# Salt stress-induced *FERROCHELATASE 1* improves resistance to salt stress by limiting sodium accumulation in *Arabidopsis thaliana*

**DOI:** 10.1038/s41598-017-13593-9

**Published:** 2017-11-07

**Authors:** Wen Ting Zhao, Sheng Jun Feng, Hua Li, Franziska Faust, Tatjana Kleine, Long Na Li, Zhi Min Yang

**Affiliations:** 1 Department of Biochemistry and Molecular Biology, College of Life Science, Nanjing Agricultural University, Nanjing, 210095 China; 2 Department of Plant Science, College of Life Science, Henan Agricultural University, Henan, 450002 China; 3 Institute of Plant Nutrition (IFZ), Justus Liebig University, Heinrich-Buff-Ring 26–32, 35392 Giessen, Germany; 4 Plant Molecular Biology (Botany), Department Biology I, Ludwig-Maximilians-University Munich, 82152 Martinsried, Germany

## Abstract

Ferrochelatase-1 as a terminal enzyme of heme biosynthesis regulates many essential metabolic and physiological processes. Whether FC1 is involved in plant response to salt stress has not been described. This study shows that Arabidopsis overexpressing *AtFC1* displays resistance to high salinity, whereas a T-DNA insertion knock-down mutant *fc1* was more sensitive to salt stress than wild-type plants. *AtFC1* conferred plant salt resistance by reducing Na^+^ concentration, enhancing K^+^ accumulation and preventing lysis of the cell membrane. Such observations were associated with the upregulation of *SOS1*, which encodes a plasma membrane Na^+^/H^+^ antiporter. *AtFC1* overexpression led to a reduced expression of several well known salt stress-responsive genes such as *NHX1* and *AVP1*, suggesting that *AtFC1*-regulated low concentration of Na^+^ in plants might not be through the mechanism for Na^+^ sequestration. To investigate the mechanism leading to the role of *AtFC1* in mediating salt stress response in plants, a transcriptome of *fc1* mutant plants under salt stress was profiled. Our data show that mutation of *AtFC1* led to 490 specific genes up-regulated and 380 specific genes down-regulated in *fc1* mutants under salt stress. Some of the genes are involved in salt-induced oxidative stress response, monovalent cation-proton (Na^+^/H^+^) exchange, and Na^+^ detoxification.

## Introduction

Soil salinity is one of the major environmental problems that seriously cause osmotic stress and ionic toxicity, thus limiting the productivity of crops. Plants have to develop remarkable capabilities for adapting themselves to adverse environments^1^. Plants also evolve various strategies to cope with the environmental challenge via perceiving stressful signals and transmitting them through diverse metabolic pathways; and upon receipt of the signal, a number of molecular and cellular responses are initiated^2^.

Plant growth responds to salt stress in three phases: Phase 0: initial response to NaCl; Phase I: osmotic phase that physiologically inhibits growth of young leaves; and Phase II: ionic phase that facilitates senescence of mature leaves^3^. Phase 0 occurs during the first minutes to hours after salt exposure. There are transient changes in turgor, growth, membrane potential^4^. During the first phase, ion accumulation in root medium may reduce the water potential and water availability to plants^5^. The second phase occurs when vacuoles no longer sequester incoming salts, but the concentration of toxic ions rises rapidly in leaves^6^. Na^+^ is the main toxic ion. Accumulation of excessive Na^+^ in cytosol is detrimental to many metabolic and physiological processes. Thus, maintaining a low concentration of cytoplasmic Na^+^ under salt stress is critical for plant growth and development^2^. Plants prevent Na^+^ accumulation in symplast through multiple ways such as extrusion, influx restriction and vacuolar sequestration^7^. Na^+^ efflux is catalyzed by a plasma membrane Na^+^/H^+^ antiporter encoded by *Salt Overly Sensitive 1* (*SOS1*)^8^. *SOS1* is mainly expressed in epidermis of root tip regions and is responsible for the long-distance Na^+^ transport from roots to shoots in xylem parenchyma to protect cells from Na^+^ toxicity^8^. Sequestration of Na^+^ into vacuoles is catalyzed by a vacuolar Na^+^/H^+^ antiporter, by which energy-consuming H^+^ transporting pumps such as H^+^-ATPase and H^+^-PPase are involved^9^. Reducing salt-induced toxicity in plants needs numerous resistant genes working together. Recent genome-wide profiling of transcriptome results in identification of a large number of genes in response to salt stress in plants^10^.

Ferrochelatase 1 (FC1, EC4.99.1.1) is the terminal enzyme of heme biosynthesis, catalyzing the insertion of ferrous iron into protoporphyrin IX^11^. Protoporphyrin IX is the branch point of the tetrapyrrole biosynthesis pathway of heme and chlorophyll. While chlorophyll is well known for its functions associated with solar energy absorption, transfer and photosynthesis in plants, heme is one of the important tetrapyrroles with functions associated with fundamental cellular aspects^12^. It is apparent that FC1 is a critical enzyme in the tetrapyrrole biosynthesis pathway for coordination of heme and apoprotein production^13^. FCs are conserved in organisms. In Arabidopsis and several other plants such as barley and cucumber, only two isoform genes of FC are available^11,14–18^. While *AtFC1* (At5g26030) is expressed in both mitochondria and chloroplasts, *AtFC2* (At2g30390) is only present in chloroplasts^11,16^. Although *AtFC1* and *AtFC2* share high identify of nucleotides (83%) and amino acids (69%), both did originate from a segmental duplication event in the Arabidopsis genome and thus belong to two distinct groups of plant ferrochelatases^11,12,18^. *AtFC1* and *AtFC2* have different expression patterns^11,13,15,16,19^. Compared to *AtFC1* that ubiquitously expresses in whole plants, *AtFC2* tends to express in stems, flowers and leaves, but not in roots^15,16^. Identifying promoters in response to biotic and abiotic stresses shows that the leaf *AtFC1* promoter activity increased in response to norflurazon (an inhibitor of carotenoid biosynthesis), wounding and viral infection, whereas *AtFC2* promoter activity was in most instances repressed^19^. Furthermore, expression of *AtFC1* could be triggered by reagents generating reactive oxygen species (ROS), inhibitors of cytoplasmic protein synthesis and other environmental stresses^13,18^. These results suggest that *FC1* is mainly involved in stress responses, whereas *FC2* is thought of generating heme for photosynthetic cytochromes^15,16,18,20^. In this paper, we showed that *AtFC1* could positively regulate Arabidopsis resistance to salinity stress. *AtFC1* overexpression improved seed germination and primary root elongation and reduced Na^+^ accumulation under salt stress, whereas the loss of function *fc1* mutant had adverse phenotypes. *AtFC1* overexpression altered expression of a set of genes responsible for Na^+^ uptake, efflux and detoxification in plants.

## Results

### Expression of *AtFC1* is upregulated by salt stress

**Figure 1:**
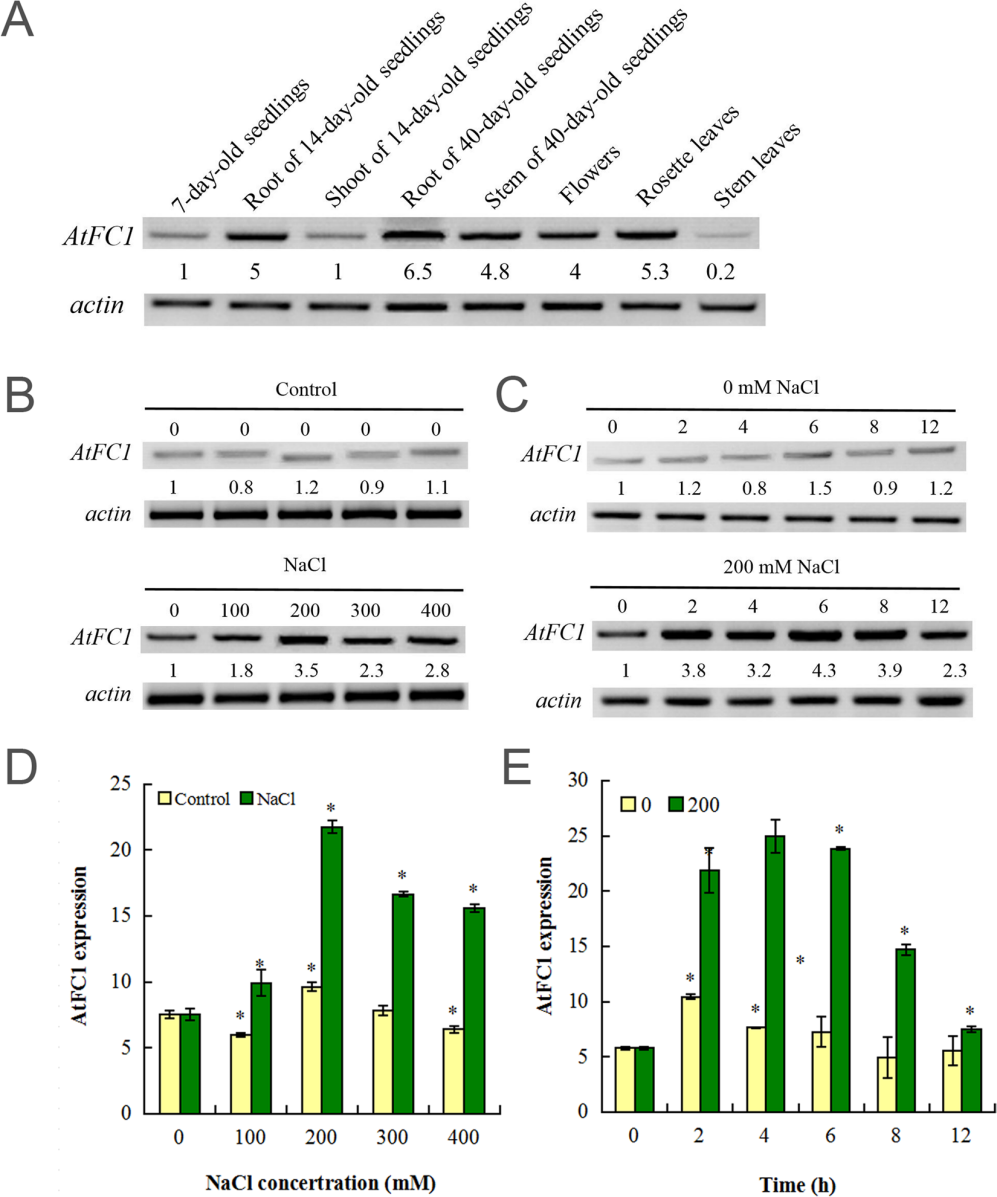
Expression pattern of *AtFC1* in Arabidopsis and its regulation by NaCl treatment. (**A**) Semi-quantitative RT-PCR (sqRT-PCR) analysis of *AtFC1* expression at different developmental stages. B-E: sqRT-PCR and qRT-PCR analyses of *AtFC1* expression in response to salt stress. Two week-old seedlings growing in MS media were exposed to 0–400 mM NaCl for 5 h (**B**,**D**) or 200 mM NaCl for 0–12 h (**C**,**E**). Total RNA was extracted from whole plants and transcripts were analyzed by sqRT-PCR (**B**,**C**) and qRT-PCR (**D**,**E**). *Actin* primers were used in PCR as an internal control. The number below the band indicates the relative abundance of the corresponding transcripts with respect to the loading control. Vertical bars represent mean values ± SE. Asterisk indicates the significant difference in expression between the treatments and control (*p* < 0.05). The color of images for sqRT-PCR was inversed. The uncropped images are shown in Supplementary Data 8.

The transcriptional expression pattern of *AtFC1* was analyzed during plant growth and development. RT-PCR analysis showed that *AtFC1* was ubiquitously expressed, including early or late developing cotyledons, roots, stems, shoots, leaves and flowers, but the abundance of their transcripts varied considerably (Fig. 1A). Roots showed a high abundance of *AtFC1* transcripts. Compared to young seedlings, mature stems and flowers also had a higher *AtFC1* expression level.

Several studies have indicated that expression of *AtFC1* was induced by wounding, ozone and oxidative stress^13,19,21,22^. To investigate the expression pattern of *AtFC1* under salt stress, transcript levels of *AtFC1* were assessed by RT-PCR. As shown in Fig. 1B, exposure of two-week old Arabidopsis seedlings to 100–400 mM NaCl induced *AtFC1* expression by 1.8–3.5 fold. The time-kinetics study, in which seedlings were treated with 200 mM NaCl for 12 hours, also showed 2.3–4.3 fold enhanced expression of *AtFC1* under salt stress (Fig. 1C). These results were well confirmed by qRT-PCR (Fig. 1D,E).

### Overexpression of *AtFC1* in Arabidopsis enhances salt resistance

**Figure 2:**
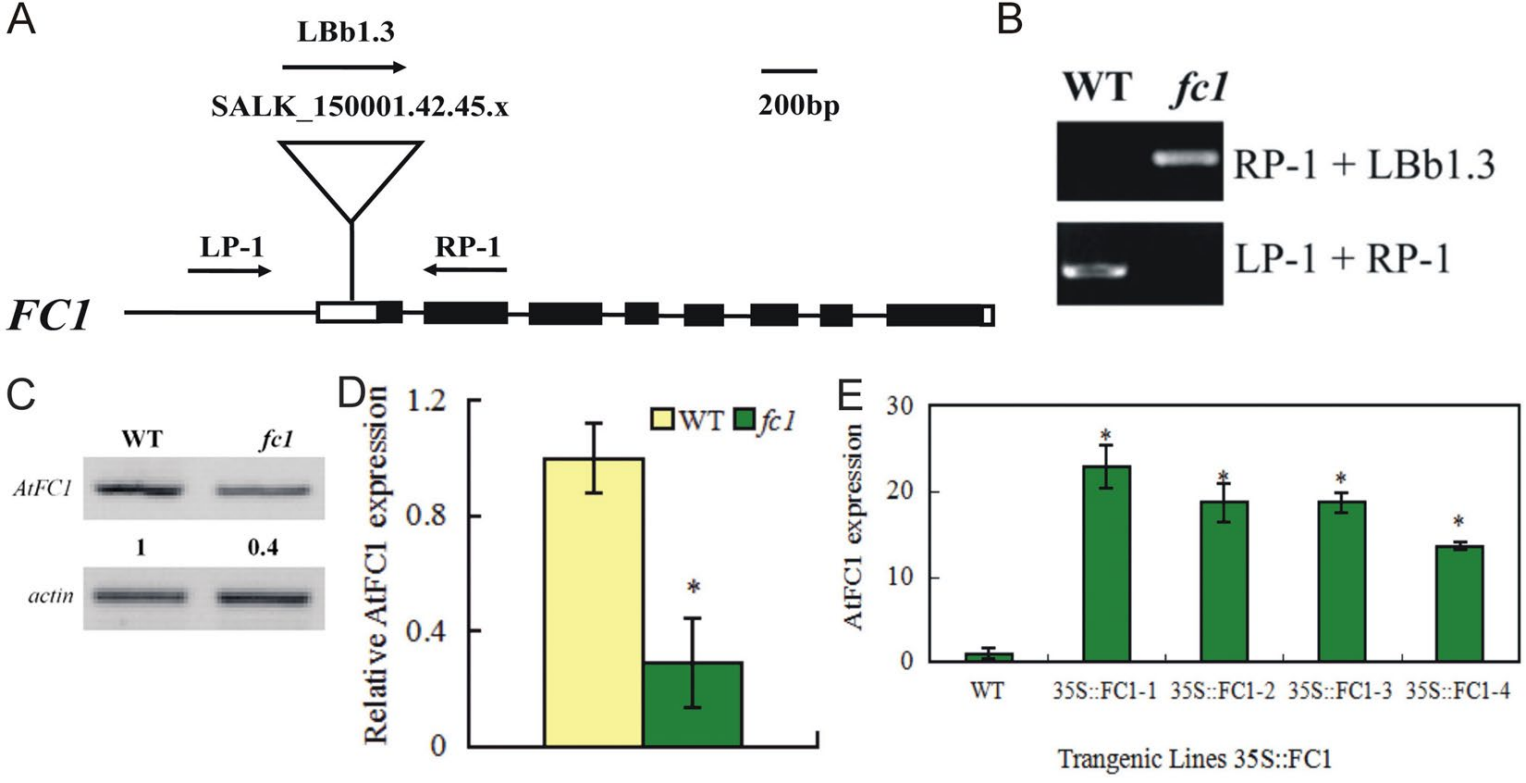
Identification of *fc1* mutant and *35 S::AtFC1* transgenic lines in Arabidopsis. (**A**) Schematic diagram of *AtFC1* structure and T-DNA diagnostic PCR and RT-PCR. The coding region and 5’ and 3’ untranslated regions are illustrated by the black and white boxes, respectively; introns are indicated by lines. The position of the T-DNA insertion is indicated by a triangle. The locations of the primer pairs used to analyze the mutation by RT-PCR are indicated by arrows. (**B**) RT-PCR analysis of the *fc1* insertion mutant. The reverse transcription products were PCR-amplified using primer pairs LP-1 + RP-1, LP-1 + LBb1.3. (**C**) RT-PCR analysis of *AtFC1* transcript levels in *fc1* mutant. (**D**) qRT-PCR analysis of *AtFC1* transcript levels in *fc1* mutant. E: qRT-PCR analysis of *AtFC1* transcript levels in *35 S::AtFC1* lines. Fourteen day-old seedlings were used for RT-PCR analysis. The number below the band indicates the relative abundance of the corresponding proteins with respect to the loading control. Vertical bars represent mean values ± SE. Asterisk indicates the significant difference in expression between the *fc1* mutant/*35 S::AtFC1* lines and wild type (*p* < 0.05). The color of image in C was inversed. The uncropped images are shown in Supplementary Data 9.

To test the biological function of *AtFC1* in Arabidopsis in response to salt stress, a T-DNA insertion mutant of *AtFC1* (SALK-150001.42.45x, *fc1*) was identified. The mutant *fc1* was verified by diagnostic PCR using gene-specific primers and found with a T-DNA insertion in the 5’-untranslated region (Fig. 2A). The RT-PCR analysis proved the right T-DNA insertion in the homozygous mutant (Fig. 2B). *AtFC1* transcripts in *fc1* were assessed by RT-PCR, showing substantial decrease compared to the wild-type (Fig. 2C,D), which was in a good agreement with the recent report^18^. We further generated transgenic lines overexpressing *AtFC1* driven by the cauliflower mosaic virus (CaMV) 35 S promoter. The homozygous transgenic lines were filtered in. The *AtFC1* mRNA level of the *35 S::AtFC1* transgenic lines was confirmed by qRT-PCR. The transgenic plants carrying *35 S::AtFC1* showed 13.5 to 22.8-fold higher transcripts of *AtFC1* than the wild-type (WT) plants (Fig. 2E).

**Figure 3:**
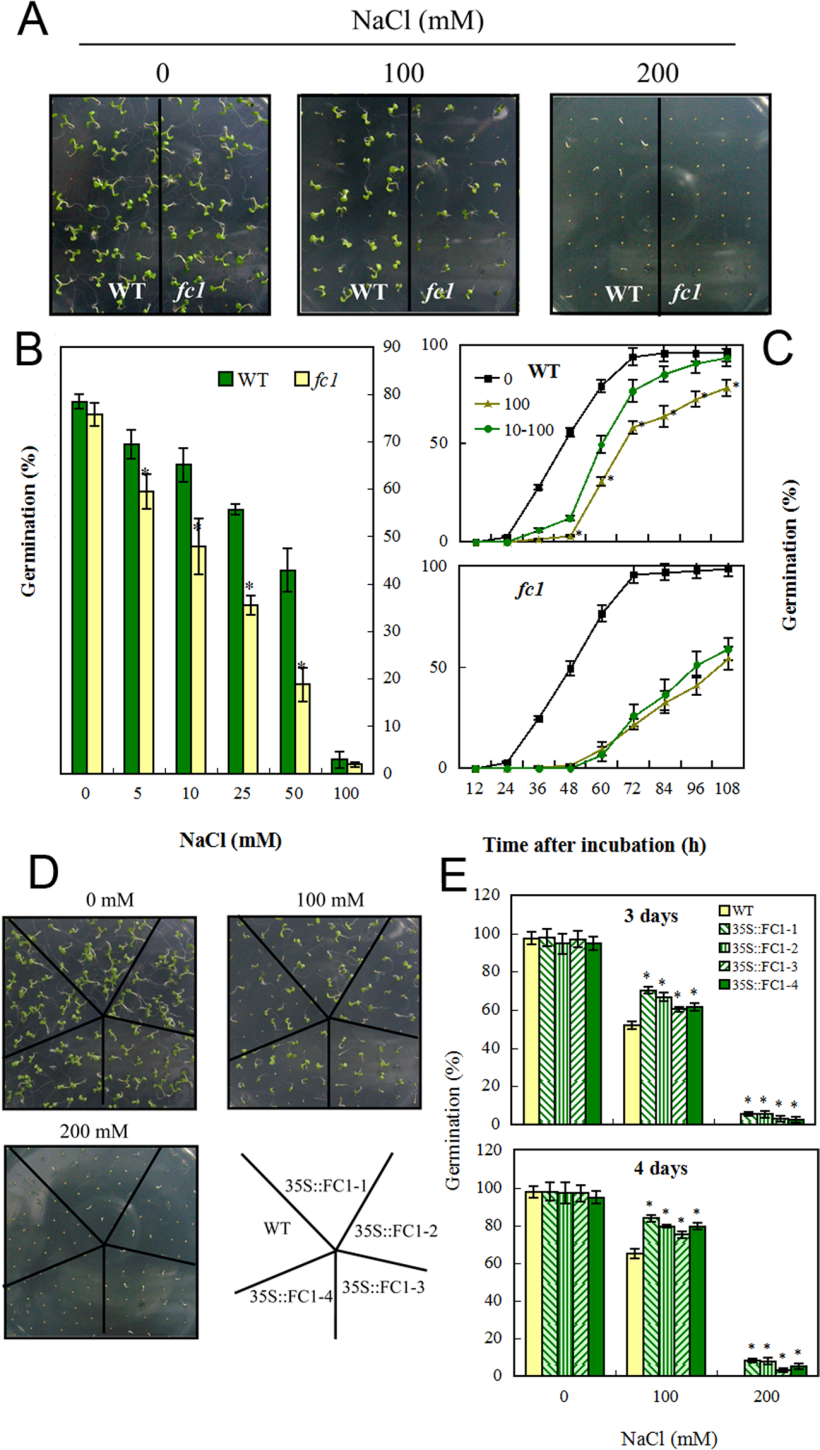
Germination responses of *fcl* mutants and *35 S::AtFC1* plants to salt stress. (**A**) Phenotypes of wild-type (WT) and *fc1* mutant seeds exposed to 0, 100 and 200 mM NaCl for 7 d. (**B**) Quantification of germination rates of WT and *fc1* seeds with 0–100 mM NaCl for 2 d. C: Quantification of germination rates of WT and *fc1* seeds in an acclimation way of 10 mM NaCl pre-treatment for 48 h (circles) and then 100 mM NaCl to the end of treatment (108 h) (triangles). Both 0 (square) and 100 (circles) mM NaCl treatments were set as control. D: Phenotypes of wild-type (WT) and *35 S::AtFC1* lines exposed the indicated concentrations of NaCl for 7 d. E: Quantification of germination rates of WT and *fc1* seeds with 0, 100 and 200 mM NaCl for 3 or 4 d. Vertical bars represent mean values ± SE. Asterisk indicates the significant difference in germination rate between *fc1* mutants/*35 S::AtFC1* lines and wild type (*p* < 0.05).

Seed germination in response to salt stress was determined in wild-type, *fc1* mutants and *AtFC1-*overexpression plants. Seeds were placed on the solid 1/2 MS medium supplemented with 0, 100 and 200 mM NaCl. First, the response of wild-type and *fc1* mutants to salt stress was compared. The *fc1* mutants always had a lower germination rate under salt stress (Fig. 3A,B). Further, a salt acclimation experiment was made with WT and *fc1* mutant seeds before exposure to 100 mM NaCl. Seeds were pretreated with 10 mM NaCl for 48 h. The WT seeds had a rapid germination after the 48 h salt acclimation, whereas *fc1* seeds had no significant increase in germination after that (Fig. 3C), indicating that loss of *AtFC1* function impaired seed germination. We then studied the effect of *AtFC1* overexpression on seed germination under salt stress. The seed germination responded differently to NaCl stress. The *35 S::AtFC1* lines always had higher germination rates than WT under salt stress (Fig. 3D,E).

**Figure 4:**
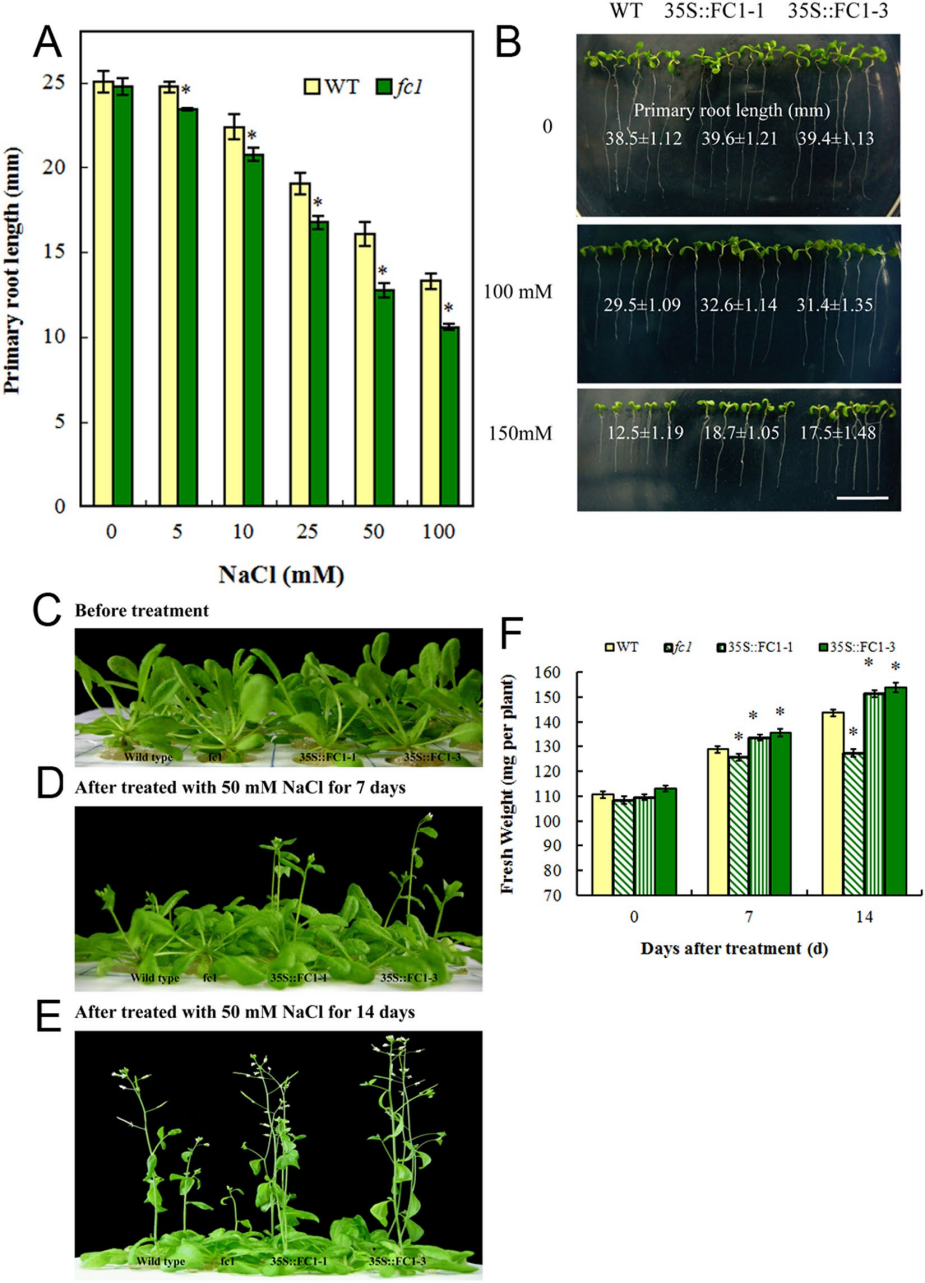
Growth responses of *fc1* and *35 S::AtFC1* plants to salt stress. (**A**) Primary root growth of wild-type and *fc1* germinating seedlings with 0–100 mM NaCl for 7 d. (**B**) Phenotype of elongation of primary roots of *35 S::AtFC1* plants with 0, 100 and 200 mM NaCl for 10 d. (**C**–**E**) Phenotype of shoots of three week-old WT, *fc1* and *35 S::AtFC1* plants exposed to 0, 100 and 150 mM NaCl for 7 and 14 d. (**F**) Quantification of fresh weight of plants treated with NaCl under the same condition of (**C**–**E**). Vertical bars represent mean values ± SE. Asterisk indicates the significant difference in fresh weight between the *fc1*/*35 S::AtFC1* lines and wild type (*p* < 0.05).

Root growth is sensitive to NaCl and is often used as a biomarker of salt stress^23^. Under 5–100 mM NaCl exposure for 7 d, the primary root elongation of *fc1* mutants was relatively weak compared to WT (Fig. 4A). In contrast, *35 S::FC1* plants displayed strong root growth compared to the wild-type under salt stress (Fig. 4B). Under 150 mM NaCl stress, elongation of *35 S::AtFC1* roots was 1.4–1.5 fold higher than that of wild-type. The *AtFC1*-improved plant growth was also found in shoots. Three-week-old WT, *fc1* and *35 S::AtFC1* seedlings were treated with 50 mM NaCl for 7 and 14 d. Thereafter, the fresh weight was measured. Compared to WT, the total fresh weight of *fc1* mutants was lower, whereas the fresh weight of *35 S::AtFC1* plants was higher (Fig. 4C–E). The fresh weight of *35 S:FC1-1* and *35 S:FC1-3* seedlings with 150 mM NaCl was increased 27–34% compared to WT (Fig. 4F).

### Overexpression of *AtFC1* attenuates electrolyte leakage and proline accumulation

**Figure 5:**
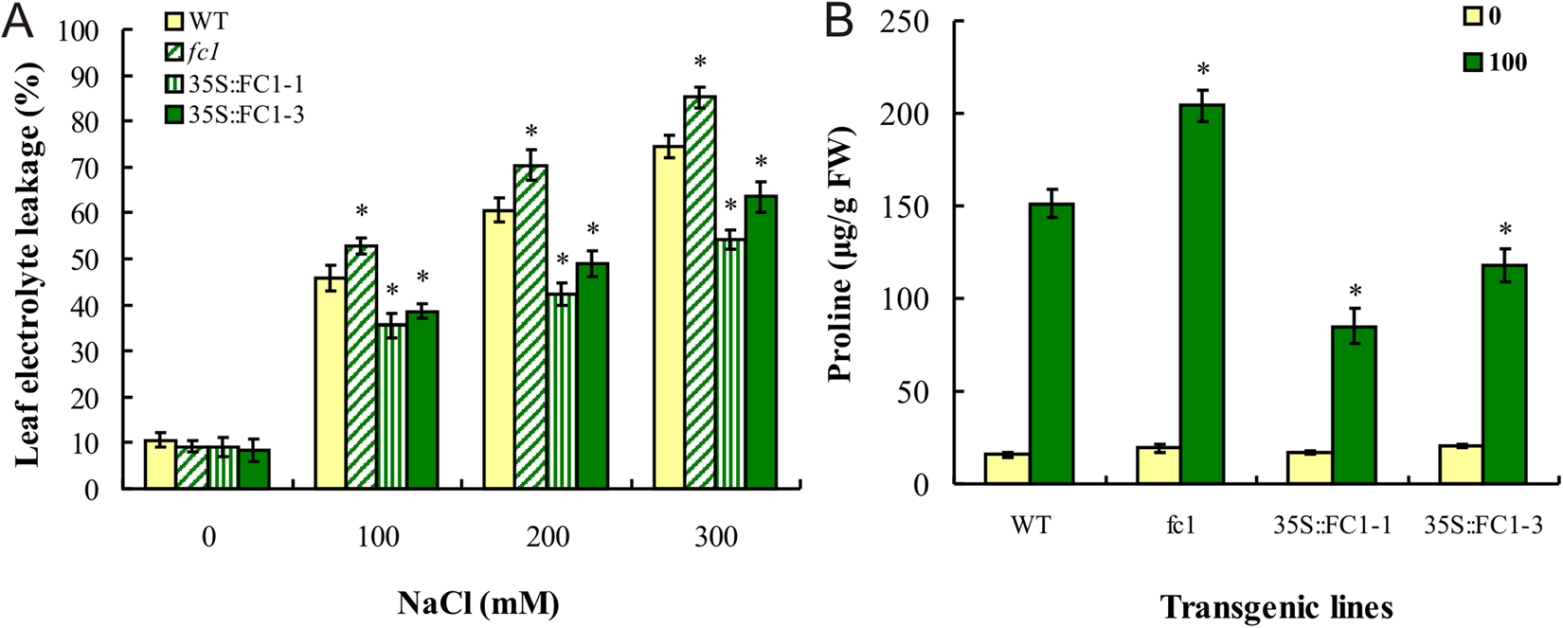
Physiological responses of *fc1* and *35 S::AtFC1* plants to salt stress. (**A**) Leaf tissues from three week-old WT, *fc1* and *35 S::AtFC1* plants were carefully excised 4 h after 0–300 mM NaCl treatments and used for electrolyte leakage (see Materials and Methods). (**B**) Three week-old WT, *fc1* and *35 S::AtFC1* plants were exposed 0 and 100 mm NaCl for 4 h before determination of free proline concentration. Vertical bars represent mean values ± SE. Asterisk indicates the significant difference between *fc1/35 S::AtFC1* and WT plants (*p* < 0.05).

The electrolyte leakage represents the damage of plasma membrane in plant cells and the degree towards abiotic stress response^24^. Compared to WT, 100–300 mM NaCl induced a higher level of electrolyte leakage in *fc1* mutants, whereas a lower level was observed in *35 S::AtFC1* plants, (Fig. 5A).

Proline acts as an osmotprotectant which plays an important role in osmotic balancing and increasing the turgor necessary for cell expansion under salt stress; in addition, it is serves as a redox potential regulator, protecting macromolecules (e.g. proteins or enzymes) from damage or denaturation caused by heat, NaCl, and other stresses^2^. Similarly, a higher level of proline was found in the *fc1* mutants than in WT under NaCl stress; in contrast, the concentration of proline in *35 S::AtFC1* plants was lower than WT (Fig. 5B).

### *AtFC1* overexpression maintains Na^+^ and K^+^ homeostasis under salt stress

**Figure 6:**
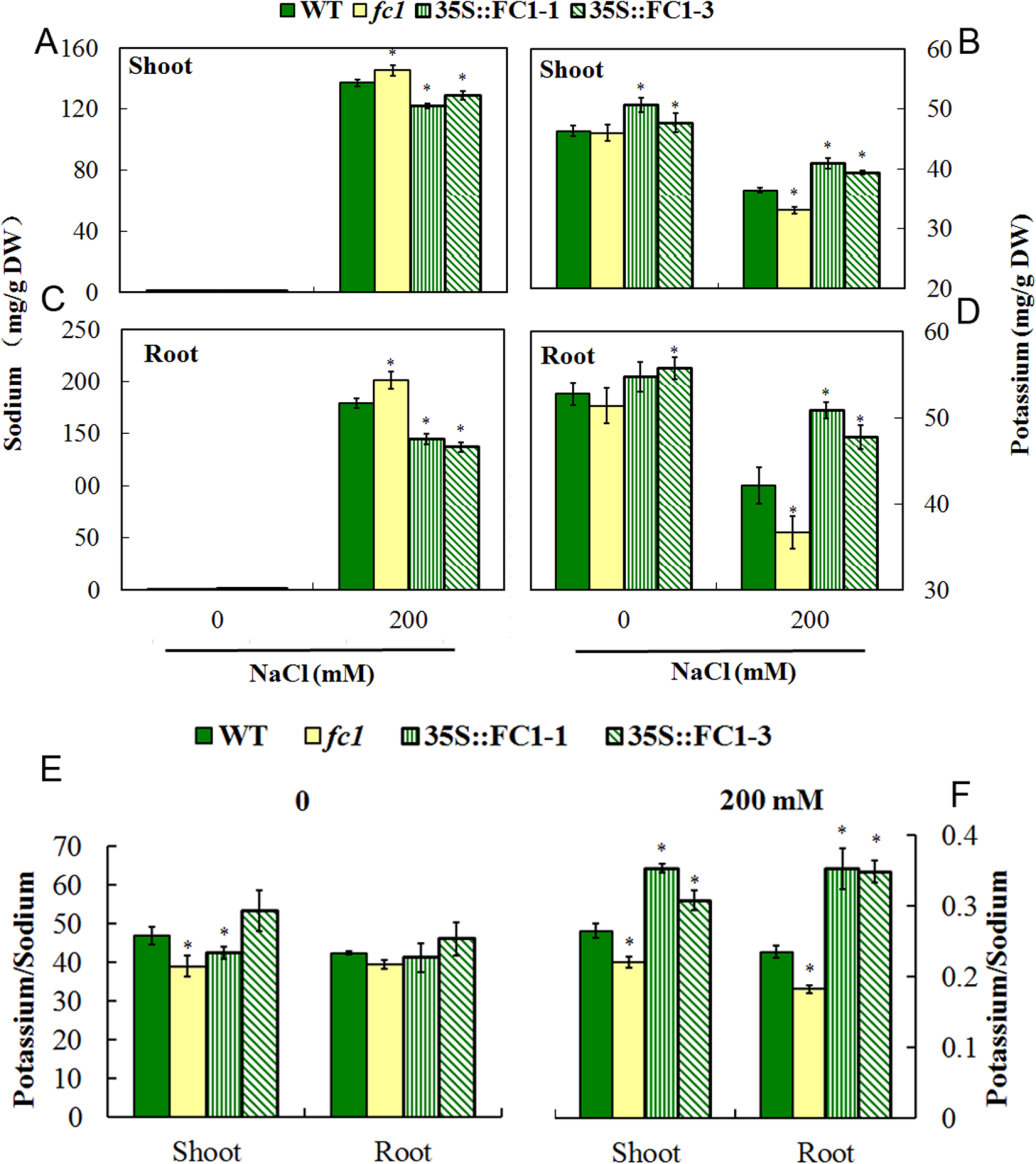
Effects of *AtFC1* overexpression on Na and K accumulation. Three week-old wild type, *fc1* mutant and over-expression transgenic plants were treated with 200 mM NaCl for 3 d. Sodium (**A**,**C**) and potassium (**B**,**D**) contents in shoots and roots were quantified. K^+^/Na^+^ ratio in shoots and roots of the *35 S::AtFC1* transgenic lines and wild type after treatment with 0 (**E**) or 200 mM NaCl (**F**) for 3 d. Vertical bars represent mean values ± SE. Asterisk indicates the significant difference between *fc1/35 S::AtFC1* and WT plants (*p* < 0.05).

Excessive Na^+^ is toxic for plants, whereas K^+^ is an opponent against Na^+^ under salt stress^25^. To figure out the physiological role of *AtFC1* in resistance to salt stress, three week-old WT, *fc1* mutant and *35 S::AtFC1* plants were treated with 200 mM NaCl for 3 d, and the concentrations of Na^+^ and K^+^ in roots and shoots were determined by coupled plasma-atomic emission spectrometry. There was no difference of Na^+^ concentrations in WT, *fc1* mutant and *35 S::AtFC1* plants grown in absence of NaCl (Fig. 6A,C). However, when exposed to 200 mM NaCl, the *fc1* mutants accumulated more Na^+^ in shoots and roots, whereas the Na^+^ concentration in the tissues was relatively lower in *35 S::AtFC1* plants compared to wild-type. In absence of NaCl, no difference of shoot K^+^ concentrations was observed between WT and *fc1* mutants (Fig. 6B); although *fc1* mutants accumulated less K^+^ in roots, there was no significant difference (Fig. 6D). Under control conditions, *35 S::AtFC1* plants accumulated a litter higher level of K^+^ in plants. However, the *35 S::AtFC1* plants accumulated more K^+^ in their shoots and roots under salt stress, whereas the K^+^ concentration was significantly lower in *fc1* mutants than in WT. We further analyzed the K^+^/Na^+^ ratio which represents the balance of the two ions under salt stress^26^. It was unclear for K^+^/Na^+^ ratio between *fc1*, *35 S::AtFC1* and WT plants under normal condition. But the imbalance of Na^+^ and K^+^ uptake led to an increase in K^+^/Na^+^ ratio in *35 S::AtFC1* plants and a decrease in K^+^/Na^+^ ratio in *fc1* mutants (Fig. 6E,F).

**Figure 7:**
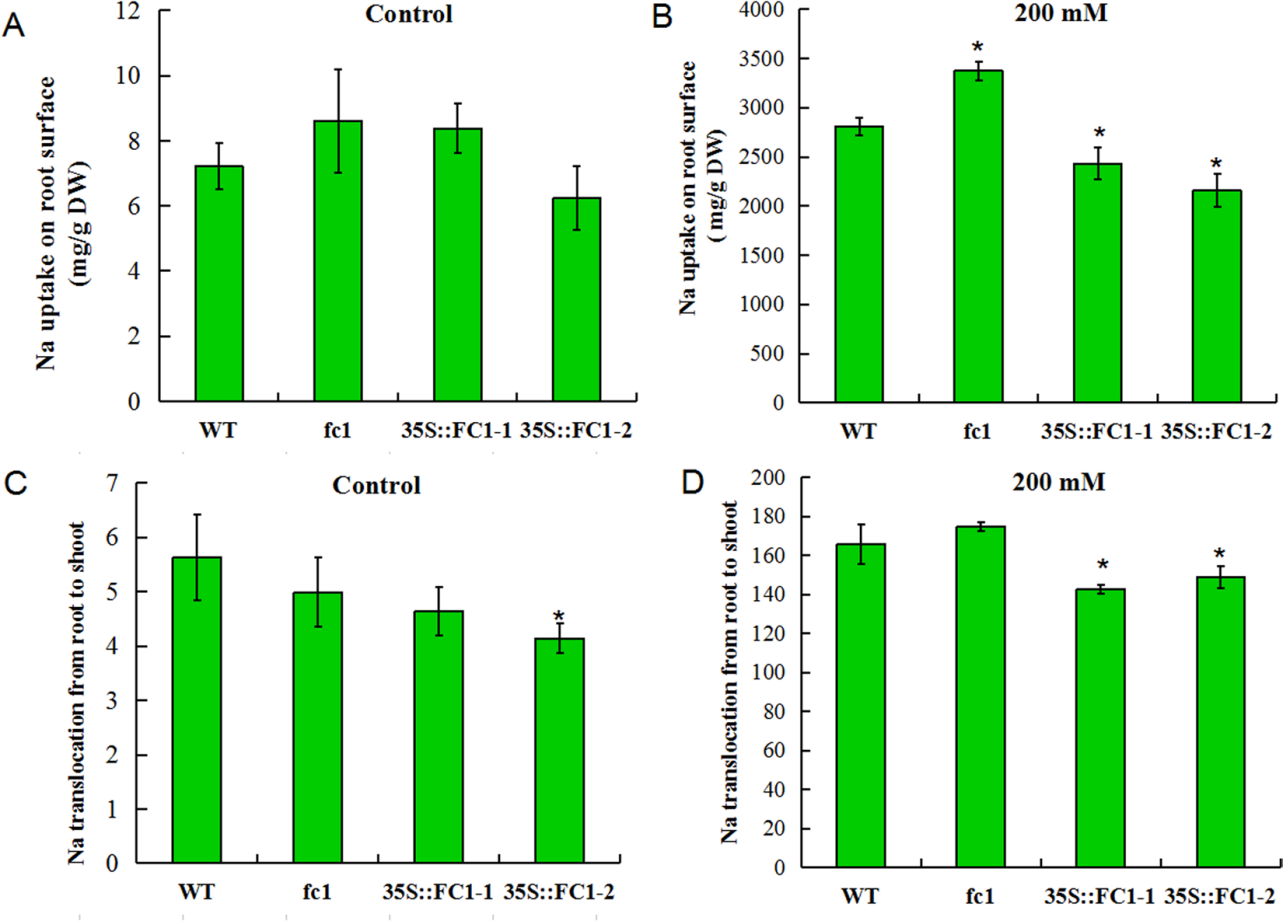
Effect of *AtFC1* over-expression on Na^+^ uptake and Na^+^ translocation. (**A**,**B**) Na^+^ uptake at root surface under control (**A**) and 200 mM NaCl (**B**) conditions. C and D: Na^+^ translocation from roots to shoots under control (**C**) and 200 mM NaCl (**D**) conditions. Vertical bars represent mean values ± SE. Asterisk indicates the significant difference between *fc1/35 S::AtFC1* and WT plants (*p* < 0.05).

To get an insight into the Na^+^ location, we analyzed the Na^+^ uptake on root surface and Na^+^ translocation from roots to shoots based on the method described previously^27^. Under normal condition, there was no significant difference of the Na^+^ uptake on root surface between WT and *35 S::AtFC1* or *fc1* mutant plants; however under salt stress (200 mM NaCl), the concentration of Na^+^ on the root surface was lower in *35 S::AtFC1* plants but higher in *fc1* mutant compared to WT (Fig. 7). Also, the *AtFC1* overexpressing plants showed less Na^+^ transfer from roots to shoots. For *fc1* mutants, no much difference was found.

**Figure 8:**
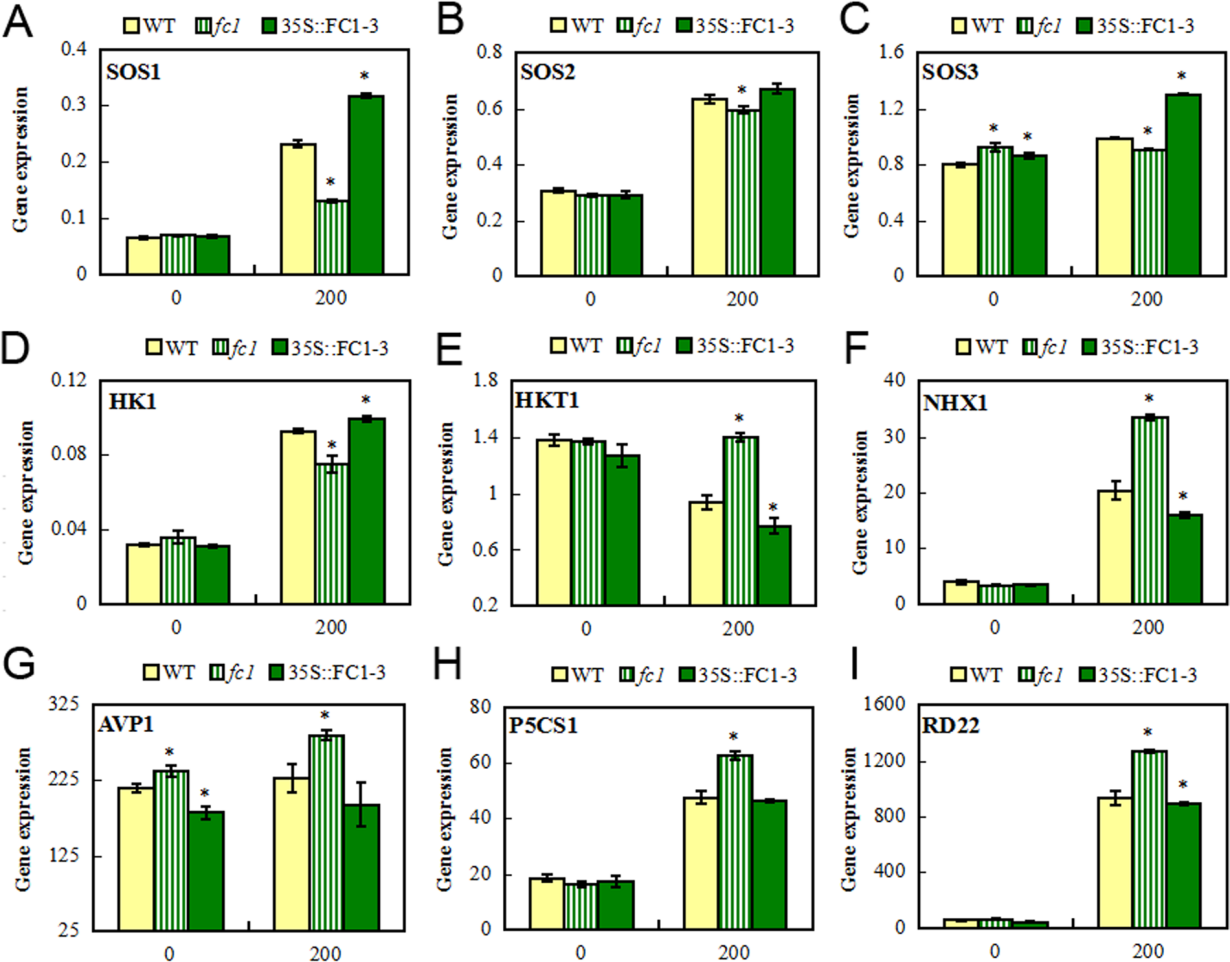
qRT-PCR analysis of salt stress-responsive genes in *fc1* mutant and *35 S::AtFC1* plants. Two week-old wild type, *fc1* mutant and *35 S::AtFC1* plants were treated with 0 and 200 mM NaCl for 4 h. Total RNA was isolated from the plants and analyzed by qRT-PCR. The graphs (*y-*axis) indicate the induction fold of the genes with 200 mM NaCl as compared with the control (0 mM NaCl) (*x-*axis). Vertical bars represent mean values ± SE. Asterisks indicate that mean values are significantly different between the *fc1*/*35 S:AtFC1* and WT (*p* < 0.05).

The altered Na^+^ accumulation due to the *AtFC1* expression prompted us to analyze genes responsible for Na^+^ and K^+^ acquisition and homeostasis. The SOS (Salt Overly Sensitive) pathway plays an important role in Arabidopsis to regulate Na^+^ concentration and maintain ionic homeostasis^2^. *SOS1* encodes a plasma membrane-localized Na^+^/H^+^ antiporter, functioning in extrusion of Na^+^ from cells and contributes to salt resistance in Arabidopsis^8^. *SOS2* encodes a member of the CBL-interacting protein kinase family, which combines with SOS3^28^. *SOS3* encodes a protein that shares significant sequence similarity with the calcineurin B subunit from yeast and neuronal calcium sensors from animals; this kind of intracellular calcium signaling through a calcineurin-like pathway mediates the beneficial effect of calcium on plant salt tolerance^2^. qRT-PCR analyses showed that compared to the controls, expression of *SOS1* and *SOS3* was lower in *fc1* mutants, whereas it was higher in *35 S::AtFC1* plants under salt stress (Fig. 8A,C). *SOS2* showed a similar expression pattern, but the *SOS2* transcription was very weak in *35 S::FC1-3* plants under salt stress (Fig. 8B).

*HK1* encodes a member of the histidine kinase family, and is considered as an osmosensor^29^. Analysis of *HK1* transcripts showed that expression of *HK1* in salt-treated *fc1* mutants was lower than in WT, accounting for about 70% of the WT (Fig. 8D). We further assessed the expression of *HKT1*, *NHX1* and *AVP1. HKT1* encodes a sodium transporter expressed in xylem parenchyma cells, but *SOS3* and other salt-related protein in the SOS pathway can inhibit the expression of *HKT1* to reduce the intracellular concentration of Na^+^^2,30^. *NHX1* encodes a vacuolar Na^+^/H^+^ antiporter involved in salt resistance and transports Na^+^ into the vacuole using the electrochemical gradient of protons generated by the vacuolar H^+^-translocating enzymes, H^+^-adenosine triphosphatase and H^+^-inorganic pyrophosphatase^31^. *AVP1* encodes an H^+^-translocating (pyrophosphate-energized) inorganic pyrophosphatase located in the vacuolar membrane^32,33^. Under salt stress, expression of *HKT1*, *NHX1* and *AVP1* was higher in *fc1* mutants, while their expression in *35 S::AtFC1* plants was lower (Fig. 8E–G). *AVP1* was an exception because it had a similar expression pattern under both –NaCl and + NaCl conditions (Fig. 8G). *P5CS1* (Delta-1-pyrroline-5-carboxylate synthase 1) is a rate-limiting enzyme in proline biosynthesis, whose mRNA is induced by drought and salinity^34^. *RD22* belongs to the DRE/CRT (drought responsive/C-repeat) elements-containing class of stress-responsive genes^35^. qRT-PCR analysis showed that both genes had a expression pattern similar to *NHX1* in *fc1* mutants and *35 S::AtFC1* plants under salt stress (Fig. 8H,I).

### Exogenous hematin alleviates tissue damage of *fc1* mutant under salt stress

**Figure 9:**
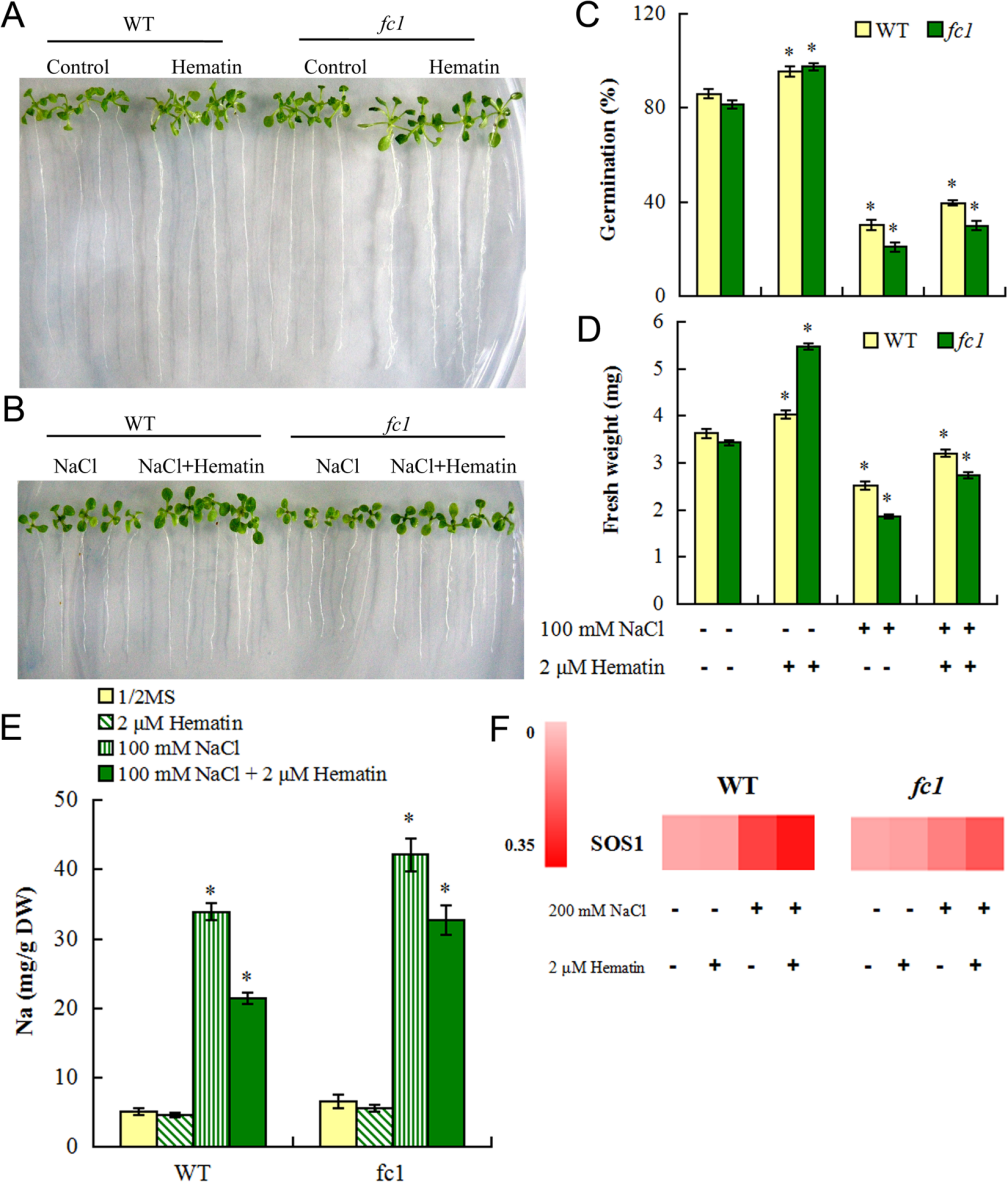
Effect of exogenous hematin on growth, Na^+^ concentration and *SOS1* expression in fc1 mutants under salt stress. (**A**,**B**) Wild-type and *fc1* mutant plants after germination were grown in MS medium containing 0 or 2 μM hematin with or without 100 mM NaCl for 14 d. Exogenous hematin effect was assayed as the whole plants fresh weight when treated with or without hematin. C: Germination rate of WT and *fc1* seeds with 100 mM NaCl and/or 2 μM hematin for 7 d. D: Fresh weight of WT and *fc1* seeds with 100 mM NaCl and/or 2 μM hematin for 7 d. E: Three-week-old wild type and *fc1* mutant plants were treated with 100 mM NaCl with and/or 2 μM hematin for 3 days. Then sodium ion contents in whole plants were detected. F: Two week-old WT and *fc1* mutant plants were treated with 0 and 200 mM NaCl with and/or 2 μM hematin for 4 h. Total RNA was isolated from the plants and analyzed by qRT-PCR. *SOS1* transcript level was analyzed by qRT-PCR. Vertical bars represent Values represent mean values ± SE. Asterisk indicates the significant difference between *fc1* mutant and wild type (*p* < 0.05).

Knowing that *AtFC1* was involved in plant salt stress response, we asked whether feeding *fc1* with heme, the product of *AtFC1* could restore the impaired seed germination caused by NaCl stress. To validate the assumption, the *fc1* mutant plants were treated with hematin (protoheme), a highly stable heme substitute^36,37^. Under salt stress (100 mM NaCl), the *fc1* seeds showed a reduced germination rate, but adding 2 μM hematin to the growth media increased the number of germinating seeds (Fig. 9A–C). Addition of hematin led to a 43% recovery in germination rate compared to the control in which no hematin was added. The exogenous hematin could also recover 47% fresh weight of *fc1* mutant plants (Fig. 9D). These results indicate that the heme produced by FC1 is partially responsible for resistance of plant to salt stress. Further functional analysis of hematin was performed by assessing Na^+^ accumulation in *fc1* mutant plants. Three week-old WT and *fc1* plants were exposed to 100 mM NaCl with or without 2 μM hematin for 3 d. Compared to NaCl treatment alone, concomitant supply of hematin resulted in a significant decrease of Na^+^ accumulation in *fc1* mutants (Fig. 9E). Furthermore, qRT-PCR analysis showed that expression of *SOS1* with NaCl was enhanced by hematin (Fig. 9F).

### Regulation of *FC1* involves extensive transcriptome remodeling

**Figure 10:**
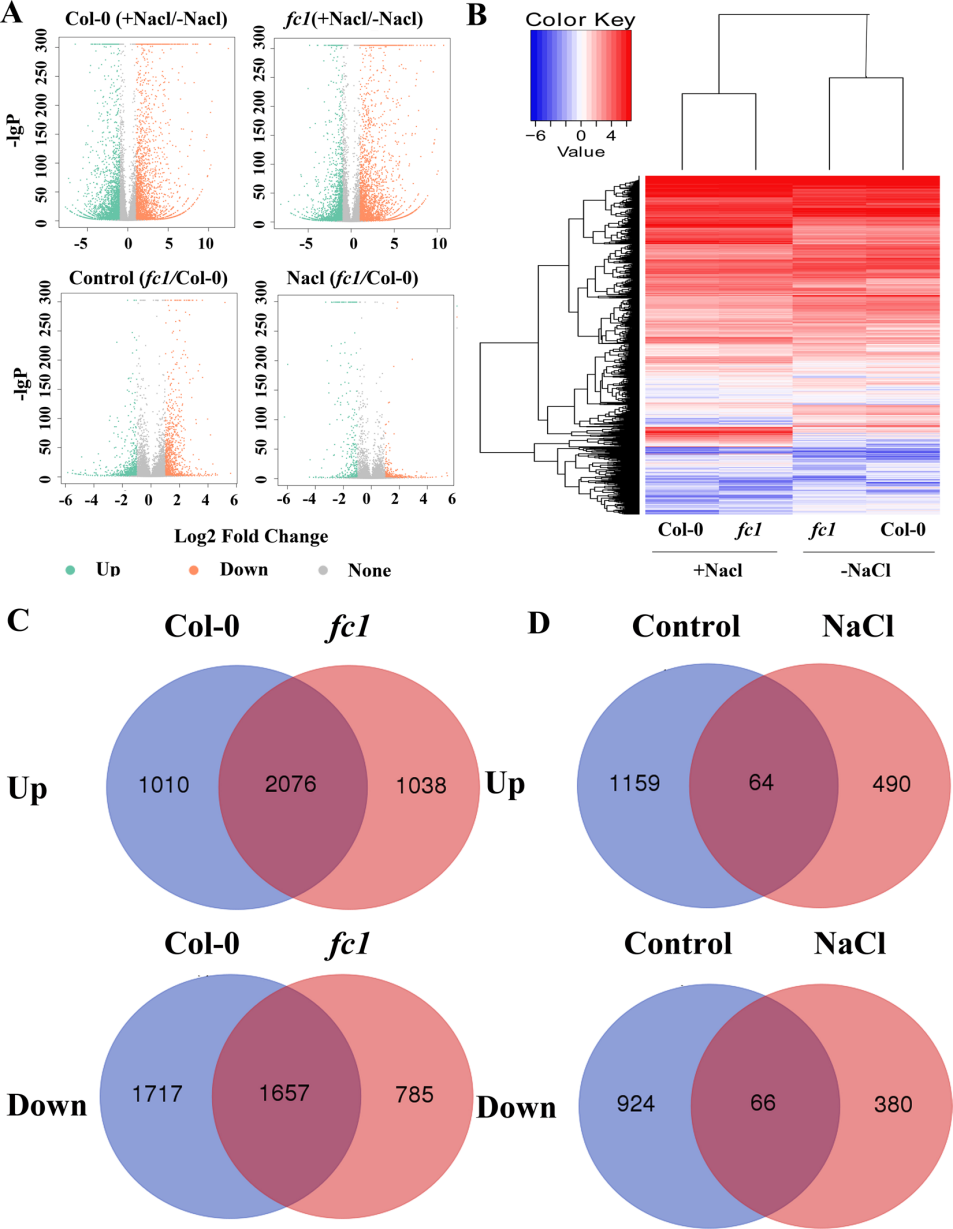
Differentially expressed genes in wild-type (WT, Col-0) and *fc1* mutant plants under salt stress as determined by mRNA-seq. (**A**) Differential transcript abundance of NaCl-free and NaCl-treated WT and *fc1* mutant plants. The *x* axis represents the log_2_ fold change under the mean normalized expression of all transcripts (*y* axis). Green dots indicate the down-regulated genes and red dots indicate the up-regulated genes. (**B**) Heatmap representation of a one-dimensional hierarchical clustering of differential gene expression for the NaCl-exposed seedlings (WT and *fc1*) relative to the control (NaCl-free). (**C**) Venn diagrams showing up- and down-regulated genes in WT and *fc1* mutant seedlings under salt stress relative to control conditions. (**D**) Venn diagrams showing up- and down-regulated genes in *fc1* seedlings relative to WT seedlings grown under the control (0 mM) and 200 mM NaCl treatment.

To identify putative *FC1* downstream genes associated with the salt stress response in Arabidopsis, we comparatively analyzed the global transcripts of *fc1* mutant and wild-type plants by RNA-sequencing. In total, 30.0–31.6 million clean reads were generated in four libraries using Illumina sequencing technology (Supplementary Data 1). Mapping the reads to Arabidopsis genome led to identification of 6460 (3086 up and 3374 down) and 5556 (3114 up and 2442 down) genes in wild-type and *fc1* mutant plants (> two fold change, *p*< 0.05) under salt stress, respectively (Supplementary Data 2–5). Figure 9A shows the distribution of the differentially expressed genes (DEGs) represented by color dots (blue, down, and red, up). Compared to the wild-type Col-0, *fc1* mutants under salt stress showed more red dots (more DEGs up-regulated) and less blue dots (less DEGs down-regulated). Under the control (-NaCl) and NaCl treatment, more red dots were detected than blue dots for *fcl* / Col-0 ratio, indicating that in the *fc1* mutant plants more genes were induced relative to wide-type (Col-0) (Fig. 10A). This observation was reflected by the heat-map graph (Fig. 10B), from which the same results could be figured out. To detail the gene expression, Venn diagrams were plotted and showed that 1010 genes were specifically induced and 1717 were repressed in Col-0, while 1038 genes were specifically induced and 785 genes were repressed in *fc1* mutant plants under salt stress (Fig. 10C). By comparative analysis of transcripts between *fc1* mutant and wild-type plants, 1159 genes were found to be specifically induced and 924 genes were repressed under control condition, whereas 490 genes were specifically induced and 380 genes were repressed under salt stress (Fig. 10D). The expression patterns of some randomly selected genes were well validated by qRT-PCR (Supplementary Data 6)

**Figure 11:**
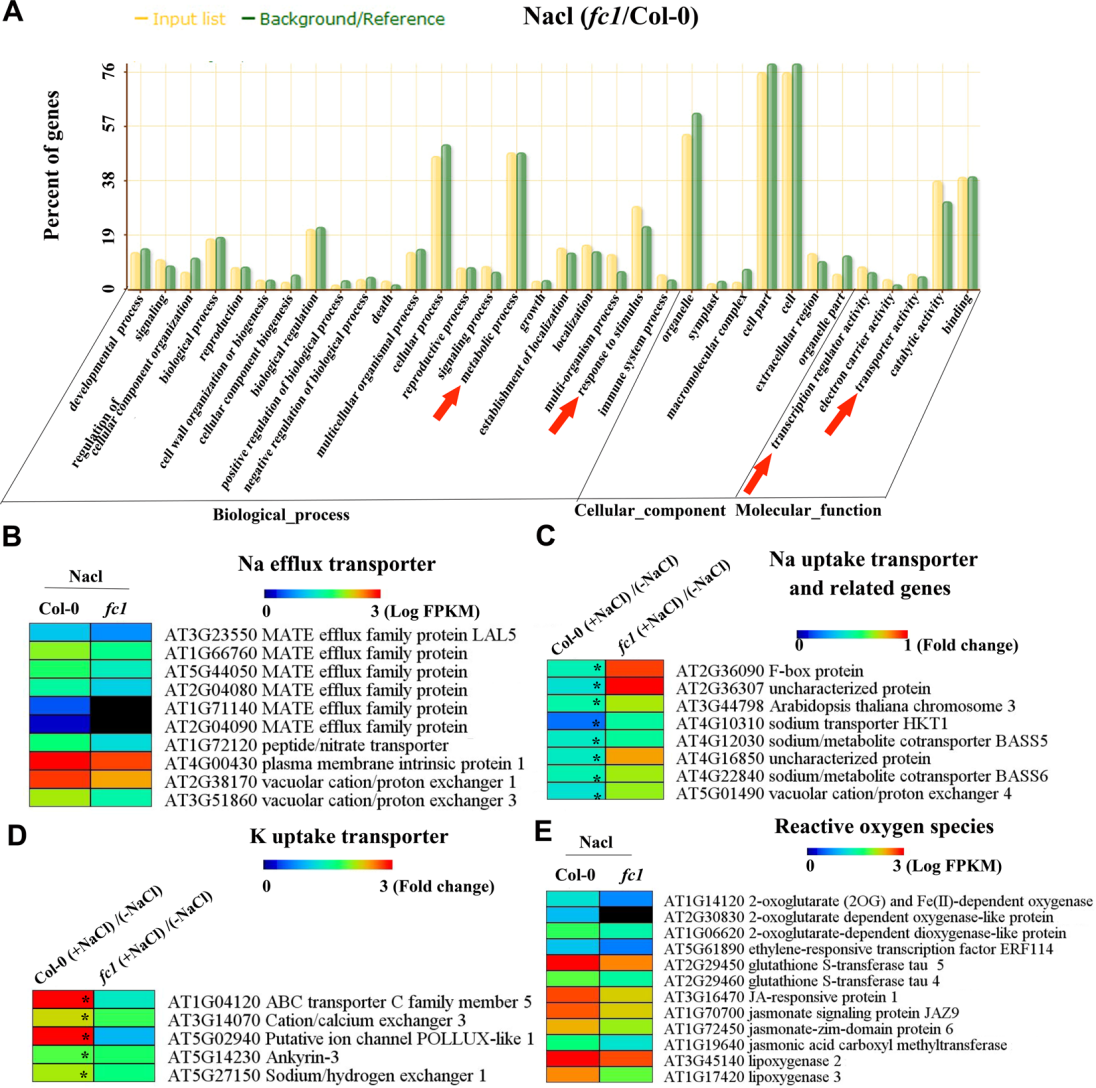
Differential gene expression as determined by mRNA-seq for *fc1* seedlings relative to the wild-type (WT, Col-0) under NaCl stress. (**A**) GO enrichment analysis of specific genes in *fc1* mutants which come from the 490 up-expression and 380 down-expression genes of DEGs in *fc1* seedlings relative to Col-0 under NaCl stress. (**B**) Heat map represents the transcript levels of genes encoding Na efflux transporter genes of *fc1* mutant and Col-1. (**C**,**D**): Heat map represents the DEGs encoding sodium (**C**) and potassium (**D**) uptake transporters/related genes in Col-0 and *fc1* mutant plants under normal and NaCl stress. Asterisks mean significantly different expressed genes (*p* < 0.05). (**E**) Heat map represents the gene expression levels of ROS (Reactive oxygen species)-related genes in Col-0 and *fc1* mutant plants.

By BGI WEGO (Web Gene Ontology Annotation Plotting), the DEGs from salt-exposed *fc1*/Col-0 samples were functionally classified. Based on their functional specificity, these DEGs were subdivided into three major groups including biological process, cellular component and molecular function (Fig. 11A). Several categories such as metabolic process, response to stimulus, transcription regulation activity and transporter activity were presented. We further specified some DEGs based on the GO categories. The first group contains genes encoding Na efflux transporters (Fig. 11B), which are responsible for Na exclusion in plants when exposed to excess salt^2^. The second group comprised genes encoding Na uptake transporters and Na tolerant-related proteins, such as sodium transporter HKT1 and sodium/metabolite co-transporter (Fig. 11C). Compared to WT, these genes in *fc1* mutants were induced under salt stress. Potassium is an important ion balancing excessive sodium during plants subjected to salt stress. Examination of K^+^ uptake transporter genes revealed that five were transcriptionally repressed in *fc1* mutants (Fig. 11D). Finally, expression of a group of ROS (reactive oxygen species)-responsive genes was also found to be lower in *fc1* mutants than in wild-type (Fig. 11E). These results indicate that disruption of *FC1* expression could modify transcription of genes responsible for salt exclusion and detoxification.

## Discussion

Although several biological and defense functions of *FC1* in Arabidopsis and other plant species have been identified in recent years^11,12,18,19,38,39^, the functional regulation of plant response to salt stress by *FC1* has not been described. A recent study using ATH1 microarray chip demonstrated that some genes (*e.g*. SALT-INDUCIBLE ZINC FINGER 2 and WRKY33) associated with salt stress could be co-regulated by *FC1* ^18^, suggesting that *FC1* would be involved in the salt stress response. The present study provided genetic and physiological evidence that *AtFC1* was able to regulate plant resistance to salt stress. *AtFC1* was transcriptionally induced by NaCl. *AtFC1* overexpression resisted salt stress by promoting seed germination (Fig. 3E), root and shoot growth (Fig. 4), accumulating less Na^+^ and more K^+^ (Fig. 6) in plants. By contrast, *AtFC1* loss of function led to adverse phenotypes. Importantly, several genes responsible for Na^+^/H^+^ antiporter were up-regulated in *35 S::AtFC1* plants. These results indicate that expression of *AtFC1* is required for plant resistance to salt stress.

Minimizing the concentration of cytoplasmic Na^+^ is critical for plant growth and development under salt stress. Strategies for fighting against excess Na^+^ in plant cells were proposed^1,2^. Na^+^ exclusion is one of the most efficiently resistant mechanisms^7^. The SOS pathway is one of the most extensively studied mechanisms for controlling Na^+^ accumulation in plants^2^. During salt stress, a myristoylated calcium-binding protein encoded by *SOS3* presumably senses the salt-elicited calcium signal and translates it to downstream responses^40^. SOS3 interacts with and activates SOS2, a serine/threonine protein kinase^28^. Both SOS2 and SOS3 regulate the expression of *SOS1*, a plasma membrane Na^+^/H^+^ antiporter which mediates Na^+^ efflux^8^. SOS1 can act as a Na^+^ sensor of adjusting Na^+^ long-distance transport from roots to shoots and protecting cells from Na^+^ toxicity^8,41^. *AtFC1-*improved resistance to salt stress was associated with activation of *SOSs* because these genes, particularly for *SOS1* were induced in *35 S::AtFC1* plants. The upregulation of *SOS1* might be responsible for the low level of Na^+^ in the *35 S::AtFC1* plants. We examined cellular and tissue Na^+^ distribution in transgenic plants and found that *AtFC1* overexpression led to a lower concentration of Na^+^ on the root cellular surface and less Na^+^ translocated from roots to shoots (Fig. 7). This is consistent with the result that *35 S::AtFC1* plants accumulated less Na in roots and shoots. In addition, RNA-seq profiled many Na efflux transporter genes repressed, while some Na uptake transporter genes were enhanced in *fc1* mutant plants (Fig. 11). Several other salt stress-responsive genes, such as *NHX1* and *AVP1* were examined. Both genes are responsible for sequestration of Na^+^ into vacuoles^31,33^. However, expression of *NHX1* and *AVP1* was down-regulated in *35 S::AtFC1* plants, suggesting that sequestration of Na^+^ into vacuoles by *NHX1* and *AVP1* was not be involved in *AtFC1-*mediated salt tolerance mechanism.

Heme is one of the most important tetrapyrroles in plants as it serves as a cofactor for many enzymes, transporters and proteins involved in the essential biological processes^36,39,42^. Furthermore, *FC1* was shown to be induced with demand for heme for respiratory cytochromes and heme-proteins as part of general defense responses^13,18,19^. To address the question whether heme is also involved in salt stress response, its regulatory function in this regard was tested by supplying hematin to *fc1* mutants and WT plants. Exogenous hematin could partially improve seed germination rate and fresh weight of *fc1* mutant plants under salt stress, and addition of hematin could also reduce the Na content in *fc1* mutants. These results suggest that *AtFC1*-improved plant resistance to salt stress not only depended on *AtFC1* itself but on heme as well. Although AtFC1 generates heme in plastids, AtFC1 could co-induced with heme-proteins outside plastids, which consequently coordinate the defense response to wounding and ozone-induced oxidative stresses^13^. One of the striking heme-containing enzymes is heme oxygenase-1 (HO1 or HY1) that regulates biosynthesis of phytochrome by taking advantage of heme as substrate to yields biliverdin IXα, carbon monoxide (CO) and iron^43^. HO1 was induced by abiotic stresses including salinity and heavy metals^36,42^. In Arabidopsis, transgenic *ho1* mutants overexpressing *AtHO1* resisted salt stress by limiting K^+^ efflux and facilitating H^+^ efflux^44^. The enhanced H^+^ efflux was associated with activation of plasma membrane H^+^-ATPases (AHA1/2/3) in root epidermis and Na + /H + antiporter (SOS1) in the plasma membrane of AtHO overexpressors^44^. In this way, AtHO1 acted as an essential component of the salt acclimation signaling pathway^36^. In addition to HO1, many other proteins such as ascorbate peroxidases and cytochrome P450 reductases with heme as co-enzyme are also involved in plant response to abiotic stress^42,43^. Thus, AtFC1-improved salt stress resistance likely depends on heme through interacting with heme-proteins.

It is mentionable that *AtFC1*-regulated salt stress resistance involved potassium homeostasis. Our data showed that the *35 S::AtFC1* plants had a higher concentration of tissue K^+^, whereas the *fc1* mutant plants had a lower concentration of K^+^ than WT under salt stress. Overload of Na^+^ can dramatically depolarize the plasma membrane, leading to K^+^ efflux via depolarization-activated outward rectifying K^+^ (KOR) channels^45^. *AtFC1* overexpression prevented the loss of K^+^ in salt-stressed roots. This was reinforced by our RNA-seq datasets, from which a group of K^+^ uptake transporter genes in response to salt stress was profiled. For example, the cation/calcium exchanger 3 (AtCCX3) identified here has been well characterized as an endomembrane H^+^-dependent K^+^ transporter^46^. ABC transporter C family member 5 (AtABCC5 or AtMRP5) mediating guild cell opening was characterized involving ABA signaling^47^. Both *AtCCX3* and *AtABCC5* are involved in abiotic stress responses, but both genes showed significantly lower abundance in *fc1* mutants than in WT under salt stress (Fig. 11D). These results suggest that the increased K^+^ which accompanied the reduced Na^+^ in *35 S::AtFC1* plants would be mediated by a mechanism for K^+^ influx and Na^+^ efflux. Further investigation will be required to highlight the essential role of *AtFC1* in mediating K^+^ and Na^+^ homeostasis in plants.

To figure out the impact of *AtFC1* on its downstream genes and the mechanism for *AtFC1* regulation, we profiled transcriptomes of *fc1* mutant plants exposed to –NaCl and + NaCl. Our data show that mutation of *AtFC1* led to 1038 specific genes up-regulated in *fc1* mutants under salt stress; by contrast there were 785 specific genes down-regulated in *fc1* mutants. These results indicate that mutation of *AtFC1* was able to modify the transcriptional pattern of more genes under salt stress. By profiling the specifically up- or down-regulated genes in *fc1* mutants, we show that many genes were involved in biological pathways including cellular components and molecular functions. A fairly number of genes that are responding to or mediate plant response to salt stress under *FC1* pathway have been identified. For example, the NaCl-specific responsive genes such as cytochrome P450^10^, was repressed in *fc1* mutants under salt stress, and many other genes related to salt stress response were also found to be altered. The vacuolar-type H + -ATPase (V-ATPase) is a multi-subunit endomembrane proton pump involved in ion vesicle trafficking and adaptation to salt stress^42^. A V-ATPase (AT4G23710) in salt-exposed *fc1* mutant plants was found to be down-regulated (< 2 fold change, *p*<0.05). Simultaneously, several other types of monovalent cation-proton antiporters including vacuolar cation/proton exchangers or sodium/hydrogen exchangers were found to be transcriptionally repressed. These cation-proton antiporters have been identified to be responsible for cellular Na^+^ efflux and maintenance of ion homeostasis and excessive Na^+^ detoxification^48^.

Taken together, this study identified a new function of *AtFC1* that involves plant response to salt stress. Overexpression of *AtFC1* conferred plant resistance to NaCl stress. The strong expression of *AtFC1*, which ensures the supply of the cofactor for a variety of heme-proteins and other putative metabolism, is required in plant resistance to salt stress. The salt stress-induced *AtFC1* expression in the tetrapyrrole pathway was associated with activation or suppression of many genes responsible for Na^+^ exclusion or sequestration, antioxidative stress, phytohormones regulation and other defense components, suggesting a cross-talk between the *FC1* pathway and salt-responsive resistance pathway. Thus, our data broaden our understanding of a new role of FC1 in mediating plant resistance to salt stress.

## Materials and Methods

### Plant materials and growth condition

*Arabidopsis thaliana* (ecotype Col-0) was used throughout the study. The *FC1* (*At5g26030*) T-DNA insertion mutant *fc1* (SALK_15000.142.45.X, *Col* background) were obtained from the Arabidopsis Biological Resource Center. Seeds were surface sterilized and germinated on half-strength MS medium containing 1 to 3% sucrose and 0.8% phytoagar (pH 5.7) in a growth chamber at 22 °C with 100 μE m^−2^ s^−1^ photosynthetically active radiation and a 16 h light/8 h dark cycle. Two week-old seedlings were used for NaCl (100–400 mM) treatments for 0–12 h depending on the experiment conducted.

### Mutant analysis

The T-DNA insertion position and homozygous lines were verified according to the instructions on the Salk Signal website (http://signal.salk.edu/isects.html). Primers were designed using the SALK T-DNA verification primer design program (http://signal.salk.edu/tdnaprimers.2.html). For qRT-PCR analysis of the insertion mutants, total RNA was isolated and reverse-transcribed. The reverse transcription products were then PCR-amplified. The internal control was normalized with *Actin*.

### RT-PCR analysis

Total RNA was extracted using column plant RNAout Kit (Tiandz). A 1% agarose gel, stained by ethidium bromide, was run to check the integrity of the RNA. All RNA samples were quantified and examined for protein contamination (A260 nm/A280 nm ratios) and reagent contamination (A260 nm/A230 nm ratios) by a Nanodrop ND 1000 spectrophotometer.

The first strand cDNA was synthesized from 1.0 µg total RNA by Moloney Murine Leukemia Virus Reverse Transcriptase (Promega) using oligo (dT) primers. Transcription of genes was analyzed by quantitative real-time RT-PCR (qRT-PCR) using the fluorescent intercalating dye SYBR-Green in a detection system (MJ Research, Opticon 2) in a final volume of 20 µL containing 2 µL of a 1/10 dilution of cDNA in water, 10 µL of the 2 × SYBR Premix Ex Taq (TaKaRa) and 200 nM of forward and reverse primers (Supplementary Data 7). The thermal cycling conditions were 40 cycles of 95 °C for 5 s for denaturation and 60 °C for 30 s for annealing and extension. All reactions were run in triplicate by monitoring the dissociation curve to control the dimers. PCR efficiency was determined by a series of 2-fold dilutions of cDNAs. The calculated efficiency of all primer pairs was 0.9 to 1.0. Gene *Actin 2* was used as a reference and relative expression levels of genes were presented by 2^−ΔCT^. For semi-quantitative RT-PCR (sqRT-PCR), the gene specific primer (Supplementary Data 7) were used for PCR reactions under the following conditions: pre-denaturation at 94 °C for 5 min, followed by 30 cycles of 30 s at 94 °C, 30 s at a specific annealing temperature (57 °C), and 30 s at 72 °C. As an internal control and to exclude genomic contamination, *Actin* was amplified (same cycling conditions as above for 28 cycles) from the same cDNA samples.

### Transformation of *AtFC1* in Arabidopsis

The *AtFC1* genomic sequence was inserted into the downstream of the CaMV35s promoter in the binary vector pBI121^23^. The constructs were then transferred into *Agrobacterium tumefaciens* for Arabidopsis transformation by the floral dip method. Positive transgenic lines were selected on the 1/2 MS medium with 50 mg/L kanamycin. More than fifteen independent transgenic lines were obtained, and four of them were presented. In this study, the homozygous lines (T4) were used.

### Germination assay, root growth and fresh weight measurement

Seeds of wild-type, mutant and transgenic plants were grown on the same plate containing MS medium with or without different concentrations of NaCl or hematin. Plants were grown under conditions of 22 °C with 100 μEm^−2^s^−1^ photosynthetically active radiation and a 16 h light/8 h dark cycle. The germination (fully emerged radicle) was recorded. The root length was measured with a ruler, and the fresh mass was weighted at the indicated times.

### Analysis of plasma membrane permeability and proline

Plasma membrane permeability of tissues was determined according to the method described previously^24^. Leaf and root segments were immersed in tubes with deionized water for 30 min, followed by measurement of conductivity of bathing medium (EC1) with a conductivity meter (METTLER TOLEDO FE30-FiveEasy™). Samples were boiled for 20 min and the conductivity of tissues (EC2) was measured. The percent leakage of electrolytes was calculated as the ratio of EC1/EC2. For proline analysis, seedlings were harvested, frozen in liquid nitrogen and dried by lyophilization. Approximately 50 mg of dried seedling tissue was ground in 3% sulfosalicylic acid to extract free proline. Proline concentration was determined as described previously^49^.

### Quantification of ion concentration

Fresh seedlings were harvested and dried at 80 °C, and digested with the mixture of nitric acid and hydrogen peroxide using microwave system (MARS, CEM). The digested samples were used to quantify Na^+^ and K^+^ in roots and shoots using inductively coupled plasma-atomic emission spectrometry (ICP-AES) (Optimal 2100DV, Perkin Elmer Instruments). The Na^+^ uptake (1) and Na^+^ translocation (2) was calculated as follows^27^: Na^+^ uptake = total Na^+^ content / root dry weight (1); and Na^+^ translocation = shoot Na^+^ content / root Na^+^ content (2).

### Preparation of total RNA libraries and mRNA sequencing

Two week-old Arabidopsis seedlings were treated with 0 and 200 mM NaCl and sampled at 1, 2 and 4 h, respectively. Total RNA from NaCl-exposed and NaCl free seedlings was isolated using the TRIzol Reagent (Invitrogen, USA) and pooled for RNA sequencing. The extracted RNA was treated with DNaseI (Qiagen, USA) at 25 °C for 30 min and confirm in quality. mRNA was purified with oligo (dT)-rich magnetic beads and broken into short fragments. The first and second strand cDNAs were synthesized. The cDNAs were end-repaired and phosphorylated using T4 DNA polymerase and Klenow DNA polymerase. The Illumina paired-end solexa adaptors were ligated to these cDNA fragments. The ligated products were purified on a 2% agarose gel. Four libraries (Col-0-NaCl, *fc1*-NaCl, Col-0 + NaCl and *fc1* + NaCl) were sequenced using an Illumina hiseq. 2500 with paired-end of Solexa RNA.

The original image data generated by the sequence providers were transferred into nucleotide sequences data by base calling, defined as raw reads and saved as ‘fastq’ files. All subsequent analyses were performed on the high-quality clean read datasets according to the bioinformatics analysis approach summarized in Supplementary Data 1 ^50^. A rigorous algorithm was used to identify differentially expressed genes (DEGs) between the samples. The expression level for each transcript was calculated as FPKM (fragments per kilobase of exon per million fragments mapped)-derived read counts based on the number of uniquely mapped reads that overlapped with exonic regions^51^. FDR was used to determine the threshold of the *p*-value in multiple tests, which corresponded to the differential gene expression test. In this study, FDR ≤ 0.001 and the absolute value of Log_2_Ratio > 1 were used as a threshold to judge the significant differences of gene expression.

### Gene Ontology analysis

The Gene Ontology (GO) category of the DEGs with functional significance was subject to the ultra-geometric test with Benjamini-Hochberg correction (http://www.geneontology.org/). GO terms with corrected *p*-value < 0.05 were regarded as significant enrichment for the DEGs compared to the genome background.

### Statistical analysis

Experiments in the study were independently performed in triplicate. Each result shown in the figures was the mean of three replicated treatments, and each treatment contained at least 15–20 seedlings. Samples for analysis were randomly selected from all transgenic lines. The significant differences between treatments were statistically evaluated by standard deviation and ANOVA methods (*p < *0.05).

## Electronic supplementary material

Supplementary Data
