# Supplementary material for: Salt stress-induced FERROCHELATASE 1 improves resistance to salt stress by limiting sodium accumulation in Arabidopsis thaliana
Source: Sci Rep. 2017 Nov 7;7:14737. doi: 10.1038/s41598-017-13593-9 (PMC5676718; doi:10.1038/s41598-017-13593-9)
Supplement: Supplementary file 1 — Supplementary Data [file 41598_2017_13593_MOESM1_ESM.pdf]

Supplementary Data

Title of article: Salt stress-induced *FERROCHELATASE 1* improves resistance to salt stress by limiting sodium accumulation in *Arabidopsis thaliana*

Running head: *AtFC1* mediation of plant resistance to salt stress

Authors: [1, 3*]Wen Ting Zhao, [1*]Sheng Jun Feng, [2]Hua Li, [3]Franziska Faust, [4]Tatjana Kleine, [1]Long Na Li, [1]Zhi Min Yang

Institutes: [1]Department of Biochemistry and Molecular Biology, College of Life Science, Nanjing Agricultural University, Nanjing 210095, China; [2]Department of Plant Science, College of Life Science, Henan Agricultural University, Henan 450002, China; [3]Institute of Plant Nutrition (IFZ), Justus Liebig University, Heinrich-Buff-Ring 26-32, 35392 Giessen, Germany; [4]Plant Molecular Biology (Botany), Department Biology I, Ludwig-Maximilians-University Munich, 82152 Martinsried, Germany.

Supplementary Data 1 Output data of RNA-seq from four rice libraries exposed to -NaCl and +NaCl.

Sample	Col-0(-NaCl)	*fc1*(-NaCl)	Col-0(+NaCl)	*fc1*(+NaCl)
Raw Reads Number	35084424	33540540	33705860	32590504
Raw Reads Length (bp)	150	150	150	150
Clean Reads Number	31643626	30471310	30741988	29950042
Clean Reads Length (bp)	150	150	150	150
Clean Reads Rate (%)	90.19	90.85	91.21	91.9
Low-quality Reads	3021236	2733958	2579344	2362418
Low-quality Reads Rate	8.61	8.15	7.65	7.25

Supplementary Data S2A Up regulated genes in fc1/Col-0 without NaCl treatment.

Gene	Log2FoldChange	pval	NT:Description
AT5G04140	1.389344288	0	Arabidopsis thaliana ferredoxin-dependent glutamate synthase 1 mRNA, complete cds
AT4G28080	2.607621419	0	Arabidopsis thaliana tetratricopeptide repeat domain protein mRNA, complete cds
AT1G01320	2.351735702	0	Arabidopsis thaliana tetratricopeptide repeat-containing protein mRNA, complete cds
AT5G40450	3.202087392	0	Arabidopsis thaliana uncharacterized protein mRNA, complete cds
AT5G53460	1.677740747	0	Arabidopsis thaliana glutamate synthase 1 [NADH] mRNA, complete cds
AT1G70320	2.654025541	0	Arabidopsis thaliana ubiquitin-protein ligase 2 mRNA, complete cds
AT2G28290	3.603087395	0	Arabidopsis thaliana chromatin structure-remodeling complex protein SYD mRNA, complete cds
AT3G02260	2.891386553	0	Arabidopsis thaliana auxin transport protein BIG mRNA, complete cds
AT1G15290	1.886368092	0	Arabidopsis thaliana tetratricopeptide repeat-containing protein mRNA, complete cds
AT1G55490	1.036839121	0	Arabidopsis thaliana chaperonin 60 subunit beta 1 mRNA, complete cds
AT1G28290	1.997791239	0	Arabidopsis thaliana arabinogalactan protein 31 mRNA, complete cds
AT4G29060	1.187031079	0	Arabidopsis thaliana elongation factor Ts family protein mRNA, complete cds
AT1G80070	2.084031631	0	Arabidopsis thaliana putative splicing factor Prp8 mRNA, complete cds
AT1G55860	2.937866497	0	Arabidopsis thaliana ubiquitin-protein ligase 1 mRNA, complete cds
AT5G41790	2.663942024	0	Arabidopsis thaliana COP1-interactive protein 1 mRNA, complete cds
AT4G25100	1.75884258	0	Arabidopsis thaliana superoxide dismutase [Fe] mRNA, complete cds
AT1G79280	2.719902076	0	Arabidopsis thaliana nuclear pore anchor mRNA, complete cds
AT1G36160	1.862679922	0	Arabidopsis thaliana acetyl-CoA carboxylase 1 mRNA, complete cds

AT4G17330	2.089028687	0	Arabidopsis thaliana G2484-1 protein mRNA, complete cds
AT1G48090	2.479172821	0	Arabidopsis thaliana calcium-dependent lipid-binding family protein mRNA, complete cds
AT3G22380	1.423424077	0	Arabidopsis thaliana clock regulator protein TIME FOR COFFEE mRNA, complete cds
AT2G46020	2.335527479	0	Arabidopsis thaliana ATP-dependent helicase BRAHMA mRNA, complete cds
AT3G41979	5.193100928	7.42E-302	Erysimum belvederense internal transcribed spacer 1, partial sequence; 5.8S ribosomal RNA gene, complete sequence; and internal transcribed spacer 2, partial sequence
AT4G02510	1.228947199	1.49E-296	Arabidopsis thaliana translocase of chloroplast 159 mRNA, complete cds
AT3G50370	1.77331863	1.54E-288	Arabidopsis thaliana uncharacterized protein mRNA, complete cds
AT4G01800	1.291762257	2.57E-288	Arabidopsis thaliana protein translocase subunit secA mRNA, complete cds
AT2G17930	2.428778749	4.16E-285	Arabidopsis thaliana phosphatidylinositol 3- and 4-kinase family protein with FAT domain mRNA, complete cds
AT5G44800	2.708577084	9.57E-281	Arabidopsis thaliana chromatin remodeling 4 protein mRNA, complete cds
AT1G64790	1.985985446	5.18E-271	Arabidopsis thaliana protein ILITHYIA mRNA, complete cds
AT1G20960	2.029056128	4.88E-270	Arabidopsis thaliana putative U5 small nuclear ribonucleoprotein helicase mRNA, complete cds
AT1G02080	1.736004464	3.71E-269	Arabidopsis thaliana CCR4-NOT transcription complex subunit 1 domain protein mRNA, complete cds
AT4G24190	1.046901471	1.10E-266	Arabidopsis thaliana HSP90-like protein GRP94 mRNA, complete cds
AT4G35800	2.018523924	9.72E-254	Arabidopsis thaliana DNA-directed RNA polymerase II subunit RPB1 mRNA, complete cds
AT5G35210	1.570448969	2.65E-252	Arabidopsis thaliana PHD type transcription factor with transmembrane domain protein mRNA, complete cds
AT4G10120	1.503637078	1.52E-249	Arabidopsis thaliana probable

			sucrose-phosphate synthase 4 mRNA, complete cds
AT2G35630	1.833857639	4.24E-238	Arabidopsis thaliana protein MICROTUBULE ORGANIZATION 1 mRNA, complete cds
AT1G03080	2.394693862	2.19E-234	Arabidopsis thaliana kinase interacting (KIP1-like) protein mRNA, complete cds
AT1G03060	2.309363504	4.17E-219	Arabidopsis thaliana WD/BEACH domain protein SPIRRIG mRNA, complete cds
AT2G22125	1.569800055	1.94E-218	Arabidopsis thaliana cellulose synthase-interactive protein 1 mRNA, complete cds
AT5G47690	1.464955189	3.43E-216	Arabidopsis thaliana uncharacterized binding protein mRNA, complete cds
AT3G08530	1.029556825	7.73E-214	Arabidopsis thaliana Clathrin, heavy chain mRNA, complete cds
AT4G17140	2.230990331	2.05E-212	Arabidopsis thaliana pleckstrin homology (PH) domain-containing protein mRNA, complete cds
AT5G04560	2.42681465	5.90E-212	Arabidopsis thaliana transcriptional activator DEMETER mRNA, complete cds
AT1G70620	1.528900354	1.14E-207	Arabidopsis thaliana cyclin-related protein mRNA, complete cds
AT4G15180	2.567930988	4.87E-207	Arabidopsis thaliana putative histone-lysine N-methyltransferase ATXR3 mRNA, complete cds
AT1G65010	2.11222719	3.18E-206	Arabidopsis thaliana flower development-related protein mRNA, complete cds
AT4G38950	1.820737397	2.28E-198	Arabidopsis thaliana ATP binding microtubule motor family protein mRNA, complete cds
AT3G60240	1.713033932	8.77E-196	Arabidopsis thaliana eukaryotic translation initiation factor 4G mRNA, complete cds
AT1G21580	1.804929681	1.78E-195	Arabidopsis thaliana zinc finger CCCH domain-containing protein mRNA, complete cds
AT1G67120	3.40809547	2.72E-195	Arabidopsis thaliana protein MIDASIN1 mRNA, complete cds
AT5G23110	2.398233585	3.28E-192	Arabidopsis thaliana uncharacterized protein mRNA, complete cds

AT1G65440	1.979316768	3.68E-189	Arabidopsis thaliana transcription elongation factor SPT6-like protein mRNA, complete cds
AT1G55350	1.579907971	9.61E-187	Arabidopsis thaliana calpain-type cysteine protease DEK1 mRNA, complete cds
AT1G35115.1	3.314774245	2.39E-184	Arabidopsis thaliana chromosome 1 sequence
AT4G11420	1.164426717	3.94E-182	Arabidopsis thaliana eukaryotic translation initiation factor 3A mRNA, complete cds
AT4G36520	2.483016725	1.88E-181	Arabidopsis thaliana chaperone DnaJ-domain containing protein mRNA, complete cds
AT1G76810	1.424865367	8.98E-181	Arabidopsis thaliana eukaryotic translation initiation factor 2 (eIF-2) family protein mRNA, complete cds
AT4G38600	1.2550525	8.51E-175	Arabidopsis thaliana HECT ubiquitin protein ligase family protein mRNA, complete cds
AT2G35050	1.261779182	1.53E-174	Arabidopsis thaliana protein kinase family protein mRNA, complete cds
AT5G13000	1.239403148	6.05E-174	Arabidopsis thaliana callose synthase 3 mRNA, complete cds
AT3G19050	3.61775215	5.47E-173	Arabidopsis thaliana phragmoplast orienting kinesin 2 mRNA, complete cds
AT3G22790	2.943427203	2.22E-171	Arabidopsis thaliana protein NETWORKED 1A mRNA, complete cds
AT3G48870	1.219402132	1.55E-170	Arabidopsis thaliana Clp ATPase mRNA, complete cds
AT1G26150	1.524127012	4.88E-170	Arabidopsis thaliana proline-rich extensin-like receptor kinase 10 mRNA, complete cds
AT1G67230	1.599328649	7.49E-170	Arabidopsis thaliana protein little nuclei1 mRNA, complete cds
AT4G31570	2.745254663	1.83E-169	Arabidopsis thaliana AUCSIA-1 interacting protein mRNA, complete cds
AT4G14760	2.717204393	1.65E-163	Arabidopsis thaliana kinase interacting-like protein mRNA, complete cds
AT1G79000	1.9424249	5.80E-163	Arabidopsis thaliana histone acetyltransferase HAC1 mRNA, complete cds
AT2G36910	1.431160939	8.00E-163	Arabidopsis thaliana ABC transporter B

			family member 1 mRNA, complete cds
AT3G52140	1.364891735	2.69E-161	Arabidopsis thaliana tetratricopeptide repeat (TPR)-containing protein mRNA, complete cds
AT3G16000	1.357564103	3.43E-159	Arabidopsis thaliana MAR-binding filament-like protein 1 mRNA, complete cds
AT5G20490	1.470773634	7.41E-153	Arabidopsis thaliana Myosin XI K mRNA, complete cds
AT1G50030	1.731194165	8.44E-152	Arabidopsis thaliana serine/threonine-protein kinase TOR mRNA, complete cds
AT4G00450	1.973509116	4.94E-151	Arabidopsis thaliana transcriptional regulator MED12-like protein mRNA, complete cds
AT1G21630	1.578626618	9.51E-149	Arabidopsis thaliana calcium-binding EF-hand-containing protein mRNA, complete cds
AT2G45540	1.377877952	1.36E-144	Arabidopsis thaliana beige-related and WD-40 repeat-containing protein mRNA, complete cds
AT3G23890	2.119925558	1.14E-143	Arabidopsis thaliana DNA topoisomerase 2 mRNA, complete cds
AT2G32240	1.231695789	1.27E-142	Arabidopsis thaliana uncharacterized protein mRNA, complete cds
AT1G58250	1.974939239	4.45E-139	Arabidopsis thaliana protein SABRE mRNA, complete cds
AT2G13370	1.810665861	9.18E-139	Arabidopsis thaliana chromatin remodeling 5 mRNA, complete cds
AT1G32750	2.115521917	1.38E-138	Arabidopsis thaliana histone acetyltransferase of the CBP family 13 mRNA, complete cds
AT4G36080	2.861492003	1.95E-138	Arabidopsis thaliana phosphotransferases/inositol or phosphatidylinositol kinases mRNA, complete cds
AT3G04340	1.301535468	1.32E-134	Arabidopsis thaliana protein EMBRYO DEFECTIVE 2458 mRNA, complete cds
AT1G23720	1.452675283	2.46E-134	Arabidopsis thaliana chromosome 1 sequence
AT4G18240	1.443881313	9.55E-134	Arabidopsis thaliana starch synthase 4 mRNA, complete cds
AT1G20970	1.69666033	3.37E-131	Arabidopsis thaliana uncharacterized protein mRNA, complete cds

AT2G31960	1.336596432	2.35E-130	Arabidopsis thaliana glucan synthase-like 3 mRNA, complete cds
AT2G43150	1.086239536	3.78E-130	Arabidopsis thaliana Proline-rich extensin-like family protein mRNA, complete cds
AT4G26630	1.122401617	1.73E-128	Arabidopsis thaliana DEK domain-containing chromatin associated protein mRNA, complete cds
AT2G42270	1.768886631	5.41E-128	Arabidopsis thaliana U5 small nuclear ribonucleoprotein helicase mRNA, complete cds
AT2G25170	1.569784179	2.32E-127	Arabidopsis thaliana CHD3-type chromatin-remodeling factor PICKLE mRNA, complete cds
AT1G77300	2.218581148	2.66E-127	Arabidopsis thaliana histone-lysine N-methyltransferase ASHH2 mRNA, complete cds
AT1G22060	2.194335433	3.94E-125	Arabidopsis thaliana uncharacterized protein mRNA, complete cds
AT2G34680	1.229832243	1.37E-124	Arabidopsis thaliana protein AUXIN-INDUCED IN ROOT CULTURES 9 mRNA, complete cds
AT3G22120	1.854190633	3.21E-124	Arabidopsis thaliana chromosome 3, complete sequence
AT1G10760	1.501611325	4.13E-124	Arabidopsis thaliana alpha-glucan water dikinase 1 mRNA, complete cds
AT3G43300	1.218319491	4.24E-123	Arabidopsis thaliana brefeldin A-inhibited guanine nucleotide-exchange protein 5 mRNA, complete cds
AT5G48570	1.355698928	2.94E-121	Arabidopsis thaliana peptidyl-prolyl cis-trans isomerase FKBP65 mRNA, complete cds
AT1G68890	1.523542687	8.06E-121	Arabidopsis thaliana protein PHYLLO mRNA, complete cds
AT2G26890	1.552456343	4.45E-120	Arabidopsis thaliana gravitropism defective 2 mRNA, complete cds
AT2G36850	1.159337144	1.35E-119	Arabidopsis thaliana glucan synthase-like 8 mRNA, complete cds
AT5G13010	1.452358799	2.74E-119	Arabidopsis thaliana RNA helicase family protein mRNA, complete cds
AT1G68790	1.980179544	5.73E-119	Arabidopsis thaliana little nuclei3 protein mRNA, complete cds
AT1G79830	1.173213996	4.27E-117	Arabidopsis thaliana golgin candidate 5 mRNA, complete cds

AT5G61140	1.647986244	6.12E-117	Arabidopsis thaliana U5 small nuclear ribonucleoprotein helicase mRNA, complete cds
AT4G01290	1.221179119	8.30E-117	Arabidopsis thaliana uncharacterized protein mRNA, complete cds
AT1G76930	2.153572564	6.19E-116	Arabidopsis thaliana extensin 4 mRNA, complete cds
AT1G24706	1.959194519	1.14E-115	Arabidopsis thaliana THO complex subunit 2 mRNA, complete cds
AT3G54670	1.965685417	4.01E-115	Arabidopsis thaliana structural maintenance of chromosomes 1 mRNA, complete cds
AT2G39580	1.29068529	5.27E-115	Arabidopsis thaliana uncharacterized protein mRNA, complete cds
AT2G16485	2.027340153	6.59E-114	Arabidopsis thaliana GW repeat- and PHD finger-containing protein NERD mRNA, complete cds
AT3G57300	1.426180763	2.39E-111	Arabidopsis thaliana DNA helicase INO80 complex-like 1 mRNA, complete cds
AT1G17360	1.337104518	3.95E-109	Arabidopsis thaliana uncharacterized protein mRNA, complete cds
AT2G03150	1.579049292	5.41E-109	Arabidopsis thaliana protein EMBRYO DEFECTIVE 1579 mRNA, complete cds
AT1G24460	1.8903701	1.72E-108	Arabidopsis thaliana TGN-localized SYP41 interacting protein mRNA, complete cds
AT4G39420	1.901236341	6.50E-107	Arabidopsis thaliana uncharacterized protein mRNA, complete cds
AT5G23150	2.125271795	1.31E-105	Arabidopsis thaliana ENHANCER OF AG-4 protein 2 mRNA, complete cds
AT3G11964	1.766549441	1.69E-105	Arabidopsis thaliana protein ribosomal RNA processing 5 mRNA, complete cds
AT3G01370	1.332661061	3.14E-105	Arabidopsis thaliana CRM family member 2 mRNA, complete cds
AT1G28420	2.025636662	3.43E-105	Arabidopsis thaliana homeobox-1 mRNA, complete cds
AT3G54580	1.434790337	7.77E-104	Arabidopsis thaliana chromosome 3, complete sequence
AT5G52640	1.032017725	1.31E-103	Arabidopsis thaliana heat shock protein 90.1 mRNA, complete cds
AT3G47910	1.723700024	1.69E-103	Arabidopsis thaliana ubiquitin carboxyl-terminal hydrolase-related protein mRNA, complete cds

AT4G24680	1.485171479	3.12E-103	Arabidopsis thaliana protein MODIFIER OF SNC1 1 mRNA, complete cds
AT5G55660	1.412830279	8.00E-102	Arabidopsis thaliana DEK domain-containing chromatin associated protein mRNA, complete cds
AT1G20390.1	3.976099557	2.65E-101	Arabidopsis thaliana chromosome 1 sequence
AT3G14172	1.920031463	6.67E-101	Arabidopsis thaliana uncharacterized protein mRNA, complete cds
AT2G46560	1.669544884	1.58E-100	Arabidopsis thaliana transducin family protein / WD-40 repeat family protein mRNA, complete cds
AT4G18600	3.152266367	2.29E-100	Arabidopsis thaliana SCAR family protein WAVE5 mRNA, complete cds
AT4G16870.1	2.290374559	8.81E-100	Arabidopsis thaliana chromosome 4 sequence
AT1G09770	1.021610263	1.55E-99	Arabidopsis thaliana cell division cycle 5-like protein mRNA, complete cds
AT4G16660	1.116397334	1.85E-98	Arabidopsis thaliana heat shock protein 70 mRNA, complete cds
AT4G39050	1.343368768	2.53E-98	Arabidopsis thaliana Kinesin motor family protein mRNA, complete cds
AT1G09750	1.004470657	5.38E-98	Arabidopsis thaliana aspartyl protease-like protein mRNA, complete cds
AT3G01320	2.036430378	8.22E-97	Arabidopsis thaliana paired amphipathic helix protein Sin3-like 1 mRNA, complete cds
AT5G58040	1.50104678	4.25E-96	Arabidopsis thaliana FIP1 [V]-like protein mRNA, complete cds
AT1G19835	1.26045509	3.37E-95	Arabidopsis thaliana uncharacterized protein mRNA, complete cds
AT1G70060	1.902610991	1.36E-94	Arabidopsis thaliana paired amphipathic helix protein Sin3-like 4 mRNA, complete cds
AT2G38440	1.488519211	1.64E-94	Arabidopsis thaliana WAVE complex SCAR2 mRNA, complete cds
AT5G47820	1.342042356	2.01E-94	Arabidopsis thaliana kinesin-like protein FRA1 mRNA, complete cds
AT5G10470	1.166950412	3.89E-94	Arabidopsis thaliana kinesin CDKA;1 associated 1 mRNA, complete cds
AT3G17360	3.180646154	8.55E-94	Arabidopsis thaliana phragmoplast orienting kinesin 1 mRNA, complete cds
AT3G28550	1.559900023	9.21E-94	Arabidopsis thaliana chromosome 3,

			complete sequence
AT5G67470	1.948752772	1.83E-93	Arabidopsis thaliana formin-like protein 6 mRNA, complete cds
AT1G72410	1.266688982	9.17E-93	Arabidopsis thaliana COP1-interacting protein-related protein mRNA, complete cds
AT2G25660	1.60392924	2.94E-92	Arabidopsis thaliana embryo defective 2410 protein mRNA, complete cds
AT3G06400	1.043531875	5.35E-92	Arabidopsis thaliana chromatin-remodeling protein 11 mRNA, complete cds
AT3G50240	1.834102213	1.54E-91	Arabidopsis thaliana kinesin-related protein mRNA, complete cds
AT3G54590	1.74446876	2.46E-91	Arabidopsis thaliana hydroxyproline-rich glycoprotein mRNA, complete cds
AT5G46070	1.788575747	4.57E-90	Arabidopsis thaliana Guanylate-binding protein mRNA, complete cds
AT1G15940	2.106055051	6.79E-90	Arabidopsis thaliana aspartyl beta-hydroxylase N-terminal region domain-containing protein mRNA, complete cds
AT3G48190	1.724996952	1.36E-89	Arabidopsis thaliana serine/threonine-protein kinase ATM mRNA, complete cds
AT1G15780	1.809806008	4.47E-89	Arabidopsis thaliana uncharacterized protein mRNA, complete cds
AT1G67140	1.522800022	1.58E-88	Arabidopsis thaliana protein SWEETIE mRNA, complete cds
AT3G01460	2.166493791	2.41E-88	Arabidopsis thaliana methyl-CPG-binding domain 9 mRNA, complete cds
AT3G50380	1.571386326	2.61E-88	Arabidopsis thaliana uncharacterized protein mRNA, complete cds
AT2G25730	1.314895655	3.25E-88	Arabidopsis thaliana uncharacterized protein mRNA, complete cds
AT5G07740	1.598087768	5.17E-88	Arabidopsis thaliana chromosome 5 sequence
AT4G00800	1.41583627	5.87E-88	Arabidopsis thaliana transducin family protein / WD-40 repeat family protein mRNA, complete cds
AT3G51740	1.246500623	8.26E-88	Arabidopsis thaliana probably inactive leucine-rich repeat receptor-like protein kinase IMK2 mRNA, complete cds
AT4G26190	1.60389543	1.94E-87	Arabidopsis thaliana Haloacid

			dehalogenase-like hydrolase (HAD) superfamily protein mRNA, complete cds
AT5G42950	1.124809096	5.06E-87	Arabidopsis thaliana GYF domain-containing protein mRNA, complete cds
AT3G13300	1.035169622	6.24E-87	Arabidopsis thaliana mRNA decapping complex VCS mRNA, complete cds
AT3G62010	1.193929533	8.48E-86	Arabidopsis thaliana uncharacterized protein mRNA, complete cds
AT1G13220	2.30631342	2.27E-85	Arabidopsis thaliana protein CROWDED NUCLEI 2 mRNA, complete cds
AT5G44180	1.466839385	2.62E-85	Arabidopsis thaliana protein RINGLET2 mRNA, complete cds
AT5G43900	1.226949713	2.79E-85	Arabidopsis thaliana myosin 2 mRNA, complete cds
AT5G63420	1.353119402	1.69E-84	Arabidopsis thaliana RNase J mRNA, complete cds
AT1G77800	1.267646632	2.17E-84	Arabidopsis thaliana PHD finger-containing protein mRNA, complete cds
AT1G58230	1.636773841	1.51E-83	Arabidopsis thaliana WD40 and Beach domain-containing protein mRNA, complete cds
AT1G02730	1.308897598	1.58E-83	Arabidopsis thaliana cellulose synthase-like protein D5 mRNA, complete cds
AT3G54280	1.600349929	2.62E-83	Arabidopsis thaliana TATA-binding protein-associated factor BTAF1 mRNA, complete cds
AT2G41960	1.747669847	3.53E-83	Arabidopsis thaliana uncharacterized protein mRNA, complete cds
AT5G60930	2.40251314	1.18E-82	Arabidopsis thaliana P-loop containing nucleoside triphosphate hydrolases superfamily protein mRNA, complete cds
AT4G30100	1.248424705	1.32E-82	Arabidopsis thaliana P-loop containing nucleoside triphosphate hydrolases superfamily protein mRNA, complete cds
AT2G26330	1.080851021	2.89E-82	Arabidopsis thaliana LRR receptor-like serine/threonine-protein kinase ERECTA mRNA, complete cds
AT2G47800	1.142515762	3.94E-82	Arabidopsis thaliana ABC transporter C

			family member 4 mRNA, complete cds
AT1G24300	1.658685408	1.23E-80	Arabidopsis thaliana GYF domain-containing protein mRNA, complete cds
AT3G12810	2.143395724	2.10E-80	Arabidopsis thaliana photoperiod-independent early flowering 1 protein mRNA, complete cds
AT3G33530	1.20816135	2.72E-80	Arabidopsis thaliana transducin/WD40 domain-containing protein mRNA, complete cds
AT3G06290	1.684239088	3.65E-80	Arabidopsis thaliana SAC3/GANP/Nin1/mts3/eIF-3 p25 family protein mRNA, complete cds
AT1G06670	1.644991308	1.82E-79	Arabidopsis thaliana nuclear DEIH-boxhelicase mRNA, complete cds
AT1G61210	1.65883284	3.73E-79	Arabidopsis thaliana protein DWD hypersensitive to ABA 3 mRNA, complete cds
AT2G20190	1.054478149	1.97E-78	Arabidopsis thaliana CLIP-associated protein mRNA, complete cds
AT5G43310	1.683421001	3.01E-78	Arabidopsis thaliana COP1-interacting protein-like protein mRNA, complete cds
AT3G12980	1.85427108	8.77E-78	Arabidopsis thaliana histone acetyltransferase HAC5 mRNA, complete cds
AT3G60860	1.441513763	5.10E-77	Arabidopsis thaliana SEC7-like guanine nucleotide exchange family protein mRNA, complete cds
AT5G51200	1.293654632	8.54E-77	Arabidopsis thaliana uncharacterized protein mRNA, complete cds
AT4G27430	1.155794885	8.07E-76	Arabidopsis thaliana COP1-interacting protein 7 mRNA, complete cds
AT4G30990	1.788755646	8.53E-76	Arabidopsis thaliana down-regulated in metastasis (DRIM) domain-containing protein mRNA, complete cds
AT5G24740	1.553613865	2.84E-75	Arabidopsis thaliana uncharacterized protein mRNA, complete cds
AT1G49340	1.168386861	2.94E-75	Arabidopsis thaliana phosphatidylinositol 4-kinase alpha mRNA, complete cds
AT4G16340	1.077113024	4.68E-75	Arabidopsis thaliana DOCK family guanine nucleotide exchange factor SPIKE1 mRNA, complete cds
AT2G48160	1.292640795	4.79E-75	Arabidopsis thaliana Tudor/PWWP/MBT domain-containing protein mRNA,

			complete cds
AT3G07770	1.043815978	6.19E-75	Arabidopsis thaliana HEAT SHOCK PROTEIN 89.1 mRNA, complete cds
AT1G32490	1.543839	1.59E-74	Arabidopsis thaliana DEAH RNA helicase homolog PRP2 mRNA, complete cds
AT3G57470	1.018078979	1.86E-74	Arabidopsis thaliana insulinase family protein mRNA, complete cds
AT5G41140	1.878386844	3.39E-74	Arabidopsis thaliana Myosin heavy chain-related protein mRNA, complete cds
AT4G22505	4.175734214	6.56E-74	Arabidopsis thaliana chromosome 4 sequence
AT2G03140	1.749202197	6.77E-74	Arabidopsis thaliana alpha/beta-Hydrolases superfamily protein mRNA, complete cds
AT2G47410	1.56504848	7.49E-74	Arabidopsis thaliana WD40 domain-containing protein mRNA, complete cds
AT2G36490	1.516090547	7.53E-74	Arabidopsis thaliana protein ROS1 mRNA, complete cds
AT1G19720	1.186447608	1.13E-73	Arabidopsis thaliana pentatricopeptide repeat-containing protein mRNA, complete cds
AT2G43800	1.931624391	4.55E-73	Arabidopsis thaliana formin-like protein 2 mRNA, complete cds
AT1G19715	1.136308734	1.43E-72	Arabidopsis thaliana Mannose-binding lectin superfamily protein mRNA, complete cds
AT5G56890	1.473099354	1.61E-72	Arabidopsis thaliana protein kinase family protein mRNA, complete cds
AT4G33240	1.313887463	1.84E-72	Arabidopsis thaliana 1-phosphatidylinositol-3-phosphate 5-kinase FAB1A mRNA, complete cds
AT1G56660	1.877500158	2.24E-72	Arabidopsis thaliana uncharacterized protein mRNA, complete cds
AT5G18620	1.457718727	9.15E-72	Arabidopsis thaliana chromatin remodeling factor17 mRNA, complete cds
AT1G71220	1.017092903	9.90E-72	Arabidopsis thaliana UDP-glucose:glycoprotein glucosyltransferase mRNA, complete cds
AT5G40480	1.50176828	5.56E-71	Arabidopsis thaliana protein EMBRYO DEFECTIVE 3012 mRNA, complete cds

AT2G31970	1.979694971	2.39E-70	Arabidopsis thaliana DNA repair protein RAD50 mRNA, complete cds
AT5G48600	2.180733022	3.34E-69	Arabidopsis thaliana structural maintenance of chromosomes protein 4 mRNA, complete cds
AT4G28710	1.419589066	5.69E-69	Arabidopsis thaliana myosin XI H mRNA, complete cds
AT3G14270	1.401379945	1.20E-68	Arabidopsis thaliana phosphatidylinositol-3P 5-kinase-like mRNA, complete cds
AT1G75310	2.31684677	4.66E-68	Arabidopsis thaliana auxin-like 1 protein mRNA, complete cds
AT4G31880	1.236621782	1.97E-67	Arabidopsis thaliana uncharacterized protein mRNA, complete cds
AT1G24190	1.506228922	2.16E-67	Arabidopsis thaliana paired amphipathic helix protein Sin3-like 3 mRNA, complete cds
AT5G40340	1.567485593	3.27E-67	Arabidopsis thaliana PWWP domain-containing protein mRNA, complete cds
AT3G63070	1.476515773	3.65E-67	Arabidopsis thaliana Tudor/PWWP/MBT domain-containing protein mRNA, complete cds
AT3G06880	1.604612913	4.26E-67	Arabidopsis thaliana transducin/WD-40 repeat-containing protein mRNA, complete cds
AT4G31160	1.607796782	5.76E-67	Arabidopsis thaliana DDB1- and CUL4-associated factor-1 mRNA, complete cds
AT3G13330	1.243818531	6.68E-67	Arabidopsis thaliana proteasome activating protein 200 mRNA, complete cds
AT4G25290	1.117456603	8.68E-67	Arabidopsis thaliana DNA photolyase mRNA, complete cds
AT1G78580	1.155469243	1.12E-65	Arabidopsis thaliana alpha,alpha-trehalose-phosphate synthase [UDP-forming] 1 mRNA, complete cds
AT5G55820	2.786424076	1.69E-65	Arabidopsis thaliana uncharacterized protein mRNA, complete cds
AT3G27260	1.166750487	2.08E-65	Arabidopsis thaliana global transcription factor group E8 mRNA, complete cds
AT3G10650	1.098576804	2.23E-65	Arabidopsis thaliana nucleoporin NUP1 mRNA, complete cds
AT3G24870	2.068683667	1.23E-64	Arabidopsis thaliana

			Helicase/SANT-associated, DNA binding protein mRNA, complete cds
AT2G34640	1.117108672	1.41E-64	Arabidopsis thaliana plastid transcriptionally active 12 mRNA, complete cds
AT3G29320	1.29830431	3.34E-64	Arabidopsis thaliana alpha-glucan phosphorylase 1 mRNA, complete cds
AT3G05680	1.118772595	4.81E-64	Arabidopsis thaliana embryo defective 2016 protein mRNA, complete cds
AT5G42540	1.012228715	8.46E-64	Arabidopsis thaliana 5'-3' exoribonuclease 2 mRNA, complete cds
AT5G55300	1.020306033	9.38E-64	Arabidopsis thaliana DNA topoisomerase 1 mRNA, complete cds
AT2G19110	1.165969743	1.22E-63	Arabidopsis thaliana putative cadmium/zinc-transporting ATPase HMA4 mRNA, complete cds
AT5G18700	2.323120341	1.28E-63	Arabidopsis thaliana protein kinase family protein with ARM repeat domain mRNA, complete cds
AT1G08600	1.743350343	1.35E-63	Arabidopsis thaliana protein ATRX mRNA, complete cds
AT3G59930	1.778352888	1.38E-63	Arabidopsis thaliana defensin-like protein 206 mRNA, complete cds
AT1G17580	1.389532064	1.42E-63	Arabidopsis thaliana myosin 1 mRNA, complete cds
AT2G37080	1.295684213	1.72E-63	Arabidopsis thaliana protein ROP interactive partner 2 mRNA, complete cds
AT3G62900	1.536939174	3.33E-63	Arabidopsis thaliana CW-type zinc-finger protein mRNA, complete cds
AT2G21300	1.261958069	3.62E-62	Arabidopsis thaliana ATP binding microtubule motor family protein mRNA, complete cds
AT1G74160	1.495479697	5.43E-62	Arabidopsis thaliana uncharacterized protein mRNA, complete cds
AT5G16210	1.273385941	5.83E-62	Arabidopsis thaliana HEAT repeat-containing protein mRNA, complete cds
AT5G02310	1.144746972	1.80E-61	Arabidopsis thaliana proteolysis 6 mRNA, complete cds
AT5G16780	1.253663263	2.49E-61	Arabidopsis thaliana SART-1 family protein DOT2 mRNA, complete cds
AT1G18370	1.918552534	3.60E-61	Arabidopsis thaliana kinesin HINKEL mRNA, complete cds

AT5G24350	1.427521104	5.12E-61	Arabidopsis thaliana uncharacterized protein mRNA, complete cds
AT3G47890	1.485174688	5.93E-61	Arabidopsis thaliana ubiquitin carboxyl-terminal hydrolase-related protein mRNA, complete cds
AT3G57660	1.697958611	7.78E-61	Arabidopsis thaliana nuclear RNA polymerase A1 mRNA, complete cds
AT1G17110	1.300325641	8.97E-61	Arabidopsis thaliana ubiquitin carboxyl-terminal hydrolase 15 mRNA, complete cds
AT1G08060	1.982781339	1.23E-60	Arabidopsis thaliana helicase protein MOM1 mRNA, complete cds
AT4G33200	1.314760211	1.94E-60	Arabidopsis thaliana myosin-15 mRNA, complete cds
AT5G15680	1.386468818	2.26E-60	Arabidopsis thaliana cell morphogenesis domain-containing protein mRNA, complete cds
AT1G79560	1.009233603	3.45E-60	Arabidopsis thaliana AT1G79560 mRNA, complete cds, clone: RAFL07-08-E14
AT4G32420	1.299986344	4.67E-60	Arabidopsis thaliana cyclophilin-like peptidyl-prolyl cis-trans isomerase family protein mRNA, complete cds
AT1G79350	1.300997249	6.76E-60	Arabidopsis thaliana protein EMBRYO DEFECTIVE 1135 mRNA, complete cds
AT1G03910	1.26485833	7.70E-60	Arabidopsis thaliana uncharacterized protein mRNA, complete cds
AT1G31810	2.100598099	7.97E-60	Arabidopsis thaliana formin-like protein 14 mRNA, complete cds
AT4G38760	1.128360191	9.75E-60	Arabidopsis thaliana uncharacterized protein mRNA, complete cds
AT3G54760	1.107267048	4.03E-59	Arabidopsis thaliana dentin sialophosphoprotein-related protein mRNA, complete cds
AT4G04350	1.114861901	4.94E-59	Arabidopsis thaliana tRNA synthetase class I (I, L, M and V) family protein mRNA, complete cds
AT4G23800	1.104888051	5.19E-59	Arabidopsis thaliana HMG (high mobility group) box protein mRNA, complete cds
AT3G45850	1.239441326	1.29E-58	Arabidopsis thaliana P-loop containing nucleoside triphosphate hydrolases superfamily protein mRNA, complete cds

AT1G13980	1.17138676	3.22E-58	Arabidopsis thaliana ARF guanine-nucleotide exchange factor GNOM mRNA, complete cds
AT5G12400	1.448829502	6.36E-58	Arabidopsis thaliana PHD-finger and DNA binding domain-containing protein mRNA, complete cds
AT2G39340	1.079468106	9.43E-58	Arabidopsis thaliana SAC3/GANP/Nin1/mts3/eIF-3 p25-family protein mRNA, complete cds
AT5G61190	1.483590398	2.10E-57	Arabidopsis thaliana putative endonuclease or glycosyl hydrolase with C2H2-type zinc finger domain mRNA, complete cds
AT5G64170	1.199120101	2.98E-57	Arabidopsis thaliana dentin sialophosphoprotein-like protein mRNA, complete cds
AT1G06720	1.545691728	3.38E-57	Arabidopsis thaliana BMS1 domain-containing protein mRNA, complete cds
AT4G27595	2.344265416	5.68E-57	Arabidopsis thaliana uncharacterized protein mRNA, complete cds
AT1G45160	1.266872128	7.03E-57	Arabidopsis thaliana protein kinase mRNA, complete cds
AT5G47480	1.015976289	7.96E-57	Arabidopsis thaliana RGPR-related protein mRNA, complete cds
AT5G15540	1.52217449	1.07E-56	Arabidopsis thaliana sister-chromatid cohesion protein 2 mRNA, complete cds
AT5G01400	1.441426793	1.11E-56	Arabidopsis thaliana Symplekin/Pta1-like protein mRNA, complete cds
AT4G18670	1.354354692	1.79E-56	Arabidopsis thaliana chromosome 4 sequence
AT1G48650	1.090771777	5.93E-56	Arabidopsis thaliana DEA(D/H)-box RNA helicase family protein mRNA, complete cds
AT2G27170	1.353252905	6.50E-56	Arabidopsis thaliana structural maintenance of chromosome 3 mRNA, complete cds
AT4G12780	1.374632889	1.03E-55	Arabidopsis thaliana auxilin-related protein 1 mRNA, complete cds
AT3G19190	1.341572773	1.68E-55	Arabidopsis thaliana protein autophagy 2 mRNA, complete cds
AT4G19020	1.524554883	1.68E-55	Arabidopsis thaliana chromomethylase 2 mRNA, complete cds
AT4G32620	1.468742201	1.82E-55	Arabidopsis thaliana Enhancer of

			polycomb-like transcription factor protein mRNA, complete cds
AT1G72250	2.034016093	2.15E-55	Arabidopsis thaliana Di-glucose binding protein with Kinesin motor domain mRNA, complete cds
AT3G10010	2.29193305	2.25E-55	Arabidopsis thaliana putative DNA glycosylase mRNA, complete cds
AT5G16730	1.109437155	6.04E-55	Arabidopsis thaliana uncharacterized protein mRNA, complete cds
AT3G52250	1.255323895	2.72E-54	Arabidopsis thaliana protein POWERDRESS mRNA, complete cds
AT2G39260	1.264855	2.98E-54	Arabidopsis thaliana regulator of nonsense transcripts UPF2 mRNA, complete cds
AT2G24120	1.022144599	7.04E-54	Arabidopsis thaliana DNA-directed RNA polymerase 3 mRNA, complete cds
AT4G19530	1.034158699	1.10E-53	Arabidopsis thaliana TIR-NBS-LRR class disease resistance protein mRNA, complete cds
AT1G47900	1.55153655	1.65E-53	Arabidopsis thaliana uncharacterized protein mRNA, complete cds
AT1G77460	1.846796694	1.78E-53	Arabidopsis thaliana armadillo/beta-catenin-like repeat and C2 domain-containing protein mRNA, complete cds
AT3G18110	1.506970998	2.31E-53	Arabidopsis thaliana pentatricopeptide repeat-containing protein mRNA, complete cds
AT3G12020	1.340125685	7.16E-53	Arabidopsis thaliana kinesin motor protein-like protein mRNA, complete cds
AT1G11720	1.139552094	7.85E-53	Arabidopsis thaliana starch synthase 3 mRNA, complete cds
AT5G65440	1.211675519	6.46E-52	Arabidopsis thaliana uncharacterized protein mRNA, complete cds
AT3G26560	1.193757546	6.55E-52	Arabidopsis thaliana probable pre-mRNA-splicing factor ATP-dependent RNA helicase mRNA, complete cds
AT2G35110	1.297940629	7.48E-52	Arabidopsis thaliana protein NAP1 mRNA, complete cds
AT3G58110	1.223183806	1.13E-51	Arabidopsis thaliana uncharacterized protein mRNA, complete cds
AT5G16680	1.466486915	1.35E-51	Arabidopsis thaliana RING/FYVE/PHD zinc finger-containing protein mRNA,

			complete cds
AT5G07940	1.444227759	1.73E-51	Arabidopsis thaliana uncharacterized protein mRNA, complete cds
AT5G02880	1.098766052	3.35E-51	Arabidopsis thaliana E3 ubiquitin-protein ligase UPL4 mRNA, complete cds
AT4G12400	1.359254917	6.70E-51	Arabidopsis thaliana carboxylate clamp-tetratricopeptide repeat protein mRNA, complete cds
AT2G28620	1.740005952	8.03E-51	Arabidopsis thaliana kinesin motor protein-related protein mRNA, complete cds
AT2G22610	2.324571399	1.00E-50	Arabidopsis thaliana Di-glucose binding protein with Kinesin motor domain mRNA, complete cds
AT5G43810	1.085475048	1.39E-50	Arabidopsis thaliana eIF2C family protein Argonaute10 mRNA, complete cds
AT1G08260	1.874925602	2.07E-50	Arabidopsis thaliana DNA polymerase epsilon catalytic subunit A mRNA, complete cds
AT2G48060	1.196055895	4.91E-50	Arabidopsis thaliana uncharacterized protein mRNA, complete cds
AT1G80020.1	1.289071367	5.92E-50	Arabidopsis thaliana chromosome 1 sequence
AT5G04020	1.92832658	1.01E-49	Arabidopsis thaliana calmodulin-binding protein mRNA, complete cds
AT5G55040	1.081819806	1.39E-49	Arabidopsis thaliana DNA-binding bromodomain-containing protein mRNA, complete cds
AT5G22450	1.176363834	2.02E-49	Arabidopsis thaliana uncharacterized protein mRNA, complete cds
AT3G24880	2.179172387	2.07E-49	Arabidopsis thaliana Helicase/SANT-associated, DNA binding protein mRNA, complete cds
AT4G31210	1.344371583	2.74E-49	Arabidopsis thaliana DNA topoisomerase, type IA, core mRNA, complete cds
AT1G55325	1.250232127	5.85E-49	Arabidopsis thaliana RNA polymerase II transcription mediator mRNA, complete cds
AT1G74260	1.047332516	1.23E-48	Arabidopsis thaliana phosphoribosylformylglycinamidine synthase mRNA, complete cds
AT1G44910	1.415694161	1.43E-48	Arabidopsis thaliana

			pre-mRNA-processing protein 40A mRNA, complete cds
AT1G16270	1.184709658	5.80E-48	Arabidopsis thaliana octicosapeptide/Phox/Bem1p domain-containing protein kinase mRNA, complete cds
AT3G13290	1.688827146	5.92E-48	Arabidopsis thaliana varicose-related protein mRNA, complete cds
AT3G24480	1.083795032	5.97E-48	Arabidopsis thaliana chromosome 3, complete sequence
AT5G17910	1.284362414	8.24E-48	Arabidopsis thaliana uncharacterized protein mRNA, complete cds
AT3G20150	1.709286838	8.59E-48	Arabidopsis thaliana Kinesin motor family protein mRNA, complete cds
AT2G22795	1.062133528	2.70E-47	Arabidopsis thaliana uncharacterized protein mRNA, complete cds
AT2G23740	1.725366006	2.93E-47	Arabidopsis thaliana histone-lysine N-methyltransferase SUVR5 mRNA, complete cds
AT1G27430	1.127446588	3.27E-47	Arabidopsis thaliana GYF domain-containing protein mRNA, complete cds
AT3G54460	1.187724374	4.78E-47	Arabidopsis thaliana SNF2, helicase and F-box domain-containing protein mRNA, complete cds
AT3G23670	2.18158694	5.85E-47	Arabidopsis thaliana kinesin-like protein KIN12B mRNA, complete cds
AT5G54280	1.201649505	9.09E-47	Arabidopsis thaliana myosin 2 mRNA, complete cds
AT1G23230	1.401613767	9.13E-47	Arabidopsis thaliana mediator of RNA polymerase II transcription subunit 23 mRNA, complete cds
AT5G39500	1.460641567	9.50E-47	Arabidopsis thaliana ARF guanine-nucleotide exchange factor GNL1 mRNA, complete cds
AT1G16710	1.314751147	2.47E-46	Arabidopsis thaliana histone acetyltransferase HAC12 mRNA, complete cds
AT4G00440	1.059326941	5.15E-46	Arabidopsis thaliana uncharacterized protein mRNA, complete cds
AT5G27970	1.053946414	9.90E-46	Arabidopsis thaliana ARM repeat superfamily protein mRNA, complete cds
AT5G25060	1.061981837	1.31E-45	Arabidopsis thaliana RNA recognition

			motif-containing protein RRC1 mRNA, complete cds
AT5G49160	1.15594998	1.63E-45	Arabidopsis thaliana DNA (cytosine-5)-methyltransferase 1 mRNA, complete cds
AT5G16270	1.067504305	1.78E-45	Arabidopsis thaliana sister chromatid cohesion 1 protein 4 mRNA, complete cds
AT5G65930	1.138059565	4.32E-45	Arabidopsis thaliana kinesin-like calmodulin-binding protein ZWICHEL mRNA, complete cds
AT2G17820	1.073248258	6.38E-45	Arabidopsis thaliana histidine kinase 1 mRNA, complete cds
AT4G32820	1.561804886	1.01E-44	Arabidopsis thaliana tetratricopeptide repeat domain-containing protein mRNA, complete cds
AT4G16630	1.462531544	2.40E-44	Arabidopsis thaliana DEAD-box ATP-dependent RNA helicase 28 mRNA, complete cds
AT1G35660	2.05479441	4.19E-44	Arabidopsis thaliana uncharacterized protein mRNA, complete cds
AT4G03550	1.087040381	4.82E-44	Arabidopsis thaliana callose synthase 12 mRNA, complete cds
AT1G72390	1.370964304	6.71E-44	Arabidopsis thaliana protein PHYTOCHROME-DEPENDENT LATE-FLOWERING mRNA, complete cds
AT2G38770	1.277613828	7.79E-44	Arabidopsis thaliana embryo defective 2765 mRNA, complete cds
AT5G53440	1.090357269	9.96E-44	Arabidopsis thaliana uncharacterized protein mRNA, complete cds
AT3G59100	1.101887282	1.63E-43	Arabidopsis thaliana putative callose synthase 6 mRNA, complete cds
AT5G15580	1.468379926	1.73E-43	Arabidopsis thaliana protein longifolia1 mRNA, complete cds
AT5G01890	1.057496593	1.95E-43	Arabidopsis thaliana leucine-rich receptor-like protein kinase mRNA, complete cds
AT3G53540	1.372885593	2.01E-43	Arabidopsis thaliana uncharacterized protein mRNA, complete cds
AT5G38840	1.683964105	3.50E-43	Arabidopsis thaliana SMAD/FHA domain-containing protein mRNA, complete cds
AT1G55970	1.973000318	7.16E-43	Arabidopsis thaliana histone

			acetyltransferase of the CBP family 4 mRNA, complete cds
AT3G15120	1.681192212	9.54E-43	Arabidopsis thaliana P-loop containing nucleoside triphosphate hydrolases superfamily protein mRNA, complete cds
AT2G33435	1.956574771	9.84E-43	Arabidopsis thaliana RNA recognition motif-containing protein mRNA, complete cds
AT5G11530	1.599102233	1.36E-42	Arabidopsis thaliana embryonic flower 1 mRNA, complete cds
AT2G21440	1.155703262	1.99E-42	Arabidopsis thaliana RNA recognition motif-containing protein mRNA, complete cds
AT3G06530	1.271945903	4.32E-42	Arabidopsis thaliana U3snoRNP10 and NUC211 domain-containing protein mRNA, complete cds
AT1G55540	1.59819995	7.52E-42	Arabidopsis thaliana nuclear pore complex protein LNO1 mRNA, complete cds
AT5G67100	1.553715848	7.96E-42	Arabidopsis thaliana DNA polymerase alpha catalytic subunit mRNA, complete cds
AT4G36180	1.035917693	1.09E-41	Arabidopsis thaliana putative LRR receptor-like serine/threonine-protein kinase mRNA, complete cds
AT2G21380	1.227925655	1.29E-41	Arabidopsis thaliana kinesin motor family protein mRNA, complete cds
AT1G66980	1.019771008	2.23E-41	Arabidopsis thaliana suppressor of npr1-1 constitutive 4 mRNA, complete cds
AT5G42400	1.432892054	3.50E-41	Arabidopsis thaliana histone methyltransferase SDG25 mRNA, complete cds
AT3G21250	1.039691375	3.69E-41	Arabidopsis thaliana multidrug resistance-associated protein 6 mRNA, complete cds
AT5G45060	1.201369552	3.92E-41	Arabidopsis thaliana TIR-NBS-LRR class disease resistance protein mRNA, complete cds
AT2G36200	1.613371514	5.80E-41	Arabidopsis thaliana kinesin family protein mRNA, complete cds
AT5G13530	1.265494325	7.88E-41	Arabidopsis thaliana E3 ubiquitin-protein ligase KEG mRNA, complete cds

AT3G61780	1.520066211	2.89E-40	Arabidopsis thaliana protein embryo defective 1703 mRNA, complete cds
AT2G26570	1.061387313	4.81E-40	Arabidopsis thaliana coiled-coil protein WEB1 mRNA, complete cds
AT4G30790	1.268372803	5.06E-40	Arabidopsis thaliana uncharacterized protein mRNA, complete cds
AT4G29940	1.822343651	7.55E-40	Arabidopsis thaliana Pathogenesis-related homeodomain protein mRNA, complete cds
AT4G02400	1.443079181	8.98E-40	Arabidopsis thaliana U3 ribonucleoprotein family protein mRNA, complete cds
AT5G08230	1.660594998	1.05E-39	Arabidopsis thaliana Tudor/PWWP/MBT domain-containing protein mRNA, complete cds
AT2G25050	1.722965142	1.25E-39	Arabidopsis thaliana actin-binding FH2 protein mRNA, complete cds
AT3G22142	2.966574049	1.37E-39	Arabidopsis thaliana chromosome 3, complete sequence
AT4G12020	1.269449402	1.85E-39	Arabidopsis thaliana mitogen-activated protein kinase kinase kinase 11 mRNA, complete cds
AT4G02560	1.218112816	2.43E-39	Arabidopsis thaliana homeobox protein LUMINIDEPENDENS mRNA, complete cds
AT5G13590	1.059711952	2.55E-39	Arabidopsis thaliana uncharacterized protein mRNA, complete cds
AT2G34357	1.031271299	2.55E-39	Arabidopsis thaliana NUC173 domain-containing protein mRNA, complete cds
AT5G52280	1.318296746	2.59E-39	Arabidopsis thaliana Myosin heavy chain-related protein mRNA, complete cds
AT1G63300	1.964962999	2.71E-39	Arabidopsis thaliana Myosin heavy chain-related protein mRNA, complete cds
AT5G62410	1.744181628	7.41E-39	Arabidopsis thaliana structural maintenance of chromosome 2 mRNA, complete cds
AT3G10310	2.117390228	3.91E-38	Arabidopsis thaliana myosin and kinesin motor and CH domain-containing protein mRNA, complete cds
AT5G48360	1.066109723	4.36E-38	Arabidopsis thaliana actin-binding FH2 (formin homology 2) family protein

			mRNA, complete cds
AT5G65460	1.197161833	5.12E-38	Arabidopsis thaliana kinesin like protein for actin based chloroplast movement 2 mRNA, complete cds
AT1G17440	1.059476631	5.71E-38	Arabidopsis thaliana transcription initiation factor TFIID subunit 12B mRNA, complete cds
AT4G32730	1.169275522	6.60E-38	Arabidopsis thaliana myb-related protein 3R-1 mRNA, complete cds
AT1G73960	1.141803083	1.09E-37	Arabidopsis thaliana TBP-associated factor 2 mRNA, complete cds
AT5G55540	1.33892515	1.47E-37	Arabidopsis thaliana protein TORNADO 1 mRNA, complete cds
AT5G07980	1.380357081	2.29E-37	Arabidopsis thaliana dentin sialophosphoprotein-like protein mRNA, complete cds
AT1G14840	1.266840771	3.15E-37	Arabidopsis thaliana microtubule-associated protein 70-4 mRNA, complete cds
AT1G01040	1.038411734	3.67E-37	Arabidopsis thaliana endoribonuclease Dicer-like 1 mRNA, complete cds
AT3G04740	1.080358679	8.20E-37	Arabidopsis thaliana mediator of RNA polymerase II transcription subunit 14 mRNA, complete cds
AT4G10930	1.946332368	1.93E-36	Arabidopsis thaliana uncharacterized protein mRNA, complete cds
AT2G40770	1.968621878	2.46E-36	Arabidopsis thaliana RING-finger, DEAD-like helicase, PHD and SNF2 domain-containing protein mRNA, complete cds
AT3G01810	1.002048851	3.93E-36	Arabidopsis thaliana uncharacterized protein mRNA, complete cds
AT3G58160	2.043099745	4.58E-36	Arabidopsis thaliana Class XI myosin mRNA, complete cds
AT1G27850	1.148571883	5.53E-36	Arabidopsis thaliana uncharacterized protein mRNA, complete cds
AT1G02990	1.665251159	6.33E-36	Arabidopsis thaliana uncharacterized protein mRNA, complete cds
AT1G15240	1.023433038	6.85E-36	Arabidopsis thaliana phox (PX) domain-containing protein mRNA, complete cds
AT5G45650	1.057328837	8.26E-36	Arabidopsis thaliana subtilase family protein mRNA, complete cds
AT5G62640	1.348439452	8.27E-36	Arabidopsis thaliana protein EARLY

			FLOWERING 5 mRNA, complete cds
AT3G44050	1.9362004	1.39E-35	Arabidopsis thaliana kinesin motor protein-like protein mRNA, complete cds
AT1G61850	1.060756488	1.73E-35	Arabidopsis thaliana galactolipase/ phospholipase mRNA, complete cds
AT4G02710	1.750333855	1.89E-35	Arabidopsis thaliana Kinase interacting (KIP1-like) family protein mRNA, complete cds
ATCG00190	1.755023188	2.14E-35	Arabidopsis thaliana chloroplast DNA, complete genome, ecotype: Columbia
AT1G02110	1.073470428	2.97E-35	Arabidopsis thaliana uncharacterized protein mRNA, complete cds
AT4G16310	1.308038496	5.23E-35	Arabidopsis thaliana protein LSD1-like 3 mRNA, complete cds
AT4G12770	1.05708721	7.00E-35	Arabidopsis thaliana auxilin-related protein 2 mRNA, complete cds
AT3G49500	1.097220255	7.09E-35	Arabidopsis thaliana RNA-dependent RNA polymerase 6 mRNA, complete cds
AT5G04290	1.609849561	1.05E-34	Arabidopsis thaliana kow domain-containing transcription factor 1 mRNA, complete cds
AT1G70070	1.095775505	3.13E-34	Arabidopsis thaliana DEAD-box ATP-dependent RNA helicase ISE2 mRNA, complete cds
AT3G17900	1.035960112	4.00E-34	Arabidopsis thaliana uncharacterized protein mRNA, complete cds
AT2G24650	1.184945952	5.59E-34	Arabidopsis thaliana B3 domain-containing protein REM13 mRNA, complete cds
AT4G00060	1.013019405	8.33E-34	Arabidopsis thaliana protein MATERNAL EFFECT EMBRYO ARREST 44 mRNA, complete cds
AT5G35750	1.09635601	1.22E-33	Arabidopsis thaliana histidine kinase 2 mRNA, complete cds
AT2G40030	2.185220935	1.37E-33	Arabidopsis thaliana nuclear RNA polymerase D1B mRNA, complete cds
AT3G28030	1.743369071	2.17E-33	Arabidopsis thaliana DNA repair protein UVH3 mRNA, complete cds
AT1G27595	1.043970746	2.36E-33	Arabidopsis thaliana symplekin tight junction protein domain-containing protein mRNA, complete cds
AT3G21430	1.349416524	2.47E-33	Arabidopsis thaliana protein ALWAYS EARLY 3 mRNA, complete cds
AT2G46240	2.227573146	4.03E-33	Arabidopsis thaliana BCL-2-associated

			athanogene 6 mRNA, complete cds
AT4G05190	1.337507602	4.36E-33	Arabidopsis thaliana kinesin 5 mRNA, complete cds
AT5G38150	1.010346451	4.37E-33	Arabidopsis thaliana plastid movement impaired protein 15 mRNA, complete cds
AT3G57060	1.643538551	4.51E-33	Arabidopsis thaliana putative condensin complex protein Cap-D2 mRNA, complete cds
AT5G66310	1.507162812	9.21E-33	Arabidopsis thaliana ATP binding microtubule motor family protein mRNA, complete cds
AT3G19670	1.003525186	9.75E-33	Arabidopsis thaliana pre-mRNA-processing protein 40B mRNA, complete cds
AT1G19220	1.509581234	1.09E-32	Arabidopsis thaliana auxin response factor 19 mRNA, complete cds
AT5G49430	1.305878713	1.56E-32	Arabidopsis thaliana WD40/YVTN repeat and Bromo-WDR9-I-like domain-containing protein mRNA, complete cds
AT4G00990	1.241196429	7.44E-32	Arabidopsis thaliana transcription factor jumonji (jmjC) domain-containing protein mRNA, complete cds
AT3G46960	1.237964751	8.74E-32	Arabidopsis thaliana DExD/H box RNA helicase mRNA, complete cds
AT1G76740	3.360730472	1.97E-31	Arabidopsis thaliana uncharacterized protein mRNA, complete cds
AT5G28400	2.270201327	2.47E-31	Arabidopsis thaliana uncharacterized protein mRNA, complete cds
AT2G46630	1.139024256	2.99E-31	Arabidopsis thaliana chromosome 2, complete sequence
AT1G79730	1.064053137	3.38E-31	Arabidopsis thaliana protein early flowering 7 mRNA, complete cds
AT5G23080	1.126327246	4.79E-31	Arabidopsis thaliana TATA-box binding protein-interacting protein TOUGH mRNA, complete cds
AT1G63640	1.184895875	8.01E-31	Arabidopsis thaliana microtubule motor protein KinG mRNA, complete cds
AT2G20290	1.753170411	1.15E-30	Arabidopsis thaliana myosin-like protein XIG mRNA, complete cds
AT3G51150	1.458427146	1.17E-30	Arabidopsis thaliana ATP binding microtubule motor family protein mRNA, complete cds

AT3G43690.1	1.243455664	1.21E-30	Arabidopsis thaliana chromosome 3, complete sequence
AT3G14570	1.244162605	1.46E-30	Arabidopsis thaliana glucan synthase-like 4 mRNA, complete cds
AT5G04240	1.145453154	4.76E-30	Arabidopsis thaliana probable lysine-specific demethylase ELF6 mRNA, complete cds
AT3G14980	1.388037818	7.38E-30	Arabidopsis thaliana histone H3 acetyltransferase IDM1 mRNA, complete cds
AT1G64570	1.079488753	1.06E-29	Arabidopsis thaliana DUO pollen 3 protein mRNA, complete cds
AT3G16840	1.007693037	1.22E-29	Arabidopsis thaliana P-loop containing nucleoside triphosphate hydrolases superfamily protein mRNA, complete cds
AT1G34355	2.381151432	1.34E-29	Arabidopsis thaliana parallel Spindle 1 protein mRNA, complete cds
AT3G43920	1.798258301	1.47E-29	Arabidopsis thaliana endoribonuclease Dicer-like 3 mRNA, complete cds
AT1G06490	1.726034281	1.63E-29	Arabidopsis thaliana callose synthase 7 mRNA, complete cds
AT5G27240	1.75834896	1.98E-29	Arabidopsis thaliana chromosome 5 sequence
AT1G63100	1.101270562	2.03E-29	Arabidopsis thaliana chromosome 1 sequence
AT5G44870	1.06174453	2.26E-29	Arabidopsis thaliana TIR-NBS-LRR class disease resistance protein LAZ5 mRNA, complete cds
AT1G19485	1.127825507	2.64E-29	Arabidopsis thaliana transducin/WD-40 repeat-containing protein mRNA, complete cds
AT2G48110	1.125406907	3.66E-29	Arabidopsis thaliana protein REDUCED EPIDERMAL FLUORESCENCE 4 mRNA, complete cds
AT1G72440	1.017611205	3.74E-29	Arabidopsis thaliana protein SLOW WALKER2 mRNA, complete cds
AT1G77030	1.155160911	5.43E-29	Arabidopsis thaliana putative DEAD-box ATP-dependent RNA helicase 29 mRNA, complete cds
AT4G02020	1.024768927	6.43E-29	Arabidopsis thaliana histone-lysine N-methyltransferase EZA1 mRNA, complete cds
AT3G20010	1.361865761	7.25E-29	Arabidopsis thaliana RING

			finger-related, SNF2 and helicase domain-containing protein mRNA, complete cds
AT3G06480	1.186054875	1.04E-28	Arabidopsis thaliana DEAD-box ATP-dependent RNA helicase 40 mRNA, complete cds
AT4G29790	1.05941507	1.20E-28	Arabidopsis thaliana uncharacterized protein mRNA, complete cds
AT1G77580	1.296763842	1.27E-28	Arabidopsis thaliana filament-like plant protein 1 mRNA, complete cds
AT1G62310	1.190654821	1.59E-28	Arabidopsis thaliana transcription factor jumonji domain-containing protein mRNA, complete cds
AT1G50240	1.356727388	1.84E-28	Arabidopsis thaliana serine/threonine-protein kinase FUSED mRNA, complete cds
AT5G58160	1.538698245	2.02E-28	Arabidopsis thaliana formin-like protein 13 mRNA, complete cds
AT3G51120	1.046056496	5.21E-28	Arabidopsis thaliana zinc finger CCCH domain-containing protein 44 mRNA, complete cds
AT2G27980	1.002435978	6.59E-28	Arabidopsis thaliana acyl-CoA N-acyltransferase with RING/FYVE/PHD-type zinc finger domain-containing protein mRNA, complete cds
AT5G44660	1.000146461	1.08E-27	Arabidopsis thaliana uncharacterized protein mRNA, complete cds
AT5G42920	1.061448346	1.35E-27	Arabidopsis thaliana THO complex, subunit 5 mRNA, complete cds
AT5G19310	1.419746677	1.37E-27	Arabidopsis thaliana homeotic protein regulator mRNA, complete cds
AT3G56410	1.669993085	1.73E-27	Arabidopsis thaliana uncharacterized protein mRNA, complete cds
AT1G61690	1.047847516	1.82E-27	Arabidopsis thaliana phosphoinositide binding protein mRNA, complete cds
AT5G48310	1.586941886	1.83E-27	Arabidopsis thaliana uncharacterized protein mRNA, complete cds
AT5G22010	1.121151086	2.17E-27	Arabidopsis thaliana replication factor C1 mRNA, complete cds
AT4G22485	2.731992219	2.27E-27	Arabidopsis thaliana chromosome 4 sequence
AT1G76720	1.485149153	2.86E-27	Arabidopsis thaliana eukaryotic translation initiation factor 2 (eIF-2)

			family protein mRNA, complete cds
AT1G07400	1.17647017	3.18E-27	Arabidopsis thaliana chromosome 1 sequence
AT4G04970	1.007657968	8.00E-27	Arabidopsis thaliana glucan synthase-like 1 mRNA, complete cds
AT2G30800	1.235267537	8.20E-27	Arabidopsis thaliana protein helicase in vascular tissue and tapetum mRNA, complete cds
AT3G07540	1.193757546	8.26E-27	Arabidopsis thaliana Actin-binding FH2 (formin homology 2) family protein mRNA, complete cds
AT5G51600	1.384380794	8.27E-27	Arabidopsis thaliana microtubule associated protein MAP65-3 mRNA, complete cds
AT1G58060	1.04737316	8.63E-27	Arabidopsis thaliana RNA helicase family protein mRNA, complete cds
AT4G27370	1.675650246	1.11E-26	Arabidopsis thaliana myosin heavy chain-like protein mRNA, complete cds
AT2G02480	1.380714477	1.21E-26	Arabidopsis thaliana protein STICHEL mRNA, complete cds
AT2G36350	1.535263771	1.28E-26	Arabidopsis thaliana protein kinase mRNA, complete cds
AT1G55250	1.148409628	1.37E-26	Arabidopsis thaliana E3 ubiquitin-protein ligase HUB2 mRNA, complete cds
AT5G28320	2.17463418	1.88E-26	Arabidopsis thaliana uncharacterized protein mRNA, complete cds
AT1G30410	1.046873504	4.10E-26	Arabidopsis thaliana multidrug resistance-associated protein 13 mRNA, complete cds
AT5G26860	1.042262981	4.23E-26	Arabidopsis thaliana lon protease 1 mRNA, complete cds
AT3G44730	1.563343374	4.59E-26	Arabidopsis thaliana kinesin-like protein 1 mRNA, complete cds
AT1G77600	1.278494971	5.33E-26	Arabidopsis thaliana ARM repeat superfamily protein mRNA, complete cds
AT1G21160	1.005849198	5.58E-26	Arabidopsis thaliana eukaryotic translation initiation factor 2 (eIF-2) family protein mRNA, complete cds
AT3G48430	1.071180712	6.11E-26	Arabidopsis thaliana lysine-specific demethylase REF6 mRNA, complete cds
AT1G19850	1.100939707	6.24E-26	Arabidopsis thaliana auxin response factor 5 mRNA, complete cds
AT2G43900	1.015158535	1.56E-25	Arabidopsis thaliana 5-inositol-phosphate

			phosphatase mRNA, complete cds
AT2G19950	1.010968169	1.59E-25	Arabidopsis thaliana golgin candidate 1 mRNA, complete cds
AT4G01020	1.671609155	1.68E-25	Arabidopsis thaliana zinc finger-related and helicase and IBR domain-containing protein mRNA, complete cds
AT1G63020	1.996031287	2.21E-25	Arabidopsis thaliana DNA-directed RNA polymerase IV subunit 1 mRNA, complete cds
AT2G34780	1.388037818	2.27E-25	Arabidopsis thaliana protein MATERNAL EFFECT EMBRYO ARREST 22 mRNA, complete cds
AT3G45830	1.2781602	3.37E-25	Arabidopsis thaliana uncharacterized protein mRNA, complete cds
AT1G21170	1.074043904	4.63E-25	Arabidopsis thaliana exocyst complex component SEC5B mRNA, complete cds
AT3G12915	1.602655095	6.76E-25	Arabidopsis thaliana ribosomal protein S5/Elongation factor G/III/V family protein mRNA, complete cds
AT5G60040	1.688827146	8.01E-25	Arabidopsis thaliana nuclear RNA polymerase C1 mRNA, complete cds
AT4G24610	1.272291917	8.33E-25	Arabidopsis thaliana uncharacterized protein mRNA, complete cds
AT3G02890	1.236034724	8.45E-25	Arabidopsis thaliana RING/FYVE/PHD zinc finger-related protein mRNA, complete cds
AT1G17450	1.640803888	1.25E-24	Arabidopsis thaliana B-block binding subunit of TFIIIC mRNA, complete cds
AT1G03830	1.823253199	1.76E-24	Arabidopsis thaliana guanylate-binding protein mRNA, complete cds
AT1G16800	1.851769574	4.30E-24	Arabidopsis thaliana P-loop containing nucleoside triphosphate hydrolases superfamily protein mRNA, complete cds
AT3G23780	1.074473196	7.09E-24	Arabidopsis thaliana nuclear RNA polymerase D2A mRNA, complete cds
AT2G13970.1	1.146191047	1.17E-23	Arabidopsis thaliana chromosome 2, complete sequence
AT1G10450	1.063343289	1.38E-23	Arabidopsis thaliana paired amphipathic helix protein Sin3-like 6 mRNA, complete cds
AT1G34047	4.34409447	1.63E-23	Arabidopsis thaliana defensin-like protein 208 mRNA, complete cds
AT3G55160	1.414429317	1.75E-23	Arabidopsis thaliana uncharacterized

			protein mRNA, complete cds
AT2G33440	1.959194519	1.92E-23	Arabidopsis thaliana RNA recognition motif-containing protein mRNA, complete cds
AT1G79150	1.269982056	2.76E-23	Arabidopsis thaliana nucleolar complex-associated protein domain-containing protein mRNA, complete cds
AT3G02930	1.651072224	2.93E-23	Arabidopsis thaliana uncharacterized protein mRNA, complete cds
AT1G13940	1.138755025	1.01E-22	Arabidopsis thaliana uncharacterized protein mRNA, complete cds
AT1G49870	1.645403474	1.41E-22	Arabidopsis thaliana uncharacterized protein mRNA, complete cds
AT5G54650	1.425391232	1.55E-22	Arabidopsis thaliana formin-like protein 5 mRNA, complete cds
AT1G03780	1.097136618	1.81E-22	Arabidopsis thaliana protein TPX2 mRNA, complete cds
AT4G27010	1.687141478	2.14E-22	Arabidopsis thaliana uncharacterized protein mRNA, complete cds
AT1G12040	1.591738084	3.15E-22	Arabidopsis thaliana chromosome 1 sequence
AT4G02660	2.351511942	3.35E-22	Arabidopsis thaliana Beige/BEACH and WD40 domain-containing protein mRNA, complete cds
AT3G42670	1.449438362	3.55E-22	Arabidopsis thaliana chromatin remodeling 38 mRNA, complete cds
AT1G07910	1.29391564	3.89E-22	Arabidopsis thaliana tRNA ligase mRNA, complete cds
AT3G44200	1.171145194	5.76E-22	Arabidopsis thaliana serine/threonine-protein kinase Nek5 mRNA, complete cds
AT3G21290	1.115805625	8.18E-22	Arabidopsis thaliana dentin sialophosphoprotein-like protein mRNA, complete cds
AT5G17880	1.063100973	8.32E-22	Arabidopsis thaliana TIR-NBS-LRR class disease resistance protein mRNA, complete cds
AT5G42140	1.245895973	1.47E-21	Arabidopsis thaliana Regulator of chromosome condensation (RCC1) family with FYVE zinc finger domain mRNA, complete cds
AT3G57980	1.407146641	1.53E-21	Arabidopsis thaliana DNA-binding bromodomain-containing protein mRNA,

			complete cds
AT4G32710	1.489791317	1.55E-21	Arabidopsis thaliana proline-rich receptor-like protein kinase PERK14 mRNA, complete cds
AT4G14150	2.004709178	1.63E-21	Arabidopsis thaliana phragmoplast-associated kinesin-related protein 1 mRNA, complete cds
AT2G37420	1.419746677	1.77E-21	Arabidopsis thaliana ATP binding microtubule motor family protein mRNA, complete cds
AT5G48610	1.412285364	2.18E-21	Arabidopsis thaliana uncharacterized protein mRNA, complete cds
AT4G13750	1.838699227	2.27E-21	Arabidopsis thaliana nuclear factor NO VEIN mRNA, complete cds
AT4G38070	1.848802591	2.52E-21	Arabidopsis thaliana transcription factor bHLH131 mRNA, complete cds
AT4G11130	1.803075317	2.56E-21	Arabidopsis thaliana RNA-dependent RNA polymerase 2 mRNA, complete cds
AT5G23480	2.057889216	3.40E-21	Arabidopsis thaliana SWIB/MDM2, Plus-3 and GYF domain-containing protein mRNA, complete cds
AT1G20720	2.074473196	3.59E-21	Arabidopsis thaliana RAD3-like DNA-binding helicase protein mRNA, complete cds
AT5G37190	1.222451752	3.96E-21	Arabidopsis thaliana COP1-interacting protein 4 mRNA, complete cds
AT2G34730	1.160437105	8.55E-21	Arabidopsis thaliana myosin heavy chain-related mRNA, complete cds
AT5G46400	1.33646747	9.21E-21	Arabidopsis thaliana pre-mRNA-processing factor 39 mRNA, complete cds
AT5G47490	1.089514327	1.07E-20	Arabidopsis thaliana RGPR-related protein mRNA, complete cds
AT1G10930	1.383139018	2.18E-20	Arabidopsis thaliana ATP-dependent DNA helicase Q-like 4A mRNA, complete cds
AT4G25280	1.032378866	2.70E-20	Arabidopsis thaliana probably adenylate kinase mRNA, complete cds
AT1G20670	1.004459253	4.38E-20	Arabidopsis thaliana DNA-binding bromodomain-containing protein mRNA, complete cds
AT3G60160	1.321669008	5.85E-20	Arabidopsis thaliana multidrug resistance-associated protein 9 mRNA, complete cds

AT4G26660	1.175734214	6.57E-20	Arabidopsis thaliana uncharacterized protein mRNA, complete cds
AT3G47460	1.380491009	8.00E-20	Arabidopsis thaliana structural maintenance of chromosomes protein 2-2 mRNA, complete cds
AT1G66730	1.01143263	1.03E-19	Arabidopsis thaliana DNA ligase 6 mRNA, complete cds
AT2G36480	1.198717218	1.16E-19	Arabidopsis thaliana ENTH/VHS-like protein mRNA, complete cds
AT1G33390	1.167893461	1.29E-19	Arabidopsis thaliana protein FASCIATED STEM 4 mRNA, complete cds
AT1G67530	1.05610255	1.39E-19	Arabidopsis thaliana ARM repeat superfamily protein mRNA, complete cds
AT5G37630	1.377877952	1.40E-19	Arabidopsis thaliana protein EMBRYO DEFECTIVE 2656 mRNA, complete cds
AT5G10370	1.708216131	1.54E-19	Arabidopsis thaliana helicase , IBR and zinc finger protein domain-containing protein mRNA, complete cds
AT1G04160	1.375213777	2.45E-19	Arabidopsis thaliana myosin XI B mRNA, complete cds
AT5G49680	1.504993964	2.69E-19	Arabidopsis thaliana SABRE-like protein mRNA, complete cds
AT5G53020	1.155544806	2.90E-19	Arabidopsis thaliana ribonuclease P protein subunit P38-like protein mRNA, complete cds
AT3G22400	1.323267562	3.72E-19	Arabidopsis thaliana lipoxygenase 5 mRNA, complete cds
AT4G12010	1.241628741	4.55E-19	Arabidopsis thaliana TIR-NBS-LRR class disease resistance protein mRNA, complete cds
AT2G33240	2.525541342	5.97E-19	Arabidopsis thaliana myosin XI D mRNA, complete cds
AT4G21270	1.286915893	6.26E-19	Arabidopsis thaliana kinesin-like motor protein heavy chain mRNA, complete cds
AT1G23935	2.703539643	6.67E-19	Arabidopsis thaliana uncharacterized protein mRNA, complete cds
AT2G36255	2.5135687	1.03E-18	Arabidopsis thaliana putative defensin-like protein 203 mRNA, complete cds
AT5G61460	1.489321154	1.88E-18	Arabidopsis thaliana protein SMC6B mRNA, complete cds

AT5G55520	1.237187497	1.97E-18	Arabidopsis thaliana uncharacterized protein mRNA, complete cds
AT5G40820	2.236034724	2.25E-18	Arabidopsis thaliana serine/threonine-protein kinase ATR mRNA, complete cds
AT1G36180	1.680819567	2.79E-18	Arabidopsis thaliana acetyl-CoA carboxylase 2 mRNA, complete cds
AT2G28240	1.517618779	2.89E-18	Arabidopsis thaliana ATP-dependent helicase-like protein mRNA, complete cds
AT2G31900	1.285200781	3.31E-18	Arabidopsis thaliana myosin-like protein XIF mRNA, complete cds
AT4G25120	1.730361133	3.52E-18	Arabidopsis thaliana helicase SRS2-like protein mRNA, complete cds
AT3G10180	2.400010459	5.80E-18	Arabidopsis thaliana kinesin motor protein-related protein mRNA, complete cds
AT3G03340	1.037681315	6.78E-18	Arabidopsis thaliana protein UNFERTILIZED EMBRYO SAC 6 mRNA, complete cds
AT4G00930	1.472734982	1.31E-17	Arabidopsis thaliana COP1-interacting protein 4.1 mRNA, complete cds
AT5G15070	1.528900354	1.48E-17	Arabidopsis thaliana phosphoglycerate mutase-like protein mRNA, complete cds
AT2G31320	1.260196775	1.99E-17	Arabidopsis thaliana poly [ADP-ribose] polymerase 2 mRNA, complete cds
AT1G14460	1.074014921	2.72E-17	Arabidopsis thaliana AAA-type ATPase family protein mRNA, complete cds
AT4G32350	1.259493697	5.03E-17	Arabidopsis thaliana regulator of Vps4 activity protein mRNA, complete cds
AT1G28010	1.032600461	5.36E-17	Arabidopsis thaliana ABC transporter B family member 14 mRNA, complete cds
AT5G26160	1.29391564	5.39E-17	Arabidopsis thaliana uncharacterized protein mRNA, complete cds
AT5G63950	1.018923037	5.60E-17	Arabidopsis thaliana protein chromatin remodeling 24 mRNA, complete cds
AT3G15550	1.177701414	5.76E-17	Arabidopsis thaliana uncharacterized protein mRNA, complete cds
AT4G32700	1.403467858	6.02E-17	Arabidopsis thaliana MUS308 and mammalian DNA polymerase-like protein mRNA, complete cds
AT4G29440	1.190342634	6.32E-17	Arabidopsis thaliana regulator of Vps4 activity protein mRNA, complete cds
AT4G08115.1	3.153572564	7.01E-17	Arabidopsis thaliana chromosome 4

			sequence
AT3G49160	1.161058127	7.29E-17	Arabidopsis thaliana pyruvate kinase-like protein mRNA, complete cds
AT4G34900	1.185573389	8.89E-17	Arabidopsis thaliana xanthine dehydrogenase 2 mRNA, complete cds
AT4G14330	1.173750446	9.81E-17	Arabidopsis thaliana phragmoplast-associated kinesin-related protein 2 mRNA, complete cds
AT1G26130	1.19570263	2.02E-16	Arabidopsis thaliana putative phospholipid-transporting ATPase 12 mRNA, complete cds
AT3G05470	1.427747528	2.08E-16	Arabidopsis thaliana Actin-binding protein FH2 mRNA, complete cds
AT3G18100	1.414033026	2.15E-16	Arabidopsis thaliana myb domain protein 4r1 mRNA, complete cds
AT1G09470	1.485823709	2.99E-16	Arabidopsis thaliana uncharacterized protein mRNA, complete cds
AT1G80810	1.357664169	3.45E-16	Arabidopsis thaliana Tudor/PWWP/MBT superfamily protein mRNA, complete cds
AT2G23360	1.234709327	3.57E-16	Arabidopsis thaliana filament-like plant protein 7 mRNA, complete cds
AT4G32970	1.252522847	4.58E-16	Arabidopsis thaliana uncharacterized protein mRNA, complete cds
AT1G62970	1.261125705	4.69E-16	Arabidopsis thaliana chromosome 1 sequence
AT4G16680	1.167749985	5.09E-16	Arabidopsis thaliana putative RNA helicase mRNA, complete cds
AT4G08100.1	2.698377938	6.28E-16	Arabidopsis thaliana chromosome 4 sequence
AT4G36120	1.493280978	7.40E-16	Arabidopsis thaliana uncharacterized protein mRNA, complete cds
AT4G02110	1.560874415	8.03E-16	Arabidopsis thaliana transcription coactivator protein mRNA, complete cds
AT1G60860	1.061809585	1.08E-15	Arabidopsis thaliana ADP-ribosylation factor GTPase-activating protein AGD2 mRNA, complete cds
AT5G38140	1.094124099	2.23E-15	Arabidopsis thaliana nuclear factor Y, subunit C12 mRNA, complete cds
AT1G76780	2.216669399	3.11E-15	Arabidopsis thaliana chromosome 1 sequence
AT5G07180	1.196068317	3.36E-15	Arabidopsis thaliana LRR receptor-like serine/threonine-protein kinase ERL2 mRNA, complete cds

AT1G68725	1.868170345	5.49E-15	Arabidopsis thaliana arabinogalactan protein 19 mRNA, complete cds
AT5G52310	1.121702049	5.64E-15	Arabidopsis thaliana protein LOW-TEMPERATURE-INDUCED 78 mRNA, complete cds
AT3G22760	1.0085587	6.81E-15	Arabidopsis thaliana CXC domain containing TSO1-like protein 1 mRNA, complete cds
AT2G15820	1.001258579	6.90E-15	Arabidopsis thaliana organelle transcript processing 51 mRNA, complete cds
AT4G17610	1.127948977	7.30E-15	Arabidopsis thaliana tRNA/rRNA methyltransferase (SpoU) family protein mRNA, complete cds
AT5G45520	3.236034724	7.78E-15	Arabidopsis thaliana chromosome 5 sequence
AT3G13440	1.507815173	8.04E-15	Arabidopsis thaliana S-adenosyl-L-methionine-dependent methyltransferase-like protein mRNA, complete cds
AT1G20060	1.730689392	8.44E-15	Arabidopsis thaliana ATP binding microtubule motor family protein mRNA, complete cds
AT5G10800	1.789592088	8.86E-15	Arabidopsis thaliana RNA recognition motif (RRM)-containing protein mRNA, complete cds
AT5G52410	1.227264515	1.20E-14	Arabidopsis thaliana uncharacterized protein mRNA, complete cds
AT4G04223	3.525541342	1.26E-14	Arabidopsis thaliana ARM repeat superfamily protein mRNA, complete cds
AT3G18090	1.305936738	1.42E-14	Arabidopsis thaliana DNA-directed RNA polymerase D subunit 2b mRNA, complete cds
AT3G14460	2.177141035	1.44E-14	Arabidopsis thaliana chromosome 3, complete sequence
AT1G77270	1.214208362	1.99E-14	Arabidopsis thaliana uncharacterized protein mRNA, complete cds
AT2G24700	1.170446383	2.73E-14	Arabidopsis thaliana transcriptional factor B3 family protein mRNA, complete cds
AT3G55060	1.690600588	2.78E-14	Arabidopsis thaliana uncharacterized protein mRNA, complete cds
AT4G22970	1.691429961	4.94E-14	Arabidopsis thaliana separase-like protein mRNA, complete cds

AT4G16970	1.492374478	6.72E-14	Arabidopsis thaliana protein kinase superfamily protein mRNA, complete cds
AT5G07660	1.788575747	7.80E-14	Arabidopsis thaliana structural maintenance of chromosomes 6A mRNA, complete cds
AT4G19430	1.436672593	7.91E-14	Arabidopsis thaliana chromosome 4 sequence
AT3G07210	1.147246486	8.60E-14	Arabidopsis thaliana uncharacterized protein mRNA, complete cds
AT5G02810	1.262506936	8.61E-14	Arabidopsis thaliana pseudo-response regulator 7 mRNA, complete cds
AT5G28390	1.703539643	1.01E-13	Arabidopsis thaliana RNA recognition motif-containing protein mRNA, complete cds
AT1G75150	1.661056312	1.03E-13	Arabidopsis thaliana uncharacterized protein mRNA, complete cds
AT3G49650	1.297735424	1.09E-13	Arabidopsis thaliana P-loop containing nucleoside triphosphate hydrolase-like protein mRNA, complete cds
AT1G15160	1.850381032	1.22E-13	Arabidopsis thaliana MATE efflux family protein mRNA, complete cds
AT5G63960	1.003178467	1.28E-13	Arabidopsis thaliana DNA polymerase delta subunit 1 mRNA, complete cds
AT4G01490.1	1.867564043	1.36E-13	Arabidopsis thaliana chromosome 4 sequence
AT1G58561.1	1.171644137	1.82E-13	Arabidopsis thaliana chromosome 1 sequence
AT3G43210	1.055985506	2.01E-13	Arabidopsis thaliana kinesin TETRASPORE mRNA, complete cds
AT5G60150	1.594488695	2.05E-13	Arabidopsis thaliana uncharacterized protein mRNA, complete cds
AT1G08840	1.486952844	2.11E-13	Arabidopsis thaliana embryo defective protein 2411 mRNA, complete cds
AT5G15920	1.188828881	2.43E-13	Arabidopsis thaliana structural maintenance of chromosomes 5 mRNA, complete cds
AT2G45900	1.641018559	2.44E-13	Arabidopsis thaliana phosphatidylinositol N-acetyglucosaminlytransferase subunit P-like protein mRNA, complete cds
AT4G21820	1.49292439	2.50E-13	Arabidopsis thaliana binding / calmodulin binding protein mRNA, complete cds
AT2G19930	1.216669399	3.34E-13	Arabidopsis thaliana probable

			RNA-dependent RNA polymerase 5 mRNA, complete cds
AT4G18490	2.632456545	3.92E-13	Arabidopsis thaliana uncharacterized protein mRNA, complete cds
AT5G52230	1.392096034	4.64E-13	Arabidopsis thaliana methyl-CPG-binding domain protein 13 mRNA, complete cds
AT5G12100	1.169203216	5.56E-13	Arabidopsis thaliana chromosome 5 sequence
AT5G24280	1.58350894	5.57E-13	Arabidopsis thaliana gamma-irradiation and mitomycin c induced 1 mRNA, complete cds
AT4G02070	1.082788464	6.49E-13	Arabidopsis thaliana DNA mismatch repair protein MSH6 mRNA, complete cds
AT2G46980	1.388037818	8.17E-13	Arabidopsis thaliana ASYNAPTIC 3 mRNA, complete cds
AT3G51290	1.451763415	1.05E-12	Arabidopsis thaliana protein ALTERED PHOSPHATE STARVATION RESPONSE 1 mRNA, complete cds
AT2G45460	1.348509454	1.23E-12	Arabidopsis thaliana SMAD/FHA domain-containing protein mRNA, complete cds
AT2G12490.1	1.520675586	1.81E-12	Arabidopsis thaliana chromosome 2, complete sequence
AT3G42640	1.009926887	2.63E-12	Arabidopsis thaliana H(+)-ATPase 8 mRNA, complete cds
AT4G14970	1.5434315	2.96E-12	Arabidopsis thaliana uncharacterized protein mRNA, complete cds
AT2G16390	1.038939522	3.00E-12	Arabidopsis thaliana putative chromatin remodeling protein mRNA, complete cds
AT2G35350	1.066109723	3.03E-12	Arabidopsis thaliana protein phosphatase 2C 29 mRNA, complete cds
AT2G35340	1.170446383	3.72E-12	Arabidopsis thaliana protein MATERNAL EFFECT EMBRYO ARREST 29 mRNA, complete cds
AT5G07810	1.549431962	6.20E-12	Arabidopsis thaliana SNF2 and helicase domain-containing protein mRNA, complete cds
AT3G58650	1.078182555	6.39E-12	Arabidopsis thaliana uncharacterized protein mRNA, complete cds
AT4G00380	1.353686313	7.93E-12	Arabidopsis thaliana XH/XS domain-containing protein mRNA, complete cds

AT5G57250	1.01682882	1.02E-11	Arabidopsis thaliana pentatricopeptide repeat-containing protein mRNA, complete cds
AT5G39580	1.28578776	1.14E-11	Arabidopsis thaliana peroxidase 62 mRNA, complete cds
AT1G10270	1.054235623	1.25E-11	Arabidopsis thaliana chromosome 1 sequence
AT2G30480	1.135151367	1.35E-11	Arabidopsis thaliana uncharacterized protein mRNA, complete cds
AT1G50890	1.066109723	1.79E-11	Arabidopsis thaliana microtubule-associated protein SPIRAL2-like mRNA, complete cds
AT2G47230	1.025792268	2.20E-11	Arabidopsis thaliana uncharacterized protein mRNA, complete cds
AT3G48770	2.803075317	3.50E-11	Arabidopsis thaliana ATP/DNA binding protein mRNA, complete cds
AT2G18760	1.019567137	3.64E-11	Arabidopsis thaliana chromatin remodeling 8 mRNA, complete cds
AT1G29560	1.945815489	3.96E-11	Arabidopsis thaliana zinc finger C-x8-C-x5-C-x3-H type family protein mRNA, complete cds
AT1G16330	1.342234128	5.16E-11	Arabidopsis thaliana cyclin B3-1 mRNA, complete cds
AT4G24450	1.176533713	5.45E-11	Arabidopsis thaliana phosphoglucan, water dikinase mRNA, complete cds
AT4G19890	1.231918616	6.70E-11	Arabidopsis thaliana Pentatricopeptide repeat domain-containing protein mRNA, complete cds
AT2G24350	1.677544435	6.88E-11	Arabidopsis thaliana RNA recognition motif-containing protein mRNA, complete cds
AT3G53800	1.018015435	8.01E-11	Arabidopsis thaliana hsp70-interacting protein FES1B-like protein mRNA, complete cds
AT2G31340	1.1602319	8.84E-11	Arabidopsis thaliana protein embryo defective 1381 mRNA, complete cds
AT1G56120	1.24897378	1.01E-10	Arabidopsis thaliana Leucine-rich repeat transmembrane protein kinase mRNA, complete cds
AT1G65470	1.000656661	1.34E-10	Arabidopsis thaliana chromatin assembly factor 1 subunit FAS1 mRNA, complete cds
AT5G61940	2.103584428	1.57E-10	Arabidopsis thaliana ubiquitin carboxyl-terminal hydrolase-related

			protein mRNA, complete cds
AT4G22860	1.256212606	1.80E-10	Arabidopsis thaliana cell cycle regulated microtubule associated protein mRNA, complete cds
AT1G59540	1.021909919	1.93E-10	Arabidopsis thaliana kinesin-like protein mRNA, complete cds
AT2G01750	1.039878181	2.05E-10	Arabidopsis thaliana microtubule-associated protein 70-3 mRNA, complete cds
AT1G22770	1.5135687	2.54E-10	Arabidopsis thaliana protein GIGANTEA mRNA, complete cds
AT1G76820	1.198560019	2.72E-10	Arabidopsis thaliana translation initiation factor 2 (eIF-2) family protein mRNA, complete cds
AT4G17000	1.103977908	3.37E-10	Arabidopsis thaliana uncharacterized protein mRNA, complete cds
AT3G28153.1	3.838699227	3.69E-10	Arabidopsis thaliana chromosome 3, complete sequence
AT4G16960	1.282146649	3.96E-10	Arabidopsis thaliana TIR-NBS-LRR class disease resistance protein mRNA, complete cds
AT3G13100	1.0455484	4.03E-10	Arabidopsis thaliana ABC transporter C family member 7 mRNA, complete cds
AT3G62455.1	2.5135687	4.23E-10	Arabidopsis thaliana chromosome 3, complete sequence
AT3G21480	1.088477536	4.34E-10	Arabidopsis thaliana BRCT domain-containing DNA repair protein mRNA, complete cds
AT1G55200	1.399093006	4.64E-10	Arabidopsis thaliana protein kinase protein with adenine nucleotide alpha hydrolases-like domain mRNA, complete cds
AT3G20200	1.55153655	4.90E-10	Arabidopsis thaliana Protein kinase protein with adenine nucleotide alpha hydrolases-like domain mRNA, complete cds
AT1G69830	1.058949931	5.78E-10	Arabidopsis thaliana alpha-amylase-like 3 mRNA, complete cds
AT3G20440	1.025467738	6.88E-10	Arabidopsis thaliana putative glycoside hydrolase mRNA, complete cds
AT4G15810	1.012670464	8.55E-10	Arabidopsis thaliana P-loop containing nucleoside triphosphate hydrolases superfamily protein mRNA, complete cds

AT5G15700	1.226574395	1.34E-09	Arabidopsis thaliana DNA-directed RNA polymerase 2 mRNA, complete cds
ATCG00180	1.734488232	1.89E-09	Arabidopsis lyrata subsp. lyrata RNA polymerase beta subunit-1, mRNA
AT4G34060	1.734488232	1.89E-09	Arabidopsis thaliana DEMETER-like protein 3 mRNA, complete cds
AT3G18730	1.400010459	2.08E-09	Arabidopsis thaliana protein BRUSHY 1 mRNA, complete cds
AT1G48120	1.099276587	2.27E-09	Arabidopsis thaliana serine/threonine-protein phosphatase 7 long form homolog mRNA, complete cds
AT2G15810.1	2.18158694	2.29E-09	Arabidopsis thaliana chromosome 2, complete sequence
AT5G27890	1.058332372	2.82E-09	Arabidopsis thaliana uncharacterized protein mRNA, complete cds
AT5G52910	1.170905913	2.87E-09	Arabidopsis thaliana protein TIMELESS mRNA, complete cds
AT1G53160	1.066109723	3.06E-09	Arabidopsis thaliana squamosa promoter binding protein-like 4 mRNA, complete cds
AT5G46880	1.0157362	3.06E-09	Arabidopsis thaliana homeobox-leucine zipper protein HDG5 mRNA, complete cds
AT5G28330	1.988107211	3.09E-09	Arabidopsis thaliana chromosome 5 sequence
AT1G14090	1.223651	3.42E-09	Arabidopsis thaliana chromosome 1 sequence
AT5G22750	1.213666911	4.86E-09	Arabidopsis thaliana mRNA for hypothetical protein, complete cds, clone: RAFL16-05-A16
AT2G42920	1.074377339	4.91E-09	Arabidopsis thaliana pentatricopeptide repeat-containing protein mRNA, complete cds
AT1G15830	1.131065165	6.13E-09	Arabidopsis thaliana chromosome 1 sequence
AT1G02065	1.319866315	6.72E-09	Arabidopsis thaliana squamosa promoter binding protein-like 8 mRNA, complete cds
AT1G65920	1.178319226	7.67E-09	Arabidopsis thaliana Regulator of chromosome condensation (RCC1) family with FYVE zinc finger domain mRNA, complete cds
AT3G66652	1.288502144	9.67E-09	Arabidopsis thaliana fip1 motif-containing protein mRNA,

			complete cds
AT3G20280	1.112206137	9.76E-09	Arabidopsis thaliana PHD finger protein mRNA, complete cds
AT5G02430	1.334042928	1.18E-08	Arabidopsis thaliana transducin/WD40 domain-containing protein mRNA, complete cds
AT1G11160	1.185050796	1.68E-08	Arabidopsis thaliana WD40 domain-containing protein mRNA, complete cds
AT4G21070	1.093943931	1.88E-08	Arabidopsis thaliana protein BREAST CANCER SUSCEPTIBILITY 1-like protein mRNA, complete cds
AT1G52030	1.075098506	1.97E-08	Arabidopsis thaliana myrosinase-binding protein 2 mRNA, complete cds
AT1G21810	1.233219709	2.31E-08	Arabidopsis thaliana uncharacterized protein mRNA, complete cds
AT4G37490	1.002684343	2.47E-08	Arabidopsis thaliana cyclin-B1-1 mRNA, complete cds
AT1G13790	1.331699834	2.51E-08	Arabidopsis thaliana SGS3-like protein FDM4 mRNA, complete cds
AT4G16910.1	2.589671679	2.70E-08	Arabidopsis thaliana chromosome 4 sequence
AT4G36515	1.585483882	3.04E-08	Arabidopsis thaliana uncharacterized protein mRNA, complete cds
AT2G22560	1.057176598	3.06E-08	Arabidopsis thaliana Kinase interacting (KIP1-like) family protein mRNA, complete cds
AT3G05130	1.257052506	3.50E-08	Arabidopsis thaliana chromosome 3, complete sequence
AT4G05631	1.301326185	4.39E-08	Arabidopsis thaliana uncharacterized protein mRNA, complete cds
AT5G44620	1.245079864	5.03E-08	Arabidopsis thaliana cytochrome P450, family 706, subfamily A, polypeptide 3 mRNA, complete cds
AT1G54575	1.651072224	5.60E-08	Arabidopsis thaliana chromosome 1 sequence
AT1G45120.1	1.107332386	6.65E-08	Arabidopsis thaliana chromosome 1 sequence
AT3G11000	1.973000318	7.31E-08	Arabidopsis thaliana DCD (Development and Cell Death) domain protein mRNA, complete cds
AT3G06630	2.120557507	8.77E-08	Arabidopsis thaliana protein kinase family protein mRNA, complete cds
AT5G27895.1	2.120557507	8.77E-08	Arabidopsis thaliana chromosome 5

			sequence
AT3G05415.1	2.18158694	9.05E-08	Arabidopsis thaliana chromosome 3, complete sequence
AT3G04690	1.220832318	1.03E-07	Arabidopsis thaliana receptor-like protein kinase ANXUR1 mRNA, complete cds
AT3G44690	1.504230835	1.38E-07	Arabidopsis thaliana ecotype Bla-1 DM2A, DM2B, DM2C, DM2D, DM2E, DM2F, DM2G, and DM2H genes, complete cds
AT5G05940	1.046744398	1.39E-07	Arabidopsis thaliana ROP guanine nucleotide exchange factor 5 mRNA, complete cds
AT2G05440	1.249331547	1.90E-07	Arabidopsis thaliana glycine-rich protein 9 mRNA, complete cds
AT2G19920	1.372771061	1.97E-07	Arabidopsis thaliana RNA-dependent RNA polymerase family protein mRNA, complete cds
AT4G34400	1.179567773	1.98E-07	Arabidopsis thaliana AP2/B3-like transcriptional factor family protein mRNA, complete cds
AT2G15880	1.066109723	2.00E-07	Arabidopsis thaliana pollen-specific leucine-rich repeat extensin-like protein 3 mRNA, complete cds
AT3G49142	2.066109723	2.41E-07	Arabidopsis thaliana putative pentatricopeptide repeat-containing protein mRNA, complete cds
AT1G52450	2.125003412	2.53E-07	Arabidopsis thaliana ubiquitin carboxyl-terminal hydrolase-related protein mRNA, complete cds
AT1G15165	1.055767779	2.75E-07	Arabidopsis thaliana U-box domain protein mRNA, complete cds
AT4G38180	1.088136029	2.84E-07	Arabidopsis thaliana protein FAR1-related sequence 5 mRNA, complete cds
AT4G29990	1.30314892	2.90E-07	Arabidopsis thaliana leucine-rich repeat transmembrane protein kinase mRNA, complete cds
AT5G35914.1	2.873464645	3.01E-07	Arabidopsis thaliana chromosome 5 sequence
AT4G19050	1.201039303	3.08E-07	Arabidopsis thaliana NB-ARC domain-containing disease resistance protein mRNA, complete cds
AT1G09050	1.469465417	3.14E-07	Arabidopsis thaliana uncharacterized protein mRNA, complete cds

AT1G30290	1.112652309	3.28E-07	Arabidopsis thaliana chromosome 1 sequence
AT1G26760	1.335296356	3.52E-07	Arabidopsis thaliana chromosome 1 sequence
AT3G24982	1.203613247	3.76E-07	Arabidopsis thaliana receptor like protein 40 mRNA, complete cds
AT2G17490.1	3.820997225	3.83E-07	Arabidopsis thaliana chromosome 2, complete sequence
AT2G36026	3.820997225	3.83E-07	Arabidopsis thaliana chromosome 2, complete sequence
AT1G25175	5.589671679	4.52E-07	Arabidopsis thaliana At1g25170/F4F7_2 mRNA, complete cds
AT2G12440.1	5.589671679	4.52E-07	Arabidopsis thaliana chromosome 2, complete sequence
AT4G13650	1.414033026	4.92E-07	Arabidopsis thaliana pentatricopeptide repeat-containing protein mRNA, complete cds
AT2G05510	3.339128217	5.76E-07	Arabidopsis thaliana glycine-rich protein mRNA, complete cds
AT1G11100	1.229048294	5.78E-07	Arabidopsis thaliana SNF2 , helicase and zinc-finger domain-containing protein mRNA, complete cds
AT1G23940	1.651072224	5.85E-07	Arabidopsis thaliana ARM repeat superfamily protein mRNA, complete cds
AT3G26050	1.273705142	6.09E-07	Arabidopsis thaliana TPX2 (targeting protein for Xklp2) family protein mRNA, complete cds
AT5G61300	1.102329911	6.80E-07	Arabidopsis thaliana uncharacterized protein mRNA, complete cds
AT4G15890	1.014394686	7.88E-07	Arabidopsis thaliana condensation complex subunit 1 domain-containing protein mRNA, complete cds
ATCG00170	1.887139582	9.01E-07	Arabidopsis thaliana chloroplast DNA, complete genome, ecotype: Columbia
AT5G12030	1.651072224	1.05E-06	Arabidopsis thaliana chromosome 5 sequence
AT1G60930	1.012867702	1.18E-06	Arabidopsis thaliana RECQ helicase L4B mRNA, complete cds
AT1G09040	1.468208166	1.20E-06	Arabidopsis thaliana uncharacterized protein mRNA, complete cds
AT5G27230	2.351511942	1.27E-06	Arabidopsis thaliana Frigida-like protein mRNA, complete cds
AT1G35530	1.53077799	1.33E-06	Arabidopsis thaliana helicase FANCM

			mRNA, complete cds
AT3G24715	1.118577143	1.33E-06	Arabidopsis thaliana octicosapeptide/Phox/Bem1p domain-containing protein kinase mRNA, complete cds
AT3G04980	1.436947418	1.51E-06	Arabidopsis thaliana chromosome 3, complete sequence
AT5G38910	4.525541342	1.55E-06	Arabidopsis thaliana putative germin-like protein subfamily 1 member 9 mRNA, complete cds
AT4G30130	1.091644815	1.72E-06	Arabidopsis thaliana uncharacterized protein mRNA, complete cds
AT5G05510	1.105638087	1.85E-06	Arabidopsis thaliana Mad3/BUB1 homology region 1 mRNA, complete cds
AT4G13880	1.407146641	1.88E-06	Arabidopsis thaliana receptor like protein 48 mRNA, complete cds
AT5G01550	1.794030177	2.08E-06	Arabidopsis thaliana chromosome 5 sequence
AT3G44765	2.136499051	2.13E-06	Arabidopsis thaliana Full-length cDNA Complete sequence from clone GSLTPGH55ZA10 of Hormone Treated Callus of strain col-0 of Arabidopsis thaliana (thale cress)
AT5G26170	1.46096934	2.15E-06	Arabidopsis thaliana putative WRKY transcription factor 50 mRNA, complete cds
AT1G26330	1.261125705	2.76E-06	Arabidopsis thaliana DNA binding protein mRNA, complete cds
AT5G48060	1.157257611	3.35E-06	Arabidopsis thaliana C2 calcium/lipid-binding plant phosphoribosyltransferase family protein mRNA, complete cds
AT3G07273	2.032942859	3.36E-06	Arabidopsis thaliana chromosome 3, complete sequence
AT3G28770	1.359840926	3.42E-06	Arabidopsis thaliana uncharacterized protein mRNA, complete cds
AT5G11470	1.325496352	3.44E-06	Arabidopsis thaliana protein ANTI-SILENCING 1 mRNA, complete cds
AT5G02950	1.197354256	3.70E-06	Arabidopsis thaliana chromosome 5 sequence
AT1G05950	1.10875406	4.14E-06	Arabidopsis thaliana uncharacterized protein mRNA, complete cds
AT1G59218	1.200410815	4.51E-06	Arabidopsis thaliana putative disease

			resistance protein RDL6/RF9 mRNA, complete cds
AT3G51490	1.998995527	5.55E-06	Arabidopsis thaliana tonoplast monosaccharide transporter3 mRNA, complete cds
AT1G49940	1.431759195	5.78E-06	Arabidopsis thaliana uncharacterized protein mRNA, complete cds
AT5G45720	1.066109723	6.06E-06	Arabidopsis thaliana AAA-type ATPase family protein mRNA, complete cds
AT5G12020	1.813343652	6.73E-06	Arabidopsis thaliana chromosome 5 sequence
AT5G38870.1	1.052689207	6.83E-06	Arabidopsis thaliana chromosome 5 sequence
AT4G09750	1.027635575	7.03E-06	Arabidopsis thaliana mRNA for hypothetical protein, complete cds, clone: RAFL14-08-C18
AT1G77310	1.288502144	7.31E-06	Arabidopsis thaliana uncharacterized protein mRNA, complete cds
AT2G30750	1.128845478	8.10E-06	Arabidopsis thaliana cytochrome P450 71A12 mRNA, complete cds
AT1G48580	1.907411977	8.34E-06	Arabidopsis thaliana uncharacterized protein mRNA, complete cds
AT3G12970	1.329144129	8.92E-06	Arabidopsis thaliana chromosome 3, complete sequence
AT3G23370	1.531773295	9.17E-06	Arabidopsis thaliana RNA recognition motif-containing protein mRNA, complete cds
AT5G61980	1.080756499	9.81E-06	Arabidopsis thaliana ADP-ribosylation factor GTPase-activating protein AGD1 mRNA, complete cds
AT5G25045.1	4.314037236	1.01E-05	Arabidopsis thaliana chromosome 5 sequence
AT3G07274	1.301737971	1.08E-05	Arabidopsis thaliana chromosome 3, complete sequence
AT4G30700	1.489321154	1.18E-05	Arabidopsis thaliana chromosome 4 sequence
AT2G35160	1.097136618	1.29E-05	Arabidopsis thaliana histone-lysine N-methyltransferase, H3 lysine-9 specific SUVH5 mRNA, complete cds
AT1G26540	1.038095347	1.41E-05	Arabidopsis thaliana agenet domain-containing protein mRNA, complete cds
AT1G15825	1.19650636	1.43E-05	Arabidopsis thaliana chromosome 1 sequence

AT5G08250	2.766549441	1.49E-05	Arabidopsis thaliana cytochrome P450 superfamily protein mRNA, complete cds
AT3G29590	1.222228925	1.49E-05	Arabidopsis thaliana chromosome 3, complete sequence
AT5G34825.1	3.458427146	1.59E-05	Arabidopsis thaliana chromosome 5 sequence
AT1G35112.1	2.55153655	1.69E-05	Arabidopsis thaliana chromosome 1 sequence
AT5G21150	1.750607897	1.71E-05	Arabidopsis thaliana argonaute 9 mRNA, complete cds
AT3G09480	2.153572564	1.81E-05	Arabidopsis thaliana chromosome 3, complete sequence
AT2G24680	1.425190816	2.23E-05	Arabidopsis thaliana B3 domain-containing protein REM12 mRNA, complete cds
AT5G66940	1.49906913	2.52E-05	Arabidopsis thaliana chromosome 5 sequence
AT3G58770	1.953634994	2.67E-05	Arabidopsis thaliana uncharacterized protein mRNA, complete cds
AT4G31610	1.757987428	3.10E-05	Arabidopsis thaliana B3 domain-containing protein REM1 mRNA, complete cds
AT5G40100	1.035736074	3.17E-05	Arabidopsis thaliana TIR-NBS-LRR class disease resistance protein mRNA, complete cds
AT5G27250.1	1.455152014	3.21E-05	Arabidopsis thaliana chromosome 5 sequence
AT5G57320	1.241196429	3.29E-05	Arabidopsis thaliana putative villin mRNA, complete cds
AT1G07390	1.188100247	3.71E-05	Arabidopsis thaliana receptor like protein 1 mRNA, complete cds
AT1G65120	1.472102083	3.83E-05	Arabidopsis thaliana ubiquitin carboxyl-terminal hydrolase-related protein mRNA, complete cds
AT3G27730	1.854605618	3.95E-05	Arabidopsis thaliana DNA helicase mRNA, complete cds
AT3G01970	1.428679802	4.82E-05	Arabidopsis thaliana WRKY DNA-binding protein 45 mRNA, complete cds
AT1G64625	1.00598873	4.86E-05	Arabidopsis thaliana transcription factor bHLH157 mRNA, complete cds
AT3G13090	1.168471441	5.28E-05	Arabidopsis thaliana multidrug resistance-associated protein 8 mRNA,

			complete cds
AT2G01510	1.510894566	5.43E-05	Arabidopsis thaliana chromosome 2, complete sequence
AT1G09080	3.314037236	5.50E-05	Arabidopsis thaliana protein BIP3 mRNA, complete cds
AT1G12210	1.123442898	5.74E-05	Arabidopsis thaliana chromosome 1 sequence
AT3G02030	1.004709178	5.95E-05	Arabidopsis thaliana transferase mRNA, complete cds
AT4G00890	1.258754801	5.95E-05	Arabidopsis thaliana chromosome 4 sequence
AT4G13410	1.388037818	6.00E-05	Arabidopsis thaliana putative mannan synthase 15 mRNA, complete cds
AT5G27220	4.066109723	6.71E-05	Arabidopsis thaliana Frigida-like protein mRNA, complete cds
AT3G48900	1.199376254	6.78E-05	Arabidopsis thaliana single-stranded DNA endonuclease family protein mRNA, complete cds
AT2G34390	1.873464645	7.12E-05	Arabidopsis thaliana aquaporin NIP2-1 mRNA, complete cds
AT5G62230	1.003373968	7.29E-05	Arabidopsis thaliana LRR receptor-like serine/threonine-protein kinase ERL1 mRNA, complete cds
AT1G74150	1.203613247	8.30E-05	Arabidopsis thaliana kelch motif-containing protein mRNA, complete cds
AT5G49390	1.126651265	8.62E-05	Arabidopsis thaliana uncharacterized protein mRNA, complete cds
AT2G38720	1.361565606	8.91E-05	Arabidopsis thaliana microtubule-associated protein 65-5 mRNA, complete cds
AT5G40010	1.314037236	8.95E-05	Arabidopsis thaliana chromosome 5 sequence
AT3G02330	1.776603106	0.000102364	Arabidopsis thaliana pentatricopeptide repeat-containing protein mRNA, complete cds
AT4G24580	1.066109723	0.000103965	Arabidopsis thaliana Rho GTPase activating protein REN1 mRNA, complete cds
AT5G25970	1.651072224	0.000122792	Arabidopsis thaliana core-2/I-branching beta-1,6-N-acetylglucosaminyltransferase family protein mRNA, complete cds
AT3G57965	3.973000318	0.000126726	Arabidopsis thaliana chromosome 3, complete sequence

AT5G23155	3.973000318	0.000126726	Arabidopsis thaliana chromosome 5 sequence
AT2G33760	2.803075317	0.000131199	Arabidopsis thaliana chromosome 2, complete sequence
AT1G04930	1.08600928	0.000138066	Arabidopsis thaliana hydroxyproline-rich glycoprotein family protein mRNA, complete cds
AT2G17770	1.55153655	0.00013877	Arabidopsis thaliana bZIP transcription factor FD-like protein mRNA, complete cds
AT3G30720	1.690600588	0.000142308	Arabidopsis thaliana qua-quine starch mRNA, complete cds
AT3G18020	1.185408651	0.000145273	Arabidopsis thaliana chromosome 3, complete sequence
AT1G64250.1	2.066109723	0.000150934	Arabidopsis thaliana chromosome 1 sequence
AT3G28945.1	1.218112816	0.000151735	Arabidopsis thaliana chromosome 3, complete sequence
AT1G69710	2.18158694	0.000157215	Arabidopsis thaliana regulator of chromosome condensation and FYVE zinc finger domain-containing protein mRNA, complete cds
AT3G18550	2.18158694	0.000157215	Arabidopsis thaliana transcription factor TCP18 mRNA, complete cds
AT4G11070	2.329144129	0.000157556	Arabidopsis thaliana putative WRKY transcription factor 41 mRNA, complete cds
AT1G05490	1.735961121	0.000163637	Arabidopsis thaliana chromatin remodeling 31 mRNA, complete cds
AT1G64572	1.735961121	0.000163637	Arabidopsis thaliana chromosome 1 sequence
AT4G35640	1.580682896	0.000163713	Arabidopsis thaliana serine acetyltransferase 3;2 mRNA, complete cds
AT2G19120	1.0470009	0.000176864	Arabidopsis thaliana P-loop containing nucleoside triphosphate hydrolases superfamily protein mRNA, complete cds
AT1G62440	1.189492138	0.000177932	Arabidopsis thaliana leucine-rich repeat extensin-like protein 2 mRNA, complete cds
AT2G26700	1.189492138	0.000177932	Arabidopsis thaliana protein kinase PINOID 2 mRNA, complete cds
AT3G14395	1.788575747	0.000186354	Arabidopsis thaliana uncharacterized

			protein mRNA, complete cds
AT3G27640	1.134281226	0.000195107	Arabidopsis thaliana transducin/WD40 repeat-like superfamily protein mRNA, complete cds
AT4G01270	1.028635017	0.000198844	Arabidopsis thaliana RING/U-box superfamily protein mRNA, complete cds
AT4G11480	1.850381032	0.000209677	Arabidopsis thaliana putative cysteine-rich receptor-like protein kinase 32 mRNA, complete cds
AT3G24780	1.046480916	0.000217205	Arabidopsis thaliana chromosome 3, complete sequence
AT5G03790	1.651072224	0.000224416	Arabidopsis thaliana homeobox 51 mRNA, complete cds
AT3G25013	1.066109723	0.000236244	Arabidopsis thaliana Synaptobrevin family protein mRNA, complete cds
AT1G20400	2.729074736	0.000237107	Arabidopsis thaliana uncharacterized protein mRNA, complete cds
AT1G48660	2.458427146	0.000263945	Arabidopsis thaliana auxin-responsive GH3 family protein mRNA, complete cds
AT5G28415.1	2.125003412	0.000266934	Arabidopsis thaliana chromosome 5 sequence
AT5G64060	2.125003412	0.000266934	Arabidopsis thaliana NAC domain containing protein 103 mRNA, complete cds
AT5G01335.1	2.125003412	0.000266934	Arabidopsis thaliana chromosome 5 sequence
AT3G57970	2.267743584	0.000272331	Arabidopsis thaliana Emsy N Terminus and plant Tudor-like domain-containing protein mRNA, complete cds
AT1G02670	1.279103446	0.000284661	Arabidopsis thaliana P-loop containing nucleoside triphosphate hydrolases superfamily protein mRNA, complete cds
AT5G59720	1.279103446	0.000284661	Arabidopsis thaliana chromosome 5 sequence
AT5G23000	1.57512337	0.000299285	Arabidopsis thaliana transcription factor RAX1 mRNA, complete cds
AT2G13130	1.008006768	0.00033581	Arabidopsis thaliana chromosome 2, complete sequence
AT5G07570	3.066109723	0.000350595	Arabidopsis thaliana glycine/proline-rich protein mRNA, complete cds
AT3G01319	3.066109723	0.000350595	Arabidopsis thaliana chromosome 3,

			complete sequence
ATCG00760	3.066109723	0.000350595	Olimarabidopsis pumila chloroplast DNA, complete sequence
AT5G60630	4.651072224	0.000359338	Arabidopsis thaliana chromosome 5 sequence
AT1G69400	1.089193336	0.000386186	Arabidopsis thaliana transducin/WD-40 repeat-containing protein mRNA, complete cds
AT1G04700	1.209067677	0.000400905	Arabidopsis thaliana PB1 domain-containing protein tyrosine kinase mRNA, complete cds
AT4G31070	1.651072224	0.000411292	Arabidopsis thaliana chromosome 4 sequence
AT3G46230	1.651072224	0.000411292	Arabidopsis thaliana chromosome 3, complete sequence
AT4G31020	1.066109723	0.000438721	Arabidopsis thaliana alpha/beta-Hydrolases superfamily protein mRNA, complete cds
AT3G11385	2.066109723	0.000450776	Arabidopsis thaliana chromosome 3, complete sequence
ATCG00160	3.766549441	0.000455101	Arabidopsis thaliana chloroplast DNA, complete genome, ecotype: Columbia
AT5G19810	2.203613247	0.000468318	Arabidopsis thaliana chromosome 5 sequence
AT1G10417	1.698377938	0.000476995	Arabidopsis thaliana uncharacterized protein mRNA, complete cds
AT1G22275	1.698377938	0.000476995	Arabidopsis thaliana synaptonemal complex protein 2 mRNA, complete cds
AT5G08600	1.147029718	0.000544979	Arabidopsis thaliana U3 ribonucleoprotein (Utp) family protein mRNA, complete cds
AT1G61080	1.043389646	0.000609878	Arabidopsis thaliana hydroxyproline-rich glycoprotein-like protein mRNA, complete cds
AT3G03480	1.267743584	0.00062043	Arabidopsis thaliana acetyl CoA:(Z)-3-hexen-1-ol acetyltransferase mRNA, complete cds
AT2G15800.1	1.820997225	0.000621436	Arabidopsis thaliana chromosome 2, complete sequence
AT1G04600	1.606678104	0.000646309	Arabidopsis thaliana myosin XI A mRNA, complete cds
ATCG00065	2.973000318	0.000647977	Arabidopsis thaliana ecotype XJalt rpl20-rps12 intergenic spacer, complete sequence; ribosomal protein S12 (rps12)

			gene, partial cds; and rps12-clpP intergenic spacer, complete sequence; chloroplast
AT1G02530	2.973000318	0.000647977	Arabidopsis thaliana P-glycoprotein 12 mRNA, complete cds
AT5G66840	1.150173988	0.000669883	Arabidopsis thaliana SAP domain-containing protein mRNA, complete cds
AT5G66960	1.150173988	0.000669883	Arabidopsis thaliana Prolyl oligopeptidase family protein mRNA, complete cds
AT4G08109.1	4.525541342	0.000680644	Arabidopsis thaliana chromosome 4 sequence
AT1G26410	4.525541342	0.000680644	Arabidopsis thaliana chromosome 1 sequence
AT5G09950	1.494953022	0.000713006	Arabidopsis thaliana chromosome 5 sequence
AT5G46460	1.04265075	0.000750625	Arabidopsis thaliana chromosome 5 sequence
AT3G54870	1.277613828	0.000758286	Arabidopsis thaliana armadillo repeat-containing kinesin-like protein 1 mRNA, complete cds
AT5G54570	2.568610063	0.000766013	Arabidopsis thaliana beta glucosidase 41 mRNA, complete cds
ATCG00150	1.120557507	0.000772799	Arabidopsis thaliana chloroplast DNA, complete genome, ecotype: Columbia
AT5G40990	2.314037236	0.000810447	Arabidopsis thaliana GDSL lipase 1 mRNA, complete cds
AT1G51880	1.236034724	0.000904593	Arabidopsis thaliana protein ROOT HAIR SPECIFIC 6 mRNA, complete cds
AT3G09080	1.157257611	0.001013173	Arabidopsis thaliana transducin/WD40 domain-containing protein mRNA, complete cds
AT5G49110	1.018804008	0.001039096	Arabidopsis thaliana uncharacterized protein mRNA, complete cds
AT5G60880	1.018804008	0.001039096	Arabidopsis thaliana protein BREAKING OF ASYMMETRY IN THE STOMATAL LINEAGE mRNA, complete cds
AT1G08730	1.453132846	0.001090421	Arabidopsis thaliana myosin class XI mRNA, complete cds
AT4G21300	1.244446964	0.001108761	Arabidopsis thaliana chromosome 4 sequence
AT3G44970	1.244446964	0.001108761	Arabidopsis thaliana cytochrome P450

			family protein mRNA, complete cds
AT2G44630	1.300574977	0.001131548	Arabidopsis thaliana chromosome 2, complete sequence
AT5G13230	1.843717302	0.001138771	Arabidopsis thaliana chromosome 5 sequence
AT1G15040	1.843717302	0.001138771	Arabidopsis thaliana chromosome 1 sequence
AT1G64310	2.873464645	0.001194738	Arabidopsis thaliana chromosome 1 sequence
AT4G11393	2.873464645	0.001194738	Arabidopsis thaliana Defensin-like (DEFL) family protein mRNA, complete cds
AT2G46840	2.873464645	0.001194738	Arabidopsis thaliana uncharacterized protein mRNA, complete cds
AT1G52120	2.873464645	0.001194738	Arabidopsis thaliana jacalin-like lectin domain-containing protein mRNA, complete cds
AT5G10880	1.940578841	0.001262583	Arabidopsis thaliana tRNA synthetase/ligase-related protein mRNA, complete cds
AT5G18633.1	2.481147222	0.001367949	Arabidopsis thaliana chromosome 5 sequence
AT4G38780	1.314037236	0.001381268	Arabidopsis thaliana Pre-mRNA-processing-splicing factor mRNA, complete cds
AT3G27980	1.040114514	0.001403359	Arabidopsis thaliana plant invertase/pectin methylesterase inhibitor superfamily mRNA, complete cds
AT2G35420	1.127510268	0.001443097	Arabidopsis thaliana chromosome 2, complete sequence
AT3G06120	1.210499632	0.001611631	Arabidopsis thaliana transcription factor MUTE mRNA, complete cds
AT5G46915	1.709965913	0.001620499	Arabidopsis thaliana putative B3 domain-containing protein mRNA, complete cds
AT4G29550	3.525541342	0.001647721	Arabidopsis thaliana uncharacterized protein mRNA, complete cds
AT1G08860	3.525541342	0.001647721	Arabidopsis thaliana protein BONZAI 3 mRNA, complete cds
AT3G59580	1.095857066	0.001656636	Arabidopsis thaliana nodule inception protein-like protein 9 mRNA, complete cds
AT5G01730	1.410064124	0.001656749	Arabidopsis thaliana protein SCAR4 mRNA, complete cds

AT1G08070	1.2640491	0.001665416	Arabidopsis thaliana chromosome 1 sequence
AT1G33615	1.329144129	0.001684952	Arabidopsis thaliana chromosome 1 sequence
ATCG00780	1.039142675	0.001730605	Arabidopsis thaliana chloroplast DNA, complete genome, ecotype: Columbia
AT2G31830	1.13024006	0.001778759	Arabidopsis thaliana Type II inositol-1,4,5-trisphosphate 5-phosphatase 14 mRNA, complete cds
AT5G65350	1.55153655	0.001863681	Arabidopsis thaliana chromosome 5 sequence
AT5G51920	1.218112816	0.001981034	Arabidopsis thaliana chromosome 5 sequence
AT1G02710	1.218112816	0.001981034	Arabidopsis thaliana chromosome 1 sequence
AT1G74350	1.218112816	0.001981034	Arabidopsis thaliana chromosome 1 sequence
AT4G22070	1.435343533	0.002001524	Arabidopsis thaliana WRKY DNA-binding protein 31 mRNA, complete cds
AT2G31751	1.435343533	0.002001524	Arabidopsis thaliana clone asmbl_6700 unknown mRNA sequence
AT1G15310	1.097136618	0.002043805	Arabidopsis thaliana signal recognition particle protein SRP54A mRNA, complete cds
AT5G06400	2.766549441	0.002196351	Arabidopsis thaliana chromosome 5 sequence
AT3G24929	1.59662444	0.002205415	Arabidopsis thaliana uncharacterized protein mRNA, complete cds
AT3G20395	1.992109141	0.002298503	Arabidopsis thaliana RING-finger domain-containing protein mRNA, complete cds
AT5G20420	1.066109723	0.002329751	Arabidopsis thaliana chromatin remodeling 42 mRNA, complete cds
AT5G65570	1.011661939	0.00240054	Arabidopsis thaliana chromosome 5 sequence
AT5G50260	1.464659099	0.002411313	Arabidopsis thaliana KDEL-tailed cysteine endopeptidase CEP1 mRNA, complete cds
AT5G61850	2.388037818	0.002430365	Arabidopsis thaliana protein LEAFY mRNA, complete cds
AT1G22260	2.388037818	0.002430365	Arabidopsis thaliana synaptonemal complex protein ZYP1a mRNA, complete cds

AT2G30670	2.388037818	0.002430365	Arabidopsis thaliana NAD(P)-binding Rossmann-fold superfamily protein mRNA, complete cds
AT4G35210	2.153572564	0.002433603	Arabidopsis thaliana chromosome 4 sequence
AT3G26614.1	4.236034724	0.00249179	Arabidopsis thaliana chromosome 3, complete sequence
AT1G31258	4.236034724	0.00249179	Arabidopsis thaliana uncharacterized protein mRNA, complete cds
AT3G28510	1.365670005	0.002500019	Arabidopsis thaliana chromosome 3, complete sequence
AT4G20730.1	1.365670005	0.002500019	Arabidopsis thaliana mRNA for putative protein, complete cds, clone: RAFL24-04-L03
AT3G47875.1	1.651072224	0.002589161	Arabidopsis thaliana chromosome 3, complete sequence
AT3G49950	1.136499051	0.002706813	Arabidopsis thaliana chromosome 3, complete sequence
AT1G11990	1.49906913	0.002894512	Arabidopsis thaliana O-fucosyltransferase family protein mRNA, complete cds
AT4G10940	1.49906913	0.002894512	Arabidopsis thaliana RING/U-box protein mRNA, complete cds
AT5G43530	1.718186419	0.003007173	Arabidopsis thaliana Helicase protein with RING/U-box domain mRNA, complete cds
AT2G24750	1.388037818	0.003038943	Arabidopsis thaliana chromosome 2, complete sequence
AT4G15165	1.388037818	0.003038943	Arabidopsis thaliana N-terminal nucleophile aminohydrolases (Ntn hydrolases) superfamily protein mRNA, complete cds
AT5G45470	1.30314892	0.003060728	Arabidopsis thaliana chromosome 5 sequence
AT5G03620	1.30314892	0.003060728	Arabidopsis thaliana Subtilisin-like serine endopeptidase family protein mRNA, complete cds
AT5G17430	1.100057055	0.003116529	Arabidopsis thaliana AP2-like ethylene-responsive transcription factor BBM mRNA, complete cds
AT1G29110	3.388037818	0.003143396	Arabidopsis thaliana cysteine proteinases superfamily protein mRNA, complete cds
AT2G18720	3.388037818	0.003143396	Arabidopsis thaliana translation

			elongation factor EF1A/initiation factor IF2gamma family protein mRNA, complete cds
AT2G30660	3.388037818	0.003143396	Arabidopsis thaliana probable 3-hydroxyisobutyryl-CoA hydrolase 3 mRNA, complete cds
AT5G67411	3.388037818	0.003143396	Arabidopsis thaliana clone asmbl_17543 unknown mRNA sequence
AT1G33430	3.388037818	0.003143396	Arabidopsis thaliana putative beta-1,3-galactosyltransferase 8 mRNA, complete cds
AT5G03250	1.035736074	0.003257316	Arabidopsis thaliana phototropic-responsive NPH3 family protein mRNA, complete cds
AT4G14170	1.140110304	0.003342018	Arabidopsis thaliana pentatricopeptide repeat-containing protein mRNA, complete cds
AT1G02190	1.140110304	0.003342018	Arabidopsis thaliana protein CER1-like 1 mRNA, complete cds
ATCG00360	1.803075317	0.003440767	Arabidopsis lyrata subsp. lyrata predicted protein, mRNA
ATCG00770	1.540040911	0.003458025	Arabidopsis thaliana chloroplast DNA, complete genome, ecotype: Columbia
AT3G16610	1.540040911	0.003458025	Arabidopsis thaliana chromosome 3, complete sequence
AT1G78340	1.188100247	0.003538182	Arabidopsis thaliana glutathione S-transferase TAU 22 mRNA, complete cds
AT3G01580	1.188100247	0.003538182	Arabidopsis thaliana pentatricopeptide repeat-containing protein mRNA, complete cds
AT5G52330	1.414033026	0.003686774	Arabidopsis thaliana meprin and TRAF homology domain-containing protein mRNA, complete cds
AT2G29500	1.101733633	0.00385241	Arabidopsis thaliana chromosome 2, complete sequence
AT4G25980	1.914106629	0.003852703	Arabidopsis thaliana peroxidase 43 mRNA, complete cds
ATMG00630	2.651072224	0.00402302	Arabidopsis thaliana ecotype Landsberg erecta mitochondrion, complete genome
AT5G38260	2.651072224	0.00402302	Arabidopsis thaliana protein kinase family protein mRNA, complete cds
AT2G07706	2.651072224	0.00402302	Arabidopsis thaliana uncharacterized protein mRNA, complete cds

AT1G35240	2.651072224	0.00402302	Arabidopsis thaliana auxin response factor 20 mRNA, complete cds
AT5G65550	2.066109723	0.004175193	Arabidopsis thaliana chromosome 5 sequence
AT5G48390	2.288502144	0.004292552	Arabidopsis thaliana ZIP4-like protein mRNA, complete cds
AT5G59330	2.288502144	0.004292552	Arabidopsis thaliana Bifunctional inhibitor/lipid-transfer protein/seed storage 2S albumin superfamily protein mRNA, complete cds
AT5G47330	1.19539274	0.004364661	Arabidopsis thaliana palmitoyl protein thioesterase family protein mRNA, complete cds
AT2G21800	1.066109723	0.004399768	Arabidopsis thaliana essential meiotic endonuclease 1A mRNA, complete cds
AT4G19080	1.444621346	0.004461017	Arabidopsis thaliana chromosome 4 sequence
AT2G34850	1.258754801	0.004528945	Arabidopsis thaliana putative UDP-arabinose 4-epimerase 2 mRNA, complete cds
AT4G16160	4.066109723	0.004820616	Arabidopsis thaliana outer envelope pore protein 16-2 mRNA, complete cds
AT5G28310	4.066109723	0.004820616	Arabidopsis thaliana NAD(P)-binding Rossmann-fold superfamily protein mRNA, complete cds
AT3G54490	4.066109723	0.004820616	Arabidopsis thaliana RNA polymerase II fifth largest subunit, E mRNA, complete cds
AT1G55010	4.066109723	0.004820616	Arabidopsis thaliana plant defensin 1.5 mRNA, complete cds
AT5G01570	1.651072224	0.004830228	Arabidopsis thaliana uncharacterized protein mRNA, complete cds
AT4G17760	1.032942859	0.004983033	Arabidopsis thaliana PCNA domain-containing protein mRNA, complete cds
AT2G12460.1	1.148571883	0.005104565	Arabidopsis thaliana chromosome 2, complete sequence
AT2G07711	1.148571883	0.005104565	Arabidopsis thaliana chromosome 2, complete sequence
AT4G00870	1.066109723	0.005446884	Arabidopsis thaliana transcription factor bHLH14 mRNA, complete cds
AT1G35513	1.2725606	0.005569213	Arabidopsis lyrata subsp. lyrata hypothetical protein, mRNA
AT2G45350	1.2725606	0.005569213	Arabidopsis thaliana chlororespiratory

			reduction 4 protein mRNA, complete cds
AT1G76530	1.361565606	0.005592948	Arabidopsis thaliana auxin efflux carrier family protein mRNA, complete cds
AT1G76065	1.361565606	0.005592948	Arabidopsis thaliana LYR family of Fe/S cluster biogenesis protein mRNA, complete cds
AT1G69120	1.001979385	0.005601013	Arabidopsis thaliana Floral homeotic protein APETALA 1 mRNA, complete cds
AT1G58889.1	1.729074736	0.005613557	Arabidopsis thaliana DNA, retrotransposon:AtRE1, complete sequence, ecotype: Niederzenz
AT3G04184	1.729074736	0.005613557	Arabidopsis thaliana chromosome 3, complete sequence
AT3G19663	3.236034724	0.006004734	Arabidopsis thaliana chromosome 3, complete sequence
AT5G35932	3.236034724	0.006004734	Arabidopsis thaliana chromosome 5 sequence
AT3G03660	3.236034724	0.006004734	Arabidopsis thaliana WUSCHEL related homeobox 11 mRNA, complete cds
AT5G34820.1	3.236034724	0.006004734	Arabidopsis thaliana chromosome 5 sequence
AT2G16005	3.236034724	0.006004734	Arabidopsis thaliana MD-2-related lipid recognition domain-containing protein mRNA, complete cds
AT1G32172	3.236034724	0.006004734	Arabidopsis thaliana chromosome 1 sequence
AT1G69920	3.236034724	0.006004734	Arabidopsis thaliana glutathione S-transferase TAU 12 mRNA, complete cds
AT1G53860	1.031344305	0.006170879	Arabidopsis thaliana remorin-like protein mRNA, complete cds
ATCG00740	1.153572564	0.006315363	Arabidopsis thaliana chloroplast DNA, complete genome, ecotype: Columbia
AT3G14640	1.831644469	0.006406111	Arabidopsis thaliana cytochrome P450, family 72, subfamily A, polypeptide 10 mRNA, complete cds
AT1G34460	1.525541342	0.006454221	Arabidopsis thaliana cyclin-B1-5 mRNA, complete cds
AT5G47600	1.066109723	0.006749262	Arabidopsis thaliana heat shock protein 14.7 mRNA, complete cds
AT3G09450	1.388037818	0.00681934	Arabidopsis thaliana uncharacterized protein mRNA, complete cds
AT5G61070	1.288502144	0.00684705	Arabidopsis thaliana histone deacetylase

			18 mRNA, complete cds
AT2G28040	1.288502144	0.00684705	Arabidopsis thaliana chromosome 2, complete sequence
AT2G19190	1.288502144	0.00684705	Arabidopsis thaliana FLG22-induced receptor-like kinase 1 mRNA, complete cds
AT2G37370	1.973000318	0.007108109	Arabidopsis thaliana uncharacterized protein mRNA, complete cds
AT2G07776	1.973000318	0.007108109	Arabidopsis thaliana uncharacterized protein mRNA, complete cds
AT4G28700	2.525541342	0.007336074	Arabidopsis thaliana chromosome 4 sequence
AT3G60970	2.525541342	0.007336074	Arabidopsis thaliana putative ABC transporter C-15 mRNA, complete cds
AT2G10735	2.525541342	0.007336074	Arabidopsis thaliana chromosome 2, complete sequence
AT5G36740	2.525541342	0.007336074	Arabidopsis thaliana Acyl-CoA N-acyltransferase domain-containing protein mRNA, complete cds
AT3G32925.1	2.525541342	0.007336074	Arabidopsis thaliana chromosome 3, complete sequence
AT1G65810	2.525541342	0.007336074	Arabidopsis thaliana P-loop containing nucleoside triphosphate hydrolases superfamily protein mRNA, complete cds
AT5G03130	2.18158694	0.007530424	Arabidopsis thaliana uncharacterized protein mRNA, complete cds
AT4G27190	2.18158694	0.007530424	Arabidopsis thaliana chromosome 4 sequence
AT3G51060	1.029583847	0.007648942	Arabidopsis thaliana protein SHI RELATED SEQUENCE 1 mRNA, complete cds
AT4G18470	1.029583847	0.007648942	Arabidopsis thaliana negative regulator of systemic acquired resistance (SNI1) mRNA, complete cds
ATCG00640	1.580682896	0.007692207	Arabidopsis thaliana chloroplast DNA, complete genome, ecotype: Columbia
AT3G60670	1.159219127	0.007819468	Arabidopsis thaliana PLATZ transcription factor family protein mRNA, complete cds
AT1G79890	1.159219127	0.007819468	Arabidopsis thaliana RAD3-like DNA-binding helicase protein mRNA, complete cds
AT1G03540	1.307117822	0.008414732	Arabidopsis thaliana chromosome 1

			sequence
AT5G46830	1.651072224	0.009078247	Arabidopsis thaliana chromosome 5 sequence
AT3G05790	1.651072224	0.009078247	Arabidopsis thaliana lon protease 4 mRNA, complete cds
AT3G25011	3.873464645	0.009402205	Arabidopsis thaliana rbpg1-1 (rbpg1-1) and rbpg1-2 (rbpg1-2) genes, complete cds
AT1G06750	3.873464645	0.009402205	Arabidopsis thaliana P-loop containing nucleoside triphosphate hydrolases superfamily protein mRNA, complete cds
AT5G20260	3.873464645	0.009402205	Arabidopsis thaliana Exostosin family protein mRNA, complete cds
AT3G10100.1	3.873464645	0.009402205	Arabidopsis thaliana At4g20730 mRNA, complete cds
AT2G33070	3.873464645	0.009402205	Arabidopsis thaliana Nitrile-specifier protein 2 mRNA, complete cds
AT3G46614	3.873464645	0.009402205	Arabidopsis thaliana chromosome 3, complete sequence
AT5G04630	3.873464645	0.009402205	Arabidopsis thaliana chromosome 5 sequence
AT3G47870	3.873464645	0.009402205	Arabidopsis thaliana LOB domain-containing protein 27 mRNA, complete cds
AT5G66700	1.458427146	0.010057994	Arabidopsis thaliana homeobox-leucine zipper protein ATHB-53 mRNA, complete cds
AT5G18350	1.458427146	0.010057994	Arabidopsis thaliana TIR-NBS-LRR class disease resistance protein mRNA, complete cds
AT5G61920	1.458427146	0.010057994	Arabidopsis thaliana protein FLX-like 4 mRNA, complete cds
AT5G19097.1	1.236034724	0.010152227	Arabidopsis thaliana chromosome 5 sequence
AT4G13992	1.329144129	0.01033424	Arabidopsis thaliana chromosome 4 sequence
AT2G01905	1.066109723	0.010394313	Arabidopsis thaliana cyclin J18 mRNA, complete cds
AT5G28340	1.066109723	0.010394313	Arabidopsis thaliana pentatricopeptide repeat-containing protein mRNA, complete cds
AT1G19394	1.744181628	0.010557732	Arabidopsis thaliana uncharacterized protein mRNA, complete cds

AT4G17085	1.744181628	0.010557732	Arabidopsis thaliana putative membrane lipoprotein mRNA, complete cds
AT4G02960.1	3.066109723	0.011480831	Arabidopsis thaliana copia-like retrotransposon AtRE2 gene for polyprotein, complete cds, ecotype: Isenburg, chromosome: 4
AT5G60470	3.066109723	0.011480831	Arabidopsis thaliana C2H2 and C2HC zinc finger-containing protein mRNA, complete cds
AT5G42120	3.066109723	0.011480831	Arabidopsis thaliana chromosome 5 sequence
AT3G12981	1.873464645	0.011996954	Arabidopsis lyrata subsp. lyrata hypothetical protein, mRNA
AT5G44630	1.873464645	0.011996954	Arabidopsis thaliana sesquiterpene synthase mRNA, complete cds
AT1G72720	1.873464645	0.011996954	Arabidopsis thaliana chromosome 1 sequence
AT3G25880	1.873464645	0.011996954	Arabidopsis thaliana NAD(P)-binding Rossmann-fold superfamily protein mRNA, complete cds
AT4G15350	1.173024927	0.012018553	Arabidopsis thaliana cytochrome P450, family 705, subfamily A, polypeptide 2 mRNA, complete cds
AT2G02061	1.506682314	0.012136768	Arabidopsis thaliana nucleotide-diphospho-sugar transferase domain-containing protein mRNA, complete cds
AT1G58460	1.506682314	0.012136768	Arabidopsis thaliana uncharacterized protein mRNA, complete cds
AT4G20420	1.250534294	0.012549702	Arabidopsis thaliana chromosome 4 sequence
AT5G33382.1	1.35561634	0.012677439	Arabidopsis thaliana chromosome 5 sequence
AT3G20730	1.35561634	0.012677439	Arabidopsis thaliana pentatricopeptide repeat-containing protein mRNA, complete cds
AT4G35190	1.066109723	0.012921694	Arabidopsis thaliana cytokinin riboside 5'-monophosphate phosphoribohydrolase LOG5 mRNA, complete cds
AT1G51670	1.066109723	0.012921694	Arabidopsis thaliana uncharacterized protein mRNA, complete cds
AT1G03800	2.066109723	0.013107783	Arabidopsis thaliana chromosome 1 sequence
AT1G52430	2.066109723	0.013107783	Arabidopsis thaliana ubiquitin

			carboxyl-terminal hydrolase-related protein mRNA, complete cds
AT3G04050	2.066109723	0.013107783	Arabidopsis thaliana pyruvate kinase mRNA, complete cds
AT3G05900	2.066109723	0.013107783	Arabidopsis thaliana neurofilament protein-related protein mRNA, complete cds
AT2G46455	2.388037818	0.013304278	Arabidopsis thaliana OxaA/YidC-like membrane insertion protein mRNA, complete cds
AT2G39240	1.023041001	0.014661756	Arabidopsis thaliana RNA polymerase I specific transcription initiation factor RRN3 protein mRNA, complete cds
AT5G59305	1.18158694	0.014921097	Arabidopsis thaliana uncharacterized protein mRNA, complete cds
AT5G46140	1.18158694	0.014921097	Arabidopsis thaliana uncharacterized protein mRNA, complete cds
ATMG00660	1.267743584	0.015516775	Arabidopsis thaliana ecotype Landsberg erecta mitochondrion, complete genome
AT4G12740	1.066109723	0.016084973	Arabidopsis thaliana HhH-GPD base excision DNA repair family protein mRNA, complete cds
AT3G56550	1.120557507	0.017428339	Arabidopsis thaliana pentatricopeptide repeat-containing protein mRNA, complete cds
AT5G06650	3.651072224	0.018501818	Arabidopsis thaliana chromosome 5 sequence
AT4G25200	3.651072224	0.018501818	Arabidopsis thaliana small heat shock protein 23.6 mRNA, complete cds
AT5G46665.1	3.651072224	0.018501818	Arabidopsis thaliana chromosome 5 sequence
AT1G13290	3.651072224	0.018501818	Arabidopsis thaliana protein DOT5 mRNA, complete cds
AT1G65630	3.651072224	0.018501818	Arabidopsis thaliana putativeDegP protease 3 mRNA, complete cds
AT5G28715.1	3.651072224	0.018501818	Arabidopsis thaliana chromosome 5 sequence
AT1G16705	3.651072224	0.018501818	Arabidopsis thaliana p300/CBP acetyltransferase-related protein mRNA, complete cds
AT2G04515	3.651072224	0.018501818	Arabidopsis thaliana chromosome 2, complete sequence
AT2G15070.1	3.651072224	0.018501818	Arabidopsis thaliana chromosome 2, complete sequence

AT3G24900	3.651072224	0.018501818	Arabidopsis thaliana chromosome 3, complete sequence
AT3G24340	1.428679802	0.018957645	Arabidopsis thaliana chromatin remodeling 40 mRNA, complete cds
AT5G64685.1	1.428679802	0.018957645	Arabidopsis thaliana chromosome 5 sequence
AT3G11680	1.288502144	0.01918588	Arabidopsis thaliana Aluminum activated malate transporter family protein mRNA, complete cds
AT5G56970	1.288502144	0.01918588	Arabidopsis thaliana cytokinin dehydrogenase 3 mRNA, complete cds
AT5G58280	1.288502144	0.01918588	Arabidopsis thaliana AP2/B3-like transcriptional factor family protein mRNA, complete cds
AT4G26260	1.766549441	0.020051575	Arabidopsis thaliana myo-inositol oxygenase 4 mRNA, complete cds
AT5G25230	1.766549441	0.020051575	Arabidopsis thaliana ribosomal protein S5/Elongation factor G/III/V family protein mRNA, complete cds
AT1G69880	1.766549441	0.020051575	Arabidopsis thaliana thioredoxin H8 mRNA, complete cds
AT2G47190	1.766549441	0.020051575	Arabidopsis thaliana mRNA for MYB transcription factor, complete cds, clone: RAFL16-42-L19
AT1G67792	1.066109723	0.020052441	Arabidopsis thaliana mRNA for hypothetical protein, complete cds, clone: RAFL07-30-D10
AT2G39510	1.066109723	0.020052441	Arabidopsis thaliana nodulin MtN21-like transporter family protein mRNA, complete cds
AT4G16015	1.125003412	0.021727318	Arabidopsis thaliana chromosome 4 sequence
AT2G20480	1.125003412	0.021727318	Arabidopsis thaliana uncharacterized protein mRNA, complete cds
AT2G15060.1	2.873464645	0.021956467	Arabidopsis thaliana chromosome 2, complete sequence
AT3G48523.1	2.873464645	0.021956467	Arabidopsis thaliana chromosome 3, complete sequence
AT1G32510	2.873464645	0.021956467	Arabidopsis thaliana NAC domain containing protein 11 mRNA, complete cds
AT4G15320	2.873464645	0.021956467	Arabidopsis thaliana cellulose synthase-like protein B6 mRNA, complete cds

AT3G62610	2.873464645	0.021956467	Arabidopsis thaliana myb domain protein 11 mRNA, complete cds
AT5G33395.1	2.873464645	0.021956467	Arabidopsis thaliana chromosome 5 sequence
AT5G28510	2.873464645	0.021956467	Arabidopsis thaliana beta glucosidase 24 mRNA, complete cds
AT2G03020	1.940578841	0.022610034	Arabidopsis thaliana Heat shock protein HSP20/alpha crystallin family protein mRNA, complete cds
AT5G55020	1.940578841	0.022610034	Arabidopsis thaliana myb domain protein 120 mRNA, complete cds
AT1G04880	1.940578841	0.022610034	Arabidopsis thaliana high mobility group B protein 15 mRNA, complete cds
AT5G64490	1.940578841	0.022610034	Arabidopsis thaliana chromosome 5 sequence
AT5G07640	1.940578841	0.022610034	Arabidopsis thaliana RING/U-box superfamily protein mRNA, complete cds
AT3G15930	1.017200122	0.022775755	Arabidopsis thaliana pentatricopeptide repeat-containing protein mRNA, complete cds
AT1G44120	1.481147222	0.02304694	Arabidopsis thaliana armadillo/beta-catenin-like repeat and C2 calcium/lipid-binding domain-containing protein mRNA, complete cds
AT5G12280	1.481147222	0.02304694	Arabidopsis thaliana SWAP (Suppressor-of-White-APricot)/surp RNA-binding domain-containing protein mRNA, complete cds
AT4G26200	1.203613247	0.023070092	Arabidopsis thaliana 1-aminocyclopropane-1-carboxylate synthase 7 mRNA, complete cds
AT2G27610	1.203613247	0.023070092	Arabidopsis thaliana chromosome 2, complete sequence
AT5G40320	1.314037236	0.023715556	Arabidopsis thaliana chromosome 5 sequence
AT2G29370	2.236034724	0.023965033	Arabidopsis thaliana tropinone reductase-like protein mRNA, complete cds
AT1G62240	2.236034724	0.023965033	Arabidopsis thaliana chromosome 1 sequence
AT4G16820	2.236034724	0.023965033	Arabidopsis thaliana chromosome 4 sequence
AT4G19760	2.236034724	0.023965033	Arabidopsis thaliana Glycosyl hydrolase

			family protein with chitinase insertion domain mRNA, complete cds
AT1G08080	2.236034724	0.023965033	Arabidopsis thaliana alpha carbonic anhydrase 7 mRNA, complete cds
AT2G27880	2.236034724	0.023965033	Arabidopsis thaliana argonaute 5 mRNA, complete cds
AT4G12890	2.236034724	0.023965033	Arabidopsis thaliana Gamma interferon responsive lysosomal thiol (GILT) reductase family protein mRNA, complete cds
AT4G14140	1.55153655	0.027813574	Arabidopsis thaliana DNA methyltransferase 2 mRNA, complete cds
AT1G58320	1.55153655	0.027813574	Arabidopsis thaliana PLAC8 family protein mRNA, complete cds
AT5G09930	1.55153655	0.027813574	Arabidopsis thaliana ABC transporter F family member 2 mRNA, complete cds
AT3G27120	1.013642303	0.028457984	Arabidopsis thaliana P-loop containing nucleoside triphosphate hydrolases superfamily protein mRNA, complete cds
AT1G13620	1.218112816	0.028733515	Arabidopsis thaliana root meristem growth factor 2 mRNA, complete cds
AT1G64260	1.218112816	0.028733515	Arabidopsis thaliana chromosome 1 sequence
AT5G24205	1.218112816	0.028733515	Arabidopsis thaliana chromosome 5 sequence
AT5G24900	1.218112816	0.028733515	Arabidopsis thaliana mRNA for cytochrome P450 like protein, complete cds, clone: RAFL09-95-E07
AT4G24170	1.346217642	0.029289793	Arabidopsis thaliana ATP binding microtubule motor family protein mRNA, complete cds
AT1G47760	1.346217642	0.029289793	Arabidopsis thaliana protein agamous-like 102 mRNA, complete cds
AT1G53540	1.651072224	0.033145571	Arabidopsis thaliana chromosome 1 sequence
AT3G05152	1.651072224	0.033145571	Arabidopsis thaliana Full-length cDNA Complete sequence from clone GSLTSIL76ZB06 of Silique of strain col-0 of Arabidopsis thaliana (thale cress)
AT5G64395	1.651072224	0.033145571	Arabidopsis thaliana chromosome 5 sequence
AT1G67270	1.651072224	0.033145571	Arabidopsis thaliana zinc-finger domain of monoamine-oxidase A repressor R1

			protein mRNA, complete cds
AT4G16590	1.651072224	0.033145571	Arabidopsis thaliana cellulose synthase-like A01 mRNA, complete cds
AT4G37780	1.136499051	0.033941986	Arabidopsis thaliana myb domain protein 87 mRNA, complete cds
AT5G16850	1.236034724	0.035826919	Arabidopsis thaliana telomerase reverse transcriptase mRNA, complete cds
AT1G61440	1.236034724	0.035826919	Arabidopsis thaliana G-type lectin S-receptor-like serine/threonine-protein kinase mRNA, complete cds
AT4G39366	1.236034724	0.035826919	Arabidopsis thaliana chromosome 4 sequence
AT1G27820	3.388037818	0.036767383	Arabidopsis thaliana chromosome 1 sequence
AT2G22821	3.388037818	0.036767383	Arabidopsis thaliana chromosome 2, complete sequence
AT4G25510	3.388037818	0.036767383	Arabidopsis thaliana uncharacterized protein mRNA, complete cds
AT3G58420	3.388037818	0.036767383	Arabidopsis thaliana TRAF-like family protein mRNA, complete cds
AT5G66340	3.388037818	0.036767383	Arabidopsis thaliana uncharacterized protein mRNA, complete cds
AT1G33830	3.388037818	0.036767383	Arabidopsis thaliana P-loop containing nucleoside triphosphate hydrolases superfamily protein mRNA, complete cds
AT3G03702	3.388037818	0.036767383	Arabidopsis thaliana mRNA for unknown protein, complete cds, clone: RAFL19-12-M17
AT3G60170.1	3.388037818	0.036767383	Arabidopsis thaliana chromosome 3, complete sequence
AT1G50830	3.388037818	0.036767383	Arabidopsis thaliana chromosome 1 sequence
AT4G16560	3.388037818	0.036767383	Arabidopsis thaliana HSP20-like chaperone mRNA, complete cds
AT1G28220	3.388037818	0.036767383	Arabidopsis thaliana purine permease 3 mRNA, complete cds
AT4G16550	3.388037818	0.036767383	Arabidopsis thaliana HSP20-like chaperone mRNA, complete cds
AT5G52740	3.388037818	0.036767383	Arabidopsis thaliana copper transport family protein mRNA, complete cds
AT4G01380	3.388037818	0.036767383	Arabidopsis thaliana plastocyanin-like domain-containing protein mRNA, complete cds

AT1G64240.1	3.388037818	0.036767383	Arabidopsis thaliana chromosome 1 sequence
AT5G24355	3.388037818	0.036767383	Arabidopsis thaliana chromosome 5 sequence
AT3G23960	3.388037818	0.036767383	Arabidopsis thaliana chromosome 3, complete sequence
AT4G14368	1.803075317	0.03859026	Arabidopsis thaliana regulator of chromosome condensation repeat-containing protein mRNA, complete cds
AT1G58390	1.803075317	0.03859026	Arabidopsis thaliana CC-NBS-LRR class disease resistance protein mRNA, complete cds
AT2G45410	1.803075317	0.03859026	Arabidopsis thaliana LOB domain-containing protein 19 mRNA, complete cds
AT4G36740	1.803075317	0.03859026	Arabidopsis thaliana homeobox protein 40 mRNA, complete cds
AT5G51330	1.803075317	0.03859026	Arabidopsis thaliana protein DYAD mRNA, complete cds
AT5G55270	1.803075317	0.03859026	Arabidopsis thaliana chromosome 5 sequence
AT3G04420	1.066109723	0.039282472	Arabidopsis thaliana NAC domain containing protein 48 mRNA, complete cds
AT1G79360	1.066109723	0.039282472	Arabidopsis thaliana chromosome 1 sequence
AT2G45940	2.651072224	0.04196297	Arabidopsis thaliana uncharacterized protein mRNA, complete cds
AT2G21187	2.651072224	0.04196297	Arabidopsis thaliana chromosome 2, complete sequence
AT2G04852	2.651072224	0.04196297	Arabidopsis thaliana chromosome 2, complete sequence
AT2G22905	2.651072224	0.04196297	Arabidopsis thaliana chromosome 2, complete sequence
AT5G13940	2.651072224	0.04196297	Arabidopsis thaliana aminopeptidase mRNA, complete cds
AT4G04510	2.651072224	0.04196297	Arabidopsis thaliana cysteine-rich receptor-like protein kinase 38 mRNA, complete cds
AT4G10265	2.651072224	0.04196297	Arabidopsis thaliana chromosome 4 sequence
AT3G49580	2.651072224	0.04196297	Arabidopsis thaliana protein RESPONSE TO LOW SULFUR 1 mRNA, complete

			cds
AT5G38750	2.651072224	0.04196297	Arabidopsis thaliana asparaginyl-tRNA synthetase-like protein mRNA, complete cds
ATMG01030	2.651072224	0.04196297	Arabidopsis thaliana ecotype Landsberg erecta mitochondrion, complete genome
AT1G57780	2.651072224	0.04196297	Arabidopsis thaliana heavy-metal-associated domain-containing protein mRNA, complete cds
AT5G47170	2.651072224	0.04196297	Arabidopsis thaliana chromosome 5 sequence
AT4G03930	2.651072224	0.04196297	Arabidopsis thaliana putative pectinesterase/pectinesterase inhibitor 42 mRNA, complete cds
AT4G32205.1	2.651072224	0.04196297	Arabidopsis thaliana clone asmbl_13272 unknown mRNA sequence
AT1G53260	2.651072224	0.04196297	Arabidopsis thaliana uncharacterized protein mRNA, complete cds
AT2G04038	2.651072224	0.04196297	Arabidopsis thaliana chromosome 2, complete sequence
AT5G57690	2.651072224	0.04196297	Arabidopsis thaliana diacylglycerol kinase 4 mRNA, complete cds
AT5G28360	1.144112235	0.042551978	Arabidopsis thaliana 1-aminocyclopropane-1-carboxylate synthase mRNA, complete cds
AT5G50940	1.144112235	0.042551978	Arabidopsis thaliana RNA-binding KH domain-containing protein mRNA, complete cds
AT1G49220	1.144112235	0.042551978	Arabidopsis thaliana chromosome 1 sequence
ATCG00200	1.144112235	0.042551978	Aster spathulifolius chloroplast, complete genome
AT2G13810	2.066109723	0.04280713	Arabidopsis thaliana AGD2-like defense response protein 1 mRNA, complete cds
AT2G35200	2.066109723	0.04280713	Arabidopsis thaliana chromosome 2, complete sequence
AT3G48010	2.066109723	0.04280713	Arabidopsis thaliana cyclic nucleotide-gated channel 16 mRNA, complete cds
AT2G33000	2.066109723	0.04280713	Arabidopsis thaliana ubiquitin-associated (UBA)/TS-N domain-containing protein-like protein mRNA, complete cds
AT2G44590	2.066109723	0.04280713	Arabidopsis thaliana dynamin-related

			protein 1d mRNA, complete cds
AT1G59265.1	2.066109723	0.04280713	Arabidopsis thaliana chromosome 1 sequence
AT5G44990	2.066109723	0.04280713	Arabidopsis thaliana Glutathione S-transferase family protein mRNA, complete cds
AT5G22570	2.066109723	0.04280713	Arabidopsis thaliana putative WRKY transcription factor 38 mRNA, complete cds
AT4G04540	2.066109723	0.04280713	Arabidopsis thaliana putative cysteine-rich receptor-like protein kinase 39 mRNA, complete cds
AT3G44705.1	1.444621346	0.044355851	Arabidopsis thaliana chromosome 3, complete sequence
AT2G23348	1.444621346	0.044355851	Arabidopsis thaliana uncharacterized protein mRNA, complete cds
AT2G46567	1.444621346	0.044355851	Arabidopsis thaliana chromosome 2, complete sequence
AT4G03816.1	1.444621346	0.044355851	Arabidopsis thaliana chromosome 4 sequence
AT1G18100	1.444621346	0.044355851	Arabidopsis thaliana protein MOTHER of FT and TF 1 mRNA, complete cds
AT5G49370	1.004709178	0.044706391	Arabidopsis thaliana Pleckstrin homology (PH) domain-containing protein-like protein mRNA, complete cds
AT5G49380	1.258754801	0.044717251	Arabidopsis thaliana uncharacterized protein mRNA, complete cds
AT1G28680	1.258754801	0.044717251	Arabidopsis thaliana HXXXD-type acyl-transferase family protein mRNA, complete cds
AT1G14490	1.066109723	0.049380462	Arabidopsis thaliana chromosome 1 sequence
AT3G04440	1.066109723	0.049380462	Arabidopsis thaliana plasma-membrane choline transporter family protein mRNA, complete cds
AT4G36770	1.066109723	0.049380462	Arabidopsis thaliana chromosome 4 sequence
AT2G37880	1.153572564	0.053474806	Arabidopsis thaliana chromosome 2, complete sequence
AT3G43110	1.153572564	0.053474806	Arabidopsis thaliana chromosome 3, complete sequence
AT4G33800	1.153572564	0.053474806	Arabidopsis thaliana uncharacterized protein mRNA, complete cds
AT1G74400	1.153572564	0.053474806	Arabidopsis thaliana chromosome 1

			sequence
AT3G51570	1.153572564	0.053474806	Arabidopsis thaliana TIR-NBS-LRR class disease resistance protein mRNA, complete cds
AT1G05894	1.525541342	0.054105249	Arabidopsis thaliana chromosome 1 sequence
AT2G12450.1	1.525541342	0.054105249	Arabidopsis thaliana chromosome 2, complete sequence
AT5G53100	1.525541342	0.054105249	Arabidopsis thaliana Rossmann-fold NAD(P)-binding domain-containing protein mRNA, complete cds
AT4G35180	1.525541342	0.054105249	Arabidopsis thaliana LYS/HIS transporter 7 mRNA, complete cds
AT1G23465	1.288502144	0.055856619	Arabidopsis thaliana peptidase-S24/S26 domain-containing protein mRNA, complete cds
AT1G52920	1.066109723	0.062260007	Arabidopsis thaliana G-protein coupled receptor 2 mRNA, complete cds
AT3G28750	1.066109723	0.062260007	Arabidopsis thaliana chromosome 3, complete sequence
AT1G04150	1.066109723	0.062260007	Arabidopsis thaliana chromosome 1 sequence
AT1G64320	1.066109723	0.062260007	Arabidopsis thaliana myosin heavy chain-related protein mRNA, complete cds

Supplementary Data S2B Down regulated genes in *fc1*/Col-0 without NaCl treatment.

Gene	Log2FoldChange	pval	NT:Description
AT4G12550	-1.641314025	0	Arabidopsis thaliana chromosome 4 sequence
AT3G16240	-1.039583315	0	Arabidopsis thaliana aquaporin TIP2-1 mRNA, complete cds
AT2G37170	-1.164119236	0	Arabidopsis thaliana aquaporin PIP2-2 mRNA, complete cds
AT1G73330	-1.187880266	0	Arabidopsis thaliana chromosome 1 sequence
AT4G02380	-1.3124019	3.34E-250	Arabidopsis thaliana senescence-associated protein SAG21 mRNA, complete cds
AT3G01190	-1.155732262	1.42E-241	Arabidopsis thaliana peroxidase 27 mRNA, complete cds
AT1G78850	-1.013200923	2.52E-237	Arabidopsis thaliana chromosome 1 sequence
AT4G19690	-2.395313743	1.69E-233	Arabidopsis thaliana Fe(2+) transport protein 1 mRNA, complete cds
AT4G23400	-1.014697285	4.57E-220	Arabidopsis thaliana putative aquaporin PIP1-5 mRNA, complete cds
AT5G63160	-1.294242531	4.36E-215	Arabidopsis thaliana BTB and TAZ domain protein 1 mRNA, complete cds
AT5G43350	-1.278448485	2.18E-179	Arabidopsis thaliana inorganic phosphate transporter 1-1 mRNA, complete cds
AT2G33830	-1.397369828	5.41E-172	Arabidopsis thaliana dormancy/auxin associated protein mRNA, complete cds
AT2G40000	-1.538877889	6.78E-172	Arabidopsis thaliana chromosome 2, complete sequence
AT4G12520	-1.589878074	7.31E-171	Arabidopsis thaliana chromosome 4 sequence
AT3G48360	-1.002162298	7.36E-170	Arabidopsis thaliana TAC1-mediated telomerase activation pathway protein BT2 mRNA, complete cds
AT1G70850	-1.43563941	7.01E-166	Arabidopsis thaliana MLP-like protein 34 mRNA, complete cds
AT1G32640	-1.658422049	3.22E-151	Arabidopsis thaliana chromosome 1 sequence
AT5G53450	-1.542839458	6.86E-149	Arabidopsis thaliana OBP3-responsive protein 1 mRNA, complete cds
AT5G19240	-1.112723151	5.33E-147	Arabidopsis thaliana GPI-anchored glycoprotein membrane precursor mRNA, complete cds
AT2G28630	-1.310945368	1.81E-145	Arabidopsis thaliana chromosome 2, complete sequence
AT5G06320	-1.124356057	2.85E-134	Arabidopsis thaliana chromosome 5 sequence
AT1G55450	-1.166105341	1.43E-129	Arabidopsis thaliana

			S-adenosyl-L-methionine-dependent methyltransferases superfamily protein mRNA, complete cds
AT5G47450	-1.118224958	1.63E-128	Arabidopsis thaliana aquaporin TIP2-3 mRNA, complete cds
AT3G05950	-1.44376759	1.79E-126	Arabidopsis thaliana mRNA for germin-like protein, complete cds, clone: RAFL16-44-D15
AT1G01580	-1.809066252	1.51E-123	Arabidopsis thaliana ferric reduction oxidase 2 mRNA, complete cds
AT4G12545	-1.192665717	2.64E-122	Arabidopsis thaliana chromosome 4 sequence
AT1G19770	-1.153849349	4.81E-119	Arabidopsis thaliana purine permease 14 mRNA, complete cds
AT5G60660	-1.376567457	7.44E-114	Arabidopsis thaliana putative aquaporin PIP2-4 mRNA, complete cds
AT2G22500	-1.585386529	3.05E-107	Arabidopsis thaliana chromosome 2, complete sequence
AT1G47600	-2.864627615	3.25E-106	Arabidopsis thaliana myrosinase 4 mRNA, complete cds
AT5G17820	-1.122551841	2.00E-103	Arabidopsis thaliana peroxidase 57 mRNA, complete cds
AT5G26030	-2.36149645	2.12E-103	Arabidopsis thaliana ferrochelatase 1 mRNA, complete cds
AT1G51470	-2.912930658	4.03E-99	Arabidopsis thaliana myrosinase 5 mRNA, complete cds
AT3G44300	-2.279665114	1.60E-97	Arabidopsis thaliana nitrilase 2 mRNA, complete cds
AT1G66100	-1.407507802	3.06E-97	Arabidopsis thaliana probable thionin-2.4 mRNA, complete cds
AT5G46890	-1.456074361	4.44E-96	Arabidopsis thaliana chromosome 5 sequence
AT2G23810	-1.191990279	1.09E-91	Arabidopsis thaliana tetraspanin8 mRNA, complete cds
AT4G12720	-1.073293334	2.60E-88	Arabidopsis thaliana nudix hydrolase 7 mRNA, complete cds
AT3G44260	-2.544305957	4.22E-88	Arabidopsis thaliana chromosome 3, complete sequence
AT4G24570	-2.448149786	4.37E-88	Arabidopsis thaliana chromosome 4 sequence
AT5G48430	-1.06182119	5.19E-87	Arabidopsis thaliana chromosome 5 sequence
AT3G55980	-1.242304586	2.11E-86	Arabidopsis thaliana salt-inducible zinc finger 1 mRNA, complete cds
AT2G01530	-1.424525395	2.94E-86	Arabidopsis thaliana MLP-like protein 329 mRNA, complete cds
AT5G63600	-1.041009122	4.08E-86	Arabidopsis thaliana flavonol synthase 5

			mRNA, complete cds
AT5G46900	-1.53776124	1.20E-83	Arabidopsis thaliana chromosome 5 sequence
AT4G00970	-1.285565715	1.84E-81	Arabidopsis thaliana cysteine-rich receptor-like protein kinase 41 mRNA, complete cds
AT3G56880	-1.10361712	3.20E-79	Arabidopsis thaliana chromosome 3, complete sequence
AT2G30930	-1.224651412	7.49E-79	Arabidopsis thaliana uncharacterized protein mRNA, complete cds
AT5G54370	-2.031187478	1.08E-78	Arabidopsis thaliana late embryogenesis abundant protein-like protein mRNA, complete cds
AT1G27730	-2.604967936	3.71E-77	Arabidopsis thaliana chromosome 1 sequence
AT4G18205	-1.147943398	8.95E-75	Arabidopsis thaliana nucleotide-sugar transporter family protein mRNA, complete cds
AT1G52070	-2.320725635	1.04E-74	Arabidopsis thaliana jacalin-like lectin domain-containing protein mRNA, complete cds
AT2G37130	-1.127178353	2.32E-72	Arabidopsis thaliana peroxidase mRNA, complete cds
AT5G61600	-1.679706789	3.52E-72	Arabidopsis thaliana chromosome 5 sequence
AT4G29780	-2.302697064	1.29E-70	Arabidopsis thaliana chromosome 4 sequence
AT1G52060	-2.07046333	3.33E-68	Arabidopsis thaliana jacalin-like lectin domain-containing protein mRNA, complete cds
AT1G74670	-1.032574552	1.82E-66	Arabidopsis thaliana gibberellin-regulated protein 6 mRNA, complete cds
AT3G19030	-1.357281055	1.88E-66	Arabidopsis thaliana chromosome 3, complete sequence
AT4G27280	-1.819985822	1.58E-65	Arabidopsis thaliana chromosome 4 sequence
AT4G33120	-1.782684473	2.57E-65	Arabidopsis thaliana S-adenosyl-L-methionine-dependent methyltransferases superfamily protein mRNA, complete cds
AT1G52050	-2.91163747	6.93E-63	Arabidopsis thaliana jacalin-like lectin domain-containing protein mRNA, complete cds
AT5G05250	-1.838030262	9.97E-62	Arabidopsis thaliana chromosome 5 sequence
AT2G24600	-1.207614961	2.24E-60	Arabidopsis thaliana Ankyrin repeat family protein mRNA, complete cds
AT2G32150	-1.283648215	7.93E-60	Arabidopsis thaliana haloacid dehalogenase-like hydrolase domain-containing protein mRNA, complete

			cds
AT3G22060	-1.401833396	1.10E-59	Arabidopsis thaliana Receptor-like protein kinase-related family protein mRNA, complete cds
AT5G45340	-1.214720266	1.92E-58	Arabidopsis thaliana abscisic acid 8'-hydroxylase 3 mRNA, complete cds
AT3G29410	-1.100980349	1.14E-55	Arabidopsis thaliana terpenoid synthase 25 mRNA, complete cds
AT2G45220	-1.327554126	3.08E-55	Arabidopsis thaliana putative pectinesterase/pectinesterase inhibitor 17 mRNA, complete cds
AT5G26130	-1.840160225	4.82E-55	Arabidopsis thaliana chromosome 5 sequence
AT4G37610	-1.014614214	2.78E-54	Arabidopsis thaliana BTB and TAZ domain protein 5 mRNA, complete cds
AT3G16440	-2.466924986	1.98E-52	Arabidopsis thaliana myrosinase-binding protein-like protein-300B mRNA, complete cds
AT3G51330	-1.030330037	2.16E-52	Arabidopsis thaliana aspartyl protease family protein mRNA, complete cds
AT4G11290	-1.057150986	3.46E-52	Arabidopsis thaliana peroxidase 39 mRNA, complete cds
AT1G06090	-2.563749316	1.71E-51	Arabidopsis thaliana delta-9 desaturase-like 1 protein mRNA, complete cds
AT5G13740	-1.026483076	4.62E-51	Arabidopsis thaliana zinc induced facilitator 1 proetin mRNA, complete cds
AT1G56430	-1.087115476	7.74E-51	Arabidopsis thaliana chromosome 1 sequence
AT3G12900	-2.015278604	7.88E-51	Arabidopsis thaliana oxidoreductase, 2OG-Fe(II) oxygenase family protein mRNA, complete cds
AT3G52400	-1.122029276	1.08E-49	Arabidopsis thaliana syntaxin-122 mRNA, complete cds
AT4G36500	-1.004619103	2.54E-48	Arabidopsis thaliana chromosome 4 sequence
AT5G57560	-1.248606885	2.62E-48	Arabidopsis thaliana xyloglucan endotransglucosylase/hydrolase protein 22 mRNA, complete cds
AT3G25930	-1.158234925	3.86E-47	Arabidopsis thaliana Adenine nucleotide alpha hydrolases-like superfamily protein mRNA, complete cds
AT5G60530	-1.618919733	2.28E-46	Arabidopsis thaliana late embryogenesis abundant protein-like protein mRNA, complete cds
AT5G44610	-1.010163019	3.94E-46	Arabidopsis thaliana microtubule-associated protein 18 mRNA, complete cds
AT5G37770	-1.164886764	5.04E-46	Arabidopsis thaliana chromosome 5 sequence

AT3G24300	-1.37056369	5.26E-46	Arabidopsis thaliana chromosome 3, complete sequence
AT1G23020	-1.177733863	1.58E-45	Arabidopsis thaliana ferric reduction oxidase 3 mRNA, complete cds
AT4G11280	-1.459959089	1.93E-44	Arabidopsis thaliana 1-aminocyclopropane-1-carboxylate synthase 6 mRNA, complete cds
AT3G22600	-1.023461018	1.14E-43	Arabidopsis thaliana protease inhibitor/seed storage/lipid transfer protein (LTP) family protein mRNA, complete cds
AT2G14878	-1.184952041	5.37E-43	Arabidopsis thaliana chromosome 2, complete sequence
AT3G07720	-1.126980563	1.51E-42	Arabidopsis thaliana galactose oxidase/kelch repeat-containing protein mRNA, complete cds
AT3G50740	-1.219627734	4.48E-42	Arabidopsis thaliana chromosome 3, complete sequence
AT2G33790	-1.135362047	1.97E-41	Arabidopsis thaliana arabinogalactan protein 30 mRNA, complete cds
AT1G73260	-2.024210884	3.20E-41	Arabidopsis thaliana chromosome 1 sequence
AT1G13609	-3.692335599	3.29E-41	Arabidopsis thaliana defensin-like protein 287 mRNA, complete cds
AT1G57990	-1.007251728	5.75E-41	Arabidopsis thaliana chromosome 1 sequence
AT5G12940	-1.407306125	1.59E-40	Arabidopsis thaliana chromosome 5 sequence
AT1G21100	-1.026421563	1.62E-39	Arabidopsis thaliana indole glucosinolate o-methyltransferase 1 mRNA, complete cds
AT2G28160	-1.404655924	1.75E-39	Arabidopsis thaliana FER-LIKE IRON DEFICIENCY-INDUCED transcription factor mRNA, complete cds
AT1G65845	-1.280646509	4.18E-38	Arabidopsis thaliana uncharacterized protein mRNA, complete cds
AT1G80840	-2.401016288	5.19E-38	Arabidopsis thaliana putative WRKY transcription factor 40 mRNA, complete cds
AT3G29250	-1.321815931	1.38E-36	Arabidopsis thaliana short-chain dehydrogenase reductase 4 mRNA, complete cds
AT1G73540	-1.347309498	2.12E-36	Arabidopsis thaliana nudix hydrolase 21 mRNA, complete cds
AT1G64980	-1.043173927	2.26E-36	Arabidopsis thaliana putative nucleotide-diphospho-sugar transferase mRNA, complete cds
AT1G07750	-1.295474572	3.53E-36	Arabidopsis thaliana cupin domain-containing protein mRNA, complete cds
AT2G14247	-4.859889696	6.73E-36	Arabidopsis thaliana chromosome 2,

			complete sequence
AT1G29025	-1.024345228	7.01E-36	Arabidopsis thaliana Calcium-binding EF-hand family protein mRNA, complete cds
AT5G51190	-2.184265111	1.37E-35	Arabidopsis thaliana chromosome 5 sequence
AT2G29350	-2.524705504	2.16E-35	Arabidopsis thaliana senescence-associated protein 13 mRNA, complete cds
AT1G53830	-1.551642713	8.02E-34	Arabidopsis thaliana pectin methylesterase 2 mRNA, complete cds
AT2G22122	-1.089081648	3.22E-33	Arabidopsis thaliana chromosome 2, complete sequence
AT1G33610	-1.224441569	1.30E-32	Arabidopsis thaliana leucine-rich repeat (LRR) family protein mRNA, complete cds
AT5G10580	-1.025812767	2.30E-32	Arabidopsis thaliana uncharacterized protein mRNA, complete cds
AT2G39200	-1.077254452	4.77E-32	Arabidopsis thaliana protein MILDEW RESISTANCE LOCUS O 12 mRNA, complete cds
AT2G36100	-1.120230778	1.25E-31	Arabidopsis thaliana casparian strip membrane protein 1 mRNA, complete cds
AT1G19180	-1.331156916	5.29E-31	Arabidopsis thaliana chromosome 1 sequence
AT1G22500	-1.323342366	2.77E-30	Arabidopsis thaliana chromosome 1 sequence
AT3G56980	-3.548600121	1.78E-29	Arabidopsis thaliana transcription factor ORG3 mRNA, complete cds
AT1G49500	-1.136927766	2.64E-29	Arabidopsis thaliana uncharacterized protein mRNA, complete cds
AT4G13620	-1.554476688	2.10E-28	Arabidopsis thaliana chromosome 4 sequence
AT3G18560	-1.200098931	2.50E-28	Arabidopsis thaliana chromosome 3, complete sequence
AT1G72910	-1.162159265	2.68E-28	Arabidopsis thaliana Toll-Interleukin-Resistance domain-containing protein mRNA, complete cds
AT5G04200	-1.507625522	2.88E-28	Arabidopsis thaliana chromosome 5 sequence
AT1G61890	-1.065239699	4.51E-28	Arabidopsis thaliana MATE efflux family protein mRNA, complete cds
AT1G29280	-1.010061958	1.58E-27	Arabidopsis thaliana putative WRKY transcription factor 65 mRNA, complete cds
AT3G46620	-1.252900889	1.76E-27	Arabidopsis thaliana chromosome 3, complete sequence
AT1G26820	-1.741245199	2.57E-27	Arabidopsis thaliana ribonuclease 3 mRNA, complete cds
AT3G32030	-1.233008489	3.01E-27	Arabidopsis thaliana terpenoid synthase 30 mRNA, complete cds

AT2G26530	-1.331545698	6.15E-27	Arabidopsis thaliana chromosome 2, complete sequence
AT4G21830	-1.029347436	1.14E-26	Arabidopsis thaliana peptide methionine sulfoxide reductase B7 mRNA, complete cds
AT3G61060	-1.232069769	3.37E-26	Arabidopsis thaliana phloem protein 2-A13 mRNA, complete cds
AT2G27830	-1.30450031	5.55E-26	Arabidopsis thaliana chromosome 2, complete sequence
AT2G41240	-4.009178404	5.91E-26	Arabidopsis thaliana transcription factor bHLH100 mRNA, complete cds
AT5G57480	-1.33525284	6.84E-26	Arabidopsis thaliana chromosome 5 sequence
AT3G45710	-1.191188313	6.87E-26	Arabidopsis thaliana putative nitrate excretion transporter 6 mRNA, complete cds
AT1G18300	-1.630821209	1.38E-25	Arabidopsis thaliana nudix hydrolase 4 mRNA, complete cds
AT5G05600	-1.790526102	2.10E-25	Arabidopsis thaliana oxidoreductase, 2OG-Fe(II) oxygenase family protein mRNA, complete cds
AT1G74450	-1.313104113	9.10E-25	Arabidopsis thaliana chromosome 1 sequence
AT4G15760	-1.149525332	2.53E-24	Arabidopsis thaliana monooxygenase 1 mRNA, complete cds
AT2G42060	-1.456925029	3.53E-24	Arabidopsis thaliana chromosome 2, complete sequence
AT1G56010	-1.021051078	4.08E-24	Arabidopsis thaliana transcription factor NAC1 mRNA, complete cds
AT3G25780	-1.038101272	7.61E-24	Arabidopsis thaliana allene oxide cyclase 3 mRNA, complete cds
AT5G66390	-1.055880801	1.03E-23	Arabidopsis thaliana peroxidase 72 mRNA, complete cds
AT1G78460	-1.159310391	1.05E-23	Arabidopsis thaliana SOUL heme-binding protein mRNA, complete cds
AT5G24410	-1.183783583	1.36E-23	Arabidopsis thaliana 6-phosphogluconolactonase 4 mRNA, complete cds
AT4G23220	-1.162421836	1.60E-23	Arabidopsis thaliana cysteine-rich receptor-like protein kinase 14 mRNA, complete cds
AT4G17490	-1.518852778	1.64E-23	Arabidopsis thaliana chromosome 4 sequence
AT3G51350	-1.080731665	2.07E-23	Arabidopsis thaliana aspartyl protease family protein mRNA, complete cds
AT3G15210	-1.188631476	2.38E-23	Arabidopsis thaliana chromosome 3, complete sequence
AT2G41660	-1.289155556	3.47E-23	Arabidopsis thaliana chromosome 2, complete sequence

AT3G52450	-1.354222076	4.08E-23	Arabidopsis thaliana chromosome 3, complete sequence
AT3G62020	-1.220771425	4.42E-23	Arabidopsis thaliana chromosome 3, complete sequence
AT1G44350	-2.126535355	5.17E-23	Arabidopsis thaliana IAA-amino acid hydrolase ILR1-like 6 mRNA, complete cds
AT5G17350	-2.119001682	8.33E-23	Arabidopsis thaliana chromosome 5 sequence
AT5G64120	-1.528836866	3.65E-22	Arabidopsis thaliana peroxidase 71 mRNA, complete cds
AT4G15093	-1.039326552	5.50E-22	Arabidopsis thaliana extradiol ring-cleavage dioxygenase mRNA, complete cds
AT4G10310	-1.029542516	7.29E-22	Arabidopsis thaliana sodium transporter HKT1 mRNA, complete cds
AT5G02090	-2.198593503	1.03E-21	Arabidopsis thaliana chromosome 5 sequence
AT3G25760	-1.873629752	1.53E-21	Arabidopsis thaliana allene oxide cyclase 1 mRNA, complete cds
AT1G05675	-1.44846345	3.10E-21	Arabidopsis thaliana UDP-Glycosyltransferase superfamily protein mRNA, complete cds
AT2G35710	-1.204192683	3.40E-21	Arabidopsis thaliana putative glucuronosyltransferase PGSIP8 mRNA, complete cds
AT3G56970	-3.606315619	3.44E-21	Arabidopsis thaliana transcription factor ORG2 mRNA, complete cds
AT1G11540	-1.064850955	3.73E-21	Arabidopsis thaliana Sulfite exporter TauE/SafE family protein mRNA, complete cds
AT5G04340	-1.738070951	5.99E-21	Arabidopsis thaliana chromosome 5 sequence
AT1G14200	-1.052363747	1.06E-20	Arabidopsis thaliana chromosome 1 sequence
AT1G62280	-1.933890277	1.57E-20	Arabidopsis thaliana S-type anion channel SLAH1 mRNA, complete cds
AT5G52750	-1.249836214	1.91E-20	Arabidopsis thaliana heavy metal transport/detoxification domain-containing protein mRNA, complete cds
AT4G11210	-1.652708525	2.05E-20	Arabidopsis thaliana chromosome 4 sequence
AT5G42180	-1.188788468	2.30E-20	Arabidopsis thaliana peroxidase mRNA, complete cds
AT1G65310	-1.253799204	2.50E-20	Arabidopsis thaliana probable xyloglucan endotransglucosylase/hydrolase protein 17 mRNA, complete cds
AT3G19580	-1.266160316	3.03E-20	Arabidopsis thaliana chromosome 3, complete sequence
AT2G39430	-1.345867977	5.52E-20	Arabidopsis thaliana chromosome 2, complete sequence

AT4G29740	-1.031258114	8.27E-20	Arabidopsis thaliana cytokinin dehydrogenase 4 mRNA, complete cds
AT3G05200	-1.097928435	8.99E-20	Arabidopsis thaliana chromosome 3, complete sequence
AT5G15180	-1.131620644	1.06E-19	Arabidopsis thaliana peroxidase 56 mRNA, complete cds
AT4G33790	-1.895822236	1.49E-19	Arabidopsis thaliana fatty acyl-CoA reductase CER4 mRNA, complete cds
AT1G72940	-1.06672596	4.21E-19	Arabidopsis thaliana Toll-Interleukin-Resistance domain-containing protein mRNA, complete cds
AT3G21550	-1.16926834	4.48E-19	Arabidopsis thaliana chromosome 3, complete sequence
AT3G16720	-1.020961985	5.55E-19	Arabidopsis thaliana chromosome 3, complete sequence
AT5G27420	-1.174898377	7.87E-19	Arabidopsis thaliana chromosome 5 sequence
AT4G21250	-1.011196003	1.14E-18	Arabidopsis thaliana Sulfite exporter TauE/SafE family protein mRNA, complete cds
AT3G49190	-3.118314848	1.33E-18	Arabidopsis thaliana O-acyltransferase (WSD1-like) family protein mRNA, complete cds
AT4G23170	-1.046000643	2.16E-18	Arabidopsis thaliana chromosome 4 sequence
AT5G47220	-2.143343643	2.88E-18	Arabidopsis thaliana chromosome 5 sequence
AT3G06460	-2.410328321	3.23E-18	Arabidopsis thaliana chromosome 3, complete sequence
AT5G47950	-1.900723413	3.62E-18	Arabidopsis thaliana chromosome 5 sequence
AT4G22666	-1.098277095	3.65E-18	Arabidopsis thaliana Bifunctional inhibitor/lipid-transfer protein/seed storage 2S albumin superfamily protein mRNA, complete cds
AT1G66090	-1.150919514	5.38E-18	Arabidopsis thaliana TIR-NBS class of disease resistance protein mRNA, complete cds
AT1G30870	-1.023441862	5.96E-18	Arabidopsis thaliana peroxidase 7 mRNA, complete cds
AT2G20670	-1.023441862	5.96E-18	Arabidopsis thaliana uncharacterized protein mRNA, complete cds
AT5G19970	-1.154917156	8.25E-18	Arabidopsis thaliana chromosome 5 sequence
AT5G65080	-3.166551034	8.34E-18	Arabidopsis thaliana protein MADS AFFECTING FLOWERING 5 mRNA, complete cds
AT1G23710	-1.163228764	9.17E-18	Arabidopsis thaliana chromosome 1 sequence

AT1G72520	-1.425030755	9.41E-18	Arabidopsis thaliana lipoxygenase 4 mRNA, complete cds
AT1G53940	-1.233098296	1.08E-17	Arabidopsis thaliana GDSL-motif lipase 2 mRNA, complete cds
AT3G14680	-1.044637343	1.72E-17	Arabidopsis thaliana cytochrome P450, family 72, subfamily A, polypeptide 14 mRNA, complete cds
AT1G72920	-1.605009422	2.38E-17	Arabidopsis thaliana Toll-Interleukin-Resistance domain-containing protein mRNA, complete cds
AT5G14650	-1.334852591	2.55E-17	Arabidopsis thaliana pectin lyase-like superfamily protein mRNA, complete cds
AT1G05650	-1.44846345	2.66E-17	Arabidopsis thaliana pectin lyase-like protein mRNA, complete cds
AT2G24710	-1.082920054	3.01E-17	Arabidopsis thaliana glutamate receptor 2.3 mRNA, complete cds
AT5G06200	-1.658577542	3.76E-17	Arabidopsis thaliana uncharacterized protein mRNA, complete cds
AT4G23870	-1.206143213	4.31E-17	Arabidopsis thaliana chromosome 4 sequence
AT5G03570	-1.481378072	5.04E-17	Arabidopsis thaliana nickel transport protein FPN2 mRNA, complete cds
AT3G58810	-1.229780511	5.81E-17	Arabidopsis thaliana chromosome 3, complete sequence
AT5G59820	-1.745280712	6.14E-17	Arabidopsis thaliana chromosome 5 sequence
AT1G47400	-2.506141726	6.14E-17	Arabidopsis thaliana chromosome 1 sequence
AT4G25250	-1.295711099	8.89E-17	Arabidopsis thaliana chromosome 4 sequence
AT3G28340	-1.65333811	9.20E-17	Arabidopsis thaliana chromosome 3, complete sequence
AT3G29035	-1.847475525	1.30E-16	Arabidopsis thaliana NAC domain-containing protein 3 mRNA, complete cds
AT1G73500	-1.243434648	1.31E-16	Arabidopsis thaliana chromosome 1 sequence
AT4G12510	-1.127770011	2.12E-16	Arabidopsis thaliana chromosome 4 sequence
AT4G37370	-1.40714239	2.23E-16	Arabidopsis thaliana cytochrome P450, family 81, subfamily D, polypeptide 8 mRNA, complete cds
AT3G04010	-1.630498134	3.15E-16	Arabidopsis thaliana O-glycosyl hydrolases family 17 protein mRNA, complete cds
AT2G27370	-1.60442851	4.04E-16	Arabidopsis thaliana uncharacterized protein mRNA, complete cds
AT1G73300	-1.44846345	5.48E-16	Arabidopsis thaliana serine carboxypeptidase-like 2 mRNA, complete cds
AT5G42590	-1.44846345	5.48E-16	Arabidopsis thaliana cytochrome P450 71A16

			mRNA, complete cds
AT1G08320	-1.077192755	5.89E-16	Arabidopsis thaliana bZIP transcription factor family protein mRNA, complete cds
AT4G26050	-1.065715489	5.97E-16	Arabidopsis thaliana plant intracellular ras group-related LRR 8 mRNA, complete cds
AT4G36850	-1.634329995	8.31E-16	Arabidopsis thaliana PQ-loop repeat family protein / transmembrane family protein mRNA, complete cds
AT2G36220	-1.156282698	8.38E-16	Arabidopsis thaliana chromosome 2, complete sequence
AT1G70690	-1.035674015	9.32E-16	Arabidopsis thaliana plasmodesmata-located protein 5 mRNA, complete cds
AT5G39020	-1.269757563	1.69E-15	Arabidopsis thaliana chromosome 5 sequence
AT5G23840	-1.060562081	1.96E-15	Arabidopsis thaliana MD-2-related lipid recognition domain-containing protein mRNA, complete cds
AT3G44860	-3.538752335	2.09E-15	Arabidopsis thaliana farnesoic acid carboxyl-O-methyltransferase mRNA, complete cds
AT1G63295	-1.355132926	2.26E-15	Arabidopsis thaliana Remorin family protein mRNA, complete cds
AT3G16690	-1.323217657	2.47E-15	Arabidopsis thaliana bidirectional sugar transporter SWEET16 mRNA, complete cds
AT1G69890	-1.034584277	2.63E-15	Arabidopsis thaliana uncharacterized protein mRNA, complete cds
AT1G50560	-1.100448427	2.88E-15	Arabidopsis thaliana cytochrome P450, family 705, subfamily A, polypeptide 25 mRNA, complete cds
AT2G27080	-1.057122043	3.07E-15	Arabidopsis thaliana chromosome 2, complete sequence
AT1G66800	-1.504206002	3.73E-15	Arabidopsis thaliana alcohol dehydrogenase-like protein mRNA, complete cds
AT5G40730	-1.351047828	5.93E-15	Arabidopsis thaliana chromosome 5 sequence
AT1G08500	-1.550561638	6.64E-15	Arabidopsis thaliana early nodulin-like protein 18 mRNA, complete cds
AT4G15290	-1.407061952	8.38E-15	Arabidopsis thaliana cellulose synthase-like protein B5 mRNA, complete cds
AT3G48340	-1.119635796	1.21E-14	Arabidopsis thaliana KDEL-tailed cysteine endopeptidase CEP2 mRNA, complete cds
AT1G73280	-1.543100324	1.61E-14	Arabidopsis thaliana serine carboxypeptidase-like 3 mRNA, complete cds
AT4G17500	-1.317495269	1.92E-14	Arabidopsis thaliana chromosome 4 sequence
AT5G06839	-1.355591507	1.99E-14	Arabidopsis thaliana bZIP transcription factor

			TGA10 mRNA, complete cds
AT5G37450	-1.087460666	2.04E-14	Arabidopsis thaliana probable LRR receptor-like serine/threonine-protein kinase mRNA, complete cds
AT3G26500	-1.165215823	3.40E-14	Arabidopsis thaliana plant intracellular ras group-related LRR 2 mRNA, complete cds
AT1G49000	-1.487991814	3.49E-14	Arabidopsis thaliana chromosome 1 sequence
AT1G17190	-1.02964926	4.40E-14	Arabidopsis thaliana glutathione S-transferase tau 26 mRNA, complete cds
AT5G59260	-1.788039411	4.43E-14	Arabidopsis thaliana chromosome 5 sequence
AT3G55970	-3.864627615	7.03E-14	Arabidopsis thaliana jasmonate-regulated protein JRG21 mRNA, complete cds
AT5G45080	-1.112764235	7.39E-14	Arabidopsis thaliana protein PHLOEM PROTEIN 2-LIKE A6 mRNA, complete cds
AT4G35770	-1.830054466	7.65E-14	Arabidopsis thaliana senescence-associated protein DIN1 mRNA, complete cds
AT5G15150	-1.13028749	8.16E-14	Arabidopsis thaliana mRNA for homeobox protein, complete cds, clone: RAFL16-61-C06
AT5G22250	-1.707297867	8.50E-14	Arabidopsis thaliana chromosome 5 sequence
AT5G08240	-1.158105434	8.60E-14	Arabidopsis thaliana uncharacterized protein mRNA, complete cds
AT1G65390	-1.281813581	9.68E-14	Arabidopsis thaliana protein PHLOEM protein 2-LIKE A5 mRNA, complete cds
AT3G26610	-1.150848812	1.03E-13	Arabidopsis thaliana putative polygalacturonase / pectinase mRNA, complete cds
AT2G25810	-1.071393801	1.07E-13	Arabidopsis thaliana aquaporin TIP4-1 mRNA, complete cds
AT1G53680	-1.143682662	1.22E-13	Arabidopsis thaliana glutathione S-transferase TAU 28 mRNA, complete cds
AT4G15330	-1.017413633	1.42E-13	Arabidopsis thaliana cytochrome P450, family 705, subfamily A, polypeptide 1 mRNA, complete cds
AT3G05920	-1.64873306	1.60E-13	Arabidopsis thaliana heavy-metal-associated domain-containing protein mRNA, complete cds
AT1G30750	-1.237282421	1.70E-13	Arabidopsis thaliana chromosome 1 sequence
AT2G39040	-1.339529078	1.97E-13	Arabidopsis thaliana peroxidase 24 mRNA, complete cds
AT2G23030	-1.493761797	2.73E-13	Arabidopsis thaliana serine/threonine-protein kinase SNRK2.9 mRNA, complete cds
AT4G27400	-2.354222076	4.57E-13	Arabidopsis thaliana late embryogenesis abundant protein-like protein mRNA,

			complete cds
AT5G04150	-4.606315619	7.19E-13	Arabidopsis thaliana transcription factor bHLH101 mRNA, complete cds
AT5G43370	-1.832010663	7.61E-13	Arabidopsis thaliana phosphate transporter Pht1;2 mRNA, complete cds
AT1G73270	-1.416041972	1.06E-12	Arabidopsis thaliana serine carboxypeptidase-like 6 mRNA, complete cds
AT5G38100	-1.884980677	1.28E-12	Arabidopsis thaliana putative S-adenosylmethionine-dependent methyltransferase mRNA, complete cds
AT3G09020	-1.273520374	1.37E-12	Arabidopsis thaliana chromosome 3, complete sequence
AT5G38820	-2.999979468	1.49E-12	Arabidopsis thaliana putative amino acid transporter mRNA, complete cds
AT1G66160	-1.528251476	1.76E-12	Arabidopsis thaliana chromosome 1 sequence
AT4G34410	-5.488479129	1.86E-12	Arabidopsis thaliana chromosome 4 sequence
AT5G06570	-1.028354961	2.10E-12	Arabidopsis thaliana probable carboxylesterase 15 mRNA, complete cds
AT2G32300	-1.070309475	2.10E-12	Arabidopsis thaliana uclacyanin 1 mRNA, complete cds
AT4G15400	-1.164670484	2.65E-12	Arabidopsis thaliana chromosome 4 sequence
AT5G24070	-2.089168503	2.99E-12	Arabidopsis thaliana probable peroxidase 61 mRNA, complete cds
AT2G21020	-1.160525922	4.21E-12	Arabidopsis thaliana chromosome 2, complete sequence
AT1G28370	-2.741245199	4.79E-12	Arabidopsis thaliana chromosome 1 sequence
AT3G52520	-1.362733576	5.25E-12	Arabidopsis thaliana chromosome 3, complete sequence
AT1G74930	-2.272692191	5.63E-12	Arabidopsis thaliana chromosome 1 sequence
AT3G11550	-1.002227407	7.72E-12	Arabidopsis thaliana uncharacterized protein mRNA, complete cds
AT2G25735	-1.109967506	1.02E-11	Arabidopsis thaliana chromosome 2, complete sequence
AT1G22550	-1.113213977	1.37E-11	Arabidopsis thaliana putative peptide/nitrate transporter mRNA, complete cds
AT2G40095	-1.103815279	1.40E-11	Arabidopsis thaliana alpha/beta hydrolase related protein mRNA, complete cds
AT5G47240	-1.550561638	1.51E-11	Arabidopsis thaliana nudix hydrolase 8 mRNA, complete cds
AT4G31380	-1.550561638	1.51E-11	Arabidopsis thaliana chromosome 4 sequence
AT1G29395	-1.585966974	1.97E-11	Arabidopsis thaliana cold regulated 314 inner membrane 1 mRNA, complete cds
AT5G47230	-1.189835258	2.31E-11	Arabidopsis thaliana chromosome 5 sequence

AT4G32280	-1.0654199	2.66E-11	Arabidopsis thaliana auxin-responsive protein IAA29 mRNA, complete cds
AT3G49845	-1.222566566	2.75E-11	Arabidopsis thaliana uncharacterized protein mRNA, complete cds
AT1G27140	-2.374462869	2.98E-11	Arabidopsis thaliana glutathione S-transferase tau 14 mRNA, complete cds
AT1G55152	-1.07506634	3.07E-11	Arabidopsis thaliana chromosome 1 sequence
AT1G47603	-1.68725031	3.35E-11	Arabidopsis thaliana purine permease 19 mRNA, complete cds
AT3G06390	-1.158596564	3.48E-11	Arabidopsis thaliana uncharacterized protein mRNA, complete cds
AT1G23830	-1.451289494	3.88E-11	Arabidopsis thaliana chromosome 1 sequence
AT5G47990	-1.563940667	4.40E-11	Arabidopsis thaliana cytochrome P450 705A5 mRNA, complete cds
AT1G14250	-1.20441922	4.66E-11	Arabidopsis thaliana probable apyrase 5 mRNA, complete cds
AT5G26340	-1.210730482	6.17E-11	Arabidopsis thaliana sugar transport protein 13 mRNA, complete cds
AT1G59590	-1.228633543	6.68E-11	Arabidopsis thaliana chromosome 1 sequence
AT3G19430	-1.646608325	7.20E-11	Arabidopsis thaliana late embryogenesis abundant protein-like protein mRNA, complete cds
AT3G61190	-2.017306285	7.93E-11	Arabidopsis thaliana BON association protein 1 mRNA, complete cds
AT4G19370	-1.367786804	8.59E-11	Arabidopsis thaliana uncharacterized protein mRNA, complete cds
AT2G45550	-1.374462869	9.70E-11	Arabidopsis thaliana cytochrome P450 76C4 mRNA, complete cds
AT2G39330	-1.013889599	1.57E-10	Arabidopsis thaliana jacalin-related lectin 23 mRNA, complete cds
AT3G16390	-1.14655116	1.62E-10	Arabidopsis thaliana Nitrile-specifier protein 3 mRNA, complete cds
AT2G39650	-1.355354046	2.01E-10	Arabidopsis thaliana uncharacterized protein mRNA, complete cds
AT3G05155	-1.208252689	2.10E-10	Arabidopsis thaliana mRNA for hypothetical protein, complete cds, clone: RAFL14-21-B09
AT2G44220	-1.707614421	2.33E-10	Arabidopsis thaliana uncharacterized protein mRNA, complete cds
AT5G42250	-1.023686947	2.48E-10	Arabidopsis thaliana alcohol dehydrogenase-like 7 mRNA, complete cds
AT1G07135	-1.770391545	2.74E-10	Arabidopsis thaliana chromosome 1 sequence
AT1G23850	-1.001867071	2.80E-10	Arabidopsis thaliana chromosome 1 sequence

AT4G22460	-1.844622939	3.06E-10	Arabidopsis thaliana chromosome 4 sequence
AT3G15300	-1.131829655	3.07E-10	Arabidopsis thaliana chromosome 3, complete sequence
AT1G14185	-1.568605813	3.13E-10	Arabidopsis thaliana glucose-methanol-choline oxidoreductase-like protein mRNA, complete cds
AT3G03500	-1.390747952	3.69E-10	Arabidopsis thaliana TatD related DNase mRNA, complete cds
AT1G47395	-1.778915617	4.43E-10	Arabidopsis thaliana chromosome 1 sequence
AT1G21910	-1.473409807	6.24E-10	Arabidopsis thaliana chromosome 1 sequence
AT5G62420	-3.196924683	6.91E-10	Arabidopsis thaliana aldo/keto reductase family protein mRNA, complete cds
AT4G15380	-1.005551262	6.92E-10	Arabidopsis thaliana cytochrome P450, family 705, subfamily A, polypeptide 4 mRNA, complete cds
AT2G44600	-1.021353118	7.07E-10	Arabidopsis thaliana chromosome 2, complete sequence
AT5G24100	-1.075664238	7.09E-10	Arabidopsis thaliana Leucine-rich repeat protein kinase family protein mRNA, complete cds
AT1G66440	-2.143343643	7.13E-10	Arabidopsis thaliana chromosome 1 sequence
AT1G76590	-1.19023003	7.52E-10	Arabidopsis thaliana PLATZ transcription factor family protein mRNA, complete cds
AT2G32620	-2.371295589	8.10E-10	Arabidopsis thaliana cellulose synthase-like protein B2 mRNA, complete cds
AT3G45730	-1.10008256	9.31E-10	Arabidopsis thaliana chromosome 3, complete sequence
AT4G31320	-1.058781525	9.66E-10	Arabidopsis thaliana chromosome 4 sequence
AT1G65970	-1.328749894	1.09E-09	Arabidopsis thaliana thioredoxin-dependent peroxidase 2 mRNA, complete cds
AT1G74660	-1.255818372	1.09E-09	Arabidopsis thaliana chromosome 1 sequence
AT1G58420	-2.123714836	1.16E-09	Arabidopsis thaliana chromosome 1 sequence
AT5G10280	-1.127662021	1.20E-09	Arabidopsis thaliana myb domain protein 92 mRNA, complete cds
AT3G32040	-1.670855871	1.35E-09	Arabidopsis thaliana geranylgeranyl pyrophosphate synthase 12 mRNA, complete cds
AT4G29050	-1.318554127	1.56E-09	Arabidopsis thaliana chromosome 4 sequence
AT5G10625	-1.348927776	1.60E-09	Arabidopsis thaliana chromosome 5 sequence
AT1G54790	-1.191688035	1.60E-09	Arabidopsis thaliana GDSL esterase/lipase mRNA, complete cds
AT4G15740	-1.399013008	2.03E-09	Arabidopsis thaliana chromosome 4 sequence
AT4G37850	-2.571320198	2.34E-09	Arabidopsis thaliana transcription factor

			bHLH25 mRNA, complete cds
AT1G22250	-1.066915696	2.37E-09	Arabidopsis thaliana uncharacterized protein mRNA, complete cds
AT3G52480	-1.153456654	2.40E-09	Arabidopsis thaliana chromosome 3, complete sequence
AT1G31885	-1.246168202	2.85E-09	Arabidopsis thaliana aquaporin NIP3-1 mRNA, complete cds
AT1G50050	-1.189729182	2.95E-09	Arabidopsis thaliana putative pathogenesis-related protein mRNA, complete cds
AT3G23630	-1.992783966	3.20E-09	Arabidopsis thaliana chromosome 3, complete sequence
AT1G65570	-3.103815279	3.80E-09	Arabidopsis thaliana polygalacturonase family protein mRNA, complete cds
AT1G30370	-1.775192531	4.48E-09	Arabidopsis thaliana chromosome 1 sequence
AT2G40113	-1.424215904	4.69E-09	Arabidopsis thaliana pollen Ole e 1 allergen and extensin family protein mRNA, complete cds
AT2G45360	-1.184116907	4.73E-09	Arabidopsis thaliana uncharacterized protein mRNA, complete cds
AT5G35490	-1.48037863	4.97E-09	Arabidopsis thaliana chromosome 5 sequence
AT1G60190	-1.492621236	5.52E-09	Arabidopsis thaliana chromosome 1 sequence
AT4G31875	-1.007139259	5.68E-09	Arabidopsis thaliana chromosome 4 sequence
AT1G71050	-1.054519982	5.84E-09	Arabidopsis thaliana heavy-metal-associated domain-containing protein mRNA, complete cds
AT1G61750	-1.402443286	6.08E-09	Arabidopsis thaliana Receptor-like protein kinase-related protein mRNA, complete cds
AT5G16170	-1.127770011	6.29E-09	Arabidopsis thaliana core-2/I-branching beta-1,6-N-acetylglucosaminyltransferase family protein mRNA, complete cds
AT4G13580	-1.231156318	6.47E-09	Arabidopsis thaliana chromosome 4 sequence
AT1G19900	-1.077856052	6.72E-09	Arabidopsis thaliana chromosome 1 sequence
AT4G37990	-1.28278542	7.33E-09	Arabidopsis thaliana cinnamyl alcohol dehydrogenase 8 mRNA, complete cds
AT4G00955	-1.103815279	7.60E-09	Arabidopsis thaliana chromosome 4 sequence
AT5G43540	-1.630498134	7.68E-09	Arabidopsis thaliana chromosome 5 sequence
AT5G35870	-4.933890277	8.80E-09	Arabidopsis thaliana chromosome 5 sequence
AT1G08890	-1.00944131	8.95E-09	Arabidopsis thaliana sugar transporter ERD6-like 1 mRNA, complete cds
AT1G14540	-1.069773705	9.12E-09	Arabidopsis thaliana peroxidase 4 mRNA, complete cds
AT2G30770	-1.294887937	9.54E-09	Arabidopsis thaliana cytochrome P450,

			family 71, subfamily A, polypeptide 13 mRNA, complete cds
AT2G23630	-1.442903925	9.62E-09	Arabidopsis thaliana protein SKU5 similar 16 mRNA, complete cds
AT4G04745	-1.716298842	1.01E-08	Arabidopsis thaliana chromosome 4 sequence
AT4G18360	-1.0953537	1.04E-08	Arabidopsis thaliana glycolate oxidase mRNA, complete cds
AT1G33817.1	-1.826975073	1.22E-08	Arabidopsis thaliana chromosome 1 sequence
AT3G29034	-1.02039927	1.22E-08	Arabidopsis thaliana chromosome 3, complete sequence
AT4G15270	-2.021353118	1.27E-08	Arabidopsis thaliana glucosyltransferase-related protein mRNA, complete cds
AT1G05310	-1.031187478	1.43E-08	Arabidopsis thaliana putative pectinesterase 8 mRNA, complete cds
AT3G23180	-1.179366312	1.62E-08	Arabidopsis thaliana HR-like lesion-inducing protein-like protein mRNA, complete cds
AT1G11190	-1.179366312	1.62E-08	Arabidopsis thaliana endonuclease 1 mRNA, complete cds
AT2G34600	-5.840780873	1.75E-08	Arabidopsis thaliana jasmonate-zim-domain protein 7 mRNA, complete cds
AT5G48290	-1.245091965	1.81E-08	Arabidopsis thaliana heavy metal transport/detoxification domain-containing protein mRNA, complete cds
AT1G34040	-1.054905678	1.94E-08	Arabidopsis thaliana Pyridoxal phosphate-dependent transferases superfamily protein mRNA, complete cds
AT2G05380	-1.549549575	2.20E-08	Arabidopsis thaliana glycine-rich protein 3 short isoform mRNA, complete cds
AT5G35480	-1.659715314	2.21E-08	Arabidopsis thaliana chromosome 5 sequence
AT1G02400	-1.014508737	2.22E-08	Arabidopsis thaliana gibberellin 2-oxidase 6 mRNA, complete cds
AT3G14260	-1.025388632	2.61E-08	Arabidopsis thaliana LURP-one-related 11 protein mRNA, complete cds
AT2G22570	-1.069667056	2.61E-08	Arabidopsis thaliana nicotinamidase 1 mRNA, complete cds
AT3G60490	-1.213998196	2.71E-08	Arabidopsis thaliana chromosome 3, complete sequence
AT3G20160	-3.978284396	2.72E-08	Arabidopsis thaliana chromosome 3, complete sequence
AT1G45015	-1.239392824	2.93E-08	Arabidopsis thaliana MD-2-related lipid recognition domain-containing protein mRNA, complete cds

AT3G59880	-1.00747141	3.46E-08	Arabidopsis thaliana chromosome 3, complete sequence
AT1G24147	-1.073165066	3.53E-08	Arabidopsis thaliana uncharacterized protein mRNA, complete cds
AT2G20880	-1.550561638	3.61E-08	Arabidopsis thaliana chromosome 2, complete sequence
AT2G47540	-1.380146507	3.85E-08	Arabidopsis thaliana pollen Ole e 1 allergen and extensin family protein mRNA, complete cds
AT2G01880	-1.149618968	4.30E-08	Arabidopsis thaliana purple acid phosphatase 7 mRNA, complete cds
AT1G27670	-2.181817791	4.51E-08	Arabidopsis thaliana chromosome 1 sequence
AT4G34790	-1.801786741	4.77E-08	Arabidopsis thaliana chromosome 4 sequence
AT4G20820	-1.353429169	8.04E-08	Arabidopsis thaliana chromosome 4 sequence
AT1G32350	-3.888086588	8.89E-08	Arabidopsis thaliana alternative oxidase 1D mRNA, complete cds
AT4G03450	-1.215661246	1.07E-07	Arabidopsis thaliana ankyrin repeat-containing protein mRNA, complete cds
AT3G47710	-1.302041389	1.13E-07	Arabidopsis thaliana atypical non-DNA binding bHLH protein BNQ3 mRNA, complete cds
AT1G34180	-1.634329995	1.37E-07	Arabidopsis thaliana NAC domain containing protein 16 mRNA, complete cds
AT4G05170	-3.840780873	1.61E-07	Arabidopsis thaliana transcription factor bHLH114 mRNA, complete cds
AT5G38120	-1.416283044	1.66E-07	Arabidopsis thaliana 4-coumarate--CoA ligase-like 8 mRNA, complete cds
AT2G47670	-1.741245199	1.78E-07	Arabidopsis thaliana chromosome 2, complete sequence
AT1G21326	-2.859889696	1.94E-07	Arabidopsis thaliana chromosome 1 sequence
AT3G62160	-1.043012	2.17E-07	Arabidopsis thaliana HXXXD-type acyl-transferase-like protein mRNA, complete cds
AT5G55110	-4.634329995	2.90E-07	Arabidopsis thaliana chromosome 5 sequence
AT4G11521	-1.29058379	2.98E-07	Arabidopsis thaliana chromosome 4 sequence
AT5G55090	-1.236453047	3.48E-07	Arabidopsis thaliana chromosome 5 sequence
AT3G49620	-2.287527232	4.24E-07	Arabidopsis thaliana 2-oxoacid-dependent dioxygenase-like protein DIN11 mRNA, complete cds
AT1G23840	-1.249092509	4.59E-07	Arabidopsis thaliana chromosome 1 sequence
AT3G26830	-1.051073816	4.63E-07	Arabidopsis thaliana protein PHYTOALEXIN DEFICIENT 3 mRNA,

			complete cds
AT5G53380	-5.518852778	4.77E-07	Arabidopsis thaliana O-acyltransferase (WSD1-like) family protein mRNA, complete cds
AT5G55250	-1.223396894	4.90E-07	Arabidopsis thaliana IAA carboxylmethyltransferase 1 mRNA, complete cds
AT4G31940	-4.577746467	5.23E-07	Arabidopsis thaliana cytochrome P450, family 82, subfamily C, polypeptide 4 mRNA, complete cds
AT1G76070	-1.098277095	5.25E-07	Arabidopsis thaliana chromosome 1 sequence
AT5G45660	-1.026336526	5.35E-07	Arabidopsis thaliana uncharacterized protein mRNA, complete cds
AT5G42580	-1.026336526	5.35E-07	Arabidopsis thaliana cytochrome P450, family 705, subfamily A, polypeptide 12 mRNA, complete cds
AT4G18425	-1.300672608	6.34E-07	Arabidopsis thaliana chromosome 4 sequence
AT2G41970	-1.537961601	6.44E-07	Arabidopsis thaliana putative protein kinase mRNA, complete cds
AT3G04060	-1.12151728	6.94E-07	Arabidopsis thaliana NAC domain containing protein 46 mRNA, complete cds
AT2G27690	-1.741245199	7.30E-07	Arabidopsis thaliana chromosome 2, complete sequence
AT4G10350	-2.59685529	7.56E-07	Arabidopsis thaliana protein BEARSKIN 2 mRNA, complete cds
AT5G59070	-1.419317104	8.25E-07	Arabidopsis thaliana glycosyl transferase family protein mRNA, complete cds
AT5G52050	-1.044073195	8.48E-07	Arabidopsis thaliana chromosome 5 sequence
AT2G28860	-1.5002371	8.69E-07	Arabidopsis thaliana chromosome 2, complete sequence
AT3G29000	-1.5002371	8.69E-07	Arabidopsis thaliana chromosome 3, complete sequence
AT1G13130	-2.44846345	9.12E-07	Arabidopsis thaliana Cellulase (glycosyl hydrolase family 5) protein mRNA, complete cds
AT1G67105	-2.44846345	9.12E-07	Arabidopsis thaliana chromosome 1 sequence
AT1G07550	-1.656356302	9.88E-07	Arabidopsis thaliana putative LRR receptor-like serine/threonine-protein kinase mRNA, complete cds
AT3G50120	-2.3262077	1.05E-06	Arabidopsis thaliana uncharacterized protein mRNA, complete cds
AT1G30757	-1.685962764	1.06E-06	Arabidopsis thaliana chromosome 1 sequence
AT1G75250	-1.263197902	1.12E-06	Arabidopsis thaliana protein RADIALIS-like 6 mRNA, complete cds

AT2G15370	-1.080731665	1.32E-06	Arabidopsis thaliana probable fucosyltransferase 5 mRNA, complete cds
AT5G51580	-1.607662045	1.39E-06	Arabidopsis thaliana chromosome 5 sequence
AT3G57157	-1.430316103	1.52E-06	Arabidopsis thaliana chromosome 3, complete sequence
AT4G27657	-1.384551686	1.75E-06	Arabidopsis thaliana chromosome 4 sequence
AT1G70420	-1.103815279	1.76E-06	Arabidopsis thaliana chromosome 1 sequence
AT4G29800	-1.085893371	1.79E-06	Arabidopsis thaliana PATATIN-like protein 8 mRNA, complete cds
AT3G03280	-1.963637621	2.14E-06	Arabidopsis thaliana chromosome 3, complete sequence
AT3G21500	-5.3262077	2.60E-06	Arabidopsis thaliana 1-deoxy-D-xylulose 5-phosphate synthase 1 mRNA, complete cds
AT1G33760	-5.3262077	2.60E-06	Arabidopsis thaliana chromosome 1 sequence
AT4G14780	-1.134188928	2.69E-06	Arabidopsis thaliana protein kinase family protein mRNA, complete cds
AT1G52830	-2.656356302	3.06E-06	Arabidopsis thaliana indole-3-acetic acid 6 mRNA, complete cds
AT4G21340	-2.156282698	3.15E-06	Arabidopsis thaliana transcription factor bHLH103 mRNA, complete cds
AT5G42440	-1.00206178	3.25E-06	Arabidopsis thaliana chromosome 5 sequence
AT5G45120	-2.071393801	3.31E-06	Arabidopsis thaliana chromosome 5 sequence
AT2G44840	-1.998020615	3.40E-06	Arabidopsis thaliana chromosome 2, complete sequence
AT1G61340	-1.407821465	3.67E-06	Arabidopsis thaliana F-box stress induced 1 mRNA, complete cds
AT5G19870	-1.407821465	3.67E-06	Arabidopsis thaliana chromosome 5 sequence
AT1G75030	-1.423275118	4.12E-06	Arabidopsis thaliana thaumatin-like protein 3 mRNA, complete cds
AT2G47360	-1.084449954	4.47E-06	Arabidopsis thaliana chromosome 2, complete sequence
AT3G13950	-5.255818372	4.62E-06	Arabidopsis thaliana uncharacterized protein mRNA, complete cds
AT4G08040	-1.239698707	4.83E-06	Arabidopsis thaliana 1-aminocyclopropane-1-carboxylate synthase 11 mRNA, complete cds
AT5G01700	-1.151481712	4.86E-06	Arabidopsis thaliana putative protein phosphatase 2C 65 mRNA, complete cds
AT5G44350	-1.566158493	5.22E-06	Arabidopsis thaliana chromosome 5 sequence
AT2G21900	-2.611962182	5.27E-06	Arabidopsis thaliana putative WRKY transcription factor 59 mRNA, complete cds
AT2G16970	-1.132670141	5.81E-06	Arabidopsis thaliana tetracycline transporter-like protein 1 mRNA, complete

			cds
AT3G49330	-1.187646869	6.24E-06	Arabidopsis thaliana chromosome 3, complete sequence
AT1G25430.1	-1.223396894	6.82E-06	Arabidopsis thaliana chromosome 1 sequence
AT5G22460	-1.114462523	6.90E-06	Arabidopsis thaliana esterase/lipase/thioesterase family protein mRNA, complete cds
AT4G12090	-1.093546943	7.03E-06	Arabidopsis thaliana Cornichon family protein mRNA, complete cds
AT5G02780	-1.518852778	7.11E-06	Arabidopsis thaliana glutathione transferase lambda 1 mRNA, complete cds
AT2G01300	-1.038859837	7.13E-06	Arabidopsis thaliana chromosome 2, complete sequence
AT3G07000	-1.038859837	7.13E-06	Arabidopsis thaliana chromosome 3, complete sequence
AT4G01970	-1.055880801	7.14E-06	Arabidopsis thaliana stachyose synthase mRNA, complete cds
ATCG00080	-2.296460357	7.29E-06	Cardamine resedifolia plastid, complete genome
AT2G28710	-1.264535589	7.31E-06	Arabidopsis thaliana C2H2-type zinc finger-containing protein mRNA, complete cds
AT3G10930	-1.7274394	7.64E-06	Arabidopsis thaliana chromosome 3, complete sequence
AT4G15417	-1.54269952	7.85E-06	Arabidopsis thaliana protein RNAse II-like 1 mRNA, complete cds
AT4G24000	-1.770391545	8.05E-06	Arabidopsis thaliana cellulose synthase-like protein G2 mRNA, complete cds
AT4G01140	-1.076848231	8.26E-06	Arabidopsis thaliana chromosome 4 sequence
AT1G33813.1	-2.085893371	8.43E-06	Arabidopsis thaliana chromosome 1 sequence
AT2G38790	-1.146193881	9.05E-06	Arabidopsis thaliana chromosome 2, complete sequence
AT4G13860	-1.126535355	1.08E-05	Arabidopsis thaliana RNA recognition motif-containing protein mRNA, complete cds
AT5G23990	-1.661810732	1.10E-05	Arabidopsis thaliana ferric reduction oxidase 5 mRNA, complete cds
AT4G06534	-1.103815279	1.10E-05	Arabidopsis thaliana chromosome 4 sequence
AT4G10540	-1.213998196	1.11E-05	Arabidopsis thaliana Subtilase family protein mRNA, complete cds
AT3G49070	-1.415016967	1.12E-05	Arabidopsis thaliana chromosome 3, complete sequence

AT1G16370	-1.255818372	1.19E-05	Arabidopsis thaliana chromosome 1 sequence
AT3G50460	-1.107538365	1.28E-05	Arabidopsis thaliana RPW8-like protein 2 mRNA, complete cds
AT5G63130	-2.143343643	1.31E-05	Arabidopsis thaliana octicosapeptide/Phox/Bem1p domain-containing protein mRNA, complete cds
AT5G46040	-1.265096186	1.37E-05	Arabidopsis thaliana peptide transporter PTR3-B mRNA, complete cds
AT4G01890	-1.969514187	1.40E-05	Arabidopsis thaliana glycoside hydrolase family 28 protein / polygalacturonase (pectinase) family protein mRNA, complete cds
AT1G74290	-1.451738582	1.41E-05	Arabidopsis thaliana esterase/lipase/thioesterase family protein mRNA, complete cds
AT3G22275	-5.103815279	1.47E-05	Arabidopsis thaliana uncharacterized protein mRNA, complete cds
AT2G32610	-1.328749894	1.63E-05	Arabidopsis thaliana cellulose synthase-like protein B1 mRNA, complete cds
AT1G15580	-1.393321896	1.64E-05	Arabidopsis thaliana auxin-responsive protein IAA5 mRNA, complete cds
AT5G61360	-1.634329995	1.69E-05	Arabidopsis thaliana uncharacterized protein mRNA, complete cds
AT5G14020	-1.016352437	1.77E-05	Arabidopsis thaliana Endosomal targeting BRO1-like domain-containing protein mRNA, complete cds
AT3G10320	-1.285362648	1.80E-05	Arabidopsis thaliana Glycosyltransferase family 61 protein mRNA, complete cds
AT4G27652	-1.670855871	1.82E-05	Arabidopsis thaliana chromosome 4 sequence
AT1G71740	-1.808359395	2.14E-05	Arabidopsis thaliana chromosome 1 sequence
AT5G42785	-1.866776081	2.21E-05	Arabidopsis thaliana uncharacterized protein mRNA, complete cds
AT3G22240	-1.099900229	2.39E-05	Arabidopsis thaliana uncharacterized protein mRNA, complete cds
AT1G43160	-1.371295589	2.39E-05	Arabidopsis thaliana ethylene-responsive transcription factor RAP2-6 mRNA, complete cds
AT2G39030	-5.021353118	2.64E-05	Arabidopsis thaliana chromosome 2, complete sequence
AT1G33280	-2.469943177	2.65E-05	Arabidopsis thaliana protein BEARSKIN1 mRNA, complete cds
AT1G23965	-2.469943177	2.65E-05	Arabidopsis thaliana chromosome 1 sequence
AT4G14380	-1.493317686	2.91E-05	Arabidopsis thaliana chromosome 4 sequence

AT2G02610	-1.493317686	2.91E-05	Arabidopsis thaliana chromosome 2, complete sequence
AT1G19320	-2.303124087	3.06E-05	Arabidopsis thaliana pathogenesis-related thaumatin-like protein mRNA, complete cds
AT4G37235	-1.725303655	3.19E-05	Arabidopsis thaliana CASP-like protein mRNA, complete cds
AT1G35625	-1.424215904	3.44E-05	Arabidopsis thaliana RING/U-box domain-containing protein mRNA, complete cds
AT1G47590	-1.897364401	3.58E-05	Arabidopsis thaliana chromosome 1 sequence
AT1G33320	-1.973418641	3.59E-05	Arabidopsis thaliana Pyridoxal phosphate (PLP)-dependent transferases superfamily protein mRNA, complete cds
AT2G31310	-2.634329995	3.73E-05	Arabidopsis thaliana LOB domain-containing protein 14 mRNA, complete cds
AT1G13430	-1.007139259	3.80E-05	Arabidopsis thaliana chromosome 1 sequence
AT1G53700	-1.087695613	3.81E-05	Arabidopsis thaliana chromosome 1 sequence
AT2G24850	-1.444852196	3.86E-05	Arabidopsis thaliana tyrosine aminotransferase 3 mRNA, complete cds
AT2G29740	-1.026999682	4.48E-05	Arabidopsis thaliana chromosome 2, complete sequence
AT4G13130	-1.21682424	4.50E-05	Arabidopsis thaliana chromosome 4 sequence
AT2G32200	-2.419317104	4.52E-05	Arabidopsis thaliana uncharacterized protein mRNA, complete cds
AT4G13395	-4.933890277	4.75E-05	Arabidopsis thaliana chromosome 4 sequence
AT2G23960	-1.225656401	5.18E-05	Arabidopsis thaliana class I glutamine amidotransferase-like superfamily protein mRNA, complete cds
AT4G40010	-1.071393801	5.23E-05	Arabidopsis thaliana SNF1-related protein kinase 2.7 mRNA, complete cds
AT3G46700	-1.071393801	5.23E-05	Arabidopsis thaliana UDP-glycosyltransferase 76E3 mRNA, complete cds
AT5G07780	-1.049367495	5.24E-05	Arabidopsis thaliana formin-like protein 19 mRNA, complete cds
AT1G73290	-1.796386753	5.51E-05	Arabidopsis thaliana serine carboxypeptidase-like 5 mRNA, complete cds
AT5G43890	-2.021353118	5.72E-05	Arabidopsis thaliana chromosome 5 sequence
AT1G61480	-1.933890277	5.77E-05	Arabidopsis thaliana G-type lectin S-receptor-like serine/threonine-protein kinase mRNA, complete cds
AT3G14440	-1.235059812	5.97E-05	Arabidopsis thaliana chromosome 3, complete sequence

AT2G47485	-1.031187478	6.10E-05	Arabidopsis thaliana chromosome 2, complete sequence
AT5G12340	-1.052071703	6.12E-05	Arabidopsis thaliana chromosome 5 sequence
AT5G28237	-2.577746467	6.45E-05	Arabidopsis thaliana tryptophan synthase beta chain-like protein mRNA, complete cds
AT3G27884	-1.581588533	6.47E-05	Arabidopsis thaliana clone 102688 mRNA sequence
AT3G20935	-1.357101708	6.52E-05	Arabidopsis thaliana cytochrome P450, family 705, subfamily A, polypeptide 28 mRNA, complete cds
AT3G18777	-1.103815279	7.00E-05	Arabidopsis thaliana chromosome 3, complete sequence
AT2G01275	-1.618388451	7.05E-05	Arabidopsis thaliana RING/FYVE/PHD zinc finger-containing protein mRNA, complete cds
AT5G50760	-1.659715314	7.62E-05	Arabidopsis thaliana chromosome 5 sequence
AT1G16820	-1.706479781	8.16E-05	Arabidopsis thaliana V-ATPase-related protein mRNA, complete cds
AT3G47050	-1.176746801	8.90E-05	Arabidopsis thaliana glycosyl hydrolase family protein mRNA, complete cds
AT3G32047	-1.821415548	9.01E-05	Arabidopsis thaliana cytochrome P450 superfamily protein mRNA, complete cds
AT1G64910	-1.267314011	9.09E-05	Arabidopsis thaliana chromosome 1 sequence
AT4G25470	-1.978284396	9.27E-05	Arabidopsis thaliana chromosome 4 sequence
AT5G43620	-1.413883218	9.45E-05	Arabidopsis thaliana chromosome 5 sequence
AT5G59930	-1.085893371	9.64E-05	Arabidopsis thaliana chromosome 5 sequence
AT5G59845	-1.550561638	9.80E-05	Arabidopsis thaliana gibberellin-regulated protein mRNA, complete cds
AT5G66400	-1.550561638	9.80E-05	Arabidopsis thaliana dehydrin Rab18 mRNA, complete cds
AT5G51870	-1.585966974	0.000107466	Arabidopsis thaliana MADS-box transcription factor AGL71 mRNA, complete cds
AT3G01760	-1.348927776	0.000107855	Arabidopsis thaliana Lysine histidine transporter-like 4 mRNA, complete cds
AT2G20562	-1.348927776	0.000107855	Arabidopsis thaliana uncharacterized protein mRNA, complete cds
AT2G22790	-1.348927776	0.000107855	Arabidopsis thaliana chromosome 2, complete sequence
AT1G79320	-1.118314848	0.000110376	Arabidopsis thaliana metacaspase 6 mRNA, complete cds
AT3G08870	-1.019281768	0.000112766	Arabidopsis thaliana chromosome 3, complete sequence
AT5G42930	-1.46113728	0.00011915	Arabidopsis thaliana lipase class 3-like

			protein mRNA, complete cds
AT4G30420	-1.094354949	0.000130921	Arabidopsis thaliana nodulin MtN21-like transporter family protein mRNA, complete cds
AT1G23120	-1.067746024	0.000132242	Arabidopsis thaliana polyketide cyclase/dehydrase and lipid transport superfamily protein mRNA, complete cds
AT2G44010	-1.067746024	0.000132242	Arabidopsis thaliana chromosome 2, complete sequence
AT2G31980	-1.933890277	0.000149485	Arabidopsis thaliana cysteine proteinase inhibitor 2 mRNA, complete cds
AT5G24220	-2.741245199	0.000153253	Arabidopsis thaliana lipase class 3-related protein mRNA, complete cds
AT5G51470	-2.741245199	0.000153253	Arabidopsis thaliana auxin-responsive GH3 family protein mRNA, complete cds
AT4G27654	-4.741245199	0.000156595	Arabidopsis thaliana chromosome 4 sequence
AT1G72260	-4.741245199	0.000156595	Arabidopsis thaliana thionin 2.1 mRNA, complete cds
AT5G22410	-1.407821465	0.000156914	Arabidopsis thaliana peroxidase 60 mRNA, complete cds
AT3G46810	-1.210730482	0.000159578	Arabidopsis thaliana chromosome 3, complete sequence
AT1G77640	-1.55280011	0.000163014	Arabidopsis thaliana chromosome 1 sequence
AT1G32928	-1.431389937	0.00017673	Arabidopsis thaliana chromosome 1 sequence
AT1G70860	-1.431389937	0.00017673	Arabidopsis thaliana SRPBCC ligand-binding domain-containing protein mRNA, complete cds
AT2G35910	-1.103815279	0.000177726	Arabidopsis thaliana chromosome 2, complete sequence
AT5G64230	-1.004279605	0.0001782	Arabidopsis thaliana uncharacterized protein mRNA, complete cds
AT3G28007	-1.591002564	0.000178736	Arabidopsis thaliana bidirectional sugar transporter SWEET4 mRNA, complete cds
AT1G62262	-3.103815279	0.000193907	Arabidopsis thaliana SLAC1 homologue 4 mRNA, complete cds
AT3G25597	-1.634329995	0.000194369	Arabidopsis thaliana chromosome 3, complete sequence
AT1G73810	-1.683912024	0.000209283	Arabidopsis thaliana core-2/I-branching beta-1,6-N-acetylglucosaminyltransferase-like protein mRNA, complete cds
AT1G17610	-1.052534774	0.000211129	Arabidopsis thaliana chromosome 1 sequence
AT1G79250	-3.840780873	0.000211237	Arabidopsis thaliana AGC kinase 1.7 mRNA,

			complete cds
AT2G23410	-1.4864313	0.000221481	Arabidopsis thaliana cis-prenyltransferase mRNA, complete cds
AT3G52072	-2.103815279	0.000233962	Arabidopsis thaliana mRNA for hypothetical protein, complete cds, clone: RAFL21-36-M10
AT5G45580	-1.148903168	0.000234621	Arabidopsis thaliana homeodomain-like superfamily protein mRNA, complete cds
AT1G05100	-1.888086588	0.000239866	Arabidopsis thaliana chromosome 1 sequence
AT1G30560	-1.196924683	0.00026083	Arabidopsis thaliana chromosome 1 sequence
AT3G28890	-2.670855871	0.000268631	Arabidopsis thaliana receptor like protein 43 mRNA, complete cds
AT3G52970	-2.670855871	0.000268631	Arabidopsis thaliana cytochrome P450, family 76, subfamily G, polypeptide 1 mRNA, complete cds
AT5G67080	-2.670855871	0.000268631	Arabidopsis thaliana chromosome 5 sequence
AT5G14180	-1.555378654	0.000271722	Arabidopsis thaliana Myzus persicae-induced lipase 1 mRNA, complete cds
AT5G42460	-1.059421159	0.000288119	Arabidopsis thaliana chromosome 5 sequence
AT3G13437	-1.206908772	0.000301857	Arabidopsis thaliana uncharacterized protein mRNA, complete cds
AT1G71030	-1.164187897	0.000316553	Arabidopsis thaliana putative myb family transcription factor mRNA, complete cds
AT1G76430	-1.64438366	0.000323405	Arabidopsis thaliana putative inorganic phosphate transporter 1-9 mRNA, complete cds
AT5G46130	-2.393321896	0.000326559	Arabidopsis thaliana uncharacterized protein mRNA, complete cds
AT3G55515	-2.393321896	0.000326559	Arabidopsis thaliana chromosome 3, complete sequence
AT5G15900	-1.126535355	0.000326994	Arabidopsis thaliana protein trichome birefringence-like 19 mRNA, complete cds
AT5G15890	-1.453264436	0.000330595	Arabidopsis thaliana protein trichome birefringence-like 21 mRNA, complete cds
AT1G62510	-1.063173294	0.000336598	Arabidopsis thaliana chromosome 1 sequence
AT5G59940	-1.063173294	0.000336598	Arabidopsis thaliana chromosome 5 sequence
AT2G31230	-1.063173294	0.000336598	Arabidopsis thaliana chromosome 2, complete sequence
AT3G25950	-1.699425023	0.000347336	Arabidopsis thaliana chromosome 3, complete sequence

AT3G46760	-3.021353118	0.000348439	Arabidopsis thaliana chromosome 3, complete sequence
AT1G28040	-1.284387524	0.000373595	Arabidopsis thaliana RING-H2 finger protein ATL20 mRNA, complete cds
AT1G33870	-1.284387524	0.000373595	Arabidopsis thaliana P-loop containing nucleoside triphosphate hydrolases superfamily protein mRNA, complete cds
AT1G10070	-1.369989392	0.00038334	Arabidopsis thaliana branched-chain-amino-acid aminotransferase 2 mRNA, complete cds
AT4G19920	-1.369989392	0.00038334	Arabidopsis thaliana Toll-Interleukin-Resistance domain-containing protein mRNA, complete cds
AT4G25490	-3.741245199	0.000388987	Arabidopsis thaliana chromosome 4 sequence
AT2G30760	-3.741245199	0.000388987	Arabidopsis thaliana uncharacterized protein mRNA, complete cds
AT2G24040	-1.098277095	0.000389081	Arabidopsis thaliana Low temperature and salt responsive protein mRNA, complete cds
AT1G05575	-1.933890277	0.000389617	Arabidopsis thaliana chromosome 1 sequence
AT2G14210	-1.014060626	0.000390904	Arabidopsis thaliana protein agamous-like 44 mRNA, complete cds
AT1G33900	-1.014060626	0.000390904	Arabidopsis thaliana P-loop containing nucleoside triphosphate hydrolases superfamily protein mRNA, complete cds
AT1G68620	-1.016352437	0.000457614	Arabidopsis thaliana chromosome 1 sequence
AT4G14060	-1.016352437	0.000457614	Arabidopsis thaliana major latex protein-like protein mRNA, complete cds
AT3G15800	-1.042414734	0.000460491	Arabidopsis thaliana glycosyl hydrolase superfamily protein mRNA, complete cds
AT1G55390	-1.318554127	0.00049058	Arabidopsis thaliana chromosome 1 sequence
AT2G45570	-1.318554127	0.00049058	Arabidopsis thaliana cytochrome P450 76C2 mRNA, complete cds
AT1G17300	-1.148015082	0.000515399	Arabidopsis thaliana uncharacterized protein mRNA, complete cds
AT5G21960	-4.518852778	0.00052662	Arabidopsis thaliana chromosome 5 sequence
AT5G44260	-1.109740113	0.000529296	Arabidopsis thaliana chromosome 5 sequence
AT3G51540	-1.109740113	0.000529296	Arabidopsis thaliana uncharacterized protein mRNA, complete cds
AT4G17470	-1.255818372	0.00053669	Arabidopsis thaliana putative

			palmitoyl-protein thioesterase mRNA, complete cds
AT3G53840	-1.075909282	0.00053679 4	Arabidopsis thaliana wall-associated receptor kinase-like 15 mRNA, complete cds
AT5G63090	-1.075909282	0.00053679 4	Arabidopsis thaliana protein LATERAL ORGAN BOUNDARIES mRNA, complete cds
AT5G52270	-2.3262077	0.0005562	Arabidopsis thaliana SNARE-like superfamily protein mRNA, complete cds
AT5G47980	-1.791871272	0.00060794 5	Arabidopsis thaliana chromosome 5 sequence
AT4G37700	-1.270925264	0.00061791	Arabidopsis thaliana chromosome 4 sequence
AT2G43310	-1.881422857	0.00062723 8	Arabidopsis thaliana chromosome 2, complete sequence
AT3G50800	-1.021353118	0.00062743 3	Arabidopsis thaliana chromosome 3, complete sequence
AT3G44700	-1.992783966	0.00062939 8	Arabidopsis thaliana uncharacterized protein mRNA, complete cds
AT5G50560	-1.049367495	0.00063024 2	Arabidopsis thaliana chromosome 5 sequence
AT1G11185	-1.213998196	0.00066323 3	Arabidopsis thaliana chromosome 1 sequence
AT2G34340	-1.518852778	0.00068796 1	Arabidopsis thaliana chromosome 2, complete sequence
AT5G11610	-1.287527232	0.00071030 4	Arabidopsis thaliana Exostosin family protein mRNA, complete cds
AT4G28530	-1.384551686	0.00072528 8	Arabidopsis thaliana NAC domain containing protein 74 mRNA, complete cds
AT4G00960	-1.384551686	0.00072528 8	Arabidopsis thaliana protein kinase family protein mRNA, complete cds
AT5G39610	-1.085893371	0.00073275	Arabidopsis thaliana NAC-domain transcription factor mRNA, complete cds
AT1G56630	-1.053189205	0.00073742 1	Arabidopsis thaliana alpha/beta-Hydrolases superfamily protein mRNA, complete cds
AT1G23160	-1.174898377	0.00080969 3	Arabidopsis thaliana auxin-responsive GH3 family protein mRNA, complete cds
AT3G48520	-1.611962182	0.00083168 7	Arabidopsis thaliana chromosome 3, complete sequence
AT2G03600	-1.670855871	0.00089993 9	Arabidopsis thaliana ureide permease 3 mRNA, complete cds
AT5G58784	-1.670855871	0.00089993 9	Arabidopsis thaliana dehydrodolichyl diphosphate synthase 5 mRNA, complete cds
AT4G30640	-1.3262077	0.00093312	Arabidopsis thaliana RNI-like superfamily protein mRNA, complete cds

AT1G05530	-1.185429044	0.000940279	Arabidopsis thaliana chromosome 1 sequence
AT5G44920	-1.185429044	0.000940279	Arabidopsis thaliana Toll-Interleukin-Resistance domain-containing protein mRNA, complete cds
AT5G20810	-2.255818372	0.000942752	Arabidopsis thaliana SAUR-like auxin-responsive protein mRNA, complete cds
AT5G64810	-1.741245199	0.000959654	Arabidopsis thaliana putative WRKY transcription factor 51 mRNA, complete cds
AT4G11310	-4.393321896	0.000973747	Arabidopsis thaliana putative cysteine proteinase mRNA, complete cds
AT5G66650	-1.00206178	0.000998225	Arabidopsis thaliana uncharacterized protein mRNA, complete cds
AT3G09960	-1.097389009	0.001000129	Arabidopsis thaliana calcineurin-like metallo-phosphoesterase-like protein mRNA, complete cds
AT1G73580	-1.826975073	0.001003974	Arabidopsis thaliana C2 domain-containing protein mRNA, complete cds
AT5G46845	-1.826975073	0.001003974	Arabidopsis thaliana chromosome 5 sequence
AT2G41850	-2.071393801	0.001007753	Arabidopsis thaliana polygalacturonase ADPG2 mRNA, complete cds
AT4G14450	-2.071393801	0.001007753	Arabidopsis thaliana chromosome 4 sequence
AT2G19660	-1.030105592	0.0010086	Arabidopsis thaliana chromosome 2, complete sequence
AT5G09670	-1.933890277	0.001023808	Arabidopsis thaliana chromosome 5 sequence
AT1G16515	-1.933890277	0.001023808	Arabidopsis thaliana chromosome 1 sequence
AT5G65070	-1.348927776	0.001065592	Arabidopsis thaliana protein MADS AFFECTING FLOWERING 4 mRNA, complete cds
AT3G13403	-2.840780873	0.00111802	Arabidopsis thaliana defensin-like protein 302 mRNA, complete cds
AT1G19968	-2.840780873	0.00111802	Arabidopsis thaliana chromosome 1 sequence
AT3G45638	-2.840780873	0.00111802	Arabidopsis thaliana clone asmbl_10177 unknown mRNA sequence
AT2G35210	-1.103815279	0.001168327	Arabidopsis thaliana root and pollen arfgap mRNA, complete cds
AT1G62045	-1.272692191	0.001178955	Arabidopsis thaliana uncharacterized protein mRNA, complete cds

AT5G56230	-1.566158493	0.001275686	Arabidopsis thaliana chromosome 5 sequence
AT3G22250	-3.518852778	0.001325341	Arabidopsis thaliana UDP-glycosyltransferase 82A1 mRNA, complete cds
AT3G53010	-1.156282698	0.0013263	Arabidopsis thaliana uncharacterized protein mRNA, complete cds
AT2G14095	-1.40337556	0.001375922	Arabidopsis thaliana uncharacterized protein mRNA, complete cds
AT1G30845	-1.40337556	0.001375922	Arabidopsis thaliana uncharacterized protein mRNA, complete cds
AT1G70985	-1.071393801	0.001383336	Arabidopsis thaliana chromosome 1 sequence
AT1G51260	-1.621946271	0.001394982	Arabidopsis thaliana 1-acyl-sn-glycerol-3-phosphate acyltransferase 3 mRNA, complete cds
AT5G54030	-1.621946271	0.001394982	Arabidopsis thaliana chromosome 5 sequence
AT3G13275	-1.223396894	0.001465716	Arabidopsis thaliana chromosome 3, complete sequence
AT1G14860	-1.166551034	0.001543855	Arabidopsis thaliana nudix hydrolase 18 mRNA, complete cds
AT4G14305	-1.166551034	0.001543855	Arabidopsis thaliana Mpv17/PMP22 domain-containing protein mRNA, complete cds
AT1G32690	-1.436390618	0.001553487	Arabidopsis thaliana chromosome 1 sequence
AT1G63820	-1.3124019	0.001556668	Arabidopsis thaliana CCT motif family protein mRNA, complete cds
AT5G65500	-2.181817791	0.001589454	Arabidopsis thaliana U-box domain-containing protein kinase family protein mRNA, complete cds
AT4G25400	-1.770391545	0.001597119	Arabidopsis thaliana transcription factor bHLH118 mRNA, complete cds
AT3G45330	-1.009178404	0.00161142	Arabidopsis thaliana chromosome 3, complete sequence
AT5G38240	-1.040805481	0.001623913	Arabidopsis thaliana Protein kinase family protein mRNA, complete cds
AT1G07430	-1.872489732	0.001655277	Arabidopsis thaliana protein phosphatase 2C 3 mRNA, complete cds
AT5G25910	-2.004279605	0.001660762	Arabidopsis thaliana receptor like protein 52 mRNA, complete cds
AT3G04530	-1.238744859	0.001695968	Arabidopsis thaliana phosphoenolpyruvate carboxylase kinase 2 mRNA, complete cds

AT4G11020	-1.474458658	0.001744136	Arabidopsis thaliana uncharacterized protein mRNA, complete cds
AT1G46552.1	-1.474458658	0.001744136	Arabidopsis thaliana chromosome 1 sequence
AT5G02170	-1.335988721	0.001782274	Arabidopsis thaliana transmembrane amino acid transporter family protein mRNA, complete cds
AT1G69720	-1.17781586	0.001796098	Arabidopsis thaliana heme oxygenase 3 mRNA, complete cds
AT5G65300	-1.17781586	0.001796098	Arabidopsis thaliana chromosome 5 sequence
AT5G45105	-4.255818372	0.001811226	Arabidopsis thaliana zinc transporter 8 precursor mRNA, complete cds
AT1G50930	-4.255818372	0.001811226	Arabidopsis thaliana uncharacterized protein mRNA, complete cds
AT1G47630	-4.255818372	0.001811226	Arabidopsis thaliana chromosome 1 sequence
AT2G22750	-4.255818372	0.001811226	Arabidopsis thaliana transcription factor bHLH18 mRNA, complete cds
AT3G57950	-1.04492159	0.001904008	Arabidopsis thaliana chromosome 3, complete sequence
AT3G50280	-1.04492159	0.001904008	Arabidopsis thaliana chromosome 3, complete sequence
AT1G15900	-2.741245199	0.001994553	Arabidopsis thaliana chromosome 1 sequence
ATCG00220	-1.089168503	0.002218776	Olimarabidopsis pumila chloroplast DNA, complete sequence
AT4G23030	-1.634329995	0.002346619	Arabidopsis thaliana chromosome 4 sequence
AT3G28650	-1.20397944	0.002425691	Arabidopsis thaliana chromosome 3, complete sequence
AT2G05518	-1.20397944	0.002425691	Arabidopsis thaliana mRNA, clone: RAFL17-49-H20
AT2G37780	-1.20397944	0.002425691	Arabidopsis thaliana chromosome 2, complete sequence
AT1G73120	-2.348927776	0.002439617	Arabidopsis thaliana uncharacterized protein mRNA, complete cds
AT5G22150	-3.393321896	0.002451299	Arabidopsis thaliana uncharacterized protein mRNA, complete cds
AT1G72590	-3.393321896	0.002451299	Arabidopsis thaliana 3-oxo-5-alpha-steroid 4-dehydrogenase family protein mRNA, complete cds
AT2G29470	-3.393321896	0.002451299	Arabidopsis thaliana glutathione S-transferase tau 3 mRNA, complete cds
AT1G74080	-2.103815279	0.00266404	Arabidopsis thaliana myb domain protein 122

		6	mRNA, complete cds
AT3G22820	-1.933890277	0.00271955 9	Arabidopsis thaliana allergen-related protein mRNA, complete cds
AT1G51480	-1.933890277	0.00271955 9	Arabidopsis thaliana CC-NBS-LRR class disease resistance protein mRNA, complete cds
AT1G06160	-1.219292496	0.00281497 5	Arabidopsis thaliana chromosome 1 sequence
AT1G63600	-1.156282698	0.00295777 5	Arabidopsis thaliana chromosome 1 sequence
AT2G15760	-1.3209134	0.00298853 8	Arabidopsis thaliana chromosome 2, complete sequence
AT1G69150	-1.518852778	0.00328659 9	Arabidopsis thaliana chromosome 1 sequence
AT2G32179	-4.103815279	0.00339029 7	Arabidopsis thaliana uncharacterized protein mRNA, complete cds
AT1G48070	-4.103815279	0.00339029 7	Arabidopsis thaliana TRX domain-containing protein mRNA, complete cds
AT1G34500	-4.103815279	0.00339029 7	Arabidopsis thaliana chromosome 1 sequence
AT1G15010	-1.348927776	0.00342126 3	Arabidopsis thaliana chromosome 1 sequence
AT3G02840	-1.168355531	0.00344872 3	Arabidopsis thaliana chromosome 3, complete sequence
AT1G69990	-2.634329995	0.00354599 9	Arabidopsis thaliana chromosome 1 sequence
AT3G18880	-1.112227518	0.00355853 3	Arabidopsis thaliana Nucleic acid-binding, OB-fold-like protein mRNA, complete cds
AT3G01175	-1.025038165	0.00359912 8	Arabidopsis thaliana uncharacterized protein mRNA, complete cds
AT4G05100	-1.255818372	0.00377590 5	Arabidopsis thaliana myb domain protein 74 mRNA, complete cds
AT4G15248	-1.255818372	0.00377590 5	Arabidopsis thaliana chromosome 4 sequence
AT2G43600	-1.381349254	0.00390144 3	Arabidopsis thaliana chitinase family protein mRNA, complete cds
AT5G57400	-1.650097311	0.00396078 6	Arabidopsis thaliana uncharacterized protein mRNA, complete cds
AT3G54070	-1.650097311	0.00396078 6	Arabidopsis thaliana ankyrin repeat-containing protein mRNA, complete cds
AT1G70110	-1.650097311	0.00396078 6	Arabidopsis thaliana putative L-type lectin-domain containing receptor kinase V.1 mRNA, complete cds

AT1G65680	-2.255818372	0.004183124	Arabidopsis thaliana expansin B2 mRNA, complete cds
AT3G02430	-2.255818372	0.004183124	Arabidopsis thaliana chromosome 3, complete sequence
AT2G44700	-1.741245199	0.004240445	Arabidopsis thaliana chromosome 2, complete sequence
AT1G55290	-1.741245199	0.004240445	Arabidopsis thaliana feruloyl CoA ortho-hydroxylase 2 mRNA, complete cds
AT2G13960	-1.277844678	0.004361533	Arabidopsis thaliana homeodomain-like superfamily protein mRNA, complete cds
AT2G43870	-1.859889696	0.004422594	Arabidopsis thaliana putative polygalacturonase /pectinase mRNA, complete cds
AT3G46410	-1.859889696	0.004422594	Arabidopsis thaliana protein kinase family protein mRNA, complete cds
AT4G12350	-1.859889696	0.004422594	Arabidopsis thaliana myb domain protein 42 mRNA, complete cds
AT1G26360	-1.419317104	0.00442706	Arabidopsis thaliana methyl esterase 13 mRNA, complete cds
AT5G39680	-1.419317104	0.00442706	Arabidopsis thaliana chromosome 5 sequence
AT1G44010	-1.419317104	0.00442706	Arabidopsis thaliana uncharacterized protein mRNA, complete cds
AT4G16220	-1.419317104	0.00442706	Arabidopsis thaliana GDSL esterase/lipase mRNA, complete cds
AT1G35310	-3.255818372	0.004538298	Arabidopsis thaliana MLP-like protein 168 mRNA, complete cds
AT4G09780	-3.255818372	0.004538298	Arabidopsis thaliana TRAF-like family protein mRNA, complete cds
AT1G58430	-3.255818372	0.004538298	Arabidopsis thaliana anther-specific proline-rich protein RXF26 mRNA, complete cds
AT4G34380	-1.196924683	0.004678871	Arabidopsis thaliana chromosome 4 sequence
AT5G38005	-1.131829655	0.004872916	Arabidopsis thaliana Unknown protein mRNA, partial cds
AT2G46495	-1.078280186	0.004964726	Arabidopsis thaliana putative RING-H2 finger protein mRNA, complete cds
AT5G11180	-1.078280186	0.004964726	Arabidopsis thaliana glutamate receptor 2.6 mRNA, complete cds
AT5G43030	-1.078280186	0.004964726	Arabidopsis thaliana chromosome 5 sequence
AT3G17890	-1.033425951	0.004974211	Arabidopsis thaliana uncharacterized protein mRNA, complete cds
AT1G31835	-1.033425951	0.004974211	Arabidopsis thaliana chromosome 1 sequence

AT3G61930	-1.464404994	0.004991457	Arabidopsis thaliana chromosome 3, complete sequence
AT1G65484	-1.464404994	0.004991457	Arabidopsis thaliana uncharacterized protein mRNA, complete cds
AT1G34050	-1.518852778	0.005580549	Arabidopsis thaliana ankyrin repeats-containing protein mRNA, complete cds
AT2G07671	-1.332439654	0.005774083	Arabidopsis thaliana ecotype Col-0 mitochondrion, complete genome
AT2G09795	-1.085893371	0.005827617	Arabidopsis thaliana Full-length cDNA Complete sequence from clone GSLTFB3ZA09 of Flowers and buds of strain col-0 of Arabidopsis thaliana (thale cress)
AT5G03795	-1.085893371	0.005827617	Arabidopsis thaliana Exostosin family protein mRNA, complete cds
AT1G48670	-1.585966974	0.006168559	Arabidopsis thaliana auxin-responsive GH3 family protein mRNA, complete cds
AT2G20700	-1.585966974	0.006168559	Arabidopsis thaliana LORELEI-like glucosylphosphatidylinositol-anchored protein 2 mRNA, complete cds
AT1G75590	-1.233450559	0.006321342	Arabidopsis thaliana chromosome 1 sequence
AT2G24545	-3.933890277	0.006388922	Arabidopsis thaliana chromosome 2, complete sequence
AT4G30730	-3.933890277	0.006388922	Arabidopsis thaliana chromosome 4 sequence
AT5G09910	-3.933890277	0.006388922	Arabidopsis thaliana Ras-related small GTP-binding family protein mRNA, complete cds
AT5G61100	-3.933890277	0.006388922	Arabidopsis thaliana uncharacterized protein mRNA, complete cds
AT1G02440	-1.366849684	0.006607638	Arabidopsis thaliana ADP-ribosylation factor D1A mRNA, complete cds
AT5G35525	-1.366849684	0.006607638	Arabidopsis thaliana PLAC8 family protein mRNA, complete cds
AT1G10585	-1.670855871	0.006711549	Arabidopsis thaliana basic helix-loop-helix domain-containing protein mRNA, complete cds
AT1G03020	-1.001004473	0.006823443	Arabidopsis thaliana chromosome 1 sequence
AT5G22380	-2.156282698	0.007127542	Arabidopsis thaliana NAC domain-containing protein mRNA, complete cds
AT2G38240	-2.156282698	0.007127542	Arabidopsis thaliana 2-oxoglutarate (2OG) and Fe(II)-dependent oxygenase-like protein

			mRNA, complete cds
AT1G57570	-1.781887184	0.007137759	Arabidopsis thaliana jacalin-like plant lectin domain-containing protein mRNA, complete cds
AT2G37740	-1.781887184	0.007137759	Arabidopsis thaliana chromosome 2, complete sequence
AT1G71890	-1.781887184	0.007137759	Arabidopsis thaliana sucrose transport protein SUC5 mRNA, complete cds
AT4G23700	-1.255818372	0.007330321	Arabidopsis thaliana cation/H(+) antiporter 17 mRNA, complete cds
AT1G08310	-1.255818372	0.007330321	Arabidopsis thaliana alpha/beta-Hydrolases superfamily protein mRNA, complete cds
AT1G52660	-1.255818372	0.007330321	Arabidopsis thaliana probable disease resistance protein mRNA, complete cds
AT4G26380	-1.933890277	0.007333811	Arabidopsis thaliana cysteine/histidine-rich C1 domain-containing protein mRNA, complete cds
AT3G22961	-1.933890277	0.007333811	Arabidopsis thaliana chromosome 3, complete sequence
AT1G77960	-1.933890277	0.007333811	Arabidopsis thaliana uncharacterized protein mRNA, complete cds
AT1G72760	-1.407821465	0.007522878	Arabidopsis thaliana putative serine/threonine protein kinase mRNA, complete cds
AT1G73410	-1.170929474	0.007788893	Arabidopsis thaliana myb domain protein 54 mRNA, complete cds
AT2G35585	-1.103815279	0.008031173	Arabidopsis thaliana uncharacterized protein mRNA, complete cds
AT2G29710	-1.103815279	0.008031173	Arabidopsis thaliana chromosome 2, complete sequence
AT4G32785	-1.004279605	0.008042423	Arabidopsis thaliana uncharacterized protein mRNA, complete cds
AT1G35330	-1.004279605	0.008042423	Arabidopsis thaliana RING-H2 finger protein ATL34 mRNA, complete cds
AT1G74000	-1.049367495	0.00810254	Arabidopsis thaliana strictosidine synthase 3 mRNA, complete cds
ATMG01220	-3.103815279	0.008407207	Arabidopsis thaliana ecotype Landsberg erecta mitochondrion, complete genome
AT3G17050.1	-3.103815279	0.008407207	Arabidopsis thaliana chromosome 3, complete sequence
AT4G23215	-1.457452233	0.008506487	Arabidopsis thaliana chromosome 4 sequence
AT3G45960	-1.007890859	0.009484211	Arabidopsis thaliana expansin-like A3 mRNA, complete cds
AT1G66860	-1.518852778	0.00952913	Arabidopsis thaliana class I glutamine

			amidotransferase-like domain-containing protein mRNA, complete cds
AT5G44690	-1.3124019	0.009785506	Arabidopsis thaliana uncharacterized protein mRNA, complete cds
AT1G51830	-1.59685529	0.010534949	Arabidopsis thaliana putative leucine-rich repeat protein kinase mRNA, complete cds
AT4G33467	-2.393321896	0.011059208	Arabidopsis thaliana uncharacterized protein mRNA, complete cds
AT1G61800	-2.393321896	0.011059208	Arabidopsis thaliana glucose-6-phosphate/phosphate translocator 2 mRNA, complete cds
AT1G33920	-2.393321896	0.011059208	Arabidopsis thaliana phloem protein 2-A4 mRNA, complete cds
AT3G45280	-2.393321896	0.011059208	Arabidopsis thaliana syntaxin-72 mRNA, complete cds
AT1G21360	-2.393321896	0.011059208	Arabidopsis thaliana glycolipid transfer protein 2 mRNA, complete cds
AT1G63930	-1.126535355	0.011067021	Arabidopsis thaliana chromosome 1 sequence
AT1G17030	-1.011892789	0.011190792	Arabidopsis thaliana uncharacterized protein mRNA, complete cds
AT2G42430	-1.348927776	0.011246261	Arabidopsis thaliana LOB domain-containing protein 16 mRNA, complete cds
AT1G24420	-1.348927776	0.011246261	Arabidopsis thaliana chromosome 1 sequence
AT3G48640	-1.348927776	0.011246261	Arabidopsis thaliana chromosome 3, complete sequence
AT5G01180	-1.699425023	0.01142448	Arabidopsis thaliana peptide transporter PTR5 mRNA, complete cds
AT4G39000	-1.699425023	0.01142448	Arabidopsis thaliana glycosyl hydrolase 9B17 mRNA, complete cds
AT1G01453	-1.699425023	0.01142448	Arabidopsis thaliana chromosome 1 sequence
AT1G68350	-1.699425023	0.01142448	Arabidopsis thaliana chromosome 1 sequence
AT3G04181	-1.699425023	0.01142448	Arabidopsis thaliana uncharacterized protein mRNA, complete cds
AT5G54790	-1.840780873	0.012026993	Arabidopsis thaliana uncharacterized protein mRNA, complete cds
AT2G36270	-1.840780873	0.012026993	Arabidopsis thaliana protein abscisic acid-insensitive 5 mRNA, complete cds
AT2G22950	-2.049367495	0.012057352	Arabidopsis thaliana putative calcium-transporting ATPase 7 mRNA, complete cds
AT3G57110	-3.741245199	0.012127161	Arabidopsis thaliana chromosome 3, complete sequence
AT2G17740	-3.741245199	0.01212716	Arabidopsis thaliana chromosome 2,

		1	complete sequence
AT1G78330	-3.741245199	0.01212716 1	Arabidopsis thaliana chromosome 1 sequence
AT1G56165	-3.741245199	0.01212716 1	Arabidopsis thaliana clone 126147 mRNA sequence
AT5G53700	-3.741245199	0.01212716 1	Arabidopsis thaliana RNA-binding (RRM/RBD/RNP motifs) family protein mRNA, complete cds
AT2G47040	-3.741245199	0.01212716 1	Arabidopsis thaliana pectinesterase 5 mRNA, complete cds
AT1G29830	-1.229346161	0.01236070 9	Arabidopsis thaliana Magnesium transporter CorA-like family protein mRNA, complete cds
AT1G06980	-1.393321896	0.01285736 4	Arabidopsis thaliana chromosome 1 sequence
AT1G59725	-1.393321896	0.01285736 4	Arabidopsis thaliana putative DNAJ heat shock protein mRNA, complete cds
AT5G23950	-1.393321896	0.01285736 4	Arabidopsis thaliana chromosome 5 sequence
AT4G22610	-1.393321896	0.01285736 4	Arabidopsis thaliana chromosome 4 sequence
AT3G01960	-1.255818372	0.01437233 4	Arabidopsis thaliana uncharacterized protein mRNA, complete cds
AT4G01670	-1.255818372	0.01437233 4	Arabidopsis thaliana uncharacterized protein mRNA, complete cds
AT1G67980	-1.44846345	0.01459215 6	Arabidopsis thaliana caffeoyl-CoA 3-O-methyltransferase mRNA, complete cds
AT3G58380	-2.933890277	0.01557563 8	Arabidopsis thaliana TRAF-like family protein mRNA, complete cds
AT2G37000	-2.933890277	0.01557563 8	Arabidopsis thaliana chromosome 2, complete sequence
AT2G17890	-2.933890277	0.01557563 8	Arabidopsis thaliana calcium-dependent protein kinase 16 mRNA, complete cds
AT5G57550	-2.933890277	0.01557563 8	Arabidopsis thaliana probable xyloglucan endotransglucosylase/hydrolase protein 25 mRNA, complete cds
AT3G56660	-2.933890277	0.01557563 8	Arabidopsis thaliana basic region/leucine zipper motif protein 49 mRNA, complete cds
AT1G07460	-2.933890277	0.01557563 8	Arabidopsis thaliana chromosome 1 sequence
AT4G31250	-2.933890277	0.01557563 8	Arabidopsis thaliana putative LRR receptor-like serine/threonine-protein kinase mRNA, complete cds
AT1G18835	-2.933890277	0.01557563	Arabidopsis thaliana chromosome 1 sequence

		8	
AT4G33985	-1.080731665	0.01560565 3	Arabidopsis thaliana uncharacterized protein mRNA, complete cds
AT5G26690	-1.080731665	0.01560565 3	Arabidopsis thaliana heavy-metal-associated domain-containing protein mRNA, complete cds
ATMG01080	-1.080731665	0.01560565 3	Arabidopsis thaliana ecotype Landsberg erecta mitochondrion, complete genome
AT3G14510	-1.518852778	0.01638814 6	Arabidopsis thaliana putative geranylgeranyl pyrophosphate synthase 8 mRNA, complete cds
AT5G11190	-1.518852778	0.01638814 6	Arabidopsis thaliana ethylene-responsive transcription factor SHINE 2 mRNA, complete cds
AT5G51930	-1.518852778	0.01638814 6	Arabidopsis thaliana Glucose-methanol-choline (GMC) oxidoreductase family protein mRNA, complete cds
AT5G38790	-1.518852778	0.01638814 6	Arabidopsis thaliana chromosome 5 sequence
AT2G03370	-1.518852778	0.01638814 6	Arabidopsis thaliana Glycosyltransferase family 61 protein mRNA, complete cds
AT2G43390	-1.518852778	0.01638814 6	Arabidopsis thaliana chromosome 2, complete sequence
AT3G56275	-1.518852778	0.01638814 6	Arabidopsis thaliana chromosome 3, complete sequence
AT5G11290	-1.174898377	0.01786403 4	Arabidopsis thaliana chromosome 5 sequence
AT2G31945	-1.174898377	0.01786403 4	Arabidopsis thaliana chromosome 2, complete sequence
AT1G63910	-1.611962182	0.018119365	Arabidopsis thaliana myb domain protein 103 mRNA, complete cds
AT3G04330	-1.611962182	0.018119365	Arabidopsis thaliana chromosome 3, complete sequence
AT4G11390	-1.611962182	0.018119365	Arabidopsis thaliana cysteine/histidine-rich C1 domain-containing protein mRNA, complete cds
AT3G49820	-1.611962182	0.018119365	Arabidopsis thaliana chromosome 3, complete sequence
AT1G19200	-1.091431554	0.01839902 5	Arabidopsis thaliana uncharacterized protein mRNA, complete cds
AT1G17285	-1.091431554	0.01839902 5	Arabidopsis thaliana uncharacterized protein mRNA, complete cds
AT1G58225	-1.091431554	0.01839902	Arabidopsis thaliana uncharacterized protein

		5	mRNA, complete cds
AT5G59510	-1.091431554	0.01839902 5	Arabidopsis thaliana chromosome 5 sequence
AT2G19050	-1.3262077	0.01925853 2	Arabidopsis thaliana GDSL esterase/lipase mRNA, complete cds
AT5G17860	-1.3262077	0.01925853 2	Arabidopsis thaliana chromosome 5 sequence
AT2G45580	-1.3262077	0.01925853 2	Arabidopsis thaliana cytochrome P450 76C3 mRNA, complete cds
AT1G07795	-2.255818372	0.01936362 1	Arabidopsis thaliana chromosome 1 sequence
AT5G10230	-2.255818372	0.01936362 1	Arabidopsis thaliana annexin D7 mRNA, complete cds
AT1G61120	-2.255818372	0.01936362 1	Arabidopsis thaliana terpene synthase 04 mRNA, complete cds
AT2G46480	-2.255818372	0.01936362 1	Arabidopsis thaliana probable galacturonosyltransferase 2 mRNA, complete cds
AT1G03580	-2.255818372	0.01936362 1	Arabidopsis thaliana chromosome 1 sequence
AT5G26630	-2.255818372	0.01936362 1	Arabidopsis thaliana chromosome 5 sequence
AT1G79680	-2.255818372	0.01936362 1	Arabidopsis thaliana wall-associated receptor kinase-like 10 mRNA, complete cds
AT1G01810	-1.741245199	0.01954689 9	Arabidopsis thaliana chromosome 1 sequence
AT3G63060	-1.741245199	0.01954689 9	Arabidopsis thaliana chromosome 3, complete sequence
AT3G44840	-1.741245199	0.01954689 9	Arabidopsis thaliana S-adenosyl-L-methionine-dependent methyltransferases superfamily protein mRNA, complete cds
AT5G51210	-1.933890277	0.02022925 6	Arabidopsis thaliana oleosin3 mRNA, complete cds
AT2G43470	-1.933890277	0.02022925 6	Arabidopsis thaliana uncharacterized protein mRNA, complete cds
AT3G48630	-1.933890277	0.02022925 6	Arabidopsis thaliana uncharacterized protein mRNA, complete cds
AT1G13145	-1.196924683	0.02092488 7	Arabidopsis thaliana chromosome 1 sequence
AT1G09480	-1.103815279	0.02169750 5	Arabidopsis thaliana alcohol dehydrogenase-like protein mRNA, complete cds
AT1G25054	-1.103815279	0.02169750	Arabidopsis thaliana

		5	UDP-3-O-[3-hydroxymyristoyl] N-acetylglucosamine deacetylase mRNA, complete cds
AT5G01480	-1.103815279	0.021697505	Arabidopsis thaliana chromosome 5 sequence
AT4G22630	-1.033425951	0.021831173	Arabidopsis thaliana bifunctional inhibitor/lipid-transfer protein/seed storage 2S albumin superfamily protein mRNA, complete cds
AT1G52530	-1.033425951	0.021831173	Arabidopsis thaliana Hus1 domain-containing protein mRNA, complete cds
AT5G53980	-1.374462869	0.022135212	Arabidopsis thaliana chromosome 5 sequence
AT3G47770	-1.374462869	0.022135212	Arabidopsis thaliana ABC transporter A family member 6 mRNA, complete cds
AT1G80470	-3.518852778	0.023200279	Arabidopsis thaliana F-box/FBD/LRR-repeat protein mRNA, complete cds
ATCG01090	-3.518852778	0.023200279	Arabidopsis thaliana chloroplast DNA, complete genome, ecotype: Columbia
AT5G35407	-3.518852778	0.023200279	Arabidopsis thaliana chromosome 5 sequence
AT3G62320	-3.518852778	0.023200279	Arabidopsis thaliana putative nucleic acid binding protein mRNA, complete cds
AT1G76230	-3.518852778	0.023200279	Arabidopsis thaliana chromosome 1 sequence
AT1G34490	-3.518852778	0.023200279	Arabidopsis thaliana chromosome 1 sequence
AT5G62040	-3.518852778	0.023200279	Arabidopsis thaliana protein BROTHER of FT and TFL 1 mRNA, complete cds
AT5G50360	-3.518852778	0.023200279	Arabidopsis thaliana chromosome 5 sequence
AT5G01080	-3.518852778	0.023200279	Arabidopsis thaliana chromosome 5 sequence
AT3G43850	-3.518852778	0.023200279	Arabidopsis thaliana uncharacterized protein mRNA, complete cds
AT5G20045	-3.518852778	0.023200279	Arabidopsis thaliana chromosome 5 sequence
AT4G34810	-1.223396894	0.024475655	Arabidopsis thaliana chromosome 4 sequence
AT3G15700	-1.223396894	0.024475655	Arabidopsis thaliana P-loop containing nucleoside triphosphate hydrolases superfamily protein mRNA, complete cds
AT5G54490	-1.223396894	0.024475655	Arabidopsis thaliana chromosome 5 sequence

AT2G32210	-1.436390618	0.025244545	Arabidopsis thaliana uncharacterized protein mRNA, complete cds
AT4G01930	-1.436390618	0.025244545	Arabidopsis thaliana chromosome 4 sequence
AT1G10790	-1.436390618	0.025244545	Arabidopsis thaliana uncharacterized protein mRNA, complete cds
AT5G41685	-1.436390618	0.025244545	Arabidopsis thaliana chromosome 5 sequence
AT4G26770	-1.436390618	0.025244545	Arabidopsis thaliana phosphatidate cytidylyltransferase mRNA, complete cds
AT4G20362	-1.040805481	0.025847187	Arabidopsis thaliana Full-length cDNA Complete sequence from clone GSLTPGH40ZC03 of Hormone Treated Callus of strain col-0 of Arabidopsis thaliana (thale cress)
AT5G47590	-1.040805481	0.025847187	Arabidopsis thaliana Heat shock protein HSP20/alpha crystallin family protein mRNA, complete cds
AT1G48220	-1.040805481	0.025847187	Arabidopsis thaliana protein kinase family prtein mRNA, complete cds
AT2G32020	-1.040805481	0.025847187	Arabidopsis thaliana chromosome 2, complete sequence
AT3G23230	-1.040805481	0.025847187	Arabidopsis thaliana chromosome 3, complete sequence
AT1G73965	-1.518852778	0.028449529	Arabidopsis thaliana chromosome 1 sequence
AT3G21351	-1.518852778	0.028449529	Arabidopsis thaliana uncharacterized protein mRNA, complete cds
AT1G70660	-1.518852778	0.028449529	Arabidopsis thaliana ubiquitin-conjugating enzyme E2 variant 1B mRNA, complete cds
AT3G09330	-1.255818372	0.028568016	Arabidopsis thaliana transmembrane amino acid transporter-like protein mRNA, complete cds
AT5G24040	-1.255818372	0.028568016	Arabidopsis thaliana uncharacterized protein mRNA, complete cds
AT2G01580	-1.255818372	0.028568016	Arabidopsis thaliana chromosome 2, complete sequence
AT3G51680	-1.255818372	0.028568016	Arabidopsis thaliana short-chain dehydrogenase reductase 2a mRNA, complete cds
AT5G35495.1	-2.741245199	0.028837806	Arabidopsis thaliana chromosome 5 sequence
AT1G11560	-2.741245199	0.028837806	Arabidopsis thaliana chromosome 1 sequence

AT2G04490.1	-2.741245199	0.028837806	Arabidopsis thaliana chromosome 2, complete sequence
AT4G26790	-2.741245199	0.028837806	Arabidopsis thaliana GDSL esterase/lipase mRNA, complete cds
AT3G11050	-1.135524138	0.030182724	Arabidopsis thaliana ferritin 2 mRNA, complete cds
AT5G43240	-1.049367495	0.030626511	Arabidopsis thaliana uncharacterized protein mRNA, complete cds
AT2G07808	-1.049367495	0.030626511	Arabidopsis thaliana chromosome 2, complete sequence
AT4G01895	-1.049367495	0.030626511	Arabidopsis thaliana chromosome 4 sequence
AT1G43590.1	-1.634329995	0.031449726	Arabidopsis thaliana chromosome 1 sequence
AT2G34330	-1.634329995	0.031449726	Arabidopsis thaliana chromosome 2, complete sequence
AT5G16330	-1.634329995	0.031449726	Arabidopsis thaliana NC domain-containing protein-like protein mRNA, complete cds
AT1G25422	-1.634329995	0.031449726	Arabidopsis thaliana chromosome 1 sequence
AT1G49832	-1.296460357	0.033236484	Arabidopsis thaliana chromosome 1 sequence
AT3G63360	-1.296460357	0.033236484	Arabidopsis thaliana defensin-like protein mRNA, complete cds
AT3G03776	-1.296460357	0.033236484	Arabidopsis thaliana hydroxyproline-rich glycoprotein family protein mRNA, complete cds
ATCG00420	-1.808359395	0.033618536	Arabidopsis thaliana chloroplast DNA, complete genome, ecotype: Columbia
AT5G07700	-1.808359395	0.033618536	Arabidopsis thaliana myb domain protein 76 mRNA, complete cds
AT5G41570	-1.808359395	0.033618536	Arabidopsis thaliana WRKY transcription factor 24 mRNA, complete cds
AT1G19371	-1.808359395	0.033618536	Arabidopsis thaliana chromosome 1 sequence
AT1G47620	-2.103815279	0.033654305	Arabidopsis thaliana chromosome 1 sequence
AT5G14110	-2.103815279	0.033654305	Arabidopsis thaliana uncharacterized protein mRNA, complete cds
AT3G16210	-2.103815279	0.033654305	Arabidopsis thaliana chromosome 3, complete sequence
AT2G37720	-2.103815279	0.033654305	Arabidopsis thaliana trichome birefringence-like 15 protein mRNA, complete cds
AT1G51770	-2.103815279	0.03365430	Arabidopsis thaliana core-2/I-branching

		5	beta-1,6-N-acetylglucosaminyltransferase family protein mRNA, complete cds
AT3G14185	-1.156282698	0.035589571	Arabidopsis thaliana Full-length cDNA Complete sequence from clone GSLTSIL56ZC04 of Silique of strain col-0 of Arabidopsis thaliana (thale cress)
AT1G61470	-1.156282698	0.035589571	Arabidopsis thaliana chromosome 1 sequence
AT1G16225	-1.156282698	0.035589571	Arabidopsis thaliana putative syntaxin-type t-SNARE protein mRNA, complete cds
AT1G77200	-1.059421159	0.036319869	Arabidopsis thaliana chromosome 1 sequence
AT5G01060	-1.059421159	0.036319869	Arabidopsis thaliana Protein kinase protein with tetratricopeptide repeat domain mRNA, complete cds
AT5G02580	-1.059421159	0.036319869	Arabidopsis thaliana uncharacterized protein mRNA, complete cds
AT3G06100	-1.348927776	0.038472987	Arabidopsis thaliana putative aquaporin NIP7-1 mRNA, complete cds
AT5G07060	-1.348927776	0.038472987	Arabidopsis thaliana MOS4-associated complex subunit 5C mRNA, complete cds
AT2G35612	-1.348927776	0.038472987	Arabidopsis thaliana chromosome 2, complete sequence
AT5G38770	-1.348927776	0.038472987	Arabidopsis thaliana chromosome 5 sequence
AT1G69430	-1.181817791	0.041938324	Arabidopsis thaliana chromosome 1 sequence
AT3G16900	-1.181817791	0.041938324	Arabidopsis thaliana uncharacterized protein mRNA, complete cds
AT2G22880	-1.071393801	0.043108412	Arabidopsis thaliana chromosome 2, complete sequence
AT1G61830	-1.071393801	0.043108412	Arabidopsis thaliana chromosome 1 sequence
AT3G14630	-1.071393801	0.043108412	Arabidopsis thaliana cytochrome P450, family 72, subfamily A, polypeptide 9 mRNA, complete cds
AT1G49370	-1.419317104	0.044174267	Arabidopsis thaliana chromosome 1 sequence
AT1G50390	-1.419317104	0.044174267	Arabidopsis thaliana pfkB-like carbohydrate kinase family protein mRNA, complete cds
AT1G78440	-1.419317104	0.044174267	Arabidopsis thaliana gibberellin 2-beta-dioxygenase 1 mRNA, complete cds
AT4G37050	-1.419317104	0.044174267	Arabidopsis thaliana PATATIN-like protein 4 mRNA, complete cds

ATMG00810	-3.255818372	0.044766751	Arabidopsis thaliana ecotype Landsberg erecta mitochondrion, complete genome
AT5G37620	-3.255818372	0.044766751	Arabidopsis thaliana chromosome 5 sequence
AT3G05780	-3.255818372	0.044766751	Arabidopsis thaliana lon protease 3 mRNA, complete cds
AT5G51105	-3.255818372	0.044766751	Arabidopsis thaliana chromosome 5 sequence
AT3G29970	-3.255818372	0.044766751	Arabidopsis thaliana B12D protein mRNA, complete cds
AT3G49300	-3.255818372	0.044766751	Arabidopsis thaliana proline-rich family protein mRNA, complete cds
AT2G21610	-3.255818372	0.044766751	Arabidopsis thaliana pectinesterase 11 mRNA, complete cds
AT3G11380	-3.255818372	0.044766751	Arabidopsis thaliana pentatricopeptide repeat-containing protein mRNA, complete cds
AT1G15050	-3.255818372	0.044766751	Arabidopsis thaliana auxin-responsive protein IAA34 mRNA, complete cds
AT2G21780	-3.255818372	0.044766751	Arabidopsis thaliana chromosome 2, complete sequence
AT4G15970	-3.255818372	0.044766751	Arabidopsis thaliana Nucleotide-diphospho-sugar transferase family protein mRNA, complete cds
AT4G26930	-3.255818372	0.044766751	Arabidopsis thaliana myb domain protein 97 mRNA, complete cds
AT1G36675	-1.213998196	0.049356736	Arabidopsis thaliana glycine-rich protein mRNA, complete cds
AT5G41590	-1.213998196	0.049356736	Arabidopsis thaliana uncharacterized protein mRNA, complete cds
AT4G22214	-1.213998196	0.049356736	Arabidopsis thaliana defensin-like protein 99 mRNA, complete cds
AT1G06923	-1.213998196	0.049356736	Arabidopsis thaliana uncharacterized protein mRNA, complete cds
AT3G10815	-1.213998196	0.049356736	Arabidopsis thaliana RING/U-box domain-containing protein mRNA, complete cds
AT2G18550	-1.518852778	0.050028779	Arabidopsis thaliana homeobox-leucine zipper protein ATHB-21 mRNA, complete cds
AT4G36230	-1.518852778	0.050028779	Arabidopsis thaliana chromosome 4 sequence
ATCG00400	-1.518852778	0.050028779	Oltmannsiellopsis viridis chloroplast, complete genome

AT3G29252	-1.518852778	0.050028779	Arabidopsis thaliana chromosome 3, complete sequence
AT5G39720	-1.518852778	0.050028779	Arabidopsis thaliana avirulence induced protein 2 like protein mRNA, complete cds
AT4G11370	-1.085893371	0.051209119	Arabidopsis thaliana chromosome 4 sequence
AT3G06490	-2.518852778	0.053304086	Arabidopsis thaliana putative transcription factor MYB108 mRNA, complete cds
AT2G29170	-2.518852778	0.053304086	Arabidopsis thaliana NAD(P)-binding Rossmann-fold superfamily protein mRNA, complete cds
AT2G25770	-2.518852778	0.053304086	Arabidopsis thaliana chromosome 2, complete sequence
AT3G45170	-2.518852778	0.053304086	Arabidopsis thaliana GATA transcription factor 14 mRNA, complete cds
AT2G01770	-2.518852778	0.053304086	Arabidopsis thaliana vacuolar iron transporter 1 mRNA, complete cds
AT5G21030	-2.518852778	0.053304086	Arabidopsis thaliana protein argonaute 8 mRNA, complete cds
AT4G31950	-2.518852778	0.053304086	Arabidopsis thaliana cytochrome P450, family 82, subfamily C, polypeptide 3 mRNA, complete cds
AT2G27220	-2.518852778	0.053304086	Arabidopsis thaliana BEL1-like homeodomain 5 mRNA, complete cds
AT2G42440	-2.518852778	0.053304086	Arabidopsis thaliana LOB domain-containing protein 17 mRNA, complete cds
AT4G11385	-2.518852778	0.053304086	Arabidopsis thaliana uncharacterized protein mRNA, complete cds
AT2G28670	-2.518852778	0.053304086	Arabidopsis thaliana protein ENHANCED SUBERIN 1 mRNA, complete cds
AT5G39260	-2.518852778	0.053304086	Arabidopsis thaliana expansin A21 mRNA, complete cds
AT4G33310	-2.518852778	0.053304086	Arabidopsis thaliana chromosome 4 sequence
AT1G62190	-2.518852778	0.053304086	Arabidopsis thaliana chromosome 1 sequence
AT4G15075	-2.518852778	0.053304086	Arabidopsis thaliana FBD-like domain family protein mRNA, complete cds
AT3G04410	-2.518852778	0.053304086	Arabidopsis thaliana no apical meristem-domain containing transcriptional regulator mRNA, complete cds
AT3G49150	-2.518852778	0.053304086	Arabidopsis thaliana putative F-box/LRR-repeat protein mRNA, complete cds
AT1G35255	-2.518852778	0.05330408	Arabidopsis thaliana chromosome 1 sequence

		6	
AT1G09380	-1.670855871	0.05525771 5	Arabidopsis thaliana nodulin MtN21-like transporter family protein mRNA, complete cds
AT5G59370	-1.670855871	0.05525771 5	Arabidopsis thaliana actin 4 mRNA, complete cds
AT2G23400	-1.670855871	0.05525771 5	Arabidopsis thaliana undecaprenyl pyrophosphate synthetase family protein mRNA, complete cds
AT2G35890	-1.255818372	0.05795039 2	Arabidopsis thaliana calcium-dependent protein kinase 25 mRNA, complete cds
AT5G10570	-1.255818372	0.05795039 2	Arabidopsis thaliana transcription factor bHLH61 mRNA, complete cds
AT4G03600	-1.255818372	0.05795039 2	Arabidopsis thaliana chromosome 4 sequence
AT3G59730	-1.255818372	0.05795039 2	Arabidopsis thaliana chromosome 3, complete sequence
AT1G69050	-1.255818372	0.05795039 2	Arabidopsis thaliana chromosome 1 sequence
AT3G21780	-1.255818372	0.05795039 2	Arabidopsis thaliana chromosome 3, complete sequence
AT3G50290	-1.255818372	0.05795039 2	Arabidopsis thaliana chromosome 3, complete sequence
AT4G29610	-1.255818372	0.05795039 2	Arabidopsis thaliana chromosome 4 sequence
AT1G34520	-1.255818372	0.05795039 2	Arabidopsis thaliana MBOAT (membrane bound O-acyl transferase) family protein mRNA, complete cds
AT4G19000	-1.933890277	0.05796523 7	Arabidopsis thaliana INTERACTS WITH SPT6-like protein IWS2 mRNA, complete cds
AT3G12835	-1.933890277	0.05796523 7	Arabidopsis thaliana chromosome 3, complete sequence
AT1G65670	-1.933890277	0.05796523 7	Arabidopsis thaliana cytochrome P450, family 702, subfamily A, polypeptide 1 mRNA, complete cds
AT1G67265	-1.933890277	0.05796523 7	Arabidopsis thaliana chromosome 1 sequence
AT1G25240	-1.933890277	0.05796523 7	Arabidopsis thaliana chromosome 1 sequence
AT1G27921	-1.933890277	0.05796523 7	Arabidopsis thaliana chromosome 1 sequence
AT4G10860	-1.933890277	0.05796523 7	Arabidopsis thaliana chromosome 4 sequence

AT4G03470	-1.103815279	0.060879843	Arabidopsis thaliana ankyrin repeat-containing protein mRNA, complete cds
AT3G01830	-1.103815279	0.060879843	Arabidopsis thaliana chromosome 3, complete sequence
AT3G49510	-1.103815279	0.060879843	Arabidopsis thaliana F-box protein mRNA, complete cds
AT5G45740	-1.103815279	0.060879843	Arabidopsis thaliana ubiquitin domain-containing protein mRNA, complete cds
AT4G30370	-1.103815279	0.060879843	Arabidopsis thaliana chromosome 4 sequence
AT5G14070	-1.103815279	0.060879843	Arabidopsis thaliana chromosome 5 sequence
AT1G34540	-1.103815279	0.060879843	Arabidopsis thaliana chromosome 1 sequence
AT5G23700	-1.004279605	0.060925936	Arabidopsis thaliana uncharacterized protein mRNA, complete cds
AT1G35515	-1.004279605	0.060925936	Arabidopsis thaliana R2R3-type MYB transcription factor mRNA, complete cds
AT3G53450	-1.004279605	0.060925936	Arabidopsis thaliana cytokinin riboside 5'-monophosphate phosphoribohydrolase LOG4 mRNA, complete cds
AT4G40065	-1.004279605	0.060925936	Arabidopsis thaliana chromosome 4 sequence
AT4G15280	-1.004279605	0.060925936	Arabidopsis thaliana chromosome 4 sequence

Supplementary Data S3A Up regulated genes in Col-0(+NaCl)/Col-0(-NaCl)

Gene	Log2FoldChange	pval	NT:Description
AT3G45140	4.374662667	0	Arabidopsis thaliana lipoxygenase 2 mRNA, complete cds
AT2G33380	5.898717978	0	Arabidopsis thaliana caleosin 3 mRNA, complete cds
AT1G20450	4.368365537	0	Arabidopsis thaliana dehydrin ERD10 mRNA, complete cds
AT5G24770	4.819055243	0	Arabidopsis thaliana acid phosphatase VSP2 mRNA, complete cds
AT4G34710	3.929306064	0	Arabidopsis thaliana chromosome 4 sequence
AT1G20440	3.086931932	0	Arabidopsis thaliana dehydrin COR47 mRNA, complete cds
AT2G34420	1.292975286	0	Arabidopsis thaliana chromosome 2, complete sequence
AT5G52310	7.310794927	0	Arabidopsis thaliana protein LOW-TEMPERATURE-INDUCED 78 mRNA, complete cds
AT2G39800	4.649626307	0	Arabidopsis thaliana delta1-pyrroline-5-carboxylate synthase 1 mRNA, complete cds
AT4G23600	6.730133552	0	Arabidopsis thaliana cystine lyase CORI3 mRNA, complete cds
AT1G52400	3.021806808	0	Arabidopsis thaliana beta glucosidase 18 mRNA, complete cds
AT4G08870	4.257371441	0	Arabidopsis thaliana arginine amidohydrolase 2 mRNA, complete cds
AT1G54100	4.276708384	0	Arabidopsis thaliana aldehyde dehydrogenase 7B4 mRNA, complete cds
AT3G23920	3.186208668	0	Arabidopsis thaliana beta-amylase 1 mRNA, complete cds
AT1G73480	5.456337718	0	Arabidopsis thaliana alpha/beta-Hydrolases superfamily protein mRNA, complete cds
AT2G42540	8.102240161	0	Arabidopsis thaliana cold-regulated protein 15a mRNA, complete cds
AT2G22240	4.86854403	0	Arabidopsis thaliana myo-inositol-1-phosphate synthase 2 mRNA, complete cds
AT4G16760	3.27838863	0	Arabidopsis thaliana peroxisomal acyl-coenzyme A oxidase 1 mRNA,

			complete cds
AT5G25610	2.345661316	0	Arabidopsis thaliana dehydration-responsive protein RD22 mRNA, complete cds
AT5G15970	5.712784765	0	Arabidopsis thaliana stress-induced protein KIN2 mRNA, complete cds
AT1G19570	2.873620907	0	Arabidopsis thaliana dehydroascorbate reductase mRNA, complete cds
AT4G27520	2.142789069	0	Arabidopsis thaliana early nodulin-like protein 2 mRNA, complete cds
AT1G61890	3.817869132	0	Arabidopsis thaliana MATE efflux family protein mRNA, complete cds
AT2G47180	5.710272199	0	Arabidopsis thaliana galactinol synthase 1 mRNA, complete cds
AT4G00430	1.86040886	0	Arabidopsis thaliana plasma membrane intrinsic protein 1;4 mRNA, complete cds
AT5G59220	6.91254613	0	Arabidopsis thaliana putative protein phosphatase 2C 78 mRNA, complete cds
AT3G25770	4.554405592	0	Arabidopsis thaliana allene oxide cyclase 2 mRNA, complete cds
AT4G26080	3.594478031	0	Arabidopsis thaliana protein phosphatase 2C 56 mRNA, complete cds
AT3G28220	4.284666517	0	Arabidopsis thaliana TRAF-like family protein mRNA, complete cds
AT3G44310	1.275214556	0	Arabidopsis thaliana nitrilase 1 mRNA, complete cds
AT2G34810	4.675503634	0	Arabidopsis thaliana chromosome 2, complete sequence
AT2G15970	3.654569603	0	Arabidopsis thaliana cold regulated 413 plasma membrane 1 mRNA, complete cds
AT5G42650	2.614129621	0	Arabidopsis thaliana chromosome 5 sequence
AT3G11410	3.344123487	0	Arabidopsis thaliana protein phosphatase 2CA mRNA, complete cds
AT1G02205	2.903990979	0	Arabidopsis thaliana protein ECERIFERUM 1 mRNA, complete cds
AT1G72770	2.733269271	0	Arabidopsis thaliana protein phosphatase 2C 16 mRNA, complete cds
AT5G50950	4.310963202	0	Arabidopsis thaliana fumarate hydratase 2 mRNA, complete cds
AT4G27410	4.848542389	0	Arabidopsis thaliana NAC transcription factor RD26 mRNA, complete cds

AT1G60190	5.755880784	0	Arabidopsis thaliana chromosome 1 sequence
AT1G32640	2.440084796	0	Arabidopsis thaliana chromosome 1 sequence
AT2G24850	6.701000525	0	Arabidopsis thaliana tyrosine aminotransferase 3 mRNA, complete cds
AT1G78070	3.495536065	0	Arabidopsis thaliana transducin/WD40 repeat-like superfamily protein mRNA, complete cds
AT5G59320	6.434739583	0	Arabidopsis thaliana non-specific lipid-transfer protein 3 mRNA, complete cds
AT3G29575	5.063470169	0	Arabidopsis thaliana Ninja-family protein AFP3 mRNA, complete cds
AT1G19180	3.655699094	0	Arabidopsis thaliana chromosome 1 sequence
AT1G76180	1.38958777	0	Arabidopsis thaliana dehydrin ERD14 mRNA, complete cds
AT2G38470	2.967639379	0	Arabidopsis thaliana putative WRKY transcription factor 33 mRNA, complete cds
AT5G61820	3.216119487	0	Arabidopsis thaliana uncharacterized protein mRNA, complete cds
AT3G61890	4.460944953	0	Arabidopsis thaliana homeobox-leucine zipper protein ATHB-12 mRNA, complete cds
AT4G04020	2.851127073	0	Arabidopsis thaliana fibrillin mRNA, complete cds
AT3G17800	2.276914513	0	Arabidopsis thaliana uncharacterized protein mRNA, complete cds
AT2G25450	2.083835016	0	Arabidopsis thaliana 1-aminocyclopropane-1-carboxylate oxidase-like protein mRNA, complete cds
AT4G22240	2.184468808	0	Arabidopsis thaliana plastid-lipid associated protein PAP / fibrillin family protein mRNA, complete cds
AT1G56600	5.767338709	0	Arabidopsis thaliana galactinol synthase 2 mRNA, complete cds
AT3G16400	1.894498096	0	Arabidopsis thaliana Nitrile-specifier protein 1 mRNA, complete cds
AT1G17420	4.090649619	0	Arabidopsis thaliana lipoxygenase 3 mRNA, complete cds
AT4G34000	3.241746441	0	Arabidopsis thaliana abscisic acid

			responsive elements-binding factor 3 mRNA, complete cds
AT2G37180	2.32386768	0	Arabidopsis thaliana aquaporin PIP2-3 mRNA, complete cds
AT3G14067	1.817781867	0	Arabidopsis thaliana chromosome 3, complete sequence
AT1G58270	3.123919325	0	Arabidopsis thaliana protein ZW9 mRNA, complete cds
AT5G67030	1.625690377	0	Arabidopsis thaliana zeaxanthin epoxidase mRNA, complete cds
AT1G01470	2.844856899	0	Arabidopsis thaliana putative desiccation-related protein LEA14 mRNA, complete cds
AT4G30960	1.958663083	0	Arabidopsis thaliana chromosome 4 sequence
AT1G69260	4.807183648	0	Arabidopsis thaliana ABI five binding protein mRNA, complete cds
AT3G25760	4.424125628	0	Arabidopsis thaliana allene oxide cyclase 1 mRNA, complete cds
AT4G34230	3.088274747	0	Arabidopsis thaliana cinnamyl alcohol dehydrogenase 5 mRNA, complete cds
AT4G24960	3.35191555	0	Arabidopsis thaliana HVA22-like protein d mRNA, complete cds
AT1G51760	2.767500963	0	Arabidopsis thaliana IAA-amino acid hydrolase IAR3 mRNA, complete cds
AT5G67300	2.523384159	0	Arabidopsis thaliana chromosome 5 sequence
AT2G23120	2.879879484	0	Arabidopsis thaliana chromosome 2, complete sequence
AT5G24780	4.37330405	0	Arabidopsis thaliana acid phosphatase VSP1 mRNA, complete cds
AT5G19110	4.471813016	0	Arabidopsis thaliana Eukaryotic aspartyl protease family protein mRNA, complete cds
AT2G29450	1.56487595	0	Arabidopsis thaliana glutathione S-transferase tau 5 mRNA, complete cds
AT1G79270	2.70078265	0	Arabidopsis thaliana uncharacterized protein mRNA, complete cds
AT5G51070	1.98919211	0	Arabidopsis thaliana chaperone protein ClpD mRNA, complete cds
AT1G49450	5.513622173	0	Arabidopsis thaliana chromosome 1 sequence
AT2G17840	2.683723954	0	Arabidopsis thaliana

			senescence/dehydration related protein mRNA, complete cds
AT1G52000	3.359140497	0	Arabidopsis thaliana mannose-binding lectin superfamily protein mRNA, complete cds
AT3G14440	5.813924396	0	Arabidopsis thaliana chromosome 3, complete sequence
AT2G41190	4.988348817	0	Arabidopsis thaliana transmembrane amino acid transporter family protein mRNA, complete cds
AT4G18440	2.955986581	0	Arabidopsis thaliana L-aspartase-like family protein mRNA, complete cds
AT2G05070	1.074790718	0	Arabidopsis thaliana photosystem II light harvesting complex protein 2.2 mRNA, complete cds
AT5G24120	2.779239115	0	Arabidopsis thaliana RNA polymerase sigma factor 5 mRNA, complete cds
AT1G43160	5.842161988	0	Arabidopsis thaliana ethylene-responsive transcription factor RAP2-6 mRNA, complete cds
AT5G50920	1.008073556	0	Arabidopsis thaliana ATP-dependent Clp protease ATP-binding subunit ClpC mRNA, complete cds
AT4G23630	1.669499103	0	Arabidopsis thaliana VIRB2-interacting protein 1 mRNA, complete cds
AT3G05640	4.343480875	0	Arabidopsis thaliana putative protein phosphatase 2C mRNA, complete cds
AT3G02480	8.645123038	0	Arabidopsis thaliana Late embryogenesis abundant protein (LEA) family protein mRNA, complete cds
AT5G05600	3.943438859	0	Arabidopsis thaliana oxidoreductase, 2OG-Fe(II) oxygenase family protein mRNA, complete cds
AT3G50930	3.62607642	0	Arabidopsis thaliana chromosome 3, complete sequence
AT1G19670	2.599569731	0	Arabidopsis thaliana chlorophyllase 1 mRNA, complete cds
AT2G06050	2.996379117	0	Arabidopsis thaliana AT2G06050 mRNA, complete cds, clone: RAFL22-42-B17
AT1G52410	3.76721523	0	Arabidopsis thaliana TSK-associating protein 1 mRNA, complete cds
AT2G46680	4.51224788	0	Arabidopsis thaliana homeobox-leucine zipper protein ATHB-7 mRNA, complete

			cds
AT4G05050	1.21097705	0	Arabidopsis thaliana chromosome 4 sequence
AT5G64310	2.292490822	0	Arabidopsis thaliana chromosome 5 sequence
AT1G09310	1.115555689	0	Arabidopsis thaliana chromosome 1 sequence
AT4G21570	2.692378935	0	Arabidopsis thaliana uncharacterized protein mRNA, complete cds
AT3G22830	7.325053324	0	Arabidopsis thaliana heat stress transcription factor A-6b mRNA, complete cds
AT5G20230	2.597393824	0	Arabidopsis thaliana blue-copper-binding protein mRNA, complete cds
AT2G46270	3.436224704	0	Arabidopsis thaliana G-box binding factor 3 mRNA, complete cds
AT1G70700	1.905601622	0	Arabidopsis thaliana putative jasmonate signaling protein JAZ9 mRNA, complete cds
AT3G27690	1.698150817	0	Arabidopsis thaliana photosystem II light harvesting complex protein 2.3 mRNA, complete cds
AT1G01720	2.910805082	0	Arabidopsis thaliana putative transcriptional activator with NAC domain mRNA, complete cds
AT3G16470	1.736633782	0	Arabidopsis thaliana JA-responsive protein 1 mRNA, complete cds
AT2G39330	3.881690307	0	Arabidopsis thaliana jacalin-related lectin 23 mRNA, complete cds
AT5G08790	3.364837164	0	Arabidopsis thaliana protein ATAF2 mRNA, complete cds
AT3G44860	5.391970918	0	Arabidopsis thaliana farnesoic acid carboxyl-O-methyltransferase mRNA, complete cds
AT4G11280	2.823016685	0	Arabidopsis thaliana 1-aminocyclopropane-1-carboxylate synthase 6 mRNA, complete cds
AT5G64840	1.268090702	0	Arabidopsis thaliana general control non-repressible 5 mRNA, complete cds
AT2G04350	2.873267839	0	Arabidopsis thaliana long chain acyl-CoA synthetase 8 mRNA, complete cds
AT1G48100	4.26748864	0	Arabidopsis thaliana polygalacturonase mRNA, complete cds

AT2G38540	1.125543446	0	Arabidopsis thaliana non-specific lipid-transfer protein 1 mRNA, complete cds
AT5G63790	3.146562864	0	Arabidopsis thaliana NAC domain-containing protein 102 mRNA, complete cds
AT3G06500	3.144343902	0	Arabidopsis thaliana protein alkaline/neutral invertase C mRNA, complete cds
AT1G73390	2.721490155	0	Arabidopsis thaliana Endosomal targeting BRO1-like domain-containing protein mRNA, complete cds
AT5G20830	2.674388591	0	Arabidopsis thaliana sucrose synthase 1 mRNA, complete cds
AT4G33150	3.005505278	0	Arabidopsis thaliana lysine-ketoglutarate reductase/saccharopine dehydrogenase bifunctional enzyme mRNA, complete cds
AT1G32900	1.621590384	0	Arabidopsis thaliana granule-bound starch synthase 1 mRNA, complete cds
AT2G46830	1.260137839	0	Arabidopsis thaliana protein CCA1 mRNA, complete cds
AT3G50970	4.486402052	0	Arabidopsis thaliana chromosome 3, complete sequence
AT1G10370	3.813788907	0	Arabidopsis thaliana glutathione S-transferase U17 mRNA, complete cds
AT5G53120	2.8754262	0	Arabidopsis thaliana Spermine synthase mRNA, complete cds
AT5G60360	1.153344373	0	Arabidopsis thaliana thiol protease aleurain mRNA, complete cds
AT1G06570	2.458676703	0	Arabidopsis thaliana 4-hydroxyphenylpyruvate dioxygenase mRNA, complete cds
AT1G68530	1.087615713	0	Arabidopsis thaliana chromosome 1 sequence
AT1G36160	2.315970919	0	Arabidopsis thaliana acetyl-CoA carboxylase 1 mRNA, complete cds
AT1G74950	2.602858529	0	Arabidopsis thaliana protein TIFY 10B mRNA, complete cds
AT1G22930	1.789760993	0	Arabidopsis thaliana T-complex protein 11 mRNA, complete cds
AT5G42050	1.374797713	0	Arabidopsis thaliana DCD (Development and Cell Death) domain protein mRNA, complete cds

AT1G62570	3.365340612	0	Arabidopsis thaliana flavin-containing monooxygenase FMO GS-OX4 mRNA, complete cds
AT5G47560	1.830421963	0	Arabidopsis thaliana tonoplast dicarboxylate transporter mRNA, complete cds
AT3G10420	1.994163775	0	Arabidopsis thaliana protein seedling plastid development 1 mRNA, complete cds
AT1G08200	1.403100266	0	Arabidopsis thaliana UDP-D-apiose/UDP-D-xylose synthase 2 mRNA, complete cds
AT4G15210	8.051172811	0	Arabidopsis thaliana beta-amylase 5 mRNA, complete cds
AT3G54500	1.257031451	0	Arabidopsis thaliana uncharacterized protein mRNA, complete cds
AT5G20190	2.137336603	0	Arabidopsis thaliana tetratricopeptide repeat domain-containing protein mRNA, complete cds
AT3G03470	2.60446412	0	Arabidopsis thaliana cytochrome P450, family 87, subfamily A, polypeptide 9 mRNA, complete cds
AT4G21910	2.572941842	0	Arabidopsis thaliana MATE efflux family protein mRNA, complete cds
AT5G06760	8.337102707	0	Arabidopsis thaliana late embryogenesis abundant protein 4-5 mRNA, complete cds
AT5G05410	4.641451228	0	Arabidopsis thaliana dehydration-responsive element-binding protein 2A mRNA, complete cds
AT4G21990	2.733647289	0	Arabidopsis thaliana 5'-adenylylsulfate reductase 3 mRNA, complete cds
AT5G48180	2.130000668	0	Arabidopsis thaliana nitrile specifier protein 5 mRNA, complete cds
AT1G17940	3.750713827	0	Arabidopsis thaliana Endosomal targeting BRO1-like domain-containing protein mRNA, complete cds
AT1G79520	3.253947411	0	Arabidopsis thaliana cation efflux family protein mRNA, complete cds
AT3G62700	2.608484456	0	Arabidopsis thaliana ABC transporter C family member 14 mRNA, complete cds
AT5G59310	8.452284744	0	Arabidopsis thaliana non-specific lipid-transfer protein 4 mRNA, complete cds

AT1G27760	2.30796845	0	Arabidopsis thaliana protein salt tolerance 32 mRNA, complete cds
AT4G23050	3.791595016	0	Arabidopsis thaliana PAS domain-containing protein tyrosine kinase family protein mRNA, complete cds
AT1G29395	4.243137292	0	Arabidopsis thaliana cold regulated 314 inner membrane 1 mRNA, complete cds
AT1G52690	10.12636579	0	Arabidopsis thaliana Late embryogenesis abundant protein (LEA) family protein mRNA, complete cds
AT1G80840	3.366452805	0	Arabidopsis thaliana putative WRKY transcription factor 40 mRNA, complete cds
AT1G52890	6.414135557	0	Arabidopsis thaliana NAC domain-containing protein 19 mRNA, complete cds
AT2G39030	7.131137878	0	Arabidopsis thaliana chromosome 2, complete sequence
AT5G14780	1.913191693	0	Arabidopsis thaliana formate dehydrogenase mRNA, complete cds
AT3G24170	1.42558696	0	Arabidopsis thaliana glutathione-disulfide reductase mRNA, complete cds
AT1G17380	4.725192015	0	Arabidopsis thaliana protein TIFY 11A mRNA, complete cds
AT3G17810	1.95332097	0	Arabidopsis thaliana putative dihydropyrimidine dehydrogenase mRNA, complete cds
AT4G05020	3.084250292	0	Arabidopsis thaliana NAD(P)H dehydrogenase B2 mRNA, complete cds
AT2G22430	1.29649343	0	Arabidopsis thaliana homeobox-leucine zipper protein ATHB-6 mRNA, complete cds
AT4G30470	2.164648351	0	Arabidopsis thaliana cinnamoyl-CoA reductase like protein mRNA, complete cds
AT5G64170	2.739497911	0	Arabidopsis thaliana dentin sialophosphoprotein-like protein mRNA, complete cds
AT4G37980	2.211601199	0	Arabidopsis thaliana cinnamyl alcohol dehydrogenase 7 mRNA, complete cds
AT5G57050	3.849239969	0	Arabidopsis thaliana protein phosphatase 2C 77 mRNA, complete cds

AT1G05100	6.091351951	0	Arabidopsis thaliana chromosome 1 sequence
AT1G44350	3.699587518	0	Arabidopsis thaliana IAA-amino acid hydrolase ILR1-like 6 mRNA, complete cds
AT3G53180	2.050194626	0	Arabidopsis thaliana nodulin/glutamine synthase-like protein mRNA, complete cds
AT3G04240	1.784999119	0	Arabidopsis thaliana putative UDP-N-acetylglucosamine--peptide N-acetylglucosaminyltransferase SEC mRNA, complete cds
AT5G11110	4.069873156	0	Arabidopsis thaliana sucrose phosphate synthase 2F mRNA, complete cds
AT4G37390	4.003320026	0	Arabidopsis thaliana indole-3-acetic acid-amido synthetase GH3.2 mRNA, complete cds
AT4G29780	2.56944442	0	Arabidopsis thaliana chromosome 4 sequence
AT1G72450	2.249790143	0	Arabidopsis thaliana jasmonate-zim-domain protein 6 mRNA, complete cds
AT1G06430	1.473579199	0	Arabidopsis thaliana ATP-dependent zinc metalloprotease FTSH 8 mRNA, complete cds
AT3G26290	5.777244418	0	Arabidopsis thaliana cytochrome P450 71B26 mRNA, complete cds
AT2G23170	4.380532509	0	Arabidopsis thaliana indole-3-acetic acid-amido synthetase GH3.3 mRNA, complete cds
AT4G11570	1.513510684	0	Arabidopsis thaliana chromosome 4 sequence
AT1G61340	4.727372301	0	Arabidopsis thaliana F-box stress induced 1 mRNA, complete cds
AT1G77450	3.973500473	0	Arabidopsis thaliana NAC domain containing protein 32 mRNA, complete cds
AT1G69480	7.383170163	0	Arabidopsis thaliana EXS (ERD1/XPR1/SYG1) family protein mRNA, complete cds
AT3G21670	1.514146005	0	Arabidopsis thaliana nitrate transporter 1.3 mRNA, complete cds
AT4G28080	1.457456021	0	Arabidopsis thaliana tetratricopeptide repeat domain protein mRNA, complete

			cds
AT1G56580	1.80507638	0	Arabidopsis thaliana chromosome 1 sequence
AT1G07430	6.300884781	0	Arabidopsis thaliana protein phosphatase 2C 3 mRNA, complete cds
AT5G40390	2.351381668	0	Arabidopsis thaliana putative galactinol--sucrose galactosyltransferase 5 mRNA, complete cds
AT5G01600	3.044361346	0	Arabidopsis thaliana ferretin 1 mRNA, complete cds
AT1G72520	3.227461613	0	Arabidopsis thaliana lipoxygenase 4 mRNA, complete cds
AT2G43570	4.241690784	0	Arabidopsis thaliana putative chitinase mRNA, complete cds
AT2G42530	6.489320654	0	Arabidopsis thaliana cold-regulated protein 15b mRNA, complete cds
AT1G52030	4.543000957	0	Arabidopsis thaliana myrosinase-binding protein 2 mRNA, complete cds
AT2G41870	3.146513407	0	Arabidopsis thaliana remorin-like protein mRNA, complete cds
AT3G51860	3.710660675	0	Arabidopsis thaliana vacuolar cation/proton exchanger 3 mRNA, complete cds
AT4G15490	3.822529534	0	Arabidopsis thaliana chromosome 4 sequence
AT4G27560	3.105894293	0	Arabidopsis thaliana chromosome 4 sequence
AT5G01820	2.136335286	0	Arabidopsis thaliana chromosome 5 sequence
AT2G40000	1.413498483	0	Arabidopsis thaliana chromosome 2, complete sequence
AT4G04610	1.684018373	0	Arabidopsis thaliana 5'-adenylylsulfate reductase 1 mRNA, complete cds
AT5G20900	1.818955852	0	Arabidopsis thaliana protein TIFY 3B mRNA, complete cds
AT5G60790	1.731811367	0	Arabidopsis thaliana ABC transporter F family member 1 mRNA, complete cds
AT1G64660	3.75563815	0	Arabidopsis thaliana methionine gamma-lyase mRNA, complete cds
AT3G07700	1.96178956	0	Arabidopsis thaliana ABC1 kinase mRNA, complete cds
AT5G07010	6.156683932	0	Arabidopsis thaliana chromosome 5 sequence
AT2G44840	5.218084476	0	Arabidopsis thaliana chromosome 2,

			complete sequence
AT3G49220	1.446882655	0	Arabidopsis thaliana putative pectinesterase/pectinesterase inhibitor 34 mRNA, complete cds
AT5G24420	2.855290764	0	Arabidopsis thaliana 6-phosphogluconolactonase 5 mRNA, complete cds
AT1G70320	2.196516264	0	Arabidopsis thaliana ubiquitin-protein ligase 2 mRNA, complete cds
AT4G30530	1.27567115	0	Arabidopsis thaliana gamma-glutamyl peptidase 1 mRNA, complete cds
AT5G15450	1.92455892	0	Arabidopsis thaliana chaperone protein ClpB3 mRNA, complete cds
AT1G04220	3.275150103	0	Arabidopsis thaliana 3-ketoacyl-CoA synthase 2 mRNA, complete cds
AT2G46370	1.74758812	0	Arabidopsis thaliana jasmonic acid-amido synthetase JAR1 mRNA, complete cds
AT3G44880	1.973136989	0	Arabidopsis thaliana pheophorbide A oxygenase mRNA, complete cds
AT1G21400	3.868915501	0	Arabidopsis thaliana thiamin diphosphate-binding fold protein mRNA, complete cds
AT5G52300	10.24583387	0	Arabidopsis thaliana protein LOW-TEMPERATURE-INDUCED 65 mRNA, complete cds
AT5G64260	1.775102338	0	Arabidopsis thaliana chromosome 5 sequence
AT1G52040	3.454201057	0	Arabidopsis thaliana myrosinase-binding protein 1 mRNA, complete cds
AT1G69490	3.689011828	0	Arabidopsis thaliana NAC transcription factor protein family mRNA, complete cds
AT2G35940	1.938633057	0	Arabidopsis thaliana BEL1-like homeodomain 1 mRNA, complete cds
AT3G17790	3.907224792	0	Arabidopsis thaliana purple acid phosphatase 17 mRNA, complete cds
AT5G15500	6.244753185	0	Arabidopsis thaliana chromosome 5 sequence
AT1G58520	1.838599645	0	Arabidopsis thaliana protein RXW8 mRNA, complete cds
AT5G53970	2.271917791	0	Arabidopsis thaliana tyrosine aminotransferase mRNA, complete cds
AT5G54940	1.033299716	0	Arabidopsis thaliana translation initiation

			factor SUI1 family protein mRNA, complete cds
AT1G01140	2.420792171	0	Arabidopsis thaliana CBL-interacting serine/threonine-protein kinase 9 mRNA, complete cds
AT5G55120	1.763164764	0	Arabidopsis thaliana GDP-L-galactose phosphorylase mRNA, complete cds
AT1G03080	2.761936573	0	Arabidopsis thaliana kinase interacting (KIP1-like) protein mRNA, complete cds
AT2G46790	5.016133532	0	Arabidopsis thaliana two-component response regulator-like APRR9 mRNA, complete cds
AT5G47640	1.89346437	0	Arabidopsis thaliana chromosome 5 sequence
AT3G23250	4.652639896	0	Arabidopsis thaliana myb domain protein 15 mRNA, complete cds
AT3G17000	2.067945012	0	Arabidopsis thaliana ubiquitin-conjugating enzyme E2 32 mRNA, complete cds
AT2G18700	2.072743188	0	Arabidopsis thaliana putative alpha,alpha-trehalose-phosphate synthase [UDP-forming] 11 mRNA, complete cds
AT5G13220	4.828849691	0	Arabidopsis thaliana protein TIFY 9 mRNA, complete cds
AT5G03190	2.371325745	0	Arabidopsis thaliana chromosome 5 sequence
AT1G36370	2.259777576	0	Arabidopsis thaliana putative serine hydroxymethyltransferase mRNA, complete cds
AT5G02020	3.56827363	0	Arabidopsis thaliana uncharacterized protein mRNA, complete cds
AT1G07720	1.884218724	0	Arabidopsis thaliana chromosome 1 sequence
AT4G39330	1.168915755	0	Arabidopsis thaliana putative cinnamyl alcohol dehydrogenase 9 mRNA, complete cds
AT3G48520	5.494696907	0	Arabidopsis thaliana chromosome 3, complete sequence
AT1G04120	1.771584295	0	Arabidopsis thaliana inositol hexakisphosphate transporter mRNA, complete cds
AT2G47780	6.706817382	0	Arabidopsis thaliana Rubber elongation factor protein mRNA, complete cds
AT1G09970	1.179797069	0	Arabidopsis thaliana leucine-rich

			receptor-like protein kinase mRNA, complete cds
AT3G25780	2.260561152	0	Arabidopsis thaliana allene oxide cyclase 3 mRNA, complete cds
AT5G54170	1.898497066	0	Arabidopsis thaliana lipid-binding START domain-containing protein mRNA, complete cds
AT3G51450	3.71531915	0	Arabidopsis thaliana strictosidine synthase family protein mRNA, complete cds
AT3G47960	1.717901986	0	Arabidopsis thaliana glucosinolate transporter 1 mRNA, complete cds
AT4G37760	1.495855038	0	Arabidopsis thaliana squalene epoxidase 3 mRNA, complete cds
AT3G48690	1.670069804	0	Arabidopsis thaliana chromosome 3, complete sequence
AT1G66760	3.535667349	0	Arabidopsis thaliana MATE efflux family protein mRNA, complete cds
AT5G50100	2.193531715	0	Arabidopsis thaliana putative thiol-disulfide oxidoreductase DCC mRNA, complete cds
AT2G28400	3.86410139	0	Arabidopsis thaliana chromosome 2, complete sequence
AT3G27250	5.795082529	0	Arabidopsis thaliana chromosome 3, complete sequence
AT3G47420	1.728316953	0	Arabidopsis thaliana putative glycerol-3-phosphate transporter 1 mRNA, complete cds
AT3G22370	2.646630427	0	Arabidopsis thaliana alternative oxidase 1A mRNA, complete cds
AT3G56275	6.273297893	0	Arabidopsis thaliana chromosome 3, complete sequence
AT5G17460	4.901410761	0	Arabidopsis thaliana uncharacterized protein mRNA, complete cds
AT5G57350	1.301101448	0	Arabidopsis thaliana H(+)-ATPase 3 mRNA, complete cds
AT3G28270	1.480890533	0	Arabidopsis thaliana chromosome 3, complete sequence
AT1G62540	3.326642639	0	Arabidopsis thaliana flavin-containing monooxygenase FMO GS-OX2 mRNA, complete cds
AT4G31780	1.56565572	0	Arabidopsis thaliana Monogalactosyldiacylglycerol synthase 1 mRNA, complete cds

AT1G01520	3.97354449	1.04E-307	Arabidopsis thaliana transcription factor ASG4 mRNA, complete cds
AT2G46510	2.459722647	4.97E-307	Arabidopsis thaliana chromosome 2, complete sequence
AT2G32150	1.73716984	4.69E-306	Arabidopsis thaliana haloacid dehalogenase-like hydrolase domain-containing protein mRNA, complete cds
AT3G47340	3.056262413	5.28E-304	Arabidopsis thaliana asparagine synthetase [glutamine-hydrolyzing] mRNA, complete cds
AT3G62260	2.822070372	1.12E-303	Arabidopsis thaliana putative protein phosphatase 2C 49 mRNA, complete cds
AT1G62180	1.207146434	3.13E-303	Arabidopsis thaliana 5'-adenylylsulfate reductase 2 mRNA, complete cds
AT5G09620	1.763609898	7.06E-301	Arabidopsis thaliana chromosome 5 sequence
AT5G15960	12.45015766	3.96E-300	Arabidopsis thaliana cold and ABA inducible protein kin1 mRNA, complete cds
AT2G29460	5.334414336	1.97E-299	Arabidopsis thaliana glutathione S-transferase tau 4 mRNA, complete cds
AT2G33590	1.945250773	4.17E-299	Arabidopsis thaliana cinnamoyl-CoA:NADP oxidoreductase-like 1 mRNA, complete cds
AT1G68620	4.265792216	1.69E-297	Arabidopsis thaliana chromosome 1 sequence
AT3G14810	2.605050777	2.95E-297	Arabidopsis thaliana mechanosensitive channel of small conductance-like 5 mRNA, complete cds
AT2G35060	2.087271674	3.96E-297	Arabidopsis thaliana potassium transporter 11 mRNA, complete cds
AT4G17330	1.950551747	2.81E-296	Arabidopsis thaliana G2484-1 protein mRNA, complete cds
AT4G31800	2.645964415	9.67E-295	Arabidopsis thaliana WRKY DNA-binding protein 18 mRNA, complete cds
AT2G30140	2.981381813	1.36E-293	Arabidopsis thaliana UDP-glycosyltransferase 87A2 mRNA, complete cds
AT4G16590	8.548380065	7.84E-293	Arabidopsis thaliana cellulose synthase-like A01 mRNA, complete cds
AT1G24580	4.451317484	1.04E-292	Arabidopsis thaliana chromosome 1

			sequence
AT2G36630	1.875838443	1.61E-292	Arabidopsis thaliana Sulfite exporter TauE/SafE family protein mRNA, complete cds
AT2G22470	1.797524514	2.67E-292	Arabidopsis thaliana chromosome 2, complete sequence
AT1G45249	2.301254837	2.08E-290	Arabidopsis thaliana abscisic acid responsive elements-binding factor 2 mRNA, complete cds
AT1G78680	1.71348027	6.12E-290	Arabidopsis thaliana gamma-glutamyl hydrolase 2 mRNA, complete cds
AT3G62010	1.927066252	2.02E-285	Arabidopsis thaliana uncharacterized protein mRNA, complete cds
AT1G10760	2.060356947	6.83E-283	Arabidopsis thaliana alpha-glucan water dikinase 1 mRNA, complete cds
AT2G29440	3.976557421	1.07E-282	Arabidopsis thaliana glutathione S-transferase tau 6 mRNA, complete cds
AT5G53460	1.100813047	1.59E-282	Arabidopsis thaliana glutamate synthase 1 [NADH] mRNA, complete cds
AT1G08920	2.406545921	8.29E-282	Arabidopsis thaliana sugar transporter ERD6-like 3 mRNA, complete cds
AT2G22010	2.10561918	3.85E-280	Arabidopsis thaliana E3 ubiquitin-protein ligase RKP mRNA, complete cds
AT1G01320	1.267557013	1.46E-277	Arabidopsis thaliana tetratricopeptide repeat-containing protein mRNA, complete cds
AT5G01520	3.058243334	7.42E-277	Arabidopsis thaliana C3HC4 type RING finger protein mRNA, complete cds
AT1G55860	2.490089755	2.31E-276	Arabidopsis thaliana ubiquitin-protein ligase 1 mRNA, complete cds
AT1G11840	1.053763841	8.20E-276	Arabidopsis thaliana glyoxalase I homolog GLX1 mRNA, complete cds
AT1G09070	1.18741348	9.63E-275	Arabidopsis thaliana chromosome 1 sequence
AT5G59450	2.484745396	1.12E-271	Arabidopsis thaliana chromosome 5 sequence
AT4G15530	1.578915155	4.76E-269	Arabidopsis thaliana pyruvate, phosphate dikinase 1 mRNA, complete cds
AT5G57610	2.276521458	6.15E-269	Arabidopsis thaliana octicosapeptide/Phox/Bem1p domain-containing protein kinase mRNA, complete cds
AT1G32970	6.744726331	1.72E-268	Arabidopsis thaliana Subtilisin-like serine endopeptidase family protein

			mRNA, complete cds
AT5G67480	2.958836628	1.23E-265	Arabidopsis thaliana BTB and TAZ domain protein 4 mRNA, complete cds
AT1G56650	5.50760058	4.84E-265	Arabidopsis thaliana transcription factor MYB75 mRNA, complete cds
AT3G09390	1.538936957	5.72E-262	Arabidopsis thaliana metallothionein 2A mRNA, complete cds
AT3G55970	4.304635483	2.08E-260	Arabidopsis thaliana jasmonate-regulated protein JRG21 mRNA, complete cds
AT3G22380	1.288457563	6.61E-259	Arabidopsis thaliana clock regulator protein TIME FOR COFFEE mRNA, complete cds
AT1G48090	2.240165043	2.27E-258	Arabidopsis thaliana calcium-dependent lipid-binding family protein mRNA, complete cds
AT4G17490	2.562662408	3.93E-258	Arabidopsis thaliana chromosome 4 sequence
AT2G38240	6.313672489	5.75E-258	Arabidopsis thaliana 2-oxoglutarate (2OG) and Fe(II)-dependent oxygenase-like protein mRNA, complete cds
AT3G02260	2.197325916	2.95E-257	Arabidopsis thaliana auxin transport protein BIG mRNA, complete cds
AT4G28300	1.538526287	3.41E-256	Arabidopsis thaliana uncharacterized protein mRNA, complete cds
AT1G73920	1.420866519	3.44E-256	Arabidopsis thaliana alpha/beta-hydrolase domain-containing protein mRNA, complete cds
AT1G13930	1.405076781	7.79E-255	Arabidopsis thaliana salt tolerance-related protein mRNA, complete cds
AT4G17840	1.372280303	2.57E-254	Arabidopsis thaliana uncharacterized protein mRNA, complete cds
AT1G21980	2.008716632	7.35E-254	Arabidopsis thaliana phosphatidylinositol-4-phosphate 5-kinase 1 mRNA, complete cds
AT1G09500	4.251223288	1.80E-253	Arabidopsis thaliana alcohol dehydrogenase-like protein mRNA, complete cds
AT4G21680	4.389602018	1.59E-251	Arabidopsis thaliana nitrate transporter 1.8 mRNA, complete cds
AT1G21790	3.136075235	5.96E-251	Arabidopsis thaliana TRAM, LAG1 and CLN8 (TLC) lipid-sensing domain containing protein mRNA, complete cds

AT5G65280	3.475164226	1.53E-250	Arabidopsis thaliana protein GCR2-like 1 mRNA, complete cds
AT5G57800	1.564277064	1.34E-249	Arabidopsis thaliana protein ECERIFERUM 3 mRNA, complete cds
AT5G54160	1.30363712	1.72E-249	Arabidopsis thaliana caffeic acid/5-hydroxyferulic acid O-methyltransferase mRNA, complete cds
AT5G64750	4.6264977	3.42E-248	Arabidopsis thaliana ethylene-responsive transcription factor ABR1 mRNA, complete cds
AT1G51140	2.203523954	1.83E-247	Arabidopsis thaliana transcription factor bHLH122 mRNA, complete cds
AT3G08730	1.150135073	1.04E-246	Arabidopsis thaliana protein-serine kinase 1 mRNA, complete cds
AT3G17860	2.035940934	6.40E-246	Arabidopsis thaliana jasmonate-zim-domain protein 3 mRNA, complete cds
AT3G63060	6.239601534	1.15E-245	Arabidopsis thaliana chromosome 3, complete sequence
AT5G08130	1.772732835	3.31E-245	Arabidopsis thaliana transcription factor BIM1 mRNA, complete cds
AT3G57520	1.113548362	5.16E-245	Arabidopsis thaliana putative galactinol--sucrose galactosyltransferase 2 mRNA, complete cds
AT2G28290	2.745070526	7.77E-245	Arabidopsis thaliana chromatin structure-remodeling complex protein SYD mRNA, complete cds
AT1G27730	2.072927235	1.59E-244	Arabidopsis thaliana chromosome 1 sequence
AT4G17550	2.496707523	3.36E-244	Arabidopsis thaliana glycerol-3-phosphate permease 4 mRNA, complete cds
AT3G02800	3.492379843	5.39E-244	Arabidopsis thaliana atypical dual-specificity phosphatase mRNA, complete cds
AT5G06980	2.567610868	6.94E-244	Arabidopsis thaliana uncharacterized protein mRNA, complete cds
AT3G22200	1.609019441	3.55E-243	Arabidopsis thaliana gamma-aminobutyrate transaminase POP2 mRNA, complete cds
AT5G66400	4.566380425	4.09E-242	Arabidopsis thaliana dehydrin Rab18 mRNA, complete cds
AT3G25250	5.081455915	1.53E-241	Arabidopsis thaliana AGC

			(cAMP-dependent, cGMP-dependent and protein kinase C) kinase family protein mRNA, complete cds
AT3G57020	1.925302727	2.79E-241	Arabidopsis thaliana calcium-dependent phosphotriesterase superfamily protein mRNA, complete cds
AT4G01070	1.185080976	3.21E-241	Arabidopsis thaliana chromosome 4 sequence
AT4G24000	4.455617953	3.96E-241	Arabidopsis thaliana cellulose synthase-like protein G2 mRNA, complete cds
AT5G03210	8.212966407	5.06E-241	Arabidopsis thaliana chromosome 5 sequence
AT2G26380	7.067916074	4.53E-240	Arabidopsis thaliana chromosome 2, complete sequence
AT5G07920	2.344805526	4.33E-239	Arabidopsis thaliana diacylglycerol kinase1 mRNA, complete cds
AT4G36040	1.556990245	9.03E-239	Arabidopsis thaliana chromosome 4 sequence
AT2G20340	2.557382691	1.15E-238	Arabidopsis thaliana tyrosine decarboxylase 1 mRNA, complete cds
AT5G64120	2.557382691	1.15E-238	Arabidopsis thaliana peroxidase 71 mRNA, complete cds
AT1G58340	4.04424088	9.43E-238	Arabidopsis thaliana MATE family protein ZRIZ1 mRNA, complete cds
AT1G26770	1.810968785	9.13E-237	Arabidopsis thaliana expansin A10 mRNA, complete cds
AT1G58360	1.538336489	1.27E-236	Arabidopsis thaliana amino acid permease 1 mRNA, complete cds
AT2G39050	3.093424888	2.30E-236	Arabidopsis thaliana Euonymus lectin S3 mRNA, complete cds
AT5G39580	3.920448743	9.07E-236	Arabidopsis thaliana peroxidase 62 mRNA, complete cds
AT3G23810	1.075295605	6.04E-230	Arabidopsis thaliana adenosylhomocysteinase 2 mRNA, complete cds
AT1G02820	4.550457511	7.93E-229	Arabidopsis thaliana late embryogenesis abundant 3-like protein mRNA, complete cds
AT1G24070	3.078971263	9.00E-229	Arabidopsis thaliana cellulose synthase-like A10 mRNA, complete cds
AT4G17470	4.384685646	5.86E-228	Arabidopsis thaliana putative palmitoyl-protein thioesterase mRNA, complete cds

AT3G51895	1.809659998	8.27E-228	Arabidopsis thaliana sulfate transporter 3;1 mRNA, complete cds
AT5G06530	1.258131496	1.17E-227	Arabidopsis thaliana ABC transporter G family member 22 mRNA, complete cds
AT1G80820	3.748596832	3.23E-227	Arabidopsis thaliana cinnamoyl-CoA reductase mRNA, complete cds
AT2G26530	2.164300611	1.96E-226	Arabidopsis thaliana chromosome 2, complete sequence
AT5G02940	1.665640866	2.04E-225	Arabidopsis thaliana uncharacterized protein mRNA, complete cds
AT5G65380	1.570890754	2.26E-225	Arabidopsis thaliana MATE efflux family protein mRNA, complete cds
AT5G59480	1.435330997	3.41E-224	Arabidopsis thaliana Haloacid dehalogenase-like hydrolase (HAD) superfamily protein mRNA, complete cds
AT2G46520	1.796608685	1.24E-222	Arabidopsis thaliana putative cellular apoptosis susceptibility protein / importin-alpha re-exporter mRNA, complete cds
AT1G63010	1.644382937	1.40E-222	Arabidopsis thaliana Major Facilitator Superfamily with SPX domain-containing protein mRNA, complete cds
AT5G52570	2.24976644	2.80E-222	Arabidopsis thaliana beta-carotene hydroxylase 2 mRNA, complete cds
AT1G16850	4.47404802	2.12E-220	Arabidopsis thaliana uncharacterized protein mRNA, complete cds
AT5G37300	2.957955862	2.89E-220	Arabidopsis thaliana o-acyltransferase WSD1 mRNA, complete cds
AT1G54130	1.711463669	2.67E-219	Arabidopsis thaliana RELA/SPOT homolog 3 mRNA, complete cds
AT1G53470	3.437865684	4.08E-218	Arabidopsis thaliana mechanosensitive channel of small conductance-like 4 mRNA, complete cds
AT1G59740	2.523461035	5.74E-218	Arabidopsis thaliana probable peptide/nitrate transporter mRNA, complete cds
AT5G42380	4.60931654	6.11E-218	Arabidopsis thaliana chromosome 5 sequence
AT3G19580	2.313725971	7.07E-217	Arabidopsis thaliana chromosome 3, complete sequence
AT2G46400	3.558617844	1.24E-216	Arabidopsis thaliana putative WRKY transcription factor 46 mRNA, complete

			cds
AT5G62090	2.116403802	4.82E-214	Arabidopsis thaliana protein SEUSS-like 2 mRNA, complete cds
AT4G17500	2.677406387	1.10E-213	Arabidopsis thaliana chromosome 4 sequence
AT4G02280	5.045548261	1.09E-212	Arabidopsis thaliana sucrose synthase 3 mRNA, complete cds
AT1G04770	3.430067314	1.38E-212	Arabidopsis thaliana tetratricopeptide repeat-containing protein mRNA, complete cds
AT1G61120	6.527751188	1.62E-212	Arabidopsis thaliana terpene synthase 04 mRNA, complete cds
AT1G76790	2.234481378	5.02E-212	Arabidopsis thaliana indole glucosinolate o-methyltransferase 5 mRNA, complete cds
AT3G08720	2.077410853	2.59E-211	Arabidopsis thaliana AT3G08720 mRNA, complete cds, clone: RAFL09-47-F23
AT5G54300	2.285840719	4.02E-209	Arabidopsis thaliana uncharacterized protein mRNA, complete cds
AT2G47770	10.41950092	1.21E-208	Arabidopsis thaliana chromosome 2, complete sequence
AT4G18010	2.091224733	1.07E-207	Arabidopsis thaliana Type I inositol-1,4,5-trisphosphate 5-phosphatase 2 mRNA, complete cds
AT4G34240	1.599895395	8.72E-206	Arabidopsis thaliana aldehyde dehydrogenase 3I1 mRNA, complete cds
AT4G17230	1.933505111	1.08E-205	Arabidopsis thaliana protein scarecrow-like 13 mRNA, complete cds
AT4G39210	2.67503857	9.05E-205	Arabidopsis thaliana glucose-1-phosphate adenylyltransferase large subunit 3 mRNA, complete cds
AT1G20510	1.710960595	1.06E-204	Arabidopsis thaliana OPC-8:0 CoA ligase1 mRNA, complete cds
AT4G34410	4.323849424	3.00E-204	Arabidopsis thaliana chromosome 4 sequence
AT1G05340	3.253701316	8.48E-204	Arabidopsis thaliana uncharacterized protein mRNA, complete cds
AT4G01080	3.280324407	4.08E-202	Arabidopsis thaliana protein TRICHOME BIREFRINGENCE-LIKE 26 mRNA, complete cds
AT2G38750	2.539845623	3.13E-201	Arabidopsis thaliana annexin D4 mRNA, complete cds
AT1G80160	4.357826186	4.98E-201	Arabidopsis thaliana GLYOXYLASE I 7

			mRNA, complete cds
AT5G43745	2.292783226	6.44E-199	Arabidopsis thaliana uncharacterized protein mRNA, complete cds
AT1G45145	2.176132371	9.02E-199	Arabidopsis thaliana thioredoxin H5 mRNA, complete cds
AT1G62510	3.812597648	3.56E-198	Arabidopsis thaliana chromosome 1 sequence
AT1G67360	1.751575369	3.51E-197	Arabidopsis thaliana REF/SRPP-like protein mRNA, complete cds
AT4G37370	2.566982348	1.04E-196	Arabidopsis thaliana cytochrome P450, family 81, subfamily D, polypeptide 8 mRNA, complete cds
AT5G04340	2.600542725	1.84E-196	Arabidopsis thaliana chromosome 5 sequence
AT4G22710	2.991286668	2.26E-195	Arabidopsis thaliana chromosome 4 sequence
AT2G03760	2.402727203	3.03E-195	Arabidopsis thaliana chromosome 2, complete sequence
AT2G40140	1.352976787	1.23E-194	Arabidopsis thaliana zinc finger CCCH domain-containing protein 29 mRNA, complete cds
AT5G13200	2.691960659	2.42E-194	Arabidopsis thaliana GRAM domain family protein mRNA, complete cds
AT4G18700	1.211155106	4.29E-194	Arabidopsis thaliana chromosome 4 sequence
AT5G47040	1.223803217	4.83E-194	Arabidopsis thaliana lon protease-like 2 mRNA, complete cds
AT1G17550	1.541734828	4.22E-193	Arabidopsis thaliana protein phosphatase 2C 7 mRNA, complete cds
AT4G05100	4.61843793	2.62E-192	Arabidopsis thaliana myb domain protein 74 mRNA, complete cds
AT2G37770	4.377744692	9.14E-191	Arabidopsis thaliana aldo-keto reductase family 4 member C9 mRNA, complete cds
AT2G35930	2.267940683	4.02E-190	Arabidopsis thaliana chromosome 2, complete sequence
AT1G77680	1.738255936	6.20E-190	Arabidopsis thaliana ribonuclease II/R family protein mRNA, complete cds
AT2G34930	2.867905347	1.86E-189	Arabidopsis thaliana chromosome 2, complete sequence
AT3G17520	10.23784108	2.46E-189	Arabidopsis thaliana chromosome 3, complete sequence
AT4G33905	7.432703979	9.55E-189	Arabidopsis thaliana Peroxisomal membrane protein Mpv17/PMP22

			mRNA, complete cds
AT1G69870	2.056748697	4.48E-187	Arabidopsis thaliana nitrate transporter 1.7 mRNA, complete cds
AT5G17760	2.545080856	2.52E-186	Arabidopsis thaliana chromosome 5 sequence
AT2G47800	1.593786727	1.19E-185	Arabidopsis thaliana ABC transporter C family member 4 mRNA, complete cds
AT2G29350	2.475475886	1.32E-185	Arabidopsis thaliana senescence-associated protein 13 mRNA, complete cds
AT4G22820	1.882715235	4.55E-184	Arabidopsis thaliana chromosome 4 sequence
AT5G27150	1.173260834	1.57E-183	Arabidopsis thaliana mRNA for Na+/H+ exchanger, complete cds, clone: RAFL07-14-P04
AT5G15410	1.438264274	3.97E-183	Arabidopsis thaliana cyclic nucleotide-gated ion channel 2 mRNA, complete cds
AT5G65990	1.920821781	1.38E-182	Arabidopsis thaliana transmembrane amino acid transporter family protein mRNA, complete cds
AT4G32770	2.365376363	1.18E-181	Arabidopsis thaliana tocopherol cyclase mRNA, complete cds
AT2G27150	2.589090835	2.61E-181	Arabidopsis thaliana abscisic-aldehyde oxidase mRNA, complete cds
AT5G61810	2.949764723	3.86E-181	Arabidopsis thaliana ATP-Mg/Pi transporter mRNA, complete cds
AT4G27260	2.22827979	4.00E-181	Arabidopsis thaliana indole-3-acetic acid-amido synthetase GH3.5 mRNA, complete cds
AT1G13990	2.399065505	3.87E-180	Arabidopsis thaliana uncharacterized protein mRNA, complete cds
AT5G52050	2.991508422	1.51E-178	Arabidopsis thaliana chromosome 5 sequence
AT4G25640	1.159659567	1.63E-178	Arabidopsis thaliana detoxifying efflux carrier 35 mRNA, complete cds
AT2G37760	2.208101274	2.75E-178	Arabidopsis thaliana aldo-keto reductase family 4 member C8 mRNA, complete cds
AT2G25625	5.249140976	8.39E-177	Arabidopsis thaliana uncharacterized protein mRNA, complete cds
AT1G01650	1.508102759	7.79E-176	Arabidopsis thaliana signal peptide peptidase-like 4 mRNA, complete cds
AT3G48460	1.765837992	1.91E-174	Arabidopsis thaliana GDSL

			esterase/lipase mRNA, complete cds
AT1G60270	2.288475094	4.45E-174	Arabidopsis thaliana beta glucosidase 6 mRNA, complete cds
AT4G30490	1.414054723	6.08E-174	Arabidopsis thaliana AFG1-like ATPase family protein mRNA, complete cds
AT2G46020	1.872632654	6.63E-174	Arabidopsis thaliana ATP-dependent helicase BRAHMA mRNA, complete cds
AT3G57260	3.43626713	7.48E-174	Arabidopsis thaliana beta 1,3-glucanase 2 mRNA, complete cds
AT5G44050	3.45736186	3.60E-173	Arabidopsis thaliana MATE efflux family protein mRNA, complete cds
AT1G02080	1.453742926	4.12E-173	Arabidopsis thaliana CCR4-NOT transcription complex subunit 1 domain protein mRNA, complete cds
AT3G15790	1.998620014	4.75E-172	Arabidopsis thaliana methyl-CPG-binding domain-containing protein 11 mRNA, complete cds
AT5G60890	1.529111543	1.35E-171	Arabidopsis thaliana myb domain protein 34 mRNA, complete cds
AT1G65890	4.960431648	1.73E-171	Arabidopsis thaliana acyl activating enzyme 12 mRNA, complete cds
AT3G22840	4.05654726	3.50E-171	Arabidopsis thaliana chlorophyll A-B binding, early light-inducible protein mRNA, complete cds
AT1G26730	3.147464799	2.27E-170	Arabidopsis thaliana phosphate transporter PHO1-7 mRNA, complete cds
AT1G01480	4.701294862	3.69E-170	Arabidopsis thaliana 1-aminocyclopropane-1-carboxylate synthase 2 mRNA, complete cds
AT2G39350	2.615482553	4.06E-170	Arabidopsis thaliana chromosome 2, complete sequence
AT5G06870	2.134499184	5.96E-170	Arabidopsis thaliana polygalacturonase inhibitor 2 mRNA, complete cds
AT1G53580	1.648916346	3.49E-169	Arabidopsis thaliana persulfide dioxygenase ETHE1-like protein mRNA, complete cds
AT2G34070	1.595117718	7.56E-168	Arabidopsis thaliana trichome birefringence-like 37 protein mRNA, complete cds
AT5G17860	5.106501473	2.50E-167	Arabidopsis thaliana chromosome 5 sequence
AT5G66760	1.16886972	1.44E-165	Arabidopsis thaliana succinate dehydrogenase [ubiquinone] flavoprotein

			subunit 1 mRNA, complete cds
AT1G14540	2.657277672	2.20E-164	Arabidopsis thaliana peroxidase 4 mRNA, complete cds
AT5G14640	1.397354024	4.77E-164	Arabidopsis thaliana Shaggy-related protein kinase 13 mRNA, complete cds
AT4G15150	3.910084844	1.32E-163	Arabidopsis thaliana glycine-rich protein mRNA, complete cds
AT4G14680	1.783328707	3.62E-163	Arabidopsis thaliana ATP sulfurylase mRNA, complete cds
AT3G02140	1.856819414	1.18E-161	Arabidopsis thaliana chromosome 3, complete sequence
AT2G18210	4.088970542	1.19E-161	Arabidopsis thaliana uncharacterized protein mRNA, complete cds
AT5G61960	1.191389514	3.11E-161	Arabidopsis thaliana MEI2-like protein 1 mRNA, complete cds
AT4G35560	2.371048859	3.58E-161	Arabidopsis thaliana DUO1-activated WD40 1 mRNA, complete cds
AT2G35630	1.569799348	5.27E-161	Arabidopsis thaliana protein MICROTUBULE ORGANIZATION 1 mRNA, complete cds
AT2G37870	6.641205363	1.16E-160	Arabidopsis thaliana protease inhibitor/seed storage/lipid transfer protein (LTP) family protein mRNA, complete cds
AT5G14700	2.645461104	1.75E-160	Arabidopsis thaliana Rossmann-fold NAD(P)-binding domain-containing protein mRNA, complete cds
AT3G06490	6.878438276	2.19E-160	Arabidopsis thaliana putative transcription factor MYB108 mRNA, complete cds
AT3G46450	1.746189375	2.70E-160	Arabidopsis thaliana SEC14 cytosolic factor family protein / phosphoglyceride transfer family protein mRNA, complete cds
AT5G11650	1.673787131	8.49E-160	Arabidopsis thaliana alpha/beta fold hydrolase family protein mRNA, complete cds
AT5G47220	2.800435764	1.39E-159	Arabidopsis thaliana chromosome 5 sequence
AT1G08650	1.246201698	1.67E-159	Arabidopsis thaliana phosphoenolpyruvate carboxylase kinase 1 mRNA, complete cds
AT1G75170	2.271780704	2.38E-158	Arabidopsis thaliana Sec14p-like phosphatidylinositol transfer family

			protein mRNA, complete cds
AT1G11960	1.956014311	3.15E-158	Arabidopsis thaliana putative ERD4 protein mRNA, complete cds
AT5G13550	1.633485392	3.48E-158	Arabidopsis thaliana sulfate transporter 4.1 mRNA, complete cds
AT2G25460	3.21380251	2.36E-157	Arabidopsis thaliana uncharacterized protein mRNA, complete cds
AT2G43530	2.905823498	1.41E-156	Arabidopsis thaliana defensin-like protein 194 mRNA, complete cds
AT5G43650	7.962422946	1.65E-156	Arabidopsis thaliana transcription factor bHLH92 mRNA, complete cds
AT1G58200	1.588546252	1.97E-156	Arabidopsis thaliana MSCS-like 3 mRNA, complete cds
AT3G11420	1.620860592	4.67E-156	Arabidopsis thaliana uncharacterized protein mRNA, complete cds
AT3G43270	2.095115476	1.49E-155	Arabidopsis thaliana probable pectinesterase/pectinesterase inhibitor 32 mRNA, complete cds
AT4G29190	2.636091794	2.73E-155	Arabidopsis thaliana chromosome 4 sequence
AT4G38600	1.188232024	2.09E-154	Arabidopsis thaliana HECT ubiquitin protein ligase family protein mRNA, complete cds
AT5G64870	4.086889057	2.72E-154	Arabidopsis thaliana SPFH/Band 7/PHB domain-containing membrane-associated protein family mRNA, complete cds
AT4G15440	2.147132211	3.16E-154	Arabidopsis thaliana hydroperoxide lyase 1 mRNA, complete cds
AT3G23030	1.046315448	3.60E-154	Arabidopsis thaliana auxin-responsive protein IAA2 mRNA, complete cds
AT2G19810	2.339556271	5.04E-154	Arabidopsis thaliana chromosome 2, complete sequence
AT2G02710	1.611085264	3.19E-153	Arabidopsis thaliana PAS/LOV protein B mRNA, complete cds
AT3G08860	5.117292869	8.81E-153	Arabidopsis thaliana PYRIMIDINE 4 mRNA, complete cds
AT5G63130	4.247605884	1.17E-151	Arabidopsis thaliana octicosapeptide/Phox/Bem1p domain-containing protein mRNA, complete cds
AT1G52565	2.401485248	1.18E-150	Arabidopsis thaliana uncharacterized protein mRNA, complete cds
AT5G28510	9.81207717	1.76E-150	Arabidopsis thaliana beta glucosidase 24 mRNA, complete cds

AT1G21410	2.132729134	1.74E-149	Arabidopsis thaliana F-box protein SKP2A mRNA, complete cds
AT2G47600	1.791502639	1.91E-149	Arabidopsis thaliana magnesium/proton exchanger mRNA, complete cds
AT3G62590	3.894362791	2.09E-149	Arabidopsis thaliana alpha/beta-Hydrolases superfamily protein mRNA, complete cds
AT1G69610	3.169179611	3.33E-149	Arabidopsis thaliana uncharacterized protein mRNA, complete cds
AT2G34660	1.400451849	4.65E-148	Arabidopsis thaliana ABC transporter C family member 2 mRNA, complete cds
AT1G61210	2.101637878	1.90E-147	Arabidopsis thaliana protein DWD hypersensitive to ABA 3 mRNA, complete cds
AT3G17690	3.493529859	2.23E-147	Arabidopsis thaliana cyclic nucleotide gated channel 19 mRNA, complete cds
AT4G24510	1.711513087	8.40E-147	Arabidopsis thaliana fatty acid elongation machinery component ECERIFERUM2 mRNA, complete cds
AT1G69295	1.405723826	4.03E-146	Arabidopsis thaliana plasmodesmata callose-binding protein 4 mRNA, complete cds
AT3G11480	9.756354695	5.42E-146	Arabidopsis thaliana SABATH methyltransferase BSMT1 mRNA, complete cds
AT5G43150	2.006454169	1.25E-145	Arabidopsis thaliana uncharacterized protein mRNA, complete cds
AT1G15290	1.13541369	3.07E-144	Arabidopsis thaliana tetratricopeptide repeat-containing protein mRNA, complete cds
AT5G13170	7.807099494	9.30E-143	Arabidopsis thaliana senescence-associated protein 29 mRNA, complete cds
AT1G09932	2.56984798	1.01E-142	Arabidopsis thaliana phosphoglycerate mutase family protein mRNA, complete cds
AT4G27830	1.96580962	1.18E-142	Arabidopsis thaliana beta glucosidase 10 mRNA, complete cds
AT5G19440	1.449734038	4.28E-142	Arabidopsis thaliana alcohol dehydrogenase-like protein mRNA, complete cds
AT5G02810	3.108044187	4.53E-142	Arabidopsis thaliana pseudo-response regulator 7 mRNA, complete cds
AT1G71080	1.617498768	4.87E-142	Arabidopsis thaliana RNA polymerase II

			transcription elongation factor mRNA, complete cds
AT3G27260	1.601799465	4.88E-142	Arabidopsis thaliana global transcription factor group E8 mRNA, complete cds
AT2G16586	1.65524572	7.93E-142	Arabidopsis thaliana chromosome 2, complete sequence
AT4G18280	3.448776458	1.70E-141	Arabidopsis thaliana chromosome 4 sequence
AT1G80300	1.187478655	2.95E-141	Arabidopsis thaliana ADP,ATP carrier protein 1 mRNA, complete cds
AT4G33240	1.715237048	4.86E-141	Arabidopsis thaliana 1-phosphatidylinositol-3-phosphate 5-kinase FAB1A mRNA, complete cds
AT2G42790	1.572188551	2.35E-140	Arabidopsis thaliana citrate synthase 3 mRNA, complete cds
AT1G80110	4.328636614	2.99E-140	Arabidopsis thaliana phloem protein 2-B11 mRNA, complete cds
AT3G20300	2.715980104	4.11E-140	Arabidopsis thaliana uncharacterized protein mRNA, complete cds
AT1G73080	1.602952828	5.43E-140	Arabidopsis thaliana leucine-rich repeat receptor-like protein kinase PEPR1 mRNA, complete cds
AT4G12000	2.153309428	7.72E-140	Arabidopsis thaliana SNARE associated Golgi family protein mRNA, complete cds
AT1G74930	3.202366274	1.23E-139	Arabidopsis thaliana chromosome 1 sequence
AT5G63450	5.225457351	1.86E-139	Arabidopsis thaliana chromosome 5 sequence
AT3G12145	1.873008961	4.06E-139	Arabidopsis thaliana leucine-rich repeat protein FLOR1 mRNA, complete cds
AT4G37990	2.751730873	1.83E-138	Arabidopsis thaliana cinnamyl alcohol dehydrogenase 8 mRNA, complete cds
AT2G29650	1.365841574	4.85E-138	Arabidopsis thaliana phosphate transporter 4;1 mRNA, complete cds
AT2G17500	2.319470983	5.03E-138	Arabidopsis thaliana auxin efflux carrier family protein mRNA, complete cds
AT5G26340	2.444755532	1.39E-137	Arabidopsis thaliana sugar transport protein 13 mRNA, complete cds
AT1G70580	1.313051525	3.41E-137	Arabidopsis thaliana glutamate--glyoxylate aminotransferase 2 mRNA, complete cds
AT4G29950	1.213283213	3.79E-137	Arabidopsis thaliana RabGAP/TBC domain-containing protein mRNA,

			complete cds
AT3G12320	1.486013903	5.48E-137	Arabidopsis thaliana uncharacterized protein mRNA, complete cds
AT5G02230	1.356071593	1.48E-136	Arabidopsis thaliana haloacid dehalogenase-like hydrolase superfamily protein mRNA, complete cds
AT3G55430	1.290818388	3.29E-136	Arabidopsis thaliana O-Glycosyl hydrolases family 17 protein mRNA, complete cds
AT3G51000	1.740558539	3.51E-136	Arabidopsis thaliana alpha/beta-Hydrolases superfamily protein mRNA, complete cds
AT5G27520	2.167838691	3.09E-135	Arabidopsis thaliana peroxisomal adenine nucleotide carrier 2 mRNA, complete cds
AT5G20490	1.395355389	3.30E-135	Arabidopsis thaliana Myosin XI K mRNA, complete cds
AT1G10585	4.880967102	4.83E-135	Arabidopsis thaliana basic helix-loop-helix domain-containing protein mRNA, complete cds
AT1G77800	1.5328851	4.92E-135	Arabidopsis thaliana PHD finger-containing protein mRNA, complete cds
AT5G48850	5.55069818	3.32E-132	Arabidopsis thaliana protein SULPHUR DEFICIENCY-INDUCED 1 mRNA, complete cds
AT3G61400	7.674904881	6.11E-132	Arabidopsis thaliana 1-aminocyclopropane-1-carboxylate oxidase-like 8 mRNA, complete cds
AT3G15210	1.745249587	2.19E-131	Arabidopsis thaliana chromosome 3, complete sequence
AT3G57010	2.515998881	2.81E-131	Arabidopsis thaliana calcium-dependent phosphotriesterase superfamily protein mRNA, complete cds
AT5G47370	1.463902596	2.88E-131	Arabidopsis thaliana homeobox-leucine zipper protein HAT2 mRNA, complete cds
AT2G26570	1.732628472	3.23E-131	Arabidopsis thaliana coiled-coil protein WEB1 mRNA, complete cds
AT5G45630	5.944401538	2.26E-130	Arabidopsis thaliana chromosome 5 sequence
AT5G16210	1.716637493	4.57E-130	Arabidopsis thaliana HEAT repeat-containing protein mRNA, complete cds

AT3G13330	1.626904023	8.90E-130	Arabidopsis thaliana proteasome activating protein 200 mRNA, complete cds
AT5G20070	1.311614787	5.07E-129	Arabidopsis thaliana nudix hydrolase 19 mRNA, complete cds
AT4G37180	2.232248976	1.11E-128	Arabidopsis thaliana myb family transcription factor mRNA, complete cds
AT2G45570	3.767083815	1.37E-128	Arabidopsis thaliana cytochrome P450 76C2 mRNA, complete cds
AT3G53230	2.393471565	2.59E-128	Arabidopsis thaliana cell division control protein 48-B mRNA, complete cds
AT5G19875	2.093969783	2.70E-128	Arabidopsis thaliana chromosome 5 sequence
AT1G54575	4.47439156	3.07E-128	Arabidopsis thaliana chromosome 1 sequence
AT3G56260	3.279889991	6.79E-128	Arabidopsis thaliana uncharacterized protein mRNA, complete cds
AT5G22630	1.439783397	9.29E-128	Arabidopsis thaliana chromosome 5 sequence
AT4G35790	1.216064652	1.08E-127	Arabidopsis thaliana phospholipase D delta mRNA, complete cds
AT1G03060	1.883660579	1.61E-127	Arabidopsis thaliana WD/BEACH domain protein SPIRRIG mRNA, complete cds
AT1G22770	3.829290626	3.99E-127	Arabidopsis thaliana protein GIGANTEA mRNA, complete cds
AT3G05630	4.060498603	1.03E-126	Arabidopsis thaliana phospholipase D P2 mRNA, complete cds
AT3G56200	2.024468543	1.35E-126	Arabidopsis thaliana putative amino acid transporter mRNA, complete cds
AT1G68600	2.687654669	1.71E-126	Arabidopsis thaliana Aluminum activated malate transporter family protein mRNA, complete cds
AT4G35800	1.533715902	9.59E-126	Arabidopsis thaliana DNA-directed RNA polymerase II subunit RPB1 mRNA, complete cds
AT5G13800	1.375884333	1.82E-125	Arabidopsis thaliana pheophytinase mRNA, complete cds
AT5G10300	2.465408144	1.92E-125	Arabidopsis thaliana methyl esterase 5 mRNA, complete cds
AT3G57540	3.394276416	5.50E-125	Arabidopsis thaliana Remorin family protein mRNA, complete cds
AT5G65470	1.539681784	1.11E-124	Arabidopsis thaliana O-fucosyltransferase family protein

			mRNA, complete cds
AT1G76810	1.218517567	1.40E-124	Arabidopsis thaliana eukaryotic translation initiation factor 2 (eIF-2) family protein mRNA, complete cds
AT2G39450	1.293559305	1.66E-124	Arabidopsis thaliana manganese transporter MTP11 mRNA, complete cds
AT5G44800	2.016755995	1.78E-124	Arabidopsis thaliana chromatin remodeling 4 protein mRNA, complete cds
AT4G05010	2.611629296	2.67E-124	Arabidopsis thaliana F-box protein mRNA, complete cds
AT5G43850	1.227894304	4.99E-124	Arabidopsis thaliana acireductone dioxygenase 4 mRNA, complete cds
AT5G44110	2.75520651	9.38E-124	Arabidopsis thaliana ABC transporter I family member 21 mRNA, complete cds
AT4G38810	1.162837371	4.71E-123	Arabidopsis thaliana SnRK2-interacting calcium sensor SCS mRNA, complete cds
AT4G00440	1.593961751	4.92E-123	Arabidopsis thaliana uncharacterized protein mRNA, complete cds
AT4G16680	2.626996278	7.10E-123	Arabidopsis thaliana putative RNA helicase mRNA, complete cds
AT3G03341	9.440010956	8.30E-123	Arabidopsis thaliana chromosome 3, complete sequence
AT5G05730	1.31985378	2.37E-122	Arabidopsis thaliana anthranilate synthase component I-1 mRNA, complete cds
AT1G76600	2.451868544	4.52E-122	Arabidopsis thaliana chromosome 1 sequence
AT4G37790	1.07427998	6.32E-122	Arabidopsis thaliana homeobox-leucine zipper protein HAT22 mRNA, complete cds
AT4G01870	2.732164542	9.19E-122	Arabidopsis thaliana chromosome 4 sequence
AT4G25100	1.11031304	9.62E-122	Arabidopsis thaliana superoxide dismutase [Fe] mRNA, complete cds
AT5G50360	6.440725338	2.52E-121	Arabidopsis thaliana chromosome 5 sequence
AT2G28470	1.210814896	1.21E-120	Arabidopsis thaliana beta-galactosidase 8 mRNA, complete cds
AT1G18870	4.259029629	2.12E-120	Arabidopsis thaliana Isochorismate synthase 2 mRNA, complete cds
AT1G32750	1.999582407	4.86E-120	Arabidopsis thaliana histone acetyltransferase of the CBP family 13

			mRNA, complete cds
AT5G66640	3.301387166	6.10E-120	Arabidopsis thaliana protein DA1-related 3 mRNA, complete cds
AT2G36850	1.155786718	1.49E-119	Arabidopsis thaliana glucan synthase-like 8 mRNA, complete cds
AT4G09500	3.613580366	1.69E-119	Arabidopsis thaliana chromosome 4 sequence
AT5G43450	2.361050087	5.45E-119	Arabidopsis thaliana 1-aminocyclopropane-1-carboxylate oxidase-like protein mRNA, complete cds
AT1G70290	1.088277873	9.73E-119	Arabidopsis thaliana putative alpha,alpha-trehalose-phosphate synthase [UDP-forming] 8 mRNA, complete cds
AT4G15180	2.095179029	1.09E-118	Arabidopsis thaliana putative histone-lysine N-methyltransferase ATXR3 mRNA, complete cds
AT2G16630	2.209523997	1.17E-118	Arabidopsis thaliana pollen Ole e 1 allergen and extensin family protein mRNA, complete cds
AT1G18460	1.199775591	2.20E-118	Arabidopsis thaliana alpha/beta-Hydrolases superfamily protein mRNA, complete cds
AT5G10625	2.596521334	5.42E-118	Arabidopsis thaliana chromosome 5 sequence
AT1G75370	1.573744718	7.70E-118	Arabidopsis thaliana Sec14p-like phosphatidylinositol transfer family protein mRNA, complete cds
AT3G14270	1.734820078	1.61E-117	Arabidopsis thaliana phosphatidylinositol-3P 5-kinase-like mRNA, complete cds
AT1G79280	1.777595787	5.71E-117	Arabidopsis thaliana nuclear pore anchor mRNA, complete cds
AT1G56250	4.510670992	6.32E-117	Arabidopsis thaliana F-box protein PP2-B14 mRNA, complete cds
AT2G32240	1.127841177	1.56E-116	Arabidopsis thaliana uncharacterized protein mRNA, complete cds
AT1G32870	2.278525433	4.06E-116	Arabidopsis thaliana NAC domain protein 13 mRNA, complete cds
AT5G54585	2.908234553	3.06E-115	Arabidopsis thaliana uncharacterized protein mRNA, complete cds
AT4G12020	1.949163725	6.61E-115	Arabidopsis thaliana mitogen-activated protein kinase kinase kinase 11 mRNA, complete cds

AT1G50260	2.186098661	9.51E-115	Arabidopsis thaliana N-terminal-transmembrane-C2 domain type 5.1 protein mRNA, complete cds
AT3G46670	2.588318326	2.14E-114	Arabidopsis thaliana UDP-glucosyl transferase 76E11 mRNA, complete cds
AT1G78610	1.90979452	2.23E-114	Arabidopsis thaliana mechanosensitive channel of small conductance-like 6 mRNA, complete cds
AT5G23110	1.974775187	3.74E-114	Arabidopsis thaliana uncharacterized protein mRNA, complete cds
AT3G59220	3.727807963	7.04E-114	Arabidopsis thaliana pirin-1 mRNA, complete cds
AT1G72120	2.645940803	1.02E-113	Arabidopsis thaliana putative peptide/nitrate transporter mRNA, complete cds
AT4G32920	1.837623491	2.51E-113	Arabidopsis thaliana glycine-rich protein mRNA, complete cds
AT5G24080	7.420587693	3.21E-113	Arabidopsis thaliana G-type lectin S-receptor-like serine/threonine protein kinase mRNA, complete cds
AT3G47500	1.389882769	5.36E-113	Arabidopsis thaliana cyclic DOF factor 3 mRNA, complete cds
AT3G47080	1.880592445	1.16E-112	Arabidopsis thaliana tetratricopeptide repeat domain-containing protein mRNA, complete cds
AT1G30320	1.894060508	1.21E-112	Arabidopsis thaliana Remorin family protein mRNA, complete cds
AT3G24460	1.956819343	1.33E-112	Arabidopsis thaliana Serinc-domain containing serine and sphingolipid biosynthesis protein mRNA, complete cds
AT3G07360	1.284892084	1.46E-112	Arabidopsis thaliana ARM domain-containing protein mRNA, complete cds
AT5G23820	1.06754099	1.66E-112	Arabidopsis thaliana MD-2-related lipid recognition domain-containing protein mRNA, complete cds
AT4G22920	1.834493384	1.69E-112	Arabidopsis thaliana protein NON-YELLOWING 1 mRNA, complete cds
AT5G04760	1.942454768	2.40E-112	Arabidopsis thaliana duplicated SANT DNA-binding domain-containing protein mRNA, complete cds
AT5G12010	1.751511655	2.52E-112	Arabidopsis thaliana chromosome 5

			sequence
AT3G58190	4.352976787	3.78E-112	Arabidopsis thaliana protein ASYMMETRIC LEAVES 2-LIKE 16 mRNA, complete cds
AT1G79000	1.67606749	4.82E-112	Arabidopsis thaliana histone acetyltransferase HAC1 mRNA, complete cds
AT5G52320	1.844785856	8.07E-112	Arabidopsis thaliana chromosome 5 sequence
AT2G41410	1.076599441	1.88E-111	Arabidopsis thaliana chromosome 2, complete sequence
AT3G22910	4.83766867	1.98E-111	Arabidopsis thaliana chromosome 3, complete sequence
AT2G36650	3.489766786	3.53E-111	Arabidopsis thaliana uncharacterized protein mRNA, complete cds
AT3G16940	1.357352139	1.82E-110	Arabidopsis thaliana calmodulin-binding transcription activator mRNA, complete cds
AT4G09030	1.545085174	3.03E-110	Arabidopsis thaliana chromosome 4 sequence
AT2G17930	1.694382945	1.35E-109	Arabidopsis thaliana phosphatidylinositol 3- and 4-kinase family protein with FAT domain mRNA, complete cds
AT4G15248	3.916265244	2.76E-109	Arabidopsis thaliana chromosome 4 sequence
AT4G17615	2.229524257	7.25E-109	Arabidopsis thaliana calcineurin B-like protein 1 mRNA, complete cds
AT2G27950	1.415295003	7.46E-109	Arabidopsis thaliana RING/U-box domain-containing protein mRNA, complete cds
AT4G36010	3.069083776	1.13E-108	Arabidopsis thaliana pathogenesis-related thaumatin family protein mRNA, complete cds
AT1G61610	4.587806311	3.51E-108	Arabidopsis thaliana putative G-type lectin S-receptor-like serine/threonine-protein kinase mRNA, complete cds
AT1G23710	1.786665499	7.82E-108	Arabidopsis thaliana chromosome 1 sequence
AT5G58160	2.561798012	8.17E-108	Arabidopsis thaliana formin-like protein 13 mRNA, complete cds
AT5G13820	2.382012449	2.79E-107	Arabidopsis thaliana Telomere repeat-binding protein 4 mRNA, complete cds

AT1G17870	2.61843793	7.17E-107	Arabidopsis thaliana S2P-like putative metalloprotease mRNA, complete cds
AT5G13750	2.024874693	1.16E-106	Arabidopsis thaliana zinc induced facilitator-like 1 protein mRNA, complete cds
AT5G35735	1.127899201	1.41E-106	Arabidopsis thaliana putative auxin-responsive protein mRNA, complete cds
AT5G60680	1.459330697	1.88E-106	Arabidopsis thaliana chromosome 5 sequence
AT4G20320	1.501789433	1.89E-106	Arabidopsis thaliana putative CTP synthase mRNA, complete cds
AT1G17745	1.426066288	2.92E-106	Arabidopsis thaliana D-3-phosphoglycerate dehydrogenase mRNA, complete cds
AT2G03240	1.951365822	1.30E-105	Arabidopsis thaliana EXS (ERD1/XPR1/SYG1) family protein mRNA, complete cds
AT1G80070	1.160581504	1.35E-105	Arabidopsis thaliana putative splicing factor Prp8 mRNA, complete cds
AT3G63070	1.761397839	3.87E-105	Arabidopsis thaliana Tudor/PWWP/MBT domain-containing protein mRNA, complete cds
AT1G78120	1.708312914	7.62E-105	Arabidopsis thaliana tetratricopeptide repeat-containing protein mRNA, complete cds
AT4G30100	1.372299228	2.11E-104	Arabidopsis thaliana P-loop containing nucleoside triphosphate hydrolases superfamily protein mRNA, complete cds
AT3G09940	1.073281657	7.37E-104	Arabidopsis thaliana monodehydroascorbate reductase (NADH) mRNA, complete cds
AT5G17380	1.272920469	1.04E-103	Arabidopsis thaliana 2-hydroxyacyl-CoA lyase mRNA, complete cds
AT3G28007	3.673838194	1.10E-103	Arabidopsis thaliana bidirectional sugar transporter SWEET4 mRNA, complete cds
AT5G19855	1.044402811	1.55E-103	Arabidopsis thaliana Chaperonin-like RbcX protein mRNA, complete cds
AT1G10480	3.411197734	1.64E-103	Arabidopsis thaliana chromosome 1 sequence
AT2G18790	1.17818603	1.66E-103	Arabidopsis thaliana phytochrome B mRNA, complete cds

AT1G64950	1.414473836	1.73E-103	Arabidopsis thaliana chromosome 1 sequence
AT1G68020	1.516148517	1.38E-102	Arabidopsis thaliana alpha,alpha-trehalose-phosphate synthase [UDP-forming] 6 mRNA, complete cds
AT4G29930	3.078195495	1.47E-102	Arabidopsis thaliana transcription factor bHLH27 mRNA, complete cds
AT1G56170	2.125200511	1.93E-102	Arabidopsis thaliana nuclear transcription factor Y subunit C-2 mRNA, complete cds
AT4G21390	2.632637063	2.57E-102	Arabidopsis thaliana G-type lectin S-receptor-like serine/threonine-protein kinase B120 mRNA, complete cds
AT1G55110	1.787344067	3.91E-102	Arabidopsis thaliana indeterminate(ID)-domain 7 protein mRNA, complete cds
AT2G02010	4.054846006	5.56E-102	Arabidopsis thaliana glutamate decarboxylase 4 mRNA, complete cds
AT4G37260	1.410261223	9.80E-102	Arabidopsis thaliana chromosome 4 sequence
AT1G10170	1.446047226	1.34E-101	Arabidopsis thaliana chromosome 1 sequence
AT3G14570	2.017773595	1.87E-101	Arabidopsis thaliana glucan synthase-like 4 mRNA, complete cds
AT2G43500	2.444818445	5.38E-101	Arabidopsis thaliana nodule inception protein-like protein 8 mRNA, complete cds
AT5G57900	1.733888502	1.04E-100	Arabidopsis thaliana F-box protein SKIP1 mRNA, complete cds
AT2G39980	2.74598798	1.91E-100	Arabidopsis thaliana chromosome 2, complete sequence
AT2G32020	4.077397127	2.24E-100	Arabidopsis thaliana chromosome 2, complete sequence
AT1G02400	2.201466405	2.28E-100	Arabidopsis thaliana gibberellin 2-oxidase 6 mRNA, complete cds
AT3G03790	1.572466625	2.90E-100	Arabidopsis thaliana ankyrin repeat and regulator of chromosome condensation (RCC1) domain-containing protein mRNA, complete cds
AT4G19230	2.66163328	4.49E-100	Arabidopsis thaliana abscisic acid 8'-hydroxylase 1 mRNA, complete cds
AT1G51090	4.945552471	9.13E-100	Arabidopsis thaliana uncharacterized protein mRNA, complete cds
AT1G02660	2.330001651	1.12E-99	Arabidopsis thaliana

			alpha/beta-Hydrolases superfamily protein mRNA, complete cds
AT1G17110	1.587777777	1.43E-99	Arabidopsis thaliana ubiquitin carboxyl-terminal hydrolase 15 mRNA, complete cds
AT5G47330	4.76664745	1.48E-99	Arabidopsis thaliana palmitoyl protein thioesterase family protein mRNA, complete cds
AT4G25490	4.942096913	1.64E-99	Arabidopsis thaliana chromosome 4 sequence
AT1G53560	1.723878506	5.90E-99	Arabidopsis thaliana ribosomal protein L18ae family mRNA, complete cds
AT2G37970	1.422885051	1.10E-98	Arabidopsis thaliana chromosome 2, complete sequence
AT5G52400	10.283953	4.93E-98	Arabidopsis thaliana cytochrome P450, family 715, subfamily A, polypeptide 1 mRNA, complete cds
AT1G13740	2.016789268	5.90E-98	Arabidopsis thaliana ABI five binding protein 2 mRNA, complete cds
AT4G11220	1.225383283	3.44E-97	Arabidopsis thaliana reticulon-like protein B2 mRNA, complete cds
AT5G02880	1.434590552	5.02E-97	Arabidopsis thaliana E3 ubiquitin-protein ligase UPL4 mRNA, complete cds
AT2G29670	1.54298157	1.08E-96	Arabidopsis thaliana tetratricopeptide repeat-containing protein mRNA, complete cds
AT3G21150	1.876948657	1.86E-96	Arabidopsis thaliana chromosome 3, complete sequence
AT3G21890	3.510929146	3.47E-96	Arabidopsis thaliana chromosome 3, complete sequence
AT4G03400	2.41788131	7.75E-96	Arabidopsis thaliana auxin-responsive GH3 family protein mRNA, complete cds
AT5G65110	1.04184746	8.19E-96	Arabidopsis thaliana acyl-coenzyme A oxidase 2 mRNA, complete cds
AT5G16010	1.010698861	9.38E-96	Arabidopsis thaliana 3-oxo-5-alpha-steroid 4-dehydrogenase family protein mRNA, complete cds
AT5G54510	1.212505364	2.33E-95	Arabidopsis thaliana indole-3-acetic acid-amido synthetase GH3.6 mRNA, complete cds
AT4G36950	6.685793198	2.63E-95	Arabidopsis thaliana chromosome 4 sequence
AT5G36160	1.224563999	2.89E-95	Arabidopsis thaliana tyrosine

			aminotransferase mRNA, complete cds
AT1G66500	2.320555309	7.47E-95	Arabidopsis thaliana chromosome 1 sequence
AT1G28380	1.111833389	1.90E-94	Arabidopsis thaliana protein necrotic spotted lesions 1 mRNA, complete cds
AT5G51630	2.010702869	7.82E-94	Arabidopsis thaliana TIR-NBS-LRR class disease resistance protein mRNA, complete cds
AT2G43330	1.141005421	1.37E-93	Arabidopsis thaliana inositol transporter 1 mRNA, complete cds
AT1G56660	2.064866439	1.66E-93	Arabidopsis thaliana uncharacterized protein mRNA, complete cds
AT4G39420	1.798617164	3.06E-93	Arabidopsis thaliana uncharacterized protein mRNA, complete cds
AT4G24415	3.400852493	7.32E-93	Arabidopsis thaliana AT4g24411 mRNA, complete cds
AT1G67900	1.23795287	8.56E-93	Arabidopsis thaliana phototropic-responsive NPH3 family protein mRNA, complete cds
AT5G53420	2.317551131	1.15E-92	Arabidopsis thaliana CCT motif family protein mRNA, complete cds
AT3G28600	6.297267354	1.96E-92	Arabidopsis thaliana chromosome 3, complete sequence
AT4G08170	2.230149839	2.58E-92	Arabidopsis thaliana inositol-tetrakisphosphate 1-kinase 2 mRNA, complete cds
AT5G35460	1.13811438	3.05E-92	Arabidopsis thaliana uncharacterized protein mRNA, complete cds
AT2G39420	1.475844641	4.69E-92	Arabidopsis thaliana alpha/beta-Hydrolases superfamily protein mRNA, complete cds
AT4G13800	5.041786343	4.85E-92	Arabidopsis thaliana uncharacterized protein mRNA, complete cds
AT1G54020	4.262073676	4.95E-92	Arabidopsis thaliana GDSL esterase/lipase mRNA, complete cds
AT5G39050	1.819514562	6.84E-92	Arabidopsis thaliana chromosome 5 sequence
AT2G02990	10.15407272	1.70E-91	Arabidopsis thaliana ribonuclease 1 mRNA, complete cds
AT3G53960	1.390212709	3.70E-91	Arabidopsis thaliana probable peptide/nitrate transporter mRNA, complete cds
AT2G22860	3.109164361	4.36E-91	Arabidopsis thaliana phytosulfokine-beta mRNA, complete cds

AT1G47510	2.866054551	5.64E-91	Arabidopsis thaliana Type I inositol-1,4,5-trisphosphate 5-phosphatase 11 mRNA, complete cds
AT2G38530	1.885921927	5.67E-91	Arabidopsis thaliana non-specific lipid-transfer protein 2 mRNA, complete cds
AT3G54680	1.563284855	1.36E-90	Arabidopsis thaliana proteophosphoglycan-related protein mRNA, complete cds
AT5G62470	1.082216971	1.57E-90	Arabidopsis thaliana Myb transcription factor mRNA, complete cds
AT3G05030	1.955809641	2.39E-90	Arabidopsis thaliana K+/H+ exchanger mRNA, complete cds
AT4G11350	3.908666455	2.64E-90	Arabidopsis thaliana uncharacterized protein mRNA, complete cds
AT5G13330	3.367476356	4.64E-90	Arabidopsis thaliana ethylene-responsive transcription factor ERF113 mRNA, complete cds
AT3G50380	1.577459991	8.95E-90	Arabidopsis thaliana uncharacterized protein mRNA, complete cds
AT3G27025	3.8983737	1.58E-89	Arabidopsis thaliana uncharacterized protein mRNA, complete cds
AT5G22450	1.502336774	2.45E-89	Arabidopsis thaliana uncharacterized protein mRNA, complete cds
AT5G10930	1.499419227	2.68E-89	Arabidopsis thaliana chromosome 5 sequence
AT4G14690	3.476598079	4.17E-89	Arabidopsis thaliana early light-inducible protein 2 mRNA, complete cds
AT5G20380	1.215473263	4.48E-89	Arabidopsis thaliana phosphate transporter 4;5 mRNA, complete cds
AT1G20970	1.446847472	9.49E-89	Arabidopsis thaliana uncharacterized protein mRNA, complete cds
AT3G15350	1.8349035	1.14E-88	Arabidopsis thaliana core-2/I-branching beta-1,6-N-acetylglucosaminyltransferase family protein mRNA, complete cds
AT5G20150	2.7816445	1.31E-88	Arabidopsis thaliana SPX domain-containing protein 1 mRNA, complete cds
AT5G04250	2.485460429	1.84E-88	Arabidopsis thaliana OTU-like cysteine protease family protein mRNA, complete cds
AT1G67310	1.232052336	2.85E-88	Arabidopsis thaliana calmodulin-binding transcription activator 4 mRNA, complete cds

AT1G06620	1.723620167	4.45E-88	Arabidopsis thaliana 2-oxoglutarate-dependent dioxygenase-like protein mRNA, complete cds
AT5G04370	3.460585761	7.94E-88	Arabidopsis thaliana methyltransferase family protein NAMT1 mRNA, complete cds
AT1G61820	1.488824112	9.43E-88	Arabidopsis thaliana beta glucosidase 46 mRNA, complete cds
AT5G57040	1.088265171	1.04E-87	Arabidopsis thaliana Lactoylglutathione lyase / glyoxalase I family protein mRNA, complete cds
AT2G18193	3.227751593	2.82E-87	Arabidopsis thaliana P-loop containing nucleoside triphosphate hydrolases superfamily protein mRNA, complete cds
AT1G70610	1.183220137	1.59E-86	Arabidopsis thaliana ABC transporter B family member 26 mRNA, complete cds
AT5G39610	3.072348321	1.79E-86	Arabidopsis thaliana NAC-domain transcription factor mRNA, complete cds
AT4G04840	2.232248976	1.96E-86	Arabidopsis thaliana methionine sulfoxide reductase B6 mRNA, complete cds
AT1G69252	1.1377015	7.77E-86	Arabidopsis thaliana chromosome 1 sequence
AT2G44970	1.210420805	8.62E-86	Arabidopsis thaliana alpha/beta-Hydrolases superfamily protein mRNA, complete cds
AT3G14720	1.259223466	8.91E-86	Arabidopsis thaliana mitogen-activated protein kinase 19 mRNA, complete cds
AT3G02990	2.407132557	9.99E-86	Arabidopsis thaliana heat stress transcription factor A-1e mRNA, complete cds
AT1G05170	1.626382876	1.17E-85	Arabidopsis thaliana putative beta-1,3-galactosyltransferase 2 mRNA, complete cds
AT4G02360	4.481647376	5.36E-85	Arabidopsis thaliana chromosome 4 sequence
AT1G10090	1.433105024	8.62E-85	Arabidopsis thaliana Early-responsive to dehydration stress protein ERD4 mRNA, complete cds
AT4G34650	2.704091894	1.49E-84	Arabidopsis thaliana squalene synthase 2 mRNA, complete cds
AT2G36895	1.060062418	1.56E-84	Arabidopsis thaliana uncharacterized

			protein mRNA, complete cds
AT1G04240	1.408764565	1.72E-84	Arabidopsis thaliana auxin-responsive protein IAA3 mRNA, complete cds
AT3G28580	4.332896125	2.39E-84	Arabidopsis thaliana chromosome 3, complete sequence
AT1G78100	1.366395281	2.49E-84	Arabidopsis thaliana chromosome 1 sequence
AT3G02840	3.465087153	4.47E-84	Arabidopsis thaliana chromosome 3, complete sequence
AT2G30040	2.168635012	4.72E-84	Arabidopsis thaliana chromosome 2, complete sequence
AT1G51950	1.244926736	5.72E-84	Arabidopsis thaliana auxin-responsive protein IAA18 mRNA, complete cds
AT2G32510	2.962759663	6.30E-84	Arabidopsis thaliana chromosome 2, complete sequence
AT5G56970	5.301888015	1.03E-83	Arabidopsis thaliana cytokinin dehydrogenase 3 mRNA, complete cds
AT1G14250	2.019145262	1.81E-83	Arabidopsis thaliana probable apyrase 5 mRNA, complete cds
AT4G28140	3.775282286	2.30E-83	Arabidopsis thaliana chromosome 4 sequence
AT2G05540	1.526566742	5.83E-83	Arabidopsis thaliana glycine-rich protein mRNA, complete cds
AT1G27910	2.08396848	6.17E-83	Arabidopsis thaliana U-box domain-containing protein 45 mRNA, complete cds
AT5G54470	4.026639799	8.71E-83	Arabidopsis thaliana B-box type zinc finger-containing protein mRNA, complete cds
AT1G21630	1.236705537	1.55E-82	Arabidopsis thaliana calcium-binding EF-hand-containing protein mRNA, complete cds
AT2G26890	1.326716596	2.77E-82	Arabidopsis thaliana gravitropism defective 2 mRNA, complete cds
AT5G13205.1	3.467505175	3.35E-82	Arabidopsis thaliana chromosome 5 sequence
AT1G21580	1.269596479	4.66E-82	Arabidopsis thaliana zinc finger CCCH domain-containing protein mRNA, complete cds
AT3G24310	7.545394148	4.97E-82	Arabidopsis thaliana myb domain protein 305 mRNA, complete cds
AT2G43010	2.090958721	8.06E-82	Arabidopsis thaliana transcription factor PIF4 mRNA, complete cds
AT1G11720	1.365801076	1.28E-81	Arabidopsis thaliana starch synthase 3

			mRNA, complete cds
AT5G37540	1.627610872	1.66E-81	Arabidopsis thaliana chromosome 5 sequence
AT5G12840	1.502076748	1.77E-81	Arabidopsis thaliana nuclear transcription factor Y subunit A-1 mRNA, complete cds
AT4G39730	1.303164479	2.69E-81	Arabidopsis thaliana Lipase/lipooxygenase, PLAT/LH2 family protein mRNA, complete cds
AT5G62220	1.318832764	2.96E-81	Arabidopsis thaliana chromosome 5 sequence
AT1G27200	1.66242001	3.82E-81	Arabidopsis thaliana chromosome 1 sequence
AT1G07900	3.324208902	3.96E-81	Arabidopsis thaliana LOB domain-containing protein 1 mRNA, complete cds
AT3G09560	1.707730265	7.10E-81	Arabidopsis thaliana phosphatidic acid phosphohydrolase 1 mRNA, complete cds
AT4G30460	2.748830729	1.41E-80	Arabidopsis thaliana chromosome 4 sequence
AT4G31570	2.092022691	4.54E-80	Arabidopsis thaliana AUCSIA-1 interacting protein mRNA, complete cds
AT3G11840	2.263353413	6.37E-80	Arabidopsis thaliana E3 ubiquitin-protein ligase PUB24 mRNA, complete cds
AT1G04300	1.116353891	7.50E-80	Arabidopsis thaliana MATH domain-containing protein mRNA, complete cds
AT5G59550	1.342625492	8.45E-80	Arabidopsis thaliana chromosome 5 sequence
AT3G50910	1.192919906	1.12E-79	Arabidopsis thaliana uncharacterized protein mRNA, complete cds
AT2G31960	1.082563104	1.28E-79	Arabidopsis thaliana glucan synthase-like 3 mRNA, complete cds
AT1G70130	9.891038312	1.65E-79	Arabidopsis thaliana putative L-type lectin-domain containing receptor kinase V.2 mRNA, complete cds
AT5G13010	1.220822888	5.41E-79	Arabidopsis thaliana RNA helicase family protein mRNA, complete cds
AT1G62975	3.123185673	5.46E-79	Arabidopsis thaliana transcription factor bHLH125 mRNA, complete cds
AT3G45960	3.396045508	5.74E-79	Arabidopsis thaliana expansin-like A3 mRNA, complete cds
AT1G51780	9.875270996	7.71E-79	Arabidopsis thaliana IAA-amino acid

			hydrolase ILR1-like 5 mRNA, complete cds
AT1G59700	1.712021841	1.17E-78	Arabidopsis thaliana glutathione S-transferase TAU 16 mRNA, complete cds
AT3G26910	1.514511642	1.48E-78	Arabidopsis thaliana hydroxyproline-rich glycoprotein family protein mRNA, complete cds
AT5G48880	2.212058598	2.29E-78	Arabidopsis thaliana 3-keto-acyl-CoA thiolase 2 mRNA, complete cds
AT1G19640	1.981504207	3.13E-78	Arabidopsis thaliana jasmonic acid carboxyl methyltransferase mRNA, complete cds
AT5G49280	1.652417615	6.29E-78	Arabidopsis thaliana chromosome 5 sequence
AT3G44870	9.852903183	6.71E-78	Arabidopsis thaliana S-adenosyl-L-methionine-dependent methyltransferase mRNA, complete cds
AT1G60470	3.407604469	7.41E-78	Arabidopsis thaliana galactinol synthase 4 mRNA, complete cds
AT1G11210	3.434245368	9.09E-78	Arabidopsis thaliana uncharacterized protein mRNA, complete cds
AT1G28420	1.797805508	1.29E-77	Arabidopsis thaliana homeobox-1 mRNA, complete cds
AT3G57680	4.2280733	1.32E-77	Arabidopsis thaliana peptidase S41 family protein mRNA, complete cds
AT1G72760	3.897547099	2.19E-77	Arabidopsis thaliana putative serine/threonine protein kinase mRNA, complete cds
AT4G32190	1.074958692	2.22E-77	Arabidopsis thaliana myosin heavy chain-related protein mRNA, complete cds
AT2G42270	1.441882322	3.06E-77	Arabidopsis thaliana U5 small nuclear ribonucleoprotein helicase mRNA, complete cds
AT5G47060	1.951945544	3.81E-77	Arabidopsis thaliana uncharacterized protein mRNA, complete cds
AT2G32990	1.297452221	4.77E-77	Arabidopsis thaliana glycosyl hydrolase 9B8 mRNA, complete cds
AT5G01260	1.555786844	1.08E-76	Arabidopsis thaliana carbohydrate-binding domain-containing protein mRNA, complete cds
AT2G23450	1.488449346	1.30E-76	Arabidopsis thaliana wall-associated receptor kinase-like 14 mRNA, complete

			cds
AT5G64220	1.477701919	1.41E-76	Arabidopsis thaliana calmodulin-binding transcription activator 2 mRNA, complete cds
AT5G59820	2.00881571	1.64E-76	Arabidopsis thaliana chromosome 5 sequence
AT4G14930	1.428932559	2.00E-76	Arabidopsis thaliana survival protein SurE-like phosphatase/nucleotidase mRNA, complete cds
AT5G58040	1.360334287	4.24E-76	Arabidopsis thaliana FIP1 [V]-like protein mRNA, complete cds
AT2G28320	1.162114599	4.53E-76	Arabidopsis thaliana pleckstrin homology (PH) and lipid-binding START domain-containing protein mRNA, complete cds
AT4G22200	1.220276773	5.54E-76	Arabidopsis thaliana potassium channel AKT2/3 mRNA, complete cds
AT1G65440	1.375418377	8.48E-76	Arabidopsis thaliana transcription elongation factor SPT6-like protein mRNA, complete cds
AT4G18170	3.134741991	2.26E-75	Arabidopsis thaliana WRKY DNA-binding protein 28 mRNA, complete cds
AT3G53540	1.714168691	2.41E-75	Arabidopsis thaliana uncharacterized protein mRNA, complete cds
AT3G60240	1.154482633	5.22E-75	Arabidopsis thaliana eukaryotic translation initiation factor 4G mRNA, complete cds
AT3G14050	1.134154375	1.04E-74	Arabidopsis thaliana RelA-SpoT like protein RSH2 mRNA, complete cds
AT5G65140	1.565923597	1.12E-74	Arabidopsis thaliana probable trehalose-phosphate phosphatase J mRNA, complete cds
AT1G19970	2.17836009	1.13E-74	Arabidopsis thaliana ER lumen protein retaining receptor mRNA, complete cds
AT1G02816	1.965377913	1.31E-74	Arabidopsis thaliana chromosome 1 sequence
AT2G36490	1.517245928	1.71E-74	Arabidopsis thaliana protein ROS1 mRNA, complete cds
AT2G47190	6.298213694	2.26E-74	Arabidopsis thaliana mRNA for MYB transcription factor, complete cds, clone: RAFL16-42-L19
AT1G49520	2.485725323	3.52E-74	Arabidopsis thaliana SWIB complex BAF60b domain-containing protein

			mRNA, complete cds
AT3G49530	1.242611077	3.53E-74	Arabidopsis thaliana transcription factor NTL6 mRNA, complete cds
AT1G15780	1.678151248	5.19E-74	Arabidopsis thaliana uncharacterized protein mRNA, complete cds
AT3G02150	1.838717277	5.33E-74	Arabidopsis thaliana transcription factor TCP13 mRNA, complete cds
AT1G30370	2.579285439	6.49E-74	Arabidopsis thaliana chromosome 1 sequence
AT2G25620	1.683727439	7.58E-74	Arabidopsis thaliana putative protein phosphatase 2C (At2g25620) mRNA, complete cds
AT2G41960	1.660672733	1.32E-73	Arabidopsis thaliana uncharacterized protein mRNA, complete cds
AT1G53090	1.368099416	1.37E-73	Arabidopsis thaliana SPA1-related 4 protein mRNA, complete cds
AT5G41790	1.364370445	2.05E-73	Arabidopsis thaliana COP1-interactive protein 1 mRNA, complete cds
AT4G33467	4.848211488	2.40E-73	Arabidopsis thaliana uncharacterized protein mRNA, complete cds
AT5G62020	1.627268749	4.51E-73	Arabidopsis thaliana heat stress transcription factor B-2a mRNA, complete cds
AT2G26150	1.154463762	4.65E-73	Arabidopsis thaliana heat stress transcription factor A-2 mRNA, complete cds
AT5G47240	2.165842495	1.67E-72	Arabidopsis thaliana nudix hydrolase 8 mRNA, complete cds
AT1G12950	2.603875636	1.77E-72	Arabidopsis thaliana root hair specific 2 mRNA, complete cds
AT5G01380	5.099624934	1.83E-72	Arabidopsis thaliana trihelix transcription factor GT-3a mRNA, complete cds
AT4G34890	1.179431974	2.74E-72	Arabidopsis thaliana xanthine dehydrogenase 1 mRNA, complete cds
AT3G61420	1.334782749	4.01E-72	Arabidopsis thaliana probable RNA polymerase II transcription factor B subunit 1-3 mRNA, complete cds
AT1G56510	1.289659507	2.93E-71	Arabidopsis thaliana TIR-NB-LRR disease resistance protein mRNA, complete cds
AT3G48240	4.410120694	3.70E-71	Arabidopsis thaliana Octicosapeptide/Phox/Bem1p family protein mRNA, complete cds
AT5G40800	4.805285163	4.76E-71	Arabidopsis thaliana chromosome 5

			sequence
AT5G16360	2.465374	4.98E-71	Arabidopsis thaliana putative lecithin retinol acyltransferase-like protein mRNA, complete cds
AT1G28480	4.689404451	6.51E-71	Arabidopsis thaliana chromosome 1 sequence
AT1G76580	1.913024176	1.21E-70	Arabidopsis thaliana squamosa promoter-binding-like protein 16 mRNA, complete cds
AT4G21940	3.329002208	1.24E-70	Arabidopsis thaliana calcium-dependent protein kinase 15 mRNA, complete cds
AT3G62900	1.602812773	1.35E-70	Arabidopsis thaliana CW-type zinc-finger protein mRNA, complete cds
AT1G58250	1.509131523	1.51E-70	Arabidopsis thaliana protein SABRE mRNA, complete cds
AT5G18130	1.439626142	1.93E-70	Arabidopsis thaliana chromosome 5 sequence
AT1G19220	2.043249145	2.06E-70	Arabidopsis thaliana auxin response factor 19 mRNA, complete cds
AT1G20960	1.181804139	3.03E-70	Arabidopsis thaliana putative U5 small nuclear ribonucleoprotein helicase mRNA, complete cds
AT5G01850	1.690745869	3.98E-70	Arabidopsis thaliana protein kinase family protein mRNA, complete cds
AT2G21300	1.322100991	6.36E-70	Arabidopsis thaliana ATP binding microtubule motor family protein mRNA, complete cds
AT1G09530	2.24042719	7.09E-70	Arabidopsis thaliana transcription factor PIF3 mRNA, complete cds
AT1G24706	1.604099131	8.21E-70	Arabidopsis thaliana THO complex subunit 2 mRNA, complete cds
AT3G15500	3.075295605	9.38E-70	Arabidopsis thaliana ATAF-like NAC-domain transcription factor mRNA, complete cds
AT1G17440	1.368799323	1.72E-69	Arabidopsis thaliana transcription initiation factor TFIID subunit 12B mRNA, complete cds
AT1G02850	2.649876087	6.39E-69	Arabidopsis thaliana beta glucosidase 11 mRNA, complete cds
AT3G27170	1.012081133	7.35E-69	Arabidopsis thaliana chloride channel protein CLC-b mRNA, complete cds
AT2G13370	1.363500162	7.60E-69	Arabidopsis thaliana chromatin remodeling 5 mRNA, complete cds
AT3G10960	1.570959958	1.06E-68	Arabidopsis thaliana chromosome 3,

			complete sequence
AT1G07040	1.603637901	1.32E-68	Arabidopsis thaliana uncharacterized protein mRNA, complete cds
AT1G21000	1.061788902	1.42E-68	Arabidopsis thaliana PLATZ transcription factor family protein mRNA, complete cds
AT5G57010	2.867047366	3.37E-68	Arabidopsis thaliana calmodulin-binding family protein mRNA, complete cds
AT4G21840	4.201259404	3.70E-68	Arabidopsis thaliana methionine sulfoxide reductase B8 mRNA, complete cds
AT3G12980	1.756218175	3.74E-68	Arabidopsis thaliana histone acetyltransferase HAC5 mRNA, complete cds
AT3G03480	4.004390937	4.80E-68	Arabidopsis thaliana acetyl CoA:(Z)-3-hexen-1-ol acetyltransferase mRNA, complete cds
AT2G47410	1.50733402	8.91E-68	Arabidopsis thaliana WD40 domain-containing protein mRNA, complete cds
AT1G73500	1.593559626	9.08E-68	Arabidopsis thaliana chromosome 1 sequence
AT5G17490	3.045548261	9.17E-68	Arabidopsis thaliana chromosome 5 sequence
AT3G26840	1.813540751	1.29E-67	Arabidopsis thaliana phytyl ester synthesis and diacylglycerol acyltransferase activity protein mRNA, complete cds
AT1G70060	1.663551024	1.39E-67	Arabidopsis thaliana paired amphipathic helix protein Sin3-like 4 mRNA, complete cds
AT4G25480	5.821652249	1.64E-67	Arabidopsis thaliana chromosome 4 sequence
AT2G04400	1.059266543	2.29E-67	Arabidopsis thaliana indole-3-glycerol phosphate synthase mRNA, complete cds
AT5G62570	1.093495744	4.21E-67	Arabidopsis thaliana calmodulin binding protein 60a mRNA, complete cds
AT3G15990	1.611424921	4.23E-67	Arabidopsis thaliana putative sulfate transporter 3;4 mRNA, complete cds
AT2G32140	2.818137765	4.74E-67	Arabidopsis thaliana transmembrane receptor protein mRNA, complete cds
AT4G01026	1.954727034	5.72E-67	Arabidopsis thaliana abscisic acid receptor PYL7 mRNA, complete cds
AT5G57340	1.737725934	6.44E-67	Arabidopsis thaliana uncharacterized

			protein mRNA, complete cds
AT1G07870	1.779154843	8.19E-67	Arabidopsis thaliana putative serine/threonine-protein kinase RLCKVII mRNA, complete cds
AT5G38130	5.805769208	8.54E-67	Arabidopsis thaliana chromosome 5 sequence
AT1G42990	1.101504667	1.09E-66	Arabidopsis thaliana bZIP transcription factor 60 mRNA, complete cds
AT5G43900	1.101504667	1.09E-66	Arabidopsis thaliana myosin 2 mRNA, complete cds
AT1G22810	5.143580344	1.10E-66	Arabidopsis thaliana chromosome 1 sequence
AT4G27310	1.900208898	1.51E-66	Arabidopsis thaliana B-box domain protein 28 mRNA, complete cds
AT5G24530	1.062378336	2.61E-66	Arabidopsis thaliana putative 2OG-Fe(II) oxygenase mRNA, complete cds
AT5G53050	1.558196557	2.97E-66	Arabidopsis thaliana hydrolase, alpha/beta fold family protein mRNA, complete cds
AT3G59770	1.043741505	3.02E-66	Arabidopsis thaliana phosphoinositide phosphatase SAC9 mRNA, complete cds
AT5G40340	1.553657843	3.32E-66	Arabidopsis thaliana PWWP domain-containing protein mRNA, complete cds
AT2G30550	1.233635591	3.50E-66	Arabidopsis thaliana phospholipase A1-Igamma2 mRNA, complete cds
AT3G55130	1.114728289	4.14E-66	Arabidopsis thaliana chromosome 3, complete sequence
AT4G00450	1.418470887	6.41E-66	Arabidopsis thaliana transcriptional regulator MED12-like protein mRNA, complete cds
AT3G03440	2.503398844	1.14E-65	Arabidopsis thaliana ARM repeat superfamily protein mRNA, complete cds
AT2G42430	3.750604608	1.15E-65	Arabidopsis thaliana LOB domain-containing protein 16 mRNA, complete cds
AT1G05870	1.323365885	2.26E-65	Arabidopsis thaliana uncharacterized protein mRNA, complete cds
AT3G15540	2.050548942	2.40E-65	Arabidopsis thaliana auxin-responsive protein IAA19 mRNA, complete cds
AT2G43240	1.87071446	2.69E-65	Arabidopsis thaliana nucleotide-sugar transporter mRNA, complete cds
AT4G27940	1.593852292	2.69E-65	Arabidopsis thaliana manganese tracking

			factor for mitochondrial SOD2 mRNA, complete cds
AT3G53830	2.388804866	4.25E-65	Arabidopsis thaliana Regulator of chromosome condensation (RCC1) family protein mRNA, complete cds
AT5G58620	1.385623703	5.17E-65	Arabidopsis thaliana zinc finger CCCH domain-containing protein 66 mRNA, complete cds
AT1G77120	1.564576492	5.66E-65	Arabidopsis thaliana alcohol dehydrogenase 1 mRNA, complete cds
AT3G55290	2.581601162	7.34E-65	Arabidopsis thaliana Rossmann-fold NAD(P)-binding domain-containing protein mRNA, complete cds
AT5G64250	1.276042139	8.83E-65	Arabidopsis thaliana Aldolase-type TIM barrel family protein mRNA, complete cds
AT1G60730	1.685778069	1.28E-64	Arabidopsis thaliana probable aldo-keto reductase 5 mRNA, complete cds
AT1G78230	1.103595547	1.37E-64	Arabidopsis thaliana Outer arm dynein light chain 1 protein mRNA, complete cds
AT5G54080	1.241535559	2.23E-64	Arabidopsis thaliana homogentisate 1,2-dioxygenase mRNA, complete cds
AT1G64900	1.213389948	4.08E-64	Arabidopsis thaliana chromosome 1 sequence
AT5G13210	2.726479821	6.31E-64	Arabidopsis thaliana chromosome 5 sequence
AT1G21590	1.04180586	8.48E-64	Arabidopsis thaliana putative protein kinase mRNA, complete cds
AT1G29330	1.557123106	8.76E-64	Arabidopsis thaliana ER lumen protein retaining receptor mRNA, complete cds
AT1G69830	2.309177716	1.27E-63	Arabidopsis thaliana alpha-amylase-like 3 mRNA, complete cds
AT1G09930	7.122363858	1.49E-63	Arabidopsis thaliana oligopeptide transporter 2 mRNA, complete cds
AT4G21440	4.045548261	1.57E-63	Arabidopsis thaliana R2R3 family MYB transcription factor mRNA, complete cds
AT5G05140	1.095444706	1.72E-63	Arabidopsis thaliana transcription elongation factor (TFIIS) family protein mRNA, complete cds
AT2G46420	1.243929709	1.91E-63	Arabidopsis thaliana uncharacterized protein mRNA, complete cds
AT1G77300	1.695636405	1.94E-63	Arabidopsis thaliana histone-lysine N-methyltransferase ASHH2 mRNA,

			complete cds
AT5G13370	1.663903425	2.67E-63	Arabidopsis thaliana auxin-responsive GH3 family protein mRNA, complete cds
AT5G50720	2.620168818	3.03E-63	Arabidopsis thaliana HVA22-like protein E mRNA, complete cds
AT1G30530	1.114880283	4.10E-63	Arabidopsis thaliana UDP-glucosyl transferase 78D1 mRNA, complete cds
AT5G43620	2.87604852	4.74E-63	Arabidopsis thaliana chromosome 5 sequence
AT1G05680	6.039901698	7.81E-63	Arabidopsis thaliana Uridine diphosphate glycosyltransferase 74E2 mRNA, complete cds
AT5G04560	1.522735018	8.31E-63	Arabidopsis thaliana transcriptional activator DEMETER mRNA, complete cds
AT3G57880	1.002268581	1.40E-62	Arabidopsis thaliana C2 domain-containing plant phosphoribosyltransferase-like protein mRNA, complete cds
AT1G28190	2.274927113	2.86E-62	Arabidopsis thaliana chromosome 1 sequence
AT5G52410	2.221290589	3.09E-62	Arabidopsis thaliana uncharacterized protein mRNA, complete cds
AT5G24660	2.956440788	4.49E-62	Arabidopsis thaliana chromosome 5 sequence
AT3G53600	6.449270447	6.02E-62	Arabidopsis thaliana chromosome 3, complete sequence
AT2G01020	2.012381397	8.81E-62	Erysimum belvederense internal transcribed spacer 1, partial sequence; 5.8S ribosomal RNA gene, complete sequence; and internal transcribed spacer 2, partial sequence
AT5G52450	1.427563506	8.82E-62	Arabidopsis thaliana MATE efflux family protein mRNA, complete cds
AT1G52855	3.942278991	1.68E-61	Arabidopsis thaliana chromosome 1 sequence
AT4G16670	1.987982974	1.82E-61	Arabidopsis thaliana uncharacterized protein mRNA, complete cds
AT1G19490	2.135113646	2.05E-61	Arabidopsis thaliana basic-leucine zipper transcription factor family protein mRNA, complete cds
AT2G38760	2.032420827	2.97E-61	Arabidopsis thaliana annexin D3 mRNA, complete cds

AT1G28960	1.251392837	4.13E-61	Arabidopsis thaliana ppGpp pyrophosphohydrolase mRNA, complete cds
AT5G23150	1.71731559	4.32E-61	Arabidopsis thaliana ENHANCER OF AG-4 protein 2 mRNA, complete cds
AT2G18090	1.059722221	4.41E-61	Arabidopsis thaliana PHD finger, SWIB/MDM2 and GYF domain-containing protein mRNA, complete cds
AT1G28370	2.585150229	6.81E-61	Arabidopsis thaliana chromosome 1 sequence
AT3G44260	1.11276671	7.26E-61	Arabidopsis thaliana chromosome 3, complete sequence
AT3G49570	6.420587693	7.99E-61	Arabidopsis thaliana chromosome 3, complete sequence
AT1G24460	1.497602569	1.09E-60	Arabidopsis thaliana TGN-localized SYP41 interacting protein mRNA, complete cds
AT2G27830	1.288079262	3.15E-60	Arabidopsis thaliana chromosome 2, complete sequence
AT5G54490	3.74598798	4.13E-60	Arabidopsis thaliana chromosome 5 sequence
AT1G68570	1.083190595	4.20E-60	Arabidopsis thaliana putative nitrite transporter mRNA, complete cds
AT3G07350	2.383964479	4.73E-60	Arabidopsis thaliana chromosome 3, complete sequence
AT3G44326	3.008181409	4.88E-60	Arabidopsis thaliana chromosome 3, complete sequence
AT3G19970	1.838782075	7.74E-60	Arabidopsis thaliana uncharacterized protein mRNA, complete cds
AT1G07150	3.26642445	7.92E-60	Arabidopsis thaliana mitogen-activated protein kinase kinase kinase 13 mRNA, complete cds
AT5G64905	4.188506215	9.60E-60	Arabidopsis thaliana chromosome 5 sequence
AT1G22640	1.717165144	1.06E-59	Arabidopsis thaliana transcription factor MYB3 mRNA, complete cds
AT5G26220	3.968380401	1.24E-59	Arabidopsis thaliana ChaC-like family protein mRNA, complete cds
AT5G63320	1.300851624	1.82E-59	Arabidopsis thaliana nuclear protein X1 mRNA, complete cds
AT5G41750	1.512674272	2.10E-59	Arabidopsis thaliana TIR-NBS-LRR class disease resistance protein mRNA, complete cds

AT3G55500	1.27445399	2.22E-59	Arabidopsis thaliana expansin A16 mRNA, complete cds
AT1G12420	1.393326007	3.07E-59	Arabidopsis thaliana ACT domain repeat 8 protein mRNA, complete cds
AT4G24380	2.713651997	3.50E-59	Arabidopsis thaliana uncharacterized protein mRNA, complete cds
AT4G13410	3.725667995	4.48E-59	Arabidopsis thaliana putative mannan synthase 15 mRNA, complete cds
AT2G01010	1.823495418	6.73E-59	Arabidopsis thaliana chromosome 2, complete sequence
AT5G27600	1.523990438	7.13E-59	Arabidopsis thaliana long-chain acyl-CoA synthetase 7 mRNA, complete cds
AT1G02930	1.106605547	1.32E-58	Arabidopsis thaliana glutathione S-transferase F6 mRNA, complete cds
AT1G50030	1.166594542	2.88E-58	Arabidopsis thaliana serine/threonine-protein kinase TOR mRNA, complete cds
AT4G25470	3.267940683	3.33E-58	Arabidopsis thaliana chromosome 4 sequence
AT5G16680	1.535514248	4.15E-58	Arabidopsis thaliana RING/FYVE/PHD zinc finger-containing protein mRNA, complete cds
AT5G41120	2.364117344	4.86E-58	Arabidopsis thaliana Esterase/lipase/thioesterase family protein mRNA, complete cds
AT5G28237	3.561563408	5.45E-58	Arabidopsis thaliana tryptophan synthase beta chain-like protein mRNA, complete cds
AT4G31870	3.36265934	6.65E-58	Arabidopsis thaliana glutathione peroxidase 7 mRNA, complete cds
AT4G18390	1.270318621	9.19E-58	Arabidopsis thaliana transcription factor TCP2 mRNA, complete cds
AT4G21830	1.067374624	1.36E-57	Arabidopsis thaliana peptide methionine sulfoxide reductase B7 mRNA, complete cds
AT3G19200	2.804540162	1.51E-57	Arabidopsis thaliana uncharacterized protein mRNA, complete cds
AT5G16270	1.178732716	1.55E-57	Arabidopsis thaliana sister chromatid cohesion 1 protein 4 mRNA, complete cds
AT1G19650	1.478146589	1.82E-57	Arabidopsis thaliana Sec14p-like phosphatidylinositol transfer family protein mRNA, complete cds

AT2G27420	1.827368091	2.85E-57	Arabidopsis thaliana cysteine proteinase-like protein mRNA, complete cds
AT3G02040	2.777059425	5.53E-57	Arabidopsis thaliana glycerophosphodiester phosphodiesterase 1 mRNA, complete cds
AT4G15100	8.067916074	8.76E-57	Arabidopsis thaliana serine carboxypeptidase-like 30 mRNA, complete cds
AT1G08800	1.454045611	1.16E-56	Arabidopsis thaliana uncharacterized protein mRNA, complete cds
AT3G23240	5.306075812	1.36E-56	Arabidopsis thaliana chromosome 3, complete sequence
AT2G22680	1.379104359	1.57E-56	Arabidopsis thaliana chromosome 2, complete sequence
AT4G15450	1.580585537	2.19E-56	Arabidopsis thaliana Senescence/dehydration-associated protein-like protein mRNA, complete cds
AT1G76650	2.683422293	2.19E-56	Arabidopsis thaliana chromosome 1 sequence
AT1G42430	1.42545954	3.07E-56	Arabidopsis thaliana uncharacterized protein mRNA, complete cds
AT2G47000	1.069171866	4.14E-56	Arabidopsis thaliana auxin efflux transmembrane transporter MDR4 mRNA, complete cds
AT1G64810	1.587806311	6.74E-56	Arabidopsis thaliana APO protein 1 mRNA, complete cds
AT1G09350	5.077397127	7.14E-56	Arabidopsis thaliana galactinol synthase 3 mRNA, complete cds
AT2G35980	4.022828185	1.24E-55	Arabidopsis thaliana chromosome 2, complete sequence
AT1G64590	1.711208821	1.25E-55	Arabidopsis thaliana NAD(P)-binding Rossmann-fold superfamily protein mRNA, complete cds
AT4G31750	1.284636972	1.35E-55	Arabidopsis thaliana putative protein phosphatase 2C 59 mRNA, complete cds
AT3G54030	1.135185474	1.42E-55	Arabidopsis thaliana Protein kinase protein with tetratricopeptide repeat domain mRNA, complete cds
AT1G68360	2.865998841	1.49E-55	Arabidopsis thaliana chromosome 1 sequence
AT3G14590	2.04261297	1.62E-55	Arabidopsis thaliana protein NTMC2T6.2 mRNA, complete cds
AT3G15670	9.225457351	1.73E-55	Arabidopsis thaliana Late embryogenesis

			abundant protein (LEA) family protein mRNA, complete cds
AT2G34310	1.013749216	2.41E-55	Arabidopsis thaliana uncharacterized protein mRNA, complete cds
AT3G55110	1.74479419	2.95E-55	Arabidopsis thaliana ABC transporter G family member 18 mRNA, complete cds
AT3G61630	1.655134549	3.35E-55	Arabidopsis thaliana chromosome 3, complete sequence
AT4G37680	1.0126649	3.90E-55	Arabidopsis thaliana heptahelical transmembrane protein HHP4 mRNA, complete cds
AT1G63720	2.272463241	4.57E-55	Arabidopsis thaliana uncharacterized protein mRNA, complete cds
AT5G47550	1.807060154	4.91E-55	Arabidopsis thaliana chromosome 5 sequence
AT3G57300	1.055625334	5.43E-55	Arabidopsis thaliana DNA helicase INO80 complex-like 1 mRNA, complete cds
AT4G10390	2.125986877	7.04E-55	Arabidopsis thaliana probable receptor-like protein kinase mRNA, complete cds
AT1G20823	1.01838593	7.41E-55	Arabidopsis thaliana chromosome 1 sequence
AT5G41400	1.842561239	7.54E-55	Arabidopsis thaliana chromosome 5 sequence
AT1G64200	1.073472263	9.86E-55	Arabidopsis thaliana V-type proton ATPase subunit E3 mRNA, complete cds
AT5G13590	1.219722736	1.01E-54	Arabidopsis thaliana uncharacterized protein mRNA, complete cds
AT3G27870	2.060750668	1.39E-54	Arabidopsis thaliana putative phospholipid-transporting ATPase 8 mRNA, complete cds
AT1G17750	2.280621264	2.03E-54	Arabidopsis thaliana leucine-rich repeat receptor-like protein kinase PEPR2 mRNA, complete cds
AT5G25930	1.296415851	2.28E-54	Arabidopsis thaliana protein kinase family protein with leucine-rich repeat domain mRNA, complete cds
AT1G71960	1.199249461	2.76E-54	Arabidopsis thaliana ABC transporter G family member 25 mRNA, complete cds
AT1G09240	2.646998885	3.01E-54	Arabidopsis thaliana chromosome 1 sequence
AT4G14760	1.803951026	3.61E-54	Arabidopsis thaliana kinase interacting-like protein mRNA, complete

			cds
AT4G39030	1.586310958	5.72E-54	Arabidopsis thaliana enhanced disease susceptibility 5 mRNA, complete cds
AT4G17650	1.339493944	6.61E-54	Arabidopsis thaliana polyketide cyclase / dehydrase and lipid transport protein mRNA, complete cds
AT2G23840	2.08681628	1.22E-53	Arabidopsis thaliana HNH endonuclease mRNA, complete cds
AT5G49520	1.734419567	1.39E-53	Arabidopsis thaliana putative WRKY transcription factor 48 mRNA, complete cds
AT1G55020	1.165079894	2.25E-53	Arabidopsis thaliana lipoxygenase 1 mRNA, complete cds
AT4G12290	1.51410127	2.39E-53	Arabidopsis thaliana copper amine oxidase family protein mRNA, complete cds
AT5G36220	1.70971803	2.51E-53	Arabidopsis thaliana cytochrome P450 81D1 mRNA, complete cds
AT1G07510	1.205508877	3.05E-53	Arabidopsis thaliana FTSH protease 10 mRNA, complete cds
AT3G52340	1.210432646	4.62E-53	Arabidopsis thaliana sucrose-phosphatase 2 mRNA, complete cds
AT3G04000	2.593984886	5.89E-53	Arabidopsis thaliana aldehyde reductase mRNA, complete cds
AT2G43620	2.523026335	5.91E-53	Arabidopsis thaliana chitinase family protein mRNA, complete cds
AT4G09750	2.638923002	6.92E-53	Arabidopsis thaliana mRNA for hypothetical protein, complete cds, clone: RAFL14-08-C18
AT2G34600	3.262778978	8.00E-53	Arabidopsis thaliana jasmonate-zim-domain protein 7 mRNA, complete cds
AT4G28290	1.769802804	8.35E-53	Arabidopsis thaliana uncharacterized protein mRNA, complete cds
AT5G55400	1.637397148	9.10E-53	Arabidopsis thaliana actin binding Calponin homology domain-containing protein mRNA, complete cds
AT1G07590	1.733604255	1.13E-52	Arabidopsis thaliana pentatricopeptide repeat-containing protein mRNA, complete cds
AT2G32680	1.684040159	1.17E-52	Arabidopsis thaliana chromosome 2, complete sequence
AT4G12400	1.374583243	1.23E-52	Arabidopsis thaliana carboxylate clamp-tetratricopeptide repeat protein

			mRNA, complete cds
AT5G20360	1.361361382	1.66E-52	Arabidopsis thaliana octicosapeptide/Phox/Bem1p and tetratricopeptide repeat domain-containing protein mRNA, complete cds
AT1G09180	3.446086191	2.42E-52	Arabidopsis thaliana secretion-associated RAS 1 protein mRNA, complete cds
AT1G53163	3.400390979	3.56E-52	Arabidopsis thaliana chromosome 1 sequence
AT1G28260	1.890154579	3.61E-52	Arabidopsis thaliana Telomerase activating protein Est1 mRNA, complete cds
AT4G21580	1.112723743	4.92E-52	Arabidopsis thaliana oxidoreductase, zinc-binding dehydrogenase family protein mRNA, complete cds
AT2G25530	2.233175265	5.49E-52	Arabidopsis thaliana AFG1-like ATPase family protein mRNA, complete cds
AT4G37470	1.336010496	5.79E-52	Arabidopsis thaliana probable esterase KAI2 mRNA, complete cds
AT4G14368	6.174831278	6.17E-52	Arabidopsis thaliana regulator of chromosome condensation repeat-containing protein mRNA, complete cds
AT2G43800	1.684865292	6.18E-52	Arabidopsis thaliana formin-like protein 2 mRNA, complete cds
AT5G41100	1.859896387	9.27E-52	Arabidopsis thaliana uncharacterized protein mRNA, complete cds
AT3G01320	1.58856258	1.12E-51	Arabidopsis thaliana paired amphipathic helix protein Sin3-like 1 mRNA, complete cds
AT5G56240	1.124136997	1.23E-51	Arabidopsis thaliana uncharacterized protein mRNA, complete cds
AT1G03090	1.278775845	1.39E-51	Arabidopsis thaliana methylcrotonoyl-CoA carboxylase subunit alpha mRNA, complete cds
AT2G36640	9.089942381	1.56E-51	Arabidopsis thaliana putative phosphotyrosine mRNA, complete cds
AT4G13110	1.890679834	1.70E-51	Arabidopsis thaliana chromosome 4 sequence
AT3G19290	1.126059937	1.87E-51	Arabidopsis thaliana ABRE binding factor 4 mRNA, complete cds
AT3G54280	1.308919391	1.90E-51	Arabidopsis thaliana TATA-binding protein-associated factor BTAF1 mRNA,

			complete cds
AT2G39260	1.232067237	2.37E-51	Arabidopsis thaliana regulator of nonsense transcripts UPF2 mRNA, complete cds
AT1G07400	1.53485041	3.19E-51	Arabidopsis thaliana chromosome 1 sequence
AT2G48160	1.094809481	3.26E-51	Arabidopsis thaliana Tudor/PWWP/MBT domain-containing protein mRNA, complete cds
AT1G23040	2.008395263	4.94E-51	Arabidopsis thaliana chromosome 1 sequence
AT1G04780	1.028325275	5.07E-51	Arabidopsis thaliana ankyrin repeat-containing protein mRNA, complete cds
AT4G14370	2.556510181	7.57E-51	Arabidopsis thaliana TIR-NBS-LRR class disease resistance protein mRNA, complete cds
AT3G20830	1.77096157	1.68E-50	Arabidopsis thaliana chromosome 3, complete sequence
AT3G54670	1.412533631	1.75E-50	Arabidopsis thaliana structural maintenance of chromosomes 1 mRNA, complete cds
AT2G20960	1.003471287	2.12E-50	Arabidopsis thaliana phospholipase-like protein (PEARLI 4) domain-containing protein mRNA, complete cds
AT1G12610	6.125921678	2.58E-50	Arabidopsis thaliana chromosome 1 sequence
AT2G40475	1.47375482	3.81E-50	Arabidopsis thaliana chromosome 2, complete sequence
AT1G72180	1.227691215	5.67E-50	Arabidopsis thaliana leucine-rich receptor-like protein kinase mRNA, complete cds
AT5G37990	1.867549959	6.71E-50	Arabidopsis thaliana probable S-adenosylmethionine-dependent methyltransferase mRNA, complete cds
AT5G22290	1.977861454	7.62E-50	Arabidopsis thaliana membrane-tethered transcription factor ANAC089 mRNA, complete cds
AT1G48650	1.033654583	8.23E-50	Arabidopsis thaliana DEA(D/H)-box RNA helicase family protein mRNA, complete cds
AT3G55940	2.404105278	8.46E-50	Arabidopsis thaliana phosphoinositide phospholipase C 7 mRNA, complete cds
AT4G32250	1.127686548	8.74E-50	Arabidopsis thaliana protein kinase

			family protein mRNA, complete cds
AT3G24840	1.461273905	1.13E-49	Arabidopsis thaliana Sec14p-like phosphatidylinositol transfer family protein mRNA, complete cds
AT1G04570	3.162814314	1.18E-49	Arabidopsis thaliana probable folate-biopterin transporter mRNA, complete cds
AT3G09350	1.022165334	1.46E-49	Arabidopsis thaliana protein Fes1A mRNA, complete cds
AT5G09930	5.367476356	1.58E-49	Arabidopsis thaliana ABC transporter F family member 2 mRNA, complete cds
AT4G03200	1.142056242	1.84E-49	Arabidopsis thaliana uncharacterized protein mRNA, complete cds
AT2G03140	1.483395644	2.00E-49	Arabidopsis thaliana alpha/beta-Hydrolases superfamily protein mRNA, complete cds
AT5G07740	1.254281283	2.73E-49	Arabidopsis thaliana chromosome 5 sequence
AT1G29640	3.03000741	3.71E-49	Arabidopsis thaliana chromosome 1 sequence
AT1G54710	1.111041561	3.86E-49	Arabidopsis thaliana autophagy 18H-like protein mRNA, complete cds
AT2G37430	3.560121434	6.15E-49	Arabidopsis thaliana chromosome 2, complete sequence
AT1G17830	2.970521216	6.19E-49	Arabidopsis thaliana uncharacterized protein mRNA, complete cds
AT1G80120	2.565805073	6.94E-49	Arabidopsis thaliana uncharacterized protein mRNA, complete cds
AT4G29920	2.062621775	6.99E-49	Arabidopsis thaliana Clp amino terminal domain-containing protein mRNA, complete cds
AT4G34280	1.474884144	9.66E-49	Arabidopsis thaliana WD40 domain-containing protein mRNA, complete cds
AT5G03890	4.202052747	1.13E-48	Arabidopsis thaliana chromosome 5 sequence
AT1G72500	1.085887191	1.15E-48	Arabidopsis thaliana uncharacterized protein mRNA, complete cds
AT5G40000	5.652878575	1.49E-48	Arabidopsis thaliana chromosome 5 sequence
AT4G25520	1.24125864	1.82E-48	Arabidopsis thaliana protein SEUSS-like 1 mRNA, complete cds
AT4G24160	1.05969875	1.87E-48	Arabidopsis thaliana lysophosphatidic acid acyltransferase mRNA, complete

			cds
AT2G47950	3.926560225	2.65E-48	Arabidopsis thaliana chromosome 2, complete sequence
AT3G54950	1.587866425	3.09E-48	Arabidopsis thaliana patatin-like protein 6 mRNA, complete cds
AT1G78490	1.051917786	3.19E-48	Arabidopsis thaliana cytochrome P450, family 708, subfamily A, polypeptide 3 mRNA, complete cds
AT5G43260	1.670039126	5.57E-48	Arabidopsis thaliana chromosome 5 sequence
AT5G61140	1.135185474	6.84E-48	Arabidopsis thaliana U5 small nuclear ribonucleoprotein helicase mRNA, complete cds
AT5G38200	2.432571385	6.96E-48	Arabidopsis thaliana class I glutamine amidotransferase-like protein mRNA, complete cds
AT3G23410	1.314453255	9.54E-48	Arabidopsis thaliana long-chain-alcohol oxidase FAO3 mRNA, complete cds
AT1G10900	1.103746508	1.15E-47	Arabidopsis thaliana 1-phosphatidylinositol-4-phosphate 5-kinase mRNA, complete cds
AT3G12510	3.299304854	1.37E-47	Arabidopsis thaliana chromosome 3, complete sequence
AT5G53760	1.41788131	1.49E-47	Arabidopsis thaliana MLO-like protein 11 mRNA, complete cds
AT5G64660	1.635617653	1.62E-47	Arabidopsis thaliana chromosome 5 sequence
AT2G45900	2.694205437	1.66E-47	Arabidopsis thaliana phosphatidylinositol N-acetyglucosaminlytransferase subunit P-like protein mRNA, complete cds
AT2G41475	1.024648181	1.94E-47	Arabidopsis thaliana PLAT domain-containing protein mRNA, complete cds
AT1G78210	1.153040982	2.13E-47	Arabidopsis thaliana alpha/beta-Hydrolases superfamily protein mRNA, complete cds
AT3G49110	1.162802041	2.16E-47	Arabidopsis thaliana peroxidase 33 mRNA, complete cds
AT1G64970	1.289002298	2.45E-47	Arabidopsis thaliana gamma-tocopherol methyltransferase mRNA, complete cds
AT3G29320	1.136504535	2.50E-47	Arabidopsis thaliana alpha-glucan phosphorylase 1 mRNA, complete cds
AT5G59580	4.51947945	3.39E-47	Arabidopsis thaliana UDP-glucosyl transferase 76E1 mRNA, complete cds

AT1G69360	1.310431092	4.01E-47	Arabidopsis thaliana uncharacterized protein mRNA, complete cds
AT3G05650	1.61540387	5.04E-47	Arabidopsis thaliana chromosome 3, complete sequence
AT4G40010	2.167317225	5.64E-47	Arabidopsis thaliana SNF1-related protein kinase 2.7 mRNA, complete cds
AT1G08630	4.51224788	6.14E-47	Arabidopsis thaliana threonine aldolase mRNA, complete cds
AT4G37730	1.949970601	6.83E-47	Arabidopsis thaliana chromosome 4 sequence
AT5G13080	3.61779971	9.93E-47	Arabidopsis thaliana putative WRKY transcription factor 75 mRNA, complete cds
AT1G16710	1.314975436	1.15E-46	Arabidopsis thaliana histone acetyltransferase HAC12 mRNA, complete cds
AT2G25690	1.907164777	1.77E-46	Arabidopsis thaliana uncharacterized protein mRNA, complete cds
AT3G47580	1.923292511	2.24E-46	Arabidopsis thaliana Leucine-rich repeat protein kinase family protein mRNA, complete cds
AT5G66700	4.645461104	2.46E-46	Arabidopsis thaliana homeobox-leucine zipper protein ATHB-53 mRNA, complete cds
AT1G76590	1.674565942	3.09E-46	Arabidopsis thaliana PLATZ transcription factor family protein mRNA, complete cds
AT1G68690	1.744546005	4.85E-46	Arabidopsis thaliana proline-rich receptor-like protein kinase PERK9 mRNA, complete cds
AT4G35770	1.837440066	6.69E-46	Arabidopsis thaliana senescence-associated protein DIN1 mRNA, complete cds
AT4G37710	8.878438276	6.88E-46	Arabidopsis thaliana chromosome 4 sequence
AT1G08230	1.526674951	7.27E-46	Arabidopsis thaliana GABA transporter 1 mRNA, complete cds
AT4G24680	1.050911446	8.06E-46	Arabidopsis thaliana protein MODIFIER OF SNC1 1 mRNA, complete cds
AT5G04460	1.270968375	8.53E-46	Arabidopsis thaliana RING/U-box domain-containing protein mRNA, complete cds
AT3G24870	1.801961618	1.43E-45	Arabidopsis thaliana Helicase/SANT-associated, DNA binding

			protein mRNA, complete cds
AT1G28010	1.580585537	1.82E-45	Arabidopsis thaliana ABC transporter B family member 14 mRNA, complete cds
AT1G65580	1.231240579	2.13E-45	Arabidopsis thaliana Type II inositol-1,4,5-trisphosphate 5-phosphatase FRA3 mRNA, complete cds
AT4G34860	1.625216462	2.45E-45	Arabidopsis thaliana beta-fructofuranosidase-like protein mRNA, complete cds
AT4G32480	1.394243266	3.43E-45	Arabidopsis thaliana uncharacterized protein mRNA, complete cds
AT1G62560	1.389827667	3.58E-45	Arabidopsis thaliana flavin-containing monooxygenase FMO GS-OX3 mRNA, complete cds
AT1G65660	1.051013022	5.21E-45	Arabidopsis thaliana putative step II splicing factor SMP1 mRNA, complete cds
AT2G38740	1.024752297	5.62E-45	Arabidopsis thaliana haloacid dehalogenase-like hydrolase family protein mRNA, complete cds
AT2G29120	1.658362883	6.97E-45	Arabidopsis thaliana glutamate receptor 2.7 mRNA, complete cds
AT5G66460	1.394443404	7.01E-45	Arabidopsis thaliana mannan endo-1,4-beta-mannosidase 7 mRNA, complete cds
AT3G22740	1.559039007	7.92E-45	Arabidopsis thaliana homocysteine S-methyltransferase 3 mRNA, complete cds
AT1G73325	4.968380401	1.57E-44	Arabidopsis thaliana chromosome 1 sequence
AT2G34720	1.018290054	1.72E-44	Arabidopsis thaliana nuclear transcription factor Y subunit A-4 mRNA, complete cds
AT3G18950	1.67050883	2.04E-44	Arabidopsis thaliana chromosome 3, complete sequence
AT3G59480	2.591516631	2.62E-44	Arabidopsis thaliana probable fructokinase-4 mRNA, complete cds
AT3G55580	2.921889794	3.25E-44	Arabidopsis thaliana regulator of chromosome condensation repeat-containing protein mRNA, complete cds
AT1G58180	1.230787684	3.68E-44	Arabidopsis thaliana beta carbonic anhydrase 6 mRNA, complete cds

AT1G74360	2.221705217	3.99E-44	Arabidopsis thaliana putative LRR receptor-like serine/threonine-protein kinase mRNA, complete cds
AT5G57510	5.50497988	6.41E-44	Arabidopsis thaliana chromosome 5 sequence
AT3G08760	1.004545287	6.95E-44	Arabidopsis thaliana osmotic stress-inducible kinase mRNA, complete cds
AT1G76390	1.511801146	8.62E-44	Arabidopsis thaliana U-box domain-containing protein 43 mRNA, complete cds
AT5G24030	1.130054557	8.71E-44	Arabidopsis thaliana SLAC1 homologue 3 mRNA, complete cds
AT2G41380	3.063470169	8.97E-44	Arabidopsis thaliana S-adenosyl-L-methionine-dependent methyltransferase-like protein mRNA, complete cds
AT4G22880	1.671234697	1.08E-43	Arabidopsis thaliana leucoanthocyanidin dioxygenase mRNA, complete cds
AT5G55310	1.005560412	1.09E-43	Arabidopsis thaliana DNA topoisomerase 1 beta mRNA, complete cds
AT4G18600	2.349609935	1.43E-43	Arabidopsis thaliana SCAR family protein WAVE5 mRNA, complete cds
AT3G16330	2.012381397	2.54E-43	Arabidopsis thaliana chromosome 3, complete sequence
AT1G15010	2.988062767	2.70E-43	Arabidopsis thaliana chromosome 1 sequence
AT4G36880	2.988062767	2.70E-43	Arabidopsis thaliana cysteine proteinase1 mRNA, complete cds
AT5G18830	1.141371029	3.34E-43	Arabidopsis thaliana squamosa promoter-binding-like protein 7 mRNA, complete cds
AT4G11890	1.906145205	3.80E-43	Arabidopsis thaliana receptor-like cytosolic kinase ARCK1 mRNA, complete cds
AT1G24190	1.246245612	4.04E-43	Arabidopsis thaliana paired amphipathic helix protein Sin3-like 3 mRNA, complete cds
AT1G59590	1.585381387	4.15E-43	Arabidopsis thaliana chromosome 1 sequence
AT2G46560	1.171853579	4.88E-43	Arabidopsis thaliana transducin family protein / WD-40 repeat family protein mRNA, complete cds
AT2G03980	1.128010422	5.06E-43	Arabidopsis thaliana GDSL

			esterase/lipase mRNA, complete cds
AT4G36080	1.861394644	5.26E-43	Arabidopsis thaliana phosphotransferases/inositol or phosphatidylinositol kinases mRNA, complete cds
AT2G18170	1.44110538	6.50E-43	Arabidopsis thaliana mitogen-activated protein kinase 7 mRNA, complete cds
AT1G79310	2.273817249	7.76E-43	Arabidopsis thaliana metacaspase 7 mRNA, complete cds
AT4G01360	4.391323098	8.58E-43	Arabidopsis thaliana BYPASS1-related protein mRNA, complete cds
AT4G33540	1.223305253	1.03E-42	Arabidopsis thaliana metallo-beta-lactamase family protein mRNA, complete cds
AT2G16485	1.367476356	1.09E-42	Arabidopsis thaliana GW repeat- and PHD finger-containing protein NERD mRNA, complete cds
AT1G21120	1.258979815	1.23E-42	Arabidopsis thaliana O-methyltransferase family protein mRNA, complete cds
AT1G14040	1.205166654	1.27E-42	Arabidopsis thaliana phosphate transporter PHO1-3 mRNA, complete cds
AT4G03820	1.567432079	1.40E-42	Arabidopsis thaliana uncharacterized protein mRNA, complete cds
AT1G70480	1.115778184	1.58E-42	Arabidopsis thaliana uncharacterized protein mRNA, complete cds
AT1G01240	1.042694264	1.63E-42	Arabidopsis thaliana chromosome 1 sequence
AT1G58230	1.233383461	1.88E-42	Arabidopsis thaliana WD40 and Beach domain-containing protein mRNA, complete cds
AT1G54160	2.998242547	2.05E-42	Arabidopsis thaliana nuclear transcription factor Y subunit A-5 mRNA, complete cds
AT3G56270	1.639175623	2.18E-42	Arabidopsis thaliana uncharacterized protein mRNA, complete cds
AT1G62710	1.837743376	2.70E-42	Arabidopsis thaliana vacuolar-processing enzyme beta mRNA, complete cds
AT1G18100	5.141472681	2.75E-42	Arabidopsis thaliana protein MOTHER of FT and TF 1 mRNA, complete cds
AT1G25520	1.196754065	2.84E-42	Arabidopsis thaliana putative transmembrane protein mRNA, complete cds
AT1G57590	2.173773972	3.63E-42	Arabidopsis thaliana pectinacetylesterase

			family protein mRNA, complete cds
AT2G22880	3.341004145	4.19E-42	Arabidopsis thaliana chromosome 2, complete sequence
AT1G16410	1.236025503	4.66E-42	Arabidopsis thaliana dihomomethionine N-hydroxylase mRNA, complete cds
AT5G05220	6.454939198	4.83E-42	Arabidopsis thaliana chromosome 5 sequence
AT1G32490	1.211333532	5.60E-42	Arabidopsis thaliana DEAH RNA helicase homolog PRP2 mRNA, complete cds
AT2G45760	3.922537371	5.86E-42	Arabidopsis thaliana chromosome 2, complete sequence
AT3G48510	5.437865684	5.96E-42	Arabidopsis thaliana chromosome 3, complete sequence
AT5G46910	1.394097237	6.31E-42	Arabidopsis thaliana transcription factor jumonji (jmj) family protein / zinc finger (C5HC2 type) family protein mRNA, complete cds
AT5G13490	1.209046994	6.56E-42	Arabidopsis thaliana ADP/ATP carrier protein 2 mRNA, complete cds
AT2G34650	1.735719644	9.04E-42	Arabidopsis thaliana protein serine/threonine kinase PINOID mRNA, complete cds
AT3G22275	3.548048602	9.33E-42	Arabidopsis thaliana uncharacterized protein mRNA, complete cds
AT5G24740	1.210382754	1.13E-41	Arabidopsis thaliana uncharacterized protein mRNA, complete cds
AT2G39020	1.194727681	1.16E-41	Arabidopsis thaliana chromosome 2, complete sequence
AT4G23140	1.654506243	1.29E-41	Arabidopsis thaliana cysteine-rich receptor-like protein kinase 6 mRNA, complete cds
AT1G53180	1.428876901	1.58E-41	Arabidopsis thaliana uncharacterized protein mRNA, complete cds
AT3G28210	3.276873808	1.92E-41	Arabidopsis thaliana zinc finger (AN1-like) family protein mRNA, complete cds
AT3G15250	2.882897924	2.28E-41	Arabidopsis thaliana chromosome 3, complete sequence
AT2G27690	2.330363183	2.33E-41	Arabidopsis thaliana chromosome 2, complete sequence
AT1G22160	1.496556305	3.55E-41	Arabidopsis thaliana uncharacterized protein mRNA, complete cds
AT3G55880	1.288819412	3.71E-41	Arabidopsis thaliana protein SULPHATE

			UTILIZATION EFFICIENCY 4 mRNA, complete cds
AT5G43380	1.991967221	5.71E-41	Arabidopsis thaliana type one serine/threonine protein phosphatase 6 mRNA, complete cds
AT4G31160	1.306876345	7.37E-41	Arabidopsis thaliana DDB1- and CUL4-associated factor-1 mRNA, complete cds
AT2G44940	1.348559143	7.47E-41	Arabidopsis thaliana chromosome 2, complete sequence
AT1G49530	2.534126226	7.61E-41	Arabidopsis thaliana chromosome 1 sequence
AT4G35190	3.652878575	7.73E-41	Arabidopsis thaliana cytokinin riboside 5'-monophosphate phosphoribohydrolase LOG5 mRNA, complete cds
AT2G44070	8.660258106	1.09E-40	Arabidopsis thaliana NagB/RpiA/CoA transferase-like superfamily protein mRNA, complete cds
AT5G10410	2.017841332	1.15E-40	Arabidopsis thaliana ENTH/ANTH/VHS superfamily protein mRNA, complete cds
AT2G43550	1.57480133	1.22E-40	Arabidopsis thaliana defensin-like protein 197 mRNA, complete cds
AT2G29490	2.5288705	1.30E-40	Arabidopsis thaliana glutathione S-transferase tau 1 mRNA, complete cds
AT3G50060	1.119992954	1.64E-40	Arabidopsis thaliana chromosome 3, complete sequence
AT1G24300	1.24419208	1.71E-40	Arabidopsis thaliana GYF domain-containing protein mRNA, complete cds
AT5G61890	6.394276416	1.82E-40	Arabidopsis thaliana ethylene-responsive transcription factor ERF114 mRNA, complete cds
AT1G76380	1.604358474	1.92E-40	Arabidopsis thaliana DNA-binding bromodomain-containing protein mRNA, complete cds
AT5G01300	7.480176489	2.40E-40	Arabidopsis thaliana putative phosphatidylethanolamine-binding protein mRNA, complete cds
AT1G22060	1.401595735	2.54E-40	Arabidopsis thaliana uncharacterized protein mRNA, complete cds
AT1G72680	1.483500153	2.57E-40	Arabidopsis thaliana cinnamyl-alcohol dehydrogenase mRNA, complete cds
AT2G36220	1.25246747	3.00E-40	Arabidopsis thaliana chromosome 2,

			complete sequence
AT2G21560	1.007375759	3.75E-40	Arabidopsis thaliana chromosome 2, complete sequence
AT5G42800	1.435668128	4.48E-40	Arabidopsis thaliana dihydroflavonol-4-reductase mRNA, complete cds
AT1G69010	1.15328983	4.76E-40	Arabidopsis thaliana transcription factor BIM2 mRNA, complete cds
AT1G15430	1.568751739	4.83E-40	Arabidopsis thaliana chromosome 1 sequence
AT2G35070	8.630510762	5.04E-40	Arabidopsis thaliana uncharacterized protein mRNA, complete cds
AT5G58690	1.8439144	5.41E-40	Arabidopsis thaliana phosphoinositide phospholipase C 5 mRNA, complete cds
AT3G01460	1.584524557	6.58E-40	Arabidopsis thaliana methyl-CPG-binding domain 9 mRNA, complete cds
AT2G45910	1.119368495	6.92E-40	Arabidopsis thaliana U-box domain-containing protein 33 mRNA, complete cds
AT3G62100	3.860808382	7.05E-40	Arabidopsis thaliana auxin-responsive protein IAA30 mRNA, complete cds
AT3G56080	1.434710872	7.75E-40	Arabidopsis thaliana probable methyltransferase PMT22 mRNA, complete cds
AT4G15480	2.458850709	8.36E-40	Arabidopsis thaliana chromosome 4 sequence
AT4G38730	1.530975089	8.39E-40	Arabidopsis thaliana uncharacterized protein mRNA, complete cds
AT5G16630	1.394581586	1.62E-39	Arabidopsis thaliana DNA repair protein Rad4 mRNA, complete cds
AT2G20320	1.394581586	1.62E-39	Arabidopsis thaliana DENN (AEX-3) domain-containing protein mRNA, complete cds
AT1G67300	1.11045856	2.36E-39	Arabidopsis thaliana putative plastidic glucose transporter 2 mRNA, complete cds
AT5G54230	2.168606658	2.51E-39	Arabidopsis thaliana putative transcription factor MYB49 mRNA, complete cds
AT4G17140	1.133455351	2.83E-39	Arabidopsis thaliana pleckstrin homology (PH) domain-containing protein mRNA, complete cds
AT3G20500	1.168197651	3.05E-39	Arabidopsis thaliana purple acid

			phosphatase 18 mRNA, complete cds
AT3G24090	2.094307573	3.51E-39	Arabidopsis thaliana putative glucosamine-fructose-6-phosphate aminotransferase mRNA, complete cds
AT5G17910	1.174004282	4.74E-39	Arabidopsis thaliana uncharacterized protein mRNA, complete cds
AT2G24100	1.097387193	4.92E-39	Arabidopsis thaliana uncharacterized protein mRNA, complete cds
AT1G52080	2.071700549	5.33E-39	Arabidopsis thaliana uncharacterized protein mRNA, complete cds
AT4G15120	3.30428253	5.97E-39	Arabidopsis thaliana chromosome 4 sequence
AT4G25390	1.600327757	8.17E-39	Arabidopsis thaliana chromosome 4 sequence
AT4G15260	1.413935667	8.19E-39	Arabidopsis thaliana chromosome 4 sequence
AT2G41700	1.070380114	8.91E-39	Arabidopsis thaliana ABC transporter A family member 1 mRNA, complete cds
AT5G28520	7.411870476	9.32E-39	Arabidopsis thaliana mannose-binding lectin-like protein mRNA, complete cds
AT3G47295	2.327812139	9.95E-39	Arabidopsis thaliana uncharacterized protein mRNA, complete cds
AT5G59490	2.272839482	1.11E-38	Arabidopsis thaliana haloacid dehalogenase-like hydrolase (HAD) superfamily protein mRNA, complete cds
AT3G02875	1.500114125	1.12E-38	Arabidopsis thaliana IAA-amino acid hydrolase ILR1 mRNA, complete cds
AT5G01400	1.221095819	1.55E-38	Arabidopsis thaliana Symplekin/Pta1-like protein mRNA, complete cds
AT3G04220	2.294720014	1.77E-38	Arabidopsis thaliana TIR-NBS-LRR class disease resistance protein mRNA, complete cds
AT5G64230	2.036935131	3.39E-38	Arabidopsis thaliana uncharacterized protein mRNA, complete cds
AT1G54570	1.498653802	3.39E-38	Arabidopsis thaliana phytyl ester synthase 1 mRNA, complete cds
AT5G39670	1.667290611	4.58E-38	Arabidopsis thaliana chromosome 5 sequence
AT3G46650	1.456974507	4.61E-38	Arabidopsis thaliana UDP-glycosyltransferase-like protein mRNA, complete cds
AT1G67140	1.062188527	5.04E-38	Arabidopsis thaliana protein SWEETIE mRNA, complete cds

AT1G20650	1.093201237	5.64E-38	Arabidopsis thaliana protein ALTERED SEED GERMINATION 5 mRNA, complete cds
AT3G48350	1.711960576	6.76E-38	Arabidopsis thaliana KDEL-tailed cysteine endopeptidase CEP3 mRNA, complete cds
AT1G10580	1.139775024	6.93E-38	Arabidopsis thaliana transducin/WD40 repeat-like superfamily protein mRNA, complete cds
AT2G14290	8.529364039	7.65E-38	Arabidopsis thaliana chromosome 2, complete sequence
AT5G17850	1.76226935	8.98E-38	Arabidopsis thaliana chromosome 5 sequence
AT5G54280	1.091576792	9.32E-38	Arabidopsis thaliana myosin 2 mRNA, complete cds
AT1G24330	2.636509503	1.53E-37	Arabidopsis thaliana U-box domain-containing protein 6 mRNA, complete cds
AT1G01010	1.864744796	2.24E-37	Arabidopsis thaliana NAC domain-containing protein 1 mRNA, complete cds
AT1G06000	1.721505294	2.61E-37	Arabidopsis thaliana chromosome 1 sequence
AT1G61140	1.199148392	2.71E-37	Arabidopsis thaliana protein EMBRYO SAC DEVELOPMENT ARREST 16 mRNA, complete cds
AT1G03840	2.041088613	4.35E-37	Arabidopsis thaliana zinc finger protein MAGPIE mRNA, complete cds
AT1G03910	1.023279098	5.75E-37	Arabidopsis thaliana uncharacterized protein mRNA, complete cds
AT4G33950	1.623953046	6.98E-37	Arabidopsis thaliana calcium-independent ABA-activated protein kinase mRNA, complete cds
AT3G06760	1.109344912	7.79E-37	Arabidopsis thaliana protein dehydration-INDUCED 19-4 mRNA, complete cds
AT2G29090	3.293475775	8.16E-37	Arabidopsis thaliana abscisic acid 8'-hydroxylase 2 mRNA, complete cds
AT3G48450	2.952438857	8.39E-37	Arabidopsis thaliana RPM1-interacting protein 4 (RIN4) mRNA, complete cds
AT3G45830	1.49620967	1.10E-36	Arabidopsis thaliana uncharacterized protein mRNA, complete cds
AT5G05270	1.125232481	1.34E-36	Arabidopsis thaliana Chalcone-flavanone isomerase family protein mRNA,

			complete cds
AT3G09910	2.33565091	1.51E-36	Arabidopsis thaliana RAB GTPase-like protein C2B mRNA, complete cds
AT2G24130	3.186029485	1.59E-36	Arabidopsis thaliana putative leucine-rich repeat receptor-like serine/threonine-protein kinase mRNA, complete cds
AT2G15880	2.140266919	1.67E-36	Arabidopsis thaliana pollen-specific leucine-rich repeat extensin-like protein 3 mRNA, complete cds
AT5G22430	2.262778978	1.72E-36	Arabidopsis thaliana pollen Ole e 1 allergen and extensin family protein mRNA, complete cds
AT1G21110	1.288483237	1.93E-36	Arabidopsis thaliana O-methyltransferase family protein mRNA, complete cds
AT4G32620	1.224230164	2.80E-36	Arabidopsis thaliana Enhancer of polycomb-like transcription factor protein mRNA, complete cds
AT1G45160	1.038853609	3.02E-36	Arabidopsis thaliana protein kinase mRNA, complete cds
AT3G11960	1.032312434	3.30E-36	Arabidopsis thaliana Cleavage and polyadenylation specificity factor (CPSF) A subunit protein mRNA, complete cds
AT4G29790	1.171264663	3.50E-36	Arabidopsis thaliana uncharacterized protein mRNA, complete cds
AT5G63350	4.679754281	4.35E-36	Arabidopsis thaliana chromosome 5 sequence
AT5G24350	1.136440843	5.97E-36	Arabidopsis thaliana uncharacterized protein mRNA, complete cds
AT1G31550	1.725835721	7.29E-36	Arabidopsis thaliana GDSL esterase/lipase mRNA, complete cds
AT2G42440	4.670039126	7.88E-36	Arabidopsis thaliana LOB domain-containing protein 17 mRNA, complete cds
AT5G40790	7.274366952	9.80E-36	Arabidopsis thaliana chromosome 5 sequence
AT4G10930	1.925770907	1.05E-35	Arabidopsis thaliana uncharacterized protein mRNA, complete cds
AT3G13672	3.313320586	1.07E-35	Arabidopsis thaliana seven in absentia (SINA) domain-containing protein mRNA, complete cds
AT3G49620	2.464500811	1.25E-35	Arabidopsis thaliana 2-oxoacid-dependent dioxygenase-like protein DIN11 mRNA, complete cds

AT1G18710	2.44515572	1.34E-35	Arabidopsis thaliana myb domain protein 47 mRNA, complete cds
AT1G79900	3.720147975	1.84E-35	Arabidopsis thaliana mitochondrial arginine transporter BAC2 mRNA, complete cds
AT5G52660	1.252535357	2.25E-35	Arabidopsis thaliana myb family transcription factor mRNA, complete cds
AT1G10560	2.040856566	2.49E-35	Arabidopsis thaliana chromosome 1 sequence
AT4G19460	2.624169235	2.75E-35	Arabidopsis thaliana chromosome 4 sequence
AT2G41170	1.23917666	3.07E-35	Arabidopsis thaliana F-box protein mRNA, complete cds
AT5G66070	1.501139859	3.25E-35	Arabidopsis thaliana RING/U-box superfamily protein mRNA, complete cds
AT1G18570	1.009682809	3.34E-35	Arabidopsis thaliana myb domain protein 51 mRNA, complete cds
AT5G44350	2.205094117	3.65E-35	Arabidopsis thaliana chromosome 5 sequence
AT4G29380	1.069395003	4.23E-35	Arabidopsis thaliana phosphoinositide-3-kinase, regulatory subunit 4, p150 mRNA, complete cds
AT5G44180	1.000560807	4.42E-35	Arabidopsis thaliana protein RINGLET2 mRNA, complete cds
AT1G67120	1.844635567	4.91E-35	Arabidopsis thaliana protein MIDASIN1 mRNA, complete cds
AT1G07500	6.164489334	5.65E-35	Arabidopsis thaliana uncharacterized protein mRNA, complete cds
AT2G20880	1.901364672	6.24E-35	Arabidopsis thaliana chromosome 2, complete sequence
AT2G47485	1.901364672	6.24E-35	Arabidopsis thaliana chromosome 2, complete sequence
AT3G01970	3.176792795	1.21E-34	Arabidopsis thaliana WRKY DNA-binding protein 45 mRNA, complete cds
AT2G44578	4.428017898	1.28E-34	Arabidopsis thaliana chromosome 2, complete sequence
AT1G07480	1.149259611	1.92E-34	Arabidopsis thaliana transcription factor IIA, alpha/beta subunit mRNA, complete cds
AT3G20720	1.180008582	2.02E-34	Arabidopsis thaliana uncharacterized protein mRNA, complete cds
AT2G04080	1.415987229	2.23E-34	Arabidopsis thaliana MATE efflux family

			protein mRNA, complete cds
AT5G15190	2.882049529	2.42E-34	Arabidopsis thaliana chromosome 5 sequence
AT1G23230	1.225348806	2.44E-34	Arabidopsis thaliana mediator of RNA polymerase II transcription subunit 23 mRNA, complete cds
AT1G23200	2.296285308	2.54E-34	Arabidopsis thaliana probable pectinesterase/pectinesterase inhibitor 6 mRNA, complete cds
AT1G52560	3.522361958	2.60E-34	Arabidopsis thaliana heat shock protein 26.5 mRNA, complete cds
AT1G42540	1.180864226	2.92E-34	Arabidopsis thaliana glutamate receptor 3.3 mRNA, complete cds
AT4G11330	1.142121521	2.94E-34	Arabidopsis thaliana mitogen-activated protein kinase 5 mRNA, complete cds
AT5G53750	1.709151211	3.41E-34	Arabidopsis thaliana CBS domain-containing protein mRNA, complete cds
AT1G35115.1	1.80613096	3.51E-34	Arabidopsis thaliana chromosome 1 sequence
AT3G19190	1.084437275	4.22E-34	Arabidopsis thaliana protein autophagy 2 mRNA, complete cds
AT2G22760	7.195295381	4.24E-34	Arabidopsis thaliana transcription factor bHLH19 mRNA, complete cds
AT5G14730	2.442438414	4.71E-34	Arabidopsis thaliana chromosome 5 sequence
AT1G63420	1.058537243	4.73E-34	Arabidopsis thaliana uncharacterized protein mRNA, complete cds
AT1G70300	1.133011103	6.19E-34	Arabidopsis thaliana potassium transporter 6 mRNA, complete cds
AT2G23790	1.003679535	6.33E-34	Arabidopsis thaliana uncharacterized protein mRNA, complete cds
AT4G39670	2.173500992	7.38E-34	Arabidopsis thaliana chromosome 4 sequence
AT2G44080	1.634149339	8.36E-34	Arabidopsis thaliana ARGOS-like protein mRNA, complete cds
AT5G59130	1.752028582	9.81E-34	Arabidopsis thaliana Subtilase family protein mRNA, complete cds
AT5G01270	1.006531203	1.07E-33	Arabidopsis thaliana RNA polymerase II C-terminal domain phosphatase-like 2 mRNA, complete cds
AT1G35910	2.143580344	1.18E-33	Arabidopsis thaliana probable trehalose-phosphate phosphatase D mRNA, complete cds

AT2G35350	1.660725375	1.21E-33	Arabidopsis thaliana protein phosphatase 2C 29 mRNA, complete cds
AT3G51090	1.530975089	1.26E-33	Arabidopsis thaliana uncharacterized protein mRNA, complete cds
AT5G12340	1.896244318	1.28E-33	Arabidopsis thaliana chromosome 5 sequence
AT5G24870	1.158140088	1.33E-33	Arabidopsis thaliana RING/U-box superfamily protein mRNA, complete cds
AT5G42930	2.355650921	1.69E-33	Arabidopsis thaliana lipase class 3-like protein mRNA, complete cds
AT3G15120	1.513256983	2.67E-33	Arabidopsis thaliana P-loop containing nucleoside triphosphate hydrolases superfamily protein mRNA, complete cds
AT3G51430	1.174132467	2.79E-33	Arabidopsis thaliana strictosidine synthase-like 5 mRNA, complete cds
AT5G28400	2.315637425	3.05E-33	Arabidopsis thaliana uncharacterized protein mRNA, complete cds
AT5G56190	1.181235361	4.32E-33	Arabidopsis thaliana transducin/WD40 domain-containing protein mRNA, complete cds
AT3G18777	1.948872242	6.00E-33	Arabidopsis thaliana chromosome 3, complete sequence
AT3G16800	1.155521103	6.47E-33	Arabidopsis thaliana putative protein phosphatase 2C mRNA, complete cds
AT5G23850	1.325656181	6.55E-33	Arabidopsis thaliana uncharacterized protein mRNA, complete cds
AT1G24145	2.256445044	9.10E-33	Arabidopsis thaliana uncharacterized protein mRNA, complete cds
AT3G50760	1.944102998	9.60E-33	Arabidopsis thaliana chromosome 3, complete sequence
AT2G30830	8.264716782	1.16E-32	Arabidopsis thaliana 2-oxoglutarate dependent oxygenase-like protein mRNA, complete cds
AT1G17580	1.039931012	1.21E-32	Arabidopsis thaliana myosin 1 mRNA, complete cds
AT2G40435	3.621232949	1.30E-32	Arabidopsis thaliana uncharacterized protein mRNA, complete cds
AT3G55640	1.419146487	1.62E-32	Arabidopsis thaliana mitochondrial substrate carrier family protein mRNA, complete cds
AT5G45820	3.805285163	1.74E-32	Arabidopsis thaliana chromosome 5 sequence

AT5G11530	1.42281939	2.26E-32	Arabidopsis thaliana embryonic flower 1 mRNA, complete cds
AT3G60160	1.619283507	2.39E-32	Arabidopsis thaliana multidrug resistance-associated protein 9 mRNA, complete cds
AT3G44200	1.382426698	2.51E-32	Arabidopsis thaliana serine/threonine-protein kinase Nek5 mRNA, complete cds
AT1G75450	1.670366751	2.54E-32	Arabidopsis thaliana cytokinin dehydrogenase 5 mRNA, complete cds
AT1G01360	1.039872077	2.56E-32	Arabidopsis thaliana regulatory component of ABA receptor 1 mRNA, complete cds
AT1G53170	1.027075601	2.71E-32	Arabidopsis thaliana chromosome 1 sequence
AT1G24600	3.259327552	2.75E-32	Arabidopsis thaliana chromosome 1 sequence
AT5G01100	1.314664165	2.81E-32	Arabidopsis thaliana O-fucosyltransferase family protein mRNA, complete cds
AT3G59080	1.082595248	3.31E-32	Arabidopsis thaliana chromosome 3, complete sequence
AT3G53810	1.264472664	3.44E-32	Arabidopsis thaliana chromosome 3, complete sequence
AT1G30640	1.228614363	3.68E-32	Arabidopsis thaliana protein kinase family protein mRNA, complete cds
AT4G34060	2.815622167	3.81E-32	Arabidopsis thaliana DEMETER-like protein 3 mRNA, complete cds
AT3G47510	1.517949956	3.89E-32	Arabidopsis thaliana uncharacterized protein mRNA, complete cds
AT5G10100	2.46935597	4.11E-32	Arabidopsis thaliana probable trehalose-phosphate phosphatase I mRNA, complete cds
AT1G16370	1.937939287	4.22E-32	Arabidopsis thaliana chromosome 1 sequence
AT5G28646	2.582982392	4.31E-32	Arabidopsis thaliana protein WAVE-DAMPENED 2 mRNA, complete cds
AT5G24110	1.874067793	6.10E-32	Arabidopsis thaliana WRKY DNA-binding protein 30 mRNA, complete cds
AT1G08620	1.067790672	6.57E-32	Arabidopsis thaliana transcription factor PKDM7D mRNA, complete cds
AT1G59640	2.218384858	6.97E-32	Arabidopsis thaliana transcription factor

			BPE mRNA, complete cds
AT1G67920	2.876490004	7.77E-32	Arabidopsis thaliana chromosome 1 sequence
AT1G52880	1.472854598	8.86E-32	Arabidopsis thaliana NAC domain-containing protein 18 mRNA, complete cds
AT4G22610	3.037327755	9.24E-32	Arabidopsis thaliana chromosome 4 sequence
AT4G20000	2.361190093	9.47E-32	Arabidopsis thaliana chromosome 4 sequence
AT4G29690	1.679838474	1.00E-31	Arabidopsis thaliana chromosome 4 sequence
AT5G13700	2.82981957	1.68E-31	Arabidopsis thaliana polyamine oxidase 1 mRNA, complete cds
AT5G02270	1.181609811	2.24E-31	Arabidopsis thaliana ABC transporter I family member 20 mRNA, complete cds
AT4G33550	3.34932901	2.55E-31	Arabidopsis thaliana chromosome 4 sequence
AT3G29000	1.943668647	3.42E-31	Arabidopsis thaliana chromosome 3, complete sequence
AT2G22200	2.024486646	3.47E-31	Arabidopsis thaliana chromosome 2, complete sequence
AT1G08060	1.514760901	3.57E-31	Arabidopsis thaliana helicase protein MOM1 mRNA, complete cds
AT4G37770	3.280013515	3.64E-31	Arabidopsis thaliana 1-aminocyclopropane-1-carboxylate synthase 8 mRNA, complete cds
AT2G23150	1.603758035	3.88E-31	Arabidopsis thaliana metal transporter Nramp3 mRNA, complete cds
AT4G38400	1.961387814	3.92E-31	Arabidopsis thaliana expansin-like A2 mRNA, complete cds
AT2G41312	2.660258106	3.92E-31	Arabidopsis thaliana Full-length cDNA Complete sequence from clone GSLTSIL38ZE01 of Silique of strain col-0 of Arabidopsis thaliana (thale cress)
AT1G52827	2.361050087	4.32E-31	Arabidopsis thaliana cadmium tolerance 1 mRNA, complete cds
AT1G72700	1.020853734	4.32E-31	Arabidopsis thaliana putative phospholipid-transporting ATPase 5 mRNA, complete cds
AT1G48840	1.238567434	4.67E-31	Arabidopsis thaliana uncharacterized protein mRNA, complete cds
AT5G52230	1.99908895	5.03E-31	Arabidopsis thaliana methyl-CPG-binding domain protein 13

			mRNA, complete cds
AT1G06180	1.626563578	5.10E-31	Arabidopsis thaliana myb domain protein 13 mRNA, complete cds
AT1G66540	1.646720989	5.73E-31	Arabidopsis thaliana cytochrome P450 superfamily protein mRNA, complete cds
AT2G46240	2.161025479	8.46E-31	Arabidopsis thaliana BCL-2-associated athanogene 6 mRNA, complete cds
AT5G15540	1.171959641	8.55E-31	Arabidopsis thaliana sister-chromatid cohesion protein 2 mRNA, complete cds
AT1G74080	3.206540138	9.11E-31	Arabidopsis thaliana myb domain protein 122 mRNA, complete cds
AT4G01550	1.72448691	9.20E-31	Arabidopsis thaliana NAC transcription factor mRNA, complete cds
AT1G48540	1.031798851	9.76E-31	Arabidopsis thaliana Outer arm dynein light chain 1 protein mRNA, complete cds
AT5G59050	1.1691033	9.91E-31	Arabidopsis thaliana uncharacterized protein mRNA, complete cds
AT3G60120	4.988062767	1.11E-30	Arabidopsis thaliana beta glucosidase 27 mRNA, complete cds
AT5G01880	3.045548261	1.25E-30	Arabidopsis thaliana chromosome 5 sequence
AT3G04070	2.734461726	1.29E-30	Arabidopsis thaliana NAC domain containing protein 47 mRNA, complete cds
AT4G24340	1.988558283	1.30E-30	Arabidopsis thaliana phosphorylase family protein mRNA, complete cds
AT1G55970	1.720147975	1.42E-30	Arabidopsis thaliana histone acetyltransferase of the CBP family 4 mRNA, complete cds
AT1G61690	1.09460908	1.75E-30	Arabidopsis thaliana phosphoinositide binding protein mRNA, complete cds
AT1G64570	1.087496443	2.14E-30	Arabidopsis thaliana DUO pollen 3 protein mRNA, complete cds
AT3G13620	1.515033545	2.20E-30	Arabidopsis thaliana POLYAMINE UPTAKE TRANSPORTER 4 mRNA, complete cds
AT1G02220	1.430770106	2.28E-30	Arabidopsis thaliana NAC domain-containing protein 3 mRNA, complete cds
AT1G01830	1.020185829	2.80E-30	Arabidopsis thaliana armadillo/beta-catenin-like repeat-containing protein mRNA,

			complete cds
AT1G76070	1.570074908	3.04E-30	Arabidopsis thaliana chromosome 1 sequence
AT3G21430	1.289171361	3.59E-30	Arabidopsis thaliana protein ALWAYS EARLY 3 mRNA, complete cds
AT1G14120	1.987727607	3.59E-30	Arabidopsis thaliana 2-oxoglutarate (2OG) and Fe(II)-dependent oxygenase-like protein mRNA, complete cds
AT2G42560	8.122363858	3.66E-30	Arabidopsis thaliana late embryogenesis abundant domain-containing protein mRNA, complete cds
AT1G69920	6.988062767	3.86E-30	Arabidopsis thaliana glutathione S-transferase TAU 12 mRNA, complete cds
AT5G22860	1.328683873	4.78E-30	Arabidopsis thaliana Serine carboxypeptidase S28 family protein mRNA, complete cds
AT1G72390	1.160209244	5.41E-30	Arabidopsis thaliana protein PHYTOCHROME-DEPENDENT LATE-FLOWERING mRNA, complete cds
AT1G69270	1.083369727	5.83E-30	Arabidopsis thaliana chromosome 1 sequence
AT3G12810	1.44347174	6.09E-30	Arabidopsis thaliana photoperiod-independent early flowering 1 protein mRNA, complete cds
AT1G34630	1.064913586	6.20E-30	Arabidopsis thaliana uncharacterized protein mRNA, complete cds
AT3G05580	1.083486744	7.03E-30	Arabidopsis thaliana serine/threonine-protein phosphatase PP1 isozyme 9 mRNA, complete cds
AT2G34850	3.437865684	7.43E-30	Arabidopsis thaliana putative UDP-arabinose 4-epimerase 2 mRNA, complete cds
AT5G41740	1.078766038	7.50E-30	Arabidopsis thaliana TIR-NBS-LRR class disease resistance protein mRNA, complete cds
AT1G08250	1.033339204	8.22E-30	Arabidopsis thaliana chromosome 1 sequence
AT4G17895	1.486120853	8.68E-30	Arabidopsis thaliana ubiquitin-specific protease 20 mRNA, complete cds
AT5G52870	1.119671516	8.74E-30	Arabidopsis thaliana membrane-associated kinase regulator

			family protein mRNA, complete cds
AT3G03170	3.515033545	9.01E-30	Arabidopsis thaliana uncharacterized protein mRNA, complete cds
AT3G62740	1.647434065	9.29E-30	Arabidopsis thaliana beta glucosidase 7 mRNA, complete cds
AT4G22214	3.224871961	1.21E-29	Arabidopsis thaliana defensin-like protein 99 mRNA, complete cds
AT4G15610	1.149640002	1.40E-29	Arabidopsis thaliana uncharacterized protein mRNA, complete cds
AT2G19710	1.44664657	1.46E-29	Arabidopsis thaliana Vps4 regulator of MVB pathway mRNA, complete cds
AT3G62090	1.911362914	1.55E-29	Arabidopsis thaliana transcription factor PIF6 mRNA, complete cds
AT5G53710	6.952438857	1.67E-29	Arabidopsis thaliana chromosome 5 sequence
AT3G01830	3.161025479	1.67E-29	Arabidopsis thaliana chromosome 3, complete sequence
AT1G73210	1.897702793	2.28E-29	Arabidopsis thaliana uncharacterized protein mRNA, complete cds
AT5G12400	1.079389415	2.30E-29	Arabidopsis thaliana PHD-finger and DNA binding domain-containing protein mRNA, complete cds
AT5G19100	2.388435975	2.31E-29	Arabidopsis thaliana chromosome 5 sequence
AT3G05660	3.418102429	2.42E-29	Arabidopsis thaliana receptor like protein 33 mRNA, complete cds
AT3G54820	1.138820416	2.96E-29	Arabidopsis thaliana putative aquaporin PIP2-5 mRNA, complete cds
AT1G08600	1.258411619	3.57E-29	Arabidopsis thaliana protein ATRX mRNA, complete cds
AT4G10590	1.002362328	4.06E-29	Arabidopsis thaliana ubiquitin-specific protease 10 mRNA, complete cds
AT2G15780	8.056775517	4.49E-29	Arabidopsis thaliana Cupredoxin superfamily protein mRNA, complete cds
AT4G29440	1.504036017	4.51E-29	Arabidopsis thaliana regulator of Vps4 activity protein mRNA, complete cds
AT2G31180	2.943668647	4.96E-29	Arabidopsis thaliana myb domain protein 14 mRNA, complete cds
AT4G34131	2.022828185	5.49E-29	Arabidopsis thaliana chromosome 4 sequence
AT4G26750	1.349833395	6.48E-29	Arabidopsis thaliana LYST INTERACTING PROTEIN 5 mRNA, complete cds

AT1G04310	1.754316964	7.14E-29	Arabidopsis thaliana ethylene response sensor 2 mRNA, complete cds
AT3G11020	3.66293224	7.53E-29	Arabidopsis thaliana dehydration-responsive element-binding protein 2B mRNA, complete cds
AT4G33040	2.225254284	8.08E-29	Arabidopsis thaliana chromosome 4 sequence
AT1G02990	1.515627124	8.37E-29	Arabidopsis thaliana uncharacterized protein mRNA, complete cds
AT3G14172	1.141160173	1.09E-28	Arabidopsis thaliana uncharacterized protein mRNA, complete cds
AT1G51440	1.413618138	1.13E-28	Arabidopsis thaliana chromosome 1 sequence
AT4G25410	3.318566756	1.14E-28	Arabidopsis thaliana transcription factor bHLH126 mRNA, complete cds
AT3G19270	2.607051789	1.40E-28	Arabidopsis thaliana abscisic acid 8'-hydroxylase 4 mRNA, complete cds
AT3G54000	1.585358726	1.77E-28	Arabidopsis thaliana uncharacterized protein mRNA, complete cds
AT2G38250	3.308582667	2.05E-28	Arabidopsis thaliana trihelix transcription factor GT-3b mRNA, complete cds
AT4G15200	8.011332546	2.42E-28	Arabidopsis thaliana formin 3 mRNA, complete cds
AT5G67470	1.191225717	2.67E-28	Arabidopsis thaliana formin-like protein 6 mRNA, complete cds
AT5G48110	1.229189256	3.99E-28	Arabidopsis thaliana terpenoid synthase 20 mRNA, complete cds
AT3G13650	1.016147345	4.89E-28	Arabidopsis thaliana chromosome 3, complete sequence
AT1G19860	1.010078918	6.82E-28	Arabidopsis thaliana zinc finger CCCH domain-containing protein 6 mRNA, complete cds
AT5G21960	3.526674951	6.91E-28	Arabidopsis thaliana chromosome 5 sequence
AT5G52760	1.921889794	1.03E-27	Arabidopsis thaliana copper transport family protein mRNA, complete cds
AT1G18360	1.123550773	1.06E-27	Arabidopsis thaliana alpha/beta-Hydrolases superfamily protein mRNA, complete cds
AT5G05340	3.608484456	1.49E-27	Arabidopsis thaliana peroxidase 52 mRNA, complete cds
AT4G24390	1.095301297	1.57E-27	Arabidopsis thaliana auxin signaling F-BOX 4 mRNA, complete cds
AT3G55720	2.291708849	1.58E-27	Arabidopsis thaliana uncharacterized

			protein mRNA, complete cds
AT1G18810	1.106731778	1.96E-27	Arabidopsis thaliana chromosome 1 sequence
AT4G23060	1.281685774	2.21E-27	Arabidopsis thaliana protein IQ-domain 22 mRNA, complete cds
AT3G55240	3.964411499	2.25E-27	Arabidopsis thaliana uncharacterized protein mRNA, complete cds
AT5G66650	1.965694954	2.36E-27	Arabidopsis thaliana uncharacterized protein mRNA, complete cds
AT1G35660	1.694205437	2.56E-27	Arabidopsis thaliana uncharacterized protein mRNA, complete cds
AT2G44200	1.150820304	2.66E-27	Arabidopsis thaliana pre-mRNA splicing factor domain-containing protein mRNA, complete cds
AT3G51750	2.045548261	3.49E-27	Arabidopsis thaliana uncharacterized protein mRNA, complete cds
AT5G52280	1.11927865	3.52E-27	Arabidopsis thaliana Myosin heavy chain-related protein mRNA, complete cds
AT5G67340	1.424809531	3.83E-27	Arabidopsis thaliana ARM repeat superfamily protein mRNA, complete cds
AT3G10490	1.034268337	4.26E-27	Arabidopsis thaliana NAC domain containing protein 52 mRNA, complete cds
AT1G20390.1	2.55078357	6.22E-27	Arabidopsis thaliana chromosome 1 sequence
AT1G32960	3.482953574	7.42E-27	Arabidopsis thaliana Subtilase family protein SBT3.3 mRNA, complete cds
AT4G01540	1.497845379	7.77E-27	Arabidopsis thaliana NAC with transmembrane motif1 mRNA, complete cds
AT3G03310	1.036388262	8.02E-27	Arabidopsis thaliana phospholipase A(1) LCAT3 mRNA, complete cds
AT4G35550	1.002607739	8.92E-27	Arabidopsis thaliana WUSCHEL-related homeobox 13 mRNA, complete cds
AT5G64900	1.937792081	9.05E-27	Arabidopsis thaliana precursor of peptide 1 mRNA, complete cds
AT5G40540	1.263728431	9.11E-27	Arabidopsis thaliana protein kinase family protein mRNA, complete cds
AT5G22300	2.390683747	1.00E-26	Arabidopsis thaliana bifunctional nitrilase/nitrile hydratase NIT4 mRNA, complete cds
AT3G56620	1.15735216	1.02E-26	Arabidopsis thaliana nodulin MtN21-like

			transporter family protein mRNA, complete cds
AT3G49580	6.787015248	1.02E-26	Arabidopsis thaliana protein RESPONSE TO LOW SULFUR 1 mRNA, complete cds
AT5G58670	1.164750968	1.10E-26	Arabidopsis thaliana phosphoinositide phospholipase C 1 mRNA, complete cds
AT4G36740	5.174831278	1.18E-26	Arabidopsis thaliana homeobox protein 40 mRNA, complete cds
AT4G23680	1.861348252	1.29E-26	Arabidopsis thaliana polyketide cyclase/dehydrase and lipid transport superfamily protein mRNA, complete cds
AT1G54050	1.208809533	1.30E-26	Arabidopsis thaliana CIII heat shock protein 17.4 mRNA, complete cds
AT1G19850	1.109206899	1.53E-26	Arabidopsis thaliana auxin response factor 5 mRNA, complete cds
AT3G62690	1.300589876	1.56E-26	Arabidopsis thaliana chromosome 3, complete sequence
AT5G01040	1.121783847	1.88E-26	Arabidopsis thaliana laccase 8 mRNA, complete cds
AT4G25433	4.491804491	1.98E-26	Arabidopsis thaliana chromosome 4 sequence
AT5G02950	2.275282658	2.01E-26	Arabidopsis thaliana chromosome 5 sequence
AT4G27020	1.000019922	2.10E-26	Arabidopsis thaliana uncharacterized protein mRNA, complete cds
AT4G18140	1.09782554	2.15E-26	Arabidopsis thaliana SCP1-like small phosphatase 4b mRNA, complete cds
AT5G62520	2.987445307	2.23E-26	Arabidopsis thaliana probable inactive poly [ADP-ribose] polymerase SRO5 mRNA, complete cds
AT5G41590	3.097636773	2.31E-26	Arabidopsis thaliana uncharacterized protein mRNA, complete cds
AT2G34610	4.773468716	2.48E-26	Arabidopsis thaliana chromosome 2, complete sequence
AT3G51120	1.014232721	2.49E-26	Arabidopsis thaliana zinc finger CCCH domain-containing protein 44 mRNA, complete cds
AT5G26770	1.397930153	2.72E-26	Arabidopsis thaliana uncharacterized protein mRNA, complete cds
AT4G17785	4.071083354	2.80E-26	Arabidopsis thaliana transcription factor MYB39 mRNA, complete cds
AT2G04050	3.654357504	3.45E-26	Arabidopsis thaliana MATE efflux family

			protein mRNA, complete cds
AT4G34310	1.146275752	3.79E-26	Arabidopsis thaliana alpha/beta-Hydrolases superfamily protein mRNA, complete cds
AT2G24840	7.865727224	4.09E-26	Arabidopsis thaliana chromosome 2, complete sequence
AT1G60750	3.903529257	4.54E-26	Arabidopsis thaliana probable aldo-keto reductase 6 mRNA, complete cds
AT1G20030	2.10783254	5.20E-26	Arabidopsis thaliana pathogenesis-related thaumatin-like protein mRNA, complete cds
AT2G21590	1.589483494	6.20E-26	Arabidopsis thaliana glucose-1-phosphate adenylyltransferase large subunit mRNA, complete cds
AT4G35110	1.177772554	7.51E-26	Arabidopsis thaliana phospholipase-like (PEARLI 4) family protein mRNA, complete cds
AT5G07940	1.068388238	8.67E-26	Arabidopsis thaliana uncharacterized protein mRNA, complete cds
AT4G31110	1.908044738	9.38E-26	Arabidopsis thaliana wall-associated receptor kinase-like 18 mRNA, complete cds
AT3G06290	1.038340777	9.84E-26	Arabidopsis thaliana SAC3/GANP/Nin1/mts3/eIF-3 p25 family protein mRNA, complete cds
AT1G67530	1.190887625	9.95E-26	Arabidopsis thaliana ARM repeat superfamily protein mRNA, complete cds
AT3G55090	1.824779583	1.15E-25	Arabidopsis thaliana chromosome 3, complete sequence
AT5G10730	1.085076626	1.26E-25	Arabidopsis thaliana Rossmann-fold NAD(P)-binding domain-containing protein mRNA, complete cds
AT1G20670	1.118398827	1.32E-25	Arabidopsis thaliana DNA-binding bromodomain-containing protein mRNA, complete cds
AT2G34730	1.269054303	1.33E-25	Arabidopsis thaliana myosin heavy chain-related mRNA, complete cds
AT2G40770	1.705057715	1.43E-25	Arabidopsis thaliana RING-finger, DEAD-like helicase, PHD and SNF2 domain-containing protein mRNA, complete cds
AT2G27550	1.688121485	1.72E-25	Arabidopsis thaliana protein CENTRORADIALIS-like mRNA,

			complete cds
AT5G04240	1.060761355	1.82E-25	Arabidopsis thaliana probable lysine-specific demethylase ELF6 mRNA, complete cds
AT4G30790	1.035865708	1.94E-25	Arabidopsis thaliana uncharacterized protein mRNA, complete cds
AT4G13180	1.082610356	2.08E-25	Arabidopsis thaliana chromosome 4 sequence
AT5G58700	1.181245782	2.27E-25	Arabidopsis thaliana phosphoinositide phospholipase C 4 mRNA, complete cds
AT2G14960	2.278209018	2.51E-25	Arabidopsis thaliana putative indole-3-acetic acid-amido synthetase GH3.1 mRNA, complete cds
AT3G14360	1.988467166	2.64E-25	Arabidopsis thaliana alpha/beta-hydrolases mRNA, complete cds
AT5G01840	1.683556996	4.63E-25	Arabidopsis thaliana chromosome 5 sequence
AT5G01720	1.391323098	4.99E-25	Arabidopsis thaliana F-box/LRR-repeat protein 3 mRNA, complete cds
AT5G07310	5.645461104	5.30E-25	Arabidopsis thaliana ethylene-responsive transcription factor ERF115 mRNA, complete cds
AT1G16730	1.832439081	6.02E-25	Arabidopsis thaliana uncharacterized protein mRNA, complete cds
AT5G28830	1.324083761	6.39E-25	Arabidopsis thaliana calcium-binding EF hand family protein mRNA, complete cds
AT2G04110	1.172740278	7.46E-25	Arabidopsis thaliana chromosome 2, complete sequence
AT3G43430	1.318871927	7.72E-25	Arabidopsis thaliana chromosome 3, complete sequence
AT1G21670	1.08387638	1.12E-24	Arabidopsis thaliana chromosome 1 sequence
AT3G24880	1.651269322	1.14E-24	Arabidopsis thaliana Helicase/SANT-associated, DNA binding protein mRNA, complete cds
AT2G42760	1.084022409	1.35E-24	Arabidopsis thaliana chromosome 2, complete sequence
AT2G16367	5.61540387	1.63E-24	Arabidopsis thaliana chromosome 2, complete sequence
AT1G65920	1.846828669	1.99E-24	Arabidopsis thaliana Regulator of chromosome condensation (RCC1) family with FYVE zinc finger domain

			mRNA, complete cds
AT3G25010	1.865340287	2.39E-24	Arabidopsis thaliana chromosome 3, complete sequence
AT3G56320	1.82315584	2.59E-24	Arabidopsis thaliana PAP/OAS1 substrate-binding domain-containing protein mRNA, complete cds
AT5G46070	1.0280305	2.59E-24	Arabidopsis thaliana Guanylate-binding protein mRNA, complete cds
AT4G13550	1.000497426	2.64E-24	Arabidopsis thaliana putative triglyceride lipase mRNA, complete cds
AT1G70640	5.600137113	2.86E-24	Arabidopsis thaliana octicosapeptide/Phox/Bem1p (PB1) domain-containing protein mRNA, complete cds
AT4G36900	2.541974088	2.94E-24	Arabidopsis thaliana chromosome 4 sequence
AT5G42010	1.470980932	3.20E-24	Arabidopsis thaliana WD40 domain-containing protein mRNA, complete cds
AT1G43650	1.090812995	3.21E-24	Arabidopsis thaliana nodulin MtN21 /EamA-like transporter protein mRNA, complete cds
AT5G67180	1.979660326	3.22E-24	Arabidopsis thaliana target of early activation tagged 3 mRNA, complete cds
AT1G42980	3.966113794	3.44E-24	Arabidopsis thaliana formin-like protein 12 mRNA, complete cds
AT2G29480	4.367476356	4.37E-24	Arabidopsis thaliana glutathione S-transferase tau 2 mRNA, complete cds
AT3G13100	1.546446497	4.40E-24	Arabidopsis thaliana ABC transporter C family member 7 mRNA, complete cds
AT3G06020	1.287552812	4.78E-24	Arabidopsis thaliana chromosome 3, complete sequence
AT4G18160	1.146388951	5.03E-24	Arabidopsis thaliana protein two-pore potassium channel 3 mRNA, complete cds
AT1G11950	1.75762268	5.27E-24	Arabidopsis thaliana transcription factor jumonji (jmjC) domain-containing protein mRNA, complete cds
AT5G43420	1.135262383	5.29E-24	Arabidopsis thaliana chromosome 5 sequence
AT1G21460	1.264382863	5.66E-24	Arabidopsis thaliana bidirectional sugar transporter SWEET1 mRNA, complete cds
AT4G19020	1.059044104	6.00E-24	Arabidopsis thaliana chromomethylase 2

			mRNA, complete cds
AT3G21420	1.175428544	6.62E-24	Arabidopsis thaliana oxidoreductase, 2OG-Fe(II) oxygenase family protein mRNA, complete cds
AT3G21700	1.042922792	7.00E-24	Arabidopsis thaliana monomeric G protein SGP2 mRNA, complete cds
AT2G43320	1.050881706	7.14E-24	Arabidopsis thaliana S-adenosyl-L-methionine-dependent methyltransferase-like protein mRNA, complete cds
AT3G28740	4.352976787	7.96E-24	Arabidopsis thaliana cytochrome P450 CYP81D11 mRNA, complete cds
AT4G37850	1.966113794	8.34E-24	Arabidopsis thaliana transcription factor bHLH25 mRNA, complete cds
AT5G39520	2.131123993	8.48E-24	Arabidopsis thaliana uncharacterized protein mRNA, complete cds
AT1G09080	5.569110217	8.84E-24	Arabidopsis thaliana protein BIP3 mRNA, complete cds
AT2G20870	1.978434066	8.89E-24	Arabidopsis thaliana chromosome 2, complete sequence
AT4G38410	2.930905695	9.28E-24	Arabidopsis thaliana putative dehydrin mRNA, complete cds
AT4G27840	1.136828768	1.11E-23	Arabidopsis thaliana chromosome 4 sequence
AT3G23150	1.687317118	1.32E-23	Arabidopsis thaliana ethylene receptor 2 mRNA, complete cds
AT4G12410	3.530975089	1.34E-23	Arabidopsis thaliana chromosome 4 sequence
AT3G52740	2.123550773	1.39E-23	Arabidopsis thaliana chromosome 3, complete sequence
AT4G17410	1.037628598	1.55E-23	Arabidopsis thaliana DWNN domain-containing protein mRNA, complete cds
AT5G08780	1.625216462	1.65E-23	Arabidopsis thaliana winged-helix DNA-binding transcription factor family protein mRNA, complete cds
AT3G30210	4.61540387	1.71E-23	Arabidopsis thaliana myb domain protein 121 mRNA, complete cds
AT3G11900	1.035017594	1.74E-23	Arabidopsis thaliana mRNA for putative amino acid transporter protein, complete cds, clone: RAFL14-20-M19
AT2G36080	2.038316692	1.89E-23	Arabidopsis thaliana protein ABNORMAL SHOOT 2 mRNA, complete cds

AT2G21900	2.394276416	1.99E-23	Arabidopsis thaliana putative WRKY transcription factor 59 mRNA, complete cds
AT2G29990	1.614743446	2.19E-23	Arabidopsis thaliana alternative NAD(P)H dehydrogenase 2 mRNA, complete cds
AT2G36270	3.092853976	2.38E-23	Arabidopsis thaliana protein abscisic acid-insensitive 5 mRNA, complete cds
AT3G09450	3.518036033	2.44E-23	Arabidopsis thaliana uncharacterized protein mRNA, complete cds
AT3G03340	1.166968062	2.61E-23	Arabidopsis thaliana protein UNFERTILIZED EMBRYO SAC 6 mRNA, complete cds
AT1G26450	1.086520042	2.70E-23	Arabidopsis thaliana carbohydrate-binding X8 domain-containing protein mRNA, complete cds
AT4G32800	1.310143181	3.35E-23	Arabidopsis thaliana chromosome 4 sequence
AT3G04420	3.406295605	3.63E-23	Arabidopsis thaliana NAC domain containing protein 48 mRNA, complete cds
AT5G46710	1.386870607	4.55E-23	Arabidopsis thaliana PLATZ transcription factor family protein mRNA, complete cds
AT4G23920	1.416996492	4.70E-23	Arabidopsis thaliana UDP-glucose 4-epimerase 2 mRNA, complete cds
AT5G38120	1.58421711	5.02E-23	Arabidopsis thaliana 4-coumarate--CoA ligase-like 8 mRNA, complete cds
AT5G06300	1.604135347	5.07E-23	Arabidopsis thaliana cytokinin riboside 5'-monophosphate phosphoribohydrolase LOG7 mRNA, complete cds
AT3G16990	1.40064322	6.11E-23	Arabidopsis thaliana heme oxygenase-like, multi-helical protein mRNA, complete cds
AT5G57620	1.508652419	6.41E-23	Arabidopsis thaliana putative transcription factor MYB36 mRNA, complete cds
AT2G22850	1.339147633	6.45E-23	Arabidopsis thaliana basic leucine-zipper 6 mRNA, complete cds
AT3G10930	1.988964733	7.19E-23	Arabidopsis thaliana chromosome 3, complete sequence
AT2G44810	7.630510762	7.23E-23	Arabidopsis thaliana chromosome 2, complete sequence

AT2G27285	1.062757551	8.85E-23	Arabidopsis thaliana Coiled-coil domain-containing protein 55 mRNA, complete cds
AT2G45600	1.476598079	9.66E-23	Arabidopsis thaliana chromosome 2, complete sequence
AT1G80810	1.569110217	9.93E-23	Arabidopsis thaliana Tudor/PWWP/MBT superfamily protein mRNA, complete cds
AT5G47610	1.163243304	1.28E-22	Arabidopsis thaliana chromosome 5 sequence
AT1G80130	1.578422251	1.33E-22	Arabidopsis thaliana tetratricopeptide repeat domain-containing protein mRNA, complete cds
AT1G75490	2.778902602	1.34E-22	Arabidopsis thaliana chromosome 1 sequence
AT5G60350	5.488491757	1.50E-22	Arabidopsis thaliana uncharacterized protein mRNA, complete cds
AT5G47990	1.385124222	1.61E-22	Arabidopsis thaliana cytochrome P450 705A5 mRNA, complete cds
AT2G35300	7.600137113	1.77E-22	Arabidopsis thaliana chromosome 2, complete sequence
AT3G41979	2.259327552	1.82E-22	Erysimum belvederense internal transcribed spacer 1, partial sequence; 5.8S ribosomal RNA gene, complete sequence; and internal transcribed spacer 2, partial sequence
AT3G28030	1.470300916	2.50E-22	Arabidopsis thaliana DNA repair protein UVH3 mRNA, complete cds
AT5G48610	1.43436824	2.59E-22	Arabidopsis thaliana uncharacterized protein mRNA, complete cds
AT4G24010	2.719320029	2.79E-22	Arabidopsis thaliana cellulose synthase-like protein G1 mRNA, complete cds
AT4G16515	1.967160363	3.00E-22	Arabidopsis thaliana chromosome 4 sequence
AT1G02340	1.416703871	3.42E-22	Arabidopsis thaliana transcription factor HFR1 mRNA, complete cds
AT4G35480	1.204746856	3.48E-22	Arabidopsis thaliana chromosome 4 sequence
AT3G14560	1.200338403	4.08E-22	Arabidopsis thaliana chromosome 3, complete sequence
AT3G17110	7.569110217	4.37E-22	Arabidopsis thaliana chromosome 3, complete sequence
AT4G05110	1.870264344	4.44E-22	Arabidopsis thaliana equilibrative

			nucleoside transporter 6 mRNA, complete cds
AT5G15240	1.870264344	4.44E-22	Arabidopsis thaliana transmembrane amino acid transporter family protein mRNA, complete cds
AT4G11911	5.454939198	4.69E-22	Arabidopsis thaliana uncharacterized protein mRNA, complete cds
AT4G18530	1.363097916	5.15E-22	Arabidopsis thaliana uncharacterized protein mRNA, complete cds
AT1G71030	1.842731378	5.86E-22	Arabidopsis thaliana putative myb family transcription factor mRNA, complete cds
AT1G56020	1.420293055	8.26E-22	Arabidopsis thaliana chromosome 1 sequence
AT5G56210	1.118864939	8.41E-22	Arabidopsis thaliana WPP domain-interacting protein 2 mRNA, complete cds
AT3G54020	1.048477592	8.75E-22	Arabidopsis thaliana phosphatidylinositol:ceramide inositolphosphotransferase 1 mRNA, complete cds
AT4G37220	3.152463465	1.36E-21	Arabidopsis thaliana cold acclimation protein WCOR413 mRNA, complete cds
AT1G34420	2.20241711	1.40E-21	Arabidopsis thaliana leucine-rich repeat transmembrane protein kinase-like protein mRNA, complete cds
AT3G19050	1.707472744	1.47E-21	Arabidopsis thaliana phragmoplast orienting kinesin 2 mRNA, complete cds
AT3G16860	1.280253944	1.74E-21	Arabidopsis thaliana COBRA-like protein 8 mRNA, complete cds
AT2G13970.1	1.094128442	1.77E-21	Arabidopsis thaliana chromosome 2, complete sequence
AT3G53160	2.774900671	1.83E-21	Arabidopsis thaliana chromosome 3, complete sequence
AT3G45060	1.076980769	1.90E-21	Arabidopsis thaliana high affinity nitrate transporter 2.6 mRNA, complete cds
AT3G44735	1.07042293	2.03E-21	Arabidopsis thaliana Phytosulfokine 3 precursor mRNA, complete cds
AT5G38240	1.882049529	2.31E-21	Arabidopsis thaliana Protein kinase family protein mRNA, complete cds
AT1G16540	1.337011076	2.33E-21	Arabidopsis thaliana molybdenum cofactor sulfurase mRNA, complete cds
AT4G16144	1.192652555	2.36E-21	Arabidopsis thaliana AMSH-like ubiquitin thiolesterase 3 mRNA, complete cds

AT5G67310	5.403100266	2.61E-21	Arabidopsis thaliana cytochrome P450, family 81, subfamily G, polypeptide 1 mRNA, complete cds
AT2G31130	1.356249101	2.65E-21	Arabidopsis thaliana uncharacterized protein mRNA, complete cds
AT1G67070	1.87481796	3.66E-21	Arabidopsis thaliana mannose-6-phosphate isomerase mRNA, complete cds
AT1G71240	1.045548261	3.67E-21	Arabidopsis thaliana uncharacterized protein mRNA, complete cds
AT3G17130	1.969997228	3.92E-21	Arabidopsis thaliana chromosome 3, complete sequence
AT3G55840	1.99875405	4.43E-21	Arabidopsis thaliana chromosome 3, complete sequence
AT1G57750	4.835625192	4.71E-21	Arabidopsis thaliana chromosome 1 sequence
AT3G63350	4.835625192	4.71E-21	Arabidopsis thaliana heat stress transcription factor A-7b mRNA, complete cds
AT3G14370	1.962219878	6.30E-21	Arabidopsis thaliana chromosome 3, complete sequence
AT3G57760	1.878898392	6.34E-21	Arabidopsis thaliana protein kinase family protein mRNA, complete cds
AT5G24470	1.4727899	6.45E-21	Arabidopsis thaliana pseudo-response regulator 5 mRNA, complete cds
AT4G21060	1.219377717	6.55E-21	Arabidopsis thaliana AGP galactosyltransferase 2 mRNA, complete cds
AT2G21130	1.389135237	6.91E-21	Arabidopsis thaliana chromosome 2, complete sequence
AT1G26620	1.099487069	6.94E-21	Arabidopsis thaliana uncharacterized protein mRNA, complete cds
AT5G09980	3.113292868	7.77E-21	Arabidopsis thaliana elicitor peptide 4 mRNA, complete cds
AT1G73470	1.02777356	8.89E-21	Arabidopsis thaliana uncharacterized protein mRNA, complete cds
AT5G10650	1.071311357	9.09E-21	Arabidopsis thaliana RING/U-box superfamily protein mRNA, complete cds
AT4G23990	1.404629355	9.28E-21	Arabidopsis thaliana cellulose synthase-like protein G3 mRNA, complete cds
AT1G17020	2.146348902	1.03E-20	Arabidopsis thaliana Fe(II)/ascorbate oxidase family protein SRG1 mRNA,

			complete cds
AT5G35450	1.000460372	1.06E-20	Arabidopsis thaliana disease resistance RPP8-like protein 3 mRNA, complete cds
AT1G30620	1.054967414	1.15E-20	Arabidopsis thaliana UDP-arabinose 4-epimerase 1 mRNA, complete cds
AT2G29380	4.800435764	1.54E-20	Arabidopsis thaliana highly ABA-induced PP2C protein 3 mRNA, complete cds
AT2G36480	1.218457121	1.60E-20	Arabidopsis thaliana ENTH/VHS-like protein mRNA, complete cds
AT4G33930	7.437865684	1.68E-20	Arabidopsis thaliana chromosome 4 sequence
AT5G07990	1.735863762	1.95E-20	Arabidopsis thaliana Flavonoid 3'-monooxygenase mRNA, complete cds
AT1G62310	1.021701519	1.96E-20	Arabidopsis thaliana transcription factor jumonji domain-containing protein mRNA, complete cds
AT4G27070	1.121212531	2.31E-20	Arabidopsis thaliana tryptophan synthase beta chain mRNA, complete cds
AT4G36670	1.245935375	2.47E-20	Arabidopsis thaliana putative polyol transporter 6 mRNA, complete cds
AT3G43210	1.27369421	2.55E-20	Arabidopsis thaliana kinesin TETRASPORE mRNA, complete cds
AT5G05965	5.33095048	2.59E-20	Arabidopsis thaliana uncharacterized protein mRNA, complete cds
AT4G35985	1.105303004	2.72E-20	Arabidopsis thaliana senescence/dehydration-associated protein mRNA, complete cds
AT2G47460	3.74598798	2.85E-20	Arabidopsis thaliana transcription factor MYB12 mRNA, complete cds
AT1G69310	1.167984337	3.59E-20	Arabidopsis thaliana putative WRKY transcription factor 57 mRNA, complete cds
AT2G38000	1.122363858	4.05E-20	Arabidopsis thaliana chaperone protein dnaJ-like protein mRNA, complete cds
AT3G21660	3.244328126	4.27E-20	Arabidopsis thaliana UBX domain-containing protein mRNA, complete cds
AT4G37780	3.338330011	5.42E-20	Arabidopsis thaliana myb domain protein 87 mRNA, complete cds
AT5G41550	2.654357504	6.01E-20	Arabidopsis thaliana TIR-NBS-LRR class disease resistance protein mRNA, complete cds

AT2G36350	1.350686698	6.80E-20	Arabidopsis thaliana protein kinase mRNA, complete cds
AT3G26280	1.186208668	6.95E-20	Arabidopsis thaliana cytochrome P450 71B4 mRNA, complete cds
AT4G39955	1.292342026	8.93E-20	Arabidopsis thaliana hydrolase, alpha/beta fold family protein mRNA, complete cds
AT1G72140	1.663985467	9.25E-20	Arabidopsis thaliana putative peptide/nitrate transporter mRNA, complete cds
AT1G71360	1.216507082	9.56E-20	Arabidopsis thaliana Galactose-binding protein mRNA, complete cds
AT3G15200	2.261276952	1.31E-19	Arabidopsis thaliana chromosome 3, complete sequence
AT4G37150	2.186904111	1.32E-19	Arabidopsis thaliana methyl esterase 9 mRNA, complete cds
AT1G11100	1.956614533	1.33E-19	Arabidopsis thaliana SNF2 , helicase and zinc-finger domain-containing protein mRNA, complete cds
AT1G18830	4.727372301	1.66E-19	Arabidopsis thaliana transport protein SEC31-like protein SEC31B mRNA, complete cds
AT3G52310	2.721113311	2.33E-19	Arabidopsis thaliana ABC transporter G family member 27 mRNA, complete cds
AT5G02170	2.102881436	3.39E-19	Arabidopsis thaliana transmembrane amino acid transporter family protein mRNA, complete cds
AT1G05575	2.325656181	3.40E-19	Arabidopsis thaliana chromosome 1 sequence
AT3G16510	1.552182401	3.72E-19	Arabidopsis thaliana chromosome 3, complete sequence
AT5G65890	1.218697164	4.05E-19	Arabidopsis thaliana ACT domain repeat 1 mRNA, complete cds
AT5G11140	4.063470169	4.07E-19	Arabidopsis thaliana chromosome 5 sequence
AT4G15233	1.188850739	4.27E-19	Arabidopsis thaliana ABC transporter G family member 42 mRNA, complete cds
AT2G45050	1.161689856	4.31E-19	Arabidopsis thaliana GATA transcription factor 2 mRNA, complete cds
AT1G75310	1.3834179	6.35E-19	Arabidopsis thaliana auxin-like 1 protein mRNA, complete cds
AT5G41070	1.3522096	6.99E-19	Arabidopsis thaliana dsRNA-binding protein 5 mRNA, complete cds
AT1G66830	3.003320026	7.90E-19	Arabidopsis thaliana probable inactive

			leucine-rich repeat receptor-like protein kinase mRNA, complete cds
AT4G36650	1.364874561	8.08E-19	Arabidopsis thaliana plant-specific TFIIB-related protein mRNA, complete cds
AT5G24600	4.312334802	8.37E-19	Arabidopsis thaliana uncharacterized protein mRNA, complete cds
AT4G32295	1.415242775	9.64E-19	Arabidopsis thaliana uncharacterized protein mRNA, complete cds
AT3G43250	6.195295381	1.20E-18	Arabidopsis thaliana chromosome 3, complete sequence
AT5G05870	1.045548261	1.24E-18	Arabidopsis thaliana mRNA for glucuronosyl transferase-like protein, complete cds, clone: RAFL14-74-G20
AT5G67160	1.08263758	1.36E-18	Arabidopsis thaliana chromosome 5 sequence
AT1G11185	1.84072847	1.44E-18	Arabidopsis thaliana chromosome 1 sequence
AT4G17245	1.059903554	1.97E-18	Arabidopsis thaliana chromosome 4 sequence
AT3G23170	1.208870528	1.97E-18	Arabidopsis thaliana chromosome 3, complete sequence
AT5G60910	1.623624377	2.09E-18	Arabidopsis thaliana agamous-like MADS-box protein AGL8 mRNA, complete cds
AT1G63860	1.308582667	2.22E-18	Arabidopsis thaliana TIR-NBS-LRR class disease resistance protein mRNA, complete cds
AT2G36780	2.974465163	2.49E-18	Arabidopsis thaliana chromosome 2, complete sequence
AT5G43890	2.128010422	2.66E-18	Arabidopsis thaliana chromosome 5 sequence
AT1G72840	1.260052773	2.66E-18	Arabidopsis thaliana TIR-NBS-LRR class disease resistance protein mRNA, complete cds
AT2G32190	2.569110217	2.76E-18	Arabidopsis thaliana uncharacterized protein mRNA, complete cds
AT1G04490	2.226120507	2.76E-18	Arabidopsis thaliana uncharacterized protein mRNA, complete cds
AT5G62170	1.341481148	3.02E-18	Arabidopsis thaliana uncharacterized protein mRNA, complete cds
AT5G67140	1.035156656	3.10E-18	Arabidopsis thaliana F-box/RNI-like superfamily protein mRNA, complete cds

AT5G54165	6.154072718	3.50E-18	Arabidopsis thaliana chromosome 5 sequence
AT5G65870	1.509712618	3.66E-18	Arabidopsis thaliana putative phytosulfokines 5 precursor mRNA, complete cds
AT1G73880	1.474859892	3.70E-18	Arabidopsis thaliana chromosome 1 sequence
AT2G30800	1.028187101	5.70E-18	Arabidopsis thaliana protein helicase in vascular tissue and tapetum mRNA, complete cds
AT1G30135	4.61033288	5.97E-18	Arabidopsis thaliana protein TIFY 5A mRNA, complete cds
AT2G36770	3.030441369	6.07E-18	Arabidopsis thaliana chromosome 2, complete sequence
AT4G16870.1	1.134553267	6.29E-18	Arabidopsis thaliana chromosome 4 sequence
AT1G67000	2.811083008	6.84E-18	Arabidopsis thaliana probable receptor-like protein kinase mRNA, complete cds
AT4G36700	7.195295381	7.38E-18	Arabidopsis thaliana cupin family protein mRNA, complete cds
AT4G28110	3.109678599	8.18E-18	Arabidopsis thaliana myb domain protein 41 mRNA, complete cds
AT3G22980	1.156750824	9.04E-18	Arabidopsis thaliana chromosome 3, complete sequence
AT5G22520	4.23537282	9.35E-18	Arabidopsis thaliana chromosome 5 sequence
AT3G58790	1.19855988	9.56E-18	Arabidopsis thaliana probable galacturonosyltransferase 15 mRNA, complete cds
AT2G35950	2.867549959	9.81E-18	Arabidopsis thaliana protein EMBRYO SAC DEVELOPMENT ARREST 12 mRNA, complete cds
AT2G30130	2.416804069	1.05E-17	Arabidopsis thaliana LOB domain-containing protein 12 mRNA, complete cds
AT2G16720	1.130939753	1.05E-17	Arabidopsis thaliana myb domain protein 7 mRNA, complete cds
AT2G23740	1.130939753	1.05E-17	Arabidopsis thaliana histone-lysine N-methyltransferase SUVR5 mRNA, complete cds
AT1G61255	3.301888015	1.38E-17	Arabidopsis thaliana chromosome 1 sequence
AT5G49665	1.056834069	1.46E-17	Arabidopsis thaliana C3H4 type zinc

			finger protein mRNA, complete cds
AT2G25820	3.420587693	1.72E-17	Arabidopsis thaliana chromosome 2, complete sequence
AT5G47230	1.027670771	1.76E-17	Arabidopsis thaliana chromosome 5 sequence
AT3G47780	1.521697275	1.78E-17	Arabidopsis thaliana ABC transporter A family member 7 mRNA, complete cds
AT5G24640	7.154072718	1.92E-17	Arabidopsis thaliana chromosome 5 sequence
AT1G15520	1.569110217	1.93E-17	Arabidopsis thaliana ABC transporter G family member 40 mRNA, complete cds
AT5G67230	1.287026524	1.93E-17	Arabidopsis thaliana probable beta-1,4-xylosyltransferase IRX14H mRNA, complete cds
AT1G33260	1.094457862	2.12E-17	Arabidopsis thaliana probable receptor-like protein kinase mRNA, complete cds
AT3G49160	1.172305404	2.47E-17	Arabidopsis thaliana pyruvate kinase-like protein mRNA, complete cds
AT3G05830	1.133544742	2.68E-17	Arabidopsis thaliana alpha-helical IF-like protein mRNA, complete cds
AT5G51990	5.089942381	2.75E-17	Arabidopsis thaliana chromosome 5 sequence
AT1G17180	2.658525138	2.92E-17	Arabidopsis thaliana glutathione S-transferase TAU 25 mRNA, complete cds
AT5G61270	1.403848746	3.12E-17	Arabidopsis thaliana transcription factor PIF7 mRNA, complete cds
AT3G11580	2.320555309	3.30E-17	Arabidopsis thaliana AP2/B3-like transcriptional factor family protein mRNA, complete cds
AT3G59900	1.969599408	3.93E-17	Arabidopsis thaliana chromosome 3, complete sequence
AT5G28320	1.818137765	4.68E-17	Arabidopsis thaliana uncharacterized protein mRNA, complete cds
AT1G46554	1.774002356	5.37E-17	Arabidopsis thaliana chromosome 1 sequence
AT1G66400	1.328855062	5.46E-17	Arabidopsis thaliana chromosome 1 sequence
AT1G53540	4.174831278	5.72E-17	Arabidopsis thaliana chromosome 1 sequence
AT1G15940	1.049400587	5.74E-17	Arabidopsis thaliana aspartyl beta-hydroxylase N-terminal region domain-containing protein mRNA,

			complete cds
AT5G66310	1.125471806	6.20E-17	Arabidopsis thaliana ATP binding microtubule motor family protein mRNA, complete cds
AT4G27970	1.249237244	7.95E-17	Arabidopsis thaliana SLAC1 homologue 2 mRNA, complete cds
AT4G22970	1.812887505	1.29E-16	Arabidopsis thaliana separase-like protein mRNA, complete cds
AT4G18050	1.290051384	1.39E-16	Arabidopsis thaliana P-glycoprotein 9 mRNA, complete cds
AT2G33435	1.311981244	1.64E-16	Arabidopsis thaliana RNA recognition motif-containing protein mRNA, complete cds
AT1G32190	1.255662353	1.64E-16	Arabidopsis thaliana alpha/beta-hydrolase-like protein mRNA, complete cds
AT5G19097.1	2.936319192	1.91E-16	Arabidopsis thaliana chromosome 5 sequence
AT5G04120	2.465087153	2.07E-16	Arabidopsis thaliana phosphoglycerate mutase-like protein mRNA, complete cds
AT1G17170	2.421415163	2.22E-16	Arabidopsis thaliana glutathione S-transferase TAU 24 mRNA, complete cds
AT5G56160	1.343727754	3.21E-16	Arabidopsis thaliana sec14p-like phosphatidylinositol transfer-like protein mRNA, complete cds
AT2G20290	1.332429409	3.28E-16	Arabidopsis thaliana myosin-like protein XIG mRNA, complete cds
AT5G50335	2.920017379	3.38E-16	Arabidopsis thaliana chromosome 5 sequence
AT4G35180	4.111637452	3.51E-16	Arabidopsis thaliana LYS/HIS transporter 7 mRNA, complete cds
AT4G04490	2.045548261	3.78E-16	Arabidopsis thaliana cysteine-rich receptor-like protein kinase 36 mRNA, complete cds
AT1G76470	4.460585761	4.00E-16	Arabidopsis thaliana NAD(P)-binding Rossmann-fold superfamily protein mRNA, complete cds
AT3G10815	2.58744204	4.54E-16	Arabidopsis thaliana RING/U-box domain-containing protein mRNA, complete cds
AT3G02890	1.011332546	4.65E-16	Arabidopsis thaliana RING/FYVE/PHD zinc finger-related protein mRNA, complete cds

AT4G34250	1.337729013	4.65E-16	Arabidopsis thaliana chromosome 4 sequence
AT5G18700	1.326504575	4.74E-16	Arabidopsis thaliana protein kinase family protein with ARM repeat domain mRNA, complete cds
AT2G18340	6.999744572	5.69E-16	Arabidopsis thaliana late embryogenesis abundant domain-containing protein mRNA, complete cds
AT2G18550	2.903529257	5.97E-16	Arabidopsis thaliana homeobox-leucine zipper protein ATHB-21 mRNA, complete cds
AT1G31810	1.210293024	6.30E-16	Arabidopsis thaliana formin-like protein 14 mRNA, complete cds
AT5G51440	1.08531879	6.33E-16	Arabidopsis thaliana mRNA for mitochondrial heat shock 22 kd protein-like, complete cds, clone: RAFL21-16-A12
AT5G16200	1.279345446	7.01E-16	Arabidopsis thaliana chromosome 5 sequence
AT1G69150	2.185478523	7.31E-16	Arabidopsis thaliana chromosome 1 sequence
AT2G17660	3.61033288	9.01E-16	Arabidopsis thaliana chromosome 2, complete sequence
AT1G20490	1.212843007	9.10E-16	Arabidopsis thaliana 4-coumarate--CoA ligase-like 3 mRNA, complete cds
AT3G56790	6.976285599	9.29E-16	Arabidopsis thaliana chromosome 3, complete sequence
AT2G18240	1.105959778	9.62E-16	Arabidopsis thaliana protein RER1D mRNA, complete cds
AT1G01500	1.591197838	9.95E-16	Arabidopsis thaliana Erythronate-4-phosphate dehydrogenase-like protein mRNA, complete cds
AT1G11410	1.01418409	1.07E-15	Arabidopsis thaliana G-type lectin S-receptor-like serine/threonine-protein kinase mRNA, complete cds
AT5G50170	1.364302108	1.08E-15	Arabidopsis thaliana C2 and GRAM domain-containing protein mRNA, complete cds
AT5G15800	2.341004145	1.14E-15	Arabidopsis thaliana MADS box transcription factor SEPALLATA 1 mRNA, complete cds
AT5G20220	1.096174334	1.25E-15	Arabidopsis thaliana CCHC-type zinc knuckle protein mRNA, complete cds

AT1G06490	1.308582667	1.42E-15	Arabidopsis thaliana callose synthase 7 mRNA, complete cds
AT1G32940	1.06293034	1.43E-15	Arabidopsis thaliana Subtilase 3.5 mRNA, complete cds
AT5G10720	1.345593238	1.62E-15	Arabidopsis thaliana histidine kinase 5 mRNA, complete cds
AT1G14550	2.662219622	1.81E-15	Arabidopsis thaliana peroxidase 5 mRNA, complete cds
AT5G12030	2.367476356	1.84E-15	Arabidopsis thaliana chromosome 5 sequence
AT2G19190	2.869976697	1.87E-15	Arabidopsis thaliana FLG22-induced receptor-like kinase 1 mRNA, complete cds
AT1G30190	2.327583629	1.91E-15	Arabidopsis thaliana chromosome 1 sequence
AT3G47540	1.10213179	1.93E-15	Arabidopsis thaliana putative chitinase mRNA, complete cds
AT5G58680	2.29017063	1.97E-15	Arabidopsis thaliana chromosome 5 sequence
AT3G46080	4.045548261	2.16E-15	Arabidopsis thaliana chromosome 3, complete sequence
AT2G41730	2.788052039	2.28E-15	Arabidopsis thaliana chromosome 2, complete sequence
AT1G33430	5.878438276	2.43E-15	Arabidopsis thaliana putative beta-1,3-galactosyltransferase 8 mRNA, complete cds
AT4G25850	2.943668647	2.63E-15	Arabidopsis thaliana OSBP(oxysterol binding protein)-related protein 4B mRNA, complete cds
AT3G46230	2.943668647	2.63E-15	Arabidopsis thaliana chromosome 3, complete sequence
AT5G57390	1.532535603	2.87E-15	Arabidopsis thaliana AP2-like ethylene-responsive transcription factor AIL5 mRNA, complete cds
AT4G26200	3.027400915	3.63E-15	Arabidopsis thaliana 1-aminocyclopropane-1-carboxylate synthase 7 mRNA, complete cds
AT4G14080	6.903529257	4.08E-15	Arabidopsis thaliana putative glucan endo-1,3-beta-glucosidase A6 mRNA, complete cds
AT4G15990	1.613537684	4.68E-15	Arabidopsis thaliana chromosome 4 sequence
AT5G64810	2.108558059	5.32E-15	Arabidopsis thaliana putative WRKY transcription factor 51 mRNA, complete

			cds
AT1G14480	1.254265746	5.97E-15	Arabidopsis thaliana Ankyrin repeat family protein mRNA, complete cds
AT2G26290	1.630510762	6.16E-15	Arabidopsis thaliana root-specific kinase 1 mRNA, complete cds
AT4G06746	2.512674272	6.86E-15	Arabidopsis thaliana chromosome 4 sequence
AT1G78340	2.512674272	6.86E-15	Arabidopsis thaliana glutathione S-transferase TAU 22 mRNA, complete cds
AT5G13880	1.60497567	7.13E-15	Arabidopsis thaliana chromosome 5 sequence
AT4G12580	2.367476356	8.47E-15	Arabidopsis thaliana chromosome 4 sequence
AT2G01008	3.526674951	9.92E-15	Arabidopsis thaliana uncharacterized protein mRNA, complete cds
AT1G07985	3.526674951	9.92E-15	Arabidopsis thaliana chromosome 1 sequence
AT3G26830	1.105669254	1.05E-14	Arabidopsis thaliana protein PHYTOALEXIN DEFICIENT 3 mRNA, complete cds
AT2G48010	1.198794522	1.09E-14	Arabidopsis thaliana chromosome 2, complete sequence
AT4G16550	6.852903183	1.10E-14	Arabidopsis thaliana HSP20-like chaperone mRNA, complete cds
AT1G09950	2.990406707	1.15E-14	Arabidopsis thaliana chromosome 1 sequence
AT3G61570	1.142121521	1.17E-14	Arabidopsis thaliana golgin candidate 3 mRNA, complete cds
AT4G12120	1.123550773	1.23E-14	Arabidopsis thaliana protein transport sec1b mRNA, complete cds
AT1G16510	1.68822626	1.36E-14	Arabidopsis thaliana chromosome 1 sequence
AT1G19200	2.057931986	1.37E-14	Arabidopsis thaliana uncharacterized protein mRNA, complete cds
AT5G17700	1.064407289	1.66E-14	Arabidopsis thaliana MATE efflux family protein mRNA, complete cds
AT4G23000	1.347203961	1.68E-14	Arabidopsis thaliana calcineurin-like metallo-phosphoesterase family protein mRNA, complete cds
AT5G62480	1.137608814	1.97E-14	Arabidopsis thaliana glutathione S-transferase tau 9 mRNA, complete cds
AT3G06780	1.125275454	2.16E-14	Arabidopsis thaliana chromosome 3, complete sequence

AT3G16175	2.126468257	2.49E-14	Arabidopsis thaliana thioesterase family protein mRNA, complete cds
AT1G13195	1.036446054	2.83E-14	Arabidopsis thaliana RING/U-box domain-containing protein mRNA, complete cds
AT5G41620	1.181920703	3.02E-14	Arabidopsis thaliana uncharacterized protein mRNA, complete cds
AT1G13480	1.858462709	3.29E-14	Arabidopsis thaliana uncharacterized protein mRNA, complete cds
AT1G17230	1.206703053	3.30E-14	Arabidopsis thaliana leucine-rich receptor-like protein kinase mRNA, complete cds
AT3G49760	2.085076626	3.97E-14	Arabidopsis thaliana chromosome 3, complete sequence
AT4G28840	2.323533009	4.08E-14	Arabidopsis thaliana TCP interactor containing EAR motif protein1 mRNA, complete cds
AT3G09375	6.773468716	4.96E-14	Arabidopsis thaliana chromosome 3, complete sequence
AT4G29110	1.586116643	5.07E-14	Arabidopsis thaliana chromosome 4 sequence
AT5G55050	1.109678599	5.71E-14	Arabidopsis thaliana GDSL esterase/lipase mRNA, complete cds
AT1G69930	4.773468716	6.02E-14	Arabidopsis thaliana glutathione S-transferase TAU 11 mRNA, complete cds
AT1G01250	2.684958546	6.53E-14	Arabidopsis thaliana chromosome 1 sequence
AT4G29940	1.156355047	6.60E-14	Arabidopsis thaliana Pathogenesis-related homeodomain protein mRNA, complete cds
AT3G22790	1.086770924	6.62E-14	Arabidopsis thaliana protein NETWORKED 1A mRNA, complete cds
AT3G03900	1.040794731	7.81E-14	Arabidopsis thaliana adenosine-5'-phosphosulfate (APS) kinase 3 mRNA, complete cds
AT3G25730	1.522361958	8.28E-14	Arabidopsis thaliana chromosome 3, complete sequence
AT1G62305	1.136456758	8.42E-14	Arabidopsis thaliana core-2/I-branching beta-1,6-N-acetylglucosaminyltransferase family protein mRNA, complete cds
AT4G15230	1.335844747	8.65E-14	Arabidopsis thaliana ABC transporter G family member 30 mRNA, complete cds
AT4G30097	1.870461555	9.96E-14	Arabidopsis thaliana chromosome 4

			sequence
AT3G21600	1.087673737	1.17E-13	Arabidopsis thaliana Senescence/dehydration-associated protein-like protein mRNA, complete cds
AT4G21930	5.689404451	1.21E-13	Arabidopsis thaliana chromosome 4 sequence
AT3G22100	5.689404451	1.21E-13	Arabidopsis thaliana chromosome 3, complete sequence
AT5G51760	5.689404451	1.21E-13	Arabidopsis thaliana probable protein phosphatase AHG1 mRNA, complete cds
AT3G22160	1.279220608	1.29E-13	Arabidopsis thaliana chromosome 3, complete sequence
AT1G76640	3.630510762	1.30E-13	Arabidopsis thaliana chromosome 1 sequence
AT5G19470	6.717973603	1.37E-13	Arabidopsis thaliana nudix hydrolase 24 mRNA, complete cds
AT1G48120	1.305784034	1.53E-13	Arabidopsis thaliana serine/threonine-protein phosphatase 7 long form homolog mRNA, complete cds
AT1G15580	1.452365254	1.58E-13	Arabidopsis thaliana auxin-responsive protein IAA5 mRNA, complete cds
AT1G12370	1.119257948	1.86E-13	Arabidopsis thaliana photolyase 1 mRNA, complete cds
AT4G36830	2.10213179	1.87E-13	Arabidopsis thaliana chromosome 4 sequence
AT2G14820	1.382989356	1.95E-13	Arabidopsis thaliana BTB/POZ domain-containing protein NPY2 mRNA, complete cds
AT5G17450	1.203089538	1.99E-13	Arabidopsis thaliana heavy metal transport/detoxification domain-containing protein mRNA, complete cds
AT1G75590	1.915912981	2.04E-13	Arabidopsis thaliana chromosome 1 sequence
AT3G57460	1.311442321	2.19E-13	Arabidopsis thaliana catalytic/ metal ion binding / metalloendopeptidase/ zinc ion binding protein mRNA, complete cds
AT1G61800	2.795570008	2.49E-13	Arabidopsis thaliana glucose-6-phosphate/phosphate translocator 2 mRNA, complete cds
AT5G04080	1.265674076	2.64E-13	Arabidopsis thaliana uncharacterized protein mRNA, complete cds
AT5G46590	2.708513274	2.97E-13	Arabidopsis thaliana NAC domain containing protein 96 mRNA, complete

			cds
AT1G62940	4.188506215	3.09E-13	Arabidopsis thaliana acyl-CoA synthetase 5 mRNA, complete cds
AT1G53110	1.30428253	3.16E-13	Arabidopsis thaliana putative proton pump interactor protein mRNA, complete cds
AT1G02190	2.305415388	3.17E-13	Arabidopsis thaliana protein CER1-like 1 mRNA, complete cds
AT4G27530	5.630510762	3.75E-13	Arabidopsis thaliana uncharacterized protein mRNA, complete cds
AT4G22530	1.3834179	4.01E-13	Arabidopsis thaliana S-adenosyl-L-methionine-dependent methyltransferase domain-containing protein mRNA, complete cds
AT2G23180	1.441032963	4.09E-13	Arabidopsis thaliana chromosome 2, complete sequence
AT2G42200	1.48738582	4.16E-13	Arabidopsis thaliana squamosa promoter-binding-like protein 9 mRNA, complete cds
AT1G17450	1.239152259	4.42E-13	Arabidopsis thaliana B-block binding subunit of TFIIIC mRNA, complete cds
AT5G62100	1.446427698	4.82E-13	Arabidopsis thaliana BCL-2-associated athanogene 2 mRNA, complete cds
AT1G15040	3.215473263	5.10E-13	Arabidopsis thaliana chromosome 1 sequence
AT1G64460	1.122907144	5.75E-13	Arabidopsis thaliana Protein kinase superfamily protein mRNA, complete cds
AT2G41210	1.265596742	6.51E-13	Arabidopsis thaliana phosphatidylinositol-4-phosphate 5-kinase 5 mRNA, complete cds
AT1G64110	4.660258106	6.62E-13	Arabidopsis thaliana transcription factor DUO1 mRNA, complete cds
AT5G49120	1.84072847	6.93E-13	Arabidopsis thaliana uncharacterized protein mRNA, complete cds
AT4G20930	1.213819744	7.25E-13	Arabidopsis thaliana probable 3-hydroxyisobutyrate dehydrogenase mRNA, complete cds
AT4G27360	2.478507669	7.36E-13	Arabidopsis thaliana Dynein light chain type 1 family protein mRNA, complete cds
AT2G01340	1.61033288	7.39E-13	Arabidopsis thaliana uncharacterized protein mRNA, complete cds
AT4G18210	1.348111031	7.41E-13	Arabidopsis thaliana purine permease 10

			mRNA, complete cds
AT5G18560	2.756041644	7.70E-13	Arabidopsis thaliana chromosome 5 sequence
AT2G32210	2.367476356	8.39E-13	Arabidopsis thaliana uncharacterized protein mRNA, complete cds
AT1G74490	1.898706873	9.07E-13	Arabidopsis thaliana protein kinase superfamily protein mRNA, complete cds
AT5G55090	1.139875644	1.11E-12	Arabidopsis thaliana chromosome 5 sequence
AT3G49690	1.260816248	1.11E-12	Arabidopsis thaliana transcription factor RAX3 mRNA, complete cds
AT5G49620	2.832144623	1.12E-12	Arabidopsis thaliana myb domain protein 78 mRNA, complete cds
AT5G04020	1.067744008	1.28E-12	Arabidopsis thaliana calmodulin-binding protein mRNA, complete cds
AT1G13220	1.035055888	1.50E-12	Arabidopsis thaliana protein CROWDED NUCLEI 2 mRNA, complete cds
AT1G01570	1.725930327	1.56E-12	Arabidopsis thaliana uncharacterized protein mRNA, complete cds
AT2G43580	6.569110217	1.77E-12	Arabidopsis thaliana chitinase family protein mRNA, complete cds
AT4G02710	1.105920879	1.84E-12	Arabidopsis thaliana Kinase interacting (KIP1-like) family protein mRNA, complete cds
AT1G52830	1.834044156	1.92E-12	Arabidopsis thaliana indole-3-acetic acid 6 mRNA, complete cds
AT5G37550	1.977161286	1.95E-12	Arabidopsis thaliana chromosome 5 sequence
AT4G31950	3.318566756	2.13E-12	Arabidopsis thaliana cytochrome P450, family 82, subfamily C, polypeptide 3 mRNA, complete cds
AT4G01020	1.213748582	2.15E-12	Arabidopsis thaliana zinc finger-related and helicase and IBR domain-containing protein mRNA, complete cds
AT3G05820	2.199353597	2.52E-12	Arabidopsis thaliana alkaline/neutral invertase H mRNA, complete cds
AT1G31820	1.34322881	2.64E-12	Arabidopsis thaliana polyamine transporter 1 mRNA, complete cds
AT1G47980	6.537401358	2.97E-12	Arabidopsis thaliana uncharacterized protein mRNA, complete cds
AT4G13290	1.363288559	3.07E-12	Arabidopsis thaliana cytochrome P450 71A19 mRNA, complete cds
AT3G13600	1.493007238	3.07E-12	Arabidopsis thaliana calmodulin-binding

			family protein mRNA, complete cds
AT5G13320	1.384858435	3.52E-12	Arabidopsis thaliana 4-substituted benzoates-glutamate ligase GH3.12 mRNA, complete cds
AT3G29590	1.77424624	3.87E-12	Arabidopsis thaliana chromosome 3, complete sequence
AT5G22570	4.569110217	4.03E-12	Arabidopsis thaliana putative WRKY transcription factor 38 mRNA, complete cds
AT5G06090	2.267940683	4.15E-12	Arabidopsis thaliana glycerol-3-phosphate acyltransferase 7 mRNA, complete cds
AT3G50260	1.172305404	4.68E-12	Arabidopsis thaliana chromosome 3, complete sequence
AT1G21520	1.255471331	4.71E-12	Arabidopsis thaliana uncharacterized protein mRNA, complete cds
AT5G52160	6.50497988	5.01E-12	Arabidopsis thaliana bifunctional inhibitor/lipid-transfer protein/seed storage 2S albumin-like protein mRNA, complete cds
AT3G50870	1.948872242	5.09E-12	Arabidopsis thaliana GATA transcription factor 18 mRNA, complete cds
AT1G44830	1.588075496	5.30E-12	Arabidopsis thaliana chromosome 1 sequence
AT1G11170	1.418591672	5.60E-12	Arabidopsis thaliana uncharacterized protein mRNA, complete cds
AT1G52430	4.045548261	6.46E-12	Arabidopsis thaliana ubiquitin carboxyl-terminal hydrolase-related protein mRNA, complete cds
AT1G58225	1.888006984	6.98E-12	Arabidopsis thaliana uncharacterized protein mRNA, complete cds
AT3G02310	2.673579484	7.21E-12	Arabidopsis thaliana developmental protein SEPALLATA 2 mRNA, complete cds
AT5G50570	2.673579484	7.21E-12	Arabidopsis thaliana squamosa promoter-binding-like protein 13 mRNA, complete cds
AT3G10010	1.204746856	7.46E-12	Arabidopsis thaliana putative DNA glycosylase mRNA, complete cds
AT3G23230	1.910618681	7.58E-12	Arabidopsis thaliana chromosome 3, complete sequence
AT4G27657	1.250238681	8.06E-12	Arabidopsis thaliana chromosome 4 sequence
AT1G32910	6.471813016	8.44E-12	Arabidopsis thaliana HXXXD-type

			acyl-transferase-like protein mRNA, complete cds
AT4G09600	6.471813016	8.44E-12	Arabidopsis thaliana gibberellin-regulated protein 3 mRNA, complete cds
AT4G11070	3.451540621	8.74E-12	Arabidopsis thaliana putative WRKY transcription factor 41 mRNA, complete cds
AT5G35110	1.986654572	9.38E-12	Arabidopsis thaliana chromosome 5 sequence
AT4G14090	1.103392336	9.52E-12	Arabidopsis thaliana chromosome 4 sequence
AT1G10530	1.852903183	1.02E-11	Arabidopsis thaliana uncharacterized protein mRNA, complete cds
AT1G46768	1.341789713	1.13E-11	Arabidopsis thaliana chromosome 1 sequence
AT2G26370	2.280013515	1.15E-11	Arabidopsis thaliana MD-2-related lipid recognition domain-containing protein mRNA, complete cds
AT2G29500	2.149884921	1.15E-11	Arabidopsis thaliana chromosome 2, complete sequence
AT1G48405	5.437865684	1.16E-11	Arabidopsis thaliana Kinase interacting (KIP1-like) family protein mRNA, complete cds
AT1G66370	5.437865684	1.16E-11	Arabidopsis thaliana transcription factor MYB113 mRNA, complete cds
AT3G21320	2.952438857	1.25E-11	Arabidopsis thaliana uncharacterized protein mRNA, complete cds
AT4G11650	1.045548261	1.39E-11	Arabidopsis thaliana osmotin-like protein OSM34 mRNA, complete cds
AT1G12540	6.437865684	1.43E-11	Arabidopsis thaliana transcription factor bHLH55 mRNA, complete cds
AT3G48390	1.619015123	1.43E-11	Arabidopsis thaliana MA3 domain-containing protein mRNA, complete cds
AT1G69600	2.569110217	1.43E-11	Arabidopsis thaliana chromosome 1 sequence
AT3G18485	2.494855663	1.58E-11	Arabidopsis thaliana protein IAA-LEUCINE RESISTANT 2 mRNA, complete cds
AT5G14230	1.017533885	1.70E-11	Arabidopsis thaliana uncharacterized protein mRNA, complete cds
AT1G19530	1.202517608	1.84E-11	Arabidopsis thaliana uncharacterized protein mRNA, complete cds

AT1G61370	1.128803765	1.96E-11	Arabidopsis thaliana S-locus lectin protein kinase family protein mRNA, complete cds
AT5G49920	5.403100266	2.06E-11	Arabidopsis thaliana octicosapeptide/Phox/Bem1p domain-containing protein mRNA, complete cds
AT1G67810	1.146075137	2.15E-11	Arabidopsis thaliana chromosome 1 sequence
AT3G43110	2.928191311	2.23E-11	Arabidopsis thaliana chromosome 3, complete sequence
AT5G48400	2.928191311	2.23E-11	Arabidopsis thaliana glutamate receptor 1.2 mRNA, complete cds
AT1G23450	3.215473263	2.28E-11	Arabidopsis thaliana pentatricopeptide repeat-containing protein mRNA, complete cds
AT3G51810	6.403100266	2.42E-11	Arabidopsis thaliana Em-like protein GEA1 mRNA, complete cds
AT4G16820	4.471813016	2.47E-11	Arabidopsis thaliana chromosome 4 sequence
AT1G15190	2.806360598	2.77E-11	Arabidopsis thaliana chromosome 1 sequence
AT2G35730	2.408118341	2.91E-11	Arabidopsis thaliana heavy-metal-associated domain-containing protein mRNA, complete cds
AT5G67080	2.115937589	3.14E-11	Arabidopsis thaliana chromosome 5 sequence
AT4G24040	1.695802222	3.17E-11	Arabidopsis thaliana trehalase 1 mRNA, complete cds
AT1G19630	2.197551355	3.24E-11	Arabidopsis thaliana cytochrome P450, family 722, subfamily A, polypeptide 1 mRNA, complete cds
AT4G22620	2.197551355	3.24E-11	Arabidopsis thaliana chromosome 4 sequence
AT5G10370	1.33207102	3.38E-11	Arabidopsis thaliana helicase , IBR and zinc finger protein domain-containing protein mRNA, complete cds
AT4G37030	1.563396566	3.78E-11	Arabidopsis thaliana uncharacterized protein mRNA, complete cds
AT5G58650	1.496927608	3.98E-11	Arabidopsis thaliana tyrosine-sulfated glycopeptide 1 mRNA, complete cds
AT1G71000	3.952438857	4.01E-11	Arabidopsis thaliana chaperone DnaJ-domain containing protein mRNA,

			complete cds
AT2G31690	6.367476356	4.10E-11	Arabidopsis thaliana chromosome 2, complete sequence
AT3G23840	1.02770026	4.22E-11	Arabidopsis thaliana HXXXD-type acyl-transferase-like protein mRNA, complete cds
AT4G32350	1.02770026	4.22E-11	Arabidopsis thaliana regulator of Vps4 activity protein mRNA, complete cds
AT3G07730	1.573479817	4.38E-11	Arabidopsis thaliana chromosome 3, complete sequence
AT5G44065	4.437865684	4.54E-11	Arabidopsis thaliana chromosome 5 sequence
AT1G64160	2.453633	4.65E-11	Arabidopsis thaliana chromosome 1 sequence
AT5G22530	2.782513856	4.88E-11	Arabidopsis thaliana chromosome 5 sequence
AT3G14070	1.24883186	4.91E-11	Arabidopsis thaliana chromosome 3, complete sequence
AT4G28703	2.062621775	4.98E-11	Arabidopsis thaliana cupin domain-containing protein mRNA, complete cds
AT1G55265	1.133011103	5.04E-11	Arabidopsis thaliana chromosome 1 sequence
AT3G12500	1.027551832	5.10E-11	Arabidopsis thaliana basic chitinase B mRNA, complete cds
AT5G27060	3.019553053	5.52E-11	Arabidopsis thaliana chromosome 5 sequence
AT5G54650	1.021701519	5.72E-11	Arabidopsis thaliana formin-like protein 5 mRNA, complete cds
AT4G36160	1.777852478	5.73E-11	Arabidopsis thaliana NAC domain containing protein 76 mRNA, complete cds
AT5G46330	1.125115519	5.80E-11	Arabidopsis thaliana LRR receptor-like serine/threonine-protein kinase FLS2 mRNA, complete cds
AT3G25870	1.326961197	5.84E-11	Arabidopsis thaliana chromosome 3, complete sequence
AT4G39190	1.039369686	5.90E-11	Arabidopsis thaliana chromosome 4 sequence
AT4G15765	1.027400915	6.16E-11	Arabidopsis thaliana FAD/NAD(P)-binding oxidoreductase family protein mRNA, complete cds
AT4G32700	1.143014071	6.37E-11	Arabidopsis thaliana MUS308 and mammalian DNA polymerase-like

			protein mRNA, complete cds
AT2G17830	1.460585761	6.43E-11	Arabidopsis thaliana chromosome 2, complete sequence
AT1G61290	5.33095048	6.58E-11	Arabidopsis thaliana syntaxin-124 mRNA, complete cds
AT5G27610	1.348739794	6.83E-11	Arabidopsis thaliana protein ALWAYS EARLY 1 mRNA, complete cds
AT5G04380	6.33095048	6.98E-11	Arabidopsis thaliana S-adenosyl-L-methionine-dependent methyltransferase-like protein mRNA, complete cds
AT4G25420	2.878438276	7.04E-11	Arabidopsis thaliana gibberellin 20 oxidase 1 mRNA, complete cds
AT1G29195	1.314037097	7.08E-11	Arabidopsis thaliana chromosome 1 sequence
AT5G05590	1.297935423	7.14E-11	Arabidopsis thaliana phosphoribosylanthranilate isomerase 2 mRNA, complete cds
AT4G15140	1.00457648	8.00E-11	Arabidopsis thaliana uncharacterized protein mRNA, complete cds
AT1G50750	1.502186665	8.18E-11	Arabidopsis thaliana plant mobile domain family protein mRNA, complete cds
AT3G48770	2.758266309	8.58E-11	Arabidopsis thaliana ATP/DNA binding protein mRNA, complete cds
AT3G50280	1.419943776	8.59E-11	Arabidopsis thaliana chromosome 3, complete sequence
AT1G03940	1.058604414	8.79E-11	Arabidopsis thaliana chromosome 1 sequence
AT1G19025	1.699552407	9.82E-11	Arabidopsis thaliana DNA repair metallo-beta-lactamase family protein mRNA, complete cds
AT5G01760	2.993080842	9.87E-11	Arabidopsis thaliana ENTH/VHS/GAT family protein mRNA, complete cds
AT1G08290	1.05872365	1.06E-10	Arabidopsis thaliana WIP domain protein 3 mRNA, complete cds
AT3G04030	1.146152202	1.12E-10	Arabidopsis thaliana myb family transcription factor mRNA, complete cds
AT5G66780	5.293475775	1.18E-10	Arabidopsis thaliana uncharacterized protein mRNA, complete cds
AT1G27710	6.293475775	1.19E-10	Arabidopsis thaliana chromosome 1 sequence
AT4G12735	3.886850515	1.36E-10	Arabidopsis thaliana chromosome 4 sequence

AT5G57790	1.250662691	1.46E-10	Arabidopsis thaliana uncharacterized protein mRNA, complete cds
AT1G72240	2.23537282	1.50E-10	Arabidopsis thaliana chromosome 1 sequence
AT2G19900	4.367476356	1.53E-10	Arabidopsis thaliana NADP-dependent malic enzyme 1 mRNA, complete cds
AT2G32030	1.630510762	1.57E-10	Arabidopsis thaliana chromosome 2, complete sequence
AT5G65300	1.507891475	1.68E-10	Arabidopsis thaliana chromosome 5 sequence
AT1G19210	3.308582667	1.74E-10	Arabidopsis thaliana chromosome 1 sequence
AT1G62970	1.026439438	1.93E-10	Arabidopsis thaliana chromosome 1 sequence
AT5G46013	5.255001627	2.12E-10	Arabidopsis thaliana chromosome 5 sequence
AT5G22380	2.325656181	2.42E-10	Arabidopsis thaliana NAC domain-containing protein mRNA, complete cds
AT5G17220	1.141763577	2.75E-10	Arabidopsis thaliana glutathione S-transferase phi 12 mRNA, complete cds
AT1G15160	1.630510762	2.77E-10	Arabidopsis thaliana MATE efflux family protein mRNA, complete cds
AT4G34990	1.114443343	2.87E-10	Arabidopsis thaliana transcription factor MYB32 mRNA, complete cds
AT4G39580	1.931377241	3.08E-10	Arabidopsis thaliana chromosome 4 sequence
AT1G74810	1.115182449	3.47E-10	Arabidopsis thaliana boron transporter 5 mRNA, complete cds
AT4G35720	1.993080842	3.52E-10	Arabidopsis thaliana chromosome 4 sequence
AT5G55820	1.314483269	3.61E-10	Arabidopsis thaliana uncharacterized protein mRNA, complete cds
AT2G34080	1.556510181	3.62E-10	Arabidopsis thaliana cysteine proteinase-like protein mRNA, complete cds
AT5G38710	1.038790895	3.95E-10	Arabidopsis thaliana proline dehydrogenase 2 mRNA, complete cds
AT4G35160	1.007413133	3.95E-10	Arabidopsis thaliana O-methyltransferase family protein mRNA, complete cds
AT5G65600	2.195295381	4.18E-10	Arabidopsis thaliana chromosome 5 sequence
AT5G38700	3.075295605	4.32E-10	Arabidopsis thaliana chromosome 5

			sequence
AT2G29300	1.145083935	4.85E-10	Arabidopsis thaliana tropinone reductase-like protein mRNA, complete cds
AT5G54060	1.630510762	4.89E-10	Arabidopsis thaliana chromosome 5 sequence
AT4G24110	1.914303728	4.94E-10	Arabidopsis thaliana chromosome 4 sequence
AT4G09820	2.581601162	5.30E-10	Arabidopsis thaliana transcription factor TT8 mRNA, complete cds
AT4G39830	1.136003212	5.59E-10	Arabidopsis thaliana putative L-ascorbate oxidase mRNA, complete cds
AT3G53790	6.174831278	6.00E-10	Arabidopsis thaliana protein TRF-like 4 mRNA, complete cds
AT1G78160	6.174831278	6.00E-10	Arabidopsis thaliana pumilio 7 mRNA, complete cds
AT5G15660	6.174831278	6.00E-10	Arabidopsis thaliana putative F-box protein mRNA, complete cds
AT4G37720	6.174831278	6.00E-10	Arabidopsis thaliana phytosulfokine 6 precursor mRNA, complete cds
AT4G37690	2.414782071	6.34E-10	Arabidopsis thaliana chromosome 4 sequence
AT2G26160	5.174831278	6.85E-10	Arabidopsis thaliana chromosome 2, complete sequence
AT1G11925	3.471813016	7.15E-10	Arabidopsis thaliana chromosome 1 sequence
AT3G05936	1.708513274	9.43E-10	Arabidopsis thaliana chromosome 3, complete sequence
AT4G30430	2.882049529	9.95E-10	Arabidopsis thaliana tetraspanin9 mRNA, complete cds
AT2G29470	2.468759692	1.01E-09	Arabidopsis thaliana glutathione S-transferase tau 3 mRNA, complete cds
AT5G44310	6.133011103	1.03E-09	Arabidopsis thaliana Late embryogenesis abundant protein family protein mRNA, complete cds
AT1G04370	6.133011103	1.03E-09	Arabidopsis thaliana chromosome 1 sequence
AT3G46700	1.101816481	1.17E-09	Arabidopsis thaliana UDP-glycosyltransferase 76E3 mRNA, complete cds
AT5G45520	2.74598798	1.21E-09	Arabidopsis thaliana chromosome 5 sequence
AT3G15280	5.133011103	1.24E-09	Arabidopsis thaliana uncharacterized protein mRNA, complete cds

AT1G75600	5.133011103	1.24E-09	Arabidopsis thaliana histone H3-like 3 mRNA, complete cds
AT1G08860	5.133011103	1.24E-09	Arabidopsis thaliana protein BONZAI 3 mRNA, complete cds
AT5G62490	3.437865684	1.31E-09	Arabidopsis thaliana HVA22-like protein b mRNA, complete cds
AT3G06520	1.693246517	1.47E-09	Arabidopsis thaliana agenet domain-containing protein mRNA, complete cds
AT4G22470	3.74598798	1.55E-09	Arabidopsis thaliana chromosome 4 sequence
AT4G19191	2.007074114	1.70E-09	Arabidopsis thaliana chromosome 4 sequence
AT4G22960	2.444097638	1.73E-09	Arabidopsis thaliana uncharacterized protein mRNA, complete cds
AT5G67430	2.852903183	1.77E-09	Arabidopsis thaliana GCN5-related N-acetyltransferase (GNAT) family protein mRNA, complete cds
AT4G35690	6.089942381	1.79E-09	Arabidopsis thaliana chromosome 4 sequence
AT4G36600	6.089942381	1.79E-09	Arabidopsis thaliana Late embryogenesis abundant (LEA) protein mRNA, complete cds
AT3G11980	6.089942381	1.79E-09	Arabidopsis thaliana fatty acyl-CoA reductase 2 mRNA, complete cds
AT5G37940	6.089942381	1.79E-09	Arabidopsis thaliana zinc-binding dehydrogenase family protein mRNA, complete cds
AT5G42290	6.089942381	1.79E-09	Arabidopsis thaliana chromosome 5 sequence
AT1G48000	2.367476356	1.84E-09	Arabidopsis thaliana myb domain protein 112 mRNA, complete cds
AT4G18490	2.299304854	1.91E-09	Arabidopsis thaliana uncharacterized protein mRNA, complete cds
AT3G15518	2.299304854	1.91E-09	Arabidopsis thaliana chromosome 3, complete sequence
AT4G11480	2.603543715	2.46E-09	Arabidopsis thaliana putative cysteine-rich receptor-like protein kinase 32 mRNA, complete cds
AT4G04223	2.984147717	2.49E-09	Arabidopsis thaliana ARM repeat superfamily protein mRNA, complete cds
AT1G15330	2.984147717	2.49E-09	Arabidopsis thaliana Cystathionine beta-synthase (CBS) protein mRNA,

			complete cds
AT5G28390	1.424059885	2.75E-09	Arabidopsis thaliana RNA recognition motif-containing protein mRNA, complete cds
AT5G11210	3.708513274	2.86E-09	Arabidopsis thaliana glutamate receptor 2.5 mRNA, complete cds
AT1G66050	1.58744204	3.09E-09	Arabidopsis thaliana E3 ubiquitin-protein ligase ORTHRUS 5 mRNA, complete cds
AT5G07330	6.045548261	3.09E-09	Arabidopsis thaliana uncharacterized protein mRNA, complete cds
AT5G57190	1.115089195	3.20E-09	Arabidopsis thaliana phosphatidylserine decarboxylase 2 mRNA, complete cds
AT4G34400	1.308582667	3.21E-09	Arabidopsis thaliana AP2/B3-like transcriptional factor family protein mRNA, complete cds
AT1G29230	1.289473844	3.22E-09	Arabidopsis thaliana chromosome 1 sequence
AT5G15160	1.46935597	3.59E-09	Arabidopsis thaliana protein banquo 2 mRNA, complete cds
AT3G22410	1.03059792	3.61E-09	Arabidopsis thaliana Sec14p-like phosphatidylinositol transfer family protein mRNA, complete cds
AT1G74590	1.115937589	3.87E-09	Arabidopsis thaliana glutathione S-transferase TAU 10 mRNA, complete cds
AT4G39700	1.776731503	4.16E-09	Arabidopsis thaliana heavy metal transport/detoxification domain-containing protein mRNA, complete cds
AT2G33710	2.576062978	4.28E-09	Arabidopsis thaliana ethylene-responsive transcription factor ERF112 mRNA, complete cds
AT5G46830	2.952438857	4.46E-09	Arabidopsis thaliana chromosome 5 sequence
AT1G24470	1.522595423	4.52E-09	Arabidopsis thaliana beta-ketoacyl reductase 2 mRNA, complete cds
AT4G15236	3.670039126	5.25E-09	Arabidopsis thaliana ABC transporter G family member 43 mRNA, complete cds
AT1G27940	1.586116643	5.45E-09	Arabidopsis thaliana ABC transporter B family member 13 mRNA, complete cds
AT4G02660	1.600137113	6.30E-09	Arabidopsis thaliana Beige/BEACH and WD40 domain-containing protein mRNA, complete cds

AT5G46350	1.759244076	6.54E-09	Arabidopsis thaliana putative WRKY transcription factor 8 mRNA, complete cds
AT2G01430	1.681136835	7.08E-09	Arabidopsis thaliana homeobox-leucine zipper protein ATHB-17 mRNA, complete cds
AT5G09610	4.999744572	7.35E-09	Arabidopsis thaliana chromosome 5 sequence
AT1G30500	1.070785553	7.48E-09	Arabidopsis thaliana nuclear transcription factor Y subunit A-7 mRNA, complete cds
AT3G48850	2.920017379	7.96E-09	Arabidopsis thaliana phosphate transporter 3;2 mRNA, complete cds
AT3G14395	2.451540621	8.14E-09	Arabidopsis thaliana uncharacterized protein mRNA, complete cds
AT1G75580	1.807388524	8.14E-09	Arabidopsis thaliana chromosome 1 sequence
AT5G12330	1.165842495	8.22E-09	Arabidopsis thaliana protein LATERAL ROOT PRIMORDIUM 1 mRNA, complete cds
AT5G41315	1.630510762	8.36E-09	Arabidopsis thaliana transcription factor GLABRA 3 mRNA, complete cds
AT4G20420	2.367476356	8.65E-09	Arabidopsis thaliana chromosome 4 sequence
AT5G22820	1.02234087	8.69E-09	Arabidopsis thaliana ARM repeat superfamily protein mRNA, complete cds
AT5G05365	2.168930677	9.08E-09	Arabidopsis thaliana Heavy metal transport/detoxification superfamily protein mRNA, complete cds
AT4G23450	1.142409801	9.13E-09	Arabidopsis thaliana C3H2C3-type RING E3 Ub ligase mRNA, complete cds
AT5G03204	5.952438857	9.36E-09	Arabidopsis thaliana chromosome 5 sequence
AT2G47520	5.952438857	9.36E-09	Arabidopsis thaliana ethylene-responsive transcription factor ERF071 mRNA, complete cds
AT5G64210	5.952438857	9.36E-09	Arabidopsis thaliana alternative oxidase 2 mRNA, complete cds
AT3G44205.1	1.257052367	9.55E-09	Arabidopsis thaliana chromosome 3, complete sequence
AT1G67980	1.893545168	1.08E-08	Arabidopsis thaliana caffeoyl-CoA 3-O-methyltransferase mRNA, complete

			cds
AT3G53650	1.110503703	1.15E-08	Arabidopsis thaliana chromosome 3, complete sequence
AT2G32120	1.110503703	1.15E-08	Arabidopsis thaliana heat-shock protein 70T-2 mRNA, complete cds
AT3G28945.1	1.682978182	1.25E-08	Arabidopsis thaliana chromosome 3, complete sequence
AT4G23215	1.788772846	1.29E-08	Arabidopsis thaliana chromosome 4 sequence
AT2G17680	4.952438857	1.34E-08	Arabidopsis thaliana chromosome 2, complete sequence
AT3G05770	4.952438857	1.34E-08	Arabidopsis thaliana uncharacterized protein mRNA, complete cds
AT3G03660	4.952438857	1.34E-08	Arabidopsis thaliana WUSCHEL related homeobox 11 mRNA, complete cds
AT4G19720	1.228994402	1.34E-08	Arabidopsis thaliana Glycosyl hydrolase family protein with chitinase insertion domain mRNA, complete cds
AT4G19050	1.303860257	1.38E-08	Arabidopsis thaliana NB-ARC domain-containing disease resistance protein mRNA, complete cds
AT5G14360	2.045548261	1.41E-08	Arabidopsis thaliana ubiquitin-like superfamily protein mRNA, complete cds
AT1G74430	2.092853976	1.47E-08	Arabidopsis thaliana putative transcription factor MYB95 mRNA, complete cds
AT1G66860	1.843055397	1.58E-08	Arabidopsis thaliana class I glutamine amidotransferase-like domain-containing protein mRNA, complete cds
AT5G06080	5.903529257	1.64E-08	Arabidopsis thaliana LOB domain-containing protein 33 mRNA, complete cds
AT2G32130	5.903529257	1.64E-08	Arabidopsis thaliana chromosome 2, complete sequence
AT5G52415	5.903529257	1.64E-08	Arabidopsis thaliana chromosome 5 sequence
AT5G56960	1.267940683	1.66E-08	Arabidopsis thaliana putative transcription factor bHLH041 mRNA, complete cds
AT1G04445	4.045548261	2.04E-08	Arabidopsis thaliana chromosome 1 sequence
AT2G26480	1.553342902	2.23E-08	Arabidopsis thaliana UDP-glucosyl transferase 76D1 mRNA, complete cds

AT3G58070	1.021025942	2.26E-08	Arabidopsis thaliana chromosome 3, complete sequence
AT1G16800	1.149384072	2.36E-08	Arabidopsis thaliana P-loop containing nucleoside triphosphate hydrolases superfamily protein mRNA, complete cds
AT3G08885	2.241945474	2.55E-08	Arabidopsis thaliana chromosome 3, complete sequence
AT2G43510	1.374171009	2.69E-08	Arabidopsis thaliana trypsin inhibitor protein 1 mRNA, complete cds
AT5G57810	5.852903183	2.87E-08	Arabidopsis thaliana tetraspanin15 mRNA, complete cds
AT5G16920	5.852903183	2.87E-08	Arabidopsis thaliana chromosome 5 sequence
AT3G18670	1.885083589	3.03E-08	Arabidopsis thaliana ankyrin repeat-containing protein mRNA, complete cds
AT3G61190	1.012758326	3.06E-08	Arabidopsis thaliana BON association protein 1 mRNA, complete cds
AT5G52390	1.750804996	3.19E-08	Arabidopsis thaliana PAR1 protein mRNA, complete cds
AT3G27270	1.139330408	3.29E-08	Arabidopsis thaliana chromosome 3, complete sequence
AT1G02065	1.263971781	3.43E-08	Arabidopsis thaliana squamosa promoter binding protein-like 8 mRNA, complete cds
AT4G28700	3.999744572	3.77E-08	Arabidopsis thaliana chromosome 4 sequence
AT3G30460	1.999744572	3.78E-08	Arabidopsis thaliana chromosome 3, complete sequence
AT3G45660	2.045548261	3.98E-08	Arabidopsis thaliana probable nitrate excretion transporter 2 mRNA, complete cds
AT5G57060	1.004443058	4.15E-08	Arabidopsis thaliana uncharacterized protein mRNA, complete cds
AT1G66170	4.852903183	4.46E-08	Arabidopsis thaliana PHD finger protein MALE MEIOCYTE DEATH 1 mRNA, complete cds
AT5G62080	4.852903183	4.46E-08	Arabidopsis thaliana protease inhibitor/seed storage/lipid transfer protein (LTP) family protein mRNA, complete cds
AT1G66390	4.852903183	4.46E-08	Arabidopsis thaliana putative transcription factor (MYB90) mRNA,

			MYB90-Col allele, complete cds
AT5G21280	1.630510762	4.62E-08	Arabidopsis thaliana hydroxyproline-rich glycoprotein family protein mRNA, complete cds
AT3G24982	1.272056791	4.95E-08	Arabidopsis thaliana receptor like protein 40 mRNA, complete cds
AT1G69120	1.731439671	5.01E-08	Arabidopsis thaliana Floral homeotic protein APETALA 1 mRNA, complete cds
AT4G21020	5.800435764	5.04E-08	Arabidopsis thaliana Late embryogenesis abundant protein (LEA) family protein mRNA, complete cds
AT3G15270	1.436007738	5.09E-08	Arabidopsis thaliana squamosa promoter-binding-like protein 5 mRNA, complete cds
AT2G47820	1.218384858	5.73E-08	Arabidopsis thaliana uncharacterized protein mRNA, complete cds
AT1G32350	1.534295447	5.97E-08	Arabidopsis thaliana alternative oxidase 1D mRNA, complete cds
AT4G22950	1.50497988	7.71E-08	Arabidopsis thaliana agamous-like MADS-box protein AGL19 mRNA, complete cds
AT1G69430	1.842014867	7.75E-08	Arabidopsis thaliana chromosome 1 sequence
AT2G15490	2.782513856	7.95E-08	Arabidopsis thaliana mRNA for putative glucosyltransferase, complete cds, clone: RAFL14-26-J02
AT5G44540	4.800435764	8.16E-08	Arabidopsis thaliana chromosome 5 sequence
AT3G62610	4.800435764	8.16E-08	Arabidopsis thaliana myb domain protein 11 mRNA, complete cds
AT5G44390	1.308582667	8.53E-08	Arabidopsis thaliana FAD-binding Berberine family protein mRNA, complete cds
AT5G17370	1.285375276	8.58E-08	Arabidopsis thaliana transducin/WD40 domain-containing protein mRNA, complete cds
AT2G40180	1.735863762	8.84E-08	Arabidopsis thaliana protein phosphatase 2C 5 mRNA, complete cds
AT3G53040	5.74598798	8.90E-08	Arabidopsis thaliana putative late embryogenesis abundant protein mRNA, complete cds
AT4G39360	5.74598798	8.90E-08	Arabidopsis thaliana chromosome 4 sequence

AT1G70920	1.430212112	8.95E-08	Arabidopsis thaliana homeobox-leucine zipper protein ATHB-18 mRNA, complete cds
AT2G46750	1.578043342	9.40E-08	Arabidopsis thaliana D-arabinono-1,4-lactone oxidase-like protein mRNA, complete cds
AT1G66725	1.001958993	1.08E-07	Arabidopsis thaliana chromosome 1 sequence
AT5G15290	3.460585761	1.10E-07	Arabidopsis thaliana casparian strip membrane protein 5 mRNA, complete cds
AT3G04040	2.099996045	1.17E-07	Arabidopsis thaliana uncharacterized protein mRNA, complete cds
AT4G17680	1.13574607	1.19E-07	Arabidopsis thaliana SBP (S-ribonuclease binding protein) family protein mRNA, complete cds
AT4G30180	1.545119271	1.23E-07	Arabidopsis thaliana chromosome 4 sequence
AT1G09050	1.500742887	1.36E-07	Arabidopsis thaliana uncharacterized protein mRNA, complete cds
AT4G37140	5.689404451	1.57E-07	Arabidopsis thaliana putative inactive methylesterase 20 mRNA, complete cds
AT1G31290	1.928191311	1.62E-07	Arabidopsis thaliana argonaute 3 mRNA, complete cds
AT1G22370	1.087983528	1.70E-07	Arabidopsis thaliana UDP-glucosyl transferase 85A5 mRNA, complete cds
AT1G59218	1.331852447	1.75E-07	Arabidopsis thaliana putative disease resistance protein RDL6/RF9 mRNA, complete cds
AT5G59730	1.238193339	1.75E-07	Arabidopsis thaliana chromosome 5 sequence
AT1G76690	1.099996045	1.80E-07	Arabidopsis thaliana 12-oxophytodienoate reductase 2 mRNA, complete cds
AT5G12020	2.019553053	1.84E-07	Arabidopsis thaliana chromosome 5 sequence
AT1G07390	1.433818851	1.86E-07	Arabidopsis thaliana receptor like protein 1 mRNA, complete cds
AT5G03840	1.527417269	1.87E-07	Arabidopsis thaliana protein TERMINAL FLOWER 1 mRNA, complete cds
AT1G65500	1.593036057	1.93E-07	Arabidopsis thaliana uncharacterized protein mRNA, complete cds
AT1G54120	2.073028998	1.93E-07	Arabidopsis thaliana chromosome 1 sequence

AT5G47920	1.399185216	1.96E-07	Arabidopsis thaliana chromosome 5 sequence
AT5G43935	3.414782071	2.02E-07	Arabidopsis thaliana flavonol synthase 6 mRNA, complete cds
AT1G14520	1.088616983	2.06E-07	Arabidopsis thaliana inositol oxygenase 1 mRNA, complete cds
AT5G62210	1.157056577	2.67E-07	Arabidopsis thaliana embryo-specific protein ATS3-like protein mRNA, complete cds
AT5G22545	5.630510762	2.80E-07	Arabidopsis thaliana chromosome 5 sequence
AT4G28395	5.630510762	2.80E-07	Arabidopsis thaliana lipid transfer protein-related mRNA, complete cds
AT3G52130	5.630510762	2.80E-07	Arabidopsis thaliana chromosome 3, complete sequence
AT3G15534	5.630510762	2.80E-07	Arabidopsis thaliana chromosome 3, complete sequence
AT1G31880	1.077969739	2.84E-07	Arabidopsis thaliana protein BREVIS RADIX mRNA, complete cds
AT1G71910	1.466391382	3.07E-07	Arabidopsis thaliana chromosome 1 sequence
AT5G06510	1.774900671	3.10E-07	Arabidopsis thaliana nuclear transcription factor Y subunit A-10 mRNA, complete cds
AT1G02310	2.10444195	3.30E-07	Arabidopsis thaliana mannan endo-1,4-beta-mannosidase 1 mRNA, complete cds
AT2G40340	2.852903183	3.66E-07	Arabidopsis thaliana dehydration-responsive element-binding protein 2C mRNA, complete cds
AT1G65690	1.056117503	3.66E-07	Arabidopsis thaliana late embryogenesis abundant (LEA) hydroxyproline-rich glycoprotein mRNA, complete cds
AT3G53450	1.697624958	3.83E-07	Arabidopsis thaliana cytokinin riboside 5'-monophosphate phosphoribohydrolase LOG4 mRNA, complete cds
AT2G04100	1.035352494	3.85E-07	Arabidopsis thaliana MATE efflux family protein mRNA, complete cds
AT1G20870	1.282587459	4.46E-07	Arabidopsis thaliana HSP20-like chaperones superfamily protein mRNA, complete cds
AT2G31945	1.554561909	4.50E-07	Arabidopsis thaliana chromosome 2, complete sequence
AT1G19040	4.630510762	5.07E-07	Arabidopsis thaliana chromosome 1

			sequence
AT5G41710.1	3.045548261	5.14E-07	Arabidopsis thaliana chromosome 5 sequence
AT4G21920	3.045548261	5.14E-07	Arabidopsis thaliana chromosome 4 sequence
AT5G67620	1.218879864	5.18E-07	Arabidopsis thaliana mRNA for hypothetical protein, complete cds, clone: RAFL14-69-L22
AT1G63820	1.314734894	5.32E-07	Arabidopsis thaliana CCT motif family protein mRNA, complete cds
AT1G65730	1.263139696	5.34E-07	Arabidopsis thaliana putative metal-nicotianamine transporter YSL7 mRNA, complete cds
AT1G16160	1.460585761	5.42E-07	Arabidopsis thaliana wall-associated receptor kinase-like 5 mRNA, complete cds
AT4G25000	1.782513856	5.48E-07	Arabidopsis thaliana alpha-amylase 1 mRNA, complete cds
AT4G12005	2.301888015	5.60E-07	Arabidopsis thaliana uncharacterized protein mRNA, complete cds
AT5G07700	2.140705494	5.63E-07	Arabidopsis thaliana myb domain protein 76 mRNA, complete cds
AT1G75770	1.035055888	5.66E-07	Arabidopsis thaliana uncharacterized protein mRNA, complete cds
AT3G17360	1.105419717	5.70E-07	Arabidopsis thaliana phragmoplast orienting kinesin 1 mRNA, complete cds
AT2G05440	1.202052747	6.10E-07	Arabidopsis thaliana glycine-rich protein 9 mRNA, complete cds
AT1G47990	2.811083008	6.51E-07	Arabidopsis thaliana gibberellin 2-oxidase 4 mRNA, complete cds
AT3G11000	1.852903183	6.73E-07	Arabidopsis thaliana DCD (Development and Cell Death) domain protein mRNA, complete cds
AT5G48390	3.318566756	6.77E-07	Arabidopsis thaliana ZIP4-like protein mRNA, complete cds
AT4G00870	1.70090009	6.79E-07	Arabidopsis thaliana transcription factor bHLH14 mRNA, complete cds
AT4G33985	1.431201954	6.84E-07	Arabidopsis thaliana uncharacterized protein mRNA, complete cds
AT1G64065	1.034901017	6.86E-07	Arabidopsis thaliana chromosome 1 sequence
AT4G26120	1.609449147	7.01E-07	Arabidopsis thaliana regulatory protein NPR2 mRNA, complete cds
AT1G71330	1.393471565	7.19E-07	Arabidopsis thaliana non-intrinsic ABC

			protein 5 mRNA, complete cds
AT2G40030	1.080595208	7.44E-07	Arabidopsis thaliana nuclear RNA polymerase D1B mRNA, complete cds
AT4G23515	2.630510762	7.69E-07	Arabidopsis thaliana Toll-Interleukin-1 receptor domain-containing protein mRNA, complete cds
AT5G59720	1.630510762	8.08E-07	Arabidopsis thaliana chromosome 5 sequence
AT2G31345	5.50497988	8.92E-07	Arabidopsis thaliana chromosome 2, complete sequence
AT2G40170	5.50497988	8.92E-07	Arabidopsis thaliana Em-like protein GEA6 mRNA, complete cds
AT1G69500	5.50497988	8.92E-07	Arabidopsis thaliana cytochrome P450, family 704, subfamily B, polypeptide 1 mRNA, complete cds
AT3G01840	2.045548261	9.02E-07	Arabidopsis thaliana LysM-containing receptor-like kinase mRNA, complete cds
AT4G16910.1	2.367476356	9.20E-07	Arabidopsis thaliana chromosome 4 sequence
AT1G03120	2.367476356	9.20E-07	Arabidopsis thaliana responsive to abscisic acid 28 mRNA, complete cds
AT1G74420	1.653230839	9.27E-07	Arabidopsis thaliana fucosyltransferase 3 mRNA, complete cds
AT3G13784	1.569110217	9.28E-07	Arabidopsis thaliana beta-fructofuranosidase, insoluble isoenzyme CWINV5 mRNA, complete cds
AT5G24860	2.109678599	9.37E-07	Arabidopsis thaliana chromosome 5 sequence
AT3G51490	2.109678599	9.37E-07	Arabidopsis thaliana tonoplast monosaccharide transporter3 mRNA, complete cds
AT1G76210	2.267940683	9.50E-07	Arabidopsis thaliana chromosome 1 sequence
AT4G16600	1.790975434	9.69E-07	Arabidopsis thaliana putative glucuronosyltransferase PGSIP8 mRNA, complete cds
AT4G28040	2.768014286	1.16E-06	Arabidopsis thaliana nodulin MtN21-like transporter family protein mRNA, complete cds
AT5G61940	1.704511344	1.21E-06	Arabidopsis thaliana ubiquitin carboxyl-terminal hydrolase-related protein mRNA, complete cds

AT5G44005	1.350402843	1.31E-06	Arabidopsis thaliana chromosome 5 sequence
AT2G02060	1.289473844	1.34E-06	Arabidopsis thaliana putative transcription factor mRNA, complete cds
AT2G38340	3.689404451	1.52E-06	Arabidopsis thaliana chromosome 2, complete sequence
AT1G68500	2.077969739	1.55E-06	Arabidopsis thaliana chromosome 1 sequence
AT1G68250	5.437865684	1.60E-06	Arabidopsis thaliana chromosome 1 sequence
AT1G55525	5.437865684	1.60E-06	Arabidopsis thaliana clone 155459 mRNA sequence
AT3G25655	5.437865684	1.60E-06	Arabidopsis thaliana chromosome 3, complete sequence
AT3G49520	5.437865684	1.60E-06	Arabidopsis thaliana chromosome 3, complete sequence
AT5G45690	5.437865684	1.60E-06	Arabidopsis thaliana uncharacterized protein mRNA, complete cds
AT5G48060	1.180197788	1.79E-06	Arabidopsis thaliana C2 calcium/lipid-binding plant phosphoribosyltransferase family protein mRNA, complete cds
AT2G43870	1.680263797	1.88E-06	Arabidopsis thaliana putative polygalacturonase /pectinase mRNA, complete cds
AT3G21780	1.839097384	1.90E-06	Arabidopsis thaliana chromosome 3, complete sequence
AT3G02410	1.586116643	1.91E-06	Arabidopsis thaliana probable isoprenylcysteine alpha-carbonyl methylesterase ICMEL2 mRNA, complete cds
AT1G01560	1.247182123	1.92E-06	Arabidopsis thaliana mitogen-activated protein kinase 11 mRNA, complete cds
AT4G27654	1.882049529	2.08E-06	Arabidopsis thaliana chromosome 4 sequence
AT5G51920	1.708513274	2.14E-06	Arabidopsis thaliana chromosome 5 sequence
AT2G30750	1.183051785	2.16E-06	Arabidopsis thaliana cytochrome P450 71A12 mRNA, complete cds
AT3G63050	1.607427149	2.21E-06	Arabidopsis thaliana chromosome 3, complete sequence
AT5G18633.1	3.215473263	2.26E-06	Arabidopsis thaliana chromosome 5 sequence
AT2G05510	3.215473263	2.26E-06	Arabidopsis thaliana glycine-rich protein

			mRNA, complete cds
AT4G10580.1	3.215473263	2.26E-06	Arabidopsis thaliana chromosome 4 sequence
AT5G44417	1.129612526	2.31E-06	Arabidopsis thaliana chromosome 5 sequence
AT1G66700	2.548048602	2.36E-06	Arabidopsis thaliana SABATH family methyltransferase PXMT1 mRNA, complete cds
AT1G51190	1.544354118	2.51E-06	Arabidopsis thaliana AP2-like ethylene-responsive transcription factor PLT2 mRNA, complete cds
AT1G09040	1.428017898	2.52E-06	Arabidopsis thaliana uncharacterized protein mRNA, complete cds
AT4G34550	2.408118341	2.56E-06	Arabidopsis thaliana chromosome 4 sequence
AT2G29370	3.630510762	2.81E-06	Arabidopsis thaliana tropinone reductase-like protein mRNA, complete cds
AT3G59740	3.630510762	2.81E-06	Arabidopsis thaliana chromosome 3, complete sequence
AT2G21820	3.630510762	2.81E-06	Arabidopsis thaliana chromosome 2, complete sequence
AT2G33000	3.630510762	2.81E-06	Arabidopsis thaliana ubiquitin-associated (UBA)/TS-N domain-containing protein-like protein mRNA, complete cds
AT5G43840	5.367476356	2.89E-06	Arabidopsis thaliana heat stress transcription factor A-6a mRNA, complete cds
AT3G26614.1	5.367476356	2.89E-06	Arabidopsis thaliana chromosome 3, complete sequence
AT3G25180	5.367476356	2.89E-06	Arabidopsis thaliana cytochrome P450, family 82, subfamily G, polypeptide 1 mRNA, complete cds
AT5G56880	5.367476356	2.89E-06	Arabidopsis thaliana chromosome 5 sequence
AT1G20150	5.367476356	2.89E-06	Arabidopsis thaliana subtilisin-like serine endopeptidase family protein mRNA, complete cds
AT5G60630	5.367476356	2.89E-06	Arabidopsis thaliana chromosome 5 sequence
AT2G31210	5.367476356	2.89E-06	Arabidopsis thaliana transcription factor bHLH91 mRNA, complete cds
AT5G07230	5.367476356	2.89E-06	Arabidopsis thaliana Tapetum-specific protein A9 mRNA, complete cds

AT2G33780	5.367476356	2.89E-06	Arabidopsis thaliana chromosome 2, complete sequence
AT1G03790	5.367476356	2.89E-06	Arabidopsis thaliana chromosome 1 sequence
AT3G10986	2.903529257	3.00E-06	Arabidopsis thaliana uncharacterized protein mRNA, complete cds
AT3G50290	1.811083008	3.03E-06	Arabidopsis thaliana chromosome 3, complete sequence
AT5G59510	1.397020632	3.15E-06	Arabidopsis thaliana chromosome 5 sequence
AT5G05490	4.437865684	3.22E-06	Arabidopsis thaliana RAD21-like protein SYN1 mRNA, complete cds
AT1G57560	1.211558213	3.26E-06	Arabidopsis thaliana myb domain protein 50 mRNA, complete cds
AT1G72260	1.852903183	3.35E-06	Arabidopsis thaliana thionin 2.1 mRNA, complete cds
AT4G16000	1.26235965	3.36E-06	Arabidopsis thaliana chromosome 4 sequence
AT3G61900	1.151342925	3.55E-06	Arabidopsis thaliana SAUR-like auxin-responsive protein mRNA, complete cds
AT5G16960	2.677816477	3.63E-06	Arabidopsis thaliana zinc-binding dehydrogenase family protein mRNA, complete cds
AT5G09430	1.899697395	3.67E-06	Arabidopsis thaliana alpha/beta-Hydrolases superfamily protein mRNA, complete cds
AT2G26390	1.117338944	3.91E-06	Arabidopsis thaliana serpin-Z3 mRNA, complete cds
AT1G52450	1.952438857	3.96E-06	Arabidopsis thaliana ubiquitin carboxyl-terminal hydrolase-related protein mRNA, complete cds
AT3G50770	1.33095048	4.00E-06	Arabidopsis thaliana chromosome 3, complete sequence
AT1G71520	3.161025479	4.13E-06	Arabidopsis thaliana chromosome 1 sequence
AT4G01060	2.367476356	4.41E-06	Arabidopsis thaliana CAPRICE-like MYB3 mRNA, complete cds
AT1G36622	2.367476356	4.41E-06	Arabidopsis thaliana uncharacterized protein mRNA, complete cds
AT1G29290	2.081172171	4.43E-06	Arabidopsis thaliana chromosome 1 sequence
AT3G09480	2.255001627	4.55E-06	Arabidopsis thaliana chromosome 3, complete sequence

AT5G61950	1.377391825	4.66E-06	Arabidopsis thaliana ubiquitin carboxyl-terminal hydrolase-related protein mRNA, complete cds
AT5G53870	1.045548261	5.10E-06	Arabidopsis thaliana early nodulin-like protein 1 mRNA, complete cds
AT5G13380	5.293475775	5.23E-06	Arabidopsis thaliana auxin-responsive GH3 family protein mRNA, complete cds
AT4G13480	5.293475775	5.23E-06	Arabidopsis thaliana myb domain protein 79 mRNA, complete cds
AT4G18190	5.293475775	5.23E-06	Arabidopsis thaliana purine permease 6 mRNA, complete cds
AT4G01895	1.432571385	5.28E-06	Arabidopsis thaliana chromosome 4 sequence
AT1G67270	2.852903183	5.39E-06	Arabidopsis thaliana zinc-finger domain of monoamine-oxidase A repressor R1 protein mRNA, complete cds
AT3G49130	2.852903183	5.39E-06	Arabidopsis thaliana SWAP (Suppressor-of-White-APricot)/surp RNA-binding domain-containing protein mRNA, complete cds
AT1G56060	2.852903183	5.39E-06	Arabidopsis thaliana uncharacterized protein mRNA, complete cds
AT5G49700	1.058845084	5.47E-06	Arabidopsis thaliana chromosome 5 sequence
AT5G11410	1.499266229	5.79E-06	Arabidopsis thaliana protein kinase family protein mRNA, complete cds
AT4G08145.1	4.367476356	5.98E-06	Arabidopsis thaliana chromosome 4 sequence
AT5G27845.1	4.367476356	5.98E-06	Arabidopsis thaliana chromosome 5 sequence
AT4G38650	1.138657666	6.04E-06	Arabidopsis thaliana glycosyl hydrolase family 10 protein mRNA, complete cds
AT1G77640	1.158442318	6.32E-06	Arabidopsis thaliana chromosome 1 sequence
AT3G20660	1.158442318	6.32E-06	Arabidopsis thaliana organic cation/carnitine transporter4 mRNA, complete cds
AT1G21550	1.286556361	7.06E-06	Arabidopsis thaliana chromosome 1 sequence
AT4G10500	1.073562638	7.10E-06	Arabidopsis thaliana oxidoreductase, 2OG-Fe(II) oxygenase family protein mRNA, complete cds
AT2G21510	2.045548261	7.33E-06	Arabidopsis thaliana DNAJ heat shock

			N-terminal domain-containing protein mRNA, complete cds
AT1G08440	3.10444195	7.53E-06	Arabidopsis thaliana aluminum-activated malate transporter 2 mRNA, complete cds
AT5G37490	3.10444195	7.53E-06	Arabidopsis thaliana chromosome 5 sequence
AT1G05560	3.10444195	7.53E-06	Arabidopsis thaliana chromosome 1 sequence
AT5G01570	2.325656181	7.56E-06	Arabidopsis thaliana uncharacterized protein mRNA, complete cds
AT2G31940	2.325656181	7.56E-06	Arabidopsis thaliana chromosome 2, complete sequence
AT2G37880	2.215473263	7.69E-06	Arabidopsis thaliana chromosome 2, complete sequence
AT5G23810	1.074117414	8.62E-06	Arabidopsis thaliana putative amino acid permease 7 mRNA, complete cds
AT4G01535	5.215473263	9.52E-06	Arabidopsis thaliana uncharacterized protein mRNA, complete cds
AT3G21410	5.215473263	9.52E-06	Arabidopsis thaliana chromosome 3, complete sequence
AT2G33440	1.019553053	9.54E-06	Arabidopsis thaliana RNA recognition motif-containing protein mRNA, complete cds
AT2G16910	2.800435764	9.64E-06	Arabidopsis thaliana transcription factor ABORTED MICROSPORES mRNA, complete cds
AT5G42325	2.800435764	9.64E-06	Arabidopsis thaliana transcription factor IIS protein mRNA, complete cds
AT5G50800	1.106948806	9.73E-06	Arabidopsis thaliana bidirectional sugar transporter SWEET13 mRNA, complete cds
AT1G13530	1.300805317	1.02E-05	Arabidopsis thaliana uncharacterized protein mRNA, complete cds
AT2G30640.1	1.493007238	1.03E-05	Arabidopsis thaliana chromosome 2, complete sequence
AT1G59865	1.689404451	1.06E-05	Arabidopsis thaliana uncharacterized protein mRNA, complete cds
AT4G23880	1.007074114	1.07E-05	Arabidopsis thaliana chromosome 4 sequence
AT5G09470	4.293475775	1.11E-05	Arabidopsis thaliana dicarboxylate carrier 3 mRNA, complete cds
AT2G18190	4.293475775	1.11E-05	Arabidopsis thaliana P-loop containing nucleoside triphosphate hydrolases

			superfamily protein mRNA, complete cds
AT3G09640	4.293475775	1.11E-05	Arabidopsis thaliana L-ascorbate peroxidase 2 mRNA, complete cds
AT5G17780	2.581601162	1.13E-05	Arabidopsis thaliana hydrolase, alpha/beta fold family protein mRNA, complete cds
AT4G30830	1.346717796	1.21E-05	Arabidopsis thaliana uncharacterized protein mRNA, complete cds
AT5G40820	1.308582667	1.23E-05	Arabidopsis thaliana serine/threonine-protein kinase ATR mRNA, complete cds
AT4G36515	1.308582667	1.23E-05	Arabidopsis thaliana uncharacterized protein mRNA, complete cds
AT5G51810	2.414782071	1.24E-05	Arabidopsis thaliana gibberellin 20 oxidase 2 mRNA, complete cds
AT5G22540	2.414782071	1.24E-05	Arabidopsis thaliana chromosome 5 sequence
AT4G16270	2.414782071	1.24E-05	Arabidopsis thaliana peroxidase 40 mRNA, complete cds
AT5G64060	2.414782071	1.24E-05	Arabidopsis thaliana NAC domain containing protein 103 mRNA, complete cds
AT2G02930	2.282587459	1.29E-05	Arabidopsis thaliana glutathione S-transferase F3 mRNA, complete cds
AT2G22790	1.006377664	1.30E-05	Arabidopsis thaliana chromosome 2, complete sequence
AT1G03990	1.045548261	1.35E-05	Arabidopsis thaliana long-chain-alcohol oxidase FAO1 mRNA, complete cds
AT4G08570	1.761755295	1.36E-05	Arabidopsis thaliana heavy-metal-associated domain-containing protein mRNA, complete cds
AT5G61850	3.045548261	1.37E-05	Arabidopsis thaliana protein LEAFY mRNA, complete cds
AT5G07640	3.045548261	1.37E-05	Arabidopsis thaliana RING/U-box superfamily protein mRNA, complete cds
AT1G17990	1.060195037	1.44E-05	Arabidopsis thaliana putative 12-oxophytodienoate reductase-like protein 2A mRNA, complete cds
AT1G01700	1.249081655	1.47E-05	Arabidopsis thaliana Rop guanine nucleotide exchange factor 2 mRNA, complete cds

AT5G39090	1.12896427	1.51E-05	Arabidopsis thaliana chromosome 5 sequence
AT1G15460	1.468759692	1.55E-05	Arabidopsis thaliana boron transporter 4 mRNA, complete cds
AT3G23220	5.133011103	1.74E-05	Arabidopsis thaliana chromosome 3, complete sequence
AT1G28360	1.486120853	1.83E-05	Arabidopsis thaliana chromosome 1 sequence
AT5G24280	1.031334402	1.85E-05	Arabidopsis thaliana gamma-irradiation and mitomycin c induced 1 mRNA, complete cds
AT1G18560	1.031334402	1.85E-05	Arabidopsis thaliana BED zinc finger and hAT dimerization domain-containing protein mRNA, complete cds
AT1G34047	2.530975089	1.98E-05	Arabidopsis thaliana defensin-like protein 208 mRNA, complete cds
AT5G02430	1.045548261	1.99E-05	Arabidopsis thaliana transducin/WD40 domain-containing protein mRNA, complete cds
AT3G25240	4.215473263	2.08E-05	Arabidopsis thaliana chromosome 3, complete sequence
AT3G20520	2.367476356	2.13E-05	Arabidopsis thaliana glycerophosphodiester phosphodiesterase-like protein mRNA, complete cds
AT2G29110	1.730046436	2.15E-05	Arabidopsis thaliana glutamate receptor 2.8 mRNA, complete cds
AT3G02493	2.238193339	2.19E-05	Arabidopsis thaliana chromosome 3, complete sequence
AT2G33240	1.444097638	2.33E-05	Arabidopsis thaliana myosin XI D mRNA, complete cds
AT4G38560	1.155172753	2.33E-05	Arabidopsis thaliana phospholipase like protein (PEARLI 4) mRNA, complete cds
AT3G07250	2.984147717	2.49E-05	Arabidopsis thaliana nuclear transport factor 2 and RNA recognition motif domain-containing protein mRNA, complete cds
AT5G67110	1.016979109	2.53E-05	Arabidopsis thaliana transcription factor ALC mRNA, complete cds
AT5G52020	1.267940683	2.59E-05	Arabidopsis thaliana chromosome 5 sequence
AT2G47560	1.077969739	2.77E-05	Arabidopsis thaliana chromosome 2, complete sequence

AT5G42710	1.404629355	2.93E-05	Arabidopsis thaliana uncharacterized protein mRNA, complete cds
AT5G10990	2.689404451	3.06E-05	Arabidopsis thaliana chromosome 5 sequence
AT1G75790	5.045548261	3.19E-05	Arabidopsis thaliana SKU5 similar 18 protein mRNA, complete cds
AT5G22470	5.045548261	3.19E-05	Arabidopsis thaliana poly [ADP-ribose] polymerase 3 mRNA, complete cds
AT3G10100.1	5.045548261	3.19E-05	Arabidopsis thaliana At4g20730 mRNA, complete cds
AT2G36750	5.045548261	3.19E-05	Arabidopsis thaliana chromosome 2, complete sequence
AT4G34210	5.045548261	3.19E-05	Arabidopsis thaliana chromosome 4 sequence
AT1G58430	1.933073532	3.24E-05	Arabidopsis thaliana anther-specific proline-rich protein RXF26 mRNA, complete cds
AT2G25470	1.933073532	3.24E-05	Arabidopsis thaliana receptor like protein 21 mRNA, complete cds
AT5G44990	3.367476356	3.31E-05	Arabidopsis thaliana Glutathione S-transferase family protein mRNA, complete cds
AT2G35290	2.004906277	3.47E-05	Arabidopsis thaliana chromosome 2, complete sequence
AT4G09490	1.186904111	3.56E-05	Arabidopsis thaliana polynucleotidyl transferase, ribonuclease H-like superfamily protein mRNA, complete cds
AT2G23010	1.097387193	3.58E-05	Arabidopsis thaliana serine carboxypeptidase-like 9 mRNA, complete cds
AT5G15430	2.318566756	3.67E-05	Arabidopsis thaliana chromosome 5 sequence
AT1G33102	1.322388467	3.73E-05	Arabidopsis thaliana chromosome 1 sequence
AT4G37890	1.322388467	3.73E-05	Arabidopsis thaliana chromosome 4 sequence
AT3G11440	1.117698047	3.79E-05	Arabidopsis thaliana myb domain protein 65 mRNA, complete cds
AT1G03020	1.061850074	3.83E-05	Arabidopsis thaliana chromosome 1 sequence
AT4G20730.1	1.737425966	3.84E-05	Arabidopsis thaliana mRNA for putative protein, complete cds, clone: RAFL24-04-L03

AT1G20350	1.737425966	3.84E-05	Arabidopsis thaliana chromosome 1 sequence
AT5G59230	4.133011103	3.90E-05	Arabidopsis thaliana transcription factor-related protein mRNA, complete cds
AT1G21525	4.133011103	3.90E-05	Arabidopsis thaliana chromosome 1 sequence
AT3G63320	4.133011103	3.90E-05	Arabidopsis thaliana putative protein phosphatase 2C 50 mRNA, complete cds
AT5G01550	1.600137113	4.00E-05	Arabidopsis thaliana chromosome 5 sequence
AT1G76740	1.600137113	4.00E-05	Arabidopsis thaliana uncharacterized protein mRNA, complete cds
AT3G01600	1.782513856	4.32E-05	Arabidopsis thaliana NAC domain containing protein 44 mRNA, complete cds
AT1G02980	2.920017379	4.51E-05	Arabidopsis thaliana cullin 2 mRNA, complete cds
AT1G72100	2.920017379	4.51E-05	Arabidopsis thaliana late embryogenesis abundant domain-containing protein mRNA, complete cds
AT1G79360	1.834044156	4.81E-05	Arabidopsis thaliana chromosome 1 sequence
AT1G22490	1.195295381	5.23E-05	Arabidopsis thaliana transcription factor bHLH94 mRNA, complete cds
AT5G56050	1.045548261	5.29E-05	Arabidopsis thaliana chromosome 5 sequence
AT2G37070	1.543047921	5.29E-05	Arabidopsis thaliana uncharacterized protein mRNA, complete cds
AT5G21150	1.664458094	5.32E-05	Arabidopsis thaliana argonaute 9 mRNA, complete cds
AT3G59580	1.34322881	5.37E-05	Arabidopsis thaliana nodule inception protein-like protein 9 mRNA, complete cds
AT2G24430	1.260561152	5.44E-05	Arabidopsis thaliana NAC domain containing protein 38 mRNA, complete cds
AT4G34850	1.963086101	5.71E-05	Arabidopsis thaliana protein LESS ADHESIVE POLLEN 5 mRNA, complete cds
AT4G23150	1.963086101	5.71E-05	Arabidopsis thaliana cysteine-rich receptor-like protein kinase 7 mRNA, complete cds
AT5G15250	4.952438857	5.89E-05	Arabidopsis thaliana ATP-dependent zinc

			metalloprotease FTSH 6 mRNA, complete cds
AT1G32880	4.952438857	5.89E-05	Arabidopsis thaliana armadillo/beta-catenin-like repeats-containing protein mRNA, complete cds
AT2G13640	4.952438857	5.89E-05	Arabidopsis thaliana chromosome 2, complete sequence
AT1G32560	4.952438857	5.89E-05	Arabidopsis thaliana Late embryogenesis abundant protein, group 1 protein mRNA, complete cds
AT3G59030	4.952438857	5.89E-05	Arabidopsis thaliana protein TRANSPARENT TESTA 12 mRNA, complete cds
AT5G43770	3.293475775	6.12E-05	Arabidopsis thaliana chromosome 5 sequence
AT2G27880	3.293475775	6.12E-05	Arabidopsis thaliana argonaute 5 mRNA, complete cds
AT3G54150	1.35487632	6.44E-05	Arabidopsis thaliana S-adenosyl-L-methionine-dependent methyltransferase-like protein mRNA, complete cds
AT5G41610	1.231961386	6.49E-05	Arabidopsis thaliana cation/H(+) antiporter 18 mRNA, complete cds
AT1G51820	1.490333104	6.86E-05	Arabidopsis thaliana putative LRR receptor-like serine/threonine protein kinase mRNA, complete cds
AT4G11910	1.029060139	7.26E-05	Arabidopsis thaliana protein STAY-GREEN2 mRNA, complete cds
AT2G45940	4.045548261	7.31E-05	Arabidopsis thaliana uncharacterized protein mRNA, complete cds
AT5G53895	4.045548261	7.31E-05	Arabidopsis thaliana uncharacterized protein mRNA, complete cds
AT2G04135.1	4.045548261	7.31E-05	Arabidopsis thaliana chromosome 2, complete sequence
AT1G16515	1.318566756	7.89E-05	Arabidopsis thaliana chromosome 1 sequence
AT1G65240	1.852903183	8.55E-05	Arabidopsis thaliana aspartyl protease family protein mRNA, complete cds
AT4G13992	1.852903183	8.55E-05	Arabidopsis thaliana chromosome 4 sequence
AT4G16146	1.178814792	9.03E-05	Arabidopsis thaliana cAMP-regulated phosphoprotein 19-related protein mRNA, complete cds

AT5G53990	1.209935079	9.32E-05	Arabidopsis thaliana chromosome 5 sequence
AT3G54340	1.920017379	9.35E-05	Arabidopsis thaliana Floral homeotic protein APETALA 3 mRNA, complete cds
AT1G07160	1.920017379	9.35E-05	Arabidopsis thaliana putative protein phosphatase 2C 2 mRNA, complete cds
AT5G23000	1.667036638	9.54E-05	Arabidopsis thaliana transcription factor RAX1 mRNA, complete cds
AT5G64395	2.569110217	9.62E-05	Arabidopsis thaliana chromosome 5 sequence
AT1G24260	1.460585761	0.000103838	Arabidopsis thaliana MADs box transcription factor SEPALLATA3 mRNA, complete cds
AT1G12030	2.367476356	0.000104383	Arabidopsis thaliana uncharacterized protein mRNA, complete cds
AT1G66380	4.852903183	0.000109223	Arabidopsis thaliana transcription factor MYB114 mRNA, complete cds
AT1G70390	4.852903183	0.000109223	Arabidopsis thaliana putative F-box protein mRNA, complete cds
AT3G13220	4.852903183	0.000109223	Arabidopsis thaliana ABC transporter G family member 26 mRNA, complete cds
AT4G25380	4.852903183	0.000109223	Arabidopsis thaliana chromosome 4 sequence
AT4G36490	4.852903183	0.000109223	Arabidopsis thaliana protein SEC14-like 12 mRNA, complete cds
AT3G63052	4.852903183	0.000109223	Arabidopsis thaliana chromosome 3, complete sequence
AT2G42340	4.852903183	0.000109223	Arabidopsis thaliana uncharacterized protein mRNA, complete cds
AT5G06820	1.396045508	0.000110128	Arabidopsis thaliana STRUBBELIG-receptor family 2 mRNA, complete cds
AT1G62440	1.215473263	0.000112889	Arabidopsis thaliana leucine-rich repeat extensin-like protein 2 mRNA, complete cds
AT5G36910	3.215473263	0.000113095	Arabidopsis thaliana thionin 2.2 mRNA, complete cds
AT4G21380	1.251999139	0.000114897	Arabidopsis thaliana receptor kinase 3 mRNA, complete cds
AT3G10180	1.293475775	0.000115338	Arabidopsis thaliana kinesin motor protein-related protein mRNA, complete cds
AT3G07273	1.756041644	0.000123189	Arabidopsis thaliana chromosome 3,

			complete sequence
AT1G27720	1.595745344	0.000128923	Arabidopsis thaliana TBP-associated factor 4B mRNA, complete cds
AT5G37800	1.595745344	0.000128923	Arabidopsis thaliana RHD SIX-LIKE 1 mRNA, complete cds
AT2G37025	1.02784626	0.000130995	Arabidopsis thaliana protein TRF-like 8 mRNA, complete cds
AT5G54400	1.412330592	0.000131333	Arabidopsis thaliana S-adenosyl-L-methionine-dependent methyltransferase-like protein mRNA, complete cds
AT1G70185	3.952438857	0.000137492	Arabidopsis thaliana chromosome 1 sequence
AT3G63095	3.952438857	0.000137492	Arabidopsis thaliana chromosome 3, complete sequence
AT5G27220	3.952438857	0.000137492	Arabidopsis thaliana Frigida-like protein mRNA, complete cds
AT2G04460.1	3.952438857	0.000137492	Arabidopsis thaliana chromosome 2, complete sequence
AT4G26950	1.10783254	0.000141093	Arabidopsis thaliana uncharacterized protein mRNA, complete cds
AT1G74350	1.430212112	0.00015634	Arabidopsis thaliana chromosome 1 sequence
AT4G28085	1.19238965	0.000161538	Arabidopsis thaliana uncharacterized protein mRNA, complete cds
AT4G01490.1	1.086190246	0.000161817	Arabidopsis thaliana chromosome 4 sequence
AT3G61450	1.952438857	0.00016538	Arabidopsis thaliana syntaxin-73 mRNA, complete cds
AT2G20470	2.045548261	0.00017574	Arabidopsis thaliana AGC (cAMP-dependent, cGMP-dependent and protein kinase C) kinase family protein mRNA, complete cds
AT5G57320	1.13574607	0.000181052	Arabidopsis thaliana putative villin mRNA, complete cds
AT3G07274	1.13574607	0.000181052	Arabidopsis thaliana chromosome 3, complete sequence
AT5G01320	1.449938516	0.000185721	Arabidopsis thaliana pyruvate decarboxylase 4 mRNA, complete cds
AT3G14225	1.382583249	0.000195969	Arabidopsis thaliana GDSL esterase/lipase 4 mRNA, complete cds
AT4G21120	1.234582086	0.00020079	Arabidopsis thaliana amino acid transporter 1 mRNA, complete cds
AT2G39725	1.325656181	0.000201441	Arabidopsis thaliana LYR family of Fe/S

			cluster biogenesis protein mRNA, complete cds
AT5G06805.1	4.74598798	0.00020366	Arabidopsis thaliana chromosome 5 sequence
AT4G11590	4.74598798	0.00020366	Arabidopsis thaliana chromosome 4 sequence
AT3G09950	4.74598798	0.00020366	Arabidopsis thaliana chromosome 3, complete sequence
AT5G45116.1	4.74598798	0.00020366	Arabidopsis thaliana chromosome 5 sequence
AT2G20555	4.74598798	0.00020366	Arabidopsis thaliana chromosome 2, complete sequence
AT1G45140.1	3.133011103	0.000208695	Arabidopsis thaliana chromosome 1 sequence
AT1G73510	3.133011103	0.000208695	Arabidopsis thaliana chromosome 1 sequence
AT1G72660	3.133011103	0.000208695	Arabidopsis thaliana developmentally regulated G-protein 2 mRNA, complete cds
AT2G42150	1.471813016	0.000220084	Arabidopsis thaliana DNA-binding bromodomain-containing protein mRNA, complete cds
AT1G79680	1.768014286	0.000221085	Arabidopsis thaliana wall-associated receptor kinase-like 10 mRNA, complete cds
AT4G11170	1.066012364	0.000224961	Arabidopsis thaliana putative disease resistance protein mRNA, complete cds
AT1G63730	1.066012364	0.000224961	Arabidopsis thaliana TIR-NBS-LRR class disease resistance protein mRNA, complete cds
AT3G04640	1.82981957	0.000246743	Arabidopsis thaliana chromosome 3, complete sequence
AT3G46680	3.852903183	0.000259126	Arabidopsis thaliana UDP-glycosyltransferase 76E6 mRNA, complete cds
AT1G78390	3.852903183	0.000259126	Arabidopsis thaliana chromosome 1 sequence
AT3G60930.1	3.852903183	0.000259126	Arabidopsis thaliana At3g60930 mRNA for unknown protein, complete cds, clone: RAFL21-22-C17
AT2G33080	2.708513274	0.000264441	Arabidopsis thaliana chromosome 2, complete sequence
AT5G55020	2.708513274	0.000264441	Arabidopsis thaliana myb domain protein 120 mRNA, complete cds

AT3G04050	2.708513274	0.000264441	Arabidopsis thaliana pyruvate kinase mRNA, complete cds
AT5G52860	1.008073556	0.000266498	Arabidopsis thaliana chromosome 5 sequence
AT4G23240	1.630510762	0.000268836	Arabidopsis thaliana cysteine-rich receptor-like protein kinase 16 mRNA, complete cds
AT3G48790	1.903529257	0.000271151	Arabidopsis thaliana Pyridoxal phosphate (PLP)-dependent transferases superfamily protein mRNA, complete cds
AT1G32690	1.066609877	0.0002742	Arabidopsis thaliana chromosome 1 sequence
AT4G33070	1.173303809	0.000279808	Arabidopsis thaliana pyruvate decarboxylase 1 mRNA, complete cds
AT4G21200	1.993080842	0.000292055	Arabidopsis thaliana gibberellin 2-beta-dioxygenase 8 mRNA, complete cds
AT1G53625	2.437865684	0.000297379	Arabidopsis thaliana chromosome 1 sequence
AT3G13900	2.10444195	0.000306293	Arabidopsis thaliana putative phospholipid-transporting ATPase 7 mRNA, complete cds
AT3G62455.1	1.673579484	0.000309204	Arabidopsis thaliana chromosome 3, complete sequence
AT1G61275	2.247182123	0.000309715	Arabidopsis thaliana chromosome 1 sequence
AT2G33100	2.247182123	0.000309715	Arabidopsis thaliana cellulose synthase-like protein D1 mRNA, complete cds
AT1G20310	1.145083935	0.00032642	Arabidopsis thaliana chromosome 1 sequence
AT4G23200	1.723620167	0.00035254	Arabidopsis thaliana putative cysteine-rich receptor-like protein kinase 12 mRNA, complete cds
AT5G64890	1.554561909	0.000359071	Arabidopsis thaliana elicitor peptide 2 mRNA, complete cds
AT5G66020	4.630510762	0.000381943	Arabidopsis thaliana phosphoinositide phosphatase SAC6 mRNA, complete cds
AT4G37420	4.630510762	0.000381943	Arabidopsis thaliana chromosome 4 sequence
AT5G09210	4.630510762	0.000381943	Arabidopsis thaliana GC-rich sequence DNA-binding factor-like protein mRNA, complete cds

AT3G17980	4.630510762	0.000381943	Arabidopsis thaliana calcium-dependent lipid-binding domain-containing protein mRNA, complete cds
AT5G09876	4.630510762	0.000381943	Arabidopsis thaliana uncharacterized protein mRNA, complete cds
AT4G27140	4.630510762	0.000381943	Arabidopsis thaliana chromosome 4 sequence
AT5G65274	4.630510762	0.000381943	Arabidopsis thaliana ARP2/3 complex subunit p16-Arc mRNA, complete cds
AT5G62800	4.630510762	0.000381943	Arabidopsis thaliana E3 ubiquitin-protein ligase SINA-like 11 mRNA, complete cds
AT5G40348	4.630510762	0.000381943	Arabidopsis thaliana clone 102435 mRNA sequence
AT2G18540	4.630510762	0.000381943	Arabidopsis thaliana cupin family protein mRNA, complete cds
AT5G60320	4.630510762	0.000381943	Arabidopsis thaliana chromosome 5 sequence
AT5G41755.1	4.630510762	0.000381943	Arabidopsis thaliana chromosome 5 sequence
AT2G36540	3.045548261	0.000384487	Arabidopsis thaliana Haloacid dehalogenase-like hydrolase (HAD) superfamily protein mRNA, complete cds
AT3G22560	1.460585761	0.000395216	Arabidopsis thaliana chromosome 3, complete sequence
AT3G21520	1.782513856	0.000397559	Arabidopsis thaliana chromosome 3, complete sequence
AT1G29560	1.183051785	0.00041374	Arabidopsis thaliana zinc finger C-x8-C-x5-C-x3-H type family protein mRNA, complete cds
AT1G48580	1.589868778	0.000418933	Arabidopsis thaliana uncharacterized protein mRNA, complete cds
AT4G22505	1.321182704	0.000429756	Arabidopsis thaliana chromosome 4 sequence
AT3G12970	1.092853976	0.000435042	Arabidopsis thaliana chromosome 3, complete sequence
AT3G48660	1.852903183	0.000442076	Arabidopsis thaliana uncharacterized protein mRNA, complete cds
AT5G67050	1.852903183	0.000442076	Arabidopsis thaliana alpha/beta-Hydrolases superfamily protein mRNA, complete cds
AT3G28500	1.120836389	0.000460848	Arabidopsis thaliana chromosome 3, complete sequence

AT1G22380	1.486120853	0.000468053	Arabidopsis thaliana UDP-glucosyl transferase 85A3 mRNA, complete cds
AT1G69485	1.486120853	0.000468053	Arabidopsis thaliana ribosomal L32p protein family mRNA, complete cds
AT4G35640	1.486120853	0.000468053	Arabidopsis thaliana serine acetyltransferase 3;2 mRNA, complete cds
AT5G18636	2.630510762	0.000473648	Arabidopsis thaliana chromosome 5 sequence
AT3G56780	2.630510762	0.000473648	Arabidopsis thaliana putative F-box/FBD/LRR-repeat protein mRNA, complete cds
AT3G49055	2.630510762	0.000473648	Arabidopsis thaliana chromosome 3 hypothetical protein AT3G49055 mRNA, complete cds, alternatively spliced
AT5G49690	1.938633057	0.000482647	Arabidopsis thaliana chromosome 5 sequence
AT4G22485	1.152463465	0.000484036	Arabidopsis thaliana chromosome 4 sequence
AT1G64572	1.630510762	0.000485748	Arabidopsis thaliana chromosome 1 sequence
AT4G01975.1	1.630510762	0.000485748	Arabidopsis thaliana chromosome 4 sequence
AT1G07850	3.74598798	0.000489384	Arabidopsis thaliana uncharacterized protein mRNA, complete cds
AT5G47850	2.045548261	0.000514165	Arabidopsis thaliana chromosome 5 sequence
AT5G44630	2.367476356	0.000519432	Arabidopsis thaliana sesquiterpene synthase mRNA, complete cds
AT2G02000	2.183051785	0.000529498	Arabidopsis thaliana glutamate decarboxylase 3 mRNA, complete cds
AT5G20240	1.094457862	0.000530684	Arabidopsis thaliana Floral homeotic protein PISTILLATA mRNA, complete cds
AT1G54330	1.003728086	0.000589589	Arabidopsis thaliana NAC domain containing protein 20 mRNA, complete cds
AT2G36660	1.350402843	0.0006226	Arabidopsis thaliana poly(A) binding protein 7 mRNA, complete cds
AT3G42800	1.733604255	0.000636769	Arabidopsis thaliana uncharacterized protein mRNA, complete cds
AT5G48100	1.548048602	0.000649011	Arabidopsis thaliana laccase-15 mRNA, complete cds
AT3G62730	1.548048602	0.000649011	Arabidopsis thaliana uncharacterized

			protein mRNA, complete cds
AT4G28280	2.952438857	0.000706943	Arabidopsis thaliana chromosome 4 sequence
AT4G25580	2.952438857	0.000706943	Arabidopsis thaliana CAP160 protein mRNA, complete cds
AT3G01319	2.952438857	0.000706943	Arabidopsis thaliana chromosome 3, complete sequence
AT2G23060	1.447646705	0.000711293	Arabidopsis thaliana acyl-CoA N-acyltransferase-like protein mRNA, complete cds
AT3G46090	1.800435764	0.000716441	Arabidopsis thaliana chromosome 3, complete sequence
AT4G32630	4.50497988	0.000720674	Arabidopsis thaliana ArfGap/RecO-like zinc finger domain-containing protein mRNA, complete cds
AT2G24210	4.50497988	0.000720674	Arabidopsis thaliana terpene synthase 10 mRNA, complete cds
AT1G27080	4.50497988	0.000720674	Arabidopsis thaliana nitrate transporter 1.6 mRNA, complete cds
AT5G05770	4.50497988	0.000720674	Arabidopsis thaliana chromosome 5 sequence
AT2G21400	4.50497988	0.000720674	Arabidopsis thaliana SHI-related sequence3 mRNA, complete cds
AT1G80590	4.50497988	0.000720674	Arabidopsis thaliana putative WRKY transcription factor 66 mRNA, complete cds
AT1G56480	4.50497988	0.000720674	Arabidopsis thaliana chromosome 1 sequence
AT3G56920	4.50497988	0.000720674	Arabidopsis thaliana putative S-acyltransferase mRNA, complete cds
AT1G29650.1	4.50497988	0.000720674	Arabidopsis thaliana chromosome 1 sequence
AT2G33280	4.50497988	0.000720674	Arabidopsis thaliana probable folate-biopterin transporter 9 mRNA, complete cds
AT2G20170	4.50497988	0.000720674	Arabidopsis thaliana uncharacterized protein mRNA, complete cds
AT3G25573	4.50497988	0.000720674	Arabidopsis thaliana chromosome 3, complete sequence
AT1G36610.1	4.50497988	0.000720674	Arabidopsis thaliana chromosome 1 sequence
AT1G09157	4.50497988	0.000720674	Arabidopsis thaliana chromosome 1 sequence
AT4G22680	1.367476356	0.000748032	Arabidopsis thaliana myb domain protein

			85 mRNA, complete cds
AT5G37760	2.548048602	0.000844969	Arabidopsis thaliana chaperone DnaJ-domain containing protein mRNA, complete cds
AT1G05550	1.984147717	0.000858056	Arabidopsis thaliana uncharacterized protein mRNA, complete cds
AT4G37430	1.984147717	0.000858056	Arabidopsis thaliana cytochrome P450 81F1 mRNA, complete cds
AT1G77390	1.165842495	0.000875464	Arabidopsis thaliana cyclin-A1-2 mRNA, complete cds
AT4G08115.1	1.630510762	0.000880547	Arabidopsis thaliana chromosome 4 sequence
AT3G60670	1.386585179	0.000897342	Arabidopsis thaliana PLATZ transcription factor family protein mRNA, complete cds
AT1G20080	2.115937589	0.00090018	Arabidopsis thaliana synaptotagmin-2 mRNA, complete cds
AT3G25882	2.293475775	0.000902714	Arabidopsis thaliana chromosome 3, complete sequence
AT2G22590	2.293475775	0.000902714	Arabidopsis thaliana chromosome 2, complete sequence
AT3G22415	1.20781969	0.000905579	Arabidopsis thaliana uncharacterized protein mRNA, complete cds
AT2G16210	3.630510762	0.000926056	Arabidopsis thaliana B3 domain-containing protein mRNA, complete cds
AT1G52990	3.630510762	0.000926056	Arabidopsis thaliana thioredoxin family protein mRNA, complete cds
AT5G23155	3.630510762	0.000926056	Arabidopsis thaliana chromosome 5 sequence
AT4G03540	3.630510762	0.000926056	Arabidopsis thaliana uncharacterized protein mRNA, complete cds
AT1G18310	3.630510762	0.000926056	Arabidopsis thaliana glycosyl hydrolase family 81 protein mRNA, complete cds
AT5G04000	3.630510762	0.000926056	Arabidopsis thaliana uncharacterized protein mRNA, complete cds
AT1G66610	3.630510762	0.000926056	Arabidopsis thaliana E3 ubiquitin-protein ligase SINA-like 1 mRNA, complete cds
AT2G20825	3.630510762	0.000926056	Arabidopsis thaliana protein ULTRAPETALA 2 mRNA, complete cds
AT4G16015	1.50497988	0.000999276	Arabidopsis thaliana chromosome 4 sequence
AT1G63245	1.682978182	0.001013516	Arabidopsis thaliana chromosome 1 sequence

AT4G20970	1.682978182	0.001013516	Arabidopsis thaliana basic helix-loop-helix domain-containing protein mRNA, complete cds
AT3G47875.1	1.74598798	0.001153702	Arabidopsis thaliana chromosome 3, complete sequence
AT4G06536	1.432571385	0.001283439	Arabidopsis thaliana chromosome 4 sequence
AT1G19550	1.82315584	0.001294124	Arabidopsis thaliana Glutathione S-transferase-like protein mRNA, complete cds
AT3G01570	1.82315584	0.001294124	Arabidopsis thaliana oleosin 5 mRNA, complete cds
AT5G06380	2.852903183	0.001296621	Arabidopsis thaliana chromosome 5 sequence
AT3G41762	2.852903183	0.001296621	Arabidopsis thaliana uncharacterized protein mRNA, complete cds
AT5G34825.1	2.852903183	0.001296621	Arabidopsis thaliana chromosome 5 sequence
AT5G53048	2.852903183	0.001296621	Arabidopsis thaliana AT5g53050/MNB8_11 mRNA, complete cds
AT4G19925	2.852903183	0.001296621	Arabidopsis thaliana Toll-Interleukin-Resistance domain-containing protein mRNA, complete cds
AT1G27740	2.852903183	0.001296621	Arabidopsis thaliana transcription factor RSL4 mRNA, complete cds
AT3G60970	2.852903183	0.001296621	Arabidopsis thaliana putative ABC transporter C-15 mRNA, complete cds
AT2G36026	2.852903183	0.001296621	Arabidopsis thaliana chromosome 2, complete sequence
AT1G19030.1	4.367476356	0.001368642	Arabidopsis thaliana chromosome 1 sequence
AT2G12440.1	4.367476356	0.001368642	Arabidopsis thaliana chromosome 2, complete sequence
AT1G61070	4.367476356	0.001368642	Arabidopsis thaliana defensin-like protein mRNA, complete cds
AT3G54410	4.367476356	0.001368642	Arabidopsis thaliana uncharacterized protein mRNA, complete cds
AT4G16160	4.367476356	0.001368642	Arabidopsis thaliana outer envelope pore protein 16-2 mRNA, complete cds
AT2G15990.1	4.367476356	0.001368642	Arabidopsis thaliana chromosome 2, complete sequence
AT1G23070	4.367476356	0.001368642	Arabidopsis thaliana uncharacterized

			protein mRNA, complete cds
AT5G45180	4.367476356	0.001368642	Arabidopsis thaliana Flavin-binding monooxygenase family protein mRNA, complete cds
AT4G34440	4.367476356	0.001368642	Arabidopsis thaliana proline-rich receptor-like protein kinase PERK5 mRNA, complete cds
AT3G57620	4.367476356	0.001368642	Arabidopsis thaliana chromosome 3, complete sequence
AT5G62040	1.920017379	0.001422885	Arabidopsis thaliana protein BROTHER of FT and TFL 1 mRNA, complete cds
AT3G11570	1.920017379	0.001422885	Arabidopsis thaliana protein trichome birefringence-like 8 mRNA, complete cds
AT5G54190	1.920017379	0.001422885	Arabidopsis thaliana protochlorophyllide reductase A mRNA, complete cds
AT3G11385	1.920017379	0.001422885	Arabidopsis thaliana chromosome 3, complete sequence
AT1G04700	1.10444195	0.001441432	Arabidopsis thaliana PB1 domain-containing protein tyrosine kinase mRNA, complete cds
AT1G63040	2.460585761	0.001500529	Arabidopsis thaliana chromosome 1 sequence
AT5G59590	2.460585761	0.001500529	Arabidopsis thaliana UDP-glucosyl transferase 76E2 mRNA, complete cds
AT1G79915	2.045548261	0.001521018	Arabidopsis thaliana putative methyltransferase family protein mRNA, complete cds
AT4G38000	2.215473263	0.001560036	Arabidopsis thaliana chromosome 4 sequence
AT2G41690	2.215473263	0.001560036	Arabidopsis thaliana heat stress transcription factor B-3 mRNA, complete cds
AT1G64195	2.215473263	0.001560036	Arabidopsis thaliana defensin-like protein 35 mRNA, complete cds
AT1G31750	1.630510762	0.00160244	Arabidopsis thaliana mRNA for hypothetical protein, complete cds, clone: RAFL16-18-G02
AT1G29860	1.233175265	0.001630021	Arabidopsis thaliana putative WRKY transcription factor 71 mRNA, complete cds
AT5G41380	1.019553053	0.001744474	Arabidopsis thaliana CCT motif family protein mRNA, complete cds
ATCG00180	1.019553053	0.001744474	Arabidopsis lyrata subsp. lyrata RNA

			polymerase beta subunit-1, mRNA
AT5G60470	3.50497988	0.001755499	Arabidopsis thaliana C2H2 and C2HC zinc finger-containing protein mRNA, complete cds
AT2G18720	3.50497988	0.001755499	Arabidopsis thaliana translation elongation factor EF1A/initiation factor IF2gamma family protein mRNA, complete cds
AT1G53420	3.50497988	0.001755499	Arabidopsis thaliana leucine-rich repeat transmembrane protein kinase mRNA, complete cds
AT1G47610	3.50497988	0.001755499	Arabidopsis thaliana chromosome 1 sequence
AT5G25045.1	3.50497988	0.001755499	Arabidopsis thaliana chromosome 5 sequence
AT5G53190	3.50497988	0.001755499	Arabidopsis thaliana bidirectional sugar transporter SWEET3 mRNA, complete cds
AT1G60450	3.50497988	0.001755499	Arabidopsis thaliana galactinol synthase 7 mRNA, complete cds
AT4G31760	3.50497988	0.001755499	Arabidopsis thaliana peroxidase 46 mRNA, complete cds
AT1G15310	1.106948806	0.001762545	Arabidopsis thaliana signal recognition particle protein SRP54A mRNA, complete cds
AT1G04600	1.493007238	0.001814093	Arabidopsis thaliana myosin XI A mRNA, complete cds
AT5G25910	1.145083935	0.001858531	Arabidopsis thaliana receptor like protein 52 mRNA, complete cds
AT4G08100.1	1.308582667	0.001988225	Arabidopsis thaliana chromosome 4 sequence
AT4G21970	1.761755295	0.002097808	Arabidopsis thaliana uncharacterized protein mRNA, complete cds
AT3G49380	1.530975089	0.002142766	Arabidopsis thaliana protein IQ-domain 15 mRNA, complete cds
AT4G21323	1.414782071	0.002322695	Arabidopsis thaliana Subtilase family protein mRNA, complete cds
AT2G31751	1.414782071	0.002322695	Arabidopsis thaliana clone asmbl_6700 unknown mRNA sequence
AT1G72070	1.197551355	0.002360749	Arabidopsis thaliana chaperone DnaJ-domain containing protein mRNA, complete cds
AT1G35610	2.74598798	0.002370956	Arabidopsis thaliana chromosome 1 sequence

AT1G02530	2.74598798	0.002370956	Arabidopsis thaliana P-glycoprotein 12 mRNA, complete cds
AT3G54510	1.255001627	0.002410718	Arabidopsis thaliana Early-responsive to dehydration stress protein (ERD4) mRNA, complete cds
AT5G59190	1.97154768	0.002552867	Arabidopsis thaliana subtilase family protein mRNA, complete cds
AT2G41445	4.215473263	0.002617218	Arabidopsis thaliana uncharacterized protein mRNA, complete cds
AT2G02340	4.215473263	0.002617218	Arabidopsis thaliana phloem protein 2-B8 mRNA, complete cds
AT5G09640	4.215473263	0.002617218	Arabidopsis thaliana serine carboxypeptidase-like 19 mRNA, complete cds
AT3G24900	4.215473263	0.002617218	Arabidopsis thaliana chromosome 3, complete sequence
AT5G02244	4.215473263	0.002617218	Arabidopsis thaliana mRNA for hypothetical protein, complete cds, clone: RAFL16-87-M17
AT2G07070.1	4.215473263	0.002617218	Arabidopsis thaliana chromosome 2, complete sequence
AT3G46520	4.215473263	0.002617218	Arabidopsis thaliana actin-12 mRNA, complete cds
AT3G15740	4.215473263	0.002617218	Arabidopsis thaliana chromosome 3, complete sequence
AT5G11050	4.215473263	0.002617218	Arabidopsis thaliana myb domain protein 64 mRNA, complete cds
AT4G27920	4.215473263	0.002617218	Arabidopsis thaliana abscisic acid receptor PYL10 mRNA, complete cds
AT1G61110	4.215473263	0.002617218	Arabidopsis thaliana NAC domain containing protein 25 mRNA, complete cds
AT2G28920	4.215473263	0.002617218	Arabidopsis thaliana chromosome 2, complete sequence
AT3G15440	4.215473263	0.002617218	Arabidopsis thaliana chromosome 3, complete sequence
AT4G17780	4.215473263	0.002617218	Arabidopsis thaliana putative F-box protein mRNA, complete cds
AT2G25540	4.215473263	0.002617218	Arabidopsis thaliana cellulose synthase 10 mRNA, complete cds
AT3G27440	4.215473263	0.002617218	Arabidopsis thaliana uridine kinase-like 5 mRNA, complete cds
AT5G66045	4.215473263	0.002617218	Arabidopsis thaliana chromosome 5 sequence

AT1G16290	4.215473263	0.002617218	Arabidopsis thaliana uncharacterized protein mRNA, complete cds
AT3G11773	4.215473263	0.002617218	Arabidopsis thaliana electron carrier/ protein disulfide oxidoreductase mRNA, complete cds
AT5G44590	4.215473263	0.002617218	Arabidopsis thaliana S-adenosyl-L-methionine-dependent methyltransferase domain-containing protein mRNA, complete cds
AT2G26610	4.215473263	0.002617218	Arabidopsis thaliana uncharacterized protein mRNA, complete cds
AT5G64530	1.112662457	0.002639241	Arabidopsis thaliana xylem NAC domain 1 mRNA, complete cds
AT2G38823	1.112662457	0.002639241	Arabidopsis thaliana uncharacterized protein mRNA, complete cds
AT3G30340	1.112662457	0.002639241	Arabidopsis thaliana nodulin MtN21 /EamA-like transporter family protein mRNA, complete cds
AT3G16360	2.367476356	0.002650863	Arabidopsis thaliana histidine-containing phosphotransfer protein 4 mRNA, complete cds
AT1G71450	2.367476356	0.002650863	Arabidopsis thaliana chromosome 1 sequence
AT1G80390	2.367476356	0.002650863	Arabidopsis thaliana auxin-responsive protein IAA15 mRNA, complete cds
AT5G60180	2.133011103	0.002679243	Arabidopsis thaliana chromosome 5 sequence
AT1G05490	1.444097638	0.002776544	Arabidopsis thaliana chromatin remodeling 31 mRNA, complete cds
AT1G67370	1.206012934	0.002880436	Arabidopsis thaliana asynaptic 1 mRNA, complete cds
AT5G20710	1.630510762	0.002929903	Arabidopsis thaliana beta-galactosidase 7 mRNA, complete cds
AT1G30220	1.630510762	0.002929903	Arabidopsis thaliana putative inositol transporter 2 mRNA, complete cds
AT2G41905	1.016401916	0.003204693	Arabidopsis thaliana chromosome 2, complete sequence
AT1G28950	3.367476356	0.003333007	Theobroma cacao Uncharacterized protein (TCM_007238) mRNA, complete cds
AT2G16005	3.367476356	0.003333007	Arabidopsis thaliana MD-2-related lipid recognition domain-containing protein mRNA, complete cds
AT1G73050	3.367476356	0.003333007	Arabidopsis thaliana

			Glucose-methanol-choline (GMC) oxidoreductase family protein mRNA, complete cds
AT2G22426	3.367476356	0.003333007	Arabidopsis thaliana chromosome 2, complete sequence
AT1G06970	3.367476356	0.003333007	Arabidopsis thaliana cation/H(+) antiporter 14 mRNA, complete cds
AT1G09176	3.367476356	0.003333007	Arabidopsis thaliana chromosome 1 sequence
AT3G56600	3.367476356	0.003333007	Arabidopsis thaliana phosphatidylinositol 4-kinase gamma 8 mRNA, complete cds
AT2G20800	3.367476356	0.003333007	Arabidopsis thaliana NAD(P)H dehydrogenase B4 mRNA, complete cds
AT4G08210	1.697624958	0.003375513	Arabidopsis thaliana chromosome 4 sequence
AT1G23935	1.161025479	0.003397258	Arabidopsis thaliana uncharacterized protein mRNA, complete cds
AT4G13395	1.215473263	0.003514985	Arabidopsis thaliana chromosome 4 sequence
AT5G67245	1.215473263	0.003514985	Arabidopsis thaliana chromosome 5 sequence
AT2G18180	1.215473263	0.003514985	Arabidopsis thaliana Sec14p-like phosphatidylinositol transfer family protein mRNA, complete cds
AT4G37410	1.782513856	0.003830608	Arabidopsis thaliana cytochrome P450, family 81, subfamily F, polypeptide 4 mRNA, complete cds
AT5G48410	1.782513856	0.003830608	Arabidopsis thaliana glutamate receptor 1.3 mRNA, complete cds
AT3G26790	1.782513856	0.003830608	Arabidopsis thaliana B3 domain-containing transcription factor FUS3 mRNA, complete cds
AT5G62850	1.119548843	0.003960728	Arabidopsis thaliana bidirectional sugar transporter SWEET5 mRNA, complete cds
AT1G03540	1.393471565	0.004218184	Arabidopsis thaliana chromosome 1 sequence
AT5G05430	1.893545168	0.004253335	Arabidopsis thaliana RNA-binding protein mRNA, complete cds
AT2G36255	1.045548261	0.00425537	Arabidopsis thaliana putative defensin-like protein 203 mRNA, complete cds
AT2G30360	2.630510762	0.004319351	Arabidopsis thaliana chromosome 2, complete sequence

AT2G23270	2.630510762	0.004319351	Arabidopsis thaliana chromosome 2, complete sequence
AT1G09510	2.630510762	0.004319351	Arabidopsis thaliana alcohol dehydrogenase-like protein mRNA, complete cds
AT5G64401	2.630510762	0.004319351	Arabidopsis thaliana chromosome 5 sequence
AT5G01730	1.299304854	0.004324298	Arabidopsis thaliana protein SCAR4 mRNA, complete cds
AT5G65550	2.045548261	0.00456954	Arabidopsis thaliana chromosome 5 sequence
AT3G47030	2.045548261	0.00456954	Arabidopsis thaliana chromosome 3, complete sequence
AT3G51680	1.081172171	0.00457011	Arabidopsis thaliana short-chain dehydrogenase reductase 2a mRNA, complete cds
AT1G68050	1.569110217	0.004616135	Arabidopsis thaliana flavin-binding, kelch repeat, f box 1 mRNA, complete cds
AT4G33800	1.569110217	0.004616135	Arabidopsis thaliana uncharacterized protein mRNA, complete cds
AT2G33760	2.267940683	0.004655256	Arabidopsis thaliana chromosome 2, complete sequence
ATMG00640	2.267940683	0.004655256	A.thaliana mitochondrial DNA for genes nad4L and orf25
AT1G04880	2.267940683	0.004655256	Arabidopsis thaliana high mobility group B protein 15 mRNA, complete cds
AT1G75360	1.123550773	0.004856487	Arabidopsis thaliana chromosome 1 sequence
AT1G44090	4.045548261	0.005042045	Arabidopsis thaliana gibberellin 20-oxidase 5 mRNA, complete cds
AT5G58080	4.045548261	0.005042045	Arabidopsis thaliana response regulator 18 mRNA, complete cds
AT1G18120	4.045548261	0.005042045	Arabidopsis thaliana chromosome 1 sequence
AT2G41997	4.045548261	0.005042045	Arabidopsis thaliana defensin-like protein 108 mRNA, complete cds
AT5G28235	4.045548261	0.005042045	Arabidopsis thaliana Ulp1 protease family protein mRNA, complete cds
AT2G40925	4.045548261	0.005042045	Arabidopsis thaliana chromosome 2, complete sequence
AT5G40260	4.045548261	0.005042045	Arabidopsis thaliana protein RUPTURED POLLEN GRAIN 1 mRNA, complete cds

AT4G02235	4.045548261	0.005042045	Arabidopsis thaliana protein agamous-like 51 mRNA, complete cds
AT2G41470	4.045548261	0.005042045	Arabidopsis thaliana uncharacterized protein mRNA, complete cds
AT5G12000	4.045548261	0.005042045	Arabidopsis thaliana Protein kinase protein with adenine nucleotide alpha hydrolases-like domain mRNA, complete cds
AT1G70720	4.045548261	0.005042045	Arabidopsis thaliana chromosome 1 sequence
AT4G14301	4.045548261	0.005042045	Arabidopsis thaliana uncharacterized protein mRNA, complete cds
AT4G10290	4.045548261	0.005042045	Arabidopsis thaliana RmlC-like cupins superfamily protein mRNA, complete cds
AT1G50960	4.045548261	0.005042045	Arabidopsis thaliana gibberellin 2-beta-dioxygenase 7 mRNA, complete cds
AT3G28470	4.045548261	0.005042045	Arabidopsis thaliana protein DEFECTIVE IN MERISTEM DEVELOPMENT AND FUNCTION 1 mRNA, complete cds
AT5G62320	4.045548261	0.005042045	Arabidopsis thaliana myb domain protein 99 mRNA, complete cds
AT4G07830.1	4.045548261	0.005042045	Arabidopsis thaliana chromosome 4 sequence
AT1G21528	4.045548261	0.005042045	Arabidopsis thaliana chromosome 1 sequence
AT5G20260	4.045548261	0.005042045	Arabidopsis thaliana Exostosin family protein mRNA, complete cds
AT1G02813	4.045548261	0.005042045	Arabidopsis thaliana chromosome 1 sequence
AT3G04660	4.045548261	0.005042045	Arabidopsis thaliana chromosome 3, complete sequence
AT5G40350	4.045548261	0.005042045	Arabidopsis thaliana myb domain protein 24 mRNA, complete cds
AT4G35655	4.045548261	0.005042045	Arabidopsis thaliana chromosome 4 sequence
AT1G69470	4.045548261	0.005042045	Arabidopsis thaliana chromosome 1 sequence
AT3G57270	4.045548261	0.005042045	Arabidopsis thaliana beta-1,3-glucanase 1 mRNA, complete cds
AT1G28160	4.045548261	0.005042045	Arabidopsis thaliana chromosome 1 sequence

AT2G41280	4.045548261	0.005042045	Arabidopsis thaliana late embryogenesis abundant protein M10 mRNA, complete cds
AT5G50790	1.174831278	0.005089642	Arabidopsis thaliana bidirectional sugar transporter SWEET10 mRNA, complete cds
AT3G22250	1.318566756	0.005246739	Arabidopsis thaliana UDP-glycosyltransferase 82A1 mRNA, complete cds
AT4G02320	1.630510762	0.005388275	Arabidopsis thaliana probable pectinesterase/pectinesterase inhibitor 40 mRNA, complete cds
AT2G22810	1.012381397	0.00592251	Arabidopsis thaliana 1-aminocyclopropane-1-carboxylate synthase mRNA, complete cds
AT1G28970	1.460585761	0.006058566	Theobroma cacao Uncharacterized protein (TCM_007238) mRNA, complete cds
AT1G51770	1.460585761	0.006058566	Arabidopsis thaliana core-2/I-branching beta-1,6-N-acetylglucosaminyltransferase family protein mRNA, complete cds
AT5G21030	1.708513274	0.00621123	Arabidopsis thaliana protein argonaute 8 mRNA, complete cds
AT1G58889.1	1.708513274	0.00621123	Arabidopsis thaliana DNA, retrotransposon:AtRE1, complete sequence, ecotype: Niederzenz
AT1G48870	1.183051785	0.006233784	Arabidopsis thaliana WD40 domain-containing protein mRNA, complete cds
AT4G10265	3.215473263	0.006335794	Arabidopsis thaliana chromosome 4 sequence
AT4G06535.1	3.215473263	0.006335794	Arabidopsis thaliana chromosome 4 sequence
AT3G52780	3.215473263	0.006335794	Arabidopsis thaliana putative purple acid phosphatase 20 mRNA, complete cds
AT1G74130	3.215473263	0.006335794	Arabidopsis thaliana Rhomboid-related intramembrane serine protease family protein mRNA, complete cds
AT1G02470	1.251999139	0.006388525	Arabidopsis thaliana SRPBCC ligand-binding domain-containing protein mRNA, complete cds
AT4G30370	1.085076626	0.00689476	Arabidopsis thaliana chromosome 4 sequence
AT3G57970	1.811083008	0.007029433	Arabidopsis thaliana Emsy N Terminus

			and plant Tudor-like domain-containing protein mRNA, complete cds
AT2G13900	1.811083008	0.007029433	Arabidopsis thaliana chromosome 2, complete sequence
AT1G14490	1.367476356	0.007692461	Arabidopsis thaliana chromosome 1 sequence
AT3G52160	1.952438857	0.007733215	Arabidopsis thaliana 3-ketoacyl-CoA synthase 15 mRNA, complete cds
AT5G15720	1.952438857	0.007733215	Arabidopsis thaliana GDSL esterase/lipase 7 mRNA, complete cds
AT3G05415.1	1.267940683	0.007794204	Arabidopsis thaliana chromosome 3, complete sequence
AT5G55150	2.50497988	0.007833144	Arabidopsis thaliana uncharacterized protein mRNA, complete cds
AT2G46455	2.50497988	0.007833144	Arabidopsis thaliana OxaA/YidC-like membrane insertion protein mRNA, complete cds
AT2G13810	2.50497988	0.007833144	Arabidopsis thaliana AGD2-like defense response protein 1 mRNA, complete cds
AT2G35658	2.50497988	0.007833144	Arabidopsis thaliana chromosome 2, complete sequence
AT1G15360	2.50497988	0.007833144	Arabidopsis thaliana ethylene-responsive transcription factor WIN1 mRNA, complete cds
AT2G47150	2.50497988	0.007833144	Arabidopsis thaliana Rossmann-fold NAD(P)-binding domain-containing protein mRNA, complete cds
AT1G19160	2.50497988	0.007833144	Arabidopsis thaliana chromosome 1 sequence
AT1G58390	2.161025479	0.008119515	Arabidopsis thaliana CC-NBS-LRR class disease resistance protein mRNA, complete cds
AT5G05420	2.161025479	0.008119515	Arabidopsis thaliana peptidyl-prolyl cis-trans isomerase FKBP15-3 mRNA, complete cds
AT5G38310	1.087368437	0.008479615	Arabidopsis thaliana chromosome 5 sequence
AT1G72490	1.560121434	0.008527487	Arabidopsis thaliana uncharacterized protein mRNA, complete cds
AT4G18980	1.560121434	0.008527487	Arabidopsis thaliana chromosome 4 sequence
AT4G01533	1.399185216	0.009284674	Arabidopsis thaliana Full-length cDNA Complete sequence from clone GSLTPGH70ZB05 of Hormone Treated

			Callus of strain col-0 of Arabidopsis thaliana (thale cress)
AT1G11610	3.852903183	0.009791452	Arabidopsis thaliana cytochrome P450 71A18 mRNA, complete cds
AT3G24093	3.852903183	0.009791452	Arabidopsis thaliana chromosome 3, complete sequence
AT4G02950	3.852903183	0.009791452	Arabidopsis thaliana chromosome 4 sequence
AT1G30780	3.852903183	0.009791452	Arabidopsis thaliana FAD-binding Berberine family protein mRNA, complete cds
AT2G28680	3.852903183	0.009791452	Arabidopsis thaliana RmlC-like cupins superfamily protein mRNA, complete cds
AT3G21880	3.852903183	0.009791452	Arabidopsis thaliana B-box type zinc finger protein with CCT domain mRNA, complete cds
AT1G01280	3.852903183	0.009791452	Arabidopsis thaliana cytochrome P450, family 703, subfamily A, polypeptide 2 mRNA, complete cds
AT3G20880	3.852903183	0.009791452	Arabidopsis thaliana WIP domain protein 4 mRNA, complete cds
AT1G24570	3.852903183	0.009791452	Arabidopsis thaliana uncharacterized protein mRNA, complete cds
AT4G29980	3.852903183	0.009791452	Arabidopsis thaliana chromosome 4 sequence
AT1G06135	3.852903183	0.009791452	Arabidopsis thaliana chromosome 1 sequence
AT5G10000	3.852903183	0.009791452	Arabidopsis thaliana chromosome 5 sequence
AT1G50830	3.852903183	0.009791452	Arabidopsis thaliana chromosome 1 sequence
AT1G73190	3.852903183	0.009791452	Arabidopsis thaliana aquaporin TIP3-1 mRNA, complete cds
AT2G03460	3.852903183	0.009791452	Arabidopsis thaliana galactose oxidase/kelch-like protein mRNA, complete cds
AT4G05049	3.852903183	0.009791452	Arabidopsis thaliana chromosome 4 sequence
AT3G51590	3.852903183	0.009791452	Arabidopsis thaliana non-specific lipid-transfer protein 12 mRNA, complete cds
AT1G30455	3.852903183	0.009791452	Arabidopsis thaliana cyclin/Brf1-like TBP-binding domain-containing protein

			mRNA, complete cds
AT5G24550	3.852903183	0.009791452	Arabidopsis thaliana beta glucosidase 32 mRNA, complete cds
AT1G62580	3.852903183	0.009791452	Arabidopsis thaliana flavin-containing monooxygenase FMO GS-OX-like 7 mRNA, complete cds
AT4G11580	3.852903183	0.009791452	Arabidopsis thaliana RNI-like superfamily protein mRNA, complete cds
AT1G49550.1	3.852903183	0.009791452	Arabidopsis thaliana chromosome 1 sequence
AT1G23201	3.852903183	0.009791452	Arabidopsis thaliana chromosome 1 sequence
AT1G60720	3.852903183	0.009791452	Arabidopsis thaliana chromosome 1 sequence
AT5G05070	3.852903183	0.009791452	Arabidopsis thaliana DHHC-type zinc finger family protein mRNA, complete cds
AT1G68765	3.852903183	0.009791452	Arabidopsis thaliana chromosome 1 sequence
AT2G41415	3.852903183	0.009791452	Arabidopsis thaliana maternally expressed family protein mRNA, complete cds
AT3G08750	3.852903183	0.009791452	Arabidopsis thaliana chromosome 3, complete sequence
AT4G09595.1	3.852903183	0.009791452	Arabidopsis thaliana chromosome 4 sequence
AT3G54530	3.852903183	0.009791452	Arabidopsis thaliana uncharacterized protein mRNA, complete cds
AT3G16040	3.852903183	0.009791452	Arabidopsis thaliana translation machinery associated TMA7 mRNA, complete cds
AT5G24090	1.089942381	0.010438855	Arabidopsis thaliana chitinase A mRNA, complete cds
AT5G47600	1.007074114	0.011028918	Arabidopsis thaliana heat shock protein 14.7 mRNA, complete cds
AT1G78478	1.437865684	0.011168969	Arabidopsis thaliana chromosome 1 sequence
AT2G47810	1.437865684	0.011168969	Arabidopsis thaliana chromosome 2, complete sequence
AT4G38140	1.723620167	0.011514356	Arabidopsis thaliana chromosome 4 sequence
AT2G29950	1.308582667	0.011583169	Arabidopsis thaliana chromosome 2, complete sequence

AT2G02700	1.308582667	0.011583169	Arabidopsis thaliana chromosome 2, complete sequence
AT1G28940	1.308582667	0.011583169	Theobroma cacao Uncharacterized protein (TCM_007238) mRNA, complete cds
AT5G60310	1.045548261	0.011976545	Arabidopsis thaliana putative L-type lectin-domain containing receptor kinase I.10 mRNA, complete cds
AT1G03982	3.045548261	0.012053009	Arabidopsis thaliana PAK-box/P21-Rho-binding family protein mRNA, complete cds
AT1G28830	3.045548261	0.012053009	Theobroma cacao Uncharacterized protein (TCM_007238) mRNA, complete cds
AT5G15140	3.045548261	0.012053009	Arabidopsis thaliana aldose 1-epimerase-like protein mRNA, complete cds
AT4G01023	3.045548261	0.012053009	Arabidopsis thaliana RING/U-box domain-containing protein mRNA, complete cds
AT2G04038	3.045548261	0.012053009	Arabidopsis thaliana chromosome 2, complete sequence
AT1G35200	3.045548261	0.012053009	Arabidopsis thaliana chromosome 1 sequence
AT2G06045.1	3.045548261	0.012053009	Arabidopsis thaliana chromosome 2, complete sequence
AT4G04510	3.045548261	0.012053009	Arabidopsis thaliana cysteine-rich receptor-like protein kinase 38 mRNA, complete cds
AT3G13130	3.045548261	0.012053009	Arabidopsis thaliana chromosome 3, complete sequence
AT3G05400	1.092853976	0.012864421	Arabidopsis thaliana sugar transporter ERD6-like 12 mRNA, complete cds
AT4G04500	1.852903183	0.012973693	Arabidopsis thaliana cysteine-rich receptor-like protein kinase 37 mRNA, complete cds
AT5G64450	1.852903183	0.012973693	Arabidopsis thaliana uncharacterized protein mRNA, complete cds
AT2G25890	1.852903183	0.012973693	Arabidopsis thaliana chromosome 2, complete sequence
AT2G30900	1.486120853	0.013370553	Arabidopsis thaliana protein TRICHOME BIREFRINGENCE-LIKE 43 mRNA, complete cds
AT3G21755	1.486120853	0.013370553	Arabidopsis thaliana clone 151422

			mRNA sequence
AT5G57520	1.152463465	0.013609661	Arabidopsis thaliana chromosome 5 sequence
AT1G80660	2.045548261	0.01405051	Arabidopsis thaliana H(+)-ATPase 9 mRNA, complete cds
AT4G29570	2.045548261	0.01405051	Arabidopsis thaliana chromosome 4 sequence
AT1G29715	2.045548261	0.01405051	Arabidopsis thaliana mRNA for hypothetical protein, complete cds, clone: RAFL21-67-K19
AT2G25440	2.045548261	0.01405051	Arabidopsis thaliana receptor like protein 20 mRNA, complete cds
AT1G03800	2.045548261	0.01405051	Arabidopsis thaliana chromosome 1 sequence
AT2G30380	2.045548261	0.01405051	Arabidopsis thaliana uncharacterized protein mRNA, complete cds
AT5G63300	1.229972833	0.014085146	Arabidopsis thaliana ribosomal protein S21 family protein mRNA, complete cds
AT2G05950.1	2.367476356	0.014126328	Arabidopsis thaliana chromosome 2, complete sequence
AT1G59265.1	2.367476356	0.014126328	Arabidopsis thaliana chromosome 1 sequence
AT2G35200	2.367476356	0.014126328	Arabidopsis thaliana chromosome 2, complete sequence
AT1G08080	2.367476356	0.014126328	Arabidopsis thaliana alpha carbonic anhydrase 7 mRNA, complete cds
AT3G08700	2.367476356	0.014126328	Arabidopsis thaliana putative ubiquitin-conjugating enzyme E2 12 mRNA, complete cds
AT5G06400	2.367476356	0.014126328	Arabidopsis thaliana chromosome 5 sequence
AT2G27280	2.367476356	0.014126328	Arabidopsis thaliana Coiled-coil domain-containing protein 55 mRNA, complete cds
AT1G18990	1.045548261	0.014776637	Arabidopsis thaliana uncharacterized protein mRNA, complete cds
AT5G12380	1.096174334	0.015872135	Arabidopsis thaliana annexin D8 mRNA, complete cds
AT3G57740	1.096174334	0.015872135	Arabidopsis thaliana chromosome 3, complete sequence
AT1G69710	1.548048602	0.015890673	Arabidopsis thaliana regulator of chromosome condensation and FYVE zinc finger domain-containing protein mRNA, complete cds

AT1G35290	1.548048602	0.015890673	Arabidopsis thaliana thioesterase family protein mRNA, complete cds
AT1G13310	1.548048602	0.015890673	Arabidopsis thaliana Endosomal targeting BRO1-like domain-containing protein mRNA, complete cds
AT4G22940	1.548048602	0.015890673	Arabidopsis thaliana protein kinase family protein mRNA, complete cds
AT1G10540	1.247182123	0.017281088	Arabidopsis thaliana nucleobase-ascorbate transporter 8 mRNA, complete cds
AT1G22275	1.247182123	0.017281088	Arabidopsis thaliana synaptonemal complex protein 2 mRNA, complete cds
AT3G13640	1.247182123	0.017281088	Arabidopsis thaliana ABC transporter E family member 1 mRNA, complete cds
AT5G09970	1.630510762	0.018675681	Arabidopsis thaliana cytochrome P450, family 78, subfamily A, polypeptide 7 mRNA, complete cds
AT5G04630	3.630510762	0.019181077	Arabidopsis thaliana chromosome 5 sequence
AT2G19890	3.630510762	0.019181077	Arabidopsis thaliana uncharacterized protein mRNA, complete cds
AT5G27010	3.630510762	0.019181077	Arabidopsis thaliana uncharacterized protein mRNA, complete cds
AT3G05310	3.630510762	0.019181077	Arabidopsis thaliana MIRO-related GTP-ase 3 mRNA, complete cds
AT2G27270	3.630510762	0.019181077	Arabidopsis thaliana uncharacterized protein mRNA, complete cds
AT4G27580	3.630510762	0.019181077	Arabidopsis thaliana uncharacterized protein mRNA, complete cds
AT2G29100	3.630510762	0.019181077	Arabidopsis thaliana glutamate receptor 2.9 mRNA, complete cds
AT1G29680	3.630510762	0.019181077	Arabidopsis thaliana uncharacterized protein mRNA, complete cds
AT4G27790	3.630510762	0.019181077	Arabidopsis thaliana Calcium-binding EF hand family protein mRNA, complete cds
AT3G57370	3.630510762	0.019181077	Arabidopsis thaliana cyclin family protein mRNA, complete cds
AT2G05450.1	3.630510762	0.019181077	Arabidopsis thaliana chromosome 2, complete sequence
AT5G59170	3.630510762	0.019181077	Arabidopsis thaliana chromosome 5 sequence
AT5G52740	3.630510762	0.019181077	Arabidopsis thaliana copper transport family protein mRNA, complete cds

AT3G17320	3.630510762	0.019181077	Arabidopsis thaliana chromosome 3, complete sequence
AT5G46610	3.630510762	0.019181077	Arabidopsis thaliana aluminum activated malate transporter family protein mRNA, complete cds
AT2G24560	3.630510762	0.019181077	Arabidopsis thaliana GDSL esterase/lipase mRNA, complete cds
AT1G03390	3.630510762	0.019181077	Arabidopsis thaliana chromosome 1 sequence
AT2G05960.1	3.630510762	0.019181077	Arabidopsis thaliana chromosome 2, complete sequence
AT4G31740	3.630510762	0.019181077	Arabidopsis thaliana sec1/munc18-like (SM) family protein mRNA, complete cds
AT4G28405	3.630510762	0.019181077	Arabidopsis thaliana uncharacterized protein mRNA, complete cds
AT3G42960	3.630510762	0.019181077	Arabidopsis thaliana protein TAPETUM 1 mRNA, complete cds
AT3G19700	3.630510762	0.019181077	Arabidopsis thaliana leucine rich repeat kinase HAIKU 2 mRNA, complete cds
AT1G32890.1	3.630510762	0.019181077	Arabidopsis thaliana chromosome 1 sequence
AT2G35670	3.630510762	0.019181077	Arabidopsis thaliana polycomb group protein FERTILIZATION-INDEPENDENT SEED 2 mRNA, complete cds
AT1G16705	3.630510762	0.019181077	Arabidopsis thaliana p300/CBP acetyltransferase-related protein mRNA, complete cds
AT5G47150	3.630510762	0.019181077	Arabidopsis thaliana YDG/SRA domain-containing protein mRNA, complete cds
AT5G17200	3.630510762	0.019181077	Arabidopsis thaliana pectin lyase-like superfamily protein mRNA, complete cds
AT1G54870	3.630510762	0.019181077	Arabidopsis thaliana glucose and ribitol dehydrogenase homolog 1 mRNA, complete cds
AT5G67060	3.630510762	0.019181077	Arabidopsis thaliana chromosome 5 sequence
AT4G36510	3.630510762	0.019181077	Arabidopsis thaliana uncharacterized protein mRNA, complete cds
AT4G04420.1	3.630510762	0.019181077	Arabidopsis thaliana chromosome 4 sequence

AT1G33160	3.630510762	0.019181077	Arabidopsis thaliana chromosome 1 sequence
AT5G04010	3.630510762	0.019181077	Arabidopsis thaliana chromosome 5 sequence
AT5G01595	3.630510762	0.019181077	Arabidopsis thaliana Full-length cDNA Complete sequence from clone GSLTSIL39ZC05 of Silique of strain col-0 of Arabidopsis thaliana (thale cress)
AT4G38310	3.630510762	0.019181077	Arabidopsis thaliana chromosome 4 sequence
AT1G52900	3.630510762	0.019181077	Arabidopsis thaliana Toll-Interleukin-Resistance domain-containing protein mRNA, complete cds
AT2G27570	3.630510762	0.019181077	Arabidopsis thaliana P-loop containing nucleoside triphosphate hydrolases superfamily protein mRNA, complete cds
AT1G54560	3.630510762	0.019181077	Arabidopsis thaliana Myosin family protein with Dil domain mRNA, complete cds
AT3G13890	3.630510762	0.019181077	Arabidopsis thaliana myb domain protein 26 mRNA, complete cds
AT4G11340	3.630510762	0.019181077	Arabidopsis thaliana TIR-NBS-LRR class disease resistance protein mRNA, complete cds
AT4G25510	3.630510762	0.019181077	Arabidopsis thaliana uncharacterized protein mRNA, complete cds
AT5G58050	3.630510762	0.019181077	Arabidopsis thaliana glycerophosphodiester phosphodiesterase like 6 mRNA, complete cds
AT5G57080	1.099996045	0.019608432	Arabidopsis thaliana uncharacterized protein mRNA, complete cds
AT4G37022	1.099996045	0.019608432	Arabidopsis thaliana uncharacterized protein mRNA, complete cds
AT1G66600	1.171079143	0.020678939	Arabidopsis thaliana putative WRKY transcription factor 63 mRNA, complete cds
AT3G12410	1.171079143	0.020678939	Arabidopsis thaliana chromosome 3, complete sequence
AT1G02030	1.408118341	0.020753741	Arabidopsis thaliana chromosome 1 sequence
AT2G04490.1	1.408118341	0.020753741	Arabidopsis thaliana chromosome 2, complete sequence

AT3G26539	1.408118341	0.020753741	Arabidopsis thaliana chromosome 3, complete sequence
AT2G02061	1.408118341	0.020753741	Arabidopsis thaliana nucleotide-diphospho-sugar transferase domain-containing protein mRNA, complete cds
AT4G31970	1.267940683	0.021201914	Arabidopsis thaliana cytochrome P450, family 82, subfamily C, polypeptide 2 mRNA, complete cds
AT3G48700	1.267940683	0.021201914	Arabidopsis thaliana chromosome 3, complete sequence
AT1G62620	1.74598798	0.021552851	Arabidopsis thaliana flavin-containing monooxygenase FMO GS-OX-like 3 mRNA, complete cds
AT1G05894	1.74598798	0.021552851	Arabidopsis thaliana chromosome 1 sequence
AT3G56770	1.045548261	0.022586348	Arabidopsis thaliana putative transcription factor bHLH107 mRNA, complete cds
AT1G74870	1.045548261	0.022586348	Arabidopsis thaliana RING-finger domain-containing protein mRNA, complete cds
AT2G34030	2.852903183	0.022931802	Arabidopsis thaliana Calcium-binding EF-hand family protein mRNA, complete cds
AT1G20180	2.852903183	0.022931802	Arabidopsis thaliana uncharacterized protein mRNA, complete cds
AT5G57310	2.852903183	0.022931802	Arabidopsis thaliana uncharacterized protein mRNA, complete cds
AT1G63870	2.852903183	0.022931802	Arabidopsis thaliana TIR-NBS-LRR class disease resistance protein mRNA, complete cds
AT2G16140.1	2.852903183	0.022931802	Arabidopsis thaliana chromosome 2, complete sequence
AT5G38750	2.852903183	0.022931802	Arabidopsis thaliana asparaginyl-tRNA synthetase-like protein mRNA, complete cds
AT1G78840	2.852903183	0.022931802	Arabidopsis thaliana putative F-box/FBD/LRR-repeat protein mRNA, complete cds
AT4G17980	2.852903183	0.022931802	Arabidopsis thaliana NAC domain containing protein 71 mRNA, complete cds
AT1G12890	2.852903183	0.022931802	Arabidopsis thaliana chromosome 1

			sequence
AT3G50390	2.852903183	0.022931802	Arabidopsis thaliana chromosome 3, complete sequence
AT1G68610	2.852903183	0.022931802	Arabidopsis thaliana chromosome 1 sequence
AT1G51530	2.852903183	0.022931802	Arabidopsis thaliana RNA recognition motif-containing protein mRNA, complete cds
AT1G19415.1	1.920017379	0.024092711	Arabidopsis thaliana chromosome 1 sequence
AT3G09620	1.920017379	0.024092711	Arabidopsis thaliana DEAD-box ATP-dependent RNA helicase 45 mRNA, complete cds
AT5G48050	1.460585761	0.025029192	Arabidopsis thaliana chromosome 5 sequence
AT2G04420	2.215473263	0.025301076	Arabidopsis thaliana chromosome 2, complete sequence
AT1G22000	2.215473263	0.025301076	Arabidopsis thaliana putative F-box/FBD/LRR-repeat protein mRNA, complete cds
AT3G28917	2.215473263	0.025301076	Arabidopsis thaliana chromosome 3, complete sequence
AT3G03760	2.215473263	0.025301076	Arabidopsis thaliana LOB domain-containing protein 20 mRNA, complete cds
AT5G02220	2.215473263	0.025301076	Arabidopsis thaliana chromosome 5 sequence
AT4G10940	1.183051785	0.025529136	Arabidopsis thaliana RING/U-box protein mRNA, complete cds
AT5G27230	1.293475775	0.026003288	Arabidopsis thaliana Frigida-like protein mRNA, complete cds
AT5G49525	1.530975089	0.029961242	Arabidopsis thaliana chromosome 5 sequence
AT1G10110	1.530975089	0.029961242	Arabidopsis thaliana chromosome 1 sequence
AT1G48740	1.109678599	0.03006163	Arabidopsis thaliana 2-oxoglutarate (2OG) and Fe(II)-dependent oxygenase superfamily protein mRNA, complete cds
AT4G31070	1.109678599	0.03006163	Arabidopsis thaliana chromosome 4 sequence
AT5G50260	1.109678599	0.03006163	Arabidopsis thaliana KDEL-tailed cysteine endopeptidase CEP1 mRNA, complete cds

AT1G51400	1.197551355	0.031550701	Arabidopsis thaliana chromosome 1 sequence
AT5G41680	1.325656181	0.031862887	Arabidopsis thaliana chromosome 5 sequence
AT1G35730	1.045548261	0.034754876	Arabidopsis thaliana protein pumilio 9 mRNA, complete cds
AT2G25130	1.045548261	0.034754876	Arabidopsis thaliana armadillo/beta-catenin-like repeats-containing protein mRNA, complete cds
AT1G77765	1.630510762	0.035409787	Arabidopsis thaliana uncharacterized protein mRNA, complete cds
AT1G69880	1.630510762	0.035409787	Arabidopsis thaliana thioredoxin H8 mRNA, complete cds
AT4G14180	1.630510762	0.035409787	Arabidopsis thaliana protein PRD1 mRNA, complete cds
AT2G38510	1.115937589	0.03731982	Arabidopsis thaliana chromosome 2, complete sequence
AT5G57820	3.367476356	0.03793829	Arabidopsis thaliana zinc ion binding protein mRNA, complete cds
AT2G02498	3.367476356	0.03793829	Arabidopsis thaliana chromosome 2, complete sequence
AT1G80090	3.367476356	0.03793829	Arabidopsis thaliana SNF1-related protein kinase regulatory subunit gamma-like PV42b mRNA, complete cds
AT1G10880	3.367476356	0.03793829	Arabidopsis thaliana core-2/I-branching beta-1,6-N-acetylglucosaminyltransferase family protein mRNA, complete cds
AT2G35990	3.367476356	0.03793829	Arabidopsis thaliana cytokinin riboside 5'-monophosphate phosphoribohydrolase LOG2 mRNA, complete cds
AT2G05025.1	3.367476356	0.03793829	Arabidopsis thaliana chromosome 2, complete sequence
AT2G04515	3.367476356	0.03793829	Arabidopsis thaliana chromosome 2, complete sequence
AT4G01160	3.367476356	0.03793829	Arabidopsis thaliana BTB/POZ/Kelch-associated protein mRNA, complete cds
AT1G05510	3.367476356	0.03793829	Arabidopsis thaliana uncharacterized protein mRNA, complete cds
AT4G22600	3.367476356	0.03793829	Arabidopsis thaliana chromosome 4 sequence
AT3G28705.1	3.367476356	0.03793829	Arabidopsis thaliana chromosome 3, complete sequence

AT5G38680	3.367476356	0.03793829	Arabidopsis thaliana putative F-box/kelch-repeat protein mRNA, complete cds
AT4G20900	3.367476356	0.03793829	Arabidopsis thaliana protein male sterile 5 mRNA, complete cds
AT2G18115	3.367476356	0.03793829	Arabidopsis thaliana chromosome 2, complete sequence
AT5G37840	3.367476356	0.03793829	Arabidopsis thaliana uncharacterized protein mRNA, complete cds
AT4G25670	3.367476356	0.03793829	Arabidopsis thaliana conserved peptide upstream open reading frame 12 mRNA, complete cds
AT1G12630	3.367476356	0.03793829	Arabidopsis thaliana chromosome 1 sequence
AT2G43261	3.367476356	0.03793829	Arabidopsis thaliana uncharacterized protein mRNA, complete cds
AT1G13070	3.367476356	0.03793829	Arabidopsis thaliana chromosome 1 sequence
AT5G57747	3.367476356	0.03793829	Arabidopsis thaliana chromosome 5 sequence
AT2G15340	3.367476356	0.03793829	Arabidopsis thaliana chromosome 2, complete sequence
AT4G16730	3.367476356	0.03793829	Arabidopsis thaliana terpene synthase 02 mRNA, complete cds
AT5G45113	3.367476356	0.03793829	Arabidopsis thaliana chromosome 5 sequence
AT1G07540	3.367476356	0.03793829	Arabidopsis thaliana putative telomere repeat-binding protein TRFL2 mRNA, complete cds
AT3G15602.1	3.367476356	0.03793829	Arabidopsis thaliana chromosome 3, complete sequence
AT4G27330	3.367476356	0.03793829	Arabidopsis thaliana putative transcription factor SPL mRNA, complete cds
AT2G31590	3.367476356	0.03793829	Arabidopsis thaliana uncharacterized protein mRNA, complete cds
AT4G33290	3.367476356	0.03793829	Arabidopsis thaliana chromosome 4 sequence
AT5G60830	3.367476356	0.03793829	Arabidopsis thaliana basic leucine-zipper 70 mRNA, complete cds
AT5G11820	3.367476356	0.03793829	Arabidopsis thaliana chromosome 5 sequence
AT5G38705.1	3.367476356	0.03793829	Arabidopsis thaliana chromosome 5 sequence

AT3G03930	3.367476356	0.03793829	Arabidopsis thaliana protein kinase-related protein mRNA, complete cds
AT3G46911	3.367476356	0.03793829	Arabidopsis thaliana chromosome 3, complete sequence
AT1G32375	3.367476356	0.03793829	Arabidopsis thaliana putative FBD-associated F-box protein mRNA, complete cds
AT5G37420	3.367476356	0.03793829	Arabidopsis thaliana uncharacterized protein mRNA, complete cds
AT1G31258	3.367476356	0.03793829	Arabidopsis thaliana uncharacterized protein mRNA, complete cds
AT1G59950	3.367476356	0.03793829	Arabidopsis thaliana putative aldo/keto reductase mRNA, complete cds
AT2G32370	3.367476356	0.03793829	Arabidopsis thaliana homeobox-leucine zipper protein HDG3 mRNA, complete cds
AT5G20690	3.367476356	0.03793829	Arabidopsis thaliana probable inactive leucine-rich repeat receptor-like protein kinase mRNA, complete cds
AT3G51190	3.367476356	0.03793829	Arabidopsis thaliana 60S ribosomal protein L8-2 mRNA, complete cds
AT2G11300.1	3.367476356	0.03793829	Arabidopsis thaliana chromosome 2, complete sequence
AT1G22015	3.367476356	0.03793829	Arabidopsis thaliana putative beta-1,3-galactosyltransferase 5 mRNA, complete cds
AT4G33860	3.367476356	0.03793829	Arabidopsis thaliana glycosyl hydrolase family 10 protein mRNA, complete cds
AT4G01985	3.367476356	0.03793829	Arabidopsis thaliana chromosome 4 sequence
AT1G71160	3.367476356	0.03793829	Arabidopsis thaliana chromosome 1 sequence
AT3G45790	3.367476356	0.03793829	Arabidopsis thaliana protein kinase-related protein mRNA, complete cds
AT1G05330	3.367476356	0.03793829	Arabidopsis thaliana chromosome 1 sequence
AT3G60650	3.367476356	0.03793829	Arabidopsis thaliana chromosome 3, complete sequence
AT4G37840	3.367476356	0.03793829	Arabidopsis thaliana hexokinase-like 3 mRNA, complete cds
AT4G35370	3.367476356	0.03793829	Arabidopsis thaliana transducin/WD40 domain-containing protein mRNA,

			complete cds
AT1G62420	3.367476356	0.03793829	Arabidopsis thaliana uncharacterized protein mRNA, complete cds
AT1G57640.1	3.367476356	0.03793829	Arabidopsis thaliana chromosome 1 sequence
AT2G35075	1.367476356	0.038968883	Arabidopsis thaliana uncharacterized protein mRNA, complete cds
AT2G34240	1.367476356	0.038968883	Arabidopsis thaliana uncharacterized protein mRNA, complete cds
AT1G14642	1.215473263	0.039033925	Arabidopsis thaliana uncharacterized protein mRNA, complete cds
AT3G50030	1.215473263	0.039033925	Arabidopsis thaliana uncharacterized protein mRNA, complete cds
AT3G14950	1.782513856	0.040874585	Arabidopsis thaliana TPR repeat-containing thioredoxin TTL2 mRNA, complete cds
AT5G65130	1.782513856	0.040874585	Arabidopsis thaliana chromosome 5 sequence
AT4G03380	1.782513856	0.040874585	Arabidopsis thaliana uncharacterized protein mRNA, complete cds
AT1G01355	1.782513856	0.040874585	Arabidopsis thaliana putative endonuclease or glycosyl hydrolase mRNA, complete cds
AT5G25920	1.782513856	0.040874585	Arabidopsis thaliana uncharacterized protein mRNA, complete cds
AT3G56700	1.782513856	0.040874585	Arabidopsis thaliana fatty acyl-CoA reductase 6 mRNA, complete cds
AT3G62990	1.782513856	0.040874585	Arabidopsis thaliana chromosome 3, complete sequence
AT1G67770	1.782513856	0.040874585	Arabidopsis thaliana terminal EAR1-like 2 protein mRNA, complete cds
AT5G08391	1.782513856	0.040874585	Arabidopsis thaliana chromosome 5 sequence
AT5G52290	2.630510762	0.043594026	Arabidopsis thaliana protein SHORTAGE IN CHIASMATA 1 mRNA, complete cds
AT4G32445	2.630510762	0.043594026	Arabidopsis thaliana chromosome 4 sequence
AT3G59230	2.630510762	0.043594026	Arabidopsis thaliana putative F-box/LRR-repeat protein mRNA, complete cds
AT5G43570	2.630510762	0.043594026	Arabidopsis thaliana PR-6 proteinase inhibitor family protein mRNA, complete cds

AT2G10400.1	2.630510762	0.043594026	Arabidopsis thaliana chromosome 2, complete sequence
AT5G58170	2.630510762	0.043594026	Arabidopsis thaliana glycerophosphodiester phosphodiesterase like 7 mRNA, complete cds
AT5G61730	2.630510762	0.043594026	Arabidopsis thaliana ABC transporter A family member 9 mRNA, complete cds
AT3G13850	2.630510762	0.043594026	Arabidopsis thaliana chromosome 3, complete sequence
AT1G07473	2.630510762	0.043594026	Arabidopsis thaliana chromosome 1 sequence
AT5G62780	2.630510762	0.043594026	Arabidopsis thaliana chaperone DnaJ-domain containing protein mRNA, complete cds
AT5G44570	2.630510762	0.043594026	Arabidopsis thaliana uncharacterized protein mRNA, complete cds
AT4G28485	2.630510762	0.043594026	Arabidopsis thaliana DUF679 domain membrane protein 7 mRNA, complete cds
AT2G21420	2.630510762	0.043594026	Arabidopsis thaliana IBR domain containing protein mRNA, complete cds
AT3G13660	2.630510762	0.043594026	Arabidopsis thaliana chromosome 3, complete sequence
AT5G41890	2.630510762	0.043594026	Arabidopsis thaliana GDSL esterase/lipase mRNA, complete cds
AT4G29550	2.630510762	0.043594026	Arabidopsis thaliana uncharacterized protein mRNA, complete cds
AT2G46685	2.630510762	0.043594026	Arabidopsis thaliana chromosome 2, complete sequence
AT1G62000	2.630510762	0.043594026	Arabidopsis thaliana chromosome 1 sequence
AT5G45570	2.630510762	0.043594026	Arabidopsis thaliana Ulp1 protease family protein mRNA, complete cds
AT4G18450	2.045548261	0.044931997	Arabidopsis thaliana chromosome 4 sequence
AT1G09260	2.045548261	0.044931997	Arabidopsis thaliana chromosome 1 sequence
AT4G24420	2.045548261	0.044931997	Arabidopsis thaliana chromosome 4 sequence
AT5G24820	2.045548261	0.044931997	Arabidopsis thaliana aspartyl protease family protein mRNA, complete cds
AT3G24230	2.045548261	0.044931997	Arabidopsis thaliana putative pectate lyase 9 mRNA, complete cds
AT4G31355	2.045548261	0.044931997	Arabidopsis thaliana chromosome 4

			sequence
AT1G62240	2.045548261	0.044931997	Arabidopsis thaliana chromosome 1 sequence
AT1G04500	2.045548261	0.044931997	Arabidopsis thaliana CCT motif-containing protein mRNA, complete cds
AT5G65165	2.045548261	0.044931997	Arabidopsis thaliana succinate dehydrogenase [ubiquinone] iron-sulfur subunit 3 mRNA, complete cds
AT2G10735	2.045548261	0.044931997	Arabidopsis thaliana chromosome 2, complete sequence
AT2G17490.1	2.045548261	0.044931997	Arabidopsis thaliana chromosome 2, complete sequence
AT1G23700	2.045548261	0.044931997	Arabidopsis thaliana protein kinase mRNA, complete cds
AT5G66710	2.045548261	0.044931997	Arabidopsis thaliana protein kinase family protein mRNA, complete cds
AT3G20395	1.424059885	0.047484054	Arabidopsis thaliana RING-finger domain-containing protein mRNA, complete cds
AT1G19394	1.424059885	0.047484054	Arabidopsis thaliana uncharacterized protein mRNA, complete cds
AT5G13230	1.238193339	0.048338603	Arabidopsis thaliana chromosome 5 sequence
AT5G51950	1.238193339	0.048338603	Arabidopsis thaliana Glucose-methanol-choline (GMC) oxidoreductase family protein mRNA, complete cds
AT3G12720	1.045548261	0.053947789	Arabidopsis thaliana myb domain protein 67 mRNA, complete cds
AT2G34390	1.045548261	0.053947789	Arabidopsis thaliana aquaporin NIP2-1 mRNA, complete cds
AT3G58770	1.045548261	0.053947789	Arabidopsis thaliana uncharacterized protein mRNA, complete cds
AT4G10020	1.50497988	0.057445822	Arabidopsis thaliana hydroxysteroid dehydrogenase 5 mRNA, complete cds
AT1G19560	1.50497988	0.057445822	Arabidopsis thaliana chromosome 1 sequence
AT4G40020	1.50497988	0.057445822	Arabidopsis thaliana chromosome 4 sequence
AT1G72720	1.50497988	0.057445822	Arabidopsis thaliana chromosome 1 sequence
AT1G74400	1.133011103	0.057891806	Arabidopsis thaliana chromosome 1 sequence

AT1G29420	1.267940683	0.059902647	Arabidopsis thaliana chromosome 1 sequence
AT5G28415.1	1.267940683	0.059902647	Arabidopsis thaliana chromosome 5 sequence
AT3G18550	1.267940683	0.059902647	Arabidopsis thaliana transcription factor TCP18 mRNA, complete cds
AT5G17810	1.267940683	0.059902647	Arabidopsis thaliana WUSCHEL-related homeobox 12 mRNA, complete cds
AT5G59390	1.267940683	0.059902647	Arabidopsis thaliana XH/XS domain-containing protein mRNA, complete cds
AT3G20898	1.045548261	0.067496279	Arabidopsis thaliana uncharacterized protein mRNA, complete cds
ATCG00770	1.045548261	0.067496279	Arabidopsis thaliana chloroplast DNA, complete genome, ecotype: Columbia
AT3G24929	1.045548261	0.067496279	Arabidopsis thaliana uncharacterized protein mRNA, complete cds
AT5G19151	1.630510762	0.068484378	Arabidopsis thaliana uncharacterized protein mRNA, complete cds
AT3G10185	1.630510762	0.068484378	Arabidopsis thaliana Gibberellin-regulated family protein mRNA, complete cds
AT4G28460	1.630510762	0.068484378	Arabidopsis thaliana chromosome 4 sequence
AT4G31351	1.630510762	0.068484378	Arabidopsis thaliana chromosome 4 sequence
AT5G58830	1.630510762	0.068484378	Arabidopsis thaliana Subtilisin-like serine endopeptidase family protein mRNA, complete cds
AT2G03020	1.630510762	0.068484378	Arabidopsis thaliana Heat shock protein HSP20/alpha crystallin family protein mRNA, complete cds
AT3G03240	1.630510762	0.068484378	Arabidopsis thaliana alpha/beta-Hydrolases superfamily protein mRNA, complete cds
AT4G29340	1.630510762	0.068484378	Arabidopsis thaliana profilin 4 mRNA, complete cds
AT2G01175	1.145083935	0.072385397	Arabidopsis thaliana chromosome 2, complete sequence
AT1G48060	1.145083935	0.072385397	Arabidopsis thaliana F-box protein mRNA, complete cds
AT5G42490	1.308582667	0.074235728	Arabidopsis thaliana ATP binding microtubule motor family protein mRNA, complete cds

AT5G10945	1.308582667	0.074235728	Arabidopsis thaliana chromosome 5 sequence
AT3G48920	1.852903183	0.078951915	Arabidopsis thaliana myb domain protein 45 mRNA, complete cds
AT2G23945	1.852903183	0.078951915	Arabidopsis thaliana chromosome 2, complete sequence
AT1G33640	1.852903183	0.078951915	Arabidopsis thaliana uncharacterized protein mRNA, complete cds
AT5G04238	1.852903183	0.078951915	Arabidopsis thaliana chromosome 5 sequence
AT4G32342	1.852903183	0.078951915	Arabidopsis thaliana uncharacterized protein mRNA, complete cds
AT5G25390	1.852903183	0.078951915	Arabidopsis thaliana ethylene-responsive transcription factor SHINE 3 mRNA, complete cds
AT4G01780	1.852903183	0.078951915	Arabidopsis thaliana XH/XS domain-containing protein mRNA, complete cds
AT1G63140	1.852903183	0.078951915	Arabidopsis thaliana O-methyltransferase 4 mRNA, complete cds
AT1G22290	1.852903183	0.078951915	Arabidopsis thaliana 14-3-3 family protein mRNA, complete cds
AT5G35932	2.367476356	0.082693952	Arabidopsis thaliana chromosome 5 sequence
ATCG00160	2.367476356	0.082693952	Arabidopsis thaliana chloroplast DNA, complete genome, ecotype: Columbia
AT5G48710	2.367476356	0.082693952	Arabidopsis thaliana putative small ubiquitin-related modifier 4 mRNA, complete cds
AT3G45000	2.367476356	0.082693952	Arabidopsis thaliana vacuolar protein sorting protein 24.2 mRNA, complete cds
AT2G21490	2.367476356	0.082693952	Arabidopsis thaliana dehydrin LEA mRNA, complete cds
AT5G46417	2.367476356	0.082693952	Arabidopsis thaliana chromosome 5 sequence
AT5G17030	2.367476356	0.082693952	Arabidopsis thaliana UDP-glucosyl transferase 78D3 mRNA, complete cds
AT5G13940	2.367476356	0.082693952	Arabidopsis thaliana aminopeptidase mRNA, complete cds
AT2G22905	2.367476356	0.082693952	Arabidopsis thaliana chromosome 2, complete sequence
AT1G66570	2.367476356	0.082693952	Arabidopsis thaliana putative sucrose transport protein SUC7 mRNA, complete

			cds
AT5G52000	2.367476356	0.082693952	Arabidopsis thaliana importin alpha isoform 8 mRNA, complete cds
AT1G79400	2.367476356	0.082693952	Arabidopsis thaliana cation/H(+) antiporter 2 mRNA, complete cds
AT3G44830	2.367476356	0.082693952	Arabidopsis thaliana putative phospholipid:diacylglycerol acyltransferase 2 mRNA, complete cds
AT3G03230	2.367476356	0.082693952	Arabidopsis thaliana alpha/beta-Hydrolases superfamily protein mRNA, complete cds
AT5G04500	2.367476356	0.082693952	Arabidopsis thaliana glycosyltransferase family protein 47 mRNA, complete cds
AT1G70380	2.367476356	0.082693952	Arabidopsis thaliana chromosome 1 sequence
AT1G50770	2.367476356	0.082693952	Arabidopsis thaliana Aminotransferase-like, plant mobile domain family protein mRNA, complete cds
AT1G53260	2.367476356	0.082693952	Arabidopsis thaliana uncharacterized protein mRNA, complete cds
AT2G31420	2.367476356	0.082693952	Arabidopsis thaliana chromosome 2, complete sequence
AT5G08210	2.367476356	0.082693952	Arabidopsis thaliana chromosome 5 sequence
AT1G03710	2.367476356	0.082693952	Arabidopsis thaliana cystatin/monellin-related protein mRNA, complete cds
AT4G20920	2.367476356	0.082693952	Arabidopsis thaliana double-stranded RNA-binding domain (DsRBD)-containing protein mRNA, complete cds
AT1G33030	1.045548261	0.08474583	Arabidopsis thaliana O-methyltransferase-like protein mRNA, complete cds
AT1G74110	1.161025479	0.090792605	Arabidopsis thaliana cytochrome P450, family 78, subfamily A, polypeptide 10 mRNA, complete cds
AT4G39363	1.161025479	0.090792605	Arabidopsis thaliana chromosome 4 sequence
AT2G20510	1.161025479	0.090792605	Arabidopsis thaliana translocase inner membrane subunit 44-1 mRNA, complete cds
AT3G05790	1.161025479	0.090792605	Arabidopsis thaliana lon protease 4

			mRNA, complete cds
AT2G16895	1.161025479	0.090792605	Arabidopsis thaliana chromosome 2, complete sequence
AT4G18960	1.161025479	0.090792605	Arabidopsis thaliana MADS domain transcription factor AGAMOUS mRNA, complete cds
AT3G60966	1.367476356	0.091859471	Arabidopsis thaliana chromosome 3, complete sequence
AT5G26582.1	1.367476356	0.091859471	Arabidopsis thaliana chromosome 5 sequence
AT5G40990	1.367476356	0.091859471	Arabidopsis thaliana GDSL lipase 1 mRNA, complete cds
AT5G63390	1.367476356	0.091859471	Arabidopsis thaliana O-fucosyltransferase family protein mRNA, complete cds
AT5G41663	1.367476356	0.091859471	Arabidopsis thaliana chromosome 5 sequence
AT3G11260	1.045548261	0.10686243	Arabidopsis thaliana WUSCHEL-related homeobox 5 mRNA, complete cds
AT1G76610	1.045548261	0.10686243	Arabidopsis thaliana chromosome 1 sequence
AT2G45420	1.045548261	0.10686243	Arabidopsis thaliana LOB domain-containing protein 18 mRNA, complete cds
AT5G12180	1.460585761	0.113059061	Arabidopsis thaliana calcium-dependent protein kinase 17 mRNA, complete cds
AT2G20150	1.460585761	0.113059061	Arabidopsis thaliana uncharacterized protein mRNA, complete cds
AT1G76770	1.460585761	0.113059061	Arabidopsis thaliana alpha-crystallin domain of heat shock protein-containing protein mRNA, complete cds
AT2G45410	1.460585761	0.113059061	Arabidopsis thaliana LOB domain-containing protein 19 mRNA, complete cds
AT4G27190	1.460585761	0.113059061	Arabidopsis thaliana chromosome 4 sequence
AT4G05018	1.460585761	0.113059061	Arabidopsis thaliana chromosome 4 sequence
AT1G22260	1.460585761	0.113059061	Arabidopsis thaliana synaptonemal complex protein ZYP1a mRNA, complete cds
AT3G50310	1.460585761	0.113059061	Arabidopsis thaliana chromosome 3, complete sequence
AT5G51480	1.460585761	0.113059061	Arabidopsis thaliana Monocopper

			oxidase-like protein SKS2 mRNA, complete cds
AT3G13898	1.460585761	0.113059061	Arabidopsis thaliana uncharacterized protein mRNA, complete cds
AT3G15536	1.460585761	0.113059061	Arabidopsis thaliana chromosome 3, complete sequence
AT4G17085	1.183051785	0.114294427	Arabidopsis thaliana putative membrane lipoprotein mRNA, complete cds
AT1G17255	1.183051785	0.114294427	Arabidopsis thaliana chromosome 1 sequence
AT2G33880	1.183051785	0.114294427	Arabidopsis thaliana WUSCHEL-related homeobox 9 mRNA, complete cds
AT2G46567	1.183051785	0.114294427	Arabidopsis thaliana chromosome 2, complete sequence
AT5G35770	1.183051785	0.114294427	Arabidopsis thaliana transcriptional regulator STERILE APETALA mRNA, complete cds
AT1G48400	1.183051785	0.114294427	Arabidopsis thaliana F-box/RNI-like/FBD-like domain-containing protein mRNA, complete cds
AT5G37620	1.183051785	0.114294427	Arabidopsis thaliana chromosome 5 sequence
AT2G30690	1.183051785	0.114294427	Arabidopsis thaliana uncharacterized protein mRNA, complete cds
AT4G17660	1.183051785	0.114294427	Arabidopsis thaliana protein kinase family protein mRNA, complete cds
AT5G39473	1.045548261	0.135477646	Arabidopsis thaliana chromosome 5 sequence
AT5G18180	1.045548261	0.135477646	Arabidopsis thaliana H/ACA ribonucleoprotein complex, subunit Gar1/Naf1 protein mRNA, complete cds
AT3G29639	1.045548261	0.135477646	Arabidopsis thaliana uncharacterized protein mRNA, complete cds
AT1G74550	1.045548261	0.135477646	Arabidopsis thaliana chromosome 1 sequence
AT4G04540	1.630510762	0.136883187	Arabidopsis thaliana putative cysteine-rich receptor-like protein kinase 39 mRNA, complete cds
ATCG00370	1.630510762	0.136883187	Cardamine resedifolia plastid, complete genome
AT2G35970	1.630510762	0.136883187	Arabidopsis thaliana chromosome 2, complete sequence
AT1G24577	1.630510762	0.136883187	Arabidopsis thaliana chromosome 1

			sequence
AT1G67623	1.630510762	0.136883187	Arabidopsis thaliana putative F-box protein mRNA, complete cds
AT2G07706	1.630510762	0.136883187	Arabidopsis thaliana uncharacterized protein mRNA, complete cds
AT1G65810	1.630510762	0.136883187	Arabidopsis thaliana P-loop containing nucleoside triphosphate hydrolases superfamily protein mRNA, complete cds
AT1G06030	1.630510762	0.136883187	Arabidopsis thaliana probable fructokinase-2 mRNA, complete cds
AT5G34830	1.630510762	0.136883187	Arabidopsis thaliana uncharacterized protein mRNA, complete cds
AT2G17690	1.630510762	0.136883187	Arabidopsis thaliana SUPPRESSOR OF drm1 drm2 cmt3 mRNA, complete cds
AT1G67990	1.630510762	0.136883187	Arabidopsis thaliana tapetum-specific methyltransferase 1 mRNA, complete cds
AT3G52270	1.630510762	0.136883187	Arabidopsis thaliana transcription initiation factor IIF, beta subunit mRNA, complete cds
AT1G48470	1.630510762	0.136883187	Arabidopsis thaliana glutamine synthetase cytosolic isozyme 1-5 mRNA, complete cds
AT5G19720	1.630510762	0.136883187	Arabidopsis thaliana ribosomal protein L25/Gln-tRNA synthetase, anti-codon-binding domain protein mRNA, complete cds
AT5G18510	1.630510762	0.136883187	Arabidopsis thaliana chromosome 5 sequence
AT2G46670	1.630510762	0.136883187	Arabidopsis thaliana CCT motif family protein mRNA, complete cds
AT1G24590	1.630510762	0.136883187	Arabidopsis thaliana chromosome 1 sequence
AT1G48660	1.215473263	0.14446956	Arabidopsis thaliana auxin-responsive GH3 family protein mRNA, complete cds
AT3G09870	1.215473263	0.14446956	Arabidopsis thaliana chromosome 3, complete sequence
AT1G09650	1.215473263	0.14446956	Arabidopsis thaliana chromosome 1 sequence
AT2G27505	1.215473263	0.14446956	Arabidopsis thaliana FBD-like domain family protein mRNA, complete cds
AT3G25880	1.215473263	0.14446956	Arabidopsis thaliana NAD(P)-binding Rossmann-fold superfamily protein

			mRNA, complete cds
AT4G16050	1.215473263	0.14446956	Arabidopsis thaliana chromosome 4 sequence
AT2G01810	1.215473263	0.14446956	Arabidopsis thaliana RING/FYVE/PHD zinc finger superfamily protein mRNA, complete cds
AT5G17960	1.215473263	0.14446956	Arabidopsis thaliana chromosome 5 sequence
AT4G26260	1.215473263	0.14446956	Arabidopsis thaliana myo-inositol oxygenase 4 mRNA, complete cds

Supplementary Data S3B Down regulated genes in Col-0(+NaCl)/Col-0(-NaCl)

Gene	Log2FoldChange	pval	NT:Description
AT5G38430	-2.013898674	0	Arabidopsis thaliana ribulose bisphosphate carboxylase small chain 1B mRNA, complete cds
AT4G12550	-4.130141763	0	Arabidopsis thaliana chromosome 4 sequence
AT3G16240	-1.794095333	0	Arabidopsis thaliana aquaporin TIP2-1 mRNA, complete cds
AT3G01190	-4.385265629	0	Arabidopsis thaliana peroxidase 27 mRNA, complete cds
AT5G20630	-2.829184459	0	Arabidopsis thaliana chromosome 5 sequence
AT1G69530	-2.115977935	0	Arabidopsis thaliana expansin A1 mRNA, complete cds
AT2G45180	-2.783647003	0	Arabidopsis thaliana chromosome 2, complete sequence
AT2G06850	-2.56422857	0	Arabidopsis thaliana endoxyloglucan transferase A1 mRNA, complete cds
AT1G73330	-1.888099504	0	Arabidopsis thaliana chromosome 1 sequence
AT3G18780	-1.309728699	0	Arabidopsis thaliana actin 2 mRNA, complete cds
AT2G10940	-1.791148539	0	Arabidopsis thaliana chromosome 2, complete sequence
AT1G65930	-1.322025237	0	Arabidopsis thaliana NADP+-dependent isocitrate dehydrogenase mRNA, complete cds
AT5G44020	-2.737001724	0	Arabidopsis thaliana HAD superfamily, subfamily IIIB acid phosphatase mRNA, complete cds
AT3G53460	-1.739242358	0	Arabidopsis thaliana chloroplast RNA-binding protein 29 mRNA, complete cds
AT4G15390	-3.444644103	0	Arabidopsis thaliana chromosome 4 sequence
AT1G66200	-1.36162712	0	Arabidopsis thaliana glutamine synthetase mRNA, complete cds
AT1G49860	-3.962973293	0	Arabidopsis thaliana glutathione S-transferase (class phi) 14 mRNA, complete cds
AT4G20260	-2.076402502	0	Arabidopsis thaliana plasma-membrane associated cation-binding protein 1

			mRNA, complete cds
AT3G32980	-2.364281271	0	Arabidopsis thaliana Peroxidase family protein mRNA, complete cds
AT1G68520	-2.287257887	0	Arabidopsis thaliana zinc finger protein CONSTANS-LIKE 6 mRNA, complete cds
AT5G43350	-3.512204995	0	Arabidopsis thaliana inorganic phosphate transporter 1-1 mRNA, complete cds
AT5G26280	-2.327566632	0	Arabidopsis thaliana TRAF-like family protein mRNA, complete cds
AT4G16980	-1.5101941	0	Arabidopsis thaliana chromosome 4 sequence
AT1G21130	-2.833789204	0	Arabidopsis thaliana Indole glucosinolate O-methyltransferase 4 mRNA, complete cds
AT1G15380	-2.623392632	0	Arabidopsis thaliana Lactoylglutathione lyase / glyoxalase I family protein mRNA, complete cds
AT1G03870	-4.15885653	0	Arabidopsis thaliana chromosome 1 sequence
AT2G41090	-2.395397409	0	Arabidopsis thaliana calmodulin-like protein 10 mRNA, complete cds
AT1G72150	-1.700646835	0	Arabidopsis thaliana patellin-1 mRNA, complete cds
AT4G23400	-1.855356799	0	Arabidopsis thaliana putative aquaporin PIP1-5 mRNA, complete cds
AT3G14310	-1.675891755	0	Arabidopsis thaliana pectin methylesterase 3 mRNA, complete cds
AT1G29660	-2.62299678	0	Arabidopsis thaliana GDSL esterase/lipase mRNA, complete cds
AT4G12545	-4.063985224	0	Arabidopsis thaliana chromosome 4 sequence
AT4G12520	-6.517330132	0	Arabidopsis thaliana chromosome 4 sequence
AT3G63160	-2.527070249	0	Arabidopsis thaliana chromosome 3, complete sequence
AT5G44340	-1.556268096	0	Arabidopsis thaliana tubulin beta-4 chain mRNA, complete cds
AT5G17820	-3.788738585	0	Arabidopsis thaliana peroxidase 57 mRNA, complete cds
AT4G30170	-3.498381492	0	Arabidopsis thaliana peroxidase 45 mRNA, complete cds
AT2G29980	-1.532311726	0	Arabidopsis thaliana omega-3 fatty acid desaturase mRNA, complete cds

AT1G04820	-1.296310741	0	Arabidopsis thaliana tubulin alpha-4 chain mRNA, complete cds
AT1G75500	-1.676506724	0	Arabidopsis thaliana protein walls are thin 1 mRNA, complete cds
AT1G52190	-3.090228518	0	Arabidopsis thaliana nitrate transporter 1.11 mRNA, complete cds
AT5G38940	-3.470075035	0	Arabidopsis thaliana germin-like protein subfamily 1 member 11 mRNA, complete cds
AT5G40850	-1.695409197	0	Arabidopsis thaliana urophorphyrin methylase 1 mRNA, complete cds
AT3G14210	-1.265286865	0	Arabidopsis thaliana epithiospecifier modifier 1 mRNA, complete cds
AT3G24420	-2.058174309	0	Arabidopsis thaliana hydrolase, alpha/beta fold family protein mRNA, complete cds
AT1G08090	-4.535652321	0	Arabidopsis thaliana nitrate transporter 2:1 mRNA, complete cds
AT3G52720	-2.394897349	0	Arabidopsis thaliana alpha carbonic anhydrase 1 mRNA, complete cds
AT2G28630	-2.948857722	0	Arabidopsis thaliana chromosome 2, complete sequence
AT4G30190	-1.286096932	0	Arabidopsis thaliana H(+)-ATPase 2 mRNA, complete cds
AT1G16880	-1.288661455	0	Arabidopsis thaliana ACT domain-containing protein mRNA, complete cds
AT2G37220	-1.036657243	0	Arabidopsis thaliana chloroplast RNA binding protein mRNA, complete cds
AT1G77760	-1.293207879	0	Arabidopsis thaliana nitrate reductase [NADH] 1 mRNA, complete cds
AT2G38310	-3.584824699	0	Arabidopsis thaliana chromosome 2, complete sequence
AT1G13110	-2.874221126	0	Arabidopsis thaliana cytochrome P450 71B7 mRNA, complete cds
AT3G58610	-1.15295731	0	Arabidopsis thaliana ketol-acid reductoisomerase mRNA, complete cds
AT5G48430	-3.505325349	0	Arabidopsis thaliana chromosome 5 sequence
AT5G14120	-1.709301371	0	Arabidopsis thaliana major facilitator protein mRNA, complete cds
AT5G47450	-2.485076329	0	Arabidopsis thaliana aquaporin TIP2-3 mRNA, complete cds
AT4G19690	-4.368428043	0	Arabidopsis thaliana Fe(2+) transport

			protein 1 mRNA, complete cds
AT3G48360	-1.723980453	0	Arabidopsis thaliana TAC1-mediated telomerase activation pathway protein BT2 mRNA, complete cds
AT4G27440	-1.63298538	0	Arabidopsis thaliana light-dependent NADPH:protochlorophyllide oxidoreductase B mRNA, complete cds
AT5G65010	-1.841150092	0	Arabidopsis thaliana asparagine synthetase 2 mRNA, complete cds
AT1G25440	-1.550896807	0	Arabidopsis thaliana zinc finger protein CONSTANS-LIKE 16 mRNA, complete cds
AT1G49240	-1.108350443	0	Arabidopsis thaliana actin 8 mRNA, complete cds
AT3G01290	-2.211480059	0	Arabidopsis thaliana SPFH/Band 7/PHB domain-containing membrane-associated protein mRNA, complete cds
AT4G14400	-1.934731809	0	Arabidopsis thaliana protein ACCELERATED CELL DEATH 6 mRNA, complete cds
AT4G16370	-1.519247491	0	Arabidopsis thaliana oligopeptide transporter mRNA, complete cds
AT2G45470	-1.854653889	0	Arabidopsis thaliana chromosome 2, complete sequence
AT1G12080	-2.396358443	0	Arabidopsis thaliana vacuolar calcium-binding protein-like protein mRNA, complete cds
AT1G12110	-1.485151713	0	Arabidopsis thaliana nitrate transporter 1.1 mRNA, complete cds
AT5G64100	-2.067998929	0	Arabidopsis thaliana peroxidase 69 mRNA, complete cds
AT5G23020	-2.221023609	0	Arabidopsis thaliana methylthioalkylmalate synthase 3 mRNA, complete cds
AT1G20010	-2.476883056	0	Arabidopsis thaliana tubulin beta-5 chain mRNA, complete cds
AT5G20250	-1.541006794	0	Arabidopsis thaliana putative galactinol--sucrose galactosyltransferase 6 mRNA, complete cds
AT5G60660	-3.187732476	0	Arabidopsis thaliana putative aquaporin PIP2-4 mRNA, complete cds
AT5G19780	-1.770859203	0	Arabidopsis thaliana tubulin alpha-5 mRNA, complete cds
AT5G19770	-1.789082716	0	Arabidopsis thaliana tubulin alpha-3

			mRNA, complete cds
AT1G70850	-2.287695147	0	Arabidopsis thaliana MLP-like protein 34 mRNA, complete cds
AT5G61420	-2.12811222	1.53E-303	Arabidopsis thaliana transcription factor MYB28 mRNA, complete cds
AT1G26250	-2.91975902	3.37E-303	Arabidopsis thaliana chromosome 1 sequence
AT1G22530	-2.693061162	1.65E-302	Arabidopsis thaliana patellin 2 mRNA, complete cds
AT1G61740	-1.180618733	1.14E-299	Arabidopsis thaliana Sulfite exporter TauE/SafE family protein mRNA, complete cds
AT1G62480	-1.027021843	2.04E-296	Arabidopsis thaliana vacuolar calcium-binding protein-like protein mRNA, complete cds
AT5G46890	-3.953449519	7.58E-296	Arabidopsis thaliana chromosome 5 sequence
AT5G14200	-1.728229998	2.68E-292	Arabidopsis thaliana isopropylmalate dehydrogenase 1 mRNA, complete cds
AT1G75750	-2.043495038	8.24E-292	Arabidopsis thaliana GA-responsive GAST1 protein-like protein mRNA, complete cds
AT3G01120	-1.13608031	1.33E-291	Arabidopsis thaliana cystathionine gamma-synthase mRNA, complete cds
AT5G08280	-1.289610603	1.16E-289	Arabidopsis thaliana Porphobilinogen deaminase mRNA, complete cds
AT1G04040	-2.761806661	2.42E-280	Arabidopsis thaliana HAD superfamily, subfamily IIIB acid phosphatase mRNA, complete cds
AT1G70410	-1.108700272	7.40E-279	Arabidopsis thaliana beta carbonic anhydrase 4 mRNA, complete cds
AT2G30930	-3.252132287	2.64E-272	Arabidopsis thaliana uncharacterized protein mRNA, complete cds
AT5G67400	-4.615041944	1.32E-270	Arabidopsis thaliana peroxidase 73 mRNA, complete cds
AT4G13770	-1.645613643	1.67E-270	Arabidopsis thaliana cytochrome P450 83A1 mRNA, complete cds
AT4G25050	-1.159559235	1.82E-269	Arabidopsis thaliana acyl carrier protein 4 mRNA, complete cds
AT4G12730	-2.040485178	2.95E-268	Arabidopsis thaliana chromosome 4 sequence
AT4G14630	-3.592577773	4.21E-266	Arabidopsis thaliana germin-like protein 9 mRNA, complete cds
AT3G04790	-1.314566622	5.48E-262	Arabidopsis thaliana chromosome 3,

			complete sequence
AT2G44790	-1.327793893	2.62E-260	Arabidopsis thaliana uclacyanin 2 mRNA, complete cds
AT5G46900	-4.698111985	1.17E-258	Arabidopsis thaliana chromosome 5 sequence
AT3G62040	-2.409045216	1.33E-252	Arabidopsis thaliana haloacid dehalogenase-like hydrolase superfamily protein mRNA, complete cds
AT2G18300	-2.878642135	1.72E-252	Arabidopsis thaliana transcription factor bHLH64 mRNA, complete cds
AT5G09220	-1.131440919	1.76E-252	Arabidopsis thaliana amino acid permease 2 mRNA, complete cds
AT5G51550	-2.013753199	7.82E-247	Arabidopsis thaliana chromosome 5 sequence
AT4G18205	-2.787117749	8.06E-247	Arabidopsis thaliana nucleotide-sugar transporter family protein mRNA, complete cds
AT3G52380	-1.318790149	2.10E-246	Arabidopsis thaliana chloroplast RNA-binding protein 33 mRNA, complete cds
AT1G21310	-1.768405771	1.09E-240	Arabidopsis thaliana extensin 3 mRNA, complete cds
AT5G11420	-2.002010358	4.78E-237	Arabidopsis thaliana uncharacterized protein mRNA, complete cds
AT2G23600	-1.69498143	5.55E-235	Arabidopsis thaliana methylesterase 2 mRNA, complete cds
AT1G66270	-2.126466089	1.01E-232	Arabidopsis thaliana beta-glucosidase 21 mRNA, complete cds
AT3G16420	-1.0681379	2.10E-230	Arabidopsis thaliana PYK10-binding protein 1 mRNA, complete cds
AT2G25510	-1.201602649	2.19E-229	Arabidopsis thaliana uncharacterized protein mRNA, complete cds
AT1G71880	-1.322148506	1.01E-228	Arabidopsis thaliana sucrose transport protein SUC1 mRNA, complete cds
AT1G78860	-3.33757999	1.34E-227	Arabidopsis thaliana chromosome 1 sequence
AT1G12520	-1.361643671	1.78E-227	Arabidopsis thaliana copper chaperone for SOD1 mRNA, complete cds
AT4G26010	-4.077071025	9.66E-227	Arabidopsis thaliana peroxidase 44 mRNA, complete cds
AT3G23430	-1.494299233	4.94E-226	Arabidopsis thaliana phosphate transporter PHO1 mRNA, complete cds
AT1G68590	-1.479926468	9.89E-225	Arabidopsis thaliana 30S ribosomal protein 3-1 mRNA, complete cds

AT2G28950	-1.89555805	9.47E-223	Arabidopsis thaliana expansin A6 mRNA, complete cds
AT3G05950	-2.173854117	1.32E-222	Arabidopsis thaliana mRNA for germin-like protein, complete cds, clone: RAFL16-44-D15
AT1G78020	-1.278896058	5.86E-219	Arabidopsis thaliana uncharacterized protein mRNA, complete cds
AT4G12420	-1.312425372	1.87E-218	Arabidopsis thaliana multi-copper oxidase-like protein SKU5 mRNA, complete cds
AT3G04210	-3.000219984	6.74E-217	Arabidopsis thaliana TIR-NBS class disease resistance protein mRNA, complete cds
AT1G16400	-3.072192008	1.38E-215	Arabidopsis thaliana Hexahomomethionine N-hydroxylase mRNA, complete cds
AT5G50200	-2.251525901	3.66E-215	Arabidopsis thaliana high-affinity nitrate transporter 3.1 mRNA, complete cds
AT1G54000	-1.947668957	8.33E-213	Arabidopsis thaliana GDSL esterase/lipase mRNA, complete cds
AT2G37660	-1.147631313	1.17E-212	Arabidopsis thaliana NAD(P)-binding Rossmann-fold-containing protein mRNA, complete cds
AT2G38120	-1.300640324	1.83E-211	Arabidopsis thaliana auxin transporter protein 1 mRNA, complete cds
AT5G25460	-1.422911725	1.87E-208	Arabidopsis thaliana UF642 l-GalL-responsive protein 1 mRNA, complete cds
AT5G19890	-2.771385637	1.30E-207	Arabidopsis thaliana peroxidase 59 mRNA, complete cds
AT2G21660	-1.619042934	7.20E-207	Arabidopsis thaliana glycine-rich RNA-binding protein 7 mRNA, complete cds
AT5G44610	-3.100608574	2.84E-205	Arabidopsis thaliana microtubule-associated protein 18 mRNA, complete cds
AT2G39530	-3.753426995	3.12E-205	Arabidopsis thaliana uncharacterized protein mRNA, complete cds
AT4G00970	-2.539414239	3.22E-204	Arabidopsis thaliana cysteine-rich receptor-like protein kinase 41 mRNA, complete cds
AT5G19510	-1.006400293	1.29E-202	Arabidopsis thaliana Elongation factor 1-beta 2 mRNA, complete cds
AT3G10720	-1.480325999	3.73E-202	Arabidopsis thaliana pectinesterase 25

			mRNA, complete cds
AT2G04780	-1.716476424	4.76E-201	Arabidopsis thaliana chromosome 2, complete sequence
AT1G72430	-2.963912068	5.39E-200	Arabidopsis thaliana chromosome 1 sequence
AT1G08930	-1.158078795	5.08E-196	Arabidopsis thaliana sugar transporter ERD6 mRNA, complete cds
AT5G62720	-2.134912022	3.48E-194	Arabidopsis thaliana HPP integral membrane domain-containing protein mRNA, complete cds
AT3G01480	-1.156224829	6.04E-193	Arabidopsis thaliana peptidyl-prolyl cis-trans isomerase CYP38 mRNA, complete cds
AT4G24780	-1.22531405	1.47E-190	Arabidopsis thaliana putative pectate lyase 18 mRNA, complete cds
AT3G58990	-2.570766658	3.92E-190	Arabidopsis thaliana chromosome 3, complete sequence
AT5G19190	-2.461036144	7.32E-190	Arabidopsis thaliana uncharacterized protein mRNA, complete cds
AT4G11320	-1.281411033	9.45E-189	Arabidopsis thaliana putative cysteine proteinase mRNA, complete cds
AT3G25930	-3.54234357	1.92E-188	Arabidopsis thaliana Adenine nucleotide alpha hydrolases-like superfamily protein mRNA, complete cds
AT3G07010	-2.485313215	3.19E-188	Arabidopsis thaliana putative pectate lyase 8 mRNA, complete cds
AT4G34881	-1.259185194	1.12E-186	Arabidopsis thaliana chromosome 4 sequence
AT2G21045	-3.25719842	1.34E-186	Arabidopsis thaliana rhodanese-like domain-containing protein mRNA, complete cds
AT1G30510	-2.21693455	5.84E-186	Arabidopsis thaliana ferredoxin--NADP reductase, root isozyme 2 mRNA, complete cds
AT1G63940	-1.389585517	2.32E-185	Arabidopsis thaliana Monodehydroascorbate reductase mRNA, complete cds
AT1G55450	-1.462820083	2.17E-184	Arabidopsis thaliana S-adenosyl-L-methionine-dependent methyltransferases superfamily protein mRNA, complete cds
AT2G01530	-2.526475184	3.40E-184	Arabidopsis thaliana MLP-like protein 329 mRNA, complete cds
AT4G31910	-3.421232813	5.32E-183	Arabidopsis thaliana BR-related

			acyltransferase 1 mRNA, complete cds
AT2G29750	-3.590488424	9.06E-178	Arabidopsis thaliana chromosome 2, complete sequence
AT2G43100	-2.104650335	1.45E-177	Arabidopsis thaliana chromosome 2, complete sequence
AT3G51330	-2.44291902	4.36E-177	Arabidopsis thaliana aspartyl protease family protein mRNA, complete cds
AT4G08300	-2.958483493	1.37E-175	Arabidopsis thaliana nodulin MtN21 /EamA-like transporter family protein mRNA, complete cds
AT3G06070	-2.544008813	2.05E-175	Arabidopsis thaliana uncharacterized protein mRNA, complete cds
AT1G21270	-3.103315125	2.33E-174	Arabidopsis thaliana wall-associated receptor kinase 2 mRNA, complete cds
AT4G02290	-1.537607483	7.03E-173	Arabidopsis thaliana glycosyl hydrolase 9B13 mRNA, complete cds
AT1G28400	-1.409629185	6.88E-172	Arabidopsis thaliana chromosome 1 sequence
AT5G63600	-1.63305317	8.26E-172	Arabidopsis thaliana flavonol synthase 5 mRNA, complete cds
AT3G01690	-1.388192883	1.15E-171	Arabidopsis thaliana alpha/beta-hydrolase domain-containing protein mRNA, complete cds
AT1G77690	-2.474888209	1.95E-171	Arabidopsis thaliana auxin influx carrier LAX3 mRNA, complete cds
AT5G49740	-1.203835621	2.59E-166	Arabidopsis thaliana ferric reduction oxidase 7 mRNA, complete cds
AT5G04730	-4.932726967	3.46E-166	Arabidopsis thaliana Ankyrin-repeat containing protein mRNA, complete cds
AT5G61590	-2.462833437	2.60E-165	Arabidopsis thaliana chromosome 5 sequence
AT1G56430	-2.509193297	2.66E-162	Arabidopsis thaliana chromosome 1 sequence
AT3G16850	-1.442689468	3.82E-162	Arabidopsis thaliana pectin lyase-like superfamily protein mRNA, complete cds
AT2G33830	-1.331399465	5.31E-161	Arabidopsis thaliana dormancy/auxin associated protein mRNA, complete cds
AT2G44490	-1.130571843	3.67E-160	Arabidopsis thaliana beta-glucosidase 26 mRNA, complete cds
AT1G49600	-1.373962714	7.17E-158	Arabidopsis thaliana RNA-binding protein 47A mRNA, complete cds
AT3G03670	-4.354828931	7.70E-157	Arabidopsis thaliana peroxidase mRNA, complete cds

AT5G13710	-1.199817496	1.19E-156	Arabidopsis thaliana cycloartenol-c-24-methyltransferase mRNA, complete cds
AT3G54040	-2.182999475	4.12E-155	Arabidopsis thaliana PAR1 protein mRNA, complete cds
AT5G54380	-2.27866801	5.10E-155	Arabidopsis thaliana chromosome 5 sequence
AT4G02330	-3.001551516	5.98E-155	Arabidopsis thaliana Probable pectinesterase/pectinesterase inhibitor 41 mRNA, complete cds
AT3G45160	-2.31302543	1.36E-154	Arabidopsis thaliana putative membrane lipoprotein mRNA, complete cds
AT1G11545	-3.13740342	6.51E-154	Arabidopsis thaliana probable xyloglucan endotransglucosylase/hydrolase protein 8 mRNA, complete cds
AT5G42530	-1.30198649	6.01E-152	Arabidopsis thaliana uncharacterized protein mRNA, complete cds
AT5G53160	-1.310661653	5.96E-149	Arabidopsis thaliana regulatory component of ABA receptor 3 mRNA, complete cds
AT5G65730	-1.764802913	1.28E-148	Arabidopsis thaliana probable xyloglucan endotransglucosylase/hydrolase protein 6 mRNA, complete cds
AT4G20890	-1.313192499	3.40E-147	Arabidopsis thaliana tubulin beta-9 chain mRNA, complete cds
AT5G59680	-3.131181918	8.00E-147	Arabidopsis thaliana leucine-rich repeat protein kinase family protein mRNA, complete cds
AT1G74670	-1.744031959	8.05E-147	Arabidopsis thaliana gibberellin-regulated protein 6 mRNA, complete cds
AT1G33470	-1.418791296	1.61E-146	Arabidopsis thaliana RNA recognition motif-containing protein mRNA, complete cds
AT1G10682	-2.036104707	1.58E-145	Arabidopsis thaliana chromosome 1 sequence
AT1G25230	-2.445474029	5.18E-145	Arabidopsis thaliana Calcineurin-like metallo-phosphoesterase superfamily protein mRNA, complete cds
AT3G55330	-1.049446169	9.10E-145	Arabidopsis thaliana PsbP-like protein 1 mRNA, complete cds
AT5G19240	-1.091270669	1.73E-143	Arabidopsis thaliana GPI-anchored glycoprotein membrane precursor mRNA, complete cds

AT1G01190	-2.736002905	1.57E-142	Arabidopsis thaliana cytochrome P450, family 78, subfamily A, polypeptide 8 mRNA, complete cds
AT1G01580	-1.981198798	2.67E-140	Arabidopsis thaliana ferric reduction oxidase 2 mRNA, complete cds
AT5G44680	-2.234913216	4.42E-140	Arabidopsis thaliana putative 3-methyladenine glycosylase I mRNA, complete cds
AT5G10580	-3.108435196	4.36E-139	Arabidopsis thaliana uncharacterized protein mRNA, complete cds
AT2G22122	-3.365037453	5.28E-139	Arabidopsis thaliana chromosome 2, complete sequence
AT1G72930	-1.779658537	7.59E-138	Arabidopsis thaliana toll/interleukin-1 receptor-like protein mRNA, complete cds
AT4G15393	-3.692803873	2.24E-137	Arabidopsis thaliana cytochrome P450, family 702, subfamily A, polypeptide 5 mRNA, complete cds
AT5G12250	-1.098579593	3.21E-137	Arabidopsis thaliana beta-6 tubulin mRNA, complete cds
AT1G20693	-1.63889021	3.87E-137	Arabidopsis thaliana high mobility group B2 protein mRNA, complete cds
AT3G54600	-1.773107202	1.53E-136	Arabidopsis thaliana class I glutamine amidotransferase-like domain-containing protein mRNA, complete cds
AT5G53250	-5.151668432	2.09E-136	Arabidopsis thaliana mRNA for predicted GPI-anchored protein, complete cds, clone: RAFL16-23-F12
AT3G15850	-1.006464542	6.03E-136	Arabidopsis thaliana palmitoyl-monogalactosyldiacylglycerol delta-7 desaturase mRNA, complete cds
AT5G26130	-4.966921091	7.71E-134	Arabidopsis thaliana chromosome 5 sequence
AT3G22231	-3.135647852	1.55E-133	Arabidopsis thaliana protein PATHOGEN AND CIRCADIAN CONTROLLED 1 mRNA, complete cds
AT5G65980	-5.20743248	2.92E-133	Arabidopsis thaliana auxin efflux carrier family protein mRNA, complete cds
AT5G04950	-2.713819031	3.41E-133	Arabidopsis thaliana chromosome 5 sequence
AT4G17520	-1.03551845	3.69E-133	Arabidopsis thaliana hyaluronan / mRNA binding family mRNA, complete cds
AT1G27450	-1.259340499	6.22E-133	Arabidopsis thaliana adenine phosphoribosyl transferase 1 mRNA,

			complete cds
AT5G45490	-1.488477274	1.23E-132	Arabidopsis thaliana chromosome 5 sequence
AT5G43060	-1.218040068	2.34E-132	Arabidopsis thaliana Granulin repeat cysteine protease family protein mRNA, complete cds
AT2G33790	-2.675297667	1.20E-131	Arabidopsis thaliana arabinogalactan protein 30 mRNA, complete cds
AT1G05260	-1.754081368	2.10E-130	Arabidopsis thaliana peroxidase 3 mRNA, complete cds
AT5G04430	-1.031832513	2.31E-130	Arabidopsis thaliana TOMV RNA binding protein mRNA, complete cds
AT1G29670	-1.028836205	3.29E-130	Arabidopsis thaliana GDSL esterase/lipase mRNA, complete cds
AT4G02270	-4.549398328	1.32E-129	Arabidopsis thaliana protein root hair specific 13 mRNA, complete cds
AT3G52060	-1.034128596	3.22E-127	Arabidopsis thaliana chromosome 3, complete sequence
AT3G18050	-1.504023379	3.36E-127	Arabidopsis thaliana uncharacterized protein mRNA, complete cds
AT3G25860	-1.266246997	1.67E-126	Arabidopsis thaliana dihydrolipoamide S-acetyltransferase mRNA, complete cds
AT2G47240	-1.390048289	2.15E-126	Arabidopsis thaliana long chain acyl-CoA synthetase 1 mRNA, complete cds
AT4G37800	-1.666749941	1.82E-124	Arabidopsis thaliana xyloglucan endotransglucosylase/hydrolase protein 7 mRNA, complete cds
AT1G10470	-1.104373061	7.97E-124	Arabidopsis thaliana two-component response regulator ARR4 mRNA, complete cds
AT5G65970	-2.917170723	1.10E-123	Arabidopsis thaliana MLO-like protein 10 mRNA, complete cds
AT5G59090	-1.123202385	1.68E-123	Arabidopsis thaliana subtilase 4.12 mRNA, complete cds
AT3G20380	-4.339115589	2.05E-123	Arabidopsis thaliana TRAF-like family protein mRNA, complete cds
AT4G21210	-1.138051677	2.13E-123	Arabidopsis thaliana pyruvate, phosphate dikinase regulatory protein 1 mRNA, complete cds
AT1G20840	-1.755109618	1.73E-122	Arabidopsis thaliana tonoplast monosaccharide transporter1 mRNA, complete cds
AT2G37710	-1.424819458	2.87E-122	Arabidopsis thaliana chromosome 2,

			complete sequence
AT1G70880	-3.880937814	1.07E-121	Arabidopsis thaliana SRPBCC domain-containing protein mRNA, complete cds
AT3G29030	-1.835223273	3.12E-121	Arabidopsis thaliana expansin A5 mRNA, complete cds
AT1G57680	-1.534243284	6.84E-121	Arabidopsis thaliana uncharacterized protein mRNA, complete cds
AT3G24300	-2.944723482	8.53E-121	Arabidopsis thaliana chromosome 3, complete sequence
AT4G10380	-1.424175189	1.01E-120	Arabidopsis thaliana putative aquaporin NIP5-1 mRNA, complete cds
AT5G14330	-4.625529397	1.65E-120	Arabidopsis thaliana uncharacterized protein mRNA, complete cds
AT4G03210	-1.783452904	1.97E-120	Arabidopsis thaliana xyloglucan endotransglucosylase/hydrolase 9 mRNA, complete cds
AT4G08950	-1.780715123	2.87E-120	Arabidopsis thaliana chromosome 4 sequence
AT1G21600	-1.565886451	6.36E-119	Arabidopsis thaliana plastid transcriptionally active 6 mRNA, complete cds
AT1G12500	-1.69209291	1.13E-118	Arabidopsis thaliana Nucleotide-sugar transporter family protein mRNA, complete cds
AT1G03475	-1.004236571	1.23E-118	Arabidopsis thaliana coproporphyrinogen III oxidase mRNA, complete cds
AT5G15350	-1.602213023	1.19E-117	Arabidopsis thaliana early nodulin-like protein 17 mRNA, complete cds
AT1G13300	-2.867218901	1.56E-117	Arabidopsis thaliana protein HRS1 mRNA, complete cds
AT3G27060	-1.729948571	1.84E-117	Arabidopsis thaliana ribonucleoside-diphosphate reductase small chain C mRNA, complete cds
AT1G51070	-1.452826554	1.40E-116	Arabidopsis thaliana transcription factor bHLH115 mRNA, complete cds
AT5G13420	-1.177043469	2.28E-116	Arabidopsis thaliana Aldolase-type TIM barrel family protein mRNA, complete cds
AT1G06830	-3.705606522	3.19E-115	Arabidopsis thaliana chromosome 1 sequence
AT1G54580	-1.221078131	3.93E-115	Arabidopsis thaliana acyl carrier protein 2 mRNA, complete cds
AT3G52150	-1.019236686	7.68E-115	Arabidopsis thaliana RNA recognition

			motif-containing protein mRNA, complete cds
AT5G07580	-2.400707968	1.70E-114	Arabidopsis thaliana chromosome 5 sequence
AT4G23820	-1.793566289	3.01E-114	Arabidopsis thaliana glycoside hydrolase family 28 protein / polygalacturonase (pectinase) family protein mRNA, complete cds
AT5G28500	-1.043567661	9.64E-113	Arabidopsis thaliana chromosome 5 sequence
AT1G75780	-2.725426225	1.55E-112	Arabidopsis thaliana tubulin beta-1 chain mRNA, complete cds
AT5G65430	-1.091845598	1.62E-112	Arabidopsis thaliana 14-3-3-like protein GF14 kappa mRNA, complete cds
AT2G36620	-1.138555675	2.73E-112	Arabidopsis thaliana 60S ribosomal protein L24-1 mRNA, complete cds
AT3G12900	-5.521266893	1.13E-111	Arabidopsis thaliana oxidoreductase, 2OG-Fe(II) oxygenase family protein mRNA, complete cds
AT5G57490	-1.426792046	2.44E-111	Arabidopsis thaliana voltage dependent anion channel 4 mRNA, complete cds
AT5G05960	-2.210106614	2.50E-111	Arabidopsis thaliana bifunctional inhibitor/lipid-transfer protein/seed storage 2S albumin superfamily protein mRNA, complete cds
AT1G11540	-4.873314976	3.28E-111	Arabidopsis thaliana Sulfite exporter TauE/SafE family protein mRNA, complete cds
AT5G46290	-1.048497924	4.82E-111	Arabidopsis thaliana beta-ketoacyl-[acyl carrier protein] synthase I mRNA, complete cds
AT4G11290	-1.749364682	4.95E-111	Arabidopsis thaliana peroxidase 39 mRNA, complete cds
AT5G65210	-1.204253888	8.24E-111	Arabidopsis thaliana transcription factor TGA1 mRNA, complete cds
AT1G44800	-1.046210062	9.87E-111	Arabidopsis thaliana bidirectional amino acid transporter SIAR1 mRNA, complete cds
AT1G30250	-1.647054963	1.41E-110	Arabidopsis thaliana chromosome 1 sequence
AT1G72910	-3.534396165	2.36E-110	Arabidopsis thaliana Toll-Interleukin-Resistance domain-containing protein mRNA, complete cds

AT4G08770	-1.869204452	3.92E-110	Arabidopsis thaliana peroxidase 37 mRNA, complete cds
AT3G13610	-2.318443893	1.05E-109	Arabidopsis thaliana feruloyl CoA ortho-hydroxylase 1 mRNA, complete cds
AT2G03440	-1.509800473	3.25E-109	Arabidopsis thaliana chromosome 2, complete sequence
AT3G49670	-1.982357735	3.66E-108	Arabidopsis thaliana CLAVATA1-related receptor kinase-like protein mRNA, complete cds
AT3G02850	-2.804117466	6.65E-108	Arabidopsis thaliana potassium channel SKOR mRNA, complete cds
AT1G80280	-1.63019347	9.59E-108	Arabidopsis thaliana alpha/beta-Hydrolases superfamily protein mRNA, complete cds
AT5G20020	-1.012750379	4.81E-107	Arabidopsis thaliana GTP-binding nuclear protein Ran-2 mRNA, complete cds
AT1G11310	-1.041239824	7.02E-106	Arabidopsis thaliana MLO-like protein 2 mRNA, complete cds
AT1G04680	-1.134234138	2.57E-105	Arabidopsis thaliana putative pectate lyase 1 mRNA, complete cds
AT5G61130	-1.409504917	3.83E-105	Arabidopsis thaliana plasmodesmata callose-binding protein 1 mRNA, complete cds
AT1G52060	-3.040751585	8.68E-105	Arabidopsis thaliana jacalin-like lectin domain-containing protein mRNA, complete cds
AT2G02130	-1.434635355	1.41E-104	Arabidopsis thaliana defensin-like protein 1 mRNA, complete cds
AT1G49750	-1.349250151	1.97E-104	Arabidopsis thaliana leucine-rich repeat (LRR) family protein mRNA, complete cds
AT5G65700	-1.006417707	7.90E-104	Arabidopsis thaliana leucine-rich repeat receptor-like serine/threonine-protein kinase BAM1 mRNA, complete cds
AT3G54580	-3.179295639	9.60E-104	Arabidopsis thaliana chromosome 3, complete sequence
AT2G05990	-1.109312868	1.27E-103	Arabidopsis thaliana enoyl-[acyl-carrier-protein] reductase [NADH] mRNA, complete cds
AT5G22880	-1.252813081	1.28E-103	Arabidopsis thaliana chromosome 5 sequence
AT3G54920	-1.113321552	1.95E-103	Arabidopsis thaliana putative pectate

			lyase mRNA, complete cds
AT1G55360	-1.032271272	9.96E-103	Arabidopsis thaliana uncharacterized protein mRNA, complete cds
AT2G25680	-2.623560246	1.10E-102	Arabidopsis thaliana chromosome 2, complete sequence
AT2G36570	-1.861007174	1.43E-102	Arabidopsis thaliana PXY/TDR-correlated protein PXC1 mRNA, complete cds
AT5G57625	-4.761806661	2.59E-102	Arabidopsis thaliana CAP (Cysteine-rich secretory proteins, Antigen 5, and Pathogenesis-related 1 protein) superfamily protein mRNA, complete cds
AT5G48230	-1.370355982	5.03E-102	Arabidopsis thaliana acetoacetyl-CoA thiolase 2 mRNA, complete cds
AT5G62890	-1.036357196	5.90E-102	Arabidopsis thaliana Xanthine/uracil permease family protein mRNA, complete cds
AT2G40490	-1.035500732	1.96E-101	Arabidopsis thaliana Uroporphyrinogen decarboxylase mRNA, complete cds
AT4G27130	-1.014653299	2.28E-101	Arabidopsis thaliana protein translation factor SUI1-1 mRNA, complete cds
AT4G37540	-1.756087977	8.74E-100	Arabidopsis thaliana LOB domain-containing protein 39 mRNA, complete cds
AT3G52500	-1.217486144	3.37E-99	Arabidopsis thaliana chromosome 3, complete sequence
AT2G28160	-2.933217904	3.41E-99	Arabidopsis thaliana FER-LIKE IRON DEFICIENCY-INDUCED transcription factor mRNA, complete cds
AT3G46280	-2.321234069	3.42E-99	Arabidopsis thaliana protein kinase-like protein mRNA, complete cds
AT3G43810	-1.412245925	6.68E-99	Arabidopsis thaliana calmodulin 7 mRNA, complete cds
AT2G36460	-1.079814699	1.17E-98	Arabidopsis thaliana fructose-bisphosphate aldolase 6 mRNA, complete cds
AT4G34950	-1.661792164	3.19E-98	Arabidopsis thaliana major facilitator family protein mRNA, complete cds
AT4G39260	-1.65939566	7.25E-98	Arabidopsis thaliana glycine-rich RNA-binding protein 8 mRNA, complete cds
AT4G07820	-3.770870743	8.11E-97	Arabidopsis thaliana chromosome 4 sequence

AT1G32450	-1.295824597	1.07E-96	Arabidopsis thaliana nitrate transporter 1.5 mRNA, complete cds
AT4G21250	-3.992586867	1.58E-96	Arabidopsis thaliana Sulfite exporter TauE/SafE family protein mRNA, complete cds
AT1G09750	-1.537221693	8.12E-96	Arabidopsis thaliana aspartyl protease-like protein mRNA, complete cds
AT5G16250	-1.608040959	1.82E-95	Arabidopsis thaliana chromosome 5 sequence
AT1G33240	-1.134242248	2.15E-95	Arabidopsis thaliana trihelix transcription factor GTL1 mRNA, complete cds
AT1G56110	-1.094962649	3.28E-95	Arabidopsis thaliana nucleolar protein NOP56-like protein mRNA, complete cds
AT5G05250	-2.609363117	7.45E-95	Arabidopsis thaliana chromosome 5 sequence
AT5G66040	-1.02348179	3.28E-94	Arabidopsis thaliana thiosulfate sulfurtransferase 16 mRNA, complete cds
AT4G37070	-2.65262485	8.68E-94	Arabidopsis thaliana patatin-related phospholipase A mRNA, complete cds
AT3G53190	-1.452315669	1.11E-93	Arabidopsis thaliana putative pectate lyase 12 mRNA, complete cds
AT1G33600	-1.672108353	1.13E-93	Arabidopsis thaliana chromosome 1 sequence
AT5G37600	-1.144343289	1.81E-93	Arabidopsis thaliana glutamine synthetase 1;1 mRNA, complete cds
AT1G60000	-1.407695242	5.56E-93	Arabidopsis thaliana RNA recognition motif-containing protein mRNA, complete cds
AT4G08380	-3.627641422	7.24E-93	Arabidopsis thaliana chromosome 4 sequence
AT4G08620	-3.721857281	8.25E-93	Arabidopsis thaliana sulfate transporter 1.1 mRNA, complete cds
AT2G37130	-1.317922096	1.07E-92	Arabidopsis thaliana peroxidase mRNA, complete cds
AT3G29250	-2.695332383	2.73E-92	Arabidopsis thaliana short-chain dehydrogenase reductase 4 mRNA, complete cds
AT4G22666	-5.561782052	2.81E-92	Arabidopsis thaliana Bifunctional inhibitor/lipid-transfer protein/seed storage 2S albumin superfamily protein mRNA, complete cds

AT5G26260	-1.407385846	4.49E-92	Arabidopsis thaliana TRAF-like family protein mRNA, complete cds
AT4G28250	-1.339531741	2.12E-91	Arabidopsis thaliana expansin B3 mRNA, complete cds
AT1G11680	-1.116656145	2.77E-91	Arabidopsis thaliana sterol 14-demethylase mRNA, complete cds
AT5G40510	-3.845222669	2.92E-91	Arabidopsis thaliana Sucrase/ferredoxin-like family protein mRNA, complete cds
AT5G24410	-3.667519065	4.47E-91	Arabidopsis thaliana 6-phosphogluconolactonase 4 mRNA, complete cds
AT2G24060	-1.340895466	5.13E-91	Arabidopsis thaliana translation initiation factor IF-3 mRNA, complete cds
AT5G53490	-1.113673815	8.19E-91	Arabidopsis thaliana thylakoid lumenal protein-17.4 mRNA, complete cds
AT4G17870	-1.333713009	9.58E-91	Arabidopsis thaliana chromosome 4 sequence
AT3G51350	-3.089220295	1.15E-90	Arabidopsis thaliana aspartyl protease family protein mRNA, complete cds
AT5G21930	-1.098187216	1.61E-90	Arabidopsis thaliana P-type ATPase mRNA, complete cds
AT5G43520	-2.243524005	8.60E-90	Arabidopsis thaliana chromosome 5 sequence
AT4G30670	-4.038516003	9.84E-90	Arabidopsis thaliana chromosome 4 sequence
AT4G23570	-1.053800825	1.43E-89	Arabidopsis thaliana phosphatase SGT1a mRNA, complete cds
AT1G54630	-1.140094974	2.84E-89	Arabidopsis thaliana acyl carrier protein 3 mRNA, complete cds
AT5G61440	-2.512696901	2.90E-89	Arabidopsis thaliana atypical CYS HIS rich thioredoxin 5 mRNA, complete cds
AT5G66920	-1.279073943	7.44E-89	Arabidopsis thaliana protein SKU5 similar 17 mRNA, complete cds
AT4G13495	-1.016608215	7.48E-89	Arabidopsis thaliana mRNA for hypothetical protein, complete cds, clone: RAFL25-35-C19
AT5G19600	-2.313800535	8.29E-89	Arabidopsis thaliana sulfate transporter 3;5 mRNA, complete cds
AT4G40090	-3.704473486	8.65E-89	Arabidopsis thaliana chromosome 4 sequence
AT1G62780	-1.202993121	1.74E-88	Arabidopsis thaliana uncharacterized protein mRNA, complete cds
AT5G05440	-2.235408052	2.46E-88	Arabidopsis thaliana chromosome 5

			sequence
AT2G18980	-4.170977153	3.21E-88	Arabidopsis thaliana peroxidase 16 mRNA, complete cds
AT5G57685	-2.011035267	3.39E-88	Arabidopsis thaliana chromosome 5 sequence
AT4G04570	-1.563014598	1.36E-87	Arabidopsis thaliana cysteine-rich receptor-like protein kinase 40 mRNA, complete cds
AT5G58250	-1.117950471	1.61E-87	Arabidopsis thaliana uncharacterized protein mRNA, complete cds
AT3G01440	-2.205334586	2.25E-87	Arabidopsis thaliana PsbQ-like protein 1 mRNA, complete cds
AT2G27402	-2.703112666	4.10E-87	Arabidopsis thaliana chromosome 2, complete sequence
AT3G10520	-2.582915878	6.07E-87	Arabidopsis thaliana non-symbiotic hemoglobin 2 mRNA, complete cds
AT5G10430	-1.579047699	7.17E-87	Arabidopsis thaliana chromosome 5 sequence
AT3G23175	-3.044740624	8.27E-87	Arabidopsis thaliana HR-like lesion-inducing protein-like protein mRNA, complete cds
AT3G59370	-4.045446271	1.03E-86	Arabidopsis thaliana vacuolar calcium-binding protein-like protein mRNA, complete cds
AT4G25820	-3.313532832	1.59E-86	Arabidopsis thaliana xyloglucan endotransglucosylase/hydrolase protein 14 mRNA, complete cds
AT3G56680	-1.010321743	9.57E-86	Arabidopsis thaliana Single-stranded nucleic acid binding R3H protein mRNA, complete cds
AT2G28960	-2.862696342	2.74E-85	Arabidopsis thaliana putative LRR receptor-like serine/threonine-protein kinase mRNA, complete cds
AT1G09560	-1.039728296	4.24E-85	Arabidopsis thaliana germin-like protein 5 mRNA, complete cds
AT3G17650	-1.002351801	9.93E-85	Arabidopsis thaliana putative metal-nicotianamine transporter YSL5 mRNA, complete cds
AT2G38380	-1.15304986	1.02E-84	Arabidopsis thaliana peroxidase 22 mRNA, complete cds
AT2G40610	-2.067432943	1.13E-84	Arabidopsis thaliana expansin A8 mRNA, complete cds
AT3G32030	-3.019104697	1.20E-84	Arabidopsis thaliana terpenoid synthase 30 mRNA, complete cds

AT2G24710	-4.512872452	1.23E-84	Arabidopsis thaliana glutamate receptor 2.3 mRNA, complete cds
AT3G12610	-1.725317739	3.63E-84	Arabidopsis thaliana chromosome 3, complete sequence
AT4G31840	-1.80497084	4.21E-84	Arabidopsis thaliana early nodulin-like protein 15 mRNA, complete cds
AT1G29025	-1.816223789	8.24E-84	Arabidopsis thaliana Calcium-binding EF-hand family protein mRNA, complete cds
AT4G22010	-1.566894976	1.21E-83	Arabidopsis thaliana protein SKU5 similar 4 mRNA, complete cds
AT5G15830	-3.372705951	1.31E-83	Arabidopsis thaliana chromosome 5 sequence
AT5G38930	-3.819522159	1.83E-83	Arabidopsis thaliana germin-like protein subfamily 1 member 10 mRNA, complete cds
AT3G28040	-1.268329323	5.72E-83	Arabidopsis thaliana probably inactive leucine-rich repeat receptor-like protein kinase mRNA, complete cds
AT3G23730	-2.147671024	6.61E-83	Arabidopsis thaliana xyloglucan endotransglucosylase/hydrolase protein 16 mRNA, complete cds
AT2G37940	-1.350049357	8.82E-83	Arabidopsis thaliana inositol phosphorylceramide synthase 2 mRNA, complete cds
AT1G01080	-1.318467839	1.01E-82	Arabidopsis thaliana RNA-binding (RRM/RBD/RNP motifs) family protein mRNA, complete cds
AT5G57180	-1.4577413	2.23E-82	Arabidopsis thaliana chloroplast import apparatus 2 protein mRNA, complete cds
AT5G38990	-1.301558396	3.49E-82	Arabidopsis thaliana chromosome 5 sequence
AT5G26880	-1.376422653	7.07E-82	Arabidopsis thaliana protein AGAMOUS-LIKE 26 mRNA, complete cds
AT5G43360	-4.466204392	1.24E-81	Arabidopsis thaliana putative inorganic phosphate transporter 1-3 mRNA, complete cds
AT2G45220	-1.73513871	1.36E-81	Arabidopsis thaliana putative pectinesterase/pectinesterase inhibitor 17 mRNA, complete cds
AT4G12980	-1.649528936	1.50E-81	Arabidopsis thaliana putative auxin-responsive protein mRNA, complete cds

AT1G30870	-3.423937022	1.70E-81	Arabidopsis thaliana peroxidase 7 mRNA, complete cds
AT3G23190	-2.878724412	1.01E-80	Arabidopsis thaliana HR-like lesion-inducing protein-like protein mRNA, complete cds
AT5G03300	-1.109442689	1.09E-80	Arabidopsis thaliana adenosine kinase 2 mRNA, complete cds
AT2G36320	-1.133220718	1.95E-80	Arabidopsis thaliana zinc finger A20 and AN1 domain-containing stress-associated protein 4 mRNA, complete cds
AT1G63570	-4.057262544	2.02E-80	Arabidopsis thaliana chromosome 1 sequence
AT1G24360	-1.008741894	6.50E-80	Arabidopsis thaliana 3-oxoacyl-[acyl-carrier-protein] reductase mRNA, complete cds
AT3G20370	-2.581539715	7.02E-80	Arabidopsis thaliana TRAF-like family protein mRNA, complete cds
AT1G48630	-1.317321412	8.74E-80	Arabidopsis thaliana receptor for activated C kinase 1B mRNA, complete cds
AT4G12600	-1.328491866	1.74E-79	Arabidopsis thaliana ribosomal protein L7Ae/L30e/S12e/Gadd45 family protein mRNA, complete cds
AT1G09740	-1.79143183	2.28E-78	Arabidopsis thaliana adenine nucleotide alpha hydrolases-like protein mRNA, complete cds
AT2G36100	-2.126484403	3.18E-78	Arabidopsis thaliana casparian strip membrane protein 1 mRNA, complete cds
AT2G01950	-1.535344575	1.30E-77	Arabidopsis thaliana chromosome 2, complete sequence
AT1G64190	-1.096972211	1.45E-77	Arabidopsis thaliana chromosome 1 sequence
AT4G25630	-1.922986969	2.77E-77	Arabidopsis thaliana mediator of RNA polymerase II transcription subunit 36a mRNA, complete cds
AT5G49770	-3.580160582	2.82E-77	Arabidopsis thaliana putative leucine-rich repeat receptor-like protein kinase mRNA, complete cds
AT3G20015	-2.828920857	1.02E-76	Arabidopsis thaliana chromosome 3, complete sequence
AT4G13620	-3.896966244	1.18E-76	Arabidopsis thaliana chromosome 4 sequence
AT3G06750	-1.409017602	3.58E-76	Arabidopsis thaliana chromosome 3,

			complete sequence
AT2G39870	-1.422889555	4.55E-76	Arabidopsis thaliana uncharacterized protein mRNA, complete cds
AT1G64650	-1.208652898	5.11E-76	Arabidopsis thaliana general substrate transporter-like protein mRNA, complete cds
AT4G38520	-1.170446389	5.93E-76	Arabidopsis thaliana putative protein phosphatase 2C 64 mRNA, complete cds
AT1G11080	-1.766076255	1.44E-75	Arabidopsis thaliana serine carboxypeptidase-like 31 mRNA, complete cds
AT1G12440	-1.172236379	1.44E-75	Arabidopsis thaliana chromosome 1 sequence
AT1G47670	-1.780107477	3.10E-75	Arabidopsis thaliana Lysine histidine transporter-like 8 mRNA, complete cds
AT2G36410	-1.370675177	4.13E-75	Arabidopsis thaliana uncharacterized protein mRNA, complete cds
AT4G12510	-7.338156031	1.07E-74	Arabidopsis thaliana chromosome 4 sequence
AT1G48480	-1.519936865	3.01E-74	Arabidopsis thaliana receptor-like kinase 1 mRNA, complete cds
AT4G02930	-1.219892642	3.39E-74	Arabidopsis thaliana putative elongation factor Tu mRNA, complete cds
AT4G25740	-1.211730836	5.57E-74	Arabidopsis thaliana 40S ribosomal protein S10-1 mRNA, complete cds
AT5G47770	-1.036685664	1.01E-73	Arabidopsis thaliana farnesyl diphosphate synthase 1 mRNA, complete cds
AT5G11450	-1.213078518	1.85E-73	Arabidopsis thaliana PsbP domain-containing protein 5 mRNA, complete cds
AT2G40330	-5.074545584	2.18E-73	Arabidopsis thaliana chromosome 2, complete sequence
AT5G41670	-1.229438264	2.32E-73	Arabidopsis thaliana chromosome 5 sequence
AT4G32570	-1.458955359	2.36E-73	Arabidopsis thaliana protein TIFY 8 mRNA, complete cds
AT1G60680	-2.793170832	7.59E-73	Arabidopsis thaliana probable aldo-keto reductase 2 mRNA, complete cds
AT3G04550	-1.333434523	1.51E-72	Arabidopsis thaliana chromosome 3, complete sequence
AT2G19760	-1.206423249	2.14E-72	Arabidopsis thaliana profilin 1 mRNA, complete cds
AT1G14720	-1.581369556	5.16E-72	Arabidopsis thaliana probable xyloglucan

			endotransglucosylase/hydrolase protein 28 mRNA, complete cds
AT1G54950	-5.86614332	1.00E-71	Arabidopsis thaliana uncharacterized protein mRNA, complete cds
AT1G64380	-1.766339165	2.59E-71	Arabidopsis thaliana chromosome 1 sequence
AT5G52780	-2.058121667	2.93E-71	Arabidopsis thaliana chromosome 5 sequence
AT3G26710	-1.075081444	6.66E-71	Arabidopsis thaliana cofactor assembly of complex C mRNA, complete cds
AT3G44990	-1.940034966	7.50E-71	Arabidopsis thaliana xyloglucan endotransglucosylase/hydrolase mRNA, complete cds
AT1G33610	-2.139730987	7.57E-71	Arabidopsis thaliana leucine-rich repeat (LRR) family protein mRNA, complete cds
AT3G15630	-1.56257196	8.38E-71	Arabidopsis thaliana chromosome 3, complete sequence
AT4G29740	-2.656410883	1.25E-70	Arabidopsis thaliana cytokinin dehydrogenase 4 mRNA, complete cds
AT1G53830	-2.821375927	1.69E-70	Arabidopsis thaliana pectin methylesterase 2 mRNA, complete cds
AT2G35120	-1.328623701	1.97E-70	Arabidopsis thaliana glycine cleavage system H protein 2 mRNA, complete cds
AT2G41480	-1.766420336	4.25E-70	Arabidopsis thaliana putative peroxidase mRNA, complete cds
AT4G23690	-1.728233553	4.44E-70	Arabidopsis thaliana chromosome 4 sequence
AT5G64620	-2.130301574	5.11E-70	Arabidopsis thaliana chromosome 5 sequence
AT3G21770	-1.123676573	6.05E-70	Arabidopsis thaliana peroxidase 30 mRNA, complete cds
AT5G63180	-1.639187374	6.15E-70	Arabidopsis thaliana putative pectate lyase 22 mRNA, complete cds
AT5G25265	-1.084728994	7.84E-70	Arabidopsis thaliana uncharacterized protein mRNA, complete cds
AT1G22500	-2.485922833	1.62E-69	Arabidopsis thaliana chromosome 1 sequence
AT4G29310	-2.904411056	2.34E-69	Arabidopsis thaliana uncharacterized protein mRNA, complete cds
AT1G23720	-1.817301773	4.16E-69	Arabidopsis thaliana chromosome 1 sequence
AT4G36540	-1.346611945	4.72E-69	Arabidopsis thaliana transcription factor BEE 2 mRNA, complete cds

AT1G80480	-1.298589432	6.12E-69	Arabidopsis thaliana protein plastid transcriptionally active 17 mRNA, complete cds
AT2G14880	-1.338214533	6.22E-69	Arabidopsis thaliana SWIB/MDM2 domain-containing protein mRNA, complete cds
AT4G30140	-2.342722328	6.76E-69	Arabidopsis thaliana GDSL esterase/lipase mRNA, complete cds
AT5G08260	-1.433493106	1.17E-68	Arabidopsis thaliana serine carboxypeptidase-like 35 mRNA, complete cds
AT2G38390	-1.359673496	1.26E-68	Arabidopsis thaliana peroxidase 23 mRNA, complete cds
AT4G38860	-1.967587141	1.28E-68	Arabidopsis thaliana chromosome 4 sequence
AT3G49720	-1.108840052	1.38E-68	Arabidopsis thaliana uncharacterized protein mRNA, complete cds
AT4G14130	-1.840976874	2.27E-68	Arabidopsis thaliana probable xyloglucan endotransglucosylase/hydrolase protein 15 mRNA, complete cds
AT1G72645	-1.587370696	2.79E-68	Arabidopsis thaliana chromosome 1 sequence
AT4G33720	-2.085809084	4.16E-68	Arabidopsis thaliana chromosome 4 sequence
AT2G19800	-2.255621273	4.56E-68	Arabidopsis thaliana myo-inositol oxygenase 2 mRNA, complete cds
AT1G14210	-1.931061918	4.57E-68	Arabidopsis thaliana ribonuclease T2 family protein mRNA, complete cds
AT5G19530	-1.464470554	5.69E-68	Arabidopsis thaliana Thermospermine synthase ACAULIS5 mRNA, complete cds
AT4G39675	-4.225914767	1.41E-67	Arabidopsis thaliana chromosome 4 sequence
AT5G66530	-1.120767293	1.49E-67	Arabidopsis thaliana aldose 1-epimerase family protein mRNA, complete cds
AT3G05490	-1.673196049	1.61E-67	Arabidopsis thaliana chromosome 3, complete sequence
AT2G28850	-7.168770859	2.07E-67	Arabidopsis thaliana chromosome 2, complete sequence
AT2G43150	-1.136540979	2.42E-67	Arabidopsis thaliana Proline-rich extensin-like family protein mRNA, complete cds
AT3G56480	-1.506073164	4.18E-67	Arabidopsis thaliana myosin heavy chain-like protein mRNA, complete cds

AT4G10310	-2.30386057	4.46E-67	Arabidopsis thaliana sodium transporter HKT1 mRNA, complete cds
AT5G62900	-2.149599324	4.58E-67	Arabidopsis thaliana uncharacterized protein mRNA, complete cds
AT1G51060	-1.045826092	4.87E-67	Arabidopsis thaliana histone H2A 10 mRNA, complete cds
AT1G76090	-1.579491679	5.51E-67	Arabidopsis thaliana chromosome 1 sequence
AT1G61790	-1.093088714	2.94E-66	Arabidopsis thaliana chromosome 1 sequence
AT3G54260	-1.374464833	3.13E-66	Arabidopsis thaliana protein TRICHOME BIREFRINGENCE-LIKE 36 mRNA, complete cds
AT2G02680	-3.821449607	4.37E-66	Arabidopsis thaliana cysteine/histidine-rich C1 domain-containing protein mRNA, complete cds
AT3G18450	-4.664945121	5.80E-66	Arabidopsis thaliana PLAC8 family protein mRNA, complete cds
AT1G55330	-2.428710775	6.73E-66	Arabidopsis thaliana chromosome 1 sequence
AT4G37450	-1.463465386	8.90E-66	Arabidopsis thaliana Lysine-rich arabinogalactan protein 18 mRNA, complete cds
AT1G10200	-1.475548035	9.30E-66	Arabidopsis thaliana transcription factor lim1 mRNA, complete cds
AT2G17710	-2.097409692	9.96E-66	Arabidopsis thaliana uncharacterized protein mRNA, complete cds
AT4G33420	-1.259165823	1.23E-65	Arabidopsis thaliana probable peroxidase mRNA, complete cds
AT4G20110	-1.344947054	1.79E-65	Arabidopsis thaliana vacuolar-sorting receptor 7 mRNA, complete cds
AT2G33430	-1.141679475	2.24E-65	Arabidopsis thaliana protein differentiation and greening-like 1 mRNA, complete cds
AT5G44380	-1.12791214	2.94E-65	Arabidopsis thaliana FAD-binding and BBE domain-containing protein mRNA, complete cds
AT3G54590	-3.856755065	4.50E-65	Arabidopsis thaliana hydroxyproline-rich glycoprotein mRNA, complete cds
AT4G21870	-2.668280834	7.53E-65	Arabidopsis thaliana heat shock protein class V 15.4 mRNA, complete cds
AT4G29080	-1.28963593	9.05E-65	Arabidopsis thaliana phytochrome-associated protein 2

			mRNA, complete cds
AT1G53940	-3.794228138	1.40E-64	Arabidopsis thaliana GDSL-motif lipase 2 mRNA, complete cds
AT4G13850	-1.242488468	7.47E-64	Arabidopsis thaliana glycine-rich RNA-binding protein 2 mRNA, complete cds
AT4G03110	-1.277639279	9.39E-64	Arabidopsis thaliana RNA-binding protein-defense related 1 mRNA, complete cds
AT1G65310	-3.186304489	9.57E-64	Arabidopsis thaliana probable xyloglucan endotransglucosylase/hydrolase protein 17 mRNA, complete cds
AT5G04960	-5.03660078	1.13E-63	Arabidopsis thaliana putative pectinesterase/pectinesterase inhibitor 46 mRNA, complete cds
AT1G52050	-2.903052586	3.91E-63	Arabidopsis thaliana jacalin-like lectin domain-containing protein mRNA, complete cds
AT2G15090	-2.379585116	5.55E-63	Arabidopsis thaliana chromosome 2, complete sequence
AT2G46330	-1.193790052	7.89E-63	Arabidopsis thaliana chromosome 2, complete sequence
AT1G74030	-1.460091787	8.94E-63	Arabidopsis thaliana enolase 1 mRNA, complete cds
AT1G12010	-1.471071449	1.37E-62	Arabidopsis thaliana 1-aminocyclopropane-1-carboxylate oxidase mRNA, complete cds
AT1G52070	-1.991541057	2.05E-62	Arabidopsis thaliana jacalin-like lectin domain-containing protein mRNA, complete cds
AT1G78460	-2.360444098	2.82E-62	Arabidopsis thaliana SOUL heme-binding protein mRNA, complete cds
AT5G65480	-1.105998211	3.64E-62	Arabidopsis thaliana uncharacterized protein mRNA, complete cds
AT5G49440	-1.043201604	4.41E-62	Arabidopsis thaliana chromosome 5 sequence
AT5G12940	-1.904305631	6.44E-62	Arabidopsis thaliana chromosome 5 sequence
AT4G19030	-2.26769759	7.39E-62	Arabidopsis thaliana aquaporin NIP1-1 mRNA, complete cds
AT3G45710	-2.239494765	1.52E-61	Arabidopsis thaliana putative nitrate excretion transporter 6 mRNA, complete cds

AT4G38160	-1.250599894	3.00E-61	Arabidopsis thaliana protein PIGMENT DEFECTIVE 191 mRNA, complete cds
AT4G38660	-1.468096835	3.88E-61	Arabidopsis thaliana pathogenesis-related thaumatin-like protein mRNA, complete cds
AT5G54370	-1.664232503	7.60E-61	Arabidopsis thaliana late embryogenesis abundant protein-like protein mRNA, complete cds
AT4G39040	-1.001757453	9.41E-61	Arabidopsis thaliana putative RNA-binding protein containing KH domain mRNA, complete cds
AT3G43800	-1.964367207	1.26E-60	Arabidopsis thaliana glutathione S-transferase tau 27 mRNA, complete cds
AT2G02450	-1.979630302	1.29E-60	Arabidopsis thaliana protein LONG VEGETATIVE PHASE 1 mRNA, complete cds
AT4G33360	-1.708572054	1.67E-60	Arabidopsis thaliana Rossmann-fold NAD(P)-binding domain-containing protein mRNA, complete cds
AT3G11800	-1.335243852	1.97E-60	Arabidopsis thaliana uncharacterized protein mRNA, complete cds
AT5G15530	-1.590238498	2.03E-60	Arabidopsis thaliana biotin carboxyl carrier protein 2 mRNA, complete cds
AT3G28550	-2.512820029	2.09E-60	Arabidopsis thaliana chromosome 3, complete sequence
AT1G47600	-1.767191867	2.27E-60	Arabidopsis thaliana myrosinase 4 mRNA, complete cds
AT2G28780	-1.530675902	2.67E-60	Arabidopsis thaliana uncharacterized protein mRNA, complete cds
AT5G28770	-1.062976195	3.26E-60	Arabidopsis thaliana basic leucine zipper 63 mRNA, complete cds
AT1G66940	-2.113467841	3.74E-60	Arabidopsis thaliana protein kinase-related protein mRNA, complete cds
AT2G42060	-3.12437674	4.25E-60	Arabidopsis thaliana chromosome 2, complete sequence
AT5G60520	-4.954451739	5.40E-60	Arabidopsis thaliana late embryogenesis abundant protein-like protein mRNA, complete cds
AT2G24762	-2.57836052	9.86E-60	Arabidopsis thaliana chromosome 2, complete sequence
AT3G02640	-1.565276303	1.13E-59	Arabidopsis thaliana chromosome 3, complete sequence

AT2G35880	-1.257984566	2.26E-59	Arabidopsis thaliana TPX2 (targeting protein for Xklp2)-like protein mRNA, complete cds
AT4G25250	-4.057739547	5.13E-59	Arabidopsis thaliana chromosome 4 sequence
AT1G23020	-1.391467607	6.98E-59	Arabidopsis thaliana ferric reduction oxidase 3 mRNA, complete cds
AT5G61340	-1.633314378	8.12E-59	Arabidopsis thaliana chromosome 5 sequence
AT1G62640	-1.34975302	9.15E-59	Arabidopsis thaliana 3-ketoacyl-acyl carrier protein synthase III mRNA, complete cds
AT5G01240	-1.42693951	1.16E-58	Arabidopsis thaliana auxin transporter-like protein 1 mRNA, complete cds
AT1G22330	-3.178315547	2.05E-58	Arabidopsis thaliana RNA recognition motif-containing protein mRNA, complete cds
AT5G37450	-3.409191662	2.98E-58	Arabidopsis thaliana probable LRR receptor-like serine/threonine-protein kinase mRNA, complete cds
AT5G06390	-1.129795587	3.53E-58	Arabidopsis thaliana fasciclin-like arabinogalactan protein 17 precursor mRNA, complete cds
AT5G22580	-1.720417204	3.91E-58	Arabidopsis thaliana stress responsive A/B Barrel domain-containing protein mRNA, complete cds
AT1G12000	-1.069581025	4.44E-58	Arabidopsis thaliana pyrophosphate--fructose-6-phosphate 1-phosphotransferase subunit beta 1 mRNA, complete cds
AT5G54630	-1.846514917	6.81E-58	Arabidopsis thaliana zinc finger-related protein mRNA, complete cds
AT2G21650	-1.929360758	7.29E-58	Arabidopsis thaliana MYB transcription factor RSM1 mRNA, complete cds
AT2G15292	-2.044649548	7.70E-58	Arabidopsis thaliana chromosome 2, complete sequence
AT5G44480	-2.186814449	9.77E-58	Arabidopsis thaliana putative UDP-arabinose 4-epimerase 4 mRNA, complete cds
AT5G57220	-2.410516506	2.03E-57	Arabidopsis thaliana cytochrome P450, family 81, subfamily F, polypeptide 2 mRNA, complete cds
AT2G44380	-2.285965882	4.46E-57	Arabidopsis thaliana chromosome 2,

			complete sequence
AT3G62680	-4.885189076	4.89E-57	Arabidopsis thaliana proline-rich protein 3 mRNA, complete cds
AT3G17185	-1.959707464	5.00E-57	Arabidopsis thaliana chromosome 3, complete sequence
AT5G44130	-1.396181356	5.23E-57	Arabidopsis thaliana chromosome 5 sequence
AT5G56320	-2.649237517	5.34E-57	Arabidopsis thaliana expansin A14 mRNA, complete cds
AT5G17630	-1.288621392	6.46E-57	Arabidopsis thaliana chromosome 5 sequence
AT3G28920	-1.324563367	1.26E-56	Arabidopsis thaliana chromosome 3, complete sequence
AT2G22170	-1.456370699	2.10E-56	Arabidopsis thaliana PLAT-plant-stress domain-containing protein mRNA, complete cds
AT2G25840	-1.204678368	5.62E-56	Arabidopsis thaliana protein ovule abortion 4 mRNA, complete cds
AT3G16250	-1.197829185	6.27E-56	Arabidopsis thaliana NDH-dependent cyclic electron flow 1 mRNA, complete cds
AT3G47350	-1.113688991	6.86E-56	Arabidopsis thaliana hydroxysteroid dehydrogenase 2 mRNA, complete cds
AT5G60710	-1.324448274	7.09E-56	Arabidopsis thaliana C3H4 type zinc finger protein mRNA, complete cds
AT5G55280	-1.333273319	1.45E-55	Arabidopsis thaliana cell division protein ftsZ-like protein 1 mRNA, complete cds
AT3G50530	-1.213473082	1.66E-55	Arabidopsis thaliana CDPK-related kinase mRNA, complete cds
AT1G33811	-1.81917288	2.12E-55	Arabidopsis thaliana GDSL esterase/lipase mRNA, complete cds
AT3G46490	-3.315639264	2.44E-55	Arabidopsis thaliana oxidoreductase, 2OG-Fe(II) oxygenase family protein mRNA, complete cds
AT5G15210	-1.959906169	5.01E-55	Arabidopsis thaliana chromosome 5 sequence
AT5G56860	-1.097265661	6.03E-55	Arabidopsis thaliana GATA transcription factor 21 mRNA, complete cds
AT4G28410	-2.821927653	9.07E-55	Arabidopsis thaliana tyrosine transaminase family protein mRNA, complete cds
AT4G26050	-2.725687235	1.11E-54	Arabidopsis thaliana plant intracellular ras group-related LRR 8 mRNA, complete cds

AT4G22212	-1.657622283	1.57E-54	Arabidopsis thaliana defensin-like protein 98 mRNA, complete cds
AT2G25810	-3.261880264	2.05E-54	Arabidopsis thaliana aquaporin TIP4-1 mRNA, complete cds
AT2G34510	-1.688424188	2.27E-54	Arabidopsis thaliana uncharacterized protein mRNA, complete cds
AT1G28580	-1.040312475	2.44E-54	Arabidopsis thaliana GDSL esterase/lipase mRNA, complete cds
AT2G03750	-1.332963362	2.47E-54	Arabidopsis thaliana chromosome 2, complete sequence
AT1G29020	-4.992586867	3.40E-54	Arabidopsis thaliana EF-hand, calcium binding motif-containing protein mRNA, complete cds
AT1G79530	-1.070116088	3.59E-54	Arabidopsis thaliana glyceraldehyde-3-phosphate dehydrogenase GAPCP1 mRNA, complete cds
AT1G18250	-1.505333244	5.04E-54	Arabidopsis thaliana Thaumatin-like protein mRNA, complete cds
AT1G48210	-1.361268731	5.75E-54	Arabidopsis thaliana serine/threonine protein kinase mRNA, complete cds
AT5G32450	-1.19866227	6.19E-54	Arabidopsis thaliana RNA recognition motif (RRM)-containing protein mRNA, complete cds
AT3G51720	-1.43737171	6.46E-54	Arabidopsis thaliana uncharacterized protein mRNA, complete cds
AT2G25930	-1.111808067	7.79E-54	Arabidopsis thaliana protein EARLY FLOWERING 3 mRNA, complete cds
AT3G51340	-2.783117167	1.03E-53	Arabidopsis thaliana aspartyl protease family protein mRNA, complete cds
AT3G14680	-2.371860142	1.43E-53	Arabidopsis thaliana cytochrome P450, family 72, subfamily A, polypeptide 14 mRNA, complete cds
AT3G62270	-2.547149888	1.48E-53	Arabidopsis thaliana putative boron transporter 2 mRNA, complete cds
AT5G66770	-2.118198165	1.51E-53	Arabidopsis thaliana chromosome 5 sequence
AT1G07090	-2.531760667	1.75E-53	Arabidopsis thaliana chromosome 1 sequence
AT3G47800	-1.258785773	1.84E-53	Arabidopsis thaliana putative aldose 1-epimerase mRNA, complete cds
AT2G37460	-1.79994179	2.79E-53	Arabidopsis thaliana nodulin MtN21-like transporter family protein mRNA, complete cds

AT3G17210	-1.06521087	2.96E-53	Arabidopsis thaliana mRNA for hypothetical protein, complete cds, clone: RAFL26-03-H08
AT4G28780	-1.412109915	4.78E-53	Arabidopsis thaliana GDSL esterase/lipase mRNA, complete cds
AT3G28910	-1.989498686	5.16E-53	Arabidopsis thaliana myb domain protein 30 mRNA, complete cds
AT1G47740	-1.084689623	5.82E-53	Arabidopsis thaliana PPPDE putative thiol peptidase family protein mRNA, complete cds
AT1G72300	-1.080515433	9.12E-53	Arabidopsis thaliana chromosome 1 sequence
AT1G64400	-1.053345001	9.27E-53	Arabidopsis thaliana long-chain acyl-CoA synthetase mRNA, complete cds
AT3G06300	-1.651524606	9.56E-53	Arabidopsis thaliana prolyl-4 hydroxylase 2 mRNA, complete cds
AT5G11480	-1.011329939	9.84E-53	Arabidopsis thaliana P-loop containing nucleoside triphosphate hydrolases superfamily protein mRNA, complete cds
AT3G22800	-3.413883357	2.00E-52	Arabidopsis thaliana chromosome 3, complete sequence
AT1G49200	-2.005499344	2.05E-52	Arabidopsis thaliana chromosome 1 sequence
AT2G01520	-1.18697484	2.90E-52	Arabidopsis thaliana MLP-like protein 328 mRNA, complete cds
AT1G60960	-1.960211008	3.17E-52	Arabidopsis thaliana Fe(2+) transport protein 3 mRNA, complete cds
AT5G39320	-1.130443188	3.22E-52	Arabidopsis thaliana putative UDP-glucose 6-dehydrogenase 2 mRNA, complete cds
AT5G64920	-1.009634654	3.79E-52	Arabidopsis thaliana chromosome 5 sequence
AT4G34290	-1.394518032	3.81E-52	Arabidopsis thaliana SWIB/MDM2 domain-containing protein mRNA, complete cds
AT3G55420	-1.745865117	5.22E-52	Arabidopsis thaliana chromosome 3, complete sequence
AT3G55630	-1.042476486	6.11E-52	Arabidopsis thaliana folylpolyglutamate synthase 3 mRNA, complete cds
AT4G26320	-6.12437674	8.07E-52	Arabidopsis thaliana chromosome 4 sequence
AT1G20950	-1.082162907	9.01E-52	Arabidopsis thaliana

			pyrophosphate--fructose 6-phosphate 1-phosphotransferase subunit alpha 1 mRNA, complete cds
AT2G43360	-1.476554215	9.67E-52	Arabidopsis thaliana biotin synthase mRNA, complete cds
AT3G18680	-1.011639212	1.56E-51	Arabidopsis thaliana uridylate kinase-like protein mRNA, complete cds
AT5G52540	-1.036451676	1.67E-51	Arabidopsis thaliana uncharacterized protein mRNA, complete cds
AT1G51470	-1.671999879	1.82E-51	Arabidopsis thaliana myrosinase 5 mRNA, complete cds
AT3G07460	-1.311185193	2.20E-51	Arabidopsis thaliana uncharacterized protein mRNA, complete cds
AT1G02640	-1.346342392	2.46E-51	Arabidopsis thaliana probable beta-D-xylosidase 2 mRNA, complete cds
AT5G60570	-1.067565819	2.50E-51	Arabidopsis thaliana F-box/kelch-repeat protein mRNA, complete cds
AT5G42180	-2.368253525	2.59E-51	Arabidopsis thaliana peroxidase mRNA, complete cds
AT5G24210	-1.210550097	2.81E-51	Arabidopsis thaliana lipase class 3 family protein mRNA, complete cds
AT3G02620	-3.346769161	3.09E-51	Arabidopsis thaliana acyl-[acyl-carrier-protein] desaturase mRNA, complete cds
AT1G13100	-2.611883878	3.58E-51	Arabidopsis thaliana cytochrome P450 71B29 mRNA, complete cds
AT2G47930	-2.095807588	4.24E-51	Arabidopsis thaliana chromosome 2, complete sequence
AT5G22920	-1.266991329	7.70E-51	Arabidopsis thaliana ring finger and CHY zinc finger domain-containing protein 1 mRNA, complete cds
AT2G40670	-2.017461536	1.29E-50	Arabidopsis thaliana two-component response regulator ARR16 mRNA, complete cds
AT5G65390	-2.617416751	1.42E-50	Arabidopsis thaliana chromosome 5 sequence
AT2G23620	-3.511828408	1.43E-50	Arabidopsis thaliana methyl esterase 1 mRNA, complete cds
AT5G05500	-4.433423544	2.00E-50	Arabidopsis thaliana chromosome 5 sequence
AT5G45550	-1.14651746	2.06E-50	Arabidopsis thaliana maintenance of ploidy protein MOB1 mRNA, complete cds

AT4G37160	-3.413883357	2.12E-50	Arabidopsis thaliana protein SKU5 similar 15 mRNA, complete cds
AT2G33330	-1.649597157	2.77E-50	Arabidopsis thaliana plasmodesmata-located protein 3 mRNA, complete cds
AT4G28270	-1.508651986	3.15E-50	Arabidopsis thaliana mRNA for hypothetical protein, complete cds, clone: RAFL14-01-C23
AT5G19970	-2.531240308	3.59E-50	Arabidopsis thaliana chromosome 5 sequence
AT5G61380	-1.024048595	4.10E-50	Arabidopsis thaliana two-component response regulator-like APRR1 mRNA, complete cds
AT3G55230	-2.064583655	4.70E-50	Arabidopsis thaliana chromosome 3, complete sequence
AT1G78260	-2.302375042	4.78E-50	Arabidopsis thaliana RNA recognition motif-containing protein mRNA, complete cds
AT2G25310	-1.394493244	8.57E-50	Arabidopsis thaliana uncharacterized protein mRNA, complete cds
AT3G04570	-1.811751092	1.37E-49	Arabidopsis thaliana chromosome 3, complete sequence
AT5G39210	-2.435909664	1.51E-49	Arabidopsis thaliana protein CHLORORESPIRATORY REDUCTION 7 mRNA, complete cds
AT5G11000	-1.378080386	1.52E-49	Arabidopsis thaliana chromosome 5 sequence
AT2G26820	-3.349514538	1.86E-49	Arabidopsis thaliana phloem protein 2-LIKE A3 mRNA, complete cds
AT1G05650	-3.773879493	1.86E-49	Arabidopsis thaliana pectin lyase-like protein mRNA, complete cds
AT1G31350	-1.340389925	2.00E-49	Arabidopsis thaliana chromosome 1 sequence
AT1G74770	-1.860629716	3.35E-49	Arabidopsis thaliana zinc ion binding protein mRNA, complete cds
AT4G38080	-1.450306765	3.57E-49	Arabidopsis thaliana chromosome 4 sequence
AT1G11350	-1.658545393	3.84E-49	Arabidopsis thaliana G-type lectin S-receptor-like serine/threonine-protein kinase SD1-13 mRNA, complete cds
AT1G09660	-1.513991642	5.19E-49	Arabidopsis thaliana RNA-binding KH domain-containing protein mRNA, complete cds
AT1G29280	-1.474925513	6.71E-49	Arabidopsis thaliana putative WRKY

			transcription factor 65 mRNA, complete cds
AT3G56650	-1.063750381	8.06E-49	Arabidopsis thaliana Mog1/PsbP/DUF1795-like photosystem II reaction center PsbP family protein mRNA, complete cds
AT2G25060	-1.50770538	1.01E-48	Arabidopsis thaliana early nodulin-like protein 14 mRNA, complete cds
AT1G01430	-1.200682518	1.12E-48	Arabidopsis thaliana protein trichome birefringence-like 25 mRNA, complete cds
AT1G13609	-5.297859561	1.21E-48	Arabidopsis thaliana defensin-like protein 287 mRNA, complete cds
AT3G04290	-1.329768809	1.68E-48	Arabidopsis thaliana Li-tolerant lipase 1 mRNA, complete cds
AT1G33840	-4.833597344	2.81E-48	Arabidopsis thaliana uncharacterized protein mRNA, complete cds
AT5G08520	-1.139897325	3.35E-48	Arabidopsis thaliana duplicated SANT DNA-binding domain-containing protein mRNA, complete cds
AT4G02540	-1.56715923	3.76E-48	Arabidopsis thaliana chromosome 4 sequence
AT1G65190	-1.201612065	5.97E-48	Arabidopsis thaliana chromosome 1 sequence
AT5G06690	-2.366834784	7.65E-48	Arabidopsis thaliana WCRKC thioredoxin 1 mRNA, complete cds
AT2G21020	-3.87410963	1.11E-47	Arabidopsis thaliana chromosome 2, complete sequence
AT2G24720	-5.020540929	1.18E-47	Arabidopsis thaliana glutamate receptor 2.2 mRNA, complete cds
AT4G14870	-1.141208672	1.31E-47	Arabidopsis thaliana chromosome 4 sequence
AT1G49470	-1.616608745	1.37E-47	Arabidopsis thaliana chromosome 1 sequence
AT3G22235	-2.49735018	1.52E-47	Arabidopsis thaliana uncharacterized protein mRNA, complete cds
AT5G11070	-1.29840614	1.57E-47	Arabidopsis thaliana chromosome 5 sequence
AT3G19850	-1.96408044	3.22E-47	Arabidopsis thaliana phototropic-responsive NPH3 family protein mRNA, complete cds
AT1G09200	-1.022196345	3.92E-47	Arabidopsis thaliana chromosome 1 sequence
AT3G07430	-1.161017384	9.48E-47	Arabidopsis thaliana chromosome 3,

			complete sequence
AT3G01260	-1.618241856	9.89E-47	Arabidopsis thaliana aldose 1-epimerase-like prtotein mRNA, complete cds
AT3G27950	-3.642507732	1.03E-46	Arabidopsis thaliana GDSL esterase/lipase mRNA, complete cds
AT1G80050	-2.173159113	1.23E-46	Arabidopsis thaliana adenine phosphoribosyl transferase 2 mRNA, complete cds
AT2G27660	-2.023493383	1.29E-46	Arabidopsis thaliana chromosome 2, complete sequence
AT3G63200	-1.43161095	1.38E-46	Arabidopsis thaliana PATATIN-like protein 9 mRNA, complete cds
AT2G42610	-1.191490936	1.47E-46	Arabidopsis thaliana uncharacterized protein mRNA, complete cds
AT3G54366	-1.234088112	1.49E-46	Arabidopsis thaliana chromosome 3, complete sequence
AT1G33800	-1.683526547	2.09E-46	Arabidopsis thaliana glucuronoxylan 4-O-methyltransferase mRNA, complete cds
AT3G46330	-2.605216298	2.38E-46	Arabidopsis thaliana putative LRR receptor-like serine/threonine-protein kinase MEE39 mRNA, complete cds
AT5G15180	-2.0962257	2.74E-46	Arabidopsis thaliana peroxidase 56 mRNA, complete cds
AT5G10030	-1.59161045	2.87E-46	Arabidopsis thaliana transcription factor TGA4 mRNA, complete cds
AT4G15380	-3.693537385	3.40E-46	Arabidopsis thaliana cytochrome P450, family 705, subfamily A, polypeptide 4 mRNA, complete cds
AT5G35220	-1.209448825	3.41E-46	Arabidopsis thaliana metalloprotease EGY1 mRNA, complete cds
AT3G23800	-2.284372624	6.22E-46	Arabidopsis thaliana selenium-binding protein 3 mRNA, complete cds
AT1G05675	-2.691417333	6.87E-46	Arabidopsis thaliana UDP-Glycosyltransferase superfamily protein mRNA, complete cds
AT2G30010	-1.721088199	7.02E-46	Arabidopsis thaliana protein trichome birefringence-like 45 mRNA, complete cds
AT3G13510	-1.129115856	7.49E-46	Arabidopsis thaliana uncharacterized protein mRNA, complete cds
AT1G12310	-1.059247929	7.56E-46	Arabidopsis thaliana chromosome 1 sequence

AT1G47840	-1.964435827	1.29E-45	Arabidopsis thaliana hexokinase 3 mRNA, complete cds
AT2G22230	-1.228587735	1.41E-45	Arabidopsis thaliana putative 3-hydroxyacyl-ACP dehydratase mRNA, complete cds
AT2G19310	-1.308295014	2.23E-45	Arabidopsis thaliana chromosome 2, complete sequence
AT5G45080	-2.813707763	2.27E-45	Arabidopsis thaliana protein PHLOEM PROTEIN 2-LIKE A6 mRNA, complete cds
AT5G60530	-1.58473823	2.57E-45	Arabidopsis thaliana late embryogenesis abundant protein-like protein mRNA, complete cds
AT1G62800	-1.527694475	3.03E-45	Arabidopsis thaliana aspartate aminotransferase 4 mRNA, complete cds
AT2G46440	-1.527637072	4.97E-45	Arabidopsis thaliana cyclic nucleotide-gated channel 11 mRNA, complete cds
AT3G09580	-1.627852204	6.15E-45	Arabidopsis thaliana chromosome 3, complete sequence
AT1G64640	-1.798255632	8.33E-45	Arabidopsis thaliana early nodulin-like protein 8 mRNA, complete cds
AT2G24980	-4.406578557	1.25E-44	Arabidopsis thaliana chromosome 2, complete sequence
AT1G51860	-2.369489238	1.37E-44	Arabidopsis thaliana putative LRR receptor-like serine/threonine-protein kinase mRNA, complete cds
AT3G05730	-1.091387384	1.50E-44	Arabidopsis thaliana defensin-like protein 205 mRNA, complete cds
AT3G46830	-1.186625181	1.55E-44	Arabidopsis thaliana Ras-related protein RABA2c mRNA, complete cds
AT3G02630	-1.230629945	1.61E-44	Arabidopsis thaliana acyl-[acyl-carrier-protein] desaturase mRNA, complete cds
AT1G16920	-1.203213421	2.35E-44	Arabidopsis thaliana Ras-related protein RABA1b mRNA, complete cds
AT3G52580	-1.003833573	2.51E-44	Arabidopsis thaliana 40S ribosomal protein S14-3 mRNA, complete cds
AT3G19930	-1.005256109	2.57E-44	Arabidopsis thaliana sugar transport protein 4 mRNA, complete cds
AT5G54610	-1.688640248	2.65E-44	Arabidopsis thaliana ankyrin-repeat transmembrane protein BDA1 mRNA, complete cds
AT5G60670	-1.235058774	3.81E-44	Arabidopsis thaliana chromosome 5

			sequence
AT5G63410	-1.358722807	4.01E-44	Arabidopsis thaliana Leucine-rich repeat protein kinase family protein mRNA, complete cds
AT5G10130	-2.443489819	4.86E-44	Arabidopsis thaliana pollen_Ole_e_I-domain containing protein mRNA, complete cds
AT4G25220	-5.462246379	5.72E-44	Arabidopsis thaliana putative glycerol-3-phosphate transporter 2 mRNA, complete cds
AT1G21100	-1.093980504	5.92E-44	Arabidopsis thaliana indole glucosinolate o-methyltransferase 1 mRNA, complete cds
AT3G50740	-1.248932828	6.66E-44	Arabidopsis thaliana chromosome 3, complete sequence
AT2G19060	-3.854455949	6.76E-44	Arabidopsis thaliana SGNH hydrolase-type esterase family protein mRNA, complete cds
AT3G43960	-2.804556308	7.67E-44	Arabidopsis thaliana putative cysteine proteinase mRNA, complete cds
AT5G45670	-1.143297602	1.44E-43	Arabidopsis thaliana GDSL esterase/lipase mRNA, complete cds
AT5G65810	-1.503965403	1.47E-43	Arabidopsis thaliana protein COTTON GOLGI-RELATED 3 mRNA, complete cds
AT3G09470	-1.118314987	2.23E-43	Arabidopsis thaliana major facilitator superfamily protein mRNA, complete cds
AT3G49960	-5.132369531	2.62E-43	Arabidopsis thaliana peroxidase 35 mRNA, complete cds
AT3G44750	-1.343977912	2.65E-43	Arabidopsis thaliana histone deacetylase HDT1 mRNA, complete cds
AT1G78090	-2.08515543	2.90E-43	Arabidopsis thaliana trehalose-6-phosphate phosphatase mRNA, complete cds
AT1G19740	-1.008206704	3.15E-43	Arabidopsis thaliana chromosome 1 sequence
AT2G47910	-1.229245858	3.60E-43	Arabidopsis thaliana chromosome 2, complete sequence
AT1G12560	-3.239853957	5.28E-43	Arabidopsis thaliana alpha-expansin family protein mRNA, complete cds
AT4G00880	-1.063108802	6.37E-43	Arabidopsis thaliana chromosome 4 sequence
AT2G33550	-2.018248389	8.26E-43	Arabidopsis thaliana Myb/SANT-like

			DNA-binding domain-containing protein mRNA, complete cds
AT1G59990	-1.510267894	9.00E-43	Arabidopsis thaliana DEAD-box ATP-dependent RNA helicase 22 mRNA, complete cds
AT5G46580	-1.037315395	9.80E-43	Arabidopsis thaliana chromosome 5 sequence
AT1G67050	-1.830004368	1.85E-42	Arabidopsis thaliana chromosome 1 sequence
AT5G08330	-1.667804776	1.97E-42	Arabidopsis thaliana chromosome 5 sequence
AT4G32650	-1.749631947	2.00E-42	Arabidopsis thaliana potassium channel KAT3 mRNA, complete cds
AT5G23210	-1.379633795	2.55E-42	Arabidopsis thaliana serine carboxypeptidase-like 34 mRNA, complete cds
AT5G50250	-1.003677091	2.93E-42	Arabidopsis thaliana chloroplast RNA-binding protein 31B mRNA, complete cds
AT2G41940	-1.514667577	3.01E-42	Arabidopsis thaliana chromosome 2, complete sequence
AT3G13760	-2.719986485	3.20E-42	Arabidopsis thaliana chromosome 3, complete sequence
AT3G13560	-1.531801055	3.85E-42	Arabidopsis thaliana glucan endo-1,3-beta-glucosidase 4 mRNA, complete cds
AT2G15050	-1.103048231	4.90E-42	Arabidopsis thaliana non-specific lipid-transfer protein 7 mRNA, complete cds
AT3G25190	-1.723014178	5.51E-42	Arabidopsis thaliana vacuolar iron transporter homolog 2.1 mRNA, complete cds
AT1G63220	-1.81487729	6.57E-42	Arabidopsis thaliana calcium-dependent lipid-binding domain-containing protein mRNA, complete cds
AT5G62340	-2.893051194	7.39E-42	Arabidopsis thaliana chromosome 5 sequence
AT1G67750	-1.366612477	7.40E-42	Arabidopsis thaliana putative pectate lyase 5 mRNA, complete cds
AT4G04830	-1.083265205	1.05E-41	Arabidopsis thaliana peptide methionine sulfoxide reductase B5 mRNA, complete cds
AT5G65683	-1.226704674	1.30E-41	Arabidopsis thaliana C3H4 type zinc finger protein mRNA, complete cds

AT1G13420	-2.468331141	1.85E-41	Arabidopsis thaliana chromosome 1 sequence
AT4G25090	-3.633931838	2.06E-41	Arabidopsis thaliana riboflavin synthase-like superfamily protein mRNA, complete cds
AT3G47220	-1.575105946	3.19E-41	Arabidopsis thaliana phosphoinositide phospholipase C9 mRNA, complete cds
AT2G27510	-1.276379833	3.38E-41	Arabidopsis thaliana ferredoxin 3 mRNA, complete cds
AT4G00330	-1.014485448	3.46E-41	Arabidopsis thaliana calmodulin-binding receptor-like cytoplasmic kinase 2 mRNA, complete cds
AT4G01330	-1.042988413	3.47E-41	Arabidopsis thaliana protein kinase family protein mRNA, complete cds
AT5G56530	-1.136781761	3.68E-41	Arabidopsis thaliana uncharacterized protein mRNA, complete cds
AT3G45310	-1.053987412	3.75E-41	Arabidopsis thaliana cysteine proteinases superfamily protein mRNA, complete cds
AT1G56010	-1.454939671	3.75E-41	Arabidopsis thaliana transcription factor NAC1 mRNA, complete cds
AT3G16440	-1.992721756	5.30E-41	Arabidopsis thaliana myrosinase-binding protein-like protein-300B mRNA, complete cds
AT1G02900	-1.936304392	7.89E-41	Arabidopsis thaliana chromosome 1 sequence
AT1G13270	-1.301434502	1.44E-40	Arabidopsis thaliana methionine aminopeptidase 1B mRNA, complete cds
AT3G11550	-2.534289207	1.85E-40	Arabidopsis thaliana uncharacterized protein mRNA, complete cds
AT2G17230	-1.958203873	2.06E-40	Arabidopsis thaliana chromosome 2, complete sequence
AT1G65590	-1.009647393	2.77E-40	Arabidopsis thaliana beta-hexosaminidase 3 mRNA, complete cds
AT3G53750	-1.145877809	2.96E-40	Arabidopsis thaliana actin 3 mRNA, complete cds
AT1G30840	-2.349136176	3.05E-40	Arabidopsis thaliana chromosome 1 sequence
AT3G06770	-1.856086609	3.63E-40	Arabidopsis thaliana polygalacturonase-like protein mRNA, complete cds
AT1G19900	-4.268342514	4.24E-40	Arabidopsis thaliana chromosome 1 sequence

AT4G19660	-1.463658402	4.85E-40	Arabidopsis thaliana NPR1-like protein 4 mRNA, complete cds
AT5G37310	-1.078001091	5.53E-40	Arabidopsis thaliana putative endomembrane protein 70 mRNA, complete cds
AT5G60760	-1.55885412	5.67E-40	Arabidopsis thaliana phytic acid metabolising protein mRNA, complete cds
AT3G25070	-1.032131102	6.72E-40	Arabidopsis thaliana RPM1 interacting protein 4 mRNA, complete cds
AT1G74070	-1.03391373	6.83E-40	Arabidopsis thaliana cyclophilin-like peptidyl-prolyl cis-trans isomerase family protein mRNA, complete cds
AT5G22940	-2.036913899	8.19E-40	Arabidopsis thaliana probable glucuronoxylan glucuronosyltransferase F8H mRNA, complete cds
AT3G59540	-1.131181918	1.09E-39	Arabidopsis thaliana 60S ribosomal protein L38 mRNA, complete cds
AT3G18130	-1.217486144	1.10E-39	Arabidopsis thaliana receptor for activated C kinase 1C mRNA, complete cds
AT1G04520	-1.536694738	1.11E-39	Arabidopsis thaliana plasmodesmata-located protein 2 mRNA, complete cds
AT4G10450	-1.148092809	1.35E-39	Arabidopsis thaliana 60S ribosomal protein L9-2 mRNA, complete cds
AT3G16410	-1.851603331	1.42E-39	Arabidopsis thaliana nitrile-specifier protein 4 mRNA, complete cds
AT3G59980	-1.296617305	1.63E-39	Arabidopsis thaliana Nucleic acid-binding, OB-fold-like protein mRNA, complete cds
AT2G38550	-1.12268839	2.09E-39	Arabidopsis thaliana transmembrane protein 14C mRNA, complete cds
AT4G01750	-1.664945121	2.33E-39	Arabidopsis thaliana rhamnogalacturonan xylosyltransferase 2 mRNA, complete cds
AT2G28310	-1.152910576	2.41E-39	Arabidopsis thaliana uncharacterized protein mRNA, complete cds
AT4G38840	-1.559196852	2.51E-39	Arabidopsis thaliana chromosome 4 sequence
AT5G45070	-2.418918947	3.09E-39	Arabidopsis thaliana protein PHLOEM PROTEIN 2-LIKE A8 mRNA, complete cds
AT1G44050	-4.380716493	4.40E-39	Arabidopsis thaliana

			cysteine/histidine-rich C1-like domain-containing protein mRNA, complete cds
AT5G16590	-1.221787447	4.87E-39	Arabidopsis thaliana leucine-rich repeat protein 1 mRNA, complete cds
AT3G15030	-1.246426611	5.59E-39	Arabidopsis thaliana transcription factor TCP4 mRNA, complete cds
AT5G66052	-1.219046658	5.87E-39	Arabidopsis thaliana uncharacterized protein mRNA, complete cds
AT3G16180	-1.487325728	7.51E-39	Arabidopsis thaliana nitrate transporter 1.12 mRNA, complete cds
AT1G73280	-3.986873216	7.66E-39	Arabidopsis thaliana serine carboxypeptidase-like 3 mRNA, complete cds
AT4G15290	-3.216119309	1.04E-38	Arabidopsis thaliana cellulose synthase-like protein B5 mRNA, complete cds
AT3G16390	-3.489040717	1.08E-38	Arabidopsis thaliana Nitrile-specifier protein 3 mRNA, complete cds
AT2G34190	-1.704473486	1.23E-38	Arabidopsis thaliana nucleobase-ascorbate transporter 2 mRNA, complete cds
AT1G58100	-1.035313571	1.27E-38	Arabidopsis thaliana chromosome 1 sequence
AT2G03350	-1.550819003	1.35E-38	Arabidopsis thaliana uncharacterized protein mRNA, complete cds
AT3G12110	-1.66840068	1.68E-38	Arabidopsis thaliana actin-11 mRNA, complete cds
AT5G53880	-1.848583103	2.25E-38	Arabidopsis thaliana chromosome 5 sequence
AT1G73300	-3.035371734	2.71E-38	Arabidopsis thaliana serine carboxypeptidase-like 2 mRNA, complete cds
AT5G16570	-1.901984319	2.90E-38	Arabidopsis thaliana glutamine synthetase 1;4 mRNA, complete cds
AT5G07080	-1.224305514	3.00E-38	Arabidopsis thaliana HXXXD-type acyl-transferase family protein mRNA, complete cds
AT1G27190	-1.072633165	3.37E-38	Arabidopsis thaliana chromosome 1 sequence
AT1G61580	-1.234268997	4.21E-38	Arabidopsis thaliana 60S ribosomal protein L3-2 mRNA, complete cds
AT5G60490	-2.744031959	4.32E-38	Arabidopsis thaliana chromosome 5 sequence

AT2G39900	-1.650950741	4.92E-38	Arabidopsis thaliana protein WLIM2A mRNA, complete cds
AT2G14560	-2.424337715	5.04E-38	Arabidopsis thaliana protein LURP1 mRNA, complete cds
AT1G58370	-2.046151573	5.15E-38	Arabidopsis thaliana xylanase 1 mRNA, complete cds
AT5G61480	-1.065095802	6.05E-38	Arabidopsis thaliana leucine-rich repeat receptor-like protein kinase TDR mRNA, complete cds
AT5G48580	-1.228485143	6.17E-38	Arabidopsis thaliana peptidyl-prolyl cis-trans isomerase FKBP15-2 mRNA, complete cds
AT3G47980	-1.970753551	8.43E-38	Arabidopsis thaliana HPP integral membrane domain-containing protein mRNA, complete cds
AT3G17170	-1.247442287	8.96E-38	Arabidopsis thaliana protein REGULATOR OF FATTY-ACID COMPOSITION 3 mRNA, complete cds
AT2G39040	-3.234559658	1.16E-37	Arabidopsis thaliana peroxidase 24 mRNA, complete cds
AT3G15260	-1.14319377	1.17E-37	Arabidopsis thaliana putative protein phosphatase 2C 39 mRNA, complete cds
AT5G66280	-1.927045581	1.25E-37	Arabidopsis thaliana chromosome 5 sequence
AT1G73110	-1.100924701	1.36E-37	Arabidopsis thaliana P-loop containing nucleoside triphosphate hydrolases superfamily protein mRNA, complete cds
AT2G39890	-1.144219012	1.58E-37	Arabidopsis thaliana proline transporter 1 mRNA, complete cds
AT2G25260	-2.062704629	2.00E-37	Arabidopsis thaliana uncharacterized protein mRNA, complete cds
AT5G14650	-2.427204736	2.04E-37	Arabidopsis thaliana pectin lyase-like superfamily protein mRNA, complete cds
AT4G10550	-1.96262567	2.11E-37	Arabidopsis thaliana subtilisin-like protease mRNA, complete cds
AT3G47450	-1.331097003	2.19E-37	Arabidopsis thaliana NO-associated protein 1 mRNA, complete cds
AT2G32270	-2.539414239	2.28E-37	Arabidopsis thaliana zinc transporter 3 mRNA, complete cds
AT5G03570	-2.833145442	3.88E-37	Arabidopsis thaliana nickel transport protein FPN2 mRNA, complete cds
AT2G14247	-6.202379252	6.35E-37	Arabidopsis thaliana chromosome 2,

			complete sequence
AT1G27020	-1.222469552	6.73E-37	Arabidopsis thaliana uncharacterized protein mRNA, complete cds
AT4G37520	-1.107636885	7.22E-37	Arabidopsis thaliana peroxidase 50 mRNA, complete cds
AT1G48240	-1.410649339	7.69E-37	Arabidopsis thaliana novel plant snare 12 mRNA, complete cds
AT5G59870	-1.036538441	9.33E-37	Arabidopsis thaliana histone H2A 6 mRNA, complete cds
AT3G06150	-1.150612618	9.55E-37	Arabidopsis thaliana uncharacterized protein mRNA, complete cds
AT5G46790	-1.740693674	1.43E-36	Arabidopsis thaliana chromosome 5 sequence
AT2G03530	-1.399092951	1.81E-36	Arabidopsis thaliana ureide permease 2 mRNA, complete cds
AT2G21050	-1.20884353	1.98E-36	Arabidopsis thaliana like AUXIN RESISTANT 2 mRNA, complete cds
AT4G04955	-1.170943559	2.82E-36	Arabidopsis thaliana allantoinase mRNA, complete cds
AT5G57480	-1.691417333	2.91E-36	Arabidopsis thaliana chromosome 5 sequence
AT5G47950	-3.795753993	4.48E-36	Arabidopsis thaliana chromosome 5 sequence
AT1G03820	-1.345844653	4.67E-36	Arabidopsis thaliana chromosome 1 sequence
AT5G27390	-1.209978608	4.92E-36	Arabidopsis thaliana Mog1/PsbP/DUF1795-like photosystem II reaction center PsbP family protein mRNA, complete cds
AT4G12830	-1.269601301	6.95E-36	Arabidopsis thaliana hydrolase, alpha/beta fold family protein mRNA, complete cds
AT3G44430	-1.113834362	7.04E-36	Arabidopsis thaliana chromosome 3, complete sequence
AT5G02890	-1.998845858	8.60E-36	Arabidopsis thaliana HXXXD-type acyl-transferase-like protein mRNA, complete cds
AT2G18800	-4.626877081	9.19E-36	Arabidopsis thaliana probable xyloglucan endotransglucosylase/hydrolase protein 21 mRNA, complete cds
AT5G44568	-2.168770859	9.35E-36	Arabidopsis thaliana uncharacterized protein mRNA, complete cds
AT1G66140	-1.50171353	1.03E-35	Arabidopsis thaliana zinc finger protein 4 mRNA, complete cds

AT4G12390	-1.372063182	1.24E-35	Arabidopsis thaliana chromosome 4 sequence
AT4G14800	-1.063352394	1.26E-35	Arabidopsis thaliana 20S proteasome beta subunit D2 mRNA, complete cds
AT3G23470	-2.023055553	1.35E-35	Arabidopsis thaliana cyclopropane-fatty-acyl-phospholipid synthase mRNA, complete cds
AT2G39430	-2.059247929	2.04E-35	Arabidopsis thaliana chromosome 2, complete sequence
AT3G06390	-2.954451739	2.07E-35	Arabidopsis thaliana uncharacterized protein mRNA, complete cds
AT4G00400	-1.081281761	2.84E-35	Arabidopsis thaliana bifunctional sn-glycerol-3-phosphate 2-O-acyltransferase/phosphatase mRNA, complete cds
AT4G26370	-1.340510171	2.89E-35	Arabidopsis thaliana antitermination NusB domain-containing protein mRNA, complete cds
AT1G72416	-2.373164896	3.27E-35	Arabidopsis thaliana chaperone DnaJ-domain containing protein mRNA, complete cds
AT1G69900	-2.748867605	3.80E-35	Arabidopsis thaliana Actin cross-linking protein mRNA, complete cds
AT1G47210	-1.498275545	4.07E-35	Arabidopsis thaliana cyclin-dependent protein kinase 3;2 mRNA, complete cds
AT1G51340	-1.39848911	4.47E-35	Arabidopsis thaliana MATE efflux family protein mRNA, complete cds
AT3G56980	-4.832195989	6.05E-35	Arabidopsis thaliana transcription factor ORG3 mRNA, complete cds
AT1G49230	-2.349514538	6.06E-35	Arabidopsis thaliana chromosome 1 sequence
AT2G13820	-1.685492499	7.30E-35	Arabidopsis thaliana Non-specific lipid-transfer protein-like protein mRNA, complete cds
AT1G14900	-1.118179792	8.02E-35	Arabidopsis thaliana high mobility group protein A protein mRNA, complete cds
AT5G40830	-1.572204174	1.13E-34	Arabidopsis thaliana S-adenosyl-L-methionine-dependent methyltransferases superfamily protein mRNA, complete cds
AT1G28670	-1.414577127	1.21E-34	Arabidopsis thaliana lipase ARAB-1 mRNA, complete cds
AT5G14920	-1.373924209	1.30E-34	Arabidopsis thaliana gibberellin-regulated protein 14 mRNA,

			complete cds
AT1G75840	-1.001864681	1.37E-34	Arabidopsis thaliana Rac-like GTP-binding protein ARAC5 mRNA, complete cds
AT5G48830	-1.457436937	1.45E-34	Arabidopsis thaliana uncharacterized protein mRNA, complete cds
AT3G11370	-4.578942604	1.64E-34	Arabidopsis thaliana chromosome 3, complete sequence
AT5G54040	-4.578942604	1.64E-34	Arabidopsis thaliana chromosome 5 sequence
AT1G48930	-3.422057289	1.99E-34	Arabidopsis thaliana glycosyl hydrolase 9C1 mRNA, complete cds
AT5G44550	-1.936964312	3.28E-34	Arabidopsis thaliana uncharacterized protein mRNA, complete cds
AT1G32540	-1.594120131	3.29E-34	Arabidopsis thaliana protein LOL1 mRNA, complete cds
AT1G65370	-1.135424233	4.05E-34	Arabidopsis thaliana meprin and TRAF homology domain-containing protein mRNA, complete cds
AT3G07570	-1.390712509	4.08E-34	Arabidopsis thaliana Cytochrome b561/ferric reductase transmembrane with DOMON related domain mRNA, complete cds
AT2G18910	-1.54919526	6.70E-34	Arabidopsis thaliana hydroxyproline-rich glycoprotein-like protein mRNA, complete cds
AT1G06090	-1.798435583	6.96E-34	Arabidopsis thaliana delta-9 desaturase-like 1 protein mRNA, complete cds
AT2G45430	-1.428382927	7.61E-34	Arabidopsis thaliana chromosome 2, complete sequence
AT4G22810	-1.903226415	7.63E-34	Arabidopsis thaliana chromosome 4 sequence
AT5G18480	-1.110793768	8.09E-34	Arabidopsis thaliana plant glycogenin-like starch initiation protein 6 mRNA, complete cds
AT3G30875	-2.065095802	8.15E-34	Arabidopsis thaliana chromosome 3, complete sequence
AT1G16170	-1.991926444	8.70E-34	Arabidopsis thaliana uncharacterized protein mRNA, complete cds
AT1G66800	-2.984199082	8.92E-34	Arabidopsis thaliana alcohol dehydrogenase-like protein mRNA, complete cds
AT3G47860	-1.23854776	9.29E-34	Arabidopsis thaliana chloroplastic

			lipocalin mRNA, complete cds
AT5G24060	-1.055276108	1.20E-33	Arabidopsis thaliana pentatricopeptide repeat-containing protein-like protein mRNA, complete cds
AT2G29660	-1.418200652	1.26E-33	Arabidopsis thaliana chromosome 2, complete sequence
AT1G24170	-1.079642001	1.29E-33	Arabidopsis thaliana chromosome 1 sequence
AT4G17800	-1.539414239	1.33E-33	Arabidopsis thaliana chromosome 4 sequence
AT1G35620	-1.065042601	1.45E-33	Arabidopsis thaliana protein disulfide isomerase 5-2 mRNA, complete cds
AT5G45930	-1.065042601	1.45E-33	Arabidopsis thaliana magnesium chelatase subunit I2 mRNA, complete cds
AT3G49260	-1.212942219	2.27E-33	Arabidopsis thaliana protein IQ-domain 21 mRNA, complete cds
AT2G45550	-4.032454251	2.77E-33	Arabidopsis thaliana cytochrome P450 76C4 mRNA, complete cds
AT2G29720	-1.504875821	3.00E-33	Arabidopsis thaliana CTF2B like oxidoreductase mRNA, complete cds
AT1G49320	-1.714624724	3.05E-33	Arabidopsis thaliana BURP domain protein USPL1 mRNA, complete cds
AT3G07470	-1.149799337	3.21E-33	Arabidopsis thaliana uncharacterized protein mRNA, complete cds
AT5G23420	-1.588442575	3.25E-33	Arabidopsis thaliana high-mobility group B6 protein mRNA, complete cds
AT2G18328	-1.870059551	4.31E-33	Arabidopsis thaliana chromosome 2, complete sequence
AT3G46940	-1.992925886	5.52E-33	Arabidopsis thaliana chromosome 3, complete sequence
AT3G63110	-1.495598007	5.81E-33	Arabidopsis thaliana chromosome 3, complete sequence
AT1G29250	-1.385704824	7.82E-33	Arabidopsis thaliana Alba DNA/RNA-binding protein mRNA, complete cds
AT3G54560	-1.447830689	9.86E-33	Arabidopsis thaliana histone H2A 11 mRNA, complete cds
AT3G27080	-1.267685132	1.07E-32	Arabidopsis thaliana mitochondrial import receptor subunit TOM20-3 mRNA, complete cds
AT2G42570	-1.353001115	1.32E-32	Arabidopsis thaliana protein trichome birefringence-like 39 mRNA, complete cds

AT1G07370	-2.035751841	1.36E-32	Arabidopsis thaliana proliferating cellular nuclear antigen 1 mRNA, complete cds
AT3G49845	-2.903052586	1.92E-32	Arabidopsis thaliana uncharacterized protein mRNA, complete cds
AT2G42380	-1.778533747	2.47E-32	Arabidopsis thaliana transcription factor BZIP34 mRNA, complete cds
AT5G08610	-1.051313278	2.92E-32	Arabidopsis thaliana DEAD-box ATP-dependent RNA helicase 26 mRNA, complete cds
AT5G55990	-1.235602696	3.37E-32	Arabidopsis thaliana calcineurin B-like protein 2 mRNA, complete cds
AT3G58810	-1.964037787	3.46E-32	Arabidopsis thaliana chromosome 3, complete sequence
AT4G25870	-1.290800349	3.76E-32	Arabidopsis thaliana core-2/I-branching beta-1,6-N-acetylglucosaminyltransferase family protein mRNA, complete cds
AT4G39770	-2.25930632	4.33E-32	Arabidopsis thaliana probable trehalose-phosphate phosphatase H mRNA, complete cds
AT2G16750	-1.435578428	4.43E-32	Arabidopsis thaliana adenine nucleotide alpha hydrolases-domain containing protein kinase mRNA, complete cds
AT2G23130	-1.157851445	4.83E-32	Arabidopsis thaliana Lysine-rich arabinogalactan protein 17 mRNA, complete cds
AT1G27480	-1.022856244	5.14E-32	Arabidopsis thaliana Lecithin-cholesterol acyltransferase-like 1 mRNA, complete cds
AT3G49860	-3.108257075	5.68E-32	Arabidopsis thaliana ADP-ribosylation factor-like A1B mRNA, complete cds
AT3G12150	-1.358173925	5.92E-32	Arabidopsis thaliana uncharacterized protein mRNA, complete cds
AT4G12310	-1.422295597	6.50E-32	Arabidopsis thaliana cytochrome P450, family 706, subfamily A, polypeptide 5 mRNA, complete cds
AT4G12030	-1.152132406	6.78E-32	Arabidopsis thaliana probable sodium/metabolite cotransporter BASS5 mRNA, complete cds
AT5G05890	-2.073392811	8.18E-32	Arabidopsis thaliana UDP-glycosyltransferase 76C5 mRNA, complete cds
AT5G03170	-1.159691213	1.63E-31	Arabidopsis thaliana chromosome 5 sequence
AT1G28110	-1.441773939	1.71E-31	Arabidopsis thaliana serine

			carboxypeptidase-like 45 mRNA, complete cds
AT4G38390	-4.687806079	1.85E-31	Arabidopsis thaliana protein root hair specific 17 mRNA, complete cds
AT5G40730	-2.423448221	1.87E-31	Arabidopsis thaliana chromosome 5 sequence
AT1G05810	-2.016736017	2.21E-31	Arabidopsis thaliana RAB GTPase homolog A5E mRNA, complete cds
AT1G08320	-1.729109623	2.53E-31	Arabidopsis thaliana bZIP transcription factor family protein mRNA, complete cds
AT5G24230	-2.725827364	2.88E-31	Arabidopsis thaliana lipase class 3-related protein mRNA, complete cds
AT2G36430	-1.512256825	3.62E-31	Arabidopsis thaliana chromosome 2, complete sequence
AT1G14700	-1.216007216	4.88E-31	Arabidopsis thaliana purple acid phosphatase 3 mRNA, complete cds
AT1G50560	-1.821449607	5.55E-31	Arabidopsis thaliana cytochrome P450, family 705, subfamily A, polypeptide 25 mRNA, complete cds
AT2G23540	-1.781615142	6.68E-31	Arabidopsis thaliana GDSL esterase/lipase mRNA, complete cds
AT1G74940	-1.553913809	7.75E-31	Arabidopsis thaliana uncharacterized protein mRNA, complete cds
AT5G67510	-1.10479833	7.93E-31	Arabidopsis thaliana chromosome 5 sequence
AT5G56540	-1.911666872	8.07E-31	Arabidopsis thaliana chromosome 5 sequence
AT3G18200	-2.93073567	8.39E-31	Arabidopsis thaliana nodulin MtN21-like transporter UMAMIT4 mRNA, complete cds
AT1G65985	-1.949502523	8.65E-31	Arabidopsis thaliana uncharacterized protein mRNA, complete cds
AT4G37010	-1.95945242	8.75E-31	Arabidopsis thaliana centrin 2 mRNA, complete cds
AT4G34750	-2.023799785	8.80E-31	Arabidopsis thaliana chromosome 4 sequence
AT1G53680	-2.078996837	1.30E-30	Arabidopsis thaliana glutathione S-transferase TAU 28 mRNA, complete cds
AT1G23480	-1.455849523	1.53E-30	Arabidopsis thaliana putative mannan synthase 3 mRNA, complete cds
AT3G46540	-1.013657328	1.61E-30	Arabidopsis thaliana ENTH/VHS family protein mRNA, complete cds

AT3G47040	-3.058788398	1.62E-30	Arabidopsis thaliana Glycosyl hydrolase family protein mRNA, complete cds
AT4G33270	-1.080360636	1.71E-30	Arabidopsis thaliana cell division cycle 20.1, cofactor of APC complex mRNA, complete cds
AT3G29110	-2.099714686	1.98E-30	Arabidopsis thaliana putative terpenoid synthase 16 mRNA, complete cds
AT4G37040	-1.041423951	2.01E-30	Arabidopsis thaliana methionine aminopeptidase 1D mRNA, complete cds
AT5G49270	-5.323685548	2.09E-30	Arabidopsis thaliana COBRA-like protein 9 mRNA, complete cds
AT3G20810	-1.949433664	2.18E-30	Arabidopsis thaliana jumonji-C domain-containing protein 30 mRNA, complete cds
AT1G22550	-2.314347684	2.23E-30	Arabidopsis thaliana putative peptide/nitrate transporter mRNA, complete cds
AT1G05700	-3.159566169	2.35E-30	Arabidopsis thaliana Leucine-rich repeat transmembrane protein kinase protein mRNA, complete cds
AT3G07990	-1.725974555	2.57E-30	Arabidopsis thaliana serine carboxypeptidase-like 27 mRNA, complete cds
AT1G24280	-1.443836579	2.57E-30	Arabidopsis thaliana glucose-6-phosphate dehydrogenase 3 mRNA, complete cds
AT2G46890	-1.42443476	2.98E-30	Arabidopsis thaliana uncharacterized protein mRNA, complete cds
AT3G58120	-1.081895756	3.15E-30	Arabidopsis thaliana transcription factor BZIP61 mRNA, complete cds
AT1G65845	-1.093668677	3.17E-30	Arabidopsis thaliana uncharacterized protein mRNA, complete cds
AT1G29790	-1.091100585	3.69E-30	Arabidopsis thaliana putative methyltransferase domain-containing protein mRNA, complete cds
AT1G63560	-3.413883357	4.06E-30	Arabidopsis thaliana chromosome 1 sequence
AT3G06145	-2.270227607	4.40E-30	Arabidopsis thaliana chromosome 3, complete sequence
AT1G62980	-3.49241676	4.46E-30	Arabidopsis thaliana expansin A18 mRNA, complete cds
AT4G13170	-1.052831638	4.60E-30	Arabidopsis thaliana 60S ribosomal protein L13a-3 mRNA, complete cds
AT3G50010	-2.946231232	5.54E-30	Arabidopsis thaliana chromosome 3,

			complete sequence
AT1G50490	-1.182580529	6.50E-30	Arabidopsis thaliana ubiquitin-conjugating enzyme E2 20 mRNA, complete cds
AT4G25790	-4.908648049	7.04E-30	Arabidopsis thaliana allergen V5/Tpx-1-related family protein mRNA, complete cds
AT2G01070	-1.225305649	7.21E-30	Arabidopsis thaliana lung seven transmembrane receptor-like protein mRNA, complete cds
AT1G16390	-3.482830711	7.91E-30	Arabidopsis thaliana chromosome 1 sequence
AT1G78450	-2.417242369	8.51E-30	Arabidopsis thaliana SOUL/heme-binding protein-like protein mRNA, complete cds
AT5G20160	-1.119305751	8.92E-30	Arabidopsis thaliana ribosomal protein L7Ae/L30e/S12e/Gadd45 family protein mRNA, complete cds
AT4G12880	-1.015600394	1.04E-29	Arabidopsis thaliana early nodulin-like protein 19 mRNA, complete cds
AT1G72310	-1.190241308	1.14E-29	Arabidopsis thaliana chromosome 1 sequence
AT2G47420	-1.018051901	1.24E-29	Arabidopsis thaliana dimethyladenosine transferase mRNA, complete cds
AT5G23840	-1.670658773	1.35E-29	Arabidopsis thaliana MD-2-related lipid recognition domain-containing protein mRNA, complete cds
AT4G34160	-1.011420551	1.35E-29	Arabidopsis thaliana cyclin-D3-1 mRNA, complete cds
AT1G49030	-2.387411146	1.61E-29	Arabidopsis thaliana PLAC8 family protein mRNA, complete cds
AT5G36180	-4.369489238	1.75E-29	Arabidopsis thaliana serine carboxypeptidase-like 1 mRNA, complete cds
AT1G01640	-1.604265384	1.80E-29	Arabidopsis thaliana BTB/POZ domain-containing protein mRNA, complete cds
AT4G33260	-1.418855315	1.91E-29	Arabidopsis thaliana cell division cycle 20.2, cofactor of APC complex mRNA, complete cds
AT2G30840	-3.314347684	2.01E-29	Arabidopsis thaliana 2-oxoglutarate dependent oxygenase-like protein mRNA, complete cds
AT3G05020	-1.115855185	2.25E-29	Arabidopsis thaliana acyl carrier protein

			1 mRNA, complete cds
AT5G42320	-1.318726124	3.02E-29	Arabidopsis thaliana Zn-dependent exopeptidases superfamily protein mRNA, complete cds
AT3G54770	-2.093105278	3.23E-29	Arabidopsis thaliana putative RNA binding protein mRNA, complete cds
AT3G07195	-1.909285423	3.26E-29	Arabidopsis thaliana RPM1-interacting protein 4 (RIN4) family protein mRNA, complete cds
AT5G49640	-1.470537435	3.67E-29	Arabidopsis thaliana chromosome 5 sequence
AT2G16780	-1.337248901	3.80E-29	Arabidopsis thaliana WD-40 repeat-containing protein MSI2 mRNA, complete cds
AT1G34040	-2.873833278	3.90E-29	Arabidopsis thaliana Pyridoxal phosphate-dependent transferases superfamily protein mRNA, complete cds
AT5G45220	-3.375915507	4.00E-29	Arabidopsis thaliana TIR-NBS-LRR class disease resistance protein mRNA, complete cds
AT1G79670	-1.368428043	5.48E-29	Arabidopsis thaliana wall-associated receptor kinase-like 22 mRNA, complete cds
AT4G04330	-1.97614681	5.68E-29	Arabidopsis thaliana Chaperonin-like RbcX protein mRNA, complete cds
AT5G43370	-4.575038149	5.89E-29	Arabidopsis thaliana phosphate transporter Pht1;2 mRNA, complete cds
AT1G47480	-1.764120527	6.23E-29	Arabidopsis thaliana probable carboxylesterase 2 mRNA, complete cds
AT3G23510	-1.445438091	6.37E-29	Arabidopsis thaliana cyclopropane-fatty-acyl-phospholipid synthase mRNA, complete cds
AT4G24460	-1.221238279	6.51E-29	Arabidopsis thaliana CRT (chloroquine-resistance transporter)-like transporter 2 mRNA, complete cds
AT3G51740	-1.034739528	7.65E-29	Arabidopsis thaliana probably inactive leucine-rich repeat receptor-like protein kinase IMK2 mRNA, complete cds
AT1G70690	-1.567197396	9.14E-29	Arabidopsis thaliana plasmodesmata-located protein 5 mRNA, complete cds
AT2G26580	-1.644383799	9.82E-29	Arabidopsis thaliana axial regulator YABBY 5 mRNA, complete cds

AT5G62740	-1.17684416	1.08E-28	Arabidopsis thaliana Hypersensitive-induced response protein 1 mRNA, complete cds
AT2G32380	-1.687038892	1.13E-28	Arabidopsis thaliana putative transmembrane protein mRNA, complete cds
AT4G34560	-1.33859523	1.32E-28	Arabidopsis thaliana chromosome 4 sequence
AT3G19680	-1.482128499	1.56E-28	Arabidopsis thaliana uncharacterized protein mRNA, complete cds
AT1G77530	-1.102612766	1.68E-28	Arabidopsis thaliana O-methyltransferase family protein mRNA, complete cds
AT1G05385	-1.459340648	1.88E-28	Arabidopsis thaliana photosystem II D1 precursor processing protein PSB27-H2 mRNA, complete cds
AT5G06640	-6.837094788	2.31E-28	Arabidopsis thaliana chromosome 5 sequence
AT1G14410	-1.395504828	2.53E-28	Arabidopsis thaliana single-stranded DNA-binding protein WHY1 mRNA, complete cds
AT2G33210	-1.066628852	2.59E-28	Arabidopsis thaliana heat shock protein 60-2 mRNA, complete cds
AT1G31690	-1.498772255	2.73E-28	Arabidopsis thaliana copper amine oxidase family protein mRNA, complete cds
AT1G55160	-1.282316859	3.07E-28	Arabidopsis thaliana uncharacterized protein mRNA, complete cds
AT5G06630	-5.214979289	3.18E-28	Arabidopsis thaliana chromosome 5 sequence
AT3G11250	-1.04244822	3.72E-28	Arabidopsis thaliana 60S acidic ribosomal protein P0-3 mRNA, complete cds
AT5G42825	-1.077107498	4.87E-28	Arabidopsis thaliana chromosome 5 sequence
AT3G05920	-3.192856478	5.20E-28	Arabidopsis thaliana heavy-metal-associated domain-containing protein mRNA, complete cds
AT4G17030	-1.282378574	5.73E-28	Arabidopsis thaliana expansin-like B1 mRNA, complete cds
AT5G61250	-1.648348611	7.12E-28	Arabidopsis thaliana glucuronidase 1 mRNA, complete cds
AT2G41950	-1.863939446	7.27E-28	Arabidopsis thaliana uncharacterized protein mRNA, complete cds

AT3G50700	-1.37507892	7.47E-28	Arabidopsis thaliana indeterminate-domain 2 protein mRNA, complete cds
AT5G26310	-2.787341753	7.82E-28	Arabidopsis thaliana chromosome 5 sequence
AT5G02400	-1.988940115	9.14E-28	Arabidopsis thaliana putative protein phosphatase 2C 66 mRNA, complete cds
AT4G15740	-3.943136425	1.01E-27	Arabidopsis thaliana chromosome 4 sequence
AT2G15370	-4.101293127	1.01E-27	Arabidopsis thaliana probable fucosyltransferase 5 mRNA, complete cds
AT1G21880	-1.039209357	1.24E-27	Arabidopsis thaliana LysM domain-containing GPI-anchored protein 1 mRNA, complete cds
AT1G56550	-2.705223132	1.28E-27	Arabidopsis thaliana rhamnogalacturonan II specific xylosyltransferase 3 mRNA, complete cds
AT5G59260	-3.393563373	1.38E-27	Arabidopsis thaliana chromosome 5 sequence
AT5G51545	-1.031501819	1.38E-27	Arabidopsis thaliana low psii accumulation2 protein mRNA, complete cds
AT1G31885	-3.173620259	1.61E-27	Arabidopsis thaliana aquaporin NIP3-1 mRNA, complete cds
AT1G23030	-1.157122019	1.64E-27	Arabidopsis thaliana ARM repeat superfamily protein mRNA, complete cds
AT4G38620	-1.034375283	1.64E-27	Arabidopsis thaliana transcription repressor MYB4 mRNA, complete cds
AT1G02335	-1.111057598	1.92E-27	Arabidopsis thaliana germin-like protein subfamily 2 member 2 mRNA, complete cds
AT1G01180	-1.531443528	1.95E-27	Arabidopsis thaliana S-adenosyl-L-methionine-dependent methyltransferase domain-containing protein mRNA, complete cds
AT2G20780	-1.311608322	1.96E-27	Arabidopsis thaliana putative polyol transporter 4 mRNA, complete cds
AT5G12110	-1.14967076	1.98E-27	Arabidopsis thaliana elongation factor 1-beta 1 mRNA, complete cds
AT3G25790	-2.630912594	1.98E-27	Arabidopsis thaliana myb-like transcription factor family protein mRNA, complete cds

AT5G48290	-3.56110931	2.91E-27	Arabidopsis thaliana heavy metal transport/detoxification domain-containing protein mRNA, complete cds
AT1G56680	-3.372953093	4.31E-27	Arabidopsis thaliana Chitinase family protein mRNA, complete cds
AT4G25890	-1.39805839	4.35E-27	Arabidopsis thaliana 60S acidic ribosomal protein P3-1 mRNA, complete cds
AT3G12930	-1.015481172	4.87E-27	Arabidopsis thaliana Lojap-related protein mRNA, complete cds
AT2G29570	-1.302085548	5.61E-27	Arabidopsis thaliana proliferating cell nuclear antigen 2 mRNA, complete cds
AT5G11340	-1.183270429	6.56E-27	Arabidopsis thaliana GCN5-related N-acetyltransferase (GNAT) family protein mRNA, complete cds
AT5G52790	-2.580286521	6.94E-27	Arabidopsis thaliana CBS domain-containing protein with a domain of unknown function (DUF21) mRNA, complete cds
AT3G53630	-1.101819882	7.71E-27	Arabidopsis thaliana uncharacterized protein mRNA, complete cds
AT4G33610	-1.806606271	9.02E-27	Arabidopsis thaliana chromosome 4 sequence
AT3G12710	-1.481928745	9.29E-27	Arabidopsis thaliana putative 3-methyladenine glycosylase I mRNA, complete cds
AT2G40590	-1.00906443	1.16E-26	Arabidopsis thaliana 40S ribosomal protein S26-1 mRNA, complete cds
AT4G15510	-1.034941656	1.21E-26	Arabidopsis thaliana photosystem II reaction center PsbP family protein mRNA, complete cds
AT5G48460	-1.355814301	1.22E-26	Arabidopsis thaliana Actin binding Calponin homology (CH) domain-containing protein mRNA, complete cds
AT4G34760	-1.202735693	1.24E-26	Arabidopsis thaliana chromosome 4 sequence
AT2G14100	-2.634192464	1.34E-26	Arabidopsis thaliana cytochrome P450, family 705, subfamily A, polypeptide 13 mRNA, complete cds
AT2G38320	-1.954451739	1.44E-26	Arabidopsis thaliana trichome birefringence-like protein 34 mRNA, complete cds

AT3G28080	-1.239290515	1.70E-26	Arabidopsis thaliana MtN21-like transporter family protein UMAMIT47 mRNA, complete cds
AT4G33740	-1.004618525	1.76E-26	Arabidopsis thaliana chromosome 4 sequence
AT4G21600	-1.685061838	2.06E-26	Arabidopsis thaliana endonuclease 5 mRNA, complete cds
AT1G72200	-1.925995293	2.20E-26	Arabidopsis thaliana chromosome 1 sequence
AT3G56930	-1.12502558	2.52E-26	Arabidopsis thaliana putative S-acyltransferase mRNA, complete cds
AT2G40750	-2.770739785	2.66E-26	Arabidopsis thaliana WRKY DNA-binding protein 54 mRNA, complete cds
AT1G18650	-1.117950471	2.99E-26	Arabidopsis thaliana plasmodesmata callose-binding protein 3 mRNA, complete cds
AT1G72480	-1.16946463	3.32E-26	Arabidopsis thaliana lung seven transmembrane receptor-like protein mRNA, complete cds
AT5G66690	-1.16946463	3.32E-26	Arabidopsis thaliana chromosome 5 sequence
AT3G25980	-1.328218765	3.75E-26	Arabidopsis thaliana mitotic spindle checkpoint protein MAD2 mRNA, complete cds
AT4G22640	-3.114323075	4.68E-26	Arabidopsis thaliana chromosome 4 sequence
AT1G77990	-1.872613447	4.89E-26	Arabidopsis thaliana sulfate transporter 2;2 mRNA, complete cds
AT2G44220	-5.097409692	5.03E-26	Arabidopsis thaliana uncharacterized protein mRNA, complete cds
AT1G32170	-1.381622994	5.06E-26	Arabidopsis thaliana probable xyloglucan endotransglucosylase/hydrolase 30 mRNA, complete cds
AT3G29430	-1.727041243	5.17E-26	Arabidopsis thaliana geranylgeranyl pyrophosphate synthase 11 mRNA, complete cds
AT4G34960	-1.352044103	5.32E-26	Arabidopsis thaliana cyclophilin-like peptidyl-prolyl cis-trans isomerase family protein mRNA, complete cds
AT3G52520	-2.547681855	5.65E-26	Arabidopsis thaliana chromosome 3, complete sequence
AT2G48140	-2.011282961	5.86E-26	Arabidopsis thaliana protein EMBRYO SAC DEVELOPMENT ARREST 4

			mRNA, complete cds
AT5G06200	-2.313854539	6.79E-26	Arabidopsis thaliana uncharacterized protein mRNA, complete cds
AT3G11340	-1.151421563	7.82E-26	Arabidopsis thaliana UDP-dependent glycosyltransferase 76B1 mRNA, complete cds
AT3G05155	-2.484071205	7.86E-26	Arabidopsis thaliana mRNA for hypothetical protein, complete cds, clone: RAFL14-21-B09
AT1G62770	-1.689776974	9.05E-26	Arabidopsis thaliana plant invertase/pectin methylesterase inhibitor domain-containing protein mRNA, complete cds
AT3G07640	-1.394493244	9.54E-26	Arabidopsis thaliana uncharacterized protein mRNA, complete cds
AT1G69780	-1.191490936	9.94E-26	Arabidopsis thaliana homeobox-leucine zipper protein ATHB-13 mRNA, complete cds
AT5G38100	-4.005077812	1.07E-25	Arabidopsis thaliana putative S-adenosylmethionine-dependent methyltransferase mRNA, complete cds
AT4G25710	-1.535114102	1.16E-25	Arabidopsis thaliana chromosome 4 sequence
AT5G63470	-1.009071323	1.17E-25	Arabidopsis thaliana nuclear transcription factor Y subunit C-4 mRNA, complete cds
AT1G60050	-4.695918725	1.19E-25	Arabidopsis thaliana Nodulin MtN21 /EamA-like transporter family protein mRNA, complete cds
AT1G10020	-1.273134174	1.27E-25	Arabidopsis thaliana uncharacterized protein mRNA, complete cds
AT2G02630	-3.391857051	1.49E-25	Arabidopsis thaliana chromosome 2, complete sequence
AT1G31050	-1.548211188	1.58E-25	Arabidopsis thaliana transcription factor bHLH111 mRNA, complete cds
AT2G48080	-3.223912414	2.03E-25	Arabidopsis thaliana oxidoreductase, 2OG-Fe(II) oxygenase family protein mRNA, complete cds
AT3G09925	-4.682372193	2.13E-25	Arabidopsis thaliana pollen Ole e 1 allergen and extensin family protein mRNA, complete cds
AT3G24140	-1.148293247	2.32E-25	Arabidopsis thaliana transcription factor FAMA mRNA, complete cds
AT1G14170	-1.50586168	2.43E-25	Arabidopsis thaliana RNA-binding KH

			domain-containing protein mRNA, complete cds
AT4G18760	-1.706792559	2.81E-25	Arabidopsis thaliana chromosome 4 sequence
AT3G22570	-2.691417333	2.90E-25	Arabidopsis thaliana chromosome 3, complete sequence
AT2G19210	-3.472300043	2.91E-25	Arabidopsis thaliana putative leucine-rich repeat receptor-like protein kinase mRNA, complete cds
AT4G00165	-1.276379833	3.15E-25	Arabidopsis thaliana chromosome 4 sequence
AT3G47710	-4.163905104	3.46E-25	Arabidopsis thaliana atypical non-DNA binding bHLH protein BNQ3 mRNA, complete cds
AT1G78110	-1.086802775	4.14E-25	Arabidopsis thaliana chromosome 1 sequence
AT5G35740	-1.474380354	4.41E-25	Arabidopsis thaliana carbohydrate-binding X8 domain-containing protein mRNA, complete cds
AT3G06460	-3.369489238	4.67E-25	Arabidopsis thaliana chromosome 3, complete sequence
AT3G27190	-1.04161254	5.01E-25	Arabidopsis thaliana uridine kinase-like 2 mRNA, complete cds
AT3G16430	-1.221238279	5.12E-25	Arabidopsis thaliana jacalin-related lectin 31 mRNA, complete cds
AT2G44740	-2.482189371	5.33E-25	Arabidopsis thaliana cyclin p4;1 mRNA, complete cds
AT1G05660	-2.035371734	5.85E-25	Arabidopsis thaliana pectin lyase-like protein mRNA, complete cds
AT1G61750	-3.6793445	5.90E-25	Arabidopsis thaliana Receptor-like protein kinase-related protein mRNA, complete cds
AT1G47400	-3.967275779	6.17E-25	Arabidopsis thaliana chromosome 1 sequence
AT3G18773	-1.378351807	6.76E-25	Arabidopsis thaliana chromosome 3, complete sequence
AT5G42500	-3.062976195	7.69E-25	Arabidopsis thaliana chromosome 5 sequence
AT5G53550	-1.003440432	8.21E-25	Arabidopsis thaliana metal-nicotianamine transporter YSL3 mRNA, complete cds
AT2G20520	-7.722636063	8.24E-25	Arabidopsis thaliana chromosome 2, complete sequence
AT1G34510	-7.722636063	8.24E-25	Arabidopsis thaliana peroxidase 8

			mRNA, complete cds
AT4G33000	-1.306529444	9.49E-25	Arabidopsis thaliana calcineurin B-like protein 10 mRNA, complete cds
AT4G25070	-1.832773182	1.09E-24	Arabidopsis thaliana uncharacterized protein mRNA, complete cds
AT2G17630	-1.548545251	1.15E-24	Arabidopsis thaliana chromosome 2, complete sequence
AT4G23170	-1.26965397	1.17E-24	Arabidopsis thaliana chromosome 4 sequence
AT3G25540	-1.022931476	1.31E-24	Arabidopsis thaliana LAG1 longevity assurance-1 mRNA, complete cds
AT3G07950	-1.043771729	1.48E-24	Arabidopsis thaliana rhomboid protein-like protein mRNA, complete cds
AT5G08100	-1.251717779	1.61E-24	Arabidopsis thaliana asparaginase mRNA, complete cds
AT4G01350	-2.434883656	1.73E-24	Arabidopsis thaliana chromosome 4 sequence
AT5G27920	-1.080940268	1.91E-24	Arabidopsis thaliana F-box protein mRNA, complete cds
AT5G26300	-4.12437674	1.99E-24	Arabidopsis thaliana TRAF-like family protein mRNA, complete cds
AT2G16850	-1.141758818	2.04E-24	Arabidopsis thaliana putative aquaporin PIP2-8 mRNA, complete cds
AT5G23250	-1.116061472	2.19E-24	Arabidopsis thaliana Succinyl-CoA ligase [GDP-forming] subunit alpha-2 mRNA, complete cds
AT3G45230	-1.821185208	2.63E-24	Arabidopsis thaliana chromosome 3, complete sequence
AT3G25290	-1.048573916	3.26E-24	Arabidopsis thaliana auxin-responsive family protein mRNA, complete cds
AT1G10550	-4.110956224	3.56E-24	Arabidopsis thaliana probable xyloglucan endotransglucosylase/hydrolase protein 33 mRNA, complete cds
AT5G04470	-1.370786043	4.27E-24	Arabidopsis thaliana chromosome 5 sequence
AT3G62930	-2.539414239	4.39E-24	Arabidopsis thaliana chromosome 3, complete sequence
AT4G13575	-1.222052671	4.44E-24	Arabidopsis thaliana uncharacterized protein mRNA, complete cds
AT3G05990	-1.08390299	4.80E-24	Arabidopsis thaliana leucine-rich repeat family protein mRNA, complete cds
AT4G00680	-3.413883357	5.13E-24	Arabidopsis thaliana actin depolymerizing factor 8 mRNA, complete cds

AT5G48485	-1.166755342	5.38E-24	Arabidopsis thaliana chromosome 5 sequence
AT1G43800	-2.268560329	5.49E-24	Arabidopsis thaliana acyl-[acyl-carrier-protein] desaturase 6 mRNA, complete cds
AT4G28890	-2.96502098	5.74E-24	Arabidopsis thaliana chromosome 4 sequence
AT4G01460	-1.246217863	6.83E-24	Arabidopsis thaliana transcription factor bHLH57 mRNA, complete cds
AT5G51570	-1.168699071	7.25E-24	Arabidopsis thaliana hypersensitive-induced response protein 4 mRNA, complete cds
AT3G60290	-1.521152033	7.42E-24	Arabidopsis thaliana oxidoreductase mRNA, complete cds
AT1G03440	-1.208457179	7.79E-24	Arabidopsis thaliana chromosome 1 sequence
AT5G63810	-1.241332886	8.30E-24	Arabidopsis thaliana beta-galactosidase 10 mRNA, complete cds
AT4G25310	-2.106454832	8.59E-24	Arabidopsis thaliana 2-oxoglutarate (2OG) and Fe(II)-dependent oxygenase superfamily protein mRNA, complete cds
AT5G02050	-1.068152238	1.01E-23	Arabidopsis thaliana mitochondrial glycoprotein family protein mRNA, complete cds
AT2G16380	-1.094176502	1.03E-23	Arabidopsis thaliana Sec14p-like phosphatidylinositol transfer family protein mRNA, complete cds
AT3G51360	-3.617416751	1.07E-23	Arabidopsis thaliana aspartyl protease family protein mRNA, complete cds
AT1G73270	-2.326420516	1.13E-23	Arabidopsis thaliana serine carboxypeptidase-like 6 mRNA, complete cds
AT5G03995	-4.083734756	1.14E-23	Arabidopsis thaliana uncharacterized protein mRNA, complete cds
AT3G43890	-4.083734756	1.14E-23	Arabidopsis thaliana chromosome 3, complete sequence
AT5G07110	-1.660166399	1.21E-23	Arabidopsis thaliana chromosome 5 sequence
AT2G18450	-2.704473486	1.33E-23	Arabidopsis thaliana succinate dehydrogenase [ubiquinone] flavoprotein subunit 2 mRNA, complete cds
AT3G05150	-2.893051194	1.36E-23	Arabidopsis thaliana sugar transporter ERD6-like 8 mRNA, complete cds

AT3G45700	-2.276379833	1.36E-23	Arabidopsis thaliana putative nitrate excretion transporter 5 mRNA, complete cds
AT4G30910	-1.168576544	1.56E-23	Ostreococcus lucimarinus CCE9901 4-hydroxy-3-methylbut-2-en-1-yl diphosphate synthase, putative chloroplast precursor (1-hydroxy-2-methyl-2-(E)-butenyl 4-diph>gi|145350195|ref|NM_119238.4| Arabidopsis thaliana cytosol aminopeptidase family protein mRNA, complete cds
AT4G24310	-2.659708473	1.66E-23	Arabidopsis thaliana chromosome 4 sequence
AT2G25980	-1.153647585	1.67E-23	Arabidopsis thaliana myrosinase-binding protein-like protein mRNA, complete cds
AT4G01440	-1.148829784	1.71E-23	Arabidopsis thaliana nodulin MtN21-like transporter family protein mRNA, complete cds
AT5G57700	-1.324635002	1.83E-23	Arabidopsis thaliana BNR/Asp-box repeat family protein mRNA, complete cds
AT2G31085	-3.135781503	1.85E-23	Arabidopsis thaliana chromosome 2, complete sequence
AT1G63260	-1.59021861	1.86E-23	Arabidopsis thaliana tetraspanin10 mRNA, complete cds
AT1G30750	-1.858236423	1.96E-23	Arabidopsis thaliana chromosome 1 sequence
AT5G56660	-1.109337341	2.20E-23	Arabidopsis thaliana IAA-amino acid hydrolase ILR1-like 2 mRNA, complete cds
AT2G28990	-2.478013695	2.46E-23	Arabidopsis thaliana putative LRR receptor-like serine/threonine-protein kinase mRNA, complete cds
AT3G62280	-3.288352475	2.53E-23	Arabidopsis thaliana GDSL esterase/lipase mRNA, complete cds
AT4G16260	-1.109992822	2.56E-23	Arabidopsis thaliana putative beta-1,3-endoglucanase mRNA, complete cds
AT4G21445	-1.413883357	2.85E-23	Arabidopsis thaliana uncharacterized protein mRNA, complete cds
AT4G11120	-1.270993287	3.06E-23	Arabidopsis thaliana putative translation elongation factor Ts mRNA, complete cds

AT2G31890	-1.040608382	3.15E-23	Arabidopsis thaliana protein RAP mRNA, complete cds
AT1G60390	-1.33608608	3.58E-23	Arabidopsis thaliana mRNA for hypothetical protein, complete cds, clone: RAFL14-16-D22
AT2G30210	-1.16946463	3.92E-23	Arabidopsis thaliana laccase 3 mRNA, complete cds
AT1G09630	-1.370844517	3.96E-23	Arabidopsis thaliana RAB GTPase 11C mRNA, complete cds
AT5G12900	-1.116302618	4.02E-23	Arabidopsis thaliana uncharacterized protein mRNA, complete cds
AT1G50050	-2.469024911	4.12E-23	Arabidopsis thaliana putative pathogenesis-related protein mRNA, complete cds
AT1G70890	-1.210791492	4.20E-23	Arabidopsis thaliana MLP-like protein 43 mRNA, complete cds
AT2G35240	-1.013766591	4.95E-23	Arabidopsis thaliana putative plastid developmental protein DAG mRNA, complete cds
AT4G02990	-1.206149911	5.05E-23	Arabidopsis thaliana mitochondrial transcription termination factor-like protein BELAYA SMERT mRNA, complete cds
AT4G30110	-1.389079966	5.22E-23	Arabidopsis thaliana cadmium/zinc-transporting ATPase HMA2 mRNA, complete cds
AT3G55010	-1.105479709	6.47E-23	Arabidopsis thaliana phosphoribosylformylglycinamidine cyclo-ligase mRNA, complete cds
AT5G66230	-1.362693676	8.19E-23	Arabidopsis thaliana Chalcone-flavanone isomerase family protein mRNA, complete cds
AT5G46240	-1.111079917	8.73E-23	Arabidopsis thaliana potassium channel KAT1 mRNA, complete cds
AT2G27400	-2.052941602	9.42E-23	Arabidopsis thaliana chromosome 2, complete sequence
AT1G51850	-1.947630473	9.50E-23	Arabidopsis thaliana Leucine-rich repeat protein kinase family protein mRNA, complete cds
AT4G15400	-1.822661866	1.10E-22	Arabidopsis thaliana chromosome 4 sequence
AT3G47560	-1.050103978	1.12E-22	Arabidopsis thaliana alpha/beta-Hydrolases superfamily protein mRNA, complete cds

AT2G29180	-1.160902616	1.20E-22	Arabidopsis thaliana uncharacterized protein mRNA, complete cds
AT2G04170	-1.534498749	1.23E-22	Arabidopsis thaliana TRAF-like family protein mRNA, complete cds
AT1G12040	-6.462246379	1.33E-22	Arabidopsis thaliana chromosome 1 sequence
AT3G19810	-1.003448772	1.34E-22	Arabidopsis thaliana uncharacterized protein mRNA, complete cds
AT1G63295	-1.79627007	1.55E-22	Arabidopsis thaliana Remorin family protein mRNA, complete cds
AT1G45015	-2.900411899	1.57E-22	Arabidopsis thaliana MD-2-related lipid recognition domain-containing protein mRNA, complete cds
AT1G61667	-2.190340002	1.84E-22	Arabidopsis thaliana uncharacterized protein mRNA, complete cds
AT2G03260	-2.954451739	2.00E-22	Arabidopsis thaliana phosphate transporter PHO1-like 2 mRNA, complete cds
AT3G32040	-4.013345428	2.12E-22	Arabidopsis thaliana geranylgeranyl pyrophosphate synthase 12 mRNA, complete cds
AT3G50460	-4.232436486	2.13E-22	Arabidopsis thaliana RPW8-like protein 2 mRNA, complete cds
AT4G28850	-4.232436486	2.13E-22	Arabidopsis thaliana xyloglucan endotransglucosylase/hydrolase 26 mRNA, complete cds
AT1G35612.1	-1.031560664	2.22E-22	Arabidopsis thaliana chromosome 1 sequence
AT1G34010	-1.412780801	2.33E-22	Arabidopsis thaliana uncharacterized protein mRNA, complete cds
AT5G24100	-1.975931466	2.44E-22	Arabidopsis thaliana Leucine-rich repeat protein kinase family protein mRNA, complete cds
AT5G49870	-3.013345428	2.49E-22	Arabidopsis thaliana Mannose-binding lectin superfamily protein mRNA, complete cds
AT1G30110	-1.094932962	3.02E-22	Arabidopsis thaliana ppGpp pyrophosphohydrolase mRNA, complete cds
AT3G54830	-1.276379833	3.04E-22	Arabidopsis thaliana transmembrane amino acid transporter family protein mRNA, complete cds
AT2G18570	-1.30416299	3.25E-22	Arabidopsis thaliana chromosome 2, complete sequence

AT1G68220	-1.025914101	3.41E-22	Arabidopsis thaliana uncharacterized protein mRNA, complete cds
AT4G14780	-3.539414239	3.43E-22	Arabidopsis thaliana protein kinase family protein mRNA, complete cds
AT3G12750	-1.707141685	3.44E-22	Arabidopsis thaliana zinc transporter 1 mRNA, complete cds
AT5G42420	-1.163698784	4.05E-22	Arabidopsis thaliana Nucleotide-sugar transporter family protein mRNA, complete cds
AT2G28970	-2.369489238	4.15E-22	Arabidopsis thaliana putative leucine-rich repeat protein kinase mRNA, complete cds
AT4G26220	-2.340105431	4.64E-22	Arabidopsis thaliana putative caffeoyl-CoA O-methyltransferase mRNA, complete cds
AT5G03120	-2.112599572	5.55E-22	Arabidopsis thaliana uncharacterized protein mRNA, complete cds
AT4G22460	-7.50904059	5.57E-22	Arabidopsis thaliana chromosome 4 sequence
AT3G29034	-2.057262544	6.00E-22	Arabidopsis thaliana chromosome 3, complete sequence
AT3G46400	-2.931731662	6.03E-22	Arabidopsis thaliana Leucine-rich repeat protein kinase family protein mRNA, complete cds
AT4G34580	-2.04002747	6.09E-22	Arabidopsis thaliana phosphatidylinositol transfer protein COW1 mRNA, complete cds
AT2G45360	-2.391091492	6.13E-22	Arabidopsis thaliana uncharacterized protein mRNA, complete cds
AT2G41240	-3.13665507	6.46E-22	Arabidopsis thaliana transcription factor bHLH100 mRNA, complete cds
AT1G06120	-3.805308299	6.72E-22	Arabidopsis thaliana delta-9 desaturase-like 3 protein mRNA, complete cds
AT5G57780	-2.482830711	6.76E-22	Arabidopsis thaliana uncharacterized protein mRNA, complete cds
AT5G15520	-1.839993014	7.64E-22	Arabidopsis thaliana 40S ribosomal protein S19-2 mRNA, complete cds
AT4G37750	-1.066532665	8.61E-22	Arabidopsis thaliana AP2-like ethylene-responsive transcription factor ANT mRNA, complete cds
AT2G42040	-1.436160278	9.01E-22	Arabidopsis thaliana uncharacterized protein mRNA, complete cds
AT5G54710	-1.044740624	9.41E-22	Arabidopsis thaliana Ankyrin repeat

			family protein mRNA, complete cds
AT2G22920	-1.954451739	9.71E-22	Arabidopsis thaliana serine carboxypeptidase-like 12 mRNA, complete cds
AT2G04790	-1.368045821	9.88E-22	Arabidopsis thaliana uncharacterized protein mRNA, complete cds
AT1G34315	-1.639355677	1.14E-21	Arabidopsis thaliana uncharacterized protein mRNA, complete cds
AT1G05880	-3.790953006	1.20E-21	Arabidopsis thaliana putative E3 ubiquitin-protein ligase ARI12 mRNA, complete cds
AT2G40480	-1.144276297	1.28E-21	Arabidopsis thaliana uncharacterized protein mRNA, complete cds
AT5G56120	-1.44749175	1.29E-21	Arabidopsis thaliana uncharacterized protein mRNA, complete cds
AT3G07340	-1.387411146	1.30E-21	Arabidopsis thaliana transcription factor bHLH62 mRNA, complete cds
AT4G23300	-1.049478346	1.52E-21	Arabidopsis thaliana cysteine-rich receptor-like protein kinase 22 mRNA, complete cds
AT1G29530	-1.156759914	1.66E-21	Arabidopsis thaliana uncharacterized protein mRNA, complete cds
AT5G26670	-1.49502012	1.69E-21	Arabidopsis thaliana pectinacetylesterase family protein mRNA, complete cds
AT4G17070	-1.000513521	1.80E-21	Arabidopsis thaliana peptidyl-prolyl cis-trans isomerase mRNA, complete cds
AT4G08035	-1.147096817	2.01E-21	Arabidopsis thaliana chromosome 4 sequence
AT3G56970	-3.626877081	2.07E-21	Arabidopsis thaliana transcription factor ORG2 mRNA, complete cds
AT4G10540	-5.363842675	2.23E-21	Arabidopsis thaliana Subtilase family protein mRNA, complete cds
AT5G52260	-4.446304835	2.24E-21	Arabidopsis thaliana myb domain protein 19 mRNA, complete cds
AT3G26500	-1.555533905	2.24E-21	Arabidopsis thaliana plant intracellular ras group-related LRR 2 mRNA, complete cds
AT4G36570	-2.05174894	2.43E-21	Arabidopsis thaliana protein RAD-like 3 mRNA, complete cds
AT3G05625	-1.307630767	2.43E-21	Arabidopsis thaliana tetratricopeptide repeat-containing protein mRNA, complete cds
AT3G24020	-1.490002045	2.49E-21	Arabidopsis thaliana chromosome 3, complete sequence

AT1G29440	-1.733852599	2.83E-21	Arabidopsis thaliana chromosome 1 sequence
AT3G03040	-1.123562808	2.91E-21	Arabidopsis thaliana F-box protein mRNA, complete cds
AT1G76240	-1.685393545	3.03E-21	Arabidopsis thaliana chromosome 1 sequence
AT1G60060	-2.304036176	3.46E-21	Arabidopsis thaliana serine/threonine-protein kinase WNK (With No lysine)-related protein mRNA, complete cds
AT3G60330	-2.061980203	3.82E-21	Arabidopsis thaliana H(+)-ATPase 7 mRNA, complete cds
AT4G26780	-1.508717442	3.88E-21	Arabidopsis thaliana molecular chaperone GrpE mRNA, complete cds
AT1G72360	-1.947072208	3.88E-21	Arabidopsis thaliana chromosome 1 sequence
AT4G04695	-1.947072208	3.88E-21	Arabidopsis thaliana calcium-dependent protein kinase 31 mRNA, complete cds
AT4G12917	-1.66386161	3.93E-21	Arabidopsis thaliana Full-length cDNA Complete sequence from clone GSLTSIL58ZA07 of Silique of strain col-0 of Arabidopsis thaliana (thale cress)
AT3G08600	-1.402332495	4.02E-21	Arabidopsis thaliana chromosome 3, complete sequence
AT3G26610	-1.561356792	4.08E-21	Arabidopsis thaliana putative polygalacturonase / pectinase mRNA, complete cds
AT2G28660	-1.350611228	4.20E-21	Arabidopsis thaliana chloroplast-targeted copper chaperone protein mRNA, complete cds
AT3G60440	-1.214514578	4.26E-21	Arabidopsis thaliana phosphoglycerate mutase family protein mRNA, complete cds
AT4G08685	-1.001575651	4.61E-21	Arabidopsis thaliana protein SAH7 mRNA, complete cds
AT4G20210	-2.001757453	6.30E-21	Arabidopsis thaliana terpenoid synthase 8 mRNA, complete cds
AT2G23770	-1.19779781	6.40E-21	Arabidopsis thaliana chromosome 2, complete sequence
AT1G26820	-1.419887091	7.47E-21	Arabidopsis thaliana ribonuclease 3 mRNA, complete cds
AT3G50210	-1.108990007	7.49E-21	Arabidopsis thaliana 2-oxoglutarate-Fe(II)-dependent oxygenase domain-containing protein

			mRNA, complete cds
AT2G40110	-1.474842238	7.90E-21	Arabidopsis thaliana Yippee family putative zinc-binding protein mRNA, complete cds
AT3G48940	-4.778880174	7.94E-21	Arabidopsis thaliana Remorin-like protein mRNA, complete cds
AT1G30900	-1.014993281	8.09E-21	Arabidopsis thaliana vacuolar sorting receptor 6 mRNA, complete cds
AT5G42680	-1.852572125	8.19E-21	Arabidopsis thaliana uncharacterized protein mRNA, complete cds
AT2G26640	-1.12437674	8.52E-21	Arabidopsis thaliana 3-ketoacyl-CoA synthase 11 mRNA, complete cds
AT2G26760	-1.479293247	8.85E-21	Arabidopsis thaliana cyclin-B1-4 mRNA, complete cds
AT3G62210	-2.43392111	8.91E-21	Arabidopsis thaliana putative endonuclease or glycosyl hydrolase mRNA, complete cds
AT1G03210	-1.215215971	9.20E-21	Arabidopsis thaliana Phenazine biosynthesis PhzC/PhzF protein mRNA, complete cds
AT5G23400	-1.33472382	1.08E-20	Arabidopsis thaliana chromosome 5 sequence
AT1G72230	-1.325139145	1.18E-20	Arabidopsis thaliana plastocyanin-like domain-containing protein mRNA, complete cds
AT5G03360	-2.930204192	1.21E-20	Arabidopsis thaliana C1 domain-containing protein mRNA, complete cds
AT2G14510	-2.809503403	1.25E-20	Arabidopsis thaliana leucine-rich repeat protein kinase family protein mRNA, complete cds
AT1G77630	-1.350143888	1.28E-20	Arabidopsis thaliana LysM domain-containing GPI-anchored protein 3 mRNA, complete cds
AT3G01080	-3.150848951	1.29E-20	Arabidopsis thaliana WRKY DNA-binding protein 58 mRNA, complete cds
AT3G15820	-1.019104697	1.32E-20	Arabidopsis thaliana phosphatidylcholine:diacylglycerol cholinephosphotransferase mRNA, complete cds
AT4G13660	-1.3066624	1.38E-20	Arabidopsis thaliana pinoresinol reductase 2 mRNA, complete cds
AT1G51790	-1.49789497	1.39E-20	Arabidopsis thaliana leucine-rich repeat

			protein kinase-like protein mRNA, complete cds
AT1G63460	-1.040792588	1.72E-20	Arabidopsis thaliana glutathione peroxidase 8 mRNA, complete cds
AT4G18800	-1.345642496	1.84E-20	Arabidopsis thaliana RAB GTPase homolog A1D mRNA, complete cds
AT4G22790	-2.606528435	1.85E-20	Arabidopsis thaliana chromosome 4 sequence
AT4G34740	-1.068365059	1.86E-20	Arabidopsis thaliana chromosome 4 sequence
AT3G60070	-1.539414239	1.99E-20	Arabidopsis thaliana major facilitator protein mRNA, complete cds
AT2G32300	-1.517881078	2.15E-20	Arabidopsis thaliana uclacyanin 1 mRNA, complete cds
AT5G20400	-1.58038602	2.45E-20	Arabidopsis thaliana flavanone 3 hydroxylase-like protein mRNA, complete cds
AT4G19370	-2.267041969	2.54E-20	Arabidopsis thaliana uncharacterized protein mRNA, complete cds
AT3G46880	-3.222940575	2.67E-20	Arabidopsis thaliana uncharacterized protein mRNA, complete cds
AT3G26200	-1.586719954	2.69E-20	Arabidopsis thaliana cytochrome P450 71B22 mRNA, complete cds
AT5G39860	-1.670658773	2.85E-20	Arabidopsis thaliana bHLH transcription factor PRE1 mRNA, complete cds
AT3G52940	-1.205259842	2.88E-20	Arabidopsis thaliana delta(14)-sterol reductase mRNA, complete cds
AT4G25110	-2.213838367	2.94E-20	Arabidopsis thaliana metacaspase 2 mRNA, complete cds
AT2G23630	-3.048427887	3.28E-20	Arabidopsis thaliana protein SKU5 similar 16 mRNA, complete cds
AT3G23290	-1.083531688	3.50E-20	Arabidopsis thaliana protein LIGHT SENSITIVE HYPOCOTYLS 4 mRNA, complete cds
AT1G33440	-1.477353271	3.94E-20	Arabidopsis thaliana probable peptide/nitrate transporter mRNA, complete cds
AT2G15390	-1.37744676	4.14E-20	Arabidopsis thaliana probable fucosyltransferase 4 mRNA, complete cds
AT1G21210	-2.230576144	4.50E-20	Arabidopsis thaliana wall-associated receptor kinase 4 mRNA, complete cds
AT1G04980	-1.225478935	4.75E-20	Arabidopsis thaliana protein disulfide-isomerase like 2-2 mRNA,

			complete cds
AT2G39140	-1.043456745	5.10E-20	Arabidopsis thaliana protein SUPPRESSOR OF VARIEGATION 1 mRNA, complete cds
AT1G55140	-1.135023984	5.26E-20	Arabidopsis thaliana ribonuclease III-like protein mRNA, complete cds
AT2G21880	-1.861342334	5.42E-20	Arabidopsis thaliana RAB GTPase-like protein 7A mRNA, complete cds
AT1G28660	-1.901984319	5.93E-20	Arabidopsis thaliana GDSL esterase/lipase mRNA, complete cds
AT3G09410	-1.059901037	6.25E-20	Arabidopsis thaliana putative pectinacetylesterase mRNA, complete cds
AT4G11230	-2.248182942	6.85E-20	Arabidopsis thaliana putative respiratory burst oxidase-I mRNA, complete cds
AT2G34180	-1.725364665	7.27E-20	Arabidopsis thaliana chromosome 2, complete sequence
AT2G26410	-4.07546714	7.31E-20	Arabidopsis thaliana protein IQ-domain 4 mRNA, complete cds
AT3G56070	-1.073189285	7.47E-20	Arabidopsis thaliana rotamase cyclophilin 2 mRNA, complete cds
AT1G48610	-1.105695319	7.51E-20	Arabidopsis thaliana AT hook motif-containing protein mRNA, complete cds
AT5G08620	-1.008446628	7.84E-20	Arabidopsis thaliana DEAD-box ATP-dependent RNA helicase 25 mRNA, complete cds
AT4G15500	-1.74481359	8.28E-20	Arabidopsis thaliana chromosome 4 sequence
AT2G01990	-1.765295647	9.35E-20	Arabidopsis thaliana uncharacterized protein mRNA, complete cds
AT3G48650	-1.908648049	9.55E-20	Arabidopsis thaliana chromosome 3, complete sequence
AT5G49215	-1.70026825	9.65E-20	Arabidopsis thaliana pectin lyase-like superfamily protein mRNA, complete cds
AT2G47540	-3.022196345	1.00E-19	Arabidopsis thaliana pollen Ole e 1 allergen and extensin family protein mRNA, complete cds
AT1G61590	-1.496021644	1.02E-19	Arabidopsis thaliana putative receptor-like cytoplasmic kinase mRNA, complete cds
AT1G51800	-1.322922419	1.09E-19	Arabidopsis thaliana putative leucine-rich repeat protein kinase mRNA, complete

			cds
AT5G42250	-1.622608175	1.16E-19	Arabidopsis thaliana alcohol dehydrogenase-like 7 mRNA, complete cds
AT3G02885	-1.630016788	1.26E-19	Arabidopsis thaliana gibberellin-regulated protein 5 mRNA, complete cds
AT2G40320	-1.377663169	1.27E-19	Arabidopsis thaliana protein trichome birefringence-like 33 mRNA, complete cds
AT1G21690	-1.303601302	1.29E-19	Arabidopsis thaliana replication factor C subunit 4 mRNA, complete cds
AT1G65970	-2.349311356	1.29E-19	Arabidopsis thaliana thioredoxin-dependent peroxidase 2 mRNA, complete cds
AT3G20110	-3.845222669	1.30E-19	Arabidopsis thaliana cytochrome P450 705A20 mRNA, complete cds
AT2G03720	-3.845222669	1.30E-19	Arabidopsis thaliana MORPHOGENESIS OF ROOT HAIR 6 mRNA, complete cds
AT3G27180	-1.084575732	1.39E-19	Arabidopsis thaliana S-adenosylmethionine-dependent methyltransferase-domain containing protein mRNA, complete cds
AT5G10210	-1.267740915	1.69E-19	Arabidopsis thaliana chromosome 5 sequence
AT4G05330	-1.500644669	1.88E-19	Arabidopsis thaliana putative ADP-ribosylation factor GTPase-activating protein AGD13 mRNA, complete cds
AT3G27906	-1.202379252	1.88E-19	Arabidopsis thaliana chromosome 3, complete sequence
AT2G03310	-2.202379252	1.96E-19	Arabidopsis thaliana uncharacterized protein mRNA, complete cds
AT4G25240	-1.4800801	1.97E-19	Arabidopsis thaliana monocopper oxidase-like protein SKS1 mRNA, complete cds
AT3G12977	-2.17684416	2.08E-19	Arabidopsis thaliana no apical meristem-domain containing transcriptional regulator mRNA, complete cds
AT5G25090	-1.511180559	2.34E-19	Arabidopsis thaliana early nodulin-like protein 13 mRNA, complete cds
AT5G42510	-2.306968153	2.36E-19	Arabidopsis thaliana chromosome 5

			sequence
AT2G04800	-2.435578428	2.47E-19	Arabidopsis thaliana chromosome 2, complete sequence
AT3G17780	-1.021417562	3.41E-19	Arabidopsis thaliana uncharacterized protein mRNA, complete cds
AT1G77020	-1.505466907	3.45E-19	Arabidopsis thaliana DNAJ heat shock N-terminal domain-containing protein mRNA, complete cds
AT5G53500	-1.02515473	3.48E-19	Arabidopsis thaliana transducin/WD40 domain-containing protein mRNA, complete cds
AT4G29260	-1.098287511	3.51E-19	Arabidopsis thaliana HAD superfamily, subfamily IIIB acid phosphatase mRNA, complete cds
AT3G63470	-2.296843936	3.90E-19	Arabidopsis thaliana serine carboxypeptidase-like 40 mRNA, complete cds
AT3G45070	-2.035371734	4.05E-19	Arabidopsis thaliana chromosome 3, complete sequence
AT5G58480	-1.351667961	4.17E-19	Arabidopsis thaliana glucan endo-1,3-beta-glucosidase 9 mRNA, complete cds
AT1G72920	-1.717201359	4.56E-19	Arabidopsis thaliana Toll-Interleukin-Resistance domain-containing protein mRNA, complete cds
AT5G26622	-1.066562105	4.67E-19	Arabidopsis thaliana chromosome 5 sequence
AT5G12420	-1.162693672	4.85E-19	Arabidopsis thaliana O-acyltransferase (WSD1-like) family protein mRNA, complete cds
AT4G00950	-1.499730538	5.09E-19	Arabidopsis thaliana uncharacterized protein mRNA, complete cds
AT1G51890	-1.673550912	5.17E-19	Arabidopsis thaliana probable LRR receptor-like protein kinase mRNA, complete cds
AT5G58310	-1.116202809	5.47E-19	Arabidopsis thaliana methyl esterase 18 mRNA, complete cds
AT4G11530	-1.863303851	5.60E-19	Arabidopsis thaliana putative cysteine-rich receptor-like protein kinase 35 mRNA, complete cds
AT1G52930	-1.011532834	5.97E-19	Arabidopsis thaliana ribosomal RNA processing Brix domain protein mRNA, complete cds

AT5G02360	-2.088307485	6.14E-19	Arabidopsis thaliana DC1 domain-containing protein mRNA, complete cds
AT4G12050	-1.649913781	6.66E-19	Arabidopsis thaliana chromosome 4 sequence
AT5G14020	-2.901984319	7.32E-19	Arabidopsis thaliana Endosomal targeting BRO1-like domain-containing protein mRNA, complete cds
AT5G26680	-1.619584588	7.75E-19	Arabidopsis thaliana flap endonuclease-1 mRNA, complete cds
AT2G19590	-1.283288203	8.47E-19	Arabidopsis thaliana 1-aminocyclopropane-1-carboxylate oxidase 1 mRNA, complete cds
AT1G74890	-1.675550927	8.49E-19	Arabidopsis thaliana two-component response regulator ARR15 mRNA, complete cds
AT5G07475	-2.12437674	9.30E-19	Arabidopsis thaliana cupredoxin superfamily protein mRNA, complete cds
AT2G36120	-1.285657647	9.73E-19	Arabidopsis thaliana chromosome 2, complete sequence
AT1G50055	-2.079147486	9.89E-19	Arabidopsis thaliana chromosome 1 sequence
AT1G49410	-1.019743199	9.97E-19	Arabidopsis thaliana translocase of the outer mitochondrial membrane 6 mRNA, complete cds
AT2G29320	-1.605604519	1.06E-18	Arabidopsis thaliana tropinone reductase-like protein mRNA, complete cds
AT5G50375	-1.288061639	1.12E-18	Arabidopsis thaliana cyclopropyl isomerase mRNA, complete cds
AT2G22510	-1.183652549	1.13E-18	Arabidopsis thaliana chromosome 2, complete sequence
AT3G59320	-1.323280808	1.17E-18	Arabidopsis thaliana uncharacterized protein mRNA, complete cds
AT5G20050	-1.031727452	1.23E-18	Arabidopsis thaliana chromosome 5 sequence
AT4G34790	-6.144276297	1.33E-18	Arabidopsis thaliana chromosome 4 sequence
AT4G01720	-1.116013267	1.38E-18	Arabidopsis thaliana putative WRKY transcription factor 47 mRNA, complete cds
AT3G57220	-1.079982621	1.39E-18	Arabidopsis thaliana Glycosyl transferase family 4 protein mRNA, complete cds

AT1G27140	-4.617416751	1.43E-18	Arabidopsis thaliana glutathione S-transferase tau 14 mRNA, complete cds
AT4G01525.1	-2.601341988	1.46E-18	Arabidopsis thaliana chromosome 4 sequence
AT3G62020	-1.048687808	1.53E-18	Arabidopsis thaliana chromosome 3, complete sequence
AT2G38600	-1.905542138	1.55E-18	Arabidopsis thaliana HAD superfamily, subfamily IIIB acid phosphatase mRNA, complete cds
AT2G18290	-1.237090702	1.84E-18	Arabidopsis thaliana anaphase-promoting complex subunit 10 mRNA, complete cds
AT2G35790	-1.091230472	1.90E-18	Arabidopsis thaliana uncharacterized protein mRNA, complete cds
AT5G51670	-1.545646193	1.97E-18	Arabidopsis thaliana uncharacterized protein mRNA, complete cds
AT4G13580	-2.180164518	2.13E-18	Arabidopsis thaliana chromosome 4 sequence
AT3G15570	-1.521266893	2.13E-18	Arabidopsis thaliana phototropic-responsive NPH3 family protein mRNA, complete cds
AT1G62280	-1.758054526	2.43E-18	Arabidopsis thaliana S-type anion channel SLAH1 mRNA, complete cds
AT4G26670	-1.207217809	2.55E-18	Arabidopsis thaliana mitochondrial import inner membrane translocase subunit TIM22-2 mRNA, complete cds
AT4G39120	-1.103153469	2.58E-18	Arabidopsis thaliana myo-inositol monophosphatase like 2 mRNA, complete cds
AT5G03552	-2.287027078	2.63E-18	Arabidopsis thaliana chromosome 5 sequence
AT4G15340	-1.163398629	2.64E-18	Arabidopsis thaliana pentacyclic triterpene synthase 1 mRNA, complete cds
AT3G47010	-1.192395163	2.74E-18	Arabidopsis thaliana glycosyl hydrolase family protein mRNA, complete cds
AT3G26700	-1.192395163	2.74E-18	Arabidopsis thaliana protein kinase family protein mRNA, complete cds
AT5G19040	-1.274032079	3.17E-18	Arabidopsis thaliana chromosome 5 sequence
AT5G50300	-2.197308263	3.28E-18	Arabidopsis thaliana chromosome 5 sequence
AT3G21550	-1.127288335	3.40E-18	Arabidopsis thaliana chromosome 3,

			complete sequence
AT3G48590	-1.069335376	3.46E-18	Arabidopsis thaliana nuclear transcription factor Y subunit C-1 mRNA, complete cds
AT5G44410	-1.172716274	3.47E-18	Arabidopsis thaliana chromosome 5 sequence
AT5G04970	-1.39502433	3.67E-18	Arabidopsis thaliana putative pectinesterase/pectinesterase inhibitor 47 mRNA, complete cds
AT3G11520	-1.750520853	3.73E-18	Arabidopsis thaliana cyclin-B1-3 mRNA, complete cds
AT3G61270	-1.631156808	3.77E-18	Arabidopsis thaliana lipopolysaccharide-modifying domain-containing protein mRNA, complete cds
AT2G16530	-1.140318284	3.85E-18	Arabidopsis thaliana putative polyprenol reductase 2 mRNA, complete cds
AT3G23830	-1.134237989	3.91E-18	Arabidopsis thaliana glycine-rich RNA-binding protein 4 mRNA, complete cds
AT1G09390	-1.288452666	3.92E-18	Arabidopsis thaliana GDSL esterase/lipase mRNA, complete cds
AT5G41050	-1.079652296	4.08E-18	Arabidopsis thaliana pollen Ole e 1 allergen and extensin family protein mRNA, complete cds
AT1G74460	-1.533155249	4.35E-18	Arabidopsis thaliana GDSL esterase/lipase mRNA, complete cds
AT5G14090	-1.647938696	4.48E-18	Arabidopsis thaliana uncharacterized protein mRNA, complete cds
AT5G52350	-4.578942604	4.58E-18	Arabidopsis thaliana exocyst subunit exo70 family protein A3 mRNA, complete cds
AT1G66440	-7.163905104	4.70E-18	Arabidopsis thaliana chromosome 1 sequence
AT1G23410	-1.810441436	4.91E-18	Arabidopsis thaliana chromosome 1 sequence
AT1G14260	-1.823557672	5.15E-18	Arabidopsis thaliana RING-variant domain-containing protein mRNA, complete cds
AT5G43540	-3.187112495	5.15E-18	Arabidopsis thaliana chromosome 5 sequence
AT3G26680	-1.345472357	5.58E-18	Arabidopsis thaliana DNA cross-link repair protein SNM1 mRNA, complete cds

AT5G58360	-5.104198858	5.80E-18	Arabidopsis thaliana chromosome 5 sequence
AT4G02630	-1.097252339	6.50E-18	Arabidopsis thaliana chromosome 4 sequence
AT3G45410	-1.963440522	6.61E-18	Arabidopsis thaliana chromosome 3, complete sequence
AT3G17160	-1.092708825	7.59E-18	Arabidopsis thaliana uncharacterized protein mRNA, complete cds
AT2G01660	-1.325818766	7.64E-18	Arabidopsis thaliana plasmodesmata-located protein 6 mRNA, complete cds
AT2G41970	-4.202379252	7.90E-18	Arabidopsis thaliana putative protein kinase mRNA, complete cds
AT1G02260	-1.053987412	8.46E-18	Arabidopsis thaliana divalent ion symporter mRNA, complete cds
AT1G60110	-2.912223503	8.50E-18	Arabidopsis thaliana jacalin-like lectin domain-containing protein mRNA, complete cds
AT4G01140	-2.775481598	8.65E-18	Arabidopsis thaliana chromosome 4 sequence
AT2G36970	-1.954451739	1.05E-17	Arabidopsis thaliana UDP-glycosyltransferase 86A1 mRNA, complete cds
AT5G01050	-2.032454251	1.05E-17	Arabidopsis thaliana laccase-9 mRNA, complete cds
AT1G26240	-3.276379833	1.06E-17	Arabidopsis thaliana chromosome 1 sequence
AT2G02020	-1.255111217	1.25E-17	Arabidopsis thaliana peptide transporter 4 mRNA, complete cds
AT3G47200	-1.259590176	1.67E-17	Arabidopsis thaliana uncharacterized protein mRNA, complete cds
AT2G27370	-1.698990552	1.79E-17	Arabidopsis thaliana uncharacterized protein mRNA, complete cds
AT1G72730	-1.108652388	1.90E-17	Arabidopsis thaliana eukaryotic initiation factor 4A-3 mRNA, complete cds
AT4G12360	-7.104198858	1.90E-17	Arabidopsis thaliana Bifunctional inhibitor/lipid-transfer protein/seed storage 2S albumin superfamily protein mRNA, complete cds
AT4G09420	-2.819522159	1.97E-17	Arabidopsis thaliana TIR-NBS class of disease resistance protein mRNA, complete cds
AT1G38131	-1.416794953	2.10E-17	Arabidopsis thaliana O-fucosyltransferase-like protein mRNA,

			complete cds
AT1G50520	-1.484272685	2.20E-17	Arabidopsis thaliana cytochrome P450, family 705, subfamily A, polypeptide 27 mRNA, complete cds
AT2G26215.1	-1.840280719	2.20E-17	Arabidopsis thaliana chromosome 2, complete sequence
AT2G28860	-3.899310184	2.53E-17	Arabidopsis thaliana chromosome 2, complete sequence
AT1G65295	-1.022397891	2.59E-17	Arabidopsis thaliana uncharacterized protein mRNA, complete cds
AT1G24320	-1.936304392	2.62E-17	Arabidopsis thaliana alpha-glucosidase 2 mRNA, complete cds
AT4G25260	-1.40665903	2.69E-17	Arabidopsis thaliana chromosome 4 sequence
AT5G15150	-1.316272561	2.91E-17	Arabidopsis thaliana mRNA for homeobox protein, complete cds, clone: RAFL16-61-C06
AT1G77750	-1.268962362	2.94E-17	Arabidopsis thaliana small ribosomal subunit protein S13 mRNA, complete cds
AT3G59310	-1.43037148	3.03E-17	Arabidopsis thaliana uncharacterized protein mRNA, complete cds
AT2G31081	-7.083734756	3.03E-17	Arabidopsis thaliana chromosome 2, complete sequence
AT3G07070	-3.243958356	3.31E-17	Arabidopsis thaliana serine/threonine-protein kinase mRNA, complete cds
AT2G16970	-2.805308299	3.40E-17	Arabidopsis thaliana tetracycline transporter-like protein 1 mRNA, complete cds
AT3G03190	-1.252132287	3.81E-17	Arabidopsis thaliana glutathione S-transferase F11 mRNA, complete cds
AT2G14460	-1.353001115	4.16E-17	Arabidopsis thaliana chromosome 2, complete sequence
AT2G20750	-1.519785433	4.28E-17	Arabidopsis thaliana expansin B1 mRNA, complete cds
AT3G16690	-1.424397579	4.41E-17	Arabidopsis thaliana bidirectional sugar transporter SWEET16 mRNA, complete cds
AT3G09035	-1.289318889	4.81E-17	Arabidopsis thaliana chromosome 3, complete sequence
AT3G50440	-1.023772799	4.83E-17	Arabidopsis thaliana methyl esterase 10 mRNA, complete cds
AT5G46280	-1.147096817	5.20E-17	Arabidopsis thaliana DNA replication licensing factor MCM3-like protein

			mRNA, complete cds
AT4G25940	-1.097576681	5.60E-17	Arabidopsis thaliana ENTH/ANTH/VHS superfamily protein mRNA, complete cds
AT3G26165	-1.097576681	5.60E-17	Arabidopsis thaliana chromosome 3, complete sequence
AT1G65610	-1.622307248	6.29E-17	Arabidopsis thaliana mRNA for hypothetical protein, complete cds, clone: RAFL16-65-K04
AT1G27460	-1.231105754	6.85E-17	Arabidopsis thaliana calmodulin-binding protein mRNA, complete cds
AT2G07690	-1.136162654	7.26E-17	Arabidopsis thaliana minichromosome maintenance protein 5 mRNA, complete cds
AT1G01750	-3.486946819	7.41E-17	Arabidopsis thaliana actin depolymerizing factor 11 mRNA, complete cds
AT2G31110	-1.525993724	7.85E-17	Arabidopsis thaliana protein TRICHOME BIREFRINGENCE-LIKE 40 mRNA, complete cds
AT5G16170	-1.814594075	8.29E-17	Arabidopsis thaliana core-2/I-branching beta-1,6-N-acetylglucosaminyltransferase family protein mRNA, complete cds
AT3G45930	-1.300447593	8.38E-17	Arabidopsis thaliana chromosome 3, complete sequence
AT3G28130	-1.01663073	8.66E-17	Arabidopsis thaliana WAT1-related protein mRNA, complete cds
AT5G40610	-1.062255028	8.67E-17	Arabidopsis thaliana glycerol-3-phosphate dehydrogenase [NAD+] mRNA, complete cds
AT1G52820	-2.180519818	8.97E-17	Arabidopsis thaliana putative 2-oxoglutarate-dependent dioxygenase mRNA, complete cds
AT5G50420	-1.207117171	9.02E-17	Arabidopsis thaliana O-fucosyltransferase family protein mRNA, complete cds
AT3G03130	-1.506549605	9.35E-17	Arabidopsis thaliana uncharacterized protein mRNA, complete cds
AT5G38550	-2.151753179	9.43E-17	Arabidopsis thaliana jacalin lectin family protein mRNA, complete cds
AT4G04745	-3.210791492	1.03E-16	Arabidopsis thaliana chromosome 4 sequence
AT3G48410	-1.678478277	1.04E-16	Arabidopsis thaliana alpha/beta-hydrolase domain-containing

			protein mRNA, complete cds
AT4G20390	-1.678478277	1.04E-16	Arabidopsis thaliana uncharacterized protein mRNA, complete cds
AT2G33400	-1.397581545	1.05E-16	Arabidopsis thaliana uncharacterized protein mRNA, complete cds
AT4G15160	-1.944928964	1.07E-16	Arabidopsis thaliana bifunctional inhibitor/lipid-transfer protein/seed storage 2S albumin-like protein mRNA, complete cds
AT3G62060	-1.442389785	1.18E-16	Arabidopsis thaliana pectinacetylesterase family protein mRNA, complete cds
AT5G41880	-1.820150647	1.35E-16	Arabidopsis thaliana DNA primase POLA3 mRNA, complete cds
AT4G14465	-1.108779885	1.40E-16	Arabidopsis thaliana chromosome 4 sequence
AT5G48920	-1.834870123	1.41E-16	Arabidopsis thaliana chromosome 5 sequence
AT1G52910	-1.475567757	1.45E-16	Arabidopsis thaliana uncharacterized protein mRNA, complete cds
AT1G33900	-3.841977009	1.47E-16	Arabidopsis thaliana P-loop containing nucleoside triphosphate hydrolases superfamily protein mRNA, complete cds
AT4G16350	-4.104198858	1.48E-16	Arabidopsis thaliana calcineurin B-like protein 6 mRNA, complete cds
AT3G60270	-4.104198858	1.48E-16	Arabidopsis thaliana cupredoxin superfamily protein mRNA, complete cds
AT5G59500	-1.035371734	1.49E-16	Arabidopsis thaliana chromosome 5 sequence
AT4G29450	-4.456952079	1.50E-16	Arabidopsis thaliana leucine-rich repeat protein kinase-like protein mRNA, complete cds
AT3G46270	-5.954451739	1.52E-16	Arabidopsis thaliana receptor protein kinase-like protein mRNA, complete cds
AT5G36120	-1.391607644	1.52E-16	Arabidopsis thaliana chromosome 5 sequence
AT1G12020	-1.327403837	1.53E-16	Arabidopsis thaliana chromosome 1 sequence
AT1G31160	-1.135314587	1.57E-16	Arabidopsis thaliana histidine triad nucleotide-binding 2 protein mRNA, complete cds
AT2G28930	-1.097754217	1.64E-16	Arabidopsis thaliana protein kinase APK1B mRNA, complete cds

AT2G01900	-2.761806661	1.74E-16	Arabidopsis thaliana DNAse I-like superfamily protein mRNA, complete cds
AT2G45270	-1.018248389	1.89E-16	Arabidopsis thaliana glycoprotease 1 mRNA, complete cds
AT3G49190	-2.691417333	2.21E-16	Arabidopsis thaliana O-acyltransferase (WSD1-like) family protein mRNA, complete cds
AT2G25735	-1.40470393	2.22E-16	Arabidopsis thaliana chromosome 2, complete sequence
AT5G63760	-1.211609578	2.62E-16	Arabidopsis thaliana putative E3 ubiquitin-protein ligase ARI15 mRNA, complete cds
AT3G52900	-1.095314274	3.05E-16	Arabidopsis thaliana uncharacterized protein mRNA, complete cds
AT1G52700	-1.788441787	3.09E-16	Arabidopsis thaliana alpha/beta-Hydrolases superfamily protein mRNA, complete cds
AT5G19790	-6.976819552	3.21E-16	Arabidopsis thaliana chromosome 5 sequence
AT5G39020	-1.30439421	3.38E-16	Arabidopsis thaliana chromosome 5 sequence
AT1G16500	-1.164486953	3.68E-16	Arabidopsis thaliana uncharacterized protein mRNA, complete cds
AT1G50580	-2.676917763	3.78E-16	Arabidopsis thaliana chromosome 1 sequence
AT4G36410	-1.609803567	3.87E-16	Arabidopsis thaliana putative ubiquitin-conjugating enzyme E2 17 mRNA, complete cds
AT4G26470	-1.740621331	3.99E-16	Arabidopsis thaliana putative calcium-binding protein CML21 mRNA, complete cds
AT5G19750	-1.091114286	4.15E-16	Arabidopsis thaliana Mpv17/PMP22 family protein mRNA, complete cds
AT2G44300	-1.038516003	4.45E-16	Arabidopsis thaliana bifunctional inhibitor/lipid-transfer protein/seed storage 2S albumin superfamily protein mRNA, complete cds
AT1G42480	-1.05858748	4.69E-16	Arabidopsis thaliana uncharacterized protein mRNA, complete cds
AT1G68110	-1.708291151	5.34E-16	Arabidopsis thaliana chromosome 1 sequence
AT4G30220	-1.053987412	5.42E-16	Arabidopsis thaliana small nuclear ribonucleoprotein F mRNA, complete

			cds
AT3G17640	-1.719986485	5.72E-16	Arabidopsis thaliana chromosome 3, complete sequence
AT5G07670	-1.039459764	6.08E-16	Arabidopsis thaliana RNI-like superfamily protein mRNA, complete cds
AT5G44040	-1.332963362	6.14E-16	Arabidopsis thaliana uncharacterized protein mRNA, complete cds
AT3G58100	-2.662270987	6.47E-16	Arabidopsis thaliana glucan endo-1,3-beta-glucosidase-like protein 1 mRNA, complete cds
AT1G55990	-1.44921643	6.68E-16	Arabidopsis thaliana glycine-rich protein mRNA, complete cds
AT3G55150	-2.055537864	6.83E-16	Arabidopsis thaliana chromosome 3, complete sequence
AT1G15000	-1.04490669	7.21E-16	Arabidopsis thaliana chromosome 1 sequence
AT5G42590	-1.432030704	7.67E-16	Arabidopsis thaliana cytochrome P450 71A16 mRNA, complete cds
AT2G16060	-1.784526737	7.79E-16	Arabidopsis thaliana non-symbiotic hemoglobin 1 mRNA, complete cds
AT1G44608	-4.04191458	8.57E-16	Arabidopsis thaliana uncharacterized protein mRNA, complete cds
AT3G45210	-1.012711137	8.65E-16	Arabidopsis thaliana chromosome 3, complete sequence
AT1G22690	-1.554364581	8.81E-16	Arabidopsis thaliana gibberellin-regulated protein 9 mRNA, complete cds
AT4G33640	-1.175220417	1.05E-15	Arabidopsis thaliana uncharacterized protein mRNA, complete cds
AT1G14080	-1.99752046	1.11E-15	Arabidopsis thaliana fucosyltransferase 6 mRNA, complete cds
AT2G29995	-1.504138765	1.14E-15	Arabidopsis thaliana uncharacterized protein mRNA, complete cds
AT1G30560	-5.861342334	1.29E-15	Arabidopsis thaliana chromosome 1 sequence
AT1G75140	-1.234559658	1.30E-15	Arabidopsis thaliana chromosome 1 sequence
AT5G62550	-1.085902592	1.42E-15	Arabidopsis thaliana uncharacterized protein mRNA, complete cds
AT3G61160	-1.490504639	1.50E-15	Arabidopsis thaliana shaggy-related protein kinase beta mRNA, complete cds
AT4G23550	-1.490504639	1.50E-15	Arabidopsis thaliana putative WRKY transcription factor 29 mRNA, complete

			cds
AT1G75190	-1.524767463	1.58E-15	Arabidopsis thaliana chromosome 1 sequence
AT5G46090	-2.142078742	1.58E-15	Arabidopsis thaliana chromosome 5 sequence
AT1G13430	-2.52520038	1.62E-15	Arabidopsis thaliana chromosome 1 sequence
AT3G14740	-2.343494029	1.67E-15	Arabidopsis thaliana RING/FYVE/PHD zinc finger-containing protein mRNA, complete cds
AT5G19700	-4.885189076	1.75E-15	Arabidopsis thaliana chromosome 5 sequence
AT1G51810	-4.885189076	1.75E-15	Arabidopsis thaliana putative LRR receptor-like serine/threonine protein kinase mRNA, complete cds
AT3G48080	-2.03367843	1.75E-15	Arabidopsis thaliana lipase class 3 family protein / disease resistance protein-related protein mRNA, complete cds
AT1G56720	-1.037867747	1.78E-15	Arabidopsis thaliana protein kinase mRNA, complete cds
AT3G04230	-1.331756591	1.88E-15	Arabidopsis thaliana chromosome 3, complete sequence
AT2G07340	-1.126698052	1.88E-15	Arabidopsis thaliana prefoldin 1 mRNA, complete cds
AT4G17810	-1.539414239	1.95E-15	Arabidopsis thaliana chromosome 4 sequence
AT1G10970	-1.578942604	1.96E-15	Arabidopsis thaliana zinc transporter 4 precursor mRNA, complete cds
AT5G10390	-1.407310703	2.08E-15	Arabidopsis thaliana chromosome 5 sequence
AT1G69240	-6.885189076	2.17E-15	Arabidopsis thaliana methyl esterase 15 mRNA, complete cds
AT4G16920	-1.706064109	2.20E-15	Arabidopsis thaliana TIR-NBS-LRR class disease resistance protein mRNA, complete cds
AT5G59670	-1.107463357	2.24E-15	Arabidopsis thaliana Leucine-rich repeat protein kinase family protein mRNA, complete cds
AT2G36110	-3.375915507	2.36E-15	Arabidopsis thaliana chromosome 2, complete sequence
AT2G23680	-1.267951211	2.38E-15	Arabidopsis thaliana Cold acclimation protein WCOR413 family mRNA, complete cds

AT4G03450	-2.101293127	2.65E-15	Arabidopsis thaliana ankyrin repeat-containing protein mRNA, complete cds
AT5G01610	-1.579603329	3.23E-15	Arabidopsis thaliana uncharacterized protein mRNA, complete cds
AT3G50470	-2.214979289	3.57E-15	Arabidopsis thaliana RPW8-like protein 3 mRNA, complete cds
AT2G18560	-1.030179065	3.76E-15	Arabidopsis thaliana chromosome 2, complete sequence
AT3G62780	-2.554364581	3.89E-15	Arabidopsis thaliana chromosome 3, complete sequence
AT4G22300	-1.190749762	4.00E-15	Arabidopsis thaliana carboxylesterase mRNA, complete cds
AT4G31730	-1.654891457	4.04E-15	Arabidopsis thaliana chromosome 4 sequence
AT5G59650	-1.606528435	4.29E-15	Arabidopsis thaliana probable LRR receptor-like serine/threonine-protein kinase PAM74 mRNA, complete cds
AT3G09020	-1.444107279	4.92E-15	Arabidopsis thaliana chromosome 3, complete sequence
AT5G39000	-3.976819552	4.98E-15	Arabidopsis thaliana chromosome 5 sequence
AT1G07560	-2.276379833	5.04E-15	Arabidopsis thaliana probable LRR receptor-like serine/threonine-protein kinase mRNA, complete cds
AT5G33355	-1.00281476	5.28E-15	Arabidopsis thaliana defensin-like protein mRNA, complete cds
AT3G10710	-3.088307485	5.42E-15	Arabidopsis thaliana root hair specific 12 mRNA, complete cds
AT5G41300	-2.887337543	6.05E-15	Arabidopsis thaliana cysteine-rich repeat secretory protein 59 mRNA, complete cds
AT3G10660	-1.45575631	6.24E-15	Arabidopsis thaliana Ccalcium-dependent protein kinase 2 mRNA, complete cds
AT1G47603	-2.13665507	6.47E-15	Arabidopsis thaliana purine permease 19 mRNA, complete cds
AT4G30420	-3.202379252	6.47E-15	Arabidopsis thaliana nodulin MtN21-like transporter family protein mRNA, complete cds
AT1G07690	-5.787341753	6.47E-15	Arabidopsis thaliana uncharacterized protein mRNA, complete cds
AT4G30320	-5.787341753	6.47E-15	Arabidopsis thaliana chromosome 4 sequence

AT5G57530	-5.787341753	6.47E-15	Arabidopsis thaliana probable xyloglucan endotransglucosylase/hydrolase protein 12 mRNA, complete cds
AT3G27970	-2.795753993	8.03E-15	Arabidopsis thaliana exonuclease-like protein mRNA, complete cds
AT5G44600	-1.501939534	8.44E-15	Arabidopsis thaliana S-adenosyl-L-methionine-dependent methyltransferase domain-containing protein mRNA, complete cds
AT4G24670	-1.008274438	8.66E-15	Arabidopsis thaliana tryptophan aminotransferase related 2 mRNA, complete cds
AT5G51310	-2.586719954	9.28E-15	Arabidopsis thaliana protein LONGER ROOT HAIRS UNDER PHOSPHATE-DEFICIENCY mRNA, complete cds
AT5G61000	-1.297935977	9.67E-15	Arabidopsis thaliana replication protein A 70 kDa DNA-binding subunit D mRNA, complete cds
AT5G35190	-4.812432734	9.84E-15	Arabidopsis thaliana chromosome 5 sequence
AT1G08990	-2.378949567	1.02E-14	Arabidopsis thaliana plant glycogenin-like starch initiation protein 5 mRNA, complete cds
AT3G21710	-1.125599881	1.03E-14	Arabidopsis thaliana uncharacterized protein mRNA, complete cds
AT5G49170	-2.713443639	1.03E-14	Arabidopsis thaliana uncharacterized protein mRNA, complete cds
AT5G01830	-1.385380611	1.04E-14	Arabidopsis thaliana chromosome 5 sequence
AT3G22930	-1.555624467	1.08E-14	Arabidopsis thaliana calmodulin-like protein 11 mRNA, complete cds
AT5G09820	-1.03340308	1.12E-14	Arabidopsis thaliana putative plastid-lipid-associated protein 7 mRNA, complete cds
AT2G30890	-1.988940115	1.14E-14	Arabidopsis thaliana Cytochrome b561/ferric reductase transmembrane protein family mRNA, complete cds
AT1G49390	-3.183270429	1.14E-14	Arabidopsis thaliana 2-oxoglutarate (2OG) and Fe(II)-dependent oxygenase-like protein mRNA, complete cds
AT4G34600	-1.07296621	1.22E-14	Arabidopsis thaliana uncharacterized protein mRNA, complete cds

AT1G19370	-1.098592714	1.23E-14	Arabidopsis thaliana chromosome 1 sequence
AT1G73620	-1.354199367	1.29E-14	Arabidopsis thaliana pathogenesis-related thaumatin-like protein mRNA, complete cds
AT1G55380	-3.317021818	1.32E-14	Arabidopsis thaliana chromosome 1 sequence
AT4G03330	-2.778880174	1.39E-14	Arabidopsis thaliana syntaxin-123 mRNA, complete cds
AT1G68150	-1.629051452	1.40E-14	Arabidopsis thaliana Group II-b WRKY transcription factor mRNA, complete cds
AT3G46320	-1.358450966	1.47E-14	Arabidopsis thaliana chromosome 3, complete sequence
AT3G02820	-1.639578401	1.52E-14	Arabidopsis thaliana CCHC-type zinc knuckle protein mRNA, complete cds
AT1G33340	-1.400011183	1.53E-14	Arabidopsis thaliana chromosome 1 sequence
AT1G44110	-1.362794585	1.67E-14	Arabidopsis thaliana cyclin-A1-1 mRNA, complete cds
AT5G10280	-1.515955267	1.73E-14	Arabidopsis thaliana myb domain protein 92 mRNA, complete cds
AT1G47655	-2.082207286	1.75E-14	Arabidopsis thaliana chromosome 1 sequence
AT5G60400	-1.162921784	1.76E-14	Arabidopsis thaliana uncharacterized protein mRNA, complete cds
AT4G29800	-2.053987412	1.79E-14	Arabidopsis thaliana PATATIN-like protein 8 mRNA, complete cds
AT2G39681	-1.146363553	1.84E-14	Arabidopsis thaliana chromosome 2, complete sequence
AT4G15330	-1.057211313	1.90E-14	Arabidopsis thaliana cytochrome P450, family 705, subfamily A, polypeptide 1 mRNA, complete cds
AT5G16480	-1.236222707	2.14E-14	Arabidopsis thaliana atypical dual-specificity phosphatase 5 mRNA, complete cds
AT4G26150	-1.046374228	2.16E-14	Arabidopsis thaliana putative GATA transcription factor 22 mRNA, complete cds
AT1G18970	-2.761806661	2.39E-14	Arabidopsis thaliana chromosome 1 sequence
AT2G48130	-1.582482961	2.39E-14	Arabidopsis thaliana protease inhibitor/seed storage/lipid transfer protein (LTP) family protein mRNA, complete cds

AT4G21903	-2.199074107	2.40E-14	Arabidopsis thaliana mate efflux domain-containing protein mRNA, complete cds
AT3G07200	-1.360444098	2.74E-14	Arabidopsis thaliana C3HC4 zinc finger domain-containing protein mRNA, complete cds
AT1G61840	-4.252132287	2.91E-14	Arabidopsis thaliana chromosome 1 sequence
AT5G17160	-1.053337403	2.99E-14	Arabidopsis thaliana uncharacterized protein mRNA, complete cds
AT3G44150	-1.010865048	3.04E-14	Arabidopsis thaliana uncharacterized protein mRNA, complete cds
AT5G27300	-1.07149116	3.07E-14	Arabidopsis thaliana pentatricopeptide (PPR) repeat-containing protein mRNA, complete cds
AT5G28630	-1.097815914	3.10E-14	Arabidopsis thaliana chromosome 5 sequence
AT3G02240	-4.761806661	3.13E-14	Arabidopsis thaliana root meristem growth factor 7 mRNA, complete cds
AT2G38940	-1.364916508	3.13E-14	Arabidopsis thaliana inorganic phosphate transporter 1-4 mRNA, complete cds
AT1G29430	-1.160902616	3.29E-14	Arabidopsis thaliana chromosome 1 sequence
AT1G51870	-5.709339241	3.30E-14	Arabidopsis thaliana putative serine/threonine protein kinase mRNA, complete cds
AT3G21340	-2.262574034	3.42E-14	Arabidopsis thaliana receptor-like protein kinase mRNA, complete cds
AT2G01120	-1.573971461	3.58E-14	Arabidopsis thaliana origin recognition complex subunit 4 mRNA, complete cds
AT4G11210	-1.258232487	3.60E-14	Arabidopsis thaliana chromosome 4 sequence
AT5G26660	-1.285860887	3.65E-14	Arabidopsis thaliana transcription factor MYB86 mRNA, complete cds
AT1G17090	-1.701003382	3.73E-14	Arabidopsis thaliana uncharacterized protein mRNA, complete cds
AT1G47395	-2.384439579	4.19E-14	Arabidopsis thaliana chromosome 1 sequence
AT3G14410	-1.092933692	4.23E-14	Arabidopsis thaliana Nucleotide/sugar transporter family protein mRNA, complete cds
AT1G54790	-1.592853498	4.35E-14	Arabidopsis thaliana GDSL esterase/lipase mRNA, complete cds
AT2G19970	-1.742043406	4.59E-14	Arabidopsis thaliana chromosome 2,

			complete sequence
AT4G29050	-1.772074996	5.18E-14	Arabidopsis thaliana chromosome 4 sequence
AT5G11690	-1.430889783	5.29E-14	Arabidopsis thaliana translocase inner membrane subunit 17-3 mRNA, complete cds
AT5G11150	-1.055989765	5.58E-14	Arabidopsis thaliana vesicle-associated membrane protein 713 mRNA, complete cds
AT4G04700	-1.492108525	5.60E-14	Arabidopsis thaliana calcium-dependent protein kinase 27 mRNA, complete cds
AT3G58550	-1.367145297	5.85E-14	Arabidopsis thaliana protease inhibitor/(LTP) family protein mRNA, complete cds
AT5G22870	-3.255621273	7.38E-14	Arabidopsis thaliana chromosome 5 sequence
AT1G33750	-1.42335611	7.71E-14	Arabidopsis thaliana terpenoid synthase 22 mRNA, complete cds
AT4G32830	-1.212763734	8.24E-14	Arabidopsis thaliana serine/threonine-protein kinase aurora-1 mRNA, complete cds
AT4G24660	-1.034491237	8.43E-14	Arabidopsis thaliana chromosome 4 sequence
AT4G18510	-2.276379833	8.54E-14	Arabidopsis thaliana chromosome 4 sequence
AT1G17960	-2.462246379	8.97E-14	Arabidopsis thaliana threonyl-tRNA synthetase mRNA, complete cds
AT3G21180	-2.462246379	8.97E-14	Arabidopsis thaliana calcium-transporting ATPase 9 mRNA, complete cds
AT3G46810	-3.609803567	9.04E-14	Arabidopsis thaliana chromosome 3, complete sequence
AT5G14100	-1.292867956	9.07E-14	Arabidopsis thaliana ABC transporter I family member 11 mRNA, complete cds
AT1G28620	-2.234559658	9.24E-14	Arabidopsis thaliana chromosome 1 sequence
AT5G59530	-1.694692465	9.38E-14	Arabidopsis thaliana 1-aminocyclopropane-1-carboxylate oxidase-like protein 11 mRNA, complete cds
AT3G23180	-1.694692465	9.38E-14	Arabidopsis thaliana HR-like lesion-inducing protein-like protein mRNA, complete cds
AT3G09790	-4.202379252	9.42E-14	Arabidopsis thaliana chromosome 3,

			complete sequence
AT5G40150	-1.462246379	9.62E-14	Arabidopsis thaliana chromosome 5 sequence
AT4G14750	-1.722005653	1.08E-13	Arabidopsis thaliana protein IQ-domain 19 mRNA, complete cds
AT1G04900	-1.369489238	1.10E-13	Arabidopsis thaliana uncharacterized protein mRNA, complete cds
AT1G22880	-1.029903967	1.13E-13	Arabidopsis thaliana cellulase 5 mRNA, complete cds
AT5G15120	-1.979113793	1.17E-13	Arabidopsis thaliana uncharacterized protein mRNA, complete cds
AT4G29430	-1.548131456	1.19E-13	Arabidopsis thaliana 40S ribosomal protein S15a-5 mRNA, complete cds
AT4G02800	-1.053305126	1.20E-13	Arabidopsis thaliana uncharacterized protein mRNA, complete cds
AT2G29130	-1.245352938	1.34E-13	Arabidopsis thaliana laccase 2 mRNA, complete cds
AT3G62760	-2.446304835	1.51E-13	Arabidopsis thaliana glutathione S-transferase F13 mRNA, complete cds
AT4G22520	-2.629051452	1.52E-13	Arabidopsis thaliana chromosome 4 sequence
AT1G14160	-2.974351296	1.57E-13	Arabidopsis thaliana uncharacterized protein mRNA, complete cds
AT1G66520	-1.836395977	1.60E-13	Arabidopsis thaliana protein pigment defective 194 mRNA, complete cds
AT5G49080.1	-4.682372193	1.78E-13	Arabidopsis thaliana chromosome 5 sequence
AT5G23990	-4.682372193	1.78E-13	Arabidopsis thaliana ferric reduction oxidase 5 mRNA, complete cds
AT2G01818	-1.33015109	1.91E-13	Arabidopsis thaliana PLATZ transcription factor family protein mRNA, complete cds
AT5G51490	-3.083734756	1.94E-13	Arabidopsis thaliana putative pectinesterase/pectinesterase inhibitor 59 mRNA, complete cds
AT3G45680	-1.595997768	1.96E-13	Arabidopsis thaliana putative nitrate excretion transporter 3 mRNA, complete cds
AT4G38340	-2.691417333	2.09E-13	Arabidopsis thaliana nodule inception protein-like protein 3 mRNA, complete cds
AT1G49000	-1.419744009	2.09E-13	Arabidopsis thaliana chromosome 1 sequence
AT4G01910	-1.67492921	2.18E-13	Arabidopsis thaliana chromosome 4

			sequence
AT5G15980	-1.043719077	2.19E-13	Arabidopsis thaliana chromosome 5 sequence
AT4G31810	-1.049829787	2.22E-13	Arabidopsis thaliana ATP-dependent caseinolytic (Clp) protease/crotonase family protein mRNA, complete cds
AT3G19400	-1.015651895	2.33E-13	Arabidopsis thaliana putative cysteine proteinase mRNA, complete cds
AT3G59680	-1.406147708	2.69E-13	Arabidopsis thaliana uncharacterized protein mRNA, complete cds
AT2G35270	-1.323096332	2.73E-13	Arabidopsis thaliana chromosome 2, complete sequence
AT3G12685	-1.167181063	2.84E-13	Arabidopsis thaliana acid phosphatase/vanadium-dependent haloperoxidase-related protein mRNA, complete cds
AT4G37530	-1.05117651	3.03E-13	Arabidopsis thaliana peroxidase 51 mRNA, complete cds
AT3G07900	-4.150848951	3.05E-13	Arabidopsis thaliana O-fucosyltransferase-like protein mRNA, complete cds
AT5G26320	-4.654891457	3.19E-13	Arabidopsis thaliana TRAF-like family protein mRNA, complete cds
AT4G17280	-1.022015022	3.26E-13	Arabidopsis thaliana putative auxin-responsive protein mRNA, complete cds
AT5G05990	-1.664945121	3.31E-13	Arabidopsis thaliana mitochondrial glycoprotein family protein mRNA, complete cds
AT3G02120	-1.513419031	3.51E-13	Arabidopsis thaliana hydroxyproline-rich glycoprotein-like protein mRNA, complete cds
AT3G17350	-1.238244705	3.54E-13	Arabidopsis thaliana uncharacterized protein mRNA, complete cds
AT1G70990	-2.473825898	3.68E-13	Arabidopsis thaliana chromosome 1 sequence
AT4G36470	-1.283233505	3.94E-13	Arabidopsis thaliana S-adenosyl-L-methionine-dependent methyltransferase-like protein mRNA, complete cds
AT5G02090	-1.458342629	4.32E-13	Arabidopsis thaliana chromosome 5 sequence
AT5G22410	-6.598307928	4.76E-13	Arabidopsis thaliana peroxidase 60 mRNA, complete cds

AT3G02650	-1.127186274	4.89E-13	Arabidopsis thaliana pentatricopeptide repeat-containing protein mRNA, complete cds
AT4G14980	-3.539414239	5.19E-13	Arabidopsis thaliana chromosome 4 sequence
AT4G10510	-3.539414239	5.19E-13	Arabidopsis thaliana Subtilase family protein mRNA, complete cds
AT1G03457	-1.047561143	5.58E-13	Arabidopsis thaliana RNA recognition motif-containing protein mRNA, complete cds
AT1G33870	-4.626877081	5.71E-13	Arabidopsis thaliana P-loop containing nucleoside triphosphate hydrolases superfamily protein mRNA, complete cds
AT2G02740	-1.234559658	5.75E-13	Arabidopsis thaliana single-stranded DNA-binding protein WHY3 mRNA, complete cds
AT4G14830	-2.261113077	5.81E-13	Arabidopsis thaliana chromosome 4 sequence
AT1G54770	-1.054715862	6.61E-13	Arabidopsis thaliana Fcf2 pre-rRNA processing protein mRNA, complete cds
AT5G45700	-1.840794957	6.61E-13	Arabidopsis thaliana chromosome 5 sequence
AT4G23770	-2.17684416	6.64E-13	Arabidopsis thaliana uncharacterized protein mRNA, complete cds
AT1G34760	-1.929360758	7.45E-13	Arabidopsis thaliana 14-3-3-like protein GF14 omicron mRNA, complete cds
AT4G30610	-1.122957463	7.81E-13	Arabidopsis thaliana serine carboxypeptidase 24 mRNA, complete cds
AT2G31270	-1.114586237	7.88E-13	Arabidopsis thaliana CDT1-like protein a mRNA, complete cds
AT1G35320	-1.076130295	7.91E-13	Arabidopsis thaliana uncharacterized protein mRNA, complete cds
AT2G13550	-1.588657758	8.01E-13	Arabidopsis thaliana chromosome 2, complete sequence
AT3G49640	-1.024841066	8.36E-13	Arabidopsis thaliana aldolase-type TIM barrel family protein mRNA, complete cds
AT2G33560	-1.548812937	8.88E-13	Arabidopsis thaliana BUB1-related protein BUBR1 mRNA, complete cds
AT2G38620	-1.548812937	8.88E-13	Arabidopsis thaliana cyclin-dependent kinase B1-2 mRNA, complete cds
AT4G38420	-1.115915161	9.18E-13	Arabidopsis thaliana protein SKU5

			similar 9 mRNA, complete cds
AT2G02950	-1.107697999	9.24E-13	Arabidopsis thaliana chromosome 2, complete sequence
AT3G49750	-1.413883357	9.36E-13	Arabidopsis thaliana chromosome 3, complete sequence
AT4G14380	-3.761806661	9.79E-13	Arabidopsis thaliana chromosome 4 sequence
AT1G24450	-1.346769161	1.00E-12	Arabidopsis thaliana nuclear fusion defective 2 protein mRNA, complete cds
AT3G50350	-1.153095557	1.01E-12	Arabidopsis thaliana uncharacterized protein mRNA, complete cds
AT4G32605	-1.207217809	1.02E-12	Arabidopsis thaliana mitochondrial glycoprotein family protein mRNA, complete cds
AT1G05310	-1.369489238	1.04E-12	Arabidopsis thaliana putative pectinesterase 8 mRNA, complete cds
AT1G63450	-2.636275779	1.05E-12	Arabidopsis thaliana chromosome 1 sequence
AT5G38820	-3.020540929	1.05E-12	Arabidopsis thaliana putative amino acid transporter mRNA, complete cds
AT1G55420	-3.020540929	1.05E-12	Arabidopsis thaliana chromosome 1 sequence
AT3G48550	-3.020540929	1.05E-12	Arabidopsis thaliana uncharacterized protein mRNA, complete cds
AT3G14260	-1.393873397	1.06E-12	Arabidopsis thaliana LURP-one-related 11 protein mRNA, complete cds
AT2G29730	-1.100895307	1.08E-12	Arabidopsis thaliana chromosome 2, complete sequence
AT5G41920	-1.019925576	1.12E-12	Arabidopsis thaliana chromosome 5 sequence
AT4G15830	-1.29452718	1.13E-12	Arabidopsis thaliana ARM repeat superfamily protein mRNA, complete cds
AT2G27970	-1.455512982	1.18E-12	Arabidopsis thaliana cyclin-dependent kinases regulatory subunit 2 mRNA, complete cds
AT5G43910	-1.209708794	1.18E-12	Arabidopsis thaliana pfkB-like carbohydrate kinase family protein mRNA, complete cds
AT1G33770	-2.055989765	1.20E-12	Arabidopsis thaliana protein kinase family protein mRNA, complete cds
AT5G38610	-1.313854539	1.20E-12	Arabidopsis thaliana chromosome 5 sequence
AT2G05160	-1.136202175	1.22E-12	Arabidopsis thaliana zinc finger CCCH

			domain-containing protein 18 mRNA, complete cds
AT3G25110	-1.003361339	1.22E-12	Arabidopsis thaliana fatA acyl-ACP thioesterase mRNA, complete cds
AT3G12270	-1.127288335	1.24E-12	Arabidopsis thaliana protein arginine N-methyltransferase 3 mRNA, complete cds
AT2G32280	-1.462792958	1.32E-12	Arabidopsis thaliana uncharacterized protein mRNA, complete cds
AT5G01330	-1.462792958	1.32E-12	Arabidopsis thaliana pyruvate decarboxylase-3 mRNA, complete cds
AT5G56080	-2.704473486	1.43E-12	Arabidopsis thaliana chromosome 5 sequence
AT2G19670	-1.302375042	1.49E-12	Arabidopsis thaliana protein arginine N-methyltransferase 1 mRNA, complete cds
AT2G25220	-1.511933503	1.59E-12	Arabidopsis thaliana protein kinase family protein mRNA, complete cds
AT2G01860	-1.612345762	1.59E-12	Arabidopsis thaliana chromosome 2, complete sequence
AT5G20470	-3.490504639	1.66E-12	Arabidopsis thaliana putative myosin mRNA, complete cds
AT4G24265	-1.903825666	1.86E-12	Arabidopsis thaliana chromosome 4 sequence
AT1G05210	-1.903825666	1.86E-12	Arabidopsis thaliana putative transmembrane protein 97 mRNA, complete cds
AT3G47050	-2.782270763	1.93E-12	Arabidopsis thaliana glycosyl hydrolase family protein mRNA, complete cds
AT4G01390	-2.011035267	1.94E-12	Arabidopsis thaliana TRAF-like family protein mRNA, complete cds
AT1G14440	-1.194278753	1.98E-12	Arabidopsis thaliana homeobox protein 31 mRNA, complete cds
AT2G01080	-1.074507491	2.00E-12	Arabidopsis thaliana late embryogenesis abundant hydroxyproline-rich glycoprotein mRNA, complete cds
AT2G06925	-1.353721922	2.15E-12	Arabidopsis thaliana phospholipase A2-alpha mRNA, complete cds
AT4G22756	-1.248634843	2.23E-12	Arabidopsis thaliana sterol C4-methyl oxidase 1-2 mRNA, complete cds
AT1G10400	-3.12437674	2.25E-12	Arabidopsis thaliana UDP-glycosyltransferase 90A2 mRNA, complete cds
AT1G12530	-1.06810852	2.32E-12	Arabidopsis thaliana uncharacterized

			protein mRNA, complete cds
AT3G20130	-1.298981262	2.43E-12	Arabidopsis thaliana cytochrome P450, family 705, subfamily A, polypeptide 22 mRNA, complete cds
AT5G59520	-2.684844679	2.46E-12	Arabidopsis thaliana zinc transporter 2 mRNA, complete cds
AT2G23560	-1.613961193	2.63E-12	Arabidopsis thaliana methyl esterase 7 mRNA, complete cds
AT3G18170	-1.061785783	2.69E-12	Arabidopsis thaliana chromosome 3, complete sequence
AT4G10530	-5.478013695	2.69E-12	Arabidopsis thaliana Subtilase family protein mRNA, complete cds
AT2G46740	-1.187765446	2.76E-12	Arabidopsis thaliana D-arabinono-1,4-lactone oxidase-like protein mRNA, complete cds
AT3G21950	-1.706524225	2.84E-12	Arabidopsis thaliana methyltransferase mRNA, complete cds
AT3G18900	-1.89088661	2.93E-12	Arabidopsis thaliana uncharacterized protein mRNA, complete cds
AT5G14000	-1.89088661	2.93E-12	Arabidopsis thaliana NAC domain containing protein 84 mRNA, complete cds
AT1G29418	-1.484966455	3.10E-12	Arabidopsis thaliana chromosome 1 sequence
AT3G48210	-1.08515543	3.20E-12	Arabidopsis thaliana kinetochore Spc25 domain-containing protein mRNA, complete cds
AT4G20450	-4.04191458	3.22E-12	Arabidopsis thaliana putative LRR receptor-like serine/threonine-protein kinase mRNA, complete cds
AT5G04150	-4.04191458	3.22E-12	Arabidopsis thaliana transcription factor bHLH101 mRNA, complete cds
AT2G32620	-2.976819552	3.22E-12	Arabidopsis thaliana cellulose synthase-like protein B2 mRNA, complete cds
AT4G28190	-1.39502433	3.25E-12	Arabidopsis thaliana protein ULTRAPETALA 1 mRNA, complete cds
AT4G32860	-1.580636902	3.28E-12	Arabidopsis thaliana chromosome 4 sequence
AT5G52180	-1.017187494	3.30E-12	Arabidopsis thaliana chromosome 5 sequence
AT4G37060	-2.761806661	3.33E-12	Arabidopsis thaliana PATATIN-like protein 5 mRNA, complete cds
AT4G27270	-1.328266575	3.39E-12	Arabidopsis thaliana Quinone reductase

			family protein mRNA, complete cds
AT4G28430	-1.755906059	3.47E-12	Arabidopsis thaliana reticulon-like protein B18 mRNA, complete cds
AT1G15125	-1.459979772	3.61E-12	Arabidopsis thaliana S-adenosyl-L-methionine-dependent methyltransferase mRNA, complete cds
AT3G26818	-2.293253652	3.64E-12	Arabidopsis thaliana chromosome 3, complete sequence
AT3G51280	-1.050103978	4.21E-12	Arabidopsis thaliana tetratricopeptide repeat domain-containing protein mRNA, complete cds
AT3G55646	-1.121349047	4.30E-12	Arabidopsis thaliana uncharacterized protein mRNA, complete cds
AT5G58520	-1.071808689	4.33E-12	Arabidopsis thaliana protein kinase family protein mRNA, complete cds
AT5G07130	-1.559734224	4.45E-12	Arabidopsis thaliana laccase 13 mRNA, complete cds
AT3G22240	-2.1560856	4.46E-12	Arabidopsis thaliana uncharacterized protein mRNA, complete cds
AT5G19560	-5.446304835	4.70E-12	Arabidopsis thaliana ROP uanine nucleotide exchange factor 10 mRNA, complete cds
AT3G03000	-1.413883357	4.75E-12	Arabidopsis thaliana chromosome 3, complete sequence
AT4G00020	-1.209265638	4.81E-12	Arabidopsis thaliana breast cancer protein 2 like 2A mRNA, complete cds
AT5G24070	-2.045599627	4.89E-12	Arabidopsis thaliana probable peroxidase 61 mRNA, complete cds
AT1G13830	-2.013345428	4.94E-12	Arabidopsis thaliana carbohydrate-binding X8 domain-containing protein mRNA, complete cds
AT5G01870	-1.197308263	5.00E-12	Arabidopsis thaliana pathogenesis-related lipid transfer protein mRNA, complete cds
AT4G19380	-2.578942604	5.18E-12	Arabidopsis thaliana Long-chain-alcohol oxidase FAO4A mRNA, complete cds
AT5G28626.1	-1.529566453	5.38E-12	Arabidopsis thaliana chromosome 5 sequence
AT2G21840	-1.49241676	5.73E-12	Arabidopsis thaliana Cysteine/Histidine-rich C1 domain family protein mRNA, complete cds
AT1G22740	-1.347794167	5.81E-12	Arabidopsis thaliana GTP-binding protein Rab7 mRNA, complete cds

AT5G01740	-2.501939534	6.18E-12	Arabidopsis thaliana chromosome 5 sequence
AT4G03010	-1.605002581	6.56E-12	Arabidopsis thaliana chromosome 4 sequence
AT1G80270	-1.067080594	6.86E-12	Arabidopsis thaliana pentatricopeptide repeat-containing protein mRNA, complete cds
AT3G54430	-1.09915675	6.93E-12	Arabidopsis thaliana SHI-related sequence 6 mRNA, complete cds
AT3G03500	-1.560172799	7.35E-12	Arabidopsis thaliana TatD related DNase mRNA, complete cds
AT2G38810	-1.168047212	7.38E-12	Arabidopsis thaliana histone H2A 8 mRNA, complete cds
AT4G30860	-1.519650985	7.99E-12	Arabidopsis thaliana histone-lysine N-methyltransferase ASHR3 mRNA, complete cds
AT1G13670	-1.749631947	8.77E-12	Arabidopsis thaliana chromosome 1 sequence
AT2G43110	-1.119700253	9.34E-12	Arabidopsis thaliana uncharacterized protein mRNA, complete cds
AT1G63550	-2.931731662	9.84E-12	Arabidopsis thaliana Receptor-like protein kinase-related family protein mRNA, complete cds
AT3G07000	-1.787341753	9.85E-12	Arabidopsis thaliana chromosome 3, complete sequence
AT1G24620	-3.654891457	1.02E-11	Arabidopsis thaliana chromosome 1 sequence
AT5G06839	-1.161414119	1.02E-11	Arabidopsis thaliana bZIP transcription factor TGA10 mRNA, complete cds
AT1G68185	-1.19800877	1.09E-11	Arabidopsis thaliana ubiquitin-related protein mRNA, complete cds
AT4G00955	-1.34993644	1.09E-11	Arabidopsis thaliana chromosome 4 sequence
AT3G13160	-1.008491578	1.11E-11	Arabidopsis thaliana chromosome 3, complete sequence
AT3G51710	-1.464936023	1.11E-11	Arabidopsis thaliana D-mannose binding lectin protein with Apple-like carbohydrate-binding domain mRNA, complete cds
AT5G06790	-1.272527508	1.14E-11	Arabidopsis thaliana chromosome 5 sequence
AT4G25990	-1.619267547	1.19E-11	Arabidopsis thaliana CIA2-like transcription factor mRNA, complete cds
AT2G25240	-2.624303137	1.24E-11	Arabidopsis thaliana serpin CCP3

			mRNA, complete cds
AT1G72220	-2.624303137	1.24E-11	Arabidopsis thaliana chromosome 1 sequence
AT1G33930	-2.624303137	1.24E-11	Arabidopsis thaliana P-loop containing nucleoside triphosphate hydrolases superfamily protein mRNA, complete cds
AT1G22065	-1.473180541	1.25E-11	Arabidopsis thaliana chromosome 1 sequence
AT1G70470	-1.203272285	1.46E-11	Arabidopsis thaliana chromosome 1 sequence
AT1G24485	-3.202379252	1.48E-11	Arabidopsis thaliana uncharacterized protein mRNA, complete cds
AT1G49310	-1.07190932	1.49E-11	Arabidopsis thaliana uncharacterized protein mRNA, complete cds
AT4G26990	-1.423221222	1.66E-11	Arabidopsis thaliana uncharacterized protein mRNA, complete cds
AT5G28919	-1.04262755	1.67E-11	Arabidopsis thaliana chromosome 5 sequence
AT4G38830	-2.698612834	1.70E-11	Arabidopsis thaliana cysteine-rich receptor-like protein kinase 26 mRNA, complete cds
AT1G14430	-1.691417333	1.80E-11	Arabidopsis thaliana glyoxal oxidase-related protein mRNA, complete cds
AT1G28040	-3.626877081	1.83E-11	Arabidopsis thaliana RING-H2 finger protein ATL20 mRNA, complete cds
AT1G48460	-1.372442555	1.85E-11	Arabidopsis thaliana uncharacterized protein mRNA, complete cds
AT1G18290	-3.954451739	1.89E-11	Arabidopsis thaliana chromosome 1 sequence
AT1G57980	-1.886064764	1.90E-11	Arabidopsis thaliana Nucleotide-sugar transporter family protein mRNA, complete cds
AT5G06900	-4.446304835	1.91E-11	Arabidopsis thaliana cytochrome P450, family 93, subfamily D, polypeptide 1 mRNA, complete cds
AT4G13890	-2.034178931	2.00E-11	Arabidopsis thaliana serine hydroxymethyltransferase 5 mRNA, complete cds
AT5G03350	-1.352211246	2.05E-11	Arabidopsis thaliana chromosome 5 sequence
AT4G01740	-1.471850956	2.06E-11	Arabidopsis thaliana cysteine/histidine-rich C1

			domain-containing protein mRNA, complete cds
AT2G36690	-2.784526737	2.29E-11	Arabidopsis thaliana 2-oxoglutarate (2OG) and Fe(II)-dependent oxygenase superfamily protein mRNA, complete cds
AT2G20390	-1.051476193	2.32E-11	Arabidopsis thaliana uncharacterized protein mRNA, complete cds
AT3G53850	-1.24225405	2.41E-11	Arabidopsis thaliana uncharacterized protein mRNA, complete cds
AT2G15042	-1.781615142	2.50E-11	Arabidopsis thaliana Leucine-rich repeat (LRR) family protein mRNA, complete cds
AT1G63600	-5.346769161	2.53E-11	Arabidopsis thaliana chromosome 1 sequence
AT3G01760	-3.17684416	2.61E-11	Arabidopsis thaliana Lysine histidine transporter-like 4 mRNA, complete cds
AT5G05400	-1.802448645	2.64E-11	Arabidopsis thaliana chromosome 5 sequence
AT3G02790	-1.044837975	2.68E-11	Arabidopsis thaliana chromosome 3, complete sequence
AT1G08280	-1.319448555	2.81E-11	Arabidopsis thaliana chromosome 1 sequence
AT1G73640	-1.695533441	2.97E-11	Arabidopsis thaliana ras-related protein RABA6a mRNA, complete cds
AT5G09978	-2.885189076	2.99E-11	Arabidopsis thaliana elicitor peptide 7 mRNA, complete cds
AT3G53235	-3.360444098	3.02E-11	Arabidopsis thaliana uncharacterized protein mRNA, complete cds
AT1G44020	-3.360444098	3.02E-11	Arabidopsis thaliana cysteine/histidine-rich C1 domain-containing protein mRNA, complete cds
AT1G07070	-1.39768613	3.09E-11	Arabidopsis thaliana 60S ribosomal protein L35a-1 mRNA, complete cds
AT3G12240	-2.018582076	3.21E-11	Arabidopsis thaliana serine carboxypeptidase-like 15 mRNA, complete cds
AT1G10030	-1.103595178	3.26E-11	Arabidopsis thaliana Ergosterol biosynthetic protein 28-like protein mRNA, complete cds
AT4G30120	-3.598307928	3.29E-11	Arabidopsis thaliana putative inactive cadmium/zinc-transporting ATPase HMA3 mRNA, complete cds

AT1G16930	-1.191490936	3.30E-11	Arabidopsis thaliana F-box/RNI-like/FBD-like domains-containing protein mRNA, complete cds
AT3G24210	-1.561609985	3.33E-11	Arabidopsis thaliana Ankyrin repeat family protein mRNA, complete cds
AT3G22210	-1.348983582	3.36E-11	Arabidopsis thaliana uncharacterized protein mRNA, complete cds
AT5G65040	-1.268342514	3.50E-11	Arabidopsis thaliana uncharacterized protein mRNA, complete cds
AT2G27770	-1.167445462	3.50E-11	Arabidopsis thaliana chromosome 2, complete sequence
AT2G43200	-1.518352624	3.62E-11	Arabidopsis thaliana S-adenosyl-L-methionine-dependent methyltransferases superfamily protein mRNA, complete cds
AT2G42170	-1.748867605	3.65E-11	Arabidopsis thaliana actin family protein mRNA, complete cds
AT2G34000	-3.005077812	3.79E-11	Arabidopsis thaliana chromosome 2, complete sequence
AT5G24910	-3.005077812	3.79E-11	Arabidopsis thaliana cytochrome P450, family 714, subfamily A, polypeptide 1 mRNA, complete cds
AT1G22030	-1.078280325	3.80E-11	Arabidopsis thaliana uncharacterized protein mRNA, complete cds
AT3G10190	-1.354631279	3.84E-11	Arabidopsis thaliana chromosome 3, complete sequence
AT5G13150	-2.761806661	3.95E-11	Arabidopsis thaliana chromosome 5 sequence
AT2G44010	-2.258232487	4.11E-11	Arabidopsis thaliana chromosome 2, complete sequence
AT4G31320	-1.158198407	4.17E-11	Arabidopsis thaliana chromosome 4 sequence
AT4G01890	-5.312003743	4.44E-11	Arabidopsis thaliana glycoside hydrolase family 28 protein / polygalacturonase (pectinase) family protein mRNA, complete cds
AT3G46240	-5.312003743	4.44E-11	Arabidopsis thaliana uncharacterized protein mRNA, complete cds
AT4G31600	-1.184186135	4.60E-11	Arabidopsis thaliana UDP-galactose transporter 7 mRNA, complete cds
AT2G37780	-6.312003743	4.60E-11	Arabidopsis thaliana chromosome 2, complete sequence
AT2G28710	-2.159566169	4.67E-11	Arabidopsis thaliana C2H2-type zinc

			finger-containing protein mRNA, complete cds
AT1G60095	-2.159566169	4.67E-11	Arabidopsis thaliana jacalin-like lectin domain-containing protein mRNA, complete cds
AT4G37340	-2.037867747	5.11E-11	Arabidopsis thaliana cytochrome P450, family 81, subfamily D, polypeptide 3 mRNA, complete cds
AT5G07780	-2.00281476	5.15E-11	Arabidopsis thaliana formin-like protein 19 mRNA, complete cds
AT3G56230	-2.356550182	5.63E-11	Arabidopsis thaliana BTB/POZ domain-containing protein mRNA, complete cds
AT5G43070	-1.162559934	5.64E-11	Arabidopsis thaliana chromosome 5 sequence
AT1G29450	-1.21594398	5.76E-11	Arabidopsis thaliana chromosome 1 sequence
AT5G23300	-1.056632133	5.94E-11	Arabidopsis thaliana dihydroorotate dehydrogenase mRNA, complete cds
AT4G09990	-1.75514293	5.99E-11	Arabidopsis thaliana uncharacterized protein mRNA, complete cds
AT3G07510	-1.099882178	6.07E-11	Arabidopsis thaliana uncharacterized protein mRNA, complete cds
AT3G50130	-3.893051194	6.14E-11	Arabidopsis thaliana uncharacterized protein mRNA, complete cds
AT4G22080	-4.380716493	6.20E-11	Arabidopsis thaliana root hair specific 14 mRNA, complete cds
AT3G06740	-1.233427688	6.37E-11	Arabidopsis thaliana GATA transcription factor 15 mRNA, complete cds
AT5G35732	-2.239853957	6.78E-11	Arabidopsis thaliana chromosome 5 sequence
AT1G79470	-1.192030564	7.20E-11	Arabidopsis thaliana inosine-5'-monophosphate dehydrogenase mRNA, complete cds
AT1G06790	-1.416355843	7.45E-11	Arabidopsis thaliana RNA polymerase Rpb7 N-terminal domain-containing protein mRNA, complete cds
AT5G25840	-1.167112622	7.63E-11	Arabidopsis thaliana chromosome 5 sequence
AT4G16850	-1.144054225	7.96E-11	Arabidopsis thaliana uncharacterized protein mRNA, complete cds
AT1G44160	-1.704473486	8.05E-11	Arabidopsis thaliana HSP40/DnaJ peptide-binding protein mRNA, complete cds

AT1G26570	-1.102731746	8.28E-11	Arabidopsis thaliana UDP-glucose dehydrogenase 1 mRNA, complete cds
AT5G67450	-1.392260546	8.41E-11	Arabidopsis thaliana chromosome 5 sequence
AT4G08780	-1.392260546	8.41E-11	Arabidopsis thaliana peroxidase 38 mRNA, complete cds
AT1G14185	-1.627641422	8.87E-11	Arabidopsis thaliana glucose-methanol-choline oxidoreductase-like protein mRNA, complete cds
AT2G13910	-2.336921375	9.38E-11	Arabidopsis thaliana chromosome 2, complete sequence
AT1G71180	-1.135023984	9.41E-11	Arabidopsis thaliana chromosome 1 sequence
AT3G20960	-1.19756545	9.70E-11	Arabidopsis thaliana cytochrome P450, family 705, subfamily A, polypeptide 33 mRNA, complete cds
AT4G35030	-1.322183523	9.94E-11	Arabidopsis thaliana salt stress-related protein kinase mRNA, complete cds
AT4G35380	-1.280679971	1.01E-10	Arabidopsis thaliana SEC7-like guanine nucleotide exchange family protein mRNA, complete cds
AT5G18030	-1.22881415	1.04E-10	Arabidopsis thaliana chromosome 5 sequence
AT4G03935	-1.303179893	1.08E-10	Arabidopsis thaliana Full-length cDNA Complete sequence from clone GSLTSIL43ZE01 of Silique of strain col-0 of Arabidopsis thaliana (thale cress)
AT5G26270	-4.346769161	1.12E-10	Arabidopsis thaliana uncharacterized protein mRNA, complete cds
AT5G06270	-1.187112495	1.17E-10	Arabidopsis thaliana uncharacterized protein mRNA, complete cds
AT3G09960	-2.954451739	1.17E-10	Arabidopsis thaliana calcineurin-like metallo-phosphoesterase-like protein mRNA, complete cds
AT5G38540	-2.715264075	1.18E-10	Arabidopsis thaliana Mannose-binding lectin superfamily protein mRNA, complete cds
AT2G32960	-1.308088693	1.24E-10	Arabidopsis thaliana atypical dual-specicity phosphatase mRNA, complete cds
AT4G40070	-1.203365036	1.31E-10	Arabidopsis thaliana chromosome 4 sequence
AT1G47590	-5.239853957	1.38E-10	Arabidopsis thaliana chromosome 1

			sequence
AT4G32340	-1.220731804	1.45E-10	Arabidopsis thaliana tetratricopeptide repeat domain-containing protein-like protein mRNA, complete cds
AT2G40700	-1.038016455	1.46E-10	Arabidopsis thaliana DEAD-box ATP-dependent RNA helicase 17 mRNA, complete cds
AT1G64910	-2.609803567	1.47E-10	Arabidopsis thaliana chromosome 1 sequence
AT5G58390	-1.255318218	1.52E-10	Arabidopsis thaliana peroxidase mRNA, complete cds
AT2G43940	-1.11908244	1.53E-10	Arabidopsis thaliana putative thiol methyltransferase 2 mRNA, complete cds
AT4G20780	-1.11908244	1.53E-10	Arabidopsis thaliana chromosome 4 sequence
AT4G27730	-1.079982621	1.54E-10	Arabidopsis thaliana oligopeptide transporter mRNA, complete cds
AT5G11750	-1.079982621	1.54E-10	Arabidopsis thaliana ribosomal protein L19 family protein mRNA, complete cds
AT5G59380	-1.098841648	1.54E-10	Arabidopsis thaliana mRNA for hypothetical protein, complete cds, clone: RAFL14-16-G11
AT1G22590	-1.179418104	1.62E-10	Arabidopsis thaliana protein AGAMOUS-like 87 mRNA, complete cds
AT1G08340	-1.276379833	1.65E-10	Arabidopsis thaliana Rho GTPase activating protein with PAK-box/P21-Rho-binding domain mRNA, complete cds
AT2G45830	-1.023613763	1.65E-10	Arabidopsis thaliana downstream target of AGL15 2 mRNA, complete cds
AT5G50560	-2.517387933	1.76E-10	Arabidopsis thaliana chromosome 5 sequence
AT4G14390	-2.517387933	1.76E-10	Arabidopsis thaliana ankyrin repeat-containing protein mRNA, complete cds
AT2G40690	-1.110369882	1.79E-10	Arabidopsis thaliana glycerol-3-phosphate dehydrogenase [NAD(+)] 2 mRNA, complete cds
AT4G13310	-1.678241944	1.88E-10	Arabidopsis thaliana cytochrome P450 71A20 mRNA, complete cds
AT1G55390	-3.50904059	1.90E-10	Arabidopsis thaliana chromosome 1 sequence
AT2G23410	-3.828920857	2.00E-10	Arabidopsis thaliana

			cis-prenyltransferase mRNA, complete cds
AT2G44340	-4.312003743	2.02E-10	Arabidopsis thaliana chromosome 2, complete sequence
AT4G39720	-1.695918725	2.03E-10	Arabidopsis thaliana chromosome 4 sequence
AT5G05180	-1.212603923	2.04E-10	Arabidopsis thaliana uncharacterized protein mRNA, complete cds
AT3G44510	-2.92845653	2.04E-10	Arabidopsis thaliana alpha/beta-hydrolases family protein mRNA, complete cds
AT5G62730	-2.435578428	2.06E-10	Arabidopsis thaliana probable peptide/nitrate transporter mRNA, complete cds
AT4G08410	-6.202379252	2.19E-10	Arabidopsis thaliana chromosome 4 sequence
AT2G29060	-1.30911662	2.33E-10	Arabidopsis thaliana scarecrow-like protein 34 mRNA, complete cds
AT1G11740	-5.202379252	2.43E-10	Arabidopsis thaliana ankyrin repeat family protein mRNA, complete cds
AT5G05640	-2.237385702	2.82E-10	Arabidopsis thaliana chromosome 5 sequence
AT5G42770	-1.085035848	2.87E-10	Arabidopsis thaliana Maf-like protein mRNA, complete cds
AT5G07610	-1.369489238	2.91E-10	Arabidopsis thaliana chromosome 5 sequence
AT1G16060	-1.018582076	3.04E-10	Arabidopsis thaliana AP2-like ethylene-responsive transcription factor mRNA, complete cds
AT1G63160	-1.319736203	3.07E-10	Arabidopsis thaliana replication factor C 2 mRNA, complete cds
AT3G52170	-1.033886206	3.16E-10	Arabidopsis thaliana DNA binding protein mRNA, complete cds
AT1G78650	-1.346769161	3.22E-10	Arabidopsis thaliana DNA polymerase delta 3 mRNA, complete cds
AT2G18650	-1.906623214	3.26E-10	Arabidopsis thaliana chromosome 2, complete sequence
AT5G52710	-1.937963616	3.32E-10	Arabidopsis thaliana copper transport family protein mRNA, complete cds
AT1G27420	-2.007563075	3.36E-10	Arabidopsis thaliana putative F-box/kelch-repeat protein mRNA, complete cds
AT3G50160	-3.478013695	3.41E-10	Arabidopsis thaliana uncharacterized protein mRNA, complete cds

AT3G45330	-2.667169787	3.48E-10	Arabidopsis thaliana chromosome 3, complete sequence
AT5G01490	-1.242028329	3.49E-10	Arabidopsis thaliana vacuolar cation/proton exchanger 4 mRNA, complete cds
AT1G56080	-1.325289434	3.52E-10	Arabidopsis thaliana uncharacterized protein mRNA, complete cds
AT5G40310	-3.795753993	3.61E-10	Arabidopsis thaliana exonuclease family protein mRNA, complete cds
AT1G14880	-1.166370829	3.63E-10	Arabidopsis thaliana cadmium resistance protein 1 mRNA, complete cds
AT2G47360	-1.619584588	3.67E-10	Arabidopsis thaliana chromosome 2, complete sequence
AT3G43790	-1.551692569	3.74E-10	Arabidopsis thaliana probable peptide/nitrate transporter mRNA, complete cds
AT2G34260	-1.141654731	3.78E-10	Arabidopsis thaliana protein WDR55 mRNA, complete cds
AT1G01940	-1.118838557	3.88E-10	Arabidopsis thaliana peptidyl-prolyl cis-trans isomerase-like 3 mRNA, complete cds
AT1G12060	-1.33101509	4.04E-10	Arabidopsis thaliana BCL-2-associated athanogene 5 mRNA, complete cds
AT3G25130	-1.33101509	4.04E-10	Arabidopsis thaliana chromosome 3, complete sequence
AT4G32840	-1.019743199	4.17E-10	Arabidopsis thaliana 6-phosphofructokinase 6 mRNA, complete cds
AT4G15417	-2.563260981	4.27E-10	Arabidopsis thaliana protein RNAse II-like 1 mRNA, complete cds
AT1G08500	-1.1560856	4.33E-10	Arabidopsis thaliana early nodulin-like protein 18 mRNA, complete cds
AT2G41780	-1.784526737	4.34E-10	Arabidopsis thaliana chromosome 2, complete sequence
AT2G27470	-1.214095555	4.44E-10	Arabidopsis thaliana nuclear factor Y, subunit B11 mRNA, complete cds
AT3G30214	-1.577888387	4.58E-10	Arabidopsis thaliana chromosome 3, complete sequence
AT2G14210	-2.163905104	4.92E-10	Arabidopsis thaliana protein agamous-like 44 mRNA, complete cds
AT5G06550	-1.271792564	5.07E-10	Arabidopsis thaliana HR demethylase-like protein mRNA, complete cds
AT1G06520	-1.271792564	5.07E-10	Arabidopsis thaliana

			sn-glycerol-3-phosphate 2-O-acyltransferase mRNA, complete cds
AT5G42780	-1.686755955	5.13E-10	Arabidopsis thaliana chromosome 5 sequence
AT2G04650	-1.145983196	5.14E-10	Arabidopsis thaliana ADP-glucose pyrophosphorylase-like protein mRNA, complete cds
AT1G68238	-1.990075648	5.38E-10	Arabidopsis thaliana chromosome 1 sequence
AT5G20550	-1.442117038	5.50E-10	Arabidopsis thaliana oxidoreductase, 2OG-Fe(II) oxygenase family protein mRNA, complete cds
AT5G43040	-1.606528435	5.54E-10	Arabidopsis thaliana chromosome 5 sequence
AT1G68730	-1.187941869	5.55E-10	Arabidopsis thaliana Zim17-type zinc finger protein mRNA, complete cds
AT4G18590	-1.02094715	5.72E-10	Arabidopsis thaliana nucleic acid-binding, OB-fold-like protein mRNA, complete cds
AT2G16230	-2.642507732	5.97E-10	Arabidopsis thaliana O-glycosyl hydrolases family 17 protein mRNA, complete cds
AT3G48185	-1.492871653	5.98E-10	Arabidopsis thaliana chromosome 3, complete sequence
AT3G01660	-1.136058545	6.08E-10	Arabidopsis thaliana S-adenosylmethionine-dependent methyltransferase domain-containing protein mRNA, complete cds
AT4G15300	-3.446304835	6.12E-10	Arabidopsis thaliana cytochrome P450, family 702, subfamily A, polypeptide 2 mRNA, complete cds
AT5G16900	-1.747009659	6.34E-10	Arabidopsis thaliana putative LRR receptor-like serine/threonine-protein kinase mRNA, complete cds
AT4G22380	-1.006446376	6.40E-10	Arabidopsis thaliana ribosomal protein L7Ae/L30e/S12e/Gadd45 family protein mRNA, complete cds
AT1G35330	-3.761806661	6.50E-10	Arabidopsis thaliana RING-H2 finger protein ATL34 mRNA, complete cds
AT1G49100	-4.239853957	6.57E-10	Arabidopsis thaliana putative leucine-rich repeat protein kinase mRNA, complete cds
AT4G02180	-4.239853957	6.57E-10	Arabidopsis thaliana DC1

			domain-containing protein mRNA, complete cds
AT5G65925	-1.240974497	6.58E-10	Arabidopsis thaliana chromosome 5 sequence
AT2G21850	-1.046194307	7.05E-10	Arabidopsis thaliana Cysteine/Histidine-rich C1 domain family protein mRNA, complete cds
AT3G58850	-1.793170832	7.12E-10	Arabidopsis thaliana chromosome 3, complete sequence
AT4G15680	-1.515166693	7.47E-10	Arabidopsis thaliana chromosome 4 sequence
AT3G10040	-1.515166693	7.47E-10	Arabidopsis thaliana chromosome 3, complete sequence
AT3G23930	-1.818390189	7.48E-10	Arabidopsis thaliana uncharacterized protein mRNA, complete cds
AT4G18250	-1.179626601	7.73E-10	Arabidopsis thaliana putative receptor serine/threonine kinase mRNA, complete cds
AT1G10990	-1.67268077	7.84E-10	Arabidopsis thaliana chromosome 1 sequence
AT1G62422	-1.030219246	7.99E-10	Arabidopsis thaliana chromosome 1 sequence
AT5G53730	-1.228212551	8.00E-10	Arabidopsis thaliana chromosome 5 sequence
AT3G61100	-1.152865297	8.12E-10	Arabidopsis thaliana putative endonuclease / glycosyl hydrolase mRNA, complete cds
AT2G37090	-1.055989765	8.35E-10	Arabidopsis thaliana probable beta-1,4-xylosyltransferase IRX9 mRNA, complete cds
AT5G13470	-1.249072487	8.80E-10	Arabidopsis thaliana uncharacterized protein mRNA, complete cds
AT1G11700	-1.480997553	8.83E-10	Arabidopsis thaliana chromosome 1 sequence
AT5G61660	-1.182479695	8.98E-10	Arabidopsis thaliana chromosome 5 sequence
AT2G27775	-1.369489238	9.01E-10	Arabidopsis thaliana uncharacterized protein mRNA, complete cds
AT1G29600	-1.539414239	9.26E-10	Arabidopsis thaliana putative zinc finger CCCH domain-containing protein 10 mRNA, complete cds
AT2G29740	-1.732059317	9.77E-10	Arabidopsis thaliana chromosome 2, complete sequence
AT5G18210	-1.411090142	1.04E-09	Arabidopsis thaliana Rossmann-fold

			NAD(P)-binding domain-containing protein mRNA, complete cds
AT1G68740	-1.023493383	1.07E-09	Arabidopsis thaliana phosphate transporter PHO1-1 mRNA, complete cds
AT1G07550	-2.29840614	1.08E-09	Arabidopsis thaliana putative LRR receptor-like serine/threonine-protein kinase mRNA, complete cds
AT5G37210	-3.413883357	1.10E-09	Arabidopsis thaliana cysteine/histidine-rich C1 domain-containing protein mRNA, complete cds
AT3G59890	-1.132369531	1.14E-09	Arabidopsis thaliana dihydrodipicolinate reductase 2 mRNA, complete cds
AT1G01390	-1.301470814	1.18E-09	Arabidopsis thaliana chromosome 1 sequence
AT5G59270	-4.202379252	1.19E-09	Arabidopsis thaliana concanavalin A-like lectin protein kinase family protein mRNA, complete cds
AT4G18430	-1.281262055	1.27E-09	Arabidopsis thaliana RAB GTPase homolog A1E mRNA, complete cds
AT5G24390	-1.134486768	1.33E-09	Arabidopsis thaliana RabGAP/TBC domain-containing protein mRNA, complete cds
AT4G19820	-2.076442263	1.35E-09	Arabidopsis thaliana Glycosyl hydrolase family protein with chitinase insertion domain mRNA, complete cds
AT4G09110	-5.083734756	1.35E-09	Arabidopsis thaliana chromosome 4 sequence
AT1G24240	-1.26211914	1.36E-09	Arabidopsis thaliana ribosomal protein L19 family protein mRNA, complete cds
AT4G28310	-1.306968153	1.36E-09	Arabidopsis thaliana chromosome 4 sequence
AT4G17860	-2.984199082	1.39E-09	Arabidopsis thaliana uncharacterized protein mRNA, complete cds
AT1G17560	-1.716952425	1.50E-09	Arabidopsis thaliana protein HUELLENLOS mRNA, complete cds
AT4G04810	-1.437534625	1.51E-09	Arabidopsis thaliana peptide methionine sulfoxide reductase B4 mRNA, complete cds
AT5G18250	-1.226704674	1.51E-09	Arabidopsis thaliana uncharacterized protein mRNA, complete cds
AT2G02590	-1.399862887	1.51E-09	Arabidopsis thaliana uncharacterized protein mRNA, complete cds

AT4G31360	-1.318505309	1.79E-09	Arabidopsis thaliana selenium binding protein mRNA, complete cds
AT3G11110	-1.102974263	1.85E-09	Arabidopsis thaliana chromosome 3, complete sequence
AT2G14660	-1.456952079	1.92E-09	Arabidopsis thaliana chromosome 2, complete sequence
AT5G18020	-1.416794953	1.95E-09	Arabidopsis thaliana chromosome 5 sequence
AT5G07650	-1.840280719	2.00E-09	Arabidopsis thaliana Actin-binding FH2 protein mRNA, complete cds
AT4G05130	-2.1560856	2.04E-09	Arabidopsis thaliana equilibrative nucleoside transporter 4 mRNA, complete cds
AT3G53220	-3.691417333	2.12E-09	Arabidopsis thaliana thioredoxin-like 3-3 mRNA, complete cds
AT4G28230	-1.354989668	2.15E-09	Arabidopsis thaliana chromosome 4 sequence
AT1G67630	-1.072448948	2.16E-09	Arabidopsis thaliana DNA polymerase alpha 2 mRNA, complete cds
AT1G54450	-1.093515776	2.17E-09	Arabidopsis thaliana calcium-binding EF-hand-containing protein mRNA, complete cds
AT1G26200	-2.056813456	2.18E-09	Arabidopsis thaliana TRAM, LAG1 and CLN8 (TLC) lipid-sensing domain containing protein mRNA, complete cds
AT1G67910	-2.013345428	2.21E-09	Arabidopsis thaliana uncharacterized protein mRNA, complete cds
AT5G07390	-1.257014509	2.22E-09	Arabidopsis thaliana Respiratory burst oxidase-A mRNA, complete cds
AT5G60590	-1.143485563	2.45E-09	Arabidopsis thaliana DHBP synthase RibB-like alpha/beta domain-containing protein mRNA, complete cds
AT2G38460	-1.106454832	2.53E-09	Arabidopsis thaliana protein RON REGULATED1 mRNA, complete cds
AT2G24645	-1.539414239	2.55E-09	Arabidopsis thaliana transcriptional factor B3 family protein mRNA, complete cds
AT5G49780	-1.745865117	2.65E-09	Arabidopsis thaliana leucine-rich repeat protein kinase-like protein mRNA, complete cds
AT1G70460	-2.323685548	2.71E-09	Arabidopsis thaliana proline-rich extensin-like receptor kinase 13 mRNA, complete cds
AT5G12920	-2.565886451	2.99E-09	Arabidopsis thaliana WD40

			domain-containing protein mRNA, complete cds
AT1G48640	-2.565886451	2.99E-09	Arabidopsis thaliana transmembrane amino acid transporter family protein mRNA, complete cds
AT4G37235	-3.12437674	3.01E-09	Arabidopsis thaliana CASP-like protein mRNA, complete cds
AT1G35230	-1.500940092	3.05E-09	Arabidopsis thaliana chromosome 1 sequence
AT4G01970	-1.500940092	3.05E-09	Arabidopsis thaliana stachyose synthase mRNA, complete cds
AT1G33890	-5.998845858	3.10E-09	Arabidopsis thaliana avirulence induced protein AIG1 mRNA, complete cds
AT5G19800	-5.998845858	3.10E-09	Arabidopsis thaliana chromosome 5 sequence
AT5G43590	-5.998845858	3.10E-09	Arabidopsis thaliana acyl transferase/acyl hydrolase/lysophospholipase-like protein mRNA, complete cds
AT5G14150	-2.191490936	3.17E-09	Arabidopsis thaliana uncharacterized protein mRNA, complete cds
AT2G22570	-1.148331472	3.33E-09	Arabidopsis thaliana nicotinamidase 1 mRNA, complete cds
AT5G11540	-1.320101211	3.38E-09	Arabidopsis thaliana L-gulono-1,4-lactone (L-GulL) oxidase 3 mRNA, complete cds
AT3G52480	-1.135023984	3.38E-09	Arabidopsis thaliana chromosome 3, complete sequence
AT5G42785	-3.346769161	3.51E-09	Arabidopsis thaliana uncharacterized protein mRNA, complete cds
AT4G30640	-3.346769161	3.51E-09	Arabidopsis thaliana RNI-like superfamily protein mRNA, complete cds
AT1G05370	-2.036913899	3.52E-09	Arabidopsis thaliana Sec14p-like phosphatidylinositol transfer family protein mRNA, complete cds
AT4G29180	-1.954451739	3.52E-09	Arabidopsis thaliana protein root hair specific 16 mRNA, complete cds
AT4G13330	-1.598307928	3.81E-09	Arabidopsis thaliana S-adenosylmethionine-dependent methyltransferase domain-containing protein mRNA, complete cds
AT5G66330	-1.707358877	3.81E-09	Arabidopsis thaliana chromosome 5 sequence
AT5G09290	-1.326420516	3.88E-09	Arabidopsis thaliana Inositol

			monophosphatase family protein mRNA, complete cds
AT3G15620	-1.08875283	4.05E-09	Arabidopsis thaliana (6-4)DNA photolyase mRNA, complete cds
AT4G16265	-1.39333598	4.13E-09	Arabidopsis thaliana RNA polymerases subunit NRP(B/D/E)9b mRNA, complete cds
AT3G20090	-1.25634208	4.18E-09	Arabidopsis thaliana cytochrome P450, family 705, subfamily A, polypeptide 18 mRNA, complete cds
AT1G30650	-1.167855377	4.42E-09	Arabidopsis thaliana putative WRKY transcription factor 14 mRNA, complete cds
AT3G45390	-2.300226575	4.50E-09	Arabidopsis thaliana concanavalin A-like lectin kinase-like protein mRNA, complete cds
AT4G04630	-1.101882102	4.73E-09	Arabidopsis thaliana chromosome 4 sequence
AT1G56630	-2.231291944	4.89E-09	Arabidopsis thaliana alpha/beta-Hydrolases superfamily protein mRNA, complete cds
AT2G48030	-1.186182025	4.99E-09	Arabidopsis thaliana endonuclease/exonuclease/phosphatase domain-containing protein mRNA, complete cds
AT5G38110	-1.021914706	5.12E-09	Arabidopsis thaliana histone chaperone ASF1B mRNA, complete cds
AT3G06140	-1.021914706	5.12E-09	Arabidopsis thaliana probable E3 ubiquitin-protein ligase LUL4 mRNA, complete cds
AT5G62865	-1.1560856	5.26E-09	Arabidopsis thaliana uncharacterized protein mRNA, complete cds
AT1G13970	-1.452702606	5.27E-09	Arabidopsis thaliana uncharacterized protein mRNA, complete cds
AT5G66985	-3.091955262	5.32E-09	Arabidopsis thaliana chromosome 5 sequence
AT1G22230	-1.049608972	5.39E-09	Arabidopsis thaliana chromosome 1 sequence
AT3G20460	-2.113650333	5.43E-09	Arabidopsis thaliana putative sugar transporter ERD6-like 13 mRNA, complete cds
AT4G34770	-2.062976195	5.58E-09	Arabidopsis thaliana chromosome 4 sequence
AT3G07130	-1.005590588	5.73E-09	Arabidopsis thaliana purple acid

			phosphatase 15 mRNA, complete cds
AT2G02610	-2.439878566	5.97E-09	Arabidopsis thaliana chromosome 2, complete sequence
AT1G67035	-3.312003743	6.27E-09	Arabidopsis thaliana uncharacterized protein mRNA, complete cds
AT5G24313	-3.312003743	6.27E-09	Arabidopsis thaliana chromosome 5 sequence
AT3G28650	-3.312003743	6.27E-09	Arabidopsis thaliana chromosome 3, complete sequence
AT5G46230	-1.429789748	6.84E-09	Arabidopsis thaliana chromosome 5 sequence
AT1G02630	-3.617416751	6.88E-09	Arabidopsis thaliana equilibrative nucleotide transporter 8 mRNA, complete cds
AT4G33730	-3.617416751	6.88E-09	Arabidopsis thaliana chromosome 4 sequence
AT4G26560	-4.083734756	7.04E-09	Arabidopsis thaliana calcineurin B-like protein 7 mRNA, complete cds
AT1G19960	-2.626877081	7.17E-09	Arabidopsis thaliana chromosome 1 sequence
AT4G33790	-1.04191458	7.28E-09	Arabidopsis thaliana fatty acyl-CoA reductase CER4 mRNA, complete cds
AT2G43140	-1.276379833	7.43E-09	Arabidopsis thaliana transcription factor bHLH129 mRNA, complete cds
AT5G18690	-1.083734756	7.55E-09	Arabidopsis thaliana chromosome 5 sequence
AT2G42110	-1.635629555	7.58E-09	Arabidopsis thaliana uncharacterized protein mRNA, complete cds
AT5G35870	-4.954451739	7.65E-09	Arabidopsis thaliana chromosome 5 sequence
AT5G08350	-1.255621273	7.90E-09	Arabidopsis thaliana GRAM domain-containing protein / ABA-responsive protein-related mRNA, complete cds
AT2G43050	-2.208208331	8.06E-09	Arabidopsis thaliana putative pectinesterase/ pectinerase inhibitor 16 mRNA, complete cds
AT5G07900	-1.14970803	8.49E-09	Arabidopsis thaliana chromosome 5 sequence
AT1G66020	-1.569161583	8.63E-09	Arabidopsis thaliana terpenoid synthase 26 mRNA, complete cds
AT2G23690	-2.512447192	8.65E-09	Arabidopsis thaliana uncharacterized protein mRNA, complete cds
AT2G45890	-1.369489238	8.70E-09	Arabidopsis thaliana mRNA for

			hypothetical protein, complete cds, clone: RAFL16-63-K09
AT1G29030	-1.063228085	8.73E-09	Arabidopsis thaliana Apoptosis inhibitory protein 5 mRNA, complete cds
AT4G15960	-1.217486144	8.74E-09	Arabidopsis thaliana alpha/beta-Hydrolases superfamily protein mRNA, complete cds
AT5G23903	-5.908648049	9.11E-09	Arabidopsis thaliana uncharacterized protein mRNA, complete cds
AT2G16250	-1.260554866	9.13E-09	Arabidopsis thaliana putative LRR receptor-like serine/threonine-protein kinase mRNA, complete cds
AT4G37660	-1.287435022	9.87E-09	Arabidopsis thaliana chromosome 4 sequence
AT3G19430	-1.377663169	9.92E-09	Arabidopsis thaliana late embryogenesis abundant protein-like protein mRNA, complete cds
AT5G08360	-2.413883357	1.01E-08	Arabidopsis thaliana uncharacterized protein mRNA, complete cds
AT2G20590	-1.52520038	1.05E-08	Arabidopsis thaliana reticulon-like protein B17 mRNA, complete cds
AT5G28650	-2.327910134	1.13E-08	Arabidopsis thaliana WRKY transcription factor 74 mRNA, complete cds
AT2G01755	-1.04490669	1.17E-08	Arabidopsis thaliana chromosome 2, complete sequence
AT1G78865	-1.140548031	1.17E-08	Arabidopsis thaliana chromosome 1 sequence
AT3G59765	-1.140548031	1.17E-08	Arabidopsis thaliana Full-length cDNA Complete sequence from clone GSLTSIL90ZF11 of Silique of strain col-0 of Arabidopsis thaliana (thale cress)
AT1G49160	-1.065812848	1.20E-08	Arabidopsis thaliana putative serine/threonine-protein kinase WNK7 mRNA, complete cds
AT3G32047	-3.578942604	1.24E-08	Arabidopsis thaliana cytochrome P450 superfamily protein mRNA, complete cds
AT5G64816	-1.39502433	1.29E-08	Arabidopsis thaliana uncharacterized protein mRNA, complete cds
AT1G08810	-1.249907622	1.29E-08	Arabidopsis thaliana putative transcription factor MYB60 mRNA, complete cds
AT1G52800	-2.184749358	1.33E-08	Arabidopsis thaliana oxidoreductase,

			2OG-Fe(II) oxygenase family protein mRNA, complete cds
AT1G66620	-1.861342334	1.36E-08	Arabidopsis thaliana E3 ubiquitin-protein ligase SINA-like 2 mRNA, complete cds
AT3G12870	-1.211360042	1.42E-08	Arabidopsis thaliana chromosome 3, complete sequence
AT2G44370	-1.36538484	1.44E-08	Arabidopsis thaliana chromosome 2, complete sequence
AT3G05727	-1.934273857	1.44E-08	Arabidopsis thaliana defensin-like protein 204 mRNA, complete cds
AT5G23190	-1.193639402	1.48E-08	Arabidopsis thaliana cytochrome P450 86B1 mRNA, complete cds
AT2G46860	-5.861342334	1.57E-08	Arabidopsis thaliana pyrophosphorylase 3 mRNA, complete cds
AT4G22630	-5.861342334	1.57E-08	Arabidopsis thaliana bifunctional inhibitor/lipid-transfer protein/seed storage 2S albumin superfamily protein mRNA, complete cds
AT3G27400	-1.131329501	1.62E-08	Arabidopsis thaliana putative pectate lyase 11 mRNA, complete cds
AT1G28710	-1.091955262	1.65E-08	Arabidopsis thaliana nucleotide-diphospho-sugar transferase family protein mRNA, complete cds
AT5G11180	-3.024841066	1.66E-08	Arabidopsis thaliana glutamate receptor 2.6 mRNA, complete cds
AT3G52460	-1.727041243	1.72E-08	Arabidopsis thaliana chromosome 3, complete sequence
AT1G75250	-1.524767463	1.74E-08	Arabidopsis thaliana protein RADIALIS-like 6 mRNA, complete cds
AT5G42580	-1.180164518	1.77E-08	Arabidopsis thaliana cytochrome P450, family 705, subfamily A, polypeptide 12 mRNA, complete cds
AT5G09320	-1.345241692	1.82E-08	Arabidopsis thaliana vacuolar sorting protein 9 (VPS9) domain protein mRNA, complete cds
AT1G17480	-1.622830248	1.91E-08	Arabidopsis thaliana protein IQ-domain 7 mRNA, complete cds
AT4G22110	-2.569161583	2.10E-08	Arabidopsis thaliana GroES-like zinc-binding dehydrogenase family protein mRNA, complete cds
AT3G53010	-2.569161583	2.10E-08	Arabidopsis thaliana uncharacterized protein mRNA, complete cds
AT5G55170	-1.841977009	2.14E-08	Arabidopsis thaliana small ubiquitin-related modifier 3 mRNA,

			complete cds
AT1G05300	-1.049229963	2.20E-08	Arabidopsis thaliana zinc transporter 5 mRNA, complete cds
AT3G03060	-1.136290062	2.20E-08	Arabidopsis thaliana P-loop containing nucleoside triphosphate hydrolases superfamily protein mRNA, complete cds
AT3G05140	-1.876449227	2.22E-08	Arabidopsis thaliana receptor-like cytosolic serine/threonine-protein kinase RBK2 mRNA, complete cds
AT4G02075	-1.07136949	2.24E-08	Arabidopsis thaliana protein pitchoun 1 mRNA, complete cds
AT5G53320	-1.913809754	2.28E-08	Arabidopsis thaliana putative inactive receptor kinase mRNA, complete cds
AT4G25560	-1.954451739	2.32E-08	Arabidopsis thaliana transcription factor LAF1 mRNA, complete cds
AT1G52660	-4.861342334	2.45E-08	Arabidopsis thaliana probable disease resistance protein mRNA, complete cds
AT1G02575	-4.861342334	2.45E-08	Arabidopsis thaliana uncharacterized protein mRNA, complete cds
AT1G33880	-4.861342334	2.45E-08	Arabidopsis thaliana avirulence-induced protein mRNA, complete cds
AT2G15300	-1.300226575	2.47E-08	Arabidopsis thaliana leucine-rich repeat protein kinase-like protein mRNA, complete cds
AT2G23030	-1.050376159	2.58E-08	Arabidopsis thaliana serine/threonine-protein kinase SNRK2.9 mRNA, complete cds
AT4G15690	-1.509970461	2.59E-08	Arabidopsis thaliana chromosome 4 sequence
AT4G28950	-1.509970461	2.59E-08	Arabidopsis thaliana Rac-like GTP-binding protein ARAC7 mRNA, complete cds
AT4G01920	-1.58832384	2.63E-08	Arabidopsis thaliana chromosome 4 sequence
AT4G13300	-1.276379833	2.66E-08	Arabidopsis thaliana (Z)-gamma-bisabolene synthase 2 mRNA, complete cds
AT5G54030	-5.812432734	2.71E-08	Arabidopsis thaliana chromosome 5 sequence
AT1G51260	-5.812432734	2.71E-08	Arabidopsis thaliana 1-acyl-sn-glycerol-3-phosphate acyltransferase 3 mRNA, complete cds
AT1G11920	-5.812432734	2.71E-08	Arabidopsis thaliana putative pectate

			lyase mRNA, complete cds
AT4G32780	-1.306753483	2.84E-08	Arabidopsis thaliana phosphoinositide binding protein mRNA, complete cds
AT1G25430.1	-1.606528435	2.89E-08	Arabidopsis thaliana chromosome 1 sequence
AT1G11735	-2.990075648	2.93E-08	Arabidopsis thaliana chromosome 1 sequence
AT2G14920	-2.990075648	2.93E-08	Arabidopsis thaliana sulfotransferase 4A mRNA, complete cds
AT3G26320	-2.990075648	2.93E-08	Arabidopsis thaliana cytochrome P450 71B36 mRNA, complete cds
AT2G15040	-1.761806661	3.02E-08	Arabidopsis thaliana chromosome 2, complete sequence
AT3G62160	-1.126767287	3.04E-08	Arabidopsis thaliana HXXXD-type acyl-transferase-like protein mRNA, complete cds
AT3G23630	-1.790953006	3.20E-08	Arabidopsis thaliana chromosome 3, complete sequence
AT4G13130	-1.822348203	3.36E-08	Arabidopsis thaliana chromosome 4 sequence
AT1G17240	-1.822348203	3.36E-08	Arabidopsis thaliana chromosome 1 sequence
AT5G13250	-1.481698741	3.43E-08	Arabidopsis thaliana RING finger protein mRNA, complete cds
AT1G45110	-1.856271345	3.50E-08	Arabidopsis thaliana tetrapyrrole (corrin/porphyrin)methylase mRNA, complete cds
AT4G27400	-1.555355783	3.57E-08	Arabidopsis thaliana late embryogenesis abundant protein-like protein mRNA, complete cds
AT1G70860	-2.539414239	3.59E-08	Arabidopsis thaliana SRPBCC ligand-binding domain-containing protein mRNA, complete cds
AT3G48490	-2.077834154	3.68E-08	Arabidopsis thaliana uncharacterized protein mRNA, complete cds
AT1G03620	-1.572204174	3.96E-08	Arabidopsis thaliana ELMO/CED-12 family protein mRNA, complete cds
AT4G09770	-2.795753993	3.97E-08	Arabidopsis thaliana TRAF-like family protein mRNA, complete cds
AT2G27740	-1.29468065	4.07E-08	Arabidopsis thaliana uncharacterized protein mRNA, complete cds
AT5G08185	-1.065483051	4.18E-08	Arabidopsis thaliana microRNA162A mRNA, complete cds
AT1G05530	-2.428382927	4.20E-08	Arabidopsis thaliana chromosome 1

			sequence
AT2G26840	-1.103315125	4.21E-08	Arabidopsis thaliana uncharacterized protein mRNA, complete cds
AT2G18735	-1.50904059	4.31E-08	Arabidopsis thaliana chromosome 2, complete sequence
AT1G44010	-5.761806661	4.68E-08	Arabidopsis thaliana uncharacterized protein mRNA, complete cds
AT3G05480	-2.332963362	4.74E-08	Arabidopsis thaliana DNA damage repair protein RAD9 mRNA, complete cds
AT3G45840	-2.332963362	4.74E-08	Arabidopsis thaliana Cysteine/Histidine-rich C1 domain family protein mRNA, complete cds
AT5G26010	-1.608955173	4.80E-08	Arabidopsis thaliana putative protein phosphatase 2C 72 mRNA, complete cds
AT2G18520	-1.523817384	4.81E-08	Arabidopsis thaliana chromosome 2, complete sequence
AT1G62360	-1.119510985	4.89E-08	Arabidopsis thaliana homeobox protein SHOOT MERISTEMLESS mRNA, complete cds
AT1G80720	-1.276379833	5.03E-08	Arabidopsis thaliana mitochondrial glycoprotein family protein mRNA, complete cds
AT1G70260	-1.46690174	5.07E-08	Arabidopsis thaliana nodulin MtN21-like transporter UMAMIT36 mRNA, complete cds
AT1G66930	-1.539414239	5.35E-08	Arabidopsis thaliana protein kinase family protein mRNA, complete cds
AT5G59940	-1.835807242	5.51E-08	Arabidopsis thaliana chromosome 5 sequence
AT1G14220	-2.17684416	5.55E-08	Arabidopsis thaliana ribonuclease T2 mRNA, complete cds
AT5G54720	-1.068407928	5.73E-08	Arabidopsis thaliana chromosome 5 sequence
AT5G59240	-2.111993016	5.81E-08	Arabidopsis thaliana 40S ribosomal protein S8-2 mRNA, complete cds
AT3G57157	-1.673269986	6.23E-08	Arabidopsis thaliana chromosome 3, complete sequence
AT1G23120	-1.673269986	6.23E-08	Arabidopsis thaliana polyketide cyclase/dehydrase and lipid transport superfamily protein mRNA, complete cds
AT3G23740	-1.351787236	6.48E-08	Arabidopsis thaliana uncharacterized protein mRNA, complete cds
AT4G30800	-1.139806781	6.58E-08	Arabidopsis thaliana 40S ribosomal

			protein S11-2 mRNA, complete cds
AT2G39675	-1.215215971	6.84E-08	Arabidopsis thaliana chromosome 2, complete sequence
AT1G67470	-1.036393352	7.58E-08	Arabidopsis thaliana chromosome 1 sequence
AT5G38300	-1.295488656	7.70E-08	Arabidopsis thaliana chromosome 5 sequence
AT1G16220	-1.084332021	7.88E-08	Arabidopsis thaliana putative protein phosphatase 2C 6 mRNA, complete cds
AT5G54050	-4.761806661	7.91E-08	Arabidopsis thaliana cysteine/histidine-rich C1 domain-containing protein mRNA, complete cds
AT5G04230	-1.219796305	7.94E-08	Arabidopsis thaliana phenylalanine ammonia-lyase 3 mRNA, complete cds
AT1G27670	-2.086902035	9.45E-08	Arabidopsis thaliana chromosome 1 sequence
AT5G46040	-1.654891457	9.52E-08	Arabidopsis thaliana peptide transporter PTR3-B mRNA, complete cds
AT4G14640	-2.029739866	9.63E-08	Arabidopsis thaliana calmodulin-like protein 8 mRNA, complete cds
AT5G56780	-1.016993205	1.00E-07	Arabidopsis thaliana effector of transcription2 mRNA, complete cds
AT2G26360	-1.492108525	1.06E-07	Arabidopsis thaliana mitochondrial substrate carrier family protein mRNA, complete cds
AT3G18970	-1.704473486	1.11E-07	Arabidopsis thaliana mitochondrial editing factor 20 mRNA, complete cds
AT3G26130	-3.12437674	1.13E-07	Arabidopsis thaliana Cellulase (glycosyl hydrolase family 5) protein mRNA, complete cds
AT3G12540	-3.12437674	1.13E-07	Arabidopsis thaliana uncharacterized protein mRNA, complete cds
AT1G76760	-1.188248923	1.16E-07	Arabidopsis thaliana thioredoxin Y1 mRNA, complete cds
AT5G44500	-1.017777674	1.17E-07	Arabidopsis thaliana small nuclear ribonucleoprotein family protein mRNA, complete cds
AT4G37700	-2.369489238	1.19E-07	Arabidopsis thaliana chromosome 4 sequence
AT2G24970	-2.727041243	1.20E-07	Arabidopsis thaliana uncharacterized protein mRNA, complete cds
AT1G48510	-1.44921643	1.24E-07	Arabidopsis thaliana Surfeit locus 1 cytochrome c oxidase biogenesis protein

			mRNA, complete cds
AT2G47670	-1.761806661	1.28E-07	Arabidopsis thaliana chromosome 2, complete sequence
AT1G77210	-1.212763734	1.29E-07	Arabidopsis thaliana sugar transport protein 14 mRNA, complete cds
AT4G11460	-3.413883357	1.30E-07	Arabidopsis thaliana putative cysteine-rich receptor-like protein kinase 30 mRNA, complete cds
AT2G28460	-2.276379833	1.32E-07	Arabidopsis thaliana chromosome 2, complete sequence
AT3G08660	-1.614376297	1.33E-07	Arabidopsis thaliana phototropic-responsive NPH3 family protein mRNA, complete cds
AT2G35000	-1.008222995	1.35E-07	Arabidopsis thaliana chromosome 2, complete sequence
AT4G15270	-1.793987066	1.35E-07	Arabidopsis thaliana glucosyltransferase-related protein mRNA, complete cds
AT3G07490	-3.861342334	1.38E-07	Arabidopsis thaliana chromosome 3, complete sequence
AT4G39630	-1.462246379	1.40E-07	Arabidopsis thaliana uncharacterized protein mRNA, complete cds
AT5G50140	-4.709339241	1.43E-07	Arabidopsis thaliana ankyrin repeat-containing protein mRNA, complete cds
AT3G11390	-4.709339241	1.43E-07	Arabidopsis thaliana chromosome 3, complete sequence
AT3G47740	-4.709339241	1.43E-07	Arabidopsis thaliana ABC transporter A family member 3 mRNA, complete cds
AT4G30330	-1.078187107	1.47E-07	Arabidopsis thaliana small nuclear ribonucleoprotein E mRNA, complete cds
AT3G12820	-1.954451739	1.54E-07	Arabidopsis thaliana myb domain protein 10 mRNA, complete cds
AT3G56730	-2.005077812	1.55E-07	Arabidopsis thaliana putative endonuclease or glycosyl hydrolase mRNA, complete cds
AT1G79320	-1.659708473	1.58E-07	Arabidopsis thaliana metacaspase 6 mRNA, complete cds
AT1G64920	-2.446304835	1.76E-07	Arabidopsis thaliana chromosome 1 sequence
AT1G08670	-2.446304835	1.76E-07	Arabidopsis thaliana ENTH/VHS family protein mRNA, complete cds
AT1G79760	-1.490504639	1.77E-07	Arabidopsis thaliana downstream target

			of AGL15-4 mRNA, complete cds
AT2G23960	-1.711881435	1.85E-07	Arabidopsis thaliana class I glutamine amidotransferase-like superfamily protein mRNA, complete cds
AT1G78320	-1.711881435	1.85E-07	Arabidopsis thaliana glutathione S-transferase TAU 23 mRNA, complete cds
AT2G40160	-1.161776712	1.93E-07	Arabidopsis thaliana protein TRICHOME BIREFRINGENCE-LIKE 30 mRNA, complete cds
AT5G54970	-1.143929537	1.97E-07	Arabidopsis thaliana chromosome 5 sequence
AT5G43250	-3.083734756	2.00E-07	Arabidopsis thaliana chromosome 5 sequence
AT5G45240	-2.691417333	2.08E-07	Arabidopsis thaliana TIR-NBS-LRR class disease resistance protein mRNA, complete cds
AT1G73290	-2.691417333	2.08E-07	Arabidopsis thaliana serine carboxypeptidase-like 5 mRNA, complete cds
AT3G44798	-1.010304973	2.17E-07	Arabidopsis thaliana chromosome 3, complete sequence
AT4G14548	-1.522136248	2.22E-07	Arabidopsis thaliana chromosome 4 sequence
AT4G24180	-1.80689455	2.22E-07	Arabidopsis thaliana thaumatin-like protein 1 mRNA, complete cds
AT2G37390	-1.319024171	2.22E-07	Arabidopsis thaliana chloroplast-targeted copper chaperone protein mRNA, complete cds
AT5G01360	-1.359708217	2.32E-07	Arabidopsis thaliana protein TRICHOME BIREFRINGENCE-LIKE 3 mRNA, complete cds
AT5G40090	-1.844268821	2.32E-07	Arabidopsis thaliana chromosome 5 sequence
AT1G16000	-1.056813456	2.33E-07	Arabidopsis thaliana uncharacterized protein mRNA, complete cds
AT4G11020	-3.369489238	2.34E-07	Arabidopsis thaliana uncharacterized protein mRNA, complete cds
AT1G27620	-1.232752901	2.34E-07	Arabidopsis thaliana HXXXD-type acyl-transferase-like protein mRNA, complete cds
AT5G23350	-1.406147708	2.36E-07	Arabidopsis thaliana chromosome 5 sequence
AT1G69520	-1.640343148	2.41E-07	Arabidopsis thaliana

			methyltransferase-like protein mRNA, complete cds
AT2G44390	-5.598307928	2.47E-07	Arabidopsis thaliana cysteine/histidine-rich C1 domain-containing protein mRNA, complete cds
AT2G37950	-1.979986831	2.49E-07	Arabidopsis thaliana RING/FYVE/PHD zinc finger-containing protein mRNA, complete cds
AT3G50120	-2.539414239	2.55E-07	Arabidopsis thaliana uncharacterized protein mRNA, complete cds
AT5G24960	-2.539414239	2.55E-07	Arabidopsis thaliana cytochrome P450 71A14 mRNA, complete cds
AT5G07322	-1.369489238	2.65E-07	Arabidopsis thaliana chromosome 5 sequence
AT4G13860	-1.369489238	2.65E-07	Arabidopsis thaliana RNA recognition motif-containing protein mRNA, complete cds
AT3G14900	-1.150371949	2.68E-07	Arabidopsis thaliana chromosome 3, complete sequence
AT2G25410	-2.841977009	2.78E-07	Arabidopsis thaliana RING-H2 finger protein ATL22 mRNA, complete cds
AT5G38970	-1.57738209	3.03E-07	Arabidopsis thaliana brassinosteroid-6-oxidase 1 mRNA, complete cds
AT1G79720	-1.087718269	3.24E-07	Arabidopsis thaliana eukaryotic aspartyl protease family protein mRNA, complete cds
AT5G55125	-1.087718269	3.24E-07	Arabidopsis thaliana ribosomal protein L31 mRNA, complete cds
AT4G30980	-1.087718269	3.24E-07	Arabidopsis thaliana transcription factor bHLH69 mRNA, complete cds
AT5G63480	-1.504648821	3.30E-07	Arabidopsis thaliana uncharacterized protein mRNA, complete cds
AT5G11610	-2.308088693	3.34E-07	Arabidopsis thaliana Exostosin family protein mRNA, complete cds
AT5G58010	-2.654891457	3.59E-07	Arabidopsis thaliana bHLH protein LJRHL1-like 3 mRNA, complete cds
AT2G05632	-1.048233885	3.71E-07	Arabidopsis thaliana chromosome 2, complete sequence
AT5G02350	-1.048233885	3.71E-07	Arabidopsis thaliana chromosome 5 sequence
AT3G09220	-1.089803592	3.79E-07	Arabidopsis thaliana laccase 7 mRNA, complete cds

AT4G04990	-2.13887631	3.83E-07	Arabidopsis thaliana uncharacterized protein mRNA, complete cds
AT5G38340	-1.456952079	3.88E-07	Arabidopsis thaliana TIR-NBS-LRR class disease resistance protein mRNA, complete cds
AT1G17300	-1.905542138	3.92E-07	Arabidopsis thaliana uncharacterized protein mRNA, complete cds
AT3G13437	-1.905542138	3.92E-07	Arabidopsis thaliana uncharacterized protein mRNA, complete cds
AT5G44910	-1.202379252	4.00E-07	Arabidopsis thaliana Toll-Interleukin-Resistance domain-containing protein mRNA, complete cds
AT1G33817.1	-1.539414239	4.11E-07	Arabidopsis thaliana chromosome 1 sequence
AT3G04770	-1.013345428	4.12E-07	Arabidopsis thaliana 40S ribosomal protein Sa-2 mRNA, complete cds
AT2G44110	-3.323685548	4.21E-07	Arabidopsis thaliana MLO-like protein 15 mRNA, complete cds
AT4G28900.1	-1.256107438	4.22E-07	Arabidopsis thaliana chromosome 4 sequence
AT1G02370	-1.160902616	4.25E-07	Arabidopsis thaliana pentatricopeptide repeat-containing protein mRNA, complete cds
AT5G53380	-5.539414239	4.34E-07	Arabidopsis thaliana O-acyltransferase (WSD1-like) family protein mRNA, complete cds
AT1G47578	-2.504648821	4.35E-07	Arabidopsis thaliana putative lipoyltransferase-like protein mRNA, complete cds
AT1G62010	-1.471510175	4.37E-07	Arabidopsis thaliana chromosome 1 sequence
AT1G26360	-3.761806661	4.56E-07	Arabidopsis thaliana methyl esterase 13 mRNA, complete cds
AT4G31940	-4.598307928	4.66E-07	Arabidopsis thaliana cytochrome P450, family 82, subfamily C, polypeptide 4 mRNA, complete cds
AT4G10370	-2.380716493	5.02E-07	Arabidopsis thaliana chromosome 4 sequence
AT1G56120	-1.578942604	5.06E-07	Arabidopsis thaliana Leucine-rich repeat transmembrane protein kinase mRNA, complete cds
AT3G07580	-1.110246411	5.18E-07	Arabidopsis thaliana uncharacterized protein mRNA, complete cds

AT1G27500	-1.078996837	5.18E-07	Arabidopsis thaliana tetratricopeptide repeat domain-containing protein mRNA, complete cds
AT5G65160	-1.38579105	5.72E-07	Arabidopsis thaliana tetratricopeptide repeat (TPR)-containing protein mRNA, complete cds
AT3G51230	-1.38579105	5.72E-07	Arabidopsis thaliana uncharacterized protein mRNA, complete cds
AT1G55430	-2.187112495	5.99E-07	Arabidopsis thaliana chromosome 1 sequence
AT1G23980	-1.130301574	5.99E-07	Arabidopsis thaliana chromosome 1 sequence
AT2G20980	-1.306437067	6.05E-07	Arabidopsis thaliana minichromosome maintenance protein 10 mRNA, complete cds
AT5G59410	-1.096470743	6.08E-07	Arabidopsis thaliana Rab5-interacting family protein mRNA, complete cds
AT2G14090	-1.096470743	6.08E-07	Arabidopsis thaliana chromosome 2, complete sequence
AT5G24140	-1.520798561	6.16E-07	Arabidopsis thaliana squalene monooxygenase 2 mRNA, complete cds
AT3G24110	-1.348730678	6.36E-07	Arabidopsis thaliana putative calcium-binding protein CML22 mRNA, complete cds
AT1G61050	-1.981932475	6.45E-07	Arabidopsis thaliana chromosome 1 sequence
AT5G01700	-1.276379833	6.51E-07	Arabidopsis thaliana putative protein phosphatase 2C 65 mRNA, complete cds
AT2G26440	-1.040608382	6.91E-07	Arabidopsis thaliana Probable pectinesterase/pectinesterase inhibitor 12 mRNA, complete cds
AT2G40010	-1.314853981	6.96E-07	Arabidopsis thaliana 60S acidic ribosomal protein P0-1 mRNA, complete cds
AT3G57860	-1.13342188	7.00E-07	Arabidopsis thaliana UV-B-insensitive 4-like protein mRNA, complete cds
AT3G54180	-1.053987412	7.00E-07	Arabidopsis thaliana cyclin-dependent kinase B1-1 mRNA, complete cds
AT4G34930	-2.469024911	7.40E-07	Arabidopsis thaliana chromosome 4 sequence
AT3G52630	-1.409646364	7.40E-07	Arabidopsis thaliana Nucleic acid-binding, OB-fold-like protein mRNA, complete cds
AT4G20740	-1.283759364	7.51E-07	Arabidopsis thaliana chromosome 4

			sequence
AT2G03600	-3.276379833	7.56E-07	Arabidopsis thaliana ureide permease 3 mRNA, complete cds
AT1G64450	-1.199074107	7.59E-07	Arabidopsis thaliana chromosome 1 sequence
AT2G43220	-5.478013695	7.63E-07	Arabidopsis thaliana chromosome 2, complete sequence
AT4G24204	-5.478013695	7.63E-07	Arabidopsis thaliana RING-finger domain-containing protein mRNA, complete cds
AT5G63090	-1.706524225	7.83E-07	Arabidopsis thaliana protein LATERAL ORGAN BOUNDARIES mRNA, complete cds
AT5G05240	-1.17684416	7.84E-07	Arabidopsis thaliana uncharacterized protein mRNA, complete cds
AT3G50810	-1.255111217	8.01E-07	Arabidopsis thaliana uncharacterized protein mRNA, complete cds
AT2G21080	-1.055537864	8.21E-07	Arabidopsis thaliana uncharacterized protein mRNA, complete cds
AT4G22840	-1.055537864	8.21E-07	Arabidopsis thaliana putative sodium/metabolite cotransporter BASS6 mRNA, complete cds
AT4G09130	-3.709339241	8.28E-07	Arabidopsis thaliana chromosome 4 sequence
AT2G46495	-2.346769161	8.44E-07	Arabidopsis thaliana putative RING-H2 finger protein mRNA, complete cds
AT3G27070	-2.346769161	8.44E-07	Arabidopsis thaliana mitochondrial import receptor subunit TOM20-1 mRNA, complete cds
AT3G46340	-4.539414239	8.44E-07	Arabidopsis thaliana putative receptor-like protein kinase mRNA, complete cds
AT3G42725	-2.761806661	8.49E-07	Arabidopsis thaliana chromosome 3, complete sequence
AT2G03200	-1.071808689	9.71E-07	Arabidopsis thaliana chromosome 2, complete sequence
AT5G37478	-1.628223506	1.02E-06	Arabidopsis thaliana TPX2 (targeting protein for Xklp2) family protein mRNA, complete cds
AT5G44920	-2.013345428	1.04E-06	Arabidopsis thaliana Toll-Interleukin-Resistance domain-containing protein mRNA, complete cds
AT1G33320	-2.578942604	1.06E-06	Arabidopsis thaliana Pyridoxal phosphate

			(PLP)-dependent transferases superfamily protein mRNA, complete cds
AT3G08490	-2.578942604	1.06E-06	Arabidopsis thaliana uncharacterized protein mRNA, complete cds
AT2G43480	-2.954451739	1.11E-06	Arabidopsis thaliana probable peroxidase 26 mRNA, complete cds
AT4G13690	-2.954451739	1.11E-06	Arabidopsis thaliana uncharacterized protein mRNA, complete cds
AT1G61080	-2.954451739	1.11E-06	Arabidopsis thaliana hydroxyproline-rich glycoprotein-like protein mRNA, complete cds
AT3G06990	-1.143485563	1.12E-06	Arabidopsis thaliana cysteine/histidine-rich C1 domain-containing protein mRNA, complete cds
AT3G26770	-1.106454832	1.14E-06	Arabidopsis thaliana NAD(P)-binding Rossmann-fold superfamily protein mRNA, complete cds
AT1G70895	-1.308088693	1.15E-06	Arabidopsis thaliana chromosome 1 sequence
AT5G15770	-1.683804149	1.21E-06	Arabidopsis thaliana chromosome 5 sequence
AT3G15150	-1.353001115	1.21E-06	Arabidopsis thaliana E3 SUMO-protein ligase MMS21 mRNA, complete cds
AT1G65032	-1.276379833	1.24E-06	Arabidopsis thaliana uncharacterized protein mRNA, complete cds
AT3G28857	-2.432499035	1.26E-06	Arabidopsis thaliana protein PACLOBUTRAZOL RESISTANCE 5 mRNA, complete cds
AT2G28270	-2.432499035	1.26E-06	Arabidopsis thaliana chromosome 2, complete sequence
AT3G60490	-1.019546767	1.26E-06	Arabidopsis thaliana chromosome 3, complete sequence
AT1G05600	-1.168047212	1.27E-06	Arabidopsis thaliana pentatricopeptide repeat-containing protein mRNA, complete cds
AT3G29120.1	-1.032454251	1.29E-06	Arabidopsis thaliana chromosome 3, complete sequence
AT2G47750	-1.147096817	1.30E-06	Arabidopsis thaliana putative indole-3-acetic acid-amido synthetase GH3.9 mRNA, complete cds
AT2G32100	-1.247233488	1.32E-06	Arabidopsis thaliana chromosome 2, complete sequence

AT5G04310	-1.060493376	1.32E-06	Arabidopsis thaliana pectin lyase-like superfamily protein mRNA, complete cds
AT3G15700	-5.413883357	1.35E-06	Arabidopsis thaliana P-loop containing nucleoside triphosphate hydrolases superfamily protein mRNA, complete cds
AT3G26815	-5.413883357	1.35E-06	Arabidopsis thaliana chromosome 3, complete sequence
AT1G33910	-5.413883357	1.35E-06	Arabidopsis thaliana P-loop containing nucleoside triphosphate hydrolases superfamily protein mRNA, complete cds
AT5G23030	-5.413883357	1.35E-06	Arabidopsis thaliana tetraspanin12 mRNA, complete cds
AT5G26790	-3.227470233	1.36E-06	Arabidopsis thaliana chromosome 5 sequence
AT5G20640	-3.227470233	1.36E-06	Arabidopsis thaliana protein LURP-one-related 16 mRNA, complete cds
AT5G66815	-3.227470233	1.36E-06	Arabidopsis thaliana chromosome 5 sequence
AT5G07760	-1.482830711	1.37E-06	Arabidopsis thaliana putative formin-like protein 21a mRNA, complete cds
AT5G45440	-1.749631947	1.39E-06	Arabidopsis thaliana chromosome 5 sequence
AT5G16530	-1.418398838	1.40E-06	Arabidopsis thaliana auxin transporter PIN5 mRNA, complete cds
AT2G15128	-1.195459838	1.44E-06	Arabidopsis thaliana chromosome 2, complete sequence
AT3G25950	-2.719986485	1.48E-06	Arabidopsis thaliana chromosome 3, complete sequence
AT4G20820	-1.172356769	1.49E-06	Arabidopsis thaliana chromosome 4 sequence
AT5G43240	-3.654891457	1.50E-06	Arabidopsis thaliana uncharacterized protein mRNA, complete cds
AT3G61930	-3.654891457	1.50E-06	Arabidopsis thaliana chromosome 3, complete sequence
AT2G16980	-1.047561143	1.53E-06	Arabidopsis thaliana major facilitator protein mRNA, complete cds
AT3G27490	-4.478013695	1.53E-06	Arabidopsis thaliana chromosome 3, complete sequence
AT3G09240	-2.210791492	1.54E-06	Arabidopsis thaliana Protein kinase protein with tetratricopeptide repeat

			domain mRNA, complete cds
AT1G63855	-1.432499035	1.59E-06	Arabidopsis thaliana putative methyltransferase family protein mRNA, complete cds
AT4G01925	-2.12437674	1.62E-06	Arabidopsis thaliana cysteine/histidine-rich C1 domain-containing protein mRNA, complete cds
AT1G28100	-1.021565934	1.74E-06	Arabidopsis thaliana uncharacterized protein mRNA, complete cds
AT1G76870	-1.336322374	1.75E-06	Arabidopsis thaliana chromosome 1 sequence
AT5G35480	-1.336322374	1.75E-06	Arabidopsis thaliana chromosome 5 sequence
AT1G03300	-1.387411146	1.80E-06	Arabidopsis thaliana uncharacterized protein mRNA, complete cds
AT4G14940	-1.06407623	1.82E-06	Arabidopsis thaliana amine oxidase 1 mRNA, complete cds
AT1G65620	-1.114916411	1.82E-06	Arabidopsis thaliana protein ASYMMETRIC LEAVES 2 mRNA, complete cds
AT4G36060	-1.114916411	1.82E-06	Arabidopsis thaliana transcription factor bHLH11 mRNA, complete cds
AT5G66005	-1.114916411	1.82E-06	Arabidopsis thaliana uncharacterized protein mRNA, complete cds
AT2G19660	-1.660720536	1.85E-06	Arabidopsis thaliana chromosome 2, complete sequence
AT3G48160	-1.205990506	1.95E-06	Arabidopsis thaliana E2F transcription factor-like E2FE mRNA, complete cds
AT1G16440	-2.908648049	1.96E-06	Arabidopsis thaliana protein root hair specific 3 mRNA, complete cds
AT4G20325	-1.022623241	2.04E-06	Arabidopsis thaliana ribonuclease H2 subunit B domain-containing protein mRNA, complete cds
AT1G16070	-1.400251492	2.05E-06	Arabidopsis thaliana Tubby-like protein 8 mRNA, complete cds
AT2G37700	-1.158810237	2.07E-06	Arabidopsis thaliana protein CER1-like 2 mRNA, complete cds
AT4G33020	-2.39502433	2.13E-06	Arabidopsis thaliana Fe(II) transporter isolog family protein mRNA, complete cds
AT2G28330	-2.39502433	2.13E-06	Arabidopsis thaliana chromosome 2, complete sequence
AT4G17215	-1.065960054	2.13E-06	Arabidopsis thaliana pollen Ole e 1

			allergen and extensin family protein mRNA, complete cds
AT5G43080	-1.309932393	2.19E-06	Arabidopsis thaliana cyclin A3-1 mRNA, complete cds
AT1G74458	-1.309932393	2.19E-06	Arabidopsis thaliana uncharacterized protein mRNA, complete cds
AT4G25780	-1.211609578	2.26E-06	Arabidopsis thaliana chromosome 4 sequence
AT2G20562	-1.761806661	2.30E-06	Arabidopsis thaliana uncharacterized protein mRNA, complete cds
AT1G07680	-5.346769161	2.39E-06	Arabidopsis thaliana uncharacterized protein mRNA, complete cds
AT1G58037	-5.346769161	2.39E-06	Arabidopsis thaliana chromosome 1 sequence
AT3G54710	-1.023714401	2.40E-06	Arabidopsis thaliana CDT1-like protein b mRNA, complete cds
AT1G49170	-1.023714401	2.40E-06	Arabidopsis thaliana uncharacterized protein mRNA, complete cds
AT5G45090	-3.17684416	2.43E-06	Arabidopsis thaliana protein PHLOEM PROTEIN 2-LIKE A7 mRNA, complete cds
AT5G39865	-1.847536535	2.55E-06	Arabidopsis thaliana chromosome 5 sequence
AT2G22890	-1.847536535	2.55E-06	Arabidopsis thaliana chromosome 2, complete sequence
AT5G46440	-1.498772255	2.56E-06	Arabidopsis thaliana chromosome 5 sequence
AT2G37560	-1.609803567	2.59E-06	Arabidopsis thaliana origin recognition complex subunit 2 mRNA, complete cds
AT4G21230	-1.609803567	2.59E-06	Arabidopsis thaliana cysteine-rich receptor-like protein kinase 27 mRNA, complete cds
AT5G19870	-1.428382927	2.64E-06	Arabidopsis thaliana chromosome 5 sequence
AT1G75720	-2.091955262	2.65E-06	Arabidopsis thaliana uncharacterized protein mRNA, complete cds
AT5G45120	-2.091955262	2.65E-06	Arabidopsis thaliana chromosome 5 sequence
AT2G43670	-4.413883357	2.79E-06	Arabidopsis thaliana carbohydrate-binding X8 domain-containing protein mRNA, complete cds
AT1G48670	-4.413883357	2.79E-06	Arabidopsis thaliana auxin-responsive GH3 family protein mRNA, complete

			cds
AT5G26080	-4.413883357	2.79E-06	Arabidopsis thaliana chromosome 5 sequence
AT2G05765	-4.413883357	2.79E-06	Arabidopsis thaliana chromosome 2, complete sequence
AT3G23010	-1.32949117	2.89E-06	Arabidopsis thaliana chromosome 3, complete sequence
AT3G27650	-1.12437674	2.92E-06	Arabidopsis thaliana LOB domain-containing protein 25 mRNA, complete cds
AT2G37330	-1.069928956	2.93E-06	Arabidopsis thaliana protein aluminum sensitive 3 mRNA, complete cds
AT3G46720	-1.223638371	3.06E-06	Arabidopsis thaliana UDP-glycosyltransferase 76E5 mRNA, complete cds
AT4G30560	-1.012399088	3.23E-06	Arabidopsis thaliana cyclic nucleotide gated channel 9 mRNA, complete cds
AT2G36307	-1.260260168	3.36E-06	Arabidopsis thaliana mRNA for hypothetical protein, complete cds, clone: RAFL22-71-B06
AT1G09170	-1.460342668	3.38E-06	Arabidopsis thaliana calponin homology and kinesin motor domain-containing protein mRNA, complete cds
AT1G64210	-2.861342334	3.45E-06	Arabidopsis thaliana putative inactive receptor-like protein kinase mRNA, complete cds
AT5G37000	-1.230086181	3.55E-06	Arabidopsis thaliana Exostosin family protein mRNA, complete cds
AT2G39880	-1.735811452	3.59E-06	Arabidopsis thaliana myb domain protein 25 mRNA, complete cds
AT2G26400	-2.356550182	3.59E-06	Arabidopsis thaliana acireductone dioxygenase 3 mRNA, complete cds
AT3G60720	-1.013345428	3.79E-06	Arabidopsis thaliana plasmodesmata-located protein 8 mRNA, complete cds
AT1G29785	-1.351341891	3.81E-06	Arabidopsis thaliana chromosome 1 sequence
AT5G36700	-1.775481598	3.82E-06	Arabidopsis thaliana 2-phosphoglycolate phosphatase 1 mRNA, complete cds
AT5G49560	-1.17684416	3.83E-06	Arabidopsis thaliana chromosome 5 sequence
AT5G01250	-1.268112218	3.89E-06	Arabidopsis thaliana chromosome 5 sequence
AT3G50150	-2.239853957	3.95E-06	Arabidopsis thaliana uncharacterized

			protein mRNA, complete cds
AT5G61350	-1.091955262	4.04E-06	Arabidopsis thaliana chromosome 5 sequence
AT5G58500	-2.142078742	4.19E-06	Arabidopsis thaliana chromosome 5 sequence
AT4G11490	-5.276379833	4.24E-06	Arabidopsis thaliana putative cysteine-rich receptor-like protein kinase 33 mRNA, complete cds
AT1G55410	-5.276379833	4.24E-06	Arabidopsis thaliana chromosome 1 sequence
AT1G19390	-5.276379833	4.24E-06	Arabidopsis thaliana putative wall-associated receptor kinase-like 11 mRNA, complete cds
AT4G14305	-1.92407809	4.31E-06	Arabidopsis thaliana Mpv17/PMP22 domain-containing protein mRNA, complete cds
AT1G02440	-3.12437674	4.34E-06	Arabidopsis thaliana ADP-ribosylation factor D1A mRNA, complete cds
AT3G61840	-3.12437674	4.34E-06	Arabidopsis thaliana chromosome 3, complete sequence
AT5G19160	-1.363257284	4.36E-06	Arabidopsis thaliana protein trichome birefringence-like 11 mRNA, complete cds
AT1G15890	-1.157543604	4.58E-06	Arabidopsis thaliana chromosome 1 sequence
AT2G35380	-1.043456745	4.63E-06	Arabidopsis thaliana peroxidase 20 mRNA, complete cds
AT3G15140	-1.043456745	4.63E-06	Arabidopsis thaliana polynucleotidyl transferase, ribonuclease H-like superfamily protein mRNA, complete cds
AT5G24980	-1.642507732	4.73E-06	Arabidopsis thaliana uncharacterized protein mRNA, complete cds
AT3G09780	-1.642507732	4.73E-06	Arabidopsis thaliana chromosome 3, complete sequence
AT4G23070	-3.539414239	4.96E-06	Arabidopsis thaliana RHOMBOID-like protein 7 mRNA, complete cds
AT5G01910	-1.375915507	4.98E-06	Arabidopsis thaliana uncharacterized protein mRNA, complete cds
AT2G19050	-4.346769161	5.09E-06	Arabidopsis thaliana GDSL esterase/lipase mRNA, complete cds
AT5G52270	-4.346769161	5.09E-06	Arabidopsis thaliana SNARE-like superfamily protein mRNA, complete cds

AT5G24220	-4.346769161	5.09E-06	Arabidopsis thaliana lipase class 3-related protein mRNA, complete cds
AT4G05200	-1.285097051	5.19E-06	Arabidopsis thaliana cysteine-rich receptor-like protein kinase 25 mRNA, complete cds
AT5G16410	-1.285097051	5.19E-06	Arabidopsis thaliana HXXXD-type acyl-transferase-like protein mRNA, complete cds
AT1G23140	-1.285097051	5.19E-06	Arabidopsis thaliana calcium-dependent lipid-binding domain-containing protein mRNA, complete cds
AT2G44580	-1.187112495	5.20E-06	Arabidopsis thaliana zinc ion binding protein mRNA, complete cds
AT3G61300	-2.456952079	5.33E-06	Arabidopsis thaliana chromosome 3, complete sequence
AT1G61795	-1.029739866	5.35E-06	Arabidopsis thaliana PAK-box/P21-Rho-binding family protein mRNA, complete cds
AT5G61740	-1.251433476	5.53E-06	Arabidopsis thaliana ABC transporter A family member 10 mRNA, complete cds
AT1G72125	-1.220731804	5.82E-06	Arabidopsis thaliana putative peptide/nitrate transporter mRNA, complete cds
AT4G21890	-1.220731804	5.82E-06	Arabidopsis thaliana chromosome 4 sequence
AT2G28140	-1.748000861	5.96E-06	Arabidopsis thaliana uncharacterized protein mRNA, complete cds
AT4G37240	-1.294301741	5.99E-06	Arabidopsis thaliana chromosome 4 sequence
AT1G15250	-1.002546027	6.00E-06	Arabidopsis thaliana 60S ribosomal protein L37-1 mRNA, complete cds
AT1G61480	-2.317021818	6.04E-06	Arabidopsis thaliana G-type lectin S-receptor-like serine/threonine-protein kinase mRNA, complete cds
AT1G48690	-2.317021818	6.04E-06	Arabidopsis thaliana auxin-responsive GH3 family protein mRNA, complete cds
AT1G03660	-2.812432734	6.06E-06	Arabidopsis thaliana chromosome 1 sequence
AT1G48780	-1.120810125	6.46E-06	Arabidopsis thaliana chromosome 1 sequence
AT3G60090	-2.202379252	6.56E-06	Arabidopsis thaliana chromosome 3, complete sequence
AT1G35625	-1.589167275	6.57E-06	Arabidopsis thaliana RING/U-box

			domain-containing protein mRNA, complete cds
AT5G44460	-1.838974521	6.63E-06	Arabidopsis thaliana chromosome 5 sequence
AT1G18000	-1.838974521	6.63E-06	Arabidopsis thaliana chromosome 1 sequence
AT5G24880	-1.227470233	6.76E-06	Arabidopsis thaliana chromosome 5 sequence
AT1G67460	-2.106454832	6.88E-06	Arabidopsis thaliana Minichromosome maintenance (MCM2/3/5) family protein mRNA, complete cds
AT1G33813.1	-2.106454832	6.88E-06	Arabidopsis thaliana chromosome 1 sequence
AT5G05320	-1.304036176	6.91E-06	Arabidopsis thaliana FAD/NAD(P)-binding oxidoreductase family protein mRNA, complete cds
AT1G71740	-1.954451739	7.01E-06	Arabidopsis thaliana chromosome 1 sequence
AT5G51870	-1.954451739	7.01E-06	Arabidopsis thaliana MADS-box transcription factor AGL71 mRNA, complete cds
AT5G39890	-2.024841066	7.02E-06	Arabidopsis thaliana mRNA for hypothetical protein, complete cds, clone: RAFL14-60-L23
AT1G78815	-1.198377321	7.06E-06	Arabidopsis thaliana chromosome 1 sequence
AT2G33735	-1.617416751	7.23E-06	Arabidopsis thaliana chaperone DnaJ-domain containing protein mRNA, complete cds
AT2G01300	-1.032454251	7.37E-06	Arabidopsis thaliana chromosome 2, complete sequence
AT3G46410	-5.202379252	7.58E-06	Arabidopsis thaliana protein kinase family protein mRNA, complete cds
AT4G03340	-1.083734756	7.61E-06	Arabidopsis thaliana core-2/I-branching beta-1,6-N-acetylglucosaminyltransferase family protein mRNA, complete cds
AT1G19320	-2.586719954	7.69E-06	Arabidopsis thaliana pathogenesis-related thaumatin-like protein mRNA, complete cds
AT1G65484	-3.069928956	7.75E-06	Arabidopsis thaliana uncharacterized protein mRNA, complete cds
AT3G60890	-3.069928956	7.75E-06	Arabidopsis thaliana protein little zipper 2 mRNA, complete cds
AT5G44690	-3.069928956	7.75E-06	Arabidopsis thaliana uncharacterized

			protein mRNA, complete cds
AT4G37810	-1.648348611	7.90E-06	Arabidopsis thaliana uncharacterized protein mRNA, complete cds
AT4G16400	-1.314347684	7.96E-06	Arabidopsis thaliana uncharacterized protein mRNA, complete cds
AT1G05720	-1.204429992	8.21E-06	Arabidopsis thaliana selenoprotein family protein mRNA, complete cds
AT5G61200	-1.435578428	8.35E-06	Arabidopsis thaliana uncharacterized protein mRNA, complete cds
AT3G62460	-1.276379833	8.58E-06	Arabidopsis thaliana putative endonuclease or glycosyl hydrolase mRNA, complete cds
AT5G59930	-1.276379833	8.58E-06	Arabidopsis thaliana chromosome 5 sequence
AT1G61950	-3.478013695	9.01E-06	Arabidopsis thaliana calcium-dependent protein kinase 19 mRNA, complete cds
AT3G44710	-3.478013695	9.01E-06	Arabidopsis thaliana chromosome 3, complete sequence
AT3G50825	-1.242028329	9.11E-06	Arabidopsis thaliana chromosome 3, complete sequence
AT1G65710	-1.131989924	1.03E-05	Arabidopsis thaliana chromosome 1 sequence
AT1G31460	-1.109729964	1.04E-05	Arabidopsis thaliana chromosome 1 sequence
AT1G36940	-1.336921375	1.05E-05	Arabidopsis thaliana uncharacterized protein mRNA, complete cds
AT4G01820	-2.761806661	1.06E-05	Arabidopsis thaliana ABC transporter B family member 3 mRNA, complete cds
AT3G27480	-2.761806661	1.06E-05	Arabidopsis thaliana cysteine/histidine-rich C1 domain-containing protein mRNA, complete cds
AT4G20362	-2.761806661	1.06E-05	Arabidopsis thaliana Full-length cDNA Complete sequence from clone GSLTPGH40ZC03 of Hormone Treated Callus of strain col-0 of Arabidopsis thaliana (thale cress)
AT2G27930	-1.591881659	1.10E-05	Arabidopsis thaliana PLATZ transcription factor family protein mRNA, complete cds
AT5G28010	-1.921284875	1.12E-05	Arabidopsis thaliana polyketide cyclase/dehydrase and lipid transport superfamily protein mRNA, complete cds

AT2G17050	-2.069928956	1.13E-05	Arabidopsis thaliana TIR-NBS-LRR class disease resistance protein mRNA, complete cds
AT1G17610	-1.295488656	1.14E-05	Arabidopsis thaliana chromosome 1 sequence
AT1G16445	-1.492871653	1.20E-05	Arabidopsis thaliana S-adenosyl-L-methionine-dependent methyltransferase domain-containing protein mRNA, complete cds
AT1G51420	-1.349311356	1.21E-05	Arabidopsis thaliana sucrose-phosphatase 1 mRNA, complete cds
AT2G16760	-1.413883357	1.23E-05	Arabidopsis thaliana uncharacterized protein mRNA, complete cds
AT2G34655	-1.224540902	1.29E-05	Arabidopsis thaliana chromosome 2, complete sequence
AT5G18540	-1.193917673	1.35E-05	Arabidopsis thaliana uncharacterized protein mRNA, complete cds
AT1G47520.1	-1.006919158	1.35E-05	Arabidopsis thaliana chromosome 1 sequence
AT2G03370	-5.12437674	1.36E-05	Arabidopsis thaliana Glycosyltransferase family 61 protein mRNA, complete cds
AT1G54940	-5.12437674	1.36E-05	Arabidopsis thaliana putative UDP-glucuronate:xylan alpha-glucuronosyltransferase 4 mRNA, complete cds
AT1G30990	-5.12437674	1.36E-05	Arabidopsis thaliana ligand-binding bet-v-1 domain-containing protein mRNA, complete cds
AT2G37820	-3.013345428	1.38E-05	Arabidopsis thaliana chromosome 2, complete sequence
AT3G20850	-3.013345428	1.38E-05	Arabidopsis thaliana chromosome 3, complete sequence
AT5G36260	-3.013345428	1.38E-05	Arabidopsis thaliana aspartyl protease family protein mRNA, complete cds
AT1G53640	-3.013345428	1.38E-05	Arabidopsis thaliana uncharacterized protein mRNA, complete cds
AT4G25600	-1.038516003	1.40E-05	Arabidopsis thaliana Oxoglutarate/iron-dependent oxygenase mRNA, complete cds
AT4G37950	-2.369489238	1.54E-05	Arabidopsis thaliana Rhamnogalacturonate lyase family protein mRNA, complete cds
AT4G22230	-2.369489238	1.54E-05	Arabidopsis thaliana defensin-like protein 96 mRNA, complete cds

AT3G55700	-1.732059317	1.54E-05	Arabidopsis thaliana UDP-glycosyltransferase 76F1 mRNA, complete cds
AT1G27110	-1.376684739	1.58E-05	Arabidopsis thaliana tetratricopeptide repeat (TPR)-like superfamily protein mRNA, complete cds
AT4G13390	-3.413883357	1.63E-05	Arabidopsis thaliana chromosome 4 sequence
AT5G22555	-3.413883357	1.63E-05	Arabidopsis thaliana uncharacterized protein mRNA, complete cds
AT3G45080	-3.413883357	1.63E-05	Arabidopsis thaliana chromosome 3, complete sequence
AT5G44585	-1.276379833	1.64E-05	Arabidopsis thaliana uncharacterized protein mRNA, complete cds
AT2G30230	-1.040181613	1.65E-05	Arabidopsis thaliana chromosome 2, complete sequence
AT3G25100	-1.144782951	1.65E-05	Arabidopsis thaliana chromosome 3, complete sequence
AT2G23530	-1.565886451	1.67E-05	Arabidopsis thaliana zinc-finger domain of monoamine-oxidase A repressor R1 mRNA, complete cds
AT3G53590	-2.234559658	1.69E-05	Arabidopsis thaliana putative leucine-rich repeat receptor-like serine/threonine-protein kinase mRNA, complete cds
AT2G40470	-1.828920857	1.73E-05	Arabidopsis thaliana LOB domain-containing protein 15 mRNA, complete cds
AT2G14095	-2.12437674	1.79E-05	Arabidopsis thaliana uncharacterized protein mRNA, complete cds
AT1G60630	-1.2068389	1.83E-05	Arabidopsis thaliana mRNA for receptor kinase like protein, complete cds, clone: RAFL14-10-N14
AT2G34360	-1.2068389	1.83E-05	Arabidopsis thaliana MATE efflux family protein mRNA, complete cds
AT5G22890	-2.709339241	1.86E-05	Arabidopsis thaliana chromosome 5 sequence
AT5G35525	-2.709339241	1.86E-05	Arabidopsis thaliana PLAC8 family protein mRNA, complete cds
AT3G50140	-2.709339241	1.86E-05	Arabidopsis thaliana uncharacterized protein mRNA, complete cds
AT1G74580	-1.008899523	1.86E-05	Arabidopsis thaliana chromosome 1 sequence
AT3G06320	-1.17684416	1.89E-05	Arabidopsis thaliana ribosomal protein

			L33 family protein mRNA, complete cds
AT4G10130	-1.024841066	1.90E-05	Arabidopsis thaliana chromosome 4 sequence
AT4G28180	-1.149467721	1.93E-05	Arabidopsis thaliana chromosome 4 sequence
AT3G18960	-1.12437674	1.96E-05	Arabidopsis thaliana AP2/B3-like transcriptional factor family protein mRNA, complete cds
AT5G47740	-1.079982621	1.98E-05	Arabidopsis thaliana Adenine nucleotide alpha hydrolases-like superfamily protein mRNA, complete cds
AT2G42730	-1.101293127	1.98E-05	Arabidopsis thaliana F-box protein mRNA, complete cds
AT3G11402	-1.626877081	2.03E-05	Arabidopsis thaliana cysteine/histidine-rich C1 domain-containing protein mRNA, complete cds
AT2G10931	-1.626877081	2.03E-05	Arabidopsis thaliana chromosome 2, complete sequence
AT4G14650	-1.662270987	2.21E-05	Arabidopsis thaliana uncharacterized protein mRNA, complete cds
AT3G45420	-1.154389309	2.26E-05	Arabidopsis thaliana chromosome 3, complete sequence
AT3G19184	-2.490504639	2.27E-05	Arabidopsis thaliana AP2/B3-like transcriptional factor family protein mRNA, complete cds
AT2G36090	-1.082775836	2.32E-05	Arabidopsis thaliana chromosome 2, complete sequence
AT3G02610	-1.701685668	2.39E-05	Arabidopsis thaliana acyl-[acyl-carrier-protein] desaturase mRNA, complete cds
AT5G48320	-1.701685668	2.39E-05	Arabidopsis thaliana chromosome 5 sequence
AT1G06923	-5.04191458	2.45E-05	Arabidopsis thaliana uncharacterized protein mRNA, complete cds
AT3G46760	-5.04191458	2.45E-05	Arabidopsis thaliana chromosome 3, complete sequence
AT2G21010	-5.04191458	2.45E-05	Arabidopsis thaliana Calcium-dependent lipid-binding (CaLB domain) family protein mRNA, complete cds
AT1G76220	-2.954451739	2.46E-05	Arabidopsis thaliana chromosome 1 sequence
AT5G01060	-2.954451739	2.46E-05	Arabidopsis thaliana Protein kinase protein with tetratricopeptide repeat

			domain mRNA, complete cds
AT1G12775	-1.308088693	2.53E-05	Arabidopsis thaliana chromosome 1 sequence
AT5G45000	-1.745865117	2.57E-05	Arabidopsis thaliana TIR-NBS-LRR class disease resistance protein mRNA, complete cds
AT3G14580	-2.323685548	2.60E-05	Arabidopsis thaliana chromosome 3, complete sequence
AT1G30757	-1.369489238	2.64E-05	Arabidopsis thaliana chromosome 1 sequence
AT5G28960	-1.13278898	2.69E-05	Arabidopsis thaliana uncharacterized protein mRNA, complete cds
AT1G78990	-1.085696272	2.72E-05	Arabidopsis thaliana HXXXD-type acyl-transferase mRNA, complete cds
AT4G39795	-1.795753993	2.73E-05	Arabidopsis thaliana uncharacterized protein mRNA, complete cds
AT5G15890	-1.795753993	2.73E-05	Arabidopsis thaliana protein trichome birefringence-like 21 mRNA, complete cds
AT5G50760	-1.795753993	2.73E-05	Arabidopsis thaliana chromosome 5 sequence
AT1G26540	-1.567428615	2.80E-05	Arabidopsis thaliana agenet domain-containing protein mRNA, complete cds
AT4G13440	-2.191490936	2.82E-05	Arabidopsis thaliana chromosome 4 sequence
AT1G07050	-2.191490936	2.82E-05	Arabidopsis thaliana CCT motif family protein mRNA, complete cds
AT3G20160	-2.191490936	2.82E-05	Arabidopsis thaliana chromosome 3, complete sequence
AT1G53340	-1.852572125	2.86E-05	Arabidopsis thaliana chromosome 1 sequence
AT4G04293.1	-1.852572125	2.86E-05	Arabidopsis thaliana chromosome 4 sequence
AT5G47635	-1.852572125	2.86E-05	Arabidopsis thaliana pollen Ole e 1 allergen and extensin family protein mRNA, complete cds
AT3G13435	-1.852572125	2.86E-05	Arabidopsis thaliana uncharacterized protein mRNA, complete cds
AT3G14185	-3.346769161	2.96E-05	Arabidopsis thaliana Full-length cDNA Complete sequence from clone GSLTSIL56ZC04 of Silique of strain col-0 of Arabidopsis thaliana (thale cress)
AT5G51470	-3.346769161	2.96E-05	Arabidopsis thaliana auxin-responsive

			GH3 family protein mRNA, complete cds
AT1G26840	-1.165018725	3.09E-05	Arabidopsis thaliana origin recognition complex subunit 6 mRNA, complete cds
AT2G15350	-4.12437674	3.13E-05	Arabidopsis thaliana chromosome 2, complete sequence
AT3G63360	-4.12437674	3.13E-05	Arabidopsis thaliana defensin-like protein mRNA, complete cds
AT3G60280	-4.12437674	3.13E-05	Arabidopsis thaliana uclacyanin 3 mRNA, complete cds
AT1G57570	-4.12437674	3.13E-05	Arabidopsis thaliana jacalin-like plant lectin domain-containing protein mRNA, complete cds
AT3G09290	-4.12437674	3.13E-05	Arabidopsis thaliana chromosome 3, complete sequence
AT4G02830	-1.276379833	3.14E-05	Arabidopsis thaliana uncharacterized protein mRNA, complete cds
AT1G73580	-2.654891457	3.24E-05	Arabidopsis thaliana C2 domain-containing protein mRNA, complete cds
AT5G24105	-1.332963362	3.35E-05	Arabidopsis thaliana mRNA for hypothetical protein, complete cds, clone: RAFL19-15-P06
AT5G62420	-1.632523644	3.40E-05	Arabidopsis thaliana aldo/keto reductase family protein mRNA, complete cds
AT4G09810	-1.013345428	3.56E-05	Arabidopsis thaliana Nucleotide-sugar transporter family protein mRNA, complete cds
AT4G19950	-1.142078742	3.69E-05	Arabidopsis thaliana chromosome 4 sequence
AT3G11120	-1.049608972	3.69E-05	Arabidopsis thaliana 60S ribosomal protein L41 mRNA, complete cds
AT4G39410	-1.512447192	3.79E-05	Arabidopsis thaliana putative WRKY transcription factor 13 mRNA, complete cds
AT3G03850	-1.346769161	3.85E-05	Arabidopsis thaliana chromosome 3, complete sequence
AT2G32610	-1.246217863	3.88E-05	Arabidopsis thaliana cellulose synthase-like protein B1 mRNA, complete cds
AT4G27480	-1.420115311	3.92E-05	Arabidopsis thaliana core-2/I-branching beta-1,6-N-acetylglucosaminyltransferase family protein mRNA, complete cds
AT1G75335	-1.713443639	4.00E-05	Arabidopsis thaliana chromosome 1

			sequence
AT5G01190	-1.209708794	4.07E-05	Arabidopsis thaliana laccase 10 mRNA, complete cds
AT4G33810	-1.05174894	4.34E-05	Arabidopsis thaliana glycosyl hydrolase family protein mRNA, complete cds
AT1G04778	-2.893051194	4.37E-05	Arabidopsis thaliana uncharacterized protein mRNA, complete cds
AT1G80340	-2.893051194	4.37E-05	Arabidopsis thaliana gibberellin 3-beta-dioxygenase 2 mRNA, complete cds
AT1G70110	-2.893051194	4.37E-05	Arabidopsis thaliana putative L-type lectin-domain containing receptor kinase V.1 mRNA, complete cds
AT2G16580	-2.276379833	4.37E-05	Arabidopsis thaliana SAUR-like auxin-responsive protein mRNA, complete cds
AT1G29179	-4.954451739	4.42E-05	Arabidopsis thaliana chromosome 1 sequence
AT5G11440	-4.954451739	4.42E-05	Arabidopsis thaliana CTC-interacting domain 5 mRNA, complete cds
AT3G46260	-4.954451739	4.42E-05	Arabidopsis thaliana protein kinase-like protein mRNA, complete cds
AT3G47170	-4.954451739	4.42E-05	Arabidopsis thaliana HXXXD-type acyl-transferase family protein mRNA, complete cds
AT4G17100	-2.147096817	4.67E-05	Arabidopsis thaliana uncharacterized protein mRNA, complete cds
AT1G78520	-2.147096817	4.67E-05	Arabidopsis thaliana carbohydrate-binding X8 domain-containing protein mRNA, complete cds
AT5G55680	-2.147096817	4.67E-05	Arabidopsis thaliana chromosome 5 sequence
AT5G25260	-1.880451157	4.71E-05	Arabidopsis thaliana SPFH/Band 7/PHB domain-containing membrane-associated protein mRNA, complete cds
AT5G42600	-1.880451157	4.71E-05	Arabidopsis thaliana marneral synthase mRNA, complete cds
AT5G42460	-1.217486144	4.74E-05	Arabidopsis thaliana chromosome 5 sequence
AT2G35585	-1.954451739	4.82E-05	Arabidopsis thaliana uncharacterized protein mRNA, complete cds
AT4G15070	-1.015852283	4.93E-05	Arabidopsis thaliana cysteine/histidine-rich C1

			domain-containing protein mRNA, complete cds
AT5G46220	-1.461411727	5.03E-05	Arabidopsis thaliana uncharacterized protein mRNA, complete cds
AT2G42260	-1.461411727	5.03E-05	Arabidopsis thaliana mRNA for hypothetical protein, complete cds, clone: RAFL14-42-A06
AT4G16563	-1.377663169	5.05E-05	Arabidopsis thaliana chromosome 4 sequence
AT2G20724	-1.098841648	5.16E-05	Arabidopsis thaliana chromosome 2, complete sequence
AT3G52670	-1.602149995	5.20E-05	Arabidopsis thaliana FBD, F-box, Skp2-like and leucine Rich Repeat domains containing protein mRNA, complete cds
AT1G23100	-1.602149995	5.20E-05	Arabidopsis thaliana GroES-like protein mRNA, complete cds
AT4G34880	-1.602149995	5.20E-05	Arabidopsis thaliana amidase family protein mRNA, complete cds
AT1G19340	-1.265653427	5.21E-05	Arabidopsis thaliana methyltransferase-like protein 2 mRNA, complete cds
AT3G52970	-3.276379833	5.37E-05	Arabidopsis thaliana cytochrome P450, family 76, subfamily G, polypeptide 1 mRNA, complete cds
AT3G55310	-3.276379833	5.37E-05	Arabidopsis thaliana Rossmann-fold NAD(P)-binding domain-containing protein mRNA, complete cds
AT5G22560	-3.276379833	5.37E-05	Arabidopsis thaliana chromosome 5 sequence
AT5G07150	-1.22575376	5.51E-05	Arabidopsis thaliana leucine-rich repeat protein kinase-like protein mRNA, complete cds
AT1G53990	-1.323685548	5.58E-05	Arabidopsis thaliana GDSL motif lipase 3 mRNA, complete cds
AT5G63270	-2.598307928	5.64E-05	Arabidopsis thaliana RPM1-interacting protein 4 family protein mRNA, complete cds
AT3G44700	-2.598307928	5.64E-05	Arabidopsis thaliana uncharacterized protein mRNA, complete cds
AT1G04660	-2.598307928	5.64E-05	Arabidopsis thaliana chromosome 1 sequence
AT3G10600	-1.484966455	5.67E-05	Arabidopsis thaliana cationic amino acid transporter 7 mRNA, complete cds

AT1G14205	-1.190079987	5.74E-05	Arabidopsis thaliana ribosomal L18p/L5e family protein mRNA, complete cds
AT4G01930	-4.04191458	5.75E-05	Arabidopsis thaliana chromosome 4 sequence
AT5G59470	-1.017187494	5.80E-05	Arabidopsis thaliana mannose-P-dolichol utilization defect 1 protein mRNA, complete cds
AT1G29740	-1.017187494	5.80E-05	Arabidopsis thaliana Leucine-rich repeat transmembrane protein kinase mRNA, complete cds
AT4G10280	-1.056331353	6.00E-05	Arabidopsis thaliana cupin domain-containing protein mRNA, complete cds
AT1G51380	-1.276379833	6.04E-05	Arabidopsis thaliana DEAD-box ATP-dependent RNA helicase 34 mRNA, complete cds
AT1G10460	-1.078440456	6.05E-05	Arabidopsis thaliana chromosome 1 sequence
AT3G55390	-1.102550378	6.05E-05	Arabidopsis thaliana uncharacterized protein mRNA, complete cds
AT1G14600	-1.680276775	6.21E-05	Arabidopsis thaliana putative Myb family transcription factor mRNA, complete cds
AT3G61950	-1.000745391	6.66E-05	Arabidopsis thaliana transcription factor bHLH67 mRNA, complete cds
AT2G30766	-1.727041243	6.70E-05	Arabidopsis thaliana chromosome 2, complete sequence
AT1G43020	-1.727041243	6.70E-05	Arabidopsis thaliana uncharacterized protein mRNA, complete cds
AT3G04320	-1.163905104	6.90E-05	Arabidopsis thaliana chromosome 3, complete sequence
AT2G33793	-1.037867747	6.96E-05	Arabidopsis thaliana uncharacterized protein mRNA, complete cds
AT2G23470	-1.037867747	6.96E-05	Arabidopsis thaliana protein ROOT UV-B SENSITIVE 4 mRNA, complete cds
AT2G33750	-2.227470233	7.33E-05	Arabidopsis thaliana purine permease 2 mRNA, complete cds
AT1G27880	-1.353001115	7.38E-05	Arabidopsis thaliana ATP-dependent DNA helicase Q-like 5 mRNA, complete cds
AT3G12170	-1.353001115	7.38E-05	Arabidopsis thaliana chaperone DnaJ-domain containing protein mRNA, complete cds

AT5G54064	-2.101293127	7.71E-05	Arabidopsis thaliana chromosome 5 sequence
AT3G27510	-2.101293127	7.71E-05	Arabidopsis thaliana chromosome 3, complete sequence
AT5G46130	-2.828920857	7.74E-05	Arabidopsis thaliana uncharacterized protein mRNA, complete cds
AT1G19510	-1.913809754	7.75E-05	Arabidopsis thaliana protein RADIALIS-like 5 mRNA, complete cds
AT4G12450	-1.913809754	7.75E-05	Arabidopsis thaliana chromosome 4 sequence
AT4G12900	-1.571123099	7.92E-05	Arabidopsis thaliana Gamma interferon responsive lysosomal thiol (GILT) reductase family protein mRNA, complete cds
AT3G13403	-4.861342334	8.04E-05	Arabidopsis thaliana defensin-like protein 302 mRNA, complete cds
AT5G39630	-4.861342334	8.04E-05	Arabidopsis thaliana vesicle transport v-SNARE family protein mRNA, complete cds
AT4G08400	-4.861342334	8.04E-05	Arabidopsis thaliana chromosome 4 sequence
AT3G02430	-4.861342334	8.04E-05	Arabidopsis thaliana chromosome 3, complete sequence
AT4G28620	-4.861342334	8.04E-05	Arabidopsis thaliana ABC transporter B family member 24 mRNA, complete cds
AT4G21260	-4.861342334	8.04E-05	Arabidopsis thaliana sulfite exporter TauE/SafE family protein mRNA, complete cds
AT4G25190	-4.861342334	8.04E-05	Arabidopsis thaliana uncharacterized protein mRNA, complete cds
AT1G24430	-1.17018043	8.07E-05	Arabidopsis thaliana chromosome 1 sequence
AT5G59070	-1.061366943	8.29E-05	Arabidopsis thaliana glycosyl transferase family protein mRNA, complete cds
AT2G23100	-1.369489238	8.46E-05	Arabidopsis thaliana chromosome 2, complete sequence
AT2G18470	-1.456952079	8.46E-05	Arabidopsis thaliana proline-rich extensin-like receptor kinase 4 mRNA, complete cds
AT4G12470	-1.646329443	9.59E-05	Arabidopsis thaliana chromosome 4 sequence
AT3G03840	-1.646329443	9.59E-05	Arabidopsis thaliana chromosome 3, complete sequence
AT5G65500	-3.202379252	9.73E-05	Arabidopsis thaliana U-box

			domain-containing protein kinase family protein mRNA, complete cds
AT1G51830	-3.202379252	9.73E-05	Arabidopsis thaliana putative leucine-rich repeat protein kinase mRNA, complete cds
AT1G26100	-1.06407623	9.75E-05	Arabidopsis thaliana probable transmembrane ascorbate ferric reductase 4 mRNA, complete cds
AT1G09610	-1.691417333	0.000104199	Arabidopsis thaliana chromosome 1 sequence
AT4G35390	-1.691417333	0.000104199	Arabidopsis thaliana chromosome 4 sequence
AT5G18910	-3.954451739	0.000105915	Arabidopsis thaliana protein kinase family protein mRNA, complete cds
AT1G09860	-3.954451739	0.000105915	Arabidopsis thaliana chromosome 1 sequence
AT3G06100	-3.954451739	0.000105915	Arabidopsis thaliana putative aquaporin NIP7-1 mRNA, complete cds
AT5G43230	-3.954451739	0.000105915	Arabidopsis thaliana uncharacterized protein mRNA, complete cds
AT5G16340	-1.327910134	0.000107372	Arabidopsis thaliana chromosome 5 sequence
AT2G28870	-1.003920415	0.000108671	Arabidopsis thaliana chromosome 2, complete sequence
AT1G77230	-1.023164489	0.000111272	Arabidopsis thaliana tetratricopeptide repeat-containing protein mRNA, complete cds
AT5G06740	-1.150002548	0.000112956	Arabidopsis thaliana chromosome 5 sequence
AT4G37580	-1.044088951	0.000113287	Arabidopsis thaliana putative N-acetyltransferase mRNA, complete cds
AT1G04425	-1.044088951	0.000113287	Arabidopsis thaliana clone 23398 mRNA sequence
AT2G43465	-1.119510985	0.000114565	Arabidopsis thaliana RNA-binding ASCH domain protein mRNA, complete cds
AT5G08070	-1.091955262	0.000115066	Arabidopsis thaliana chromosome 5 sequence
AT1G76430	-1.802448645	0.000118909	Arabidopsis thaliana putative inorganic phosphate transporter 1-9 mRNA, complete cds
AT5G07720	-1.343494029	0.000123531	Arabidopsis thaliana chromosome 5 sequence
AT1G48500	-1.428382927	0.000125664	Arabidopsis thaliana protein TIFY 6A

			mRNA, complete cds
AT1G78780	-1.428382927	0.000125664	Arabidopsis thaliana pathogenesis-related protein mRNA, complete cds
AT3G06435	-1.428382927	0.000125664	Arabidopsis thaliana chromosome 3, complete sequence
AT2G19990	-2.053987412	0.00012681	Arabidopsis thaliana chromosome 2, complete sequence
AT5G58784	-2.053987412	0.00012681	Arabidopsis thaliana dehydrodolichyl diphosphate synthase 5 mRNA, complete cds
AT4G01575	-2.053987412	0.00012681	Arabidopsis thaliana chromosome 4 sequence
AT4G23720	-1.954451739	0.000127119	Arabidopsis thaliana chromosome 4 sequence
AT1G63930	-1.954451739	0.000127119	Arabidopsis thaliana chromosome 1 sequence
AT3G09660	-1.191490936	0.000128577	Arabidopsis thaliana minichromosome maintenance 8 mRNA, complete cds
AT1G11120	-1.024841066	0.000131014	Arabidopsis thaliana uncharacterized protein mRNA, complete cds
AT1G55205	-1.573361571	0.000133441	Arabidopsis thaliana uncharacterized protein mRNA, complete cds
AT5G19060	-1.573361571	0.000133441	Arabidopsis thaliana chromosome 5 sequence
AT1G60130	-1.573361571	0.000133441	Arabidopsis thaliana Mannose-binding lectin superfamily protein mRNA, complete cds
AT3G20590	-1.573361571	0.000133441	Arabidopsis thaliana chromosome 3, complete sequence
AT3G60580	-1.573361571	0.000133441	Arabidopsis thaliana chromosome 3, complete sequence
AT4G00905	-1.069928956	0.000134746	Arabidopsis thaliana NC domain-containing protein-like protein mRNA, complete cds
AT5G56795	-2.761806661	0.000136807	Arabidopsis thaliana metallothionein 1B mRNA, complete cds
AT5G51520	-1.241332886	0.000143372	Arabidopsis thaliana chromosome 5 sequence
AT5G64110	-1.241332886	0.000143372	Arabidopsis thaliana peroxidase 70 mRNA, complete cds
AT5G62330	-4.761806661	0.000146751	Arabidopsis thaliana uncharacterized protein mRNA, complete cds
AT1G19840	-1.00629067	0.000150789	Arabidopsis thaliana chromosome 1 sequence

AT3G60660	-1.478013695	0.000161021	Arabidopsis thaliana spindle and kinetochore-associated protein 1-like protein mRNA, complete cds
AT3G03820	-1.378949567	0.000162534	Arabidopsis thaliana chromosome 3, complete sequence
AT5G13900	-2.478013695	0.000168738	Arabidopsis thaliana lipid transfer-like protein VAS mRNA, complete cds
AT5G01120	-3.12437674	0.000175951	Arabidopsis thaliana uncharacterized protein mRNA, complete cds
AT1G13500	-3.12437674	0.000175951	Arabidopsis thaliana uncharacterized protein mRNA, complete cds
AT5G38790	-3.12437674	0.000175951	Arabidopsis thaliana chromosome 5 sequence
AT2G43390	-3.12437674	0.000175951	Arabidopsis thaliana chromosome 2, complete sequence
AT3G19320	-3.12437674	0.000175951	Arabidopsis thaliana leucine-rich repeat-containing protein mRNA, complete cds
AT1G68510	-3.12437674	0.000175951	Arabidopsis thaliana LOB domain-containing protein 42 mRNA, complete cds
AT1G34540	-3.12437674	0.000175951	Arabidopsis thaliana chromosome 1 sequence
AT5G06430	-1.007563075	0.000177665	Arabidopsis thaliana thioredoxin-related protein mRNA, complete cds
AT1G74453	-1.317021818	0.000179575	Arabidopsis thaliana chromosome 1 sequence
AT2G28440	-1.506992762	0.000181119	Arabidopsis thaliana chromosome 2, complete sequence
AT5G25750	-1.506992762	0.000181119	Arabidopsis thaliana uncharacterized protein mRNA, complete cds
AT2G26520	-1.506992762	0.000181119	Arabidopsis thaliana chromosome 2, complete sequence
AT4G31805	-1.02845232	0.000181705	Arabidopsis thaliana protein POLAR mRNA, complete cds
AT2G38430	-1.051313278	0.00018467	Arabidopsis thaliana uncharacterized protein mRNA, complete cds
AT1G11915	-1.399236581	0.000185763	Arabidopsis thaliana uncharacterized protein mRNA, complete cds
AT1G47440	-2.276379833	0.000190821	Arabidopsis thaliana chromosome 1 sequence
AT5G36870	-2.276379833	0.000190821	Arabidopsis thaliana callose synthase 4 mRNA, complete cds
AT5G61360	-1.332963362	0.000206975	Arabidopsis thaliana uncharacterized

			protein mRNA, complete cds
AT5G14340	-2.005077812	0.000207476	Arabidopsis thaliana myb domain protein 40 mRNA, complete cds
AT1G06930	-2.005077812	0.000207476	Arabidopsis thaliana chromosome 1 sequence
AT5G25380	-2.005077812	0.000207476	Arabidopsis thaliana cyclin a2;1 mRNA, complete cds
AT1G70340	-1.008899523	0.000209368	Arabidopsis thaliana uncharacterized protein mRNA, complete cds
AT4G33100	-1.030400592	0.000214036	Arabidopsis thaliana uncharacterized protein mRNA, complete cds
AT5G65510	-1.079982621	0.000219136	Arabidopsis thaliana AINTEGUMENTA-like 7 protein mRNA, complete cds
AT5G60100	-1.276379833	0.000224344	Arabidopsis thaliana pseudo-response regulator 3 mRNA, complete cds
AT2G39920	-1.575940115	0.000225365	Arabidopsis thaliana acid phosphatase class IIIB protein mRNA, complete cds
AT5G14130	-1.575940115	0.000225365	Arabidopsis thaliana peroxidase 55 mRNA, complete cds
AT4G21220	-1.227470233	0.000237838	Arabidopsis thaliana trimeric LpxA-like protein mRNA, complete cds
AT5G24040	-2.691417333	0.000241028	Arabidopsis thaliana uncharacterized protein mRNA, complete cds
AT2G29620	-2.691417333	0.000241028	Arabidopsis thaliana uncharacterized protein mRNA, complete cds
AT1G49910	-1.032454251	0.000252157	Arabidopsis thaliana cell cycle arrest protein BUB3 mRNA, complete cds
AT1G70350	-1.147096817	0.000253949	Arabidopsis thaliana uncharacterized protein mRNA, complete cds
AT2G17036	-1.147096817	0.000253949	Arabidopsis thaliana F-box protein mRNA, complete cds
AT3G46950	-1.083734756	0.000257724	Arabidopsis thaliana chromosome 3, complete sequence
AT5G65320	-1.29005477	0.000259872	Arabidopsis thaliana transcription factor bHLH99 mRNA, complete cds
AT5G36270	-4.654891457	0.000269336	Arabidopsis thaliana chromosome 5 sequence
AT1G69990	-4.654891457	0.000269336	Arabidopsis thaliana chromosome 1 sequence
AT2G22950	-4.654891457	0.000269336	Arabidopsis thaliana putative calcium-transporting ATPase 7 mRNA, complete cds
AT1G02570	-4.654891457	0.000269336	Arabidopsis thaliana uncharacterized

			protein mRNA, complete cds
AT1G70450	-4.654891457	0.000269336	Arabidopsis thaliana protein kinase superfamily protein mRNA, complete cds
AT4G12270	-4.654891457	0.000269336	Arabidopsis thaliana copper amine oxidase family protein mRNA, complete cds
AT3G18880	-1.473825898	0.000271994	Arabidopsis thaliana Nucleic acid-binding, OB-fold-like protein mRNA, complete cds
AT3G20935	-1.193238598	0.000289378	Arabidopsis thaliana cytochrome P450, family 705, subfamily A, polypeptide 28 mRNA, complete cds
AT2G20700	-2.413883357	0.000290209	Arabidopsis thaliana LORELEI-like glucosylphosphatidylinositol-anchored protein 2 mRNA, complete cds
AT1G04070	-1.011784914	0.000290912	Arabidopsis thaliana chromosome 1 sequence
AT3G11150	-1.719986485	0.000294376	Arabidopsis thaliana 2-oxoglutarate (2OG) and Fe(II)-dependent oxygenase superfamily protein mRNA, complete cds
AT3G05740	-1.304948986	0.000300606	Arabidopsis thaliana ATP-dependent DNA helicase Q-like 1 mRNA, complete cds
AT5G55830	-1.087718269	0.000303119	Arabidopsis thaliana chromosome 5 sequence
AT1G54540	-1.087718269	0.000303119	Arabidopsis thaliana chromosome 1 sequence
AT5G03545	-1.390550853	0.00031294	Arabidopsis thaliana chromosome 5 sequence
AT5G04390	-3.04191458	0.000317692	Arabidopsis thaliana chromosome 5 sequence
AT2G14440	-3.04191458	0.000317692	Arabidopsis thaliana putative leucine-rich repeat receptor-like serine/threonine-protein kinase mRNA, complete cds
AT1G01453	-3.04191458	0.000317692	Arabidopsis thaliana chromosome 1 sequence
AT4G12440	-2.217486144	0.000321361	Arabidopsis thaliana adenine phosphoribosyl transferase 4 mRNA, complete cds
AT1G24420	-2.217486144	0.000321361	Arabidopsis thaliana chromosome 1 sequence

AT5G09670	-2.217486144	0.000321361	Arabidopsis thaliana chromosome 5 sequence
AT2G35210	-1.249907622	0.000322117	Arabidopsis thaliana root and pollen arfgap mRNA, complete cds
AT4G01240	-1.249907622	0.000322117	Arabidopsis thaliana putative methyltransferase mRNA, complete cds
AT1G52530	-1.861342334	0.000329397	Arabidopsis thaliana Hus1 domain-containing protein mRNA, complete cds
AT3G18217	-1.861342334	0.000329397	Arabidopsis thaliana chromosome 3, complete sequence
AT1G71760	-2.069928956	0.000336172	Arabidopsis thaliana uncharacterized protein mRNA, complete cds
AT5G61550	-1.539414239	0.000342919	Arabidopsis thaliana U-box domain-containing protein 52 mRNA, complete cds
AT4G31620	-1.013345428	0.000343013	Arabidopsis thaliana transcriptional factor B3 family protein mRNA, complete cds
AT2G34010	-1.321234069	0.000347164	Arabidopsis thaliana uncharacterized protein mRNA, complete cds
AT4G22560	-1.160902616	0.000348699	Arabidopsis thaliana chromosome 4 sequence
AT2G35910	-1.036913899	0.000350143	Arabidopsis thaliana chromosome 2, complete sequence
AT5G16950	-1.12437674	0.000354675	Arabidopsis thaliana chromosome 5 sequence
AT2G02300	-3.761806661	0.000361511	Arabidopsis thaliana phloem protein 2-B5 mRNA, complete cds
AT1G53635	-3.761806661	0.000361511	Arabidopsis thaliana chromosome 1 sequence
AT2G19150	-3.761806661	0.000361511	Arabidopsis thaliana putative pectinesterase 10 mRNA, complete cds
AT1G73160	-1.578942604	0.000381401	Arabidopsis thaliana chromosome 1 sequence
AT5G02140	-1.339115589	0.000400185	Arabidopsis thaliana pathogenesis-related thaumatin-like protein mRNA, complete cds
AT2G45695	-1.339115589	0.000400185	Arabidopsis thaliana ubiquitin-related modifier 1-1 mRNA, complete cds
AT1G72690	-1.439878566	0.000406402	Arabidopsis thaliana uncharacterized protein mRNA, complete cds
AT1G15885	-1.168576544	0.000408431	Arabidopsis thaliana uncharacterized protein mRNA, complete cds

AT5G13990	-1.130301574	0.00041644	Arabidopsis thaliana chromosome 5 sequence
AT3G04370	-2.617416751	0.000423222	Arabidopsis thaliana plasmodesmata-located protein 4 mRNA, complete cds
AT3G47770	-2.617416751	0.000423222	Arabidopsis thaliana ABC transporter A family member 6 mRNA, complete cds
AT1G44740	-1.676917763	0.000459522	Arabidopsis thaliana uncharacterized protein mRNA, complete cds
AT3G10470	-1.676917763	0.000459522	Arabidopsis thaliana chromosome 3, complete sequence
AT1G34245	-1.676917763	0.000459522	Arabidopsis thaliana protein EPIDERMAL PATTERNING FACTOR 2 mRNA, complete cds
AT1G26390	-1.016736017	0.000477164	Arabidopsis thaliana chromosome 1 sequence
AT3G19440	-1.016736017	0.000477164	Arabidopsis thaliana RNA pseudourine synthase 4 mRNA, complete cds
AT3G46020	-1.04191458	0.000486523	Arabidopsis thaliana RNA recognition motif-containing protein mRNA, complete cds
AT1G66810	-1.13665507	0.000488894	Arabidopsis thaliana zinc finger CCCH domain-containing protein mRNA, complete cds
AT2G17620	-1.13665507	0.000488894	Arabidopsis thaliana cyclin-B2-1 mRNA, complete cds
AT1G20470	-1.13665507	0.000488894	Arabidopsis thaliana chromosome 1 sequence
AT5G25570	-1.101293127	0.000493235	Arabidopsis thaliana uncharacterized protein mRNA, complete cds
AT1G33760	-2.346769161	0.000496953	Arabidopsis thaliana chromosome 1 sequence
AT2G45580	-2.346769161	0.000496953	Arabidopsis thaliana cytochrome P450 76C3 mRNA, complete cds
AT3G29252	-4.539414239	0.000496992	Arabidopsis thaliana chromosome 3, complete sequence
AT5G02000	-4.539414239	0.000496992	Arabidopsis thaliana chromosome 5 sequence
AT3G20557	-4.539414239	0.000496992	Arabidopsis thaliana uncharacterized protein mRNA, complete cds
AT1G44030	-4.539414239	0.000496992	Arabidopsis thaliana chromosome 1 sequence
AT2G17060	-1.812432734	0.000526024	Arabidopsis thaliana TIR-NBS-LRR class disease resistance protein mRNA,

			complete cds
AT5G24240	-1.812432734	0.000526024	Arabidopsis thaliana phosphatidylinositol 3- and 4-kinase / ubiquitin family protein mRNA, complete cds
AT4G26960	-1.380716493	0.000528091	Arabidopsis thaliana uncharacterized protein mRNA, complete cds
AT3G01070	-1.234559658	0.000536648	Arabidopsis thaliana early nodulin-like protein 16 mRNA, complete cds
AT3G30737.1	-2.1560856	0.000538547	Arabidopsis thaliana chromosome 3, complete sequence
AT2G45040	-2.1560856	0.000538547	Arabidopsis thaliana chromosome 2, complete sequence
AT5G01420	-1.901984319	0.000546859	Arabidopsis thaliana chromosome 5 sequence
AT1G29830	-1.901984319	0.000546859	Arabidopsis thaliana Magnesium transporter CorA-like family protein mRNA, complete cds
AT2G18480	-2.013345428	0.000553047	Arabidopsis thaliana putative polyol transporter 3 mRNA, complete cds
AT5G64800	-2.013345428	0.000553047	Arabidopsis thaliana chromosome 5 sequence
AT1G10155	-1.185777285	0.000559722	Arabidopsis thaliana phloem protein 2-A10 mRNA, complete cds
AT4G33880	-1.073750667	0.000579712	Arabidopsis thaliana mRNA for putative bHLH transcription factor, complete cds, clone: RAFL14-58-P18
AT4G21065	-1.539414239	0.000581615	Arabidopsis thaliana chromosome 4 sequence
AT3G48540	-1.539414239	0.000581615	Arabidopsis thaliana cytidine/deoxycytidylate deaminase family protein mRNA, complete cds
AT5G58750	-1.308088693	0.000583005	Arabidopsis thaliana chromosome 5 sequence
AT4G28530	-1.405113148	0.000604041	Arabidopsis thaliana NAC domain containing protein 74 mRNA, complete cds
AT5G05840	-1.582482961	0.000647008	Arabidopsis thaliana uncharacterized protein mRNA, complete cds
AT5G46370	-1.195459838	0.000654787	Arabidopsis thaliana two-pore potassium channel 2 mRNA, complete cds
AT5G51580	-1.020540929	0.000664348	Arabidopsis thaliana chromosome 5 sequence
AT1G35270	-3.654891457	0.000669645	Arabidopsis thaliana chromosome 1 sequence

AT1G65481	-3.654891457	0.000669645	Arabidopsis thaliana uncharacterized protein mRNA, complete cds
AT1G73780	-3.654891457	0.000669645	Arabidopsis thaliana chromosome 1 sequence
AT5G16330	-3.654891457	0.000669645	Arabidopsis thaliana NC domain-containing protein-like protein mRNA, complete cds
AT1G77870	-3.654891457	0.000669645	Arabidopsis thaliana membrane-anchored ubiquitin-fold protein 5 mRNA, complete cds
AT2G02980	-1.111993016	0.000682363	Arabidopsis thaliana chromosome 2, complete sequence
AT1G67260	-1.632523644	0.000713278	Arabidopsis thaliana transcription factor TCP1 mRNA, complete cds
AT1G33475	-1.261113077	0.000727157	Arabidopsis thaliana chromosome 1 sequence
AT5G06590	-1.261113077	0.000727157	Arabidopsis thaliana uncharacterized protein mRNA, complete cds
AT5G18730	-2.539414239	0.000740331	Arabidopsis thaliana uncharacterized protein mRNA, complete cds
AT1G62262	-2.539414239	0.000740331	Arabidopsis thaliana SLAC1 homologue 4 mRNA, complete cds
AT3G24400	-1.346769161	0.000776502	Arabidopsis thaliana chromosome 3, complete sequence
AT3G03650	-1.346769161	0.000776502	Arabidopsis thaliana Exostosin family protein mRNA, complete cds
AT2G32630	-1.346769161	0.000776502	Arabidopsis thaliana chromosome 2, complete sequence
AT4G15360	-1.463465386	0.000781232	Arabidopsis thaliana cytochrome P450, family 705, subfamily A, polypeptide 3 mRNA, complete cds
AT2G20710	-1.022623241	0.000784166	Arabidopsis thaliana chromosome 2, complete sequence
AT1G31835	-1.276379833	0.00084494	Arabidopsis thaliana chromosome 1 sequence
AT3G52550	-1.276379833	0.00084494	Arabidopsis thaliana chromosome 3, complete sequence
AT3G13432	-2.276379833	0.000846911	Arabidopsis thaliana uncharacterized protein mRNA, complete cds
AT3G22820	-2.276379833	0.000846911	Arabidopsis thaliana allergen-related protein mRNA, complete cds
AT1G51880	-2.276379833	0.000846911	Arabidopsis thaliana protein ROOT HAIR SPECIFIC 6 mRNA, complete cds
AT1G51480	-2.276379833	0.000846911	Arabidopsis thaliana CC-NBS-LRR class

			disease resistance protein mRNA, complete cds
AT5G11290	-1.847536535	0.000880382	Arabidopsis thaliana chromosome 5 sequence
AT4G35130	-1.498772255	0.000881902	Arabidopsis thaliana chromosome 4 sequence
AT3G48970	-1.369489238	0.000892897	Arabidopsis thaliana Heavy metal transport/detoxification superfamily protein mRNA, complete cds
AT3G55515	-2.091955262	0.000897709	Arabidopsis thaliana chromosome 3, complete sequence
AT1G15540	-4.413883357	0.000922314	Arabidopsis thaliana 2-oxoglutarate (2OG) and Fe(II)-dependent oxygenase-like protein mRNA, complete cds
AT2G31082	-4.413883357	0.000922314	Arabidopsis thaliana chromosome 2, complete sequence
AT1G33920	-4.413883357	0.000922314	Arabidopsis thaliana phloem protein 2-A4 mRNA, complete cds
AT3G51030	-1.053987412	0.000941301	Arabidopsis thaliana thioredoxin H1 mRNA, complete cds
AT5G15265	-1.12437674	0.000943856	Arabidopsis thaliana uncharacterized protein mRNA, complete cds
AT3G62050	-1.086902035	0.000947984	Arabidopsis thaliana putative endonuclease or glycosyl hydrolase mRNA, complete cds
AT3G15720	-1.086902035	0.000947984	Arabidopsis thaliana probable polygalacturonase mRNA, complete cds
AT1G50040	-1.293253652	0.000980355	Arabidopsis thaliana uncharacterized protein mRNA, complete cds
AT2G22410	-1.293253652	0.000980355	Arabidopsis thaliana chromosome 2, complete sequence
AT3G30350	-1.539414239	0.000989156	Arabidopsis thaliana protein ROOT MERISTEM GROWTH FACTOR 4 mRNA, complete cds
AT1G68930	-1.539414239	0.000989156	Arabidopsis thaliana chromosome 1 sequence
AT3G22690	-1.39502433	0.001023603	Arabidopsis thaliana protein YELLOW SEEDLINGS 1 mRNA, complete cds
AT2G46192	-1.39502433	0.001023603	Arabidopsis thaliana chromosome 2, complete sequence
AT2G35890	-2.861342334	0.001029379	Arabidopsis thaliana calcium-dependent protein kinase 25 mRNA, complete cds

AT1G79250	-2.861342334	0.001029379	Arabidopsis thaliana AGC kinase 1.7 mRNA, complete cds
AT5G54790	-2.861342334	0.001029379	Arabidopsis thaliana uncharacterized protein mRNA, complete cds
AT1G67150	-1.027208081	0.001093324	Arabidopsis thaliana uncharacterized protein mRNA, complete cds
AT4G10600	-1.586719954	0.001100602	Arabidopsis thaliana chromosome 4 sequence
AT2G26211	-1.131329501	0.001109932	Arabidopsis thaliana chromosome 2, complete sequence
AT1G67650	-1.131329501	0.001109932	Arabidopsis thaliana SRP72 RNA-binding domain protein mRNA, complete cds
AT4G01580	-1.131329501	0.001109932	Arabidopsis thaliana AP2/B3 domain-containing protein mRNA, complete cds
AT4G00960	-1.312003743	0.001135483	Arabidopsis thaliana protein kinase family protein mRNA, complete cds
AT5G61800	-1.423937022	0.001169112	Arabidopsis thaliana chromosome 5 sequence
ATMG00690	-1.642507732	0.001212275	Arabidopsis thaliana mitochondrial ORF240a, ecotype Kz-9
AT5G46060	-1.243958356	0.00121799	Arabidopsis thaliana uncharacterized protein mRNA, complete cds
AT1G09400	-3.539414239	0.001242361	Arabidopsis thaliana putative 12-oxophytodienoate reductase-like protein 1 mRNA, complete cds
AT5G24200	-3.539414239	0.001242361	Arabidopsis thaliana uncharacterized protein mRNA, complete cds
AT5G09805	-3.539414239	0.001242361	Arabidopsis thaliana chromosome 5 sequence
AT1G53610	-3.539414239	0.001242361	Arabidopsis thaliana uncharacterized protein mRNA, complete cds
AT2G14760	-3.539414239	0.001242361	Arabidopsis thaliana transcription factor bHLH84 mRNA, complete cds
AT2G02580	-3.539414239	0.001242361	Arabidopsis thaliana cytochrome P450 71B9 mRNA, complete cds
AT2G31930	-3.539414239	0.001242361	Arabidopsis thaliana chromosome 2, complete sequence
AT1G11112	-3.539414239	0.001242361	Arabidopsis thaliana chromosome 1 sequence
AT3G48630	-3.539414239	0.001242361	Arabidopsis thaliana uncharacterized protein mRNA, complete cds
AT1G24290	-1.001757453	0.001261386	Arabidopsis thaliana chromosome 1

			sequence
AT4G14723	-2.456952079	0.001289518	Arabidopsis thaliana epidermal patterning factor-like protein 4 mRNA, complete cds
AT5G44900	-2.456952079	0.001289518	Arabidopsis thaliana Toll-Interleukin-Resistance domain-containing protein mRNA, complete cds
AT5G22608	-1.061366943	0.001310749	Arabidopsis thaliana uncharacterized protein mRNA, complete cds
AT3G46800	-1.332963362	0.001312402	Arabidopsis thaliana chromosome 3, complete sequence
AT5G39110	-1.332963362	0.001312402	Arabidopsis thaliana germin-like protein subfamily 1 member 14 mRNA, complete cds
AT1G13150	-1.097409692	0.001316344	Arabidopsis thaliana chromosome 1 sequence
AT2G26220	-1.709339241	0.001318012	Arabidopsis thaliana chromosome 2, complete sequence
AT2G31920	-1.456952079	0.001329293	Arabidopsis thaliana uncharacterized protein mRNA, complete cds
AT3G12910	-1.456952079	0.001329293	Arabidopsis thaliana no apical meristem domain-containing transcriptional regulator mRNA, complete cds
AT5G46650	-1.790953006	0.001408657	Arabidopsis thaliana chromosome 5 sequence
AT5G06470	-1.25930632	0.001419002	Arabidopsis thaliana chromosome 5 sequence
AT1G10680	-2.202379252	0.001435705	Arabidopsis thaliana ABC transporter B family member 10 mRNA, complete cds
AT3G54070	-1.893051194	0.001471162	Arabidopsis thaliana ankyrin repeat-containing protein mRNA, complete cds
AT2G45120	-2.024841066	0.001487743	Arabidopsis thaliana chromosome 2, complete sequence
AT3G58865.1	-1.198377321	0.001491608	Arabidopsis thaliana chromosome 3, complete sequence
AT1G54530	-1.49502012	0.001503023	Arabidopsis thaliana chromosome 1 sequence
AT5G12880	-1.356550182	0.001513055	Arabidopsis thaliana chromosome 5 sequence
AT3G09730	-1.356550182	0.001513055	Arabidopsis thaliana uncharacterized protein mRNA, complete cds
AT1G67105	-1.276379833	0.001650937	Arabidopsis thaliana chromosome 1

			sequence
AT3G18630	-1.539414239	0.001687647	Arabidopsis thaliana uracil DNA glycosylase mRNA, complete cds
ATMG01370	-1.539414239	0.001687647	Arabidopsis thaliana ecotype Landsberg erecta mitochondrion, complete genome
AT4G09780	-4.276379833	0.001721979	Arabidopsis thaliana TRAF-like family protein mRNA, complete cds
AT5G45105	-4.276379833	0.001721979	Arabidopsis thaliana zinc transporter 8 precursor mRNA, complete cds
AT2G13422	-4.276379833	0.001721979	Arabidopsis thaliana chromosome 2, complete sequence
AT5G32621.1	-4.276379833	0.001721979	Arabidopsis thaliana chromosome 5 sequence
AT5G26070	-4.276379833	0.001721979	Arabidopsis thaliana chromosome 5 sequence
AT3G46420	-4.276379833	0.001721979	Arabidopsis thaliana leucine-rich repeat protein kinase-like protein mRNA, complete cds
AT2G05910	-1.383295037	0.001739026	Arabidopsis thaliana uncharacterized protein mRNA, complete cds
AT1G27285.1	-1.1560856	0.00180321	Arabidopsis thaliana chromosome 1 sequence
AT5G40942	-1.109729964	0.001828208	Arabidopsis thaliana chromosome 5 sequence
AT5G61280	-2.761806661	0.001845616	Arabidopsis thaliana Remorin family protein mRNA, complete cds
AT3G44840	-2.761806661	0.001845616	Arabidopsis thaliana S-adenosyl-L-methionine-dependent methyltransferases superfamily protein mRNA, complete cds
AT1G30835.2	-1.591881659	0.001878221	Arabidopsis thaliana chromosome 1 sequence
AT4G34970	-1.295488656	0.00191762	Arabidopsis thaliana actin depolymerizing factor 9 mRNA, complete cds
AT1G21326	-1.295488656	0.00191762	Arabidopsis thaliana chromosome 1 sequence
AT4G25760	-1.413883357	0.001991196	Arabidopsis thaliana chromosome 4 sequence
AT3G28840	-1.413883357	0.001991196	Arabidopsis thaliana uncharacterized protein mRNA, complete cds
AT5G54020	-1.654891457	0.002066442	Arabidopsis thaliana chromosome 5 sequence
AT1G21510	-1.654891457	0.002066442	Arabidopsis thaliana chromosome 1

			sequence
AT2G37195	-1.006919158	0.002086328	Arabidopsis thaliana uncharacterized protein mRNA, complete cds
AT5G59360	-1.074745972	0.002156855	Arabidopsis thaliana chromosome 5 sequence
AT5G05810	-1.317021818	0.002222915	Arabidopsis thaliana chromosome 5 sequence
AT2G42365	-1.317021818	0.002222915	Arabidopsis thaliana mRNA for unknown protein, complete cds, clone: RAFL17-34-N18
AT5G23980	-1.317021818	0.002222915	Arabidopsis thaliana ferric reduction oxidase 4 mRNA, complete cds
AT2G35612	-2.369489238	0.002235263	Arabidopsis thaliana chromosome 2, complete sequence
AT2G11810	-2.369489238	0.002235263	Arabidopsis thaliana Monogalactosyldiacylglycerol synthase 3 mRNA, complete cds
AT1G75920	-2.369489238	0.002235263	Arabidopsis thaliana GDSL esterase/lipase EXL5 mRNA, complete cds
AT2G04680	-2.369489238	0.002235263	Arabidopsis thaliana chromosome 2, complete sequence
AT1G01590	-2.369489238	0.002235263	Arabidopsis thaliana ferric reduction oxidase 1 mRNA, complete cds
AT5G56810	-1.732059317	0.002239197	Arabidopsis thaliana putative F-box/FBD/LRR-repeat protein mRNA, complete cds
AT1G26970	-1.732059317	0.002239197	Arabidopsis thaliana putative protein kinase mRNA, complete cds
AT5G26690	-1.44921643	0.002269227	Arabidopsis thaliana heavy-metal-associated domain-containing protein mRNA, complete cds
AT3G42658.1	-3.413883357	0.002308036	Arabidopsis thaliana chromosome 3, complete sequence
AT5G01280	-3.413883357	0.002308036	Arabidopsis thaliana uncharacterized protein mRNA, complete cds
AT4G01735	-3.413883357	0.002308036	Arabidopsis thaliana uncharacterized protein mRNA, complete cds
AT1G63590	-3.413883357	0.002308036	Arabidopsis thaliana chromosome 1 sequence
AT1G29100	-3.413883357	0.002308036	Arabidopsis thaliana heavy-metal-associated domain-containing protein mRNA,

			complete cds
AT5G15725	-3.413883357	0.002308036	Arabidopsis thaliana uncharacterized protein mRNA, complete cds
AT3G01730	-3.413883357	0.002308036	Arabidopsis thaliana chromosome 3, complete sequence
AT4G34510	-1.828920857	0.002376661	Arabidopsis thaliana chromosome 4 sequence
AT3G59120	-1.828920857	0.002376661	Arabidopsis thaliana Cysteine/Histidine-rich C1 domain family protein mRNA, complete cds
AT4G34810	-1.828920857	0.002376661	Arabidopsis thaliana chromosome 4 sequence
AT1G62910	-1.239853957	0.002386143	Arabidopsis thaliana pentatricopeptide repeat-containing protein mRNA, complete cds
AT4G25707	-1.239853957	0.002386143	Arabidopsis thaliana chromosome 4 sequence
AT2G40085	-1.239853957	0.002386143	Arabidopsis thaliana uncharacterized protein mRNA, complete cds
AT1G80970	-1.239853957	0.002386143	Arabidopsis thaliana XH domain-containing protein mRNA, complete cds
AT3G03776	-2.12437674	0.002419681	Arabidopsis thaliana hydroxyproline-rich glycoprotein family protein mRNA, complete cds
AT4G18940	-1.954451739	0.002450061	Arabidopsis thaliana RNA ligase/cyclic nucleotide phosphodiesterase family protein mRNA, complete cds
AT5G03960	-1.17684416	0.002488511	Arabidopsis thaliana IQ-domain 12 protein mRNA, complete cds
AT1G64170	-1.17684416	0.002488511	Arabidopsis thaliana cation/H(+) antiporter 16 mRNA, complete cds
AT1G50270	-1.04191458	0.002521453	Arabidopsis thaliana chromosome 1 sequence
AT5G14830.1	-1.04191458	0.002521453	Arabidopsis thaliana chromosome 5 sequence
AT2G27035	-1.079982621	0.00254732	Arabidopsis thaliana early nodulin-like protein 20 mRNA, complete cds
AT5G27110	-1.490504639	0.002570776	Arabidopsis thaliana chromosome 5 sequence
AT1G67148	-1.490504639	0.002570776	Arabidopsis thaliana uncharacterized protein mRNA, complete cds
AT3G22540	-1.490504639	0.002570776	Arabidopsis thaliana chromosome 3, complete sequence

AT3G27027	-1.257014509	0.002784735	Arabidopsis thaliana chromosome 3, complete sequence
AT1G10980	-1.257014509	0.002784735	Arabidopsis thaliana Lung seven transmembrane receptor family protein mRNA, complete cds
AT3G21310	-1.539414239	0.002890334	Arabidopsis thaliana core-2/I-branching beta-1,6-N-acetylglucosaminyltransferase family protein mRNA, complete cds
AT2G43310	-1.539414239	0.002890334	Arabidopsis thaliana chromosome 2, complete sequence
AT3G03830	-1.539414239	0.002890334	Arabidopsis thaliana chromosome 3, complete sequence
AT1G71690	-1.539414239	0.002890334	Arabidopsis thaliana uncharacterized protein mRNA, complete cds
AT1G13130	-1.188916992	0.00292141	Arabidopsis thaliana Cellulase (glycosyl hydrolase family 5) protein mRNA, complete cds
AT1G50090	-1.188916992	0.00292141	Arabidopsis thaliana putative branched-chain-amino-acid aminotransferase 7 mRNA, complete cds
AT1G71380	-1.011035267	0.002923139	Arabidopsis thaliana cellulase 3 mRNA, complete cds
AT1G14960	-1.598307928	0.003217505	Arabidopsis thaliana polyyketide cyclase/dehydrase and lipid transport superfamily protein mRNA, complete cds
AT3G54310	-4.12437674	0.003235659	Arabidopsis thaliana uncharacterized protein mRNA, complete cds
AT5G14110	-4.12437674	0.003235659	Arabidopsis thaliana uncharacterized protein mRNA, complete cds
AT1G34500	-4.12437674	0.003235659	Arabidopsis thaliana chromosome 1 sequence
AT5G17590	-4.12437674	0.003235659	Arabidopsis thaliana chromosome 5 sequence
AT3G16210	-4.12437674	0.003235659	Arabidopsis thaliana chromosome 3, complete sequence
AT5G59662	-4.12437674	0.003235659	Arabidopsis thaliana chromosome 5 sequence
AT1G48070	-4.12437674	0.003235659	Arabidopsis thaliana TRX domain-containing protein mRNA, complete cds
AT2G22290	-4.12437674	0.003235659	Arabidopsis thaliana RAB GTPase-like protein H1D mRNA, complete cds
AT1G31260	-4.12437674	0.003235659	Arabidopsis thaliana putative zinc

			transporter 10 mRNA, complete cds
AT1G58130	-4.12437674	0.003235659	Arabidopsis thaliana chromosome 1 sequence
AT4G19770	-4.12437674	0.003235659	Arabidopsis thaliana Glycosyl hydrolase family protein with chitinase insertion domain mRNA, complete cds
AT5G65090	-1.276379833	0.003245042	Arabidopsis thaliana protein DEFORMED ROOT HAIRS 4 mRNA, complete cds
AT2G14115	-1.276379833	0.003245042	Arabidopsis thaliana chromosome 2, complete sequence
AT3G24540	-2.654891457	0.003297902	Arabidopsis thaliana proline-rich receptor-like protein kinase PERK3 mRNA, complete cds
AT1G64930	-2.654891457	0.003297902	Arabidopsis thaliana chromosome 1 sequence
AT2G34330	-2.654891457	0.003297902	Arabidopsis thaliana chromosome 2, complete sequence
AT4G25010	-2.654891457	0.003297902	Arabidopsis thaliana bidirectional sugar transporter SWEET14 mRNA, complete cds
AT3G27095	-2.654891457	0.003297902	Arabidopsis thaliana chromosome 3, complete sequence
AT2G20030	-2.654891457	0.003297902	Arabidopsis thaliana chromosome 2, complete sequence
AT5G02502	-1.401910716	0.003402961	Arabidopsis thaliana Oligosaccaryltransferase mRNA, complete cds
AT4G05220	-1.401910716	0.003402961	Arabidopsis thaliana chromosome 4 sequence
AT4G05170	-1.401910716	0.003402961	Arabidopsis thaliana transcription factor bHLH114 mRNA, complete cds
AT1G20700	-1.202379252	0.003427496	Arabidopsis thaliana WUSCHEL-related homeobox 14 mRNA, complete cds
AT3G30122	-1.013345428	0.003462119	Arabidopsis thaliana chromosome 3, complete sequence
AT4G00220	-1.091955262	0.003554981	Arabidopsis thaliana protein JAGGED LATERAL ORGANS mRNA, complete cds
AT1G02430	-1.29840614	0.003774375	Arabidopsis thaliana ADP-ribosylation factor D1B mRNA, complete cds
AT3G48580	-1.29840614	0.003774375	Arabidopsis thaliana probable xyloglucan endotransglucosylase/hydrolase 11 mRNA, complete cds

AT1G16640	-1.29840614	0.003774375	Arabidopsis thaliana AP2/B3-like transcriptional factor family protein mRNA, complete cds
AT5G51270	-1.761806661	0.003812566	Arabidopsis thaliana U-box domain-containing protein 53 mRNA, complete cds
AT1G16225	-1.761806661	0.003812566	Arabidopsis thaliana putative syntaxin-type t-SNARE protein mRNA, complete cds
AT1G66450	-1.761806661	0.003812566	Arabidopsis thaliana chromosome 1 sequence
AT4G08450	-1.761806661	0.003812566	Arabidopsis thaliana TIR-NBS-LRR class disease resistance protein mRNA, complete cds
AT5G15845	-1.761806661	0.003812566	Arabidopsis thaliana AT5g15850/F14F8_230 mRNA sequence
AT1G05990	-2.276379833	0.003853427	Arabidopsis thaliana chromosome 1 sequence
AT1G34520	-2.276379833	0.003853427	Arabidopsis thaliana MBOAT (membrane bound O-acyl transferase) family protein mRNA, complete cds
AT1G08430	-2.276379833	0.003853427	Arabidopsis thaliana aluminum-activated malate transporter 1 mRNA, complete cds
AT3G45638	-2.276379833	0.003853427	Arabidopsis thaliana clone asmbl_10177 unknown mRNA sequence
AT4G03965	-2.276379833	0.003853427	Arabidopsis thaliana chromosome 4 sequence
AT2G32650	-2.276379833	0.003853427	Arabidopsis thaliana RmlC-like cupins superfamily protein mRNA, complete cds
AT3G26120	-2.276379833	0.003853427	Arabidopsis thaliana terminal EAR1-like 1 mRNA, complete cds
AT2G32200	-1.439878566	0.00388882	Arabidopsis thaliana uncharacterized protein mRNA, complete cds
AT4G16220	-1.439878566	0.00388882	Arabidopsis thaliana GDSL esterase/lipase mRNA, complete cds
AT1G03700	-1.880451157	0.004007112	Arabidopsis thaliana uncharacterized protein mRNA, complete cds
AT4G05210	-1.880451157	0.004007112	Arabidopsis thaliana probable UDP-3-O-acylglucosamine N-acyltransferase 1 mRNA, complete cds
AT3G16900	-1.880451157	0.004007112	Arabidopsis thaliana uncharacterized protein mRNA, complete cds

AT5G65850	-1.880451157	0.004007112	Arabidopsis thaliana chromosome 5 sequence
AT3G57130	-1.880451157	0.004007112	Arabidopsis thaliana protein BLADE ON PETIOLE 1 mRNA, complete cds
AT2G29000	-2.04191458	0.004051759	Arabidopsis thaliana leucine-rich repeat protein kinase family protein mRNA, complete cds
AT1G67760	-2.04191458	0.004051759	Arabidopsis thaliana TCP-1/cpn60 chaperonin family protein mRNA, complete cds
AT4G22370	-2.04191458	0.004051759	Arabidopsis thaliana uncharacterized protein mRNA, complete cds
AT5G41685	-2.04191458	0.004051759	Arabidopsis thaliana chromosome 5 sequence
AT4G26770	-2.04191458	0.004051759	Arabidopsis thaliana phosphatidate cytidylyltransferase mRNA, complete cds
AT2G22140	-1.152391116	0.004153478	Arabidopsis thaliana essential meiotic endonuclease 1B mRNA, complete cds
AT1G06160	-1.152391116	0.004153478	Arabidopsis thaliana chromosome 1 sequence
AT2G01275	-1.053987412	0.004178202	Arabidopsis thaliana RING/FYVE/PHD zinc finger-containing protein mRNA, complete cds
AT1G28170	-3.276379833	0.004292497	Arabidopsis thaliana chromosome 1 sequence
AT5G18810	-1.323685548	0.004379731	Arabidopsis thaliana SC35-like splicing factor 28 mRNA, complete cds
AT5G05900	-1.323685548	0.004379731	Arabidopsis thaliana UDP-glycosyltransferase 76C3 mRNA, complete cds
AT2G02750	-1.484966455	0.004415983	Arabidopsis thaliana chromosome 2, complete sequence
AT5G57980	-1.484966455	0.004415983	Arabidopsis thaliana RNA polymerase II fifth largest subunit, C mRNA, complete cds
AT1G09155	-1.484966455	0.004415983	Arabidopsis thaliana phloem protein 2-B15 mRNA, complete cds
AT3G12965	-1.018582076	0.004862911	Arabidopsis thaliana clone asmbl_8715 unknown mRNA sequence
AT4G19920	-1.018582076	0.004862911	Arabidopsis thaliana Toll-Interleukin-Resistance domain-containing protein mRNA, complete cds

AT5G39160	-1.163905104	0.004891761	Arabidopsis thaliana germin-like protein subfamily 1 member 18 mRNA, complete cds
AT3G24535	-1.163905104	0.004891761	Arabidopsis thaliana chromosome 3, complete sequence
AT2G38060	-1.058788398	0.004947638	Arabidopsis thaliana phosphate transporter 4;2 mRNA, complete cds
AT3G48640	-1.539414239	0.004972881	Arabidopsis thaliana chromosome 3, complete sequence
AT4G24080	-1.539414239	0.004972881	Arabidopsis thaliana aldolase like protein mRNA, complete cds
AT1G23965	-1.353001115	0.00506697	Arabidopsis thaliana chromosome 1 sequence
AT4G22900	-1.25401202	0.005503936	Arabidopsis thaliana chromosome 4 sequence
AT1G62760	-1.606528435	0.005537185	Arabidopsis thaliana chromosome 1 sequence
AT5G57670	-1.021565934	0.00576741	Arabidopsis thaliana probable receptor-like serine/threonine-protein kinase mRNA, complete cds
AT5G24330	-1.387411146	0.005839427	Arabidopsis thaliana histone-lysine N-methyltransferase ATXR6 mRNA, complete cds
AT3G52072	-1.387411146	0.005839427	Arabidopsis thaliana mRNA for hypothetical protein, complete cds, clone: RAFL21-36-M10
AT4G12540	-1.06407623	0.005860862	Arabidopsis thaliana uncharacterized protein mRNA, complete cds
AT5G60335	-2.539414239	0.005868946	Arabidopsis thaliana thioesterase-like protein mRNA, complete cds
AT5G39480	-2.539414239	0.005868946	Arabidopsis thaliana chromosome 5 sequence
AT5G55770	-2.539414239	0.005868946	Arabidopsis thaliana chromosome 5 sequence
AT2G02640	-2.539414239	0.005868946	Arabidopsis thaliana chromosome 2, complete sequence
AT2G33460	-2.539414239	0.005868946	Arabidopsis thaliana ROP-interactive CRIB motif-containing protein 1 mRNA, complete cds
AT4G14860	-1.691417333	0.006069594	Arabidopsis thaliana chromosome 4 sequence
AT2G01580	-1.691417333	0.006069594	Arabidopsis thaliana chromosome 2, complete sequence
AT1G05730	-1.691417333	0.006069594	Arabidopsis thaliana uncharacterized

			protein mRNA, complete cds
AT4G10160	-3.954451739	0.006121747	Arabidopsis thaliana E3 ubiquitin-protein ligase ATL59 mRNA, complete cds
AT5G06905	-3.954451739	0.006121747	Arabidopsis thaliana cytochrome P450, family 712, subfamily A, polypeptide 2 mRNA, complete cds
AT2G01200	-3.954451739	0.006121747	Arabidopsis thaliana auxin-responsive protein IAA32 mRNA, complete cds
AT1G25053	-3.954451739	0.006121747	Arabidopsis thaliana chromosome 1 sequence
AT3G29780	-3.954451739	0.006121747	Arabidopsis thaliana chromosome 3, complete sequence
AT3G05860	-3.954451739	0.006121747	Arabidopsis thaliana MADS-box transcription factor family protein mRNA, complete cds
AT4G31250	-3.954451739	0.006121747	Arabidopsis thaliana putative LRR receptor-like serine/threonine-protein kinase mRNA, complete cds
AT3G56500	-3.954451739	0.006121747	Arabidopsis thaliana chromosome 3, complete sequence
AT4G30730	-3.954451739	0.006121747	Arabidopsis thaliana chromosome 4 sequence
AT5G59860	-3.954451739	0.006121747	Arabidopsis thaliana RNA recognition motif-containing protein mRNA, complete cds
AT3G49630	-1.276379833	0.006426869	Arabidopsis thaliana 2-oxoglutarate (2OG) and Fe(II)-dependent oxygenase superfamily protein mRNA, complete cds
AT5G22660	-1.802448645	0.006504451	Arabidopsis thaliana F-box/FBD/LRR-repeat protein mRNA, complete cds
AT1G30850	-1.802448645	0.006504451	Arabidopsis thaliana chromosome 1 sequence
AT5G23780	-1.802448645	0.006504451	Arabidopsis thaliana uncharacterized protein mRNA, complete cds
AT2G35860	-1.802448645	0.006504451	Arabidopsis thaliana fasciclin-like arabinogalactan protein 16 mRNA, complete cds
AT5G45740	-1.802448645	0.006504451	Arabidopsis thaliana ubiquitin domain-containing protein mRNA, complete cds
AT1G52790	-2.17684416	0.006601664	Arabidopsis thaliana oxidoreductase, 2OG-Fe(II) oxygenase family protein

			mRNA, complete cds
AT4G22513	-2.17684416	0.006601664	Arabidopsis thaliana chromosome 4 sequence
AT2G30760	-2.17684416	0.006601664	Arabidopsis thaliana uncharacterized protein mRNA, complete cds
AT1G49370	-2.17684416	0.006601664	Arabidopsis thaliana chromosome 1 sequence
AT5G42450	-1.428382927	0.006695632	Arabidopsis thaliana pentatricopeptide repeat-containing protein mRNA, complete cds
AT5G11570	-1.428382927	0.006695632	Arabidopsis thaliana major facilitator protein mRNA, complete cds
AT5G22355	-1.428382927	0.006695632	Arabidopsis thaliana chromosome 5 sequence
AT3G04330	-1.954451739	0.006736136	Arabidopsis thaliana chromosome 3, complete sequence
AT4G11390	-1.954451739	0.006736136	Arabidopsis thaliana cysteine/histidine-rich C1 domain-containing protein mRNA, complete cds
AT3G49820	-1.954451739	0.006736136	Arabidopsis thaliana chromosome 3, complete sequence
AT3G05685	-1.024841066	0.006843638	Arabidopsis thaliana cystatin-related protein mRNA, complete cds
ATCG00080	-1.12437674	0.006935039	Cardamine resedifolia plastid, complete genome
AT4G11140	-1.069928956	0.006945181	Arabidopsis thaliana chromosome 4 sequence
AT2G33205	-1.302375042	0.007488351	Arabidopsis thaliana Serinc-domain containing serine and sphingolipid biosynthesis protein mRNA, complete cds
AT1G31760	-1.302375042	0.007488351	Arabidopsis thaliana SWIB/MDM2 domain-containing protein mRNA, complete cds
AT2G15630	-1.302375042	0.007488351	Arabidopsis thaliana chromosome 2, complete sequence
AT2G23830	-3.12437674	0.007988918	Arabidopsis thaliana vesicle-associated protein 3-1 mRNA, complete cds
AT1G43010	-3.12437674	0.007988918	Arabidopsis thaliana pentatricopeptide repeat-containing protein mRNA, complete cds
AT3G61090	-3.12437674	0.007988918	Arabidopsis thaliana putative endonuclease or glycosyl hydrolase

			mRNA, complete cds
AT5G24180	-3.12437674	0.007988918	Arabidopsis thaliana lipase class 3-related protein mRNA, complete cds
AT4G17788	-3.12437674	0.007988918	Arabidopsis thaliana chromosome 4 sequence
AT3G17050.1	-3.12437674	0.007988918	Arabidopsis thaliana chromosome 3, complete sequence
AT3G51560	-3.12437674	0.007988918	Arabidopsis thaliana TIR-NBS-LRR class disease resistance protein mRNA, complete cds
AT3G08500	-1.02845232	0.008125102	Arabidopsis thaliana myb domain protein 83 mRNA, complete cds
AT5G01660	-1.02845232	0.008125102	Arabidopsis thaliana uncharacterized protein mRNA, complete cds
AT5G48780	-1.135023984	0.008197101	Arabidopsis thaliana TIR-NBS class disease resistance protein mRNA, complete cds
AT1G01880	-1.135023984	0.008197101	Arabidopsis thaliana 5'-3' exonuclease family protein mRNA, complete cds
AT5G45730	-1.076442263	0.008233153	Arabidopsis thaliana cysteine/histidine-rich C1 domain-containing protein mRNA, complete cds
AT2G34925	-1.076442263	0.008233153	Arabidopsis thaliana chromosome 2, complete sequence
AT1G07270	-1.539414239	0.008604574	Arabidopsis thaliana cell division control protein 6 mRNA, complete cds
AT5G46845	-1.332963362	0.008700331	Arabidopsis thaliana chromosome 5 sequence
AT1G78190	-1.332963362	0.008700331	Arabidopsis thaliana chromosome 1 sequence
AT1G11340	-1.332963362	0.008700331	Arabidopsis thaliana G-type lectin S-receptor-like serine/threonine-protein kinase mRNA, complete cds
AT1G13610	-1.332963362	0.008700331	Arabidopsis thaliana alpha/beta-hydrolase-like protein mRNA, complete cds
AT4G26830	-1.332963362	0.008700331	Arabidopsis thaliana O-Glycosyl hydrolases family 17 protein mRNA, complete cds
AT4G03940	-1.227470233	0.009359803	Arabidopsis thaliana uncharacterized protein mRNA, complete cds
AT4G11550	-1.227470233	0.009359803	Arabidopsis thaliana chromosome 4 sequence

AT3G56220	-1.227470233	0.009359803	Arabidopsis thaliana transcription regulator mRNA, complete cds
AT1G16420	-1.617416751	0.009583292	Arabidopsis thaliana metacaspase 8 mRNA, complete cds
AT2G20875	-1.617416751	0.009583292	Arabidopsis thaliana protein EPIDERMAL PATTERNING FACTOR 1 mRNA, complete cds
AT4G36105	-1.617416751	0.009583292	Arabidopsis thaliana uncharacterized protein mRNA, complete cds
AT2G31083	-1.617416751	0.009583292	Arabidopsis thaliana chromosome 2, complete sequence
AT1G73165	-1.617416751	0.009583292	Arabidopsis thaliana chromosome 1 sequence
AT3G28960	-1.032454251	0.009652083	Arabidopsis thaliana transmembrane amino acid transporter family protein mRNA, complete cds
AT4G14120	-1.083734756	0.009763591	Arabidopsis thaliana uncharacterized protein mRNA, complete cds
AT3G47342	-1.083734756	0.009763591	Arabidopsis thaliana chromosome 3, complete sequence
AT3G60700	-1.369489238	0.010070044	Arabidopsis thaliana uncharacterized protein mRNA, complete cds
AT5G37072	-1.369489238	0.010070044	Arabidopsis thaliana chromosome 5 sequence
AT5G38230.1	-2.413883357	0.010392925	Arabidopsis thaliana chromosome 5 sequence
AT1G53633	-2.413883357	0.010392925	Arabidopsis thaliana uncharacterized protein mRNA, complete cds
AT5G10660	-2.413883357	0.010392925	Arabidopsis thaliana chromosome 5 sequence
AT5G52220	-2.413883357	0.010392925	Arabidopsis thaliana chromosome transmission fidelity protein 8 domain-containing protein mRNA, complete cds
AT5G21080	-2.413883357	0.010392925	Arabidopsis thaliana uncharacterized protein mRNA, complete cds
AT2G38152	-2.413883357	0.010392925	Arabidopsis thaliana alpha 1,4-glycosyltransferase-like protein mRNA, complete cds
AT4G39000	-1.719986485	0.01047122	Arabidopsis thaliana glycosyl hydrolase 9B17 mRNA, complete cds
AT2G02310	-1.719986485	0.01047122	Arabidopsis thaliana putative F-box protein PP2-B6 mRNA, complete cds
AT4G31710	-1.249907622	0.010979764	Arabidopsis thaliana glutamate receptor

			2.4 mRNA, complete cds
AT1G25054	-1.249907622	0.010979764	Arabidopsis thaliana UDP-3-O-[3-hydroxymyristoyl] N-acetylglucosamine deacetylase mRNA, complete cds
AT4G26170	-1.861342334	0.011109739	Arabidopsis thaliana uncharacterized protein mRNA, complete cds
AT3G50200	-1.861342334	0.011109739	Arabidopsis thaliana uncharacterized protein mRNA, complete cds
AT5G49350	-1.861342334	0.011109739	Arabidopsis thaliana glycine-rich protein mRNA, complete cds
AT2G11150.1	-2.069928956	0.011229519	Arabidopsis thaliana chromosome 2, complete sequence
AT3G28510	-2.069928956	0.011229519	Arabidopsis thaliana chromosome 3, complete sequence
AT4G17160	-2.069928956	0.011229519	Arabidopsis thaliana RAB GTPase homolog B1A mRNA, complete cds
AT3G59250	-1.160902616	0.01144812	Arabidopsis thaliana F-box/LRR-repeat protein mRNA, complete cds
AT1G61390	-1.160902616	0.01144812	Arabidopsis thaliana G-type lectin S-receptor-like serine/threonine-protein kinase mRNA, complete cds
AT5G25020	-1.160902616	0.01144812	Arabidopsis thaliana uncharacterized protein mRNA, complete cds
AT4G09012	-1.160902616	0.01144812	Arabidopsis thaliana mitochondrial ribosomal protein L27 mRNA, complete cds
AT3G21080	-1.091955262	0.011582734	Arabidopsis thaliana ABC transporter-like protein mRNA, complete cds
AT3G14630	-1.413883357	0.011595101	Arabidopsis thaliana cytochrome P450, family 72, subfamily A, polypeptide 9 mRNA, complete cds
AT1G55700	-1.413883357	0.011595101	Arabidopsis thaliana chromosome 1 sequence
AT5G38565	-1.413883357	0.011595101	Arabidopsis thaliana putative FBD-associated F-box protein mRNA, complete cds
AT5G51795	-1.413883357	0.011595101	Arabidopsis thaliana DNA/RNA-binding protein Kin17, conserved region-containing protein mRNA, complete cds
AT1G59725	-1.413883357	0.011595101	Arabidopsis thaliana putative DNAJ heat shock protein mRNA, complete cds

AT1G06980	-1.413883357	0.011595101	Arabidopsis thaliana chromosome 1 sequence
AT2G15130	-1.413883357	0.011595101	Arabidopsis thaliana chromosome 2, complete sequence
AT1G21230	-3.761806661	0.011667826	Arabidopsis thaliana wall-associated receptor kinase 5 mRNA, complete cds
AT5G39490	-3.761806661	0.011667826	Arabidopsis thaliana chromosome 5 sequence
AT2G23440	-3.761806661	0.011667826	Arabidopsis thaliana chromosome 2, complete sequence
AT4G02190	-3.761806661	0.011667826	Arabidopsis thaliana chromosome 4 sequence
AT2G47040	-3.761806661	0.011667826	Arabidopsis thaliana pectinesterase 5 mRNA, complete cds
AT5G57540	-3.761806661	0.011667826	Arabidopsis thaliana xyloglucan endotransglucosylase/hydrolase 13 mRNA, complete cds
AT2G38100	-3.761806661	0.011667826	Arabidopsis thaliana putative peptide/nitrate transporter mRNA, complete cds
AT1G79130	-3.761806661	0.011667826	Arabidopsis thaliana chromosome 1 sequence
AT5G41765	-3.761806661	0.011667826	Arabidopsis thaliana DNA-binding storekeeper protein-related transcriptional regulator mRNA, complete cds
AT1G76135	-3.761806661	0.011667826	Arabidopsis thaliana chromosome 1 sequence
AT3G24130	-3.761806661	0.011667826	Arabidopsis thaliana putative pectinesterase 29 mRNA, complete cds
AT3G18460	-3.761806661	0.011667826	Arabidopsis thaliana PLAC8 family protein mRNA, complete cds
AT3G50710	-3.761806661	0.011667826	Arabidopsis thaliana putative FBD-associated F-box protein mRNA, complete cds
AT1G57835	-3.761806661	0.011667826	Arabidopsis thaliana mRNA for hypothetical protein, partial cds, clone: RAFL14-03-A17
AT4G20235	-1.276379833	0.012856245	Arabidopsis thaliana putative cytochrome P450 71A28 mRNA, complete cds
AT2G02620	-1.469024911	0.013254939	Arabidopsis thaliana chromosome 2, complete sequence
AT1G70885	-1.469024911	0.013254939	Arabidopsis thaliana chromosome 1 sequence

AT3G48290	-1.469024911	0.013254939	Arabidopsis thaliana cytochrome P450 71A24 mRNA, complete cds
AT1G48220	-1.17684416	0.013523208	Arabidopsis thaliana protein kinase family prtein mRNA, complete cds
AT1G65480	-1.04191458	0.013646544	Arabidopsis thaliana protein FLOWERING LOCUS T mRNA, complete cds
AT4G39361	-1.04191458	0.013646544	Arabidopsis thaliana chromosome 4 sequence
AT5G44010	-1.04191458	0.013646544	Arabidopsis thaliana uncharacterized protein mRNA, complete cds
AT3G20360	-1.04191458	0.013646544	Arabidopsis thaliana TRAF-like family protein mRNA, complete cds
AT5G55720	-1.101293127	0.013745597	Arabidopsis thaliana probable pectate lyase 21 mRNA, complete cds
AT3G20610	-2.954451739	0.014871496	Arabidopsis thaliana chromosome 3, complete sequence
AT1G68320	-2.954451739	0.014871496	Arabidopsis thaliana R2R3-MYB transcription family mRNA, complete cds
AT1G27921	-2.954451739	0.014871496	Arabidopsis thaliana chromosome 1 sequence
AT1G07460	-2.954451739	0.014871496	Arabidopsis thaliana chromosome 1 sequence
AT3G07425	-2.954451739	0.014871496	Arabidopsis thaliana chromosome 3, complete sequence
AT1G79860	-2.954451739	0.014871496	Arabidopsis thaliana RHO guanyl-nucleotide exchange factor 12 mRNA, complete cds
AT1G25240	-2.954451739	0.014871496	Arabidopsis thaliana chromosome 1 sequence
AT2G37740	-1.539414239	0.014995859	Arabidopsis thaliana chromosome 2, complete sequence
AT2G04500	-1.539414239	0.014995859	Arabidopsis thaliana chromosome 2, complete sequence
AT5G49850	-1.539414239	0.014995859	Arabidopsis thaliana mRNA for hypothetical protein, complete cds, clone: RAFL14-29-F23
AT5G03020	-1.539414239	0.014995859	Arabidopsis thaliana chromosome 5 sequence
AT1G61130	-1.539414239	0.014995859	Arabidopsis thaliana serine carboxypeptidase-like 32 mRNA, complete cds
AT3G09190	-1.047561143	0.016243047	Arabidopsis thaliana chromosome 3,

			complete sequence
AT2G44735	-1.047561143	0.016243047	Arabidopsis thaliana uncharacterized protein mRNA, complete cds
AT1G32415	-1.111993016	0.016317497	Arabidopsis thaliana chromosome 1 sequence
AT2G26420	-1.632523644	0.016704484	Arabidopsis thaliana phosphatidylinositol-4-phosphate 5-kinase 3 mRNA, complete cds
AT5G60060	-1.632523644	0.016704484	Arabidopsis thaliana chromosome 5 sequence
AT5G10850.1	-1.346769161	0.017472481	Arabidopsis thaliana chromosome 5 sequence
AT5G01070	-1.761806661	0.018159851	Arabidopsis thaliana RING/FYVE/PHD zinc finger-containing protein mRNA, complete cds
AT2G42140	-1.761806661	0.018159851	Arabidopsis thaliana chromosome 2, complete sequence
AT1G10220	-1.761806661	0.018159851	Arabidopsis thaliana uncharacterized protein mRNA, complete cds
AT1G50390	-1.761806661	0.018159851	Arabidopsis thaliana pfkB-like carbohydrate kinase family protein mRNA, complete cds
ATCG00100	-1.761806661	0.018159851	Liverwort chloroplast Gly-tRNA-tcc
AT5G21050	-1.761806661	0.018159851	Arabidopsis thaliana chromosome 5 sequence
AT2G26695	-1.761806661	0.018159851	Arabidopsis thaliana Ran BP2/NZF zinc finger-like protein mRNA, complete cds
AT3G11640	-1.761806661	0.018159851	Arabidopsis thaliana chromosome 3, complete sequence
AT2G33175	-1.761806661	0.018159851	Arabidopsis thaliana chromosome 2, complete sequence
AT5G40860	-2.276379833	0.018294268	Arabidopsis thaliana uncharacterized protein mRNA, complete cds
AT5G59370	-2.276379833	0.018294268	Arabidopsis thaliana actin 4 mRNA, complete cds
AT1G07795	-2.276379833	0.018294268	Arabidopsis thaliana chromosome 1 sequence
AT1G50930	-2.276379833	0.018294268	Arabidopsis thaliana uncharacterized protein mRNA, complete cds
AT2G23400	-2.276379833	0.018294268	Arabidopsis thaliana undecaprenyl pyrophosphate synthetase family protein mRNA, complete cds
AT1G01695	-2.276379833	0.018294268	Arabidopsis thaliana phosphatidylinositol N-acetyglucosaminlytransferase subunit

			P-like protein mRNA, complete cds
AT2G45780	-2.276379833	0.018294268	Arabidopsis thaliana chromosome 2, complete sequence
AT3G13840	-2.276379833	0.018294268	Arabidopsis thaliana chromosome 3, complete sequence
AT1G79075	-2.276379833	0.018294268	Arabidopsis thaliana chromosome 1 sequence
AT5G14380	-1.954451739	0.018945825	Arabidopsis thaliana chromosome 5 sequence
AT2G43470	-1.954451739	0.018945825	Arabidopsis thaliana uncharacterized protein mRNA, complete cds
AT5G56200	-1.954451739	0.018945825	Arabidopsis thaliana chromosome 5 sequence
AT1G31173	-1.954451739	0.018945825	Arabidopsis thaliana chromosome 1 sequence
AT5G41290	-1.053987412	0.019347884	Arabidopsis thaliana cysteine-rich repeat secretory protein 58 mRNA, complete cds
AT1G69180	-1.39502433	0.020227131	Arabidopsis thaliana putative transcription factor CRABS CLAW mRNA, complete cds
AT1G15640	-1.39502433	0.020227131	Arabidopsis thaliana uncharacterized protein mRNA, complete cds
AT5G62310	-1.39502433	0.020227131	Arabidopsis thaliana putative serine/threonine protein kinase IRE mRNA, complete cds
AT5G48670	-1.39502433	0.020227131	Arabidopsis thaliana chromosome 5 sequence
AT1G32583	-1.243958356	0.022182394	Arabidopsis thaliana uncharacterized protein mRNA, complete cds
AT3G48400	-1.243958356	0.022182394	Arabidopsis thaliana cysteine/histidine-rich C1 domain-containing protein mRNA, complete cds
AT5G57640	-1.243958356	0.022182394	Arabidopsis thaliana chromosome 5 sequence
AT2G04300	-3.539414239	0.022416951	Arabidopsis thaliana putative leucine-rich repeat receptor-like serine/threonine-protein kinase mRNA, complete cds
AT1G57943	-3.539414239	0.022416951	Arabidopsis thaliana purine permease 17 mRNA, complete cds
ATCG01090	-3.539414239	0.022416951	Arabidopsis thaliana chloroplast DNA, complete genome, ecotype: Columbia

AT1G80470	-3.539414239	0.022416951	Arabidopsis thaliana F-box/FBD/LRR-repeat protein mRNA, complete cds
AT5G28280	-3.539414239	0.022416951	Arabidopsis thaliana chromosome 5 sequence
AT2G17055	-3.539414239	0.022416951	Arabidopsis thaliana chromosome 2, complete sequence
AT2G21030	-3.539414239	0.022416951	Arabidopsis thaliana uncharacterized protein mRNA, complete cds
AT5G35407	-3.539414239	0.022416951	Arabidopsis thaliana chromosome 5 sequence
AT3G09340	-3.539414239	0.022416951	Arabidopsis thaliana transmembrane amino acid transporter-like protein mRNA, complete cds
AT1G13490	-3.539414239	0.022416951	Arabidopsis thaliana uncharacterized protein mRNA, complete cds
AT5G27200	-3.539414239	0.022416951	Arabidopsis thaliana acyl carrier protein 5 mRNA, complete cds
AT1G34490	-3.539414239	0.022416951	Arabidopsis thaliana chromosome 1 sequence
AT3G45170	-3.539414239	0.022416951	Arabidopsis thaliana GATA transcription factor 14 mRNA, complete cds
AT1G11655	-3.539414239	0.022416951	Arabidopsis thaliana chromosome 1 sequence
AT4G15075	-3.539414239	0.022416951	Arabidopsis thaliana FBD-like domain family protein mRNA, complete cds
AT4G11385	-3.539414239	0.022416951	Arabidopsis thaliana uncharacterized protein mRNA, complete cds
AT5G01080	-3.539414239	0.022416951	Arabidopsis thaliana chromosome 5 sequence
AT1G11482	-3.539414239	0.022416951	Arabidopsis thaliana chromosome 1 sequence
AT1G52618	-1.13887631	0.023011026	Arabidopsis thaliana chromosome 1 sequence
AT1G19940	-1.13887631	0.023011026	Arabidopsis thaliana glycosyl hydrolase 9B5 mRNA, complete cds
AT3G28630	-1.061366943	0.023064282	Arabidopsis thaliana uncharacterized protein mRNA, complete cds
AT1G31430	-1.061366943	0.023064282	Arabidopsis thaliana chromosome 1 sequence
AT4G27660	-1.061366943	0.023064282	Arabidopsis thaliana uncharacterized protein mRNA, complete cds
AT5G01445	-1.456952079	0.023237052	Arabidopsis thaliana uncharacterized protein mRNA, complete cds

AT2G05180	-1.456952079	0.023237052	Arabidopsis thaliana cytochrome P450, family 705, subfamily A, polypeptide 6 mRNA, complete cds
AT4G27510	-1.276379833	0.02607518	Arabidopsis thaliana uncharacterized protein mRNA, complete cds
AT4G09920	-1.276379833	0.02607518	Arabidopsis thaliana FBD, F-box and leucine rich repeat domain-containing protein mRNA, complete cds
AT5G09720	-1.276379833	0.02607518	Arabidopsis thaliana putative inactive magnesium transporter MRS2-8 mRNA, complete cds
AT5G44569	-1.276379833	0.02607518	Arabidopsis thaliana chromosome 5 sequence
ATMG00110	-1.276379833	0.02607518	Arabidopsis thaliana ecotype Landsberg erecta mitochondrion, complete genome
AT3G19040	-1.539414239	0.026382182	Arabidopsis thaliana transcription initiation factor TFIID subunit 1-B mRNA, complete cds
AT3G21351	-1.539414239	0.026382182	Arabidopsis thaliana uncharacterized protein mRNA, complete cds
AT4G22545	-1.539414239	0.026382182	Arabidopsis thaliana chromosome 4 sequence
AT2G21260	-1.539414239	0.026382182	Arabidopsis thaliana putative mannose-6P reductase mRNA, complete cds
AT1G05020	-1.005077812	0.02700755	Arabidopsis thaliana chromosome 1 sequence
AT4G16807	-1.005077812	0.02700755	Arabidopsis thaliana uncharacterized protein mRNA, complete cds
AT5G62150	-1.005077812	0.02700755	Arabidopsis thaliana chromosome 5 sequence
AT3G47110	-1.005077812	0.02700755	Arabidopsis thaliana putative receptor-like protein kinase mRNA, complete cds
AT5G58910	-1.1560856	0.027329202	Arabidopsis thaliana laccase 16 mRNA, complete cds
AT1G25360	-1.069928956	0.027517287	Arabidopsis thaliana chromosome 1 sequence
AT2G29260	-1.069928956	0.027517287	Arabidopsis thaliana NAD(P)-binding Rossmann-fold superfamily protein mRNA, complete cds
AT5G64667	-1.069928956	0.027517287	Arabidopsis thaliana chromosome 5 sequence
AT2G31310	-1.069928956	0.027517287	Arabidopsis thaliana LOB

			domain-containing protein 14 mRNA, complete cds
AT4G13680	-2.761806661	0.027669598	Arabidopsis thaliana chromosome 4 sequence
ATCG01010	-2.761806661	0.027669598	Arabidopsis thaliana chloroplast DNA, complete genome, ecotype: Columbia
AT3G02590	-2.761806661	0.027669598	Arabidopsis thaliana putative Delta(7)-sterol-C5(6)-desaturase 2 mRNA, complete cds
AT1G15385	-2.761806661	0.027669598	Arabidopsis thaliana chromosome 1 sequence
AT5G61610	-2.761806661	0.027669598	Arabidopsis thaliana oleosin mRNA, complete cds
AT1G78030	-2.761806661	0.027669598	Arabidopsis thaliana uncharacterized protein mRNA, complete cds
AT5G53700	-2.761806661	0.027669598	Arabidopsis thaliana RNA-binding (RRM/RBD/RNP motifs) family protein mRNA, complete cds
AT5G18661	-2.761806661	0.027669598	Arabidopsis thaliana chromosome 5 sequence
AT1G71770	-2.761806661	0.027669598	Arabidopsis thaliana polyadenylate-binding protein 5 mRNA, complete cds
AT1G49938	-2.761806661	0.027669598	Arabidopsis thaliana uncharacterized protein mRNA, complete cds
AT5G24190	-2.761806661	0.027669598	Arabidopsis thaliana lipase class 3-related protein mRNA, complete cds
AT5G39150	-2.761806661	0.027669598	Arabidopsis thaliana germin-like protein subfamily 1 member 17 mRNA, complete cds
AT2G14580	-2.761806661	0.027669598	Arabidopsis thaliana chromosome 2, complete sequence
AT2G17940	-1.654891457	0.029387336	Arabidopsis thaliana uncharacterized protein mRNA, complete cds
AT4G10720	-1.654891457	0.029387336	Arabidopsis thaliana ankyrin repeat-containing protein mRNA, complete cds
AT3G61113	-1.654891457	0.029387336	Arabidopsis thaliana ubiquitin-related modifier 1 mRNA, complete cds
AT5G35760	-1.654891457	0.029387336	Arabidopsis thaliana Beta-galactosidase related protein mRNA, complete cds
AT4G20190	-1.654891457	0.029387336	Arabidopsis thaliana chromosome 4 sequence
AT4G04900	-1.654891457	0.029387336	Arabidopsis thaliana ROP-interactive

			CRIB motif-containing protein 10 mRNA, complete cds
AT2G04480	-1.654891457	0.029387336	Arabidopsis thaliana uncharacterized protein mRNA, complete cds
AT2G16960	-1.654891457	0.029387336	Arabidopsis thaliana ARM repeat superfamily protein mRNA, complete cds
AT2G19200	-1.654891457	0.029387336	Arabidopsis thaliana hypothetical protein (At2g19200/T20K24.25) mRNA, complete cds
AT1G63580	-1.654891457	0.029387336	Arabidopsis thaliana receptor-like protein kinase-related family protein mRNA, complete cds
AT3G18530	-1.317021818	0.030553911	Arabidopsis thaliana ARM repeat family protein-like protein mRNA, complete cds
AT1G74540	-1.317021818	0.030553911	Arabidopsis thaliana chromosome 1 sequence
AT3G09400	-1.828920857	0.031663991	Arabidopsis thaliana putative protein phosphatase 2C 36 mRNA, complete cds
AT1G32385	-1.828920857	0.031663991	Arabidopsis thaliana chromosome 1 sequence
AT3G29370	-1.828920857	0.031663991	Arabidopsis thaliana chromosome 3, complete sequence
AT3G29772.1	-1.828920857	0.031663991	Arabidopsis thaliana chromosome 3, complete sequence
AT2G31480	-1.828920857	0.031663991	Arabidopsis thaliana chromosome 2, complete sequence
AT5G22150	-1.828920857	0.031663991	Arabidopsis thaliana uncharacterized protein mRNA, complete cds
ATCG00200	-2.12437674	0.031968666	Aster spathulifolius chloroplast, complete genome
AT1G24792	-2.12437674	0.031968666	Arabidopsis thaliana chromosome 1 sequence
ATMG01220	-2.12437674	0.031968666	Arabidopsis thaliana ecotype Landsberg erecta mitochondrion, complete genome
AT3G02832	-2.12437674	0.031968666	Arabidopsis thaliana chromosome 3, complete sequence
AT5G16230	-2.12437674	0.031968666	Arabidopsis thaliana acyl-[acyl-carrier-protein] desaturase mRNA, complete cds
AT1G13620	-2.12437674	0.031968666	Arabidopsis thaliana root meristem growth factor 2 mRNA, complete cds
AT4G38495	-2.12437674	0.031968666	Arabidopsis thaliana uncharacterized protein mRNA, complete cds

AT2G32179	-2.12437674	0.031968666	Arabidopsis thaliana uncharacterized protein mRNA, complete cds
AT1G49610	-2.12437674	0.031968666	Arabidopsis thaliana putative F-box protein mRNA, complete cds
AT4G26020	-2.12437674	0.031968666	Arabidopsis thaliana uncharacterized protein mRNA, complete cds
AT5G08150	-2.12437674	0.031968666	Arabidopsis thaliana chromosome 5 sequence
AT2G42850	-1.008899523	0.032315473	Arabidopsis thaliana cytochrome P450, family 718 mRNA, complete cds
AT5G51390	-1.008899523	0.032315473	Arabidopsis thaliana chromosome 5 sequence
AT5G19650	-1.008899523	0.032315473	Arabidopsis thaliana chromosome 5 sequence
AT5G53950	-1.17684416	0.032451091	Arabidopsis thaliana protein CUP-SHAPED COTYLEDON 2 mRNA, complete cds
AT1G35515	-1.17684416	0.032451091	Arabidopsis thaliana R2R3-type MYB transcription factor mRNA, complete cds
AT3G09550	-1.079982621	0.032858434	Arabidopsis thaliana ankyrin repeat-containing protein mRNA, complete cds
AT1G52330	-1.079982621	0.032858434	Arabidopsis thaliana chromosome 1 sequence
AT1G72870	-1.079982621	0.032858434	Arabidopsis thaliana TIR-NBS class of disease resistance protein mRNA, complete cds
AT1G63910	-1.369489238	0.035624814	Arabidopsis thaliana myb domain protein 103 mRNA, complete cds
AT1G18410	-1.369489238	0.035624814	Arabidopsis thaliana kinesin motor domain-containing protein mRNA, complete cds
AT2G20721	-1.369489238	0.035624814	Arabidopsis thaliana chromosome 2, complete sequence
AT4G04972	-1.369489238	0.035624814	Arabidopsis thaliana uncharacterized protein mRNA, complete cds
AT1G76890	-1.369489238	0.035624814	Arabidopsis thaliana trihelix transcription factor GT-2 mRNA, complete cds
AT5G53980	-1.202379252	0.038510522	Arabidopsis thaliana chromosome 5 sequence
AT4G14170	-1.202379252	0.038510522	Arabidopsis thaliana pentatricopeptide repeat-containing protein mRNA, complete cds
AT4G19590	-1.202379252	0.038510522	Arabidopsis thaliana chaperone

			DnaJ-domain containing protein mRNA, complete cds
AT4G35810	-1.202379252	0.038510522	Arabidopsis thaliana oxidoreductase, 2OG-Fe(II) oxygenase family protein mRNA, complete cds
AT4G31877	-1.202379252	0.038510522	Arabidopsis thaliana mRNA for unknown protein, complete cds, clone: RAFL17-06-E12
AT1G33055	-1.202379252	0.038510522	Arabidopsis thaliana uncharacterized protein mRNA, complete cds
AT3G29140	-1.091955262	0.039271239	Arabidopsis thaliana uncharacterized protein mRNA, complete cds
AT4G16640	-1.091955262	0.039271239	Arabidopsis thaliana chromosome 4 sequence
AT1G21220.1	-1.439878566	0.04120646	Arabidopsis thaliana chromosome 1 sequence
AT5G14810.1	-1.439878566	0.04120646	Arabidopsis thaliana chromosome 5 sequence
AT3G18510	-1.439878566	0.04120646	Arabidopsis thaliana uncharacterized protein mRNA, complete cds
AT1G70581	-1.439878566	0.04120646	Arabidopsis thaliana chromosome 1 sequence
AT1G20990	-1.439878566	0.04120646	Arabidopsis thaliana Cysteine/Histidine-rich C1 domain family protein mRNA, complete cds
AT5G17720	-3.276379833	0.043448349	Arabidopsis thaliana hydrolase, alpha/beta fold family protein mRNA, complete cds
AT3G13404	-3.276379833	0.043448349	Arabidopsis thaliana uncharacterized protein mRNA, complete cds
AT3G13400	-3.276379833	0.043448349	Arabidopsis thaliana protein SKU5-like 13 mRNA, complete cds
AT3G01345	-3.276379833	0.043448349	Arabidopsis thaliana chromosome 3, complete sequence
AT1G15050	-3.276379833	0.043448349	Arabidopsis thaliana auxin-responsive protein IAA34 mRNA, complete cds
AT2G42480	-3.276379833	0.043448349	Arabidopsis thaliana TRAF-like family protein mRNA, complete cds
AT1G44542	-3.276379833	0.043448349	Arabidopsis thaliana cyclase family protein mRNA, complete cds
AT5G54620	-3.276379833	0.043448349	Arabidopsis thaliana ankyrin repeat-containing protein mRNA, complete cds
AT3G46350	-3.276379833	0.043448349	Arabidopsis thaliana Leucine-rich repeat

			protein kinase family protein mRNA, complete cds
AT3G11380	-3.276379833	0.043448349	Arabidopsis thaliana pentatricopeptide repeat-containing protein mRNA, complete cds
AT2G36020	-3.276379833	0.043448349	Arabidopsis thaliana HVA22-like protein j mRNA, complete cds
AT1G61270	-3.276379833	0.043448349	Arabidopsis thaliana transmembrane amino acid transporter family protein mRNA, complete cds
AT2G29010	-3.276379833	0.043448349	Arabidopsis thaliana chromosome 2, complete sequence
AT3G29970	-3.276379833	0.043448349	Arabidopsis thaliana B12D protein mRNA, complete cds
AT2G34320	-3.276379833	0.043448349	Arabidopsis thaliana chromosome 2, complete sequence
AT2G17590	-3.276379833	0.043448349	Arabidopsis thaliana chromosome 2, complete sequence
AT3G48346	-3.276379833	0.043448349	Arabidopsis thaliana chromosome 3, complete sequence
AT5G12235	-3.276379833	0.043448349	Arabidopsis thaliana chromosome 5 sequence
ATCG00290	-3.276379833	0.043448349	Pachycladon cheesemanii chloroplast, complete genome
AT3G05780	-3.276379833	0.043448349	Arabidopsis thaliana lon protease 3 mRNA, complete cds
AT5G18260	-3.276379833	0.043448349	Arabidopsis thaliana protein binding / zinc ion binding protein mRNA, complete cds
AT1G36675	-1.234559658	0.04564636	Arabidopsis thaliana glycine-rich protein mRNA, complete cds
AT4G14280	-1.234559658	0.04564636	Arabidopsis thaliana ARM repeat superfamily protein mRNA, complete cds
AT5G38344	-1.234559658	0.04564636	Arabidopsis thaliana chromosome 5 sequence
AT4G10270	-1.234559658	0.04564636	Arabidopsis thaliana chromosome 4 sequence
AT5G49190	-1.106454832	0.046977345	Arabidopsis thaliana sucrose synthase 2 mRNA, complete cds
AT5G10278	-1.106454832	0.046977345	Arabidopsis thaliana Full-length cDNA Complete sequence from clone GSLTPGH59ZH07 of Hormone Treated Callus of strain col-0 of Arabidopsis

			thaliana (thale cress)
ATCG00400	-1.539414239	0.047021045	Oltmannsiellopsis viridis chloroplast, complete genome
AT5G51210	-1.539414239	0.047021045	Arabidopsis thaliana oleosin3 mRNA, complete cds
AT1G12100	-1.539414239	0.047021045	Arabidopsis thaliana Bifunctional inhibitor/lipid-transfer protein/seed storage 2S albumin superfamily protein mRNA, complete cds
AT5G49070	-1.539414239	0.047021045	Arabidopsis thaliana chromosome 5 sequence
AT5G55010	-2.539414239	0.051404249	Arabidopsis thaliana chromosome 5 sequence
AT3G56891	-2.539414239	0.051404249	Arabidopsis thaliana metal ion binding protein mRNA, complete cds
AT3G09680	-2.539414239	0.051404249	Arabidopsis thaliana 40S ribosomal protein S23-1 mRNA, complete cds
AT4G22050	-2.539414239	0.051404249	Arabidopsis thaliana aspartyl protease family protein mRNA, complete cds
AT5G45040	-2.539414239	0.051404249	Arabidopsis thaliana cytochrome c6 mRNA, complete cds
AT5G48130	-2.539414239	0.051404249	Arabidopsis thaliana phototropic-responsive NPH3 family protein mRNA, complete cds
AT4G00530	-2.539414239	0.051404249	Arabidopsis thaliana uncharacterized protein mRNA, complete cds
AT1G62190	-2.539414239	0.051404249	Arabidopsis thaliana chromosome 1 sequence
AT5G41730	-2.539414239	0.051404249	Arabidopsis thaliana protein kinase mRNA, complete cds
AT4G31640	-2.539414239	0.051404249	Arabidopsis thaliana B3 domain-containing protein REM5 mRNA, complete cds
AT5G39460	-2.539414239	0.051404249	Arabidopsis thaliana chromosome 5 sequence
AT4G33310	-2.539414239	0.051404249	Arabidopsis thaliana chromosome 4 sequence
AT2G38890	-2.539414239	0.051404249	Arabidopsis thaliana uncharacterized protein mRNA, complete cds
AT1G28730	-2.539414239	0.051404249	Arabidopsis thaliana chromosome 1 sequence
AT2G27120	-2.539414239	0.051404249	Arabidopsis thaliana DNA polymerase epsilon catalytic subunit B mRNA, complete cds

AT3G62320	-2.539414239	0.051404249	Arabidopsis thaliana putative nucleic acid binding protein mRNA, complete cds
AT4G22210	-2.539414239	0.051404249	Arabidopsis thaliana clone asmbl_12628 defensin-like mRNA sequence
AT5G26630	-1.691417333	0.052343347	Arabidopsis thaliana chromosome 5 sequence
AT1G52110	-1.691417333	0.052343347	Arabidopsis thaliana jacalin-like lectin domain-containing protein mRNA, complete cds
AT3G30530	-1.691417333	0.052343347	Arabidopsis thaliana chromosome 3, complete sequence
AT1G60989	-1.691417333	0.052343347	Arabidopsis thaliana protein SCR-like 7 mRNA, complete cds
AT1G47630	-1.691417333	0.052343347	Arabidopsis thaliana chromosome 1 sequence
AT4G32540	-1.691417333	0.052343347	Arabidopsis thaliana flavin-containing monooxygenase YUCCA1 mRNA, complete cds
AT1G35310	-1.691417333	0.052343347	Arabidopsis thaliana MLP-like protein 168 mRNA, complete cds
AT4G01500	-1.691417333	0.052343347	Arabidopsis thaliana B3 domain-containing transcription factor NGA4 mRNA, complete cds
AT5G37240	-1.691417333	0.052343347	Arabidopsis thaliana uncharacterized protein mRNA, complete cds
AT3G44120	-1.276379833	0.053981564	Arabidopsis thaliana chromosome 3, complete sequence
AT5G10570	-1.276379833	0.053981564	Arabidopsis thaliana transcription factor bHLH61 mRNA, complete cds
AT5G07040	-1.276379833	0.053981564	Arabidopsis thaliana chromosome 5 sequence
AT4G08990	-1.276379833	0.053981564	Arabidopsis thaliana DNA (cytosine-5-)-methyltransferase mRNA, complete cds
AT4G29610	-1.276379833	0.053981564	Arabidopsis thaliana chromosome 4 sequence
AT1G64480	-1.954451739	0.05536677	Arabidopsis thaliana calcineurin B-like protein 8 mRNA, complete cds
AT5G44420	-1.954451739	0.05536677	Arabidopsis thaliana ethylene- and jasmonate-responsive plant defensin mRNA, complete cds
AT4G10860	-1.954451739	0.05536677	Arabidopsis thaliana chromosome 4 sequence

AT3G14380	-1.954451739	0.05536677	Arabidopsis thaliana uncharacterized protein mRNA, complete cds
AT1G16230	-1.954451739	0.05536677	Arabidopsis thaliana Target SNARE coiled-coil domain protein mRNA, complete cds
AT5G54148	-1.954451739	0.05536677	Arabidopsis thaliana uncharacterized protein mRNA, complete cds
AT4G19000	-1.954451739	0.05536677	Arabidopsis thaliana INTERACTS WITH SPT6-like protein IWS2 mRNA, complete cds
AT1G31983	-1.954451739	0.05536677	Arabidopsis thaliana chromosome 1 sequence
AT1G74875	-1.954451739	0.05536677	Arabidopsis thaliana uncharacterized protein mRNA, complete cds
AT1G69320	-1.954451739	0.05536677	Arabidopsis thaliana chromosome 1 sequence
AT4G03811	-1.954451739	0.05536677	Arabidopsis thaliana mRNA for hypothetical protein, complete cds, clone: RAFL22-88-K15
AT2G34315	-1.954451739	0.05536677	Arabidopsis thaliana avirulence induced family protein mRNA, complete cds
AT4G03823	-1.954451739	0.05536677	Arabidopsis thaliana chromosome 4 sequence
AT3G51642	-1.954451739	0.05536677	Arabidopsis thaliana chromosome 3, complete sequence
AT4G01190	-1.954451739	0.05536677	Arabidopsis thaliana phosphatidylinositol phosphate kinase 10 mRNA, complete cds
ATCG00650	-1.954451739	0.05536677	Arabidopsis thaliana chloroplast DNA, complete genome, ecotype: Columbia
AT1G55290	-1.024841066	0.05584165	Arabidopsis thaliana feruloyl CoA ortho-hydroxylase 2 mRNA, complete cds
AT3G43580	-1.024841066	0.05584165	Arabidopsis thaliana chromosome 3, complete sequence
AT3G49510	-1.12437674	0.056242678	Arabidopsis thaliana F-box protein mRNA, complete cds
AT1G61230	-1.332963362	0.063571665	Arabidopsis thaliana jacalin-like lectin domain-containing protein mRNA, complete cds
AT1G22600	-1.332963362	0.063571665	Arabidopsis thaliana Late embryogenesis abundant protein (LEA) family protein mRNA, complete cds
AT5G40410	-1.332963362	0.063571665	Arabidopsis thaliana chromosome 5

			sequence
AT3G57440	-1.332963362	0.063571665	Arabidopsis thaliana uncharacterized protein mRNA, complete cds
AT1G23510	-1.147096817	0.067381599	Arabidopsis thaliana uncharacterized protein mRNA, complete cds
AT3G62725.1	-1.147096817	0.067381599	Arabidopsis thaliana chromosome 3, complete sequence
AT3G62499	-1.147096817	0.067381599	Arabidopsis thaliana chromosome 3, complete sequence
AT5G23160	-1.147096817	0.067381599	Arabidopsis thaliana uncharacterized protein mRNA, complete cds
AT1G05220	-1.413883357	0.074277221	Arabidopsis thaliana putative transmembrane protein 97 mRNA, complete cds
AT4G02670	-1.413883357	0.074277221	Arabidopsis thaliana indeterminate-domain 12 protein mRNA, complete cds
AT1G52270	-1.413883357	0.074277221	Arabidopsis thaliana chromosome 1 sequence
AT5G49340	-1.413883357	0.074277221	Arabidopsis thaliana protein TRICHOME BIREFRINGENCE-LIKE mRNA, complete cds
AT1G13510	-1.413883357	0.074277221	Arabidopsis thaliana uncharacterized protein mRNA, complete cds
ATCG01130	-1.413883357	0.074277221	Arabidopsis thaliana chloroplast DNA, complete genome, ecotype: Columbia
AT1G72590	-1.413883357	0.074277221	Arabidopsis thaliana 3-oxo-5-alpha-steroid 4-dehydrogenase family protein mRNA, complete cds
AT1G49830	-1.413883357	0.074277221	Arabidopsis thaliana basic helix-loop-helix (bHLH) DNA-binding superfamily protein mRNA, complete cds
AT3G25060	-1.17684416	0.080753545	Arabidopsis thaliana chromosome 3, complete sequence
AT2G39510	-1.17684416	0.080753545	Arabidopsis thaliana nodulin MtN21-like transporter family protein mRNA, complete cds
AT2G36210	-1.04191458	0.081153453	Arabidopsis thaliana chromosome 2, complete sequence
AT1G49920	-1.539414239	0.085428419	Arabidopsis thaliana MuDR family transposase mRNA, complete cds
AT3G28380	-1.539414239	0.085428419	Arabidopsis thaliana ABC transporter B family member 17 mRNA, complete cds

AT4G32990	-1.539414239	0.085428419	Arabidopsis thaliana WD40 domain-containing protein mRNA, complete cds
AT4G21902	-1.539414239	0.085428419	Arabidopsis thaliana chromosome 4 sequence
AT5G55780	-1.539414239	0.085428419	Arabidopsis thaliana chromosome 5 sequence
AT1G78360	-1.539414239	0.085428419	Arabidopsis thaliana glutathione S-transferase TAU 21 mRNA, complete cds
AT1G61410	-1.539414239	0.085428419	Arabidopsis thaliana chromosome 1 sequence
AT4G21745	-1.539414239	0.085428419	Arabidopsis thaliana PAK-box/P21-Rho-binding family protein mRNA, complete cds
AT3G44800	-1.539414239	0.085428419	Arabidopsis thaliana Meprin and TRAF (MATH) homology domain-containing protein mRNA, complete cds
AT3G58780	-1.539414239	0.085428419	Arabidopsis thaliana agamous-like MADS-box protein AGL1 mRNA, complete cds
AT3G57110	-1.761806661	0.094834662	Arabidopsis thaliana chromosome 3, complete sequence
AT3G05858	-1.761806661	0.094834662	Arabidopsis thaliana chromosome 3, complete sequence
AT1G78206	-1.761806661	0.094834662	Arabidopsis thaliana chromosome 1 sequence
AT5G44140	-1.761806661	0.094834662	Arabidopsis thaliana prohibitin 7 mRNA, complete cds
AT5G35495.1	-1.761806661	0.094834662	Arabidopsis thaliana chromosome 5 sequence
AT5G03010	-1.761806661	0.094834662	Arabidopsis thaliana chromosome 5 sequence
AT4G10780	-1.761806661	0.094834662	Arabidopsis thaliana chromosome 4 sequence
AT3G44755	-1.761806661	0.094834662	Arabidopsis thaliana uncharacterized protein mRNA, complete cds
AT5G65575	-1.761806661	0.094834662	Arabidopsis thaliana Full-length cDNA Complete sequence from clone GSLTSIL28ZH03 of Silique of strain col-0 of Arabidopsis thaliana (thale cress)
AT2G28426	-2.276379833	0.095217602	Arabidopsis thaliana chromosome 2, complete sequence
AT1G23110	-2.276379833	0.095217602	Arabidopsis thaliana uncharacterized

			protein mRNA, complete cds
AT5G55507	-2.276379833	0.095217602	Arabidopsis thaliana chromosome 5 sequence
AT2G47530	-2.276379833	0.095217602	Arabidopsis thaliana Pollen Ole e 1 allergen and extensin family protein mRNA, complete cds
AT3G07150	-2.276379833	0.095217602	Arabidopsis thaliana uncharacterized protein mRNA, complete cds
AT5G19810	-2.276379833	0.095217602	Arabidopsis thaliana chromosome 5 sequence
AT5G51174	-2.276379833	0.095217602	Arabidopsis thaliana chromosome 5 sequence
AT3G49300	-2.276379833	0.095217602	Arabidopsis thaliana proline-rich family protein mRNA, complete cds
AT4G09120	-2.276379833	0.095217602	Arabidopsis thaliana chromosome 4 sequence
AT3G47760	-2.276379833	0.095217602	Arabidopsis thaliana ABC transporter A family member 5 mRNA, complete cds
AT1G79770	-2.276379833	0.095217602	Arabidopsis thaliana chromosome 1 sequence
ATMG00810	-2.276379833	0.095217602	Arabidopsis thaliana ecotype Landsberg erecta mitochondrion, complete genome
AT1G70220	-2.276379833	0.095217602	Arabidopsis thaliana anticodon-binding domain-containing protein mRNA, complete cds
AT1G80540	-2.276379833	0.095217602	Arabidopsis thaliana uncharacterized protein mRNA, complete cds
AT5G18240	-2.276379833	0.095217602	Arabidopsis thaliana myb-related protein 1 mRNA, complete cds
AT5G52720	-2.276379833	0.095217602	Arabidopsis thaliana copper transport family protein mRNA, complete cds
AT3G28155	-2.276379833	0.095217602	Arabidopsis thaliana uncharacterized protein mRNA, complete cds
AT2G19140.1	-2.276379833	0.095217602	Arabidopsis thaliana chromosome 2, complete sequence
AT5G22490	-2.276379833	0.095217602	Arabidopsis thaliana O-acyltransferase (WSD1-like) family protein mRNA, complete cds
AT1G49005	-2.276379833	0.095217602	Arabidopsis thaliana CLAVATA3/ESR (CLE)-related protein 11 mRNA, complete cds
AT3G60870	-2.276379833	0.095217602	Arabidopsis thaliana chromosome 3, complete sequence
AT1G12940	-2.276379833	0.095217602	Arabidopsis thaliana nitrate

			transporter2.5 mRNA, complete cds
AT2G33775	-2.276379833	0.095217602	Arabidopsis thaliana chromosome 2, complete sequence
AT2G05330	-2.276379833	0.095217602	Arabidopsis thaliana BTB/POZ domain-containing protein mRNA, complete cds
AT1G30950	-2.276379833	0.095217602	Arabidopsis thaliana chromosome 1 sequence
AT1G59930	-2.276379833	0.095217602	Arabidopsis thaliana chromosome 1 sequence
AT3G28360	-2.276379833	0.095217602	Arabidopsis thaliana ABC transporter B family member 16 mRNA, complete cds
AT5G37950	-1.217486144	0.096736616	Arabidopsis thaliana UDP-glycosyltransferase-like protein mRNA, complete cds
AT3G58060	-1.217486144	0.096736616	Arabidopsis thaliana putative metal tolerance protein C3 mRNA, complete cds
AT4G22070	-1.217486144	0.096736616	Arabidopsis thaliana WRKY DNA-binding protein 31 mRNA, complete cds
AT5G59305	-1.217486144	0.096736616	Arabidopsis thaliana uncharacterized protein mRNA, complete cds
AT3G27620	-1.217486144	0.096736616	Arabidopsis thaliana alternative oxidase 1C mRNA, complete cds
AT5G42655	-1.053987412	0.098177886	Arabidopsis thaliana chromosome 5 sequence
AT5G50175	-1.053987412	0.098177886	Arabidopsis thaliana chromosome 5 sequence
AT3G44765	-1.276379833	0.115631763	Arabidopsis thaliana Full-length cDNA Complete sequence from clone GSLTPGH55ZA10 of Hormone Treated Callus of strain col-0 of Arabidopsis thaliana (thale cress)
AT3G56890	-1.276379833	0.115631763	Arabidopsis thaliana F-box associated ubiquitination effector family protein mRNA, complete cds
AT1G63370	-1.276379833	0.115631763	Arabidopsis thaliana flavin-containing monooxygenase FMO GS-OX-like 5 mRNA, complete cds
AT5G46871	-1.276379833	0.115631763	Arabidopsis thaliana defensin-like protein 308 mRNA, complete cds
AT5G52471	-1.276379833	0.115631763	Arabidopsis thaliana chromosome 5 sequence

AT1G34044	-1.276379833	0.115631763	Arabidopsis thaliana uncharacterized protein mRNA, complete cds
AT2G46480	-1.276379833	0.115631763	Arabidopsis thaliana probable galacturonosyltransferase 2 mRNA, complete cds
AT2G37530	-1.276379833	0.115631763	Arabidopsis thaliana chromosome 2, complete sequence
AT3G17600	-1.276379833	0.115631763	Arabidopsis thaliana auxin-responsive protein IAA31 mRNA, complete cds
AT1G09380	-1.276379833	0.115631763	Arabidopsis thaliana nodulin MtN21-like transporter family protein mRNA, complete cds
AT2G35742	-1.276379833	0.115631763	Arabidopsis thaliana chromosome 2, complete sequence
AT1G04090	-1.069928956	0.11910675	Arabidopsis thaliana uncharacterized protein mRNA, complete cds
AT1G53690	-1.069928956	0.11910675	Arabidopsis thaliana uncharacterized protein mRNA, complete cds
AT1G55790	-1.069928956	0.11910675	Arabidopsis thaliana uncharacterized protein mRNA, complete cds
AT5G23270	-1.069928956	0.11910675	Arabidopsis thaliana sugar transport protein 11 mRNA, complete cds
AT2G28056	-1.369489238	0.137342286	Arabidopsis thaliana mRNA for unknown protein, complete cds, clone: RAFL21-01-E23
AT4G16230	-1.369489238	0.137342286	Arabidopsis thaliana GDSL-like lipase mRNA, complete cds
AT1G05577	-1.369489238	0.137342286	Arabidopsis thaliana uncharacterized protein mRNA, complete cds
AT2G37000	-1.369489238	0.137342286	Arabidopsis thaliana chromosome 2, complete sequence
AT4G19750	-1.369489238	0.137342286	Arabidopsis thaliana Glycosyl hydrolase family protein with chitinase insertion domain mRNA, complete cds
AT1G28840	-1.369489238	0.137342286	Theobroma cacao Uncharacterized protein (TCM_007238) mRNA, complete cds
AT3G51570	-1.369489238	0.137342286	Arabidopsis thaliana TIR-NBS-LRR class disease resistance protein mRNA, complete cds
AT3G07260	-1.369489238	0.137342286	Arabidopsis thaliana SMAD/FHA domain-containing protein mRNA, complete cds
AT1G58300	-1.369489238	0.137342286	Arabidopsis thaliana heme oxygenase 4

			mRNA, complete cds
AT2G05995	-1.369489238	0.137342286	Arabidopsis thaliana clone 10529 mRNA sequence
AT3G44410	-1.369489238	0.137342286	Arabidopsis thaliana chromosome 3, complete sequence
AT5G28440.1	-1.369489238	0.137342286	Arabidopsis thaliana chromosome 5 sequence
AT5G28460	-1.091955262	0.144955657	Arabidopsis thaliana chromosome 5 sequence
AT1G03445	-1.091955262	0.144955657	Arabidopsis thaliana serine/threonine-protein phosphatase BSU1 mRNA, complete cds
AT1G19371	-1.091955262	0.144955657	Arabidopsis thaliana chromosome 1 sequence
ATCG00590	-1.091955262	0.144955657	Arabidopsis thaliana chloroplast petL gene for cyt b6/f complex subunit VI, strain Wassilewskija (Ws-2)
AT3G62510	-1.091955262	0.144955657	Arabidopsis thaliana protein disulfide isomerase-like protein mRNA, complete cds
AT1G73700	-1.091955262	0.144955657	Arabidopsis thaliana MATE efflux family protein mRNA, complete cds
AT1G02405	-1.091955262	0.144955657	Arabidopsis thaliana chromosome 1 sequence
AT1G65110	-1.091955262	0.144955657	Arabidopsis thaliana ubiquitin carboxyl-terminal hydrolase-related protein mRNA, complete cds

Supplementary Data S4A Up regulated genes in fc1(+NaCl)/fc1(-NaCl)

Gene	Log2FoldChange	pval	NT:Description
AT1G20450	4.20612252	0	Arabidopsis thaliana dehydrin ERD10 mRNA, complete cds
AT2G34420	1.353324149	0	Arabidopsis thaliana chromosome 2, complete sequence
AT2G39800	4.851747735	0	Arabidopsis thaliana delta1-pyrroline-5-carboxylate synthase 1 mRNA, complete cds
AT1G20440	3.275308071	0	Arabidopsis thaliana dehydrin COR47 mRNA, complete cds
AT2G33380	5.804558282	0	Arabidopsis thaliana caleosin 3 mRNA, complete cds
AT5G52310	5.926963564	0	Arabidopsis thaliana protein LOW-TEMPERATURE-INDUCED 78 mRNA, complete cds
AT5G25610	2.67937577	0	Arabidopsis thaliana dehydration-responsive protein RD22 mRNA, complete cds
AT1G29920	1.135393832	0	Arabidopsis thaliana chromosome 1 sequence
AT2G05100	1.38735047	0	Arabidopsis thaliana photosystem II light harvesting complex protein 2.1 mRNA, complete cds
AT1G76180	2.318867841	0	Arabidopsis thaliana dehydrin ERD14 mRNA, complete cds
AT2G38470	4.137053101	0	Arabidopsis thaliana putative WRKY transcription factor 33 mRNA, complete cds
AT2G22240	4.605142303	0	Arabidopsis thaliana myo-inositol-1-phosphate synthase 2 mRNA, complete cds
AT2G40000	3.789025987	0	Arabidopsis thaliana chromosome 2, complete sequence
AT3G23920	2.847797663	0	Arabidopsis thaliana beta-amylase 1 mRNA, complete cds
AT5G59320	6.445376903	0	Arabidopsis thaliana non-specific lipid-transfer protein 3 mRNA, complete cds
AT1G54100	3.863245601	0	Arabidopsis thaliana aldehyde dehydrogenase 7B4 mRNA, complete cds
AT3G50930	5.148589056	0	Arabidopsis thaliana chromosome 3, complete sequence
AT2G42540	7.649977661	0	Arabidopsis thaliana cold-regulated protein 15a mRNA, complete cds
AT2G15970	4.082433422	0	Arabidopsis thaliana cold regulated 413

			plasma membrane 1 mRNA, complete cds
AT5G15970	5.449439775	0	Arabidopsis thaliana stress-induced protein KIN2 mRNA, complete cds
AT1G32640	3.775105286	0	Arabidopsis thaliana chromosome 1 sequence
AT4G11280	4.897770152	0	Arabidopsis thaliana 1-aminocyclopropane-1-carboxylate synthase 6 mRNA, complete cds
AT3G54890	1.084257903	0	Arabidopsis thaliana chlorophyll a-b binding protein 6 mRNA, complete cds
AT2G05070	1.403033275	0	Arabidopsis thaliana photosystem II light harvesting complex protein 2.2 mRNA, complete cds
AT4G34710	2.740994651	0	Arabidopsis thaliana chromosome 4 sequence
AT4G30960	2.624611741	0	Arabidopsis thaliana chromosome 4 sequence
AT3G11410	3.598712373	0	Arabidopsis thaliana protein phosphatase 2CA mRNA, complete cds
AT1G61890	4.299741484	0	Arabidopsis thaliana MATE efflux family protein mRNA, complete cds
AT1G73480	4.905426666	0	Arabidopsis thaliana alpha/beta-Hydrolases superfamily protein mRNA, complete cds
AT5G67030	1.812955219	0	Arabidopsis thaliana zeaxanthin epoxidase mRNA, complete cds
AT1G02205	2.692611156	0	Arabidopsis thaliana protein ECERIFERUM 1 mRNA, complete cds
AT1G78070	3.62004448	0	Arabidopsis thaliana transducin/WD40 repeat-like superfamily protein mRNA, complete cds
AT4G27520	2.035522517	0	Arabidopsis thaliana early nodulin-like protein 2 mRNA, complete cds
AT4G29780	5.51551842	0	Arabidopsis thaliana chromosome 4 sequence
AT5G42050	2.142939812	0	Arabidopsis thaliana DCD (Development and Cell Death) domain protein mRNA, complete cds
AT1G09310	1.434478661	0	Arabidopsis thaliana chromosome 1 sequence
AT5G24770	3.036414863	0	Arabidopsis thaliana acid phosphatase VSP2 mRNA, complete cds
AT4G34000	3.119212781	0	Arabidopsis thaliana abscisic acid responsive elements-binding factor 3 mRNA, complete cds
AT1G56600	5.993343631	0	Arabidopsis thaliana galactinol synthase 2 mRNA, complete cds
AT4G26080	3.473111349	0	Arabidopsis thaliana protein phosphatase 2C 56 mRNA, complete cds

AT1G19180	4.625602813	0	Arabidopsis thaliana chromosome 1 sequence
AT5G64310	2.757463522	0	Arabidopsis thaliana chromosome 5 sequence
AT5G60360	1.566682058	0	Arabidopsis thaliana thiol protease aleurain mRNA, complete cds
AT2G41190	5.459233935	0	Arabidopsis thaliana transmembrane amino acid transporter family protein mRNA, complete cds
AT5G59220	6.752968469	0	Arabidopsis thaliana putative protein phosphatase 2C 78 mRNA, complete cds
AT5G08790	4.156772863	0	Arabidopsis thaliana protein ATAF2 mRNA, complete cds
AT5G63790	3.883054637	0	Arabidopsis thaliana NAC domain-containing protein 102 mRNA, complete cds
AT1G72770	2.484545511	0	Arabidopsis thaliana protein phosphatase 2C 16 mRNA, complete cds
AT3G29575	5.293229403	0	Arabidopsis thaliana Ninja-family protein AFP3 mRNA, complete cds
AT4G23600	5.097039447	0	Arabidopsis thaliana cystine lyase CORI3 mRNA, complete cds
AT4G05050	1.49297138	0	Arabidopsis thaliana chromosome 4 sequence
AT3G14067	1.623289321	0	Arabidopsis thaliana chromosome 3, complete sequence
AT1G60190	6.822459912	0	Arabidopsis thaliana chromosome 1 sequence
AT3G61890	4.44881732	0	Arabidopsis thaliana homeobox-leucine zipper protein ATHB-12 mRNA, complete cds
AT2G23120	2.822808094	0	Arabidopsis thaliana chromosome 2, complete sequence
AT3G22830	8.449760623	0	Arabidopsis thaliana heat stress transcription factor A-6b mRNA, complete cds
AT4G04020	2.69926394	0	Arabidopsis thaliana fibrillin mRNA, complete cds
AT1G01470	3.192814763	0	Arabidopsis thaliana putative desiccation-related protein LEA14 mRNA, complete cds
AT3G27690	1.949052524	0	Arabidopsis thaliana photosystem II light harvesting complex protein 2.3 mRNA, complete cds
AT4G22240	2.158684756	0	Arabidopsis thaliana plastid-lipid associated protein PAP / fibrillin family protein mRNA, complete cds
AT4G21990	3.199070039	0	Arabidopsis thaliana 5'-adenylylsulfate reductase 3 mRNA, complete cds
AT5G05410	5.763545866	0	Arabidopsis thaliana dehydration-responsive

			element-binding protein 2A mRNA, complete cds
AT4G16760	2.315523441	0	Arabidopsis thaliana peroxisomal acyl-coenzyme A oxidase 1 mRNA, complete cds
AT3G02480	9.710971482	0	Arabidopsis thaliana Late embryogenesis abundant protein (LEA) family protein mRNA, complete cds
AT1G68530	1.418869423	0	Arabidopsis thaliana chromosome 1 sequence
AT4G24960	3.383619189	0	Arabidopsis thaliana HVA22-like protein d mRNA, complete cds
AT5G50950	3.841779423	0	Arabidopsis thaliana fumarate hydratase 2 mRNA, complete cds
AT4G27410	5.012607218	0	Arabidopsis thaliana NAC transcription factor RD26 mRNA, complete cds
AT5G24120	2.793100825	0	Arabidopsis thaliana RNA polymerase sigma factor 5 mRNA, complete cds
AT1G62510	6.761967494	0	Arabidopsis thaliana chromosome 1 sequence
AT5G61820	3.316096307	0	Arabidopsis thaliana uncharacterized protein mRNA, complete cds
AT5G59310	8.057894273	0	Arabidopsis thaliana non-specific lipid-transfer protein 4 mRNA, complete cds
AT4G32020	1.56382103	0	Arabidopsis thaliana chromosome 4 sequence
AT1G49450	5.768958121	0	Arabidopsis thaliana chromosome 1 sequence
AT1G01720	3.171012671	0	Arabidopsis thaliana putative transcriptional activator with NAC domain mRNA, complete cds
AT3G55980	2.825285356	0	Arabidopsis thaliana salt-inducible zinc finger 1 mRNA, complete cds
AT3G14440	6.836543791	0	Arabidopsis thaliana chromosome 3, complete sequence
AT1G69260	5.125621191	0	Arabidopsis thaliana ABI five binding protein mRNA, complete cds
AT2G46270	3.753647813	0	Arabidopsis thaliana G-box binding factor 3 mRNA, complete cds
AT2G17840	2.552903469	0	Arabidopsis thaliana senescence/dehydration related protein mRNA, complete cds
AT1G09070	2.110271655	0	Arabidopsis thaliana chromosome 1 sequence
AT4G37390	4.737528805	0	Arabidopsis thaliana indole-3-acetic acid-amido synthetase GH3.2 mRNA, complete cds
AT2G46680	4.23427168	0	Arabidopsis thaliana homeobox-leucine zipper protein ATHB-7 mRNA, complete cds

AT1G27730	5.109970771	0	Arabidopsis thaliana chromosome 1 sequence
AT2G34430	1.312124483	0	Arabidopsis thaliana chromosome 2, complete sequence
AT2G40140	2.767687953	0	Arabidopsis thaliana zinc finger CCCH domain-containing protein 29 mRNA, complete cds
AT1G73390	2.795340439	0	Arabidopsis thaliana Endosomal targeting BRO1-like domain-containing protein mRNA, complete cds
AT1G32900	1.903088897	0	Arabidopsis thaliana granule-bound starch synthase 1 mRNA, complete cds
AT4G23630	1.64228376	0	Arabidopsis thaliana VIRB2-interacting protein 1 mRNA, complete cds
AT1G79270	2.342382968	0	Arabidopsis thaliana uncharacterized protein mRNA, complete cds
AT3G17800	1.967347262	0	Arabidopsis thaliana uncharacterized protein mRNA, complete cds
AT3G56880	2.468222562	0	Arabidopsis thaliana chromosome 3, complete sequence
AT1G58270	2.856819089	0	Arabidopsis thaliana protein ZW9 mRNA, complete cds
AT5G11110	4.247584597	0	Arabidopsis thaliana sucrose phosphate synthase 2F mRNA, complete cds
AT5G47560	2.127852192	0	Arabidopsis thaliana tonoplast dicarboxylate transporter mRNA, complete cds
AT1G80840	5.615074355	0	Arabidopsis thaliana putative WRKY transcription factor 40 mRNA, complete cds
AT2G22430	1.581042774	0	Arabidopsis thaliana homeobox-leucine zipper protein ATHB-6 mRNA, complete cds
AT2G47180	4.357924126	0	Arabidopsis thaliana galactinol synthase 1 mRNA, complete cds
AT1G14540	4.954052628	0	Arabidopsis thaliana peroxidase 4 mRNA, complete cds
AT3G50970	4.086915535	0	Arabidopsis thaliana chromosome 3, complete sequence
AT5G47640	2.312498452	0	Arabidopsis thaliana chromosome 5 sequence
AT5G63160	1.916697453	0	Arabidopsis thaliana BTB and TAZ domain protein 1 mRNA, complete cds
AT1G48100	3.934718256	0	Arabidopsis thaliana polygalacturonase mRNA, complete cds
AT4G04610	2.162894933	0	Arabidopsis thaliana 5'-adenylylsulfate reductase 1 mRNA, complete cds
AT1G06570	2.409850906	0	Arabidopsis thaliana 4-hydroxyphenylpyruvate dioxygenase

			mRNA, complete cds
AT2G46830	1.08946562	0	Arabidopsis thaliana protein CCA1 mRNA, complete cds
AT1G36370	2.816184519	0	Arabidopsis thaliana putative serine hydroxymethyltransferase mRNA, complete cds
AT2G23170	4.248927599	0	Arabidopsis thaliana indole-3-acetic acid-amido synthetase GH3.3 mRNA, complete cds
AT4G17490	4.546234635	0	Arabidopsis thaliana chromosome 4 sequence
AT4G39800	1.251622453	0	Arabidopsis thaliana myo-inositol-1-phosphate synthase 1 mRNA, complete cds
AT2G37180	1.978502036	0	Arabidopsis thaliana aquaporin PIP2-3 mRNA, complete cds
AT2G46400	5.034278257	0	Arabidopsis thaliana putative WRKY transcription factor 46 mRNA, complete cds
AT5G59450	3.096005743	0	Arabidopsis thaliana chromosome 5 sequence
AT1G52690	8.824953366	0	Arabidopsis thaliana Late embryogenesis abundant protein (LEA) family protein mRNA, complete cds
AT2G04350	2.562025107	0	Arabidopsis thaliana long chain acyl-CoA synthetase 8 mRNA, complete cds
AT1G61340	6.343380008	0	Arabidopsis thaliana F-box stress induced 1 mRNA, complete cds
AT1G29395	5.689897682	0	Arabidopsis thaliana cold regulated 314 inner membrane 1 mRNA, complete cds
AT5G52300	10.75016279	0	Arabidopsis thaliana protein LOW-TEMPERATURE-INDUCED 65 mRNA, complete cds
AT2G22470	2.749898516	0	Arabidopsis thaliana chromosome 2, complete sequence
AT1G80920	1.586044981	0	Arabidopsis thaliana chaperone protein dnaJ 8 mRNA, complete cds
AT4G20830	1.505888851	0	Arabidopsis thaliana FAD-binding Berberine family protein mRNA, complete cds
AT1G62570	3.199390264	0	Arabidopsis thaliana flavin-containing monooxygenase FMO GS-OX4 mRNA, complete cds
AT3G15450	1.429157682	0	Arabidopsis thaliana aluminum induced protein with YGL and LRDR motif mRNA, complete cds
AT5G67300	2.257221185	0	Arabidopsis thaliana chromosome 5 sequence
AT5G02020	3.954705874	0	Arabidopsis thaliana uncharacterized protein

			mRNA, complete cds
AT5G19110	4.387504747	0	Arabidopsis thaliana Eukaryotic aspartyl protease family protein mRNA, complete cds
AT3G05640	3.973327697	0	Arabidopsis thaliana putative protein phosphatase 2C mRNA, complete cds
AT1G19570	1.609921935	0	Arabidopsis thaliana dehydroascorbate reductase mRNA, complete cds
AT3G54500	1.025951	0	Arabidopsis thaliana uncharacterized protein mRNA, complete cds
AT5G06320	1.852215735	0	Arabidopsis thaliana chromosome 5 sequence
AT3G62260	3.441564718	0	Arabidopsis thaliana putative protein phosphatase 2C 49 mRNA, complete cds
AT2G41100	1.353002552	0	Arabidopsis thaliana calmodulin-like protein 4 mRNA, complete cds
AT3G23250	5.499652696	0	Arabidopsis thaliana myb domain protein 15 mRNA, complete cds
AT5G66400	7.0079195	0	Arabidopsis thaliana dehydrin Rab18 mRNA, complete cds
AT4G30470	2.323956849	0	Arabidopsis thaliana cinnamoyl-CoA reductase like protein mRNA, complete cds
AT4G24570	4.148673581	0	Arabidopsis thaliana chromosome 4 sequence
AT1G73540	3.394462363	0	Arabidopsis thaliana nudix hydrolase 21 mRNA, complete cds
AT1G06430	1.449169982	0	Arabidopsis thaliana ATP-dependent zinc metalloprotease FTSH 8 mRNA, complete cds
AT2G32150	2.755670587	0	Arabidopsis thaliana haloacid dehalogenase-like hydrolase domain-containing protein mRNA, complete cds
AT5G57560	2.894094291	0	Arabidopsis thaliana xyloglucan endotransglucosylase/hydrolase protein 22 mRNA, complete cds
AT4G37370	4.507683395	0	Arabidopsis thaliana cytochrome P450, family 81, subfamily D, polypeptide 8 mRNA, complete cds
AT2G41430	1.087662171	0	Arabidopsis thaliana dehydration-induced protein ERD15 mRNA, complete cds
AT5G54940	1.281288708	0	Arabidopsis thaliana translation initiation factor SUI1 family protein mRNA, complete cds
AT1G69490	4.065763015	0	Arabidopsis thaliana NAC transcription factor protein family mRNA, complete cds
AT2G35930	3.464301011	0	Arabidopsis thaliana chromosome 2,

			complete sequence
AT4G05020	3.301662855	0	Arabidopsis thaliana NAD(P)H dehydrogenase B2 mRNA, complete cds
AT3G19580	3.847796815	0	Arabidopsis thaliana chromosome 3, complete sequence
AT3G25250	6.446741827	0	Arabidopsis thaliana AGC (cAMP-dependent, cGMP-dependent and protein kinase C) kinase family protein mRNA, complete cds
AT5G64260	2.079873543	0	Arabidopsis thaliana chromosome 5 sequence
AT2G41870	3.410515972	0	Arabidopsis thaliana remorin-like protein mRNA, complete cds
AT5G01520	4.155791378	0	Arabidopsis thaliana C3HC4 type RING finger protein mRNA, complete cds
AT4G39330	1.338583468	0	Arabidopsis thaliana putative cinnamyl alcohol dehydrogenase 9 mRNA, complete cds
AT3G02800	4.584684553	0	Arabidopsis thaliana atypical dual-specificity phosphatase mRNA, complete cds
AT5G61600	3.126869769	0	Arabidopsis thaliana chromosome 5 sequence
AT5G42380	6.250379441	0	Arabidopsis thaliana chromosome 5 sequence
AT2G39010	1.328190374	0	Arabidopsis thaliana plasma membrane intrinsic protein 2E mRNA, complete cds
AT1G10370	3.42466912	0	Arabidopsis thaliana glutathione S-transferase U17 mRNA, complete cds
AT1G04220	3.225408491	0	Arabidopsis thaliana 3-ketoacyl-CoA synthase 2 mRNA, complete cds
AT3G14990	1.241874641	0	Arabidopsis thaliana protein DJ-1-like A mRNA, complete cds
AT3G10420	1.634901618	0	Arabidopsis thaliana protein seedling plastid development 1 mRNA, complete cds
AT1G74950	2.67992472	0	Arabidopsis thaliana protein TIFY 10B mRNA, complete cds
AT3G22370	3.008475957	0	Arabidopsis thaliana alternative oxidase 1A mRNA, complete cds
AT3G03470	2.290763478	0	Arabidopsis thaliana cytochrome P450, family 87, subfamily A, polypeptide 9 mRNA, complete cds
AT5G42650	1.98326109	0	Arabidopsis thaliana chromosome 5 sequence
AT2G25450	1.60161517	0	Arabidopsis thaliana 1-aminocyclopropane-1-carboxylate oxidase-like protein mRNA, complete cds
AT1G22930	1.209299996	0	Arabidopsis thaliana T-complex protein 11 mRNA, complete cds

AT5G18670	1.135675648	0	Arabidopsis thaliana putative beta-amylase BMY3 mRNA, complete cds
AT2G18700	1.943492313	0	Arabidopsis thaliana putative alpha,alpha-trehalose-phosphate synthase [UDP-forming] 11 mRNA, complete cds
AT2G44840	7.191739674	0	Arabidopsis thaliana chromosome 2, complete sequence
AT5G57050	3.768299799	0	Arabidopsis thaliana protein phosphatase 2C 77 mRNA, complete cds
AT2G46790	5.159044026	0	Arabidopsis thaliana two-component response regulator-like APRR9 mRNA, complete cds
AT5G20830	2.008135294	0	Arabidopsis thaliana sucrose synthase 1 mRNA, complete cds
AT5G15500	6.026194099	0	Arabidopsis thaliana chromosome 5 sequence
AT3G08720	2.914668953	0	Arabidopsis thaliana AT3G08720 mRNA, complete cds, clone: RAFL09-47-F23
AT1G22190	2.031088664	0	Arabidopsis thaliana chromosome 1 sequence
AT2G34810	3.463913673	0	Arabidopsis thaliana chromosome 2, complete sequence
AT2G26530	3.470164956	0	Arabidopsis thaliana chromosome 2, complete sequence
AT5G14780	1.821075127	0	Arabidopsis thaliana formate dehydrogenase mRNA, complete cds
AT5G06760	9.343380008	0	Arabidopsis thaliana late embryogenesis abundant protein 4-5 mRNA, complete cds
AT1G77450	4.187178168	0	Arabidopsis thaliana NAC domain containing protein 32 mRNA, complete cds
AT1G58520	1.795721499	0	Arabidopsis thaliana protein RXW8 mRNA, complete cds
AT1G22710	1.277105024	0	Arabidopsis thaliana sucrose transport protein SUC2 mRNA, complete cds
AT4G21570	2.122551336	0	Arabidopsis thaliana uncharacterized protein mRNA, complete cds
AT3G07700	1.888826642	0	Arabidopsis thaliana ABC1 kinase mRNA, complete cds
AT1G21400	4.02085316	0	Arabidopsis thaliana thiamin diphosphate-binding fold protein mRNA, complete cds
AT5G20230	2.504429067	0	Arabidopsis thaliana blue-copper-binding protein mRNA, complete cds
AT5G47220	5.445905724	0	Arabidopsis thaliana chromosome 5 sequence
AT1G04770	3.753014798	0	Arabidopsis thaliana tetratricopeptide repeat-containing protein mRNA, complete cds

AT3G17520	9.224906538	0	Arabidopsis thaliana chromosome 3, complete sequence
AT5G41750	3.111181374	0	Arabidopsis thaliana TIR-NBS-LRR class disease resistance protein mRNA, complete cds
AT3G04240	1.46855209	0	Arabidopsis thaliana putative UDP-N-acetylglucosamine--peptide N-acetylglucosaminyltransferase SEC mRNA, complete cds
AT1G17380	4.871315672	0	Arabidopsis thaliana protein TIFY 11A mRNA, complete cds
AT3G44880	1.930683069	0	Arabidopsis thaliana pheophorbide A oxygenase mRNA, complete cds
AT1G78680	2.03959926	0	Arabidopsis thaliana gamma-glutamyl hydrolase 2 mRNA, complete cds
AT3G23030	1.521237363	0	Arabidopsis thaliana auxin-responsive protein IAA2 mRNA, complete cds
AT3G06500	2.436336622	0	Arabidopsis thaliana protein alkaline/neutral invertase C mRNA, complete cds
AT1G17940	3.192041904	0	Arabidopsis thaliana Endosomal targeting BRO1-like domain-containing protein mRNA, complete cds
AT4G08870	2.105171988	3.28E-307	Arabidopsis thaliana arginine amidohydrolase 2 mRNA, complete cds
AT4G17500	4.056325454	2.33E-305	Arabidopsis thaliana chromosome 4 sequence
AT1G01520	3.964690059	2.44E-305	Arabidopsis thaliana transcription factor ASG4 mRNA, complete cds
AT1G79520	2.583430605	3.12E-304	Arabidopsis thaliana cation efflux family protein mRNA, complete cds
AT1G45249	2.333731952	8.50E-304	Arabidopsis thaliana abscisic acid responsive elements-binding factor 2 mRNA, complete cds
AT5G53120	2.164679181	1.36E-303	Arabidopsis thaliana Spermine synthase mRNA, complete cds
AT3G45140	1.033783554	1.84E-303	Arabidopsis thaliana lipoxygenase 2 mRNA, complete cds
AT1G23710	3.443530358	9.35E-303	Arabidopsis thaliana chromosome 1 sequence
AT3G47340	3.241582775	1.50E-302	Arabidopsis thaliana asparagine synthetase [glutamine-hydrolyzing] mRNA, complete cds
AT3G45640	1.70817684	2.23E-302	Arabidopsis thaliana mitogen-activated protein kinase 3 mRNA, complete cds
AT5G64870	5.618794202	5.91E-301	Arabidopsis thaliana SPFH/Band 7/PHB domain-containing membrane-associated

			protein family mRNA, complete cds
AT4G33150	2.355615616	1.96E-299	Arabidopsis thaliana lysine-ketoglutarate reductase/saccharopine dehydrogenase bifunctional enzyme mRNA, complete cds
AT3G57530	1.780112763	5.38E-298	Arabidopsis thaliana calcium-dependent protein kinase 32 mRNA, complete cds
AT4G22710	3.819803522	1.99E-295	Arabidopsis thaliana chromosome 4 sequence
AT4G11570	1.32726719	1.29E-292	Arabidopsis thaliana chromosome 4 sequence
AT1G66180	2.222225225	1.94E-292	Arabidopsis thaliana chromosome 1 sequence
AT1G72520	3.957185517	1.37E-290	Arabidopsis thaliana lipoxygenase 4 mRNA, complete cds
AT3G62700	1.617797751	2.70E-290	Arabidopsis thaliana ABC transporter C family member 14 mRNA, complete cds
AT1G07720	2.078779108	5.55E-290	Arabidopsis thaliana chromosome 1 sequence
AT5G40390	2.288263501	1.99E-289	Arabidopsis thaliana putative galactinol--sucrose galactosyltransferase 5 mRNA, complete cds
AT2G39200	2.581632197	7.69E-286	Arabidopsis thaliana protein MILDEW RESISTANCE LOCUS O 12 mRNA, complete cds
AT4G37760	1.415438602	1.32E-284	Arabidopsis thaliana squalene epoxidase 3 mRNA, complete cds
AT1G17870	3.698479959	3.94E-281	Arabidopsis thaliana S2P-like putative metalloprotease mRNA, complete cds
AT5G13170	8.078308828	1.02E-280	Arabidopsis thaliana senescence-associated protein 29 mRNA, complete cds
AT1G51140	2.281837591	5.03E-280	Arabidopsis thaliana transcription factor bHLH122 mRNA, complete cds
AT4G02890	1.350280818	2.91E-278	Arabidopsis thaliana chromosome 4 sequence
AT2G24850	5.862197196	2.28E-274	Arabidopsis thaliana tyrosine aminotransferase 3 mRNA, complete cds
AT3G17790	3.747410646	4.44E-272	Arabidopsis thaliana purple acid phosphatase 17 mRNA, complete cds
AT1G30370	5.586573748	8.65E-272	Arabidopsis thaliana chromosome 1 sequence
AT5G53970	2.243053885	1.27E-271	Arabidopsis thaliana tyrosine aminotransferase mRNA, complete cds
AT4G15530	1.605429388	1.90E-270	Arabidopsis thaliana pyruvate, phosphate dikinase 1 mRNA, complete cds
AT3G44260	3.708331012	4.37E-268	Arabidopsis thaliana chromosome 3, complete sequence
AT2G47780	6.064981443	7.48E-268	Arabidopsis thaliana Rubber elongation factor protein mRNA, complete cds
AT1G76600	3.809592475	1.97E-267	Arabidopsis thaliana chromosome 1 sequence

AT3G27250	6.351481464	3.55E-267	Arabidopsis thaliana chromosome 3, complete sequence
AT4G34230	2.215514757	2.69E-265	Arabidopsis thaliana cinnamyl alcohol dehydrogenase 5 mRNA, complete cds
AT1G58340	3.986996726	4.04E-265	Arabidopsis thaliana MATE family protein ZRIZ1 mRNA, complete cds
AT3G15210	3.049400504	9.40E-265	Arabidopsis thaliana chromosome 3, complete sequence
AT1G19660	1.091363836	1.35E-264	Arabidopsis thaliana putative wound-responsive protein mRNA, complete cds
AT3G50060	3.014528071	9.51E-264	Arabidopsis thaliana chromosome 3, complete sequence
AT5G15960	9.551700388	9.80E-262	Arabidopsis thaliana cold and ABA inducible protein kin1 mRNA, complete cds
AT1G64660	3.516169545	1.39E-260	Arabidopsis thaliana methionine gamma-lyase mRNA, complete cds
AT3G56275	7.641162015	3.47E-259	Arabidopsis thaliana chromosome 3, complete sequence
AT4G24160	2.573987548	1.51E-256	Arabidopsis thaliana lysophosphatidic acid acyltransferase mRNA, complete cds
AT4G31800	2.585054808	2.51E-256	Arabidopsis thaliana WRKY DNA-binding protein 18 mRNA, complete cds
AT5G17460	4.541573099	1.97E-255	Arabidopsis thaliana uncharacterized protein mRNA, complete cds
AT1G20510	2.275357778	2.17E-252	Arabidopsis thaliana OPC-8:0 CoA ligase1 mRNA, complete cds
AT5G01600	2.141830458	6.84E-251	Arabidopsis thaliana ferretin 1 mRNA, complete cds
AT1G51760	2.138029626	1.12E-250	Arabidopsis thaliana IAA-amino acid hydrolase IAR3 mRNA, complete cds
AT5G15450	1.301781442	1.70E-250	Arabidopsis thaliana chaperone protein ClpB3 mRNA, complete cds
AT1G07430	7.572597729	1.15E-248	Arabidopsis thaliana protein phosphatase 2C 3 mRNA, complete cds
AT3G25780	2.656063703	9.86E-248	Arabidopsis thaliana allene oxide cyclase 3 mRNA, complete cds
AT1G01140	2.038454998	4.36E-247	Arabidopsis thaliana CBL-interacting serine/threonine-protein kinase 9 mRNA, complete cds
AT1G24580	4.445003501	2.30E-246	Arabidopsis thaliana chromosome 1 sequence
AT1G51700	2.361228009	2.67E-246	Arabidopsis thaliana chromosome 1 sequence
AT1G09932	3.199113199	2.34E-245	Arabidopsis thaliana phosphoglycerate mutase family protein mRNA, complete cds

AT3G26290	5.268994292	7.32E-244	Arabidopsis thaliana cytochrome P450 71B26 mRNA, complete cds
AT5G43850	1.774528079	4.05E-241	Arabidopsis thaliana acireductone dioxygenase 4 mRNA, complete cds
AT2G47060	1.97370419	4.11E-241	Arabidopsis thaliana Pto-interacting 1-4 mRNA, complete cds
AT5G51070	1.097391383	3.84E-240	Arabidopsis thaliana chaperone protein ClpD mRNA, complete cds
AT3G44860	7.497925032	1.18E-237	Arabidopsis thaliana farnesoic acid carboxyl-O-methyltransferase mRNA, complete cds
AT3G17810	1.604234855	1.32E-236	Arabidopsis thaliana putative dihydropyrimidine dehydrogenase mRNA, complete cds
AT3G08730	1.232723973	2.80E-236	Arabidopsis thaliana protein-serine kinase 1 mRNA, complete cds
AT1G27760	1.786964622	1.65E-233	Arabidopsis thaliana protein salt tolerance 32 mRNA, complete cds
AT3G17690	4.443115211	1.19E-231	Arabidopsis thaliana cyclic nucleotide gated channel 19 mRNA, complete cds
AT1G62180	1.082124212	2.27E-231	Arabidopsis thaliana 5'-adenylylsulfate reductase 2 mRNA, complete cds
AT1G62540	3.138677908	6.98E-230	Arabidopsis thaliana flavin-containing monooxygenase FMO GS-OX2 mRNA, complete cds
AT5G37300	3.026211575	7.74E-229	Arabidopsis thaliana o-acyltransferase WSD1 mRNA, complete cds
AT4G14680	2.400863228	1.79E-228	Arabidopsis thaliana ATP sulfurylase mRNA, complete cds
AT5G66640	4.273190209	2.60E-227	Arabidopsis thaliana protein DA1-related 3 mRNA, complete cds
AT1G59590	3.880712168	2.89E-227	Arabidopsis thaliana chromosome 1 sequence
AT4G23050	2.683556908	7.45E-226	Arabidopsis thaliana PAS domain-containing protein tyrosine kinase family protein mRNA, complete cds
AT5G03190	1.921540709	1.04E-225	Arabidopsis thaliana chromosome 5 sequence
AT4G17550	2.746833922	1.75E-225	Arabidopsis thaliana glycerol-3-phosphate permease 4 mRNA, complete cds
AT5G35735	2.020300245	3.74E-224	Arabidopsis thaliana putative auxin-responsive protein mRNA, complete cds
AT1G56660	1.790792164	5.77E-224	Arabidopsis thaliana uncharacterized protein mRNA, complete cds
AT5G64170	1.519665357	1.61E-223	Arabidopsis thaliana dentin

			sialophosphoprotein-like protein mRNA, complete cds
AT3G24170	1.062044107	1.11E-222	Arabidopsis thaliana glutathione-disulfide reductase mRNA, complete cds
AT3G49530	2.166104162	1.43E-222	Arabidopsis thaliana transcription factor NTL6 mRNA, complete cds
AT3G52800	1.735646341	5.48E-222	Arabidopsis thaliana zinc finger A20 and AN1 domain-containing stress-associated protein 6 mRNA, complete cds
AT5G51190	4.066881411	3.78E-220	Arabidopsis thaliana chromosome 5 sequence
AT2G37870	6.725111421	1.47E-219	Arabidopsis thaliana protease inhibitor/seed storage/lipid transfer protein (LTP) family protein mRNA, complete cds
AT3G57520	1.223903117	2.17E-219	Arabidopsis thaliana putative galactinol--sucrose galactosyltransferase 2 mRNA, complete cds
AT2G28400	4.148347443	1.20E-216	Arabidopsis thaliana chromosome 2, complete sequence
AT5G59820	4.279665769	6.35E-216	Arabidopsis thaliana chromosome 5 sequence
AT1G01480	5.83403535	9.75E-216	Arabidopsis thaliana 1-aminocyclopropane-1-carboxylate synthase 2 mRNA, complete cds
AT4G02280	4.830593839	1.87E-215	Arabidopsis thaliana sucrose synthase 3 mRNA, complete cds
AT5G20900	1.65397895	2.27E-215	Arabidopsis thaliana protein TIFY 3B mRNA, complete cds
AT1G13930	1.239747161	9.63E-215	Arabidopsis thaliana salt tolerance-related protein mRNA, complete cds
AT5G06980	2.334771628	3.06E-213	Arabidopsis thaliana uncharacterized protein mRNA, complete cds
AT5G48850	5.363376476	5.32E-213	Arabidopsis thaliana protein SULPHUR DEFICIENCY-INDUCED 1 mRNA, complete cds
AT4G12720	1.56002969	8.37E-212	Arabidopsis thaliana nudix hydrolase 7 mRNA, complete cds
AT4G34150	1.418536402	8.40E-212	Arabidopsis thaliana calcium-dependent lipid-binding domain-containing protein mRNA, complete cds
AT3G24460	2.416246954	1.37E-211	Arabidopsis thaliana Serinc-domain containing serine and sphingolipid biosynthesis protein mRNA, complete cds
AT3G53180	1.509108321	1.80E-211	Arabidopsis thaliana nodulin/glutamine synthase-like protein mRNA, complete cds
AT3G28580	4.881435824	2.10E-210	Arabidopsis thaliana chromosome 3,

			complete sequence
AT3G48690	1.406878114	2.25E-208	Arabidopsis thaliana chromosome 3, complete sequence
AT2G42530	5.465014547	2.67E-208	Arabidopsis thaliana cold-regulated protein 15b mRNA, complete cds
AT5G64120	3.503293206	3.51E-208	Arabidopsis thaliana peroxidase 71 mRNA, complete cds
AT1G56250	5.256762807	3.17E-206	Arabidopsis thaliana F-box protein PP2-B14 mRNA, complete cds
AT2G43570	3.458899087	4.18E-206	Arabidopsis thaliana putative chitinase mRNA, complete cds
AT5G39580	2.865260236	7.62E-206	Arabidopsis thaliana peroxidase 62 mRNA, complete cds
AT1G56580	1.373032518	6.01E-205	Arabidopsis thaliana chromosome 1 sequence
AT3G17860	2.42448799	1.99E-204	Arabidopsis thaliana jasmonate-zim-domain protein 3 mRNA, complete cds
AT5G59480	1.601520763	5.04E-204	Arabidopsis thaliana Haloacid dehalogenase-like hydrolase (HAD) superfamily protein mRNA, complete cds
AT3G58190	5.664392262	7.31E-204	Arabidopsis thaliana protein ASYMMETRIC LEAVES 2-LIKE 16 mRNA, complete cds
AT3G25770	2.384999288	2.48E-203	Arabidopsis thaliana allene oxide cyclase 2 mRNA, complete cds
AT2G23810	1.666017698	1.08E-202	Arabidopsis thaliana tetraspanin8 mRNA, complete cds
AT2G03760	2.783219466	1.12E-202	Arabidopsis thaliana chromosome 2, complete sequence
AT1G17420	2.801599816	2.49E-202	Arabidopsis thaliana lipoxygenase 3 mRNA, complete cds
AT5G60680	1.995191323	6.30E-202	Arabidopsis thaliana chromosome 5 sequence
AT1G69480	5.280535407	9.48E-201	Arabidopsis thaliana EXS (ERD1/XPR1/SYG1) family protein mRNA, complete cds
AT3G47420	1.62472158	1.62E-200	Arabidopsis thaliana putative glycerol-3-phosphate transporter 1 mRNA, complete cds
AT3G46600	1.597048822	8.04E-200	Arabidopsis thaliana chromosome 3, complete sequence
AT3G21670	1.311978851	4.51E-199	Arabidopsis thaliana nitrate transporter 1.3 mRNA, complete cds
AT3G57020	1.959447763	2.33E-198	Arabidopsis thaliana calcium-dependent phosphotriesterase superfamily protein mRNA, complete cds
AT3G09390	1.443127977	2.90E-197	Arabidopsis thaliana metallothionein 2A

			mRNA, complete cds
AT2G33590	1.821428845	7.73E-196	Arabidopsis thaliana cinnamoyl-CoA:NADP oxidoreductase-like 1 mRNA, complete cds
AT1G02820	4.522413161	1.28E-195	Arabidopsis thaliana late embryogenesis abundant 3-like protein mRNA, complete cds
AT1G72450	2.058423211	3.00E-195	Arabidopsis thaliana jasmonate-zim-domain protein 6 mRNA, complete cds
AT3G07360	1.721183072	3.52E-195	Arabidopsis thaliana ARM domain-containing protein mRNA, complete cds
AT2G47770	7.83403535	1.04E-194	Arabidopsis thaliana chromosome 2, complete sequence
AT5G59550	2.477979597	2.57E-193	Arabidopsis thaliana chromosome 5 sequence
AT2G25460	3.855722717	4.47E-193	Arabidopsis thaliana uncharacterized protein mRNA, complete cds
AT5G02230	1.81206623	6.54E-193	Arabidopsis thaliana haloacid dehalogenase-like hydrolase superfamily protein mRNA, complete cds
AT4G17230	2.016258023	2.26E-192	Arabidopsis thaliana protein scarecrow-like 13 mRNA, complete cds
AT3G17000	1.810550629	1.41E-190	Arabidopsis thaliana ubiquitin-conjugating enzyme E2 32 mRNA, complete cds
AT4G27260	2.015453172	4.78E-190	Arabidopsis thaliana indole-3-acetic acid-amido synthetase GH3.5 mRNA, complete cds
AT1G19770	1.414363651	4.66E-189	Arabidopsis thaliana purine permease 14 mRNA, complete cds
AT5G01820	1.416768722	7.91E-189	Arabidopsis thaliana chromosome 5 sequence
AT4G21910	1.816744094	2.05E-188	Arabidopsis thaliana MATE efflux family protein mRNA, complete cds
AT5G05600	3.676364526	7.54E-188	Arabidopsis thaliana oxidoreductase, 2OG-Fe(II) oxygenase family protein mRNA, complete cds
AT1G51500	1.008157238	1.14E-187	Arabidopsis thaliana ABC transporter G family member 12 mRNA, complete cds
AT5G13740	1.775839655	2.58E-187	Arabidopsis thaliana zinc induced facilitator 1 proetin mRNA, complete cds
AT5G11650	1.914628803	5.80E-187	Arabidopsis thaliana alpha/beta fold hydrolase family protein mRNA, complete cds
AT3G47960	1.526471713	9.22E-186	Arabidopsis thaliana glucosinolate transporter 1 mRNA, complete cds
AT1G05100	6.644809793	1.25E-185	Arabidopsis thaliana chromosome 1 sequence
AT5G20190	1.546226251	2.06E-184	Arabidopsis thaliana tetratricopeptide repeat domain-containing protein mRNA, complete

			cds
AT1G66500	3.464414175	2.31E-184	Arabidopsis thaliana chromosome 1 sequence
AT4G17840	1.274234014	4.84E-184	Arabidopsis thaliana uncharacterized protein mRNA, complete cds
AT1G42990	2.018002855	1.34E-182	Arabidopsis thaliana bZIP transcription factor 60 mRNA, complete cds
AT5G52570	2.609723415	3.84E-182	Arabidopsis thaliana beta-carotene hydroxylase 2 mRNA, complete cds
AT1G05340	3.224548771	4.46E-182	Arabidopsis thaliana uncharacterized protein mRNA, complete cds
AT4G22820	2.23171401	1.63E-181	Arabidopsis thaliana chromosome 4 sequence
AT4G15490	2.780736819	4.91E-180	Arabidopsis thaliana chromosome 4 sequence
AT5G17760	2.352049317	5.31E-180	Arabidopsis thaliana chromosome 5 sequence
AT1G11960	2.076736914	1.05E-179	Arabidopsis thaliana putative ERD4 protein mRNA, complete cds
AT1G70290	1.299046261	2.38E-179	Arabidopsis thaliana putative alpha,alpha-trehalose-phosphate synthase [UDP-forming] 8 mRNA, complete cds
AT1G74930	5.480659429	2.49E-179	Arabidopsis thaliana chromosome 1 sequence
AT1G70700	1.373005177	1.30E-178	Arabidopsis thaliana putative jasmonate signaling protein JAZ9 mRNA, complete cds
AT1G21000	1.961191579	2.54E-178	Arabidopsis thaliana PLATZ transcription factor family protein mRNA, complete cds
AT2G22500	1.952664219	3.16E-178	Arabidopsis thaliana chromosome 2, complete sequence
AT1G78120	2.048681978	3.83E-178	Arabidopsis thaliana tetratricopeptide repeat-containing protein mRNA, complete cds
AT5G50100	1.959515001	6.42E-178	Arabidopsis thaliana putative thiol-disulfide oxidoreductase DCC mRNA, complete cds
AT3G02140	2.291898498	3.41E-177	Arabidopsis thaliana chromosome 3, complete sequence
AT4G35560	2.076020787	2.56E-176	Arabidopsis thaliana DUO1-activated WD40 1 mRNA, complete cds
AT3G22910	4.64809665	2.74E-176	Arabidopsis thaliana chromosome 3, complete sequence
AT1G67360	1.768031697	1.33E-175	Arabidopsis thaliana REF/SRPP-like protein mRNA, complete cds
AT5G47370	1.699701248	1.82E-175	Arabidopsis thaliana homeobox-leucine zipper protein HAT2 mRNA, complete cds
AT4G39210	2.380351306	2.33E-175	Arabidopsis thaliana glucose-1-phosphate adenylyltransferase large subunit 3 mRNA, complete cds

AT4G37790	1.420636771	2.41E-175	Arabidopsis thaliana homeobox-leucine zipper protein HAT22 mRNA, complete cds
AT4G36500	1.734241334	4.55E-175	Arabidopsis thaliana chromosome 4 sequence
AT5G48180	1.446910555	1.17E-174	Arabidopsis thaliana nitrile specifier protein 5 mRNA, complete cds
AT2G30020	2.380682022	4.40E-174	Arabidopsis thaliana putative protein phosphatase 2C 25 mRNA, complete cds
AT2G06050	2.129973378	1.14E-173	Arabidopsis thaliana AT2G06050 mRNA, complete cds, clone: RAFL22-42-B17
AT3G25760	3.917088043	1.84E-172	Arabidopsis thaliana allene oxide cyclase 1 mRNA, complete cds
AT1G43160	4.954052628	1.98E-172	Arabidopsis thaliana ethylene-responsive transcription factor RAP2-6 mRNA, complete cds
AT1G08920	2.172081351	3.24E-172	Arabidopsis thaliana sugar transporter ERD6-like 3 mRNA, complete cds
AT5G13200	2.979209652	1.09E-171	Arabidopsis thaliana GRAM domain family protein mRNA, complete cds
AT3G08860	5.126961488	1.40E-171	Arabidopsis thaliana PYRIMIDINE 4 mRNA, complete cds
AT4G37180	2.660885217	2.41E-171	Arabidopsis thaliana myb family transcription factor mRNA, complete cds
AT1G18740	2.043359169	3.51E-171	Arabidopsis thaliana uncharacterized protein mRNA, complete cds
AT1G53470	3.570871398	4.78E-171	Arabidopsis thaliana mechanosensitive channel of small conductance-like 4 mRNA, complete cds
AT2G30250	2.010937691	8.94E-171	Arabidopsis thaliana WRKY transcription factor 25 mRNA, complete cds
AT2G18193	4.115772706	1.28E-170	Arabidopsis thaliana P-loop containing nucleoside triphosphate hydrolases superfamily protein mRNA, complete cds
AT3G11420	1.975205582	4.59E-170	Arabidopsis thaliana uncharacterized protein mRNA, complete cds
AT1G28380	1.612539232	4.95E-170	Arabidopsis thaliana protein necrotic spotted lesions 1 mRNA, complete cds
AT4G23810	2.251893368	5.91E-170	Arabidopsis thaliana putative WRKY transcription factor 53 mRNA, complete cds
AT4G27280	2.614197493	7.32E-170	Arabidopsis thaliana chromosome 4 sequence
AT5G13220	4.480659429	1.28E-169	Arabidopsis thaliana protein TIFY 9 mRNA, complete cds
AT5G37540	2.620628894	2.70E-169	Arabidopsis thaliana chromosome 5 sequence
AT3G22200	1.661601734	6.37E-169	Arabidopsis thaliana gamma-aminobutyrate transaminase POP2 mRNA, complete cds

AT1G32870	2.511964733	7.96E-169	Arabidopsis thaliana NAC domain protein 13 mRNA, complete cds
AT4G15150	3.64908952	1.12E-168	Arabidopsis thaliana glycine-rich protein mRNA, complete cds
AT2G19810	2.79732049	3.39E-168	Arabidopsis thaliana chromosome 2, complete sequence
AT3G49220	1.121112585	4.08E-168	Arabidopsis thaliana putative pectinesterase/pectinesterase inhibitor 34 mRNA, complete cds
AT4G18010	1.988486125	8.52E-168	Arabidopsis thaliana Type I inositol-1,4,5-trisphosphate 5-phosphatase 2 mRNA, complete cds
AT1G17550	1.48000127	2.00E-167	Arabidopsis thaliana protein phosphatase 2C 7 mRNA, complete cds
AT5G43650	7.577695597	5.50E-167	Arabidopsis thaliana transcription factor bHLH92 mRNA, complete cds
AT4G01870	3.288471667	2.22E-165	Arabidopsis thaliana chromosome 4 sequence
AT5G51630	2.210358229	2.50E-165	Arabidopsis thaliana TIR-NBS-LRR class disease resistance protein mRNA, complete cds
AT5G35460	1.698145521	4.54E-165	Arabidopsis thaliana uncharacterized protein mRNA, complete cds
AT5G22630	2.070126079	1.75E-164	Arabidopsis thaliana chromosome 5 sequence
AT3G28180	1.637984217	1.25E-163	Arabidopsis thaliana xyloglucan glycosyltransferase 4 mRNA, complete cds
AT2G42790	1.863621329	3.71E-163	Arabidopsis thaliana citrate synthase 3 mRNA, complete cds
AT4G34410	9.935806028	1.45E-162	Arabidopsis thaliana chromosome 4 sequence
AT5G57800	1.323326397	2.11E-162	Arabidopsis thaliana protein ECERIFERUM 3 mRNA, complete cds
AT4G18280	3.579354058	7.63E-161	Arabidopsis thaliana chromosome 4 sequence
AT5G57010	3.962985753	6.10E-160	Arabidopsis thaliana calmodulin-binding family protein mRNA, complete cds
AT1G20823	2.059220823	5.08E-159	Arabidopsis thaliana chromosome 1 sequence
AT4G37980	1.528217084	2.90E-158	Arabidopsis thaliana cinnamyl alcohol dehydrogenase 7 mRNA, complete cds
AT5G65380	1.520496097	4.16E-158	Arabidopsis thaliana MATE efflux family protein mRNA, complete cds
AT2G46510	2.167695896	8.13E-158	Arabidopsis thaliana chromosome 2, complete sequence
AT1G80820	3.268433914	4.14E-157	Arabidopsis thaliana cinnamoyl-CoA reductase mRNA, complete cds
AT4G23190	1.846020273	8.28E-156	Arabidopsis thaliana cysteine-rich

			receptor-like protein kinase 11 mRNA, complete cds
AT3G45960	5.129469433	1.02E-155	Arabidopsis thaliana expansin-like A3 mRNA, complete cds
AT5G45630	7.092268769	1.10E-154	Arabidopsis thaliana chromosome 5 sequence
AT4G25470	6.35974908	2.14E-154	Arabidopsis thaliana chromosome 4 sequence
AT3G63060	7.447995073	2.20E-154	Arabidopsis thaliana chromosome 3, complete sequence
AT2G36630	1.76739253	3.57E-154	Arabidopsis thaliana Sulfite exporter TauE/SafE family protein mRNA, complete cds
AT5G43150	2.211373382	2.06E-152	Arabidopsis thaliana uncharacterized protein mRNA, complete cds
AT5G14640	1.41031279	3.58E-152	Arabidopsis thaliana Shaggy-related protein kinase 13 mRNA, complete cds
AT2G26380	7.064212484	5.15E-152	Arabidopsis thaliana chromosome 2, complete sequence
AT4G15210	5.999586767	1.23E-151	Arabidopsis thaliana beta-amylase 5 mRNA, complete cds
AT3G57540	4.017501001	1.55E-150	Arabidopsis thaliana Remorin family protein mRNA, complete cds
AT4G20320	1.772302191	7.52E-150	Arabidopsis thaliana putative CTP synthase mRNA, complete cds
AT3G55500	2.351296014	2.42E-149	Arabidopsis thaliana expansin A16 mRNA, complete cds
AT2G42750	1.127832102	3.38E-149	Arabidopsis thaliana DNAJ heat shock N-terminal domain-containing protein mRNA, complete cds
AT5G07920	1.843272197	3.48E-149	Arabidopsis thaliana diacylglycerol kinase1 mRNA, complete cds
AT5G62020	2.534552297	4.55E-149	Arabidopsis thaliana heat stress transcription factor B-2a mRNA, complete cds
AT1G08650	1.347744437	2.98E-148	Arabidopsis thaliana phosphoenolpyruvate carboxylase kinase 1 mRNA, complete cds
AT5G04340	3.545754457	3.84E-147	Arabidopsis thaliana chromosome 5 sequence
AT5G57610	1.601189859	3.87E-146	Arabidopsis thaliana octicosapeptide/Phox/Bem1p domain-containing protein kinase mRNA, complete cds
AT5G09620	1.428963433	5.04E-146	Arabidopsis thaliana chromosome 5 sequence
AT3G15540	3.155774461	1.06E-145	Arabidopsis thaliana auxin-responsive protein IAA19 mRNA, complete cds
AT4G18700	1.120915267	1.65E-145	Arabidopsis thaliana chromosome 4 sequence

AT3G48460	1.717830984	2.93E-145	Arabidopsis thaliana GDSL esterase/lipase mRNA, complete cds
AT5G24590	1.706739649	4.63E-145	Arabidopsis thaliana TCV-interacting protein mRNA, complete cds
AT2G29440	3.240873286	1.15E-144	Arabidopsis thaliana glutathione S-transferase tau 6 mRNA, complete cds
AT4G25490	9.714157839	3.89E-144	Arabidopsis thaliana chromosome 4 sequence
AT5G04760	2.463163907	1.97E-143	Arabidopsis thaliana duplicated SANT DNA-binding domain-containing protein mRNA, complete cds
AT1G62300	1.981900613	3.35E-143	Arabidopsis thaliana WRKY transcription factor 6 mRNA, complete cds
AT3G26910	1.95701988	8.24E-143	Arabidopsis thaliana hydroxyproline-rich glycoprotein family protein mRNA, complete cds
AT3G02840	5.149577452	1.37E-141	Arabidopsis thaliana chromosome 3, complete sequence
AT4G29190	2.477010048	3.37E-141	Arabidopsis thaliana chromosome 4 sequence
AT1G21790	2.621699232	3.86E-141	Arabidopsis thaliana TRAM, LAG1 and CLN8 (TLC) lipid-sensing domain containing protein mRNA, complete cds
AT2G46370	1.352750922	4.39E-141	Arabidopsis thaliana jasmonic acid-amido synthetase JAR1 mRNA, complete cds
AT3G22840	3.116539298	2.94E-140	Arabidopsis thaliana chlorophyll A-B binding, early light-inducible protein mRNA, complete cds
AT2G32020	5.507306269	3.32E-140	Arabidopsis thaliana chromosome 2, complete sequence
AT5G54160	1.096825352	6.53E-140	Arabidopsis thaliana caffeic acid/5-hydroxyferulic acid O-methyltransferase mRNA, complete cds
AT1G52890	5.303319431	8.83E-140	Arabidopsis thaliana NAC domain-containing protein 19 mRNA, complete cds
AT1G18570	2.09384703	5.31E-139	Arabidopsis thaliana myb domain protein 51 mRNA, complete cds
AT1G27200	2.416551481	9.41E-139	Arabidopsis thaliana chromosome 1 sequence
AT3G06490	9.639334662	2.26E-138	Arabidopsis thaliana putative transcription factor MYB108 mRNA, complete cds
AT5G63130	6.187880681	6.27E-138	Arabidopsis thaliana octicosapeptide/Phox/Bem1p domain-containing protein mRNA, complete cds
AT1G73920	1.057350058	3.84E-137	Arabidopsis thaliana alpha/beta-hydrolase domain-containing protein mRNA, complete

			cds
AT1G26730	3.081084146	6.47E-137	Arabidopsis thaliana phosphate transporter PHO1-7 mRNA, complete cds
AT5G03210	9.619079517	7.51E-137	Arabidopsis thaliana chromosome 5 sequence
AT4G05100	5.362916359	3.02E-136	Arabidopsis thaliana myb domain protein 74 mRNA, complete cds
AT4G32940	1.006965518	9.49E-136	Arabidopsis thaliana vacuolar-processing enzyme gamma mRNA, complete cds
AT2G41410	1.345785968	1.43E-135	Arabidopsis thaliana chromosome 2, complete sequence
AT2G35060	1.508123156	5.53E-135	Arabidopsis thaliana potassium transporter 11 mRNA, complete cds
AT5G56980	1.615845714	1.44E-134	Arabidopsis thaliana chromosome 5 sequence
AT1G44350	3.933748531	4.79E-134	Arabidopsis thaliana IAA-amino acid hydrolase ILR1-like 6 mRNA, complete cds
AT4G36040	1.376277704	3.49E-133	Arabidopsis thaliana chromosome 4 sequence
AT1G27770	1.188911861	1.11E-132	Arabidopsis thaliana autoinhibited Ca2+-ATPase 1 mRNA, complete cds
AT3G21150	2.420709651	2.25E-132	Arabidopsis thaliana chromosome 3, complete sequence
AT3G59220	3.319233921	2.99E-132	Arabidopsis thaliana pirin-1 mRNA, complete cds
AT5G24110	3.663741609	3.82E-131	Arabidopsis thaliana WRKY DNA-binding protein 30 mRNA, complete cds
AT1G58360	1.256625429	1.19E-130	Arabidopsis thaliana amino acid permease 1 mRNA, complete cds
AT1G21110	2.391601658	5.05E-130	Arabidopsis thaliana O-methyltransferase family protein mRNA, complete cds
AT3G59060	1.103902472	8.84E-130	Arabidopsis thaliana transcription factor PIF5 mRNA, complete cds
AT2G39050	2.687456242	1.15E-129	Arabidopsis thaliana Euonymus lectin S3 mRNA, complete cds
AT4G29950	1.293103116	6.14E-129	Arabidopsis thaliana RabGAP/TBC domain-containing protein mRNA, complete cds
AT2G33310	1.537768381	1.70E-128	Arabidopsis thaliana auxin-responsive protein IAA13 mRNA, complete cds
AT5G62570	1.571286636	1.99E-127	Arabidopsis thaliana calmodulin binding protein 60a mRNA, complete cds
AT1G13990	2.400960063	2.58E-127	Arabidopsis thaliana uncharacterized protein mRNA, complete cds
AT4G28300	1.091522998	2.14E-126	Arabidopsis thaliana uncharacterized protein mRNA, complete cds

AT3G46450	1.602215667	3.80E-126	Arabidopsis thaliana SEC14 cytosolic factor family protein / phosphoglyceride transfer family protein mRNA, complete cds
AT3G14810	1.850692516	9.79E-126	Arabidopsis thaliana mechanosensitive channel of small conductance-like 5 mRNA, complete cds
AT5G62470	1.562538449	1.93E-125	Arabidopsis thaliana Myb transcription factor mRNA, complete cds
AT3G59080	2.417551616	7.18E-125	Arabidopsis thaliana chromosome 3, complete sequence
AT1G73500	2.774445073	1.95E-124	Arabidopsis thaliana chromosome 1 sequence
AT4G14370	3.132984049	2.18E-124	Arabidopsis thaliana TIR-NBS-LRR class disease resistance protein mRNA, complete cds
AT2G29350	3.869789462	5.22E-124	Arabidopsis thaliana senescence-associated protein 13 mRNA, complete cds
AT1G02660	2.644592273	8.43E-124	Arabidopsis thaliana alpha/beta-Hydrolases superfamily protein mRNA, complete cds
AT3G47500	1.481857318	1.07E-123	Arabidopsis thaliana cyclic DOF factor 3 mRNA, complete cds
AT5G54080	1.822808094	1.77E-123	Arabidopsis thaliana homogentisate 1,2-dioxygenase mRNA, complete cds
AT5G19875	2.267410051	2.30E-123	Arabidopsis thaliana chromosome 5 sequence
AT2G43330	1.446984875	4.13E-123	Arabidopsis thaliana inositol transporter 1 mRNA, complete cds
AT4G05010	2.972616975	5.98E-123	Arabidopsis thaliana F-box protein mRNA, complete cds
AT3G12320	1.483161894	8.10E-123	Arabidopsis thaliana uncharacterized protein mRNA, complete cds
AT5G52760	3.775190074	1.36E-122	Arabidopsis thaliana copper transport family protein mRNA, complete cds
AT3G03341	9.416332609	1.86E-122	Arabidopsis thaliana chromosome 3, complete sequence
AT2G22010	1.291441156	2.68E-122	Arabidopsis thaliana E3 ubiquitin-protein ligase RKP mRNA, complete cds
AT2G31880	1.151835285	2.87E-122	Arabidopsis thaliana chromosome 2, complete sequence
AT4G12000	2.332769558	2.42E-121	Arabidopsis thaliana SNARE associated Golgi family protein mRNA, complete cds
AT1G45145	2.07836738	4.92E-121	Arabidopsis thaliana thioredoxin H5 mRNA, complete cds
AT5G57040	1.319563521	8.11E-121	Arabidopsis thaliana Lactoylglutathione lyase / glyoxalase I family protein mRNA, complete cds

AT1G16850	4.383791732	1.19E-120	Arabidopsis thaliana uncharacterized protein mRNA, complete cds
AT1G21120	2.006472383	2.81E-120	Arabidopsis thaliana O-methyltransferase family protein mRNA, complete cds
AT2G28470	1.12695156	3.65E-119	Arabidopsis thaliana beta-galactosidase 8 mRNA, complete cds
AT2G36220	2.620315231	8.40E-119	Arabidopsis thaliana chromosome 2, complete sequence
AT5G25930	2.052151115	8.78E-119	Arabidopsis thaliana protein kinase family protein with leucine-rich repeat domain mRNA, complete cds
AT1G09930	6.387592713	2.04E-118	Arabidopsis thaliana oligopeptide transporter 2 mRNA, complete cds
AT1G80110	4.455630234	5.40E-118	Arabidopsis thaliana phloem protein 2-B11 mRNA, complete cds
AT4G33920	1.757575201	1.97E-117	Arabidopsis thaliana putative protein phosphatase 2C 63 mRNA, complete cds
AT1G53580	1.576971949	2.02E-117	Arabidopsis thaliana persulfide dioxygenase ETHE1-like protein mRNA, complete cds
AT3G28220	1.873887726	5.96E-117	Arabidopsis thaliana TRAF-like family protein mRNA, complete cds
AT4G32770	1.889107439	6.05E-117	Arabidopsis thaliana tocopherol cyclase mRNA, complete cds
AT3G51000	1.86056757	1.63E-116	Arabidopsis thaliana alpha/beta-Hydrolases superfamily protein mRNA, complete cds
AT1G78100	1.616324257	2.33E-116	Arabidopsis thaliana chromosome 1 sequence
AT5G43620	4.736192452	2.37E-116	Arabidopsis thaliana chromosome 5 sequence
AT2G26150	1.387795028	7.74E-116	Arabidopsis thaliana heat stress transcription factor A-2 mRNA, complete cds
AT1G74450	2.439592879	7.87E-116	Arabidopsis thaliana chromosome 1 sequence
AT4G01070	1.038796619	2.33E-115	Arabidopsis thaliana chromosome 4 sequence
AT2G29460	4.870504837	2.35E-115	Arabidopsis thaliana glutathione S-transferase tau 4 mRNA, complete cds
AT4G14690	3.75756207	2.42E-115	Arabidopsis thaliana early light-inducible protein 2 mRNA, complete cds
AT4G14368	5.594391886	4.64E-115	Arabidopsis thaliana regulator of chromosome condensation repeat-containing protein mRNA, complete cds
AT1G70130	8.097602214	8.42E-115	Arabidopsis thaliana putative L-type lectin-domain containing receptor kinase V.2 mRNA, complete cds
AT2G39450	1.327319812	1.05E-114	Arabidopsis thaliana manganese transporter MTP11 mRNA, complete cds

AT2G30140	2.362472248	1.75E-114	Arabidopsis thaliana UDP-glycosyltransferase 87A2 mRNA, complete cds
AT4G27560	2.121247836	3.42E-114	Arabidopsis thaliana chromosome 4 sequence
AT3G51895	1.418862669	6.79E-114	Arabidopsis thaliana sulfate transporter 3;1 mRNA, complete cds
AT5G44110	2.875505518	9.33E-114	Arabidopsis thaliana ABC transporter I family member 21 mRNA, complete cds
AT4G27450	1.413700556	9.89E-114	Arabidopsis thaliana aluminum induced protein with YGL and LRDR motifs mRNA, complete cds
AT3G61630	2.529507297	4.57E-113	Arabidopsis thaliana chromosome 3, complete sequence
AT5G39670	2.775372752	4.94E-113	Arabidopsis thaliana chromosome 5 sequence
AT4G37260	1.650666523	6.55E-113	Arabidopsis thaliana chromosome 4 sequence
AT1G21980	1.317213832	9.45E-113	Arabidopsis thaliana phosphatidylinositol-4-phosphate 5-kinase 1 mRNA, complete cds
AT2G27830	2.35772513	1.54E-112	Arabidopsis thaliana chromosome 2, complete sequence
AT2G25625	4.624262415	2.21E-112	Arabidopsis thaliana uncharacterized protein mRNA, complete cds
AT5G64660	2.860943223	4.53E-112	Arabidopsis thaliana chromosome 5 sequence
AT3G52400	1.590382983	1.00E-111	Arabidopsis thaliana syntaxin-122 mRNA, complete cds
AT5G65990	1.548121166	1.06E-111	Arabidopsis thaliana transmembrane amino acid transporter family protein mRNA, complete cds
AT1G59740	1.815515602	2.84E-111	Arabidopsis thaliana probable peptide/nitrate transporter mRNA, complete cds
AT5G08130	1.109183224	3.89E-111	Arabidopsis thaliana transcription factor BIM1 mRNA, complete cds
AT4G30490	1.212997702	4.21E-111	Arabidopsis thaliana AFG1-like ATPase family protein mRNA, complete cds
AT3G45650	1.935066619	7.71E-111	Arabidopsis thaliana nitrate excretion transporter1 mRNA, complete cds
AT2G17040	2.188517881	2.03E-110	Arabidopsis thaliana NAC transcription factor family protein NAC036 mRNA, complete cds
AT1G71697	1.862425438	1.35E-109	Arabidopsis thaliana choline kinase 1 mRNA, complete cds
AT5G37770	1.683272606	3.83E-109	Arabidopsis thaliana chromosome 5 sequence
AT3G46620	2.199877744	9.62E-109	Arabidopsis thaliana chromosome 3, complete sequence

AT2G36650	3.564152129	1.03E-108	Arabidopsis thaliana uncharacterized protein mRNA, complete cds
AT2G02010	3.635506338	1.52E-108	Arabidopsis thaliana glutamate decarboxylase 4 mRNA, complete cds
AT2G37430	4.456888362	1.97E-108	Arabidopsis thaliana chromosome 2, complete sequence
AT5G41740	1.784172948	3.11E-108	Arabidopsis thaliana TIR-NBS-LRR class disease resistance protein mRNA, complete cds
AT2G27420	2.267151344	4.41E-108	Arabidopsis thaliana cysteine proteinase-like protein mRNA, complete cds
AT1G32970	4.637890379	5.59E-108	Arabidopsis thaliana Subtilisin-like serine endopeptidase family protein mRNA, complete cds
AT5G48900	1.863188981	1.20E-107	Arabidopsis thaliana putative pectate lyase 20 mRNA, complete cds
AT2G22680	2.015453172	2.84E-107	Arabidopsis thaliana chromosome 2, complete sequence
AT2G37970	1.610087408	5.45E-107	Arabidopsis thaliana chromosome 2, complete sequence
AT5G57340	2.208567801	1.22E-106	Arabidopsis thaliana uncharacterized protein mRNA, complete cds
AT2G35980	5.231390572	1.31E-106	Arabidopsis thaliana chromosome 2, complete sequence
AT4G33905	5.630163017	2.43E-106	Arabidopsis thaliana Peroxisomal membrane protein Mpv17/PMP22 mRNA, complete cds
AT5G57900	1.831910302	5.46E-106	Arabidopsis thaliana F-box protein SKIP1 mRNA, complete cds
AT5G10930	1.820880646	5.73E-106	Arabidopsis thaliana chromosome 5 sequence
AT2G29650	1.202346107	7.82E-106	Arabidopsis thaliana phosphate transporter 4;1 mRNA, complete cds
AT5G18130	2.142145177	1.02E-105	Arabidopsis thaliana chromosome 5 sequence
AT1G68620	3.896657124	1.53E-105	Arabidopsis thaliana chromosome 1 sequence
AT1G02520	2.036603007	1.76E-105	Arabidopsis thaliana P-glycoprotein 11 mRNA, complete cds
AT2G35940	1.04061222	3.02E-105	Arabidopsis thaliana BEL1-like homeodomain 1 mRNA, complete cds
AT1G61820	1.729245653	5.27E-105	Arabidopsis thaliana beta glucosidase 46 mRNA, complete cds
AT3G14050	1.598252712	8.51E-105	Arabidopsis thaliana RelA-SpoT like protein RSH2 mRNA, complete cds
AT5G02810	2.084280297	2.77E-104	Arabidopsis thaliana pseudo-response regulator 7 mRNA, complete cds

AT2G42430	5.600415673	3.50E-104	Arabidopsis thaliana LOB domain-containing protein 16 mRNA, complete cds
AT4G33120	2.139188541	2.50E-102	Arabidopsis thaliana S-adenosyl-L-methionine-dependent methyltransferases superfamily protein mRNA, complete cds
AT1G28370	5.424844109	3.08E-102	Arabidopsis thaliana chromosome 1 sequence
AT1G22810	6.777004405	5.57E-102	Arabidopsis thaliana chromosome 1 sequence
AT4G21390	2.789539248	5.91E-102	Arabidopsis thaliana G-type lectin S-receptor-like serine/threonine-protein kinase B120 mRNA, complete cds
AT4G09030	1.451324175	1.45E-101	Arabidopsis thaliana chromosome 4 sequence
AT1G70580	1.033773356	1.67E-101	Arabidopsis thaliana glutamate--glyoxylate aminotransferase 2 mRNA, complete cds
AT1G21410	2.032674913	1.83E-101	Arabidopsis thaliana F-box protein SKP2A mRNA, complete cds
AT1G10480	2.867637876	2.72E-101	Arabidopsis thaliana chromosome 1 sequence
AT5G62090	1.232285094	7.86E-101	Arabidopsis thaliana protein SEUSS-like 2 mRNA, complete cds
AT1G02400	2.850959135	1.04E-100	Arabidopsis thaliana gibberellin 2-oxidase 6 mRNA, complete cds
AT4G35790	1.11835308	1.85E-100	Arabidopsis thaliana phospholipase D delta mRNA, complete cds
AT2G39330	2.594074146	5.82E-100	Arabidopsis thaliana jacalin-related lectin 23 mRNA, complete cds
AT5G24780	2.563262675	5.94E-100	Arabidopsis thaliana acid phosphatase VSP1 mRNA, complete cds
AT5G02260	1.439951001	8.44E-100	Arabidopsis thaliana expansin A9 mRNA, complete cds
AT5G63800	1.178407324	1.31E-99	Arabidopsis thaliana beta-galactosidase 6 mRNA, complete cds
AT5G01380	5.535526142	1.32E-99	Arabidopsis thaliana trihelix transcription factor GT-3a mRNA, complete cds
AT2G02710	1.375806028	1.84E-99	Arabidopsis thaliana PAS/LOV protein B mRNA, complete cds
AT4G21650	1.202173416	2.68E-99	Arabidopsis thaliana Subtilase family protein mRNA, complete cds
AT5G40800	5.246019525	6.49E-99	Arabidopsis thaliana chromosome 5 sequence
AT5G40000	6.726259606	9.05E-99	Arabidopsis thaliana chromosome 5 sequence
AT5G47230	2.851614943	1.67E-98	Arabidopsis thaliana chromosome 5 sequence
AT1G07180	1.186848062	2.56E-98	Arabidopsis thaliana internal alternative NAD(P)H-ubiquinone oxidoreductase A1 mRNA, complete cds

AT2G20960	1.162955613	3.06E-98	Arabidopsis thaliana phospholipase-like protein (PEARLI 4) domain-containing protein mRNA, complete cds
AT5G52750	2.364974637	1.17E-97	Arabidopsis thaliana heavy metal transport/detoxification domain-containing protein mRNA, complete cds
AT2G05540	2.005930398	1.50E-97	Arabidopsis thaliana glycine-rich protein mRNA, complete cds
AT1G07900	3.947766365	1.92E-97	Arabidopsis thaliana LOB domain-containing protein 1 mRNA, complete cds
AT4G11220	1.331589987	7.06E-97	Arabidopsis thaliana reticulon-like protein B2 mRNA, complete cds
AT4G38810	1.19216734	1.11E-96	Arabidopsis thaliana SnRK2-interacting calcium sensor SCS mRNA, complete cds
AT4G17615	2.455437029	1.17E-96	Arabidopsis thaliana calcineurin B-like protein 1 mRNA, complete cds
AT4G24380	3.860943223	1.72E-96	Arabidopsis thaliana uncharacterized protein mRNA, complete cds
AT1G42430	1.750183762	2.64E-96	Arabidopsis thaliana uncharacterized protein mRNA, complete cds
AT1G77680	1.196633637	2.66E-96	Arabidopsis thaliana ribonuclease II/R family protein mRNA, complete cds
AT2G32140	3.633932799	2.68E-96	Arabidopsis thaliana transmembrane receptor protein mRNA, complete cds
AT1G53170	2.064882882	2.85E-96	Arabidopsis thaliana chromosome 1 sequence
AT3G20830	2.492898845	3.03E-96	Arabidopsis thaliana chromosome 3, complete sequence
AT2G36895	1.117835077	1.17E-95	Arabidopsis thaliana uncharacterized protein mRNA, complete cds
AT1G66760	2.725306921	2.67E-95	Arabidopsis thaliana MATE efflux family protein mRNA, complete cds
AT1G68020	1.393574667	4.40E-95	Arabidopsis thaliana alpha,alpha-trehalose-phosphate synthase [UDP-forming] 6 mRNA, complete cds
AT4G15248	4.855556677	2.73E-94	Arabidopsis thaliana chromosome 4 sequence
AT3G55880	2.300610389	3.48E-94	Arabidopsis thaliana protein SULPHATE UTILIZATION EFFICIENCY 4 mRNA, complete cds
AT4G09500	3.603495066	9.47E-94	Arabidopsis thaliana chromosome 4 sequence
AT4G24510	1.544942337	9.59E-94	Arabidopsis thaliana fatty acid elongation machinery component ECERIFERUM2 mRNA, complete cds
AT5G54490	5.437985208	4.64E-93	Arabidopsis thaliana chromosome 5 sequence
AT2G41010	2.044914635	6.20E-93	Arabidopsis thaliana chromosome 2,

			complete sequence
AT2G30040	2.413045537	8.85E-93	Arabidopsis thaliana chromosome 2, complete sequence
AT4G16680	1.784447621	2.70E-92	Arabidopsis thaliana putative RNA helicase mRNA, complete cds
AT4G32920	1.387186887	3.02E-92	Arabidopsis thaliana glycine-rich protein mRNA, complete cds
AT1G73080	1.351423384	6.65E-92	Arabidopsis thaliana leucine-rich repeat receptor-like protein kinase PEPR1 mRNA, complete cds
AT2G27150	1.675324643	1.14E-91	Arabidopsis thaliana abscisic-aldehyde oxidase mRNA, complete cds
AT4G39730	1.408672561	1.48E-91	Arabidopsis thaliana Lipase/lipooxygenase, PLAT/LH2 family protein mRNA, complete cds
AT3G29000	4.039527307	2.11E-91	Arabidopsis thaliana chromosome 3, complete sequence
AT1G56650	3.493500469	2.16E-91	Arabidopsis thaliana transcription factor MYB75 mRNA, complete cds
AT1G21590	1.192609743	5.97E-91	Arabidopsis thaliana putative protein kinase mRNA, complete cds
AT4G13010	1.154489847	2.33E-90	Arabidopsis thaliana putative quinone-oxidoreductase-like protein mRNA, complete cds
AT2G23320	1.108036578	2.46E-90	Arabidopsis thaliana WRKY DNA-binding protein 15 mRNA, complete cds
AT5G10625	3.270805793	4.42E-90	Arabidopsis thaliana chromosome 5 sequence
AT3G24310	10.10026156	8.15E-90	Arabidopsis thaliana myb domain protein 305 mRNA, complete cds
AT2G41640	1.95938711	9.83E-90	Arabidopsis thaliana Glycosyltransferase family 61 protein mRNA, complete cds
AT3G45060	2.264392748	1.33E-89	Arabidopsis thaliana high affinity nitrate transporter 2.6 mRNA, complete cds
AT5G52050	3.049400504	1.37E-89	Arabidopsis thaliana chromosome 5 sequence
AT3G15350	1.847267825	4.07E-89	Arabidopsis thaliana core-2/I-branching beta-1,6-N-acetylglucosaminyltransferase family protein mRNA, complete cds
AT2G47190	4.877255879	4.10E-89	Arabidopsis thaliana mRNA for MYB transcription factor, complete cds, clone: RAFL16-42-L19
AT1G11210	4.153669314	5.44E-89	Arabidopsis thaliana uncharacterized protein mRNA, complete cds
AT1G05170	1.613460021	7.63E-89	Arabidopsis thaliana putative beta-1,3-galactosyltransferase 2 mRNA,

			complete cds
AT2G05940	1.564670012	1.99E-88	Arabidopsis thaliana RPM1-induced protein kinase mRNA, complete cds
AT5G26340	2.828083037	2.19E-88	Arabidopsis thaliana sugar transport protein 13 mRNA, complete cds
AT1G69252	1.285761216	2.98E-88	Arabidopsis thaliana chromosome 1 sequence
AT5G54585	2.779434685	2.99E-88	Arabidopsis thaliana uncharacterized protein mRNA, complete cds
AT3G54680	1.696923654	1.12E-87	Arabidopsis thaliana proteophosphoglycan-related protein mRNA, complete cds
AT1G75170	1.702087091	1.54E-87	Arabidopsis thaliana Sec14p-like phosphatidylinositol transfer family protein mRNA, complete cds
AT2G46800	1.060321941	1.81E-87	Arabidopsis thaliana zinc transporter ZAT mRNA, complete cds
AT1G10170	1.164596612	2.90E-87	Arabidopsis thaliana chromosome 1 sequence
AT5G56970	4.237845594	4.01E-87	Arabidopsis thaliana cytokinin dehydrogenase 3 mRNA, complete cds
AT5G64750	3.574184132	9.20E-87	Arabidopsis thaliana ethylene-responsive transcription factor ABR1 mRNA, complete cds
AT3G57010	2.263904255	1.25E-86	Arabidopsis thaliana calcium-dependent phosphotriesterase superfamily protein mRNA, complete cds
AT5G65470	1.210196537	1.66E-86	Arabidopsis thaliana O-fucosyltransferase family protein mRNA, complete cds
AT2G47000	1.217087034	1.67E-86	Arabidopsis thaliana auxin efflux transmembrane transporter MDR4 mRNA, complete cds
AT2G46500	1.066482825	2.45E-86	Arabidopsis thaliana phosphoinositide 4-kinase gamma 4 mRNA, complete cds
AT3G11840	2.96541249	2.79E-86	Arabidopsis thaliana E3 ubiquitin-protein ligase PUB24 mRNA, complete cds
AT1G18460	1.05567765	3.64E-86	Arabidopsis thaliana alpha/beta-Hydrolases superfamily protein mRNA, complete cds
AT4G27830	1.582252006	5.76E-86	Arabidopsis thaliana beta glucosidase 10 mRNA, complete cds
AT5G24660	3.107375662	5.91E-86	Arabidopsis thaliana chromosome 5 sequence
AT1G77120	1.850790507	7.80E-86	Arabidopsis thaliana alcohol dehydrogenase 1 mRNA, complete cds
AT5G58620	1.766313208	2.02E-85	Arabidopsis thaliana zinc finger CCCH domain-containing protein 66 mRNA, complete cds

AT5G15410	1.16118189	2.67E-85	Arabidopsis thaliana cyclic nucleotide-gated ion channel 2 mRNA, complete cds
AT1G01650	1.323815865	3.91E-85	Arabidopsis thaliana signal peptide peptidase-like 4 mRNA, complete cds
AT5G55120	1.012810873	9.21E-85	Arabidopsis thaliana GDP-L-galactose phosphorylase mRNA, complete cds
AT2G31865	2.195749858	1.41E-84	Arabidopsis thaliana poly(ADP-ribose) glycohydrolase 2 mRNA, complete cds
AT1G18300	2.602944499	1.96E-84	Arabidopsis thaliana nudix hydrolase 4 mRNA, complete cds
AT3G27025	3.603846579	2.34E-84	Arabidopsis thaliana uncharacterized protein mRNA, complete cds
AT3G10960	1.794426293	2.89E-84	Arabidopsis thaliana chromosome 3, complete sequence
AT4G24000	4.703953277	3.57E-84	Arabidopsis thaliana cellulose synthase-like protein G2 mRNA, complete cds
AT2G43010	1.876407484	4.98E-84	Arabidopsis thaliana transcription factor PIF4 mRNA, complete cds
AT5G52400	9.975455104	5.02E-84	Arabidopsis thaliana cytochrome P450, family 715, subfamily A, polypeptide 1 mRNA, complete cds
AT1G54130	1.11622283	1.08E-83	Arabidopsis thaliana RELA/SPOT homolog 3 mRNA, complete cds
AT2G39350	2.01733046	1.09E-83	Arabidopsis thaliana chromosome 2, complete sequence
AT4G34650	2.874854842	2.05E-83	Arabidopsis thaliana squalene synthase 2 mRNA, complete cds
AT1G30730	1.829441187	2.64E-83	Arabidopsis thaliana chromosome 1 sequence
AT1G05870	1.406066289	6.11E-83	Arabidopsis thaliana uncharacterized protein mRNA, complete cds
AT3G49570	5.621726179	1.17E-82	Arabidopsis thaliana chromosome 3, complete sequence
AT4G29930	3.101372199	1.59E-82	Arabidopsis thaliana transcription factor bHLH27 mRNA, complete cds
AT5G28510	5.617489187	2.04E-82	Arabidopsis thaliana beta glucosidase 24 mRNA, complete cds
AT4G01120	1.389473913	2.25E-82	Arabidopsis thaliana basic leucine zipper transcription factor mRNA, complete cds
AT5G27420	2.16862681	8.62E-82	Arabidopsis thaliana chromosome 5 sequence
AT5G47240	3.298387136	8.74E-82	Arabidopsis thaliana nudix hydrolase 8 mRNA, complete cds
AT1G12420	1.65912498	1.47E-81	Arabidopsis thaliana ACT domain repeat 8 protein mRNA, complete cds

AT1G53163	4.753805307	2.77E-81	Arabidopsis thaliana chromosome 1 sequence
AT5G62430	1.326261155	3.64E-81	Arabidopsis thaliana cycling DOF factor 1 mRNA, complete cds
AT5G38200	3.266678276	4.02E-81	Arabidopsis thaliana class I glutamine amidotransferase-like protein mRNA, complete cds
AT5G14700	2.255042991	4.38E-81	Arabidopsis thaliana Rossmann-fold NAD(P)-binding domain-containing protein mRNA, complete cds
AT3G56200	2.214034632	5.33E-81	Arabidopsis thaliana putative amino acid transporter mRNA, complete cds
AT5G54470	4.09129693	5.42E-81	Arabidopsis thaliana B-box type zinc finger-containing protein mRNA, complete cds
AT3G47080	1.706260761	5.71E-81	Arabidopsis thaliana tetratricopeptide repeat domain-containing protein mRNA, complete cds
AT1G67310	1.091274607	1.14E-80	Arabidopsis thaliana calmodulin-binding transcription activator 4 mRNA, complete cds
AT3G28600	5.578785657	2.97E-80	Arabidopsis thaliana chromosome 3, complete sequence
AT5G22460	3.271792926	8.83E-80	Arabidopsis thaliana esterase/lipase/thioesterase family protein mRNA, complete cds
AT3G09350	1.308500147	1.05E-79	Arabidopsis thaliana protein Fes1A mRNA, complete cds
AT2G27080	2.132343412	1.16E-79	Arabidopsis thaliana chromosome 2, complete sequence
AT3G22060	1.594713314	1.29E-79	Arabidopsis thaliana Receptor-like protein kinase-related family protein mRNA, complete cds
AT2G43290	1.268349131	3.45E-79	Arabidopsis thaliana chromosome 2, complete sequence
AT1G04240	1.389500063	8.44E-79	Arabidopsis thaliana auxin-responsive protein IAA3 mRNA, complete cds
AT2G24550	1.058220102	9.64E-79	Arabidopsis thaliana uncharacterized protein mRNA, complete cds
AT5G12010	1.65853972	3.52E-78	Arabidopsis thaliana chromosome 5 sequence
AT4G28140	4.102916014	3.92E-78	Arabidopsis thaliana chromosome 4 sequence
AT1G09500	3.226600837	4.01E-78	Arabidopsis thaliana alcohol dehydrogenase-like protein mRNA, complete cds
AT1G28480	5.539015128	4.41E-78	Arabidopsis thaliana chromosome 1 sequence
AT2G21560	1.40415028	5.41E-78	Arabidopsis thaliana chromosome 2,

			complete sequence
AT3G02990	2.320591609	1.16E-77	Arabidopsis thaliana heat stress transcription factor A-1e mRNA, complete cds
AT3G43270	1.965233817	2.46E-77	Arabidopsis thaliana probable pectinesterase/pectinesterase inhibitor 32 mRNA, complete cds
AT5G65280	2.277066904	2.56E-77	Arabidopsis thaliana protein GCR2-like 1 mRNA, complete cds
AT3G21070	1.365716614	2.71E-77	Arabidopsis thaliana NAD(H) kinase 1 mRNA, complete cds
AT3G19390	1.412548501	2.88E-77	Arabidopsis thaliana Granulin repeat cysteine protease family protein mRNA, complete cds
AT2G41630	1.145681544	3.63E-77	Arabidopsis thaliana transcription initiation factor IIB-1 mRNA, complete cds
AT1G22770	2.320450871	5.04E-77	Arabidopsis thaliana protein GIGANTEA mRNA, complete cds
AT2G24570	1.149184003	1.07E-76	Arabidopsis thaliana putative WRKY transcription factor 17 mRNA, complete cds
AT1G69840	1.10312658	1.09E-76	Arabidopsis thaliana Hypersensitive-induced response protein 2 mRNA, complete cds
AT5G65140	1.920418891	1.48E-76	Arabidopsis thaliana probable trehalose-phosphate phosphatase J mRNA, complete cds
AT3G07350	3.005759776	1.71E-76	Arabidopsis thaliana chromosome 3, complete sequence
AT5G06870	1.97973125	2.08E-76	Arabidopsis thaliana polygalacturonase inhibitor 2 mRNA, complete cds
AT4G35770	3.456552968	4.89E-76	Arabidopsis thaliana senescence-associated protein DIN1 mRNA, complete cds
AT4G36010	2.867202214	7.82E-76	Arabidopsis thaliana pathogenesis-related thaumatin family protein mRNA, complete cds
AT3G55430	1.068160223	8.06E-76	Arabidopsis thaliana O-Glycosyl hydrolases family 17 protein mRNA, complete cds
AT5G64905	4.659309362	1.06E-75	Arabidopsis thaliana chromosome 5 sequence
AT4G34240	1.00882685	1.17E-75	Arabidopsis thaliana aldehyde dehydrogenase 3I1 mRNA, complete cds
AT4G33550	3.951322835	2.26E-75	Arabidopsis thaliana chromosome 4 sequence
AT2G21900	5.969649483	2.80E-75	Arabidopsis thaliana putative WRKY transcription factor 59 mRNA, complete cds
AT1G22640	2.412343325	3.06E-75	Arabidopsis thaliana transcription factor MYB3 mRNA, complete cds
AT4G37730	2.617693665	3.36E-75	Arabidopsis thaliana chromosome 4 sequence

AT1G63010	1.111758559	3.68E-75	Arabidopsis thaliana Major Facilitator Superfamily with SPX domain-containing protein mRNA, complete cds
AT4G32480	2.003033913	4.98E-75	Arabidopsis thaliana uncharacterized protein mRNA, complete cds
AT5G10100	3.409626152	7.61E-75	Arabidopsis thaliana probable trehalose-phosphate phosphatase I mRNA, complete cds
AT3G62590	2.83857541	9.06E-75	Arabidopsis thaliana alpha/beta-Hydrolases superfamily protein mRNA, complete cds
AT1G73870	1.520091367	1.41E-74	Arabidopsis thaliana zinc finger protein CONSTANS-LIKE 7 mRNA, complete cds
AT4G11350	3.707944137	2.70E-74	Arabidopsis thaliana uncharacterized protein mRNA, complete cds
AT3G47780	2.564031523	2.95E-74	Arabidopsis thaliana ABC transporter A family member 7 mRNA, complete cds
AT5G62480	2.816549104	3.42E-74	Arabidopsis thaliana glutathione S-transferase tau 9 mRNA, complete cds
AT5G43890	5.277548018	4.32E-74	Arabidopsis thaliana chromosome 5 sequence
AT5G53450	1.16820303	7.55E-74	Arabidopsis thaliana OBP3-responsive protein 1 mRNA, complete cds
AT1G11050	1.77416835	1.13E-73	Arabidopsis thaliana chromosome 1 sequence
AT5G43450	2.04176445	1.25E-73	Arabidopsis thaliana 1-aminocyclopropane-1-carboxylate oxidase-like protein mRNA, complete cds
AT5G20380	1.186118967	1.49E-73	Arabidopsis thaliana phosphate transporter 4;5 mRNA, complete cds
AT5G05140	1.313230368	1.83E-73	Arabidopsis thaliana transcription elongation factor (TFIIS) family protein mRNA, complete cds
AT5G47550	2.414490193	2.52E-73	Arabidopsis thaliana chromosome 5 sequence
AT3G09830	1.531702923	2.72E-73	Arabidopsis thaliana protein kinase family protein mRNA, complete cds
AT1G14040	1.528980535	3.54E-73	Arabidopsis thaliana phosphate transporter PHO1-3 mRNA, complete cds
AT1G14480	2.671407505	4.10E-73	Arabidopsis thaliana Ankyrin repeat family protein mRNA, complete cds
AT3G53230	1.528767632	6.04E-73	Arabidopsis thaliana cell division control protein 48-B mRNA, complete cds
AT5G49520	2.011153035	7.14E-73	Arabidopsis thaliana putative WRKY transcription factor 48 mRNA, complete cds
AT4G36880	3.683831681	9.24E-73	Arabidopsis thaliana cysteine proteinase1 mRNA, complete cds
AT5G12840	1.416517729	1.12E-72	Arabidopsis thaliana nuclear transcription

			factor Y subunit A-1 mRNA, complete cds
AT2G29670	1.361903586	2.76E-72	Arabidopsis thaliana tetratricopeptide repeat-containing protein mRNA, complete cds
AT3G48520	4.936699061	4.63E-72	Arabidopsis thaliana chromosome 3, complete sequence
AT2G45760	4.808686986	6.00E-72	Arabidopsis thaliana chromosome 2, complete sequence
AT4G37470	1.589372574	1.18E-71	Arabidopsis thaliana probable esterase KAI2 mRNA, complete cds
AT5G45100	1.297063812	1.35E-71	Arabidopsis thaliana chromosome 5 sequence
AT5G17350	3.223048592	2.97E-71	Arabidopsis thaliana chromosome 5 sequence
AT4G19230	2.748169294	3.78E-71	Arabidopsis thaliana abscisic acid 8'-hydroxylase 1 mRNA, complete cds
AT1G53180	2.028915432	4.08E-71	Arabidopsis thaliana uncharacterized protein mRNA, complete cds
AT1G63720	3.005011426	4.14E-71	Arabidopsis thaliana uncharacterized protein mRNA, complete cds
AT1G07040	1.790092519	4.96E-71	Arabidopsis thaliana uncharacterized protein mRNA, complete cds
AT1G65890	3.691018222	1.15E-70	Arabidopsis thaliana acyl activating enzyme 12 mRNA, complete cds
AT3G24840	1.826728464	1.47E-70	Arabidopsis thaliana Sec14p-like phosphatidylinositol transfer family protein mRNA, complete cds
AT1G64950	1.351357286	1.72E-70	Arabidopsis thaliana chromosome 1 sequence
AT4G13800	3.922343768	1.86E-70	Arabidopsis thaliana uncharacterized protein mRNA, complete cds
AT5G26220	4.093955146	2.35E-70	Arabidopsis thaliana ChaC-like family protein mRNA, complete cds
AT5G62220	1.293526978	3.55E-70	Arabidopsis thaliana chromosome 5 sequence
AT3G51450	2.418355346	3.94E-70	Arabidopsis thaliana strictosidine synthase family protein mRNA, complete cds
AT2G22860	3.528862412	3.99E-70	Arabidopsis thaliana phytosulfokine-beta mRNA, complete cds
AT1G71030	3.72440439	4.16E-70	Arabidopsis thaliana putative myb family transcription factor mRNA, complete cds
AT3G16940	1.00671676	5.49E-70	Arabidopsis thaliana calmodulin-binding transcription activator mRNA, complete cds
AT4G16780	1.185900227	1.17E-69	Arabidopsis thaliana homeobox protein 2 mRNA, complete cds
AT3G56260	2.963489751	1.60E-69	Arabidopsis thaliana uncharacterized protein mRNA, complete cds

AT4G14365	1.234866357	2.54E-69	Arabidopsis thaliana putative E3 ubiquitin-protein ligase XBAT34 mRNA, complete cds
AT3G54200	1.449701276	2.68E-69	Arabidopsis thaliana chromosome 3, complete sequence
AT1G50600	1.103149781	2.89E-69	Arabidopsis thaliana scarecrow-like protein 5 mRNA, complete cds
AT4G27940	1.765821471	3.34E-69	Arabidopsis thaliana manganese tracking factor for mitochondrial SOD2 mRNA, complete cds
AT1G03290	1.331712517	4.40E-69	Arabidopsis thaliana uncharacterized protein mRNA, complete cds
AT5G14730	3.026452171	1.53E-68	Arabidopsis thaliana chromosome 5 sequence
AT4G24390	1.600415673	1.73E-68	Arabidopsis thaliana auxin signaling F-BOX 4 mRNA, complete cds
AT1G68360	3.25361291	3.18E-68	Arabidopsis thaliana chromosome 1 sequence
AT1G01360	1.60210205	3.33E-68	Arabidopsis thaliana regulatory component of ABA receptor 1 mRNA, complete cds
AT1G29340	1.003298972	3.49E-68	Arabidopsis thaliana chromosome 1 sequence
AT1G28960	1.474511084	4.45E-68	Arabidopsis thaliana ppGpp pyrophosphohydrolase mRNA, complete cds
AT4G39640	1.099252399	4.69E-68	Arabidopsis thaliana gamma-glutamyl transpeptidase 1 mRNA, complete cds
AT4G25480	7.215125517	6.44E-68	Arabidopsis thaliana chromosome 4 sequence
AT3G08760	1.396736545	9.03E-68	Arabidopsis thaliana osmotic stress-inducible kinase mRNA, complete cds
AT4G11600	1.153367779	9.53E-68	Arabidopsis thaliana glutathione peroxidase 6 mRNA, complete cds
AT4G40010	3.217087034	1.44E-67	Arabidopsis thaliana SNF1-related protein kinase 2.7 mRNA, complete cds
AT5G46710	2.384686982	8.51E-67	Arabidopsis thaliana PLATZ transcription factor family protein mRNA, complete cds
AT1G03840	2.477931667	9.85E-67	Arabidopsis thaliana zinc finger protein MAGPIE mRNA, complete cds
AT5G07010	3.313133721	1.05E-66	Arabidopsis thaliana chromosome 5 sequence
AT1G56170	1.689046972	2.18E-66	Arabidopsis thaliana nuclear transcription factor Y subunit C-2 mRNA, complete cds
AT3G05030	1.603896242	2.72E-66	Arabidopsis thaliana K+/H+ exchanger mRNA, complete cds
AT1G52565	2.032412	3.21E-66	Arabidopsis thaliana uncharacterized protein mRNA, complete cds
AT1G30700	1.58675692	3.87E-66	Arabidopsis thaliana FAD-binding Berberine family protein mRNA, complete cds

AT2G33580	1.916215299	4.27E-66	Arabidopsis thaliana chromosome 2, complete sequence
AT5G57620	2.676183181	6.02E-66	Arabidopsis thaliana putative transcription factor MYB36 mRNA, complete cds
AT2G37760	1.465293644	1.47E-65	Arabidopsis thaliana aldo-keto reductase family 4 member C8 mRNA, complete cds
AT5G24080	5.759614268	2.10E-65	Arabidopsis thaliana G-type lectin S-receptor-like serine/threonine protein kinase mRNA, complete cds
AT3G05650	1.939149805	4.22E-65	Arabidopsis thaliana chromosome 3, complete sequence
AT5G22290	2.099722089	4.77E-65	Arabidopsis thaliana membrane-tethered transcription factor ANAC089 mRNA, complete cds
AT3G04220	2.988911385	9.54E-65	Arabidopsis thaliana TIR-NBS-LRR class disease resistance protein mRNA, complete cds
AT2G45660	1.082058571	9.87E-65	Arabidopsis thaliana MADS-box protein SOC1 mRNA, complete cds
AT1G71960	1.403378826	1.50E-64	Arabidopsis thaliana ABC transporter G family member 25 mRNA, complete cds
AT1G29690	1.392805306	1.58E-64	Arabidopsis thaliana protein constitutively activated cell death 1 mRNA, complete cds
AT2G16900	1.295746779	1.94E-64	Arabidopsis thaliana phospholipase-like protein (PEARLI 4) family mRNA, complete cds
AT1G68840	1.470545855	2.94E-64	Arabidopsis thaliana AP2-EREBP family, RAVE subfamily protein RAV2 mRNA, complete cds
AT3G15790	1.33806647	3.04E-64	Arabidopsis thaliana methyl-CPG-binding domain-containing protein 11 mRNA, complete cds
AT3G28340	2.797717114	3.61E-64	Arabidopsis thaliana chromosome 3, complete sequence
AT5G13210	2.3584869	3.80E-64	Arabidopsis thaliana chromosome 5 sequence
AT3G59480	3.711261442	3.96E-64	Arabidopsis thaliana probable fructokinase-4 mRNA, complete cds
AT5G54230	3.110975783	4.77E-64	Arabidopsis thaliana putative transcription factor MYB49 mRNA, complete cds
AT5G17050	1.159785253	5.03E-64	Arabidopsis thaliana anthocyanidin 3-O-glucosyltransferase mRNA, complete cds
AT1G22160	1.821728637	6.82E-64	Arabidopsis thaliana uncharacterized protein mRNA, complete cds
AT1G69270	1.718889143	8.39E-64	Arabidopsis thaliana chromosome 1 sequence

AT2G30550	1.356584295	1.23E-63	Arabidopsis thaliana phospholipase A1-Igamma2 mRNA, complete cds
AT3G19030	1.347924762	1.43E-63	Arabidopsis thaliana chromosome 3, complete sequence
AT5G39610	3.5689047	1.60E-63	Arabidopsis thaliana NAC-domain transcription factor mRNA, complete cds
AT4G01026	2.010503956	1.61E-63	Arabidopsis thaliana abscisic acid receptor PYL7 mRNA, complete cds
AT3G57680	3.35043742	1.95E-63	Arabidopsis thaliana peptidase S41 family protein mRNA, complete cds
AT3G14590	1.984062753	2.13E-63	Arabidopsis thaliana protein NTMC2T6.2 mRNA, complete cds
AT2G18210	2.926185834	2.70E-63	Arabidopsis thaliana uncharacterized protein mRNA, complete cds
AT1G01010	2.404644494	8.36E-63	Arabidopsis thaliana NAC domain-containing protein 1 mRNA, complete cds
AT1G07870	1.85356549	1.03E-62	Arabidopsis thaliana putative serine/threonine-protein kinase RLCKVII mRNA, complete cds
AT2G44970	1.090850621	1.62E-62	Arabidopsis thaliana alpha/beta-Hydrolases superfamily protein mRNA, complete cds
AT5G66070	2.16071612	1.71E-62	Arabidopsis thaliana RING/U-box superfamily protein mRNA, complete cds
AT3G20300	2.362255936	1.88E-62	Arabidopsis thaliana uncharacterized protein mRNA, complete cds
AT4G11330	1.898535421	2.35E-62	Arabidopsis thaliana mitogen-activated protein kinase 5 mRNA, complete cds
AT2G44940	1.873146578	2.49E-62	Arabidopsis thaliana chromosome 2, complete sequence
AT1G43910	1.589486113	2.59E-62	Arabidopsis thaliana P-loop containing nucleoside triphosphate hydrolases superfamily protein mRNA, complete cds
AT4G29920	1.806357574	6.86E-62	Arabidopsis thaliana Clp amino terminal domain-containing protein mRNA, complete cds
AT3G57260	2.301610136	7.41E-62	Arabidopsis thaliana beta 1,3-glucanase 2 mRNA, complete cds
AT1G10090	1.332434476	7.74E-62	Arabidopsis thaliana Early-responsive to dehydration stress protein ERD4 mRNA, complete cds
AT1G21140	1.706232518	8.35E-62	Arabidopsis thaliana chromosome 1 sequence
AT3G44190	1.250749929	8.92E-62	Arabidopsis thaliana FAD/NAD(P)-binding oxidoreductase family protein mRNA, complete cds

AT2G38530	1.467100815	1.18E-61	Arabidopsis thaliana non-specific lipid-transfer protein 2 mRNA, complete cds
AT4G22920	1.446157613	1.62E-61	Arabidopsis thaliana protein NON-YELLOWING 1 mRNA, complete cds
AT3G14595	1.185081841	1.71E-61	Arabidopsis thaliana ribosomal protein L18ae family protein mRNA, complete cds
AT5G44670	1.466664284	2.01E-61	Arabidopsis thaliana uncharacterized protein mRNA, complete cds
AT5G13800	1.096252283	2.10E-61	Arabidopsis thaliana pheophytinase mRNA, complete cds
AT3G19200	3.04039953	3.35E-61	Arabidopsis thaliana uncharacterized protein mRNA, complete cds
AT3G19290	1.210566506	3.71E-61	Arabidopsis thaliana ABRE binding factor 4 mRNA, complete cds
AT1G08890	2.331162745	7.22E-61	Arabidopsis thaliana sugar transporter ERD6-like 1 mRNA, complete cds
AT2G24600	1.226092412	7.33E-61	Arabidopsis thaliana Ankyrin repeat family protein mRNA, complete cds
AT5G54170	1.008092467	7.98E-61	Arabidopsis thaliana lipid-binding START domain-containing protein mRNA, complete cds
AT3G10930	4.190711618	1.88E-60	Arabidopsis thaliana chromosome 3, complete sequence
AT1G62710	2.28590505	4.06E-60	Arabidopsis thaliana vacuolar-processing enzyme beta mRNA, complete cds
AT4G24415	3.019640969	5.65E-60	Arabidopsis thaliana AT4g24411 mRNA, complete cds
AT3G61400	5.94619051	1.17E-59	Arabidopsis thaliana 1-aminocyclopropane-1-carboxylate oxidase-like 8 mRNA, complete cds
AT5G20150	2.254240032	1.25E-59	Arabidopsis thaliana SPX domain-containing protein 1 mRNA, complete cds
AT5G03890	4.348436456	1.32E-59	Arabidopsis thaliana chromosome 5 sequence
AT1G13740	1.696050952	1.56E-59	Arabidopsis thaliana ABI five binding protein 2 mRNA, complete cds
AT2G40900	1.108856734	1.79E-59	Arabidopsis thaliana nodulin MtN21-like transporter family protein mRNA, complete cds
AT5G03230	1.634791253	1.83E-59	Arabidopsis thaliana chromosome 5 sequence
AT3G56080	1.855365068	1.85E-59	Arabidopsis thaliana probable methyltransferase PMT22 mRNA, complete cds
AT5G10300	2.2188953	2.31E-59	Arabidopsis thaliana methyl esterase 5 mRNA, complete cds

AT3G51860	2.370548131	4.64E-59	Arabidopsis thaliana vacuolar cation/proton exchanger 3 mRNA, complete cds
AT5G41100	1.581357337	4.75E-59	Arabidopsis thaliana uncharacterized protein mRNA, complete cds
AT1G76040	1.227852485	5.04E-59	Arabidopsis thaliana calcium-dependent protein kinase 29 mRNA, complete cds
AT5G64810	4.957967678	6.39E-59	Arabidopsis thaliana putative WRKY transcription factor 51 mRNA, complete cds
AT2G03240	1.384818813	8.47E-59	Arabidopsis thaliana EXS (ERD1/XPR1/SYG1) family protein mRNA, complete cds
AT1G76590	2.517953513	1.01E-58	Arabidopsis thaliana PLATZ transcription factor family protein mRNA, complete cds
AT1G19490	2.085274622	2.85E-58	Arabidopsis thaliana basic-leucine zipper transcription factor family protein mRNA, complete cds
AT1G01240	1.196281011	5.18E-58	Arabidopsis thaliana chromosome 1 sequence
AT1G60470	3.251829823	6.49E-58	Arabidopsis thaliana galactinol synthase 4 mRNA, complete cds
AT5G13205.1	2.63490405	1.08E-57	Arabidopsis thaliana chromosome 5 sequence
AT1G58180	1.349063072	1.20E-57	Arabidopsis thaliana beta carbonic anhydrase 6 mRNA, complete cds
AT2G39980	1.92494088	1.77E-57	Arabidopsis thaliana chromosome 2, complete sequence
AT1G03610	1.269428239	2.46E-57	Arabidopsis thaliana uncharacterized protein mRNA, complete cds
AT1G30190	3.731660206	2.48E-57	Arabidopsis thaliana chromosome 1 sequence
AT3G15250	3.844341256	3.01E-57	Arabidopsis thaliana chromosome 3, complete sequence
AT5G43380	2.6273109	3.16E-57	Arabidopsis thaliana type one serine/threonine protein phosphatase 6 mRNA, complete cds
AT1G07150	3.450390229	3.58E-57	Arabidopsis thaliana mitogen-activated protein kinase kinase kinase 13 mRNA, complete cds
AT1G09530	1.90633145	3.72E-57	Arabidopsis thaliana transcription factor PIF3 mRNA, complete cds
AT3G54140	1.235563931	5.69E-57	Arabidopsis thaliana peptide transporter PTR1 mRNA, complete cds
AT2G18690	1.358185882	6.81E-57	Arabidopsis thaliana chromosome 2, complete sequence
AT3G17770	1.018476635	8.38E-57	Arabidopsis thaliana dihydroxyacetone kinase mRNA, complete cds

AT1G47510	3.011047993	8.64E-57	Arabidopsis thaliana Type I inositol-1,4,5-trisphosphate 5-phosphatase 11 mRNA, complete cds
AT1G04250	1.25390194	1.05E-56	Arabidopsis thaliana auxin-responsive protein IAA17 mRNA, complete cds
AT4G31750	1.408192005	1.13E-56	Arabidopsis thaliana putative protein phosphatase 2C 59 mRNA, complete cds
AT3G05580	1.707503479	1.97E-56	Arabidopsis thaliana serine/threonine-protein phosphatase PP1 isozyme 9 mRNA, complete cds
AT5G08240	2.111153589	2.42E-56	Arabidopsis thaliana uncharacterized protein mRNA, complete cds
AT1G53560	1.496424942	2.78E-56	Arabidopsis thaliana ribosomal protein L18ae family mRNA, complete cds
AT1G73805	1.433988116	3.21E-56	Arabidopsis thaliana protein SAR Deficient 1 mRNA, complete cds
AT3G16720	1.648364635	3.42E-56	Arabidopsis thaliana chromosome 3, complete sequence
AT2G38750	2.012632653	4.57E-56	Arabidopsis thaliana annexin D4 mRNA, complete cds
AT1G03090	1.131536692	5.17E-56	Arabidopsis thaliana methylcrotonoyl-CoA carboxylase subunit alpha mRNA, complete cds
AT1G74710	1.521805838	8.81E-56	Arabidopsis thaliana Isochorismate synthase 1 mRNA, complete cds
AT2G38240	6.26969446	9.05E-56	Arabidopsis thaliana 2-oxoglutarate (2OG) and Fe(II)-dependent oxygenase-like protein mRNA, complete cds
AT5G13820	1.84580992	1.19E-55	Arabidopsis thaliana Telomere repeat-binding protein 4 mRNA, complete cds
AT1G78610	1.392610788	1.67E-55	Arabidopsis thaliana mechanosensitive channel of small conductance-like 6 mRNA, complete cds
AT5G41550	3.544232838	1.89E-55	Arabidopsis thaliana TIR-NBS-LRR class disease resistance protein mRNA, complete cds
AT4G16670	1.844677274	3.17E-55	Arabidopsis thaliana uncharacterized protein mRNA, complete cds
AT2G38740	1.20809825	3.34E-55	Arabidopsis thaliana haloacid dehalogenase-like hydrolase family protein mRNA, complete cds
AT5G17860	4.867202214	3.82E-55	Arabidopsis thaliana chromosome 5 sequence
AT5G63450	4.867202214	3.82E-55	Arabidopsis thaliana chromosome 5 sequence
AT5G59960	1.139281759	5.35E-55	Arabidopsis thaliana uncharacterized protein

			mRNA, complete cds
AT1G66090	1.862408798	7.17E-55	Arabidopsis thaliana TIR-NBS class of disease resistance protein mRNA, complete cds
AT2G39650	2.679773465	7.73E-55	Arabidopsis thaliana uncharacterized protein mRNA, complete cds
AT2G43620	2.303433935	1.27E-54	Arabidopsis thaliana chitinase family protein mRNA, complete cds
AT2G29490	2.970784123	1.38E-54	Arabidopsis thaliana glutathione S-transferase tau 1 mRNA, complete cds
AT4G01540	2.501637519	1.45E-54	Arabidopsis thaliana NAC with transmembrane motif1 mRNA, complete cds
AT2G25690	2.274429362	1.46E-54	Arabidopsis thaliana uncharacterized protein mRNA, complete cds
AT4G30460	2.612388315	1.47E-54	Arabidopsis thaliana chromosome 4 sequence
AT3G15990	1.528894376	1.48E-54	Arabidopsis thaliana putative sulfate transporter 3;4 mRNA, complete cds
AT5G04720	1.020731975	1.54E-54	Arabidopsis thaliana ADR1-like 2 protein mRNA, complete cds
AT3G13600	2.428051626	1.57E-54	Arabidopsis thaliana calmodulin-binding family protein mRNA, complete cds
AT2G23840	2.104458178	1.95E-54	Arabidopsis thaliana HNH endonuclease mRNA, complete cds
AT5G52320	1.816472138	2.16E-54	Arabidopsis thaliana chromosome 5 sequence
AT3G44100	1.217368288	2.58E-54	Arabidopsis thaliana MD-2-related lipid recognition domain-containing protein mRNA, complete cds
AT3G62100	4.22190405	5.94E-54	Arabidopsis thaliana auxin-responsive protein IAA30 mRNA, complete cds
AT4G01250	1.606899707	6.31E-54	Arabidopsis thaliana WRKY transcription factor 22 mRNA, complete cds
AT2G43500	1.658177393	1.01E-53	Arabidopsis thaliana nodule inception protein-like protein 8 mRNA, complete cds
AT1G52000	1.142395003	1.99E-53	Arabidopsis thaliana mannose-binding lectin superfamily protein mRNA, complete cds
AT5G55400	1.692585517	2.51E-53	Arabidopsis thaliana actin binding Calponin homology domain-containing protein mRNA, complete cds
AT1G02930	1.071429378	2.63E-53	Arabidopsis thaliana glutathione S-transferase F6 mRNA, complete cds
AT5G18470	1.671498771	3.04E-53	Arabidopsis thaliana chromosome 5 sequence
AT5G13750	1.589689267	3.38E-53	Arabidopsis thaliana zinc induced facilitator-like 1 protein mRNA, complete cds

AT5G27520	1.693052914	4.29E-53	Arabidopsis thaliana peroxisomal adenine nucleotide carrier 2 mRNA, complete cds
AT4G30850	1.392545868	4.83E-53	Arabidopsis thaliana heptahelical transmembrane protein2 mRNA, complete cds
AT1G21670	1.704572904	6.56E-53	Arabidopsis thaliana chromosome 1 sequence
AT3G53960	1.275688945	7.35E-53	Arabidopsis thaliana probable peptide/nitrate transporter mRNA, complete cds
AT5G24530	1.161046596	9.66E-53	Arabidopsis thaliana putative 2OG-Fe(II) oxygenase mRNA, complete cds
AT3G10020	1.236854391	1.05E-52	Arabidopsis thaliana chromosome 3, complete sequence
AT3G18950	2.043407234	1.35E-52	Arabidopsis thaliana chromosome 3, complete sequence
AT3G29410	1.083899149	3.03E-52	Arabidopsis thaliana terpenoid synthase 25 mRNA, complete cds
AT2G40475	1.673664655	7.50E-52	Arabidopsis thaliana chromosome 2, complete sequence
AT4G27310	1.737416603	9.00E-52	Arabidopsis thaliana B-box domain protein 28 mRNA, complete cds
AT3G15500	3.421445532	1.04E-51	Arabidopsis thaliana ATAF-like NAC-domain transcription factor mRNA, complete cds
AT2G38400	1.216791126	1.15E-51	Arabidopsis thaliana alanine:glyoxylate aminotransferase 3 mRNA, complete cds
AT1G32920	1.744805582	1.86E-51	Arabidopsis thaliana chromosome 1 sequence
AT5G17380	1.07958351	2.51E-51	Arabidopsis thaliana 2-hydroxyacyl-CoA lyase mRNA, complete cds
AT2G34720	1.153781329	3.76E-51	Arabidopsis thaliana nuclear transcription factor Y subunit A-4 mRNA, complete cds
AT2G47600	1.19302595	5.00E-51	Arabidopsis thaliana magnesium/proton exchanger mRNA, complete cds
AT2G14960	3.211850385	7.00E-51	Arabidopsis thaliana putative indole-3-acetic acid-amido synthetase GH3.1 mRNA, complete cds
AT4G25810	2.04787465	1.12E-50	Arabidopsis thaliana probable xyloglucan endotransglucosylase/hydrolase protein 23 mRNA, complete cds
AT1G55110	1.489384361	1.33E-50	Arabidopsis thaliana indeterminate(ID)-domain 7 protein mRNA, complete cds
AT1G18870	2.722671423	2.01E-50	Arabidopsis thaliana Isochorismate synthase 2 mRNA, complete cds
AT5G04460	1.172554134	2.05E-50	Arabidopsis thaliana RING/U-box domain-containing protein mRNA, complete

			cds
AT2G43240	1.659916685	2.75E-50	Arabidopsis thaliana nucleotide-sugar transporter mRNA, complete cds
AT3G54030	1.233189231	2.83E-50	Arabidopsis thaliana Protein kinase protein with tetratricopeptide repeat domain mRNA, complete cds
AT4G28240	1.0907413	6.28E-50	Arabidopsis thaliana chromosome 4 sequence
AT4G37780	3.571502981	6.38E-50	Arabidopsis thaliana myb domain protein 87 mRNA, complete cds
AT2G35710	1.747163041	6.99E-50	Arabidopsis thaliana putative glucuronosyltransferase PGSIP8 mRNA, complete cds
AT3G15670	7.822808094	8.92E-50	Arabidopsis thaliana Late embryogenesis abundant protein (LEA) family protein mRNA, complete cds
AT3G44326	3.185378174	1.24E-49	Arabidopsis thaliana chromosome 3, complete sequence
AT5G48070	2.059022187	1.35E-49	Arabidopsis thaliana xyloglucan endotransglucosylase/hydrolase protein 20 mRNA, complete cds
AT5G63320	1.007504419	1.43E-49	Arabidopsis thaliana nuclear protein X1 mRNA, complete cds
AT5G42570	1.164567987	2.07E-49	Arabidopsis thaliana B-cell receptor-associated 31-like protein mRNA, complete cds
AT4G15760	1.573194204	2.11E-49	Arabidopsis thaliana monooxygenase 1 mRNA, complete cds
AT5G15870	1.219269865	2.59E-49	Arabidopsis thaliana chromosome 5 sequence
AT5G57510	4.421445532	5.99E-49	Arabidopsis thaliana chromosome 5 sequence
AT1G76070	2.54117647	7.03E-49	Arabidopsis thaliana chromosome 1 sequence
AT5G44050	2.426386273	9.75E-49	Arabidopsis thaliana MATE efflux family protein mRNA, complete cds
AT3G47580	1.803179288	1.23E-48	Arabidopsis thaliana Leucine-rich repeat protein kinase family protein mRNA, complete cds
AT5G54300	1.401869994	2.10E-48	Arabidopsis thaliana uncharacterized protein mRNA, complete cds
AT5G09930	3.99600081	2.52E-48	Arabidopsis thaliana ABC transporter F family member 2 mRNA, complete cds
AT5G13080	3.644042606	2.81E-48	Arabidopsis thaliana putative WRKY transcription factor 75 mRNA, complete cds
AT3G53830	1.892969166	3.26E-48	Arabidopsis thaliana Regulator of chromosome condensation (RCC1) family protein mRNA, complete cds

AT4G21940	2.427713587	4.25E-48	Arabidopsis thaliana calcium-dependent protein kinase 15 mRNA, complete cds
AT5G47060	1.565650255	6.06E-48	Arabidopsis thaliana uncharacterized protein mRNA, complete cds
AT1G60730	1.531650088	6.14E-48	Arabidopsis thaliana probable aldo-keto reductase 5 mRNA, complete cds
AT1G16670	1.433241283	7.01E-48	Arabidopsis thaliana putative serine/threonine kinase mRNA, complete cds
AT3G50910	1.001491499	7.24E-48	Arabidopsis thaliana uncharacterized protein mRNA, complete cds
AT5G61810	1.698819377	7.41E-48	Arabidopsis thaliana ATP-Mg/Pi transporter mRNA, complete cds
AT2G42440	7.750162793	1.02E-47	Arabidopsis thaliana LOB domain-containing protein 17 mRNA, complete cds
AT1G75400	1.085440691	1.13E-47	Arabidopsis thaliana RING/U-box domain-containing protein mRNA, complete cds
AT1G30320	1.25893217	1.16E-47	Arabidopsis thaliana Remorin family protein mRNA, complete cds
AT1G19970	1.929479949	1.32E-47	Arabidopsis thaliana ER lumen protein retaining receptor mRNA, complete cds
AT5G01100	1.87077342	1.76E-47	Arabidopsis thaliana O-fucosyltransferase family protein mRNA, complete cds
AT1G14200	1.524818054	2.03E-47	Arabidopsis thaliana chromosome 1 sequence
AT2G47485	2.783945272	2.28E-47	Arabidopsis thaliana chromosome 2, complete sequence
AT5G45110	1.392367127	2.69E-47	Arabidopsis thaliana NPR1-like protein 3 mRNA, complete cds
AT1G35910	2.69926906	2.87E-47	Arabidopsis thaliana probable trehalose-phosphate phosphatase D mRNA, complete cds
AT2G04050	3.678418185	3.33E-47	Arabidopsis thaliana MATE efflux family protein mRNA, complete cds
AT1G76680	1.04107898	5.30E-47	Arabidopsis thaliana 12-oxophytodienoate reductase 1 mRNA, complete cds
AT5G23850	1.834880927	7.38E-47	Arabidopsis thaliana uncharacterized protein mRNA, complete cds
AT1G64590	1.734852894	1.03E-46	Arabidopsis thaliana NAD(P)-binding Rossmann-fold superfamily protein mRNA, complete cds
AT2G39030	8.891970119	1.08E-46	Arabidopsis thaliana chromosome 2, complete sequence
AT3G18777	2.938285312	1.24E-46	Arabidopsis thaliana chromosome 3, complete sequence

AT2G32510	2.684865995	1.47E-46	Arabidopsis thaliana chromosome 2, complete sequence
AT3G47510	2.001906641	1.64E-46	Arabidopsis thaliana uncharacterized protein mRNA, complete cds
AT3G51910	1.245289562	1.85E-46	Arabidopsis thaliana heat stress transcription factor A-7a mRNA, complete cds
AT2G34650	1.833455339	1.96E-46	Arabidopsis thaliana protein serine/threonine kinase PINOID mRNA, complete cds
AT5G41400	1.712442291	2.50E-46	Arabidopsis thaliana chromosome 5 sequence
AT5G66460	1.202989716	2.65E-46	Arabidopsis thaliana mannan endo-1,4-beta-mannosidase 7 mRNA, complete cds
AT5G60350	5.255767502	2.81E-46	Arabidopsis thaliana uncharacterized protein mRNA, complete cds
AT2G15880	1.805033393	3.18E-46	Arabidopsis thaliana pollen-specific leucine-rich repeat extensin-like protein 3 mRNA, complete cds
AT2G45050	1.740188478	3.29E-46	Arabidopsis thaliana GATA transcription factor 2 mRNA, complete cds
AT2G20880	3.108562577	3.71E-46	Arabidopsis thaliana chromosome 2, complete sequence
AT3G21890	2.735930644	4.34E-46	Arabidopsis thaliana chromosome 3, complete sequence
AT1G02816	1.618498032	4.83E-46	Arabidopsis thaliana chromosome 1 sequence
AT4G34390	1.060265186	4.91E-46	Arabidopsis thaliana extra-large GTP-binding protein 2 mRNA, complete cds
AT3G01830	4.622783486	5.20E-46	Arabidopsis thaliana chromosome 3, complete sequence
AT5G50720	2.358754918	8.75E-46	Arabidopsis thaliana HVA22-like protein E mRNA, complete cds
AT3G18690	2.062998757	9.13E-46	Arabidopsis thaliana chromosome 3, complete sequence
AT5G04370	2.817646389	9.92E-46	Arabidopsis thaliana methyltransferase family protein NAMT1 mRNA, complete cds
AT1G52560	3.58130685	1.00E-45	Arabidopsis thaliana heat shock protein 26.5 mRNA, complete cds
AT1G68690	1.517953513	1.11E-45	Arabidopsis thaliana proline-rich receptor-like protein kinase PERK9 mRNA, complete cds
AT3G61190	3.468625801	1.35E-45	Arabidopsis thaliana BON association protein 1 mRNA, complete cds
AT2G44080	2.124682242	1.64E-45	Arabidopsis thaliana ARGOS-like protein mRNA, complete cds
AT1G19220	1.146183468	2.79E-45	Arabidopsis thaliana auxin response factor 19

			mRNA, complete cds
AT3G25610	1.390623289	3.40E-45	Arabidopsis thaliana ATPase E1-E2 type family protein / haloacid dehalogenase-like hydrolase family protein mRNA, complete cds
AT1G69830	1.55422604	3.77E-45	Arabidopsis thaliana alpha-amylase-like 3 mRNA, complete cds
AT3G53810	1.490677678	4.44E-45	Arabidopsis thaliana chromosome 3, complete sequence
AT2G03470	1.094586133	1.13E-44	Arabidopsis thaliana ELM2 domain-containing protein mRNA, complete cds
AT1G52855	4.185378174	1.28E-44	Arabidopsis thaliana chromosome 1 sequence
AT4G01360	5.201319718	1.52E-44	Arabidopsis thaliana BYPASS1-related protein mRNA, complete cds
AT4G38410	3.548885373	1.89E-44	Arabidopsis thaliana putative dehydrin mRNA, complete cds
AT5G43420	1.605690615	1.90E-44	Arabidopsis thaliana chromosome 5 sequence
AT4G18170	3.068564509	2.00E-44	Arabidopsis thaliana WRKY DNA-binding protein 28 mRNA, complete cds
AT5G52660	1.621989085	2.31E-44	Arabidopsis thaliana myb family transcription factor mRNA, complete cds
AT5G59490	2.742076822	2.35E-44	Arabidopsis thaliana haloacid dehalogenase-like hydrolase (HAD) superfamily protein mRNA, complete cds
AT4G08170	1.751423461	2.55E-44	Arabidopsis thaliana inositol-tetrakisphosphate 1-kinase 2 mRNA, complete cds
AT1G80160	2.914076152	2.60E-44	Arabidopsis thaliana GLYOXYLASE I 7 mRNA, complete cds
AT5G66390	1.402642687	2.68E-44	Arabidopsis thaliana peroxidase 72 mRNA, complete cds
AT1G09490	1.271070107	2.68E-44	Arabidopsis thaliana alcohol dehydrogenase mRNA, complete cds
AT3G51830	1.15455525	2.77E-44	Arabidopsis thaliana putative transmembrane protein G5p mRNA, complete cds
AT2G27690	3.600415673	3.90E-44	Arabidopsis thaliana chromosome 2, complete sequence
AT3G16510	2.543832145	3.92E-44	Arabidopsis thaliana chromosome 3, complete sequence
AT1G68600	1.502330673	5.90E-44	Arabidopsis thaliana Aluminum activated malate transporter family protein mRNA, complete cds
AT5G65640	1.269498795	8.88E-44	Arabidopsis thaliana transcription factor

			bHLH93 mRNA, complete cds
AT4G17785	4.407770595	9.07E-44	Arabidopsis thaliana transcription factor MYB39 mRNA, complete cds
AT5G63350	4.930336559	9.33E-44	Arabidopsis thaliana chromosome 5 sequence
AT4G20000	2.605416354	1.12E-43	Arabidopsis thaliana chromosome 4 sequence
AT1G60270	1.229506293	1.20E-43	Arabidopsis thaliana beta glucosidase 6 mRNA, complete cds
AT3G20500	1.296117472	1.25E-43	Arabidopsis thaliana purple acid phosphatase 18 mRNA, complete cds
AT4G16590	4.052542491	1.27E-43	Arabidopsis thaliana cellulose synthase-like A01 mRNA, complete cds
AT1G60750	4.155934396	1.37E-43	Arabidopsis thaliana probable aldo-keto reductase 6 mRNA, complete cds
AT1G27910	1.424491931	1.53E-43	Arabidopsis thaliana U-box domain-containing protein 45 mRNA, complete cds
AT3G10250	1.088362751	1.83E-43	Arabidopsis thaliana uncharacterized protein mRNA, complete cds
AT1G76650	2.658026396	2.07E-43	Arabidopsis thaliana chromosome 1 sequence
AT3G45300	1.364148177	2.48E-43	Arabidopsis thaliana isovaleryl-CoA-dehydrogenase mRNA, complete cds
AT1G70300	1.384868055	2.69E-43	Arabidopsis thaliana potassium transporter 6 mRNA, complete cds
AT5G64900	2.674962627	3.00E-43	Arabidopsis thaliana precursor of peptide 1 mRNA, complete cds
AT5G48110	1.486428843	3.17E-43	Arabidopsis thaliana terpenoid synthase 20 mRNA, complete cds
AT3G48240	3.780987919	3.22E-43	Arabidopsis thaliana Octicosapeptide/Phox/Bem1p family protein mRNA, complete cds
AT4G11890	2.206317512	3.33E-43	Arabidopsis thaliana receptor-like cytosolic kinase ARCK1 mRNA, complete cds
AT4G39030	1.667939667	3.89E-43	Arabidopsis thaliana enhanced disease susceptibility 5 mRNA, complete cds
AT4G02410	1.306737055	4.47E-43	Arabidopsis thaliana chromosome 4 sequence
AT1G79700	1.374628311	5.14E-43	Arabidopsis thaliana AP2-like ethylene-responsive transcription factor WRI4 mRNA, complete cds
AT3G28210	3.45507631	5.46E-43	Arabidopsis thaliana zinc finger (AN1-like) family protein mRNA, complete cds
AT2G16630	1.62274022	5.75E-43	Arabidopsis thaliana pollen Ole e 1 allergen and extensin family protein mRNA, complete cds

AT3G54000	2.113856876	5.76E-43	Arabidopsis thaliana uncharacterized protein mRNA, complete cds
AT1G23200	2.910983906	5.80E-43	Arabidopsis thaliana probable pectinesterase/pectinesterase inhibitor 6 mRNA, complete cds
AT5G28646	3.35043742	6.89E-43	Arabidopsis thaliana protein WAVE-DAMPENED 2 mRNA, complete cds
AT3G60120	5.865118899	7.61E-43	Arabidopsis thaliana beta glucosidase 27 mRNA, complete cds
AT4G35985	1.643484395	7.73E-43	Arabidopsis thaliana senescence/dehydration-associated protein mRNA, complete cds
AT5G66050	1.110116368	7.83E-43	Arabidopsis thaliana Wound-responsive family protein mRNA, complete cds
AT4G22780	1.505898074	1.30E-42	Arabidopsis thaliana ACT domain repeat 7 protein mRNA, complete cds
AT1G59700	1.393073142	1.35E-42	Arabidopsis thaliana glutathione S-transferase TAU 16 mRNA, complete cds
AT1G54575	2.172994449	1.52E-42	Arabidopsis thaliana chromosome 1 sequence
AT3G55290	2.399596664	1.87E-42	Arabidopsis thaliana Rossmann-fold NAD(P)-binding domain-containing protein mRNA, complete cds
AT2G35350	1.378023252	5.44E-42	Arabidopsis thaliana protein phosphatase 2C 29 mRNA, complete cds
AT1G04490	3.673664655	5.53E-42	Arabidopsis thaliana uncharacterized protein mRNA, complete cds
AT1G20650	1.131752204	8.37E-42	Arabidopsis thaliana protein ALTERED SEED GERMINATION 5 mRNA, complete cds
AT1G79310	2.800353244	1.08E-41	Arabidopsis thaliana metacaspase 7 mRNA, complete cds
AT5G58160	1.189848878	1.11E-41	Arabidopsis thaliana formin-like protein 13 mRNA, complete cds
AT2G01150	1.533421367	1.32E-41	Arabidopsis thaliana chromosome 2, complete sequence
AT4G03820	1.603241717	1.77E-41	Arabidopsis thaliana uncharacterized protein mRNA, complete cds
AT2G25530	1.773193059	1.81E-41	Arabidopsis thaliana AFG1-like ATPase family protein mRNA, complete cds
AT2G19710	1.406605722	2.09E-41	Arabidopsis thaliana Vps4 regulator of MVB pathway mRNA, complete cds
AT5G46780	1.041871521	2.58E-41	Arabidopsis thaliana chromosome 5 sequence
AT5G66700	3.35784537	2.71E-41	Arabidopsis thaliana homeobox-leucine zipper protein ATHB-53 mRNA, complete

			cds
AT1G12950	2.158057568	2.89E-41	Arabidopsis thaliana root hair specific 2 mRNA, complete cds
AT2G12400	1.210224551	3.83E-41	Arabidopsis thaliana uncharacterized protein mRNA, complete cds
AT4G37770	3.517953513	4.47E-41	Arabidopsis thaliana 1-aminocyclopropane-1-carboxylate synthase 8 mRNA, complete cds
AT2G38820	1.589188418	4.73E-41	Arabidopsis thaliana uncharacterized protein mRNA, complete cds
AT2G37770	2.710333365	5.87E-41	Arabidopsis thaliana aldo-keto reductase family 4 member C9 mRNA, complete cds
AT5G50360	8.652077793	6.61E-41	Arabidopsis thaliana chromosome 5 sequence
AT3G54950	1.586367899	7.13E-41	Arabidopsis thaliana patatin-like protein 6 mRNA, complete cds
AT1G69010	1.300068109	9.22E-41	Arabidopsis thaliana transcription factor BIM2 mRNA, complete cds
AT2G04240	1.488522793	1.16E-40	Arabidopsis thaliana chromosome 2, complete sequence
AT5G01300	7.466664284	2.01E-40	Arabidopsis thaliana putative phosphatidylethanolamine-binding protein mRNA, complete cds
AT1G16130	2.342182254	2.08E-40	Arabidopsis thaliana mRNA for hypothetical protein, complete cds, clone: RAFL21-09-D16
AT3G16800	1.450668554	2.10E-40	Arabidopsis thaliana putative protein phosphatase 2C mRNA, complete cds
AT3G23170	1.847899075	2.84E-40	Arabidopsis thaliana chromosome 3, complete sequence
AT1G47960	1.58334216	3.13E-40	Arabidopsis thaliana cell wall / vacuolar inhibitor of fructosidase 1 mRNA, complete cds
AT3G12510	2.788042676	4.15E-40	Arabidopsis thaliana chromosome 3, complete sequence
AT4G14560	1.535926947	4.29E-40	Arabidopsis thaliana auxin-responsive protein IAA1 mRNA, complete cds
AT1G05575	4.608977687	4.61E-40	Arabidopsis thaliana chromosome 1 sequence
AT1G66160	2.472310847	4.73E-40	Arabidopsis thaliana chromosome 1 sequence
AT5G17850	1.90372809	8.88E-40	Arabidopsis thaliana chromosome 5 sequence
AT4G19720	2.805033393	1.01E-39	Arabidopsis thaliana Glycosyl hydrolase family protein with chitinase insertion domain mRNA, complete cds
AT5G06370	1.151887109	1.03E-39	Arabidopsis thaliana lecithin retinol acyltransferase domain protein mRNA,

			complete cds
AT1G69610	1.822808094	1.46E-39	Arabidopsis thaliana uncharacterized protein mRNA, complete cds
AT4G13110	1.980120099	1.58E-39	Arabidopsis thaliana chromosome 4 sequence
AT4G37030	2.48820617	3.14E-39	Arabidopsis thaliana uncharacterized protein mRNA, complete cds
AT3G05630	3.158411126	4.61E-39	Arabidopsis thaliana phospholipase D P2 mRNA, complete cds
AT2G32990	1.037055427	4.90E-39	Arabidopsis thaliana glycosyl hydrolase 9B8 mRNA, complete cds
AT4G15450	1.32057298	5.55E-39	Arabidopsis thaliana Senescence/dehydration-associated protein-like protein mRNA, complete cds
AT1G08630	4.128153305	6.43E-39	Arabidopsis thaliana threonine aldolase mRNA, complete cds
AT1G13260	1.03552309	6.61E-39	Arabidopsis thaliana chromosome 1 sequence
AT1G11185	3.288471667	8.96E-39	Arabidopsis thaliana chromosome 1 sequence
AT3G49580	4.76140755	1.07E-38	Arabidopsis thaliana protein RESPONSE TO LOW SULFUR 1 mRNA, complete cds
AT3G54020	1.440907288	1.10E-38	Arabidopsis thaliana phosphatidylinositol:ceramide inositolphosphotransferase 1 mRNA, complete cds
AT3G48510	5.706615077	1.42E-38	Arabidopsis thaliana chromosome 3, complete sequence
AT2G24130	3.445441013	1.56E-38	Arabidopsis thaliana putative leucine-rich repeat receptor-like serine/threonine-protein kinase mRNA, complete cds
AT2G43820	1.231710808	1.71E-38	Arabidopsis thaliana UDP-glucosyltransferase 74F2 mRNA, complete cds
AT1G10560	2.312719213	1.95E-38	Arabidopsis thaliana chromosome 1 sequence
AT1G55920	1.005090003	2.98E-38	Arabidopsis thaliana chromosome 1 sequence
AT4G28703	3.88992229	3.26E-38	Arabidopsis thaliana cupin domain-containing protein mRNA, complete cds
AT3G01510	1.441059913	3.69E-38	Arabidopsis thaliana phosphoglucan phosphatase LSF1 mRNA, complete cds
AT4G34131	2.178391744	4.39E-38	Arabidopsis thaliana chromosome 4 sequence
AT3G55840	2.909462477	4.69E-38	Arabidopsis thaliana chromosome 3, complete sequence
AT1G17180	3.266991939	5.08E-38	Arabidopsis thaliana glutathione S-transferase TAU 25 mRNA, complete cds
AT5G16360	2.14014892	7.32E-38	Arabidopsis thaliana putative lecithin retinol

			acyltransferase-like protein mRNA, complete cds
AT1G34050	4.364181327	8.17E-38	Arabidopsis thaliana ankyrin repeats-containing protein mRNA, complete cds
AT1G54160	2.756534875	8.29E-38	Arabidopsis thaliana nuclear transcription factor Y subunit A-5 mRNA, complete cds
AT1G04310	1.744251446	9.15E-38	Arabidopsis thaliana ethylene response sensor 2 mRNA, complete cds
AT4G01280	1.208388152	9.35E-38	Arabidopsis thaliana homeodomain-like superfamily protein mRNA, complete cds
AT1G12610	8.507306269	9.42E-38	Arabidopsis thaliana chromosome 1 sequence
AT4G17650	1.325699462	9.67E-38	Arabidopsis thaliana polyketide cyclase / dehydrase and lipid transport protein mRNA, complete cds
AT1G66400	2.124836632	1.19E-37	Arabidopsis thaliana chromosome 1 sequence
AT3G19240	1.115758078	1.35E-37	Arabidopsis thaliana vacuolar import/degradation, Vid27-related protein mRNA, complete cds
AT5G26920	1.333814652	1.51E-37	Arabidopsis thaliana Cam-binding protein 60-like G mRNA, complete cds
AT2G17500	1.59113786	1.66E-37	Arabidopsis thaliana auxin efflux carrier family protein mRNA, complete cds
AT4G24480	1.294581554	2.40E-37	Arabidopsis thaliana protein kinase family protein mRNA, complete cds
AT2G36640	7.328336128	2.94E-37	Arabidopsis thaliana putative phosphotyrosine mRNA, complete cds
AT1G15010	3.856755426	3.49E-37	Arabidopsis thaliana chromosome 1 sequence
AT2G41880	1.244339356	3.55E-37	Arabidopsis thaliana guanylate kinase 1 mRNA, complete cds
AT4G21680	2.662343422	4.28E-37	Arabidopsis thaliana nitrate transporter 1.8 mRNA, complete cds
AT3G14280	1.512609292	5.03E-37	Arabidopsis thaliana uncharacterized protein mRNA, complete cds
AT1G15430	1.688735476	5.31E-37	Arabidopsis thaliana chromosome 1 sequence
AT1G62560	1.412078635	7.22E-37	Arabidopsis thaliana flavin-containing monooxygenase FMO GS-OX3 mRNA, complete cds
AT1G24145	2.656268908	7.35E-37	Arabidopsis thaliana uncharacterized protein mRNA, complete cds
AT4G38400	2.210210027	7.95E-37	Arabidopsis thaliana expansin-like A2 mRNA, complete cds
AT1G51090	3.749641682	1.03E-36	Arabidopsis thaliana uncharacterized protein mRNA, complete cds

AT5G05270	1.105416549	1.28E-36	Arabidopsis thaliana Chalcone-flavanone isomerase family protein mRNA, complete cds
AT5G58690	1.584613817	1.89E-36	Arabidopsis thaliana phosphoinositide phospholipase C 5 mRNA, complete cds
AT4G21580	1.00450584	2.91E-36	Arabidopsis thaliana oxidoreductase, zinc-binding dehydrogenase family protein mRNA, complete cds
AT3G46080	6.205277731	2.99E-36	Arabidopsis thaliana chromosome 3, complete sequence
AT4G34280	1.191610128	3.19E-36	Arabidopsis thaliana WD40 domain-containing protein mRNA, complete cds
AT5G01260	1.091255997	3.36E-36	Arabidopsis thaliana carbohydrate-binding domain-containing protein mRNA, complete cds
AT3G49690	2.124574895	3.67E-36	Arabidopsis thaliana transcription factor RAX3 mRNA, complete cds
AT1G63840	1.707880371	4.04E-36	Arabidopsis thaliana chromosome 1 sequence
AT4G27657	2.856755426	4.07E-36	Arabidopsis thaliana chromosome 4 sequence
AT5G66675	1.171283344	4.12E-36	Arabidopsis thaliana uncharacterized protein mRNA, complete cds
AT2G18550	5.205277731	4.41E-36	Arabidopsis thaliana homeobox-leucine zipper protein ATHB-21 mRNA, complete cds
AT3G21420	1.372843013	4.47E-36	Arabidopsis thaliana oxidoreductase, 2OG-Fe(II) oxygenase family protein mRNA, complete cds
AT2G46600	1.12617999	4.72E-36	Arabidopsis thaliana chromosome 2, complete sequence
AT3G28740	4.300855391	5.17E-36	Arabidopsis thaliana cytochrome P450 CYP81D11 mRNA, complete cds
AT2G21590	1.721340455	5.52E-36	Arabidopsis thaliana glucose-1-phosphate adenylyltransferase large subunit mRNA, complete cds
AT5G12340	2.564791763	5.78E-36	Arabidopsis thaliana chromosome 5 sequence
AT4G35190	2.790240232	6.35E-36	Arabidopsis thaliana cytokinin riboside 5'-monophosphate phosphoribohydrolase LOG5 mRNA, complete cds
AT4G18390	1.021996018	6.60E-36	Arabidopsis thaliana transcription factor TCP2 mRNA, complete cds
AT2G02230	1.520492129	7.42E-36	Arabidopsis thaliana F-box protein PP2-B1 mRNA, complete cds
AT4G20070	1.033917235	8.14E-36	Arabidopsis thaliana allantoate

			amidohydrolase mRNA, complete cds
AT1G61120	6.185378174	8.57E-36	Arabidopsis thaliana terpene synthase 04 mRNA, complete cds
AT3G14370	3.022648674	1.04E-35	Arabidopsis thaliana chromosome 3, complete sequence
AT5G06300	2.068094652	1.50E-35	Arabidopsis thaliana cytokinin riboside 5'-monophosphate phosphoribohydrolase LOG7 mRNA, complete cds
AT5G40880	1.807168971	1.59E-35	Arabidopsis thaliana zinc finger CCCH domain-containing protein 59 mRNA, complete cds
AT3G28007	3.707330877	1.99E-35	Arabidopsis thaliana bidirectional sugar transporter SWEET4 mRNA, complete cds
AT2G43120	1.025483538	2.13E-35	Arabidopsis thaliana mRNA for putative pirin protein, complete cds, clone: RAFL16-13-C22
AT3G48100	1.248572	2.45E-35	Arabidopsis thaliana two-component response regulator ARR5 mRNA, complete cds
AT2G38250	3.291087615	2.50E-35	Arabidopsis thaliana trihelix transcription factor GT-3b mRNA, complete cds
AT2G22850	1.993015783	3.15E-35	Arabidopsis thaliana basic leucine-zipper 6 mRNA, complete cds
AT3G53600	8.381775387	3.30E-35	Arabidopsis thaliana chromosome 3, complete sequence
AT2G44070	8.381775387	3.30E-35	Arabidopsis thaliana NagB/RpiA/CoA transferase-like superfamily protein mRNA, complete cds
AT5G24640	8.381775387	3.30E-35	Arabidopsis thaliana chromosome 5 sequence
AT2G02990	8.381775387	3.30E-35	Arabidopsis thaliana ribonuclease 1 mRNA, complete cds
AT5G56960	2.687070055	3.39E-35	Arabidopsis thaliana putative transcription factor bHLH041 mRNA, complete cds
AT4G02360	3.232683889	3.61E-35	Arabidopsis thaliana chromosome 4 sequence
AT5G10410	2.089707727	4.13E-35	Arabidopsis thaliana ENTH/ANTH/VHS superfamily protein mRNA, complete cds
AT5G13700	3.283225497	4.46E-35	Arabidopsis thaliana polyamine oxidase 1 mRNA, complete cds
AT3G02040	2.320955719	4.94E-35	Arabidopsis thaliana glycerophosphodiester phosphodiesterase 1 mRNA, complete cds
AT5G35110	3.608977687	5.74E-35	Arabidopsis thaliana chromosome 5 sequence
AT3G25710	1.096807867	5.77E-35	Arabidopsis thaliana transcription factor BHLH32 mRNA, complete cds
AT1G67300	1.138621215	5.86E-35	Arabidopsis thaliana putative plastidic glucose transporter 2 mRNA, complete cds

AT4G17470	3.224906538	6.43E-35	Arabidopsis thaliana putative palmitoyl-protein thioesterase mRNA, complete cds
AT2G42760	1.666504864	8.81E-35	Arabidopsis thaliana chromosome 2, complete sequence
AT1G72760	4.112314712	9.26E-35	Arabidopsis thaliana putative serine/threonine protein kinase mRNA, complete cds
AT5G47330	2.674072715	1.01E-34	Arabidopsis thaliana palmitoyl protein thioesterase family protein mRNA, complete cds
AT5G41590	4.417551616	1.11E-34	Arabidopsis thaliana uncharacterized protein mRNA, complete cds
AT3G03170	3.217087034	1.14E-34	Arabidopsis thaliana uncharacterized protein mRNA, complete cds
AT2G36270	5.144736189	1.37E-34	Arabidopsis thaliana protein abscisic acid-insensitive 5 mRNA, complete cds
AT3G19970	1.574004042	1.60E-34	Arabidopsis thaliana uncharacterized protein mRNA, complete cds
AT3G11690	1.574004042	1.60E-34	Arabidopsis thaliana chromosome 3, complete sequence
AT3G05660	3.591802543	1.86E-34	Arabidopsis thaliana receptor like protein 33 mRNA, complete cds
AT1G64970	1.093879819	2.17E-34	Arabidopsis thaliana gamma-tocopherol methyltransferase mRNA, complete cds
AT3G06020	1.614388616	2.48E-34	Arabidopsis thaliana chromosome 3, complete sequence
AT3G10260	1.053781291	2.72E-34	Arabidopsis thaliana reticulon-like protein B8 mRNA, complete cds
AT4G25390	1.590219906	3.03E-34	Arabidopsis thaliana chromosome 4 sequence
AT2G41730	4.397922809	3.61E-34	Arabidopsis thaliana chromosome 2, complete sequence
AT2G38760	2.006054474	3.89E-34	Arabidopsis thaliana annexin D3 mRNA, complete cds
AT4G20860	1.055991332	4.06E-34	Arabidopsis thaliana chromosome 4 sequence
AT1G75860	1.172002452	4.08E-34	Arabidopsis thaliana uncharacterized protein mRNA, complete cds
AT1G61610	3.015453172	4.11E-34	Arabidopsis thaliana putative G-type lectin S-receptor-like serine/threonine-protein kinase mRNA, complete cds
AT1G18710	2.768615994	4.24E-34	Arabidopsis thaliana myb domain protein 47 mRNA, complete cds
AT1G59910	1.0241389	4.81E-34	Arabidopsis thaliana formin-like protein 7 mRNA, complete cds
AT1G28260	1.401382267	4.83E-34	Arabidopsis thaliana Telomerase activating

			protein Est1 mRNA, complete cds
AT1G50750	2.343140537	5.36E-34	Arabidopsis thaliana plant mobile domain family protein mRNA, complete cds
AT5G66650	2.76169658	7.36E-34	Arabidopsis thaliana uncharacterized protein mRNA, complete cds
AT1G76700	1.038824444	7.42E-34	Arabidopsis thaliana chaperone protein dnaJ 10 mRNA, complete cds
AT1G57590	2.151915415	8.02E-34	Arabidopsis thaliana pectinacetylesterase family protein mRNA, complete cds
AT5G22520	4.215125517	1.08E-33	Arabidopsis thaliana chromosome 5 sequence
AT5G23750	1.317841081	1.22E-33	Arabidopsis thaliana Remorin family protein mRNA, complete cds
AT3G55720	2.459553137	1.45E-33	Arabidopsis thaliana uncharacterized protein mRNA, complete cds
AT3G27870	1.336038601	1.50E-33	Arabidopsis thaliana putative phospholipid-transporting ATPase 8 mRNA, complete cds
AT4G35550	1.16649093	1.55E-33	Arabidopsis thaliana WUSCHEL-related homeobox 13 mRNA, complete cds
AT5G65790	1.645153988	1.88E-33	Arabidopsis thaliana myb domain protein 68 mRNA, complete cds
AT5G19230	1.317663033	2.43E-33	Arabidopsis thaliana GPI-anchored glycoprotein membrane precursor mRNA, complete cds
AT4G01950	1.020747472	2.50E-33	Arabidopsis thaliana putative sn-glycerol-3-phosphate 2-O-acyltransferase mRNA, complete cds
AT1G07000	1.047039515	2.63E-33	Arabidopsis thaliana exocyst subunit exo70 family protein B2 mRNA, complete cds
AT5G11140	4.79155716	2.97E-33	Arabidopsis thaliana chromosome 5 sequence
AT1G69920	4.054981537	3.27E-33	Arabidopsis thaliana glutathione S-transferase TAU 12 mRNA, complete cds
AT1G02850	2.21598616	3.27E-33	Arabidopsis thaliana beta glucosidase 11 mRNA, complete cds
AT2G34600	8.272841015	3.92E-33	Arabidopsis thaliana jasmonate-zim-domain protein 7 mRNA, complete cds
AT5G42010	1.810134264	4.17E-33	Arabidopsis thaliana WD40 domain-containing protein mRNA, complete cds
AT5G46910	1.199877744	4.83E-33	Arabidopsis thaliana transcription factor jumonji (jmj) family protein / zinc finger (C5HC2 type) family protein mRNA, complete cds
AT3G10500	1.264008081	5.37E-33	Arabidopsis thaliana NAC domain containing

			protein 53 mRNA, complete cds
AT5G44350	3.161130628	6.41E-33	Arabidopsis thaliana chromosome 5 sequence
AT5G10730	1.344334028	7.78E-33	Arabidopsis thaliana Rossmann-fold NAD(P)-binding domain-containing protein mRNA, complete cds
AT4G38730	1.41264267	8.03E-33	Arabidopsis thaliana uncharacterized protein mRNA, complete cds
AT5G22430	2.50088	8.69E-33	Arabidopsis thaliana pollen Ole e 1 allergen and extensin family protein mRNA, complete cds
AT5G60850	1.055824355	9.21E-33	Arabidopsis thaliana chromosome 5 sequence
AT3G03310	1.214233037	1.15E-32	Arabidopsis thaliana phospholipase A(1) LCAT3 mRNA, complete cds
AT5G52870	1.230553101	1.35E-32	Arabidopsis thaliana membrane-associated kinase regulator family protein mRNA, complete cds
AT1G06210	1.039922168	1.77E-32	Arabidopsis thaliana ENTH/VHS/GAT family protein mRNA, complete cds
AT4G30280	1.212548375	1.91E-32	Arabidopsis thaliana mRNA for hypothetical protein, complete cds, clone: RAFL24-01-A19
AT5G49280	1.27641325	1.99E-32	Arabidopsis thaliana chromosome 5 sequence
AT1G61360	1.128794645	2.10E-32	Arabidopsis thaliana G-type lectin S-receptor-like serine/threonine-protein kinase mRNA, complete cds
AT3G61060	1.366527613	2.13E-32	Arabidopsis thaliana phloem protein 2-A13 mRNA, complete cds
AT4G36850	2.20809825	2.36E-32	Arabidopsis thaliana PQ-loop repeat family protein / transmembrane family protein mRNA, complete cds
AT1G59620	1.171247845	2.41E-32	Arabidopsis thaliana protein CW9 mRNA, complete cds
AT3G30210	5.441717927	2.74E-32	Arabidopsis thaliana myb domain protein 121 mRNA, complete cds
AT3G55580	2.628430049	3.30E-32	Arabidopsis thaliana regulator of chromosome condensation repeat-containing protein mRNA, complete cds
AT1G43650	1.33319347	3.34E-32	Arabidopsis thaliana nodulin MtN21 /EamA-like transporter protein mRNA, complete cds
AT2G41170	1.249768761	5.11E-32	Arabidopsis thaliana F-box protein mRNA, complete cds
AT3G13672	3.237845594	5.68E-32	Arabidopsis thaliana seven in absentia (SINA) domain-containing protein mRNA,

			complete cds
AT4G11360	1.431538761	6.18E-32	Arabidopsis thaliana chromosome 4 sequence
AT1G14550	3.294312545	7.01E-32	Arabidopsis thaliana peroxidase 5 mRNA, complete cds
AT4G24340	2.319233921	7.98E-32	Arabidopsis thaliana phosphorylase family protein mRNA, complete cds
AT3G55240	3.668895411	8.69E-32	Arabidopsis thaliana uncharacterized protein mRNA, complete cds
AT4G37220	3.668895411	8.69E-32	Arabidopsis thaliana cold acclimation protein WCOR413 mRNA, complete cds
AT5G40790	8.195362262	9.94E-32	Arabidopsis thaliana chromosome 5 sequence
AT1G07630	1.004646417	1.02E-31	Arabidopsis thaliana putative protein phosphatase 2C mRNA, complete cds
AT3G55970	5.015453172	1.38E-31	Arabidopsis thaliana jasmonate-regulated protein JRG21 mRNA, complete cds
AT3G43430	1.811684716	1.43E-31	Arabidopsis thaliana chromosome 3, complete sequence
AT4G13180	1.274758994	1.55E-31	Arabidopsis thaliana chromosome 4 sequence
AT4G18880	1.305883114	1.57E-31	Arabidopsis thaliana heat stress transcription factor A-4a mRNA, complete cds
AT1G05680	4.715892891	1.79E-31	Arabidopsis thaliana Uridine diphosphate glycosyltransferase 74E2 mRNA, complete cds
AT3G49760	3.220567602	1.80E-31	Arabidopsis thaliana chromosome 3, complete sequence
AT2G35760	1.422078432	1.94E-31	Arabidopsis thaliana CASP-like protein mRNA, complete cds
AT2G43320	1.343198594	2.03E-31	Arabidopsis thaliana S-adenosyl-L-methionine-dependent methyltransferase-like protein mRNA, complete cds
AT4G08570	3.484222656	2.15E-31	Arabidopsis thaliana heavy-metal-associated domain-containing protein mRNA, complete cds
AT4G15260	1.50088	2.17E-31	Arabidopsis thaliana chromosome 4 sequence
AT1G34630	1.098414621	2.67E-31	Arabidopsis thaliana uncharacterized protein mRNA, complete cds
AT2G41475	1.030883213	3.25E-31	Arabidopsis thaliana PLAT domain-containing protein mRNA, complete cds
AT1G30640	1.361476943	3.47E-31	Arabidopsis thaliana protein kinase family protein mRNA, complete cds
AT2G04570	1.183575931	3.84E-31	Arabidopsis thaliana GDSL esterase/lipase mRNA, complete cds

AT5G13190	1.056095157	4.23E-31	Arabidopsis thaliana GSH-induced LITAF domain protein mRNA, complete cds
AT1G35210	2.770340675	5.13E-31	Arabidopsis thaliana chromosome 1 sequence
AT4G09750	1.680468925	5.20E-31	Arabidopsis thaliana mRNA for hypothetical protein, complete cds, clone: RAFL14-08-C18
AT2G41660	1.476587086	5.91E-31	Arabidopsis thaliana chromosome 2, complete sequence
AT5G19340	1.501585646	1.24E-30	Arabidopsis thaliana chromosome 5 sequence
AT1G50260	1.240485476	1.31E-30	Arabidopsis thaliana N-terminal-transmembrane-C2 domain type 5.1 protein mRNA, complete cds
AT1G74360	1.616226188	1.33E-30	Arabidopsis thaliana putative LRR receptor-like serine/threonine-protein kinase mRNA, complete cds
AT1G74080	4.969649483	1.40E-30	Arabidopsis thaliana myb domain protein 122 mRNA, complete cds
AT1G09180	3.534181975	1.45E-30	Arabidopsis thaliana secretion-associated RAS 1 protein mRNA, complete cds
AT1G07590	1.434909343	1.60E-30	Arabidopsis thaliana pentatricopeptide repeat-containing protein mRNA, complete cds
AT5G53050	1.270267071	1.71E-30	Arabidopsis thaliana hydrolase, alpha/beta fold family protein mRNA, complete cds
AT5G62460	1.021274411	1.71E-30	Arabidopsis thaliana RING/FYVE/PHD zinc finger-containing protein mRNA, complete cds
AT4G28290	1.510217864	1.89E-30	Arabidopsis thaliana uncharacterized protein mRNA, complete cds
AT1G75590	3.52446682	2.61E-30	Arabidopsis thaliana chromosome 1 sequence
AT1G75490	3.705768673	3.38E-30	Arabidopsis thaliana chromosome 1 sequence
AT4G33950	1.372223017	3.51E-30	Arabidopsis thaliana calcium-independent ABA-activated protein kinase mRNA, complete cds
AT1G54740	1.555677358	4.33E-30	Arabidopsis thaliana chromosome 1 sequence
AT2G34930	1.281886155	4.52E-30	Arabidopsis thaliana chromosome 2, complete sequence
AT1G21460	1.369090127	5.08E-30	Arabidopsis thaliana bidirectional sugar transporter SWEET1 mRNA, complete cds
AT4G14746	1.160736668	5.29E-30	Arabidopsis thaliana uncharacterized protein mRNA, complete cds
AT3G46230	2.585768897	6.20E-30	Arabidopsis thaliana chromosome 3, complete sequence
AT2G14820	2.004646417	7.21E-30	Arabidopsis thaliana BTB/POZ

			domain-containing protein NPY2 mRNA, complete cds
AT3G49620	3.922343768	7.40E-30	Arabidopsis thaliana 2-oxoacid-dependent dioxygenase-like protein DIN11 mRNA, complete cds
AT1G51170	1.275096989	7.75E-30	Arabidopsis thaliana chromosome 1 sequence
AT1G11950	1.604417604	7.85E-30	Arabidopsis thaliana transcription factor jumonji (jmjC) domain-containing protein mRNA, complete cds
AT1G21520	2.191530401	1.41E-29	Arabidopsis thaliana uncharacterized protein mRNA, complete cds
AT2G32930	1.209224916	1.44E-29	Arabidopsis thaliana zinc finger nuclease 2 mRNA, complete cds
AT1G32350	5.885817892	1.59E-29	Arabidopsis thaliana alternative oxidase 1D mRNA, complete cds
AT3G23240	5.885817892	1.59E-29	Arabidopsis thaliana chromosome 3, complete sequence
AT2G29090	2.822808094	2.26E-29	Arabidopsis thaliana abscisic acid 8'-hydroxylase 2 mRNA, complete cds
AT1G75900	1.429348847	2.29E-29	Arabidopsis thaliana GDSL esterase/lipase EXL3 mRNA, complete cds
AT1G77640	3.407770595	2.32E-29	Arabidopsis thaliana chromosome 1 sequence
AT4G34060	1.787647255	2.67E-29	Arabidopsis thaliana DEMETER-like protein 3 mRNA, complete cds
AT4G31950	6.922343768	2.92E-29	Arabidopsis thaliana cytochrome P450, family 82, subfamily C, polypeptide 3 mRNA, complete cds
AT5G04250	1.808356938	3.46E-29	Arabidopsis thaliana OTU-like cysteine protease family protein mRNA, complete cds
AT2G20560	1.009462735	4.92E-29	Arabidopsis thaliana DNAJ heat shock family protein mRNA, complete cds
AT1G30620	1.337381267	5.07E-29	Arabidopsis thaliana UDP-arabinose 4-epimerase 1 mRNA, complete cds
AT1G72240	3.13093039	5.66E-29	Arabidopsis thaliana chromosome 1 sequence
AT3G06760	1.011428916	6.23E-29	Arabidopsis thaliana protein dehydration-INDUCED 19-4 mRNA, complete cds
AT1G25520	1.128123199	6.42E-29	Arabidopsis thaliana putative transmembrane protein mRNA, complete cds
AT5G17490	2.3652603	6.85E-29	Arabidopsis thaliana chromosome 5 sequence
AT1G44830	2.699951347	7.15E-29	Arabidopsis thaliana chromosome 1 sequence
AT1G74870	4.015453172	8.64E-29	Arabidopsis thaliana RING-finger domain-containing protein mRNA, complete cds

AT1G18100	3.464760574	8.84E-29	Arabidopsis thaliana protein MOTHER of FT and TF 1 mRNA, complete cds
AT5G44060	1.205784385	1.10E-28	Arabidopsis thaliana chromosome 5 sequence
AT3G03440	1.755586046	1.29E-28	Arabidopsis thaliana ARM repeat superfamily protein mRNA, complete cds
AT3G55090	1.793759562	1.31E-28	Arabidopsis thaliana chromosome 3, complete sequence
AT3G26280	1.549514775	1.62E-28	Arabidopsis thaliana cytochrome P450 71B4 mRNA, complete cds
AT2G45570	3.112314712	1.78E-28	Arabidopsis thaliana cytochrome P450 76C2 mRNA, complete cds
AT4G39830	1.955896328	1.86E-28	Arabidopsis thaliana putative L-ascorbate oxidase mRNA, complete cds
AT3G11020	2.997305826	3.25E-28	Arabidopsis thaliana dehydration-responsive element-binding protein 2B mRNA, complete cds
AT4G33930	6.860943223	3.34E-28	Arabidopsis thaliana chromosome 4 sequence
AT2G20670	1.268980493	3.35E-28	Arabidopsis thaliana uncharacterized protein mRNA, complete cds
AT3G52450	1.490232755	3.89E-28	Arabidopsis thaliana chromosome 3, complete sequence
AT1G49500	1.129273072	4.26E-28	Arabidopsis thaliana uncharacterized protein mRNA, complete cds
AT1G29330	1.293360855	4.28E-28	Arabidopsis thaliana ER lumen protein retaining receptor mRNA, complete cds
AT2G32030	2.703509166	5.31E-28	Arabidopsis thaliana chromosome 2, complete sequence
AT3G62690	1.452858485	5.81E-28	Arabidopsis thaliana chromosome 3, complete sequence
AT4G01550	1.754038862	5.82E-28	Arabidopsis thaliana NAC transcription factor mRNA, complete cds
AT5G05220	7.969649483	5.88E-28	Arabidopsis thaliana chromosome 5 sequence
AT5G67340	1.388439868	6.34E-28	Arabidopsis thaliana ARM repeat superfamily protein mRNA, complete cds
AT1G80130	1.939217587	7.51E-28	Arabidopsis thaliana tetratricopeptide repeat domain-containing protein mRNA, complete cds
AT4G12580	3.715892891	7.74E-28	Arabidopsis thaliana chromosome 4 sequence
AT1G16150	1.830613497	8.98E-28	Arabidopsis thaliana wall associated kinase-like 4 mRNA, complete cds
AT1G78210	1.028509325	9.77E-28	Arabidopsis thaliana alpha/beta-Hydrolases superfamily protein mRNA, complete cds
AT1G78340	2.413330121	1.13E-27	Arabidopsis thaliana glutathione S-transferase

			TAU 22 mRNA, complete cds
AT3G47295	1.93361488	1.19E-27	Arabidopsis thaliana uncharacterized protein mRNA, complete cds
AT4G23220	1.262246937	1.34E-27	Arabidopsis thaliana cysteine-rich receptor-like protein kinase 14 mRNA, complete cds
AT1G52880	1.636598009	1.35E-27	Arabidopsis thaliana NAC domain-containing protein 18 mRNA, complete cds
AT2G45600	1.783007086	1.44E-27	Arabidopsis thaliana chromosome 2, complete sequence
AT5G01880	2.561102749	1.71E-27	Arabidopsis thaliana chromosome 5 sequence
AT5G13320	2.033676931	1.75E-27	Arabidopsis thaliana 4-substituted benzoates-glutamate ligase GH3.12 mRNA, complete cds
AT3G56790	5.770340675	2.17E-27	Arabidopsis thaliana chromosome 3, complete sequence
AT5G59130	1.820196881	2.20E-27	Arabidopsis thaliana Subtilase family protein mRNA, complete cds
AT3G62090	1.752418767	2.62E-27	Arabidopsis thaliana transcription factor PIF6 mRNA, complete cds
AT5G24870	1.050051834	2.66E-27	Arabidopsis thaliana RING/U-box superfamily protein mRNA, complete cds
AT4G15120	2.422956999	2.95E-27	Arabidopsis thaliana chromosome 4 sequence
AT4G18530	1.765933573	3.16E-27	Arabidopsis thaliana uncharacterized protein mRNA, complete cds
AT1G16410	1.243894638	4.58E-27	Arabidopsis thaliana dihomomethionine N-hydroxylase mRNA, complete cds
AT4G31870	2.249508511	4.72E-27	Arabidopsis thaliana glutathione peroxidase 7 mRNA, complete cds
AT2G39420	1.00899815	4.85E-27	Arabidopsis thaliana alpha/beta-Hydrolases superfamily protein mRNA, complete cds
AT1G07480	1.004774404	5.13E-27	Arabidopsis thaliana transcription factor IIA, alpha/beta subunit mRNA, complete cds
AT1G16370	2.51529906	5.61E-27	Arabidopsis thaliana chromosome 1 sequence
AT3G13784	2.486758891	6.28E-27	Arabidopsis thaliana beta-fructofuranosidase, insoluble isoenzyme CWINV5 mRNA, complete cds
AT3G25730	2.040544153	7.76E-27	Arabidopsis thaliana chromosome 3, complete sequence
AT4G39190	1.509100506	8.88E-27	Arabidopsis thaliana chromosome 4 sequence
AT3G09910	2.425328967	1.29E-26	Arabidopsis thaliana RAB GTPase-like protein C2B mRNA, complete cds
AT3G47640	1.160132925	1.34E-26	Arabidopsis thaliana bHLH transcription

			factor POPEYE mRNA, complete cds
AT4G37900	1.534263338	1.36E-26	Arabidopsis thaliana uncharacterized protein mRNA, complete cds
AT1G48840	1.164075788	1.37E-26	Arabidopsis thaliana uncharacterized protein mRNA, complete cds
AT3G05200	1.267747079	1.43E-26	Arabidopsis thaliana chromosome 3, complete sequence
AT3G06780	1.604945332	1.45E-26	Arabidopsis thaliana chromosome 3, complete sequence
AT5G35450	1.08410536	1.88E-26	Arabidopsis thaliana disease resistance RPP8-like protein 3 mRNA, complete cds
AT5G67140	1.410591114	1.97E-26	Arabidopsis thaliana F-box/RNI-like superfamily protein mRNA, complete cds
AT1G09350	3.452858485	2.03E-26	Arabidopsis thaliana galactinol synthase 3 mRNA, complete cds
AT1G56020	1.72092848	2.08E-26	Arabidopsis thaliana chromosome 1 sequence
AT5G15190	3.087861668	2.23E-26	Arabidopsis thaliana chromosome 5 sequence
AT2G39110	1.582659202	2.26E-26	Arabidopsis thaliana protein kinase superfamily protein mRNA, complete cds
AT3G44870	4.756920159	2.83E-26	Arabidopsis thaliana S-adenosyl-L-methionine-dependent methyltransferase mRNA, complete cds
AT5G48530	1.140489218	3.33E-26	Arabidopsis thaliana uncharacterized protein mRNA, complete cds
AT1G29640	2.543190804	3.69E-26	Arabidopsis thaliana chromosome 1 sequence
AT1G42980	4.237845594	3.87E-26	Arabidopsis thaliana formin-like protein 12 mRNA, complete cds
AT4G36950	7.848343187	4.12E-26	Arabidopsis thaliana chromosome 4 sequence
AT1G19650	1.00007014	4.51E-26	Arabidopsis thaliana Sec14p-like phosphatidylinositol transfer family protein mRNA, complete cds
AT5G07310	6.72969869	4.63E-26	Arabidopsis thaliana ethylene-responsive transcription factor ERF115 mRNA, complete cds
AT1G07135	2.600415673	4.80E-26	Arabidopsis thaliana chromosome 1 sequence
AT3G51750	2.041448381	5.55E-26	Arabidopsis thaliana uncharacterized protein mRNA, complete cds
AT4G06534	2.214233037	5.80E-26	Arabidopsis thaliana chromosome 4 sequence
AT1G06620	1.29268146	7.17E-26	Arabidopsis thaliana 2-oxoglutarate-dependent dioxygenase-like protein mRNA, complete cds
AT1G77890	1.158647808	8.69E-26	Arabidopsis thaliana DNA-directed RNA polymerase II protein mRNA, complete cds

AT5G13370	1.275842857	9.06E-26	Arabidopsis thaliana auxin-responsive GH3 family protein mRNA, complete cds
AT2G40970	1.258525847	9.52E-26	Arabidopsis thaliana chromosome 2, complete sequence
AT1G59640	2.076564687	9.60E-26	Arabidopsis thaliana transcription factor BPE mRNA, complete cds
AT5G21960	7.822808094	9.73E-26	Arabidopsis thaliana chromosome 5 sequence
AT1G80120	2.121842517	9.92E-26	Arabidopsis thaliana uncharacterized protein mRNA, complete cds
AT3G10820	1.254110648	1.14E-25	Arabidopsis thaliana probable mediator of RNA polymerase II transcription subunit 26a mRNA, complete cds
AT3G60140	1.842985976	1.51E-25	Arabidopsis thaliana beta-glucosidase 30 mRNA, complete cds
AT1G26450	1.256461272	1.59E-25	Arabidopsis thaliana carbohydrate-binding X8 domain-containing protein mRNA, complete cds
AT2G26660	1.13093039	2.04E-25	Arabidopsis thaliana SPX domain-containing protein 2 mRNA, complete cds
AT5G59580	4.198674996	2.31E-25	Arabidopsis thaliana UDP-glucosyl transferase 76E1 mRNA, complete cds
AT2G27310	1.40098143	2.40E-25	Arabidopsis thaliana chromosome 2, complete sequence
AT5G45820	3.102916014	2.84E-25	Arabidopsis thaliana chromosome 5 sequence
AT4G23140	1.559773689	2.97E-25	Arabidopsis thaliana cysteine-rich receptor-like protein kinase 6 mRNA, complete cds
AT4G11070	2.706615077	3.75E-25	Arabidopsis thaliana putative WRKY transcription factor 41 mRNA, complete cds
AT1G53540	3.396274956	3.79E-25	Arabidopsis thaliana chromosome 1 sequence
AT5G64230	2.263380686	3.85E-25	Arabidopsis thaliana uncharacterized protein mRNA, complete cds
AT2G32680	1.44146179	4.05E-25	Arabidopsis thaliana chromosome 2, complete sequence
AT1G70590	1.045615143	5.45E-25	Arabidopsis thaliana F-box protein mRNA, complete cds
AT4G29110	2.276945413	6.14E-25	Arabidopsis thaliana chromosome 4 sequence
AT4G15233	1.321160915	6.85E-25	Arabidopsis thaliana ABC transporter G family member 42 mRNA, complete cds
AT1G58280	1.321160915	6.85E-25	Arabidopsis thaliana phosphoglycerate mutase-like protein mRNA, complete cds
AT3G51890	1.071306407	8.38E-25	Arabidopsis thaliana Clathrin light chain protein mRNA, complete cds

AT5G28830	1.525510864	8.69E-25	Arabidopsis thaliana calcium-binding EF hand family protein mRNA, complete cds
AT5G38240	2.618794202	9.04E-25	Arabidopsis thaliana Protein kinase family protein mRNA, complete cds
AT1G49520	1.42369511	9.46E-25	Arabidopsis thaliana SWIB complex BAF60b domain-containing protein mRNA, complete cds
AT1G15330	4.673664655	9.63E-25	Arabidopsis thaliana Cystathionine beta-synthase (CBS) protein mRNA, complete cds
AT5G57910	1.113398223	9.77E-25	Arabidopsis thaliana uncharacterized protein mRNA, complete cds
AT2G34850	2.433305687	1.09E-24	Arabidopsis thaliana putative UDP-arabinose 4-epimerase 2 mRNA, complete cds
AT3G26760	1.550110591	1.14E-24	Arabidopsis thaliana Rossmann-fold NAD(P)-binding domain-containing protein mRNA, complete cds
AT1G18830	3.822808094	1.17E-24	Arabidopsis thaliana transport protein SEC31-like protein SEC31B mRNA, complete cds
AT3G03870	1.01066811	1.47E-24	Arabidopsis thaliana uncharacterized protein mRNA, complete cds
AT4G11660	1.049035773	1.65E-24	Arabidopsis thaliana heat stress transcription factor B-2b mRNA, complete cds
AT3G03480	2.128515837	1.89E-24	Arabidopsis thaliana acetyl CoA:(Z)-3-hexen-1-ol acetyltransferase mRNA, complete cds
AT3G50760	1.940165045	2.07E-24	Arabidopsis thaliana chromosome 3, complete sequence
AT1G02880	1.025402833	2.48E-24	Arabidopsis thaliana thiamin pyrophosphokinase1 mRNA, complete cds
AT1G07985	4.144736189	2.50E-24	Arabidopsis thaliana chromosome 1 sequence
AT2G40435	4.144736189	2.50E-24	Arabidopsis thaliana uncharacterized protein mRNA, complete cds
AT5G56210	1.105018557	2.53E-24	Arabidopsis thaliana WPP domain-interacting protein 2 mRNA, complete cds
AT1G52830	4.36595042	2.72E-24	Arabidopsis thaliana indole-3-acetic acid 6 mRNA, complete cds
AT3G09375	5.585308781	2.88E-24	Arabidopsis thaliana chromosome 3, complete sequence
AT2G22880	3.551506073	3.03E-24	Arabidopsis thaliana chromosome 2, complete sequence
AT1G72120	1.844181745	3.07E-24	Arabidopsis thaliana putative peptide/nitrate transporter mRNA, complete cds

AT3G20340	1.853702102	3.31E-24	Arabidopsis thaliana chromosome 3, complete sequence
AT1G06180	1.39315452	3.40E-24	Arabidopsis thaliana myb domain protein 13 mRNA, complete cds
AT3G47210	1.294525788	3.61E-24	Arabidopsis thaliana uncharacterized protein mRNA, complete cds
AT4G15610	1.140439949	5.62E-24	Arabidopsis thaliana uncharacterized protein mRNA, complete cds
AT1G77000	1.472660126	5.93E-24	Arabidopsis thaliana F-box protein SKP2B mRNA, complete cds
AT5G49120	2.520981206	5.93E-24	Arabidopsis thaliana uncharacterized protein mRNA, complete cds
AT1G28190	1.518731876	6.49E-24	Arabidopsis thaliana chromosome 1 sequence
AT1G54830	1.114163033	6.50E-24	Arabidopsis thaliana chromosome 1 sequence
AT1G62975	2.259378755	7.43E-24	Arabidopsis thaliana transcription factor bHLH125 mRNA, complete cds
AT3G13100	1.162962424	8.87E-24	Arabidopsis thaliana ABC transporter C family member 7 mRNA, complete cds
AT5G24030	1.020524162	1.09E-23	Arabidopsis thaliana SLAC1 homologue 3 mRNA, complete cds
AT4G28640	1.337381267	1.16E-23	Arabidopsis thaliana auxin-responsive protein IAA11 mRNA, complete cds
AT2G20340	1.146843572	1.16E-23	Arabidopsis thaliana tyrosine decarboxylase 1 mRNA, complete cds
AT3G22275	7.659309362	1.82E-23	Arabidopsis thaliana uncharacterized protein mRNA, complete cds
AT4G35110	1.343338723	1.87E-23	Arabidopsis thaliana phospholipase-like (PEARLI 4) family protein mRNA, complete cds
AT5G22300	2.108562577	2.19E-23	Arabidopsis thaliana bifunctional nitrilase/nitrile hydratase NIT4 mRNA, complete cds
AT4G15480	2.562940968	2.28E-23	Arabidopsis thaliana chromosome 4 sequence
AT3G21700	1.106218636	2.39E-23	Arabidopsis thaliana monomeric G protein SGP2 mRNA, complete cds
AT4G21440	2.527352211	2.59E-23	Arabidopsis thaliana R2R3 family MYB transcription factor mRNA, complete cds
AT3G15760	1.430490672	2.88E-23	Arabidopsis thaliana uncharacterized protein mRNA, complete cds
AT5G38700	4.585308781	3.32E-23	Arabidopsis thaliana chromosome 5 sequence
AT3G55940	2.101028904	3.57E-23	Arabidopsis thaliana phosphoinositide phospholipase C 7 mRNA, complete cds
AT4G33040	2.173601006	3.57E-23	Arabidopsis thaliana chromosome 4 sequence

AT1G66830	3.613712496	3.67E-23	Arabidopsis thaliana probable inactive leucine-rich repeat receptor-like protein kinase mRNA, complete cds
AT1G75230	1.225124353	3.86E-23	Arabidopsis thaliana DNA glycosylase superfamily protein mRNA, complete cds
AT4G14550	1.46458387	4.35E-23	Arabidopsis thaliana auxin-responsive protein IAA14 mRNA, complete cds
AT5G40540	1.204214816	4.55E-23	Arabidopsis thaliana protein kinase family protein mRNA, complete cds
AT4G33467	5.507306269	4.73E-23	Arabidopsis thaliana uncharacterized protein mRNA, complete cds
AT5G37990	1.179612328	5.17E-23	Arabidopsis thaliana probable S-adenosylmethionine-dependent methyltransferase mRNA, complete cds
AT1G04180	1.483132151	5.25E-23	Arabidopsis thaliana flavin-containing monooxygenase YUCCA9 mRNA, complete cds
AT3G25510	2.128347229	5.90E-23	Arabidopsis thaliana putative TIR-NBS-LRR class disease resistance protein mRNA, complete cds
AT4G26200	2.695572906	6.21E-23	Arabidopsis thaliana 1-aminocyclopropane-1-carboxylate synthase 7 mRNA, complete cds
AT4G35480	1.600415673	6.99E-23	Arabidopsis thaliana chromosome 4 sequence
AT3G45970	1.448869201	7.05E-23	Arabidopsis thaliana expansin-like A1 mRNA, complete cds
AT1G61370	1.584818818	8.19E-23	Arabidopsis thaliana S-locus lectin protein kinase family protein mRNA, complete cds
AT5G42630	1.695835238	9.04E-23	Arabidopsis thaliana probable transcription factor KAN4 mRNA, complete cds
AT2G47260	1.269498795	1.00E-22	Arabidopsis thaliana WRKY DNA-binding protein 23 mRNA, complete cds
AT1G63820	3.051077082	1.12E-22	Arabidopsis thaliana CCT motif family protein mRNA, complete cds
AT4G09890	1.077994638	1.39E-22	Arabidopsis thaliana chromosome 4 sequence
AT3G53650	1.697033863	1.55E-22	Arabidopsis thaliana chromosome 3, complete sequence
AT1G45976	1.029102234	1.83E-22	Arabidopsis thaliana S-ribonuclease binding protein 1 mRNA, complete cds
AT1G64065	1.952868635	1.87E-22	Arabidopsis thaliana chromosome 1 sequence
AT5G16600	1.261965122	2.02E-22	Arabidopsis thaliana myb domain protein 43 mRNA, complete cds
AT1G67000	3.263380686	2.30E-22	Arabidopsis thaliana probable receptor-like protein kinase mRNA, complete cds

AT1G65390	1.605870103	2.32E-22	Arabidopsis thaliana protein PHLOEM protein 2-LIKE A5 mRNA, complete cds
AT5G28610	2.456025764	2.41E-22	Arabidopsis thaliana chromosome 5 sequence
AT3G62740	1.250358205	2.45E-22	Arabidopsis thaliana beta glucosidase 7 mRNA, complete cds
AT2G29480	4.906224103	2.47E-22	Arabidopsis thaliana glutathione S-transferase tau 2 mRNA, complete cds
AT5G28520	7.570042024	2.61E-22	Arabidopsis thaliana mannose-binding lectin-like protein mRNA, complete cds
AT2G18050	2.393964796	2.87E-22	Arabidopsis thaliana histone H1-3 mRNA, complete cds
AT2G43870	4.248113929	3.18E-22	Arabidopsis thaliana putative polygalacturonase /pectinase mRNA, complete cds
AT5G03490	1.374913324	3.42E-22	Arabidopsis thaliana chromosome 5 sequence
AT1G61290	6.458396668	4.14E-22	Arabidopsis thaliana syntaxin-124 mRNA, complete cds
AT2G44578	5.441717927	4.50E-22	Arabidopsis thaliana chromosome 2, complete sequence
AT1G51470	1.672565459	4.99E-22	Arabidopsis thaliana myrosinase 5 mRNA, complete cds
AT1G33480	1.175917845	5.57E-22	Arabidopsis thaliana RING-H2 finger protein ATL58 mRNA, complete cds
AT3G48450	2.470019036	6.24E-22	Arabidopsis thaliana RPM1-interacting protein 4 (RIN4) mRNA, complete cds
AT4G39670	2.229232463	6.51E-22	Arabidopsis thaliana chromosome 4 sequence
AT5G22250	2.089034305	6.77E-22	Arabidopsis thaliana chromosome 5 sequence
AT1G02220	1.37549664	6.79E-22	Arabidopsis thaliana NAC domain-containing protein 3 mRNA, complete cds
AT3G56710	1.729892198	6.85E-22	Arabidopsis thaliana chromosome 3, complete sequence
AT4G15100	6.441717927	6.92E-22	Arabidopsis thaliana serine carboxypeptidase-like 30 mRNA, complete cds
AT1G07500	5.424844109	7.92E-22	Arabidopsis thaliana uncharacterized protein mRNA, complete cds
AT1G64810	1.026680428	8.52E-22	Arabidopsis thaliana APO protein 1 mRNA, complete cds
AT1G69890	1.229128377	9.83E-22	Arabidopsis thaliana uncharacterized protein mRNA, complete cds
AT2G27000	1.217087034	9.85E-22	Arabidopsis thaliana cytochrome P450, family 705, subfamily A, polypeptide 8 mRNA, complete cds

AT2G14290	7.523247813	1.00E-21	Arabidopsis thaliana chromosome 2, complete sequence
AT1G52030	1.242957008	1.15E-21	Arabidopsis thaliana myrosinase-binding protein 2 mRNA, complete cds
AT5G53420	1.212310502	1.17E-21	Arabidopsis thaliana CCT motif family protein mRNA, complete cds
AT2G31130	1.26436647	1.31E-21	Arabidopsis thaliana uncharacterized protein mRNA, complete cds
AT4G32280	1.44841258	1.37E-21	Arabidopsis thaliana auxin-responsive protein IAA29 mRNA, complete cds
AT1G74590	1.567994195	1.41E-21	Arabidopsis thaliana glutathione S-transferase TAU 10 mRNA, complete cds
AT5G57100	1.128731159	1.48E-21	Arabidopsis thaliana nucleotide/sugar transporter family protein mRNA, complete cds
AT5G53710	7.507306269	1.57E-21	Arabidopsis thaliana chromosome 5 sequence
AT1G52080	1.483770754	1.74E-21	Arabidopsis thaliana uncharacterized protein mRNA, complete cds
AT4G08040	2.211850385	1.78E-21	Arabidopsis thaliana 1-aminocyclopropane-1-carboxylate synthase 11 mRNA, complete cds
AT4G36740	3.407770595	1.95E-21	Arabidopsis thaliana homeobox protein 40 mRNA, complete cds
AT1G67810	1.873434168	2.51E-21	Arabidopsis thaliana chromosome 1 sequence
AT1G67110	1.346232185	2.68E-21	Arabidopsis thaliana cytokinin hydroxylase mRNA, complete cds
AT5G11210	3.969649483	3.19E-21	Arabidopsis thaliana glutamate receptor 2.5 mRNA, complete cds
AT4G05110	1.843972704	3.42E-21	Arabidopsis thaliana equilibrative nucleoside transporter 6 mRNA, complete cds
AT4G14450	4.185378174	3.45E-21	Arabidopsis thaliana chromosome 4 sequence
AT1G64110	4.458396668	3.79E-21	Arabidopsis thaliana transcription factor DUO1 mRNA, complete cds
AT3G46110	1.546577522	4.16E-21	Arabidopsis thaliana uncharacterized protein mRNA, complete cds
AT5G55090	2.023445963	4.44E-21	Arabidopsis thaliana chromosome 5 sequence
AT1G09950	3.630163017	4.73E-21	Arabidopsis thaliana chromosome 1 sequence
AT2G31020	1.087439285	4.93E-21	Arabidopsis thaliana oxysterol binding protein-related protein 1A mRNA, complete cds
AT1G73260	1.556528029	5.44E-21	Arabidopsis thaliana chromosome 1 sequence
AT5G12030	1.779640235	5.50E-21	Arabidopsis thaliana chromosome 5 sequence
AT5G55970	1.156497054	5.69E-21	Arabidopsis thaliana RING/U-box

			domain-containing protein mRNA, complete cds
AT1G63750	1.162294561	6.91E-21	Arabidopsis thaliana TIR-NBS-LRR class disease resistance protein mRNA, complete cds
AT4G37850	3.489384361	7.43E-21	Arabidopsis thaliana transcription factor bHLH25 mRNA, complete cds
AT1G59865	3.489384361	7.43E-21	Arabidopsis thaliana uncharacterized protein mRNA, complete cds
AT1G12030	5.355303175	7.65E-21	Arabidopsis thaliana uncharacterized protein mRNA, complete cds
AT5G56180	1.173556465	8.50E-21	Arabidopsis thaliana actin-related protein 8 mRNA, complete cds
AT5G05965	7.441717927	9.62E-21	Arabidopsis thaliana uncharacterized protein mRNA, complete cds
AT4G11780	1.912883439	1.38E-20	Arabidopsis thaliana uncharacterized protein mRNA, complete cds
AT3G51430	1.060043323	1.43E-20	Arabidopsis thaliana strictosidine synthase-like 5 mRNA, complete cds
AT4G38940	1.77550238	1.46E-20	Arabidopsis thaliana chromosome 4 sequence
AT4G37710	7.424844109	1.52E-20	Arabidopsis thaliana chromosome 4 sequence
AT3G45660	2.941452591	1.84E-20	Arabidopsis thaliana probable nitrate excretion transporter 2 mRNA, complete cds
AT4G15990	2.189950904	2.12E-20	Arabidopsis thaliana chromosome 4 sequence
AT4G12735	7.407770595	2.40E-20	Arabidopsis thaliana chromosome 4 sequence
AT2G18340	7.407770595	2.40E-20	Arabidopsis thaliana late embryogenesis abundant domain-containing protein mRNA, complete cds
AT2G34610	3.460238015	2.41E-20	Arabidopsis thaliana chromosome 2, complete sequence
AT1G15580	2.496579862	3.12E-20	Arabidopsis thaliana auxin-responsive protein IAA5 mRNA, complete cds
AT1G01250	4.119789832	3.75E-20	Arabidopsis thaliana chromosome 1 sequence
AT4G30290	1.068402052	3.79E-20	Arabidopsis thaliana xyloglucan endotransglucosylase/hydrolase 19 mRNA, complete cds
AT4G14080	7.390492604	3.79E-20	Arabidopsis thaliana putative glucan endo-1,3-beta-glucosidase A6 mRNA, complete cds
AT3G48390	2.389848687	4.13E-20	Arabidopsis thaliana MA3 domain-containing protein mRNA, complete cds
AT5G16200	1.600415673	4.48E-20	Arabidopsis thaliana chromosome 5 sequence
AT3G03900	1.359040148	4.67E-20	Arabidopsis thaliana

			adenosine-5'-phosphosulfate (APS) kinase 3 mRNA, complete cds
AT4G39700	2.525314218	4.72E-20	Arabidopsis thaliana heavy metal transport/detoxification domain-containing protein mRNA, complete cds
AT5G43260	1.177724601	5.62E-20	Arabidopsis thaliana chromosome 5 sequence
AT2G47950	3.715892891	5.70E-20	Arabidopsis thaliana chromosome 2, complete sequence
AT4G30430	4.102916014	6.81E-20	Arabidopsis thaliana tetraspanin9 mRNA, complete cds
AT1G52827	1.944680098	6.94E-20	Arabidopsis thaliana cadmium tolerance 1 mRNA, complete cds
AT4G01080	1.72969869	6.96E-20	Arabidopsis thaliana protein TRICHOME BIREFRINGENCE-LIKE 26 mRNA, complete cds
AT5G35580	1.29748854	7.23E-20	Arabidopsis thaliana Ser/Thr protein kinase ACIK1b mRNA, complete cds
AT5G01760	3.13093039	7.45E-20	Arabidopsis thaliana ENTH/VHS/GAT family protein mRNA, complete cds
AT2G35950	2.976282575	7.69E-20	Arabidopsis thaliana protein EMBRYO SAC DEVELOPMENT ARREST 12 mRNA, complete cds
AT2G20870	1.990649781	7.98E-20	Arabidopsis thaliana chromosome 2, complete sequence
AT1G18390	1.222852879	8.30E-20	Arabidopsis thaliana probable serine/threonine-protein kinase mRNA, complete cds
AT3G60550	2.078779108	9.22E-20	Arabidopsis thaliana cyclin p3;2 mRNA, complete cds
AT4G10390	1.67511304	9.67E-20	Arabidopsis thaliana probable receptor-like protein kinase mRNA, complete cds
AT2G41040	1.153710258	1.03E-19	Arabidopsis thaliana uncharacterized methyltransferase mRNA, complete cds
AT3G62720	1.003480531	1.08E-19	Arabidopsis thaliana chromosome 3, complete sequence
AT2G29380	4.0858425	1.24E-19	Arabidopsis thaliana highly ABA-induced PP2C protein 3 mRNA, complete cds
AT1G13480	1.982286308	1.28E-19	Arabidopsis thaliana uncharacterized protein mRNA, complete cds
AT1G72680	1.149938505	1.43E-19	Arabidopsis thaliana cinnamyl-alcohol dehydrogenase mRNA, complete cds
AT1G71400	1.012380338	1.55E-19	Arabidopsis thaliana chromosome 1 sequence
AT3G14560	1.146156864	1.98E-19	Arabidopsis thaliana chromosome 3, complete sequence

AT1G70420	1.854172265	2.01E-19	Arabidopsis thaliana chromosome 1 sequence
AT2G32210	3.856755426	2.07E-19	Arabidopsis thaliana uncharacterized protein mRNA, complete cds
AT1G57750	5.244271863	2.35E-19	Arabidopsis thaliana chromosome 1 sequence
AT2G47520	5.244271863	2.35E-19	Arabidopsis thaliana ethylene-responsive transcription factor ERF071 mRNA, complete cds
AT1G22510	1.70894013	2.56E-19	Arabidopsis thaliana C3HC4-type RING finger protein mRNA, complete cds
AT2G04110	1.198760386	2.71E-19	Arabidopsis thaliana chromosome 2, complete sequence
AT3G44400	1.052577198	2.98E-19	Arabidopsis thaliana TIR-NBS-LRR class disease resistance protein mRNA, complete cds
AT2G04070	3.015453172	3.18E-19	Arabidopsis thaliana MATE efflux family protein mRNA, complete cds
AT3G50840	1.504388785	3.46E-19	Arabidopsis thaliana phototropic-responsive NPH3 family protein mRNA, complete cds
AT3G02410	3.088702154	4.17E-19	Arabidopsis thaliana probable isoprenylcysteine alpha-carbonyl methylesterase ICMEL2 mRNA, complete cds
AT2G15480	1.355145547	5.31E-19	Arabidopsis thaliana UDP-glucosyl transferase 73B5 mRNA, complete cds
AT5G62080	7.282239713	6.06E-19	Arabidopsis thaliana protease inhibitor/seed storage/lipid transfer protein (LTP) family protein mRNA, complete cds
AT2G47460	3.651041746	6.13E-19	Arabidopsis thaliana transcription factor MYB12 mRNA, complete cds
AT2G28210	1.191610128	6.31E-19	Arabidopsis thaliana alpha carbonic anhydrase 2 mRNA, complete cds
AT4G39955	1.294876539	1.13E-18	Arabidopsis thaliana hydrolase, alpha/beta fold family protein mRNA, complete cds
AT5G17650	1.050386963	1.40E-18	Arabidopsis thaliana glycine/proline-rich protein mRNA, complete cds
AT1G17830	2.315848106	1.54E-18	Arabidopsis thaliana uncharacterized protein mRNA, complete cds
AT4G32295	1.480834057	1.68E-18	Arabidopsis thaliana uncharacterized protein mRNA, complete cds
AT1G58225	2.973224937	1.75E-18	Arabidopsis thaliana uncharacterized protein mRNA, complete cds
AT1G74660	1.697881483	1.78E-18	Arabidopsis thaliana chromosome 1 sequence
AT5G50120	1.544562438	1.78E-18	Arabidopsis thaliana chromosome 5 sequence
AT1G51440	1.145741202	2.18E-18	Arabidopsis thaliana chromosome 1 sequence

AT1G17750	1.460238015	2.75E-18	Arabidopsis thaliana leucine-rich repeat receptor-like protein kinase PEPR2 mRNA, complete cds
AT3G26840	1.083624675	2.87E-18	Arabidopsis thaliana phytyl ester synthesis and diacylglycerol acyltransferase activity protein mRNA, complete cds
AT4G21840	2.822808094	2.88E-18	Arabidopsis thaliana methionine sulfoxide reductase B8 mRNA, complete cds
AT4G16515	2.074346861	2.89E-18	Arabidopsis thaliana chromosome 4 sequence
AT3G26830	1.658421277	2.99E-18	Arabidopsis thaliana protein PHYTOALEXIN DEFICIENT 3 mRNA, complete cds
AT2G17660	4.620315231	3.00E-18	Arabidopsis thaliana chromosome 2, complete sequence
AT1G30040	1.504599886	3.42E-18	Arabidopsis thaliana gibberellin 2-beta-dioxygenase 2 mRNA, complete cds
AT1G20190	1.189704099	3.48E-18	Arabidopsis thaliana expansin-A11 mRNA, complete cds
AT5G39680	3.217087034	3.82E-18	Arabidopsis thaliana chromosome 5 sequence
AT3G04070	2.006175359	4.31E-18	Arabidopsis thaliana NAC domain containing protein 47 mRNA, complete cds
AT4G19460	2.20659466	4.73E-18	Arabidopsis thaliana chromosome 4 sequence
AT2G15490	4.244271863	4.82E-18	Arabidopsis thaliana mRNA for putative glucosyltransferase, complete cds, clone: RAFL14-26-J02
AT5G49920	4.600415673	5.42E-18	Arabidopsis thaliana octicosapeptide/Phox/Bem1p domain-containing protein mRNA, complete cds
AT5G52160	7.185378174	6.32E-18	Arabidopsis thaliana bifunctional inhibitor/lipid-transfer protein/seed storage 2S albumin-like protein mRNA, complete cds
AT5G10695	1.961681916	6.36E-18	Arabidopsis thaliana chromosome 5 sequence
AT2G34790	1.181039238	6.78E-18	Arabidopsis thaliana protein MATERNAL EFFECT EMBRYO ARREST 23 mRNA, complete cds
AT3G53450	3.201319718	6.82E-18	Arabidopsis thaliana cytokinin riboside 5'-monophosphate phosphoribohydrolase LOG4 mRNA, complete cds
AT1G69790	1.996837494	6.94E-18	Arabidopsis thaliana putative protein serine/threonine kinase mRNA, complete cds
AT1G24600	2.866309733	7.00E-18	Arabidopsis thaliana chromosome 1 sequence
AT4G33985	2.866309733	7.00E-18	Arabidopsis thaliana uncharacterized protein mRNA, complete cds

AT3G44735	1.048201755	7.82E-18	Arabidopsis thaliana Phytosulfokine 3 precursor mRNA, complete cds
AT2G16990	1.411381849	8.87E-18	Arabidopsis thaliana major facilitator protein mRNA, complete cds
AT4G35180	3.095180365	9.52E-18	Arabidopsis thaliana LYS/HIS transporter 7 mRNA, complete cds
AT5G56100	1.359407574	9.76E-18	Arabidopsis thaliana chromosome 5 sequence
AT1G69430	3.424844109	1.02E-17	Arabidopsis thaliana chromosome 1 sequence
AT2G33710	3.424844109	1.02E-17	Arabidopsis thaliana ethylene-responsive transcription factor ERF112 mRNA, complete cds
AT1G31290	2.212754613	1.26E-17	Arabidopsis thaliana argonaute 3 mRNA, complete cds
AT5G37490	5.102916014	1.33E-17	Arabidopsis thaliana chromosome 5 sequence
AT3G06420	1.048704978	1.33E-17	Arabidopsis thaliana autophagy-related protein 8h mRNA, complete cds
AT5G13880	1.926916498	1.54E-17	Arabidopsis thaliana chromosome 5 sequence
AT4G39580	2.914926296	1.68E-17	Arabidopsis thaliana chromosome 4 sequence
AT5G24470	1.4129323	1.76E-17	Arabidopsis thaliana pseudo-response regulator 5 mRNA, complete cds
AT5G62520	2.325793293	1.80E-17	Arabidopsis thaliana probable inactive poly [ADP-ribose] polymerase SRO5 mRNA, complete cds
AT1G67980	3.547948253	2.14E-17	Arabidopsis thaliana caffeoyl-CoA 3-O-methyltransferase mRNA, complete cds
AT3G08885	3.547948253	2.14E-17	Arabidopsis thaliana chromosome 3, complete sequence
AT1G19310	1.25781401	2.26E-17	Arabidopsis thaliana RING/U-box superfamily protein mRNA, complete cds
AT1G66540	1.202823308	2.31E-17	Arabidopsis thaliana cytochrome P450 superfamily protein mRNA, complete cds
AT5G63650	1.026233011	2.35E-17	Arabidopsis thaliana serine/threonine-protein kinase SRK2H mRNA, complete cds
AT3G09640	6.059847292	2.55E-17	Arabidopsis thaliana L-ascorbate peroxidase 2 mRNA, complete cds
AT5G51990	7.123977629	2.62E-17	Arabidopsis thaliana chromosome 5 sequence
AT4G23420	1.300855391	3.42E-17	Arabidopsis thaliana NAD(P)-binding Rossmann-fold superfamily protein mRNA, complete cds
AT2G30130	2.163845012	3.48E-17	Arabidopsis thaliana LOB domain-containing protein 12 mRNA, complete cds
AT2G40080	1.892969166	3.66E-17	Arabidopsis thaliana chromosome 2, complete sequence

AT5G44540	7.102916014	4.22E-17	Arabidopsis thaliana chromosome 5 sequence
AT3G10986	3.902978443	4.86E-17	Arabidopsis thaliana uncharacterized protein mRNA, complete cds
AT1G56060	4.165200292	5.26E-17	Arabidopsis thaliana uncharacterized protein mRNA, complete cds
AT1G63860	1.019091749	6.08E-17	Arabidopsis thaliana TIR-NBS-LRR class disease resistance protein mRNA, complete cds
AT5G54370	1.081041514	6.36E-17	Arabidopsis thaliana late embryogenesis abundant protein-like protein mRNA, complete cds
AT2G38000	1.011859908	6.46E-17	Arabidopsis thaliana chaperone protein dnaJ-like protein mRNA, complete cds
AT2G29120	1.144186487	6.70E-17	Arabidopsis thaliana glutamate receptor 2.7 mRNA, complete cds
AT3G51810	7.081542363	6.81E-17	Arabidopsis thaliana Em-like protein GEA1 mRNA, complete cds
AT4G18160	1.0907413	6.90E-17	Arabidopsis thaliana protein two-pore potassium channel 3 mRNA, complete cds
AT5G67230	1.280595241	7.16E-17	Arabidopsis thaliana probable beta-1,4-xylosyltransferase IRX14H mRNA, complete cds
AT5G67310	5.037820985	7.58E-17	Arabidopsis thaliana cytochrome P450, family 81, subfamily G, polypeptide 1 mRNA, complete cds
AT5G22530	3.237845594	8.69E-17	Arabidopsis thaliana chromosome 5 sequence
AT1G14780	1.213679283	9.13E-17	Arabidopsis thaliana MAC/Perforin domain-containing protein mRNA, complete cds
AT1G32190	1.158411126	9.92E-17	Arabidopsis thaliana alpha/beta-hydrolase-like protein mRNA, complete cds
AT3G63350	7.059847292	1.10E-16	Arabidopsis thaliana heat stress transcription factor A-7b mRNA, complete cds
AT3G22100	5.992733096	1.28E-16	Arabidopsis thaliana chromosome 3, complete sequence
AT2G18170	1.00459218	1.39E-16	Arabidopsis thaliana mitogen-activated protein kinase 7 mRNA, complete cds
AT1G46768	2.058521894	1.48E-16	Arabidopsis thaliana chromosome 1 sequence
AT2G16720	1.251751196	1.50E-16	Arabidopsis thaliana myb domain protein 7 mRNA, complete cds
AT1G67920	2.13093039	1.54E-16	Arabidopsis thaliana chromosome 1 sequence
AT3G16860	1.456962883	1.57E-16	Arabidopsis thaliana COBRA-like protein 8 mRNA, complete cds

AT3G14360	1.456962883	1.57E-16	Arabidopsis thaliana alpha/beta-hydrolases mRNA, complete cds
AT5G02600	1.008111197	1.77E-16	Arabidopsis thaliana heavy metal transport/detoxification domain-containing protein mRNA, complete cds
AT3G54530	7.037820985	1.78E-16	Arabidopsis thaliana uncharacterized protein mRNA, complete cds
AT1G10070	2.600415673	1.96E-16	Arabidopsis thaliana branched-chain-amino-acid aminotransferase 2 mRNA, complete cds
AT5G65690	1.093455684	1.99E-16	Arabidopsis thaliana phosphoenolpyruvate carboxykinase 2 mRNA, complete cds
AT1G73010	1.855974958	2.29E-16	Arabidopsis thaliana inorganic pyrophosphatase 1 mRNA, complete cds
AT1G17170	2.266414746	2.33E-16	Arabidopsis thaliana glutathione S-transferase TAU 24 mRNA, complete cds
AT1G21010	1.374534266	2.89E-16	Arabidopsis thaliana chromosome 1 sequence
AT1G23040	1.145600683	3.16E-16	Arabidopsis thaliana chromosome 1 sequence
AT2G01010	1.152026225	3.23E-16	Arabidopsis thaliana chromosome 2, complete sequence
AT2G26290	1.481771177	3.27E-16	Arabidopsis thaliana root-specific kinase 1 mRNA, complete cds
AT1G55265	1.481771177	3.27E-16	Arabidopsis thaliana chromosome 1 sequence
AT1G03070	2.324781231	3.53E-16	Arabidopsis thaliana protein lifeguard 4 mRNA, complete cds
AT5G01200	1.395225342	3.71E-16	Arabidopsis thaliana duplicated SANT DNA-binding domain-containing protein mRNA, complete cds
AT5G47990	1.861540489	3.88E-16	Arabidopsis thaliana cytochrome P450 705A5 mRNA, complete cds
AT1G54330	2.059847292	3.98E-16	Arabidopsis thaliana NAC domain containing protein 20 mRNA, complete cds
AT4G11480	2.083624675	4.05E-16	Arabidopsis thaliana putative cysteine-rich receptor-like protein kinase 32 mRNA, complete cds
AT2G31690	6.992733096	4.67E-16	Arabidopsis thaliana chromosome 2, complete sequence
AT1G27710	6.992733096	4.67E-16	Arabidopsis thaliana chromosome 1 sequence
AT5G66780	6.992733096	4.67E-16	Arabidopsis thaliana uncharacterized protein mRNA, complete cds
AT3G16990	1.319964214	5.04E-16	Arabidopsis thaliana heme oxygenase-like, multi-helical protein mRNA, complete cds
AT5G15160	1.964469244	5.58E-16	Arabidopsis thaliana protein banquo 2 mRNA, complete cds

AT2G29300	1.182909918	5.83E-16	Arabidopsis thaliana tropinone reductase-like protein mRNA, complete cds
AT3G15300	1.406962136	5.89E-16	Arabidopsis thaliana chromosome 3, complete sequence
AT1G19630	2.312070179	5.90E-16	Arabidopsis thaliana cytochrome P450, family 722, subfamily A, polypeptide 1 mRNA, complete cds
AT5G22380	4.430490672	6.30E-16	Arabidopsis thaliana NAC domain-containing protein mRNA, complete cds
AT1G02340	1.269636661	6.80E-16	Arabidopsis thaliana transcription factor HFR1 mRNA, complete cds
AT5G25160	1.259956295	6.90E-16	Arabidopsis thaliana chromosome 5 sequence
AT1G03730	1.365639527	7.13E-16	Arabidopsis thaliana chromosome 1 sequence
AT1G62940	6.969649483	7.59E-16	Arabidopsis thaliana acyl-CoA synthetase 5 mRNA, complete cds
AT5G07330	6.969649483	7.59E-16	Arabidopsis thaliana uncharacterized protein mRNA, complete cds
AT2G21130	1.208837147	8.43E-16	Arabidopsis thaliana chromosome 2, complete sequence
AT4G25850	3.167456266	8.85E-16	Arabidopsis thaliana OSBP(oxysterol binding protein)-related protein 4B mRNA, complete cds
AT5G14690	1.4194524	9.33E-16	Arabidopsis thaliana chromosome 5 sequence
AT4G18220	1.164741881	9.39E-16	Arabidopsis thaliana putative purine permease 9 mRNA, complete cds
AT5G36220	1.245122759	9.90E-16	Arabidopsis thaliana cytochrome P450 81D1 mRNA, complete cds
AT3G22160	1.584818818	9.92E-16	Arabidopsis thaliana chromosome 3, complete sequence
AT5G53290	1.423796019	1.09E-15	Arabidopsis thaliana chromosome 5 sequence
AT2G38823	2.167456266	1.10E-15	Arabidopsis thaliana uncharacterized protein mRNA, complete cds
AT3G21510	1.165815907	1.12E-15	Arabidopsis thaliana histidine-containing phosphotransmitter 1 mRNA, complete cds
AT5G15260	1.07255358	1.13E-15	Arabidopsis thaliana ribosomal protein L34e superfamily protein mRNA, complete cds
AT1G33970	1.104047096	1.21E-15	Arabidopsis thaliana AIG1 domain-containing protein mRNA, complete cds
AT2G35070	6.94619051	1.24E-15	Arabidopsis thaliana uncharacterized protein mRNA, complete cds
AT1G17285	2.790746885	1.49E-15	Arabidopsis thaliana uncharacterized protein mRNA, complete cds
AT1G10585	3.580237791	1.56E-15	Arabidopsis thaliana basic helix-loop-helix

			domain-containing protein mRNA, complete cds
AT1G34180	2.250669634	1.71E-15	Arabidopsis thaliana NAC domain containing protein 16 mRNA, complete cds
AT4G28110	4.037820985	1.91E-15	Arabidopsis thaliana myb domain protein 41 mRNA, complete cds
AT1G71450	6.922343768	2.01E-15	Arabidopsis thaliana chromosome 1 sequence
AT1G52430	2.856755426	2.09E-15	Arabidopsis thaliana ubiquitin carboxyl-terminal hydrolase-related protein mRNA, complete cds
AT3G01600	2.647721388	2.17E-15	Arabidopsis thaliana NAC domain containing protein 44 mRNA, complete cds
AT5G62770	1.218168109	2.36E-15	Arabidopsis thaliana chromosome 5 sequence
AT2G23180	1.691755985	2.42E-15	Arabidopsis thaliana chromosome 2, complete sequence
AT5G02780	2.351056204	2.47E-15	Arabidopsis thaliana glutathione transferase lambda 1 mRNA, complete cds
AT4G17670	1.298985889	2.52E-15	Arabidopsis thaliana uncharacterized protein mRNA, complete cds
AT2G24400	1.972384451	2.52E-15	Arabidopsis thaliana chromosome 2, complete sequence
AT1G55280	1.337381267	2.66E-15	Arabidopsis thaliana Lipase/lipooxygenase, PLAT/LH2 family protein mRNA, complete cds
AT2G41380	2.015453172	2.73E-15	Arabidopsis thaliana S-adenosyl-L-methionine-dependent methyltransferase-like protein mRNA, complete cds
AT1G69150	3.13093039	2.81E-15	Arabidopsis thaliana chromosome 1 sequence
AT3G21600	1.101183046	2.82E-15	Arabidopsis thaliana Senescence/dehydration-associated protein-like protein mRNA, complete cds
AT5G51260	1.221482148	3.33E-15	Arabidopsis thaliana HAD superfamily, subfamily IIIB acid phosphatase mRNA, complete cds
AT2G27010	1.672565459	3.33E-15	Arabidopsis thaliana cytochrome P450, family 705, subfamily A, polypeptide 9 mRNA, complete cds
AT4G11470	1.941452591	3.84E-15	Arabidopsis thaliana putative cysteine-rich receptor-like protein kinase 31 mRNA, complete cds
AT3G07080	1.126097236	3.84E-15	Arabidopsis thaliana EamA-like transporter mRNA, complete cds
AT4G03510	1.189347076	3.88E-15	Arabidopsis thaliana E3 ubiquitin-protein

			ligase RMA1 mRNA, complete cds
AT1G02610	1.827826169	4.06E-15	Arabidopsis thaliana RING/FYVE/PHD zinc finger-containing protein mRNA, complete cds
AT5G44417	1.982286308	4.23E-15	Arabidopsis thaliana chromosome 5 sequence
AT4G00850	1.392610788	4.27E-15	Arabidopsis thaliana GRF1-interacting factor 3 mRNA, complete cds
AT5G55150	6.873434168	5.37E-15	Arabidopsis thaliana uncharacterized protein mRNA, complete cds
AT5G26770	1.134239325	5.62E-15	Arabidopsis thaliana uncharacterized protein mRNA, complete cds
AT5G59720	1.673944709	5.72E-15	Arabidopsis thaliana chromosome 5 sequence
AT1G75600	5.822808094	5.85E-15	Arabidopsis thaliana histone H3-like 3 mRNA, complete cds
AT5G43840	5.822808094	5.85E-15	Arabidopsis thaliana heat stress transcription factor A-6a mRNA, complete cds
AT4G28840	2.822808094	6.40E-15	Arabidopsis thaliana TCP interactor containing EAR motif protein1 mRNA, complete cds
AT5G05880	1.83307643	6.92E-15	Arabidopsis thaliana UDP-glycosyltransferase 76C4 mRNA, complete cds
AT1G13195	1.156051503	7.20E-15	Arabidopsis thaliana RING/U-box domain-containing protein mRNA, complete cds
AT3G08505	1.092931925	7.56E-15	Arabidopsis thaliana zinc finger (CCCH-type/C3HC4-type RING finger) family protein mRNA, complete cds
AT3G15200	2.211850385	7.79E-15	Arabidopsis thaliana chromosome 3, complete sequence
AT3G10815	3.35784537	7.80E-15	Arabidopsis thaliana RING/U-box domain-containing protein mRNA, complete cds
AT5G15800	1.865557742	8.04E-15	Arabidopsis thaliana MADS box transcription factor SEPALLATA 1 mRNA, complete cds
AT1G71520	4.848343187	8.17E-15	Arabidopsis thaliana chromosome 1 sequence
AT5G62490	6.848343187	8.80E-15	Arabidopsis thaliana HVA22-like protein b mRNA, complete cds
AT5G41620	1.204930971	9.26E-15	Arabidopsis thaliana uncharacterized protein mRNA, complete cds
AT3G13620	1.143777269	9.77E-15	Arabidopsis thaliana POLYAMINE UPTAKE TRANSPORTER 4 mRNA, complete cds
AT3G27270	1.919593157	9.78E-15	Arabidopsis thaliana chromosome 3, complete sequence

AT5G54840	1.061549587	9.94E-15	Arabidopsis thaliana monomeric G protein SGP1 mRNA, complete cds
AT2G26480	2.396882279	1.02E-14	Arabidopsis thaliana UDP-glucosyl transferase 76D1 mRNA, complete cds
AT3G27880	1.41857735	1.08E-14	Arabidopsis thaliana chromosome 3, complete sequence
AT5G46500	1.189482572	1.08E-14	Arabidopsis thaliana uncharacterized protein mRNA, complete cds
AT4G22610	3.205277731	1.14E-14	Arabidopsis thaliana chromosome 4 sequence
AT1G61255	3.969649483	1.15E-14	Arabidopsis thaliana chromosome 1 sequence
AT1G16160	1.872088997	1.37E-14	Arabidopsis thaliana wall-associated receptor kinase-like 5 mRNA, complete cds
AT1G55740	1.015453172	1.42E-14	Arabidopsis thaliana raffinose synthase 1 mRNA, complete cds
AT4G23920	1.237845594	1.55E-14	Arabidopsis thaliana UDP-glucose 4-epimerase 2 mRNA, complete cds
AT3G17130	1.79745252	1.57E-14	Arabidopsis thaliana chromosome 3, complete sequence
AT4G16820	3.074346861	1.58E-14	Arabidopsis thaliana chromosome 4 sequence
AT5G67430	3.496579862	1.67E-14	Arabidopsis thaliana GCN5-related N-acetyltransferase (GNAT) family protein mRNA, complete cds
AT1G25400	1.113563258	1.71E-14	Arabidopsis thaliana chromosome 1 sequence
AT5G10720	1.047463567	1.75E-14	Arabidopsis thaliana histidine kinase 5 mRNA, complete cds
AT2G46640	1.443134881	2.29E-14	Arabidopsis thaliana protein TILLER ANGLE CONTROL 1 mRNA, complete cds
AT2G16367	6.796812886	2.37E-14	Arabidopsis thaliana chromosome 2, complete sequence
AT4G09600	6.796812886	2.37E-14	Arabidopsis thaliana gibberellin-regulated protein 3 mRNA, complete cds
AT3G58070	1.467149142	2.42E-14	Arabidopsis thaliana chromosome 3, complete sequence
AT3G51090	1.196782937	2.58E-14	Arabidopsis thaliana uncharacterized protein mRNA, complete cds
AT5G18560	2.415383779	2.68E-14	Arabidopsis thaliana chromosome 5 sequence
AT4G10350	3.474884791	3.02E-14	Arabidopsis thaliana protein BEARSKIN 2 mRNA, complete cds
AT5G39520	2.171957658	3.50E-14	Arabidopsis thaliana uncharacterized protein mRNA, complete cds
AT5G01840	1.218270055	3.66E-14	Arabidopsis thaliana chromosome 5 sequence
AT3G02493	4.263380686	4.14E-14	Arabidopsis thaliana chromosome 3, complete sequence

AT5G38120	1.905270255	4.19E-14	Arabidopsis thaliana 4-coumarate--CoA ligase-like 8 mRNA, complete cds
AT4G36670	1.229819453	4.34E-14	Arabidopsis thaliana putative polyol transporter 6 mRNA, complete cds
AT1G52040	1.13093039	4.39E-14	Arabidopsis thaliana myrosinase-binding protein 1 mRNA, complete cds
AT3G29035	1.738935537	4.91E-14	Arabidopsis thaliana NAC domain-containing protein 3 mRNA, complete cds
AT4G15230	1.389577478	4.93E-14	Arabidopsis thaliana ABC transporter G family member 30 mRNA, complete cds
AT1G01570	1.856755426	5.79E-14	Arabidopsis thaliana uncharacterized protein mRNA, complete cds
AT1G61560	1.204723524	6.11E-14	Arabidopsis thaliana MLO-like protein 6 mRNA, complete cds
AT3G52740	1.483058723	6.42E-14	Arabidopsis thaliana chromosome 3, complete sequence
AT1G15190	3.144736189	6.48E-14	Arabidopsis thaliana chromosome 1 sequence
AT1G74490	1.780987919	6.54E-14	Arabidopsis thaliana protein kinase superfamily protein mRNA, complete cds
AT1G53110	1.206395955	7.25E-14	Arabidopsis thaliana putative proton pump interactor protein mRNA, complete cds
AT1G15520	1.370718451	8.92E-14	Arabidopsis thaliana ABC transporter G family member 40 mRNA, complete cds
AT5G08780	1.076853717	9.17E-14	Arabidopsis thaliana winged-helix DNA-binding transcription factor family protein mRNA, complete cds
AT2G42560	5.687878514	9.33E-14	Arabidopsis thaliana late embryogenesis abundant domain-containing protein mRNA, complete cds
AT4G27652	2.534827332	9.64E-14	Arabidopsis thaliana chromosome 4 sequence
AT5G56160	1.340939087	9.88E-14	Arabidopsis thaliana sec14p-like phosphatidylinositol transfer-like protein mRNA, complete cds
AT5G14750	1.649659192	1.04E-13	Arabidopsis thaliana transcription factor WER mRNA, complete cds
AT5G15290	6.715892891	1.06E-13	Arabidopsis thaliana casparian strip membrane protein 5 mRNA, complete cds
AT5G42325	3.624262415	1.14E-13	Arabidopsis thaliana transcription factor IIS protein mRNA, complete cds
AT4G23515	3.123977629	1.16E-13	Arabidopsis thaliana Toll-Interleukin-1 receptor domain-containing protein mRNA, complete cds
AT1G78600	1.077737451	1.31E-13	Arabidopsis thaliana light-regulated zinc finger protein 1 mRNA, complete cds

AT5G20910	1.02485187	1.35E-13	Arabidopsis thaliana E3 ubiquitin-protein ligase AIP2 mRNA, complete cds
AT1G46554	1.745364009	1.44E-13	Arabidopsis thaliana chromosome 1 sequence
AT5G61890	3.25249237	1.46E-13	Arabidopsis thaliana ethylene-responsive transcription factor ERF114 mRNA, complete cds
AT5G46350	2.041925384	1.49E-13	Arabidopsis thaliana putative WRKY transcription factor 8 mRNA, complete cds
AT1G17020	2.13093039	1.56E-13	Arabidopsis thaliana Fe(II)/ascorbate oxidase family protein SRG1 mRNA, complete cds
AT1G58420	2.458396668	1.84E-13	Arabidopsis thaliana chromosome 1 sequence
AT1G13340	1.610001722	1.90E-13	Arabidopsis thaliana Regulator of Vps4 activity in the MVB pathway protein mRNA, complete cds
AT1G61460	1.721167833	2.02E-13	Arabidopsis thaliana G-type lectin S-receptor-like serine/threonine-protein kinase mRNA, complete cds
AT1G04570	1.931621582	2.02E-13	Arabidopsis thaliana probable folate-biopterin transporter mRNA, complete cds
AT5G01720	1.217087034	2.04E-13	Arabidopsis thaliana F-box/LRR-repeat protein 3 mRNA, complete cds
AT5G65300	2.30668447	2.30E-13	Arabidopsis thaliana chromosome 5 sequence
AT3G06520	2.002514117	2.33E-13	Arabidopsis thaliana agenet domain-containing protein mRNA, complete cds
AT2G26160	6.659309362	2.91E-13	Arabidopsis thaliana chromosome 2, complete sequence
AT1G30500	1.319459359	2.96E-13	Arabidopsis thaliana nuclear transcription factor Y subunit A-7 mRNA, complete cds
AT5G66870	1.389268009	3.34E-13	Arabidopsis thaliana LOB domain-containing protein 36 (ASYMMETRIC LEAVES 2-like protein 1) mRNA, complete cds
AT3G28850	1.372523073	3.55E-13	Arabidopsis thaliana chromosome 3, complete sequence
AT4G38560	1.964827099	3.59E-13	Arabidopsis thaliana phospholipase like protein (PEARLI 4) mRNA, complete cds
AT5G48400	3.081542363	3.67E-13	Arabidopsis thaliana glutamate receptor 1.2 mRNA, complete cds
AT2G32190	2.618794202	3.70E-13	Arabidopsis thaliana uncharacterized protein mRNA, complete cds
AT5G47850	3.576168127	3.75E-13	Arabidopsis thaliana chromosome 5 sequence
AT5G44260	2.015453172	3.90E-13	Arabidopsis thaliana chromosome 5 sequence
AT2G39370	2.015453172	3.90E-13	Arabidopsis thaliana chromosome 2, complete sequence

AT2G01340	1.462136298	4.15E-13	Arabidopsis thaliana uncharacterized protein mRNA, complete cds
AT5G44005	2.170731398	4.17E-13	Arabidopsis thaliana chromosome 5 sequence
AT1G70640	3.822808094	4.21E-13	Arabidopsis thaliana octicosapeptide/Phox/Bem1p (PB1) domain-containing protein mRNA, complete cds
AT5G44065	4.158411126	4.57E-13	Arabidopsis thaliana chromosome 5 sequence
AT2G22200	1.699951347	4.82E-13	Arabidopsis thaliana chromosome 2, complete sequence
AT5G25190	1.381405891	4.87E-13	Arabidopsis thaliana ethylene-responsive transcription factor ERF003 mRNA, complete cds
AT5G50335	2.954052628	4.91E-13	Arabidopsis thaliana chromosome 5 sequence
AT3G55640	1.020385468	6.36E-13	Arabidopsis thaliana mitochondrial substrate carrier family protein mRNA, complete cds
AT3G51680	3.059847292	6.52E-13	Arabidopsis thaliana short-chain dehydrogenase reductase 2a mRNA, complete cds
AT3G25010	1.115803698	6.54E-13	Arabidopsis thaliana chromosome 3, complete sequence
AT1G21910	1.676651259	6.65E-13	Arabidopsis thaliana chromosome 1 sequence
AT2G29990	1.199877744	6.74E-13	Arabidopsis thaliana alternative NAD(P)H dehydrogenase 2 mRNA, complete cds
AT4G16000	2.192991358	6.79E-13	Arabidopsis thaliana chromosome 4 sequence
AT1G67070	1.756210346	7.26E-13	Arabidopsis thaliana mannose-6-phosphate isomerase mRNA, complete cds
AT5G65600	2.530026345	7.30E-13	Arabidopsis thaliana chromosome 5 sequence
AT1G22370	1.233358203	8.03E-13	Arabidopsis thaliana UDP-glucosyl transferase 85A5 mRNA, complete cds
AT4G36600	6.600415673	8.04E-13	Arabidopsis thaliana Late embryogenesis abundant (LEA) protein mRNA, complete cds
AT5G64890	3.185378174	8.39E-13	Arabidopsis thaliana elicitor peptide 2 mRNA, complete cds
AT1G70140	1.102916014	8.70E-13	Arabidopsis thaliana formin-like protein 8 mRNA, complete cds
AT1G34060	1.148135656	8.72E-13	Arabidopsis thaliana tryptophan aminotransferase-related protein 4 mRNA, complete cds
AT1G30135	5.570042024	8.79E-13	Arabidopsis thaliana protein TIFY 5A mRNA, complete cds
AT2G19190	1.962985753	9.70E-13	Arabidopsis thaliana FLG22-induced receptor-like kinase 1 mRNA, complete cds

AT5G24600	3.337381267	1.04E-12	Arabidopsis thaliana uncharacterized protein mRNA, complete cds
AT1G24330	1.479400272	1.10E-12	Arabidopsis thaliana U-box domain-containing protein 6 mRNA, complete cds
AT4G14819	3.526415092	1.23E-12	Arabidopsis thaliana chromosome 4 sequence
AT3G59900	1.691324169	1.28E-12	Arabidopsis thaliana chromosome 3, complete sequence
AT2G24840	6.570042024	1.34E-12	Arabidopsis thaliana chromosome 2, complete sequence
AT5G04380	6.570042024	1.34E-12	Arabidopsis thaliana S-adenosyl-L-methionine-dependent methyltransferase-like protein mRNA, complete cds
AT1G74430	2.911617361	1.53E-12	Arabidopsis thaliana putative transcription factor MYB95 mRNA, complete cds
AT4G11170	1.655277608	1.57E-12	Arabidopsis thaliana putative disease resistance protein mRNA, complete cds
AT1G12740	1.71873564	1.58E-12	Arabidopsis thaliana cytochrome P450, family 87, subfamily A, polypeptide 2 mRNA, complete cds
AT3G17110	4.600415673	1.69E-12	Arabidopsis thaliana chromosome 3, complete sequence
AT4G22950	1.829040048	1.77E-12	Arabidopsis thaliana agamous-like MADS-box protein AGL19 mRNA, complete cds
AT1G19250	1.44841258	1.80E-12	Arabidopsis thaliana flavin-dependent monooxygenase 1 mRNA, complete cds
AT5G62280	1.171022881	1.83E-12	Arabidopsis thaliana uncharacterized protein mRNA, complete cds
AT5G04080	1.257113927	1.86E-12	Arabidopsis thaliana uncharacterized protein mRNA, complete cds
AT1G65500	1.848343187	1.91E-12	Arabidopsis thaliana uncharacterized protein mRNA, complete cds
AT3G48340	1.048119361	2.04E-12	Arabidopsis thaliana KDEL-tailed cysteine endopeptidase CEP2 mRNA, complete cds
AT5G46013	6.539015128	2.24E-12	Arabidopsis thaliana chromosome 5 sequence
AT2G20555	6.539015128	2.24E-12	Arabidopsis thaliana chromosome 2, complete sequence
AT4G24110	2.373005177	2.56E-12	Arabidopsis thaliana chromosome 4 sequence
AT1G75450	1.086419694	2.68E-12	Arabidopsis thaliana cytokinin dehydrogenase 5 mRNA, complete cds
AT1G48405	5.507306269	2.72E-12	Arabidopsis thaliana Kinase interacting (KIP1-like) family protein mRNA, complete

			cds
AT1G73210	1.321699645	2.78E-12	Arabidopsis thaliana uncharacterized protein mRNA, complete cds
AT4G01010	1.093455684	2.79E-12	Arabidopsis thaliana cyclic nucleotide-gated channel 13 mRNA, complete cds
AT3G52310	2.271792926	2.86E-12	Arabidopsis thaliana ABC transporter G family member 27 mRNA, complete cds
AT3G04420	2.110610405	3.04E-12	Arabidopsis thaliana NAC domain containing protein 48 mRNA, complete cds
AT3G25400	1.445693078	3.09E-12	Arabidopsis thaliana uncharacterized protein mRNA, complete cds
AT5G53870	1.506701238	3.36E-12	Arabidopsis thaliana early nodulin-like protein 1 mRNA, complete cds
AT4G15140	1.101426396	3.46E-12	Arabidopsis thaliana uncharacterized protein mRNA, complete cds
AT2G34500	1.345601774	3.69E-12	Arabidopsis thaliana chromosome 2, complete sequence
AT4G10250	6.507306269	3.75E-12	Arabidopsis thaliana chromosome 4 sequence
AT1G31820	1.550490448	3.90E-12	Arabidopsis thaliana polyamine transporter 1 mRNA, complete cds
AT1G76980	1.168927653	4.31E-12	Arabidopsis thaliana uncharacterized protein mRNA, complete cds
AT3G24750	1.973224937	4.41E-12	Arabidopsis thaliana uncharacterized protein mRNA, complete cds
AT3G24090	1.020786617	4.53E-12	Arabidopsis thaliana putative glucosamine-fructose-6-phosphate aminotransferase mRNA, complete cds
AT4G04490	1.436590871	4.56E-12	Arabidopsis thaliana cysteine-rich receptor-like protein kinase 36 mRNA, complete cds
AT2G47890	1.043359169	4.72E-12	Arabidopsis thaliana zinc finger protein CONSTANS-LIKE 13 mRNA, complete cds
AT5G37550	1.842616576	5.15E-12	Arabidopsis thaliana chromosome 5 sequence
AT1G10280	1.01017823	5.76E-12	Arabidopsis thaliana core-2/I-branching beta-1,6-N-acetylglucosaminyltransferase family protein mRNA, complete cds
AT5G22545	6.474884791	6.28E-12	Arabidopsis thaliana chromosome 5 sequence
AT1G47600	1.214233037	6.34E-12	Arabidopsis thaliana myrosinase 4 mRNA, complete cds
AT3G21660	2.393964796	6.77E-12	Arabidopsis thaliana UBX domain-containing protein mRNA, complete cds
AT3G11580	2.337381267	7.26E-12	Arabidopsis thaliana AP2/B3-like transcriptional factor family protein mRNA, complete cds

AT1G34670	1.266261276	7.31E-12	Arabidopsis thaliana myb domain protein 93 mRNA, complete cds
AT1G72070	2.580237791	8.34E-12	Arabidopsis thaliana chaperone DnaJ-domain containing protein mRNA, complete cds
AT4G22530	1.412343325	8.46E-12	Arabidopsis thaliana S-adenosyl-L-methionine-dependent methyltransferase domain-containing protein mRNA, complete cds
AT1G49530	1.39217374	9.08E-12	Arabidopsis thaliana chromosome 1 sequence
AT5G64180	1.038173249	9.15E-12	Arabidopsis thaliana uncharacterized protein mRNA, complete cds
AT1G19200	2.504838013	9.48E-12	Arabidopsis thaliana uncharacterized protein mRNA, complete cds
AT5G02170	2.436916941	1.05E-11	Arabidopsis thaliana transmembrane amino acid transporter family protein mRNA, complete cds
AT5G62040	6.441717927	1.06E-11	Arabidopsis thaliana protein BROTHER of FT and TFL 1 mRNA, complete cds
AT1G13530	1.944370074	1.14E-11	Arabidopsis thaliana uncharacterized protein mRNA, complete cds
AT5G08760	1.044717767	1.38E-11	Arabidopsis thaliana uncharacterized protein mRNA, complete cds
AT1G67970	1.278487578	1.43E-11	Arabidopsis thaliana heat stress transcription factor A-8 mRNA, complete cds
AT3G03280	2.559773689	1.43E-11	Arabidopsis thaliana chromosome 3, complete sequence
AT5G59570	1.032662462	1.48E-11	Arabidopsis thaliana protein BROTHER OF LUX ARRHYTHMO mRNA, complete cds
AT2G30340	1.032662462	1.48E-11	Arabidopsis thaliana LOB domain-containing protein 13 mRNA, complete cds
AT1G69600	2.484938456	1.62E-11	Arabidopsis thaliana chromosome 1 sequence
AT5G15660	6.407770595	1.78E-11	Arabidopsis thaliana putative F-box protein mRNA, complete cds
AT2G35300	6.407770595	1.78E-11	Arabidopsis thaliana chromosome 2, complete sequence
AT3G50260	1.393964796	1.81E-11	Arabidopsis thaliana chromosome 3, complete sequence
AT3G05936	1.929723298	1.82E-11	Arabidopsis thaliana chromosome 3, complete sequence
AT4G25410	2.35649009	1.93E-11	Arabidopsis thaliana transcription factor bHLH126 mRNA, complete cds
AT5G22270	1.547173652	1.99E-11	Arabidopsis thaliana uncharacterized protein mRNA, complete cds
AT1G65510	1.450390229	2.10E-11	Arabidopsis thaliana uncharacterized protein

			mRNA, complete cds
AT3G50770	2.04787465	2.15E-11	Arabidopsis thaliana chromosome 3, complete sequence
AT2G22790	2.082567368	2.19E-11	Arabidopsis thaliana chromosome 2, complete sequence
AT2G31180	2.159843082	2.22E-11	Arabidopsis thaliana myb domain protein 14 mRNA, complete cds
AT4G04500	3.393964796	2.39E-11	Arabidopsis thaliana cysteine-rich receptor-like protein kinase 37 mRNA, complete cds
AT2G12646	1.164316558	2.40E-11	Arabidopsis thaliana PLATZ transcription factor family protein mRNA, complete cds
AT3G54150	1.866309733	2.55E-11	Arabidopsis thaliana S-adenosyl-L-methionine-dependent methyltransferase-like protein mRNA, complete cds
AT5G38130	5.373005177	2.67E-11	Arabidopsis thaliana chromosome 5 sequence
AT2G31990	1.498775411	2.94E-11	Arabidopsis thaliana Exostosin family protein mRNA, complete cds
AT4G27654	6.373005177	3.00E-11	Arabidopsis thaliana chromosome 4 sequence
AT4G28395	6.373005177	3.00E-11	Arabidopsis thaliana lipid transfer protein-related mRNA, complete cds
AT2G40340	3.954052628	3.09E-11	Arabidopsis thaliana dehydration-responsive element-binding protein 2C mRNA, complete cds
AT5G50820	1.44047476	3.11E-11	Arabidopsis thaliana NAC domain containing protein 97 mRNA, complete cds
AT5G13330	1.829897519	3.73E-11	Arabidopsis thaliana ethylene-responsive transcription factor ERF113 mRNA, complete cds
AT3G28945.1	1.375349118	3.87E-11	Arabidopsis thaliana chromosome 3, complete sequence
AT5G35940	1.423918018	3.93E-11	Arabidopsis thaliana jacalin-like lectin family protein mRNA, complete cds
AT4G31110	1.254240032	4.15E-11	Arabidopsis thaliana wall-associated receptor kinase-like 18 mRNA, complete cds
AT1G19530	1.202656165	4.20E-11	Arabidopsis thaliana uncharacterized protein mRNA, complete cds
AT1G79915	3.36595042	4.32E-11	Arabidopsis thaliana putative methyltransferase family protein mRNA, complete cds
AT4G12410	3.36595042	4.32E-11	Arabidopsis thaliana chromosome 4 sequence
AT4G03960	1.319787207	4.46E-11	Arabidopsis thaliana atypical dual-specificity phosphatase 4 mRNA, complete cds

AT5G19100	1.525314218	4.49E-11	Arabidopsis thaliana chromosome 5 sequence
AT5G46330	1.027425814	4.91E-11	Arabidopsis thaliana LRR receptor-like serine/threonine-protein kinase FLS2 mRNA, complete cds
AT2G15780	3.600415673	5.07E-11	Arabidopsis thaliana Cupredoxin superfamily protein mRNA, complete cds
AT2G43580	6.337381267	5.07E-11	Arabidopsis thaliana chitinase family protein mRNA, complete cds
AT1G01560	1.83745487	6.37E-11	Arabidopsis thaliana mitogen-activated protein kinase 11 mRNA, complete cds
AT1G21326	3.337381267	7.80E-11	Arabidopsis thaliana chromosome 1 sequence
AT5G40500	1.021502217	7.93E-11	Arabidopsis thaliana uncharacterized protein mRNA, complete cds
AT1G79900	2.423537911	7.99E-11	Arabidopsis thaliana mitochondrial arginine transporter BAC2 mRNA, complete cds
AT1G47990	5.300855391	8.43E-11	Arabidopsis thaliana gibberellin 2-oxidase 4 mRNA, complete cds
AT5G44310	6.300855391	8.60E-11	Arabidopsis thaliana Late embryogenesis abundant protein family protein mRNA, complete cds
AT3G25240	6.300855391	8.60E-11	Arabidopsis thaliana chromosome 3, complete sequence
AT2G32130	6.300855391	8.60E-11	Arabidopsis thaliana chromosome 2, complete sequence
AT1G69930	6.300855391	8.60E-11	Arabidopsis thaliana glutathione S-transferase TAU 11 mRNA, complete cds
AT3G52130	6.300855391	8.60E-11	Arabidopsis thaliana chromosome 3, complete sequence
AT3G45730	1.166617246	9.59E-11	Arabidopsis thaliana chromosome 3, complete sequence
AT1G31880	1.254919107	9.83E-11	Arabidopsis thaliana protein BREVIS RADIX mRNA, complete cds
AT2G19900	3.88992229	1.03E-10	Arabidopsis thaliana NADP-dependent malic enzyme 1 mRNA, complete cds
AT4G38140	3.13093039	1.12E-10	Arabidopsis thaliana chromosome 4 sequence
AT5G65207	1.021605399	1.13E-10	Arabidopsis thaliana chromosome 5 sequence
AT1G64470	1.04787465	1.18E-10	Arabidopsis thaliana chromosome 1 sequence
AT1G20030	1.328043403	1.24E-10	Arabidopsis thaliana pathogenesis-related thaumatin-like protein mRNA, complete cds
AT4G01060	2.402476296	1.36E-10	Arabidopsis thaliana CAPRICE-like MYB3 mRNA, complete cds
AT5G05340	2.337381267	1.46E-10	Arabidopsis thaliana peroxidase 52 mRNA, complete cds

AT2G22760	6.263380686	1.46E-10	Arabidopsis thaliana transcription factor bHLH19 mRNA, complete cds
AT4G27970	1.034646738	1.52E-10	Arabidopsis thaliana SLAC1 homologue 2 mRNA, complete cds
AT5G45310	1.808011093	1.58E-10	Arabidopsis thaliana uncharacterized protein mRNA, complete cds
AT5G04120	2.175917845	1.62E-10	Arabidopsis thaliana phosphoglycerate mutase-like protein mRNA, complete cds
AT1G16515	3.102916014	2.01E-10	Arabidopsis thaliana chromosome 1 sequence
AT5G57190	1.132176779	2.11E-10	Arabidopsis thaliana phosphatidylserine decarboxylase 2 mRNA, complete cds
AT2G17080	1.772182021	2.27E-10	Arabidopsis thaliana chromosome 2, complete sequence
AT5G66630	1.041925384	2.29E-10	Arabidopsis thaliana DA1-related protein 5 mRNA, complete cds
AT5G19470	6.224906538	2.49E-10	Arabidopsis thaliana nudix hydrolase 24 mRNA, complete cds
AT4G13395	6.224906538	2.49E-10	Arabidopsis thaliana chromosome 4 sequence
AT4G15200	6.224906538	2.49E-10	Arabidopsis thaliana formin 3 mRNA, complete cds
AT1G29650.1	6.224906538	2.49E-10	Arabidopsis thaliana chromosome 1 sequence
AT3G15280	6.224906538	2.49E-10	Arabidopsis thaliana uncharacterized protein mRNA, complete cds
AT1G66170	5.224906538	2.68E-10	Arabidopsis thaliana PHD finger protein MALE MEIOCYTE DEATH 1 mRNA, complete cds
AT5G09876	5.224906538	2.68E-10	Arabidopsis thaliana uncharacterized protein mRNA, complete cds
AT4G12490	2.936018705	2.69E-10	Arabidopsis thaliana chromosome 4 sequence
AT2G43890	1.003069448	2.78E-10	Arabidopsis thaliana putative polygalacturonase /pectinase mRNA, complete cds
AT2G26390	1.719468345	2.90E-10	Arabidopsis thaliana serpin-Z3 mRNA, complete cds
AT5G47920	1.88992229	3.39E-10	Arabidopsis thaliana chromosome 5 sequence
AT2G46940	1.23489831	3.96E-10	Arabidopsis thaliana uncharacterized protein mRNA, complete cds
AT5G62100	1.206879243	3.96E-10	Arabidopsis thaliana BCL-2-associated athanogene 2 mRNA, complete cds
AT5G62420	3.248113929	4.57E-10	Arabidopsis thaliana aldo/keto reductase family protein mRNA, complete cds
AT2G36770	2.908537969	4.76E-10	Arabidopsis thaliana chromosome 2,

			complete sequence
AT2G27550	1.398310266	4.98E-10	Arabidopsis thaliana protein CENTRORADIALIS-like mRNA, complete cds
AT5G41120	1.05809751	5.19E-10	Arabidopsis thaliana Esterase/lipase/thioesterase family protein mRNA, complete cds
AT1G75000	1.187513918	6.58E-10	Arabidopsis thaliana GNS1/SUR4 membrane-like protein mRNA, complete cds
AT5G21950	1.228856811	6.66E-10	Arabidopsis thaliana hydrolase, alpha/beta fold family protein mRNA, complete cds
AT1G72800	1.051077082	7.08E-10	Arabidopsis thaliana chromosome 1 sequence
AT3G43110	2.11781489	7.23E-10	Arabidopsis thaliana chromosome 3, complete sequence
AT5G64210	6.144736189	7.28E-10	Arabidopsis thaliana alternative oxidase 2 mRNA, complete cds
AT5G47530	1.806866551	7.30E-10	Arabidopsis thaliana putative auxin-responsive protein mRNA, complete cds
AT4G27950	1.141716945	7.30E-10	Arabidopsis thaliana chromosome 4 sequence
AT3G23840	1.058954811	7.43E-10	Arabidopsis thaliana HXXXD-type acyl-transferase-like protein mRNA, complete cds
AT1G24470	1.526415092	7.85E-10	Arabidopsis thaliana beta-ketoacyl reductase 2 mRNA, complete cds
AT5G14470	3.217087034	8.22E-10	Arabidopsis thaliana GHMP kinase family protein mRNA, complete cds
AT1G78440	3.217087034	8.22E-10	Arabidopsis thaliana gibberellin 2-beta-dioxygenase 1 mRNA, complete cds
AT1G15040	1.856755426	8.57E-10	Arabidopsis thaliana chromosome 1 sequence
AT5G42290	5.144736189	8.61E-10	Arabidopsis thaliana chromosome 5 sequence
AT1G19040	5.144736189	8.61E-10	Arabidopsis thaliana chromosome 1 sequence
AT2G31945	2.462912149	9.44E-10	Arabidopsis thaliana chromosome 2, complete sequence
AT5G59730	1.600415673	1.01E-09	Arabidopsis thaliana chromosome 5 sequence
AT3G54510	2.743373627	1.05E-09	Arabidopsis thaliana Early-responsive to dehydration stress protein (ERD4) mRNA, complete cds
AT2G34910	1.978927296	1.09E-09	Arabidopsis thaliana chromosome 2, complete sequence
AT5G59510	2.315013454	1.10E-09	Arabidopsis thaliana chromosome 5 sequence
AT5G67080	3.752418767	1.15E-09	Arabidopsis thaliana chromosome 5 sequence
AT2G44280	1.022372586	1.17E-09	Arabidopsis thaliana major facilitator protein

			mRNA, complete cds
AT4G20420	1.814540478	1.25E-09	Arabidopsis thaliana chromosome 4 sequence
AT5G09430	2.626887885	1.26E-09	Arabidopsis thaliana alpha/beta-Hydrolases superfamily protein mRNA, complete cds
AT4G23680	1.060307408	1.27E-09	Arabidopsis thaliana polyketide cyclase/dehydrase and lipid transport superfamily protein mRNA, complete cds
AT1G03905	1.257341769	1.31E-09	Arabidopsis thaliana ABC transporter I family member 19 mRNA, complete cds
AT5G43440	1.389428015	1.46E-09	Arabidopsis thaliana 1-aminocyclopropane-1-carboxylate oxidase-like protein 9 mRNA, complete cds
AT2G23150	1.096071633	1.51E-09	Arabidopsis thaliana metal transporter Nramp3 mRNA, complete cds
AT2G33080	5.102916014	1.55E-09	Arabidopsis thaliana chromosome 2, complete sequence
AT4G21930	5.102916014	1.55E-09	Arabidopsis thaliana chromosome 4 sequence
AT4G18210	1.244271863	1.57E-09	Arabidopsis thaliana purine permease 10 mRNA, complete cds
AT1G49210	1.547466793	1.78E-09	Arabidopsis thaliana chromosome 1 sequence
AT2G46150	1.371596983	1.82E-09	Arabidopsis thaliana chromosome 2, complete sequence
AT3G21780	2.985079523	2.03E-09	Arabidopsis thaliana chromosome 3, complete sequence
AT1G32960	2.985079523	2.03E-09	Arabidopsis thaliana Subtilase family protein SBT3.3 mRNA, complete cds
AT5G22540	3.715892891	2.10E-09	Arabidopsis thaliana chromosome 5 sequence
AT1G78160	6.059847292	2.15E-09	Arabidopsis thaliana pumilio 7 mRNA, complete cds
AT3G04620	1.251081421	2.22E-09	Arabidopsis thaliana DUO1-activated nucleic acid binding protein mRNA, complete cds
AT5G06510	1.849443221	2.32E-09	Arabidopsis thaliana nuclear transcription factor Y subunit A-10 mRNA, complete cds
AT5G55050	1.098869181	2.59E-09	Arabidopsis thaliana GDSL esterase/lipase mRNA, complete cds
AT4G25400	2.822808094	2.61E-09	Arabidopsis thaliana transcription factor bHLH118 mRNA, complete cds
AT4G34850	3.152956696	2.65E-09	Arabidopsis thaliana protein LESS ADHESIVE POLLEN 5 mRNA, complete cds
AT1G24140	1.360281669	2.65E-09	Arabidopsis thaliana chromosome 1 sequence
AT1G52240	1.071101202	2.73E-09	Arabidopsis thaliana RHO guanyl-nucleotide exchange factor 11 mRNA, complete cds

AT2G20800	5.059847292	2.78E-09	Arabidopsis thaliana NAD(P)H dehydrogenase B4 mRNA, complete cds
AT3G15518	1.941452591	2.82E-09	Arabidopsis thaliana chromosome 3, complete sequence
AT3G25597	2.337381267	2.95E-09	Arabidopsis thaliana chromosome 3, complete sequence
AT3G16175	1.498235278	3.04E-09	Arabidopsis thaliana thioesterase family protein mRNA, complete cds
AT1G51620	1.805530103	3.38E-09	Arabidopsis thaliana protein kinase superfamily protein mRNA, complete cds
AT2G29500	1.46525609	3.38E-09	Arabidopsis thaliana chromosome 2, complete sequence
AT1G70500	1.508992645	3.51E-09	Arabidopsis thaliana pectin lyase-like protein mRNA, complete cds
AT4G35720	1.699951347	3.56E-09	Arabidopsis thaliana chromosome 4 sequence
AT2G02000	2.954052628	3.60E-09	Arabidopsis thaliana glutamate decarboxylase 3 mRNA, complete cds
AT4G23610	2.954052628	3.60E-09	Arabidopsis thaliana chromosome 4 sequence
AT5G60910	1.100844664	3.70E-09	Arabidopsis thaliana agamous-like MADS-box protein AGL8 mRNA, complete cds
AT5G65830	1.015453172	3.88E-09	Arabidopsis thaliana chromosome 5 sequence
AT4G22980	1.072355564	3.91E-09	Arabidopsis thaliana chromosome 4 sequence
AT5G13910	1.647721388	4.37E-09	Arabidopsis thaliana chromosome 5 sequence
AT1G06090	1.265996634	4.38E-09	Arabidopsis thaliana delta-9 desaturase-like 1 protein mRNA, complete cds
AT4G19191	1.922343768	4.53E-09	Arabidopsis thaliana chromosome 4 sequence
AT3G21320	2.793060751	4.60E-09	Arabidopsis thaliana uncharacterized protein mRNA, complete cds
AT3G61450	2.793060751	4.60E-09	Arabidopsis thaliana syntaxin-73 mRNA, complete cds
AT3G46090	2.313133721	4.97E-09	Arabidopsis thaliana chromosome 3, complete sequence
AT2G18540	5.015453172	5.01E-09	Arabidopsis thaliana cupin family protein mRNA, complete cds
AT5G14360	2.185378174	5.31E-09	Arabidopsis thaliana ubiquitin-like superfamily protein mRNA, complete cds
AT5G03180	1.064362773	5.35E-09	Arabidopsis thaliana RING/U-box domain-containing protein mRNA, complete cds
AT4G23215	2.659309362	5.57E-09	Arabidopsis thaliana chromosome 4 sequence
AT3G46700	1.461709402	5.83E-09	Arabidopsis thaliana UDP-glycosyltransferase 76E3 mRNA,

			complete cds
AT1G10880	5.969649483	6.41E-09	Arabidopsis thaliana core-2/I-branching beta-1,6-N-acetylglucosaminyltransferase family protein mRNA, complete cds
AT3G62990	5.969649483	6.41E-09	Arabidopsis thaliana chromosome 3, complete sequence
AT5G09980	2.545967889	6.47E-09	Arabidopsis thaliana elicitor peptide 4 mRNA, complete cds
AT5G02950	1.031050027	7.47E-09	Arabidopsis thaliana chromosome 5 sequence
AT3G23630	1.974811188	8.08E-09	Arabidopsis thaliana chromosome 3, complete sequence
AT5G58650	1.303698141	8.40E-09	Arabidopsis thaliana tyrosine-sulfated glycopeptide 1 mRNA, complete cds
AT5G42930	2.015453172	8.41E-09	Arabidopsis thaliana lipase class 3-like protein mRNA, complete cds
AT3G62730	3.0858425	8.53E-09	Arabidopsis thaliana uncharacterized protein mRNA, complete cds
AT4G16550	3.0858425	8.53E-09	Arabidopsis thaliana HSP20-like chaperone mRNA, complete cds
AT1G74830	2.22190405	8.65E-09	Arabidopsis thaliana uncharacterized protein mRNA, complete cds
AT3G23230	2.22190405	8.65E-09	Arabidopsis thaliana chromosome 3, complete sequence
AT5G55110	4.969649483	9.06E-09	Arabidopsis thaliana chromosome 5 sequence
AT1G66470	1.355682084	9.12E-09	Arabidopsis thaliana protein ROOT HAIR DEFECTIVE6 mRNA, complete cds
AT1G65690	1.287532718	1.02E-08	Arabidopsis thaliana late embryogenesis abundant (LEA) hydroxyproline-rich glycoprotein mRNA, complete cds
AT1G19210	3.300855391	1.08E-08	Arabidopsis thaliana chromosome 1 sequence
AT3G19270	1.426085493	1.10E-08	Arabidopsis thaliana abscisic acid 8'-hydroxylase 4 mRNA, complete cds
AT1G04370	5.922343768	1.11E-08	Arabidopsis thaliana chromosome 1 sequence
AT5G37760	5.922343768	1.11E-08	Arabidopsis thaliana chaperone DnaJ-domain containing protein mRNA, complete cds
AT1G11925	5.922343768	1.11E-08	Arabidopsis thaliana chromosome 1 sequence
AT4G27530	5.922343768	1.11E-08	Arabidopsis thaliana uncharacterized protein mRNA, complete cds
AT5G16920	5.922343768	1.11E-08	Arabidopsis thaliana chromosome 5 sequence
AT3G49520	5.922343768	1.11E-08	Arabidopsis thaliana chromosome 3, complete sequence
AT3G25655	5.922343768	1.11E-08	Arabidopsis thaliana chromosome 3, complete sequence

AT4G24010	1.883349636	1.16E-08	Arabidopsis thaliana cellulose synthase-like protein G1 mRNA, complete cds
AT4G23200	2.421445532	1.24E-08	Arabidopsis thaliana putative cysteine-rich receptor-like protein kinase 12 mRNA, complete cds
AT5G65870	1.200808214	1.26E-08	Arabidopsis thaliana putative phytosulfokines 5 precursor mRNA, complete cds
AT4G17970	1.049005732	1.43E-08	Arabidopsis thaliana aluminum-activated, malate transporter 12 mRNA, complete cds
AT5G62820	1.349872211	1.56E-08	Arabidopsis thaliana uncharacterized protein mRNA, complete cds
AT4G01535	5.873434168	1.94E-08	Arabidopsis thaliana uncharacterized protein mRNA, complete cds
AT5G06805.1	5.873434168	1.94E-08	Arabidopsis thaliana chromosome 5 sequence
AT5G45690	5.873434168	1.94E-08	Arabidopsis thaliana uncharacterized protein mRNA, complete cds
AT3G53160	1.634363005	2.01E-08	Arabidopsis thaliana chromosome 3, complete sequence
AT2G04100	1.264812641	2.09E-08	Arabidopsis thaliana MATE efflux family protein mRNA, complete cds
AT5G67520	1.193240292	2.12E-08	Arabidopsis thaliana adenosine-5'-phosphosulfate kinase 4 mRNA, complete cds
AT5G06090	1.972384451	2.21E-08	Arabidopsis thaliana glycerol-3-phosphate acyltransferase 7 mRNA, complete cds
AT5G14890	1.124574895	2.29E-08	Arabidopsis thaliana NHL domain-containing protein mRNA, complete cds
AT5G17370	1.29046022	2.43E-08	Arabidopsis thaliana transducin/WD40 domain-containing protein mRNA, complete cds
AT4G00080	1.343779039	2.67E-08	Arabidopsis thaliana chromosome 4 sequence
AT1G64160	3.015453172	2.73E-08	Arabidopsis thaliana chromosome 1 sequence
AT3G50800	1.539015128	2.75E-08	Arabidopsis thaliana chromosome 3, complete sequence
AT1G74420	1.693525078	2.84E-08	Arabidopsis thaliana fucosyltransferase 3 mRNA, complete cds
AT1G32880	4.873434168	2.97E-08	Arabidopsis thaliana armadillo/beta-catenin-like repeats-containing protein mRNA, complete cds
AT1G08290	1.023816645	2.99E-08	Arabidopsis thaliana WIP domain protein 3 mRNA, complete cds
AT1G19025	1.21598616	3.02E-08	Arabidopsis thaliana DNA repair metallo-beta-lactamase family protein

			mRNA, complete cds
AT5G45580	1.617489187	3.07E-08	Arabidopsis thaliana homeodomain-like superfamily protein mRNA, complete cds
AT1G09080	1.876050116	3.15E-08	Arabidopsis thaliana protein BIP3 mRNA, complete cds
AT1G29860	1.911617361	3.36E-08	Arabidopsis thaliana putative WRKY transcription factor 71 mRNA, complete cds
AT3G15440	5.822808094	3.38E-08	Arabidopsis thaliana chromosome 3, complete sequence
AT2G18190	5.822808094	3.38E-08	Arabidopsis thaliana P-loop containing nucleoside triphosphate hydrolases superfamily protein mRNA, complete cds
AT5G06080	5.822808094	3.38E-08	Arabidopsis thaliana LOB domain-containing protein 33 mRNA, complete cds
AT4G36160	1.385821622	3.49E-08	Arabidopsis thaliana NAC domain containing protein 76 mRNA, complete cds
AT4G25433	2.822808094	3.54E-08	Arabidopsis thaliana chromosome 4 sequence
AT3G01960	2.36595042	3.57E-08	Arabidopsis thaliana uncharacterized protein mRNA, complete cds
AT3G22410	1.023914751	3.58E-08	Arabidopsis thaliana Sec14p-like phosphatidylinositol transfer family protein mRNA, complete cds
AT1G16510	1.425737142	3.79E-08	Arabidopsis thaliana chromosome 1 sequence
AT4G36900	1.471132656	4.03E-08	Arabidopsis thaliana chromosome 4 sequence
AT4G34990	1.061549587	4.42E-08	Arabidopsis thaliana transcription factor MYB32 mRNA, complete cds
AT5G42610	1.482579183	4.66E-08	Arabidopsis thaliana uncharacterized protein mRNA, complete cds
AT2G01880	1.159288945	4.90E-08	Arabidopsis thaliana purple acid phosphatase 7 mRNA, complete cds
AT4G23880	1.372146685	5.10E-08	Arabidopsis thaliana chromosome 4 sequence
AT5G12300	1.024118034	5.14E-08	Arabidopsis thaliana chromosome 5 sequence
AT5G41380	1.88992229	5.38E-08	Arabidopsis thaliana CCT motif family protein mRNA, complete cds
ATCG00280	1.494621009	5.40E-08	Arabidopsis thaliana chloroplast DNA, complete genome, ecotype: Columbia
AT4G28390	1.132636712	5.58E-08	Arabidopsis thaliana ADP/ATP carrier 3 protein mRNA, complete cds
AT3G57460	1.161531432	5.85E-08	Arabidopsis thaliana catalytic/ metal ion binding / metalloendopeptidase/ zinc ion binding protein mRNA, complete cds
AT4G21020	5.770340675	5.92E-08	Arabidopsis thaliana Late embryogenesis abundant protein (LEA) family protein

			mRNA, complete cds
AT2G25470	2.788042676	6.24E-08	Arabidopsis thaliana receptor like protein 21 mRNA, complete cds
AT1G08860	2.256461272	6.35E-08	Arabidopsis thaliana protein BONZAI 3 mRNA, complete cds
AT3G49055	3.185378174	6.36E-08	Arabidopsis thaliana chromosome 3 hypothetical protein AT3G49055 mRNA, complete cds, alternatively spliced
AT5G57810	3.185378174	6.36E-08	Arabidopsis thaliana tetraspanin15 mRNA, complete cds
AT1G70920	1.656999202	6.78E-08	Arabidopsis thaliana homeobox-leucine zipper protein ATHB-18 mRNA, complete cds
AT2G38790	1.358340886	7.45E-08	Arabidopsis thaliana chromosome 2, complete sequence
AT1G67365	1.358340886	7.45E-08	Arabidopsis thaliana chromosome 1 sequence
AT5G05490	3.474884791	7.77E-08	Arabidopsis thaliana RAD21-like protein SYN1 mRNA, complete cds
AT4G21120	1.833614849	7.95E-08	Arabidopsis thaliana amino acid transporter 1 mRNA, complete cds
AT4G09820	1.833614849	7.95E-08	Arabidopsis thaliana transcription factor TT8 mRNA, complete cds
AT2G36780	2.941452591	8.67E-08	Arabidopsis thaliana chromosome 2, complete sequence
AT5G54165	3.922343768	8.77E-08	Arabidopsis thaliana chromosome 5 sequence
AT2G28500	1.905270255	9.18E-08	Arabidopsis thaliana LOB domain-containing protein 11 mRNA, complete cds
AT1G29060	1.123977629	9.27E-08	Arabidopsis thaliana Bet1-like protein mRNA, complete cds
AT1G66700	2.400117023	9.56E-08	Arabidopsis thaliana SABATH family methyltransferase PXMT1 mRNA, complete cds
AT2G03770	4.770340675	9.84E-08	Arabidopsis thaliana chromosome 2, complete sequence
AT4G39360	5.715892891	1.04E-07	Arabidopsis thaliana chromosome 4 sequence
AT1G21850	5.715892891	1.04E-07	Arabidopsis thaliana SKU5 similar 8 protein mRNA, complete cds
AT2G25540	5.715892891	1.04E-07	Arabidopsis thaliana cellulose synthase 10 mRNA, complete cds
AT1G20150	5.715892891	1.04E-07	Arabidopsis thaliana subtilisin-like serine endopeptidase family protein mRNA, complete cds
AT3G09450	1.638383523	1.04E-07	Arabidopsis thaliana uncharacterized protein mRNA, complete cds

AT2G23060	2.228446896	1.06E-07	Arabidopsis thaliana acyl-CoA N-acyltransferase-like protein mRNA, complete cds
AT5G15340	2.158411126	1.08E-07	Arabidopsis thaliana chromosome 5 sequence
AT1G36622	3.144736189	1.15E-07	Arabidopsis thaliana uncharacterized protein mRNA, complete cds
AT2G30395	1.780987919	1.15E-07	Arabidopsis thaliana ovate family protein 17 mRNA, complete cds
AT1G24147	1.044404546	1.42E-07	Arabidopsis thaliana uncharacterized protein mRNA, complete cds
AT1G07390	1.044404546	1.42E-07	Arabidopsis thaliana receptor like protein 1 mRNA, complete cds
AT4G30370	2.474884791	1.49E-07	Arabidopsis thaliana chromosome 4 sequence
AT3G16490	1.194423313	1.71E-07	Arabidopsis thaliana protein IQ-domain 26 mRNA, complete cds
AT1G54870	5.659309362	1.83E-07	Arabidopsis thaliana glucose and ribitol dehydrogenase homolog 1 mRNA, complete cds
AT5G41755.1	5.659309362	1.83E-07	Arabidopsis thaliana chromosome 5 sequence
AT2G46410	1.283942008	1.99E-07	Arabidopsis thaliana transcription factor CPC mRNA, complete cds
AT5G16770	1.162294561	1.99E-07	Arabidopsis thaliana myb domain protein 9 mRNA, complete cds
AT3G50030	3.102916014	2.06E-07	Arabidopsis thaliana uncharacterized protein mRNA, complete cds
AT2G31860	2.565650255	2.27E-07	Arabidopsis thaliana chromosome 2, complete sequence
AT2G16910	2.565650255	2.27E-07	Arabidopsis thaliana transcription factor ABORTED MICROSPORES mRNA, complete cds
AT5G57400	2.565650255	2.27E-07	Arabidopsis thaliana uncharacterized protein mRNA, complete cds
AT1G61280	1.600415673	2.45E-07	Arabidopsis thaliana phosphatidylinositol N-acetylglucosaminyltransferase subunit P mRNA, complete cds
AT3G20520	2.441717927	2.55E-07	Arabidopsis thaliana glycerophosphodiester phosphodiesterase-like protein mRNA, complete cds
AT1G02980	3.384686982	2.58E-07	Arabidopsis thaliana cullin 2 mRNA, complete cds
AT3G15270	1.430490672	2.65E-07	Arabidopsis thaliana squamosa promoter-binding-like protein 5 mRNA, complete cds

AT2G37210	1.941452591	2.67E-07	Arabidopsis thaliana cytokinin riboside 5'-monophosphate phosphoribohydrolase LOG3 mRNA, complete cds
AT2G41312	1.295561092	2.79E-07	Arabidopsis thaliana Full-length cDNA Complete sequence from clone GSLTSIL38ZE01 of Silique of strain col-0 of Arabidopsis thaliana (thale cress)
AT2G27280	3.822808094	2.97E-07	Arabidopsis thaliana Coiled-coil domain-containing protein 55 mRNA, complete cds
AT5G41710.1	3.822808094	2.97E-07	Arabidopsis thaliana chromosome 5 sequence
AT1G04445	3.822808094	2.97E-07	Arabidopsis thaliana chromosome 1 sequence
AT5G25830	1.069091136	3.23E-07	Arabidopsis thaliana GATA transcription factor 12 mRNA, complete cds
AT2G31345	5.600415673	3.24E-07	Arabidopsis thaliana chromosome 2, complete sequence
AT2G29470	4.659309362	3.28E-07	Arabidopsis thaliana glutathione S-transferase tau 3 mRNA, complete cds
AT4G30830	1.301757358	3.30E-07	Arabidopsis thaliana uncharacterized protein mRNA, complete cds
AT1G67030	1.152956696	3.32E-07	Arabidopsis thaliana chromosome 1 sequence
AT5G46590	1.664546011	3.53E-07	Arabidopsis thaliana NAC domain containing protein 96 mRNA, complete cds
AT5G15430	3.059847292	3.71E-07	Arabidopsis thaliana chromosome 5 sequence
AT3G01990	1.155383434	3.97E-07	Arabidopsis thaliana ACT domain-containing protein 6 mRNA, complete cds
AT3G50870	1.233358203	4.12E-07	Arabidopsis thaliana GATA transcription factor 18 mRNA, complete cds
AT2G40180	1.915917499	4.29E-07	Arabidopsis thaliana protein phosphatase 2C 5 mRNA, complete cds
AT5G24860	2.407770595	4.36E-07	Arabidopsis thaliana chromosome 5 sequence
AT1G73630	1.058521894	4.42E-07	Arabidopsis thaliana chromosome 1 sequence
AT3G16360	3.337381267	4.70E-07	Arabidopsis thaliana histidine-containing phosphotransfer protein 4 mRNA, complete cds
AT3G63050	2.015453172	4.73E-07	Arabidopsis thaliana chromosome 3, complete sequence
AT3G12720	2.140984055	4.90E-07	Arabidopsis thaliana myb domain protein 67 mRNA, complete cds
AT4G25000	1.744805582	4.90E-07	Arabidopsis thaliana alpha-amylase 1 mRNA, complete cds
AT5G58680	1.543832145	4.95E-07	Arabidopsis thaliana chromosome 5 sequence

AT4G23340	1.776265509	5.40E-07	Arabidopsis thaliana chromosome 4 sequence
AT1G61800	3.770340675	5.46E-07	Arabidopsis thaliana glucose-6-phosphate/phosphate translocator 2 mRNA, complete cds
AT1G19968	3.770340675	5.46E-07	Arabidopsis thaliana chromosome 1 sequence
AT1G47980	5.539015128	5.75E-07	Arabidopsis thaliana uncharacterized protein mRNA, complete cds
AT3G43250	5.539015128	5.75E-07	Arabidopsis thaliana chromosome 3, complete sequence
AT4G08145.1	5.539015128	5.75E-07	Arabidopsis thaliana chromosome 4 sequence
AT1G58430	4.600415673	6.02E-07	Arabidopsis thaliana anther-specific proline-rich protein RXF26 mRNA, complete cds
AT5G53750	1.04787465	6.04E-07	Arabidopsis thaliana CBS domain-containing protein mRNA, complete cds
AT4G26120	1.580237791	6.48E-07	Arabidopsis thaliana regulatory protein NPR2 mRNA, complete cds
AT5G66800	1.182103042	6.89E-07	Arabidopsis thaliana uncharacterized protein mRNA, complete cds
AT1G23760	1.30668447	7.89E-07	Arabidopsis thaliana putative polygalacturonase non-catalytic subunit JP630 mRNA, complete cds
AT1G01500	1.166012849	8.11E-07	Arabidopsis thaliana Erythronate-4-phosphate dehydrogenase-like protein mRNA, complete cds
AT1G04500	2.780987919	8.57E-07	Arabidopsis thaliana CCT motif-containing protein mRNA, complete cds
AT3G48790	2.780987919	8.57E-07	Arabidopsis thaliana Pyridoxal phosphate (PLP)-dependent transferases superfamily protein mRNA, complete cds
AT5G63300	2.780987919	8.57E-07	Arabidopsis thaliana ribosomal protein S21 family protein mRNA, complete cds
AT4G34550	2.780987919	8.57E-07	Arabidopsis thaliana chromosome 4 sequence
AT1G61550	1.645503563	9.47E-07	Arabidopsis thaliana G-type lectin S-receptor-like serine/threonine-protein kinase mRNA, complete cds
AT3G21410	5.474884791	1.02E-06	Arabidopsis thaliana chromosome 3, complete sequence
AT3G50290	2.600415673	1.03E-06	Arabidopsis thaliana chromosome 3, complete sequence
AT4G27460	1.863450079	1.10E-06	Arabidopsis thaliana cystathionine beta-synthase domain-containing protein mRNA, complete cds

AT2G21820	4.539015128	1.10E-06	Arabidopsis thaliana chromosome 2, complete sequence
AT5G45116.1	4.539015128	1.10E-06	Arabidopsis thaliana chromosome 5 sequence
AT1G07410	1.026341489	1.12E-06	Arabidopsis thaliana RAB GTPase homolog A2B mRNA, complete cds
AT3G62610	2.456025764	1.17E-06	Arabidopsis thaliana myb domain protein 11 mRNA, complete cds
AT4G22960	2.456025764	1.17E-06	Arabidopsis thaliana uncharacterized protein mRNA, complete cds
AT5G49620	1.908537969	1.18E-06	Arabidopsis thaliana myb domain protein 78 mRNA, complete cds
AT5G28237	2.969649483	1.19E-06	Arabidopsis thaliana tryptophan synthase beta chain-like protein mRNA, complete cds
AT1G65240	2.969649483	1.19E-06	Arabidopsis thaliana aspartyl protease family protein mRNA, complete cds
AT5G09990	2.337381267	1.26E-06	Arabidopsis thaliana elicitor peptide 5 mRNA, complete cds
AT1G76410	1.137443697	1.33E-06	Arabidopsis thaliana chromosome 1 sequence
AT2G17830	1.137443697	1.33E-06	Arabidopsis thaliana chromosome 2, complete sequence
AT1G01725	1.337381267	1.54E-06	Arabidopsis thaliana uncharacterized protein mRNA, complete cds
AT4G36700	3.237845594	1.55E-06	Arabidopsis thaliana cupin family protein mRNA, complete cds
AT3G01175	1.557980407	1.72E-06	Arabidopsis thaliana uncharacterized protein mRNA, complete cds
AT2G01430	1.383184957	1.72E-06	Arabidopsis thaliana homeobox-leucine zipper protein ATHB-17 mRNA, complete cds
AT1G08500	1.051077082	1.80E-06	Arabidopsis thaliana early nodulin-like protein 18 mRNA, complete cds
AT3G29020	5.407770595	1.83E-06	Arabidopsis thaliana myb domain protein 110 mRNA, complete cds
AT2G22426	5.407770595	1.83E-06	Arabidopsis thaliana chromosome 2, complete sequence
AT1G27565	5.407770595	1.83E-06	Arabidopsis thaliana uncharacterized protein mRNA, complete cds
AT1G02310	2.417551616	2.00E-06	Arabidopsis thaliana mannan endo-1,4-beta-mannosidase 1 mRNA, complete cds
AT3G12955	2.417551616	2.00E-06	Arabidopsis thaliana chromosome 3, complete sequence
AT5G63580	1.093455684	2.06E-06	Arabidopsis thaliana flavonol synthase 2 mRNA, complete cds

AT2G31980	2.300855391	2.14E-06	Arabidopsis thaliana cysteine proteinase inhibitor 2 mRNA, complete cds
AT5G19097.1	1.450390229	2.17E-06	Arabidopsis thaliana chromosome 5 sequence
AT5G48100	2.203080176	2.22E-06	Arabidopsis thaliana laccase-15 mRNA, complete cds
AT5G42440	1.027041147	2.32E-06	Arabidopsis thaliana chromosome 5 sequence
AT1G22490	1.185378174	2.36E-06	Arabidopsis thaliana transcription factor bHLH94 mRNA, complete cds
AT5G55900	1.185378174	2.36E-06	Arabidopsis thaliana Sucrase/ferredoxin-like family protein mRNA, complete cds
AT1G66920	1.09488764	2.47E-06	Arabidopsis thaliana putative serine/threonine protein kinase mRNA, complete cds
AT2G24430	1.328611058	2.64E-06	Arabidopsis thaliana NAC domain containing protein 38 mRNA, complete cds
AT1G65570	2.693525078	2.66E-06	Arabidopsis thaliana polygalacturonase family protein mRNA, complete cds
AT3G14510	2.517953513	3.10E-06	Arabidopsis thaliana putative geranylgeranyl pyrophosphate synthase 8 mRNA, complete cds
AT4G06536	1.899975955	3.25E-06	Arabidopsis thaliana chromosome 4 sequence
AT3G25180	5.337381267	3.29E-06	Arabidopsis thaliana cytochrome P450, family 82, subfamily G, polypeptide 1 mRNA, complete cds
AT1G72100	5.337381267	3.29E-06	Arabidopsis thaliana late embryogenesis abundant domain-containing protein mRNA, complete cds
AT1G78390	5.337381267	3.29E-06	Arabidopsis thaliana chromosome 1 sequence
AT1G45140.1	5.337381267	3.29E-06	Arabidopsis thaliana chromosome 1 sequence
AT5G22470	5.337381267	3.29E-06	Arabidopsis thaliana poly [ADP-ribose] polymerase 3 mRNA, complete cds
AT5G09610	5.337381267	3.29E-06	Arabidopsis thaliana chromosome 5 sequence
AT5G45113	5.337381267	3.29E-06	Arabidopsis thaliana chromosome 5 sequence
AT5G02220	3.600415673	3.40E-06	Arabidopsis thaliana chromosome 5 sequence
AT1G66390	3.600415673	3.40E-06	Arabidopsis thaliana putative transcription factor (MYB90) mRNA, MYB90-Col allele, complete cds
AT3G63095	3.600415673	3.40E-06	Arabidopsis thaliana chromosome 3, complete sequence
AT3G20160	3.600415673	3.40E-06	Arabidopsis thaliana chromosome 3, complete sequence
AT1G29290	2.378023252	3.42E-06	Arabidopsis thaliana chromosome 1 sequence

AT1G67270	1.954052628	3.45E-06	Arabidopsis thaliana zinc-finger domain of monoamine-oxidase A repressor R1 protein mRNA, complete cds
AT1G16825	1.386290868	3.48E-06	Arabidopsis thaliana reticulon-like protein mRNA, complete cds
AT4G30097	1.003957534	3.56E-06	Arabidopsis thaliana chromosome 4 sequence
AT3G61900	1.346659081	3.68E-06	Arabidopsis thaliana SAUR-like auxin-responsive protein mRNA, complete cds
AT3G51940	1.114988846	3.68E-06	Arabidopsis thaliana uncharacterized protein mRNA, complete cds
AT3G30460	2.0858425	3.69E-06	Arabidopsis thaliana chromosome 3, complete sequence
AT4G23450	1.053588301	3.73E-06	Arabidopsis thaliana C3H2C3-type RING E3 Ub ligase mRNA, complete cds
AT1G07850	4.407770595	3.75E-06	Arabidopsis thaliana uncharacterized protein mRNA, complete cds
AT3G56660	4.407770595	3.75E-06	Arabidopsis thaliana basic region/leucine zipper motif protein 49 mRNA, complete cds
AT2G20150	2.873434168	3.80E-06	Arabidopsis thaliana uncharacterized protein mRNA, complete cds
AT4G40020	2.873434168	3.80E-06	Arabidopsis thaliana chromosome 4 sequence
AT4G21200	2.873434168	3.80E-06	Arabidopsis thaliana gibberellin 2-beta-dioxygenase 8 mRNA, complete cds
ATCG00350	1.067920592	3.91E-06	Arabidopsis thaliana chloroplast DNA, complete genome, ecotype: Columbia
AT5G66670	1.780987919	4.42E-06	Arabidopsis thaliana chromosome 5 sequence
AT4G27360	1.533301477	4.53E-06	Arabidopsis thaliana Dynein light chain type 1 family protein mRNA, complete cds
AT5G01180	2.647721388	4.67E-06	Arabidopsis thaliana peptide transporter PTR5 mRNA, complete cds
AT1G76640	3.13093039	5.10E-06	Arabidopsis thaliana chromosome 1 sequence
AT1G23450	1.869602306	5.22E-06	Arabidopsis thaliana pentatricopeptide repeat-containing protein mRNA, complete cds
AT1G65730	1.119075803	5.29E-06	Arabidopsis thaliana putative metal-nicotianamine transporter YSL7 mRNA, complete cds
AT4G21920	2.474884791	5.36E-06	Arabidopsis thaliana chromosome 4 sequence
AT3G11440	1.13830992	5.45E-06	Arabidopsis thaliana myb domain protein 65 mRNA, complete cds
AT4G37690	1.922343768	5.57E-06	Arabidopsis thaliana chromosome 4 sequence
AT2G21180	1.069900956	5.64E-06	Arabidopsis thaliana chromosome 2,

			complete sequence
AT2G33100	2.337381267	5.83E-06	Arabidopsis thaliana cellulose synthase-like protein D1 mRNA, complete cds
AT5G05365	1.491891216	5.92E-06	Arabidopsis thaliana Heavy metal transport/detoxification superfamily protein mRNA, complete cds
AT3G13950	5.263380686	5.92E-06	Arabidopsis thaliana uncharacterized protein mRNA, complete cds
AT1G80090	5.263380686	5.92E-06	Arabidopsis thaliana SNF1-related protein kinase regulatory subunit gamma-like PV42b mRNA, complete cds
AT4G35690	5.263380686	5.92E-06	Arabidopsis thaliana chromosome 4 sequence
AT1G01280	5.263380686	5.92E-06	Arabidopsis thaliana cytochrome P450, family 703, subfamily A, polypeptide 2 mRNA, complete cds
AT2G24140	1.378023252	6.02E-06	Arabidopsis thaliana uncharacterized protein mRNA, complete cds
AT4G17980	2.822808094	6.77E-06	Arabidopsis thaliana NAC domain containing protein 71 mRNA, complete cds
AT5G27845.1	2.822808094	6.77E-06	Arabidopsis thaliana chromosome 5 sequence
AT4G30180	1.600415673	6.80E-06	Arabidopsis thaliana chromosome 4 sequence
AT1G48605	1.600415673	6.80E-06	Arabidopsis thaliana putative phosphopantothenoylcysteine decarboxylase mRNA, complete cds
AT5G42710	1.210469155	6.83E-06	Arabidopsis thaliana uncharacterized protein mRNA, complete cds
AT2G04460.1	4.337381267	6.93E-06	Arabidopsis thaliana chromosome 2, complete sequence
AT3G11980	4.337381267	6.93E-06	Arabidopsis thaliana fatty acyl-CoA reductase 2 mRNA, complete cds
AT3G58270	1.165695808	7.99E-06	Arabidopsis thaliana phospholipase-like protein (PEARLI 4) with TRAF-like domain mRNA, complete cds
AT2G46950	1.88992229	8.98E-06	Arabidopsis thaliana cytochrome P450, family 709, subfamily B, polypeptide 2 mRNA, complete cds
AT3G18715	1.88992229	8.98E-06	Arabidopsis thaliana chromosome 3, complete sequence
AT2G23270	2.430490672	9.23E-06	Arabidopsis thaliana chromosome 2, complete sequence
AT5G02580	1.948338977	9.54E-06	Arabidopsis thaliana uncharacterized protein mRNA, complete cds
AT1G77200	1.948338977	9.54E-06	Arabidopsis thaliana chromosome 1 sequence

AT1G53700	1.169258509	9.57E-06	Arabidopsis thaliana chromosome 1 sequence
AT4G22214	2.295561092	9.91E-06	Arabidopsis thaliana defensin-like protein 99 mRNA, complete cds
AT1G33430	2.015453172	9.97E-06	Arabidopsis thaliana putative beta-1,3-galactosyltransferase 8 mRNA, complete cds
AT3G22560	2.185378174	1.02E-05	Arabidopsis thaliana chromosome 3, complete sequence
AT2G15310	1.042933909	1.06E-05	Arabidopsis thaliana ADP-ribosylation factor B1A mRNA, complete cds
AT5G58080	5.185378174	1.07E-05	Arabidopsis thaliana response regulator 18 mRNA, complete cds
AT1G09157	5.185378174	1.07E-05	Arabidopsis thaliana chromosome 1 sequence
AT5G52415	5.185378174	1.07E-05	Arabidopsis thaliana chromosome 5 sequence
AT2G31210	5.185378174	1.07E-05	Arabidopsis thaliana transcription factor bHLH91 mRNA, complete cds
AT1G54120	1.723272421	1.09E-05	Arabidopsis thaliana chromosome 1 sequence
AT4G16750	1.05809751	1.12E-05	Arabidopsis thaliana chromosome 4 sequence
AT5G53990	1.430490672	1.14E-05	Arabidopsis thaliana chromosome 5 sequence
AT2G26610	4.263380686	1.28E-05	Arabidopsis thaliana uncharacterized protein mRNA, complete cds
AT5G06230	1.628430049	1.35E-05	Arabidopsis thaliana protein trichome birefringence-like 9 mRNA, complete cds
AT2G33585	1.075574165	1.41E-05	Arabidopsis thaliana uncharacterized protein mRNA, complete cds
AT5G25910	2.551506073	1.43E-05	Arabidopsis thaliana receptor like protein 52 mRNA, complete cds
AT4G17680	1.133634599	1.57E-05	Arabidopsis thaliana SBP (S-ribonuclease binding protein) family protein mRNA, complete cds
AT2G30400	1.307219297	1.59E-05	Arabidopsis thaliana chromosome 2, complete sequence
AT1G01670	1.237845594	1.65E-05	Arabidopsis thaliana U-box domain-containing protein 56 mRNA, complete cds
AT5G05430	3.015453172	1.67E-05	Arabidopsis thaliana RNA-binding protein mRNA, complete cds
AT1G24130	2.25249237	1.68E-05	Arabidopsis thaliana chromosome 1 sequence
AT4G18425	1.159843082	1.93E-05	Arabidopsis thaliana chromosome 4 sequence
AT1G28360	1.278487578	1.94E-05	Arabidopsis thaliana chromosome 1 sequence
AT1G68765	5.102916014	1.95E-05	Arabidopsis thaliana chromosome 1 sequence
AT1G33760	5.102916014	1.95E-05	Arabidopsis thaliana chromosome 1 sequence
AT5G65274	5.102916014	1.95E-05	Arabidopsis thaliana ARP2/3 complex

			subunit p16-Arc mRNA, complete cds
AT5G07230	5.102916014	1.95E-05	Arabidopsis thaliana Tapetum-specific protein A9 mRNA, complete cds
AT3G15800	1.244271863	1.97E-05	Arabidopsis thaliana glycosyl hydrolase superfamily protein mRNA, complete cds
AT1G14860	1.50088	2.08E-05	Arabidopsis thaliana nudix hydrolase 18 mRNA, complete cds
AT5G67620	1.096983058	2.11E-05	Arabidopsis thaliana mRNA for hypothetical protein, complete cds, clone: RAFL14-69-L22
AT3G20660	1.117332786	2.19E-05	Arabidopsis thaliana organic cation/carnitine transporter4 mRNA, complete cds
AT4G37990	1.001778236	2.23E-05	Arabidopsis thaliana cinnamyl alcohol dehydrogenase 8 mRNA, complete cds
AT5G27060	2.50088	2.48E-05	Arabidopsis thaliana chromosome 5 sequence
AT1G48000	1.877949649	2.49E-05	Arabidopsis thaliana myb domain protein 112 mRNA, complete cds
AT1G54020	1.337381267	2.64E-05	Arabidopsis thaliana GDSL esterase/lipase mRNA, complete cds
AT4G28040	2.337381267	2.71E-05	Arabidopsis thaliana nodulin MtN21-like transporter family protein mRNA, complete cds
AT1G61470	2.015453172	2.78E-05	Arabidopsis thaliana chromosome 1 sequence
AT2G04495	2.015453172	2.78E-05	Arabidopsis thaliana chromosome 2, complete sequence
AT3G53150	2.20809825	2.83E-05	Arabidopsis thaliana chromosome 3, complete sequence
AT5G07700	2.954052628	3.01E-05	Arabidopsis thaliana myb domain protein 76 mRNA, complete cds
AT1G19230	1.030403514	3.02E-05	Arabidopsis thaliana riboflavin synthase-like superfamily protein mRNA, complete cds
AT3G07730	1.030403514	3.02E-05	Arabidopsis thaliana chromosome 3, complete sequence
AT1G05280	1.231181863	3.36E-05	Arabidopsis thaliana uncharacterized protein mRNA, complete cds
AT1G61070	5.015453172	3.56E-05	Arabidopsis thaliana defensin-like protein mRNA, complete cds
AT1G18310	5.015453172	3.56E-05	Arabidopsis thaliana glycosyl hydrolase family 81 protein mRNA, complete cds
AT3G15534	5.015453172	3.56E-05	Arabidopsis thaliana chromosome 3, complete sequence
AT3G05770	5.015453172	3.56E-05	Arabidopsis thaliana uncharacterized protein mRNA, complete cds

AT1G32910	5.015453172	3.56E-05	Arabidopsis thaliana HXXXD-type acyl-transferase-like protein mRNA, complete cds
AT5G43935	5.015453172	3.56E-05	Arabidopsis thaliana flavonol synthase 6 mRNA, complete cds
AT3G50940	5.015453172	3.56E-05	Arabidopsis thaliana cytochrome BC1 synthesis-like protein mRNA, complete cds
AT1G58889.1	1.495446114	3.64E-05	Arabidopsis thaliana DNA, retrotransposon:AtRE1, complete sequence, ecotype: Niederzenz
AT1G04330	1.788042676	3.67E-05	Arabidopsis thaliana chromosome 1 sequence
AT5G36925	1.125077664	3.79E-05	Arabidopsis thaliana uncharacterized protein mRNA, complete cds
AT2G20510	3.337381267	3.89E-05	Arabidopsis thaliana translocase inner membrane subunit 44-1 mRNA, complete cds
AT5G37800	1.841423773	4.00E-05	Arabidopsis thaliana RHD SIX-LIKE 1 mRNA, complete cds
AT3G03200	1.430490672	4.03E-05	Arabidopsis thaliana NAC domain containing protein 45 mRNA, complete cds
AT4G12350	2.44841258	4.30E-05	Arabidopsis thaliana myb domain protein 42 mRNA, complete cds
AT2G47150	4.102916014	4.44E-05	Arabidopsis thaliana Rossmann-fold NAD(P)-binding domain-containing protein mRNA, complete cds
AT4G17780	4.102916014	4.44E-05	Arabidopsis thaliana putative F-box protein mRNA, complete cds
AT2G47810	1.974811188	4.55E-05	Arabidopsis thaliana chromosome 2, complete sequence
AT3G11570	1.974811188	4.55E-05	Arabidopsis thaliana protein trichome birefringence-like 8 mRNA, complete cds
AT3G04040	1.667529869	4.67E-05	Arabidopsis thaliana uncharacterized protein mRNA, complete cds
AT1G77855	1.152956696	4.67E-05	Arabidopsis thaliana uncharacterized protein mRNA, complete cds
AT5G14070	2.059847292	4.71E-05	Arabidopsis thaliana chromosome 5 sequence
AT2G37880	1.542700175	4.86E-05	Arabidopsis thaliana chromosome 2, complete sequence
AT4G33790	1.00034628	5.60E-05	Arabidopsis thaliana fatty acyl-CoA reductase CER4 mRNA, complete cds
AT4G03480	1.489384361	6.38E-05	Arabidopsis thaliana Ankyrin repeat family protein mRNA, complete cds
AT1G61275	1.803949067	6.39E-05	Arabidopsis thaliana chromosome 1 sequence
AT3G19920	4.922343768	6.54E-05	Arabidopsis thaliana uncharacterized protein mRNA, complete cds

AT4G25380	4.922343768	6.54E-05	Arabidopsis thaliana chromosome 4 sequence
AT3G08750	4.922343768	6.54E-05	Arabidopsis thaliana chromosome 3, complete sequence
AT1G66370	4.922343768	6.54E-05	Arabidopsis thaliana transcription factor MYB113 mRNA, complete cds
AT4G28460	2.600415673	6.62E-05	Arabidopsis thaliana chromosome 4 sequence
AT3G22961	2.600415673	6.62E-05	Arabidopsis thaliana chromosome 3, complete sequence
AT4G23150	1.863450079	6.94E-05	Arabidopsis thaliana cysteine-rich receptor-like protein kinase 7 mRNA, complete cds
AT1G02230	1.863450079	6.94E-05	Arabidopsis thaliana NAC domain-containing protein 4 mRNA, complete cds
AT1G17147	1.068564509	7.02E-05	Arabidopsis thaliana chromosome 1 sequence
AT3G13220	3.263380686	7.15E-05	Arabidopsis thaliana ABC transporter G family member 26 mRNA, complete cds
AT5G66020	3.263380686	7.15E-05	Arabidopsis thaliana phosphoinositide phosphatase SAC6 mRNA, complete cds
AT5G55250	1.015453172	7.17E-05	Arabidopsis thaliana IAA carboxylmethyltransferase 1 mRNA, complete cds
AT3G05820	1.634363005	7.25E-05	Arabidopsis thaliana alkaline/neutral invertase H mRNA, complete cds
AT3G02310	1.634363005	7.25E-05	Arabidopsis thaliana developmental protein SEPALLATA 2 mRNA, complete cds
AT2G46750	1.089453754	7.34E-05	Arabidopsis thaliana D-arabinono-1,4-lactone oxidase-like protein mRNA, complete cds
AT4G21340	1.932991012	7.42E-05	Arabidopsis thaliana transcription factor bHLH103 mRNA, complete cds
AT4G01533	2.393964796	7.43E-05	Arabidopsis thaliana Full-length cDNA Complete sequence from clone GSLTPGH70ZB05 of Hormone Treated Callus of strain col-0 of Arabidopsis thaliana (thale cress)
AT1G74290	1.363376476	7.53E-05	Arabidopsis thaliana esterase/lipase/thioesterase family protein mRNA, complete cds
AT1G02030	2.237845594	7.85E-05	Arabidopsis thaliana chromosome 1 sequence
AT4G32630	4.015453172	8.28E-05	Arabidopsis thaliana ArfGap/RecO-like zinc finger domain-containing protein mRNA, complete cds
AT1G63030	4.015453172	8.28E-05	Arabidopsis thaliana chromosome 1 sequence
AT1G70185	4.015453172	8.28E-05	Arabidopsis thaliana chromosome 1 sequence
AT1G21550	1.539015128	8.53E-05	Arabidopsis thaliana chromosome 1 sequence

AT1G73040	1.140984055	9.45E-05	Arabidopsis thaliana Mannose-binding lectin superfamily protein mRNA, complete cds
AT5G46830	1.460238015	9.60E-05	Arabidopsis thaliana chromosome 5 sequence
AT5G51451	1.169781319	9.65E-05	Arabidopsis thaliana root meristem growth factor 5 mRNA, complete cds
AT4G15975	1.393964796	0.000104485	Arabidopsis thaliana chromosome 4 sequence
AT3G11050	1.822808094	0.000111404	Arabidopsis thaliana ferritin 2 mRNA, complete cds
AT3G04530	1.482579183	0.000111824	Arabidopsis thaliana phosphoenolpyruvate carboxylase kinase 2 mRNA, complete cds
AT5G56050	1.174651767	0.000115738	Arabidopsis thaliana chromosome 5 sequence
AT3G53040	2.539015128	0.000116144	Arabidopsis thaliana putative late embryogenesis abundant protein mRNA, complete cds
AT3G60930.1	2.539015128	0.000116144	Arabidopsis thaliana At3g60930 mRNA for unknown protein, complete cds, clone: RAFL21-22-C17
AT1G56150	1.20809825	0.000116883	Arabidopsis thaliana chromosome 1 sequence
AT5G50800	1.20809825	0.000116883	Arabidopsis thaliana bidirectional sugar transporter SWEET13 mRNA, complete cds
AT4G11370	1.88992229	0.000120333	Arabidopsis thaliana chromosome 4 sequence
AT3G03660	1.88992229	0.000120333	Arabidopsis thaliana WUSCHEL related homeobox 11 mRNA, complete cds
AT1G63870	4.822808094	0.000120588	Arabidopsis thaliana TIR-NBS-LRR class disease resistance protein mRNA, complete cds
AT2G17680	4.822808094	0.000120588	Arabidopsis thaliana chromosome 2, complete sequence
AT5G53190	4.822808094	0.000120588	Arabidopsis thaliana bidirectional sugar transporter SWEET3 mRNA, complete cds
AT4G33290	4.822808094	0.000120588	Arabidopsis thaliana chromosome 4 sequence
AT5G43030	1.411381849	0.000122776	Arabidopsis thaliana chromosome 5 sequence
AT2G28105	1.969649483	0.000127607	Arabidopsis thaliana uncharacterized protein mRNA, complete cds
AT4G15236	1.969649483	0.000127607	Arabidopsis thaliana ABC transporter G family member 43 mRNA, complete cds
AT1G71330	1.095623521	0.000127676	Arabidopsis thaliana non-intrinsic ABC protein 5 mRNA, complete cds
AT5G67050	2.337381267	0.000127813	Arabidopsis thaliana alpha/beta-Hydrolases superfamily protein mRNA, complete cds

AT1G51820	1.507306269	0.00012976	Arabidopsis thaliana putative LRR receptor-like serine/threonine protein kinase mRNA, complete cds
AT4G05170	3.185378174	0.000131152	Arabidopsis thaliana transcription factor bHLH114 mRNA, complete cds
AT5G65130	3.185378174	0.000131152	Arabidopsis thaliana chromosome 5 sequence
AT4G08555	3.185378174	0.000131152	Arabidopsis thaliana chromosome 4 sequence
AT1G78955	1.120806173	0.000132387	Arabidopsis thaliana camelliol C synthase 1 mRNA, complete cds
AT2G04515	2.185378174	0.000132712	Arabidopsis thaliana chromosome 2, complete sequence
AT3G01513	1.254240032	0.000139125	Arabidopsis thaliana chromosome 3, complete sequence
AT3G21680	1.214761981	0.00013986	Arabidopsis thaliana chromosome 3, complete sequence
AT1G10530	1.430490672	0.000143963	Arabidopsis thaliana uncharacterized protein mRNA, complete cds
AT1G33102	1.430490672	0.000143963	Arabidopsis thaliana chromosome 1 sequence
AT5G43770	3.922343768	0.000154701	Arabidopsis thaliana chromosome 5 sequence
AT3G17320	3.922343768	0.000154701	Arabidopsis thaliana chromosome 3, complete sequence
AT5G46417	3.922343768	0.000154701	Arabidopsis thaliana chromosome 5 sequence
AT1G52990	3.922343768	0.000154701	Arabidopsis thaliana thioredoxin family protein mRNA, complete cds
AT2G36540	3.922343768	0.000154701	Arabidopsis thaliana Haloacid dehalogenase-like hydrolase (HAD) superfamily protein mRNA, complete cds
AT4G26680	1.123977629	0.000159146	Arabidopsis thaliana chromosome 4 sequence
AT4G21323	1.310909056	0.000161837	Arabidopsis thaliana Subtilase family protein mRNA, complete cds
AT1G01680	1.263380686	0.000165944	Arabidopsis thaliana U-box domain-containing protein 54 mRNA, complete cds
AT1G09880	1.451552287	0.000168387	Arabidopsis thaliana rhamnogalacturonate lyase-like protein mRNA, complete cds
AT5G55570	1.451552287	0.000168387	Arabidopsis thaliana uncharacterized protein mRNA, complete cds
AT5G21280	1.05392732	0.000168793	Arabidopsis thaliana hydroxyproline-rich glycoprotein family protein mRNA, complete cds

AT5G48390	1.565650255	0.000172585	Arabidopsis thaliana ZIP4-like protein mRNA, complete cds
AT5G50790	1.780987919	0.000177975	Arabidopsis thaliana bidirectional sugar transporter SWEET10 mRNA, complete cds
AT5G26730	1.323575468	0.000192071	Arabidopsis thaliana Fasciclin-like arabinogalactan family protein mRNA, complete cds
AT1G03020	1.323575468	0.000192071	Arabidopsis thaliana chromosome 1 sequence
AT5G52390	1.323575468	0.000192071	Arabidopsis thaliana PAR1 protein mRNA, complete cds
AT1G76470	1.845528171	0.000194279	Arabidopsis thaliana NAD(P)-binding Rossmann-fold superfamily protein mRNA, complete cds
AT1G51190	1.157472177	0.00019636	Arabidopsis thaliana AP2-like ethylene-responsive transcription factor PLT2 mRNA, complete cds
AT3G42800	1.474884791	0.000196387	Arabidopsis thaliana uncharacterized protein mRNA, complete cds
AT5G64450	2.474884791	0.000202994	Arabidopsis thaliana uncharacterized protein mRNA, complete cds
AT5G20810	2.474884791	0.000202994	Arabidopsis thaliana SAUR-like auxin-responsive protein mRNA, complete cds
AT3G59740	2.474884791	0.000202994	Arabidopsis thaliana chromosome 3, complete sequence
AT3G19430	1.054981537	0.000203314	Arabidopsis thaliana late embryogenesis abundant protein-like protein mRNA, complete cds
AT5G20710	1.922343768	0.000208495	Arabidopsis thaliana beta-galactosidase 7 mRNA, complete cds
AT5G63390	1.922343768	0.000208495	Arabidopsis thaliana O-fucosyltransferase family protein mRNA, complete cds
AT2G25820	1.922343768	0.000208495	Arabidopsis thaliana chromosome 2, complete sequence
AT1G29195	1.077737451	0.000213367	Arabidopsis thaliana chromosome 1 sequence
AT3G15536	4.715892891	0.000223659	Arabidopsis thaliana chromosome 3, complete sequence
AT1G67990	4.715892891	0.000223659	Arabidopsis thaliana tapetum-specific methyltransferase 1 mRNA, complete cds
AT5G13380	4.715892891	0.000223659	Arabidopsis thaliana auxin-responsive GH3 family protein mRNA, complete cds
AT1G69500	4.715892891	0.00022365	Arabidopsis thaliana cytochrome P450,

		9	family 704, subfamily B, polypeptide 1 mRNA, complete cds
AT5G09210	4.715892891	0.00022365 9	Arabidopsis thaliana GC-rich sequence DNA-binding factor-like protein mRNA, complete cds
AT1G04560	4.715892891	0.00022365 9	Arabidopsis thaliana AWPM-19-like family protein mRNA, complete cds
AT3G51590	4.715892891	0.00022365 9	Arabidopsis thaliana non-specific lipid-transfer protein 12 mRNA, complete cds
AT5G40260	4.715892891	0.00022365 9	Arabidopsis thaliana protein RUPTURED POLLEN GRAIN 1 mRNA, complete cds
AT3G49070	1.237845594	0.00023924 8	Arabidopsis thaliana chromosome 3, complete sequence
AT3G15740	3.102916014	0.00024025 6	Arabidopsis thaliana chromosome 3, complete sequence
AT4G37140	3.102916014	0.00024025 6	Arabidopsis thaliana putative inactive methylesterase 20 mRNA, complete cds
AT3G56600	3.102916014	0.00024025 6	Arabidopsis thaliana phosphatidylinositol 4-kinase gamma 8 mRNA, complete cds
AT5G52020	1.167456266	0.00028317 4	Arabidopsis thaliana chromosome 5 sequence
AT1G27720	1.246778719	0.00028593 4	Arabidopsis thaliana TBP-associated factor 4B mRNA, complete cds
AT5G48175	3.822808094	0.00028970 8	Arabidopsis thaliana uncharacterized protein mRNA, complete cds
AT2G27220	3.822808094	0.00028970 8	Arabidopsis thaliana BEL1-like homeodomain 5 mRNA, complete cds
AT2G13960	1.562940968	0.00030442 5	Arabidopsis thaliana homeodomain-like superfamily protein mRNA, complete cds
AT2G29110	1.799724481	0.000311997	Arabidopsis thaliana glutamate receptor 2.8 mRNA, complete cds
AT1G20350	1.799724481	0.000311997	Arabidopsis thaliana chromosome 1 sequence
AT4G23590	1.015453172	0.00031791 2	Arabidopsis thaliana probable aminotransferase TAT4 mRNA, complete cds
AT3G23610	1.035917275	0.00033727 2	Arabidopsis thaliana dual specificity protein phosphatase 1 mRNA, complete cds
AT1G32690	1.600415673	0.00034867 1	Arabidopsis thaliana chromosome 1 sequence
AT4G17660	2.407770595	0.00035329 6	Arabidopsis thaliana protein kinase family protein mRNA, complete cds
AT5G39720	2.407770595	0.00035329 6	Arabidopsis thaliana avirulence induced protein 2 like protein mRNA, complete cds
AT1G29230	1.058521894	0.00035584 4	Arabidopsis thaliana chromosome 1 sequence

AT3G04640	1.962985753	0.000360602	Arabidopsis thaliana chromosome 3, complete sequence
AT1G73510	1.962985753	0.000360602	Arabidopsis thaliana chromosome 1 sequence
AT4G16270	2.217087034	0.000372933	Arabidopsis thaliana peroxidase 40 mRNA, complete cds
AT5G02570	2.217087034	0.000372933	Arabidopsis thaliana chromosome 5 sequence
AT4G11911	2.217087034	0.000372933	Arabidopsis thaliana uncharacterized protein mRNA, complete cds
AT5G53390	2.074346861	0.000373549	Arabidopsis thaliana bifunctional wax ester synthase/diacylglycerol acyltransferase mRNA, complete cds
AT3G26790	2.074346861	0.000373549	Arabidopsis thaliana B3 domain-containing transcription factor FUS3 mRNA, complete cds
AT1G76210	2.074346861	0.000373549	Arabidopsis thaliana chromosome 1 sequence
AT5G35770	2.074346861	0.000373549	Arabidopsis thaliana transcriptional regulator STERILE APETALA mRNA, complete cds
AT1G32928	1.38742195	0.00037612	Arabidopsis thaliana chromosome 1 sequence
AT5G55020	1.643484395	0.000396507	Arabidopsis thaliana myb domain protein 120 mRNA, complete cds
AT2G45930	1.322114511	0.000396524	Arabidopsis thaliana uncharacterized protein mRNA, complete cds
AT2G37070	1.14320872	0.000400246	Arabidopsis thaliana uncharacterized protein mRNA, complete cds
AT1G22470	1.493500469	0.000402429	Arabidopsis thaliana chromosome 1 sequence
AT3G28705.1	4.600415673	0.000417174	Arabidopsis thaliana chromosome 3, complete sequence
AT1G07540	4.600415673	0.000417174	Arabidopsis thaliana putative telomere repeat-binding protein TRFL2 mRNA, complete cds
AT3G59030	4.600415673	0.000417174	Arabidopsis thaliana protein TRANSPARENT TESTA 12 mRNA, complete cds
AT5G52690	4.600415673	0.000417174	Arabidopsis thaliana copper transport family protein mRNA, complete cds
AT4G11590	4.600415673	0.000417174	Arabidopsis thaliana chromosome 4 sequence
AT2G07070.1	4.600415673	0.000417174	Arabidopsis thaliana chromosome 2, complete sequence
AT4G28405	4.600415673	0.00041717	Arabidopsis thaliana uncharacterized protein

		4	mRNA, complete cds
AT3G23220	4.600415673	0.000417174	Arabidopsis thaliana chromosome 3, complete sequence
AT5G03204	4.600415673	0.000417174	Arabidopsis thaliana chromosome 5 sequence
AT1G73050	4.600415673	0.000417174	Arabidopsis thaliana Glucose-methanol-choline (GMC) oxidoreductase family protein mRNA, complete cds
AT1G79680	3.015453172	0.000439373	Arabidopsis thaliana wall-associated receptor kinase-like 10 mRNA, complete cds
AT3G13130	3.015453172	0.000439373	Arabidopsis thaliana chromosome 3, complete sequence
AT4G13480	3.015453172	0.000439373	Arabidopsis thaliana myb domain protein 79 mRNA, complete cds
AT1G68500	1.407770595	0.000442942	Arabidopsis thaliana chromosome 1 sequence
AT5G18633.1	1.407770595	0.000442942	Arabidopsis thaliana chromosome 5 sequence
AT2G03540.1	1.037148244	0.00049067	Arabidopsis thaliana chromosome 2, complete sequence
AT2G21510	1.752418767	0.000498222	Arabidopsis thaliana DNAJ heat shock N-terminal domain-containing protein mRNA, complete cds
AT5G45810	1.559773689	0.000538393	Arabidopsis thaliana chromosome 5 sequence
AT1G06135	3.715892891	0.000543596	Arabidopsis thaliana chromosome 1 sequence
AT3G11480	3.715892891	0.000543596	Arabidopsis thaliana SABATH methyltransferase BSMT1 mRNA, complete cds
AT4G18450	3.715892891	0.000543596	Arabidopsis thaliana chromosome 4 sequence
AT3G50980	3.715892891	0.000543596	Arabidopsis thaliana dehydrin xero 1 mRNA, complete cds
AT5G15140	3.715892891	0.000543596	Arabidopsis thaliana aldose 1-epimerase-like protein mRNA, complete cds
AT1G06330	2.600415673	0.000550182	Arabidopsis thaliana heavy metal transport/detoxification superfamily protein mRNA, complete cds
ATCG00420	2.600415673	0.000550182	Arabidopsis thaliana chloroplast DNA, complete genome, ecotype: Columbia
AT5G17260	1.118546665	0.000563113	Arabidopsis thaliana NAC domain containing protein 86 mRNA, complete cds

AT1G76960	1.192330935	0.000588812	Arabidopsis thaliana uncharacterized protein mRNA, complete cds
AT1G69485	1.908537969	0.000590728	Arabidopsis thaliana ribosomal L32p protein family mRNA, complete cds
AT1G77960	2.337381267	0.000612035	Arabidopsis thaliana uncharacterized protein mRNA, complete cds
AT1G13310	2.337381267	0.000612035	Arabidopsis thaliana Endosomal targeting BRO1-like domain-containing protein mRNA, complete cds
AT2G47560	1.062758887	0.000624381	Arabidopsis thaliana chromosome 2, complete sequence
AT3G01840	1.373005177	0.000659838	Arabidopsis thaliana LysM-containing receptor-like kinase mRNA, complete cds
AT4G37890	1.122368376	0.000678483	Arabidopsis thaliana chromosome 4 sequence
AT1G75030	1.122368376	0.000678483	Arabidopsis thaliana thaumatin-like protein 3 mRNA, complete cds
AT3G02515.1	1.647721388	0.00070215	Arabidopsis thaliana chromosome 3, complete sequence
AT1G53625	1.647721388	0.00070215	Arabidopsis thaliana chromosome 1 sequence
AT5G24090	1.647721388	0.00070215	Arabidopsis thaliana chitinase A mRNA, complete cds
AT5G43660	1.484938456	0.000711274	Arabidopsis thaliana 2-oxoglutarate (2OG) and Fe(II)-dependent oxygenase superfamily protein mRNA, complete cds
AT1G32950	1.484938456	0.000711274	Arabidopsis thaliana subtilase family protein mRNA, complete cds
AT5G36000	1.393964796	0.000779088	Arabidopsis thaliana uncharacterized protein mRNA, complete cds
AT5G37250	4.474884791	0.000782773	Arabidopsis thaliana chromosome 5 sequence
AT1G19415.1	4.474884791	0.000782773	Arabidopsis thaliana chromosome 1 sequence
AT3G49540	4.474884791	0.000782773	Arabidopsis thaliana uncharacterized protein mRNA, complete cds
AT3G54520	4.474884791	0.000782773	Arabidopsis thaliana uncharacterized protein mRNA, complete cds
AT5G59590	4.474884791	0.000782773	Arabidopsis thaliana UDP-glucosyl transferase 76E2 mRNA, complete cds
AT4G37720	4.474884791	0.000782773	Arabidopsis thaliana phytosulfokine 6 precursor mRNA, complete cds
AT4G25580	4.474884791	0.000782773	Arabidopsis thaliana CAP160 protein mRNA, complete cds
AT5G39090	1.093455684	0.000788511	Arabidopsis thaliana chromosome 5 sequence

AT1G51770	2.922343768	0.000801825	Arabidopsis thaliana core-2/I-branching beta-1,6-N-acetylglucosaminyltransferase family protein mRNA, complete cds
AT5G64401	2.922343768	0.000801825	Arabidopsis thaliana chromosome 5 sequence
AT3G50280	1.126484485	0.00081766	Arabidopsis thaliana chromosome 3, complete sequence
AT5G60610	1.517953513	0.000826459	Arabidopsis thaliana FBD-associated F-box protein mRNA, complete cds
AT5G01480	1.517953513	0.000826459	Arabidopsis thaliana chromosome 5 sequence
AT4G12480	1.164316558	0.000838638	Arabidopsis thaliana chromosome 4 sequence
AT3G14362	1.770340675	0.000879523	Arabidopsis thaliana chromosome 3, complete sequence
AT5G44990	1.770340675	0.000879523	Arabidopsis thaliana Glutathione S-transferase family protein mRNA, complete cds
AT5G38310	1.417551616	0.00091764	Arabidopsis thaliana chromosome 5 sequence
AT3G05400	1.556021554	0.000955097	Arabidopsis thaliana sugar transporter ERD6-like 12 mRNA, complete cds
AT4G06746	1.556021554	0.000955097	Arabidopsis thaliana chromosome 4 sequence
AT5G37750	1.85195444	0.000961894	Arabidopsis thaliana chromosome 5 sequence
AT5G03860	1.85195444	0.000961894	Arabidopsis thaliana malate synthase mRNA, complete cds
AT1G21340	2.517953513	0.000973675	Arabidopsis thaliana chromosome 1 sequence
AT1G70910	3.600415673	0.001021846	Arabidopsis thaliana chromosome 1 sequence
AT2G04490.1	3.600415673	0.001021846	Arabidopsis thaliana chromosome 2, complete sequence
AT1G12890	3.600415673	0.001021846	Arabidopsis thaliana chromosome 1 sequence
AT1G80660	3.600415673	0.001021846	Arabidopsis thaliana H(+)-ATPase 9 mRNA, complete cds
AT5G41680	1.954052628	0.001028495	Arabidopsis thaliana chromosome 5 sequence
AT5G54190	1.954052628	0.001028495	Arabidopsis thaliana protochlorophyllide reductase A mRNA, complete cds
AT2G24040	1.040115227	0.001042684	Arabidopsis thaliana Low temperature and salt responsive protein mRNA, complete cds
AT3G48850	2.263380686	0.00105483	Arabidopsis thaliana phosphate transporter

		3	3;2 mRNA, complete cds
AT3G16020	2.0858425	0.001065642	Arabidopsis thaliana F-box associated ubiquitination effector protein mRNA, complete cds
AT4G19925	2.0858425	0.001065642	Arabidopsis thaliana Toll-Interleukin-Resistance domain-containing protein mRNA, complete cds
AT5G23700	1.600415673	0.001096276	Arabidopsis thaliana uncharacterized protein mRNA, complete cds
AT1G19420	1.35649009	0.001159652	Arabidopsis thaliana chromosome 1 sequence
AT1G03120	1.652883093	0.001247479	Arabidopsis thaliana responsive to abscisic acid 28 mRNA, complete cds
AT2G41905	1.069900956	0.001327539	Arabidopsis thaliana chromosome 2, complete sequence
AT1G02700	1.069900956	0.001327539	Arabidopsis thaliana uncharacterized protein mRNA, complete cds
AT1G19780	2.822808094	0.0014595	Arabidopsis thaliana cyclic nucleotide gated channel 8 mRNA, complete cds
AT1G70390	2.822808094	0.0014595	Arabidopsis thaliana putative F-box protein mRNA, complete cds
AT3G18485	1.237845594	0.001465924	Arabidopsis thaliana protein IAA-LEUCINE RESISTANT 2 mRNA, complete cds
AT1G03390	4.337381267	0.001478095	Arabidopsis thaliana chromosome 1 sequence
AT3G51915	4.337381267	0.001478095	Arabidopsis thaliana uncharacterized protein mRNA, complete cds
AT1G18120	4.337381267	0.001478095	Arabidopsis thaliana chromosome 1 sequence
AT1G27990	4.337381267	0.001478095	Arabidopsis thaliana AT1G27990 mRNA, complete cds, clone: RAFL22-35-E15
AT4G05018	4.337381267	0.001478095	Arabidopsis thaliana chromosome 4 sequence
AT4G34440	4.337381267	0.001478095	Arabidopsis thaliana proline-rich receptor-like protein kinase PERK5 mRNA, complete cds
AT1G59530	4.337381267	0.001478095	Arabidopsis thaliana chromosome 1 sequence
AT2G11310.1	4.337381267	0.001478095	Arabidopsis thaliana chromosome 2, complete sequence
AT4G29340	4.337381267	0.001478095	Arabidopsis thaliana profilin 4 mRNA, complete cds
AT1G12540	4.337381267	0.001478095	Arabidopsis thaliana transcription factor bHLH55 mRNA, complete cds

AT5G50770	4.337381267	0.001478095	Arabidopsis thaliana hydroxysteroid dehydrogenase 6 mRNA, complete cds
AT2G02340	4.337381267	0.001478095	Arabidopsis thaliana phloem protein 2-B8 mRNA, complete cds
AT1G33510	4.337381267	0.001478095	Arabidopsis thaliana chromosome 1 sequence
AT1G55720	4.337381267	0.001478095	Arabidopsis thaliana cation exchanger 6 mRNA, complete cds
AT2G41415	4.337381267	0.001478095	Arabidopsis thaliana maternally expressed family protein mRNA, complete cds
AT1G28160	4.337381267	0.001478095	Arabidopsis thaliana chromosome 1 sequence
AT2G40170	4.337381267	0.001478095	Arabidopsis thaliana Em-like protein GEA6 mRNA, complete cds
AT2G40925	4.337381267	0.001478095	Arabidopsis thaliana chromosome 2, complete sequence
AT3G59510	1.402476296	0.001622418	Arabidopsis thaliana chromosome 3, complete sequence
AT3G25990	1.402476296	0.001622418	Arabidopsis thaliana trihelix transcription factor GT-4 mRNA, complete cds
AT5G60310	1.551506073	0.001700467	Arabidopsis thaliana putative L-type lectin-domain containing receptor kinase I.10 mRNA, complete cds
AT5G67245	1.551506073	0.001700467	Arabidopsis thaliana chromosome 5 sequence
AT3G07250	2.430490672	0.001715144	Arabidopsis thaliana nuclear transport factor 2 and RNA recognition motif domain-containing protein mRNA, complete cds
AT3G18610	1.249918426	0.001757582	Arabidopsis thaliana protein NUCLEOLIN LIKE 2 mRNA, complete cds
AT5G11410	1.249918426	0.001757582	Arabidopsis thaliana protein kinase family protein mRNA, complete cds
AT1G71890	2.015453172	0.001785177	Arabidopsis thaliana sucrose transport protein SUC5 mRNA, complete cds
AT1G73965	2.015453172	0.001785177	Arabidopsis thaliana chromosome 1 sequence
AT1G53980	2.185378174	0.00180768	Arabidopsis thaliana chromosome 1 sequence
AT1G27740	2.185378174	0.00180768	Arabidopsis thaliana transcription factor RSL4 mRNA, complete cds
AT1G77525	3.474884791	0.001924016	Arabidopsis thaliana uncharacterized protein mRNA, complete cds
AT3G04410	3.474884791	0.001924016	Arabidopsis thaliana no apical meristem-domain containing transcriptional

			regulator mRNA, complete cds
AT2G42340	3.474884791	0.001924016	Arabidopsis thaliana uncharacterized protein mRNA, complete cds
AT5G19880	3.474884791	0.001924016	Arabidopsis thaliana peroxidase mRNA, complete cds
AT1G05400	1.337381267	0.002042191	Arabidopsis thaliana uncharacterized protein mRNA, complete cds
AT2G43510	1.015453172	0.002101614	Arabidopsis thaliana trypsin inhibitor protein 1 mRNA, complete cds
AT5G22570	1.659309362	0.002224995	Arabidopsis thaliana putative WRKY transcription factor 38 mRNA, complete cds
AT4G02650	1.659309362	0.002224995	Arabidopsis thaliana putative clathrin assembly protein mRNA, complete cds
AT5G60650	1.044022325	0.002230083	Arabidopsis thaliana chromosome 5 sequence
AT1G03170	1.044022325	0.002230083	Arabidopsis thaliana chromosome 1 sequence
AT2G18000	1.044022325	0.002230083	Arabidopsis thaliana TBP-associated factor 14 mRNA, complete cds
AT3G26350	1.076853717	0.002347175	Arabidopsis thaliana chromosome 3, complete sequence
AT4G23310	1.359407574	0.002426657	Arabidopsis thaliana putative cysteine-rich receptor-like protein kinase 23 mRNA, complete cds
AT5G57520	1.359407574	0.002426657	Arabidopsis thaliana chromosome 5 sequence
AT2G02700	1.731660206	0.002500246	Arabidopsis thaliana chromosome 2, complete sequence
AT3G21720	1.731660206	0.002500246	Arabidopsis thaliana isocitrate lyase mRNA, complete cds
AT3G57740	1.21339255	0.002545981	Arabidopsis thaliana chromosome 3, complete sequence
AT3G26742	2.715892891	0.002648256	Arabidopsis thaliana chromosome 3, complete sequence
AT2G16210	2.715892891	0.002648256	Arabidopsis thaliana B3 domain-containing protein mRNA, complete cds
AT2G24681	2.715892891	0.002648256	Arabidopsis thaliana AP2/B3-like transcriptional factor family protein mRNA, complete cds
AT4G05049	2.715892891	0.002648256	Arabidopsis thaliana chromosome 4 sequence
AT4G28485	2.715892891	0.002648256	Arabidopsis thaliana DUF679 domain membrane protein 7 mRNA, complete cds
AT4G35783	2.715892891	0.00264825	Arabidopsis thaliana chromosome 4 sequence

		6	
AT2G02240	1.822808094	0.002759965	Arabidopsis thaliana F-box domain protein MEE66 mRNA, complete cds
AT4G26770	1.822808094	0.002759965	Arabidopsis thaliana phosphatidate cytidylyltransferase mRNA, complete cds
AT3G14950	1.822808094	0.002759965	Arabidopsis thaliana TPR repeat-containing thioredoxin TTL2 mRNA, complete cds
AT1G77765	1.822808094	0.002759965	Arabidopsis thaliana uncharacterized protein mRNA, complete cds
AT4G03600	1.822808094	0.002759965	Arabidopsis thaliana chromosome 4 sequence
AT1G27080	4.185378174	0.002809939	Arabidopsis thaliana nitrate transporter 1.6 mRNA, complete cds
AT2G45610	4.185378174	0.002809939	Arabidopsis thaliana chromosome 2, complete sequence
AT3G16040	4.185378174	0.002809939	Arabidopsis thaliana translation machinery associated TMA7 mRNA, complete cds
AT1G30780	4.185378174	0.002809939	Arabidopsis thaliana FAD-binding Berberine family protein mRNA, complete cds
AT4G01985	4.185378174	0.002809939	Arabidopsis thaliana chromosome 4 sequence
AT5G05070	4.185378174	0.002809939	Arabidopsis thaliana DHHC-type zinc finger family protein mRNA, complete cds
AT2G13640	4.185378174	0.002809939	Arabidopsis thaliana chromosome 2, complete sequence
AT5G62800	4.185378174	0.002809939	Arabidopsis thaliana E3 ubiquitin-protein ligase SINA-like 11 mRNA, complete cds
AT4G20900	4.185378174	0.002809939	Arabidopsis thaliana protein male sterile 5 mRNA, complete cds
AT1G72260	4.185378174	0.002809939	Arabidopsis thaliana thionin 2.1 mRNA, complete cds
AT2G24545	4.185378174	0.002809939	Arabidopsis thaliana chromosome 2, complete sequence
AT5G52700	4.185378174	0.002809939	Arabidopsis thaliana copper transport family protein mRNA, complete cds
AT3G11773	4.185378174	0.002809939	Arabidopsis thaliana electron carrier/ protein disulfide oxidoreductase mRNA, complete cds
AT4G16050	4.185378174	0.002809939	Arabidopsis thaliana chromosome 4 sequence
AT4G14301	4.185378174	0.002809939	Arabidopsis thaliana uncharacterized protein mRNA, complete cds
AT2G17010	4.185378174	0.002809939	Arabidopsis thaliana mechanosensitive ion channel protein 8 mRNA, complete cds

AT2G35990	4.185378174	0.002809939	Arabidopsis thaliana cytokinin riboside 5'-monophosphate phosphoribohydrolase LOG2 mRNA, complete cds
AT3G42960	4.185378174	0.002809939	Arabidopsis thaliana protein TAPETUM 1 mRNA, complete cds
AT3G25233	4.185378174	0.002809939	Arabidopsis thaliana chromosome 3, complete sequence
AT4G34210	4.185378174	0.002809939	Arabidopsis thaliana chromosome 4 sequence
AT5G40350	4.185378174	0.002809939	Arabidopsis thaliana myb domain protein 24 mRNA, complete cds
AT3G48920	1.941452591	0.002970384	Arabidopsis thaliana myb domain protein 45 mRNA, complete cds
AT2G32200	1.941452591	0.002970384	Arabidopsis thaliana uncharacterized protein mRNA, complete cds
AT4G01780	1.941452591	0.002970384	Arabidopsis thaliana XH/XS domain-containing protein mRNA, complete cds
AT1G37150	1.941452591	0.002970384	Arabidopsis thaliana holocarboxylase synthetase 2 mRNA, complete cds
AT4G01975.1	1.941452591	0.002970384	Arabidopsis thaliana chromosome 4 sequence
AT5G49690	1.941452591	0.002970384	Arabidopsis thaliana chromosome 5 sequence
AT5G08030	2.337381267	0.003005284	Arabidopsis thaliana glycerophosphoryl diester phosphodiesterase mRNA, complete cds
AT2G28680	2.337381267	0.003005284	Arabidopsis thaliana RmlC-like cupins superfamily protein mRNA, complete cds
AT3G13275	1.167456266	0.003036041	Arabidopsis thaliana chromosome 3, complete sequence
AT2G02380	1.545967889	0.003040723	Arabidopsis thaliana putative glutathione S-transferase zeta-class 2 mRNA, complete cds
AT2G31940	1.545967889	0.003040723	Arabidopsis thaliana chromosome 2, complete sequence
AT1G33813.1	1.545967889	0.003040723	Arabidopsis thaliana chromosome 1 sequence
AT4G04760	2.102916014	0.003078355	Arabidopsis thaliana sugar transporter ERD6-like 15 mRNA, complete cds
AT3G07120	2.102916014	0.003078355	Arabidopsis thaliana chromosome 3, complete sequence
AT1G54400	2.102916014	0.003078355	Arabidopsis thaliana alpha-crystallin domain of heat shock protein-containing protein

			mRNA, complete cds
AT1G31750	1.414002549	0.003401109	Arabidopsis thaliana mRNA for hypothetical protein, complete cds, clone: RAFL16-18-G02
AT4G10580.1	1.600415673	0.003501142	Arabidopsis thaliana chromosome 4 sequence
AT1G80970	1.125077664	0.003576642	Arabidopsis thaliana XH domain-containing protein mRNA, complete cds
ATCG00730	1.315013454	0.003604657	Arabidopsis thaliana chloroplast DNA, complete genome, ecotype: Columbia
AT3G46680	3.337381267	0.003627755	Arabidopsis thaliana UDP-glycosyltransferase 76E6 mRNA, complete cds
AT1G07473	3.337381267	0.003627755	Arabidopsis thaliana chromosome 1 sequence
AT2G34030	3.337381267	0.003627755	Arabidopsis thaliana Calcium-binding EF-hand family protein mRNA, complete cds
AT1G12672	3.337381267	0.003627755	Arabidopsis thaliana uncharacterized protein mRNA, complete cds
AT1G35255	3.337381267	0.003627755	Arabidopsis thaliana chromosome 1 sequence
AT4G19829	3.337381267	0.003627755	Arabidopsis thaliana chromosome 4 sequence
AT3G27884	1.237845594	0.003682424	Arabidopsis thaliana clone 102688 mRNA sequence
AT3G55820	1.015453172	0.003736469	Arabidopsis thaliana chromosome 3, complete sequence
AT1G07290	1.04787465	0.00396508	Arabidopsis thaliana GDP-mannose transporter mRNA, complete cds
AT1G44130	1.667529869	0.003987212	Arabidopsis thaliana aspartyl protease family protein mRNA, complete cds
AT2G41240	1.667529869	0.003987212	Arabidopsis thaliana transcription factor bHLH100 mRNA, complete cds
AT1G13130	1.667529869	0.003987212	Arabidopsis thaliana Cellulase (glycosyl hydrolase family 5) protein mRNA, complete cds
AT5G40210	1.0858425	0.00416783	Arabidopsis thaliana nodulin MtN21 /EamA-like transporter family protein mRNA, complete cds
AT1G16820	1.337381267	0.004300244	Arabidopsis thaliana V-ATPase-related protein mRNA, complete cds
AT5G51500	1.13093039	0.004327966	Arabidopsis thaliana putative pectinesterase/pectinesterase inhibitor 60 mRNA, complete cds

AT1G01380	1.185378174	0.004423431	Arabidopsis thaliana MYB-like transcription factor ETC1 mRNA, complete cds
AT2G35730	1.25249237	0.00442589	Arabidopsis thaliana heavy-metal-associated domain-containing protein mRNA, complete cds
AT5G59190	1.752418767	0.004471853	Arabidopsis thaliana subtilase family protein mRNA, complete cds
AT2G35550	1.752418767	0.004471853	Arabidopsis thaliana basic pentacysteine 7 mRNA, complete cds
AT5G05300	1.752418767	0.004471853	Arabidopsis thaliana chromosome 5 sequence
AT4G04510	1.752418767	0.004471853	Arabidopsis thaliana cysteine-rich receptor-like protein kinase 38 mRNA, complete cds
AT5G10230	2.600415673	0.004786824	Arabidopsis thaliana annexin D7 mRNA, complete cds
AT1G21525	2.600415673	0.004786824	Arabidopsis thaliana chromosome 1 sequence
AT3G50310	2.600415673	0.004786824	Arabidopsis thaliana chromosome 3, complete sequence
AT5G06520	2.600415673	0.004786824	Arabidopsis thaliana SWAP/Surp domain-containing protein mRNA, complete cds
AT5G51760	2.600415673	0.004786824	Arabidopsis thaliana probable protein phosphatase AHG1 mRNA, complete cds
AT5G66110	1.863450079	0.004905953	Arabidopsis thaliana heavy metal associated isoprenylated plant protein 27 mRNA, complete cds
AT1G76955	1.863450079	0.004905953	Arabidopsis thaliana uncharacterized protein mRNA, complete cds
AT1G24480	1.863450079	0.004905953	Arabidopsis thaliana chromosome 1 sequence
AT3G47030	1.863450079	0.004905953	Arabidopsis thaliana chromosome 3, complete sequence
AT5G16960	1.863450079	0.004905953	Arabidopsis thaliana zinc-binding dehydrogenase family protein mRNA, complete cds
AT1G05894	1.363376476	0.005119526	Arabidopsis thaliana chromosome 1 sequence
AT1G49900	2.015453172	0.00520553	Arabidopsis thaliana C2H2 type zinc finger transcription factor-like protein mRNA, complete cds
AT1G32560	2.237845594	0.005234101	Arabidopsis thaliana Late embryogenesis abundant protein, group 1 protein mRNA, complete cds

AT3G49130	2.237845594	0.005234101	Arabidopsis thaliana SWAP (Suppressor-of-White-APricot)/surp RNA-binding domain-containing protein mRNA, complete cds
AT2G02930	1.269209765	0.005315995	Arabidopsis thaliana glutathione S-transferase F3 mRNA, complete cds
AT5G47590	1.269209765	0.005315995	Arabidopsis thaliana Heat shock protein HSP20/alpha crystallin family protein mRNA, complete cds
AT4G34380	1.196025418	0.005339904	Arabidopsis thaliana chromosome 4 sequence
AT4G31760	4.015453172	0.005380568	Arabidopsis thaliana peroxidase 46 mRNA, complete cds
AT2G27505	4.015453172	0.005380568	Arabidopsis thaliana FBD-like domain family protein mRNA, complete cds
AT4G18090	4.015453172	0.005380568	Arabidopsis thaliana chromosome 4 sequence
AT1G63760	4.015453172	0.005380568	Arabidopsis thaliana chromosome 1 sequence
AT4G27790	4.015453172	0.005380568	Arabidopsis thaliana Calcium-binding EF hand family protein mRNA, complete cds
AT1G07120	4.015453172	0.005380568	Arabidopsis thaliana uncharacterized protein mRNA, complete cds
AT4G36350	4.015453172	0.005380568	Arabidopsis thaliana purple acid phosphatase 25 mRNA, complete cds
AT3G56277.1	4.015453172	0.005380568	Arabidopsis thaliana chromosome 3, complete sequence
AT3G09950	4.015453172	0.005380568	Arabidopsis thaliana chromosome 3, complete sequence
AT5G04010	4.015453172	0.005380568	Arabidopsis thaliana chromosome 5 sequence
AT2G44810	4.015453172	0.005380568	Arabidopsis thaliana chromosome 2, complete sequence
AT1G16290	4.015453172	0.005380568	Arabidopsis thaliana uncharacterized protein mRNA, complete cds
AT2G26135	4.015453172	0.005380568	Arabidopsis thaliana RING/U-box protein with C6HC-type zinc finger mRNA, complete cds
AT5G12000	4.015453172	0.005380568	Arabidopsis thaliana Protein kinase protein with adenine nucleotide alpha hydrolases-like domain mRNA, complete cds
AT5G44590	4.015453172	0.005380568	Arabidopsis thaliana S-adenosyl-L-methionine-dependent methyltransferase domain-containing protein

			mRNA, complete cds
AT2G33780	4.015453172	0.005380568	Arabidopsis thaliana chromosome 2, complete sequence
AT1G22670	4.015453172	0.005380568	Arabidopsis thaliana Protease-associated (PA) RING/U-box zinc finger family protein mRNA, complete cds
AT1G23070	4.015453172	0.005380568	Arabidopsis thaliana uncharacterized protein mRNA, complete cds
AT1G03982	4.015453172	0.005380568	Arabidopsis thaliana PAK-box/P21-Rho-binding family protein mRNA, complete cds
AT1G68250	4.015453172	0.005380568	Arabidopsis thaliana chromosome 1 sequence
AT3G21500	4.015453172	0.005380568	Arabidopsis thaliana 1-deoxy-D-xylulose 5-phosphate synthase 1 mRNA, complete cds
AT4G25670	4.015453172	0.005380568	Arabidopsis thaliana conserved peptide upstream open reading frame 12 mRNA, complete cds
AT1G47950.1	4.015453172	0.005380568	Arabidopsis thaliana chromosome 1 sequence
AT3G41762	4.015453172	0.005380568	Arabidopsis thaliana uncharacterized protein mRNA, complete cds
AT5G41090	4.015453172	0.005380568	Arabidopsis thaliana NAC domain containing protein 95 mRNA, complete cds
AT4G34300	4.015453172	0.005380568	Arabidopsis thaliana chromosome 4 sequence
AT5G56230	1.393964796	0.006078337	Arabidopsis thaliana chromosome 5 sequence
AT4G24150	1.093455684	0.006128764	Arabidopsis thaliana growth-regulating factor 8 mRNA, complete cds
AT1G72660	1.600415673	0.006307721	Arabidopsis thaliana developmentally regulated G-protein 2 mRNA, complete cds
AT1G07160	1.288471667	0.006379344	Arabidopsis thaliana putative protein phosphatase 2C 2 mRNA, complete cds
AT1G74550	1.288471667	0.006379344	Arabidopsis thaliana chromosome 1 sequence
AT2G01275	1.20809825	0.006446154	Arabidopsis thaliana RING/FYVE/PHD zinc finger-containing protein mRNA, complete cds
AT5G41663	3.185378174	0.006847363	Arabidopsis thaliana chromosome 5 sequence
AT2G33280	3.185378174	0.006847363	Arabidopsis thaliana probable folate-biopterin transporter 9 mRNA, complete cds
AT3G05260	3.185378174	0.00684736	Arabidopsis thaliana NAD(P)-binding

		3	Rossmann-fold superfamily protein mRNA, complete cds
AT5G06380	3.185378174	0.00684736 3	Arabidopsis thaliana chromosome 5 sequence
AT5G25390	3.185378174	0.00684736 3	Arabidopsis thaliana ethylene-responsive transcription factor SHINE 3 mRNA, complete cds
AT4G18190	3.185378174	0.00684736 3	Arabidopsis thaliana purine permease 6 mRNA, complete cds
AT5G49525	3.185378174	0.00684736 3	Arabidopsis thaliana chromosome 5 sequence
AT1G71000	1.678418185	0.00718669 2	Arabidopsis thaliana chaperone DnaJ-domain containing protein mRNA, complete cds
AT5G18636	1.678418185	0.00718669 2	Arabidopsis thaliana chromosome 5 sequence
AT1G69050	1.678418185	0.00718669 2	Arabidopsis thaliana chromosome 1 sequence
AT3G24900	1.678418185	0.00718669 2	Arabidopsis thaliana chromosome 3, complete sequence
AT1G16140	1.430490672	0.00719040 4	Arabidopsis thaliana chromosome 1 sequence
AT5G17780	1.310909056	0.00764582 8	Arabidopsis thaliana hydrolase, alpha/beta fold family protein mRNA, complete cds
AT1G73810	1.310909056	0.00764582 8	Arabidopsis thaliana core-2/I-branching beta-1,6-N-acetylglucosaminyltransferase-lik e protein mRNA, complete cds
AT1G28940	1.310909056	0.00764582 8	Theobroma cacao Uncharacterized protein (TCM_007238) mRNA, complete cds
AT1G73325	1.780987919	0.00803703 8	Arabidopsis thaliana chromosome 1 sequence
AT1G59265. 1	1.474884791	0.00846355 8	Arabidopsis thaliana chromosome 1 sequence
AT3G29639	2.474884791	0.008611954	Arabidopsis thaliana uncharacterized protein mRNA, complete cds
AT1G17710	2.474884791	0.008611954	Arabidopsis thaliana phosphoethanolamine/phosphocholine phosphatase mRNA, complete cds
AT3G56920	2.474884791	0.008611954	Arabidopsis thaliana putative S-acyltransferase mRNA, complete cds
AT1G13448	2.474884791	0.008611954	Arabidopsis thaliana clone 270701 mRNA sequence
AT2G27630	2.474884791	0.008611954	Arabidopsis thaliana ubiquitin carboxyl-terminal hydrolase-related protein mRNA, complete cds

AT5G05420	1.922343768	0.008733822	Arabidopsis thaliana peptidyl-prolyl cis-trans isomerase FKBP15-3 mRNA, complete cds
AT4G28280	1.922343768	0.008733822	Arabidopsis thaliana chromosome 4 sequence
AT5G56880	1.922343768	0.008733822	Arabidopsis thaliana chromosome 5 sequence
AT3G07380	1.922343768	0.008733822	Arabidopsis thaliana uncharacterized protein mRNA, complete cds
AT3G21620	1.102916014	0.00903585	Arabidopsis thaliana early-responsive to dehydration stress-related protein mRNA, complete cds
AT5G51210	2.13093039	0.009052858	Arabidopsis thaliana oleosin3 mRNA, complete cds
AT3G01620	2.13093039	0.009052858	Arabidopsis thaliana beta-1,4-N-acetylglucosaminyltransferase family protein mRNA, complete cds
AT2G24683	2.13093039	0.009052858	Arabidopsis thaliana chromosome 2, complete sequence
AT4G05053	2.13093039	0.009052858	Arabidopsis thaliana chromosome 4 sequence
AT3G28890	2.13093039	0.009052858	Arabidopsis thaliana receptor like protein 43 mRNA, complete cds
AT4G01670	1.337381267	0.009147871	Arabidopsis thaliana uncharacterized protein mRNA, complete cds
AT1G05770	1.015453172	0.00988199	Arabidopsis thaliana mannose-binding lectin-like protein mRNA, complete cds
ATCG00080	1.530026345	0.00989279	Cardamine resedifolia plastid, complete genome
AT2G35290	1.530026345	0.00989279	Arabidopsis thaliana chromosome 2, complete sequence
AT1G14642	1.530026345	0.00989279	Arabidopsis thaliana uncharacterized protein mRNA, complete cds
AT1G63245	1.530026345	0.00989279	Arabidopsis thaliana chromosome 1 sequence
AT5G20260	1.530026345	0.00989279	Arabidopsis thaliana Exostosin family protein mRNA, complete cds
AT3G42550	3.822808094	0.010383467	Arabidopsis thaliana aspartyl protease family protein mRNA, complete cds
AT1G30455	3.822808094	0.010383467	Arabidopsis thaliana cyclin/Brf1-like TBP-binding domain-containing protein mRNA, complete cds
AT3G52080	3.822808094	0.010383467	Arabidopsis thaliana cation/H(+) antiporter 28 mRNA, complete cds
AT3G17720	3.822808094	0.010383467	Arabidopsis thaliana pyridoxal phosphate (PLP)-dependent transferases superfamily

			protein mRNA, complete cds
AT3G20541	3.822808094	0.010383467	Arabidopsis thaliana chromosome 3, complete sequence
AT2G27320	3.822808094	0.010383467	Arabidopsis thaliana uncharacterized protein mRNA, complete cds
AT2G23510	3.822808094	0.010383467	Arabidopsis thaliana spermidine disinapoyl acyltransferase mRNA, complete cds
AT5G09640	3.822808094	0.010383467	Arabidopsis thaliana serine carboxypeptidase-like 19 mRNA, complete cds
AT1G28695	3.822808094	0.010383467	Arabidopsis thaliana nucleotide-diphospho-sugar transferase-like protein mRNA, complete cds
AT1G71120	3.822808094	0.010383467	Arabidopsis thaliana GDSL-motif lipase/hydrolase 6 mRNA, complete cds
AT5G45830	3.822808094	0.010383467	Arabidopsis thaliana protein DELAY OF GERMINATION 1 mRNA, complete cds
AT2G29150	3.822808094	0.010383467	Arabidopsis thaliana tropinone reductase-like protein mRNA, complete cds
AT2G23808	3.822808094	0.010383467	Arabidopsis thaliana chromosome 2, complete sequence
AT1G55230	3.822808094	0.010383467	Arabidopsis thaliana chromosome 1 sequence
AT2G30830	3.822808094	0.010383467	Arabidopsis thaliana 2-oxoglutarate dependent oxygenase-like protein mRNA, complete cds
AT4G37420	3.822808094	0.010383467	Arabidopsis thaliana chromosome 4 sequence
AT1G44090	3.822808094	0.010383467	Arabidopsis thaliana gibberellin 20-oxidase 5 mRNA, complete cds
AT2G21780	3.822808094	0.010383467	Arabidopsis thaliana chromosome 2, complete sequence
AT4G24890	3.822808094	0.010383467	Arabidopsis thaliana purple acid phosphatase 24 mRNA, complete cds
AT1G52680	3.822808094	0.010383467	Arabidopsis thaliana late embryogenesis abundant protein-like protein mRNA, complete cds
AT1G19115	3.822808094	0.010383467	Arabidopsis thaliana uncharacterized protein mRNA, complete cds
AT4G27210.1	3.822808094	0.010383467	Arabidopsis thaliana chromosome 4 sequence
AT2G25482	3.822808094	0.010383467	Arabidopsis thaliana chromosome 2, complete sequence
AT1G66060	3.822808094	0.01038346	Arabidopsis thaliana chromosome 1 sequence

		7	
AT5G37970	3.822808094	0.010383467	Arabidopsis thaliana probable S-adenosylmethionine-dependent methyltransferase mRNA, complete cds
AT1G57650	3.822808094	0.010383467	Arabidopsis thaliana ATP binding protein mRNA, complete cds
AT3G54410	3.822808094	0.010383467	Arabidopsis thaliana uncharacterized protein mRNA, complete cds
AT5G25430	3.822808094	0.010383467	Arabidopsis thaliana putative boron transporter 6 mRNA, complete cds
AT2G41997	3.822808094	0.010383467	Arabidopsis thaliana defensin-like protein 108 mRNA, complete cds
AT5G64540	3.822808094	0.010383467	Arabidopsis thaliana uncharacterized protein mRNA, complete cds
AT5G24820	3.822808094	0.010383467	Arabidopsis thaliana aspartyl protease family protein mRNA, complete cds
AT5G59230	3.822808094	0.010383467	Arabidopsis thaliana transcription factor-related protein mRNA, complete cds
AT2G47040	3.822808094	0.010383467	Arabidopsis thaliana pectinesterase 5 mRNA, complete cds
AT5G01900	3.822808094	0.010383467	Arabidopsis thaliana putative WRKY transcription factor 62 mRNA, complete cds
AT5G02330	3.822808094	0.010383467	Arabidopsis thaliana chromosome 5 sequence
AT4G19220	1.057273348	0.010486669	Arabidopsis thaliana pentatricopeptide repeat-containing protein mRNA, complete cds
AT2G35345	1.057273348	0.010486669	Arabidopsis thaliana chromosome 2, complete sequence
AT4G39925	1.369090127	0.010918491	Arabidopsis thaliana AT hook motif DNA-binding family protein mRNA, complete cds
AT5G51810	1.369090127	0.010918491	Arabidopsis thaliana gibberellin 20 oxidase 2 mRNA, complete cds
AT2G04040	1.369090127	0.010918491	Arabidopsis thaliana MATE efflux family protein DTX1 mRNA, complete cds
AT2G29370	1.369090127	0.010918491	Arabidopsis thaliana tropinone reductase-like protein mRNA, complete cds
AT5G14180	1.172994449	0.011297814	Arabidopsis thaliana Myzus persicae-induced lipase 1 mRNA, complete cds
AT4G28700	1.256461272	0.011324296	Arabidopsis thaliana chromosome 4 sequence
AT1G18990	1.256461272	0.011324296	Arabidopsis thaliana uncharacterized protein mRNA, complete cds
AT4G13000	1.256461272	0.011324296	Arabidopsis thaliana chromosome 4 sequence

AT2G44700	1.600415673	0.011447604	Arabidopsis thaliana chromosome 2, complete sequence
AT3G12910	1.015453172	0.012034849	Arabidopsis thaliana no apical meristem domain-containing transcriptional regulator mRNA, complete cds
AT1G17030	1.015453172	0.012034849	Arabidopsis thaliana uncharacterized protein mRNA, complete cds
AT5G15480	3.015453172	0.012931772	Arabidopsis thaliana chromosome 5 sequence
AT4G18350	3.015453172	0.012931772	Arabidopsis thaliana chromosome 4 sequence
AT1G64195	3.015453172	0.012931772	Arabidopsis thaliana defensin-like protein 35 mRNA, complete cds
AT5G37940	3.015453172	0.012931772	Arabidopsis thaliana zinc-binding dehydrogenase family protein mRNA, complete cds
AT2G25890	3.015453172	0.012931772	Arabidopsis thaliana chromosome 2, complete sequence
AT5G57820	3.015453172	0.012931772	Arabidopsis thaliana zinc ion binding protein mRNA, complete cds
AT5G60320	3.015453172	0.012931772	Arabidopsis thaliana chromosome 5 sequence
AT1G73175	3.015453172	0.012931772	Arabidopsis thaliana chromosome 1 sequence
AT5G19360	3.015453172	0.012931772	Arabidopsis thaliana calcium-dependent protein kinase 34 mRNA, complete cds
AT1G14930	3.015453172	0.012931772	Arabidopsis thaliana polyketide cyclase/dehydrase and lipid transport superfamily protein mRNA, complete cds
AT2G04135.1	3.015453172	0.012931772	Arabidopsis thaliana chromosome 2, complete sequence
AT3G17050.1	3.015453172	0.012931772	Arabidopsis thaliana chromosome 3, complete sequence
AT5G21030	3.015453172	0.012931772	Arabidopsis thaliana protein argonaute 8 mRNA, complete cds
AT4G36490	3.015453172	0.012931772	Arabidopsis thaliana protein SEC14-like 12 mRNA, complete cds
AT5G42340	3.015453172	0.012931772	Arabidopsis thaliana U-box domain-containing protein 15 mRNA, complete cds
AT5G50570	1.407770595	0.012987117	Arabidopsis thaliana squamosa promoter-binding-like protein 13 mRNA, complete cds
AT1G68050	1.407770595	0.012987117	Arabidopsis thaliana flavin-binding, kelch

			repeat, f box 1 mRNA, complete cds
AT1G55790	1.693525078	0.013048796	Arabidopsis thaliana uncharacterized protein mRNA, complete cds
AT1G78478	1.114988846	0.013361601	Arabidopsis thaliana chromosome 1 sequence
AT4G38850	1.114988846	0.013361601	Arabidopsis thaliana chromosome 4 sequence
AT2G31083	1.278487578	0.013647964	Arabidopsis thaliana chromosome 2, complete sequence
AT2G42990	1.278487578	0.013647964	Arabidopsis thaliana GDSL esterase/lipase mRNA, complete cds
AT3G13540	1.278487578	0.013647964	Arabidopsis thaliana transcription repressor MYB5 mRNA, complete cds
AT5G25990	1.185378174	0.013703473	Arabidopsis thaliana uncharacterized protein mRNA, complete cds
AT4G34320	1.185378174	0.013703473	Arabidopsis thaliana uncharacterized protein mRNA, complete cds
AT4G03380	1.822808094	0.014525326	Arabidopsis thaliana uncharacterized protein mRNA, complete cds
AT5G10990	1.822808094	0.014525326	Arabidopsis thaliana chromosome 5 sequence
AT5G41780	1.822808094	0.014525326	Arabidopsis thaliana myosin heavy chain-like protein mRNA, complete cds
AT1G42560	1.822808094	0.014525326	Arabidopsis thaliana MLO-like protein 9 mRNA, complete cds
AT2G22460	1.015453172	0.014672197	Arabidopsis thaliana chromosome 2, complete sequence
AT1G67600	1.456025764	0.015371116	Arabidopsis thaliana acid phosphatase/vanadium-dependent haloperoxidase-related protein mRNA, complete cds
AT5G65165	2.337381267	0.015405391	Arabidopsis thaliana succinate dehydrogenase [ubiquinone] iron-sulfur subunit 3 mRNA, complete cds
AT2G35658	2.337381267	0.015405391	Arabidopsis thaliana chromosome 2, complete sequence
AT2G34990	2.337381267	0.015405391	Arabidopsis thaliana chromosome 2, complete sequence
ATCG00330	2.337381267	0.015405391	Arabidopsis thaliana chloroplast DNA, complete genome, ecotype: Columbia
AT3G56380	2.337381267	0.015405391	Arabidopsis thaliana two-component response regulator ARR17 mRNA, complete cds
AT1G02250	2.337381267	0.015405391	Arabidopsis thaliana NAC domain-containing protein 5 mRNA, complete cds

AT5G42230	2.337381267	0.015405391	Arabidopsis thaliana serine carboxypeptidase-like 41 mRNA, complete cds
AT2G30690	2.015453172	0.015533202	Arabidopsis thaliana uncharacterized protein mRNA, complete cds
AT1G64100	2.015453172	0.015533202	Arabidopsis thaliana pentatricopeptide repeat-containing protein mRNA, complete cds
AT4G23160	2.015453172	0.015533202	Arabidopsis thaliana cysteine-rich receptor-like protein kinase 8 mRNA, complete cds
AT4G18980	2.015453172	0.015533202	Arabidopsis thaliana chromosome 4 sequence
AT2G45420	2.015453172	0.015533202	Arabidopsis thaliana LOB domain-containing protein 18 mRNA, complete cds
AT5G41570	2.015453172	0.015533202	Arabidopsis thaliana WRKY transcription factor 24 mRNA, complete cds
AT2G38340	2.015453172	0.015533202	Arabidopsis thaliana chromosome 2, complete sequence
AT5G52000	2.015453172	0.015533202	Arabidopsis thaliana importin alpha isoform 8 mRNA, complete cds
AT5G44574	2.015453172	0.015533202	Arabidopsis thaliana uncharacterized protein mRNA, complete cds
AT1G23700	2.015453172	0.015533202	Arabidopsis thaliana protein kinase mRNA, complete cds
AT2G44990	1.30495979	0.016428269	Arabidopsis thaliana carotenoid cleavage dioxygenase 7 mRNA, complete cds
AT4G14700	1.199877744	0.016625745	Arabidopsis thaliana chromosome 4 sequence
AT1G77570	1.199877744	0.016625745	Arabidopsis thaliana chromosome 1 sequence
AT5G07060	1.517953513	0.018058959	Arabidopsis thaliana MOS4-associated complex subunit 5C mRNA, complete cds
AT1G05450	1.517953513	0.018058959	Arabidopsis thaliana protease inhibitor/seed storage/LTP family protein mRNA, complete cds
AT2G01008	1.337381267	0.019737309	Arabidopsis thaliana uncharacterized protein mRNA, complete cds
AT4G23030	1.337381267	0.019737309	Arabidopsis thaliana chromosome 4 sequence
AT2G21450	1.337381267	0.019737309	Arabidopsis thaliana chromatin remodeling 34 mRNA, complete cds
AT1G11460	1.337381267	0.019737309	Arabidopsis thaliana nodulin MtN21-like transporter family protein mRNA, complete

			cds
AT1G50400	1.337381267	0.019737309	Arabidopsis thaliana eukaryotic porin family protein mRNA, complete cds
AT5G23600	1.13093039	0.019825313	Arabidopsis thaliana chromosome 5 sequence
AT4G15280	1.217087034	0.020173925	Arabidopsis thaliana chromosome 4 sequence
AT1G30220	1.217087034	0.020173925	Arabidopsis thaliana putative inositol transporter 2 mRNA, complete cds
AT2G35670	3.600415673	0.020208664	Arabidopsis thaliana polycomb group protein FERTILIZATION-INDEPENDENT SEED 2 mRNA, complete cds
AT5G44416.1	3.600415673	0.020208664	Arabidopsis thaliana chromosome 5 sequence
AT4G29570	3.600415673	0.020208664	Arabidopsis thaliana chromosome 4 sequence
AT1G62095	3.600415673	0.020208664	Arabidopsis thaliana chromosome 1 sequence
AT5G11820	3.600415673	0.020208664	Arabidopsis thaliana chromosome 5 sequence
AT1G66990	3.600415673	0.020208664	Arabidopsis thaliana chromosome 1 sequence
AT1G12211	3.600415673	0.020208664	Arabidopsis thaliana chromosome 1 sequence
AT1G29680	3.600415673	0.020208664	Arabidopsis thaliana uncharacterized protein mRNA, complete cds
AT1G73190	3.600415673	0.020208664	Arabidopsis thaliana aquaporin TIP3-1 mRNA, complete cds
AT1G62000	3.600415673	0.020208664	Arabidopsis thaliana chromosome 1 sequence
AT3G43200	3.600415673	0.020208664	Arabidopsis thaliana chromosome 3, complete sequence
AT2G05450.1	3.600415673	0.020208664	Arabidopsis thaliana chromosome 2, complete sequence
AT2G03460	3.600415673	0.020208664	Arabidopsis thaliana galactose oxidase/kelch-like protein mRNA, complete cds
AT1G32480	3.600415673	0.020208664	Arabidopsis thaliana chromosome 1 sequence
AT3G02470	3.600415673	0.020208664	Arabidopsis thaliana conserved peptide upstream open reading frame 9 mRNA, complete cds
AT4G18080	3.600415673	0.020208664	Arabidopsis thaliana chromosome 4 sequence

AT5G53520	3.600415673	0.020208664	Arabidopsis thaliana oligopeptide transporter 8 mRNA, complete cds
AT4G37840	3.600415673	0.020208664	Arabidopsis thaliana hexokinase-like 3 mRNA, complete cds
AT1G03790	3.600415673	0.020208664	Arabidopsis thaliana chromosome 1 sequence
AT5G41200	3.600415673	0.020208664	Arabidopsis thaliana chromosome 5 sequence
AT2G47870	3.600415673	0.020208664	Arabidopsis thaliana chromosome 2, complete sequence
AT3G04660	3.600415673	0.020208664	Arabidopsis thaliana chromosome 3, complete sequence
AT1G51150	3.600415673	0.020208664	Arabidopsis thaliana DegP protease 6 mRNA, complete cds
AT4G02655	3.600415673	0.020208664	Arabidopsis thaliana chromosome 4 sequence
AT5G17200	3.600415673	0.020208664	Arabidopsis thaliana pectin lyase-like superfamily protein mRNA, complete cds
AT1G51780	3.600415673	0.020208664	Arabidopsis thaliana IAA-amino acid hydrolase ILR1-like 5 mRNA, complete cds
AT5G20690	3.600415673	0.020208664	Arabidopsis thaliana probable inactive leucine-rich repeat receptor-like protein kinase mRNA, complete cds
AT1G22015	3.600415673	0.020208664	Arabidopsis thaliana putative beta-1,3-galactosyltransferase 5 mRNA, complete cds
AT3G63052	3.600415673	0.020208664	Arabidopsis thaliana chromosome 3, complete sequence
AT1G02770	3.600415673	0.020208664	Arabidopsis thaliana uncharacterized protein mRNA, complete cds
AT5G63900	3.600415673	0.020208664	Arabidopsis thaliana chromosome 5 sequence
AT4G33870	3.600415673	0.020208664	Arabidopsis thaliana putative peroxidase mRNA, complete cds
AT3G53790	3.600415673	0.020208664	Arabidopsis thaliana protein TRF-like 4 mRNA, complete cds
AT5G44330	3.600415673	0.020208664	Arabidopsis thaliana tetratricopeptide repeat domain-containing protein mRNA, complete cds
AT5G54450	3.600415673	0.020208664	Arabidopsis thaliana chromosome 5 sequence
AT2G32310	3.600415673	0.020208664	Arabidopsis thaliana CCT motif family protein mRNA, complete cds
AT3G46911	3.600415673	0.02020866	Arabidopsis thaliana chromosome 3,

		4	complete sequence
AT1G66850	3.600415673	0.020208664	Arabidopsis thaliana chromosome 1 sequence
AT2G41690	1.600415673	0.020976676	Arabidopsis thaliana heat stress transcription factor B-3 mRNA, complete cds
AT5G59845	1.015453172	0.021886672	Arabidopsis thaliana gibberellin-regulated protein mRNA, complete cds
AT4G22620	1.015453172	0.021886672	Arabidopsis thaliana chromosome 4 sequence
AT4G37022	1.015453172	0.021886672	Arabidopsis thaliana uncharacterized protein mRNA, complete cds
AT1G68200	1.015453172	0.021886672	Arabidopsis thaliana zinc finger CCCH domain-containing protein 15 mRNA, complete cds
AT1G06750	1.378023252	0.023642636	Arabidopsis thaliana P-loop containing nucleoside triphosphate hydrolases superfamily protein mRNA, complete cds
AT5G07880	1.715892891	0.023919491	Arabidopsis thaliana SNAP25-like protein SNAP29 mRNA, complete cds
AT4G26380	1.715892891	0.023919491	Arabidopsis thaliana cysteine/histidine-rich C1 domain-containing protein mRNA, complete cds
AT2G17690	1.715892891	0.023919491	Arabidopsis thaliana SUPPRESSOR OF drm1 drm2 cmt3 mRNA, complete cds
AT2G33130	2.822808094	0.024420357	Arabidopsis thaliana chromosome 2, complete sequence
AT5G38705.1	2.822808094	0.024420357	Arabidopsis thaliana chromosome 5 sequence
AT4G27550	2.822808094	0.024420357	Arabidopsis thaliana putative alpha,alpha-trehalose-phosphate synthase [UDP-forming] 4 mRNA, complete cds
AT1G70720	2.822808094	0.024420357	Arabidopsis thaliana chromosome 1 sequence
AT1G72590	2.822808094	0.024420357	Arabidopsis thaliana 3-oxo-5-alpha-steroid 4-dehydrogenase family protein mRNA, complete cds
AT5G35380	2.822808094	0.024420357	Arabidopsis thaliana protein kinase protein with adenine nucleotide alpha hydrolases-like domain mRNA, complete cds
AT2G32740	2.822808094	0.024420357	Arabidopsis thaliana chromosome 2, complete sequence
AT5G10340	2.822808094	0.024420357	Arabidopsis thaliana F-box protein mRNA, complete cds
AT5G37050	2.822808094	0.02442035	Arabidopsis thaliana uncharacterized protein

		7	mRNA, complete cds
AT4G07830.1	2.822808094	0.024420357	Arabidopsis thaliana chromosome 4 sequence
AT5G47150	2.822808094	0.024420357	Arabidopsis thaliana YDG/SRA domain-containing protein mRNA, complete cds
AT4G38310	2.822808094	0.024420357	Arabidopsis thaliana chromosome 4 sequence
AT3G51478	1.237845594	0.024477505	Arabidopsis thaliana chromosome 3, complete sequence
AT2G29950	1.237845594	0.024477505	Arabidopsis thaliana chromosome 2, complete sequence
AT1G28990	1.88992229	0.026407276	Arabidopsis thaliana chromosome 1 sequence
AT3G13210	1.88992229	0.026407276	Arabidopsis thaliana putative crooked neck protein / cell cycle protein mRNA, complete cds
AT4G03540	1.88992229	0.026407276	Arabidopsis thaliana uncharacterized protein mRNA, complete cds
AT2G29220	1.88992229	0.026407276	Arabidopsis thaliana chromosome 2, complete sequence
AT1G15360	2.185378174	0.027365204	Arabidopsis thaliana ethylene-responsive transcription factor WIN1 mRNA, complete cds
AT1G21360	2.185378174	0.027365204	Arabidopsis thaliana glycolipid transfer protein 2 mRNA, complete cds
AT1G27610	2.185378174	0.027365204	Arabidopsis thaliana uncharacterized protein mRNA, complete cds
AT3G45638	2.185378174	0.027365204	Arabidopsis thaliana clone asmbl_10177 unknown mRNA sequence
AT1G36680.1	2.185378174	0.027365204	Arabidopsis thaliana chromosome 1 sequence
AT4G33280	1.430490672	0.028188244	Arabidopsis thaliana AP2/B3-like transcriptional factor family protein mRNA, complete cds
AT1G51960	1.430490672	0.028188244	Arabidopsis thaliana IQ-domain 27 protein mRNA, complete cds
AT3G59730	1.430490672	0.028188244	Arabidopsis thaliana chromosome 3, complete sequence
AT3G04050	1.074346861	0.028416652	Arabidopsis thaliana pyruvate kinase mRNA, complete cds
AT1G61830	1.152956696	0.029527703	Arabidopsis thaliana chromosome 1 sequence
AT5G23950	1.263380686	0.02968658	Arabidopsis thaliana chromosome 5 sequence

		5	
AT4G11950	1.263380686	0.029686585	Arabidopsis thaliana chromosome 4 sequence
AT4G08250	1.263380686	0.029686585	Arabidopsis thaliana chromosome 4 sequence
AT5G64190	1.295561092	0.035967957	Arabidopsis thaliana uncharacterized protein mRNA, complete cds
AT1G28930	1.167456266	0.036090219	Theobroma cacao Uncharacterized protein (TCM_007238) mRNA, complete cds
AT4G22470	1.167456266	0.036090219	Arabidopsis thaliana chromosome 4 sequence
AT3G16120	1.167456266	0.036090219	Arabidopsis thaliana Dynein light chain type 1 family protein mRNA, complete cds
AT4G36230	1.600415673	0.038951471	Arabidopsis thaliana chromosome 4 sequence
AT4G19680	1.600415673	0.038951471	Arabidopsis thaliana Fe(2+) transport protein 2 mRNA, complete cds
AT1G53080	1.600415673	0.038951471	Arabidopsis thaliana chromosome 1 sequence
AT1G06970	1.600415673	0.038951471	Arabidopsis thaliana cation/H(+) antiporter 14 mRNA, complete cds
AT3G27440	1.600415673	0.038951471	Arabidopsis thaliana uridine kinase-like 5 mRNA, complete cds
AT3G43850	3.337381267	0.039699834	Arabidopsis thaliana uncharacterized protein mRNA, complete cds
AT5G42965	3.337381267	0.039699834	Arabidopsis thaliana chromosome 5 sequence
AT1G66380	3.337381267	0.039699834	Arabidopsis thaliana transcription factor MYB114 mRNA, complete cds
AT5G25180	3.337381267	0.039699834	Arabidopsis thaliana cytochrome P450 71B14 mRNA, complete cds
AT1G69660	3.337381267	0.039699834	Arabidopsis thaliana TRAF-like family protein mRNA, complete cds
AT3G46140	3.337381267	0.039699834	Arabidopsis thaliana chromosome 3, complete sequence
AT1G48150	3.337381267	0.039699834	Arabidopsis thaliana chromosome 1 sequence
AT3G20075	3.337381267	0.039699834	Arabidopsis thaliana chromosome 3, complete sequence
AT2G39160	3.337381267	0.039699834	Arabidopsis thaliana uncharacterized protein mRNA, complete cds
AT1G27461	3.337381267	0.039699834	Arabidopsis thaliana chromosome 1 sequence

AT1G50760	3.337381267	0.039699834	Arabidopsis thaliana Aminotransferase-like, plant mobile domain family protein mRNA, complete cds
AT5G11050	3.337381267	0.039699834	Arabidopsis thaliana myb domain protein 64 mRNA, complete cds
AT5G65100	3.337381267	0.039699834	Arabidopsis thaliana chromosome 5 sequence
AT3G10240	3.337381267	0.039699834	Arabidopsis thaliana chromosome 3, complete sequence
AT2G21610	3.337381267	0.039699834	Arabidopsis thaliana pectinesterase 11 mRNA, complete cds
AT1G36610.1	3.337381267	0.039699834	Arabidopsis thaliana chromosome 1 sequence
AT2G10400.1	3.337381267	0.039699834	Arabidopsis thaliana chromosome 2, complete sequence
AT2G30615	3.337381267	0.039699834	Arabidopsis thaliana uncharacterized protein mRNA, complete cds
AT1G75790	3.337381267	0.039699834	Arabidopsis thaliana SKU5 similar 18 protein mRNA, complete cds
AT5G40940	3.337381267	0.039699834	Arabidopsis thaliana chromosome 5 sequence
AT3G55860	3.337381267	0.039699834	Arabidopsis thaliana uncharacterized protein mRNA, complete cds
AT5G20045	3.337381267	0.039699834	Arabidopsis thaliana chromosome 5 sequence
AT3G49700	3.337381267	0.039699834	Arabidopsis thaliana 1-aminocyclopropane-1-carboxylate synthase 9 mRNA, complete cds
AT5G05770	3.337381267	0.039699834	Arabidopsis thaliana chromosome 5 sequence
AT4G25920	3.337381267	0.039699834	Arabidopsis thaliana uncharacterized protein mRNA, complete cds
AT3G42047	3.337381267	0.039699834	Arabidopsis thaliana chromosome 3, complete sequence
AT1G21528	3.337381267	0.039699834	Arabidopsis thaliana chromosome 1 sequence
AT1G55525	3.337381267	0.039699834	Arabidopsis thaliana clone 155459 mRNA sequence
AT4G09595.1	3.337381267	0.039699834	Arabidopsis thaliana chromosome 4 sequence
AT3G01270	3.337381267	0.039699834	Arabidopsis thaliana putative pectate lyase 7 mRNA, complete cds
AT3G12850	3.337381267	0.039699834	Arabidopsis thaliana COP9 signalosome complex-related protein mRNA, complete cds

AT3G05310	3.337381267	0.039699834	Arabidopsis thaliana MIRO-related GTP-ase 3 mRNA, complete cds
AT1G68630	3.337381267	0.039699834	Arabidopsis thaliana PLAC8 family protein mRNA, complete cds
AT2G04840	3.337381267	0.039699834	Arabidopsis thaliana uncharacterized protein mRNA, complete cds
AT1G20925	3.337381267	0.039699834	Arabidopsis thaliana auxin efflux carrier-like protein mRNA, complete cds
AT1G69470	3.337381267	0.039699834	Arabidopsis thaliana chromosome 1 sequence
AT1G66610	3.337381267	0.039699834	Arabidopsis thaliana E3 ubiquitin-protein ligase SINA-like 1 mRNA, complete cds
AT5G54550	3.337381267	0.039699834	Arabidopsis thaliana chromosome 5 sequence
AT4G25330	3.337381267	0.039699834	Arabidopsis thaliana uncharacterized protein mRNA, complete cds
AT5G27570	3.337381267	0.039699834	Arabidopsis thaliana chromosome 5 sequence
AT2G20970	3.337381267	0.039699834	Arabidopsis thaliana uncharacterized protein mRNA, complete cds
AT3G56420	3.337381267	0.039699834	Arabidopsis thaliana putative thioredoxin H10 mRNA, complete cds
AT1G43895	3.337381267	0.039699834	Arabidopsis thaliana chromosome 1 sequence
AT2G30300	3.337381267	0.039699834	Arabidopsis thaliana major facilitator protein mRNA, complete cds
AT3G62320	3.337381267	0.039699834	Arabidopsis thaliana putative nucleic acid binding protein mRNA, complete cds
AT2G15340	3.337381267	0.039699834	Arabidopsis thaliana chromosome 2, complete sequence
AT2G47050	3.337381267	0.039699834	Arabidopsis thaliana chromosome 2, complete sequence
AT2G31100	3.337381267	0.039699834	Arabidopsis thaliana phospholipase A1-IIbeta mRNA, complete cds
AT5G16020	3.337381267	0.039699834	Arabidopsis thaliana gamete-expressed 3 mRNA, complete cds
AT5G52680	3.337381267	0.039699834	Arabidopsis thaliana copper transport family protein mRNA, complete cds
AT1G26140	3.337381267	0.039699834	Arabidopsis thaliana uncharacterized protein mRNA, complete cds
AT4G00236	3.337381267	0.039699834	Arabidopsis thaliana chromosome 4 sequence
AT1G20320	3.337381267	0.039699834	Arabidopsis thaliana chromosome 1 sequence

AT4G12920	3.337381267	0.03969983<br>4	Arabidopsis thaliana chromosome 4 sequence
AT2G21400	3.337381267	0.03969983<br>4	Arabidopsis thaliana SHI-related sequence3 mRNA, complete cds
AT1G09370	3.337381267	0.03969983<br>4	Arabidopsis thaliana chromosome 1 sequence
AT5G57380	3.337381267	0.03969983<br>4	Arabidopsis thaliana protein VERNALIZATION INSENSITIVE 3 mRNA, complete cds
AT3G19700	3.337381267	0.03969983<br>4	Arabidopsis thaliana leucine rich repeat kinase HAIKU 2 mRNA, complete cds
AT1G68610	3.337381267	0.03969983<br>4	Arabidopsis thaliana chromosome 1 sequence
AT5G56510	3.337381267	0.03969983<br>4	Arabidopsis thaliana pumilio 12 mRNA, complete cds
ATCG01090	3.337381267	0.03969983<br>4	Arabidopsis thaliana chloroplast DNA, complete genome, ecotype: Columbia
AT5G20560	3.337381267	0.03969983<br>4	Arabidopsis thaliana chromosome 5 sequence
AT5G22670	3.337381267	0.03969983<br>4	Arabidopsis thaliana F-box/FBD/LRR-repeat protein mRNA, complete cds
AT3G54550	3.337381267	0.03969983<br>4	Arabidopsis thaliana uncharacterized protein mRNA, complete cds
AT1G65140	3.337381267	0.03969983<br>4	Arabidopsis thaliana ubiquitin carboxyl-terminal hydrolase family protein mRNA, complete cds
AT5G53380	3.337381267	0.03969983<br>4	Arabidopsis thaliana O-acyltransferase (WSD1-like) family protein mRNA, complete cds
AT4G26880	3.337381267	0.03969983<br>4	Arabidopsis thaliana chromosome 4 sequence
AT4G34170	3.337381267	0.03969983<br>4	Arabidopsis thaliana putative F-box/kelch-repeat protein mRNA, complete cds
AT4G31960	3.337381267	0.03969983<br>4	Arabidopsis thaliana chromosome 4 sequence
AT1G20500	3.337381267	0.03969983<br>4	Arabidopsis thaliana 4-coumarate--CoA ligase-like 4 mRNA, complete cds
AT1G07645	3.337381267	0.03969983<br>4	Arabidopsis thaliana dessication-induced 1VOC-like protein mRNA, complete cds
AT4G37682	1.015453172	0.040333118	Arabidopsis thaliana uncharacterized protein mRNA, complete cds
AT1G63380	1.0858425	0.04276668<br>9	Arabidopsis thaliana Rossmann-fold NAD(P)-binding domain-containing protein

			mRNA, complete cds
AT5G59760	1.0858425	0.042766689	Arabidopsis thaliana uncharacterized protein mRNA, complete cds
AT3G12460	1.337381267	0.043490474	Arabidopsis thaliana chromosome 3, complete sequence
AT4G27980	1.337381267	0.043490474	Arabidopsis thaliana chromosome 4 sequence
AT5G18180	1.337381267	0.043490474	Arabidopsis thaliana H/ACA ribonucleoprotein complex, subunit Gar1/Naf1 protein mRNA, complete cds
AT2G40740	1.337381267	0.043490474	Arabidopsis thaliana WRKY transcription factor 55 mRNA, complete cds
AT4G03470	1.185378174	0.044154853	Arabidopsis thaliana ankyrin repeat-containing protein mRNA, complete cds
AT2G35075	1.185378174	0.044154853	Arabidopsis thaliana uncharacterized protein mRNA, complete cds
AT1G08440	1.752418767	0.044413649	Arabidopsis thaliana aluminum-activated malate transporter 2 mRNA, complete cds
AT4G02320	1.752418767	0.044413649	Arabidopsis thaliana probable pectinesterase/pectinesterase inhibitor 40 mRNA, complete cds
AT3G60966	1.752418767	0.044413649	Arabidopsis thaliana chromosome 3, complete sequence
AT4G10950	1.752418767	0.044413649	Arabidopsis thaliana SGNH hydrolase-type esterase superfamily protein mRNA, complete cds
AT1G48400	1.752418767	0.044413649	Arabidopsis thaliana F-box/RNI-like/FBD-like domain-containing protein mRNA, complete cds
AT4G03220	2.600415673	0.046067282	Arabidopsis thaliana putative F-box/FBD/LRR-repeat protein mRNA, complete cds
AT5G51480	2.600415673	0.046067282	Arabidopsis thaliana Monocopper oxidase-like protein SKS2 mRNA, complete cds
AT3G62850	2.600415673	0.046067282	Arabidopsis thaliana zinc finger protein-like protein mRNA, complete cds
AT1G18835	2.600415673	0.046067282	Arabidopsis thaliana chromosome 1 sequence
AT2G07080.1	2.600415673	0.046067282	Arabidopsis thaliana chromosome 2, complete sequence
AT2G22590	2.600415673	0.046067282	Arabidopsis thaliana chromosome 2, complete sequence

AT4G21490	2.600415673	0.046067282	Arabidopsis thaliana NAD(P)H dehydrogenase B3 mRNA, complete cds
AT1G65200	2.600415673	0.046067282	Arabidopsis thaliana ubiquitin carboxyl-terminal hydrolase-related protein mRNA, complete cds
ATCG01070	2.600415673	0.046067282	Arabidopsis thaliana chloroplast DNA, complete genome, ecotype: Columbia
AT1G24570	2.600415673	0.046067282	Arabidopsis thaliana uncharacterized protein mRNA, complete cds
AT5G05530	2.600415673	0.046067282	Arabidopsis thaliana chromosome 5 sequence
AT5G58050	2.600415673	0.046067282	Arabidopsis thaliana glycerophosphodiester phosphodiesterase like 6 mRNA, complete cds
AT1G35310	2.600415673	0.046067282	Arabidopsis thaliana MLP-like protein 168 mRNA, complete cds
AT4G37290	2.600415673	0.046067282	Arabidopsis thaliana chromosome 4 sequence
AT2G23945	2.600415673	0.046067282	Arabidopsis thaliana chromosome 2, complete sequence
AT4G02950	2.600415673	0.046067282	Arabidopsis thaliana chromosome 4 sequence
AT2G30760	2.600415673	0.046067282	Arabidopsis thaliana uncharacterized protein mRNA, complete cds
AT5G09500	2.600415673	0.046067282	Arabidopsis thaliana 40S ribosomal protein S15-3 mRNA, complete cds
AT3G08900	2.600415673	0.046067282	Arabidopsis thaliana UDP-arabinopyranose mutase 3 mRNA, complete cds
AT2G03230	2.600415673	0.046067282	Arabidopsis thaliana chromosome 2, complete sequence
AT2G20825	2.015453172	0.048191164	Arabidopsis thaliana protein ULTRAPETALA 2 mRNA, complete cds
AT1G15900	2.015453172	0.048191164	Arabidopsis thaliana chromosome 1 sequence
AT5G63750	2.015453172	0.048191164	Arabidopsis thaliana putative E3 ubiquitin-protein ligase ARI13 mRNA, complete cds
AT4G12500	2.015453172	0.048191164	Arabidopsis thaliana chromosome 4 sequence
AT5G04150	2.015453172	0.048191164	Arabidopsis thaliana transcription factor bHLH101 mRNA, complete cds
AT1G28950	2.015453172	0.048191164	Theobroma cacao Uncharacterized protein (TCM_007238) mRNA, complete cds
AT3G45280	2.015453172	0.048191164	Arabidopsis thaliana syntaxin-72 mRNA, complete cds
AT1G19750	2.015453172	0.048191164	Arabidopsis thaliana UV-B response

			protein-like protein CSAat1b mRNA, complete cds
AT1G24260	1.015453172	0.049642363	Arabidopsis thaliana MADs box transcription factor SEPALLATA3 mRNA, complete cds
AT2G43600	1.015453172	0.049642363	Arabidopsis thaliana chitinase family protein mRNA, complete cds
AT1G02450	1.393964796	0.052383971	Arabidopsis thaliana chromosome 1 sequence
AT4G38092	1.093455684	0.05261766	Arabidopsis thaliana uncharacterized protein mRNA, complete cds
AT1G26558	1.093455684	0.05261766	Arabidopsis thaliana Full-length cDNA Complete sequence from clone GSLTLS18ZE08 of Adult vegetative tissue of strain col-0 of Arabidopsis thaliana (thale cress)
AT5G66150	1.20809825	0.054068822	Arabidopsis thaliana Glycosyl hydrolase family protein mRNA, complete cds
AT4G18990	1.20809825	0.054068822	Arabidopsis thaliana probable xyloglucan endotransglucosylase/hydrolase protein 29 mRNA, complete cds
AT1G32510	1.20809825	0.054068822	Arabidopsis thaliana NAC domain containing protein 11 mRNA, complete cds
AT5G66710	1.20809825	0.054068822	Arabidopsis thaliana protein kinase family protein mRNA, complete cds
AT1G78476	1.015453172	0.061251132	Arabidopsis thaliana uncharacterized protein mRNA, complete cds
AT4G27810	1.474884791	0.062630127	Arabidopsis thaliana uncharacterized protein mRNA, complete cds
AT2G36750	1.474884791	0.062630127	Arabidopsis thaliana chromosome 2, complete sequence
AT5G41540	1.474884791	0.062630127	Arabidopsis thaliana TIR-NBS-LRR class disease resistance protein mRNA, complete cds
AT1G33640	1.474884791	0.062630127	Arabidopsis thaliana uncharacterized protein mRNA, complete cds
AT3G56890	1.474884791	0.062630127	Arabidopsis thaliana F-box associated ubiquitination effector family protein mRNA, complete cds
AT4G10020	1.474884791	0.062630127	Arabidopsis thaliana hydroxysteroid dehydrogenase 5 mRNA, complete cds
AT3G09870	1.474884791	0.062630127	Arabidopsis thaliana chromosome 3, complete sequence
AT1G20080	1.474884791	0.062630127	Arabidopsis thaliana synaptotagmin-2 mRNA, complete cds

AT3G54450	1.102916014	0.064886511	Arabidopsis thaliana probable peptide/nitrate transporter mRNA, complete cds
AT4G27590	1.102916014	0.064886511	Arabidopsis thaliana heavy metal associated domain-containing protein mRNA, complete cds
AT2G33000	1.102916014	0.064886511	Arabidopsis thaliana ubiquitin-associated (UBA)/TS-N domain-containing protein-like protein mRNA, complete cds
AT1G22600	1.237845594	0.066246832	Arabidopsis thaliana Late embryogenesis abundant protein (LEA) family protein mRNA, complete cds
AT5G43290	1.237845594	0.066246832	Arabidopsis thaliana putative WRKY transcription factor 49 mRNA, complete cds
AT1G43000	1.237845594	0.066246832	Arabidopsis thaliana PLATZ transcription factor family protein mRNA, complete cds
AT5G53820	1.237845594	0.066246832	Arabidopsis thaliana Late embryogenesis abundant protein (LEA) family protein mRNA, complete cds
ATCG00440	1.600415673	0.073762805	Arabidopsis thaliana chloroplast DNA, complete genome, ecotype: Columbia
AT2G27120	1.600415673	0.073762805	Arabidopsis thaliana DNA polymerase epsilon catalytic subunit B mRNA, complete cds
AT1G65680	1.600415673	0.073762805	Arabidopsis thaliana expansin B2 mRNA, complete cds
AT3G14880	1.600415673	0.073762805	Arabidopsis thaliana uncharacterized protein mRNA, complete cds
AT1G55800	1.015453172	0.075793955	Arabidopsis thaliana uncharacterized protein mRNA, complete cds
AT1G69540	1.114988846	0.080227149	Arabidopsis thaliana protein agamous-like 94 mRNA, complete cds
AT1G66860	1.114988846	0.080227149	Arabidopsis thaliana class I glutamine amidotransferase-like domain-containing protein mRNA, complete cds
AT3G56780	1.114988846	0.080227149	Arabidopsis thaliana putative F-box/FBD/LRR-repeat protein mRNA, complete cds
AT2G43310	1.114988846	0.080227149	Arabidopsis thaliana chromosome 2, complete sequence
AT1G52270	1.278487578	0.081158012	Arabidopsis thaliana chromosome 1 sequence
AT1G80390	1.278487578	0.081158012	Arabidopsis thaliana auxin-responsive protein IAA15 mRNA, complete cds
AT5G52290	1.278487578	0.081158012	Arabidopsis thaliana protein SHORTAGE IN CHIASMATA 1 mRNA, complete cds

AT4G01980.1	1.822808094	0.083957658	Arabidopsis thaliana chromosome 4 sequence
AT1G48020	1.822808094	0.083957658	Arabidopsis thaliana chromosome 1 sequence
AT1G66173	1.822808094	0.083957658	Arabidopsis thaliana chromosome 1 sequence
AT5G01225	1.822808094	0.083957658	Arabidopsis thaliana chromosome 5 sequence
AT4G10490	1.822808094	0.083957658	Arabidopsis thaliana oxidoreductase, 2OG-Fe(II) oxygenase family protein mRNA, complete cds
AT3G22860	1.822808094	0.083957658	Arabidopsis thaliana eukaryotic translation initiation factor 3 subunit C2 mRNA, complete cds
AT1G76610	1.822808094	0.083957658	Arabidopsis thaliana chromosome 1 sequence
AT3G21880	1.822808094	0.083957658	Arabidopsis thaliana B-box type zinc finger protein with CCT domain mRNA, complete cds
AT4G28170	1.822808094	0.083957658	Arabidopsis thaliana uncharacterized protein mRNA, complete cds
AT5G53048	1.822808094	0.083957658	Arabidopsis thaliana AT5g53050/MNB8_11 mRNA, complete cds
AT4G19000	1.822808094	0.083957658	Arabidopsis thaliana INTERACTS WITH SPT6-like protein IWS2 mRNA, complete cds
AT1G62420	1.822808094	0.083957658	Arabidopsis thaliana uncharacterized protein mRNA, complete cds
AT3G55210	1.822808094	0.083957658	Arabidopsis thaliana NAC domain containing protein 63 mRNA, complete cds
AT3G03240	1.822808094	0.083957658	Arabidopsis thaliana alpha/beta-Hydrolases superfamily protein mRNA, complete cds
AT2G12420.1	1.822808094	0.083957658	Arabidopsis thaliana chromosome 2, complete sequence
AT4G27580	2.337381267	0.086691999	Arabidopsis thaliana uncharacterized protein mRNA, complete cds
AT5G43300	2.337381267	0.086691999	Arabidopsis thaliana glycerophosphodiester phosphodiesterase mRNA, complete cds
AT2G36710	2.337381267	0.086691999	Arabidopsis thaliana putative pectinesterase 15 mRNA, complete cds
AT1G05330	2.337381267	0.086691999	Arabidopsis thaliana chromosome 1 sequence
AT4G27920	2.337381267	0.086691999	Arabidopsis thaliana abscisic acid receptor PYL10 mRNA, complete cds

AT4G35170	2.337381267	0.086691999	Arabidopsis thaliana late embryogenesis abundant hydroxyproline-rich glycoprotein mRNA, complete cds
AT5G27345.1	2.337381267	0.086691999	Arabidopsis thaliana chromosome 5 sequence
AT2G47430	2.337381267	0.086691999	Arabidopsis thaliana histidine kinase CKI1 mRNA, complete cds
AT3G29190	2.337381267	0.086691999	Arabidopsis thaliana terpenoid synthase 15 mRNA, complete cds
AT4G05095	2.337381267	0.086691999	Arabidopsis thaliana chromosome 4 sequence
AT1G21890	2.337381267	0.086691999	Arabidopsis thaliana WAT1-related protein mRNA, complete cds
AT3G28915.1	2.337381267	0.086691999	Arabidopsis thaliana chromosome 3, complete sequence
AT5G47670	2.337381267	0.086691999	Arabidopsis thaliana chromosome 5 sequence
AT2G27650	2.337381267	0.086691999	Arabidopsis thaliana ubiquitin carboxyl-terminal hydrolase-related protein mRNA, complete cds
AT1G11590	2.337381267	0.086691999	Arabidopsis thaliana probable pectinesterase/pectinesterase inhibitor 19 mRNA, complete cds
AT5G66890	2.337381267	0.086691999	Arabidopsis thaliana putative disease resistance protein mRNA, complete cds
AT1G32850	2.337381267	0.086691999	Arabidopsis thaliana putative ubiquitin carboxyl-terminal hydrolase 11 mRNA, complete cds
AT2G31590	2.337381267	0.086691999	Arabidopsis thaliana uncharacterized protein mRNA, complete cds
AT1G22110	2.337381267	0.086691999	Arabidopsis thaliana chromosome 1 sequence
AT1G31570.1	2.337381267	0.086691999	Arabidopsis thaliana chromosome 1 sequence
AT2G23110	2.337381267	0.086691999	Arabidopsis thaliana Late embryogenesis abundant protein, group 6 mRNA, complete cds
AT5G49200	2.337381267	0.086691999	Arabidopsis thaliana chromosome 5 sequence
AT3G13500	2.337381267	0.086691999	Arabidopsis thaliana chromosome 3, complete sequence
AT4G26790	2.337381267	0.086691999	Arabidopsis thaliana GDSL esterase/lipase mRNA, complete cds
AT3G22250	2.337381267	0.08669199	Arabidopsis thaliana

		9	UDP-glycosyltransferase 82A1 mRNA, complete cds
AT1G14686	2.337381267	0.086691999	Arabidopsis thaliana chromosome 1 sequence
AT4G06535.1	2.337381267	0.086691999	Arabidopsis thaliana chromosome 4 sequence
AT1G07885	2.337381267	0.086691999	Arabidopsis thaliana uncharacterized protein mRNA, complete cds
AT2G39820	2.337381267	0.086691999	Arabidopsis thaliana translation initiation factor eIF-6 mRNA, complete cds
AT5G09470	2.337381267	0.086691999	Arabidopsis thaliana dicarboxylate carrier 3 mRNA, complete cds
AT3G58380	2.337381267	0.086691999	Arabidopsis thaliana TRAF-like family protein mRNA, complete cds
AT1G27890	2.337381267	0.086691999	Arabidopsis thaliana chromosome 1 sequence
AT5G41310	2.337381267	0.086691999	Arabidopsis thaliana kinesin-like protein mRNA, complete cds
AT5G62240	2.337381267	0.086691999	Arabidopsis thaliana Cell cycle regulated microtubule associated protein mRNA, complete cds
AT1G21470	2.337381267	0.086691999	Arabidopsis thaliana uncharacterized protein mRNA, complete cds
AT5G02244	2.337381267	0.086691999	Arabidopsis thaliana mRNA for hypothetical protein, complete cds, clone: RAFL16-87-M17
AT4G31680	2.337381267	0.086691999	Arabidopsis thaliana transcriptional factor B3 family protein mRNA, complete cds
AT2G17890	2.337381267	0.086691999	Arabidopsis thaliana calcium-dependent protein kinase 16 mRNA, complete cds
AT4G35590	1.015453172	0.094114928	Arabidopsis thaliana RWP-RK domain-containing protein mRNA, complete cds
AT1G04580	1.015453172	0.094114928	Arabidopsis thaliana aldehyde oxidase 4 mRNA, complete cds
AT5G20790	1.015453172	0.094114928	Arabidopsis thaliana chromosome 5 sequence
AT4G37410	1.015453172	0.094114928	Arabidopsis thaliana cytochrome P450, family 81, subfamily F, polypeptide 4 mRNA, complete cds
AT5G66830	1.337381267	0.099254037	Arabidopsis thaliana chromosome 5 sequence
AT3G21755	1.337381267	0.099254037	Arabidopsis thaliana clone 151422 mRNA sequence
AT1G49830	1.337381267	0.09925403	Arabidopsis thaliana basic helix-loop-helix

		7	(bHLH) DNA-binding superfamily protein mRNA, complete cds
AT2G34230	1.337381267	0.09925403 7	Arabidopsis thaliana uncharacterized protein mRNA, complete cds
AT3G19310	1.337381267	0.09925403 7	Arabidopsis thaliana PLC-like phosphodiesterase-like protein mRNA, complete cds
AT2G36815	1.337381267	0.09925403 7	Arabidopsis thaliana uncharacterized protein mRNA, complete cds
AT1G17400	1.337381267	0.09925403 7	Arabidopsis thaliana uncharacterized protein mRNA, complete cds
AT5G10680	1.337381267	0.09925403 7	Arabidopsis thaliana chromosome 5 sequence
AT1G73550	1.337381267	0.09925403 7	Arabidopsis thaliana protease inhibitor/seed storage/LTP family protein mRNA, complete cds
AT3G53820	1.337381267	0.09925403 7	Arabidopsis thaliana chromosome 3, complete sequence
AT5G58830	1.13093039	0.09949597 9	Arabidopsis thaliana Subtilisin-like serine endopeptidase family protein mRNA, complete cds
AT5G09970	1.13093039	0.09949597 9	Arabidopsis thaliana cytochrome P450, family 78, subfamily A, polypeptide 7 mRNA, complete cds
AT5G11190	1.13093039	0.09949597 9	Arabidopsis thaliana ethylene-responsive transcription factor SHINE 2 mRNA, complete cds
AT1G62620	1.13093039	0.09949597 9	Arabidopsis thaliana flavin-containing monooxygenase FMO GS-OX-like 3 mRNA, complete cds
AT1G66950	1.13093039	0.09949597 9	Arabidopsis thaliana ABC transporter G family member 39 mRNA, complete cds
AT2G13900	1.13093039	0.09949597 9	Arabidopsis thaliana chromosome 2, complete sequence
AT3G25882	1.015453172	0.117358418	Arabidopsis thaliana chromosome 3, complete sequence
AT2G26750	1.015453172	0.117358418	Arabidopsis thaliana alpha/beta-hydrolases superfamily protein mRNA, complete cds
AT5G66045	1.430490672	0.12069662 1	Arabidopsis thaliana chromosome 5 sequence
AT1G77340	1.430490672	0.12069662 1	Arabidopsis thaliana pentatricopeptide repeat-containing protein mRNA, complete cds
AT1G73445.	1.430490672	0.12069662	Arabidopsis thaliana chromosome 1 sequence

1		1	
AT1G18960	1.430490672	0.120696621	Arabidopsis thaliana myb-like HTH transcriptional regulator family protein mRNA, complete cds
AT1G51920	1.430490672	0.120696621	Arabidopsis thaliana uncharacterized protein mRNA, complete cds
AT1G09176	1.430490672	0.120696621	Arabidopsis thaliana chromosome 1 sequence
AT5G43570	1.430490672	0.120696621	Arabidopsis thaliana PR-6 proteinase inhibitor family protein mRNA, complete cds
AT3G49340	1.430490672	0.120696621	Arabidopsis thaliana putative cysteine proteinase mRNA, complete cds
AT3G44755	1.430490672	0.120696621	Arabidopsis thaliana uncharacterized protein mRNA, complete cds
AT3G46190	1.430490672	0.120696621	Arabidopsis thaliana TRAF-like family protein mRNA, complete cds
AT1G76770	1.152956696	0.123822691	Arabidopsis thaliana alpha-crystallin domain of heat shock protein-containing protein mRNA, complete cds
AT2G28420	1.152956696	0.123822691	Arabidopsis thaliana chromosome 2, complete sequence
AT2G37740	1.152956696	0.123822691	Arabidopsis thaliana chromosome 2, complete sequence
AT1G35610	1.152956696	0.123822691	Arabidopsis thaliana chromosome 1 sequence
AT3G01870	1.152956696	0.123822691	Arabidopsis thaliana chromosome 3, complete sequence

Supplementary Data S4 B Down regulated genes in fc1(+NaCl)/fc1(-NaCl))

Gene	Log2FoldChange	pval	NT:Description
AT5G38420	-1.42752512	0	Arabidopsis thaliana ribulose bisphosphate carboxylase small chain 2B mRNA, complete cds
AT5G02500	-1.08871868	0	Arabidopsis thaliana heat shock cognate protein 70-1 mRNA, complete cds
AT5G38430	-1.717589592	0	Arabidopsis thaliana ribulose bisphosphate carboxylase small chain 1B mRNA, complete cds
AT2G10940	-2.237727333	0	Arabidopsis thaliana chromosome 2, complete sequence
AT3G53460	-1.934053246	0	Arabidopsis thaliana chloroplast RNA-binding protein 29 mRNA, complete cds
AT5G20630	-2.552093816	0	Arabidopsis thaliana chromosome 5 sequence
AT1G77760	-1.752580675	0	Arabidopsis thaliana nitrate reductase [NADH] 1 mRNA, complete cds
AT1G55490	-1.217138552	0	Arabidopsis thaliana chaperonin 60 subunit beta 1 mRNA, complete cds
AT3G14310	-1.902235664	0	Arabidopsis thaliana pectin methylesterase 3 mRNA, complete cds
AT1G69530	-1.914379544	0	Arabidopsis thaliana expansin A1 mRNA, complete cds
AT5G40850	-1.965396872	0	Arabidopsis thaliana urophorphyrin methylase 1 mRNA, complete cds
AT2G45180	-2.34820729	0	Arabidopsis thaliana chromosome 2, complete sequence
AT4G29060	-1.324852447	0	Arabidopsis thaliana elongation factor Ts family protein mRNA, complete cds
AT3G63160	-2.510908915	0	Arabidopsis thaliana chromosome 3, complete sequence
AT1G68520	-1.983667582	0	Arabidopsis thaliana zinc finger protein CONSTANS-LIKE 6 mRNA, complete cds
AT2G41090	-2.048707673	0	Arabidopsis thaliana calmodulin-like protein 10 mRNA, complete cds
AT1G28290	-1.88712903	0	Arabidopsis thaliana arabinogalactan protein 31 mRNA, complete cds
AT2G28000	-1.33424754	0	Arabidopsis thaliana chaperonin-60 alpha mRNA, complete cds
AT1G66200	-1.169471456	0	Arabidopsis thaliana glutamine

			synthetase mRNA, complete cds
AT5G49910	-1.470250148	0	Arabidopsis thaliana heat shock protein 70-2 mRNA, complete cds
AT2G04030	-1.371774593	0	Arabidopsis thaliana chloroplast heat shock protein 90 mRNA, complete cds
AT4G27440	-1.510964431	0	Arabidopsis thaliana light-dependent NADPH:protochlorophyllide oxidoreductase B mRNA, complete cds
AT1G49860	-3.112327124	0	Arabidopsis thaliana glutathione S-transferase (class phi) 14 mRNA, complete cds
AT2G38310	-3.92665682	0	Arabidopsis thaliana chromosome 2, complete sequence
AT1G26250	-2.947634222	0	Arabidopsis thaliana chromosome 1 sequence
AT5G44020	-2.117957092	0	Arabidopsis thaliana HAD superfamily, subfamily IIIB acid phosphatase mRNA, complete cds
AT1G08090	-4.826780754	0	Arabidopsis thaliana nitrate transporter 2:1 mRNA, complete cds
AT2G37660	-1.431835263	0	Arabidopsis thaliana NAD(P)-binding Rossmann-fold-containing protein mRNA, complete cds
AT3G52380	-1.571373276	0	Arabidopsis thaliana chloroplast RNA-binding protein 33 mRNA, complete cds
AT2G45470	-1.882157685	0	Arabidopsis thaliana chromosome 2, complete sequence
AT3G01190	-2.667120125	0	Arabidopsis thaliana peroxidase 27 mRNA, complete cds
AT3G58610	-1.019909189	0	Arabidopsis thaliana ketol-acid reductoisomerase mRNA, complete cds
AT5G12860	-1.157745899	0	Arabidopsis thaliana dicarboxylate transporter 1 mRNA, complete cds
AT4G14400	-2.061113877	0	Arabidopsis thaliana protein ACCELERATED CELL DEATH 6 mRNA, complete cds
AT3G54580	-3.868850738	0	Arabidopsis thaliana chromosome 3, complete sequence
AT4G03210	-2.649097593	0	Arabidopsis thaliana xyloglucan endotransglucosylase/hydrolase 9 mRNA, complete cds
AT2G06850	-1.679451344	1.43E-305	Arabidopsis thaliana endoxyloglucan transferase A1 mRNA, complete cds

AT5G40450	-1.122389649	6.53E-304	Arabidopsis thaliana uncharacterized protein mRNA, complete cds
AT4G12550	-2.284332965	7.95E-304	Arabidopsis thaliana chromosome 4 sequence
AT4G16980	-1.197282777	2.41E-302	Arabidopsis thaliana chromosome 4 sequence
AT5G19780	-1.735207027	1.64E-301	Arabidopsis thaliana tubulin alpha-5 mRNA, complete cds
AT3G24420	-1.911501041	6.60E-298	Arabidopsis thaliana hydrolase, alpha/beta fold family protein mRNA, complete cds
AT4G15390	-2.385727526	2.00E-292	Arabidopsis thaliana chromosome 4 sequence
AT1G21310	-1.573279111	1.01E-287	Arabidopsis thaliana extensin 3 mRNA, complete cds
AT5G61420	-1.996694798	1.91E-286	Arabidopsis thaliana transcription factor MYB28 mRNA, complete cds
AT1G09750	-2.106335308	5.79E-282	Arabidopsis thaliana aspartyl protease-like protein mRNA, complete cds
AT1G03870	-3.170773641	7.14E-281	Arabidopsis thaliana chromosome 1 sequence
AT5G19770	-1.666738199	1.88E-280	Arabidopsis thaliana tubulin alpha-3 mRNA, complete cds
AT1G04820	-1.021957104	2.33E-271	Arabidopsis thaliana tubulin alpha-4 chain mRNA, complete cds
AT5G42530	-1.71907028	1.97E-268	Arabidopsis thaliana uncharacterized protein mRNA, complete cds
AT2G25510	-1.403739956	1.20E-264	Arabidopsis thaliana uncharacterized protein mRNA, complete cds
AT1G16880	-1.038141067	4.81E-256	Arabidopsis thaliana ACT domain-containing protein mRNA, complete cds
AT3G13470	-1.468901418	8.22E-256	Arabidopsis thaliana TCP-1/cpn60 chaperonin family protein mRNA, complete cds
AT4G30190	-1.049571486	4.85E-253	Arabidopsis thaliana H(+)-ATPase 2 mRNA, complete cds
AT2G15620	-1.079493574	1.31E-248	Arabidopsis thaliana Ferredoxin--nitrite reductase mRNA, complete cds
AT1G23720	-2.325187183	1.80E-245	Arabidopsis thaliana chromosome 1 sequence
AT5G08280	-1.18888011	4.75E-244	Arabidopsis thaliana Porphobilinogen deaminase mRNA, complete cds

AT1G29660	-2.052883957	1.10E-243	Arabidopsis thaliana GDSL esterase/lipase mRNA, complete cds
AT1G49750	-1.728349852	1.72E-242	Arabidopsis thaliana leucine-rich repeat (LRR) family protein mRNA, complete cds
AT1G13110	-2.616640076	6.66E-239	Arabidopsis thaliana cytochrome P450 71B7 mRNA, complete cds
AT3G54590	-5.003330303	7.75E-233	Arabidopsis thaliana hydroxyproline-rich glycoprotein mRNA, complete cds
AT5G61790	-1.387115642	2.55E-232	Arabidopsis thaliana calnexin 1 mRNA, complete cds
AT2G43150	-1.616083886	2.18E-231	Arabidopsis thaliana Proline-rich extensin-like family protein mRNA, complete cds
AT5G44340	-1.162490777	1.46E-228	Arabidopsis thaliana tubulin beta-4 chain mRNA, complete cds
AT1G75500	-1.258246642	2.31E-227	Arabidopsis thaliana protein walls are thin 1 mRNA, complete cds
AT5G28540	-1.268324719	3.78E-226	Arabidopsis thaliana Luminal-binding protein 1 mRNA, complete cds
AT3G27850	-1.031935645	5.27E-226	Arabidopsis thaliana chromosome 3, complete sequence
AT3G01120	-1.035574923	1.40E-221	Arabidopsis thaliana cystathionine gamma-synthase mRNA, complete cds
AT1G25440	-1.168258781	1.15E-219	Arabidopsis thaliana zinc finger protein CONSTANS-LIKE 16 mRNA, complete cds
AT1G75750	-1.798224748	2.36E-218	Arabidopsis thaliana GA-responsive GAST1 protein-like protein mRNA, complete cds
AT3G28550	-3.11800552	1.21E-209	Arabidopsis thaliana chromosome 3, complete sequence
AT4G12420	-1.227723591	1.38E-209	Arabidopsis thaliana multi-copper oxidase-like protein SKU5 mRNA, complete cds
AT1G06950	-1.321774059	3.60E-206	Arabidopsis thaliana protein TIC110 mRNA, complete cds
AT4G20260	-1.24257252	5.23E-203	Arabidopsis thaliana plasma-membrane associated cation-binding protein 1 mRNA, complete cds
AT1G68590	-1.377085133	9.85E-202	Arabidopsis thaliana 30S ribosomal protein 3-1 mRNA, complete cds
AT3G04790	-1.204028388	1.24E-198	Arabidopsis thaliana chromosome 3, complete sequence

AT5G14120	-1.373589118	2.48E-196	Arabidopsis thaliana major facilitator protein mRNA, complete cds
AT2G29980	-1.0368316	5.29E-196	Arabidopsis thaliana omega-3 fatty acid desaturase mRNA, complete cds
AT5G38940	-2.590914692	5.20E-195	Arabidopsis thaliana germin-like protein subfamily 1 member 11 mRNA, complete cds
AT4G25050	-1.003361137	2.82E-190	Arabidopsis thaliana acyl carrier protein 4 mRNA, complete cds
AT1G20010	-1.832969999	6.90E-190	Arabidopsis thaliana tubulin beta-5 chain mRNA, complete cds
AT5G51550	-1.697443872	3.54E-188	Arabidopsis thaliana chromosome 5 sequence
AT3G52720	-1.585411823	9.75E-188	Arabidopsis thaliana alpha carbonic anhydrase 1 mRNA, complete cds
AT1G72150	-1.030866731	1.11E-186	Arabidopsis thaliana patellin-1 mRNA, complete cds
AT3G32980	-1.358540908	2.28E-185	Arabidopsis thaliana Peroxidase family protein mRNA, complete cds
AT1G09690	-1.013858008	2.39E-183	Arabidopsis thaliana 60S ribosomal protein L21-1 mRNA, complete cds
AT3G01480	-1.084034816	2.57E-183	Arabidopsis thaliana peptidyl-prolyl cis-trans isomerase CYP38 mRNA, complete cds
AT5G42020	-1.177465781	2.61E-182	Arabidopsis thaliana Luminal-binding protein 2 mRNA, complete cds
AT4G17520	-1.149662224	1.47E-181	Arabidopsis thaliana hyaluronan / mRNA binding family mRNA, complete cds
AT1G15380	-1.810383876	1.23E-180	Arabidopsis thaliana Lactoylglutathione lyase / glyoxalase I family protein mRNA, complete cds
AT4G02290	-1.334100828	3.67E-180	Arabidopsis thaliana glycosyl hydrolase 9B13 mRNA, complete cds
AT1G28400	-1.202119551	1.42E-179	Arabidopsis thaliana chromosome 1 sequence
AT2G18300	-2.522642723	1.70E-177	Arabidopsis thaliana transcription factor bHLH64 mRNA, complete cds
AT1G63940	-1.307636981	2.14E-177	Arabidopsis thaliana Monodehydroascorbate reductase mRNA, complete cds
AT4G30170	-2.300300755	2.38E-175	Arabidopsis thaliana peroxidase 45 mRNA, complete cds
AT4G34950	-2.980442429	4.85E-175	Arabidopsis thaliana major facilitator family protein mRNA, complete cds

AT3G22120	-2.500425016	1.17E-174	Arabidopsis thaliana chromosome 3, complete sequence
AT3G44890	-1.096866836	2.07E-174	Arabidopsis thaliana 50S ribosomal protein L9 mRNA, complete cds
AT5G67400	-4.167166902	4.01E-172	Arabidopsis thaliana peroxidase 73 mRNA, complete cds
AT2G23350	-1.170903613	9.60E-170	Arabidopsis thaliana poly(A) binding protein 4 mRNA, complete cds
AT4G12545	-2.906689507	1.01E-169	Arabidopsis thaliana chromosome 4 sequence
AT2G21660	-1.535230702	1.39E-169	Arabidopsis thaliana glycine-rich RNA-binding protein 7 mRNA, complete cds
AT4G12520	-4.775960206	4.59E-165	Arabidopsis thaliana chromosome 4 sequence
AT1G12110	-1.253528164	5.14E-164	Arabidopsis thaliana nitrate transporter 1.1 mRNA, complete cds
AT2G23600	-1.44998218	3.37E-161	Arabidopsis thaliana methylesterase 2 mRNA, complete cds
AT5G13510	-1.134229927	6.40E-161	Arabidopsis thaliana chromosome 5 sequence
AT1G62780	-1.472868233	2.56E-157	Arabidopsis thaliana uncharacterized protein mRNA, complete cds
AT3G62250	-1.055686777	1.71E-156	Arabidopsis thaliana chromosome 3, complete sequence
AT4G22490	-2.789016359	1.27E-155	Arabidopsis thaliana chromosome 4 sequence
AT5G17820	-2.712113724	2.51E-154	Arabidopsis thaliana peroxidase 57 mRNA, complete cds
AT5G43350	-2.182421077	2.14E-153	Arabidopsis thaliana inorganic phosphate transporter 1-1 mRNA, complete cds
AT5G14200	-1.412408367	8.20E-153	Arabidopsis thaliana isopropylmalate dehydrogenase 1 mRNA, complete cds
AT1G16400	-3.178403338	1.69E-150	Arabidopsis thaliana Hexahomomethionine N-hydroxylase mRNA, complete cds
AT4G17390	-1.074968207	1.86E-148	Arabidopsis thaliana 60S ribosomal protein L15-2 mRNA, complete cds
AT5G62720	-1.956839898	7.38E-148	Arabidopsis thaliana HPP integral membrane domain-containing protein mRNA, complete cds
AT3G08740	-1.155279448	2.33E-147	Arabidopsis thaliana elongation factor EF-P mRNA, complete cds
AT1G60960	-2.732216091	1.56E-144	Arabidopsis thaliana Fe(2+) transport

			protein 3 mRNA, complete cds
AT3G23990	-1.168270679	1.10E-143	Arabidopsis thaliana heat shock protein 60 mRNA, complete cds
AT4G13495	-1.275173985	5.99E-142	Arabidopsis thaliana mRNA for hypothetical protein, complete cds, clone: RAFL25-35-C19
AT1G22530	-2.032960178	2.34E-140	Arabidopsis thaliana patellin 2 mRNA, complete cds
AT3G23940	-1.45060833	4.48E-139	Arabidopsis thaliana dehydratase family protein mRNA, complete cds
AT5G26280	-1.276095296	9.82E-139	Arabidopsis thaliana TRAF-like family protein mRNA, complete cds
AT1G23090	-1.486443454	1.35E-138	Arabidopsis thaliana sulfate transporter 3.3 mRNA, complete cds
AT4G12730	-1.721512422	1.46E-138	Arabidopsis thaliana chromosome 4 sequence
AT5G28500	-1.120261633	1.61E-137	Arabidopsis thaliana chromosome 5 sequence
AT1G03475	-1.082871785	2.14E-137	Arabidopsis thaliana coproporphyrinogen III oxidase mRNA, complete cds
AT5G13420	-1.303747423	1.03E-135	Arabidopsis thaliana Aldolase-type TIM barrel family protein mRNA, complete cds
AT2G47240	-1.395011597	5.55E-133	Arabidopsis thaliana long chain acyl-CoA synthetase 1 mRNA, complete cds
AT3G28040	-1.54245303	3.97E-132	Arabidopsis thaliana probably inactive leucine-rich repeat receptor-like protein kinase mRNA, complete cds
AT5G22880	-1.35773239	1.85E-131	Arabidopsis thaliana chromosome 5 sequence
AT3G52960	-1.098983515	8.22E-131	Arabidopsis thaliana chromosome 3, complete sequence
AT5G65010	-1.20600213	1.14E-129	Arabidopsis thaliana asparagine synthetase 2 mRNA, complete cds
AT1G56340	-1.06193686	2.19E-129	Arabidopsis thaliana calreticulin-1 mRNA, complete cds
AT5G48430	-2.391722209	1.15E-128	Arabidopsis thaliana chromosome 5 sequence
AT2G28950	-1.419347582	1.73E-127	Arabidopsis thaliana expansin A6 mRNA, complete cds
AT1G30510	-1.779666807	3.57E-127	Arabidopsis thaliana ferredoxin--NADP reductase, root isozyme 2 mRNA, complete cds

AT3G48730	-1.14862126	1.13E-126	Arabidopsis thaliana glutamate-1-semialdehyde 2,1-aminomutase 2 mRNA, complete cds
AT1G66270	-1.724473822	2.16E-126	Arabidopsis thaliana beta-glucosidase 21 mRNA, complete cds
AT3G60245	-1.023803188	7.75E-126	Arabidopsis thaliana zinc-binding ribosomal protein family protein mRNA, complete cds
AT3G52150	-1.029957287	2.75E-125	Arabidopsis thaliana RNA recognition motif-containing protein mRNA, complete cds
AT5G55220	-1.037692493	3.93E-125	Arabidopsis thaliana trigger factor type chaperone family protein mRNA, complete cds
AT5G63420	-1.791366327	9.41E-123	Arabidopsis thaliana RNase J mRNA, complete cds
AT3G22231	-4.131751753	3.75E-122	Arabidopsis thaliana protein PATHOGEN AND CIRCADIAN CONTROLLED 1 mRNA, complete cds
AT1G09590	-1.153211283	2.62E-121	Arabidopsis thaliana 60S ribosomal protein L21-1 mRNA, complete cds
AT1G04040	-2.156552139	3.62E-121	Arabidopsis thaliana HAD superfamily, subfamily IIIB acid phosphatase mRNA, complete cds
AT1G21130	-1.436266125	1.48E-120	Arabidopsis thaliana Indole glucosinolate O-methyltransferase 4 mRNA, complete cds
AT5G56850	-1.027885702	2.90E-120	Arabidopsis thaliana uncharacterized protein mRNA, complete cds
AT1G52190	-1.800786736	1.18E-119	Arabidopsis thaliana nitrate transporter 1.11 mRNA, complete cds
AT5G64290	-1.091462031	1.39E-119	Arabidopsis thaliana dicarboxylate transport 2.1 mRNA, complete cds
AT3G53190	-1.533799388	1.47E-119	Arabidopsis thaliana putative pectate lyase 12 mRNA, complete cds
AT3G46740	-1.242176429	1.06E-118	Arabidopsis thaliana protein TOC75-3 mRNA, complete cds
AT4G21210	-1.157215557	1.60E-118	Arabidopsis thaliana pyruvate, phosphate dikinase regulatory protein 1 mRNA, complete cds
AT5G58250	-1.290412535	3.71E-118	Arabidopsis thaliana uncharacterized protein mRNA, complete cds
AT5G07580	-2.594600309	1.35E-117	Arabidopsis thaliana chromosome 5 sequence

AT1G54000	-1.698075867	3.80E-117	Arabidopsis thaliana GDSL esterase/lipase mRNA, complete cds
AT1G56110	-1.230661392	2.21E-114	Arabidopsis thaliana nucleolar protein NOP56-like protein mRNA, complete cds
AT5G33355	-3.11938937	1.66E-113	Arabidopsis thaliana defensin-like protein mRNA, complete cds
AT5G14920	-2.204386258	1.07E-112	Arabidopsis thaliana gibberellin-regulated protein 14 mRNA, complete cds
AT3G04210	-2.499631504	4.66E-112	Arabidopsis thaliana TIR-NBS class disease resistance protein mRNA, complete cds
AT4G34830	-1.090777154	7.80E-112	Arabidopsis thaliana pentatricopeptide repeat protein MRL1 mRNA, complete cds
AT4G17560	-1.009361596	1.96E-111	Arabidopsis thaliana 50S ribosomal protein L19-1 mRNA, complete cds
AT3G51740	-1.485838628	4.40E-111	Arabidopsis thaliana probably inactive leucine-rich repeat receptor-like protein kinase IMK2 mRNA, complete cds
AT3G54920	-1.070846674	2.33E-110	Arabidopsis thaliana putative pectate lyase mRNA, complete cds
AT4G13770	-1.112626641	3.65E-110	Arabidopsis thaliana cytochrome P450 83A1 mRNA, complete cds
AT1G71880	-1.07861909	4.21E-110	Arabidopsis thaliana sucrose transport protein SUC1 mRNA, complete cds
AT5G12110	-2.052009795	7.48E-108	Arabidopsis thaliana elongation factor 1-beta 1 mRNA, complete cds
AT3G47650	-1.023929366	5.16E-107	Arabidopsis thaliana DnaJ/Hsp40 cysteine-rich domain-containing protein mRNA, complete cds
AT2G40490	-1.064235716	1.53E-106	Arabidopsis thaliana Uroporphyrinogen decarboxylase mRNA, complete cds
AT5G65730	-1.738246438	2.27E-106	Arabidopsis thaliana probable xyloglucan endotransglucosylase/hydrolase protein 6 mRNA, complete cds
AT1G72430	-2.453316311	1.38E-105	Arabidopsis thaliana chromosome 1 sequence
AT4G26010	-3.14486983	1.60E-105	Arabidopsis thaliana peroxidase 44 mRNA, complete cds
AT4G09320	-1.058395857	4.61E-103	Arabidopsis thaliana nucleoside diphosphate kinase 1 mRNA, complete cds

AT1G06830	-3.72814116	5.37E-103	Arabidopsis thaliana chromosome 1 sequence
AT5G61590	-2.072788031	6.48E-103	Arabidopsis thaliana chromosome 5 sequence
AT5G63310	-1.110247829	7.46E-103	Arabidopsis thaliana nucleoside diphosphate kinase II mRNA, complete cds
AT3G07770	-1.300869106	9.55E-103	Arabidopsis thaliana HEAT SHOCK PROTEIN 89.1 mRNA, complete cds
AT1G80280	-1.610151313	6.10E-102	Arabidopsis thaliana alpha/beta-Hydrolases superfamily protein mRNA, complete cds
AT5G19890	-2.297541798	1.34E-101	Arabidopsis thaliana peroxidase 59 mRNA, complete cds
AT5G54380	-1.893197881	4.61E-101	Arabidopsis thaliana chromosome 5 sequence
AT5G50200	-1.785364833	3.10E-100	Arabidopsis thaliana high-affinity nitrate transporter 3.1 mRNA, complete cds
AT1G21270	-2.484392715	3.60E-100	Arabidopsis thaliana wall-associated receptor kinase 2 mRNA, complete cds
AT4G08950	-1.710371864	1.43E-99	Arabidopsis thaliana chromosome 4 sequence
AT2G36620	-1.054693808	1.65E-97	Arabidopsis thaliana 60S ribosomal protein L24-1 mRNA, complete cds
AT5G38930	-4.286666441	8.28E-96	Arabidopsis thaliana germin-like protein subfamily 1 member 10 mRNA, complete cds
AT3G49670	-1.758050008	5.82E-94	Arabidopsis thaliana CLAVATA1-related receptor kinase-like protein mRNA, complete cds
AT1G78860	-2.719926171	9.19E-93	Arabidopsis thaliana chromosome 1 sequence
AT5G47500	-1.183798672	1.20E-92	Arabidopsis thaliana pectin methylesterase 5 mRNA, complete cds
AT1G21600	-1.356403503	1.83E-92	Arabidopsis thaliana plastid transcriptionally active 6 mRNA, complete cds
AT2G36460	-1.052574064	2.85E-92	Arabidopsis thaliana fructose-bisphosphate aldolase 6 mRNA, complete cds
AT5G19190	-1.620448249	1.00E-91	Arabidopsis thaliana uncharacterized protein mRNA, complete cds
AT2G24060	-1.234975402	1.77E-91	Arabidopsis thaliana translation initiation factor IF-3 mRNA, complete cds

AT3G06070	-1.771863996	2.26E-90	Arabidopsis thaliana uncharacterized protein mRNA, complete cds
AT5G11420	-1.422871179	2.78E-90	Arabidopsis thaliana uncharacterized protein mRNA, complete cds
AT1G29900	-1.032361071	6.54E-90	Arabidopsis thaliana carbamoyl phosphate synthetase B mRNA, complete cds
AT3G07110	-1.016573147	1.03E-89	Arabidopsis thaliana 60S ribosomal protein L13a-1 mRNA, complete cds
AT2G32230	-1.254225581	1.52E-89	Arabidopsis thaliana proteinaceous RNase P 1 mRNA, complete cds
AT4G11320	-1.012768235	3.59E-89	Arabidopsis thaliana putative cysteine proteinase mRNA, complete cds
AT3G18080	-1.08898844	3.97E-89	Arabidopsis thaliana beta-glucosidase 44 mRNA, complete cds
AT1G01190	-2.214844447	1.33E-88	Arabidopsis thaliana cytochrome P450, family 78, subfamily A, polypeptide 8 mRNA, complete cds
AT4G15393	-2.664104261	2.11E-87	Arabidopsis thaliana cytochrome P450, family 702, subfamily A, polypeptide 5 mRNA, complete cds
AT4G28660	-1.046005507	2.17E-87	Arabidopsis thaliana photosystem II reaction center PSB28 protein mRNA, complete cds
AT2G34640	-1.378169739	1.33E-86	Arabidopsis thaliana plastid transcriptionally active 12 mRNA, complete cds
AT1G02730	-1.365325705	1.50E-86	Arabidopsis thaliana cellulose synthase-like protein D5 mRNA, complete cds
AT4G37080	-1.66765042	7.83E-86	Arabidopsis thaliana uncharacterized protein mRNA, complete cds
AT5G46580	-1.470671339	8.85E-86	Arabidopsis thaliana chromosome 5 sequence
AT5G23020	-1.200636759	9.25E-85	Arabidopsis thaliana methylthioalkylmalate synthase 3 mRNA, complete cds
AT4G13340	-1.774171371	3.03E-84	Arabidopsis thaliana chromosome 4 sequence
AT1G72930	-1.342550713	3.67E-84	Arabidopsis thaliana toll/interleukin-1 receptor-like protein mRNA, complete cds
AT4G31910	-2.366212869	3.98E-84	Arabidopsis thaliana BR-related acyltransferase 1 mRNA, complete cds

AT3G59930	-2.264349447	4.83E-84	Arabidopsis thaliana defensin-like protein 206 mRNA, complete cds
AT3G04550	-1.373384973	5.85E-84	Arabidopsis thaliana chromosome 3, complete sequence
AT4G14630	-1.980917404	1.75E-83	Arabidopsis thaliana germin-like protein 9 mRNA, complete cds
AT3G62680	-5.838376179	3.02E-83	Arabidopsis thaliana proline-rich protein 3 mRNA, complete cds
AT2G28630	-1.675193229	7.36E-83	Arabidopsis thaliana chromosome 2, complete sequence
AT1G13270	-1.801606013	7.66E-83	Arabidopsis thaliana methionine aminopeptidase 1B mRNA, complete cds
AT5G53160	-1.029546812	2.45E-82	Arabidopsis thaliana regulatory component of ABA receptor 3 mRNA, complete cds
AT4G37450	-1.762154406	6.81E-82	Arabidopsis thaliana Lysine-rich arabinogalactan protein 18 mRNA, complete cds
AT4G37540	-1.94856753	1.58E-81	Arabidopsis thaliana LOB domain-containing protein 39 mRNA, complete cds
AT5G10430	-1.403399973	2.09E-81	Arabidopsis thaliana chromosome 5 sequence
AT5G46890	-2.86575078	3.51E-81	Arabidopsis thaliana chromosome 5 sequence
AT1G70600	-1.012869737	3.83E-81	Arabidopsis thaliana chromosome 1 sequence
AT2G16660	-1.486838641	4.15E-81	Arabidopsis thaliana Major facilitator superfamily protein mRNA, complete cds
AT4G28250	-1.282185719	4.86E-81	Arabidopsis thaliana expansin B3 mRNA, complete cds
AT4G12600	-1.383506439	9.46E-81	Arabidopsis thaliana ribosomal protein L7Ae/L30e/S12e/Gadd45 family protein mRNA, complete cds
AT1G02150	-1.141959218	1.52E-80	Arabidopsis thaliana pentatricopeptide repeat-containing protein mRNA, complete cds
AT2G24980	-5.787601612	1.92E-80	Arabidopsis thaliana chromosome 2, complete sequence
AT3G18680	-1.205106091	3.58E-80	Arabidopsis thaliana uridylate kinase-like protein mRNA, complete cds
AT5G14330	-4.060390586	8.40E-80	Arabidopsis thaliana uncharacterized protein mRNA, complete cds

AT1G68890	-1.175288363	1.64E-79	Arabidopsis thaliana protein PHYLLO mRNA, complete cds
AT3G10520	-2.4502104	2.18E-79	Arabidopsis thaliana non-symbiotic hemoglobin 2 mRNA, complete cds
AT1G33470	-1.05779581	2.51E-79	Arabidopsis thaliana RNA recognition motif-containing protein mRNA, complete cds
AT5G64620	-2.478620187	6.27E-79	Arabidopsis thaliana chromosome 5 sequence
AT5G61170	-1.231268578	7.44E-79	Arabidopsis thaliana 40S ribosomal protein S19-3 mRNA, complete cds
AT2G21045	-2.18094404	1.36E-78	Arabidopsis thaliana rhodanese-like domain-containing protein mRNA, complete cds
AT5G05960	-1.699184862	1.67E-78	Arabidopsis thaliana bifunctional inhibitor/lipid-transfer protein/seed storage 2S albumin superfamily protein mRNA, complete cds
AT3G14240	-1.131084804	2.88E-78	Arabidopsis thaliana chromosome 3, complete sequence
AT2G33430	-1.189507427	4.38E-78	Arabidopsis thaliana protein differentiation and greening-like 1 mRNA, complete cds
AT1G15140	-1.273824136	8.56E-78	Arabidopsis thaliana FAD/NAD(P)-binding oxidoreductase mRNA, complete cds
AT3G48500	-1.432460483	6.96E-77	Arabidopsis thaliana RNA binding protein PDE312 mRNA, complete cds
AT5G04730	-2.965438005	1.47E-75	Arabidopsis thaliana Ankyrin-repeat containing protein mRNA, complete cds
AT3G18050	-1.208900765	1.64E-75	Arabidopsis thaliana uncharacterized protein mRNA, complete cds
AT3G03670	-3.828530672	1.59E-74	Arabidopsis thaliana peroxidase mRNA, complete cds
AT4G40090	-4.150247243	2.06E-74	Arabidopsis thaliana chromosome 4 sequence
AT2G14880	-1.400000749	2.14E-74	Arabidopsis thaliana SWIB/MDM2 domain-containing protein mRNA, complete cds
AT5G59680	-2.541603332	3.33E-73	Arabidopsis thaliana leucine-rich repeat protein kinase family protein mRNA, complete cds
AT1G13640	-1.144737946	5.21E-73	Arabidopsis thaliana phosphatidylinositol 4-kinase gamma 6 mRNA, complete cds

AT1G60000	-1.196948246	9.78E-73	Arabidopsis thaliana RNA recognition motif-containing protein mRNA, complete cds
AT2G43100	-1.575622452	1.15E-72	Arabidopsis thaliana chromosome 2, complete sequence
AT1G79560	-1.154947417	1.90E-72	Arabidopsis thaliana AT1G79560 mRNA, complete cds, clone: RAFL07-08-E14
AT2G39530	-2.246102304	2.17E-72	Arabidopsis thaliana uncharacterized protein mRNA, complete cds
AT2G21650	-2.374219529	4.35E-72	Arabidopsis thaliana MYB transcription factor RSM1 mRNA, complete cds
AT3G44990	-1.632461234	5.67E-72	Arabidopsis thaliana xyloglucan endotransglucosylase/hydrolase mRNA, complete cds
AT4G08380	-3.086084854	6.03E-72	Arabidopsis thaliana chromosome 4 sequence
AT1G18250	-1.634304451	6.27E-72	Arabidopsis thaliana Thaumatin-like protein mRNA, complete cds
AT4G02270	-4.046323025	6.93E-72	Arabidopsis thaliana protein root hair specific 13 mRNA, complete cds
AT2G02130	-1.20728328	1.16E-71	Arabidopsis thaliana defensin-like protein 1 mRNA, complete cds
AT1G14980	-1.254183968	1.61E-71	Arabidopsis thaliana chaperonin 10 mRNA, complete cds
AT3G04290	-1.710506062	1.77E-71	Arabidopsis thaliana Li-tolerant lipase 1 mRNA, complete cds
AT4G22010	-1.416974464	2.97E-71	Arabidopsis thaliana protein SKU5 similar 4 mRNA, complete cds
AT1G12080	-1.202114177	6.11E-71	Arabidopsis thaliana vacuolar calcium-binding protein-like protein mRNA, complete cds
AT3G48110	-1.023717425	2.03E-70	Arabidopsis thaliana glycyl-tRNA synthetase 2 mRNA, complete cds
AT4G08620	-3.758543153	3.25E-70	Arabidopsis thaliana sulfate transporter 1.1 mRNA, complete cds
AT4G38520	-1.129487432	5.74E-70	Arabidopsis thaliana putative protein phosphatase 2C 64 mRNA, complete cds
AT3G54600	-1.508294902	6.84E-70	Arabidopsis thaliana class I glutamine amidotransferase-like domain-containing protein mRNA, complete cds
AT5G26880	-1.292895127	1.88E-69	Arabidopsis thaliana protein AGAMOUS-LIKE 26 mRNA, complete cds

AT5G35220	-1.385509142	4.06E-69	Arabidopsis thaliana metalloprotease EGY1 mRNA, complete cds
AT3G57150	-1.084728725	6.13E-69	Arabidopsis thaliana chromosome 3, complete sequence
AT1G12500	-1.381882325	2.33E-68	Arabidopsis thaliana Nucleotide-sugar transporter family protein mRNA, complete cds
AT5G53250	-5.139364937	7.39E-68	Arabidopsis thaliana mRNA for predicted GPI-anchored protein, complete cds, clone: RAFL16-23-F12
AT1G41880	-1.124983653	9.56E-68	Arabidopsis thaliana 60S ribosomal protein L35a-2 mRNA, complete cds
AT3G01440	-1.939018488	2.55E-67	Arabidopsis thaliana PsbQ-like protein 1 mRNA, complete cds
AT3G23730	-2.515738072	3.73E-67	Arabidopsis thaliana xyloglucan endotransglucosylase/hydrolase protein 16 mRNA, complete cds
AT2G14560	-3.90511236	4.50E-67	Arabidopsis thaliana protein LURP1 mRNA, complete cds
AT4G31840	-1.481243577	7.34E-67	Arabidopsis thaliana early nodulin-like protein 15 mRNA, complete cds
AT5G42100	-1.002735774	8.70E-67	Arabidopsis thaliana glucan endo-1,3-beta-glucosidase 10 mRNA, complete cds
AT5G04950	-1.894675797	1.10E-66	Arabidopsis thaliana chromosome 5 sequence
AT4G38160	-1.285055939	2.77E-66	Arabidopsis thaliana protein PIGMENT DEFECTIVE 191 mRNA, complete cds
AT5G65970	-2.153188863	3.65E-66	Arabidopsis thaliana MLO-like protein 10 mRNA, complete cds
AT3G62040	-1.358189959	9.98E-66	Arabidopsis thaliana haloacid dehalogenase-like hydrolase superfamily protein mRNA, complete cds
AT4G39040	-1.007494449	1.28E-65	Arabidopsis thaliana putative RNA-binding protein containing KH domain mRNA, complete cds
AT1G48480	-1.551341206	2.26E-65	Arabidopsis thaliana receptor-like kinase 1 mRNA, complete cds
AT5G61480	-1.229833385	5.48E-65	Arabidopsis thaliana leucine-rich repeat receptor-like protein kinase TDR mRNA, complete cds
AT5G57180	-1.293763871	9.76E-65	Arabidopsis thaliana chloroplast import apparatus 2 protein mRNA, complete cds
AT5G41670	-1.252207211	1.24E-64	Arabidopsis thaliana chromosome 5

			sequence
AT3G07430	-1.359198026	1.34E-64	Arabidopsis thaliana chromosome 3, complete sequence
AT2G04780	-1.045271348	2.72E-64	Arabidopsis thaliana chromosome 2, complete sequence
AT4G21870	-2.31519214	3.86E-64	Arabidopsis thaliana heat shock protein class V 15.4 mRNA, complete cds
AT3G23390	-1.094431559	4.13E-64	Arabidopsis thaliana 60S ribosomal protein L36a mRNA, complete cds
AT2G30930	-1.919316456	1.13E-63	Arabidopsis thaliana uncharacterized protein mRNA, complete cds
AT3G58990	-1.829092309	1.43E-63	Arabidopsis thaliana chromosome 3, complete sequence
AT5G16250	-1.311132001	1.50E-63	Arabidopsis thaliana chromosome 5 sequence
AT3G02850	-2.31528082	1.62E-63	Arabidopsis thaliana potassium channel SKOR mRNA, complete cds
AT5G57625	-3.840156918	1.62E-63	Arabidopsis thaliana CAP (Cysteine-rich secretory proteins, Antigen 5, and Pathogenesis-related 1 protein) superfamily protein mRNA, complete cds
AT1G11545	-2.297792679	1.87E-63	Arabidopsis thaliana probable xyloglucan endotransglucosylase/hydrolase protein 8 mRNA, complete cds
AT2G47910	-1.402537644	1.90E-63	Arabidopsis thaliana chromosome 2, complete sequence
AT5G44680	-1.540940176	1.91E-63	Arabidopsis thaliana putative 3-methyladenine glycosylase I mRNA, complete cds
AT3G26710	-1.052778889	2.00E-63	Arabidopsis thaliana cofactor assembly of complex C mRNA, complete cds
AT4G36180	-1.385607883	2.99E-63	Arabidopsis thaliana putative LRR receptor-like serine/threonine-protein kinase mRNA, complete cds
AT5G65980	-4.014294171	3.34E-63	Arabidopsis thaliana auxin efflux carrier family protein mRNA, complete cds
AT4G38860	-2.068815744	3.35E-63	Arabidopsis thaliana chromosome 4 sequence
AT4G23800	-1.174301054	4.10E-63	Arabidopsis thaliana HMG (high mobility group) box protein mRNA, complete cds
AT1G12040	-5.433007328	4.22E-63	Arabidopsis thaliana chromosome 1 sequence

AT2G29750	-2.133294274	4.84E-63	Arabidopsis thaliana chromosome 2, complete sequence
AT4G34290	-1.526341469	5.09E-63	Arabidopsis thaliana SWIB/MDM2 domain-containing protein mRNA, complete cds
AT3G17170	-1.557349274	1.03E-62	Arabidopsis thaliana protein REGULATOR OF FATTY-ACID COMPOSITION 3 mRNA, complete cds
AT4G25630	-1.74638709	1.07E-62	Arabidopsis thaliana mediator of RNA polymerase II transcription subunit 36a mRNA, complete cds
AT2G17240	-1.197593823	1.53E-62	Arabidopsis thaliana uncharacterized protein mRNA, complete cds
AT2G30010	-2.327580555	1.59E-62	Arabidopsis thaliana protein trichome birefringence-like 45 mRNA, complete cds
AT2G27402	-2.26884985	6.60E-62	Arabidopsis thaliana chromosome 2, complete sequence
AT5G65390	-2.784522219	8.06E-62	Arabidopsis thaliana chromosome 5 sequence
AT1G70880	-3.025887623	5.69E-61	Arabidopsis thaliana SRPBCC domain-containing protein mRNA, complete cds
AT2G02450	-2.049155909	9.87E-61	Arabidopsis thaliana protein LONG VEGETATIVE PHASE 1 mRNA, complete cds
AT2G36570	-1.208577279	1.27E-60	Arabidopsis thaliana PXY/TDR-correlated protein PXC1 mRNA, complete cds
AT5G61130	-1.10037743	1.94E-60	Arabidopsis thaliana plasmodesmata callose-binding protein 1 mRNA, complete cds
AT5G08610	-1.351947246	2.15E-60	Arabidopsis thaliana DEAD-box ATP-dependent RNA helicase 26 mRNA, complete cds
AT4G26370	-1.733603109	4.20E-60	Arabidopsis thaliana antitermination NusB domain-containing protein mRNA, complete cds
AT2G24120	-1.113149488	5.80E-60	Arabidopsis thaliana DNA-directed RNA polymerase 3 mRNA, complete cds
AT3G56480	-1.365462442	5.84E-60	Arabidopsis thaliana myosin heavy chain-like protein mRNA, complete cds
AT3G54040	-1.604274746	6.12E-60	Arabidopsis thaliana PAR1 protein mRNA, complete cds

AT1G12000	-1.073814166	8.96E-60	Arabidopsis thaliana pyrophosphate--fructose-6-phosphate 1-phosphotransferase subunit beta 1 mRNA, complete cds
AT5G64580	-1.226844775	3.67E-59	Arabidopsis thaliana AAA-type ATPase family protein mRNA, complete cds
AT3G27060	-1.279751239	6.42E-59	Arabidopsis thaliana ribonucleoside-diphosphate reductase small chain C mRNA, complete cds
AT1G33140	-1.014413367	7.06E-59	Arabidopsis thaliana 60S ribosomal protein L9-1 mRNA, complete cds
AT2G25060	-1.526600737	1.13E-58	Arabidopsis thaliana early nodulin-like protein 14 mRNA, complete cds
AT4G36540	-1.173386689	1.21E-58	Arabidopsis thaliana transcription factor BEE 2 mRNA, complete cds
AT5G18660	-1.000550787	1.25E-58	Arabidopsis thaliana chromosome 5 sequence
AT3G05730	-1.361217887	2.03E-58	Arabidopsis thaliana defensin-like protein 205 mRNA, complete cds
AT4G18205	-1.668451178	2.94E-58	Arabidopsis thaliana nucleotide-sugar transporter family protein mRNA, complete cds
AT4G00810	-1.210384492	6.67E-58	Arabidopsis thaliana 60S acidic ribosomal protein P1-2 mRNA, complete cds
AT4G29310	-2.971910002	8.01E-58	Arabidopsis thaliana uncharacterized protein mRNA, complete cds
AT3G23890	-1.139291571	8.37E-58	Arabidopsis thaliana DNA topoisomerase 2 mRNA, complete cds
AT2G06950.1	-1.087518979	1.21E-57	Arabidopsis thaliana chromosome 2, complete sequence
AT3G06700	-1.026015155	2.03E-57	Arabidopsis thaliana 60S ribosomal protein L29-1 mRNA, complete cds
AT1G09200	-1.144941191	3.07E-57	Arabidopsis thaliana chromosome 1 sequence
AT5G46900	-2.64371027	5.22E-57	Arabidopsis thaliana chromosome 5 sequence
AT4G34160	-1.311738866	1.75E-56	Arabidopsis thaliana cyclin-D3-1 mRNA, complete cds
AT5G49770	-3.092506076	3.17E-56	Arabidopsis thaliana putative leucine-rich repeat receptor-like protein kinase mRNA, complete cds
AT3G09580	-1.70011691	7.44E-56	Arabidopsis thaliana chromosome 3, complete sequence

AT1G25230	-1.577569098	1.08E-55	Arabidopsis thaliana Calcineurin-like metallo-phosphoesterase superfamily protein mRNA, complete cds
AT5G60660	-1.629719946	1.09E-55	Arabidopsis thaliana putative aquaporin PIP2-4 mRNA, complete cds
AT5G54630	-1.843157408	2.41E-55	Arabidopsis thaliana zinc finger-related protein mRNA, complete cds
AT2G01950	-1.184873308	2.59E-55	Arabidopsis thaliana chromosome 2, complete sequence
AT3G22230	-1.015440836	2.62E-55	Arabidopsis thaliana chromosome 3, complete sequence
AT5G11450	-1.097115971	6.29E-55	Arabidopsis thaliana PsbP domain-containing protein 5 mRNA, complete cds
AT2G40670	-2.201617609	6.61E-55	Arabidopsis thaliana two-component response regulator ARR16 mRNA, complete cds
AT5G45650	-1.428646792	6.94E-55	Arabidopsis thaliana subtilase family protein mRNA, complete cds
AT5G47450	-1.072571575	7.16E-55	Arabidopsis thaliana aquaporin TIP2-3 mRNA, complete cds
AT4G39260	-1.309574161	1.07E-54	Arabidopsis thaliana glycine-rich RNA-binding protein 8 mRNA, complete cds
AT5G44610	-1.829010808	1.34E-54	Arabidopsis thaliana microtubule-associated protein 18 mRNA, complete cds
AT1G01080	-1.122537831	1.75E-54	Arabidopsis thaliana RNA-binding (RRM/RBD/RNP motifs) family protein mRNA, complete cds
AT2G33330	-1.763190453	2.75E-54	Arabidopsis thaliana plasmodesmata-located protein 3 mRNA, complete cds
AT1G69200	-1.573631317	3.17E-54	Arabidopsis thaliana fructokinase-like 2 mRNA, complete cds
AT3G45030	-1.052397656	3.91E-54	Arabidopsis thaliana 40S ribosomal protein S20-1 mRNA, complete cds
AT5G52780	-1.772372585	8.82E-54	Arabidopsis thaliana chromosome 5 sequence
AT2G34710	-1.00103495	9.14E-54	Arabidopsis thaliana homeobox-leucine zipper protein ATHB-14 mRNA, complete cds
AT1G62500	-1.599656292	1.20E-53	Arabidopsis thaliana chromosome 1 sequence

AT1G20840	-1.228547666	1.23E-53	Arabidopsis thaliana tonoplast monosaccharide transporter1 mRNA, complete cds
AT5G59850	-1.103468926	3.21E-53	Arabidopsis thaliana 40S ribosomal protein S15a-1 mRNA, complete cds
AT4G17870	-1.083408581	3.49E-53	Arabidopsis thaliana chromosome 4 sequence
AT2G23130	-1.878016907	4.43E-53	Arabidopsis thaliana Lysine-rich arabinogalactan protein 17 mRNA, complete cds
AT5G15350	-1.184816438	4.46E-53	Arabidopsis thaliana early nodulin-like protein 17 mRNA, complete cds
AT4G08300	-1.661251897	5.58E-53	Arabidopsis thaliana nodulin MtN21 /EamA-like transporter family protein mRNA, complete cds
AT4G30110	-1.712020973	6.27E-53	Arabidopsis thaliana cadmium/zinc-transporting ATPase HMA2 mRNA, complete cds
AT4G30670	-3.569509328	1.15E-52	Arabidopsis thaliana chromosome 4 sequence
AT5G11480	-1.03445635	3.26E-52	Arabidopsis thaliana P-loop containing nucleoside triphosphate hydrolases superfamily protein mRNA, complete cds
AT3G43810	-1.072453917	3.91E-52	Arabidopsis thaliana calmodulin 7 mRNA, complete cds
AT5G56860	-1.195679133	4.71E-52	Arabidopsis thaliana GATA transcription factor 21 mRNA, complete cds
AT1G48630	-1.141380133	6.17E-52	Arabidopsis thaliana receptor for activated C kinase 1B mRNA, complete cds
AT2G21790	-1.040220425	1.19E-51	Arabidopsis thaliana ribonucleoside-diphosphate reductase large subunit mRNA, complete cds
AT2G22122	-2.661767836	1.51E-51	Arabidopsis thaliana chromosome 2, complete sequence
AT1G74030	-1.339521823	4.16E-51	Arabidopsis thaliana enolase 1 mRNA, complete cds
AT5G60670	-1.375296955	4.69E-51	Arabidopsis thaliana chromosome 5 sequence
AT2G20450	-1.074901605	5.40E-51	Arabidopsis thaliana 60S ribosomal protein L14-1 mRNA, complete cds
AT1G57680	-1.098762396	6.25E-51	Arabidopsis thaliana uncharacterized protein mRNA, complete cds

AT3G29030	-1.28385662	1.46E-50	Arabidopsis thaliana expansin A5 mRNA, complete cds
AT2G18980	-3.679133819	2.83E-50	Arabidopsis thaliana peroxidase 16 mRNA, complete cds
AT3G15030	-1.483976514	4.25E-50	Arabidopsis thaliana transcription factor TCP4 mRNA, complete cds
AT3G18110	-1.464327092	8.04E-50	Arabidopsis thaliana pentatricopeptide repeat-containing protein mRNA, complete cds
AT3G12930	-1.371146976	9.87E-50	Arabidopsis thaliana Lojap-related protein mRNA, complete cds
AT3G08680	-1.085899498	1.16E-49	Arabidopsis thaliana leucine-rich repeat protein kinase family protein mRNA, complete cds
AT3G02640	-1.398322678	1.29E-49	Arabidopsis thaliana chromosome 3, complete sequence
AT1G75780	-1.790536208	3.22E-49	Arabidopsis thaliana tubulin beta-1 chain mRNA, complete cds
AT3G25930	-2.120683516	3.24E-49	Arabidopsis thaliana Adenine nucleotide alpha hydrolases-like superfamily protein mRNA, complete cds
AT1G80480	-1.071537248	4.74E-49	Arabidopsis thaliana protein plastid transcriptionally active 17 mRNA, complete cds
AT1G11350	-1.61538969	6.01E-49	Arabidopsis thaliana G-type lectin S-receptor-like serine/threonine-protein kinase SD1-13 mRNA, complete cds
AT3G06880	-1.315245132	6.15E-49	Arabidopsis thaliana transducin/WD-40 repeat-containing protein mRNA, complete cds
AT5G44130	-1.392557682	1.25E-48	Arabidopsis thaliana chromosome 5 sequence
AT3G16780	-1.075694716	1.33E-48	Arabidopsis thaliana 60S ribosomal protein L19-2 mRNA, complete cds
AT3G22235	-2.994815163	1.79E-48	Arabidopsis thaliana uncharacterized protein mRNA, complete cds
AT3G47860	-1.326299901	1.89E-48	Arabidopsis thaliana chloroplastic lipocalin mRNA, complete cds
AT5G66770	-2.031413433	2.24E-48	Arabidopsis thaliana chromosome 5 sequence
AT1G14210	-1.722391846	2.42E-48	Arabidopsis thaliana ribonuclease T2 family protein mRNA, complete cds
AT4G02680	-1.021710117	2.48E-48	Arabidopsis thaliana ETO1-like protein 1 mRNA, complete cds

AT5G08260	-1.253860296	3.26E-48	Arabidopsis thaliana serine carboxypeptidase-like 35 mRNA, complete cds
AT5G06630	-5.050636018	3.35E-48	Arabidopsis thaliana chromosome 5 sequence
AT4G38840	-1.762154406	3.51E-48	Arabidopsis thaliana chromosome 4 sequence
AT1G53730	-1.138281376	3.63E-48	Arabidopsis thaliana STRUBBELIG-receptor family 6 mRNA, complete cds
AT3G10690	-1.258258026	4.28E-48	Arabidopsis thaliana DNA gyrase subunit A mRNA, complete cds
AT1G65370	-1.316328823	5.04E-48	Arabidopsis thaliana meprin and TRAF homology domain-containing protein mRNA, complete cds
AT1G54950	-6.021636146	5.66E-48	Arabidopsis thaliana uncharacterized protein mRNA, complete cds
AT4G33270	-1.158501194	5.99E-48	Arabidopsis thaliana cell division cycle 20.1, cofactor of APC complex mRNA, complete cds
AT3G07010	-1.465761403	7.19E-48	Arabidopsis thaliana putative pectate lyase 8 mRNA, complete cds
AT5G15210	-1.896010153	8.10E-48	Arabidopsis thaliana chromosome 5 sequence
AT3G46280	-1.63555972	9.71E-48	Arabidopsis thaliana protein kinase-like protein mRNA, complete cds
AT3G43690.1	-1.728707923	1.22E-47	Arabidopsis thaliana chromosome 3, complete sequence
AT1G66940	-1.765732817	1.61E-47	Arabidopsis thaliana protein kinase-related protein mRNA, complete cds
AT5G06640	-6.613903448	2.14E-47	Arabidopsis thaliana chromosome 5 sequence
AT5G32450	-1.164314447	6.66E-47	Arabidopsis thaliana RNA recognition motif (RRM)-containing protein mRNA, complete cds
AT3G16250	-1.006205709	9.20E-47	Arabidopsis thaliana NDH-dependent cyclic electron flow 1 mRNA, complete cds
AT2G40330	-5.006914641	1.03E-46	Arabidopsis thaliana chromosome 2, complete sequence
AT1G01300	-1.011559779	2.48E-46	Arabidopsis thaliana chromosome 1 sequence
AT1G55330	-2.42266794	3.58E-46	Arabidopsis thaliana chromosome 1

			sequence
AT5G40480	-1.144394756	5.38E-46	Arabidopsis thaliana protein EMBRYO DEFECTIVE 3012 mRNA, complete cds
AT4G18760	-2.340027482	6.39E-46	Arabidopsis thaliana chromosome 4 sequence
AT5G27390	-1.265661688	6.99E-46	Arabidopsis thaliana Mog1/PsbP/DUF1795-like photosystem II reaction center PsbP family protein mRNA, complete cds
AT5G63180	-1.416458145	8.09E-46	Arabidopsis thaliana putative pectate lyase 22 mRNA, complete cds
AT4G34220	-1.072859763	1.14E-45	Arabidopsis thaliana leucine-rich repeat protein kinase family protein mRNA, complete cds
AT4G22505	-2.435208237	2.25E-45	Arabidopsis thaliana chromosome 4 sequence
AT2G25840	-1.037495708	3.00E-45	Arabidopsis thaliana protein ovule abortion 4 mRNA, complete cds
AT5G15830	-2.921353001	4.30E-45	Arabidopsis thaliana chromosome 5 sequence
AT3G45160	-1.163546653	5.13E-45	Arabidopsis thaliana putative membrane lipoprotein mRNA, complete cds
AT3G22790	-1.084692391	1.76E-44	Arabidopsis thaliana protein NETWORKED 1A mRNA, complete cds
AT5G60710	-1.22319514	2.69E-44	Arabidopsis thaliana C3H4 type zinc finger protein mRNA, complete cds
AT1G63100	-1.473219314	4.46E-44	Arabidopsis thaliana chromosome 1 sequence
AT5G27120	-1.020105694	5.09E-44	Arabidopsis thaliana putative SAR DNA-binding protein mRNA, complete cds
AT2G36885	-1.343364021	6.98E-44	Arabidopsis thaliana uncharacterized protein mRNA, complete cds
AT5G61440	-1.810801955	7.63E-44	Arabidopsis thaliana atypical CYS HIS rich thioredoxin 5 mRNA, complete cds
AT1G05200	-1.013271117	8.10E-44	Arabidopsis thaliana glutamate receptor 3.4 mRNA, complete cds
AT3G50530	-1.033548728	9.87E-44	Arabidopsis thaliana CDPK-related kinase mRNA, complete cds
AT2G19760	-1.038576288	2.12E-43	Arabidopsis thaliana profilin 1 mRNA, complete cds
AT3G09210	-1.135674234	2.81E-43	Arabidopsis thaliana plastid transcriptionally active 13 mRNA, complete cds

AT5G19600	-1.801382803	2.85E-43	Arabidopsis thaliana sulfate transporter 3;5 mRNA, complete cds
AT4G08770	-1.345060321	2.97E-43	Arabidopsis thaliana peroxidase 37 mRNA, complete cds
AT1G49200	-1.806209586	3.05E-43	Arabidopsis thaliana chromosome 1 sequence
AT4G31210	-1.253875563	3.18E-43	Arabidopsis thaliana DNA topoisomerase, type IA, core mRNA, complete cds
AT3G20015	-2.361267395	4.63E-43	Arabidopsis thaliana chromosome 3, complete sequence
AT2G39140	-1.427065063	5.99E-43	Arabidopsis thaliana protein SUPPRESSOR OF VARIEGATION 1 mRNA, complete cds
AT3G07540	-1.690815625	6.50E-43	Arabidopsis thaliana Actin-binding FH2 (formin homology 2) family protein mRNA, complete cds
AT1G67750	-1.196597305	6.81E-43	Arabidopsis thaliana putative pectate lyase 5 mRNA, complete cds
AT5G56840	-2.923146283	1.08E-42	Arabidopsis thaliana myb-like transcription factor family protein mRNA, complete cds
AT1G60680	-2.310907169	1.63E-42	Arabidopsis thaliana probable aldo-keto reductase 2 mRNA, complete cds
AT4G24660	-1.887998339	3.05E-42	Arabidopsis thaliana chromosome 4 sequence
AT3G22800	-2.343233472	3.53E-42	Arabidopsis thaliana chromosome 3, complete sequence
AT1G77690	-1.38632823	4.73E-42	Arabidopsis thaliana auxin influx carrier LAX3 mRNA, complete cds
AT5G49160	-1.115414353	6.48E-42	Arabidopsis thaliana DNA (cytosine-5)-methyltransferase 1 mRNA, complete cds
AT2G35500	-1.083003851	6.83E-42	Arabidopsis thaliana shikimate kinase like 2 mRNA, complete cds
AT3G44750	-1.321037217	6.88E-42	Arabidopsis thaliana histone deacetylase HDT1 mRNA, complete cds
AT4G37750	-1.321142972	1.32E-41	Arabidopsis thaliana AP2-like ethylene-responsive transcription factor ANT mRNA, complete cds
AT2G40610	-1.805137577	1.43E-41	Arabidopsis thaliana expansin A8 mRNA, complete cds
AT2G39870	-1.054790289	1.43E-41	Arabidopsis thaliana uncharacterized protein mRNA, complete cds

AT1G22330	-2.738063598	1.45E-41	Arabidopsis thaliana RNA recognition motif-containing protein mRNA, complete cds
AT3G51330	-1.413568556	1.81E-41	Arabidopsis thaliana aspartyl protease family protein mRNA, complete cds
AT3G20380	-2.54424257	2.59E-41	Arabidopsis thaliana TRAF-like family protein mRNA, complete cds
AT4G34760	-1.482046487	3.12E-41	Arabidopsis thaliana chromosome 4 sequence
AT4G04570	-1.288327576	3.89E-41	Arabidopsis thaliana cysteine-rich receptor-like protein kinase 40 mRNA, complete cds
AT2G02070	-1.311711571	4.71E-41	Arabidopsis thaliana indeterminate(ID)-domain 5 protein mRNA, complete cds
AT1G63570	-3.242344585	5.78E-41	Arabidopsis thaliana chromosome 1 sequence
AT1G59990	-1.365740872	6.70E-41	Arabidopsis thaliana DEAD-box ATP-dependent RNA helicase 22 mRNA, complete cds
AT4G22666	-4.620135401	8.17E-41	Arabidopsis thaliana Bifunctional inhibitor/lipid-transfer protein/seed storage 2S albumin superfamily protein mRNA, complete cds
AT4G33260	-1.369836983	1.03E-40	Arabidopsis thaliana cell division cycle 20.2, cofactor of APC complex mRNA, complete cds
AT2G28605	-1.308579511	1.11E-40	Arabidopsis thaliana photosystem II reaction center PsbP family protein mRNA, complete cds
AT1G09660	-1.336245001	1.72E-40	Arabidopsis thaliana RNA-binding KH domain-containing protein mRNA, complete cds
AT4G23820	-1.135986258	1.76E-40	Arabidopsis thaliana glycoside hydrolase family 28 protein / polygalacturonase (pectinase) family protein mRNA, complete cds
AT5G22940	-1.862556303	1.83E-40	Arabidopsis thaliana probable glucuronoxylan glucuronosyltransferase F8H mRNA, complete cds
AT2G02100	-1.221586025	1.95E-40	Arabidopsis thaliana defensin-like protein 2 mRNA, complete cds
AT5G43360	-2.960299281	2.04E-40	Arabidopsis thaliana putative inorganic phosphate transporter 1-3 mRNA,

			complete cds
AT5G48580	-1.278146199	2.22E-40	Arabidopsis thaliana peptidyl-prolyl cis-trans isomerase FKBP15-2 mRNA, complete cds
AT1G30250	-1.080060115	2.72E-40	Arabidopsis thaliana chromosome 1 sequence
AT1G48210	-1.225029706	4.27E-40	Arabidopsis thaliana serine/threonine protein kinase mRNA, complete cds
AT2G19310	-1.17927522	6.32E-40	Arabidopsis thaliana chromosome 2, complete sequence
AT5G39210	-2.074078049	6.54E-40	Arabidopsis thaliana protein CHLORORESPIRATORY REDUCTION 7 mRNA, complete cds
AT5G07240	-1.278680437	9.53E-40	Arabidopsis thaliana IQ-domain 24 protein mRNA, complete cds
AT4G29080	-1.118355868	9.73E-40	Arabidopsis thaliana phytochrome-associated protein 2 mRNA, complete cds
AT3G47800	-1.118355868	9.73E-40	Arabidopsis thaliana putative aldose 1-epimerase mRNA, complete cds
AT4G02330	-1.575222979	1.09E-39	Arabidopsis thaliana Probable pectinesterase/pectinesterase inhibitor 41 mRNA, complete cds
AT3G18450	-4.410811582	1.36E-39	Arabidopsis thaliana PLAC8 family protein mRNA, complete cds
AT3G51720	-1.032943652	1.73E-39	Arabidopsis thaliana uncharacterized protein mRNA, complete cds
AT5G18570	-1.112988378	2.12E-39	Arabidopsis thaliana OBGC-like protein mRNA, complete cds
AT4G32980	-1.332470131	2.48E-39	Arabidopsis thaliana homeobox protein ATH1 mRNA, complete cds
AT5G53880	-1.894236057	2.58E-39	Arabidopsis thaliana chromosome 5 sequence
AT1G33811	-1.615023988	2.71E-39	Arabidopsis thaliana GDSL esterase/lipase mRNA, complete cds
AT5G39080	-1.000267286	3.34E-39	Arabidopsis thaliana chromosome 5 sequence
AT4G21250	-3.108874963	3.64E-39	Arabidopsis thaliana Sulfite exporter TauE/SafE family protein mRNA, complete cds
AT1G74070	-1.00650608	9.08E-39	Arabidopsis thaliana cyclophilin-like peptidyl-prolyl cis-trans isomerase family protein mRNA, complete cds
AT4G02540	-1.356932348	2.05E-38	Arabidopsis thaliana chromosome 4

			sequence
AT5G51545	-1.240201703	2.16E-38	Arabidopsis thaliana low psii accumulation2 protein mRNA, complete cds
AT3G47350	-1.086223528	2.28E-38	Arabidopsis thaliana hydroxysteroid dehydrogenase 2 mRNA, complete cds
AT4G19660	-1.300936739	2.65E-38	Arabidopsis thaliana NPR1-like protein 4 mRNA, complete cds
AT4G38660	-1.159573794	2.65E-38	Arabidopsis thaliana pathogenesis-related thaumatin-like protein mRNA, complete cds
AT4G16390	-1.250940246	3.78E-38	Arabidopsis thaliana chromosome 4 sequence
AT3G28920	-1.228150703	3.84E-38	Arabidopsis thaliana chromosome 3, complete sequence
AT5G48360	-1.081454858	6.75E-38	Arabidopsis thaliana actin-binding FH2 (formin homology 2) family protein mRNA, complete cds
AT3G63110	-2.014294171	7.30E-38	Arabidopsis thaliana chromosome 3, complete sequence
AT4G25220	-4.517041908	8.87E-38	Arabidopsis thaliana putative glycerol-3-phosphate transporter 2 mRNA, complete cds
AT3G54090	-1.34269511	1.80E-37	Arabidopsis thaliana fructokinase-like 1 mRNA, complete cds
AT1G56050	-1.187363711	2.04E-37	Arabidopsis thaliana putative GTP-binding protein mRNA, complete cds
AT1G28670	-1.39873343	2.96E-37	Arabidopsis thaliana lipase ARAB-1 mRNA, complete cds
AT5G50460	-1.454866762	3.20E-37	Arabidopsis thaliana protein transport protein sec61 subunit gamma-2 mRNA, complete cds
AT2G47930	-1.903410065	3.26E-37	Arabidopsis thaliana chromosome 2, complete sequence
AT2G42610	-1.156862376	4.82E-37	Arabidopsis thaliana uncharacterized protein mRNA, complete cds
AT2G28960	-2.21672027	6.18E-37	Arabidopsis thaliana putative LRR receptor-like serine/threonine-protein kinase mRNA, complete cds
AT3G18130	-1.290608517	6.56E-37	Arabidopsis thaliana receptor for activated C kinase 1C mRNA, complete cds
AT5G44660	-1.22747366	7.08E-37	Arabidopsis thaliana uncharacterized

			protein mRNA, complete cds
AT4G28780	-1.213171203	7.73E-37	Arabidopsis thaliana GDSL esterase/lipase mRNA, complete cds
AT2G15090	-1.560407121	8.44E-37	Arabidopsis thaliana chromosome 2, complete sequence
AT2G36170	-1.019465244	8.91E-37	Arabidopsis thaliana 60S ribosomal protein L40-1 mRNA, complete cds
AT2G17230	-1.955567761	1.09E-36	Arabidopsis thaliana chromosome 2, complete sequence
AT3G59980	-1.311197333	1.38E-36	Arabidopsis thaliana Nucleic acid-binding, OB-fold-like protein mRNA, complete cds
AT2G28850	-7.279167576	1.46E-36	Arabidopsis thaliana chromosome 2, complete sequence
AT1G80050	-1.780818251	1.85E-36	Arabidopsis thaliana adenine phosphoribosyl transferase 2 mRNA, complete cds
AT2G02680	-2.466609421	1.95E-36	Arabidopsis thaliana cysteine/histidine-rich C1 domain-containing protein mRNA, complete cds
AT5G25090	-2.048677165	2.11E-36	Arabidopsis thaliana early nodulin-like protein 13 mRNA, complete cds
AT1G18370	-1.343949628	2.44E-36	Arabidopsis thaliana kinesin HINKEL mRNA, complete cds
AT3G20540	-1.110409784	2.52E-36	Arabidopsis thaliana polymerase gamma 1 mRNA, complete cds
AT2G28900	-1.089149039	2.55E-36	Arabidopsis thaliana outer plastid envelope protein 16-1 mRNA, complete cds
AT4G19690	-2.006640598	3.24E-36	Arabidopsis thaliana Fe(2+) transport protein 1 mRNA, complete cds
AT2G31150	-1.293671575	3.80E-36	Arabidopsis thaliana ATP binding / ATPase mRNA, complete cds
AT2G24710	-3.808975263	4.14E-36	Arabidopsis thaliana glutamate receptor 2.3 mRNA, complete cds
AT3G16080	-1.040269302	4.98E-36	Arabidopsis thaliana 60S ribosomal protein L37-3 mRNA, complete cds
AT5G17630	-1.107731591	7.68E-36	Arabidopsis thaliana chromosome 5 sequence
AT5G46240	-1.513731871	7.75E-36	Arabidopsis thaliana potassium channel KAT1 mRNA, complete cds
AT3G51280	-1.670287098	1.35E-35	Arabidopsis thaliana tetratricopeptide repeat domain-containing protein mRNA,

			complete cds
AT4G32570	-1.003655651	1.38E-35	Arabidopsis thaliana protein TIFY 8 mRNA, complete cds
AT3G59040	-1.08382393	2.75E-35	Arabidopsis thaliana pentatricopeptide repeat-containing protein mRNA, complete cds
AT3G50700	-1.659146541	2.83E-35	Arabidopsis thaliana indeterminate-domain 2 protein mRNA, complete cds
AT1G57820	-1.131804919	3.09E-35	Arabidopsis thaliana E3 ubiquitin-protein ligase ORTHRUS 2 mRNA, complete cds
AT1G23480	-1.559986554	3.12E-35	Arabidopsis thaliana putative mannan synthase 3 mRNA, complete cds
AT4G39675	-3.528867344	3.34E-35	Arabidopsis thaliana chromosome 4 sequence
AT2G39890	-1.113355975	3.38E-35	Arabidopsis thaliana proline transporter 1 mRNA, complete cds
AT5G10580	-1.75998657	4.18E-35	Arabidopsis thaliana uncharacterized protein mRNA, complete cds
AT2G33210	-1.064515132	4.26E-35	Arabidopsis thaliana heat shock protein 60-2 mRNA, complete cds
AT1G11540	-2.728055268	4.49E-35	Arabidopsis thaliana Sulfite exporter TauE/SafE family protein mRNA, complete cds
AT5G55280	-1.019756705	6.34E-35	Arabidopsis thaliana cell division protein ftsZ-like protein 1 mRNA, complete cds
AT1G04520	-1.305236026	1.21E-34	Arabidopsis thaliana plasmodesmata-located protein 2 mRNA, complete cds
AT4G25820	-2.068419385	1.53E-34	Arabidopsis thaliana xyloglucan endotransglucosylase/hydrolase protein 14 mRNA, complete cds
AT3G44430	-1.187039691	1.78E-34	Arabidopsis thaliana chromosome 3, complete sequence
AT3G12110	-1.492150446	1.99E-34	Arabidopsis thaliana actin-11 mRNA, complete cds
AT4G28210	-1.425527594	2.10E-34	Arabidopsis thaliana embryo defective 1923 protein mRNA, complete cds
AT1G18590	-1.372817417	2.31E-34	Arabidopsis thaliana chromosome 1 sequence
AT5G60520	-3.427490323	3.00E-34	Arabidopsis thaliana late embryogenesis abundant protein-like protein mRNA, complete cds

AT1G76090	-1.36905946	5.53E-34	Arabidopsis thaliana chromosome 1 sequence
AT1G49630	-1.07016361	6.10E-34	Arabidopsis thaliana presequence protease 2 mRNA, complete cds
AT3G14390	-1.019228667	8.62E-34	Arabidopsis thaliana diaminopimelate decarboxylase 1 mRNA, complete cds
AT1G13420	-2.474872454	1.01E-33	Arabidopsis thaliana chromosome 1 sequence
AT1G10682	-1.077907376	1.07E-33	Arabidopsis thaliana chromosome 1 sequence
AT2G22230	-1.068759831	1.15E-33	Arabidopsis thaliana putative 3-hydroxyacyl-ACP dehydratase mRNA, complete cds
AT1G34210	-1.142088105	1.28E-33	Arabidopsis thaliana somatic embryogenesis receptor kinase 2 mRNA, complete cds
AT2G34510	-1.301358977	2.02E-33	Arabidopsis thaliana uncharacterized protein mRNA, complete cds
AT5G45670	-1.015948927	2.15E-33	Arabidopsis thaliana GDSL esterase/lipase mRNA, complete cds
AT1G14720	-1.031379992	2.47E-33	Arabidopsis thaliana probable xyloglucan endotransglucosylase/hydrolase protein 28 mRNA, complete cds
AT2G23930	-1.086567765	2.61E-33	Arabidopsis thaliana probable small nuclear ribonucleoprotein G mRNA, complete cds
AT2G31890	-1.115215779	2.66E-33	Arabidopsis thaliana protein RAP mRNA, complete cds
AT2G48080	-3.212352745	3.27E-33	Arabidopsis thaliana oxidoreductase, 2OG-Fe(II) oxygenase family protein mRNA, complete cds
AT2G37460	-1.48916722	4.38E-33	Arabidopsis thaliana nodulin MtN21-like transporter family protein mRNA, complete cds
AT2G27510	-1.302588	4.50E-33	Arabidopsis thaliana ferredoxin 3 mRNA, complete cds
AT5G13840	-1.201605805	4.99E-33	Arabidopsis thaliana protein FIZZY-related 3 mRNA, complete cds
AT5G23210	-1.298725423	5.51E-33	Arabidopsis thaliana serine carboxypeptidase-like 34 mRNA, complete cds
AT1G50490	-1.195570435	7.06E-33	Arabidopsis thaliana ubiquitin-conjugating enzyme E2 20 mRNA, complete cds

AT4G33360	-1.347116907	1.01E-32	Arabidopsis thaliana Rossmann-fold NAD(P)-binding domain-containing protein mRNA, complete cds
AT1G58370	-1.747631483	1.98E-32	Arabidopsis thaliana xylanase 1 mRNA, complete cds
AT3G13560	-1.335733864	2.23E-32	Arabidopsis thaliana glucan endo-1,3-beta-glucosidase 4 mRNA, complete cds
AT3G20150	-1.31601569	3.85E-32	Arabidopsis thaliana Kinesin motor family protein mRNA, complete cds
AT2G16850	-1.250621688	6.92E-32	Arabidopsis thaliana putative aquaporin PIP2-8 mRNA, complete cds
AT4G33720	-1.176533592	8.37E-32	Arabidopsis thaliana chromosome 4 sequence
AT3G51350	-2.324775739	1.03E-31	Arabidopsis thaliana aspartyl protease family protein mRNA, complete cds
AT4G12510	-8.174371386	1.12E-31	Arabidopsis thaliana chromosome 4 sequence
AT5G51560	-1.178200901	3.03E-31	Arabidopsis thaliana leucine-rich repeat protein kinase mRNA, complete cds
AT1G07090	-2.259553875	3.44E-31	Arabidopsis thaliana chromosome 1 sequence
AT1G12330	-1.456298718	3.45E-31	Arabidopsis thaliana uncharacterized protein mRNA, complete cds
AT5G63550	-1.272280003	3.49E-31	Arabidopsis thaliana DEK domain-containing chromatin associated protein mRNA, complete cds
AT3G54830	-1.59598154	3.70E-31	Arabidopsis thaliana transmembrane amino acid transporter family protein mRNA, complete cds
AT5G17160	-1.337218446	5.84E-31	Arabidopsis thaliana uncharacterized protein mRNA, complete cds
AT2G22170	-1.075873688	6.88E-31	Arabidopsis thaliana PLAT-plant-stress domain-containing protein mRNA, complete cds
AT1G70090	-1.197985965	7.55E-31	Arabidopsis thaliana chromosome 1 sequence
AT3G61310	-1.164492632	9.57E-31	Arabidopsis thaliana AT hook motif DNA-binding family protein mRNA, complete cds
AT2G42380	-1.926443873	9.93E-31	Arabidopsis thaliana transcription factor BZIP34 mRNA, complete cds
AT1G18650	-1.191561148	1.07E-30	Arabidopsis thaliana plasmodesmata callose-binding protein 3 mRNA,

			complete cds
AT5G23420	-1.417826158	1.14E-30	Arabidopsis thaliana high-mobility group B6 protein mRNA, complete cds
AT4G12830	-1.079325052	1.15E-30	Arabidopsis thaliana hydrolase, alpha/beta fold family protein mRNA, complete cds
AT2G26820	-2.598364191	1.21E-30	Arabidopsis thaliana phloem protein 2-LIKE A3 mRNA, complete cds
AT4G13170	-1.09574939	1.30E-30	Arabidopsis thaliana 60S ribosomal protein L13a-3 mRNA, complete cds
AT1G05700	-3.236933989	1.43E-30	Arabidopsis thaliana Leucine-rich repeat transmembrane protein kinase protein mRNA, complete cds
AT3G59370	-2.471328934	1.50E-30	Arabidopsis thaliana vacuolar calcium-binding protein-like protein mRNA, complete cds
AT2G47840	-1.064834617	1.92E-30	Arabidopsis thaliana chromosome 2, complete sequence
AT1G29020	-4.42195214	2.25E-30	Arabidopsis thaliana EF-hand, calcium binding motif-containing protein mRNA, complete cds
AT4G00970	-1.200275519	2.73E-30	Arabidopsis thaliana cysteine-rich receptor-like protein kinase 41 mRNA, complete cds
AT4G12310	-1.342847312	3.34E-30	Arabidopsis thaliana cytochrome P450, family 706, subfamily A, polypeptide 5 mRNA, complete cds
AT4G37520	-1.100753969	3.55E-30	Arabidopsis thaliana peroxidase 50 mRNA, complete cds
AT2G18328	-1.979310179	4.90E-30	Arabidopsis thaliana chromosome 2, complete sequence
AT3G54560	-1.363058451	5.15E-30	Arabidopsis thaliana histone H2A 11 mRNA, complete cds
AT1G24280	-1.472060988	6.73E-30	Arabidopsis thaliana glucose-6-phosphate dehydrogenase 3 mRNA, complete cds
AT5G67510	-1.169193335	7.76E-30	Arabidopsis thaliana chromosome 5 sequence
AT3G49500	-1.007816607	9.33E-30	Arabidopsis thaliana RNA-dependent RNA polymerase 6 mRNA, complete cds
AT3G06770	-1.700138771	9.37E-30	Arabidopsis thaliana polygalacturonase-like protein mRNA, complete cds
AT5G24020	-1.235830228	1.16E-29	Arabidopsis thaliana chromosome 5

			sequence
AT4G20940	-1.415181182	1.70E-29	Arabidopsis thaliana leucine-rich receptor-like protein kinase mRNA, complete cds
AT1G60600	-1.065630756	1.88E-29	Arabidopsis thaliana 1,4-dihydroxy-2-naphthoate polyprenyltransferase mRNA, complete cds
AT2G33550	-1.597758805	2.30E-29	Arabidopsis thaliana Myb/SANT-like DNA-binding domain-containing protein mRNA, complete cds
AT1G64380	-1.368151819	2.53E-29	Arabidopsis thaliana chromosome 1 sequence
AT2G41940	-1.393011673	4.33E-29	Arabidopsis thaliana chromosome 2, complete sequence
AT1G78450	-2.729505212	4.80E-29	Arabidopsis thaliana SOUL/heme-binding protein-like protein mRNA, complete cds
AT5G65810	-1.189804902	5.16E-29	Arabidopsis thaliana protein COTTON GOLGI-RELATED 3 mRNA, complete cds
AT4G07820	-2.151444136	5.39E-29	Arabidopsis thaliana chromosome 4 sequence
AT5G23310	-1.178286653	6.23E-29	Arabidopsis thaliana Fe superoxide dismutase 3 mRNA, complete cds
AT4G37040	-1.054936155	8.51E-29	Arabidopsis thaliana methionine aminopeptidase 1D mRNA, complete cds
AT4G05190	-1.235953231	1.12E-28	Arabidopsis thaliana kinesin 5 mRNA, complete cds
AT3G46550	-1.212352745	1.26E-28	Arabidopsis thaliana chromosome 3, complete sequence
AT4G20440	-1.119196354	1.35E-28	Arabidopsis thaliana chromosome 4 sequence
AT5G60930	-1.123010867	1.60E-28	Arabidopsis thaliana P-loop containing nucleoside triphosphate hydrolases superfamily protein mRNA, complete cds
AT2G29180	-1.295971636	1.64E-28	Arabidopsis thaliana uncharacterized protein mRNA, complete cds
AT3G13610	-1.370306534	1.79E-28	Arabidopsis thaliana feruloyl CoA ortho-hydroxylase 1 mRNA, complete cds
AT3G10610	-1.216110895	2.36E-28	Arabidopsis thaliana 40S ribosomal protein S17-3 mRNA, complete cds

AT1G78260	-1.697516941	2.85E-28	Arabidopsis thaliana RNA recognition motif-containing protein mRNA, complete cds
AT3G27950	-3.213365518	2.94E-28	Arabidopsis thaliana GDSL esterase/lipase mRNA, complete cds
AT5G40510	-2.044418284	3.89E-28	Arabidopsis thaliana Sucrase/ferredoxin-like family protein mRNA, complete cds
AT3G02885	-1.87838127	3.99E-28	Arabidopsis thaliana gibberellin-regulated protein 5 mRNA, complete cds
AT3G47450	-1.082111606	4.74E-28	Arabidopsis thaliana NO-associated protein 1 mRNA, complete cds
AT1G27460	-1.435926174	5.14E-28	Arabidopsis thaliana calmodulin-binding protein mRNA, complete cds
AT1G47670	-1.123523241	5.85E-28	Arabidopsis thaliana Lysine histidine transporter-like 8 mRNA, complete cds
AT1G10020	-1.450384334	5.99E-28	Arabidopsis thaliana uncharacterized protein mRNA, complete cds
AT5G19090	-1.013947744	6.40E-28	Arabidopsis thaliana heavy metal transport/detoxification domain-containing protein mRNA, complete cds
AT4G30140	-1.774828236	7.64E-28	Arabidopsis thaliana GDSL esterase/lipase mRNA, complete cds
AT2G25680	-1.458890156	8.91E-28	Arabidopsis thaliana chromosome 2, complete sequence
AT3G22142	-2.177191906	9.00E-28	Arabidopsis thaliana chromosome 3, complete sequence
AT2G36120	-1.973231514	9.19E-28	Arabidopsis thaliana chromosome 2, complete sequence
AT1G24170	-1.090548181	1.56E-27	Arabidopsis thaliana chromosome 1 sequence
AT4G02800	-1.314512242	1.90E-27	Arabidopsis thaliana uncharacterized protein mRNA, complete cds
AT5G40830	-1.399584327	2.15E-27	Arabidopsis thaliana S-adenosyl-L-methionine-dependent methyltransferases superfamily protein mRNA, complete cds
AT2G02950	-1.658970956	2.96E-27	Arabidopsis thaliana chromosome 2, complete sequence
AT5G46280	-1.382139192	4.29E-27	Arabidopsis thaliana DNA replication licensing factor MCM3-like protein mRNA, complete cds

AT2G01530	-1.258580232	4.78E-27	Arabidopsis thaliana MLP-like protein 329 mRNA, complete cds
AT4G16450	-1.072009669	4.98E-27	Arabidopsis thaliana uncharacterized protein mRNA, complete cds
AT3G46490	-2.594171383	5.18E-27	Arabidopsis thaliana oxidoreductase, 2OG-Fe(II) oxygenase family protein mRNA, complete cds
AT1G64450	-2.399584327	7.86E-27	Arabidopsis thaliana chromosome 1 sequence
AT5G62900	-1.352055957	1.13E-26	Arabidopsis thaliana uncharacterized protein mRNA, complete cds
AT5G59670	-1.400802305	1.32E-26	Arabidopsis thaliana Leucine-rich repeat protein kinase family protein mRNA, complete cds
AT5G10030	-1.238536094	1.43E-26	Arabidopsis thaliana transcription factor TGA4 mRNA, complete cds
AT4G25790	-5.141051313	1.47E-26	Arabidopsis thaliana allergen V5/Tpx-1-related family protein mRNA, complete cds
AT3G12900	-4.469973655	1.60E-26	Arabidopsis thaliana oxidoreductase, 2OG-Fe(II) oxygenase family protein mRNA, complete cds
AT5G62550	-1.257565322	1.76E-26	Arabidopsis thaliana uncharacterized protein mRNA, complete cds
AT1G11080	-1.038115509	1.77E-26	Arabidopsis thaliana serine carboxypeptidase-like 31 mRNA, complete cds
AT2G43360	-1.020616082	1.91E-26	Arabidopsis thaliana biotin synthase mRNA, complete cds
AT4G19430	-2.484392715	2.33E-26	Arabidopsis thaliana chromosome 4 sequence
AT4G02990	-1.164933768	2.93E-26	Arabidopsis thaliana mitochondrial transcription termination factor-like protein BELAYA SMERT mRNA, complete cds
AT2G03340	-1.101730367	4.23E-26	Arabidopsis thaliana WRKY DNA-binding protein 3 mRNA, complete cds
AT3G23175	-1.811010573	5.12E-26	Arabidopsis thaliana HR-like lesion-inducing protein-like protein mRNA, complete cds
AT2G24762	-1.523573044	5.29E-26	Arabidopsis thaliana chromosome 2, complete sequence
AT5G63470	-1.021541035	7.21E-26	Arabidopsis thaliana nuclear

			transcription factor Y subunit C-4 mRNA, complete cds
AT5G04960	-3.254007503	7.29E-26	Arabidopsis thaliana putative pectinesterase/pectinesterase inhibitor 46 mRNA, complete cds
AT1G56430	-1.13624807	7.65E-26	Arabidopsis thaliana chromosome 1 sequence
AT1G70820	-1.1034879	8.15E-26	Arabidopsis thaliana phosphoglucomutase-like protein mRNA, complete cds
AT4G30620	-1.090286081	8.94E-26	Arabidopsis thaliana uncharacterized protein mRNA, complete cds
AT1G15000	-1.467537355	1.05E-25	Arabidopsis thaliana chromosome 1 sequence
AT5G20160	-1.108874963	1.59E-25	Arabidopsis thaliana ribosomal protein L7Ae/L30e/S12e/Gadd45 family protein mRNA, complete cds
AT5G42320	-1.202912184	1.89E-25	Arabidopsis thaliana Zn-dependent exopeptidases superfamily protein mRNA, complete cds
AT4G24350	-1.148836294	2.40E-25	Arabidopsis thaliana phosphorylase family protein mRNA, complete cds
AT4G29740	-2.024075192	3.03E-25	Arabidopsis thaliana cytokinin dehydrogenase 4 mRNA, complete cds
AT4G33000	-1.212108352	3.49E-25	Arabidopsis thaliana calcineurin B-like protein 10 mRNA, complete cds
AT3G53900	-1.115016759	3.66E-25	Arabidopsis thaliana uracil phosphoribosyltransferase mRNA, complete cds
AT5G11270	-1.041617013	4.12E-25	Arabidopsis thaliana overexpressor of cationic peroxidase 3 mRNA, complete cds
AT1G14030	-1.039182084	5.55E-25	Arabidopsis thaliana [fructose-bisphosphate aldolase]-lysine N-methyltransferase mRNA, complete cds
AT1G26240	-3.37686425	5.67E-25	Arabidopsis thaliana chromosome 1 sequence
AT4G22212	-1.40883664	6.55E-25	Arabidopsis thaliana defensin-like protein 98 mRNA, complete cds
AT4G32915	-1.162574809	7.42E-25	Arabidopsis thaliana uncharacterized protein mRNA, complete cds
AT5G54040	-3.684986546	7.93E-25	Arabidopsis thaliana chromosome 5 sequence

AT1G09830	-1.096875528	1.24E-24	Arabidopsis thaliana phosphoribosylamine--glycine ligase mRNA, complete cds
AT5G05500	-3.061362425	1.47E-24	Arabidopsis thaliana chromosome 5 sequence
AT5G23480	-2.39136382	1.65E-24	Arabidopsis thaliana SWIB/MDM2, Plus-3 and GYF domain-containing protein mRNA, complete cds
AT2G22610	-1.356964693	2.11E-24	Arabidopsis thaliana Di-glucose binding protein with Kinesin motor domain mRNA, complete cds
AT1G49570	-1.137193838	2.28E-24	Arabidopsis thaliana peroxidase mRNA, complete cds
AT5G22580	-1.250280383	2.41E-24	Arabidopsis thaliana stress responsive A/B Barrel domain-containing protein mRNA, complete cds
AT4G36570	-2.410811582	2.50E-24	Arabidopsis thaliana protein RAD-like 3 mRNA, complete cds
AT5G67100	-1.091880872	2.71E-24	Arabidopsis thaliana DNA polymerase alpha catalytic subunit mRNA, complete cds
AT1G49410	-1.287109598	3.13E-24	Arabidopsis thaliana translocase of the outer mitochondrial membrane 6 mRNA, complete cds
AT1G30870	-2.095293893	3.32E-24	Arabidopsis thaliana peroxidase 7 mRNA, complete cds
AT2G36255	-3.432005805	3.70E-24	Arabidopsis thaliana putative defensin-like protein 203 mRNA, complete cds
AT1G14440	-1.524280074	3.73E-24	Arabidopsis thaliana homeobox protein 31 mRNA, complete cds
AT4G35380	-1.643239717	4.13E-24	Arabidopsis thaliana SEC7-like guanine nucleotide exchange family protein mRNA, complete cds
AT3G58700	-1.066076713	4.37E-24	Arabidopsis thaliana 60S ribosomal protein L11-2 mRNA, complete cds
AT2G20780	-1.319616022	6.58E-24	Arabidopsis thaliana putative polyol transporter 4 mRNA, complete cds
AT1G77630	-1.438637103	8.20E-24	Arabidopsis thaliana LysM domain-containing GPI-anchored protein 3 mRNA, complete cds
AT5G48460	-1.17584548	9.73E-24	Arabidopsis thaliana Actin binding Calponin homology (CH) domain-containing protein mRNA,

			complete cds
AT1G51860	-1.819324445	1.11E-23	Arabidopsis thaliana putative LRR receptor-like serine/threonine-protein kinase mRNA, complete cds
AT3G49960	-5.523705639	1.55E-23	Arabidopsis thaliana peroxidase 35 mRNA, complete cds
AT1G16390	-3.90511236	1.69E-23	Arabidopsis thaliana chromosome 1 sequence
AT2G27400	-2.405389949	1.76E-23	Arabidopsis thaliana chromosome 2, complete sequence
AT4G00950	-1.762989058	1.89E-23	Arabidopsis thaliana uncharacterized protein mRNA, complete cds
AT4G26150	-1.419641979	1.94E-23	Arabidopsis thaliana putative GATA transcription factor 22 mRNA, complete cds
AT3G18900	-2.194000193	1.99E-23	Arabidopsis thaliana uncharacterized protein mRNA, complete cds
AT3G17185	-1.212528931	2.00E-23	Arabidopsis thaliana chromosome 3, complete sequence
AT1G34047	-4.58445967	2.01E-23	Arabidopsis thaliana defensin-like protein 208 mRNA, complete cds
AT3G23190	-1.683615814	2.07E-23	Arabidopsis thaliana HR-like lesion-inducing protein-like protein mRNA, complete cds
AT1G75240	-1.043659921	2.13E-23	Arabidopsis thaliana homeobox protein 33 mRNA, complete cds
AT5G05440	-1.281004235	2.72E-23	Arabidopsis thaliana chromosome 5 sequence
AT1G09740	-1.004584581	2.87E-23	Arabidopsis thaliana adenine nucleotide alpha hydrolases-like protein mRNA, complete cds
AT3G29250	-1.816669655	2.88E-23	Arabidopsis thaliana short-chain dehydrogenase reductase 4 mRNA, complete cds
AT5G49080.1	-6.523705639	3.10E-23	Arabidopsis thaliana chromosome 5 sequence
AT4G00400	-1.012610746	3.26E-23	Arabidopsis thaliana bifunctional sn-glycerol-3-phosphate 2-O-acyltransferase/phosphatase mRNA, complete cds
AT1G49975	-1.007306731	3.54E-23	Arabidopsis thaliana uncharacterized protein mRNA, complete cds
AT5G58480	-1.376347618	4.09E-23	Arabidopsis thaliana glucan endo-1,3-beta-glucosidase 9 mRNA,

			complete cds
AT1G29530	-1.084488265	4.10E-23	Arabidopsis thaliana uncharacterized protein mRNA, complete cds
AT1G29250	-1.185195129	4.83E-23	Arabidopsis thaliana Alba DNA/RNA-binding protein mRNA, complete cds
AT5G06690	-1.45646939	5.10E-23	Arabidopsis thaliana WCRKC thioredoxin 1 mRNA, complete cds
AT1G50055	-2.359586259	5.43E-23	Arabidopsis thaliana chromosome 1 sequence
AT2G32270	-1.977594067	5.46E-23	Arabidopsis thaliana zinc transporter 3 mRNA, complete cds
AT4G05330	-1.61787235	5.49E-23	Arabidopsis thaliana putative ADP-ribosylation factor GTPase-activating protein AGD13 mRNA, complete cds
AT1G44050	-4.277328577	5.99E-23	Arabidopsis thaliana cysteine/histidine-rich C1-like domain-containing protein mRNA, complete cds
AT5G35740	-1.374780936	6.81E-23	Arabidopsis thaliana carbohydrate-binding X8 domain-containing protein mRNA, complete cds
AT1G07370	-1.618418929	9.20E-23	Arabidopsis thaliana proliferating cellular nuclear antigen 1 mRNA, complete cds
AT5G61000	-1.554402436	1.14E-22	Arabidopsis thaliana replication protein A 70 kDa DNA-binding subunit D mRNA, complete cds
AT2G33180	-1.087424008	1.30E-22	Arabidopsis thaliana uncharacterized protein mRNA, complete cds
AT2G19870	-1.17016692	1.30E-22	Arabidopsis thaliana chromosome 2, complete sequence
AT3G02120	-1.675119516	1.35E-22	Arabidopsis thaliana hydroxyproline-rich glycoprotein-like protein mRNA, complete cds
AT1G13300	-1.254204176	1.37E-22	Arabidopsis thaliana protein HRS1 mRNA, complete cds
AT2G03350	-1.203500165	1.44E-22	Arabidopsis thaliana uncharacterized protein mRNA, complete cds
AT5G05180	-1.682138183	1.49E-22	Arabidopsis thaliana uncharacterized protein mRNA, complete cds
AT1G33840	-3.345294171	2.12E-22	Arabidopsis thaliana uncharacterized

			protein mRNA, complete cds
AT2G27840	-1.289173909	2.16E-22	Arabidopsis thaliana histone deacetylase HDT4 mRNA, complete cds
AT4G02060	-1.104577531	2.29E-22	Arabidopsis thaliana protein PROLIFERA mRNA, complete cds
AT1G07610	-1.203441805	3.23E-22	Arabidopsis thaliana metallothionein 1C mRNA, complete cds
AT1G30840	-1.936106473	3.38E-22	Arabidopsis thaliana chromosome 1 sequence
AT3G15570	-1.464290016	3.55E-22	Arabidopsis thaliana phototropic-responsive NPH3 family protein mRNA, complete cds
AT3G21300	-1.331349591	3.64E-22	Arabidopsis thaliana RNA methyltransferase family protein mRNA, complete cds
AT2G33790	-1.249819706	3.73E-22	Arabidopsis thaliana arabinogalactan protein 30 mRNA, complete cds
AT5G23830	-1.304483625	3.75E-22	Arabidopsis thaliana MD-2-related lipid recognition domain-containing protein mRNA, complete cds
AT4G25090	-2.690261488	4.59E-22	Arabidopsis thaliana riboflavin synthase-like superfamily protein mRNA, complete cds
AT5G58310	-1.068727915	4.99E-22	Arabidopsis thaliana methyl esterase 18 mRNA, complete cds
AT2G36970	-2.158576227	5.97E-22	Arabidopsis thaliana UDP-glycosyltransferase 86A1 mRNA, complete cds
AT2G15292	-1.247581233	6.14E-22	Arabidopsis thaliana chromosome 2, complete sequence
AT4G37070	-1.286846116	6.86E-22	Arabidopsis thaliana patatin-related phospholipase A mRNA, complete cds
AT3G45930	-1.524185338	7.44E-22	Arabidopsis thaliana chromosome 3, complete sequence
AT5G52010	-1.130466366	7.63E-22	Arabidopsis thaliana chromosome 5 sequence
AT1G69900	-2.679692246	7.88E-22	Arabidopsis thaliana Actin cross-linking protein mRNA, complete cds
AT1G64640	-1.333995985	8.13E-22	Arabidopsis thaliana early nodulin-like protein 8 mRNA, complete cds
AT1G28110	-1.059314596	8.92E-22	Arabidopsis thaliana serine carboxypeptidase-like 45 mRNA, complete cds
AT4G25990	-2.192945977	9.15E-22	Arabidopsis thaliana CIA2-like

			transcription factor mRNA, complete cds
AT4G01037	-1.006241899	1.12E-21	Arabidopsis thaliana RNA recognition domain-containing protein WTF1 mRNA, complete cds
AT2G27970	-1.877631624	1.17E-21	Arabidopsis thaliana cyclin-dependent kinases regulatory subunit 2 mRNA, complete cds
AT5G42750	-1.078058714	1.20E-21	Arabidopsis thaliana chromosome 5 sequence
AT3G06730	-1.183725634	1.23E-21	Arabidopsis thaliana thioredoxin-like protein CITRX mRNA, complete cds
AT4G11120	-1.269704044	1.24E-21	Arabidopsis thaliana putative translation elongation factor Ts mRNA, complete cds
AT3G19680	-1.2789192	1.38E-21	Arabidopsis thaliana uncharacterized protein mRNA, complete cds
AT3G43800	-1.147777176	1.48E-21	Arabidopsis thaliana glutathione S-transferase tau 27 mRNA, complete cds
AT4G15160	-1.999813584	1.60E-21	Arabidopsis thaliana bifunctional inhibitor/lipid-transfer protein/seed storage 2S albumin-like protein mRNA, complete cds
AT3G08600	-1.591964179	2.01E-21	Arabidopsis thaliana chromosome 3, complete sequence
AT1G18970	-3.047556625	2.05E-21	Arabidopsis thaliana chromosome 1 sequence
AT5G03995	-4.186180689	2.06E-21	Arabidopsis thaliana uncharacterized protein mRNA, complete cds
AT5G46790	-1.3729492	2.24E-21	Arabidopsis thaliana chromosome 5 sequence
AT3G30875	-1.350869042	2.26E-21	Arabidopsis thaliana chromosome 3, complete sequence
AT5G62340	-1.948020952	2.36E-21	Arabidopsis thaliana chromosome 5 sequence
AT4G34750	-1.829824233	2.43E-21	Arabidopsis thaliana chromosome 4 sequence
AT2G33860	-1.003764743	2.46E-21	Arabidopsis thaliana auxin response transcription factor 3 mRNA, complete cds
AT3G54910	-1.388937083	2.73E-21	Arabidopsis thaliana leucine rich repeat and F-Box domain-containing protein mRNA, complete cds
AT3G23290	-1.054936155	3.73E-21	Arabidopsis thaliana protein LIGHT

			SENSITIVE HYPOCOTYLS 4 mRNA, complete cds
AT5G02050	-1.089040995	3.97E-21	Arabidopsis thaliana mitochondrial glycoprotein family protein mRNA, complete cds
AT1G47840	-1.339641786	4.04E-21	Arabidopsis thaliana hexokinase 3 mRNA, complete cds
AT3G27180	-1.217019255	4.14E-21	Arabidopsis thaliana S-adenosylmethionine-dependent methyltransferase-domain containing protein mRNA, complete cds
AT4G26320	-2.780406111	6.25E-21	Arabidopsis thaliana chromosome 4 sequence
AT1G36390	-1.013229582	7.55E-21	Arabidopsis thaliana Co-chaperone GrpE family protein mRNA, complete cds
AT3G25717	-1.17927522	8.66E-21	Arabidopsis thaliana chromosome 3, complete sequence
AT2G07690	-1.016575682	1.10E-20	Arabidopsis thaliana minichromosome maintenance protein 5 mRNA, complete cds
AT3G24140	-1.1186835	1.17E-20	Arabidopsis thaliana transcription factor FAMA mRNA, complete cds
AT2G48120	-1.026194525	1.23E-20	Arabidopsis thaliana protein PALE CRESS mRNA, complete cds
AT1G49470	-1.226621615	1.25E-20	Arabidopsis thaliana chromosome 1 sequence
AT4G20210	-2.234773457	1.50E-20	Arabidopsis thaliana terpenoid synthase 8 mRNA, complete cds
AT5G04230	-1.847043304	1.84E-20	Arabidopsis thaliana phenylalanine ammonia-lyase 3 mRNA, complete cds
AT2G18910	-1.145637172	1.98E-20	Arabidopsis thaliana hydroxyproline-rich glycoprotein-like protein mRNA, complete cds
AT4G04330	-1.638371186	2.00E-20	Arabidopsis thaliana Chaperonin-like RbcX protein mRNA, complete cds
AT2G16380	-1.061852554	2.01E-20	Arabidopsis thaliana Sec14p-like phosphatidylinositol transfer family protein mRNA, complete cds
AT2G28780	-1.033456428	2.18E-20	Arabidopsis thaliana uncharacterized protein mRNA, complete cds
AT5G60490	-1.816992731	2.55E-20	Arabidopsis thaliana chromosome 5 sequence
AT1G49650	-1.186936224	2.66E-20	Arabidopsis thaliana chromosome 1 sequence

AT5G54610	-1.351085809	2.69E-20	Arabidopsis thaliana ankyrin-repeat transmembrane protein BDA1 mRNA, complete cds
AT1G13100	-2.017713692	2.86E-20	Arabidopsis thaliana cytochrome P450 71B29 mRNA, complete cds
AT2G24720	-3.443978446	2.91E-20	Arabidopsis thaliana glutamate receptor 2.2 mRNA, complete cds
AT4G26670	-1.302096482	3.17E-20	Arabidopsis thaliana mitochondrial import inner membrane translocase subunit TIM22-2 mRNA, complete cds
AT5G26670	-1.391511026	3.24E-20	Arabidopsis thaliana pectinacetylesterase family protein mRNA, complete cds
AT3G46320	-1.723730247	3.35E-20	Arabidopsis thaliana chromosome 3, complete sequence
AT4G25240	-1.380475504	3.53E-20	Arabidopsis thaliana monocopper oxidase-like protein SKS1 mRNA, complete cds
AT3G11402	-3.716350717	3.55E-20	Arabidopsis thaliana cysteine/histidine-rich C1 domain-containing protein mRNA, complete cds
AT4G13493	-1.809868296	3.96E-20	Arabidopsis thaliana chromosome 4 sequence
AT1G52700	-2.145538704	4.49E-20	Arabidopsis thaliana alpha/beta-Hydrolases superfamily protein mRNA, complete cds
AT1G58561.1	-1.593655416	4.50E-20	Arabidopsis thaliana chromosome 1 sequence
AT3G50010	-2.910546246	4.58E-20	Arabidopsis thaliana chromosome 3, complete sequence
AT3G60330	-2.044847338	4.68E-20	Arabidopsis thaliana H(+)-ATPase 7 mRNA, complete cds
AT3G51340	-1.717066517	5.14E-20	Arabidopsis thaliana aspartyl protease family protein mRNA, complete cds
AT3G23800	-1.449215095	5.42E-20	Arabidopsis thaliana selenium-binding protein 3 mRNA, complete cds
AT5G48470	-1.072009669	5.73E-20	Arabidopsis thaliana uncharacterized protein mRNA, complete cds
AT3G10525	-1.140975987	6.12E-20	Arabidopsis thaliana chromosome 3, complete sequence
AT5G44040	-1.227403351	6.32E-20	Arabidopsis thaliana uncharacterized protein mRNA, complete cds
AT2G39900	-1.163870527	7.22E-20	Arabidopsis thaliana protein WLIM2A mRNA, complete cds

AT1G48570	-1.268339794	7.35E-20	Arabidopsis thaliana zinc finger (Ran-binding) domain-containing protein mRNA, complete cds
AT2G02740	-1.626842503	7.69E-20	Arabidopsis thaliana single-stranded DNA-binding protein WHY3 mRNA, complete cds
AT5G24410	-1.84908341	7.91E-20	Arabidopsis thaliana 6-phosphogluconolactonase 4 mRNA, complete cds
AT1G51940	-1.098879503	9.05E-20	Arabidopsis thaliana LysM type receptor kinase-like protein mRNA, complete cds
AT3G20370	-1.202970347	9.41E-20	Arabidopsis thaliana TRAF-like family protein mRNA, complete cds
AT2G19060	-2.830036879	1.04E-19	Arabidopsis thaliana SGNH hydrolase-type esterase family protein mRNA, complete cds
AT5G65410	-1.095440419	1.24E-19	Arabidopsis thaliana chromosome 5 sequence
AT4G24120	-1.022184485	1.63E-19	Arabidopsis thaliana metal-nicotianamine transporter YSL1 mRNA, complete cds
AT1G31690	-1.389530662	1.74E-19	Arabidopsis thaliana copper amine oxidase family protein mRNA, complete cds
AT3G50560	-1.415409294	2.76E-19	Arabidopsis thaliana Rossmann-fold NAD(P)-binding domain-containing protein mRNA, complete cds
AT3G13760	-1.707706622	3.39E-19	Arabidopsis thaliana chromosome 3, complete sequence
AT4G30220	-1.237312898	3.65E-19	Arabidopsis thaliana small nuclear ribonucleoprotein F mRNA, complete cds
AT2G40750	-2.306474922	3.72E-19	Arabidopsis thaliana WRKY DNA-binding protein 54 mRNA, complete cds
AT3G12750	-1.517670397	3.72E-19	Arabidopsis thaliana zinc transporter 1 mRNA, complete cds
AT1G12560	-2.433007328	4.28E-19	Arabidopsis thaliana alpha-expansin family protein mRNA, complete cds
AT3G55010	-1.041261219	4.49E-19	Arabidopsis thaliana phosphoribosylformylglycinamidine cyclo-ligase mRNA, complete cds
AT4G12917	-1.818868797	4.72E-19	Arabidopsis thaliana Full-length cDNA Complete sequence from clone GSLTSIL58ZA07 of Silique of strain

			col-0 of Arabidopsis thaliana (thale cress)
AT4G26780	-1.360055963	4.94E-19	Arabidopsis thaliana molecular chaperone GrpE mRNA, complete cds
AT4G31360	-2.100024045	5.09E-19	Arabidopsis thaliana selenium binding protein mRNA, complete cds
AT5G53500	-1.030559225	5.64E-19	Arabidopsis thaliana transducin/WD40 domain-containing protein mRNA, complete cds
AT3G25890	-1.003242921	5.73E-19	Arabidopsis thaliana chromosome 3, complete sequence
AT1G04980	-1.177766113	5.86E-19	Arabidopsis thaliana protein disulfide-isomerase like 2-2 mRNA, complete cds
AT3G05625	-1.227117131	6.16E-19	Arabidopsis thaliana tetratricopeptide repeat-containing protein mRNA, complete cds
AT4G25890	-1.236293162	7.02E-19	Arabidopsis thaliana 60S acidic ribosomal protein P3-1 mRNA, complete cds
AT4G12360	-6.174371386	9.09E-19	Arabidopsis thaliana Bifunctional inhibitor/lipid-transfer protein/seed storage 2S albumin superfamily protein mRNA, complete cds
AT4G19030	-1.387394918	9.30E-19	Arabidopsis thaliana aquaporin NIP1-1 mRNA, complete cds
AT1G79670	-1.038738928	9.96E-19	Arabidopsis thaliana wall-associated receptor kinase-like 22 mRNA, complete cds
AT3G54750	-1.042603929	1.03E-18	Arabidopsis thaliana uncharacterized protein mRNA, complete cds
AT5G56120	-1.216992914	1.03E-18	Arabidopsis thaliana uncharacterized protein mRNA, complete cds
AT3G55420	-1.071134513	1.07E-18	Arabidopsis thaliana chromosome 3, complete sequence
AT1G63220	-1.339293548	1.10E-18	Arabidopsis thaliana calcium-dependent lipid-binding domain-containing protein mRNA, complete cds
AT3G17160	-1.084594004	1.15E-18	Arabidopsis thaliana uncharacterized protein mRNA, complete cds
AT4G28850	-5.174371386	1.19E-18	Arabidopsis thaliana xyloglucan endotransglucosylase/hydrolase 26 mRNA, complete cds
AT1G50240	-1.03541091	1.34E-18	Arabidopsis thaliana serine/threonine-protein kinase FUSED

			mRNA, complete cds
AT2G46890	-1.120524497	1.79E-18	Arabidopsis thaliana uncharacterized protein mRNA, complete cds
AT5G51600	-1.090898809	1.92E-18	Arabidopsis thaliana microtubule associated protein MAP65-3 mRNA, complete cds
AT5G44480	-1.353780637	2.29E-18	Arabidopsis thaliana putative UDP-arabinose 4-epimerase 4 mRNA, complete cds
AT4G35050	-1.202639111	2.42E-18	Arabidopsis thaliana WD-40 repeat-containing protein MSI3 mRNA, complete cds
AT1G56680	-3.217207584	2.52E-18	Arabidopsis thaliana Chitinase family protein mRNA, complete cds
AT2G28740	-1.056337511	2.59E-18	Arabidopsis thaliana chromosome 2, complete sequence
AT2G28930	-1.260671233	2.76E-18	Arabidopsis thaliana protein kinase APK1B mRNA, complete cds
AT5G62730	-4.609037692	2.78E-18	Arabidopsis thaliana probable peptide/nitrate transporter mRNA, complete cds
AT1G14410	-1.010898155	2.79E-18	Arabidopsis thaliana single-stranded DNA-binding protein WHY1 mRNA, complete cds
AT1G51800	-1.204053858	2.84E-18	Arabidopsis thaliana putative leucine-rich repeat protein kinase mRNA, complete cds
AT5G37450	-2.235090289	2.95E-18	Arabidopsis thaliana probable LRR receptor-like serine/threonine-protein kinase mRNA, complete cds
AT1G21560	-1.060974775	3.15E-18	Arabidopsis thaliana uncharacterized protein mRNA, complete cds
AT1G78865	-1.520599728	3.34E-18	Arabidopsis thaliana chromosome 1 sequence
AT5G63960	-1.245744575	3.45E-18	Arabidopsis thaliana DNA polymerase delta subunit 1 mRNA, complete cds
AT3G02620	-2.284941761	4.34E-18	Arabidopsis thaliana acyl-[acyl-carrier-protein] desaturase mRNA, complete cds
AT5G26320	-6.113829845	4.41E-18	Arabidopsis thaliana TRAF-like family protein mRNA, complete cds
AT2G44740	-2.254007503	4.62E-18	Arabidopsis thaliana cyclin p4;1 mRNA, complete cds
AT5G01870	-1.497359543	5.73E-18	Arabidopsis thaliana pathogenesis-related

			lipid transfer protein mRNA, complete cds
AT2G33400	-1.303963703	7.03E-18	Arabidopsis thaliana uncharacterized protein mRNA, complete cds
AT2G04790	-1.246641673	7.77E-18	Arabidopsis thaliana uncharacterized protein mRNA, complete cds
AT3G11370	-4.213365518	8.36E-18	Arabidopsis thaliana chromosome 3, complete sequence
AT1G62980	-2.999497169	8.91E-18	Arabidopsis thaliana expansin A18 mRNA, complete cds
AT2G16650	-1.20099611	1.05E-17	Arabidopsis thaliana proteinaceous RNase P 2 mRNA, complete cds
AT1G08580	-1.193723629	1.06E-17	Arabidopsis thaliana uncharacterized protein mRNA, complete cds
AT3G26200	-1.659707137	1.10E-17	Arabidopsis thaliana cytochrome P450 71B22 mRNA, complete cds
AT2G19210	-3.410811582	1.11E-17	Arabidopsis thaliana putative leucine-rich repeat receptor-like protein kinase mRNA, complete cds
AT2G44380	-1.556273501	1.15E-17	Arabidopsis thaliana chromosome 2, complete sequence
AT1G10990	-2.391722209	1.44E-17	Arabidopsis thaliana chromosome 1 sequence
AT3G06300	-1.069209225	1.44E-17	Arabidopsis thaliana prolyl-4 hydroxylase 2 mRNA, complete cds
AT5G42680	-1.745359164	1.47E-17	Arabidopsis thaliana uncharacterized protein mRNA, complete cds
AT1G78460	-1.61257805	1.68E-17	Arabidopsis thaliana SOUL heme-binding protein mRNA, complete cds
AT4G03100	-1.205423016	1.69E-17	Arabidopsis thaliana Rho GTPase activating protein with PAK-box/P21-Rho-binding domain mRNA, complete cds
AT1G49870	-1.388269014	1.85E-17	Arabidopsis thaliana uncharacterized protein mRNA, complete cds
AT4G26660	-1.094171319	1.90E-17	Arabidopsis thaliana uncharacterized protein mRNA, complete cds
AT5G07670	-1.060897714	1.90E-17	Arabidopsis thaliana RNI-like superfamily protein mRNA, complete cds
AT5G35190	-5.072009669	2.08E-17	Arabidopsis thaliana chromosome 5 sequence
AT1G34510	-5.072009669	2.08E-17	Arabidopsis thaliana peroxidase 8

			mRNA, complete cds
AT5G66750	-1.193434985	2.39E-17	Arabidopsis thaliana ATP-dependent DNA helicase DDM1 mRNA, complete cds
AT1G05385	-1.095420045	2.64E-17	Arabidopsis thaliana photosystem II D1 precursor processing protein PSB27-H2 mRNA, complete cds
AT3G60440	-1.100688422	2.69E-17	Arabidopsis thaliana phosphoglycerate mutase family protein mRNA, complete cds
AT1G28660	-1.873843363	2.86E-17	Arabidopsis thaliana GDSL esterase/lipase mRNA, complete cds
AT5G66230	-1.080762143	2.93E-17	Arabidopsis thaliana Chalcone-flavanone isomerase family protein mRNA, complete cds
AT2G28990	-2.272791796	3.06E-17	Arabidopsis thaliana putative LRR receptor-like serine/threonine-protein kinase mRNA, complete cds
AT5G27300	-1.057952769	3.59E-17	Arabidopsis thaliana pentatricopeptide (PPR) repeat-containing protein mRNA, complete cds
AT4G35880	-1.091462031	3.61E-17	Arabidopsis thaliana aspartyl protease family protein mRNA, complete cds
AT1G51870	-5.050636018	3.69E-17	Arabidopsis thaliana putative serine/threonine protein kinase mRNA, complete cds
AT5G48830	-1.028490175	3.98E-17	Arabidopsis thaliana uncharacterized protein mRNA, complete cds
AT2G47230	-1.393755248	4.10E-17	Arabidopsis thaliana uncharacterized protein mRNA, complete cds
AT1G51100	-1.108221198	4.47E-17	Arabidopsis thaliana chromosome 1 sequence
AT4G15830	-1.351917893	4.54E-17	Arabidopsis thaliana ARM repeat superfamily protein mRNA, complete cds
AT3G59890	-1.583793554	5.15E-17	Arabidopsis thaliana dihydrodipicolinate reductase 2 mRNA, complete cds
AT3G28080	-1.024878922	5.35E-17	Arabidopsis thaliana MtN21-like transporter family protein UMAMIT47 mRNA, complete cds
AT3G50270	-1.127151223	6.43E-17	Arabidopsis thaliana chromosome 3, complete sequence
AT3G46940	-1.535721015	6.45E-17	Arabidopsis thaliana chromosome 3, complete sequence

AT2G01755	-1.443978446	6.65E-17	Arabidopsis thaliana chromosome 2, complete sequence
AT3G20810	-1.692891744	6.92E-17	Arabidopsis thaliana jumonji-C domain-containing protein 30 mRNA, complete cds
AT1G44110	-1.361913909	7.03E-17	Arabidopsis thaliana cyclin-A1-1 mRNA, complete cds
AT4G34740	-1.041880003	7.28E-17	Arabidopsis thaliana chromosome 4 sequence
AT1G60060	-2.318447564	7.32E-17	Arabidopsis thaliana serine/threonine-protein kinase WNK (With No lysine)-related protein mRNA, complete cds
AT5G51350	-1.069554853	8.69E-17	Arabidopsis thaliana receptor-like kinase MOL1 mRNA, complete cds
AT2G46440	-1.117813358	1.03E-16	Arabidopsis thaliana cyclic nucleotide-gated channel 11 mRNA, complete cds
AT3G44590	-1.017798633	1.14E-16	Arabidopsis thaliana 60S acidic ribosomal protein P2-4 mRNA, complete cds
AT4G36410	-1.69504021	1.16E-16	Arabidopsis thaliana putative ubiquitin-conjugating enzyme E2 17 mRNA, complete cds
AT1G47210	-1.071010918	1.43E-16	Arabidopsis thaliana cyclin-dependent protein kinase 3;2 mRNA, complete cds
AT1G60050	-3.852443292	1.54E-16	Arabidopsis thaliana Nodulin MtN21 /EamA-like transporter family protein mRNA, complete cds
AT1G21210	-2.148933645	1.54E-16	Arabidopsis thaliana wall-associated receptor kinase 4 mRNA, complete cds
AT1G77990	-1.338183782	1.57E-16	Arabidopsis thaliana sulfate transporter 2;2 mRNA, complete cds
AT3G14900	-1.484812164	1.90E-16	Arabidopsis thaliana chromosome 3, complete sequence
AT4G28410	-1.541358288	2.06E-16	Arabidopsis thaliana tyrosine transaminase family protein mRNA, complete cds
AT3G47980	-1.317746035	2.06E-16	Arabidopsis thaliana HPP integral membrane domain-containing protein mRNA, complete cds
AT3G62270	-1.548135678	2.32E-16	Arabidopsis thaliana putative boron transporter 2 mRNA, complete cds
AT4G08410	-7.006914641	2.40E-16	Arabidopsis thaliana chromosome 4

			sequence
AT2G20520	-7.006914641	2.40E-16	Arabidopsis thaliana chromosome 2, complete sequence
AT5G43020	-1.002925357	2.63E-16	Arabidopsis thaliana leucine-rich repeat protein kinase family protein mRNA, complete cds
AT2G37600	-1.058547409	3.02E-16	Arabidopsis thaliana 60S ribosomal protein L36-1 mRNA, complete cds
AT2G01610	-1.338830296	3.02E-16	Arabidopsis thaliana chromosome 2, complete sequence
AT5G14090	-1.439112691	3.13E-16	Arabidopsis thaliana uncharacterized protein mRNA, complete cds
AT2G29320	-1.342098832	3.50E-16	Arabidopsis thaliana tropinone reductase-like protein mRNA, complete cds
AT2G40690	-1.342098832	3.50E-16	Arabidopsis thaliana glycerol-3-phosphate dehydrogenase [NAD(+)] 2 mRNA, complete cds
AT4G24265	-1.908597974	3.53E-16	Arabidopsis thaliana chromosome 4 sequence
AT1G10522	-1.049764487	3.97E-16	Arabidopsis thaliana protein PRIN2 mRNA, complete cds
AT2G41950	-1.471328934	4.14E-16	Arabidopsis thaliana uncharacterized protein mRNA, complete cds
AT1G14080	-2.074744637	4.18E-16	Arabidopsis thaliana fucosyltransferase 6 mRNA, complete cds
AT5G03360	-2.891437423	4.50E-16	Arabidopsis thaliana C1 domain-containing protein mRNA, complete cds
AT2G17710	-1.05051009	5.54E-16	Arabidopsis thaliana uncharacterized protein mRNA, complete cds
AT1G23410	-1.652925336	5.72E-16	Arabidopsis thaliana chromosome 1 sequence
AT1G12320	-1.266211052	5.93E-16	Arabidopsis thaliana uncharacterized protein mRNA, complete cds
AT1G16630	-1.20355661	8.40E-16	Arabidopsis thaliana chromosome 1 sequence
AT5G36120	-1.38080712	8.94E-16	Arabidopsis thaliana chromosome 5 sequence
AT1G56120	-1.704438908	9.18E-16	Arabidopsis thaliana Leucine-rich repeat transmembrane protein kinase mRNA, complete cds
AT3G27970	-4.050636018	9.52E-16	Arabidopsis thaliana exonuclease-like protein mRNA, complete cds

AT4G37160	-1.984546828	1.05E-15	Arabidopsis thaliana protein SKU5 similar 15 mRNA, complete cds
AT2G23620	-2.028940947	1.08E-15	Arabidopsis thaliana methyl esterase 1 mRNA, complete cds
AT1G73620	-1.49912	1.32E-15	Arabidopsis thaliana pathogenesis-related thaumatin-like protein mRNA, complete cds
AT2G24700	-1.267853628	1.34E-15	Arabidopsis thaliana transcriptional factor B3 family protein mRNA, complete cds
AT5G14450	-1.247581233	1.44E-15	Arabidopsis thaliana GDSL esterase/lipase mRNA, complete cds
AT5G49870	-2.64751184	1.55E-15	Arabidopsis thaliana Mannose-binding lectin superfamily protein mRNA, complete cds
AT1G80870	-1.034015504	1.56E-15	Arabidopsis thaliana chromosome 1 sequence
AT1G72416	-1.718565138	1.68E-15	Arabidopsis thaliana chaperone DnaJ-domain containing protein mRNA, complete cds
AT1G61080	-4.37686425	1.80E-15	Arabidopsis thaliana hydroxyproline-rich glycoprotein-like protein mRNA, complete cds
AT3G03130	-1.328031066	2.32E-15	Arabidopsis thaliana uncharacterized protein mRNA, complete cds
AT3G46880	-2.842527823	2.39E-15	Arabidopsis thaliana uncharacterized protein mRNA, complete cds
AT4G17810	-1.602299263	2.45E-15	Arabidopsis thaliana chromosome 4 sequence
AT5G19700	-6.891437423	2.68E-15	Arabidopsis thaliana chromosome 5 sequence
AT5G14100	-1.4154757	2.75E-15	Arabidopsis thaliana ABC transporter I family member 11 mRNA, complete cds
AT5G56320	-1.460058186	2.89E-15	Arabidopsis thaliana expansin A14 mRNA, complete cds
AT4G10510	-4.006914641	3.12E-15	Arabidopsis thaliana Subtilase family protein mRNA, complete cds
AT4G34580	-1.465673517	3.29E-15	Arabidopsis thaliana phosphatidylinositol transfer protein COW1 mRNA, complete cds
AT3G06145	-1.585719555	3.30E-15	Arabidopsis thaliana chromosome 3, complete sequence
AT2G18570	-1.060144446	3.56E-15	Arabidopsis thaliana chromosome 2, complete sequence

AT2G13820	-1.105446261	3.60E-15	Arabidopsis thaliana Non-specific lipid-transfer protein-like protein mRNA, complete cds
AT1G03300	-2.293299534	3.67E-15	Arabidopsis thaliana uncharacterized protein mRNA, complete cds
AT1G07560	-2.51179383	3.68E-15	Arabidopsis thaliana probable LRR receptor-like serine/threonine-protein kinase mRNA, complete cds
AT3G02930	-1.231093111	4.02E-15	Arabidopsis thaliana uncharacterized protein mRNA, complete cds
AT2G39675	-2.019035204	4.55E-15	Arabidopsis thaliana chromosome 2, complete sequence
AT3G62280	-2.616815043	4.56E-15	Arabidopsis thaliana GDSL esterase/lipase mRNA, complete cds
AT5G61030	-1.23317079	4.70E-15	Arabidopsis thaliana glycine-rich RNA-binding protein 3 mRNA, complete cds
AT4G34960	-1.031295087	4.83E-15	Arabidopsis thaliana cyclophilin-like peptidyl-prolyl cis-trans isomerase family protein mRNA, complete cds
AT3G10660	-1.24531106	5.34E-15	Arabidopsis thaliana Ccalcium-dependent protein kinase 2 mRNA, complete cds
AT1G70430	-1.381882325	5.56E-15	Arabidopsis thaliana protein kinase mRNA, complete cds
AT2G01990	-1.495886465	6.28E-15	Arabidopsis thaliana uncharacterized protein mRNA, complete cds
AT3G60270	-3.213365518	6.44E-15	Arabidopsis thaliana cupredoxin superfamily protein mRNA, complete cds
AT3G18090	-1.352697939	6.52E-15	Arabidopsis thaliana DNA-directed RNA polymerase D subunit 2b mRNA, complete cds
AT2G25260	-1.451246447	7.13E-15	Arabidopsis thaliana uncharacterized protein mRNA, complete cds
AT1G75190	-1.502395132	7.13E-15	Arabidopsis thaliana chromosome 1 sequence
AT1G51850	-1.325583745	7.47E-15	Arabidopsis thaliana Leucine-rich repeat protein kinase family protein mRNA, complete cds
AT1G14430	-1.727771412	7.90E-15	Arabidopsis thaliana glyoxal oxidase-related protein mRNA, complete cds
AT4G29270	-1.037334242	9.75E-15	Arabidopsis thaliana HAD superfamily,

			subfamily IIIB acid phosphatase mRNA, complete cds
AT1G16220	-1.651971488	1.17E-14	Arabidopsis thaliana putative protein phosphatase 2C 6 mRNA, complete cds
AT2G26215.1	-1.474512814	1.21E-14	Arabidopsis thaliana chromosome 2, complete sequence
AT3G02820	-1.845143771	1.40E-14	Arabidopsis thaliana CCHC-type zinc knuckle protein mRNA, complete cds
AT1G43800	-1.622948587	1.44E-14	Arabidopsis thaliana acyl-[acyl-carrier-protein] desaturase 6 mRNA, complete cds
AT3G42670	-1.094424445	1.78E-14	Arabidopsis thaliana chromatin remodeling 38 mRNA, complete cds
AT5G44635	-1.17487804	1.92E-14	Arabidopsis thaliana minichromosome maintenance protein 6 mRNA, complete cds
AT3G28910	-1.150556779	1.94E-14	Arabidopsis thaliana myb domain protein 30 mRNA, complete cds
AT2G27770	-1.604698757	1.96E-14	Arabidopsis thaliana chromosome 2, complete sequence
AT1G29430	-1.232474341	2.05E-14	Arabidopsis thaliana chromosome 1 sequence
AT3G02650	-1.267277035	2.17E-14	Arabidopsis thaliana pentatricopeptide repeat-containing protein mRNA, complete cds
AT4G24670	-1.033912905	2.17E-14	Arabidopsis thaliana tryptophan aminotransferase related 2 mRNA, complete cds
AT5G46690	-1.306474922	2.19E-14	Arabidopsis thaliana transcription factor bHLH71 mRNA, complete cds
AT3G09780	-2.365975934	2.22E-14	Arabidopsis thaliana chromosome 3, complete sequence
AT5G42500	-3.306474922	2.44E-14	Arabidopsis thaliana chromosome 5 sequence
AT1G55200	-1.888686812	2.59E-14	Arabidopsis thaliana protein kinase protein with adenine nucleotide alpha hydrolases-like domain mRNA, complete cds
AT3G13160	-1.137703616	2.67E-14	Arabidopsis thaliana chromosome 3, complete sequence
AT4G25250	-2.202451858	2.89E-14	Arabidopsis thaliana chromosome 4 sequence
AT2G33560	-1.440226311	2.95E-14	Arabidopsis thaliana BUB1-related protein BUBR1 mRNA, complete cds

AT2G18450	-2.047556625	3.14E-14	Arabidopsis thaliana succinate dehydrogenase [ubiquinone] flavoprotein subunit 2 mRNA, complete cds
AT5G26680	-1.399584327	3.16E-14	Arabidopsis thaliana flap endonuclease-1 mRNA, complete cds
AT2G20180	-1.044958344	3.24E-14	Arabidopsis thaliana transcription factor PIF1 mRNA, complete cds
AT3G47460	-1.110995163	3.56E-14	Arabidopsis thaliana structural maintenance of chromosomes protein 2-2 mRNA, complete cds
AT3G23830	-1.034851909	3.58E-14	Arabidopsis thaliana glycine-rich RNA-binding protein 4 mRNA, complete cds
AT2G02020	-1.025061987	3.93E-14	Arabidopsis thaliana peptide transporter 4 mRNA, complete cds
AT2G24645	-1.677885489	4.01E-14	Arabidopsis thaliana transcriptional factor B3 family protein mRNA, complete cds
AT4G39300	-1.352055957	4.06E-14	Arabidopsis thaliana uncharacterized protein mRNA, complete cds
AT2G38810	-1.371569951	4.34E-14	Arabidopsis thaliana histone H2A 8 mRNA, complete cds
AT3G16410	-1.035498015	5.00E-14	Arabidopsis thaliana nitrile-specifier protein 4 mRNA, complete cds
AT4G39470	-1.24635393	5.26E-14	Arabidopsis thaliana tetratricopeptide repeat domain-containing protein mRNA, complete cds
AT2G28160	-1.299902269	6.06E-14	Arabidopsis thaliana FER-LIKE IRON DEFICIENCY-INDUCED transcription factor mRNA, complete cds
AT1G76240	-1.668362715	6.12E-14	Arabidopsis thaliana chromosome 1 sequence
AT4G30320	-5.684986546	7.29E-14	Arabidopsis thaliana chromosome 4 sequence
AT1G68725	-1.78660745	7.84E-14	Arabidopsis thaliana arabinogalactan protein 19 mRNA, complete cds
AT5G02360	-1.819487581	8.98E-14	Arabidopsis thaliana DC1 domain-containing protein mRNA, complete cds
AT3G23470	-1.357356091	9.08E-14	Arabidopsis thaliana cyclopropane-fatty-acyl-phospholipid synthase mRNA, complete cds
AT5G48490	-1.14760511	9.86E-14	Arabidopsis thaliana chromosome 5 sequence

AT2G23560	-1.855263811	1.01E-13	Arabidopsis thaliana methyl esterase 7 mRNA, complete cds
AT3G07195	-1.616815043	1.04E-13	Arabidopsis thaliana RPM1-interacting protein 4 (RIN4) family protein mRNA, complete cds
AT3G06140	-1.165876592	1.16E-13	Arabidopsis thaliana probable E3 ubiquitin-protein ligase LUL4 mRNA, complete cds
AT5G49270	-2.891437423	1.20E-13	Arabidopsis thaliana COBRA-like protein 9 mRNA, complete cds
AT1G50900	-1.236383027	1.22E-13	Arabidopsis thaliana protein GRANA DEFICIENT CHLOROPLAST 1 mRNA, complete cds
AT3G16660	-1.007195567	1.28E-13	Arabidopsis thaliana pollen Ole e 1 allergen and extensin family protein mRNA, complete cds
AT2G04530	-1.238788115	1.43E-13	Arabidopsis thaliana ribonuclease Z mRNA, complete cds
AT2G37420	-1.048677165	1.46E-13	Arabidopsis thaliana ATP binding microtubule motor family protein mRNA, complete cds
AT4G21270	-1.037896252	1.61E-13	Arabidopsis thaliana kinesin-like motor protein heavy chain mRNA, complete cds
AT2G18290	-1.007483639	1.79E-13	Arabidopsis thaliana anaphase-promoting complex subunit 10 mRNA, complete cds
AT1G33610	-1.161674515	1.89E-13	Arabidopsis thaliana leucine-rich repeat (LRR) family protein mRNA, complete cds
AT5G07660	-1.781013433	2.04E-13	Arabidopsis thaliana structural maintenance of chromosomes 6A mRNA, complete cds
AT1G49030	-1.781013433	2.04E-13	Arabidopsis thaliana PLAC8 family protein mRNA, complete cds
AT3G62060	-1.283070318	2.04E-13	Arabidopsis thaliana pectinacetylesterase family protein mRNA, complete cds
AT3G46240	-4.18094404	2.07E-13	Arabidopsis thaliana uncharacterized protein mRNA, complete cds
AT5G01740	-2.872072098	2.10E-13	Arabidopsis thaliana chromosome 5 sequence
AT4G14750	-1.639411342	2.16E-13	Arabidopsis thaliana protein IQ-domain 19 mRNA, complete cds
AT1G27435	-1.067602567	2.22E-13	Arabidopsis thaliana uncharacterized

			protein mRNA, complete cds
AT2G31081	-6.65697217	2.27E-13	Arabidopsis thaliana chromosome 2, complete sequence
AT3G20240	-1.517979028	2.36E-13	Arabidopsis thaliana mitochondrial substrate carrier family protein mRNA, complete cds
AT5G45070	-1.487047168	2.39E-13	Arabidopsis thaliana protein PHLOEM PROTEIN 2-LIKE A8 mRNA, complete cds
AT4G19380	-2.388937083	2.42E-13	Arabidopsis thaliana Long-chain-alcohol oxidase FAO4A mRNA, complete cds
AT5G49330	-1.302994354	2.64E-13	Arabidopsis thaliana myb domain protein 111 mRNA, complete cds
AT1G17850	-1.017822982	2.71E-13	Arabidopsis thaliana rhodanese homology domain-containing protein mRNA, complete cds
AT3G47040	-1.912993567	3.06E-13	Arabidopsis thaliana Glycosyl hydrolase family protein mRNA, complete cds
AT4G32710	-1.075245138	3.17E-13	Arabidopsis thaliana proline-rich receptor-like protein kinase PERK14 mRNA, complete cds
AT4G29180	-3.569509328	3.31E-13	Arabidopsis thaliana protein root hair specific 16 mRNA, complete cds
AT3G24300	-1.088619282	3.32E-13	Arabidopsis thaliana chromosome 3, complete sequence
AT3G49860	-2.168971399	3.32E-13	Arabidopsis thaliana ADP-ribosylation factor-like A1B mRNA, complete cds
AT1G77750	-1.023279222	3.33E-13	Arabidopsis thaliana small ribosomal subunit protein S13 mRNA, complete cds
AT5G49640	-1.06311588	3.56E-13	Arabidopsis thaliana chromosome 5 sequence
AT5G26300	-3.817436842	3.58E-13	Arabidopsis thaliana TRAF-like family protein mRNA, complete cds
AT2G14660	-1.588618151	3.59E-13	Arabidopsis thaliana chromosome 2, complete sequence
AT1G14880	-1.588618151	3.59E-13	Arabidopsis thaliana cadmium resistance protein 1 mRNA, complete cds
AT5G52790	-1.803306513	3.65E-13	Arabidopsis thaliana CBS domain-containing protein with a domain of unknown function (DUF21) mRNA, complete cds
AT2G30890	-1.821048095	3.90E-13	Arabidopsis thaliana Cytochrome b561/ferric reductase transmembrane protein family mRNA, complete cds

AT4G38390	-2.606035204	4.06E-13	Arabidopsis thaliana protein root hair specific 17 mRNA, complete cds
AT1G63260	-1.211182473	4.78E-13	Arabidopsis thaliana tetraspanin10 mRNA, complete cds
AT1G53940	-1.775093462	5.29E-13	Arabidopsis thaliana GDSL-motif lipase 2 mRNA, complete cds
AT1G19370	-1.113131034	5.73E-13	Arabidopsis thaliana chromosome 1 sequence
AT3G61100	-1.454688926	6.75E-13	Arabidopsis thaliana putative endonuclease / glycosyl hydrolase mRNA, complete cds
AT3G25790	-1.910546246	8.00E-13	Arabidopsis thaliana myb-like transcription factor family protein mRNA, complete cds
AT3G06740	-1.388937083	8.04E-13	Arabidopsis thaliana GATA transcription factor 15 mRNA, complete cds
AT2G03260	-1.958551619	8.59E-13	Arabidopsis thaliana phosphate transporter PHO1-like 2 mRNA, complete cds
AT1G10930	-1.014294171	9.96E-13	Arabidopsis thaliana ATP-dependent DNA helicase Q-like 4A mRNA, complete cds
AT4G25710	-1.117441098	1.11E-12	Arabidopsis thaliana chromosome 4 sequence
AT4G09420	-3.765906541	1.17E-12	Arabidopsis thaliana TIR-NBS class of disease resistance protein mRNA, complete cds
AT5G56540	-1.431229953	1.27E-12	Arabidopsis thaliana chromosome 5 sequence
AT1G70460	-2.244413954	1.31E-12	Arabidopsis thaliana proline-rich extensin-like receptor kinase 13 mRNA, complete cds
AT5G05510	-1.920981699	1.32E-12	Arabidopsis thaliana Mad3/BUB1 homology region 1 mRNA, complete cds
AT1G65470	-1.145369622	1.58E-12	Arabidopsis thaliana chromatin assembly factor 1 subunit FAS1 mRNA, complete cds
AT1G77520	-1.350499546	1.58E-12	Arabidopsis thaliana O-methyltransferase family protein mRNA, complete cds
AT1G68238	-3.306474922	1.67E-12	Arabidopsis thaliana chromosome 1 sequence
AT1G63560	-2.628403017	1.71E-12	Arabidopsis thaliana chromosome 1 sequence
AT1G14260	-1.842527823	1.80E-12	Arabidopsis thaliana RING-variant

			domain-containing protein mRNA, complete cds
AT1G78090	-1.037788849	1.91E-12	Arabidopsis thaliana trehalose-6-phosphate phosphatase mRNA, complete cds
AT1G15125	-1.600536224	1.92E-12	Arabidopsis thaliana S-adenosyl-L-methionine-dependent methyltransferase mRNA, complete cds
AT1G29600	-1.611634804	2.14E-12	Arabidopsis thaliana putative zinc finger CCCH domain-containing protein 10 mRNA, complete cds
AT3G13960	-1.522511849	2.15E-12	Arabidopsis thaliana growth-regulating factor 5 mRNA, complete cds
AT5G63920	-1.091462031	2.38E-12	Arabidopsis thaliana topoisomerase 3alpha mRNA, complete cds
AT3G20440	-1.223058914	2.40E-12	Arabidopsis thaliana putative glycoside hydrolase mRNA, complete cds
AT4G15620	-1.200275519	2.49E-12	Arabidopsis thaliana uncharacterized protein mRNA, complete cds
AT4G26050	-1.441753781	2.81E-12	Arabidopsis thaliana plant intracellular ras group-related LRR 8 mRNA, complete cds
AT5G06790	-1.330321664	2.83E-12	Arabidopsis thaliana chromosome 5 sequence
AT5G52910	-1.471812654	2.84E-12	Arabidopsis thaliana protein TIMELESS mRNA, complete cds
AT1G02900	-1.004866812	2.96E-12	Arabidopsis thaliana chromosome 1 sequence
AT4G28950	-1.872072098	3.16E-12	Arabidopsis thaliana Rac-like GTP-binding protein ARAC7 mRNA, complete cds
AT1G74890	-1.479311519	3.24E-12	Arabidopsis thaliana two-component response regulator ARR15 mRNA, complete cds
AT3G22210	-1.479311519	3.24E-12	Arabidopsis thaliana uncharacterized protein mRNA, complete cds
AT3G09035	-1.101005804	3.38E-12	Arabidopsis thaliana chromosome 3, complete sequence
AT2G15370	-3.469973655	3.47E-12	Arabidopsis thaliana probable fucosyltransferase 5 mRNA, complete cds
AT4G29450	-3.469973655	3.47E-12	Arabidopsis thaliana leucine-rich repeat protein kinase-like protein mRNA, complete cds

AT4G13620	-1.762154406	3.58E-12	Arabidopsis thaliana chromosome 4 sequence
AT5G10390	-1.361401133	4.10E-12	Arabidopsis thaliana chromosome 5 sequence
AT1G08280	-1.495031112	4.19E-12	Arabidopsis thaliana chromosome 1 sequence
AT1G07070	-1.495031112	4.19E-12	Arabidopsis thaliana 60S ribosomal protein L35a-1 mRNA, complete cds
AT1G07690	-4.539135679	4.29E-12	Arabidopsis thaliana uncharacterized protein mRNA, complete cds
AT1G22690	-1.579925944	4.37E-12	Arabidopsis thaliana gibberellin-regulated protein 9 mRNA, complete cds
AT2G19670	-1.221083431	4.63E-12	Arabidopsis thaliana protein arginine N-methyltransferase 1 mRNA, complete cds
AT5G57530	-6.476399924	4.73E-12	Arabidopsis thaliana probable xyloglucan endotransglucosylase/hydrolase protein 12 mRNA, complete cds
AT5G42510	-2.342098832	4.89E-12	Arabidopsis thaliana chromosome 5 sequence
AT1G33930	-2.342098832	4.89E-12	Arabidopsis thaliana P-loop containing nucleoside triphosphate hydrolases superfamily protein mRNA, complete cds
AT4G37810	-2.589408886	5.03E-12	Arabidopsis thaliana uncharacterized protein mRNA, complete cds
AT3G45070	-1.249046642	5.13E-12	Arabidopsis thaliana chromosome 3, complete sequence
AT1G18140	-1.881453335	5.25E-12	Arabidopsis thaliana laccase 1 mRNA, complete cds
AT5G26130	-1.453316311	6.18E-12	Arabidopsis thaliana chromosome 5 sequence
AT3G27400	-1.402537644	6.67E-12	Arabidopsis thaliana putative pectate lyase 11 mRNA, complete cds
AT2G27660	-1.149606074	6.86E-12	Arabidopsis thaliana chromosome 2, complete sequence
AT1G06120	-3.684986546	6.93E-12	Arabidopsis thaliana delta-9 desaturase-like 3 protein mRNA, complete cds
AT3G43980	-1.7058308	7.22E-12	Arabidopsis thaliana 40S ribosomal protein S29 mRNA, complete cds
AT2G29720	-1.041130356	7.30E-12	Arabidopsis thaliana CTF2B like oxidoreductase mRNA, complete cds

AT4G22110	-4.508108784	7.75E-12	Arabidopsis thaliana GroES-like zinc-binding dehydrogenase family protein mRNA, complete cds
AT2G48130	-1.322869361	7.88E-12	Arabidopsis thaliana protease inhibitor/seed storage/lipid transfer protein (LTP) family protein mRNA, complete cds
AT5G02760	-1.123310898	7.94E-12	Arabidopsis thaliana putative protein phosphatase 2C 67 mRNA, complete cds
AT1G19900	-2.32439683	8.17E-12	Arabidopsis thaliana chromosome 1 sequence
AT2G38160	-1.73790686	8.58E-12	Arabidopsis thaliana uncharacterized protein mRNA, complete cds
AT4G24310	-1.891437423	8.71E-12	Arabidopsis thaliana chromosome 4 sequence
AT3G55660	-1.231559216	8.74E-12	Arabidopsis thaliana ROP (rho of plants) guanine nucleotide exchange factor 6 mRNA, complete cds
AT5G23300	-1.090997769	8.82E-12	Arabidopsis thaliana dihydroorotate dehydrogenase mRNA, complete cds
AT5G07760	-1.916159853	9.09E-12	Arabidopsis thaliana putative formin-like protein 21a mRNA, complete cds
AT4G31600	-1.206939249	9.13E-12	Arabidopsis thaliana UDP-galactose transporter 7 mRNA, complete cds
AT3G43960	-1.393659493	9.72E-12	Arabidopsis thaliana putative cysteine proteinase mRNA, complete cds
AT5G03120	-2.03084048	1.01E-11	Arabidopsis thaliana uncharacterized protein mRNA, complete cds
AT5G45780	-1.294568156	1.03E-11	Arabidopsis thaliana putative LRR receptor-like serine/threonine-protein kinase mRNA, complete cds
AT2G16270	-1.055513349	1.09E-11	Arabidopsis thaliana uncharacterized protein mRNA, complete cds
AT2G25810	-1.569509328	1.11E-11	Arabidopsis thaliana aquaporin TIP4-1 mRNA, complete cds
AT1G61840	-5.410811582	1.11E-11	Arabidopsis thaliana chromosome 1 sequence
AT4G11460	-5.410811582	1.11E-11	Arabidopsis thaliana putative cysteine-rich receptor-like protein kinase 30 mRNA, complete cds
AT2G36110	-3.417506235	1.12E-11	Arabidopsis thaliana chromosome 2, complete sequence
AT5G52260	-3.417506235	1.12E-11	Arabidopsis thaliana myb domain protein 19 mRNA, complete cds

AT1G15510	-1.458478016	1.19E-11	Arabidopsis thaliana chromosome 1 sequence
AT1G28710	-1.166487205	1.32E-11	Arabidopsis thaliana nucleotide-diphospho-sugar transferase family protein mRNA, complete cds
AT4G01390	-1.742574037	1.44E-11	Arabidopsis thaliana TRAF-like family protein mRNA, complete cds
AT3G13940	-1.190451126	1.52E-11	Arabidopsis thaliana DNA binding / DNA-directed RNA polymerase mRNA, complete cds
AT1G48930	-2.015573723	1.62E-11	Arabidopsis thaliana glycosyl hydrolase 9C1 mRNA, complete cds
AT5G52350	-3.206939249	1.70E-11	Arabidopsis thaliana exocyst subunit exo70 family protein A3 mRNA, complete cds
AT2G39681	-1.06454615	1.85E-11	Arabidopsis thaliana chromosome 2, complete sequence
AT3G55150	-1.697264875	1.87E-11	Arabidopsis thaliana chromosome 3, complete sequence
AT1G64270.1	-1.072774011	2.24E-11	Arabidopsis thaliana chromosome 1 sequence
AT4G01350	-1.91346373	2.39E-11	Arabidopsis thaliana chromosome 4 sequence
AT1G61570	-1.209420238	2.43E-11	Arabidopsis thaliana mitochondrial import inner membrane translocase subunit Tim13 mRNA, complete cds
AT2G41650	-1.282227376	2.43E-11	Arabidopsis thaliana uncharacterized protein mRNA, complete cds
AT3G46400	-2.528867344	2.51E-11	Arabidopsis thaliana Leucine-rich repeat protein kinase family protein mRNA, complete cds
AT3G45230	-1.174149314	2.52E-11	Arabidopsis thaliana chromosome 3, complete sequence
AT3G46560	-1.319337073	2.55E-11	Arabidopsis thaliana mitochondrial import inner membrane translocase subunit Tim9 mRNA, complete cds
AT5G54970	-1.604274746	2.62E-11	Arabidopsis thaliana chromosome 5 sequence
AT5G45220	-1.785238019	2.82E-11	Arabidopsis thaliana TIR-NBS-LRR class disease resistance protein mRNA, complete cds
AT5G24230	-1.785238019	2.82E-11	Arabidopsis thaliana lipase class 3-related protein mRNA, complete cds
AT1G68780	-1.038541717	3.19E-11	Arabidopsis thaliana leucine-rich

			repeats-ribonuclease inhibitor domain-containing protein mRNA, complete cds
AT5G39760	-1.347116907	3.21E-11	Arabidopsis thaliana chromosome 5 sequence
AT1G26540	-1.849617247	3.42E-11	Arabidopsis thaliana agenet domain-containing protein mRNA, complete cds
AT4G27730	-1.11674144	3.43E-11	Arabidopsis thaliana oligopeptide transporter mRNA, complete cds
AT5G57780	-1.592779108	3.97E-11	Arabidopsis thaliana uncharacterized protein mRNA, complete cds
AT1G07050	-3.599256672	4.09E-11	Arabidopsis thaliana CCT motif family protein mRNA, complete cds
AT5G19790	-3.599256672	4.09E-11	Arabidopsis thaliana chromosome 5 sequence
AT1G09470	-1.147485399	4.14E-11	Arabidopsis thaliana uncharacterized protein mRNA, complete cds
AT5G65450	-1.053708852	4.76E-11	Arabidopsis thaliana ubiquitin-specific protease 17 mRNA, complete cds
AT1G09630	-1.007630441	5.16E-11	Arabidopsis thaliana RAB GTPase 11C mRNA, complete cds
AT3G07990	-1.077656232	6.10E-11	Arabidopsis thaliana serine carboxypeptidase-like 27 mRNA, complete cds
AT1G76740	-1.348430239	6.23E-11	Arabidopsis thaliana uncharacterized protein mRNA, complete cds
AT2G26860	-1.08594886	6.24E-11	Arabidopsis thaliana FBD-associated F-box protein mRNA, complete cds
AT2G15042	-1.910546246	6.27E-11	Arabidopsis thaliana Leucine-rich repeat (LRR) family protein mRNA, complete cds
AT1G66820	-1.199285676	6.59E-11	Arabidopsis thaliana chromosome 1 sequence
AT3G07910	-1.131888544	6.71E-11	Arabidopsis thaliana uncharacterized protein mRNA, complete cds
AT1G10970	-1.152669586	6.77E-11	Arabidopsis thaliana zinc transporter 4 precursor mRNA, complete cds
AT1G74180	-1.0080058	7.22E-11	Arabidopsis thaliana receptor like protein 14 mRNA, complete cds
AT2G26410	-2.745359164	7.35E-11	Arabidopsis thaliana protein IQ-domain 4 mRNA, complete cds
AT4G19100	-1.104546604	7.65E-11	Arabidopsis thaliana uncharacterized protein mRNA, complete cds

AT2G25210	-1.079406014	8.52E-11	Arabidopsis thaliana 60S ribosomal protein L39-1 mRNA, complete cds
AT1G79470	-1.280788279	9.15E-11	Arabidopsis thaliana inosine-5'-monophosphate dehydrogenase mRNA, complete cds
AT5G02890	-1.056207813	9.31E-11	Arabidopsis thaliana HXXXD-type acyl-transferase-like protein mRNA, complete cds
AT4G30800	-1.485240411	9.61E-11	Arabidopsis thaliana 40S ribosomal protein S11-2 mRNA, complete cds
AT3G05470	-1.072009669	9.83E-11	Arabidopsis thaliana Actin-binding protein FH2 mRNA, complete cds
AT2G17630	-1.072009669	9.83E-11	Arabidopsis thaliana chromosome 2, complete sequence
AT4G11230	-1.744596035	1.06E-10	Arabidopsis thaliana putative respiratory burst oxidase-I mRNA, complete cds
AT2G07709	-1.181583675	1.10E-10	Arabidopsis thaliana chromosome 2, complete sequence
AT2G01210	-1.147167028	1.11E-10	Arabidopsis thaliana leucine-rich repeat transmembrane protein kinase-like protein mRNA, complete cds
AT2G01120	-1.370825334	1.12E-10	Arabidopsis thaliana origin recognition complex subunit 4 mRNA, complete cds
AT2G34360	-1.662618733	1.13E-10	Arabidopsis thaliana MATE efflux family protein mRNA, complete cds
AT5G57220	-1.042390902	1.22E-10	Arabidopsis thaliana cytochrome P450, family 81, subfamily F, polypeptide 2 mRNA, complete cds
AT5G16570	-1.108178177	1.26E-10	Arabidopsis thaliana glutamine synthetase 1;4 mRNA, complete cds
AT1G65710	-1.513799896	1.41E-10	Arabidopsis thaliana chromosome 1 sequence
AT5G06900	-3.859015945	1.44E-10	Arabidopsis thaliana cytochrome P450, family 93, subfamily D, polypeptide 1 mRNA, complete cds
AT5G07150	-1.879849449	1.57E-10	Arabidopsis thaliana leucine-rich repeat protein kinase-like protein mRNA, complete cds
AT5G37010	-1.028940947	1.59E-10	Arabidopsis thaliana uncharacterized protein mRNA, complete cds
AT1G56710	-2.267480791	1.63E-10	Arabidopsis thaliana pectin lyase-like protein mRNA, complete cds
AT3G12870	-1.345734353	2.02E-10	Arabidopsis thaliana chromosome 3, complete sequence

AT1G17090	-1.502395132	2.10E-10	Arabidopsis thaliana uncharacterized protein mRNA, complete cds
AT4G21600	-1.166153634	2.13E-10	Arabidopsis thaliana endonuclease 5 mRNA, complete cds
AT4G25110	-1.607983476	2.13E-10	Arabidopsis thaliana metacaspase 2 mRNA, complete cds
AT1G75120	-1.557436496	2.29E-10	Arabidopsis thaliana protein REDUCED RESIDUAL ARABINOSE 1 mRNA, complete cds
AT4G31730	-1.306474922	2.30E-10	Arabidopsis thaliana chromosome 4 sequence
AT1G78650	-1.379406445	2.50E-10	Arabidopsis thaliana DNA polymerase delta 3 mRNA, complete cds
AT2G14090	-1.20972169	2.85E-10	Arabidopsis thaliana chromosome 2, complete sequence
AT2G14460	-1.104841061	2.85E-10	Arabidopsis thaliana chromosome 2, complete sequence
AT5G36180	-1.984546828	2.90E-10	Arabidopsis thaliana serine carboxypeptidase-like 1 mRNA, complete cds
AT5G20470	-2.502395132	3.12E-10	Arabidopsis thaliana putative myosin mRNA, complete cds
AT3G32030	-1.087116561	3.24E-10	Arabidopsis thaliana terpenoid synthase 30 mRNA, complete cds
AT5G03350	-1.297137058	3.29E-10	Arabidopsis thaliana chromosome 5 sequence
AT3G04450	-1.341481372	3.39E-10	Arabidopsis thaliana myb family transcription factor mRNA, complete cds
AT1G11920	-5.194000193	3.42E-10	Arabidopsis thaliana putative pectate lyase mRNA, complete cds
AT1G49100	-5.194000193	3.42E-10	Arabidopsis thaliana putative leucine-rich repeat protein kinase mRNA, complete cds
AT3G48940	-3.247581233	3.73E-10	Arabidopsis thaliana Remorin-like protein mRNA, complete cds
AT2G17050	-2.3501963	3.90E-10	Arabidopsis thaliana TIR-NBS-LRR class disease resistance protein mRNA, complete cds
AT5G03870	-1.187830426	4.05E-10	Arabidopsis thaliana chromosome 5 sequence
AT1G47480	-1.265959763	4.20E-10	Arabidopsis thaliana probable carboxylesterase 2 mRNA, complete cds
AT1G11160	-1.375567446	4.20E-10	Arabidopsis thaliana WD40 domain-containing protein mRNA,

			complete cds
AT5G30495	-1.06254934	4.21E-10	Arabidopsis thaliana Fcf2 pre-rRNA processing protein mRNA, complete cds
AT4G38420	-1.010309924	4.60E-10	Arabidopsis thaliana protein SKU5 similar 9 mRNA, complete cds
AT5G02820	-1.190356365	4.76E-10	Arabidopsis thaliana DNA topoisomerase 6 subunit A mRNA, complete cds
AT5G40310	-3.79190175	4.76E-10	Arabidopsis thaliana exonuclease family protein mRNA, complete cds
AT4G31060	-1.331565903	4.88E-10	Arabidopsis thaliana ethylene-responsive transcription factor ERF015 mRNA, complete cds
AT1G29440	-1.221586025	5.40E-10	Arabidopsis thaliana chromosome 1 sequence
AT1G51890	-1.100024045	5.45E-10	Arabidopsis thaliana probable LRR receptor-like protein kinase mRNA, complete cds
AT5G06610	-1.274053445	5.74E-10	Arabidopsis thaliana uncharacterized protein mRNA, complete cds
AT1G59540	-1.003912152	6.18E-10	Arabidopsis thaliana kinesin-like protein mRNA, complete cds
AT3G04770	-1.224750525	6.33E-10	Arabidopsis thaliana 40S ribosomal protein Sa-2 mRNA, complete cds
AT1G17480	-1.509637872	6.79E-10	Arabidopsis thaliana protein IQ-domain 7 mRNA, complete cds
AT3G43890	-2.877631624	7.25E-10	Arabidopsis thaliana chromosome 3, complete sequence
AT4G25310	-1.403259985	7.45E-10	Arabidopsis thaliana 2-oxoglutarate (2OG) and Fe(II)-dependent oxygenase superfamily protein mRNA, complete cds
AT4G04990	-2.545261782	7.83E-10	Arabidopsis thaliana uncharacterized protein mRNA, complete cds
AT3G26320	-3.443978446	7.83E-10	Arabidopsis thaliana cytochrome P450 71B36 mRNA, complete cds
AT5G64150	-1.103845756	8.98E-10	Arabidopsis thaliana RNA methyltransferase family protein mRNA, complete cds
AT4G05130	-2.455852547	9.00E-10	Arabidopsis thaliana equilibrative nucleoside transporter 4 mRNA, complete cds
AT5G22555	-4.232474341	9.06E-10	Arabidopsis thaliana uncharacterized protein mRNA, complete cds
AT1G10550	-2.73943433	9.28E-10	Arabidopsis thaliana probable xyloglucan

			endotransglucosylase/hydrolase protein 33 mRNA, complete cds
AT2G06925	-1.31152815	1.01E-09	Arabidopsis thaliana phospholipase A2-alpha mRNA, complete cds
AT2G18800	-1.870375808	1.09E-09	Arabidopsis thaliana probable xyloglucan endotransglucosylase/hydrolase protein 21 mRNA, complete cds
AT1G27050	-1.175972898	1.09E-09	Arabidopsis thaliana RNA recognition motif-containing protein mRNA, complete cds
AT3G53590	-2.621976748	1.14E-09	Arabidopsis thaliana putative leucine-rich repeat receptor-like serine/threonine-protein kinase mRNA, complete cds
AT3G61300	-2.621976748	1.14E-09	Arabidopsis thaliana chromosome 3, complete sequence
AT2G30840	-1.90015464	1.14E-09	Arabidopsis thaliana 2-oxoglutarate dependent oxygenase-like protein mRNA, complete cds
AT5G46880	-1.067439836	1.15E-09	Arabidopsis thaliana homeobox-leucine zipper protein HDG5 mRNA, complete cds
AT3G20110	-3.186180689	1.19E-09	Arabidopsis thaliana cytochrome P450 705A20 mRNA, complete cds
AT2G02630	-1.731780757	1.20E-09	Arabidopsis thaliana chromosome 2, complete sequence
AT4G34770	-2.186180689	1.22E-09	Arabidopsis thaliana chromosome 4 sequence
AT4G30980	-1.399584327	1.25E-09	Arabidopsis thaliana transcription factor bHLH69 mRNA, complete cds
AT5G05890	-1.206939249	1.25E-09	Arabidopsis thaliana UDP-glycosyltransferase 76C5 mRNA, complete cds
AT5G16690	-1.322128187	1.37E-09	Arabidopsis thaliana origin recognition complex subunit 3 mRNA, complete cds
AT5G28626.1	-1.583184265	1.40E-09	Arabidopsis thaliana chromosome 5 sequence
AT3G50460	-3.410811582	1.41E-09	Arabidopsis thaliana RPW8-like protein 2 mRNA, complete cds
AT5G03800	-1.407237899	1.44E-09	Arabidopsis thaliana chromosome 5 sequence
AT1G31885	-2.432005805	1.52E-09	Arabidopsis thaliana aquaporin NIP3-1 mRNA, complete cds
AT2G21020	-1.677569074	1.55E-09	Arabidopsis thaliana chromosome 2,

			complete sequence
AT4G17000	-1.069209225	1.61E-09	Arabidopsis thaliana uncharacterized protein mRNA, complete cds
AT4G37060	-2.712467282	1.61E-09	Arabidopsis thaliana PATATIN-like protein 5 mRNA, complete cds
AT2G47750	-1.228965555	1.69E-09	Arabidopsis thaliana putative indole-3-acetic acid-amido synthetase GH3.9 mRNA, complete cds
AT5G42880	-1.144025042	1.80E-09	Arabidopsis thaliana uncharacterized protein mRNA, complete cds
AT1G55380	-2.59598154	1.95E-09	Arabidopsis thaliana chromosome 1 sequence
AT4G22640	-2.024075192	2.06E-09	Arabidopsis thaliana chromosome 4 sequence
AT1G51880	-3.154471829	2.12E-09	Arabidopsis thaliana protein ROOT HAIR SPECIFIC 6 mRNA, complete cds
AT3G45410	-1.645060361	2.15E-09	Arabidopsis thaliana chromosome 3, complete sequence
AT5G58360	-2.495508747	2.28E-09	Arabidopsis thaliana chromosome 5 sequence
AT1G08810	-1.219566857	2.38E-09	Arabidopsis thaliana putative transcription factor MYB60 mRNA, complete cds
AT4G22485	-1.161044483	2.50E-09	Arabidopsis thaliana chromosome 4 sequence
AT4G31620	-1.482797695	2.54E-09	Arabidopsis thaliana transcriptional factor B3 family protein mRNA, complete cds
AT1G63550	-3.37686425	2.54E-09	Arabidopsis thaliana Receptor-like protein kinase-related family protein mRNA, complete cds
AT3G21180	-2.407758258	2.58E-09	Arabidopsis thaliana calcium-transporting ATPase 9 mRNA, complete cds
AT1G60095	-1.836024303	2.71E-09	Arabidopsis thaliana jacalin-like lectin domain-containing protein mRNA, complete cds
AT1G09450	-1.411556969	2.80E-09	Arabidopsis thaliana protein kinase HASPIN mRNA, complete cds
AT3G63470	-1.700753862	2.87E-09	Arabidopsis thaliana serine carboxypeptidase-like 40 mRNA, complete cds
AT1G52820	-1.700753862	2.87E-09	Arabidopsis thaliana putative 2-oxoglutarate-dependent dioxygenase

			mRNA, complete cds
AT2G42170	-1.351917893	2.88E-09	Arabidopsis thaliana actin family protein mRNA, complete cds
AT4G04695	-1.177191906	2.93E-09	Arabidopsis thaliana calcium-dependent protein kinase 31 mRNA, complete cds
AT5G16900	-1.613903448	2.95E-09	Arabidopsis thaliana putative LRR receptor-like serine/threonine-protein kinase mRNA, complete cds
AT4G31050	-1.247581233	3.74E-09	Arabidopsis thaliana Biotin/lipoate A/B protein ligase family mRNA, complete cds
AT1G51810	-3.122050351	3.79E-09	Arabidopsis thaliana putative LRR receptor-like serine/threonine protein kinase mRNA, complete cds
AT2G03720	-2.79190175	3.89E-09	Arabidopsis thaliana MORPHOGENESIS OF ROOT HAIR 6 mRNA, complete cds
AT5G44600	-1.094600373	3.89E-09	Arabidopsis thaliana S-adenosyl-L-methionine-dependent methyltransferase domain-containing protein mRNA, complete cds
AT1G05650	-1.79190175	3.98E-09	Arabidopsis thaliana pectin lyase-like protein mRNA, complete cds
AT3G46330	-1.168146766	4.08E-09	Arabidopsis thaliana putative LRR receptor-like serine/threonine-protein kinase MEE39 mRNA, complete cds
AT5G44620	-1.399584327	4.09E-09	Arabidopsis thaliana cytochrome P450, family 706, subfamily A, polypeptide 3 mRNA, complete cds
AT1G65620	-1.340690638	4.17E-09	Arabidopsis thaliana protein ASYMMETRIC LEAVES 2 mRNA, complete cds
AT5G61520	-1.21672027	4.63E-09	Arabidopsis thaliana sugar transport protein 3 mRNA, complete cds
AT3G24400	-2.306474922	4.74E-09	Arabidopsis thaliana chromosome 3, complete sequence
AT1G77020	-1.056496669	4.94E-09	Arabidopsis thaliana DNAJ heat shock N-terminal domain-containing protein mRNA, complete cds
AT3G51360	-2.122050351	5.41E-09	Arabidopsis thaliana aspartyl protease family protein mRNA, complete cds
AT3G46270	-4.113829845	5.47E-09	Arabidopsis thaliana receptor protein kinase-like protein mRNA, complete cds
AT3G26815	-4.113829845	5.47E-09	Arabidopsis thaliana chromosome 3,

			complete sequence
AT5G49215	-1.203715348	5.54E-09	Arabidopsis thaliana pectin lyase-like superfamily protein mRNA, complete cds
AT5G41880	-1.145538704	5.68E-09	Arabidopsis thaliana DNA primase POLA3 mRNA, complete cds
AT5G64110	-1.750081574	5.75E-09	Arabidopsis thaliana peroxidase 70 mRNA, complete cds
AT4G28890	-1.774623758	6.21E-09	Arabidopsis thaliana chromosome 4 sequence
AT2G34050	-1.190997705	6.61E-09	Arabidopsis thaliana ATP11 molecular chaperone domain-containing protein mRNA, complete cds
AT5G55125	-1.16142459	6.70E-09	Arabidopsis thaliana ribosomal protein L31 mRNA, complete cds
AT2G13550	-1.434349745	7.16E-09	Arabidopsis thaliana chromosome 2, complete sequence
AT2G28970	-1.478086301	7.25E-09	Arabidopsis thaliana putative leucine-rich repeat protein kinase mRNA, complete cds
AT3G62930	-1.689090944	7.47E-09	Arabidopsis thaliana chromosome 3, complete sequence
AT2G27775	-1.367875467	7.52E-09	Arabidopsis thaliana uncharacterized protein mRNA, complete cds
AT1G24620	-5.938743138	7.67E-09	Arabidopsis thaliana chromosome 1 sequence
AT1G22500	-1.014920477	8.13E-09	Arabidopsis thaliana chromosome 1 sequence
AT1G05880	-1.964368946	8.58E-09	Arabidopsis thaliana putative E3 ubiquitin-protein ligase ARI12 mRNA, complete cds
AT2G38600	-1.231186795	8.77E-09	Arabidopsis thaliana HAD superfamily, subfamily IIIB acid phosphatase mRNA, complete cds
AT4G15380	-1.454032111	9.39E-09	Arabidopsis thaliana cytochrome P450, family 705, subfamily A, polypeptide 4 mRNA, complete cds
AT3G18730	-1.348821213	9.41E-09	Arabidopsis thaliana protein BRUSHY 1 mRNA, complete cds
AT1G16280	-1.015243625	9.63E-09	Arabidopsis thaliana DEAD-box ATP-dependent RNA helicase 36 mRNA, complete cds
AT4G22080	-4.072009669	9.97E-09	Arabidopsis thaliana root hair specific 14 mRNA, complete cds

AT4G37490	-1.050889322	1.11E-08	Arabidopsis thaliana cyclin-B1-1 mRNA, complete cds
AT1G67630	-1.024075192	1.19E-08	Arabidopsis thaliana DNA polymerase alpha 2 mRNA, complete cds
AT4G13572	-1.239119655	1.21E-08	Arabidopsis thaliana uncharacterized protein mRNA, complete cds
AT4G17540	-1.032909849	1.23E-08	Arabidopsis thaliana uncharacterized protein mRNA, complete cds
AT3G10040	-1.20355661	1.26E-08	Arabidopsis thaliana chromosome 3, complete sequence
AT1G29450	-1.17163238	1.29E-08	Arabidopsis thaliana chromosome 1 sequence
AT1G62870	-1.17163238	1.29E-08	Arabidopsis thaliana chromosome 1 sequence
AT2G32280	-1.312351489	1.45E-08	Arabidopsis thaliana uncharacterized protein mRNA, complete cds
AT1G05810	-1.033456428	1.46E-08	Arabidopsis thaliana RAB GTPase homolog A5E mRNA, complete cds
AT5G20400	-1.1450115	1.53E-08	Arabidopsis thaliana flavanone 3 hydroxylase-like protein mRNA, complete cds
AT4G25070	-1.007879331	1.54E-08	Arabidopsis thaliana uncharacterized protein mRNA, complete cds
AT3G07190	-1.007879331	1.54E-08	Arabidopsis thaliana B-cell receptor-associated protein 31-like protein mRNA, complete cds
AT5G47950	-2.859015945	1.62E-08	Arabidopsis thaliana chromosome 5 sequence
AT3G07070	-2.859015945	1.62E-08	Arabidopsis thaliana serine/threonine-protein kinase mRNA, complete cds
AT3G05150	-1.76476562	1.63E-08	Arabidopsis thaliana sugar transporter ERD6-like 8 mRNA, complete cds
AT1G05205	-1.22847241	1.71E-08	Arabidopsis thaliana uncharacterized protein mRNA, complete cds
AT5G13060	-1.107605224	1.75E-08	Arabidopsis thaliana ARMADILLO BTB protein 1 mRNA, complete cds
AT1G12060	-1.147276328	1.80E-08	Arabidopsis thaliana BCL-2-associated athanogene 5 mRNA, complete cds
AT5G43370	-4.028940947	1.82E-08	Arabidopsis thaliana phosphate transporter Pht1;2 mRNA, complete cds
AT3G18010	-1.386645271	1.95E-08	Arabidopsis thaliana WUSCHEL-related homeobox 1 mRNA, complete cds
AT5G45700	-1.386645271	1.95E-08	Arabidopsis thaliana chromosome 5

			sequence
AT1G69240	-4.891437423	2.01E-08	Arabidopsis thaliana methyl esterase 15 mRNA, complete cds
AT1G23100	-2.700753862	2.05E-08	Arabidopsis thaliana GroES-like protein mRNA, complete cds
AT1G20720	-1.044135624	2.12E-08	Arabidopsis thaliana RAD3-like DNA-binding helicase protein mRNA, complete cds
AT5G43070	-1.164455918	2.12E-08	Arabidopsis thaliana chromosome 5 sequence
AT3G11520	-1.054087761	2.19E-08	Arabidopsis thaliana cyclin-B1-3 mRNA, complete cds
AT3G26818	-2.232474341	2.20E-08	Arabidopsis thaliana chromosome 3, complete sequence
AT5G46220	-1.963173177	2.27E-08	Arabidopsis thaliana uncharacterized protein mRNA, complete cds
AT5G49560	-1.721512422	2.33E-08	Arabidopsis thaliana chromosome 5 sequence
AT3G23805	-1.236610116	2.35E-08	Arabidopsis thaliana chromosome 3, complete sequence
AT3G44960	-1.447518804	2.69E-08	Arabidopsis thaliana uncharacterized protein mRNA, complete cds
AT3G16390	-1.639050262	2.69E-08	Arabidopsis thaliana Nitrile-specifier protein 3 mRNA, complete cds
AT4G13310	-1.773042722	2.74E-08	Arabidopsis thaliana cytochrome P450 71A20 mRNA, complete cds
AT2G25220	-1.373589118	2.85E-08	Arabidopsis thaliana protein kinase family protein mRNA, complete cds
AT3G52630	-1.569509328	2.94E-08	Arabidopsis thaliana Nucleic acid-binding, OB-fold-like protein mRNA, complete cds
AT3G28510	-3.984546828	3.32E-08	Arabidopsis thaliana chromosome 3, complete sequence
AT5G08400	-1.056696613	3.63E-08	Arabidopsis thaliana uncharacterized protein mRNA, complete cds
AT2G33680	-1.60345666	3.68E-08	Arabidopsis thaliana chromosome 2, complete sequence
AT4G16350	-1.984546828	3.75E-08	Arabidopsis thaliana calcineurin B-like protein 6 mRNA, complete cds
AT1G44020	-2.984546828	3.77E-08	Arabidopsis thaliana cysteine/histidine-rich C1 domain-containing protein mRNA, complete cds
AT4G00680	-2.031852542	3.81E-08	Arabidopsis thaliana actin

			depolymerizing factor 8 mRNA, complete cds
AT4G29240	-1.727771412	3.94E-08	Arabidopsis thaliana chromosome 4 sequence
AT1G54385	-1.009493185	4.25E-08	Arabidopsis thaliana ARM repeat superfamily protein mRNA, complete cds
AT4G28430	-1.276727579	4.26E-08	Arabidopsis thaliana reticulon-like protein B18 mRNA, complete cds
AT3G03060	-1.027874259	4.60E-08	Arabidopsis thaliana P-loop containing nucleoside triphosphate hydrolases superfamily protein mRNA, complete cds
AT5G25820	-1.306474922	4.73E-08	Arabidopsis thaliana Exostosin family protein mRNA, complete cds
AT1G14160	-3.194000193	4.75E-08	Arabidopsis thaliana uncharacterized protein mRNA, complete cds
AT1G10030	-1.009784119	5.03E-08	Arabidopsis thaliana Ergosterol biosynthetic protein 28-like protein mRNA, complete cds
AT5G59360	-1.845143771	5.27E-08	Arabidopsis thaliana chromosome 5 sequence
AT5G05990	-1.312872694	5.51E-08	Arabidopsis thaliana mitochondrial glycoprotein family protein mRNA, complete cds
AT3G12240	-1.880711017	5.57E-08	Arabidopsis thaliana serine carboxypeptidase-like 15 mRNA, complete cds
AT4G39630	-1.522203614	5.88E-08	Arabidopsis thaliana uncharacterized protein mRNA, complete cds
AT4G11190	-1.01008192	5.96E-08	Arabidopsis thaliana chromosome 4 sequence
AT4G33020	-3.938743138	6.06E-08	Arabidopsis thaliana Fe(II) transporter isolog family protein mRNA, complete cds
AT3G62780	-2.00839357	6.16E-08	Arabidopsis thaliana chromosome 3, complete sequence
AT3G30122	-1.604698757	6.25E-08	Arabidopsis thaliana chromosome 3, complete sequence
AT4G28190	-1.082727222	6.34E-08	Arabidopsis thaliana protein ULTRAPETALA 1 mRNA, complete cds
AT5G14580	-1.028940947	6.46E-08	Arabidopsis thaliana polyribonucleotide nucleotidyltransferase mRNA, complete cds

AT3G12540	-4.79190175	6.56E-08	Arabidopsis thaliana uncharacterized protein mRNA, complete cds
AT2G26840	-1.108282196	6.58E-08	Arabidopsis thaliana uncharacterized protein mRNA, complete cds
AT4G17860	-2.948020952	6.68E-08	Arabidopsis thaliana uncharacterized protein mRNA, complete cds
AT5G49780	-1.269949046	7.11E-08	Arabidopsis thaliana leucine-rich repeat protein kinase-like protein mRNA, complete cds
AT1G65700	-1.072009669	7.33E-08	Arabidopsis thaliana Small nuclear ribonucleoprotein family protein mRNA, complete cds
AT1G78770	-1.029494234	7.65E-08	Arabidopsis thaliana anaphase-promoting complex subunit 6 mRNA, complete cds
AT2G39040	-1.666370868	8.55E-08	Arabidopsis thaliana peroxidase 24 mRNA, complete cds
AT4G10530	-2.757136331	8.70E-08	Arabidopsis thaliana Subtilase family protein mRNA, complete cds
AT1G05720	-1.451672838	8.81E-08	Arabidopsis thaliana selenoprotein family protein mRNA, complete cds
AT2G45890	-1.451672838	8.81E-08	Arabidopsis thaliana mRNA for hypothetical protein, complete cds, clone: RAFL16-63-K09
AT2G45360	-1.859015945	8.83E-08	Arabidopsis thaliana uncharacterized protein mRNA, complete cds
AT1G07300	-1.030061487	9.07E-08	Arabidopsis thaliana chromosome 1 sequence
AT5G19800	-3.443978446	1.01E-07	Arabidopsis thaliana chromosome 5 sequence
AT1G01390	-1.074432331	1.03E-07	Arabidopsis thaliana chromosome 1 sequence
AT1G61750	-2.60345666	1.07E-07	Arabidopsis thaliana Receptor-like protein kinase-related protein mRNA, complete cds
AT3G47710	-2.60345666	1.07E-07	Arabidopsis thaliana atypical non-DNA binding bHLH protein BNQ3 mRNA, complete cds
AT2G36690	-2.60345666	1.07E-07	Arabidopsis thaliana 2-oxoglutarate (2OG) and Fe(II)-dependent oxygenase superfamily protein mRNA, complete cds
AT1G48460	-1.100024045	1.07E-07	Arabidopsis thaliana uncharacterized protein mRNA, complete cds
AT4G15300	-3.891437423	1.11E-07	Arabidopsis thaliana cytochrome P450,

			family 702, subfamily A, polypeptide 2 mRNA, complete cds
AT4G30860	-1.143745422	1.11E-07	Arabidopsis thaliana histone-lysine N-methyltransferase ASHR3 mRNA, complete cds
AT3G15140	-1.052110111	1.15E-07	Arabidopsis thaliana polynucleotidyl transferase, ribonuclease H-like superfamily protein mRNA, complete cds
AT5G03570	-1.626092857	1.18E-07	Arabidopsis thaliana nickel transport protein FPN2 mRNA, complete cds
AT5G24313	-5.684986546	1.22E-07	Arabidopsis thaliana chromosome 5 sequence
AT1G05210	-1.436242797	1.30E-07	Arabidopsis thaliana putative transmembrane protein 97 mRNA, complete cds
AT1G08610	-1.268339794	1.39E-07	Arabidopsis thaliana pentatricopeptide repeat-containing protein mRNA, complete cds
AT1G61667	-1.327434541	1.46E-07	Arabidopsis thaliana uncharacterized protein mRNA, complete cds
AT1G33900	-3.113829845	1.52E-07	Arabidopsis thaliana P-loop containing nucleoside triphosphate hydrolases superfamily protein mRNA, complete cds
AT4G24175	-1.165876592	1.54E-07	Arabidopsis thaliana uncharacterized protein mRNA, complete cds
AT1G62010	-1.915284165	1.55E-07	Arabidopsis thaliana chromosome 1 sequence
AT5G42780	-1.365975934	1.58E-07	Arabidopsis thaliana chromosome 5 sequence
AT3G51290	-1.002468736	1.58E-07	Arabidopsis thaliana protein ALTERED PHOSPHATE STARVATION RESPONSE 1 mRNA, complete cds
AT4G11210	-1.69504021	1.60E-07	Arabidopsis thaliana chromosome 4 sequence
AT2G14510	-1.587887858	1.61E-07	Arabidopsis thaliana leucine-rich repeat protein kinase family protein mRNA, complete cds
AT4G19645	-2.065466823	1.65E-07	Arabidopsis thaliana TRAM, LAG1 and CLN8 (TLC) lipid-sensing domain containing protein mRNA, complete cds
AT2G39920	-2.065466823	1.65E-07	Arabidopsis thaliana acid phosphatase class IIIB protein mRNA, complete cds

AT3G06680	-1.012027564	1.65E-07	Arabidopsis thaliana 60S ribosomal protein L29-2 mRNA, complete cds
AT2G38620	-1.250136938	1.69E-07	Arabidopsis thaliana cyclin-dependent kinase B1-2 mRNA, complete cds
AT2G19990	-3.399584327	1.83E-07	Arabidopsis thaliana chromosome 2, complete sequence
AT4G28180	-1.054936155	1.91E-07	Arabidopsis thaliana chromosome 4 sequence
AT4G22790	-1.53474391	1.92E-07	Arabidopsis thaliana chromosome 4 sequence
AT1G73360	-1.190997705	2.12E-07	Arabidopsis thaliana homeobox-leucine zipper protein HDG11 mRNA, complete cds
AT1G30560	-5.628403017	2.14E-07	Arabidopsis thaliana chromosome 1 sequence
AT1G28620	-1.851280297	2.34E-07	Arabidopsis thaliana chromosome 1 sequence
AT1G55420	-2.247581233	2.52E-07	Arabidopsis thaliana chromosome 1 sequence
AT3G18200	-1.443978446	2.53E-07	Arabidopsis thaliana nodulin MtN21-like transporter UMAMIT4 mRNA, complete cds
AT2G16760	-1.935637227	2.57E-07	Arabidopsis thaliana uncharacterized protein mRNA, complete cds
AT4G13070	-1.04508837	2.60E-07	Arabidopsis thaliana RNA-binding CRS1 / YhbY (CRM) domain protein mRNA, complete cds
AT5G59260	-2.684986546	2.65E-07	Arabidopsis thaliana chromosome 5 sequence
AT5G61310	-1.266582195	2.71E-07	Arabidopsis thaliana chromosome 5 sequence
AT3G18217	-3.072009669	2.72E-07	Arabidopsis thaliana chromosome 3, complete sequence
AT4G15290	-1.588618151	2.74E-07	Arabidopsis thaliana cellulose synthase-like protein B5 mRNA, complete cds
AT1G27420	-1.198671633	2.94E-07	Arabidopsis thaliana putative F-box/kelch-repeat protein mRNA, complete cds
AT1G06790	-1.126565832	2.97E-07	Arabidopsis thaliana RNA polymerase Rpb7 N-terminal domain-containing protein mRNA, complete cds
AT2G34925	-2.53474391	3.16E-07	Arabidopsis thaliana chromosome 2, complete sequence

AT1G10400	-2.53474391	3.16E-07	Arabidopsis thaliana UDP-glycosyltransferase 90A2 mRNA, complete cds
AT1G04425	-1.272527591	3.18E-07	Arabidopsis thaliana clone 23398 mRNA sequence
AT5G42110	-1.533440074	3.26E-07	Arabidopsis thaliana uncharacterized protein mRNA, complete cds
AT3G50130	-3.353780637	3.31E-07	Arabidopsis thaliana uncharacterized protein mRNA, complete cds
AT1G01750	-1.79190175	3.46E-07	Arabidopsis thaliana actin depolymerizing factor 11 mRNA, complete cds
AT5G63140	-1.145701619	3.53E-07	Arabidopsis thaliana purple acid phosphatase 29 mRNA, complete cds
AT4G24710	-1.378825767	3.57E-07	Arabidopsis thaliana P-loop containing nucleoside triphosphate hydrolases superfamily protein mRNA, complete cds
AT3G44970	-2.410811582	3.60E-07	Arabidopsis thaliana cytochrome P450 family protein mRNA, complete cds
AT3G29110	-1.046831106	3.65E-07	Arabidopsis thaliana putative terpenoid synthase 16 mRNA, complete cds
AT5G26080	-5.569509328	3.76E-07	Arabidopsis thaliana chromosome 5 sequence
AT1G33890	-5.569509328	3.76E-07	Arabidopsis thaliana avirulence induced protein AIG1 mRNA, complete cds
AT2G24395	-1.072009669	3.87E-07	Arabidopsis thaliana chaperone protein dnaJ-related protein mRNA, complete cds
AT5G66985	-4.628403017	3.92E-07	Arabidopsis thaliana chromosome 5 sequence
AT2G25410	-4.628403017	3.92E-07	Arabidopsis thaliana RING-H2 finger protein ATL22 mRNA, complete cds
AT2G15350	-4.628403017	3.92E-07	Arabidopsis thaliana chromosome 2, complete sequence
AT2G12170	-1.958551619	4.26E-07	Arabidopsis thaliana uncharacterized protein mRNA, complete cds
AT3G12977	-1.439741453	4.28E-07	Arabidopsis thaliana no apical meristem-domain containing transcriptional regulator mRNA, complete cds
AT2G04800	-1.439741453	4.28E-07	Arabidopsis thaliana chromosome 2, complete sequence
AT5G41010	-1.285206306	4.35E-07	Arabidopsis thaliana DNA-directed RNA

			polymerase NRPB12/NRPD12/NRPE12 mRNA, complete cds
AT1G66800	-1.707012852	4.58E-07	Arabidopsis thaliana alcohol dehydrogenase-like protein mRNA, complete cds
AT4G13390	-2.64751184	4.60E-07	Arabidopsis thaliana chromosome 4 sequence
AT1G19960	-2.64751184	4.60E-07	Arabidopsis thaliana chromosome 1 sequence
AT5G48920	-1.101903778	4.79E-07	Arabidopsis thaliana chromosome 5 sequence
AT1G68150	-1.211317689	4.79E-07	Arabidopsis thaliana Group II-b WRKY transcription factor mRNA, complete cds
AT5G45080	-1.117441098	4.86E-07	Arabidopsis thaliana protein PHLOEM PROTEIN 2-LIKE A6 mRNA, complete cds
AT1G65032	-1.291975353	5.08E-07	Arabidopsis thaliana uncharacterized protein mRNA, complete cds
AT5G05940	-1.004175634	5.22E-07	Arabidopsis thaliana ROP guanine nucleotide exchange factor 5 mRNA, complete cds
AT5G35732	-1.768818137	5.42E-07	Arabidopsis thaliana chromosome 5 sequence
AT4G31805	-1.215872374	5.64E-07	Arabidopsis thaliana protein POLAR mRNA, complete cds
AT5G49170	-1.803974582	5.83E-07	Arabidopsis thaliana uncharacterized protein mRNA, complete cds
AT3G10710	-2.37686425	6.11E-07	Arabidopsis thaliana root hair specific 12 mRNA, complete cds
AT3G17640	-1.004446385	6.20E-07	Arabidopsis thaliana chromosome 3, complete sequence
AT2G19970	-1.42266794	6.32E-07	Arabidopsis thaliana chromosome 2, complete sequence
AT4G10540	-2.79190175	6.46E-07	Arabidopsis thaliana Subtilase family protein mRNA, complete cds
AT1G49230	-1.015135147	6.47E-07	Arabidopsis thaliana chromosome 1 sequence
AT1G34245	-1.885011154	6.56E-07	Arabidopsis thaliana protein EPIDERMAL PATTERNING FACTOR 2 mRNA, complete cds
AT2G26400	-2.274053445	6.62E-07	Arabidopsis thaliana acireductone dioxygenase 3 mRNA, complete cds
AT3G60280	-5.508108784	6.64E-07	Arabidopsis thaliana uclacyanin 3 mRNA, complete cds

AT5G22560	-5.508108784	6.64E-07	Arabidopsis thaliana chromosome 5 sequence
AT2G26440	-1.198142301	6.76E-07	Arabidopsis thaliana Probable pectinesterase/pectinesterase inhibitor 12 mRNA, complete cds
AT4G15740	-2.186180689	6.96E-07	Arabidopsis thaliana chromosome 4 sequence
AT4G13690	-1.984546828	7.06E-07	Arabidopsis thaliana uncharacterized protein mRNA, complete cds
AT1G33612	-1.435208237	7.27E-07	Arabidopsis thaliana Leucine-rich repeat (LRR) family protein mRNA, complete cds
AT3G08660	-1.590267888	7.92E-07	Arabidopsis thaliana phototropic-responsive NPH3 family protein mRNA, complete cds
AT1G79490	-1.107929243	7.93E-07	Arabidopsis thaliana chromosome 1 sequence
AT5G25580	-1.202451858	7.95E-07	Arabidopsis thaliana uncharacterized protein mRNA, complete cds
AT1G49490	-1.314252273	8.07E-07	Arabidopsis thaliana chromosome 1 sequence
AT1G29418	-1.038994612	8.27E-07	Arabidopsis thaliana chromosome 1 sequence
AT1G72200	-1.051661023	8.55E-07	Arabidopsis thaliana chromosome 1 sequence
AT1G61050	-2.984546828	8.64E-07	Arabidopsis thaliana chromosome 1 sequence
AT4G30560	-1.079183702	9.04E-07	Arabidopsis thaliana cyclic nucleotide gated channel 9 mRNA, complete cds
AT3G50890	-1.016025059	9.10E-07	Arabidopsis thaliana homeobox protein 28 mRNA, complete cds
AT3G60580	-2.462594124	9.28E-07	Arabidopsis thaliana chromosome 3, complete sequence
AT3G45700	-1.206939249	9.36E-07	Arabidopsis thaliana putative nitrate excretion transporter 5 mRNA, complete cds
AT2G44370	-1.11007771	9.39E-07	Arabidopsis thaliana chromosome 2, complete sequence
AT3G26490	-1.027615549	9.46E-07	Arabidopsis thaliana phototropic-responsive NPH3 family protein mRNA, complete cds
AT4G25600	-1.1450115	9.56E-07	Arabidopsis thaliana Oxoglutarate/iron-dependent oxygenase mRNA, complete cds

AT5G24140	-1.817436842	9.82E-07	Arabidopsis thaliana squalene monooxygenase 2 mRNA, complete cds
AT4G11140	-1.817436842	9.82E-07	Arabidopsis thaliana chromosome 4 sequence
AT1G70895	-1.366417463	1.02E-06	Arabidopsis thaliana chromosome 1 sequence
AT1G65610	-1.005305388	1.03E-06	Arabidopsis thaliana mRNA for hypothetical protein, complete cds, clone: RAFL16-65-K04
AT2G26211	-1.859015945	1.04E-06	Arabidopsis thaliana chromosome 2, complete sequence
AT4G01820	-3.257565322	1.08E-06	Arabidopsis thaliana ABC transporter B family member 3 mRNA, complete cds
AT5G56810	-2.240886581	1.11E-06	Arabidopsis thaliana putative F-box/FBD/LRR-repeat protein mRNA, complete cds
AT5G46090	-1.167768652	1.13E-06	Arabidopsis thaliana chromosome 5 sequence
AT3G09220	-1.14804556	1.13E-06	Arabidopsis thaliana laccase 7 mRNA, complete cds
AT5G20550	-1.298655418	1.16E-06	Arabidopsis thaliana oxidoreductase, 2OG-Fe(II) oxygenase family protein mRNA, complete cds
AT2G13910	-2.079704061	1.17E-06	Arabidopsis thaliana chromosome 2, complete sequence
AT5G23903	-5.443978446	1.18E-06	Arabidopsis thaliana uncharacterized protein mRNA, complete cds
AT5G38910	-5.443978446	1.18E-06	Arabidopsis thaliana putative germin-like protein subfamily 1 member 9 mRNA, complete cds
AT1G07680	-5.443978446	1.18E-06	Arabidopsis thaliana uncharacterized protein mRNA, complete cds
AT2G28860	-3.684986546	1.23E-06	Arabidopsis thaliana chromosome 2, complete sequence
AT1G19390	-3.684986546	1.23E-06	Arabidopsis thaliana putative wall-associated receptor kinase-like 11 mRNA, complete cds
AT2G33793	-1.082394151	1.27E-06	Arabidopsis thaliana uncharacterized protein mRNA, complete cds
AT2G05632	-1.016968305	1.28E-06	Arabidopsis thaliana chromosome 2, complete sequence
AT3G12860	-1.016968305	1.28E-06	Arabidopsis thaliana NOP56-like pre RNA processing ribonucleoprotein mRNA, complete cds

AT5G01910	-1.216493556	1.30E-06	Arabidopsis thaliana uncharacterized protein mRNA, complete cds
AT3G20850	-4.508108784	1.31E-06	Arabidopsis thaliana chromosome 3, complete sequence
AT2G42730	-1.193133449	1.32E-06	Arabidopsis thaliana F-box protein mRNA, complete cds
AT3G08520	-1.041577773	1.38E-06	Arabidopsis thaliana 60S ribosomal protein L41 mRNA, complete cds
AT1G79420	-1.054936155	1.43E-06	Arabidopsis thaliana uncharacterized protein mRNA, complete cds
AT1G55430	-2.938743138	1.54E-06	Arabidopsis thaliana chromosome 1 sequence
AT2G45550	-1.79190175	1.55E-06	Arabidopsis thaliana cytochrome P450 76C4 mRNA, complete cds
AT5G14000	-1.175230389	1.57E-06	Arabidopsis thaliana NAC domain containing protein 84 mRNA, complete cds
AT3G28840	-2.425119419	1.58E-06	Arabidopsis thaliana uncharacterized protein mRNA, complete cds
AT5G66940	-1.832543734	1.65E-06	Arabidopsis thaliana chromosome 5 sequence
AT1G18980	-1.25373346	1.74E-06	Arabidopsis thaliana chromosome 1 sequence
AT5G15770	-1.877631624	1.75E-06	Arabidopsis thaliana chromosome 5 sequence
AT3G50470	-1.548447713	1.82E-06	Arabidopsis thaliana RPW8-like protein 3 mRNA, complete cds
AT1G67180	-1.411968051	1.82E-06	Arabidopsis thaliana RING-finger and BRCT domain-containing protein mRNA, complete cds
AT3G05727	-1.667356652	1.86E-06	Arabidopsis thaliana defensin-like protein 204 mRNA, complete cds
AT4G18510	-1.667356652	1.86E-06	Arabidopsis thaliana chromosome 4 sequence
AT2G18735	-1.290355257	1.95E-06	Arabidopsis thaliana chromosome 2, complete sequence
AT1G67910	-1.569509328	2.05E-06	Arabidopsis thaliana uncharacterized protein mRNA, complete cds
AT4G11490	-5.37686425	2.10E-06	Arabidopsis thaliana putative cysteine-rich receptor-like protein kinase 33 mRNA, complete cds
AT3G49170	-1.232474341	2.11E-06	Arabidopsis thaliana pentatricopeptide repeat-containing protein mRNA, complete cds

AT5G64816	-1.141051313	2.20E-06	Arabidopsis thaliana uncharacterized protein mRNA, complete cds
AT3G03850	-1.729974	2.24E-06	Arabidopsis thaliana chromosome 3, complete sequence
AT2G23690	-1.729974	2.24E-06	Arabidopsis thaliana uncharacterized protein mRNA, complete cds
AT4G22460	-3.628403017	2.26E-06	Arabidopsis thaliana chromosome 4 sequence
AT1G16590	-1.38143698	2.31E-06	Arabidopsis thaliana putative translesion synthesis polymerase zeta subunit REV7 mRNA, complete cds
AT3G62760	-1.38143698	2.31E-06	Arabidopsis thaliana glutathione S-transferase F13 mRNA, complete cds
AT4G26220	-1.211957324	2.54E-06	Arabidopsis thaliana putative caffeoyl-CoA O-methyltransferase mRNA, complete cds
AT3G56020	-1.393352373	2.67E-06	Arabidopsis thaliana 60S ribosomal protein L41 mRNA, complete cds
AT5G26270	-2.891437423	2.72E-06	Arabidopsis thaliana uncharacterized protein mRNA, complete cds
AT4G33730	-2.891437423	2.72E-06	Arabidopsis thaliana chromosome 4 sequence
AT3G01080	-1.64350991	2.86E-06	Arabidopsis thaliana WRKY DNA-binding protein 58 mRNA, complete cds
AT4G14980	-2.269949046	2.93E-06	Arabidopsis thaliana chromosome 4 sequence
AT2G43480	-2.269949046	2.93E-06	Arabidopsis thaliana probable peroxidase 26 mRNA, complete cds
AT2G16230	-1.954173179	3.05E-06	Arabidopsis thaliana O-glycosyl hydrolases family 17 protein mRNA, complete cds
AT3G06390	-1.250826893	3.41E-06	Arabidopsis thaliana uncharacterized protein mRNA, complete cds
AT4G34930	-2.662618733	3.46E-06	Arabidopsis thaliana chromosome 4 sequence
AT5G51310	-1.419483884	3.56E-06	Arabidopsis thaliana protein LONGER ROOT HAIRS UNDER PHOSPHATE-DEFICIENCY mRNA, complete cds
AT2G22870	-1.033456428	3.72E-06	Arabidopsis thaliana GTP-binding protein EMB2001 mRNA, complete cds
AT1G33870	-5.306474922	3.74E-06	Arabidopsis thaliana P-loop containing nucleoside triphosphate hydrolases

			superfamily protein mRNA, complete cds
AT4G03330	-1.73943433	3.80E-06	Arabidopsis thaliana syntaxin-123 mRNA, complete cds
AT3G26330	-1.0080058	4.08E-06	Arabidopsis thaliana cytochrome P450 71B37 mRNA, complete cds
AT1G67260	-2.487047168	4.09E-06	Arabidopsis thaliana transcription factor TCP1 mRNA, complete cds
AT4G30420	-2.487047168	4.09E-06	Arabidopsis thaliana nodulin MtN21-like transporter family protein mRNA, complete cds
AT3G11750	-1.505378991	4.11E-06	Arabidopsis thaliana Dihydroneopterin aldolase mRNA, complete cds
AT1G66930	-1.334131265	4.22E-06	Arabidopsis thaliana protein kinase family protein mRNA, complete cds
AT1G02575	-4.37686425	4.38E-06	Arabidopsis thaliana uncharacterized protein mRNA, complete cds
AT3G59130	-4.37686425	4.38E-06	Arabidopsis thaliana Cysteine/Histidine-rich C1 domain family protein mRNA, complete cds
AT5G21050	-4.37686425	4.38E-06	Arabidopsis thaliana chromosome 5 sequence
AT4G02180	-2.347116907	4.57E-06	Arabidopsis thaliana DC1 domain-containing protein mRNA, complete cds
AT1G18075	-2.347116907	4.57E-06	Arabidopsis thaliana chromosome 1 sequence
AT5G28650	-1.86906961	4.68E-06	Arabidopsis thaliana WRKY transcription factor 74 mRNA, complete cds
AT5G40730	-1.264654747	4.70E-06	Arabidopsis thaliana chromosome 5 sequence
AT2G46110	-1.08030581	4.83E-06	Arabidopsis thaliana ketopantoate hydroxymethyltransferase 1 mRNA, complete cds
AT1G68110	-1.00839357	4.84E-06	Arabidopsis thaliana chromosome 1 sequence
AT5G55170	-1.64751184	4.87E-06	Arabidopsis thaliana small ubiquitin-related modifier 3 mRNA, complete cds
AT2G10931	-1.64751184	4.87E-06	Arabidopsis thaliana chromosome 2, complete sequence
AT4G26990	-1.021383596	5.04E-06	Arabidopsis thaliana uncharacterized protein mRNA, complete cds

AT3G58865.1	-1.984546828	5.06E-06	Arabidopsis thaliana chromosome 3, complete sequence
AT3G44765	-2.054936155	5.13E-06	Arabidopsis thaliana Full-length cDNA Complete sequence from clone GSLTPGH55ZA10 of Hormone Treated Callus of strain col-0 of Arabidopsis thaliana (thale cress)
AT3G49950	-2.054936155	5.13E-06	Arabidopsis thaliana chromosome 3, complete sequence
AT4G16563	-1.399584327	5.24E-06	Arabidopsis thaliana chromosome 4 sequence
AT3G54710	-1.065466823	5.58E-06	Arabidopsis thaliana CDT1-like protein b mRNA, complete cds
AT1G21150	-1.240886581	5.71E-06	Arabidopsis thaliana chromosome 1 sequence
AT2G14100	-1.212352745	5.86E-06	Arabidopsis thaliana cytochrome P450, family 705, subfamily A, polypeptide 13 mRNA, complete cds
AT4G23770	-1.212352745	5.86E-06	Arabidopsis thaliana uncharacterized protein mRNA, complete cds
AT2G26520	-1.712467282	5.92E-06	Arabidopsis thaliana chromosome 2, complete sequence
AT2G44220	-2.616815043	6.02E-06	Arabidopsis thaliana uncharacterized protein mRNA, complete cds
AT1G11735	-2.616815043	6.02E-06	Arabidopsis thaliana chromosome 1 sequence
AT3G46950	-1.413390126	6.05E-06	Arabidopsis thaliana chromosome 3, complete sequence
AT3G28857	-3.100024045	6.32E-06	Arabidopsis thaliana protein PACLOBUTRAZOL RESISTANCE 5 mRNA, complete cds
AT1G76780	-1.051042239	6.43E-06	Arabidopsis thaliana chromosome 1 sequence
AT3G52670	-1.750081574	6.45E-06	Arabidopsis thaliana FBD, F-box, Skp2-like and leucine Rich Repeat domains containing protein mRNA, complete cds
AT5G56080	-1.594600309	6.70E-06	Arabidopsis thaliana chromosome 5 sequence
AT3G22570	-1.247581233	6.71E-06	Arabidopsis thaliana chromosome 3, complete sequence
AT1G67620	-1.084082501	6.79E-06	Arabidopsis thaliana Lojap-related protein mRNA, complete cds
AT1G25360	-1.79190175	6.96E-06	Arabidopsis thaliana chromosome 1

			sequence
AT5G26010	-1.428153479	6.97E-06	Arabidopsis thaliana putative protein phosphatase 2C 72 mRNA, complete cds
AT5G09978	-2.443978446	7.01E-06	Arabidopsis thaliana elicitor peptide 7 mRNA, complete cds
AT3G14020	-1.122050351	7.02E-06	Arabidopsis thaliana nuclear transcription factor Y subunit A-6 mRNA, complete cds
AT5G44460	-3.508108784	7.54E-06	Arabidopsis thaliana chromosome 5 sequence
AT3G46340	-3.508108784	7.54E-06	Arabidopsis thaliana putative receptor-like protein kinase mRNA, complete cds
AT4G28900.1	-1.379406445	7.69E-06	Arabidopsis thaliana chromosome 4 sequence
AT4G13890	-1.522966742	7.98E-06	Arabidopsis thaliana serine hydroxymethyltransferase 5 mRNA, complete cds
AT3G19184	-2.194000193	8.18E-06	Arabidopsis thaliana AP2/B3-like transcriptional factor family protein mRNA, complete cds
AT3G47348	-1.104841061	8.21E-06	Arabidopsis thaliana chromosome 3, complete sequence
AT3G26050	-1.125028051	8.31E-06	Arabidopsis thaliana TPX2 (targeting protein for Xklp2) family protein mRNA, complete cds
AT5G44010	-2.100024045	8.38E-06	Arabidopsis thaliana uncharacterized protein mRNA, complete cds
AT1G56550	-1.023366076	8.45E-06	Arabidopsis thaliana rhamnogalacturonan II specific xylosyltransferase 3 mRNA, complete cds
AT1G52800	-1.392631566	8.90E-06	Arabidopsis thaliana oxidoreductase, 2OG-Fe(II) oxygenase family protein mRNA, complete cds
AT2G38070	-1.053588472	9.06E-06	Arabidopsis thaliana chromosome 2, complete sequence
AT1G17960	-1.460984872	9.19E-06	Arabidopsis thaliana threonyl-tRNA synthetase mRNA, complete cds
AT3G57670	-1.460984872	9.19E-06	Arabidopsis thaliana transcription factor NO TRANSMITTING TRACT mRNA, complete cds
AT3G62210	-1.230303242	9.55E-06	Arabidopsis thaliana putative endonuclease or glycosyl hydrolase mRNA, complete cds

AT4G37240	-1.347116907	9.68E-06	Arabidopsis thaliana chromosome 4 sequence
AT4G12620	-1.107403575	9.73E-06	Arabidopsis thaliana chromosome 4 sequence
AT2G19720	-1.024075192	1.00E-05	Arabidopsis thaliana 40S ribosomal protein S15ab mRNA, complete cds
AT5G46230	-1.406779828	1.03E-05	Arabidopsis thaliana chromosome 5 sequence
AT3G32040	-2.569509328	1.05E-05	Arabidopsis thaliana geranylgeranyl pyrophosphate synthase 12 mRNA, complete cds
AT3G45420	-1.072009669	1.11E-05	Arabidopsis thaliana chromosome 3, complete sequence
AT3G11120	-1.072009669	1.11E-05	Arabidopsis thaliana 60S ribosomal protein L41 mRNA, complete cds
AT3G45840	-3.043440517	1.13E-05	Arabidopsis thaliana Cysteine/Histidine-rich C1 domain family protein mRNA, complete cds
AT1G53640	-3.043440517	1.13E-05	Arabidopsis thaliana uncharacterized protein mRNA, complete cds
AT2G44340	-3.043440517	1.13E-05	Arabidopsis thaliana chromosome 2, complete sequence
AT1G04660	-3.043440517	1.13E-05	Arabidopsis thaliana chromosome 1 sequence
AT2G47010	-1.010542036	1.14E-05	Arabidopsis thaliana uncharacterized protein mRNA, complete cds
AT4G22760	-1.59598154	1.14E-05	Arabidopsis thaliana pentatricopeptide repeat-containing protein mRNA, complete cds
AT5G63540	-1.807669065	1.18E-05	Arabidopsis thaliana protein RECQ MEDIATED INSTABILITY 1 mRNA, complete cds
AT2G15040	-1.807669065	1.18E-05	Arabidopsis thaliana chromosome 2, complete sequence
AT3G12170	-1.316390391	1.21E-05	Arabidopsis thaliana chaperone DnaJ-domain containing protein mRNA, complete cds
AT4G23070	-5.154471829	1.21E-05	Arabidopsis thaliana RHOMBOID-like protein 7 mRNA, complete cds
AT5G36270	-5.154471829	1.21E-05	Arabidopsis thaliana chromosome 5 sequence
AT1G63600	-5.154471829	1.21E-05	Arabidopsis thaliana chromosome 1 sequence
AT4G08400	-5.154471829	1.21E-05	Arabidopsis thaliana chromosome 4

			sequence
AT3G28370	-1.278278031	1.27E-05	Arabidopsis thaliana uncharacterized protein mRNA, complete cds
AT4G15360	-1.917432632	1.32E-05	Arabidopsis thaliana cytochrome P450, family 705, subfamily A, polypeptide 3 mRNA, complete cds
AT2G47540	-1.984546828	1.36E-05	Arabidopsis thaliana pollen Ole e 1 allergen and extensin family protein mRNA, complete cds
AT2G25240	-1.984546828	1.36E-05	Arabidopsis thaliana serpin CCP3 mRNA, complete cds
AT1G29270	-1.112870925	1.37E-05	Arabidopsis thaliana uncharacterized protein mRNA, complete cds
AT3G27480	-3.443978446	1.38E-05	Arabidopsis thaliana cysteine/histidine-rich C1 domain-containing protein mRNA, complete cds
AT2G21880	-1.025574096	1.42E-05	Arabidopsis thaliana RAB GTPase-like protein 7A mRNA, complete cds
AT1G02630	-4.232474341	1.48E-05	Arabidopsis thaliana equilibrative nucleotide transporter 8 mRNA, complete cds
AT5G50140	-4.232474341	1.48E-05	Arabidopsis thaliana ankyrin repeat-containing protein mRNA, complete cds
AT1G55410	-4.232474341	1.48E-05	Arabidopsis thaliana chromosome 1 sequence
AT1G74150	-1.385084757	1.51E-05	Arabidopsis thaliana kelch motif-containing protein mRNA, complete cds
AT2G42260	-1.543974236	1.55E-05	Arabidopsis thaliana mRNA for hypothetical protein, complete cds, clone: RAFL14-42-A06
AT3G09790	-1.731780757	1.72E-05	Arabidopsis thaliana chromosome 3, complete sequence
AT1G70990	-1.569509328	1.74E-05	Arabidopsis thaliana chromosome 1 sequence
AT5G03960	-1.399584327	1.75E-05	Arabidopsis thaliana IQ-domain 12 protein mRNA, complete cds
AT4G30700	-1.474872454	1.80E-05	Arabidopsis thaliana chromosome 4 sequence
AT1G77210	-1.059314596	1.80E-05	Arabidopsis thaliana sugar transport protein 14 mRNA, complete cds
AT2G34000	-2.520599728	1.81E-05	Arabidopsis thaliana chromosome 2,

			complete sequence
AT1G24485	-1.775960206	1.86E-05	Arabidopsis thaliana uncharacterized protein mRNA, complete cds
AT3G21950	-1.167410885	1.93E-05	Arabidopsis thaliana methyltransferase mRNA, complete cds
AT1G18290	-2.984546828	2.03E-05	Arabidopsis thaliana chromosome 1 sequence
AT4G10370	-2.984546828	2.03E-05	Arabidopsis thaliana chromosome 4 sequence
AT4G35210	-5.072009669	2.19E-05	Arabidopsis thaliana chromosome 4 sequence
AT5G23030	-5.072009669	2.19E-05	Arabidopsis thaliana tetraspanin12 mRNA, complete cds
AT3G51230	-1.079704061	2.20E-05	Arabidopsis thaliana uncharacterized protein mRNA, complete cds
AT2G35160	-1.079704061	2.20E-05	Arabidopsis thaliana histone-lysine N-methyltransferase, H3 lysine-9 specific SUVH5 mRNA, complete cds
AT5G14350	-1.079704061	2.20E-05	Arabidopsis thaliana pentatricopeptide repeat repeat-containing protein mRNA, complete cds
AT1G70340	-1.079704061	2.20E-05	Arabidopsis thaliana uncharacterized protein mRNA, complete cds
AT3G07900	-1.948020952	2.21E-05	Arabidopsis thaliana O-fucosyltransferase-like protein mRNA, complete cds
AT3G48550	-1.948020952	2.21E-05	Arabidopsis thaliana uncharacterized protein mRNA, complete cds
AT5G25970	-1.948020952	2.21E-05	Arabidopsis thaliana core-2/I-branching beta-1,6-N-acetylglucosaminyltransferase family protein mRNA, complete cds
AT3G27640	-1.363058451	2.22E-05	Arabidopsis thaliana transducin/WD40 repeat-like superfamily protein mRNA, complete cds
AT4G34360	-1.200864735	2.26E-05	Arabidopsis thaliana S-adenosyl-L-methionine-dependent methyltransferase-like protein mRNA, complete cds
AT1G08390	-1.200864735	2.26E-05	Arabidopsis thaliana uncharacterized protein mRNA, complete cds
AT2G29760	-1.200864735	2.26E-05	Arabidopsis thaliana RNA editing factor OTP81 mRNA, complete cds
AT1G60110	-1.432005805	2.33E-05	Arabidopsis thaliana jacalin-like lectin domain-containing protein mRNA,

			complete cds
AT3G27980	-1.517979028	2.34E-05	Arabidopsis thaliana plant invertase/pectin methylesterase inhibitor superfamily mRNA, complete cds
AT5G39120	-1.517979028	2.34E-05	Arabidopsis thaliana germin-like protein subfamily 1 member 15 mRNA, complete cds
AT5G08360	-1.662618733	2.43E-05	Arabidopsis thaliana uncharacterized protein mRNA, complete cds
AT1G11340	-1.662618733	2.43E-05	Arabidopsis thaliana G-type lectin S-receptor-like serine/threonine-protein kinase mRNA, complete cds
AT5G56795	-3.37686425	2.51E-05	Arabidopsis thaliana metallothionein 1B mRNA, complete cds
AT2G01900	-1.542542281	2.65E-05	Arabidopsis thaliana DNAse I-like superfamily protein mRNA, complete cds
AT1G47440	-4.154471829	2.74E-05	Arabidopsis thaliana chromosome 1 sequence
AT2G11150.1	-4.154471829	2.74E-05	Arabidopsis thaliana chromosome 2, complete sequence
AT2G29000	-4.154471829	2.74E-05	Arabidopsis thaliana leucine-rich repeat protein kinase family protein mRNA, complete cds
AT1G50580	-1.328501229	2.78E-05	Arabidopsis thaliana chromosome 1 sequence
AT5G01330	-1.045947372	2.93E-05	Arabidopsis thaliana pyruvate decarboxylase-3 mRNA, complete cds
AT2G41780	-1.285716362	2.94E-05	Arabidopsis thaliana chromosome 2, complete sequence
AT2G23530	-1.391722209	2.98E-05	Arabidopsis thaliana zinc-finger domain of monoamine-oxidase A repressor R1 mRNA, complete cds
AT5G14020	-1.569509328	2.99E-05	Arabidopsis thaliana Endosomal targeting BRO1-like domain-containing protein mRNA, complete cds
AT3G05480	-1.469973655	3.07E-05	Arabidopsis thaliana DNA damage repair protein RAD9 mRNA, complete cds
AT5G62370	-1.213365518	3.15E-05	Arabidopsis thaliana chromosome 5 sequence
AT1G14205	-1.182486205	3.19E-05	Arabidopsis thaliana ribosomal L18p/L5e family protein mRNA, complete cds
AT5G13250	-1.340690638	3.24E-05	Arabidopsis thaliana RING finger protein mRNA, complete cds

AT1G64170	-1.599256672	3.36E-05	Arabidopsis thaliana cation/H(+) antiporter 16 mRNA, complete cds
AT2G17060	-1.599256672	3.36E-05	Arabidopsis thaliana TIR-NBS-LRR class disease resistance protein mRNA, complete cds
AT5G38550	-1.295748516	3.45E-05	Arabidopsis thaliana jacalin lectin family protein mRNA, complete cds
AT3G09925	-1.407758258	3.45E-05	Arabidopsis thaliana pollen Ole e 1 allergen and extensin family protein mRNA, complete cds
AT1G73280	-1.407758258	3.45E-05	Arabidopsis thaliana serine carboxypeptidase-like 3 mRNA, complete cds
AT1G13610	-1.910546246	3.56E-05	Arabidopsis thaliana alpha/beta-hydrolase-like protein mRNA, complete cds
AT3G11150	-2.923146283	3.64E-05	Arabidopsis thaliana 2-oxoglutarate (2OG) and Fe(II)-dependent oxygenase superfamily protein mRNA, complete cds
AT3G58100	-1.220175076	3.71E-05	Arabidopsis thaliana glucan endo-1,3-beta-glucosidase-like protein 1 mRNA, complete cds
AT4G19820	-1.632245084	3.75E-05	Arabidopsis thaliana Glycosyl hydrolase family protein with chitinase insertion domain mRNA, complete cds
AT3G53220	-1.632245084	3.75E-05	Arabidopsis thaliana thioredoxin-like 3-3 mRNA, complete cds
AT3G62890	-1.632245084	3.75E-05	Arabidopsis thaliana pentatricopeptide repeat-containing protein mRNA, complete cds
AT4G01270	-1.188080222	3.77E-05	Arabidopsis thaliana RING/U-box superfamily protein mRNA, complete cds
AT5G43040	-1.188080222	3.77E-05	Arabidopsis thaliana chromosome 5 sequence
AT4G15070	-1.132645467	3.79E-05	Arabidopsis thaliana cysteine/histidine-rich C1 domain-containing protein mRNA, complete cds
AT2G38300	-1.159044559	3.80E-05	Arabidopsis thaliana myb-like HTH transcriptional regulator-like protein mRNA, complete cds
AT4G12270	-4.984546828	3.98E-05	Arabidopsis thaliana copper amine

			oxidase family protein mRNA, complete cds
AT5G36790	-1.425119419	3.98E-05	Arabidopsis thaliana 2-phosphoglycolate phosphatase 1 mRNA, complete cds
AT4G14650	-1.425119419	3.98E-05	Arabidopsis thaliana uncharacterized protein mRNA, complete cds
AT2G15300	-1.048677165	4.13E-05	Arabidopsis thaliana leucine-rich repeat protein kinase-like protein mRNA, complete cds
AT4G20450	-1.669045002	4.16E-05	Arabidopsis thaliana putative LRR receptor-like serine/threonine-protein kinase mRNA, complete cds
AT5G07400	-1.088883487	4.36E-05	Arabidopsis thaliana forkhead-associated domainand FHA domain-containing protein mRNA, complete cds
AT1G05600	-1.088883487	4.36E-05	Arabidopsis thaliana pentatricopeptide repeat-containing protein mRNA, complete cds
AT5G48320	-1.540940176	4.54E-05	Arabidopsis thaliana chromosome 5 sequence
AT2G47360	-1.540940176	4.54E-05	Arabidopsis thaliana chromosome 2, complete sequence
AT4G29150	-1.710371864	4.58E-05	Arabidopsis thaliana protein IQ-domain 25 mRNA, complete cds
AT5G43590	-3.306474922	4.59E-05	Arabidopsis thaliana acyl transferase/acyl hydrolase/lysophospholipase-like protein mRNA, complete cds
AT3G18320	-1.050135169	4.91E-05	Arabidopsis thaliana F-box and associated interaction domain-containing protein mRNA, complete cds
AT1G24290	-1.050135169	4.91E-05	Arabidopsis thaliana chromosome 1 sequence
AT3G58520	-1.274053445	4.97E-05	Arabidopsis thaliana plant organelle RNA recognition domain-containing protein mRNA, complete cds
AT4G14050	-1.274053445	4.97E-05	Arabidopsis thaliana chromosome 4 sequence
AT1G30990	-4.072009669	5.06E-05	Arabidopsis thaliana ligand-binding bet-v-1 domain-containing protein mRNA, complete cds
AT2G37780	-4.072009669	5.06E-05	Arabidopsis thaliana chromosome 2, complete sequence
AT2G43220	-4.072009669	5.06E-05	Arabidopsis thaliana chromosome 2, complete sequence

AT3G27490	-4.072009669	5.06E-05	Arabidopsis thaliana chromosome 3, complete sequence
AT1G31460	-1.091462031	5.18E-05	Arabidopsis thaliana chromosome 1 sequence
AT5G26310	-1.091462031	5.18E-05	Arabidopsis thaliana chromosome 5 sequence
AT1G17240	-1.810517428	5.38E-05	Arabidopsis thaliana chromosome 1 sequence
AT1G72540	-1.032909849	5.65E-05	Arabidopsis thaliana putative receptor-like protein kinase mRNA, complete cds
AT2G01913	-1.032909849	5.65E-05	Arabidopsis thaliana uncharacterized protein mRNA, complete cds
AT3G30720	-1.872072098	5.72E-05	Arabidopsis thaliana qua-quine starch mRNA, complete cds
AT5G22870	-1.601218188	5.76E-05	Arabidopsis thaliana chromosome 5 sequence
AT5G20640	-2.257565322	5.85E-05	Arabidopsis thaliana protein LURP-one-related 16 mRNA, complete cds
AT5G64667	-1.943904843	5.97E-05	Arabidopsis thaliana chromosome 5 sequence
AT5G39190	-1.072009669	6.02E-05	Arabidopsis thaliana germin-like protein 2a mRNA, complete cds
AT5G43250	-2.131388216	6.08E-05	Arabidopsis thaliana chromosome 5 sequence
AT5G39000	-2.028940947	6.11E-05	Arabidopsis thaliana chromosome 5 sequence
AT3G42725	-2.028940947	6.11E-05	Arabidopsis thaliana chromosome 3, complete sequence
AT3G24780	-1.174371386	6.29E-05	Arabidopsis thaliana chromosome 3, complete sequence
AT1G02970	-1.174371386	6.29E-05	Arabidopsis thaliana Wee1-like protein kinase mRNA, complete cds
AT3G56870	-1.1450115	6.30E-05	Arabidopsis thaliana uncharacterized protein mRNA, complete cds
AT3G58850	-1.343627921	6.41E-05	Arabidopsis thaliana chromosome 3, complete sequence
AT5G59520	-1.636623524	6.43E-05	Arabidopsis thaliana zinc transporter 2 mRNA, complete cds
AT3G19210	-1.016255687	6.46E-05	Arabidopsis thaliana Rad54-like protein mRNA, complete cds
AT5G19810	-2.859015945	6.49E-05	Arabidopsis thaliana chromosome 5 sequence

AT2G26220	-2.859015945	6.49E-05	Arabidopsis thaliana chromosome 2, complete sequence
AT3G29034	-1.034015504	6.71E-05	Arabidopsis thaliana chromosome 3, complete sequence
AT3G10880	-1.417506235	6.81E-05	Arabidopsis thaliana uncharacterized protein mRNA, complete cds
AT3G29300	-4.891437423	7.26E-05	Arabidopsis thaliana chromosome 3, complete sequence
AT2G30670	-4.891437423	7.26E-05	Arabidopsis thaliana NAD(P)-binding Rossmann-fold superfamily protein mRNA, complete cds
AT5G16230	-4.891437423	7.26E-05	Arabidopsis thaliana acyl-[acyl-carrier-protein] desaturase mRNA, complete cds
AT3G56330	-1.180097637	7.44E-05	Arabidopsis thaliana N2,N2-dimethylguanosine tRNA methyltransferase mRNA, complete cds
AT1G63450	-1.721512422	7.83E-05	Arabidopsis thaliana chromosome 1 sequence
AT3G48490	-1.306474922	7.99E-05	Arabidopsis thaliana uncharacterized protein mRNA, complete cds
AT3G50570	-2.569509328	8.08E-05	Arabidopsis thaliana chromosome 3, complete sequence
AT5G40320	-3.232474341	8.37E-05	Arabidopsis thaliana chromosome 5 sequence
AT4G09110	-3.232474341	8.37E-05	Arabidopsis thaliana chromosome 4 sequence
AT1G08670	-1.773042722	8.52E-05	Arabidopsis thaliana ENTH/VHS family protein mRNA, complete cds
AT3G61920	-1.373589118	8.68E-05	Arabidopsis thaliana chromosome 3, complete sequence
AT3G04980	-1.125902677	8.80E-05	Arabidopsis thaliana chromosome 3, complete sequence
ATCG00530	-1.154471829	8.84E-05	Arabidopsis thaliana chloroplast DNA, complete genome, ecotype: Columbia
AT3G30350	-1.458478016	9.03E-05	Arabidopsis thaliana protein ROOT MERISTEM GROWTH FACTOR 4 mRNA, complete cds
ATCG00040	-1.458478016	9.03E-05	Arabidopsis thaliana chloroplast DNA, complete genome, ecotype: Columbia
AT3G48160	-1.017713692	9.14E-05	Arabidopsis thaliana E2F transcription factor-like E2FE mRNA, complete cds
AT2G15128	-1.017713692	9.14E-05	Arabidopsis thaliana chromosome 2, complete sequence

AT1G67460	-2.363058451	9.20E-05	Arabidopsis thaliana Minichromosome maintenance (MCM2/3/5) family protein mRNA, complete cds
AT3G07490	-3.984546828	9.39E-05	Arabidopsis thaliana chromosome 3, complete sequence
AT1G13510	-3.984546828	9.39E-05	Arabidopsis thaliana uncharacterized protein mRNA, complete cds
AT1G28680	-3.984546828	9.39E-05	Arabidopsis thaliana HXXXD-type acyl-transferase family protein mRNA, complete cds
AT3G45330	-2.206939249	9.85E-05	Arabidopsis thaliana chromosome 3, complete sequence
AT3G59250	-1.60345666	9.91E-05	Arabidopsis thaliana F-box/LRR-repeat protein mRNA, complete cds
AT4G22520	-1.390539187	0.000100711	Arabidopsis thaliana chromosome 4 sequence
AT2G04450	-1.159633534	0.000104771	Arabidopsis thaliana nudix hydrolase 6 mRNA, complete cds
AT5G19020	-1.332470131	0.000109181	Arabidopsis thaliana mitochondrial editing factor 18 mRNA, complete cds
AT5G16950	-1.332470131	0.000109181	Arabidopsis thaliana chromosome 5 sequence
AT2G42110	-1.037658164	0.000113115	Arabidopsis thaliana uncharacterized protein mRNA, complete cds
AT5G58280	-2.79190175	0.000115626	Arabidopsis thaliana AP2/B3-like transcriptional factor family protein mRNA, complete cds
AT5G16350	-1.508108784	0.000118095	Arabidopsis thaliana O-acyltransferase (WSD1-like) family protein mRNA, complete cds
AT5G08070	-1.199559719	0.000122934	Arabidopsis thaliana chromosome 5 sequence
AT5G49010	-1.001225569	0.000124092	Arabidopsis thaliana SLD5 component of GINS complex-like protein mRNA, complete cds
AT3G57500	-1.347116907	0.000127347	Arabidopsis thaliana chromosome 3, complete sequence
AT1G33910	-4.79190175	0.000133296	Arabidopsis thaliana P-loop containing nucleoside triphosphate hydrolases superfamily protein mRNA, complete cds
AT4G11393	-4.79190175	0.000133296	Arabidopsis thaliana Defensin-like (DEFL) family protein mRNA, complete cds

AT4G24204	-4.79190175	0.000133296	Arabidopsis thaliana RING-finger domain-containing protein mRNA, complete cds
AT3G17600	-4.79190175	0.000133296	Arabidopsis thaliana auxin-responsive protein IAA31 mRNA, complete cds
AT3G18460	-4.79190175	0.000133296	Arabidopsis thaliana PLAC8 family protein mRNA, complete cds
AT2G40205	-1.734568575	0.000134212	Arabidopsis thaliana 60S ribosomal protein L41 mRNA, complete cds
AT1G02430	-1.734568575	0.000134212	Arabidopsis thaliana ADP-ribosylation factor D1B mRNA, complete cds
AT2G25090	-1.537087851	0.000134238	Arabidopsis thaliana SNF1-related kinase mRNA, complete cds
AT4G20740	-1.060495681	0.000139103	Arabidopsis thaliana chromosome 4 sequence
AT2G36730	-1.247581233	0.000141548	Arabidopsis thaliana chromosome 2, complete sequence
AT3G50825	-1.084082501	0.000142789	Arabidopsis thaliana chromosome 3, complete sequence
AT2G37310	-1.206939249	0.000145248	Arabidopsis thaliana chromosome 2, complete sequence
AT4G35130	-1.363058451	0.000148294	Arabidopsis thaliana chromosome 4 sequence
AT1G76420	-1.569509328	0.0001518	Arabidopsis thaliana protein CUP-SHAPED COTYLEDON 3 mRNA, complete cds
AT5G22410	-3.154471829	0.000152367	Arabidopsis thaliana peroxidase 60 mRNA, complete cds
AT3G44510	-1.859015945	0.000155222	Arabidopsis thaliana alpha/beta-hydrolases family protein mRNA, complete cds
AT3G09960	-2.306474922	0.000156838	Arabidopsis thaliana calcineurin-like metallo-phosphoesterase-like protein mRNA, complete cds
AT3G49845	-1.040400062	0.000160284	Arabidopsis thaliana uncharacterized protein mRNA, complete cds
AT3G50160	-2.154471829	0.000165005	Arabidopsis thaliana uncharacterized protein mRNA, complete cds
AT2G32940	-1.06254934	0.000165526	Arabidopsis thaliana argonaute 6 mRNA, complete cds
AT5G43540	-2.035172901	0.00016641	Arabidopsis thaliana chromosome 5 sequence
AT1G01200	-1.606035204	0.000170609	Arabidopsis thaliana RAB GTPase homolog A3 mRNA, complete cds

AT1G13830	-1.380475504	0.000172364	Arabidopsis thaliana carbohydrate-binding X8 domain-containing protein mRNA, complete cds
AT3G60700	-3.891437423	0.000174322	Arabidopsis thaliana uncharacterized protein mRNA, complete cds
AT2G23410	-3.891437423	0.000174322	Arabidopsis thaliana cis-prenyltransferase mRNA, complete cds
AT4G25190	-3.891437423	0.000174322	Arabidopsis thaliana uncharacterized protein mRNA, complete cds
AT1G49940	-1.143745422	0.000174345	Arabidopsis thaliana uncharacterized protein mRNA, complete cds
AT3G52550	-1.476399924	0.000177934	Arabidopsis thaliana chromosome 3, complete sequence
AT1G45015	-1.320149859	0.000185987	Arabidopsis thaliana MD-2-related lipid recognition domain-containing protein mRNA, complete cds
AT5G19060	-1.399584327	0.000199909	Arabidopsis thaliana chromosome 5 sequence
AT1G48260	-1.223333687	0.000202529	Arabidopsis thaliana SNF1-related kinase mRNA, complete cds
AT3G18630	-1.503920987	0.000203409	Arabidopsis thaliana uracil DNA glycosylase mRNA, complete cds
AT1G35330	-2.721512422	0.000205254	Arabidopsis thaliana RING-H2 finger protein ATL34 mRNA, complete cds
AT1G80340	-2.721512422	0.000205254	Arabidopsis thaliana gibberellin 3-beta-dioxygenase 2 mRNA, complete cds
AT1G33880	-2.721512422	0.000205254	Arabidopsis thaliana avirulence-induced protein mRNA, complete cds
AT1G53035	-1.117813358	0.000205328	Arabidopsis thaliana chromosome 1 sequence
AT1G67035	-1.750081574	0.000230197	Arabidopsis thaliana uncharacterized protein mRNA, complete cds
AT5G09730	-1.280002711	0.000230295	Arabidopsis thaliana beta-xylosidase 3 mRNA, complete cds
AT5G40010	-1.232474341	0.000238985	Arabidopsis thaliana chromosome 5 sequence
AT1G61130	-2.443978446	0.000243629	Arabidopsis thaliana serine carboxypeptidase-like 32 mRNA, complete cds
AT5G19560	-2.443978446	0.000243629	Arabidopsis thaliana ROP uanine nucleotide exchange factor 10 mRNA,

			complete cds
AT4G14830	-1.122050351	0.000243942	Arabidopsis thaliana chromosome 4 sequence
AT3G12965	-1.154471829	0.000245321	Arabidopsis thaliana clone asmbl_8715 unknown mRNA sequence
AT4G01920	-1.154471829	0.000245321	Arabidopsis thaliana chromosome 4 sequence
AT3G47050	-1.891437423	0.000263395	Arabidopsis thaliana glycosyl hydrolase family protein mRNA, complete cds
AT1G26970	-2.247581233	0.000266286	Arabidopsis thaliana putative protein kinase mRNA, complete cds
AT5G59270	-2.247581233	0.000266286	Arabidopsis thaliana concanavalin A-like lectin protein kinase family protein mRNA, complete cds
AT2G28040	-1.984546828	0.000273191	Arabidopsis thaliana chromosome 2, complete sequence
AT1G11850	-2.100024045	0.000275152	Arabidopsis thaliana chromosome 1 sequence
AT2G46860	-3.072009669	0.000277065	Arabidopsis thaliana pyrophosphorylase 3 mRNA, complete cds
AT3G13840	-3.072009669	0.000277065	Arabidopsis thaliana chromosome 3, complete sequence
AT4G17160	-3.072009669	0.000277065	Arabidopsis thaliana RAB GTPase homolog B1A mRNA, complete cds
AT2G37510	-1.242344585	0.000281845	Arabidopsis thaliana RNA recognition motif-containing protein mRNA, complete cds
AT5G15760	-1.126565832	0.000289828	Arabidopsis thaliana 30S ribosomal protein 3-2 mRNA, complete cds
AT1G55940	-1.160396663	0.000290964	Arabidopsis thaliana cytochrome P450, family 708, subfamily A, polypeptide 1 mRNA, complete cds
AT3G60660	-1.609037692	0.000294439	Arabidopsis thaliana spindle and kinetochore-associated protein 1-like protein mRNA, complete cds
AT5G24960	-1.609037692	0.000294439	Arabidopsis thaliana cytochrome P450 71A14 mRNA, complete cds
AT1G55580	-1.469973655	0.000306674	Arabidopsis thaliana chromosome 1 sequence
AT3G06320	-1.046831106	0.000322577	Arabidopsis thaliana ribosomal protein L33 family protein mRNA, complete cds
AT2G32650	-3.79190175	0.000324352	Arabidopsis thaliana RmlC-like cupins superfamily protein mRNA, complete cds

AT1G15540	-3.79190175	0.000324352	Arabidopsis thaliana 2-oxoglutarate (2OG) and Fe(II)-dependent oxygenase-like protein mRNA, complete cds
AT1G76220	-3.79190175	0.000324352	Arabidopsis thaliana chromosome 1 sequence
AT4G36000	-3.79190175	0.000324352	Arabidopsis thaliana pathogenesis-related thaumatin family protein mRNA, complete cds
AT4G22630	-3.79190175	0.000324352	Arabidopsis thaliana bifunctional inhibitor/lipid-transfer protein/seed storage 2S albumin superfamily protein mRNA, complete cds
AT1G45229	-1.206939249	0.000341256	Arabidopsis thaliana uncharacterized protein mRNA, complete cds
AT2G38490	-1.49912	0.000351096	Arabidopsis thaliana chromosome 2, complete sequence
AT3G53235	-1.49912	0.000351096	Arabidopsis thaliana uncharacterized protein mRNA, complete cds
AT5G10660	-2.64751184	0.000363165	Arabidopsis thaliana chromosome 5 sequence
AT4G26560	-2.64751184	0.000363165	Arabidopsis thaliana calcineurin B-like protein 7 mRNA, complete cds
AT4G01140	-1.321581815	0.000371355	Arabidopsis thaliana chromosome 4 sequence
AT4G09990	-1.048677165	0.000384412	Arabidopsis thaliana uncharacterized protein mRNA, complete cds
AT5G45440	-1.264654747	0.000391191	Arabidopsis thaliana chromosome 5 sequence
AT4G21065	-1.215872374	0.000403485	Arabidopsis thaliana chromosome 4 sequence
AT3G53010	-2.37686425	0.000420523	Arabidopsis thaliana uncharacterized protein mRNA, complete cds
AT5G18350	-2.37686425	0.000420523	Arabidopsis thaliana TIR-NBS-LRR class disease resistance protein mRNA, complete cds
AT2G44110	-2.37686425	0.000420523	Arabidopsis thaliana MLO-like protein 15 mRNA, complete cds
AT1G76530	-1.932079408	0.000446107	Arabidopsis thaliana auxin efflux carrier family protein mRNA, complete cds
AT3G05685	-1.569509328	0.000453011	Arabidopsis thaliana cystatin-related protein mRNA, complete cds
AT5G03790	-1.569509328	0.000453011	Arabidopsis thaliana homeobox 51 mRNA, complete cds

AT5G38260	-4.569509328	0.000456151	Arabidopsis thaliana protein kinase family protein mRNA, complete cds
AT1G54940	-4.569509328	0.000456151	Arabidopsis thaliana putative UDP-glucuronate:xylan alpha-glucuronosyltransferase 4 mRNA, complete cds
AT4G21260	-4.569509328	0.000456151	Arabidopsis thaliana sulfite exporter TauE/SafE family protein mRNA, complete cds
AT3G44710	-4.569509328	0.000456151	Arabidopsis thaliana chromosome 3, complete sequence
AT3G05900	-4.569509328	0.000456151	Arabidopsis thaliana neurofilament protein-related protein mRNA, complete cds
AT3G28153.1	-1.435208237	0.000459945	Arabidopsis thaliana chromosome 3, complete sequence
AT1G53887	-2.984546828	0.000502911	Arabidopsis thaliana chromosome 1 sequence
AT5G07570	-2.984546828	0.000502911	Arabidopsis thaliana glycine/proline-rich protein mRNA, complete cds
AT5G38565	-1.61257805	0.000509366	Arabidopsis thaliana putative FBD-associated F-box protein mRNA, complete cds
AT2G20875	-1.61257805	0.000509366	Arabidopsis thaliana protein EPIDERMAL PATTERNING FACTOR 1 mRNA, complete cds
AT5G19170	-1.462594124	0.000529718	Arabidopsis thaliana uncharacterized protein mRNA, complete cds
AT5G24880	-1.291208166	0.000541022	Arabidopsis thaliana chromosome 5 sequence
AT4G22560	-1.080762143	0.000561863	Arabidopsis thaliana chromosome 4 sequence
AT3G28150	-1.236085595	0.000563217	Arabidopsis thaliana protein trichome birefringence-like 22 mRNA, complete cds
AT3G48970	-1.662618733	0.000567636	Arabidopsis thaliana Heavy metal transport/detoxification superfamily protein mRNA, complete cds
AT4G30120	-1.662618733	0.000567636	Arabidopsis thaliana putative inactive cadmium/zinc-transporting ATPase HMA3 mRNA, complete cds
AT5G55720	-1.188905326	0.000574873	Arabidopsis thaliana probable pectate lyase 21 mRNA, complete cds
AT4G31610	-1.37686425	0.00059047	Arabidopsis thaliana B3

			domain-containing protein REM1 mRNA, complete cds
AT3G06120	-1.37686425	0.00059047	Arabidopsis thaliana transcription factor MUTE mRNA, complete cds
AT1G75920	-3.684986546	0.000604614	Arabidopsis thaliana GDSL esterase/lipase EXL5 mRNA, complete cds
AT1G49390	-1.493560475	0.000607477	Arabidopsis thaliana 2-oxoglutarate (2OG) and Fe(II)-dependent oxygenase-like protein mRNA, complete cds
AT1G06475	-1.493560475	0.000607477	Arabidopsis thaliana chromosome 1 sequence
AT4G09770	-1.721512422	0.000625508	Arabidopsis thaliana TRAF-like family protein mRNA, complete cds
AT1G70080	-1.306474922	0.000635143	Arabidopsis thaliana terpenoid synthase 6 mRNA, complete cds
AT5G01420	-1.306474922	0.000635143	Arabidopsis thaliana chromosome 5 sequence
AT1G61950	-2.569509328	0.000640188	Arabidopsis thaliana calcium-dependent protein kinase 19 mRNA, complete cds
AT5G24105	-1.084082501	0.000669531	Arabidopsis thaliana mRNA for hypothetical protein, complete cds, clone: RAFL19-15-P06
AT1G64250.1	-1.79190175	0.000679452	Arabidopsis thaliana chromosome 1 sequence
AT1G16445	-1.116997124	0.000681626	Arabidopsis thaliana S-adenosyl-L-methionine-dependent methyltransferase domain-containing protein mRNA, complete cds
AT5G48290	-1.154471829	0.000686111	Arabidopsis thaliana heavy metal transport/detoxification domain-containing protein mRNA, complete cds
AT5G54020	-1.528867344	0.00069305	Arabidopsis thaliana chromosome 5 sequence
ATMG00690	-2.306474922	0.000722435	Arabidopsis thaliana mitochondrial ORF240a, ecotype Kz-9
AT3G04370	-2.306474922	0.000722435	Arabidopsis thaliana plasmodesmata-located protein 4 mRNA, complete cds
AT5G45090	-2.306474922	0.000722435	Arabidopsis thaliana protein PHLOEM PROTEIN 2-LIKE A7 mRNA, complete cds

AT5G49015	-2.306474922	0.000722435	Arabidopsis thaliana uncharacterized protein mRNA, complete cds
AT1G04778	-1.877631624	0.000724339	Arabidopsis thaliana uncharacterized protein mRNA, complete cds
AT1G35830	-1.030350517	0.000750523	Arabidopsis thaliana chromosome 1 sequence
AT1G65850	-1.984546828	0.000753041	Arabidopsis thaliana TIR-NBS-LRR class disease resistance protein mRNA, complete cds
AT4G22230	-2.122050351	0.000756187	Arabidopsis thaliana defensin-like protein 96 mRNA, complete cds
AT5G45470	-1.569509328	0.000785663	Arabidopsis thaliana chromosome 5 sequence
AT1G61390	-1.425119419	0.000794746	Arabidopsis thaliana G-type lectin S-receptor-like serine/threonine-protein kinase mRNA, complete cds
AT3G46900	-1.206939249	0.0008068	Arabidopsis thaliana chromosome 3, complete sequence
AT3G21460	-1.122050351	0.000811331	Arabidopsis thaliana chromosome 3, complete sequence
AT1G78320	-1.16142459	0.000815047	Arabidopsis thaliana glutathione S-transferase TAU 23 mRNA, complete cds
AT3G01730	-4.443978446	0.000851102	Arabidopsis thaliana chromosome 3, complete sequence
AT3G47170	-4.443978446	0.000851102	Arabidopsis thaliana HXXXD-type acyl-transferase family protein mRNA, complete cds
AT1G26100	-1.006914641	0.000859447	Arabidopsis thaliana probable transmembrane ascorbate ferric reductase 4 mRNA, complete cds
AT3G45880	-1.342098832	0.000871329	Arabidopsis thaliana 2-oxoglutarate (2OG) and Fe(II)-dependent oxygenase superfamily protein mRNA, complete cds
AT3G62460	-1.031852542	0.000895943	Arabidopsis thaliana putative endonuclease or glycosyl hydrolase mRNA, complete cds
AT1G13500	-2.891437423	0.000910834	Arabidopsis thaliana uncharacterized protein mRNA, complete cds
AT3G54870	-1.274053445	0.000923979	Arabidopsis thaliana armadillo repeat-containing kinesin-like protein 1 mRNA, complete cds
AT5G05640	-1.059834955	0.000927209	Arabidopsis thaliana chromosome 5

			sequence
AT2G04025	-1.672602821	0.000983915	Arabidopsis thaliana root meristem growth factor 3 mRNA, complete cds
AT5G11930	-1.73943433	0.001081572	Arabidopsis thaliana chromosome 5 sequence
AT1G05135	-1.289401409	0.001087586	Arabidopsis thaliana chromosome 1 sequence
AT1G09860	-2.487047168	0.001123809	Arabidopsis thaliana chromosome 1 sequence
AT3G60890	-2.487047168	0.001123809	Arabidopsis thaliana protein little zipper 2 mRNA, complete cds
AT3G51570	-2.487047168	0.001123809	Arabidopsis thaliana TIR-NBS-LRR class disease resistance protein mRNA, complete cds
AT1G53130	-2.487047168	0.001123809	Arabidopsis thaliana chromosome 1 sequence
AT5G64800	-2.487047168	0.001123809	Arabidopsis thaliana chromosome 5 sequence
AT4G20160	-3.569509328	0.001128951	Arabidopsis thaliana uncharacterized protein mRNA, complete cds
AT1G21230	-3.569509328	0.001128951	Arabidopsis thaliana wall-associated receptor kinase 5 mRNA, complete cds
AT3G46420	-3.569509328	0.001128951	Arabidopsis thaliana leucine-rich repeat protein kinase-like protein mRNA, complete cds
AT3G44770	-1.133410213	0.001149734	Arabidopsis thaliana uncharacterized protein mRNA, complete cds
AT4G04745	-1.821048095	0.00116897	Arabidopsis thaliana chromosome 4 sequence
AT1G71140	-1.386645271	0.001185426	Arabidopsis thaliana MATE efflux family protein mRNA, complete cds
AT3G01580	-1.386645271	0.001185426	Arabidopsis thaliana pentatricopeptide repeat-containing protein mRNA, complete cds
AT2G15400	-1.525115209	0.001204484	Arabidopsis thaliana mRNA for DNA-directed RNA polymerase II, third largest subunit, complete cds, clone: RAFL14-92-F19
AT2G33750	-1.923146283	0.0012349	Arabidopsis thaliana purine permease 2 mRNA, complete cds
AT2G24970	-1.923146283	0.0012349	Arabidopsis thaliana uncharacterized protein mRNA, complete cds
AT1G53330	-1.923146283	0.0012349	Arabidopsis thaliana chromosome 1 sequence

AT3G11680	-2.054936155	0.001263664	Arabidopsis thaliana Aluminum activated malate transporter family protein mRNA, complete cds
AT3G01760	-2.054936155	0.001263664	Arabidopsis thaliana Lysine histidine transporter-like 4 mRNA, complete cds
AT3G55390	-1.035172901	0.001278008	Arabidopsis thaliana uncharacterized protein mRNA, complete cds
AT1G26200	-1.306474922	0.001278456	Arabidopsis thaliana TRAM, LAG1 and CLN8 (TLC) lipid-sensing domain containing protein mRNA, complete cds
AT4G16146	-1.240886581	0.001336719	Arabidopsis thaliana cAMP-regulated phosphoprotein 19-related protein mRNA, complete cds
AT4G03610	-1.240886581	0.001336719	Arabidopsis thaliana metallo-beta-lactamase domain-containing protein mRNA, complete cds
AT5G46440	-1.186180689	0.001365101	Arabidopsis thaliana chromosome 5 sequence
AT2G43050	-1.569509328	0.001366982	Arabidopsis thaliana putative pectinesterase/ pectinerase inhibitor 16 mRNA, complete cds
AT5G23980	-1.413390126	0.001376778	Arabidopsis thaliana ferric reduction oxidase 4 mRNA, complete cds
AT3G02240	-1.413390126	0.001376778	Arabidopsis thaliana root meristem growth factor 7 mRNA, complete cds
AT1G49910	-1.009208882	0.001466041	Arabidopsis thaliana cell cycle arrest protein BUB3 mRNA, complete cds
AT3G03820	-1.068611092	0.001577664	Arabidopsis thaliana chromosome 3, complete sequence
AT5G16340	-1.443978446	0.001593068	Arabidopsis thaliana chromosome 5 sequence
AT1G44010	-4.306474922	0.001597854	Arabidopsis thaliana uncharacterized protein mRNA, complete cds
AT1G16360	-4.306474922	0.001597854	Arabidopsis thaliana ligand-effect modulator 3 family protein / CDC50 family protein mRNA, complete cds
AT1G68510	-4.306474922	0.001597854	Arabidopsis thaliana LOB domain-containing protein 42 mRNA, complete cds
AT1G26360	-4.306474922	0.001597854	Arabidopsis thaliana methyl esterase 13 mRNA, complete cds
AT5G24180	-4.306474922	0.001597854	Arabidopsis thaliana lipase class 3-related protein mRNA, complete cds

AT1G20230	-1.104841061	0.001613432	Arabidopsis thaliana chromosome 1 sequence
AT2G22290	-2.79190175	0.001645198	Arabidopsis thaliana RAB GTPase-like protein H1D mRNA, complete cds
AT1G60989	-2.79190175	0.001645198	Arabidopsis thaliana protein SCR-like 7 mRNA, complete cds
AT5G59662	-2.79190175	0.001645198	Arabidopsis thaliana chromosome 5 sequence
AT1G64220	-2.79190175	0.001645198	Arabidopsis thaliana chromosome 1 sequence
AT5G54050	-2.79190175	0.001645198	Arabidopsis thaliana cysteine/histidine-rich C1 domain-containing protein mRNA, complete cds
AT3G24540	-2.79190175	0.001645198	Arabidopsis thaliana proline-rich receptor-like protein kinase PERK3 mRNA, complete cds
AT2G46840	-2.79190175	0.001645198	Arabidopsis thaliana uncharacterized protein mRNA, complete cds
AT2G28270	-1.684986546	0.001710584	Arabidopsis thaliana chromosome 2, complete sequence
AT3G11390	-1.684986546	0.001710584	Arabidopsis thaliana chromosome 3, complete sequence
AT4G25760	-1.347116907	0.001757412	Arabidopsis thaliana chromosome 4 sequence
AT5G42600	-1.479311519	0.0018348	Arabidopsis thaliana marneral synthase mRNA, complete cds
AT4G12470	-1.269949046	0.001864713	Arabidopsis thaliana chromosome 4 sequence
AT5G27230	-1.269949046	0.001864713	Arabidopsis thaliana Frigida-like protein mRNA, complete cds
AT1G52100	-1.269949046	0.001864713	Arabidopsis thaliana jacalin-like lectin domain-containing protein mRNA, complete cds
ATMG01370	-1.762154406	0.001874293	Arabidopsis thaliana ecotype Landsberg erecta mitochondrion, complete genome
AT1G19510	-1.762154406	0.001874293	Arabidopsis thaliana protein RADIALIS-like 5 mRNA, complete cds
AT2G32660	-1.762154406	0.001874293	Arabidopsis thaliana chromosome 2, complete sequence
AT5G12920	-1.072009669	0.001884623	Arabidopsis thaliana WD40 domain-containing protein mRNA, complete cds
AT2G37950	-1.072009669	0.001884623	Arabidopsis thaliana RING/FYVE/PHD

			zinc finger-containing protein mRNA, complete cds
AT5G25500	-1.206939249	0.001922733	Arabidopsis thaliana chromosome 5 sequence
AT5G41300	-1.154471829	0.001940047	Arabidopsis thaliana cysteine-rich repeat secretory protein 59 mRNA, complete cds
AT1G53690	-2.399584327	0.001963435	Arabidopsis thaliana uncharacterized protein mRNA, complete cds
AT1G48500	-1.371569951	0.002053464	Arabidopsis thaliana protein TIFY 6A mRNA, complete cds
AT5G20480	-1.011019039	0.002097043	Arabidopsis thaliana LRR receptor-like serine/threonine-protein kinase EFR mRNA, complete cds
AT5G43230	-2.154471829	0.002097913	Arabidopsis thaliana uncharacterized protein mRNA, complete cds
AT5G14340	-1.984546828	0.002098539	Arabidopsis thaliana myb domain protein 40 mRNA, complete cds
AT1G11112	-1.984546828	0.002098539	Arabidopsis thaliana chromosome 1 sequence
AT1G28040	-1.984546828	0.002098539	Arabidopsis thaliana RING-H2 finger protein ATL20 mRNA, complete cds
AT1G62090	-3.443978446	0.002111174	Arabidopsis thaliana chromosome 1 sequence
AT2G01200	-3.443978446	0.002111174	Arabidopsis thaliana auxin-responsive protein IAA32 mRNA, complete cds
AT5G18404	-3.443978446	0.002111174	Arabidopsis thaliana chromosome 5 sequence
AT4G11020	-3.443978446	0.002111174	Arabidopsis thaliana uncharacterized protein mRNA, complete cds
AT3G05415.1	-1.041130356	0.002183149	Arabidopsis thaliana chromosome 3, complete sequence
AT4G09012	-1.287109598	0.002198763	Arabidopsis thaliana mitochondrial ribosomal protein L27 mRNA, complete cds
AT3G28960	-1.219012081	0.002280432	Arabidopsis thaliana transmembrane amino acid transporter family protein mRNA, complete cds
AT4G17760	-1.162884069	0.002309571	Arabidopsis thaliana PCNA domain-containing protein mRNA, complete cds
AT3G62050	-1.162884069	0.002309571	Arabidopsis thaliana putative endonuclease or glycosyl hydrolase mRNA, complete cds

AT3G56550	-1.569509328	0.002387543	Arabidopsis thaliana pentatricopeptide repeat-containing protein mRNA, complete cds
AT4G14280	-1.399584327	0.002392174	Arabidopsis thaliana ARM repeat superfamily protein mRNA, complete cds
AT3G06360	-1.399584327	0.002392174	Arabidopsis thaliana chromosome 3, complete sequence
AT1G31200	-1.012027564	0.002509702	Arabidopsis thaliana protein PHLOEM protein 2-LIKE A9 mRNA, complete cds
AT3G52460	-1.012027564	0.002509702	Arabidopsis thaliana chromosome 3, complete sequence
AT3G15130	-1.306474922	0.002588867	Arabidopsis thaliana chromosome 3, complete sequence
AT5G26720	-1.043440517	0.002611844	Arabidopsis thaliana chromosome 5 sequence
AT3G45080	-1.628403017	0.002686845	Arabidopsis thaliana chromosome 3, complete sequence
AT5G55490	-1.172173831	0.002749213	Arabidopsis thaliana gamete expressed protein 1 mRNA, complete cds
AT5G38100	-1.432005805	0.002776224	Arabidopsis thaliana putative S-adenosylmethionine-dependent methyltransferase mRNA, complete cds
AT2G45350	-1.432005805	0.002776224	Arabidopsis thaliana chlororespiratory reduction 4 protein mRNA, complete cds
AT1G64930	-2.684986546	0.002961946	Arabidopsis thaliana chromosome 1 sequence
AT3G44705.1	-2.684986546	0.002961946	Arabidopsis thaliana chromosome 3, complete sequence
AT5G39480	-2.684986546	0.002961946	Arabidopsis thaliana chromosome 5 sequence
AT5G62330	-4.154471829	0.003019591	Arabidopsis thaliana uncharacterized protein mRNA, complete cds
AT5G26070	-4.154471829	0.003019591	Arabidopsis thaliana chromosome 5 sequence
AT5G45770	-4.154471829	0.003019591	Arabidopsis thaliana chromosome 5 sequence
AT4G19760	-4.154471829	0.003019591	Arabidopsis thaliana Glycosyl hydrolase family protein with chitinase insertion domain mRNA, complete cds
AT5G55770	-4.154471829	0.003019591	Arabidopsis thaliana chromosome 5 sequence
AT1G76190	-4.154471829	0.003019591	Arabidopsis thaliana chromosome 1 sequence

AT1G79770	-4.154471829	0.003019591	Arabidopsis thaliana chromosome 1 sequence
AT1G03700	-4.154471829	0.003019591	Arabidopsis thaliana uncharacterized protein mRNA, complete cds
AT1G05577	-4.154471829	0.003019591	Arabidopsis thaliana uncharacterized protein mRNA, complete cds
AT5G25750	-1.328501229	0.003042616	Arabidopsis thaliana uncharacterized protein mRNA, complete cds
AT2G18660	-1.328501229	0.003042616	Arabidopsis thaliana plant natriuretic peptide A mRNA, complete cds
AT2G28140	-1.045947372	0.003126091	Arabidopsis thaliana uncharacterized protein mRNA, complete cds
AT2G07711	-1.247581233	0.003201622	Arabidopsis thaliana chromosome 2, complete sequence
AT1G02950	-1.469973655	0.003206426	Arabidopsis thaliana glutathione S-transferase F4 mRNA, complete cds
AT5G52330	-1.469973655	0.003206426	Arabidopsis thaliana meprin and TRAF homology domain-containing protein mRNA, complete cds
AT2G37560	-1.084082501	0.003219012	Arabidopsis thaliana origin recognition complex subunit 2 mRNA, complete cds
AT1G14220	-1.084082501	0.003219012	Arabidopsis thaliana ribonuclease T2 mRNA, complete cds
AT1G26600	-1.79190175	0.003255445	Arabidopsis thaliana chromosome 1 sequence
AT3G61170	-1.79190175	0.003255445	Arabidopsis thaliana pentatricopeptide (PPR) repeat-containing protein mRNA, complete cds
AT4G05210	-1.79190175	0.003255445	Arabidopsis thaliana probable UDP-3-O-acylglucosamine N-acyltransferase 1 mRNA, complete cds
AT4G04360	-1.182486205	0.003272017	Arabidopsis thaliana uncharacterized protein mRNA, complete cds
AT4G16835	-1.128936737	0.00327231	Arabidopsis thaliana pentatricopeptide repeat-containing protein mRNA, complete cds
AT4G25750	-2.306474922	0.003411909	Arabidopsis thaliana chromosome 4 sequence
ATCG00065	-2.306474922	0.003411909	Arabidopsis thaliana ecotype XJalt rpl20-rps12 intergenic spacer, complete sequence; ribosomal protein S12 (rps12) gene, partial cds; and rps12-clpP intergenic spacer, complete sequence; chloroplast

AT2G22600	-2.306474922	0.003411909	Arabidopsis thaliana RNA-binding KH domain-containing protein mRNA, complete cds
AT2G37530	-2.306474922	0.003411909	Arabidopsis thaliana chromosome 2, complete sequence
AT1G65481	-2.306474922	0.003411909	Arabidopsis thaliana uncharacterized protein mRNA, complete cds
AT2G46495	-1.910546246	0.003461271	Arabidopsis thaliana putative RING-H2 finger protein mRNA, complete cds
AT4G18940	-2.072009669	0.003542058	Arabidopsis thaliana RNA ligase/cyclic nucleotide phosphodiesterase family protein mRNA, complete cds
AT5G23270	-2.072009669	0.003542058	Arabidopsis thaliana sugar transport protein 11 mRNA, complete cds
AT5G54570	-2.072009669	0.003542058	Arabidopsis thaliana beta glucosidase 41 mRNA, complete cds
AT1G13800	-1.353780637	0.003567658	Arabidopsis thaliana chromosome 1 sequence
AT5G54240	-1.014294171	0.003599831	Arabidopsis thaliana uncharacterized protein mRNA, complete cds
AT5G16080	-1.014294171	0.003599831	Arabidopsis thaliana chromosome 5 sequence
AT3G46810	-1.515061544	0.003680236	Arabidopsis thaliana chromosome 3, complete sequence
AT5G05900	-1.515061544	0.003680236	Arabidopsis thaliana UDP-glycosyltransferase 76C3 mRNA, complete cds
AT5G40030	-1.048677165	0.003743327	Arabidopsis thaliana protein kinase family protein mRNA, complete cds
AT4G12540	-1.048677165	0.003743327	Arabidopsis thaliana uncharacterized protein mRNA, complete cds
AT3G49740	-1.264654747	0.003788442	Arabidopsis thaliana chromosome 3, complete sequence
AT1G04840	-1.194000193	0.003893289	Arabidopsis thaliana pentatricopeptide repeat-containing protein mRNA, complete cds
AT3G61898	-1.194000193	0.003893289	Arabidopsis thaliana uncharacterized protein mRNA, complete cds
AT4G04972	-3.306474922	0.003952856	Arabidopsis thaliana uncharacterized protein mRNA, complete cds
AT1G02570	-3.306474922	0.003952856	Arabidopsis thaliana uncharacterized protein mRNA, complete cds
AT2G02620	-3.306474922	0.003952856	Arabidopsis thaliana chromosome 2, complete sequence

AT5G04390	-3.306474922	0.003952856	Arabidopsis thaliana chromosome 5 sequence
AT4G01890	-3.306474922	0.003952856	Arabidopsis thaliana glycoside hydrolase family 28 protein / polygalacturonase (pectinase) family protein mRNA, complete cds
AT1G29110	-3.306474922	0.003952856	Arabidopsis thaliana cysteine proteinases superfamily protein mRNA, complete cds
AT5G15725	-3.306474922	0.003952856	Arabidopsis thaliana uncharacterized protein mRNA, complete cds
AT5G44690	-3.306474922	0.003952856	Arabidopsis thaliana uncharacterized protein mRNA, complete cds
AT3G02832	-3.306474922	0.003952856	Arabidopsis thaliana chromosome 3, complete sequence
AT1G56630	-1.383096204	0.004171024	Arabidopsis thaliana alpha/beta-Hydrolases superfamily protein mRNA, complete cds
AT5G37210	-1.569509328	0.004189382	Arabidopsis thaliana cysteine/histidine-rich C1 domain-containing protein mRNA, complete cds
AT2G23550	-1.569509328	0.004189382	Arabidopsis thaliana methyl esterase 6 mRNA, complete cds
AT2G43690	-1.015573723	0.004314754	Arabidopsis thaliana chromosome 2, complete sequence
AT4G10600	-1.094171319	0.004607847	Arabidopsis thaliana chromosome 4 sequence
AT3G22240	-1.094171319	0.004607847	Arabidopsis thaliana uncharacterized protein mRNA, complete cds
AT2G32487	-1.206939249	0.00463089	Arabidopsis thaliana uncharacterized protein mRNA, complete cds
AT2G07811	-1.206939249	0.00463089	Arabidopsis thaliana ecotype Col-0 mitochondrion, complete genome
AT5G10880	-1.636623524	0.004716144	Arabidopsis thaliana tRNA synthetase/ligase-related protein mRNA, complete cds
AT5G51270	-1.636623524	0.004716144	Arabidopsis thaliana U-box domain-containing protein 53 mRNA, complete cds
AT2G16385	-1.636623524	0.004716144	Arabidopsis thaliana uncharacterized protein mRNA, complete cds
AT3G27120	-1.417506235	0.004857947	Arabidopsis thaliana P-loop containing nucleoside triphosphate hydrolases

			superfamily protein mRNA, complete cds
AT1G76065	-1.417506235	0.004857947	Arabidopsis thaliana LYR family of Fe/S cluster biogenesis protein mRNA, complete cds
AT1G07550	-1.721512422	0.005227541	Arabidopsis thaliana putative LRR receptor-like serine/threonine-protein kinase mRNA, complete cds
AT1G44180	-1.721512422	0.005227541	Arabidopsis thaliana peptidase M20/M25/M40 family protein mRNA, complete cds
AT5G52600	-1.306474922	0.005282699	Arabidopsis thaliana transcription factor MYB82 mRNA, complete cds
AT1G07270	-1.306474922	0.005282699	Arabidopsis thaliana cell division control protein 6 mRNA, complete cds
AT4G35390	-1.306474922	0.005282699	Arabidopsis thaliana chromosome 4 sequence
AT5G65090	-1.306474922	0.005282699	Arabidopsis thaliana protein DEFORMED ROOT HAIRS 4 mRNA, complete cds
AT1G52660	-2.569509328	0.005311458	Arabidopsis thaliana probable disease resistance protein mRNA, complete cds
AT1G29540	-2.569509328	0.005311458	Arabidopsis thaliana uncharacterized protein mRNA, complete cds
AT2G17770	-1.054936155	0.00537559	Arabidopsis thaliana bZIP transcription factor FD-like protein mRNA, complete cds
AT2G28710	-1.221586025	0.005505494	Arabidopsis thaliana C2H2-type zinc finger-containing protein mRNA, complete cds
AT1G51402	-1.221586025	0.005505494	Arabidopsis thaliana uncharacterized protein mRNA, complete cds
AT1G61450	-1.100024045	0.005516111	Arabidopsis thaliana uncharacterized protein mRNA, complete cds
AT2G23630	-1.100024045	0.005516111	Arabidopsis thaliana protein SKU5 similar 16 mRNA, complete cds
AT4G13440	-1.458478016	0.005629869	Arabidopsis thaliana chromosome 4 sequence
AT1G64910	-1.458478016	0.005629869	Arabidopsis thaliana chromosome 1 sequence
AT3G56220	-1.458478016	0.005629869	Arabidopsis thaliana transcription regulator mRNA, complete cds
AT1G48690	-1.832543734	0.005666377	Arabidopsis thaliana auxin-responsive GH3 family protein mRNA, complete

			cds
AT2G47200	-1.832543734	0.005666377	Arabidopsis thaliana chromosome 2, complete sequence
AT2G44390	-1.832543734	0.005666377	Arabidopsis thaliana cysteine/histidine-rich C1 domain-containing protein mRNA, complete cds
AT1G34540	-3.984546828	0.005746653	Arabidopsis thaliana chromosome 1 sequence
AT4G22210	-3.984546828	0.005746653	Arabidopsis thaliana clone asmbl_12628 defensin-like mRNA sequence
AT5G61412	-3.984546828	0.005746653	Arabidopsis thaliana chromosome 5 sequence
AT1G62035	-3.984546828	0.005746653	Arabidopsis thaliana chromosome 1 sequence
AT2G19150	-3.984546828	0.005746653	Arabidopsis thaliana putative pectinesterase 10 mRNA, complete cds
AT3G50710	-3.984546828	0.005746653	Arabidopsis thaliana putative FBD-associated F-box protein mRNA, complete cds
AT5G40860	-2.206939249	0.005892604	Arabidopsis thaliana uncharacterized protein mRNA, complete cds
AT1G63930	-2.206939249	0.005892604	Arabidopsis thaliana chromosome 1 sequence
AT1G23510	-2.206939249	0.005892604	Arabidopsis thaliana uncharacterized protein mRNA, complete cds
AT1G77870	-2.206939249	0.005892604	Arabidopsis thaliana membrane-anchored ubiquitin-fold protein 5 mRNA, complete cds
AT1G16420	-2.206939249	0.005892604	Arabidopsis thaliana metacaspase 8 mRNA, complete cds
AT2G33460	-2.206939249	0.005892604	Arabidopsis thaliana ROP-interactive CRIB motif-containing protein 1 mRNA, complete cds
AT5G23990	-1.984546828	0.005937981	Arabidopsis thaliana ferric reduction oxidase 5 mRNA, complete cds
AT1G66440	-1.984546828	0.005937981	Arabidopsis thaliana chromosome 1 sequence
AT2G31920	-1.01849416	0.006209771	Arabidopsis thaliana uncharacterized protein mRNA, complete cds
AT1G27110	-1.01849416	0.006209771	Arabidopsis thaliana tetratricopeptide repeat (TPR)-like superfamily protein mRNA, complete cds
AT1G61930	-1.332470131	0.006219947	Arabidopsis thaliana chromosome 1

			sequence
AT1G25460	-1.332470131	0.006219947	Arabidopsis thaliana tetraketide alpha-pyrone reductase 2-like protein mRNA, complete cds
AT4G31710	-1.058547409	0.006447127	Arabidopsis thaliana glutamate receptor 2.4 mRNA, complete cds
AT2G14920	-1.508108784	0.006481091	Arabidopsis thaliana sulfotransferase 4A mRNA, complete cds
AT2G04190	-1.508108784	0.006481091	Arabidopsis thaliana TRAF-like family protein mRNA, complete cds
AT1G80730	-1.23830342	0.006540774	Arabidopsis thaliana chromosome 1 sequence
AT4G38830	-1.165119073	0.006651764	Arabidopsis thaliana cysteine-rich receptor-like protein kinase 26 mRNA, complete cds
AT1G11740	-1.363058451	0.00730311	Arabidopsis thaliana ankyrin repeat family protein mRNA, complete cds
AT5G59305	-1.363058451	0.00730311	Arabidopsis thaliana uncharacterized protein mRNA, complete cds
AT3G04854	-1.569509328	0.007393183	Arabidopsis thaliana chromosome 3, complete sequence
AT5G39630	-3.154471829	0.00740764	Arabidopsis thaliana vesicle transport v-SNARE family protein mRNA, complete cds
AT3G09290	-3.154471829	0.00740764	Arabidopsis thaliana chromosome 3, complete sequence
AT3G18250	-3.154471829	0.00740764	Arabidopsis thaliana chromosome 3, complete sequence
AT2G07667	-3.154471829	0.00740764	Arabidopsis thaliana uncharacterized protein mRNA, complete cds
AT1G29179	-3.154471829	0.00740764	Arabidopsis thaliana chromosome 1 sequence
AT4G12890	-3.154471829	0.00740764	Arabidopsis thaliana Gamma interferon responsive lysosomal thiol (GILT) reductase family protein mRNA, complete cds
AT1G62240	-3.154471829	0.00740764	Arabidopsis thaliana chromosome 1 sequence
AT4G19750	-3.154471829	0.00740764	Arabidopsis thaliana Glycosyl hydrolase family protein with chitinase insertion domain mRNA, complete cds
AT5G45230	-3.154471829	0.00740764	Arabidopsis thaliana TIR-NBS-LRR class disease resistance protein mRNA, complete cds

AT1G16225	-3.154471829	0.00740764	Arabidopsis thaliana putative syntaxin-type t-SNARE protein mRNA, complete cds
AT1G70450	-3.154471829	0.00740764	Arabidopsis thaliana protein kinase superfamily protein mRNA, complete cds
AT5G39820	-3.154471829	0.00740764	Arabidopsis thaliana NAC domain containing protein 94 mRNA, complete cds
AT5G21140	-1.020170737	0.007456936	Arabidopsis thaliana protein EMBRYO DEFECTIVE 1379 mRNA, complete cds
AT1G18000	-1.06254934	0.00773685	Arabidopsis thaliana chromosome 1 sequence
AT5G59600	-1.06254934	0.00773685	Arabidopsis thaliana chromosome 5 sequence
AT4G24180	-1.06254934	0.00773685	Arabidopsis thaliana thaumatin-like protein 1 mRNA, complete cds
AT5G61550	-1.257565322	0.007763379	Arabidopsis thaliana U-box domain-containing protein 52 mRNA, complete cds
AT3G03650	-1.177191906	0.0079429	Arabidopsis thaliana Exostosin family protein mRNA, complete cds
AT1G58460	-1.64751184	0.008325669	Arabidopsis thaliana uncharacterized protein mRNA, complete cds
AT1G49220	-1.64751184	0.008325669	Arabidopsis thaliana chromosome 1 sequence
AT5G24240	-1.399584327	0.008542965	Arabidopsis thaliana phosphatidylinositol 3- and 4-kinase / ubiquitin family protein mRNA, complete cds
AT1G31840	-1.399584327	0.008542965	Arabidopsis thaliana pentatricopeptide (PPR) repeat-containing protein mRNA, complete cds
AT2G21010	-1.750081574	0.009200459	Arabidopsis thaliana Calcium-dependent lipid-binding (CaLB domain) family protein mRNA, complete cds
AT1G70860	-1.750081574	0.009200459	Arabidopsis thaliana SRPBCC ligand-binding domain-containing protein mRNA, complete cds
AT1G54930	-1.750081574	0.009200459	Arabidopsis thaliana GRF zinc finger / zinc knuckle protein mRNA, complete cds
AT2G37820	-1.750081574	0.009200459	Arabidopsis thaliana chromosome 2, complete sequence
AT3G08490	-1.280002711	0.009202536	Arabidopsis thaliana uncharacterized

			protein mRNA, complete cds
AT2G02580	-2.443978446	0.009478951	Arabidopsis thaliana cytochrome P450 71B9 mRNA, complete cds
AT1G58130	-2.443978446	0.009478951	Arabidopsis thaliana chromosome 1 sequence
AT1G73165	-2.443978446	0.009478951	Arabidopsis thaliana chromosome 1 sequence
AT5G42785	-2.443978446	0.009478951	Arabidopsis thaliana uncharacterized protein mRNA, complete cds
AT1G53660	-2.443978446	0.009478951	Arabidopsis thaliana probable sugar phosphate/phosphate translocator mRNA, complete cds
AT5G08150	-2.443978446	0.009478951	Arabidopsis thaliana chromosome 5 sequence
AT3G58060	-2.443978446	0.009478951	Arabidopsis thaliana putative metal tolerance protein C3 mRNA, complete cds
AT5G28010	-1.190997705	0.009483191	Arabidopsis thaliana polyketide cyclase/dehydrase and lipid transport superfamily protein mRNA, complete cds
AT4G19590	-1.443978446	0.009942566	Arabidopsis thaliana chaperone DnaJ-domain containing protein mRNA, complete cds
AT5G50760	-2.100024045	0.010105561	Arabidopsis thaliana chromosome 5 sequence
AT1G53635	-2.100024045	0.010105561	Arabidopsis thaliana chromosome 1 sequence
AT5G52471	-2.100024045	0.010105561	Arabidopsis thaliana chromosome 5 sequence
AT5G48780	-1.024075192	0.010776975	Arabidopsis thaliana TIR-NBS class disease resistance protein mRNA, complete cds
AT3G57160	-1.024075192	0.010776975	Arabidopsis thaliana uncharacterized protein mRNA, complete cds
AT3G44716	-1.024075192	0.010776975	Arabidopsis thaliana uncharacterized protein mRNA, complete cds
AT1G31430	-1.306474922	0.010888927	Arabidopsis thaliana chromosome 1 sequence
AT5G18910	-3.79190175	0.011019776	Arabidopsis thaliana protein kinase family protein mRNA, complete cds
AT1G06923	-3.79190175	0.011019776	Arabidopsis thaliana uncharacterized protein mRNA, complete cds
AT4G15320	-3.79190175	0.011019776	Arabidopsis thaliana cellulose

			synthase-like protein B6 mRNA, complete cds
AT2G19230	-3.79190175	0.011019776	Arabidopsis thaliana leucine-rich repeat transmembrane protein kinase protein mRNA, complete cds
AT1G73000	-3.79190175	0.011019776	Arabidopsis thaliana chromosome 1 sequence
AT3G59190	-3.79190175	0.011019776	Arabidopsis thaliana putative F-box protein mRNA, complete cds
AT2G14580	-3.79190175	0.011019776	Arabidopsis thaliana chromosome 2, complete sequence
AT2G31082	-3.79190175	0.011019776	Arabidopsis thaliana chromosome 2, complete sequence
AT2G02640	-3.79190175	0.011019776	Arabidopsis thaliana chromosome 2, complete sequence
AT5G04630	-3.79190175	0.011019776	Arabidopsis thaliana chromosome 5 sequence
AT5G54700	-3.79190175	0.011019776	Arabidopsis thaliana ankyrin repeat-containing protein mRNA, complete cds
AT5G57540	-3.79190175	0.011019776	Arabidopsis thaliana xyloglucan endotransglucosylase/hydrolase 13 mRNA, complete cds
AT4G14403	-3.79190175	0.011019776	Arabidopsis thaliana ecotype Sf-2 At4g14390 pseudogene, complete sequence; accelerated cell death 6 (ACD6A) gene, complete cds; and ACD6B pseudogene, complete sequence
AT4G25010	-3.79190175	0.011019776	Arabidopsis thaliana bidirectional sugar transporter SWEET14 mRNA, complete cds
AT2G27930	-1.072009669	0.011163561	Arabidopsis thaliana PLATZ transcription factor family protein mRNA, complete cds
AT3G03830	-1.206939249	0.011318715	Arabidopsis thaliana chromosome 3, complete sequence
AT3G57157	-1.206939249	0.011318715	Arabidopsis thaliana chromosome 3, complete sequence
AT1G35112.1	-1.206939249	0.011318715	Arabidopsis thaliana chromosome 1 sequence
AT4G39795	-1.131388216	0.011372683	Arabidopsis thaliana uncharacterized protein mRNA, complete cds
AT1G13620	-1.49912	0.011489496	Arabidopsis thaliana root meristem growth factor 2 mRNA, complete cds

AT5G01120	-1.49912	0.011489496	Arabidopsis thaliana uncharacterized protein mRNA, complete cds
AT4G22545	-1.338183782	0.012852216	Arabidopsis thaliana chromosome 4 sequence
AT2G26420	-1.569509328	0.013142048	Arabidopsis thaliana phosphatidylinositol-4-phosphate 5-kinase 3 mRNA, complete cds
AT2G16580	-1.569509328	0.013142048	Arabidopsis thaliana SAUR-like auxin-responsive protein mRNA, complete cds
AT3G57130	-1.569509328	0.013142048	Arabidopsis thaliana protein BLADE ON PETIOLE 1 mRNA, complete cds
AT1G31720	-1.569509328	0.013142048	Arabidopsis thaliana uncharacterized protein mRNA, complete cds
AT5G10850.1	-1.569509328	0.013142048	Arabidopsis thaliana chromosome 5 sequence
AT2G03010	-1.077656232	0.013423976	Arabidopsis thaliana uncharacterized protein mRNA, complete cds
AT5G44565	-1.225554927	0.013502464	Arabidopsis thaliana uncharacterized protein mRNA, complete cds
AT4G14640	-1.142088105	0.013640244	Arabidopsis thaliana calmodulin-like protein 8 mRNA, complete cds
AT3G16300	-2.984546828	0.013887254	Arabidopsis thaliana uncharacterized protein mRNA, complete cds
AT5G39130	-2.984546828	0.013887254	Arabidopsis thaliana germin-like protein subfamily 1 member 16 mRNA, complete cds
AT1G79860	-2.984546828	0.013887254	Arabidopsis thaliana RHO guanyl-nucleotide exchange factor 12 mRNA, complete cds
ATCG00030	-2.984546828	0.013887254	Cyanidiaceae sp. MX-AZ01 chloroplast, complete genome
AT1G12805	-2.984546828	0.013887254	Arabidopsis thaliana chromosome 1 sequence
AT4G02960.1	-2.984546828	0.013887254	Arabidopsis thaliana copia-like retrotransposon AtRE2 gene for polyprotein, complete cds, ecotype: Isenburg, chromosome: 4
AT3G54490	-2.984546828	0.013887254	Arabidopsis thaliana RNA polymerase II fifth largest subunit, E mRNA, complete cds
AT3G50120	-2.984546828	0.013887254	Arabidopsis thaliana uncharacterized protein mRNA, complete cds
AT3G48660	-1.662618733	0.014804314	Arabidopsis thaliana uncharacterized

			protein mRNA, complete cds
AT1G18410	-1.662618733	0.014804314	Arabidopsis thaliana kinesin motor domain-containing protein mRNA, complete cds
AT2G35860	-1.37686425	0.015116092	Arabidopsis thaliana fasciclin-like arabinogalactan protein 16 mRNA, complete cds
AT2G02750	-1.028940947	0.015628401	Arabidopsis thaliana chromosome 2, complete sequence
AT5G39110	-1.028940947	0.015628401	Arabidopsis thaliana germin-like protein subfamily 1 member 14 mRNA, complete cds
AT4G09920	-1.247581233	0.016094091	Arabidopsis thaliana FBD, F-box and leucine rich repeat domain-containing protein mRNA, complete cds
AT3G47840	-1.247581233	0.016094091	Arabidopsis thaliana chromosome 3, complete sequence
AT4G39110	-1.247581233	0.016094091	Arabidopsis thaliana chromosome 4 sequence
AT5G62310	-1.247581233	0.016094091	Arabidopsis thaliana putative serine/threonine protein kinase IRE mRNA, complete cds
AT4G23720	-1.247581233	0.016094091	Arabidopsis thaliana chromosome 4 sequence
AT5G28460	-1.79190175	0.016280604	Arabidopsis thaliana chromosome 5 sequence
AT3G47740	-1.79190175	0.016280604	Arabidopsis thaliana ABC transporter A family member 3 mRNA, complete cds
AT3G44800	-1.79190175	0.016280604	Arabidopsis thaliana Meprin and TRAF (MATH) homology domain-containing protein mRNA, complete cds
AT1G68800	-1.79190175	0.016280604	Arabidopsis thaliana transcription factor TCP12 mRNA, complete cds
AT1G52120	-1.79190175	0.016280604	Arabidopsis thaliana jacalin-like lectin domain-containing protein mRNA, complete cds
AT1G47590	-2.306474922	0.016817598	Arabidopsis thaliana chromosome 1 sequence
AT3G15170	-2.306474922	0.016817598	Arabidopsis thaliana protein CUP-SHAPED COTYLEDON 1 mRNA, complete cds
AT3G51560	-2.306474922	0.016817598	Arabidopsis thaliana TIR-NBS-LRR class disease resistance protein mRNA, complete cds

AT3G25950	-2.306474922	0.016817598	Arabidopsis thaliana chromosome 3, complete sequence
AT5G23160	-2.306474922	0.016817598	Arabidopsis thaliana uncharacterized protein mRNA, complete cds
AT5G54148	-2.306474922	0.016817598	Arabidopsis thaliana uncharacterized protein mRNA, complete cds
AT1G30850	-2.306474922	0.016817598	Arabidopsis thaliana chromosome 1 sequence
AT3G46770	-2.306474922	0.016817598	Arabidopsis thaliana AP2/B3-like transcriptional factor family protein mRNA, complete cds
AT5G35525	-2.306474922	0.016817598	Arabidopsis thaliana PLAC8 family protein mRNA, complete cds
AT2G23830	-2.306474922	0.016817598	Arabidopsis thaliana vesicle-associated protein 3-1 mRNA, complete cds
AT2G30660	-2.306474922	0.016817598	Arabidopsis thaliana probable 3-hydroxyisobutyryl-CoA hydrolase 3 mRNA, complete cds
AT5G55270	-2.306474922	0.016817598	Arabidopsis thaliana chromosome 5 sequence
AT1G02940	-2.306474922	0.016817598	Arabidopsis thaliana glutathione S-transferase F5 mRNA, complete cds
AT5G06165	-1.984546828	0.017190988	Arabidopsis thaliana chromosome 5 sequence
AT3G05152	-1.984546828	0.017190988	Arabidopsis thaliana Full-length cDNA Complete sequence from clone GSLTSIL76ZB06 of Silique of strain col-0 of Arabidopsis thaliana (thale cress)
AT2G07706	-1.984546828	0.017190988	Arabidopsis thaliana uncharacterized protein mRNA, complete cds
AT4G26330	-1.984546828	0.017190988	Arabidopsis thaliana protein UNFERTILIZED EMBRYO SAC 17 mRNA, complete cds
ATMG00630	-1.984546828	0.017190988	Arabidopsis thaliana ecotype Landsberg erecta mitochondrion, complete genome
AT3G50150	-1.425119419	0.017688629	Arabidopsis thaliana uncharacterized protein mRNA, complete cds
AT1G64210	-1.425119419	0.017688629	Arabidopsis thaliana putative inactive receptor-like protein kinase mRNA, complete cds
AT3G44420.2	-1.425119419	0.017688629	Arabidopsis thaliana chromosome 3, complete sequence
AT3G27510	-1.425119419	0.017688629	Arabidopsis thaliana chromosome 3, complete sequence

AT1G26780	-1.274053445	0.019158319	Arabidopsis thaliana myb-domain transcription factor LOF1 mRNA, complete cds
AT4G01925	-1.091462031	0.01945622	Arabidopsis thaliana cysteine/histidine-rich C1 domain-containing protein mRNA, complete cds
AT5G58750	-1.091462031	0.01945622	Arabidopsis thaliana chromosome 5 sequence
AT1G44890	-1.091462031	0.01945622	Arabidopsis thaliana uncharacterized protein mRNA, complete cds
AT1G50090	-1.091462031	0.01945622	Arabidopsis thaliana putative branched-chain-amino-acid aminotransferase 7 mRNA, complete cds
AT2G20480	-1.168971399	0.019637955	Arabidopsis thaliana uncharacterized protein mRNA, complete cds
AT4G14103	-1.168971399	0.019637955	Arabidopsis thaliana F-box/LRR-repeat protein mRNA, complete cds
AT5G41290	-1.168971399	0.019637955	Arabidopsis thaliana cysteine-rich repeat secretory protein 58 mRNA, complete cds
AT3G10290	-1.487047168	0.020543725	Arabidopsis thaliana nucleotide-sugar transporter-like protein mRNA, complete cds
AT5G47960	-1.487047168	0.020543725	Arabidopsis thaliana RAB GTPase homolog A4C mRNA, complete cds
AT1G47760	-1.487047168	0.020543725	Arabidopsis thaliana protein agamous-like 102 mRNA, complete cds
ATMG01090	-1.487047168	0.020543725	Arabidopsis thaliana ecotype Col-0 mitochondrion, complete genome
AT4G36105	-1.487047168	0.020543725	Arabidopsis thaliana uncharacterized protein mRNA, complete cds
AT5G06905	-3.569509328	0.021306155	Arabidopsis thaliana cytochrome P450, family 712, subfamily A, polypeptide 2 mRNA, complete cds
AT1G16230	-3.569509328	0.021306155	Arabidopsis thaliana Target SNARE coiled-coil domain protein mRNA, complete cds
AT4G19770	-3.569509328	0.021306155	Arabidopsis thaliana Glycosyl hydrolase family protein with chitinase insertion domain mRNA, complete cds
AT5G28715.1	-3.569509328	0.021306155	Arabidopsis thaliana chromosome 5 sequence
AT1G57780	-3.569509328	0.021306155	Arabidopsis thaliana

			heavy-metal-associated domain-containing protein mRNA, complete cds
AT3G12143	-3.569509328	0.021306155	Arabidopsis thaliana chromosome 3, complete sequence
ATMG01030	-3.569509328	0.021306155	Arabidopsis thaliana ecotype Landsberg erecta mitochondrion, complete genome
AT3G20610	-3.569509328	0.021306155	Arabidopsis thaliana chromosome 3, complete sequence
AT1G63590	-3.569509328	0.021306155	Arabidopsis thaliana chromosome 1 sequence
AT4G20190	-3.569509328	0.021306155	Arabidopsis thaliana chromosome 4 sequence
AT5G27910	-3.569509328	0.021306155	Arabidopsis thaliana chromosome 5 sequence
AT1G53260	-3.569509328	0.021306155	Arabidopsis thaliana uncharacterized protein mRNA, complete cds
AT3G61090	-3.569509328	0.021306155	Arabidopsis thaliana putative endonuclease or glycosyl hydrolase mRNA, complete cds
AT1G65630	-3.569509328	0.021306155	Arabidopsis thaliana putativeDegP protease 3 mRNA, complete cds
AT5G50060	-3.569509328	0.021306155	Arabidopsis thaliana plant invertase/pectin methylesterase inhibitor superfamily protein mRNA, complete cds
AT1G36640	-3.569509328	0.021306155	Arabidopsis thaliana uncharacterized protein mRNA, complete cds
AT2G03370	-3.569509328	0.021306155	Arabidopsis thaliana Glycosyltransferase family 61 protein mRNA, complete cds
AT5G24910	-1.035172901	0.022756416	Arabidopsis thaliana cytochrome P450, family 714, subfamily A, polypeptide 1 mRNA, complete cds
AT2G23960	-1.035172901	0.022756416	Arabidopsis thaliana class I glutamine amidotransferase-like superfamily protein mRNA, complete cds
AT5G59240	-1.035172901	0.022756416	Arabidopsis thaliana 40S ribosomal protein S8-2 mRNA, complete cds
AT1G43020	-1.306474922	0.022760621	Arabidopsis thaliana uncharacterized protein mRNA, complete cds
AT4G15417	-1.306474922	0.022760621	Arabidopsis thaliana protein RNAse II-like 1 mRNA, complete cds
AT1G73160	-1.306474922	0.022760621	Arabidopsis thaliana chromosome 1 sequence

AT5G10572	-1.306474922	0.022760621	Arabidopsis thaliana chromosome 5 sequence
AT2G07739	-1.306474922	0.022760621	Arabidopsis thaliana ecotype Col-0 mitochondrion, complete genome
AT5G37072	-1.100024045	0.023453055	Arabidopsis thaliana chromosome 5 sequence
AT2G04090	-1.186180689	0.023567641	Arabidopsis thaliana MATE efflux family protein mRNA, complete cds
AT5G62150	-1.186180689	0.023567641	Arabidopsis thaliana chromosome 5 sequence
AT1G78190	-1.186180689	0.023567641	Arabidopsis thaliana chromosome 1 sequence
AT2G46192	-1.186180689	0.023567641	Arabidopsis thaliana chromosome 2, complete sequence
AT2G37370	-1.569509328	0.023584856	Arabidopsis thaliana uncharacterized protein mRNA, complete cds
AT3G48400	-1.569509328	0.023584856	Arabidopsis thaliana cysteine/histidine-rich C1 domain-containing protein mRNA, complete cds
AT3G57965	-1.569509328	0.023584856	Arabidopsis thaliana chromosome 3, complete sequence
AT5G09805	-2.79190175	0.026026853	Arabidopsis thaliana chromosome 5 sequence
AT3G46150	-2.79190175	0.026026853	Arabidopsis thaliana uncharacterized protein mRNA, complete cds
AT5G36960	-2.79190175	0.026026853	Arabidopsis thaliana uncharacterized protein mRNA, complete cds
AT3G63360	-2.79190175	0.026026853	Arabidopsis thaliana defensin-like protein mRNA, complete cds
AT1G17345	-2.79190175	0.026026853	Arabidopsis thaliana chromosome 1 sequence
AT1G31983	-2.79190175	0.026026853	Arabidopsis thaliana chromosome 1 sequence
AT3G20530	-2.79190175	0.026026853	Arabidopsis thaliana protein kinase family protein mRNA, complete cds
AT4G37235	-1.684986546	0.02657245	Arabidopsis thaliana CASP-like protein mRNA, complete cds
AT1G69880	-1.684986546	0.02657245	Arabidopsis thaliana thioredoxin H8 mRNA, complete cds
AT2G28560	-1.684986546	0.02657245	Arabidopsis thaliana DNA repair protein RAD51B mRNA, complete cds
AT1G78172	-1.684986546	0.02657245	Arabidopsis thaliana uncharacterized protein mRNA, complete cds

AT3G10590	-1.684986546	0.02657245	Arabidopsis thaliana SANT DNA-binding domain-containing protein mRNA, complete cds
AT1G04150	-1.347116907	0.026957323	Arabidopsis thaliana chromosome 1 sequence
AT3G62960	-1.347116907	0.026957323	Arabidopsis thaliana chromosome 3, complete sequence
AT4G25980	-1.347116907	0.026957323	Arabidopsis thaliana peroxidase 43 mRNA, complete cds
AT5G50930	-1.038994612	0.027509234	Arabidopsis thaliana Histone superfamily protein mRNA, complete cds
AT2G36026	-1.038994612	0.027509234	Arabidopsis thaliana chromosome 2, complete sequence
AT5G53100	-1.859015945	0.028972363	Arabidopsis thaliana Rossmann-fold NAD(P)-binding domain-containing protein mRNA, complete cds
AT4G29550	-1.859015945	0.028972363	Arabidopsis thaliana uncharacterized protein mRNA, complete cds
AT2G12450.1	-1.859015945	0.028972363	Arabidopsis thaliana chromosome 2, complete sequence
AT5G61610	-1.859015945	0.028972363	Arabidopsis thaliana oleosin mRNA, complete cds
ATMG01330	-1.859015945	0.028972363	Arabidopsis thaliana ecotype Landsberg erecta mitochondrion, complete genome
AT3G01015	-1.859015945	0.028972363	Arabidopsis thaliana TPX2 (targeting protein for Xklp2) family protein mRNA, complete cds
AT1G32385	-1.859015945	0.028972363	Arabidopsis thaliana chromosome 1 sequence
AT3G50170	-1.859015945	0.028972363	Arabidopsis thaliana uncharacterized protein mRNA, complete cds
AT3G32925.1	-1.859015945	0.028972363	Arabidopsis thaliana chromosome 3, complete sequence
AT5G50175	-1.859015945	0.028972363	Arabidopsis thaliana chromosome 5 sequence
AT2G06002	-2.154471829	0.029625115	Arabidopsis thaliana chromosome 2, complete sequence
AT3G15700	-2.154471829	0.029625115	Arabidopsis thaliana P-loop containing nucleoside triphosphate hydrolases superfamily protein mRNA, complete cds
AT1G31258	-2.154471829	0.029625115	Arabidopsis thaliana uncharacterized protein mRNA, complete cds
AT5G54030	-2.154471829	0.029625115	Arabidopsis thaliana chromosome 5

			sequence
AT2G03600	-2.154471829	0.029625115	Arabidopsis thaliana ureide permease 3 mRNA, complete cds
AT2G37810	-2.154471829	0.029625115	Arabidopsis thaliana chromosome 2, complete sequence
AT1G32172	-2.154471829	0.029625115	Arabidopsis thaliana chromosome 1 sequence
AT3G61930	-2.154471829	0.029625115	Arabidopsis thaliana chromosome 3, complete sequence
AT1G30280	-1.399584327	0.031774174	Arabidopsis thaliana chaperone DnaJ-domain-containing protein mRNA, complete cds
AT1G59850	-1.043440517	0.033300442	Arabidopsis thaliana ARM repeat superfamily protein mRNA, complete cds
AT3G16610	-1.043440517	0.033300442	Arabidopsis thaliana chromosome 3, complete sequence
AT3G60090	-1.232474341	0.03391692	Arabidopsis thaliana chromosome 3, complete sequence
AT2G14610	-1.232474341	0.03391692	Arabidopsis thaliana chromosome 2, complete sequence
AT2G02061	-1.232474341	0.03391692	Arabidopsis thaliana nucleotide-diphospho-sugar transferase domain-containing protein mRNA, complete cds
AT3G27290	-1.232474341	0.03391692	Arabidopsis thaliana mRNA for hypothetical protein, complete cds, clone: RAFL09-70-M16
AT1G65480	-1.232474341	0.03391692	Arabidopsis thaliana protein FLOWERING LOCUS T mRNA, complete cds
AT5G11180	-1.232474341	0.03391692	Arabidopsis thaliana glutamate receptor 2.6 mRNA, complete cds
AT5G48010	-1.122050351	0.034172646	Arabidopsis thaliana thalianol synthase 1 mRNA, complete cds
AT3G20730	-1.122050351	0.034172646	Arabidopsis thaliana pentatricopeptide repeat-containing protein mRNA, complete cds
AT4G35200	-1.469973655	0.037160145	Arabidopsis thaliana chromosome 4 sequence
AT3G27095	-1.469973655	0.037160145	Arabidopsis thaliana chromosome 3, complete sequence
AT1G27140	-1.469973655	0.037160145	Arabidopsis thaliana glutathione S-transferase tau 14 mRNA, complete

			cds
AT3G59270	-1.048677165	0.040372769	Arabidopsis thaliana FBD domain-containing protein mRNA, complete cds
AT1G72870	-1.136549921	0.041308113	Arabidopsis thaliana TIR-NBS class of disease resistance protein mRNA, complete cds
AT1G64260	-1.136549921	0.041308113	Arabidopsis thaliana chromosome 1 sequence
AT1G57570	-3.306474922	0.041568869	Arabidopsis thaliana jacalin-like plant lectin domain-containing protein mRNA, complete cds
AT3G51642	-3.306474922	0.041568869	Arabidopsis thaliana chromosome 3, complete sequence
AT5G66340	-3.306474922	0.041568869	Arabidopsis thaliana uncharacterized protein mRNA, complete cds
AT1G28830	-3.306474922	0.041568869	Theobroma cacao Uncharacterized protein (TCM_007238) mRNA, complete cds
AT1G65485.1	-3.306474922	0.041568869	Arabidopsis thaliana chromosome 1 sequence
AT2G45130	-3.306474922	0.041568869	Arabidopsis thaliana SPX domain protein 3 mRNA, complete cds
AT1G33830	-3.306474922	0.041568869	Arabidopsis thaliana P-loop containing nucleoside triphosphate hydrolases superfamily protein mRNA, complete cds
AT1G01453	-3.306474922	0.041568869	Arabidopsis thaliana chromosome 1 sequence
AT1G28170	-3.306474922	0.041568869	Arabidopsis thaliana chromosome 1 sequence
AT3G55734	-3.306474922	0.041568869	Arabidopsis thaliana chromosome 3, complete sequence
AT3G23635	-3.306474922	0.041568869	Arabidopsis thaliana chromosome 3, complete sequence
AT4G39000	-3.306474922	0.041568869	Arabidopsis thaliana glycosyl hydrolase 9B17 mRNA, complete cds
AT3G06868	-3.306474922	0.041568869	Arabidopsis thaliana chromosome 3, complete sequence
AT2G43660	-3.306474922	0.041568869	Arabidopsis thaliana carbohydrate-binding X8 domain-containing protein mRNA, complete cds
AT1G28890	-3.306474922	0.041568869	Theobroma cacao Uncharacterized

			protein (TCM_007238) mRNA, complete cds
AT1G12340	-3.306474922	0.041568869	Arabidopsis thaliana putative protein cornichon mRNA, complete cds
AT2G35765	-3.306474922	0.041568869	Arabidopsis thaliana uncharacterized protein mRNA, complete cds
AT2G42050.1	-3.306474922	0.041568869	Arabidopsis thaliana chromosome 2, complete sequence
AT2G23347	-3.306474922	0.041568869	Arabidopsis thaliana chromosome 2, complete sequence
AT3G58420	-3.306474922	0.041568869	Arabidopsis thaliana TRAF-like family protein mRNA, complete cds
AT4G16355	-3.306474922	0.041568869	Arabidopsis thaliana chromosome 4 sequence
AT5G09700	-3.306474922	0.041568869	Arabidopsis thaliana Glycosyl hydrolase family protein mRNA, complete cds
AT1G64240.1	-3.306474922	0.041568869	Arabidopsis thaliana chromosome 1 sequence
AT5G57200	-3.306474922	0.041568869	Arabidopsis thaliana putative clathrin assembly protein mRNA, complete cds
AT5G51800	-3.306474922	0.041568869	Arabidopsis thaliana protein kinase family protein mRNA, complete cds
AT4G01830	-3.306474922	0.041568869	Arabidopsis thaliana ABC transporter B family member 5 mRNA, complete cds
AT2G28056	-3.306474922	0.041568869	Arabidopsis thaliana mRNA for unknown protein, complete cds, clone: RAFL21-01-E23
AT3G03702	-3.306474922	0.041568869	Arabidopsis thaliana mRNA for unknown protein, complete cds, clone: RAFL19-12-M17
AT5G39460	-3.306474922	0.041568869	Arabidopsis thaliana chromosome 5 sequence
AT2G36610	-3.306474922	0.041568869	Arabidopsis thaliana homeobox-leucine zipper protein ATHB-22 mRNA, complete cds
AT3G42820.1	-3.306474922	0.041568869	Arabidopsis thaliana chromosome 3, complete sequence
AT2G31480	-3.306474922	0.041568869	Arabidopsis thaliana chromosome 2, complete sequence
AT3G47760	-3.306474922	0.041568869	Arabidopsis thaliana ABC transporter A family member 5 mRNA, complete cds
AT2G31930	-3.306474922	0.041568869	Arabidopsis thaliana chromosome 2, complete sequence
AT5G61120	-1.569509328	0.042885146	Arabidopsis thaliana uncharacterized

			protein mRNA, complete cds
AT1G70885	-1.569509328	0.042885146	Arabidopsis thaliana chromosome 1 sequence
AT5G11440	-1.569509328	0.042885146	Arabidopsis thaliana CTC-interacting domain 5 mRNA, complete cds
AT2G14440	-1.721512422	0.04830065	Arabidopsis thaliana putative leucine-rich repeat receptor-like serine/threonine-protein kinase mRNA, complete cds
AT1G43010	-1.721512422	0.04830065	Arabidopsis thaliana pentatricopeptide repeat-containing protein mRNA, complete cds
AT1G51055	-1.721512422	0.04830065	Arabidopsis thaliana FBD-like domain family protein mRNA, complete cds
AT5G45120	-1.721512422	0.04830065	Arabidopsis thaliana chromosome 5 sequence
AT1G64540	-1.721512422	0.04830065	Arabidopsis thaliana putative F-box protein mRNA, complete cds
AT2G42140	-1.721512422	0.04830065	Arabidopsis thaliana chromosome 2, complete sequence
AT3G61113	-1.721512422	0.04830065	Arabidopsis thaliana ubiquitin-related modifier 1 mRNA, complete cds
AT2G18720	-1.721512422	0.04830065	Arabidopsis thaliana translation elongation factor EF1A/initiation factor IF2gamma family protein mRNA, complete cds
AT3G46260	-1.721512422	0.04830065	Arabidopsis thaliana protein kinase-like protein mRNA, complete cds
AT1G60525	-1.306474922	0.048574099	Arabidopsis thaliana Full-length cDNA Complete sequence from clone GSLTFB47ZB08 of Flowers and buds of strain col-0 of Arabidopsis thaliana (thale cress)
AT2G07776	-1.306474922	0.048574099	Arabidopsis thaliana uncharacterized protein mRNA, complete cds
AT2G07722	-2.569509328	0.048716089	Arabidopsis thaliana chromosome 2, complete sequence
AT5G44140	-2.569509328	0.048716089	Arabidopsis thaliana prohibitin 7 mRNA, complete cds
AT2G05330	-2.569509328	0.048716089	Arabidopsis thaliana BTB/POZ domain-containing protein mRNA, complete cds
AT2G33175	-2.569509328	0.048716089	Arabidopsis thaliana chromosome 2, complete sequence

AT1G28920	-2.569509328	0.048716089	Theobroma cacao Uncharacterized protein (TCM_007238) mRNA, complete cds
AT4G09480.1	-2.569509328	0.048716089	Arabidopsis thaliana chromosome 4 sequence
AT2G04852	-2.569509328	0.048716089	Arabidopsis thaliana chromosome 2, complete sequence
AT1G31990	-2.569509328	0.048716089	Arabidopsis thaliana chromosome 1 sequence
AT1G34520	-2.569509328	0.048716089	Arabidopsis thaliana MBOAT (membrane bound O-acyl transferase) family protein mRNA, complete cds
AT3G61118	-2.569509328	0.048716089	Arabidopsis thaliana Full-length cDNA Complete sequence from clone GSLTSIL92ZG07 of Silique of strain col-0 of Arabidopsis thaliana (thale cress)
AT4G08874	-2.569509328	0.048716089	Arabidopsis thaliana chromosome 4 sequence
AT2G21220	-2.569509328	0.048716089	Arabidopsis thaliana chromosome 2, complete sequence
AT5G32621.1	-2.569509328	0.048716089	Arabidopsis thaliana chromosome 5 sequence
AT4G29770	-2.569509328	0.048716089	Arabidopsis thaliana target of trans acting-siR480/255. mRNA, complete cds
AT2G38100	-2.569509328	0.048716089	Arabidopsis thaliana putative peptide/nitrate transporter mRNA, complete cds
AT1G31835	-1.054936155	0.049031703	Arabidopsis thaliana chromosome 1 sequence
AT5G61920	-1.054936155	0.049031703	Arabidopsis thaliana protein FLX-like 4 mRNA, complete cds
AT2G29590	-1.154471829	0.049979096	Arabidopsis thaliana thioesterase-like protein mRNA, complete cds
AT1G09155	-1.154471829	0.049979096	Arabidopsis thaliana phloem protein 2-B15 mRNA, complete cds
AT5G36260	-1.154471829	0.049979096	Arabidopsis thaliana aspartyl protease family protein mRNA, complete cds
AT1G55010	-1.984546828	0.05172897	Arabidopsis thaliana plant defensin 1.5 mRNA, complete cds
AT3G28360	-1.984546828	0.05172897	Arabidopsis thaliana ABC transporter B family member 16 mRNA, complete cds
AT3G20087	-1.984546828	0.05172897	Arabidopsis thaliana chromosome 3, complete sequence
AT2G19050	-1.984546828	0.05172897	Arabidopsis thaliana GDSL

			esterase/lipase mRNA, complete cds
AT2G13422	-1.984546828	0.05172897	Arabidopsis thaliana chromosome 2, complete sequence
AT3G07600	-1.984546828	0.05172897	Arabidopsis thaliana heavy-metal-associated domain-containing protein mRNA, complete cds
AT3G44120	-1.984546828	0.05172897	Arabidopsis thaliana chromosome 3, complete sequence
AT2G16960	-1.984546828	0.05172897	Arabidopsis thaliana ARM repeat superfamily protein mRNA, complete cds
AT2G26940	-1.984546828	0.05172897	Arabidopsis thaliana chromosome 2, complete sequence
AT1G48315	-1.363058451	0.057834172	Arabidopsis thaliana mRNA for hypothetical protein, partial cds, clone: RAFL24-01-H06
AT5G15845	-1.363058451	0.057834172	Arabidopsis thaliana AT5g15850/F14F8_230 mRNA sequence
AT2G05765	-1.363058451	0.057834172	Arabidopsis thaliana chromosome 2, complete sequence
AT1G58037	-1.363058451	0.057834172	Arabidopsis thaliana chromosome 1 sequence
AT1G71691	-1.363058451	0.057834172	Arabidopsis thaliana GDSL esterase/lipase mRNA, complete cds
AT5G01280	-1.363058451	0.057834172	Arabidopsis thaliana uncharacterized protein mRNA, complete cds
AT5G59390	-1.363058451	0.057834172	Arabidopsis thaliana XH/XS domain-containing protein mRNA, complete cds
AT4G04900	-1.363058451	0.057834172	Arabidopsis thaliana ROP-interactive CRIB motif-containing protein 10 mRNA, complete cds
AT2G02300	-1.363058451	0.057834172	Arabidopsis thaliana phloem protein 2-B5 mRNA, complete cds
AT3G57440	-1.363058451	0.057834172	Arabidopsis thaliana uncharacterized protein mRNA, complete cds
AT1G10000	-1.363058451	0.057834172	Arabidopsis thaliana chromosome 1 sequence
AT4G28560	-1.363058451	0.057834172	Arabidopsis thaliana chromosome 4 sequence
AT4G20362	-1.363058451	0.057834172	Arabidopsis thaliana Full-length cDNA Complete sequence from clone GSLTPGH40ZC03 of Hormone Treated

			Callus of strain col-0 of Arabidopsis thaliana (thale cress)
AT5G25045.1	-1.06254934	0.059664186	Arabidopsis thaliana chromosome 5 sequence
AT3G26130	-1.06254934	0.059664186	Arabidopsis thaliana Cellulase (glycosyl hydrolase family 5) protein mRNA, complete cds
AT3G04184	-1.06254934	0.059664186	Arabidopsis thaliana chromosome 3, complete sequence
AT4G14723	-1.177191906	0.06051736	Arabidopsis thaliana epidermal patterning factor-like protein 4 mRNA, complete cds
AT5G27220	-1.177191906	0.06051736	Arabidopsis thaliana Frigida-like protein mRNA, complete cds
AT1G12100	-1.177191906	0.06051736	Arabidopsis thaliana Bifunctional inhibitor/lipid-transfer protein/seed storage 2S albumin superfamily protein mRNA, complete cds
AT2G15130	-1.177191906	0.06051736	Arabidopsis thaliana chromosome 2, complete sequence
AT1G16440	-1.177191906	0.06051736	Arabidopsis thaliana protein root hair specific 3 mRNA, complete cds
AT3G05240	-1.443978446	0.068334617	Arabidopsis thaliana mitochondrial editing factor 19 mRNA, complete cds
AT4G14860	-1.443978446	0.068334617	Arabidopsis thaliana chromosome 4 sequence
AT3G01345	-1.443978446	0.068334617	Arabidopsis thaliana chromosome 3, complete sequence
AT4G08109.1	-1.443978446	0.068334617	Arabidopsis thaliana chromosome 4 sequence
AT2G11810	-1.443978446	0.068334617	Arabidopsis thaliana Monogalactosyldiacylglycerol synthase 3 mRNA, complete cds
AT2G10735	-1.443978446	0.068334617	Arabidopsis thaliana chromosome 2, complete sequence
AT2G07671	-1.443978446	0.068334617	Arabidopsis thaliana ecotype Col-0 mitochondrion, complete genome
AT5G01175	-1.443978446	0.068334617	Arabidopsis thaliana mRNA for hypothetical protein, complete cds, clone: RAFL21-63-L07
AT3G19320	-1.443978446	0.068334617	Arabidopsis thaliana leucine-rich repeat-containing protein mRNA, complete cds
AT5G18245	-1.072009669	0.072763896	Arabidopsis thaliana mRNA for

			hypothetical protein, complete cds, clone: RAFL19-67-L10
AT4G20770	-1.206939249	0.073310924	Arabidopsis thaliana pentatricopeptide repeat-containing protein mRNA, complete cds
AT1G13550	-1.206939249	0.073310924	Arabidopsis thaliana uncharacterized protein mRNA, complete cds
AT3G59340	-1.206939249	0.073310924	Arabidopsis thaliana uncharacterized protein mRNA, complete cds
AT3G53365	-1.206939249	0.073310924	Arabidopsis thaliana chromosome 3, complete sequence
AT2G42395	-1.206939249	0.073310924	Arabidopsis thaliana uncharacterized protein mRNA, complete cds
AT5G59330	-1.206939249	0.073310924	Arabidopsis thaliana Bifunctional inhibitor/lipid-transfer protein/seed storage 2S albumin superfamily protein mRNA, complete cds
AT3G28380	-1.206939249	0.073310924	Arabidopsis thaliana ABC transporter B family member 17 mRNA, complete cds
AT3G60470	-1.206939249	0.073310924	Arabidopsis thaliana uncharacterized protein mRNA, complete cds
ATCG00100	-1.206939249	0.073310924	Liverwort chloroplast Gly-tRNA-tcc
ATMG00070	-1.569509328	0.079507406	Arabidopsis thaliana mitochondrial genome
AT1G48820	-1.569509328	0.079507406	Arabidopsis thaliana terpene synthase/cyclase family protein mRNA, complete cds
AT2G16005	-1.569509328	0.079507406	Arabidopsis thaliana MD-2-related lipid recognition domain-containing protein mRNA, complete cds
AT1G63580	-1.569509328	0.079507406	Arabidopsis thaliana receptor-like protein kinase-related family protein mRNA, complete cds
AT1G67760	-1.569509328	0.079507406	Arabidopsis thaliana TCP-1/cpn60 chaperonin family protein mRNA, complete cds
AT5G65575	-1.247581233	0.08878418	Arabidopsis thaliana Full-length cDNA Complete sequence from clone GSLTSIL28ZH03 of Silique of strain col-0 of Arabidopsis thaliana (thale cress)
AT3G20710	-1.084082501	0.088965731	Arabidopsis thaliana chromosome 3, complete sequence
AT5G17390	-1.084082501	0.088965731	Arabidopsis thaliana Adenine nucleotide alpha hydrolases-like superfamily protein

			mRNA, complete cds
AT4G16640	-1.084082501	0.088965731	Arabidopsis thaliana chromosome 4 sequence
AT4G23320	-1.084082501	0.088965731	Arabidopsis thaliana cysteine-rich receptor-like protein kinase 24 mRNA, complete cds
AT1G04380	-1.79190175	0.08934391	Arabidopsis thaliana 1-aminocyclopropane-1-carboxylate oxidase-like protein 7 mRNA, complete cds
ATMG00640	-1.79190175	0.08934391	A.thaliana mitochondrial DNA for genes nad4L and orf25
AT4G28620	-1.79190175	0.08934391	Arabidopsis thaliana ABC transporter B family member 24 mRNA, complete cds
AT3G13404	-1.79190175	0.08934391	Arabidopsis thaliana uncharacterized protein mRNA, complete cds
AT2G20700	-1.79190175	0.08934391	Arabidopsis thaliana LORELEI-like glucosylphosphatidylinositol-anchored protein 2 mRNA, complete cds
AT5G52940	-1.79190175	0.08934391	Arabidopsis thaliana chromosome 5 sequence
AT3G20557	-1.79190175	0.08934391	Arabidopsis thaliana uncharacterized protein mRNA, complete cds
AT1G54970	-1.79190175	0.08934391	Arabidopsis thaliana proline-rich protein 1 mRNA, complete cds
AT1G65130	-1.79190175	0.08934391	Arabidopsis thaliana ubiquitin carboxyl-terminal hydrolase-related protein mRNA, complete cds
ATCG00710	-1.79190175	0.08934391	Arabidopsis thaliana chloroplast DNA, complete genome, ecotype: Columbia
AT1G26218	-1.79190175	0.08934391	Arabidopsis thaliana mRNA for hypothetical protein, complete cds, clone: RAFL25-17-G23
AT4G03292	-1.79190175	0.08934391	Arabidopsis thaliana chromosome 4 sequence
AT1G53610	-1.79190175	0.08934391	Arabidopsis thaliana uncharacterized protein mRNA, complete cds
AT1G61270	-1.79190175	0.08934391	Arabidopsis thaliana transmembrane amino acid transporter family protein mRNA, complete cds
AT2G15060.1	-1.79190175	0.08934391	Arabidopsis thaliana chromosome 2, complete sequence
AT3G29370	-1.79190175	0.08934391	Arabidopsis thaliana chromosome 3, complete sequence

AT1G75160	-2.306474922	0.090940021	Arabidopsis thaliana uncharacterized protein mRNA, complete cds
AT4G31354	-2.306474922	0.090940021	Arabidopsis thaliana chromosome 4 sequence
AT4G22050	-2.306474922	0.090940021	Arabidopsis thaliana aspartyl protease family protein mRNA, complete cds
AT3G44805	-2.306474922	0.090940021	Arabidopsis thaliana TRAF-like family protein mRNA, complete cds
AT2G14760	-2.306474922	0.090940021	Arabidopsis thaliana transcription factor bHLH84 mRNA, complete cds
AT1G75890	-2.306474922	0.090940021	Arabidopsis thaliana GDSL esterase/lipase EXL2 mRNA, complete cds
AT5G07560	-2.306474922	0.090940021	Arabidopsis thaliana glycine-rich protein 20 mRNA, complete cds
AT5G18710	-2.306474922	0.090940021	Arabidopsis thaliana uncharacterized protein mRNA, complete cds
AT1G63040	-2.306474922	0.090940021	Arabidopsis thaliana chromosome 1 sequence
AT1G64480	-2.306474922	0.090940021	Arabidopsis thaliana calcineurin B-like protein 8 mRNA, complete cds
AT5G24355	-2.306474922	0.090940021	Arabidopsis thaliana chromosome 5 sequence
AT3G59230	-2.306474922	0.090940021	Arabidopsis thaliana putative F-box/LRR-repeat protein mRNA, complete cds
AT5G58400	-2.306474922	0.090940021	Arabidopsis thaliana peroxidase mRNA, complete cds
AT3G23960	-2.306474922	0.090940021	Arabidopsis thaliana chromosome 3, complete sequence
AT5G17590	-2.306474922	0.090940021	Arabidopsis thaliana chromosome 5 sequence
AT2G34315	-2.306474922	0.090940021	Arabidopsis thaliana avirulence induced family protein mRNA, complete cds
AT1G49370	-2.306474922	0.090940021	Arabidopsis thaliana chromosome 1 sequence
AT4G33590	-2.306474922	0.090940021	Arabidopsis thaliana chromosome 4 sequence
AT5G15100	-2.306474922	0.090940021	Arabidopsis thaliana auxin transporter PIN8 mRNA, complete cds
AT5G55350	-2.306474922	0.090940021	Arabidopsis thaliana chromosome 5 sequence
AT1G80555	-2.306474922	0.090940021	Arabidopsis thaliana Isocitrate/isopropylmalate dehydrogenase

			family protein mRNA, complete cds
AT2G15120	-2.306474922	0.090940021	Arabidopsis thaliana chromosome 2, complete sequence
AT2G22821	-2.306474922	0.090940021	Arabidopsis thaliana chromosome 2, complete sequence
AT3G14380	-2.306474922	0.090940021	Arabidopsis thaliana uncharacterized protein mRNA, complete cds
AT1G59124	-2.306474922	0.090940021	Arabidopsis thaliana putative disease resistance protein mRNA, complete cds
AT5G48575	-2.306474922	0.090940021	Arabidopsis thaliana uncharacterized protein mRNA, complete cds
AT4G31690	-2.306474922	0.090940021	Arabidopsis thaliana transcriptional factor B3 family protein mRNA, complete cds
AT1G78360	-2.306474922	0.090940021	Arabidopsis thaliana glutathione S-transferase TAU 21 mRNA, complete cds
AT1G10710	-2.306474922	0.090940021	Arabidopsis thaliana poor homologous synapsis 1 mRNA, complete cds
AT2G19410	-2.306474922	0.090940021	Arabidopsis thaliana U-box domain-containing protein 34 mRNA, complete cds
AT1G70581	-1.306474922	0.107313807	Arabidopsis thaliana chromosome 1 sequence
AT5G49140	-1.306474922	0.107313807	Arabidopsis thaliana TIR-NBS-LRR class disease resistance protein mRNA, complete cds
AT1G44030	-1.306474922	0.107313807	Arabidopsis thaliana chromosome 1 sequence
AT5G67411	-1.306474922	0.107313807	Arabidopsis thaliana clone asmbl_17543 unknown mRNA sequence
AT3G08790	-1.306474922	0.107313807	Arabidopsis thaliana ENTH/VHS/GAT family protein mRNA, complete cds
AT1G63370	-1.306474922	0.107313807	Arabidopsis thaliana flavin-containing monooxygenase FMO GS-OX-like 5 mRNA, complete cds
AT5G11570	-1.100024045	0.109092786	Arabidopsis thaliana major facilitator protein mRNA, complete cds
AT5G46650	-1.100024045	0.109092786	Arabidopsis thaliana chromosome 5 sequence
AT1G49790	-1.100024045	0.109092786	Arabidopsis thaliana F-box associated ubiquitination effector family protein mRNA, complete cds
AT2G04680	-1.100024045	0.109092786	Arabidopsis thaliana chromosome 2,

			complete sequence
AT1G01590	-1.100024045	0.109092786	Arabidopsis thaliana ferric reduction oxidase 1 mRNA, complete cds
AT5G39150	-1.100024045	0.109092786	Arabidopsis thaliana germin-like protein subfamily 1 member 17 mRNA, complete cds
AT3G62499	-1.100024045	0.109092786	Arabidopsis thaliana chromosome 3, complete sequence
ATMG00160	-1.100024045	0.109092786	Raphanus sativus cytochrome c oxidase subunit II (cox2) mRNA, mitochondrial mRNA encoding mitochondrial protein, complete cds

Supplementary Data S5A Up regulated genes in fc1/Col-0 with NaCl treatment.

Gene	Log2FoldChange	pval	NT:Description
AT1G62510	1.886192859	0	Arabidopsis thaliana chromosome 1 sequence
AT5G40450	1.827819181	4.91E-292	Arabidopsis thaliana uncharacterized protein mRNA, complete cds
AT3G41979	2.926102248	2.05E-205	Erysimum belvederense internal transcribed spacer 1, partial sequence; 5.8S ribosomal RNA gene, complete sequence; and internal transcribed spacer 2, partial sequence
AT1G56660	1.603422191	2.86E-193	Arabidopsis thaliana uncharacterized protein mRNA, complete cds
AT1G73540	1.28814207	2.37E-132	Arabidopsis thaliana nudix hydrolase 21 mRNA, complete cds
AT1G14540	1.226997559	2.41E-131	Arabidopsis thaliana peroxidase 4 mRNA, complete cds
AT5G41750	1.2378803	4.93E-99	Arabidopsis thaliana TIR-NBS-LRR class disease resistance protein mRNA, complete cds
AT2G46400	1.0653284	7.38E-95	Arabidopsis thaliana putative WRKY transcription factor 46 mRNA, complete cds
AT4G19170	1.011703608	4.98E-94	Arabidopsis thaliana chromosome 4 sequence
AT4G24570	1.016130971	1.67E-79	Arabidopsis thaliana chromosome 4 sequence
AT1G02520	1.623975931	1.94E-78	Arabidopsis thaliana P-glycoprotein 11 mRNA, complete cds
AT1G30370	1.232092086	9.56E-62	Arabidopsis thaliana chromosome 1 sequence
AT5G13170	1.074280959	3.32E-58	Arabidopsis thaliana senescence-associated protein 29 mRNA, complete cds
AT1G17870	1.00744179	5.83E-55	Arabidopsis thaliana S2P-like putative metalloprotease mRNA, complete cds
AT3G28580	1.320914528	9.80E-55	Arabidopsis thaliana chromosome 3, complete sequence
AT1G73805	1.316617977	6.34E-50	Arabidopsis thaliana protein SAR Deficient 1 mRNA, complete cds
AT1G59590	1.066693546	2.22E-47	Arabidopsis thaliana chromosome 1 sequence
AT5G51190	1.052950804	9.07E-44	Arabidopsis thaliana chromosome 5 sequence
AT4G36520	1.063557269	6.74E-37	Arabidopsis thaliana chaperone DnaJ-domain containing protein mRNA, complete cds
AT4G25490	1.030812035	1.51E-34	Arabidopsis thaliana chromosome 4 sequence
AT5G52760	1.249554913	8.62E-34	Arabidopsis thaliana copper transport family protein mRNA, complete cds
AT1G74710	1.036010942	1.36E-31	Arabidopsis thaliana Isochorismate synthase

			1 mRNA, complete cds
AT4G14370	1.075539306	1.74E-31	Arabidopsis thaliana TIR-NBS-LRR class disease resistance protein mRNA, complete cds
AT5G24110	1.113276068	6.51E-31	Arabidopsis thaliana WRKY DNA-binding protein 30 mRNA, complete cds
AT4G25470	1.113520309	7.81E-31	Arabidopsis thaliana chromosome 4 sequence
AT3G47780	1.265981555	1.14E-29	Arabidopsis thaliana ABC transporter A family member 7 mRNA, complete cds
AT5G22460	1.383324355	8.71E-29	Arabidopsis thaliana esterase/lipase/thioesterase family protein mRNA, complete cds
AT2G31865	1.003313904	4.38E-28	Arabidopsis thaliana poly(ADP-ribose) glycohydrolase 2 mRNA, complete cds
AT1G18300	1.127268687	8.44E-28	Arabidopsis thaliana nudix hydrolase 4 mRNA, complete cds
AT3G13600	1.429244071	8.97E-28	Arabidopsis thaliana calmodulin-binding family protein mRNA, complete cds
AT1G66090	1.138525852	1.98E-27	Arabidopsis thaliana TIR-NBS class of disease resistance protein mRNA, complete cds
AT4G14368	1.222632232	1.45E-26	Arabidopsis thaliana regulator of chromosome condensation repeat-containing protein mRNA, complete cds
AT4G17030	1.255179462	6.26E-26	Arabidopsis thaliana expansin-like B1 mRNA, complete cds
AT4G25810	1.235082289	1.76E-25	Arabidopsis thaliana probable xyloglucan endotransglucosylase/hydrolase protein 23 mRNA, complete cds
AT2G30210	1.138977521	3.02E-21	Arabidopsis thaliana laccase 3 mRNA, complete cds
AT5G40000	1.139487062	3.63E-21	Arabidopsis thaliana chromosome 5 sequence
AT2G37430	1.029987154	1.52E-20	Arabidopsis thaliana chromosome 2, complete sequence
AT1G14480	1.003254848	8.90E-20	Arabidopsis thaliana Ankyrin repeat family protein mRNA, complete cds
AT1G30190	1.403068412	1.96E-19	Arabidopsis thaliana chromosome 1 sequence
AT4G33550	1.191661812	2.97E-19	Arabidopsis thaliana chromosome 4 sequence
AT1G34050	1.86117714	2.09E-17	Arabidopsis thaliana ankyrin repeats-containing protein mRNA, complete cds
AT1G74870	2.22865602	2.86E-17	Arabidopsis thaliana RING-finger domain-containing protein mRNA, complete cds

AT1G34060	1.413315794	3.30E-17	Arabidopsis thaliana tryptophan aminotransferase-related protein 4 mRNA, complete cds
AT5G48600	1.128834975	4.28E-17	Arabidopsis thaliana structural maintenance of chromosomes protein 4 mRNA, complete cds
AT4G37780	1.369668329	5.58E-17	Arabidopsis thaliana myb domain protein 87 mRNA, complete cds
AT4G08040	1.758476966	2.13E-16	Arabidopsis thaliana 1-aminocyclopropane-1-carboxylate synthase 11 mRNA, complete cds
AT5G41550	1.21901577	5.44E-16	Arabidopsis thaliana TIR-NBS-LRR class disease resistance protein mRNA, complete cds
AT4G08570	1.788573391	7.90E-16	Arabidopsis thaliana heavy-metal-associated domain-containing protein mRNA, complete cds
AT5G43890	1.128180786	1.07E-15	Arabidopsis thaliana chromosome 5 sequence
AT1G72910	1.74566919	1.56E-15	Arabidopsis thaliana Toll-Interleukin-Resistance domain-containing protein mRNA, complete cds
AT4G37030	1.14619386	2.11E-14	Arabidopsis thaliana uncharacterized protein mRNA, complete cds
AT3G22400	1.017977019	6.12E-14	Arabidopsis thaliana lipoxygenase 5 mRNA, complete cds
AT3G13784	1.443186323	6.45E-14	Arabidopsis thaliana beta-fructofuranosidase, insoluble isoenzyme CWINV5 mRNA, complete cds
AT4G11070	1.584214892	1.44E-13	Arabidopsis thaliana putative WRKY transcription factor 41 mRNA, complete cds
AT3G54530	3.251023833	1.85E-13	Arabidopsis thaliana uncharacterized protein mRNA, complete cds
AT3G17360	1.121402433	1.87E-13	Arabidopsis thaliana phragmoplast orienting kinesin 1 mRNA, complete cds
AT1G16150	1.046427558	7.06E-13	Arabidopsis thaliana wall associated kinase-like 4 mRNA, complete cds
AT3G46230	1.293168781	9.55E-13	Arabidopsis thaliana chromosome 3, complete sequence
AT5G64810	1.108160728	1.50E-12	Arabidopsis thaliana putative WRKY transcription factor 51 mRNA, complete cds
AT4G33720	1.015101952	3.63E-12	Arabidopsis thaliana chromosome 4 sequence
AT3G25510	1.271389005	6.83E-12	Arabidopsis thaliana putative TIR-NBS-LRR class disease resistance protein mRNA,

			complete cds
AT1G74770	1.01447778	7.64E-12	Arabidopsis thaliana zinc ion binding protein mRNA, complete cds
AT1G25175	5.393562946	2.24E-11	Arabidopsis thaliana At1g25170/F4F7_2 mRNA, complete cds
AT4G10250	3.942901537	4.47E-11	Arabidopsis thaliana chromosome 4 sequence
AT5G59720	1.322533701	4.66E-11	Arabidopsis thaliana chromosome 5 sequence
AT5G24640	1.293808699	5.73E-11	Arabidopsis thaliana chromosome 5 sequence
AT3G48650	1.484471443	9.21E-11	Arabidopsis thaliana chromosome 3, complete sequence
AT5G05250	1.07703821	1.13E-10	Arabidopsis thaliana chromosome 5 sequence
AT5G60350	1.15530987	1.43E-10	Arabidopsis thaliana uncharacterized protein mRNA, complete cds
AT5G12030	1.06323241	1.76E-10	Arabidopsis thaliana chromosome 5 sequence
AT3G23670	1.010699853	3.17E-10	Arabidopsis thaliana kinesin-like protein KIN12B mRNA, complete cds
AT1G78340	1.088752404	5.32E-10	Arabidopsis thaliana glutathione S-transferase TAU 22 mRNA, complete cds
AT2G38823	1.483469919	5.71E-10	Arabidopsis thaliana uncharacterized protein mRNA, complete cds
AT5G61460	1.332403945	6.72E-10	Arabidopsis thaliana protein SMC6B mRNA, complete cds
AT2G04070	1.607167643	9.43E-10	Arabidopsis thaliana MATE efflux family protein mRNA, complete cds
AT4G26470	1.387085382	1.80E-09	Arabidopsis thaliana putative calcium-binding protein CML21 mRNA, complete cds
AT5G55150	2.434560318	2.01E-09	Arabidopsis thaliana uncharacterized protein mRNA, complete cds
AT4G11480	1.3304583	2.83E-09	Arabidopsis thaliana putative cysteine-rich receptor-like protein kinase 32 mRNA, complete cds
AT3G46080	1.225835501	3.48E-09	Arabidopsis thaliana chromosome 3, complete sequence
AT4G28703	1.086051624	3.93E-09	Arabidopsis thaliana cupin domain-containing protein mRNA, complete cds
AT1G72240	1.131588602	1.01E-08	Arabidopsis thaliana chromosome 1 sequence
AT5G10080	1.036010942	1.01E-08	Arabidopsis thaliana aspartyl protease family protein mRNA, complete cds
AT1G12030	1.732004755	1.23E-08	Arabidopsis thaliana uncharacterized protein mRNA, complete cds
AT5G04660	1.451048441	1.38E-08	Arabidopsis thaliana chromosome 5 sequence
AT4G38070	1.185758061	1.39E-08	Arabidopsis thaliana transcription factor bHLH131 mRNA, complete cds

AT3G13090	1.269808126	1.48E-08	Arabidopsis thaliana multidrug resistance-associated protein 8 mRNA, complete cds
AT1G31290	1.208650328	2.46E-08	Arabidopsis thaliana argonaute 3 mRNA, complete cds
AT1G61460	1.179846714	2.83E-08	Arabidopsis thaliana G-type lectin S-receptor-like serine/threonine-protein kinase mRNA, complete cds
AT5G01630	1.011207551	3.68E-08	Arabidopsis thaliana protein BRCA2-like B mRNA, complete cds
AT1G71450	2.036010942	4.60E-08	Arabidopsis thaliana chromosome 1 sequence
AT2G18050	1.05462662	6.24E-08	Arabidopsis thaliana histone H1-3 mRNA, complete cds
AT3G23630	1.772976536	7.41E-08	Arabidopsis thaliana chromosome 3, complete sequence
AT4G14020	1.288777012	7.42E-08	Arabidopsis thaliana chromosome 4 sequence
AT3G09640	1.832477548	9.05E-08	Arabidopsis thaliana L-ascorbate peroxidase 2 mRNA, complete cds
AT4G38340	2.151488159	1.36E-07	Arabidopsis thaliana nodule inception protein-like protein 3 mRNA, complete cds
AT5G22520	1.045858728	1.81E-07	Arabidopsis thaliana chromosome 5 sequence
AT4G14819	1.96201036	1.99E-07	Arabidopsis thaliana chromosome 4 sequence
AT4G12490	2.063491678	2.20E-07	Arabidopsis thaliana chromosome 4 sequence
AT3G61880	1.26307985	3.78E-07	Arabidopsis thaliana cytochrome p450 78a9 mRNA, complete cds
AT4G23610	2.237644803	3.79E-07	Arabidopsis thaliana chromosome 4 sequence
AT5G11210	1.327242239	5.28E-07	Arabidopsis thaliana glutamate receptor 2.5 mRNA, complete cds
AT5G62080	1.49544256	5.89E-07	Arabidopsis thaliana protease inhibitor/seed storage/lipid transfer protein (LTP) family protein mRNA, complete cds
AT4G39770	1.170940522	6.05E-07	Arabidopsis thaliana probable trehalose-phosphate phosphatase H mRNA, complete cds
AT4G04500	1.929095738	9.04E-07	Arabidopsis thaliana cysteine-rich receptor-like protein kinase 37 mRNA, complete cds
AT4G31950	1.084920542	1.19E-06	Arabidopsis thaliana cytochrome P450, family 82, subfamily C, polypeptide 3 mRNA, complete cds
AT1G04050	1.181861808	1.27E-06	Arabidopsis thaliana histone-lysine N-methyltransferase SUVR1 mRNA, complete cds
AT5G39680	1.274170679	1.34E-06	Arabidopsis thaliana chromosome 5 sequence

AT3G01600	1.253241658	1.61E-06	Arabidopsis thaliana NAC domain containing protein 44 mRNA, complete cds
AT1G27565	5.428328365	1.67E-06	Arabidopsis thaliana uncharacterized protein mRNA, complete cds
AT1G15330	1.170660468	2.17E-06	Arabidopsis thaliana Cystathionine beta-synthase (CBS) protein mRNA, complete cds
AT2G03770	3.205935943	2.43E-06	Arabidopsis thaliana chromosome 2, complete sequence
AT2G46950	2.036010942	2.86E-06	Arabidopsis thaliana cytochrome P450, family 709, subfamily B, polypeptide 2 mRNA, complete cds
AT5G37490	1.479617593	3.21E-06	Arabidopsis thaliana chromosome 5 sequence
AT3G10986	1.387483312	3.68E-06	Arabidopsis thaliana uncharacterized protein mRNA, complete cds
AT5G26130	1.673440862	3.81E-06	Arabidopsis thaliana chromosome 5 sequence
AT1G10880	2.668279157	3.94E-06	Arabidopsis thaliana core-2/I-branching beta-1,6-N-acetylglucosaminyltransferase family protein mRNA, complete cds
AT5G08250	3.151488159	4.42E-06	Arabidopsis thaliana cytochrome P450 superfamily protein mRNA, complete cds
AT5G22750	1.048834982	4.54E-06	Arabidopsis thaliana mRNA for hypothetical protein, complete cds, clone: RAFL16-05-A16
AT2G47520	1.357939037	4.56E-06	Arabidopsis thaliana ethylene-responsive transcription factor ERF071 mRNA, complete cds
AT2G14210	1.73645066	4.85E-06	Arabidopsis thaliana protein agamous-like 44 mRNA, complete cds
AT4G38140	1.73645066	4.85E-06	Arabidopsis thaliana chromosome 4 sequence
AT2G31860	2.071634851	4.90E-06	Arabidopsis thaliana chromosome 2, complete sequence
AT1G21326	1.772976536	5.42E-06	Arabidopsis thaliana chromosome 1 sequence
AT5G61280	3.559572898	5.53E-06	Arabidopsis thaliana Remorin family protein mRNA, complete cds
AT1G56060	1.378403139	6.44E-06	Arabidopsis thaliana uncharacterized protein mRNA, complete cds
AT2G20555	1.85913318	6.60E-06	Arabidopsis thaliana chromosome 2, complete sequence
AT1G20060	1.036010942	7.52E-06	Arabidopsis thaliana ATP binding microtubule motor family protein mRNA, complete cds
AT5G66670	1.708436284	7.62E-06	Arabidopsis thaliana chromosome 5 sequence
AT4G14450	1.170312033	7.79E-06	Arabidopsis thaliana chromosome 4 sequence

AT1G61290	1.193552219	8.01E-06	Arabidopsis thaliana syntaxin-124 mRNA, complete cds
AT3G24750	1.07951258	9.08E-06	Arabidopsis thaliana uncharacterized protein mRNA, complete cds
AT5G53320	1.620973442	9.15E-06	Arabidopsis thaliana putative inactive receptor kinase mRNA, complete cds
AT1G70260	1.252328849	9.92E-06	Arabidopsis thaliana nodulin MtN21-like transporter UMAMIT36 mRNA, complete cds
AT5G02780	1.036010942	1.10E-05	Arabidopsis thaliana glutathione transferase lambda 1 mRNA, complete cds
AT5G44540	1.368586281	1.13E-05	Arabidopsis thaliana chromosome 5 sequence
AT1G33960	2.405244751	1.34E-05	Arabidopsis thaliana protein AIG1 mRNA, complete cds
AT5G05400	1.299045348	1.42E-05	Arabidopsis thaliana chromosome 5 sequence
AT5G43840	1.521437769	1.60E-05	Arabidopsis thaliana heat stress transcription factor A-6a mRNA, complete cds
AT4G14150	1.022071751	1.82E-05	Arabidopsis thaliana phragmoplast-associated kinesin-related protein 1 mRNA, complete cds
AT1G21850	2.73645066	1.84E-05	Arabidopsis thaliana SKU5 similar 8 protein mRNA, complete cds
AT1G53990	1.405244751	1.89E-05	Arabidopsis thaliana GDSL motif lipase 3 mRNA, complete cds
AT1G65310	1.16312286	1.93E-05	Arabidopsis thaliana probable xyloglucan endotransglucosylase/hydrolase protein 17 mRNA, complete cds
AT1G59865	1.036010942	1.95E-05	Arabidopsis thaliana uncharacterized protein mRNA, complete cds
AT3G21340	1.476583533	2.07E-05	Arabidopsis thaliana receptor-like protein kinase mRNA, complete cds
AT1G01680	1.476583533	2.07E-05	Arabidopsis thaliana U-box domain-containing protein 54 mRNA, complete cds
AT1G22990	1.050961283	2.09E-05	Arabidopsis thaliana heavy-metal-associated domain-containing protein mRNA, complete cds
AT5G62420	1.683709198	2.11E-05	Arabidopsis thaliana aldo/keto reductase family protein mRNA, complete cds
AT2G37800	2.036010942	2.30E-05	Arabidopsis thaliana cysteine/histidine-rich C1 domain-containing protein mRNA, complete cds
AT1G04500	2.123473783	2.37E-05	Arabidopsis thaliana CCT motif-containing protein mRNA, complete cds

ATCG00170	1.381785779	2.80E-05	Arabidopsis thaliana chloroplast DNA, complete genome, ecotype: Columbia
AT1G58889.1	1.516003883	2.84E-05	Arabidopsis thaliana DNA, retrotransposon:AtRE1, complete sequence, ecotype: Niederzenz
AT3G30214	1.148485671	3.29E-05	Arabidopsis thaliana chromosome 3, complete sequence
AT1G63030	5.036010942	3.31E-05	Arabidopsis thaliana chromosome 1 sequence
AT3G22800	1.346351062	3.46E-05	Arabidopsis thaliana chromosome 3, complete sequence
AT2G04515	2.468970349	3.71E-05	Arabidopsis thaliana chromosome 2, complete sequence
AT1G60630	1.177366791	4.12E-05	Arabidopsis thaliana mRNA for receptor kinase like protein, complete cds, clone: RAFL14-10-N14
AT5G65230	1.357939037	4.13E-05	Arabidopsis thaliana myb domain protein 53 mRNA, complete cds
AT1G05280	1.205935943	4.22E-05	Arabidopsis thaliana uncharacterized protein mRNA, complete cds
AT1G73040	1.205935943	4.22E-05	Arabidopsis thaliana Mannose-binding lectin superfamily protein mRNA, complete cds
AT3G45070	1.108160728	4.42E-05	Arabidopsis thaliana chromosome 3, complete sequence
AT1G51620	1.089122278	5.07E-05	Arabidopsis thaliana protein kinase superfamily protein mRNA, complete cds
AT1G55390	2.620973442	5.79E-05	Arabidopsis thaliana chromosome 1 sequence
AT5G47850	1.33369149	6.07E-05	Arabidopsis thaliana chromosome 5 sequence
AT4G38850	2.135546615	6.76E-05	Arabidopsis thaliana chromosome 4 sequence
AT1G47578	2.135546615	6.76E-05	Arabidopsis thaliana putative lipoyltransferase-like protein mRNA, complete cds
AT5G04730	1.345339	7.25E-05	Arabidopsis thaliana Ankyrin-repeat containing protein mRNA, complete cds
AT1G47655	1.258403363	7.46E-05	Arabidopsis thaliana chromosome 1 sequence
AT2G18470	1.480795784	7.64E-05	Arabidopsis thaliana proline-rich extensin-like receptor kinase 4 mRNA, complete cds
AT1G29650.1	1.786032689	8.42E-05	Arabidopsis thaliana chromosome 1 sequence
AT1G61400	1.165293959	8.52E-05	Arabidopsis thaliana S-locus lectin protein kinase family protein mRNA, complete cds
AT3G29020	2.843365864	8.62E-05	Arabidopsis thaliana myb domain protein 110 mRNA, complete cds

AT2G31085	1.620973442	9.14E-05	Arabidopsis thaliana chromosome 2, complete sequence
AT2G28460	1.843365864	9.31E-05	Arabidopsis thaliana chromosome 2, complete sequence
AT5G38350	1.141363942	9.93E-05	Arabidopsis thaliana TIR-NBS-LRR class disease resistance protein mRNA, complete cds
AT1G79915	1.386508189	0.000122728	Arabidopsis thaliana putative methyltransferase family protein mRNA, complete cds
AT4G30570	1.01873295	0.000124642	Arabidopsis thaliana Glucose-1-phosphate adenylyltransferase family protein mRNA, complete cds
AT5G14470	1.472110057	0.000135819	Arabidopsis thaliana GHMP kinase family protein mRNA, complete cds
AT1G06250	1.120899839	0.000139595	Arabidopsis thaliana phospholipase A1-IIalpha mRNA, complete cds
AT4G12735	1.00206361	0.000140276	Arabidopsis thaliana chromosome 4 sequence
AT1G04330	1.586208024	0.000141497	Arabidopsis thaliana chromosome 1 sequence
AT3G22690	1.586208024	0.000141497	Arabidopsis thaliana protein YELLOW SEEDLINGS 1 mRNA, complete cds
AT3G48080	1.147903822	0.000145674	Arabidopsis thaliana lipase class 3 family protein / disease resistance protein-related protein mRNA, complete cds
AT2G18100	1.075539306	0.000152249	Arabidopsis thaliana uncharacterized protein mRNA, complete cds
AT2G36307	1.075539306	0.000152249	Arabidopsis thaliana mRNA for hypothetical protein, complete cds, clone: RAFL22-71-B06
AT5G42325	1.211860777	0.000154312	Arabidopsis thaliana transcription factor IIS protein mRNA, complete cds
AT3G09925	2.49544256	0.000179504	Arabidopsis thaliana pollen Ole e 1 allergen and extensin family protein mRNA, complete cds
AT1G27420	1.357939037	0.000181909	Arabidopsis thaliana putative F-box/kelch-repeat protein mRNA, complete cds
AT1G76780	1.304499778	0.000186717	Arabidopsis thaliana chromosome 1 sequence
AT5G09876	1.660501807	0.000186933	Arabidopsis thaliana uncharacterized protein mRNA, complete cds
AT3G12955	1.660501807	0.00018693	Arabidopsis thaliana chromosome 3,

		3	complete sequence
AT4G17980	2.036010942	0.00018847 9	Arabidopsis thaliana NAC domain containing protein 71 mRNA, complete cds
AT5G24900	2.299045348	0.00019173 1	Arabidopsis thaliana mRNA for cytochrome P450 like protein, complete cds, clone: RAFL09-95-E07
AT3G30737.1	2.299045348	0.00019173 1	Arabidopsis thaliana chromosome 3, complete sequence
AT5G59940	1.373045929	0.00021697 9	Arabidopsis thaliana chromosome 5 sequence
AT3G55700	1.550584115	0.00021801 2	Arabidopsis thaliana UDP-glycosyltransferase 76F1 mRNA, complete cds
AT2G20800	1.758476966	0.00023914 9	Arabidopsis thaliana NAD(P)H dehydrogenase B4 mRNA, complete cds
AT3G26480	1.583498737	0.00025307 9	Arabidopsis thaliana transducin/WD40 domain-containing protein mRNA, complete cds
AT3G28770	1.056475044	0.00025607 4	Arabidopsis thaliana uncharacterized protein mRNA, complete cds
AT3G23610	1.056475044	0.00025607 4	Arabidopsis thaliana dual specificity protein phosphatase 1 mRNA, complete cds
AT4G23340	1.159393357	0.00025893	Arabidopsis thaliana chromosome 4 sequence
AT4G14840	1.159393357	0.00025893	Arabidopsis thaliana uncharacterized protein mRNA, complete cds
AT5G41780	3.843365864	0.00026849 6	Arabidopsis thaliana myosin heavy chain-like protein mRNA, complete cds
AT1G76960	1.277019041	0.00027236 9	Arabidopsis thaliana uncharacterized protein mRNA, complete cds
AT2G24140	1.036010942	0.00029089 7	Arabidopsis thaliana uncharacterized protein mRNA, complete cds
AT2G20150	1.893991937	0.000291118	Arabidopsis thaliana uncharacterized protein mRNA, complete cds
AT3G12910	1.620973442	0.00029208 8	Arabidopsis thaliana no apical meristem domain-containing transcriptional regulator mRNA, complete cds
AT3G46090	1.163766489	0.00031372 8	Arabidopsis thaliana chromosome 3, complete sequence
AT4G26770	2.428328365	0.00031415 9	Arabidopsis thaliana phosphatidate cytidylyltransferase mRNA, complete cds
AT3G16020	2.428328365	0.00031415 9	Arabidopsis thaliana F-box associated ubiquitination effector protein mRNA, complete cds
AT4G01525.	1.34267228	0.000321189	Arabidopsis thaliana chromosome 4 sequence

1			
AT1G54870	2.094904631	0.00032629 7	Arabidopsis thaliana glucose and ribitol dehydrogenase homolog 1 mRNA, complete cds
AT3G21720	2.237644803	0.0003286	Arabidopsis thaliana isocitrate lyase mRNA, complete cds
AT1G63410	1.016382135	0.00032888	Arabidopsis thaliana uncharacterized protein mRNA, complete cds
AT1G71520	1.168461238	0.00038016 5	Arabidopsis thaliana chromosome 1 sequence
AT5G40990	1.714082847	0.00038025 2	Arabidopsis thaliana GDSL lipase 1 mRNA, complete cds
AT1G78780	1.357939037	0.00038402 5	Arabidopsis thaliana pathogenesis-related protein mRNA, complete cds
AT5G61200	1.205935943	0.00039034 3	Arabidopsis thaliana uncharacterized protein mRNA, complete cds
AT5G52690	4.620973442	0.00039282 6	Arabidopsis thaliana copper transport family protein mRNA, complete cds
AT3G12720	1.299045348	0.00039378 5	Arabidopsis thaliana myb domain protein 67 mRNA, complete cds
AT1G80880	1.01583306	0.00039966 5	Arabidopsis thaliana pentatricopeptide repeat-containing protein mRNA, complete cds
AT5G13150	1.772976536	0.00042722 8	Arabidopsis thaliana chromosome 5 sequence
AT1G18265	1.374812855	0.000458511	Arabidopsis thaliana chromosome 1 sequence
AT1G05370	1.374812855	0.000458511	Arabidopsis thaliana Sec14p-like phosphatidylinositol transfer family protein mRNA, complete cds
AT2G03310	1.058378755	0.00045942 5	Arabidopsis thaliana uncharacterized protein mRNA, complete cds
AT4G26320	2.620973442	0.00049679 4	Arabidopsis thaliana chromosome 4 sequence
AT1G06750	2.620973442	0.00049679 4	Arabidopsis thaliana P-loop containing nucleoside triphosphate hydrolases superfamily protein mRNA, complete cds
AT4G13680	3.73645066	0.00050603 2	Arabidopsis thaliana chromosome 4 sequence
AT2G24681	3.73645066	0.00050603 2	Arabidopsis thaliana AP2/B3-like transcriptional factor family protein mRNA, complete cds
AT3G26922	1.036010942	0.00052235 9	Arabidopsis thaliana leucine rich repeat and F-box domain-containing protein mRNA, complete cds

AT2G31083	2.036010942	0.000546242	Arabidopsis thaliana chromosome 2, complete sequence
AT1G80970	1.393562946	0.000546533	Arabidopsis thaliana XH domain-containing protein mRNA, complete cds
AT1G44608	2.357939037	0.000547304	Arabidopsis thaliana uncharacterized protein mRNA, complete cds
AT1G09420	1.220435513	0.000571032	Arabidopsis thaliana glucose-6-phosphate dehydrogenase 4 mRNA, complete cds
AT3G62455.1	1.014637291	0.000590816	Arabidopsis thaliana chromosome 3, complete sequence
AT3G62990	1.668279157	0.000601079	Arabidopsis thaliana chromosome 3, complete sequence
AT1G57980	1.114013454	0.000626176	Arabidopsis thaliana Nucleotide-sugar transporter family protein mRNA, complete cds
AT3G09240	1.724066935	0.000682336	Arabidopsis thaliana Protein kinase protein with tetratricopeptide repeat domain mRNA, complete cds
AT2G03380	1.538511282	0.000701044	Arabidopsis thaliana chromosome 2, complete sequence
AT3G49630	1.538511282	0.000701044	Arabidopsis thaliana 2-oxoglutarate (2OG) and Fe(II)-dependent oxygenase superfamily protein mRNA, complete cds
AT2G31910	2.942901537	0.000735873	Arabidopsis thaliana cation/H(+) antiporter 21 mRNA, complete cds
AT1G56550	1.116930937	0.000761079	Arabidopsis thaliana rhamnogalacturonan II specific xylosyltransferase 3 mRNA, complete cds
AT2G15810.1	1.438109385	0.00077154	Arabidopsis thaliana chromosome 2, complete sequence
AT3G53150	1.576579323	0.00081638	Arabidopsis thaliana chromosome 3, complete sequence
AT1G16070	1.060672996	0.000826812	Arabidopsis thaliana Tubby-like protein 8 mRNA, complete cds
AT1G47400	1.872512209	0.000843245	Arabidopsis thaliana chromosome 1 sequence
AT2G28330	1.872512209	0.000843245	Arabidopsis thaliana chromosome 2, complete sequence
AT2G18800	1.974610397	0.000909069	Arabidopsis thaliana probable xyloglucan endotransglucosylase/hydrolase protein 21 mRNA, complete cds
AT2G12440.1	1.620973442	0.0009443	Arabidopsis thaliana chromosome 2, complete sequence
AT5G63390	1.620973442	0.0009443	Arabidopsis thaliana O-fucosyltransferase

			family protein mRNA, complete cds
AT5G36870	2.10640027	0.000949911	Arabidopsis thaliana callose synthase 4 mRNA, complete cds
ATCG00160	2.10640027	0.000949911	Arabidopsis thaliana chloroplast DNA, complete genome, ecotype: Columbia
AT5G49380	2.10640027	0.000949911	Arabidopsis thaliana uncharacterized protein mRNA, complete cds
ATCG01010	3.620973442	0.000955539	Arabidopsis thaliana chloroplast DNA, complete genome, ecotype: Columbia
AT3G09660	1.061546034	0.001006492	Arabidopsis thaliana minichromosome maintenance 8 mRNA, complete cds
AT2G17036	1.061546034	0.001006492	Arabidopsis thaliana F-box protein mRNA, complete cds
AT1G52570	1.012551969	0.001064913	Arabidopsis thaliana phospholipase D alpha 2 mRNA, complete cds
AT3G15440	1.673440862	0.001082929	Arabidopsis thaliana chromosome 3, complete sequence
AT1G56120	1.123473783	0.001125416	Arabidopsis thaliana Leucine-rich repeat transmembrane protein kinase mRNA, complete cds
AT5G16530	1.036010942	0.001146012	Arabidopsis thaliana auxin transporter PIN5 mRNA, complete cds
AT2G42485	1.036010942	0.001146012	Arabidopsis thaliana chromosome 2, complete sequence
AT3G05740	1.205935943	0.001205728	Arabidopsis thaliana ATP-dependent DNA helicase Q-like 1 mRNA, complete cds
AT1G73860	1.258403363	0.001219483	Arabidopsis thaliana putative Kinesin motor protein-related protein mRNA, complete cds
AT1G33720	1.062483153	0.001225745	Arabidopsis thaliana cytochrome P450, family 76, subfamily C, polypeptide 6 mRNA, complete cds
AT5G23600	1.73645066	0.001228166	Arabidopsis thaliana chromosome 5 sequence
AT1G34460	1.530775634	0.001263061	Arabidopsis thaliana cyclin-B1-5 mRNA, complete cds
AT5G40510	1.011763396	0.001297085	Arabidopsis thaliana Sucrase/ferredoxin-like family protein mRNA, complete cds
AT5G45970	1.011763396	0.001297085	Arabidopsis thaliana Rac-like GTP-binding protein ARAC2 mRNA, complete cds
ATCG00200	2.843365864	0.001346417	Aster spathulifolius chloroplast, complete genome
AT3G20160	1.81361852	0.00137249	Arabidopsis thaliana chromosome 3, complete sequence
AT2G15800.	1.423034065	0.00138313	Arabidopsis thaliana chromosome 2,

1		3	complete sequence
AT5G60610	1.423034065	0.001383133	Arabidopsis thaliana FBD-associated F-box protein mRNA, complete cds
AT1G33510	4.357939037	0.001402584	Arabidopsis thaliana chromosome 1 sequence
AT4G10950	4.357939037	0.001402584	Arabidopsis thaliana SGNH hydrolase-type esterase superfamily protein mRNA, complete cds
AT3G51915	4.357939037	0.001402584	Arabidopsis thaliana uncharacterized protein mRNA, complete cds
AT1G09880	1.167255475	0.001424632	Arabidopsis thaliana rhamnogalacturonate lyase-like protein mRNA, complete cds
AT3G14740	1.167255475	0.001424632	Arabidopsis thaliana RING/FYVE/PHD zinc finger-containing protein mRNA, complete cds
AT5G01660	1.214348183	0.001461643	Arabidopsis thaliana uncharacterized protein mRNA, complete cds
AT5G15340	1.063491678	0.001493441	Arabidopsis thaliana chromosome 5 sequence
AT3G61840	2.451048441	0.001565806	Arabidopsis thaliana chromosome 3, complete sequence
AT3G09400	2.451048441	0.001565806	Arabidopsis thaliana putative protein phosphatase 2C 36 mRNA, complete cds
AT1G32510	2.451048441	0.001565806	Arabidopsis thaliana NAC domain containing protein 11 mRNA, complete cds
AT5G15120	1.010919961	0.001580614	Arabidopsis thaliana uncharacterized protein mRNA, complete cds
AT5G45113	2.036010942	0.001600659	Arabidopsis thaliana chromosome 5 sequence
AT2G21450	2.036010942	0.001600659	Arabidopsis thaliana chromatin remodeling 34 mRNA, complete cds
AT2G41970	2.205935943	0.001635018	Arabidopsis thaliana putative protein kinase mRNA, complete cds
AT2G18540	1.451048441	0.001641568	Arabidopsis thaliana cupin family protein mRNA, complete cds
ATCG00730	1.451048441	0.001641568	Arabidopsis thaliana chloroplast DNA, complete genome, ecotype: Columbia
AT2G23270	1.451048441	0.001641568	Arabidopsis thaliana chromosome 2, complete sequence
AT5G57785	1.173514466	0.001731292	Arabidopsis thaliana chromosome 5 sequence
AT3G10600	1.223637945	0.001771762	Arabidopsis thaliana cationic amino acid transporter 7 mRNA, complete cds
AT1G09400	3.49544256	0.00180748	Arabidopsis thaliana putative

		9	12-oxophytodienoate reductase-like protein 1 mRNA, complete cds
AT1G28730	3.49544256	0.00180748 9	Arabidopsis thaliana chromosome 1 sequence
AT1G15385	3.49544256	0.00180748 9	Arabidopsis thaliana chromosome 1 sequence
AT4G12480	1.064580094	0.00182048 7	Arabidopsis thaliana chromosome 4 sequence
AT1G03170	1.064580094	0.00182048 7	Arabidopsis thaliana chromosome 1 sequence
AT1G51405	1.010015733	0.00192707 8	Arabidopsis thaliana myosin-related protein mRNA, complete cds
AT2G14095	1.679867132	0.00195872	Arabidopsis thaliana uncharacterized protein mRNA, complete cds
AT5G11620	1.135546615	0.00202893 9	Arabidopsis thaliana SWIM zinc finger family protein / mitogen-activated protein kinase kinase kinase (MAPKKK)-related mRNA, complete cds
AT4G15380	1.233950319	0.00214741 4	Arabidopsis thaliana cytochrome P450, family 705, subfamily A, polypeptide 4 mRNA, complete cds
AT1G75720	1.521437769	0.00228459 1	Arabidopsis thaliana uncharacterized protein mRNA, complete cds
AT2G27280	1.521437769	0.00228459 1	Arabidopsis thaliana Coiled-coil domain-containing protein 55 mRNA, complete cds
AT1G78440	1.100141279	0.00235492 3	Arabidopsis thaliana gibberellin 2-beta-dioxygenase 1 mRNA, complete cds
AT2G02680	1.100141279	0.00235492 3	Arabidopsis thaliana cysteine/histidine-rich C1 domain-containing protein mRNA, complete cds
AT2G02000	1.100141279	0.00235492 3	Arabidopsis thaliana glutamate decarboxylase 3 mRNA, complete cds
AT3G50980	2.73645066	0.00245596 2	Arabidopsis thaliana dehydrin xero 1 mRNA, complete cds
AT1G06320	2.73645066	0.00245596 2	Arabidopsis thaliana uncharacterized protein mRNA, complete cds
AT4G05053	2.73645066	0.00245596 2	Arabidopsis thaliana chromosome 4 sequence
AT3G02240	2.73645066	0.00245596 2	Arabidopsis thaliana root meristem growth factor 7 mRNA, complete cds
AT3G01620	2.73645066	0.00245596 2	Arabidopsis thaliana beta-1,4-N-acetylglucosaminyltransferase family protein mRNA, complete cds

AT5G36180	1.843365864	0.002468841	Arabidopsis thaliana serine carboxypeptidase-like 1 mRNA, complete cds
AT2G30766	1.405244751	0.002486904	Arabidopsis thaliana chromosome 2, complete sequence
AT4G12450	1.566525658	0.002672489	Arabidopsis thaliana chromosome 4 sequence
AT5G11610	1.566525658	0.002672489	Arabidopsis thaliana Exostosin family protein mRNA, complete cds
AT1G33813.1	1.566525658	0.002672489	Arabidopsis thaliana chromosome 1 sequence
AT2G25540	1.566525658	0.002672489	Arabidopsis thaliana cellulose synthase 10 mRNA, complete cds
AT1G26410	4.205935943	0.002677101	Arabidopsis thaliana chromosome 1 sequence
AT5G65850	1.96201036	0.002679137	Arabidopsis thaliana chromosome 5 sequence
AT1G80090	1.96201036	0.002679137	Arabidopsis thaliana SNF1-related protein kinase regulatory subunit gamma-like PV42b mRNA, complete cds
AT3G53220	2.357939037	0.002758977	Arabidopsis thaliana thioredoxin-like 3-3 mRNA, complete cds
AT3G19920	2.357939037	0.002758977	Arabidopsis thaliana uncharacterized protein mRNA, complete cds
AT3G28153.1	2.357939037	0.002758977	Arabidopsis thaliana chromosome 3, complete sequence
AT1G33760	2.123473783	0.002800453	Arabidopsis thaliana chromosome 1 sequence
AT1G78950	1.007996566	0.00286917	Arabidopsis thaliana beta-amyrin synthase mRNA, complete cds
AT4G40020	1.434560318	0.002962274	Arabidopsis thaliana chromosome 4 sequence
AT3G09550	1.434560318	0.002962274	Arabidopsis thaliana ankyrin repeat-containing protein mRNA, complete cds
AT1G50270	1.036010942	0.003092511	Arabidopsis thaliana chromosome 1 sequence
AT5G09720	1.620973442	0.003101171	Arabidopsis thaliana putative inactive magnesium transporter MRS2-8 mRNA, complete cds
AT1G13430	1.196475614	0.003110708	Arabidopsis thaliana chromosome 1 sequence
AT1G32415	1.335571224	0.003112197	Arabidopsis thaliana chromosome 1 sequence
AT4G34790	3.357939037	0.003424157	Arabidopsis thaliana chromosome 4 sequence
AT3G42658.1	3.357939037	0.003424157	Arabidopsis thaliana chromosome 3, complete sequence

AT1G12672	3.357939037	0.003424157	Arabidopsis thaliana uncharacterized protein mRNA, complete cds
AT1G64310	3.357939037	0.003424157	Arabidopsis thaliana chromosome 1 sequence
AT1G11740	3.357939037	0.003424157	Arabidopsis thaliana ankyrin repeat family protein mRNA, complete cds
AT3G27473	1.10640027	0.003505826	Arabidopsis thaliana chromosome 3, complete sequence
AT1G44130	1.688087638	0.003559627	Arabidopsis thaliana aspartyl protease family protein mRNA, complete cds
AT3G25240	1.151488159	0.003670539	Arabidopsis thaliana chromosome 3, complete sequence
AT5G60440	1.357939037	0.003740733	Arabidopsis thaliana agamous-like MADS-box protein AGL62 mRNA, complete cds
AT1G76952	1.205935943	0.003782815	Arabidopsis thaliana inflorescence deficient in abscission (IDA)-like 5 protein mRNA, complete cds
AT1G20700	1.205935943	0.003782815	Arabidopsis thaliana WUSCHEL-related homeobox 14 mRNA, complete cds
AT1G71760	1.772976536	0.004024375	Arabidopsis thaliana uncharacterized protein mRNA, complete cds
AT4G02650	1.50994213	0.004151975	Arabidopsis thaliana putative clathrin assembly protein mRNA, complete cds
AT3G13240	1.005637293	0.004282309	Arabidopsis thaliana uncharacterized protein mRNA, complete cds
AT4G22070	1.884007848	0.004451328	Arabidopsis thaliana WRKY DNA-binding protein 31 mRNA, complete cds
AT5G39100	2.620973442	0.00446305	Arabidopsis thaliana germin-like protein 6 mRNA, complete cds
ATCG00650	2.620973442	0.00446305	Arabidopsis thaliana chloroplast DNA, complete genome, ecotype: Columbia
AT5G02502	1.383934245	0.004487135	Arabidopsis thaliana Oligosaccaryltransferase mRNA, complete cds
AT1G01880	1.216583187	0.00460048	Arabidopsis thaliana 5'-3' exonuclease family protein mRNA, complete cds
AT5G41160	1.216583187	0.00460048	Arabidopsis thaliana chromosome 5 sequence
AT4G16350	2.036010942	0.004763282	Arabidopsis thaliana calcineurin B-like protein 6 mRNA, complete cds
AT4G03816.1	2.258403363	0.00483235	Arabidopsis thaliana chromosome 4 sequence
AT3G09790	2.258403363	0.00483235	Arabidopsis thaliana chromosome 3, complete sequence
AT5G66815	2.258403363	0.00483235	Arabidopsis thaliana chromosome 5 sequence

AT4G20450	2.258403363	0.00483235	Arabidopsis thaliana putative LRR receptor-like serine/threonine-protein kinase mRNA, complete cds
AT5G02570	1.559572898	0.004871891	Arabidopsis thaliana chromosome 5 sequence
AT2G42510	1.071634851	0.004942033	Arabidopsis thaliana uncharacterized protein mRNA, complete cds
AT4G34300	4.036010942	0.005147447	Arabidopsis thaliana chromosome 4 sequence
AT1G51920	4.036010942	0.005147447	Arabidopsis thaliana uncharacterized protein mRNA, complete cds
AT1G63760	4.036010942	0.005147447	Arabidopsis thaliana chromosome 1 sequence
AT1G45020.1	4.036010942	0.005147447	Arabidopsis thaliana chromosome 1 sequence
AT5G39890	1.414522565	0.005368072	Arabidopsis thaliana mRNA for hypothetical protein, complete cds, clone: RAFL14-60-L23
AT2G32550	1.414522565	0.005368072	Arabidopsis thaliana cell differentiation, Rcd1-like protein mRNA, complete cds
AT2G44010	1.165293959	0.005461069	Arabidopsis thaliana chromosome 2, complete sequence
AT1G08730	1.309029436	0.005584572	Arabidopsis thaliana myosin class XI mRNA, complete cds
AT1G74550	1.309029436	0.005584572	Arabidopsis thaliana chromosome 1 sequence
AT4G17100	1.620973442	0.005665924	Arabidopsis thaliana uncharacterized protein mRNA, complete cds
AT5G02220	1.451048441	0.006398872	Arabidopsis thaliana chromosome 5 sequence
AT5G55590	1.451048441	0.006398872	Arabidopsis thaliana pectinesterase QRT1 mRNA, complete cds
AT5G12920	1.451048441	0.006398872	Arabidopsis thaliana WD40 domain-containing protein mRNA, complete cds
AT4G28460	1.451048441	0.006398872	Arabidopsis thaliana chromosome 4 sequence
AT4G14780	1.451048441	0.006398872	Arabidopsis thaliana protein kinase family protein mRNA, complete cds
AT5G62040	1.002844078	0.006409702	Arabidopsis thaliana protein BROTHER of FT and TFL 1 mRNA, complete cds
AT5G33395.1	3.205935943	0.006494383	Arabidopsis thaliana chromosome 5 sequence
AT2G28426	3.205935943	0.00649438	Arabidopsis thaliana chromosome 2,

		3	complete sequence
AT3G25233	3.205935943	0.006494383	Arabidopsis thaliana chromosome 3, complete sequence
AT2G27120	3.205935943	0.006494383	Arabidopsis thaliana DNA polymerase epsilon catalytic subunit B mRNA, complete cds
AT3G13435	1.331466825	0.006743802	Arabidopsis thaliana uncharacterized protein mRNA, complete cds
AT2G42850	1.242461819	0.006803635	Arabidopsis thaliana cytochrome P450, family 718 mRNA, complete cds
AT5G27250.1	1.036010942	0.006920886	Arabidopsis thaliana chromosome 5 sequence
AT1G69570	1.801545688	0.007336094	Arabidopsis thaliana Dof zinc finger protein DOF1.10 mRNA, complete cds
AT2G45040	1.801545688	0.007336094	Arabidopsis thaliana chromosome 2, complete sequence
AT2G39500	1.075539306	0.007404681	Arabidopsis thaliana uncharacterized protein mRNA, complete cds
AT2G19530	1.001245524	0.007851427	Arabidopsis thaliana uncharacterized protein mRNA, complete cds
AT1G10680	1.942901537	0.008038974	Arabidopsis thaliana ABC transporter B family member 10 mRNA, complete cds
AT5G08030	1.942901537	0.008038974	Arabidopsis thaliana glycerophosphoryl diester phosphodiesterase mRNA, complete cds
AT3G49540	2.49544256	0.008073218	Arabidopsis thaliana uncharacterized protein mRNA, complete cds
AT4G26020	2.49544256	0.008073218	Arabidopsis thaliana uncharacterized protein mRNA, complete cds
AT5G55650	2.49544256	0.008073218	Arabidopsis thaliana chromosome 5 sequence
AT1G28990	2.49544256	0.008073218	Arabidopsis thaliana chromosome 1 sequence
AT5G49370	1.357939037	0.008129875	Arabidopsis thaliana Pleckstrin homology (PH) domain-containing protein-like protein mRNA, complete cds
AT5G25260	1.357939037	0.008129875	Arabidopsis thaliana SPFH/Band 7/PHB domain-containing membrane-associated protein mRNA, complete cds
AT5G45220	1.18285233	0.008138996	Arabidopsis thaliana TIR-NBS-LRR class disease resistance protein mRNA, complete cds
AT3G20590	1.18285233	0.008138996	Arabidopsis thaliana chromosome 3, complete sequence

AT5G01180	1.18285233	0.008138996	Arabidopsis thaliana peptide transporter PTR5 mRNA, complete cds
AT2G02630	1.258403363	0.008271524	Arabidopsis thaliana chromosome 2, complete sequence
AT2G33070	2.151488159	0.008405916	Arabidopsis thaliana Nitrile-specifier protein 2 mRNA, complete cds
AT1G61410	2.151488159	0.008405916	Arabidopsis thaliana chromosome 1 sequence
AT1G04560	2.151488159	0.008405916	Arabidopsis thaliana AWPM-19-like family protein mRNA, complete cds
AT2G24683	2.151488159	0.008405916	Arabidopsis thaliana chromosome 2, complete sequence
AT3G05140	1.036010942	0.00848225	Arabidopsis thaliana receptor-like cytosolic serine/threonine-protein kinase RBK2 mRNA, complete cds
AT5G45730	1.077831117	0.009075366	Arabidopsis thaliana cysteine/histidine-rich C1 domain-containing protein mRNA, complete cds
AT4G12900	1.129120346	0.009584551	Arabidopsis thaliana Gamma interferon responsive lysosomal thiol (GILT) reductase family protein mRNA, complete cds
ATCG01050	1.389647896	0.009777566	Arabidopsis thaliana chloroplast DNA, complete genome, ecotype: Columbia
AT5G06805.1	1.193552219	0.009942263	Arabidopsis thaliana chromosome 5 sequence
AT5G37970	3.843365864	0.009976169	Arabidopsis thaliana probable S-adenosylmethionine-dependent methyltransferase mRNA, complete cds
AT2G47040	3.843365864	0.009976169	Arabidopsis thaliana pectinesterase 5 mRNA, complete cds
AT5G05840	1.277019041	0.010051655	Arabidopsis thaliana uncharacterized protein mRNA, complete cds
AT5G02140	1.036010942	0.010405943	Arabidopsis thaliana pathogenesis-related thaumatin-like protein mRNA, complete cds
AT3G49055	1.036010942	0.010405943	Arabidopsis thaliana chromosome 3 hypothetical protein AT3G49055 mRNA, complete cds, alternatively spliced
AT5G14820	1.620973442	0.010428318	Arabidopsis thaliana chromosome 5 sequence
AT1G24480	1.620973442	0.010428318	Arabidopsis thaliana chromosome 1 sequence
AT5G66110	1.620973442	0.010428318	Arabidopsis thaliana heavy metal associated isoprenylated plant protein 27 mRNA, complete cds

AT4G39361	1.080405061	0.011133691	Arabidopsis thaliana chromosome 4 sequence
AT1G24130	1.080405061	0.011133691	Arabidopsis thaliana chromosome 1 sequence
AT1G11540	1.080405061	0.011133691	Arabidopsis thaliana Sulfite exporter TauE/SafE family protein mRNA, complete cds
AT4G14170	1.428328365	0.011719549	Arabidopsis thaliana pentatricopeptide repeat-containing protein mRNA, complete cds
AT1G45616	1.135546615	0.011746237	Arabidopsis thaliana chromosome 1 sequence
AT2G22890	1.135546615	0.011746237	Arabidopsis thaliana chromosome 2, complete sequence
AT3G50940	1.714082847	0.011982851	Arabidopsis thaliana cytochrome BC1 synthesis-like protein mRNA, complete cds
AT1G33500	1.714082847	0.011982851	Arabidopsis thaliana uncharacterized protein mRNA, complete cds
AT1G13609	1.714082847	0.011982851	Arabidopsis thaliana defensin-like protein 287 mRNA, complete cds
AT3G19040	1.714082847	0.011982851	Arabidopsis thaliana transcription initiation factor TFIID subunit 1-B mRNA, complete cds
AT3G45390	1.205935943	0.012149486	Arabidopsis thaliana concanavalin A-like lectin kinase-like protein mRNA, complete cds
AT3G29140	1.299045348	0.012205847	Arabidopsis thaliana uncharacterized protein mRNA, complete cds
AT3G11390	3.036010942	0.01232611	Arabidopsis thaliana chromosome 3, complete sequence
AT5G52270	3.036010942	0.01232611	Arabidopsis thaliana SNARE-like superfamily protein mRNA, complete cds
AT1G47950.1	3.036010942	0.01232611	Arabidopsis thaliana chromosome 1 sequence
AT2G43670	3.036010942	0.01232611	Arabidopsis thaliana carbohydrate-binding X8 domain-containing protein mRNA, complete cds
AT5G42340	3.036010942	0.01232611	Arabidopsis thaliana U-box domain-containing protein 15 mRNA, complete cds
AT1G71770	3.036010942	0.01232611	Arabidopsis thaliana polyadenylate-binding protein 5 mRNA, complete cds
AT3G49340	3.036010942	0.01232611	Arabidopsis thaliana putative cysteine proteinase mRNA, complete cds
AT5G24200	3.036010942	0.01232611	Arabidopsis thaliana uncharacterized protein mRNA, complete cds
AT1G73175	3.036010942	0.01232611	Arabidopsis thaliana chromosome 1 sequence

AT3G17050.1	3.036010942	0.01232611	Arabidopsis thaliana chromosome 3, complete sequence
AT2G22410	1.036010942	0.012779526	Arabidopsis thaliana chromosome 2, complete sequence
AT2G22140	1.036010942	0.012779526	Arabidopsis thaliana essential meiotic endonuclease 1B mRNA, complete cds
AT4G24910	1.036010942	0.012779526	Arabidopsis thaliana chromosome 4 sequence
AT1G43000	1.843365864	0.013449312	Arabidopsis thaliana PLATZ transcription factor family protein mRNA, complete cds
AT1G19780	1.843365864	0.013449312	Arabidopsis thaliana cyclic nucleotide gated channel 8 mRNA, complete cds
AT1G42560	1.843365864	0.013449312	Arabidopsis thaliana MLO-like protein 9 mRNA, complete cds
AT5G13900	1.843365864	0.013449312	Arabidopsis thaliana lipid transfer-like protein VAS mRNA, complete cds
AT5G36700	1.083316657	0.013673254	Arabidopsis thaliana 2-phosphoglycolate phosphatase 1 mRNA, complete cds
AT3G58100	1.083316657	0.013673254	Arabidopsis thaliana glucan endo-1,3-beta-glucosidase-like protein 1 mRNA, complete cds
AT5G55680	1.476583533	0.013978673	Arabidopsis thaliana chromosome 5 sequence
AT1G01280	1.476583533	0.013978673	Arabidopsis thaliana cytochrome P450, family 703, subfamily A, polypeptide 2 mRNA, complete cds
AT3G48920	1.476583533	0.013978673	Arabidopsis thaliana myb domain protein 45 mRNA, complete cds
AT4G01780	1.476583533	0.013978673	Arabidopsis thaliana XH/XS domain-containing protein mRNA, complete cds
AT1G05894	1.142926146	0.014407965	Arabidopsis thaliana chromosome 1 sequence
AT1G16640	1.142926146	0.014407965	Arabidopsis thaliana AP2/B3-like transcriptional factor family protein mRNA, complete cds
AT2G38720	1.142926146	0.014407965	Arabidopsis thaliana microtubule-associated protein 65-5 mRNA, complete cds
AT1G70910	2.036010942	0.014506857	Arabidopsis thaliana chromosome 1 sequence
AT1G08430	2.036010942	0.014506857	Arabidopsis thaliana aluminum-activated malate transporter 1 mRNA, complete cds
AT2G05995	2.036010942	0.014506857	Arabidopsis thaliana clone 10529 mRNA sequence

AT2G11310.1	2.357939037	0.014521731	Arabidopsis thaliana chromosome 2, complete sequence
AT1G17400	2.357939037	0.014521731	Arabidopsis thaliana uncharacterized protein mRNA, complete cds
AT4G19829	2.357939037	0.014521731	Arabidopsis thaliana chromosome 4 sequence
AT1G27990	2.357939037	0.014521731	Arabidopsis thaliana AT1G27990 mRNA, complete cds, clone: RAFL22-35-E15
AT5G58360	2.357939037	0.014521731	Arabidopsis thaliana chromosome 5 sequence
AT3G20190	2.357939037	0.014521731	Arabidopsis thaliana leucine-rich repeat protein kinase-like protein mRNA, complete cds
AT1G55720	2.357939037	0.014521731	Arabidopsis thaliana cation exchanger 6 mRNA, complete cds
AT4G01190	2.357939037	0.014521731	Arabidopsis thaliana phosphatidylinositol phosphate kinase 10 mRNA, complete cds
AT3G05858	2.357939037	0.014521731	Arabidopsis thaliana chromosome 3, complete sequence
AT5G57980	1.325517559	0.014804201	Arabidopsis thaliana RNA polymerase II fifth largest subunit, C mRNA, complete cds
AT3G04440	1.220435513	0.014851064	Arabidopsis thaliana plasma-membrane choline transporter family protein mRNA, complete cds
AT5G35770	1.220435513	0.014851064	Arabidopsis thaliana transcriptional regulator STERILE APETALA mRNA, complete cds
AT5G42840	1.220435513	0.014851064	Arabidopsis thaliana cysteine/histidine-rich C1 domain-containing protein mRNA, complete cds
AT1G05730	1.538511282	0.016552289	Arabidopsis thaliana uncharacterized protein mRNA, complete cds
AT3G12470	1.538511282	0.016552289	Arabidopsis thaliana chromosome 3, complete sequence
AT1G54400	1.538511282	0.016552289	Arabidopsis thaliana alpha-crystallin domain of heat shock protein-containing protein mRNA, complete cds
AT2G18480	1.538511282	0.016552289	Arabidopsis thaliana putative polyol transporter 3 mRNA, complete cds
AT3G47110	1.086637015	0.016811599	Arabidopsis thaliana putative receptor-like protein kinase mRNA, complete cds
AT2G16970	1.086637015	0.016811599	Arabidopsis thaliana tetracycline transporter-like protein 1 mRNA, complete cds
AT4G04510	1.357939037	0.01792248	Arabidopsis thaliana cysteine-rich

		4	receptor-like protein kinase 38 mRNA, complete cds
AT5G20260	1.357939037	0.017922484	Arabidopsis thaliana Exostosin family protein mRNA, complete cds
AT1G78476	1.357939037	0.017922484	Arabidopsis thaliana uncharacterized protein mRNA, complete cds
AT5G51795	1.357939037	0.017922484	Arabidopsis thaliana DNA/RNA-binding protein Kin17, conserved region-containing protein mRNA, complete cds
ATCG00360	1.237644803	0.018156466	Arabidopsis lyrata subsp. lyrata predicted protein, mRNA
AT1G68200	1.036010942	0.019345207	Arabidopsis thaliana zinc finger CCCH domain-containing protein 15 mRNA, complete cds
AT2G21020	1.036010942	0.019345207	Arabidopsis thaliana chromosome 2, complete sequence
AT3G18880	1.036010942	0.019345207	Arabidopsis thaliana Nucleic acid-binding, OB-fold-like protein mRNA, complete cds
AT3G16360	1.036010942	0.019345207	Arabidopsis thaliana histidine-containing phosphotransfer protein 4 mRNA, complete cds
AT1G73700	1.620973442	0.019380487	Arabidopsis thaliana MATE efflux family protein mRNA, complete cds
AT5G45276	1.620973442	0.019380487	Arabidopsis thaliana chromosome 5 sequence
AT5G46417	1.620973442	0.019380487	Arabidopsis thaliana chromosome 5 sequence
AT4G15710	3.620973442	0.019502397	Arabidopsis thaliana uncharacterized protein mRNA, complete cds
AT1G12211	3.620973442	0.019502397	Arabidopsis thaliana chromosome 1 sequence
AT5G59860	3.620973442	0.019502397	Arabidopsis thaliana RNA recognition motif-containing protein mRNA, complete cds
AT3G03272	3.620973442	0.019502397	Arabidopsis thaliana chromosome 3, complete sequence
AT5G53520	3.620973442	0.019502397	Arabidopsis thaliana oligopeptide transporter 8 mRNA, complete cds
AT3G43200	3.620973442	0.019502397	Arabidopsis thaliana chromosome 3, complete sequence
AT1G62095	3.620973442	0.019502397	Arabidopsis thaliana chromosome 1 sequence
AT2G15030	1.090458726	0.020696872	Arabidopsis thaliana chromosome 2, complete sequence

AT5G51390	1.090458726	0.020696872	Arabidopsis thaliana chromosome 5 sequence
AT3G16380	1.398581021	0.021634821	Arabidopsis thaliana poly(A) binding protein 6 mRNA, complete cds
AT1G76955	1.398581021	0.021634821	Arabidopsis thaliana uncharacterized protein mRNA, complete cds
AT5G01420	1.398581021	0.021634821	Arabidopsis thaliana chromosome 5 sequence
AT1G05880	1.398581021	0.021634821	Arabidopsis thaliana putative E3 ubiquitin-protein ligase ARI12 mRNA, complete cds
AT1G01750	1.398581021	0.021634821	Arabidopsis thaliana actin depolymerizing factor 11 mRNA, complete cds
AT4G04293.1	1.161541824	0.021738951	Arabidopsis thaliana chromosome 4 sequence
AT1G68930	1.161541824	0.021738951	Arabidopsis thaliana chromosome 1 sequence
AT1G72220	1.161541824	0.021738951	Arabidopsis thaliana chromosome 1 sequence
AT5G48290	1.161541824	0.021738951	Arabidopsis thaliana heavy metal transport/detoxification domain-containing protein mRNA, complete cds
AT4G14390	1.161541824	0.021738951	Arabidopsis thaliana ankyrin repeat-containing protein mRNA, complete cds
AT1G16140	1.161541824	0.021738951	Arabidopsis thaliana chromosome 1 sequence
AT5G38540	1.258403363	0.022196826	Arabidopsis thaliana Mannose-binding lectin superfamily protein mRNA, complete cds
AT3G09590	1.258403363	0.022196826	Arabidopsis thaliana chromosome 3, complete sequence
AT3G24340	1.258403363	0.022196826	Arabidopsis thaliana chromatin remodeling 40 mRNA, complete cds
AT1G05530	1.258403363	0.022196826	Arabidopsis thaliana chromosome 1 sequence
AT4G13690	1.73645066	0.022280586	Arabidopsis thaliana uncharacterized protein mRNA, complete cds
AT5G51210	1.73645066	0.022280586	Arabidopsis thaliana oleosin3 mRNA, complete cds
AT4G35783	1.73645066	0.022280586	Arabidopsis thaliana chromosome 4 sequence
AT4G26170	1.73645066	0.022280586	Arabidopsis thaliana uncharacterized protein mRNA, complete cds
AT2G17940	1.73645066	0.02228058	Arabidopsis thaliana uncharacterized protein

		6	mRNA, complete cds
AT5G52720	2.843365864	0.023395599	Arabidopsis thaliana copper transport family protein mRNA, complete cds
AT2G12420.1	2.843365864	0.023395599	Arabidopsis thaliana chromosome 2, complete sequence
AT3G07150	2.843365864	0.023395599	Arabidopsis thaliana uncharacterized protein mRNA, complete cds
AT4G27210.1	2.843365864	0.023395599	Arabidopsis thaliana chromosome 4 sequence
AT1G19115	2.843365864	0.023395599	Arabidopsis thaliana uncharacterized protein mRNA, complete cds
AT1G55230	2.843365864	0.023395599	Arabidopsis thaliana chromosome 1 sequence
AT5G10340	2.843365864	0.023395599	Arabidopsis thaliana F-box protein mRNA, complete cds
AT2G29150	2.843365864	0.023395599	Arabidopsis thaliana tropinone reductase-like protein mRNA, complete cds
AT1G35130.1	2.843365864	0.023395599	Arabidopsis thaliana chromosome 1 sequence
AT5G64540	2.843365864	0.023395599	Arabidopsis thaliana uncharacterized protein mRNA, complete cds
AT3G56891	2.843365864	0.023395599	Arabidopsis thaliana metal ion binding protein mRNA, complete cds
AT2G27630	1.91048006	0.024807276	Arabidopsis thaliana ubiquitin carboxyl-terminal hydrolase-related protein mRNA, complete cds
AT4G01500	1.91048006	0.024807276	Arabidopsis thaliana B3 domain-containing transcription factor NGA4 mRNA, complete cds
AT2G29220	1.91048006	0.024807276	Arabidopsis thaliana chromosome 2, complete sequence
AT5G37250	1.91048006	0.024807276	Arabidopsis thaliana chromosome 5 sequence
AT5G64490	1.91048006	0.024807276	Arabidopsis thaliana chromosome 5 sequence
AT3G54520	1.91048006	0.024807276	Arabidopsis thaliana uncharacterized protein mRNA, complete cds
AT1G31173	1.91048006	0.024807276	Arabidopsis thaliana chromosome 1 sequence
AT5G44900	1.91048006	0.024807276	Arabidopsis thaliana Toll-Interleukin-Resistance domain-containing protein mRNA, complete cds
AT5G41755.	1.094904631	0.02551657	Arabidopsis thaliana chromosome 5 sequence

1		1	
AT1G33615	1.094904631	0.025516571	Arabidopsis thaliana chromosome 1 sequence
AT1G01695	2.205935943	0.025941022	Arabidopsis thaliana phosphatidylinositol N-acetyglucosaminlytransferase subunit P-like protein mRNA, complete cds
AT4G09130	2.205935943	0.025941022	Arabidopsis thaliana chromosome 4 sequence
AT3G55310	2.205935943	0.025941022	Arabidopsis thaliana Rossmann-fold NAD(P)-binding domain-containing protein mRNA, complete cds
AT1G58320	2.205935943	0.025941022	Arabidopsis thaliana PLAC8 family protein mRNA, complete cds
AT5G52700	2.205935943	0.025941022	Arabidopsis thaliana copper transport family protein mRNA, complete cds
AT1G53100	2.205935943	0.025941022	Arabidopsis thaliana core-2/I-branching beta-1,6-N-acetylglucosaminyltransferase-like protein mRNA, complete cds
AT5G66150	1.451048441	0.025996305	Arabidopsis thaliana Glycosyl hydrolase family protein mRNA, complete cds
AT1G55790	1.451048441	0.025996305	Arabidopsis thaliana uncharacterized protein mRNA, complete cds
AT4G18990	1.451048441	0.025996305	Arabidopsis thaliana probable xyloglucan endotransglucosylase/hydrolase protein 29 mRNA, complete cds
AT4G03130	1.173514466	0.02674327	Arabidopsis thaliana BRCT domain-containing DNA repair protein mRNA, complete cds
AT1G59265.1	1.173514466	0.02674327	Arabidopsis thaliana chromosome 1 sequence
AT5G51490	1.283938455	0.02712622	Arabidopsis thaliana putative pectinesterase/pectinesterase inhibitor 59 mRNA, complete cds
AT1G64920	1.283938455	0.02712622	Arabidopsis thaliana chromosome 1 sequence
AT1G52800	1.036010942	0.029457109	Arabidopsis thaliana oxidoreductase, 2OG-Fe(II) oxygenase family protein mRNA, complete cds
AT1G22600	1.521437769	0.03100332	Arabidopsis thaliana Late embryogenesis abundant protein (LEA) family protein mRNA, complete cds
AT4G33290	1.521437769	0.03100332	Arabidopsis thaliana chromosome 4 sequence
AT2G21260	1.521437769	0.03100332	Arabidopsis thaliana putative mannose-6P reductase mRNA, complete cds
AT3G53235	1.521437769	0.03100332	Arabidopsis thaliana uncharacterized protein

			mRNA, complete cds
AT5G48175	1.521437769	0.03100332	Arabidopsis thaliana uncharacterized protein mRNA, complete cds
AT1G20990	1.521437769	0.03100332	Arabidopsis thaliana Cysteine/Histidine-rich C1 domain family protein mRNA, complete cds

Supplementary Data S5B Down regulated genes in fc1/Col-0 with NaCl treatment.

Gene	Log2FoldChange	pval	NT:Description
AT3G45140	-2.579790665	0	Arabidopsis thaliana lipoxygenase 2 mRNA, complete cds
AT1G52400	-2.405542018	0	Arabidopsis thaliana beta glucosidase 18 mRNA, complete cds
AT5G24770	-1.822833833	0	Arabidopsis thaliana acid phosphatase VSP2 mRNA, complete cds
AT4G08870	-2.449415885	0	Arabidopsis thaliana arginine amidohydrolase 2 mRNA, complete cds
AT4G34710	-1.462707474	0	Arabidopsis thaliana chromosome 4 sequence
AT4G23600	-1.955390447	0	Arabidopsis thaliana cystine lyase CORI3 mRNA, complete cds
AT3G44310	-1.065971906	0	Arabidopsis thaliana nitrilase 1 mRNA, complete cds
AT3G28220	-2.679696512	0	Arabidopsis thaliana TRAF-like family protein mRNA, complete cds
AT3G25770	-2.30619652	0	Arabidopsis thaliana allene oxide cyclase 2 mRNA, complete cds
AT1G19570	-1.372529046	0	Arabidopsis thaliana dehydroascorbate reductase mRNA, complete cds
AT4G00430	-1.179446715	0	Arabidopsis thaliana plasma membrane intrinsic protein 1;4 mRNA, complete cds
AT5G42650	-1.566314619	0	Arabidopsis thaliana chromosome 5 sequence
AT1G19670	-2.480004205	0	Arabidopsis thaliana chlorophyllase 1 mRNA, complete cds
AT2G34810	-1.942991066	0	Arabidopsis thaliana chromosome 2, complete sequence
AT2G24850	-2.283659218	0	Arabidopsis thaliana tyrosine aminotransferase 3 mRNA, complete cds
AT4G18440	-2.420969661	0	Arabidopsis thaliana L-aspartase-like family protein mRNA, complete cds
AT2G29450	-1.214300183	0	Arabidopsis thaliana glutathione S-transferase tau 5 mRNA,

			complete cds
AT3G16470	-1.550698586	0	Arabidopsis thaliana JA-responsive protein 1 mRNA, complete cds
AT3G16400	-1.261283464	0	Arabidopsis thaliana Nitrile-specifier protein 1 mRNA, complete cds
AT5G24780	-2.575537813	0	Arabidopsis thaliana acid phosphatase VSP1 mRNA, complete cds
AT4G16760	-1.063638769	0	Arabidopsis thaliana peroxisomal acyl-coenzyme A oxidase 1 mRNA, complete cds
AT3G25760	-2.380671029	0	Arabidopsis thaliana allene oxide cyclase 1 mRNA, complete cds
AT2G47180	-1.513419526	0	Arabidopsis thaliana galactinol synthase 1 mRNA, complete cds
AT1G52000	-2.078272486	0	Arabidopsis thaliana mannose-binding lectin superfamily protein mRNA, complete cds
AT1G17420	-1.955247989	0	Arabidopsis thaliana lipoxygenase 3 mRNA, complete cds
AT1G73480	-1.093226494	0	Arabidopsis thaliana alpha/beta-Hydrolases superfamily protein mRNA, complete cds
AT1G43160	-2.259408642	0	Arabidopsis thaliana ethylene-responsive transcription factor RAP2-6 mRNA, complete cds
AT5G05600	-2.057604126	0	Arabidopsis thaliana oxidoreductase, 2OG-Fe(II) oxygenase family protein mRNA, complete cds
AT1G70700	-1.330853457	0	Arabidopsis thaliana putative jasmonate signaling protein JAZ9 mRNA, complete cds
AT2G38170	-1.158367103	0	Arabidopsis thaliana vacuolar cation/proton exchanger 1 mRNA, complete cds
AT2G39330	-2.301509452	0	Arabidopsis thaliana jacalin-related lectin 23 mRNA, complete cds
AT1G51760	-1.472374538	0	Arabidopsis thaliana IAA-amino acid hydrolase IAR3 mRNA, complete cds
AT2G39030	-3.260524569	0	Arabidopsis thaliana chromosome 2,

			complete sequence
AT1G52410	-1.988538366	0	Arabidopsis thaliana TSK-associating protein 1 mRNA, complete cds
AT2G25450	-1.046842505	0	Arabidopsis thaliana 1-aminocyclopropane-1-carboxylate oxidase-like protein mRNA, complete cds
AT2G06050	-1.640444565	0	Arabidopsis thaliana AT2G06050 mRNA, complete cds, clone: RAFL22-42-B17
AT5G24420	-2.833496705	0	Arabidopsis thaliana 6-phosphogluconolactonase 5 mRNA, complete cds
AT4G30530	-1.218047508	0	Arabidopsis thaliana gamma-glutamyl peptidase 1 mRNA, complete cds
AT4G34230	-1.316400043	0	Arabidopsis thaliana cinnamyl alcohol dehydrogenase 5 mRNA, complete cds
AT4G15210	-2.122983537	0	Arabidopsis thaliana beta-amylase 5 mRNA, complete cds
AT2G22330	-1.620045899	2.20E-302	Arabidopsis thaliana tryptophan N-monooxygenase 2 mRNA, complete cds
AT1G52040	-2.803446669	7.21E-302	Arabidopsis thaliana myrosinase-binding protein 1 mRNA, complete cds
AT4G31500	-1.184891958	4.24E-250	Arabidopsis thaliana cytochrome P450 83B1 mRNA, complete cds
AT1G44350	-1.892378034	8.35E-234	Arabidopsis thaliana IAA-amino acid hydrolase ILR1-like 6 mRNA, complete cds
AT3G51860	-2.160624713	8.84E-232	Arabidopsis thaliana vacuolar cation/proton exchanger 3 mRNA, complete cds
AT4G16590	-2.844769043	4.62E-228	Arabidopsis thaliana cellulose synthase-like A01 mRNA, complete cds
AT5G20230	-1.069342058	4.72E-216	Arabidopsis thaliana blue-copper-binding protein mRNA, complete cds
AT1G52890	-1.892707002	5.04E-216	Arabidopsis thaliana NAC domain-containing protein 19

			mRNA, complete cds
AT1G52030	-2.224949136	1.72E-215	Arabidopsis thaliana myrosinase-binding protein 2 mRNA, complete cds
AT1G74100	-1.443629965	5.83E-213	Arabidopsis thaliana chromosome 1 sequence
AT3G55970	-3.153813617	6.00E-203	Arabidopsis thaliana jasmonate-regulated protein JRG21 mRNA, complete cds
AT1G26770	-1.628192068	1.49E-198	Arabidopsis thaliana expansin A10 mRNA, complete cds
AT3G11480	-5.974355774	1.19E-196	Arabidopsis thaliana SABATH methyltransferase BSMT1 mRNA, complete cds
AT5G07010	-2.192481679	1.73E-194	Arabidopsis thaliana chromosome 5 sequence
AT3G44860	-1.432801913	1.65E-193	Arabidopsis thaliana farnesoic acid carboxyl-O-methyltransferase mRNA, complete cds
AT5G23820	-1.536455911	3.97E-191	Arabidopsis thaliana MD-2-related lipid recognition domain-containing protein mRNA, complete cds
AT5G20190	-1.001284902	2.59E-188	Arabidopsis thaliana tetratricopeptide repeat domain-containing protein mRNA, complete cds
AT1G66100	-1.715533117	1.31E-182	Arabidopsis thaliana probable thionin-2.4 mRNA, complete cds
AT1G76790	-1.887975648	2.29E-166	Arabidopsis thaliana indole glucosinolate o-methyltransferase 5 mRNA, complete cds
AT4G39950	-1.615968312	8.30E-165	Arabidopsis thaliana tryptophan N-monooxygenase 1 mRNA, complete cds
AT5G26030	-3.039698718	1.27E-164	Arabidopsis thaliana ferrochelatase 1 mRNA, complete cds
AT3G48520	-2.16996372	3.82E-162	Arabidopsis thaliana chromosome 3, complete sequence
AT2G34070	-1.513558515	2.56E-151	Arabidopsis thaliana trichome birefringence-like 37 protein mRNA, complete cds
AT1G72450	-1.051260319	3.46E-151	Arabidopsis thaliana jasmonate-zim-domain protein 6 mRNA, complete cds

AT4G37990	-3.03274175	3.54E-148	Arabidopsis thaliana cinnamyl alcohol dehydrogenase 8 mRNA, complete cds
AT2G20340	-1.776149328	1.66E-147	Arabidopsis thaliana tyrosine decarboxylase 1 mRNA, complete cds
AT1G24070	-2.066674722	4.41E-143	Arabidopsis thaliana cellulose synthase-like A10 mRNA, complete cds
AT4G01080	-2.342500682	2.58E-139	Arabidopsis thaliana protein TRICHOME BIREFRINGENCE-LIKE 26 mRNA, complete cds
AT5G54170	-1.052970412	3.51E-134	Arabidopsis thaliana lipid-binding START domain-containing protein mRNA, complete cds
AT4G17470	-2.415601172	6.18E-134	Arabidopsis thaliana putative palmitoyl-protein thioesterase mRNA, complete cds
AT5G67480	-1.696705179	1.51E-132	Arabidopsis thaliana BTB and TAZ domain protein 4 mRNA, complete cds
AT4G21680	-2.194584765	9.15E-132	Arabidopsis thaliana nitrate transporter 1.8 mRNA, complete cds
AT3G51450	-1.630944931	4.52E-129	Arabidopsis thaliana strictosidine synthase family protein mRNA, complete cds
AT1G61120	-2.598195078	1.44E-128	Arabidopsis thaliana terpene synthase 04 mRNA, complete cds
AT2G38240	-2.20026442	9.43E-128	Arabidopsis thaliana 2-oxoglutarate (2OG) and Fe(II)-dependent oxygenase-like protein mRNA, complete cds
AT1G05100	-1.334632438	1.50E-120	Arabidopsis thaliana chromosome 1 sequence
AT2G46520	-1.198454312	1.94E-119	Arabidopsis thaliana putative cellular apoptosis susceptibility protein / importin-alpha re-exporter mRNA, complete cds
AT4G27560	-1.191991448	1.66E-114	Arabidopsis thaliana chromosome 4 sequence
AT5G02940	-1.068032083	7.33E-112	Arabidopsis thaliana uncharacterized protein mRNA,

			complete cds
AT2G43530	-2.188420625	2.79E-111	Arabidopsis thaliana defensin-like protein 194 mRNA, complete cds
AT5G05730	-1.25536809	3.15E-110	Arabidopsis thaliana anthranilate synthase component I-1 mRNA, complete cds
AT3G12145	-1.59155494	4.25E-108	Arabidopsis thaliana leucine-rich repeat protein FLOR1 mRNA, complete cds
AT1G51780	-6.208749293	3.04E-107	Arabidopsis thaliana IAA-amino acid hydrolase ILR1-like 5 mRNA, complete cds
AT4G15440	-1.656460342	2.14E-106	Arabidopsis thaliana hydroperoxide lyase 1 mRNA, complete cds
AT1G66760	-1.399118911	1.90E-105	Arabidopsis thaliana MATE efflux family protein mRNA, complete cds
AT4G39940	-1.290252533	1.07E-102	Arabidopsis thaliana adenosine-5'-phosphosulfate-kinase 2 mRNA, complete cds
AT1G14250	-2.453227499	1.35E-102	Arabidopsis thaliana probable apyrase 5 mRNA, complete cds
AT1G54010	-2.061972612	9.50E-102	Arabidopsis thaliana GDSL esterase/lipase mRNA, complete cds
AT1G10585	-2.971588874	6.76E-95	Arabidopsis thaliana basic helix-loop-helix domain-containing protein mRNA, complete cds
AT3G09940	-1.023728947	1.23E-93	Arabidopsis thaliana monodehydroascorbate reductase (NADH) mRNA, complete cds
AT1G69480	-1.162059608	2.04E-92	Arabidopsis thaliana EXS (ERD1/XPR1/SYG1) family protein mRNA, complete cds
AT5G60890	-1.034689211	1.38E-91	Arabidopsis thaliana myb domain protein 34 mRNA, complete cds
AT2G38750	-1.421108871	2.40E-91	Arabidopsis thaliana annexin D4 mRNA, complete cds
AT2G04400	-1.288194494	7.59E-89	Arabidopsis thaliana indole-3-glycerol phosphate synthase mRNA, complete cds
AT1G68620	-1.385491221	3.12E-84	Arabidopsis thaliana chromosome 1 sequence
AT4G35630	-1.256625284	2.25E-80	Arabidopsis thaliana chromosome 4 sequence
AT1G80160	-1.740214083	2.05E-78	Arabidopsis thaliana

			GLYOXYLASE I 7 mRNA, complete cds
AT4G24000	-1.522059913	5.77E-77	Arabidopsis thaliana cellulose synthase-like protein G2 mRNA, complete cds
AT5G54300	-1.135664903	5.09E-76	Arabidopsis thaliana uncharacterized protein mRNA, complete cds
AT5G43745	-1.165900032	3.99E-75	Arabidopsis thaliana uncharacterized protein mRNA, complete cds
AT1G09500	-1.389150775	2.31E-72	Arabidopsis thaliana alcohol dehydrogenase-like protein mRNA, complete cds
AT2G29460	-1.310340627	2.34E-72	Arabidopsis thaliana glutathione S-transferase tau 4 mRNA, complete cds
AT2G37770	-1.671694624	2.55E-70	Arabidopsis thaliana aldo-keto reductase family 4 member C9 mRNA, complete cds
AT4G21830	-1.208941105	4.86E-68	Arabidopsis thaliana peptide methionine sulfoxide reductase B7 mRNA, complete cds
AT2G45570	-1.973326923	7.89E-65	Arabidopsis thaliana cytochrome P450 76C2 mRNA, complete cds
AT1G32970	-1.252234027	7.02E-62	Arabidopsis thaliana Subtilisin-like serine endopeptidase family protein mRNA, complete cds
AT2G16660	-1.324391301	1.01E-61	Arabidopsis thaliana Major facilitator superfamily protein mRNA, complete cds
AT2G29350	-1.13039562	2.86E-61	Arabidopsis thaliana senescence-associated protein 13 mRNA, complete cds
AT4G04840	-1.726191944	1.90E-60	Arabidopsis thaliana methionine sulfoxide reductase B6 mRNA, complete cds
AT1G54020	-2.617578278	2.54E-60	Arabidopsis thaliana GDSL esterase/lipase mRNA, complete cds
AT3G54640	-1.196891493	3.01E-60	Arabidopsis thaliana tryptophan synthase alpha chain mRNA, complete cds
AT1G72120	-1.623760147	3.12E-60	Arabidopsis thaliana putative peptide/nitrate transporter mRNA,

			complete cds
AT5G64750	-1.268607268	1.42E-59	Arabidopsis thaliana ethylene-responsive transcription factor ABR1 mRNA, complete cds
AT3G16530	-1.050329907	3.76E-59	Arabidopsis thaliana chromosome 3, complete sequence
AT2G03980	-1.398391882	6.99E-58	Arabidopsis thaliana GDSL esterase/lipase mRNA, complete cds
AT1G54030	-1.096526329	4.41E-56	Arabidopsis thaliana protein MODIFIED VACUOLE PHENOTYPE 1 mRNA, complete cds
AT1G56650	-1.199532847	6.21E-56	Arabidopsis thaliana transcription factor MYB75 mRNA, complete cds
AT5G13330	-2.168080663	1.03E-55	Arabidopsis thaliana ethylene-responsive transcription factor ERF113 mRNA, complete cds
AT2G34930	-1.149544711	4.78E-55	Arabidopsis thaliana chromosome 2, complete sequence
AT5G17860	-1.565510651	1.83E-53	Arabidopsis thaliana chromosome 5 sequence
AT5G65280	-1.0501972	4.51E-53	Arabidopsis thaliana protein GCR2-like 1 mRNA, complete cds
AT5G44050	-1.312792859	2.24E-52	Arabidopsis thaliana MATE efflux family protein mRNA, complete cds
AT3G46670	-1.432758541	6.78E-52	Arabidopsis thaliana UDP-glucosyl transferase 76E11 mRNA, complete cds
AT5G28510	-1.321127031	1.47E-51	Arabidopsis thaliana beta glucosidase 24 mRNA, complete cds
AT5G28237	-3.169664085	2.34E-51	Arabidopsis thaliana tryptophan synthase beta chain-like protein mRNA, complete cds
AT2G17500	-1.158184588	3.15E-51	Arabidopsis thaliana auxin efflux carrier family protein mRNA, complete cds
AT3G44870	-2.029876994	4.66E-49	Arabidopsis thaliana S-adenosyl-L-methionine-dependent methyltransferase mRNA, complete cds
AT1G06620	-1.173351531	7.52E-49	Arabidopsis thaliana

			2-oxoglutarate-dependent dioxygenase-like protein mRNA, complete cds
AT1G19640	-1.414518177	6.82E-48	Arabidopsis thaliana jasmonic acid carboxyl methyltransferase mRNA, complete cds
AT2G02990	-1.706191301	8.02E-48	Arabidopsis thaliana ribonuclease 1 mRNA, complete cds
AT3G02150	-1.327373989	1.79E-45	Arabidopsis thaliana transcription factor TCP13 mRNA, complete cds
AT2G46650	-1.124021255	3.12E-45	Arabidopsis thaliana cytochrome B5 isoform C mRNA, complete cds
AT2G43550	-1.714269006	1.96E-44	Arabidopsis thaliana defensin-like protein 197 mRNA, complete cds
AT4G24350	-1.448511658	1.02E-43	Arabidopsis thaliana phosphorylase family protein mRNA, complete cds
AT4G13410	-2.644108792	1.37E-42	Arabidopsis thaliana putative mannan synthase 15 mRNA, complete cds
AT5G38130	-2.688586095	1.04E-41	Arabidopsis thaliana chromosome 5 sequence
AT3G55110	-1.434209473	6.81E-41	Arabidopsis thaliana ABC transporter G family member 18 mRNA, complete cds
AT5G59920	-1.249762388	5.43E-40	Arabidopsis thaliana chromosome 5 sequence
AT3G04000	-2.079466276	5.50E-40	Arabidopsis thaliana aldehyde reductase mRNA, complete cds
AT3G46650	-1.535455716	6.65E-40	Arabidopsis thaliana UDP-glycosyltransferase-like protein mRNA, complete cds
AT3G05630	-1.420943947	6.08E-39	Arabidopsis thaliana phospholipase D P2 mRNA, complete cds
AT3G28007	-1.557513572	7.06E-39	Arabidopsis thaliana bidirectional sugar transporter SWEET4 mRNA, complete cds
AT5G23660	-1.105244717	2.77E-38	Arabidopsis thaliana bidirectional sugar transporter SWEET12 mRNA, complete cds
AT3G22740	-1.407090476	1.06E-37	Arabidopsis thaliana homocysteine S-methyltransferase 3 mRNA, complete cds
AT4G36950	-1.77134398	7.68E-37	Arabidopsis thaliana chromosome 4 sequence

AT1G73325	-3.384320857	3.72E-35	Arabidopsis thaliana chromosome 1 sequence
AT2G30830	-4.375802657	3.57E-34	Arabidopsis thaliana 2-oxoglutarate dependent oxygenase-like protein mRNA, complete cds
AT5G63450	-1.292149107	8.55E-34	Arabidopsis thaliana chromosome 5 sequence
AT3G61400	-1.247570841	8.12E-33	Arabidopsis thaliana 1-aminocyclopropane-1-carboxylate oxidase-like 8 mRNA, complete cds
AT5G13490	-1.039540091	2.39E-32	Arabidopsis thaliana ADP/ATP carrier protein 2 mRNA, complete cds
AT4G03400	-1.091209609	2.84E-31	Arabidopsis thaliana auxin-responsive GH3 family protein mRNA, complete cds
AT5G50360	-1.307504015	3.70E-30	Arabidopsis thaliana chromosome 5 sequence
AT4G29700	-1.074936617	3.80E-30	Arabidopsis thaliana chromosome 4 sequence
AT2G04080	-1.312175219	5.63E-30	Arabidopsis thaliana MATE efflux family protein mRNA, complete cds
AT1G65890	-1.033382394	1.00E-29	Arabidopsis thaliana acyl activating enzyme 12 mRNA, complete cds
AT5G36220	-1.140552702	6.49E-29	Arabidopsis thaliana cytochrome P450 81D1 mRNA, complete cds
AT3G28930	-1.250641678	2.98E-28	Arabidopsis thaliana avrRpt2-induced protein AIG2 mRNA, complete cds
AT4G33467	-1.734230807	6.00E-28	Arabidopsis thaliana uncharacterized protein mRNA, complete cds
AT1G52720	-1.035504172	1.37E-26	Arabidopsis thaliana chromosome 1 sequence
AT5G53750	-1.445594174	1.89E-26	Arabidopsis thaliana CBS domain-containing protein mRNA, complete cds
AT1G61610	-1.243212702	5.71E-26	Arabidopsis thaliana putative G-type lectin S-receptor-like serine/threonine-protein kinase mRNA, complete cds
AT4G34950	-1.447071945	5.83E-26	Arabidopsis thaliana major facilitator family protein mRNA, complete cds

AT5G42800	-1.084501866	9.81E-26	Arabidopsis thaliana dihydroflavonol-4-reductase mRNA, complete cds
AT5G04250	-1.030222757	1.00E-25	Arabidopsis thaliana OTU-like cysteine protease family protein mRNA, complete cds
AT1G14120	-1.787523907	1.11E-25	Arabidopsis thaliana 2-oxoglutarate (2OG) and Fe(II)-dependent oxygenase-like protein mRNA, complete cds
AT1G31550	-1.375341511	1.66E-25	Arabidopsis thaliana GDSL esterase/lipase mRNA, complete cds
AT1G09240	-1.457636392	4.29E-25	Arabidopsis thaliana chromosome 1 sequence
AT3G02875	-1.129048305	7.60E-25	Arabidopsis thaliana IAA-amino acid hydrolase ILR1 mRNA, complete cds
AT1G62975	-1.250213091	9.21E-25	Arabidopsis thaliana transcription factor bHLH125 mRNA, complete cds
AT1G22400	-1.239452109	1.17E-24	Arabidopsis thaliana UDP-glycosyltransferase 85A1 mRNA, complete cds
AT1G18590	-1.17726009	2.69E-24	Arabidopsis thaliana chromosome 1 sequence
AT5G67210	-1.196010181	8.62E-24	Arabidopsis thaliana chromosome 5 sequence
AT5G42930	-1.801338721	1.37E-23	Arabidopsis thaliana lipase class 3-like protein mRNA, complete cds
AT1G51090	-1.236719963	2.77E-23	Arabidopsis thaliana uncharacterized protein mRNA, complete cds
AT4G21840	-1.492917524	1.47E-22	Arabidopsis thaliana methionine sulfoxide reductase B8 mRNA, complete cds
AT2G36590	-1.049044828	1.79E-22	Arabidopsis thaliana proline transporter 3 mRNA, complete cds
AT1G77520	-1.684606658	6.95E-21	Arabidopsis thaliana O-methyltransferase family protein mRNA, complete cds
AT4G15100	-1.560092117	8.70E-21	Arabidopsis thaliana serine carboxypeptidase-like 30 mRNA, complete cds
AT3G16330	-1.176474522	3.77E-20	Arabidopsis thaliana chromosome 3,

			complete sequence
AT2G44810	-3.548951559	5.32E-20	Arabidopsis thaliana chromosome 2, complete sequence
AT4G10390	-1.044427674	7.88E-20	Arabidopsis thaliana probable receptor-like protein kinase mRNA, complete cds
AT1G72760	-1.193057544	2.72E-19	Arabidopsis thaliana putative serine/threonine protein kinase mRNA, complete cds
AT3G51440	-1.189055614	3.78E-19	Arabidopsis thaliana strictosidine synthase family protein mRNA, complete cds
AT4G22214	-2.143312758	4.02E-19	Arabidopsis thaliana defensin-like protein 99 mRNA, complete cds
AT5G67160	-1.1022251	1.32E-18	Arabidopsis thaliana chromosome 5 sequence
AT5G15240	-1.648980377	2.64E-18	Arabidopsis thaliana transmembrane amino acid transporter family protein mRNA, complete cds
AT5G41120	-1.022322369	5.63E-18	Arabidopsis thaliana Esterase/lipase/thioesterase family protein mRNA, complete cds
AT4G27070	-1.0470708	6.45E-18	Arabidopsis thaliana tryptophan synthase beta chain mRNA, complete cds
AT5G07990	-1.590174222	9.34E-18	Arabidopsis thaliana Flavonoid 3'-monooxygenase mRNA, complete cds
AT2G35070	-1.618214221	1.57E-16	Arabidopsis thaliana uncharacterized protein mRNA, complete cds
AT4G37710	-1.387488136	1.02E-15	Arabidopsis thaliana chromosome 4 sequence
AT5G23830	-1.110509455	1.30E-15	Arabidopsis thaliana MD-2-related lipid recognition domain-containing protein mRNA, complete cds
AT5G47990	-1.087528093	1.75E-15	Arabidopsis thaliana cytochrome P450 705A5 mRNA, complete cds
AT2G22760	-1.865808664	4.57E-15	Arabidopsis thaliana transcription factor bHLH19 mRNA, complete cds
AT1G71100	-1.013742093	6.49E-15	Arabidopsis thaliana chromosome 1 sequence
AT1G04570	-1.165086701	2.82E-14	Arabidopsis thaliana probable

			folate-biopterin transporter mRNA, complete cds
AT5G38120	-1.095233592	2.29E-13	Arabidopsis thaliana 4-coumarate--CoA ligase-like 8 mRNA, complete cds
AT5G17450	-1.207260209	3.70E-13	Arabidopsis thaliana heavy metal transport/detoxification domain-containing protein mRNA, complete cds
AT2G47950	-1.244096977	3.82E-13	Arabidopsis thaliana chromosome 2, complete sequence
AT4G36830	-2.079466276	5.83E-13	Arabidopsis thaliana chromosome 4 sequence
AT2G27550	-1.043132943	1.17E-12	Arabidopsis thaliana protein CENTRORADIALIS-like mRNA, complete cds
AT1G17830	-1.043132943	1.17E-12	Arabidopsis thaliana uncharacterized protein mRNA, complete cds
AT4G15200	-1.720319977	3.56E-12	Arabidopsis thaliana formin 3 mRNA, complete cds
AT4G11210	-2.08951994	3.84E-12	Arabidopsis thaliana chromosome 4 sequence
AT5G16190	-1.475888097	4.91E-12	Arabidopsis thaliana cellulose synthase like A11 mRNA, complete cds
AT4G37150	-1.492368031	6.23E-12	Arabidopsis thaliana methyl esterase 9 mRNA, complete cds
AT1G70790	-1.025916807	1.09E-10	Arabidopsis thaliana calcium-dependent lipid-binding domain-containing protein mRNA, complete cds
AT4G22610	-1.225375611	1.71E-10	Arabidopsis thaliana chromosome 4 sequence
AT5G67180	-1.076099425	1.97E-10	Arabidopsis thaliana target of early activation tagged 3 mRNA, complete cds
AT5G33355	-1.213967311	2.06E-10	Arabidopsis thaliana defensin-like protein mRNA, complete cds
AT1G66860	-2.246923022	2.37E-10	Arabidopsis thaliana class I glutamine amidotransferase-like domain-containing protein mRNA, complete cds
AT5G61890	-1.268323093	2.43E-10	Arabidopsis thaliana

			ethylene-responsive transcription factor ERF114 mRNA, complete cds
AT5G55930	-1.036968504	2.93E-10	Arabidopsis thaliana oligopeptide transporter 1 mRNA, complete cds
AT1G79900	-1.230504033	4.39E-10	Arabidopsis thaliana mitochondrial arginine transporter BAC2 mRNA, complete cds
AT2G26370	-2.03798964	4.45E-10	Arabidopsis thaliana MD-2-related lipid recognition domain-containing protein mRNA, complete cds
AT1G32960	-1.43176802	5.48E-10	Arabidopsis thaliana Subtilase family protein SBT3.3 mRNA, complete cds
AT4G37850	-1.048053323	5.70E-10	Arabidopsis thaliana transcription factor bHLH25 mRNA, complete cds
AT5G38710	-1.040487196	6.55E-10	Arabidopsis thaliana proline dehydrogenase 2 mRNA, complete cds
AT2G26560	-1.249391277	9.27E-10	Arabidopsis thaliana phospholipase A 2A mRNA, complete cds
AT3G50280	-1.338384573	9.53E-10	Arabidopsis thaliana chromosome 3, complete sequence
AT2G42560	-1.368379313	9.99E-10	Arabidopsis thaliana late embryogenesis abundant domain-containing protein mRNA, complete cds
AT2G15780	-1.390253813	1.50E-09	Arabidopsis thaliana Cupredoxin superfamily protein mRNA, complete cds
AT4G11911	-1.849818038	1.65E-09	Arabidopsis thaliana uncharacterized protein mRNA, complete cds
AT1G29195	-1.127124894	1.24E-08	Arabidopsis thaliana chromosome 1 sequence
AT3G23550	-1.051451899	2.61E-08	Arabidopsis thaliana MATE efflux family protein LAL5 mRNA, complete cds
AT1G62370	-1.247295859	2.71E-08	Arabidopsis thaliana chromosome 1 sequence
AT4G06746	-1.51857791	3.54E-08	Arabidopsis thaliana chromosome 4 sequence
AT4G01895	-1.881526898	4.90E-08	Arabidopsis thaliana chromosome 4

			sequence
AT4G16146	-1.881526898	4.90E-08	Arabidopsis thaliana cAMP-regulated phosphoprotein 19-related protein mRNA, complete cds
AT1G72260	-2.408773901	6.29E-08	Arabidopsis thaliana thionin 2.1 mRNA, complete cds
AT4G25433	-1.33985596	6.59E-08	Arabidopsis thaliana chromosome 4 sequence
AT5G65080	-1.860153247	7.75E-08	Arabidopsis thaliana protein MADS AFFECTING FLOWERING 5 mRNA, complete cds
AT5G56840	-1.45875375	1.03E-07	Arabidopsis thaliana myb-like transcription factor family protein mRNA, complete cds
AT5G48010	-2.285917153	1.17E-07	Arabidopsis thaliana thalianol synthase 1 mRNA, complete cds
AT1G66050	-1.345418165	2.41E-07	Arabidopsis thaliana E3 ubiquitin-protein ligase ORTHRUS 5 mRNA, complete cds
AT2G36780	-1.345418165	2.41E-07	Arabidopsis thaliana chromosome 2, complete sequence
AT2G24840	-1.229579169	2.68E-07	Arabidopsis thaliana chromosome 2, complete sequence
AT5G02170	-1.001956908	3.56E-07	Arabidopsis thaliana transmembrane amino acid transporter family protein mRNA, complete cds
AT4G15660	-1.174206766	4.00E-07	Arabidopsis thaliana chromosome 4 sequence
AT2G01008	-1.708150154	4.07E-07	Arabidopsis thaliana uncharacterized protein mRNA, complete cds
AT5G64700	-1.074171976	4.07E-07	Arabidopsis thaliana nodulin MtN21 /EamA-like transporter family protein mRNA, complete cds
AT1G58590	-1.142959199	5.65E-07	Arabidopsis thaliana RXW18 mRNA
AT5G50570	-1.659134477	5.76E-07	Arabidopsis thaliana squamosa promoter-binding-like protein 13 mRNA, complete cds
AT2G25820	-1.432137894	7.10E-07	Arabidopsis thaliana chromosome 2, complete sequence
AT3G43250	-1.590174222	7.24E-07	Arabidopsis thaliana chromosome 3, complete sequence

AT5G51760	-2.022882747	9.24E-07	Arabidopsis thaliana probable protein phosphatase AHG1 mRNA, complete cds
AT3G05800	-1.017627022	1.15E-06	Arabidopsis thaliana chromosome 3, complete sequence
AT3G22231	-1.373379994	1.17E-06	Arabidopsis thaliana protein PATHOGEN AND CIRCADIAN CONTROLLED 1 mRNA, complete cds
AT4G22620	-1.379026558	2.23E-06	Arabidopsis thaliana chromosome 4 sequence
AT4G12410	-1.098918638	2.83E-06	Arabidopsis thaliana chromosome 4 sequence
AT3G53790	-2.508309574	2.87E-06	Arabidopsis thaliana protein TRF-like 4 mRNA, complete cds
AT1G61800	-1.418554922	3.34E-06	Arabidopsis thaliana glucose-6-phosphate/phosphate translocator 2 mRNA, complete cds
AT4G30450	-1.126927629	4.59E-06	Arabidopsis thaliana chromosome 4 sequence
AT2G36255	-1.963989058	6.33E-06	Arabidopsis thaliana putative defensin-like protein 203 mRNA, complete cds
AT1G10530	-1.093272075	6.33E-06	Arabidopsis thaliana uncharacterized protein mRNA, complete cds
AT5G47980	-2.034378386	6.36E-06	Arabidopsis thaliana chromosome 5 sequence
AT1G12540	-2.034378386	6.36E-06	Arabidopsis thaliana transcription factor bHLH55 mRNA, complete cds
AT1G05135	-1.657885931	7.01E-06	Arabidopsis thaliana chromosome 1 sequence
AT5G20670	-1.015519359	9.45E-06	Arabidopsis thaliana chromosome 5 sequence
AT1G34047	-2.77134398	9.92E-06	Arabidopsis thaliana defensin-like protein 208 mRNA, complete cds
AT5G09980	-1.031733665	1.14E-05	Arabidopsis thaliana elicitor peptide 4 mRNA, complete cds
AT2G35300	-1.126260487	1.21E-05	Arabidopsis thaliana chromosome 2, complete sequence
AT4G36700	-1.306381256	1.91E-05	Arabidopsis thaliana cupin family protein mRNA, complete cds
AT3G22250	-2.500041958	2.12E-05	Arabidopsis thaliana

			UDP-glycosyltransferase 82A1 mRNA, complete cds
AT5G20480	-1.317626013	2.21E-05	Arabidopsis thaliana LRR receptor-like serine/threonine-protein kinase EFR mRNA, complete cds
AT2G25090	-1.642060963	3.06E-05	Arabidopsis thaliana SNF1-related kinase mRNA, complete cds
AT2G32487	-1.642060963	3.06E-05	Arabidopsis thaliana uncharacterized protein mRNA, complete cds
AT3G48660	-2.449415885	3.63E-05	Arabidopsis thaliana uncharacterized protein mRNA, complete cds
AT4G36110	-1.138486789	5.20E-05	Arabidopsis thaliana chromosome 4 sequence
AT5G18050	-1.285917153	5.32E-05	Arabidopsis thaliana chromosome 5 sequence
AT3G43980	-1.094385696	7.22E-05	Arabidopsis thaliana 40S ribosomal protein S29 mRNA, complete cds
AT5G37940	-2.008383178	7.25E-05	Arabidopsis thaliana zinc-binding dehydrogenase family protein mRNA, complete cds
AT3G05820	-1.013457735	9.34E-05	Arabidopsis thaliana alkaline/neutral invertase H mRNA, complete cds
AT3G18670	-1.193470904	9.69E-05	Arabidopsis thaliana ankyrin repeat-containing protein mRNA, complete cds
AT1G71910	-1.053626271	9.80E-05	Arabidopsis thaliana chromosome 1 sequence
AT1G63040	-3.963989058	0.000101958	Arabidopsis thaliana chromosome 1 sequence
AT4G16745	-1.240829264	0.000109204	Arabidopsis thaliana Exostosin family protein mRNA, complete cds
AT5G67080	-1.034378386	0.000113366	Arabidopsis thaliana chromosome 5 sequence
AT5G54165	-1.165622919	0.000116343	Arabidopsis thaliana chromosome 5 sequence
AT2G23010	-1.01582799	0.000130456	Arabidopsis thaliana serine carboxypeptidase-like 9 mRNA, complete cds
AT1G74000	-1.139075765	0.000138619	Arabidopsis thaliana strictosidine synthase 3 mRNA, complete cds

AT1G71140	-1.548951559	0.000185042	Arabidopsis thaliana MATE efflux family protein mRNA, complete cds
AT2G35730	-1.08951994	0.000192684	Arabidopsis thaliana heavy-metal-associated domain-containing protein mRNA, complete cds
AT2G43520	-1.455842155	0.000218706	Arabidopsis thaliana trypsin inhibitor protein 2 mRNA, complete cds
AT1G25250	-1.04199157	0.000220942	Arabidopsis thaliana shoot gravitropism5-like protein mRNA, complete cds
AT2G42150	-1.483363217	0.000248227	Arabidopsis thaliana DNA-binding bromodomain-containing protein mRNA, complete cds
AT2G29470	-1.202775918	0.000258676	Arabidopsis thaliana glutathione S-transferase tau 3 mRNA, complete cds
AT5G16080	-1.211916572	0.00030312	Arabidopsis thaliana chromosome 5 sequence
AT1G76640	-1.170439936	0.000311625	Arabidopsis thaliana chromosome 1 sequence
AT1G20310	-1.170439936	0.000311625	Arabidopsis thaliana chromosome 1 sequence
AT1G66370	-1.449415885	0.000371945	Arabidopsis thaliana transcription factor MYB113 mRNA, complete cds
AT2G34350	-1.449415885	0.000371945	Arabidopsis thaliana nodulin-like domain-containing protein mRNA, complete cds
AT4G37720	-1.633840457	0.000389146	Arabidopsis thaliana phytosulfokine 6 precursor mRNA, complete cds
AT1G32910	-1.390253813	0.000483114	Arabidopsis thaliana HXXXD-type acyl-transferase-like protein mRNA, complete cds
AT4G37430	-2.165622919	0.000508875	Arabidopsis thaliana cytochrome P450 81F1 mRNA, complete cds
AT4G25420	-1.153022883	0.000515983	Arabidopsis thaliana gibberellin 20 oxidase 1 mRNA, complete cds
AT4G00870	-1.083287987	0.000517722	Arabidopsis thaliana transcription factor bHLH14 mRNA, complete cds
AT4G23240	-1.548951559	0.000537618	Arabidopsis thaliana cysteine-rich receptor-like protein kinase 16

			mRNA, complete cds
AT3G30340	-1.335957836	0.000615954	Arabidopsis thaliana nodulin MtN21 /EamA-like transporter family protein mRNA, complete cds
AT4G36000	-3.664428776	0.000648411	Arabidopsis thaliana pathogenesis-related thaumatin family protein mRNA, complete cds
AT1G22340	-1.642060963	0.000663784	Arabidopsis thaliana UDP-glucosyl transferase 85A7 mRNA, complete cds
AT2G14560	-1.215527825	0.000695146	Arabidopsis thaliana protein LURP1 mRNA, complete cds
AT3G21520	-1.700954652	0.000725996	Arabidopsis thaliana chromosome 3, complete sequence
AT2G07671	-2.285917153	0.000805516	Arabidopsis thaliana ecotype Col-0 mitochondrion, complete genome
AT1G60450	-4.423420677	0.000899232	Arabidopsis thaliana galactinol synthase 7 mRNA, complete cds
AT2G24210	-4.423420677	0.000899232	Arabidopsis thaliana terpene synthase 10 mRNA, complete cds
AT1G56480	-4.423420677	0.000899232	Arabidopsis thaliana chromosome 1 sequence
AT5G54400	-1.18638148	0.000989526	Arabidopsis thaliana S-adenosyl-L-methionine-dependent methyltransferase-like protein mRNA, complete cds
AT5G15250	-2.870879654	0.000990416	Arabidopsis thaliana ATP-dependent zinc metalloprotease FTSH 6 mRNA, complete cds
AT1G70080	-1.253495675	0.001116651	Arabidopsis thaliana terpenoid synthase 6 mRNA, complete cds
AT3G17980	-3.548951559	0.001205409	Arabidopsis thaliana calcium-dependent lipid-binding domain-containing protein mRNA, complete cds
AT1G09510	-3.548951559	0.001205409	Arabidopsis thaliana alcohol dehydrogenase-like protein mRNA, complete cds
AT5G40348	-3.548951559	0.001205409	Arabidopsis thaliana clone 102435 mRNA sequence
AT4G27140	-3.548951559	0.001205409	Arabidopsis thaliana chromosome 4 sequence
AT5G17390	-1.800490326	0.001328277	Arabidopsis thaliana Adenine nucleotide alpha hydrolases-like

			superfamily protein mRNA, complete cds
AT1G18075	-1.800490326	0.001328277	Arabidopsis thaliana chromosome 1 sequence
AT2G24165	-1.800490326	0.001328277	Arabidopsis thaliana chromosome 2, complete sequence
AT1G71000	-1.207914641	0.001367068	Arabidopsis thaliana chaperone DnaJ-domain containing protein mRNA, complete cds
AT2G30900	-2.211916572	0.001369026	Arabidopsis thaliana protein TRICHOME BIREFRINGENCE-LIKE 43 mRNA, complete cds
AT1G55525	-2.034378386	0.00141314	Arabidopsis thaliana clone 155459 mRNA sequence
AT2G37580	-1.119267284	0.001669284	Arabidopsis thaliana chromosome 2, complete sequence
ATMG00640	-2.77134398	0.001779905	A.thaliana mitochondrial DNA for genes nad4L and orf25
AT3G48850	-1.175493163	0.001947467	Arabidopsis thaliana phosphate transporter 3;2 mRNA, complete cds
AT1G33030	-2.379026558	0.002145725	Arabidopsis thaliana O-methyltransferase-like protein mRNA, complete cds
AT3G12890	-1.249391277	0.002208119	Arabidopsis thaliana protein activator of spomin::LUC2 mRNA, complete cds
AT3G59130	-3.423420677	0.002244033	Arabidopsis thaliana Cysteine/Histidine-rich C1 domain family protein mRNA, complete cds
AT2G29710	-1.018436842	0.002245721	Arabidopsis thaliana chromosome 2, complete sequence
AT3G23440	-1.838458176	0.002255171	Arabidopsis thaliana chromosome 3, complete sequence
AT3G61170	-1.838458176	0.002255171	Arabidopsis thaliana pentatricopeptide (PPR) repeat-containing protein mRNA, complete cds
AT1G24260	-1.08951994	0.002332682	Arabidopsis thaliana MADs box transcription factor SEPALLATA3 mRNA, complete cds
AT1G48260	-1.020572587	0.002667115	Arabidopsis thaliana SNF1-related kinase mRNA, complete cds

AT2G22810	-1.1423263	0.002758334	Arabidopsis thaliana 1-aminocyclopropane-1-carboxylate synthase mRNA, complete cds
AT2G27480	-1.095233592	0.002764234	Arabidopsis thaliana putative calcium-binding protein CML48 mRNA, complete cds
AT1G05560	-1.607845248	0.003039975	Arabidopsis thaliana chromosome 1 sequence
AT2G41445	-4.13391406	0.003165883	Arabidopsis thaliana uncharacterized protein mRNA, complete cds
AT1G61110	-4.13391406	0.003165883	Arabidopsis thaliana NAC domain containing protein 25 mRNA, complete cds
AT5G44460	-2.664428776	0.003188031	Arabidopsis thaliana chromosome 5 sequence
AT3G45130	-1.680196092	0.00335106	Arabidopsis thaliana lanosterol synthase 1 mRNA, complete cds
AT1G65850	-1.77134398	0.003627472	Arabidopsis thaliana TIR-NBS-LRR class disease resistance protein mRNA, complete cds
AT3G50170	-2.285917153	0.003708385	Arabidopsis thaliana uncharacterized protein mRNA, complete cds
AT4G29180	-1.889988477	0.003826415	Arabidopsis thaliana protein root hair specific 16 mRNA, complete cds
AT5G09470	-1.889988477	0.003826415	Arabidopsis thaliana dicarboxylate carrier 3 mRNA, complete cds
AT1G68350	-1.889988477	0.003826415	Arabidopsis thaliana chromosome 1 sequence
AT3G57500	-1.063524732	0.003849521	Arabidopsis thaliana chromosome 3, complete sequence
AT3G53232	-1.494503775	0.004169748	Arabidopsis thaliana chromosome 3, complete sequence
AT5G53815.1	-1.548951559	0.004711549	Arabidopsis thaliana chromosome 5 sequence
AT2G04190	-1.548951559	0.004711549	Arabidopsis thaliana TRAF-like family protein mRNA, complete cds
AT2G05518	-1.26354934	0.005157876	Arabidopsis thaliana mRNA, clone: RAFL17-49-H20
AT5G23950	-1.073613549	0.005434246	Arabidopsis thaliana chromosome 5 sequence
AT3G27120	-1.396948466	0.005510854	Arabidopsis thaliana P-loop

			containing nucleoside triphosphate hydrolases superfamily protein mRNA, complete cds
AT1G68238	-1.700954652	0.005790656	Arabidopsis thaliana chromosome 1 sequence
AT1G11850	-1.700954652	0.005790656	Arabidopsis thaliana chromosome 1 sequence
AT1G03790	-1.700954652	0.005790656	Arabidopsis thaliana chromosome 1 sequence
AT2G06045.1	-3.963989058	0.006000848	Arabidopsis thaliana chromosome 2, complete sequence
AT1G09260	-3.963989058	0.006000848	Arabidopsis thaliana chromosome 1 sequence
AT4G10290	-3.963989058	0.006000848	Arabidopsis thaliana RmlC-like cupins superfamily protein mRNA, complete cds
AT1G28830	-3.963989058	0.006000848	Theobroma cacao Uncharacterized protein (TCM_007238) mRNA, complete cds
AT3G23450	-1.285917153	0.006042479	Arabidopsis thaliana chromosome 3, complete sequence
AT5G03204	-1.285917153	0.006042479	Arabidopsis thaliana chromosome 5 sequence
AT5G46600	-1.034378386	0.006343774	Arabidopsis thaliana aluminum activated malate transporter family protein mRNA, complete cds
AT1G06540	-2.18638148	0.006369368	Arabidopsis thaliana uncharacterized protein mRNA, complete cds
AT5G49015	-1.963989058	0.006473638	Arabidopsis thaliana uncharacterized protein mRNA, complete cds
AT4G28330	-1.21774565	0.007488488	Arabidopsis thaliana chromosome 4 sequence
AT3G57240	-3.13391406	0.007800789	Arabidopsis thaliana beta-1,3-glucanase 3 mRNA, complete cds
AT2G28920	-3.13391406	0.007800789	Arabidopsis thaliana chromosome 2, complete sequence
AT3G03760	-3.13391406	0.007800789	Arabidopsis thaliana LOB domain-containing protein 20 mRNA, complete cds
AT3G60700	-3.13391406	0.007800789	Arabidopsis thaliana uncharacterized protein mRNA,

			complete cds
AT2G41850	-1.548951559	0.008203103	Arabidopsis thaliana polygalacturonase ADPG2 mRNA, complete cds
AT1G22380	-1.04199157	0.009004145	Arabidopsis thaliana UDP-glucosyl transferase 85A3 mRNA, complete cds
AT2G34340	-1.156634136	0.009101606	Arabidopsis thaliana chromosome 2, complete sequence
AT2G22590	-1.626954071	0.00916793	Arabidopsis thaliana chromosome 2, complete sequence
AT3G12410	-1.379026558	0.009562078	Arabidopsis thaliana chromosome 3, complete sequence
AT5G07640	-1.379026558	0.009562078	Arabidopsis thaliana RING/U-box superfamily protein mRNA, complete cds
AT5G37620	-2.423420677	0.010095566	Arabidopsis thaliana chromosome 5 sequence
AT1G53420	-2.423420677	0.010095566	Arabidopsis thaliana leucine-rich repeat transmembrane protein kinase mRNA, complete cds
AT1G80590	-2.423420677	0.010095566	Arabidopsis thaliana putative WRKY transcription factor 66 mRNA, complete cds
AT1G19160	-2.423420677	0.010095566	Arabidopsis thaliana chromosome 1 sequence
AT1G11610	-3.77134398	0.011459365	Arabidopsis thaliana cytochrome P450 71A18 mRNA, complete cds
AT3G44006	-3.77134398	0.011459365	Arabidopsis thaliana chromosome 3, complete sequence
AT1G08440	-1.285917153	0.012201689	Arabidopsis thaliana aluminum-activated malate transporter 2 mRNA, complete cds
AT1G69720	-1.003517422	0.012463538	Arabidopsis thaliana heme oxygenase 3 mRNA, complete cds
AT3G20470	-1.18638148	0.012787198	Arabidopsis thaliana chromosome 3, complete sequence
AT1G65880	-2.963989058	0.014553698	Arabidopsis thaliana benzoate-CoA ligase mRNA, complete cds
AT1G11560	-2.963989058	0.014553698	Arabidopsis thaliana chromosome 1 sequence
AT1G62240	-2.963989058	0.014553698	Arabidopsis thaliana chromosome 1 sequence
AT3G57270	-2.963989058	0.014553698	Arabidopsis thaliana

			beta-1,3-glucanase 1 mRNA, complete cds
AT5G28235	-2.963989058	0.014553698	Arabidopsis thaliana Ulp1 protease family protein mRNA, complete cds
AT2G47360	-1.005809234	0.014897874	Arabidopsis thaliana chromosome 2, complete sequence
AT5G06990	-1.121530335	0.015423536	Arabidopsis thaliana chromosome 5 sequence
AT5G53895	-1.642060963	0.01608095	Arabidopsis thaliana uncharacterized protein mRNA, complete cds
AT1G75790	-1.642060963	0.01608095	Arabidopsis thaliana SKU5 similar 18 protein mRNA, complete cds
AT1G31240	-1.356306481	0.016693673	Arabidopsis thaliana chromosome 1 sequence
AT1G68250	-1.356306481	0.016693673	Arabidopsis thaliana chromosome 1 sequence
AT3G44970	-1.356306481	0.016693673	Arabidopsis thaliana cytochrome P450 family protein mRNA, complete cds
AT4G09420	-1.77134398	0.017545366	Arabidopsis thaliana TIR-NBS class of disease resistance protein mRNA, complete cds
AT5G60220	-1.77134398	0.017545366	Arabidopsis thaliana tetraspanin4 mRNA, complete cds
AT2G06002	-2.285917153	0.017815121	Arabidopsis thaliana chromosome 2, complete sequence
AT3G15170	-2.285917153	0.017815121	Arabidopsis thaliana protein CUP-SHAPED COTYLEDON 1 mRNA, complete cds
AT1G79680	-1.008383178	0.017823963	Arabidopsis thaliana wall-associated receptor kinase-like 10 mRNA, complete cds
AT2G04090	-1.227023464	0.017923684	Arabidopsis thaliana MATE efflux family protein mRNA, complete cds
AT2G17680	-1.063524732	0.018281469	Arabidopsis thaliana chromosome 2, complete sequence
AT5G50760	-1.963989058	0.018374136	Arabidopsis thaliana chromosome 5 sequence
AT5G62730	-1.963989058	0.018374136	Arabidopsis thaliana probable peptide/nitrate transporter mRNA, complete cds
AT1G25422	-1.963989058	0.018374136	Arabidopsis thaliana chromosome 1 sequence

AT2G32660	-1.40456165	0.019390947	Arabidopsis thaliana chromosome 2, complete sequence
AT5G04630	-3.548951559	0.022060374	Arabidopsis thaliana chromosome 5 sequence
AT1G60740	-3.548951559	0.022060374	Arabidopsis thaliana peroxiredoxin-2D mRNA, complete cds
AT1G24590	-3.548951559	0.022060374	Arabidopsis thaliana chromosome 1 sequence
AT2G46685	-3.548951559	0.022060374	Arabidopsis thaliana chromosome 2, complete sequence
AT2G07765	-3.548951559	0.022060374	Eruca vesicaria subsp. sativa mitochondrion, complete genome
AT3G04200	-3.548951559	0.022060374	Arabidopsis thaliana RmlC-like cupins superfamily protein mRNA, complete cds
AT2G05960.1	-3.548951559	0.022060374	Arabidopsis thaliana chromosome 2, complete sequence
AT5G01595	-3.548951559	0.022060374	Arabidopsis thaliana Full-length cDNA Complete sequence from clone GSLTSIL39ZC05 of Silique of strain col-0 of Arabidopsis thaliana (thale cress)
AT5G62780	-3.548951559	0.022060374	Arabidopsis thaliana chaperone DnaJ-domain containing protein mRNA, complete cds
AT2G21420	-3.548951559	0.022060374	Arabidopsis thaliana IBR domain containing protein mRNA, complete cds
AT5G54700	-3.548951559	0.022060374	Arabidopsis thaliana ankyrin repeat-containing protein mRNA, complete cds
AT3G29300	-3.548951559	0.022060374	Arabidopsis thaliana chromosome 3, complete sequence
AT3G13660	-3.548951559	0.022060374	Arabidopsis thaliana chromosome 3, complete sequence
AT3G13850	-3.548951559	0.022060374	Arabidopsis thaliana chromosome 3, complete sequence
AT1G64185	-1.466489399	0.022352869	Arabidopsis thaliana lactoylglutathione lyase / glyoxalase I-like protein mRNA, complete cds
AT1G72490	-1.285917153	0.024983085	Arabidopsis thaliana uncharacterized protein mRNA, complete cds

AT2G33780	-1.285917153	0.024983085	Arabidopsis thaliana chromosome 2, complete sequence
AT1G54930	-1.548951559	0.025467004	Arabidopsis thaliana GRF zinc finger / zinc knuckle protein mRNA, complete cds
AT1G19610	-1.548951559	0.025467004	Arabidopsis thaliana defensin-like protein 19 mRNA, complete cds
AT2G38490	-1.014615131	0.025592439	Arabidopsis thaliana chromosome 2, complete sequence
AT1G11570	-1.165622919	0.026085495	Arabidopsis thaliana chromosome 1 sequence
AT2G10975	-2.77134398	0.027140465	Arabidopsis thaliana uncharacterized protein mRNA, complete cds
AT4G31354	-2.77134398	0.027140465	Arabidopsis thaliana chromosome 4 sequence
AT1G60720	-2.77134398	0.027140465	Arabidopsis thaliana chromosome 1 sequence
AT5G55360	-2.77134398	0.027140465	Arabidopsis thaliana chromosome 5 sequence
ATCG00030	-2.77134398	0.027140465	Cyanidiaceae sp. MX-AZ01 chloroplast, complete genome
AT3G50120	-2.77134398	0.027140465	Arabidopsis thaliana uncharacterized protein mRNA, complete cds
AT2G21220	-2.77134398	0.027140465	Arabidopsis thaliana chromosome 2, complete sequence
AT2G02620	-2.77134398	0.027140465	Arabidopsis thaliana chromosome 2, complete sequence
AT1G61050	-1.664428776	0.028469628	Arabidopsis thaliana chromosome 1 sequence
AT4G38000	-1.326559138	0.02937285	Arabidopsis thaliana chromosome 4 sequence
AT3G27920	-1.018436842	0.030720803	Arabidopsis thaliana trichome differentiation protein GL1 mRNA, complete cds
AT1G44890	-1.018436842	0.030720803	Arabidopsis thaliana uncharacterized protein mRNA, complete cds
AT2G33880	-1.838458176	0.030789633	Arabidopsis thaliana WUSCHEL-related homeobox 9 mRNA, complete cds
AT1G47610	-1.838458176	0.030789633	Arabidopsis thaliana chromosome 1 sequence

AT4G11310	-1.838458176	0.030789633	Arabidopsis thaliana putative cysteine proteinase mRNA, complete cds
AT2G18720	-1.838458176	0.030789633	Arabidopsis thaliana translation elongation factor EF1A/initiation factor IF2gamma family protein mRNA, complete cds
AT1G23965	-1.838458176	0.030789633	Arabidopsis thaliana chromosome 1 sequence
AT4G31970	-1.18638148	0.031076442	Arabidopsis thaliana cytochrome P450, family 82, subfamily C, polypeptide 2 mRNA, complete cds
AT1G29540	-2.13391406	0.031210313	Arabidopsis thaliana uncharacterized protein mRNA, complete cds
AT5G40320	-2.13391406	0.031210313	Arabidopsis thaliana chromosome 5 sequence

A **B**

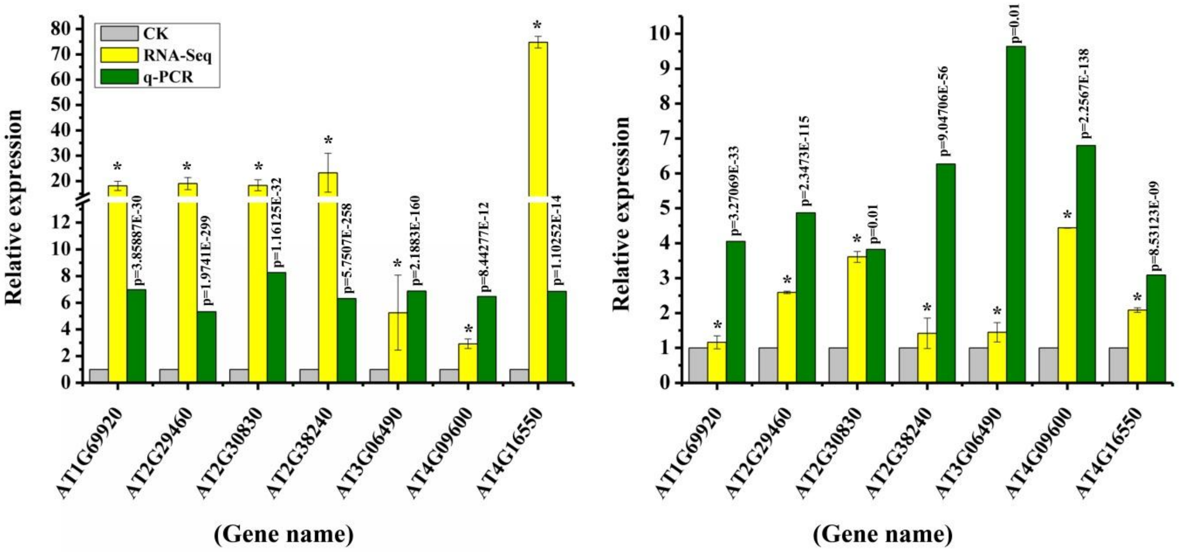

Supplementary Data 6 Quantitative RT-PCR validation of randomly selected genes from Arabidopsis. Two week-old Arabidopsis seedlings were treated with 0 and 200 mM NaCl and sampled at 1, 2 and 4 h, respectively. Total RNA was extracted and qPCR-PCR was performed. A: wild-type; B: *fc1* mutant plants.

Supplementary Data 7A. Primer sequences used for semi-quantitative RT-PCR analysis

Primer name	Forward (5'-3')	Reverse (5'-3')
AtFC1	TGCTATTGGTGGTGGCTCT	GTCGGTGATGAAGGATGGT
ACTIN	TCGTTTCGCTTTCCTTAG	CTTCACCATTCCAGTTCC

Supplementary Data 7B. Primer sequences used for mutant identification

Primer name	
LP-1	TTTTGGATTGAGGAACTTTTACAAC
RP-1	AAACTCAATCCACGATTCGTG
LBb1.3	ATTTTGCCGATTTCGGAAC

Supplementary Data 7C. Primer sequences used for quantitative real-time RT-PCR analysis

Primer name	Forward (5'-3')	Reverse (5'-3')
AtFC1 qRT	ACCAAAGGCGAGGCTATGTCAATTC	TCTGCTTCTGGTACGGATCTCCAGG
SOS1 qRT	CACTTCTGGGAAATGGTTGCA	TGCCTTCAGCAATGACAACAC
SOS2 qRT	GCGGGGTTATTCTTTTCGTTATATT	GATTGGGGTCAAGTATCCTATGTAT
SOS3 qRT	ATGGGCTGCTCTGTATCGAAG	TCTACCGTGAATGGCGTGAC
HK1 qRT	CAAACATTAGCGTATGGCCTTAGA	TGGTGTTTAAAACGCTCCACAT
HKT1 qRT	TCAGTGCATATGGAAACGTTGG	CAGCCACCATCGCTGATG
NHX1 qRT	CAGCACAGTGGTGTTTGGTATGCTG	GTATGGATTTTGGGGTGTTGTCATCA
AVP1 qRT	GGAAACACCACTGCTGCTATTGGAA	GGGTCAAAACATCTACGGTGTGGAT
P5CS1 qRT	AGCAGCCTGTAATGCGATGG	AAGTGACGCCTTTGGTTTGC
RD22 qRT	AGGGCTGTTTCCACTGAGG	CACCACAGATTTATCGTCAGACA
AT1G69920	GCCATAGTAGGTCCAATCTCTG	GATGAACTCGGCAACGGTAG
AT2G29460	GATGAACAGGTTGGACCAGTAG	TTCTTGAGCCTCCTTGATTGC
AT2G30830	GAGCAGGACTCAGAAGTTAAGA	GAACATCAGGAGCCATAACCAT
AT2G38240	AGCGTGGCGAGAGTTCTT	CAAGGCGGCTTCCATATCC
AT3G06490	GGTGCTGCTTCTTCTTCAGACA	GTTGAACTGTTGTTGCTGCTGT
AT4G09600	CAAGTCGGTGAAGGCGTAGT	CACAGTTGCAGCGGTAACAA
AT4G16550	TACGACTACTCTGAACGGAACT	GGAGAAGCCTAAGAACACCATC
ACTIN	CATCAGGAAGGACTTGTACGG	GATGGACCTGACTCGTCATAC

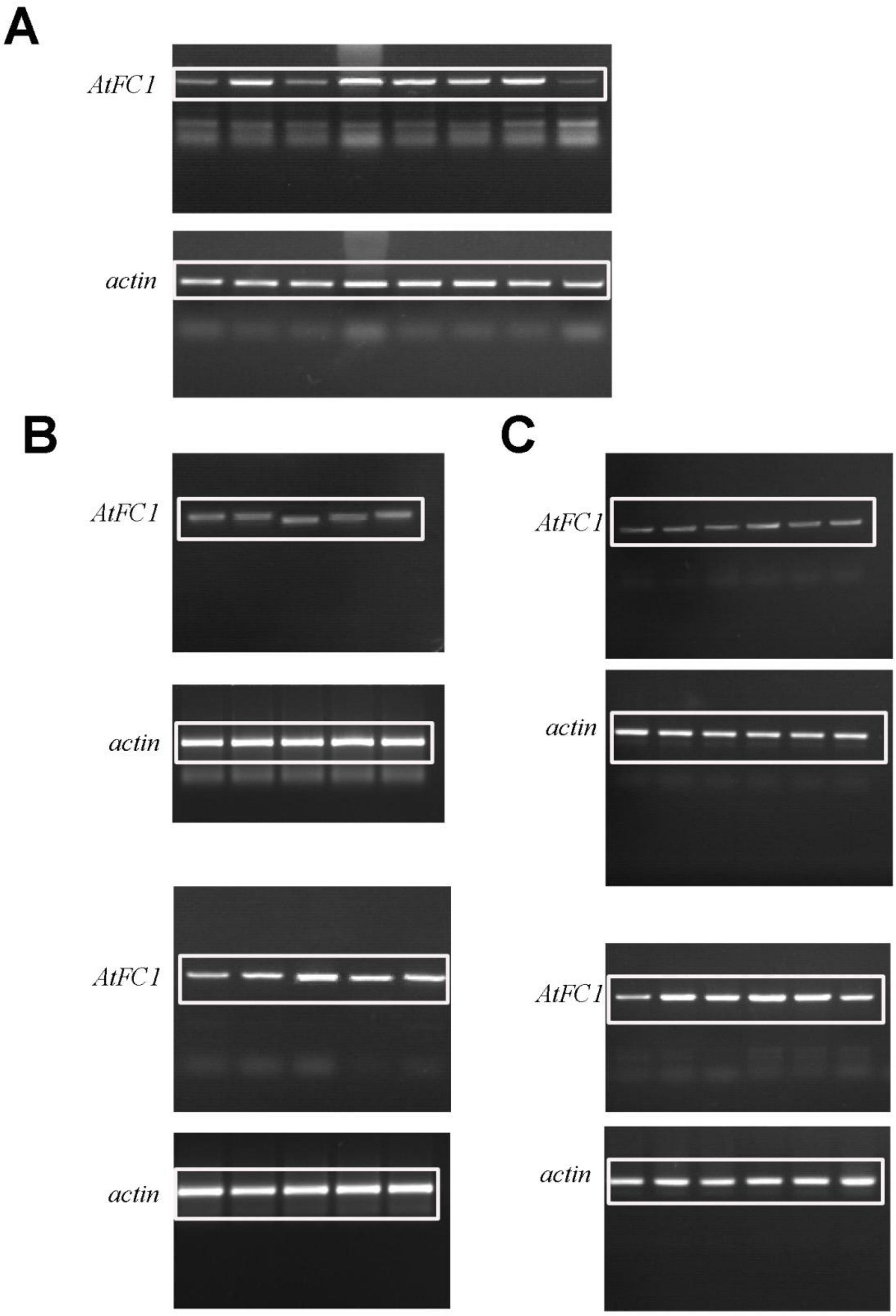

Supplementary Data 8 Uncropped images of Figure 1.

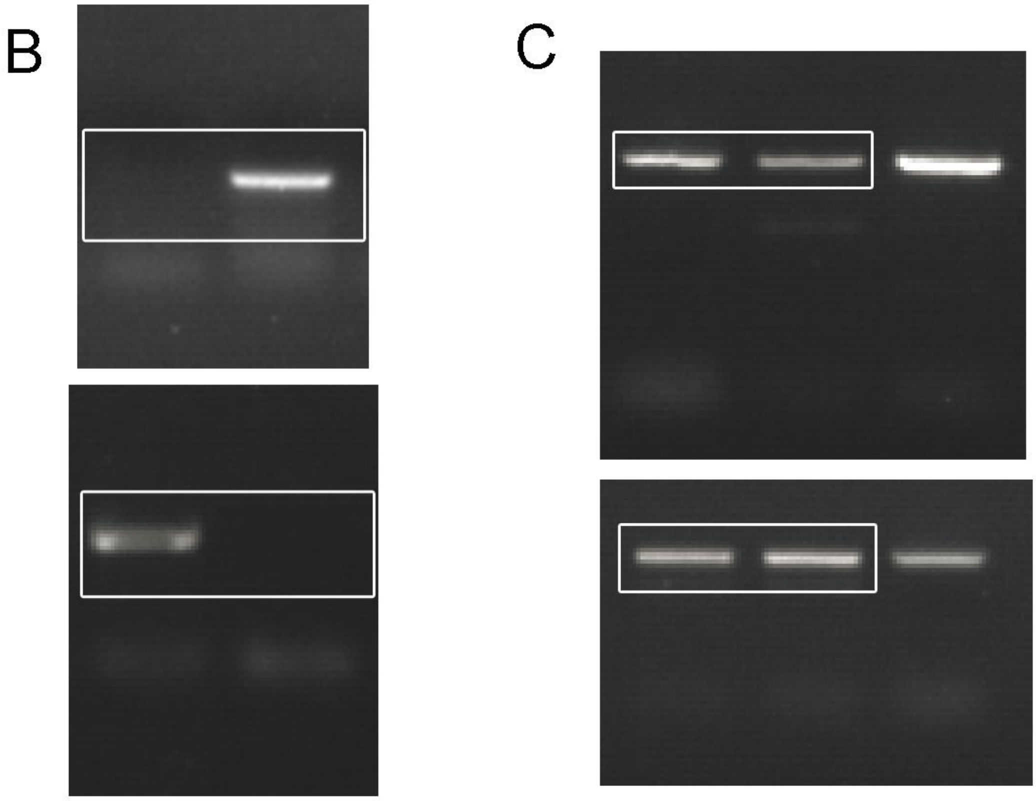

Supplementary Data 9 Uncropped images of Figure 2.
